# Single-cell analysis of senescent epithelia reveals targetable mechanisms promoting fibrosis

**DOI:** 10.1172/jci.insight.154124

**Published:** 2022-11-22

**Authors:** Eoin D. O’Sullivan, Katie J. Mylonas, Rachel Bell, Cyril Carvalho, David P. Baird, Carolynn Cairns, Kevin M. Gallagher, Ross Campbell, Marie Docherty, Alexander Laird, Neil C. Henderson, Tamir Chandra, Kristina Kirschner, Bryan Conway, Gry H. Dihazi, Michael Zeisberg, Jeremy Hughes, Laura Denby, Hassan Dihazi, David A. Ferenbach

**Affiliations:** 1 Centre for Inflammation Research, Queen’s Medical Research Institute, College of Medicine and Veterinary Medicine, University of Edinburgh, Edinburgh, United Kingdom.; 2 Kidney Health Service, Royal Brisbane and Women’s Hospital, Brisbane, Queensland, Australia.; 3 Centre for Cardiovascular Science, Queen’s Medical Research Institute, College of Medicine and Veterinary Medicine, University of Edinburgh, Edinburgh, United Kingdom.; 4 Department of Urology, Western General Hospital, Edinburgh, United Kingdom.; 5 MRC Human Genetics Unit, Institute of Genetics and Molecular Medicine, College of Medicine and Veterinary Medicine, University of Edinburgh, Edinburgh, United Kingdom.; 6 The Institute of Cancer Sciences, University of Glasgow, Glasgow, United Kingdom.; 7 Cancer Research UK Beatson Institute, Glasgow, United Kingdom.; 8 Institute for Clinical Chemistry/UMG-Laboratories,; 9 Clinic for Nephrology and Rheumatology, and; 10 Center for Biostructural Imaging of Neurodegeneration (BIN), University Medical Center Göttingen, Göttingen, Germany.

**Keywords:** Cell Biology, Nephrology, Bioinformatics, Cellular senescence, Fibrosis

## Abstract

Progressive fibrosis and maladaptive organ repair result in significant morbidity and millions of premature deaths annually. Senescent cells accumulate with aging and after injury and are implicated in organ fibrosis, but the mechanisms by which senescence influences repair are poorly understood. Using 2 murine models of injury and repair, we show that obstructive injury generated senescent epithelia, which persisted after resolution of the original injury, promoted ongoing fibrosis, and impeded adaptive repair. Depletion of senescent cells with ABT-263 reduced fibrosis in reversed ureteric obstruction and after renal ischemia/reperfusion injury. We validated these findings in humans, showing that senescence and fibrosis persisted after relieved renal obstruction. We next characterized senescent epithelia in murine renal injury using single-cell RNA-Seq. We extended our classification to human kidney and liver disease and identified conserved profibrotic proteins, which we validated in vitro and in human disease. We demonstrated that increased levels of protein disulfide isomerase family A member 3 (PDIA3) augmented TGF-β–mediated fibroblast activation. Inhibition of PDIA3 in vivo significantly reduced kidney fibrosis during ongoing renal injury and as such represented a new potential therapeutic pathway. Analysis of the signaling pathways of senescent epithelia connected senescence to organ fibrosis, permitting rational design of antifibrotic therapies.

## Graphical abstract

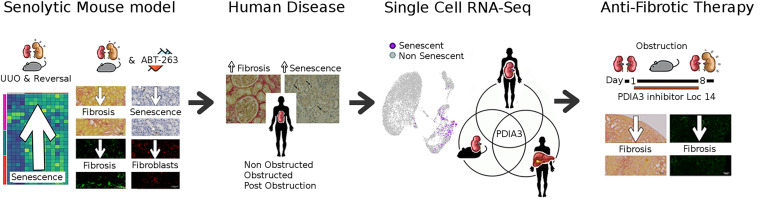

## Introduction

Effective repair following injury is essential for maintaining organ function and health. Kidneys are susceptible to injury during illness, with acute kidney injury (AKI) complicating up to 25% of hospital admissions (1). Maladaptive repair can lead to scarred kidneys and chronic kidney disease (CKD) (2, 3). Furthermore, CKD itself, a disease affecting almost 700 million people worldwide, increases the risk of AKI and maladaptive repair, which can lead to a vicious cycle of acute injury and disease progression (2, 4). Hence, there is an urgent need to understand the mechanisms of maladaptive repair following injury and develop novel interventions and treatments.

Cellular senescence is a heterogeneous phenotype assumed by cells during development and in response to age, injury, or stress. Senescence is an important physiological defense against malignant transformation and plays an important role in wound healing (5, 6). Senescent cells undergo permanent growth arrest and accumulate in multiple organs with increasing age and disease (7). Senescent cells can adopt a secretory phenotype (known as the senescence-associated secretory phenotype, or SASP). In some contexts, such as wound healing, the SASP can have a beneficial effect, but when chronically present the SASP can alter the tissue microenvironment, exerting a detrimental effect on surrounding cells and ultimately disrupting organ function.

The elderly and those with CKD have the highest burden of senescent cells and are the most susceptible to maladaptive repair after injury (8). We have previously demonstrated that removal of preexisting senescent cells prior to kidney injury is protective (9). However, the role of emergent senescent cells after acute injury and during repair remains unknown.

Although multiple cell types in the kidney can express markers of permanent cell cycle arrest, it is renal epithelial cell senescence that correlates most strongly to progressive fibrosis and functional loss in human biopsy samples (10, 11). Increased senescent cell epithelial numbers are found in renal biopsies from older patients or those with kidney disease, and a greater epithelial senescent cell burden is associated with worse clinical outcomes in several renal conditions, including renal transplantation and glomerulonephritis (10, 12–16). Unlike with other cell types, epithelial cell senescence has been shown to accumulate with advancing age in murine models of aging and injury (9, 17). The importance of renal epithelial senescence is supported by data which demonstrate that a variety of injuries can induce renal tubular epithelial cells into senescence (18).

For these reasons, while not discounting the potential importance of senescence in other cell types within the kidney, we chose to address the role of epithelial senescence in maladaptive repair after resolved injury. We demonstrate that senescent epithelia persisted in human kidneys alongside persistent fibrosis following the resolution of ureteric obstruction. Using experimental models of reversible ureteric obstruction and renal ischemia/reperfusion injury in mice, we show that depletion of senescent cells in the aftermath of injury promoted complete repair. In contrast, depletion of senescent cells exacerbated fibrosis during the “initiation” phase of obstructive injury. We characterize senescent epithelial transcriptomes at a single-cell resolution in vivo and explore conserved senescence transcripts across organs and species to identify important profibrotic molecules. One such molecule, protein disulfide isomerase family A member 3 (PDIA3), potentiated TGF-β–mediated fibrosis in vivo. We demonstrate that PDIA3 inhibition during obstructive renal injury was antifibrotic in vivo.

## Results

### Senescent epithelia drive fibrosis during postinjury renal repair.

**Figure 1:**
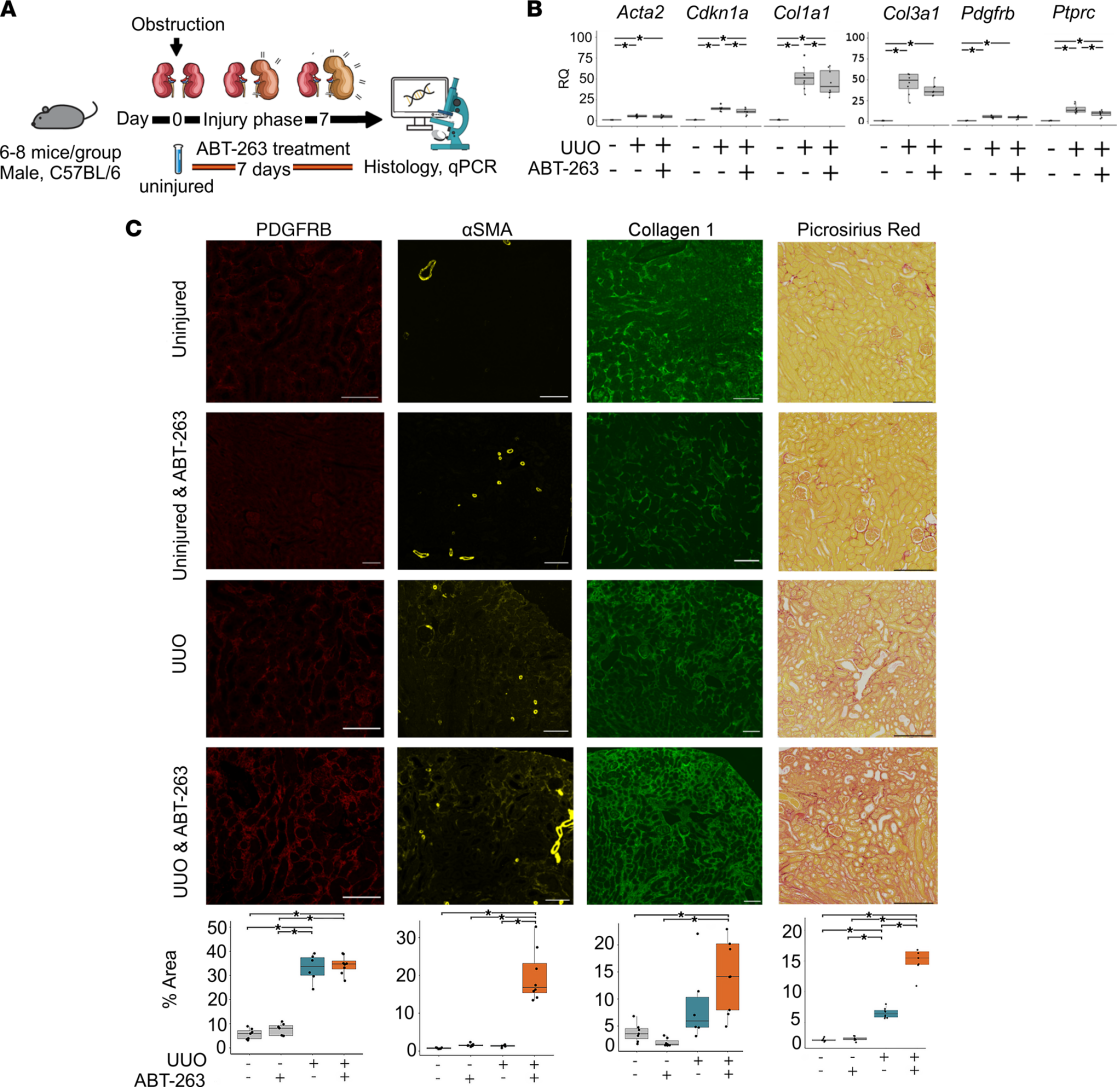
Senescent cell depletion during UUO results in increased renal fibrosis. (**A**) Schematic of murine senescent cell depletion during UUO experiment, with samples taken at days 0 and 7. Note senolytic ABT-263 administration daily from time of injury. *n* = 6–8 per group. (**B**) qPCR analysis of renal tissue after UUO showing reduction in markers of senescence cyclin dependent kinase inhibitor 1A (*Cdkn1a*), no change in myofibroblast markers *Acta2*, *Ptprc*, *Pdgfrb*, or *Col3a1*. * denotes *P* < 0.05 by ANOVA and Tukey test. RQ, relative quantification (fold change). (**C**) Representative images and quantification of immunofluorescence and of Picrosirius red staining of kidneys. Quantification of total staining per renal cortex. * denotes *P* < 0.05. PDGFRB: uninjured and vehicle-treated 5.69% (s.d. 2.23), uninjured and ABT-263–treated 7.53% (s.d. 2.48). UUO and vehicle-treated 33% (s.d. 5.67) versus UUO and ABT-263–treated 34.3% (s.d. 3.77). ANOVA, *P* = 0.921 95% CI: –4.2–6.75. α–Smooth muscle actin (α-SMA): uninjured and vehicle-treated 0.72% (s.d. 0.16), uninjured and ABT-263–treated 1.56% (s.d. 0.39). UUO and vehicle-treated 1.31% (s.d. 0.335) versus UUO and ABT-263–treated 19.9% (s.d. 6.96). ANOVA, *P* < 0.000 95% CI: 12.33–24.8. Collagen 1: uninjured and vehicle-treated 3.75% (s.d. 1.73), uninjured and ABT-263–treated 1.95% (s.d. 0.74). UUO and vehicle-treated 8.85% (s.d. 7.08) versus UUO and ABT-263–treated 14.5% (s.d. 6.58). ANOVA, *P* = 0.921 95% CI: -4.2–6.75. Picrosirius red: uninjured and vehicle-treated 2.1% (s.d. 0.26), uninjured and ABT-263–treated 2.33% (s.d. 0.34). UUO and vehicle-treated 6.5% (s.d. 0.81) versus UUO and ABT-263–treated 15.6% (s.d. 3.08). ANOVA, *P* < 0.000 95% CI: 6.8–11.3. For box plots, the center line represents the mean, the box limits the first and third quartiles, the whiskers ± 1.5 × IQR, and the points all the data. Scale bar = 100 μm.

**Figure 2:**
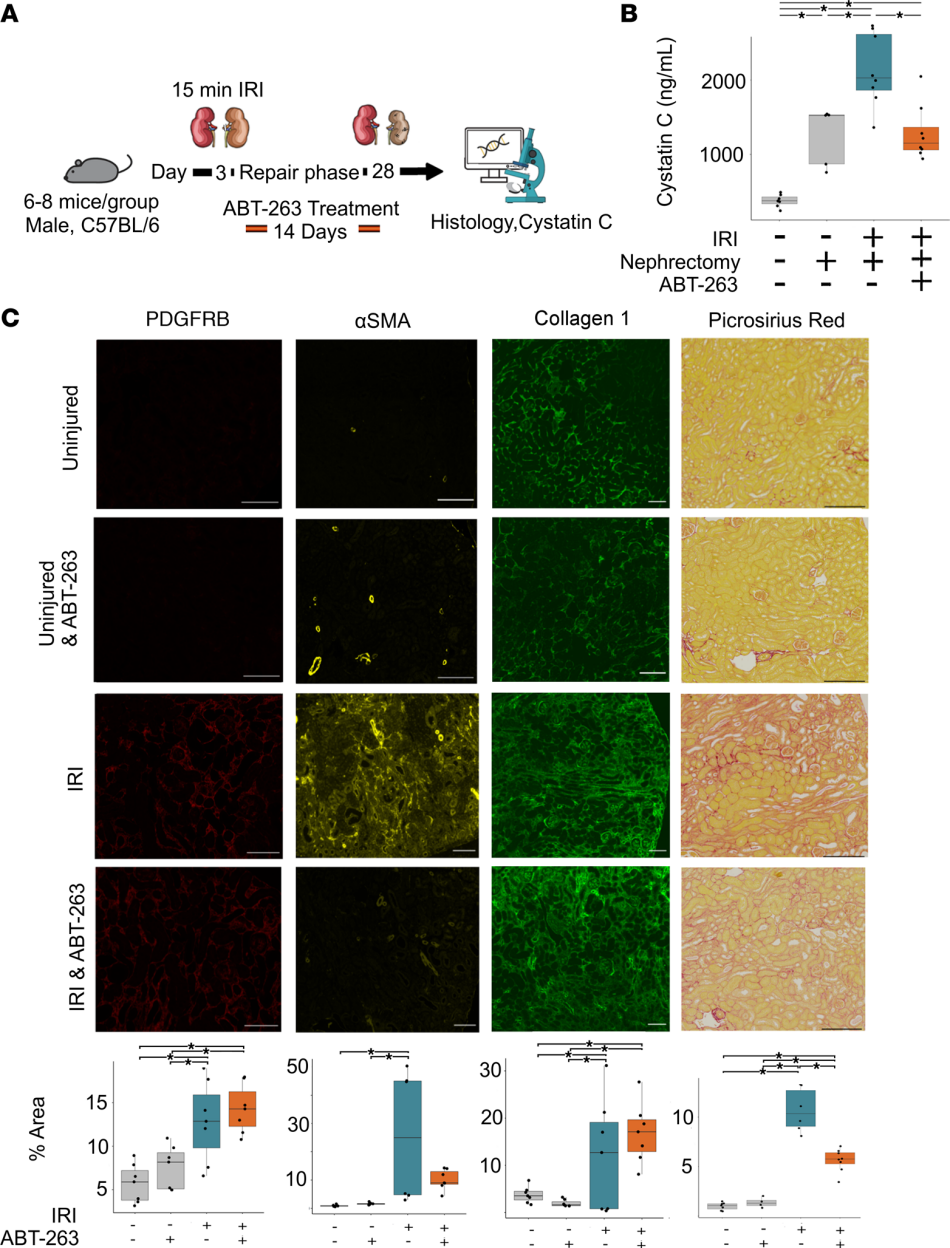
Senescent cell depletion following IRI results in decreased renal fibrosis. (**A**) Schematic of murine senescent cell depletion following IRI experiment, with samples taken at days 0 and 28. Note senolytic ABT-263 administration for 2 weeks following injury. *n* = 6–8 per group. (**B**) Cystatin C measurement (ng/mL) — baseline group, mean cystatin C 377 (s.d. 84), and postnephrectomy group, mean 1,244 (s.d. 395). IRI and vehicle treatment group, mean 2,136 (s.d. 491), versus IRI and ABT-263 treatment, mean 1,286 (s.d. 372), 2-sided *t* test, *P* = 0.0006, difference 850, CI: 315–1,384. (**C**) Representative images and quantification of immunofluorescence and of Picrosirius red staining of kidneys. Quantification of total staining per renal cortex. * denotes *P* < 0.05. PDGFRB: uninjured 5.69% (s.d. 2.23), uninjured and ABT-263–treated 7.53% (s.d. 2.48). IRI and vehicle-treated 12.8% (s.d. 4.66) versus IRI and ABT-263–treated 14.3% (s.d. 2.82). ANOVA, *P* = 0.921 95% CI: –4.24–6.7. α-SMA: uninjured 0.84% (s.d. 0.33). uninjured and ABT-263–treated 1.56% (s.d. 0.39). IRI and vehicle-treated 25.5% (s.d. 23.4) versus IRI and ABT-263–treated 10.1% (s.d. 3.56). ANOVA, *P* = 0.108 95% CI: –33.74–2.92. Collagen 1: uninjured and vehicle-treated 3.75% (s.d. 1.73), uninjured and ABT-263–treated 1.95% (s.d. 0.74). UUO and vehicle-treated 12% (s.d. 12) versus UUO and ABT-263–treated 16.9% (s.d. 6.39). ANOVA, *P* = 0.921 95% CI: –4.2–6.75. Picrosirius red: control and vehicle-treated 0.98% (s.d. 0.37), control and ABT-263–treated 1.35% (s.d. 0.51). IRI and vehicle-treated 10.6% (s.d. 2.2) versus IRI and ABT-263–treated 5.6% (s.d. 1.14), difference 5.06%, (CI: 3.1–7.01) *P* < 0.005. For box plots, the center line represents the mean, the box limits the first and third quartiles, the whiskers ± 1.5 × IQR, and the points all the data. Scale bar = 100 μm.

To explore the role of senescent cells in acute renal injury, we first depleted senescent cells using the senolytic drug ABT-263 following unilateral ureteric obstruction (UUO) in mice (Figure 1A). This is a persistent injury model where the insult remains present and tissue damage persists so long as the ureter remains obstructed. Administration of ABT-263 resulted in an increase in renal fibrosis following UUO injury measured by Picrosirius red and a decrease in quantitative PCR (qPCR) markers of senescence (Figure 1, B and C). Hypothesizing that senescence may thus be important in limiting ongoing injury, we next assessed whether senescent cell depletion following a single discrete injury would have a different effect. We administered ABT-263 to mice following ischemia/reperfusion injury (IRI) commencing at postinjury day 3 (Figure 2A). Administration of ABT-263 resulted in reduced kidney fibrosis and improved function by measured cystatin C clearance in kidneys after IRI (Figure 2, B and C). These results suggested senescence may be a desirable phenomenon during ongoing tissue injury but harmful during postinjury repair.

**Figure 3:**
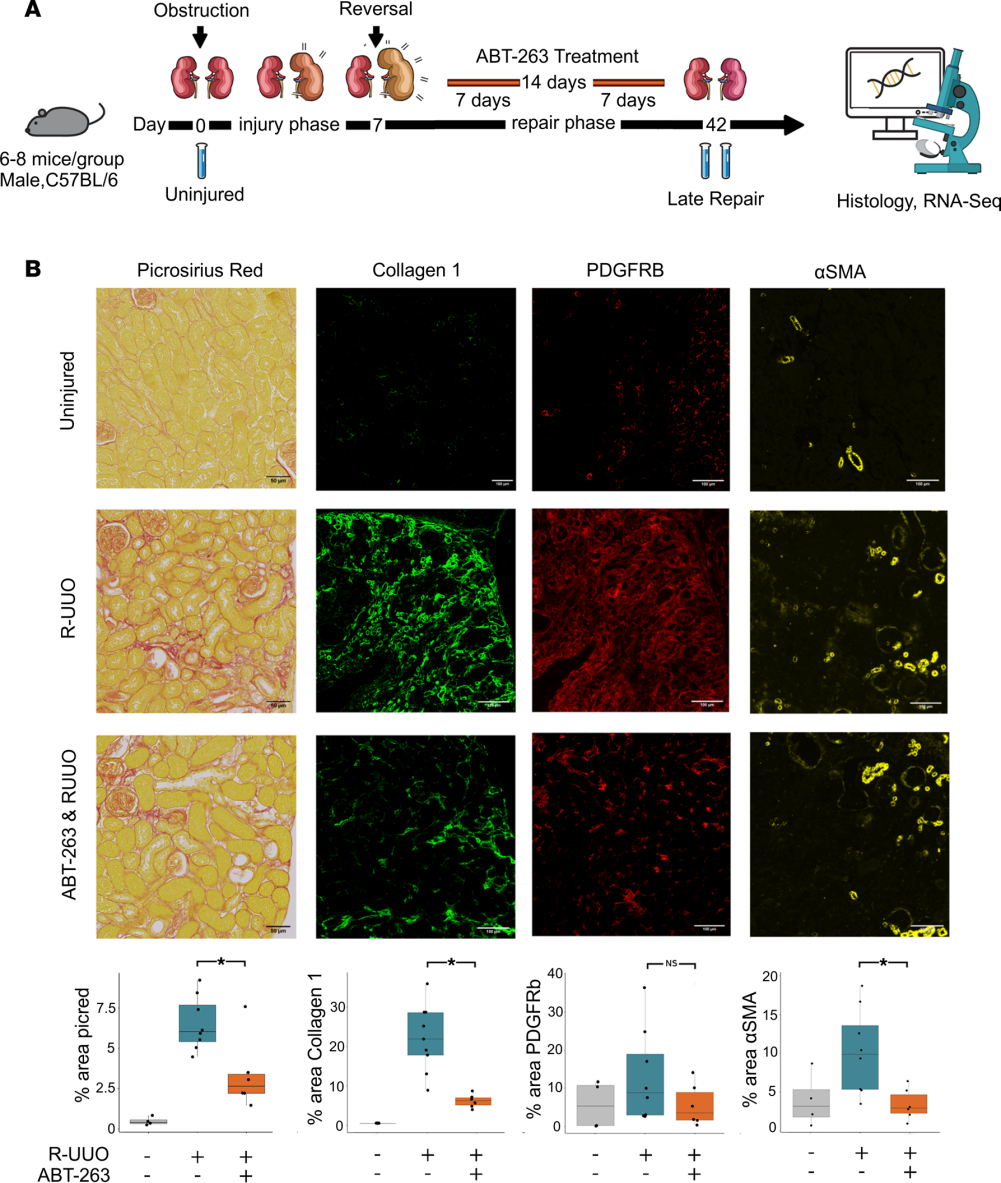
ABT-263 reduces fibrosis after R-UUO. (**A**) Schematic of murine R-UUO experiment, with samples taken at days 0, 14 (7 days following reversal), and 42 (35 days following reversal). Note senolytic ABT-263 administration for 14 days during the repair phase. *n* = 6–8 per group. (**B**) Representative images and quantification of Picrosirius red staining of kidneys and immunofluorescence of key proteins. Quantification of total staining per renal cortex. * denotes *P* < 0.05. Picrosirius Red: vehicle-treated 6.5% (s.d. 1.6) versus ABT-263–treated 3.3% (s.d. 2.2), 2-sided *t* test, *P* = 0.01, CI: 0.7–5.6. Collagen 1: vehicle-treated 5.7% (s.d. 1.6) versus ABT-263–treated 21.5% (s.d. 8.3), 2-sided *t* test, *P* = 0.0003, CI: 9.2–22.3. PDGFRB: vehicle-treated 13.1% (s.d. 12.2) versus ABT-263–treated 5.5% (s.d. 5.5), 2-sided *t* test, *P* = 0.1, CI: –3.3–18. α-SMA: vehicle-treated 10.2% (s.d. 5.5) versus ABT-263–treated 3.4% (s.d. 2) 2-sided *t* test, *P* = 0.01, 95% CI: 2–11.5.

To further explore whether senescent epithelial cells promote maladaptive repair and fibrosis following removal of an injurious stimulus, we used murine reversible unilateral ureteric obstruction (R-UUO), where a single ureter was surgically obstructed before reversal 7 days later. This experimental model is analogous to reversed obstructive uropathy in humans. To establish the role of senescence in maladaptive repair, ABT-263 was administered during the repair phase after injury, and effects were compared with those of a vehicle-treated group. Bulk RNA-Seq was performed on uninjured kidneys and 35 days after reversal (day 42 in Figure 3A) to identify the transcriptomic pathways that might prevent full repair and the effect of senolytic treatment.

**Figure 4:**
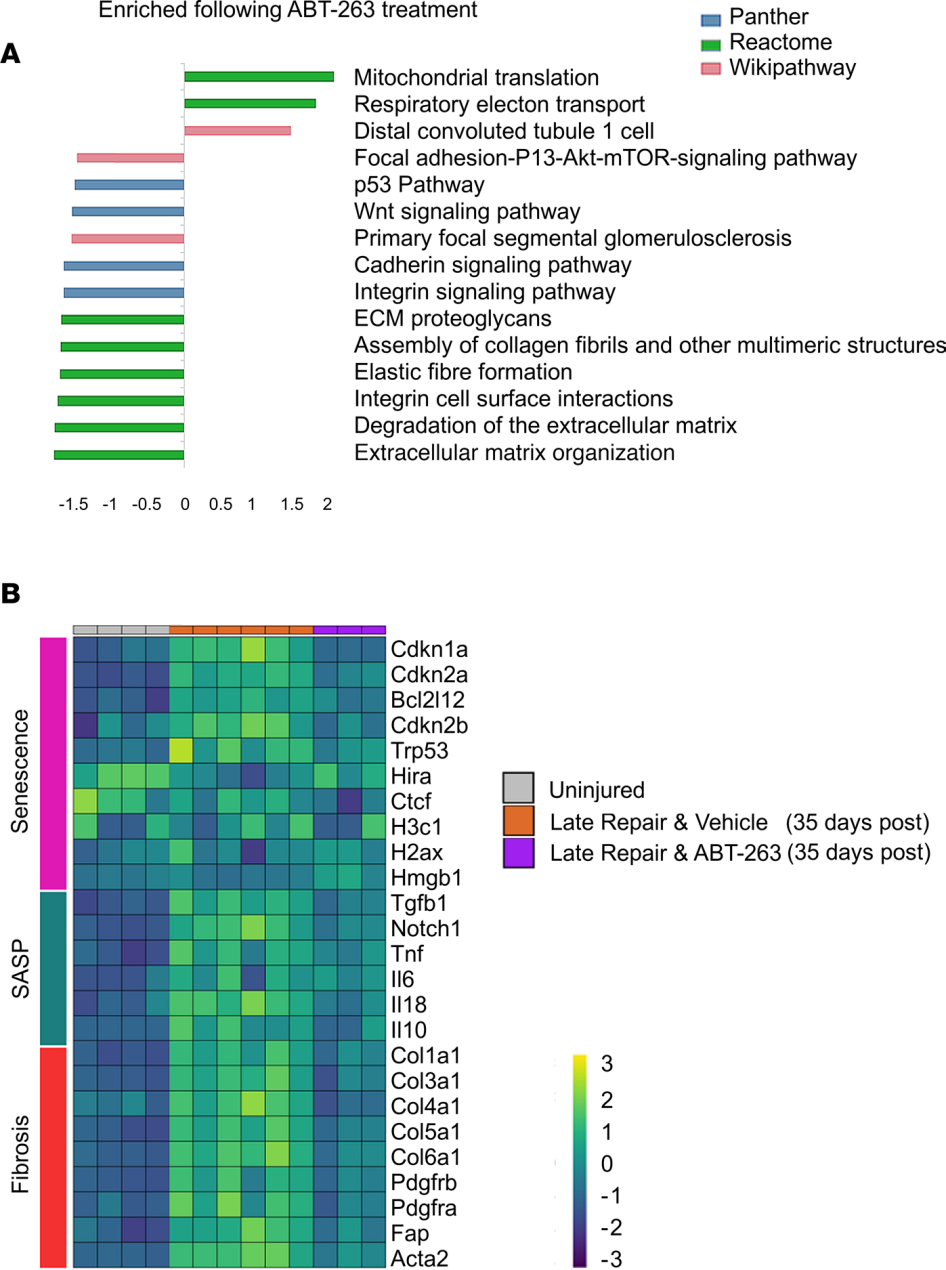
ABT-263 alters the renal transcriptome by depleting senescent cells after R-UUO. (**A**) Gene set enrichment analysis (GSEA) of differentially expressed genes (DEGs) between ABT-263–treated animals and vehicle-treated controls at 35 days after reversal. All FDR values for all pathways shown are <0.05. GSEA performed on WebGestalt. (**B**) Heatmap showing senescence-associated and fibrosis-associated genes are persistent during repair, which return toward baseline following administration of ABT-263. The color scheme is based on *z* score distribution. All genes during late repair are significant DEGs (*q* < 0.1) as compared with uninjured group. ABT-263–treated genes compared with vehicle-treated were all were significant DEGs (*q* < 0.1) except *H3c1*, *H2ax*, *Hmgb1*, *Il6*, and *Il10*.

Mice treated with ABT-263 had significantly less cortical fibrosis, as measured by Picrosirius red staining and collagen I immunofluorescence, and reduction of fibroblast activation, by α-SMA immunofluorescence (Figure 3B). ABT-263 resulted in a decrease in senescent cells as measured by senescence-associated β-galactosidase (SA-β-gal) staining and cyclin-dependent kinase inhibitor p21^Cip1+^ tubules (Supplemental Figure 1, A–C; supplemental material available online with this article; https://doi.org/10.1172/jci.insight.154124DS1). There were marked transcriptional changes during repair and following ABT-263 treatment (Figure 4A). GSEA revealed pathways associated with persistent inflammation and fibrosis, including interleukin signaling and extracellular matrix reorganization, remained persistently elevated after reversal of obstruction (Figure 4A). At day 35 after reversal, mice treated with ABT-263 had a significant downregulation of pathways associated with senescence, fibrosis, and the SASP, including the p53 pathway, extracellular matrix organization, and elastic fibre formation, when compared with vehicle-treated mice (Figure 4B and Supplemental Table 1).

Taken together, these data indicated that senescent epithelia persisting in the aftermath of injury promote maladaptive repair, and the depletion of senescent cells by ABT-263 in this setting reduces subsequent renal fibrosis and mesenchymal activation following deobstruction.

### Senescent epithelial cells are associated with postinjury fibrosis in human kidneys.

**Figure 5:**
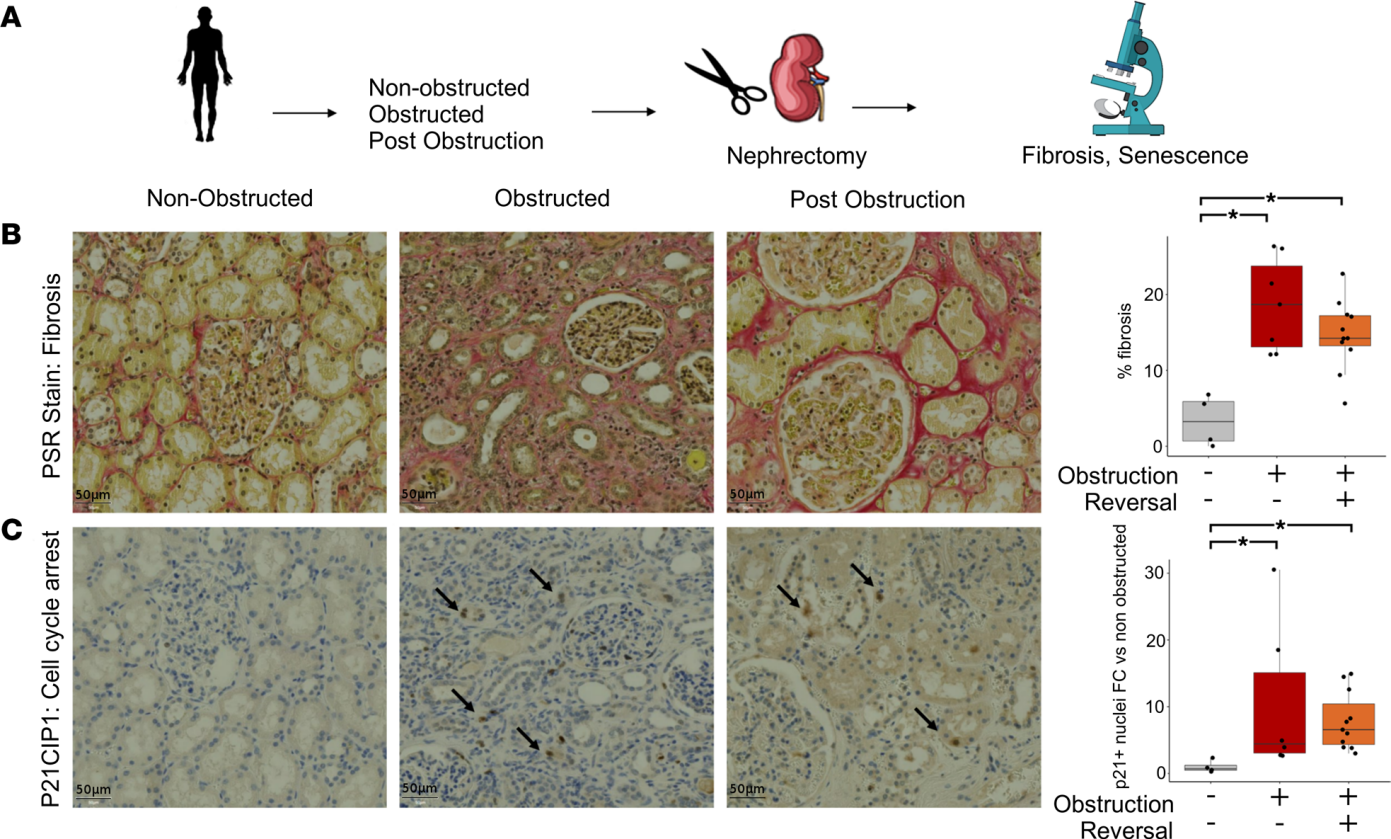
Senescent cells persist following renal injury with subsequent maladaptive repair. (**A**) Schematic of human kidney sample retrieval (*n* = 22). (**B**) Human kidney fibrosis. Red staining indicates Picrosirius red staining of collagen networks. * denotes significance at *P* < 0.05. Uninjured 3.3% (s.d. 3.3) versus obstruction 18.6% (s.d. 6.1) versus after obstruction 14% (s.d. 4.5), ANOVA, uninjured versus obstructed, *P* = 0.002 CI 7.4–23.3, uninjured versus after obstruction *P* = 0.002 CI 3.9–18.7, obstruction versus after obstruction *P* = 0.6 CI –10.14–2.11. For all box plots, the center line represents the mean, the box limits the first and third quartiles, and the whiskers are ± 1.5 × IQR. Scale bar = 50 μm. (**C**) Human kidney senescence. P21^CIP1^ staining demonstrating senescent epithelial cells. Arrows point to P21^CIP1+^ nuclei. *Y* axis shows fold change of senescent tubules per kidney relative to the mean number of senescent tubules in the uninjured control group. Uninjured 0.9 (s.d. 0.9) versus obstruction 10.5 (s.d. 11.4) versus after obstruction 7.8 (s.d. 4.3), Kruskal-Wallis rank sum test, uninjured versus obstructed, *P* = 0.009 CI 1.5–29.6, uninjured versus after obstruction *P* = 0.001 CI 2.7–12.5, obstruction versus after obstruction *P* = 0.2 CI: –5.5–14.5.

Having observed persistence of senescence in murine epithelia following injury, we next examined whether senescent cells induced during a similar obstructive kidney injury also persisted in humans despite resolution of injury. To do this, we utilized the nontumorous portion of tumor nephrectomy specimens from 3 cohorts of patients; 1) kidneys with no evidence of obstruction, 2) persistently obstructed kidneys, and 3) previously obstructed kidneys where obstruction had been relieved by prior placement of a ureteric stent (Figure 5A). Patients who had persistent obstruction had increased levels of fibrosis and expression of P21^CIP1^ (typically upregulated in senescence) (Figure 5, B and C). Notably, P21^CIP1^ expression and fibrosis levels did not return to baseline after obstruction, suggesting persistent senescence may be present alongside maladaptive repair even after relief of obstruction (Figure 5, B and C).

### Single-cell RNA-Seq allows analysis of senescent cells in vivo.

**Figure 6:**
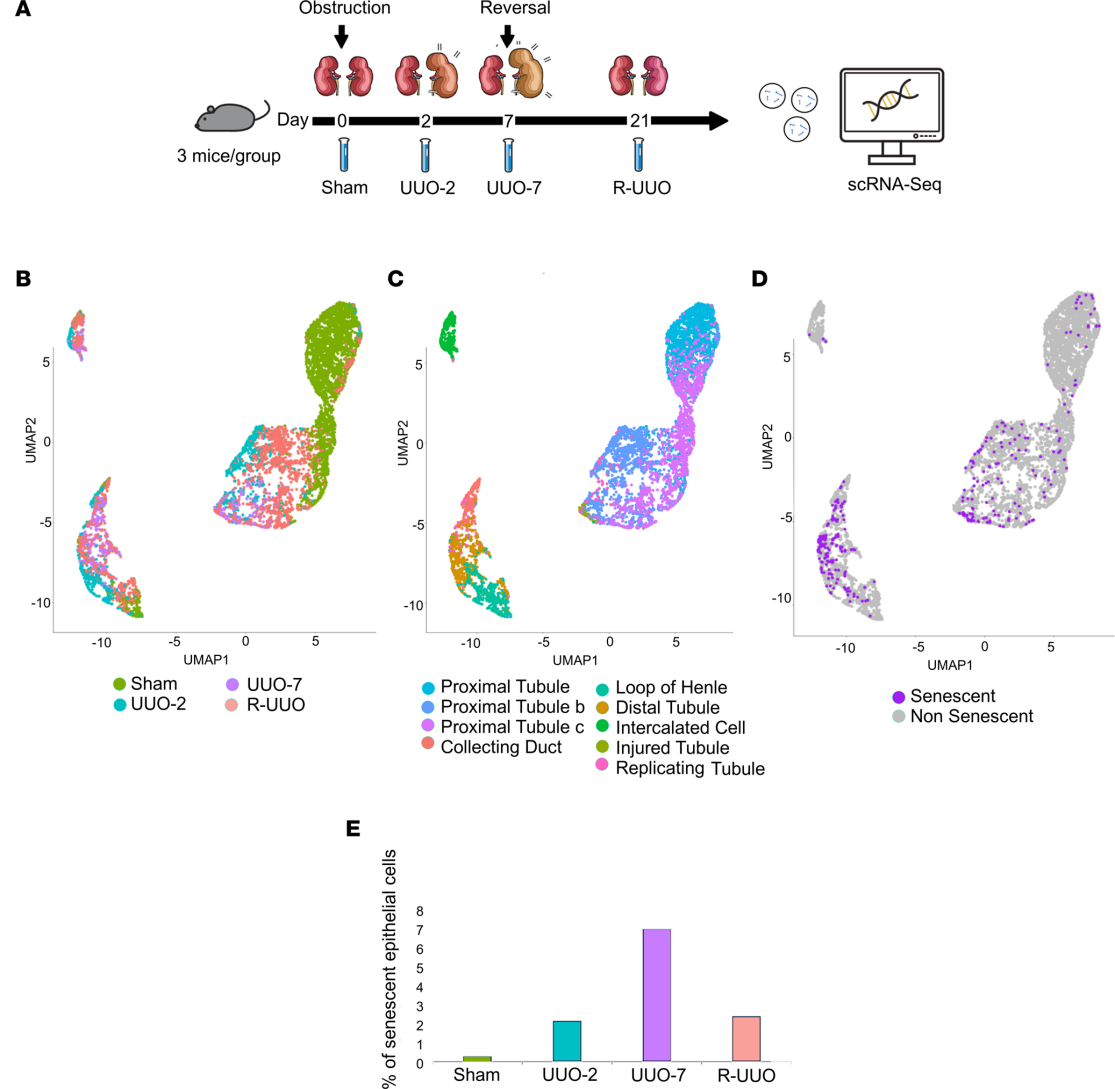
Senescent cells can be identified using single-cell RNA-Seq. (**A**) Schematic of single-cell RNA-Seq R-UUO experimental workflow. Uniform manifold approximation and projection (UMAP) plots of 7,392 epithelial cells colored by (**B**) injury time point, (**C**) unbiased cell cluster, and (**D**) senescent status (320 cells). (**E**) Percentage of epithelial cells classified as senescent at each injury time point. The proportion of epithelial cells that are senescent increases over time following injury and decreases during repair but does not return to baseline.

To better understand how senescent cell persistence inhibits repair and promotes fibrosis, we performed subsequent studies using single-cell RNA-Seq. Single-cell libraries were prepared using whole-kidney digests of 3 pooled mice per library at multiple time points: day 2 after UUO, day 7 post-UUO, and 14 days post-R-UUO (Figure 6A). This data set analyzing the myeloid compartment was recently published (19). All cells can be explored via our online atlas: http://www.ruuo-kidney-gene-atlas.com As we have previously described the varied cell types during R-UUO in detail, and as senescent renal epithelia are implicated in the induction of fibrosis after injury as discussed, we focused this analysis on the epithelial compartment.

Epithelial cells were first identified following unsupervised clustering and classification before epithelial subtypes (e.g., proximal tubule, loop of Henle) were classified based on characteristic marker genes (Figure 6, B and C; Supplemental Figure 1, D and E; and Supplemental Figure 2, A and B). An “injured tubule” cluster was identified and uniquely characterized by elevated injury marker *Havcr1* (KIM-1), which was transcriptomically distinct from other clusters (Figure 6C). Next, to classify a subgroup as senescent, we overpartitioned the epithelia into multiple clusters. A single cluster was identified expressing high *Cdkn1a* and an absence of cells in S phase — and was thus classified as senescent. These cells demonstrated upregulation of typical senescent pathways (SASP genes, TGF-β and Notch signaling) (Supplemental Figures 3 and 4).

In total, 322 of the 7,392 (4.2%) epithelial cells were classified as senescent (Figure 6D). Senescent cells had a markedly different transcriptome, accumulated over time following injury, and decreased following deobstruction but did not return to baseline, consistent with our earlier observations in postobstruction human kidneys (Figure 6E and Supplemental Table 2). Senescent cells were most frequently found among distal tubular cells, consistent with previous observations in human renal senescence (13).

### Senescent cells exhibit a profibrotic, proinflammatory transcriptome.

**Figure 7:**
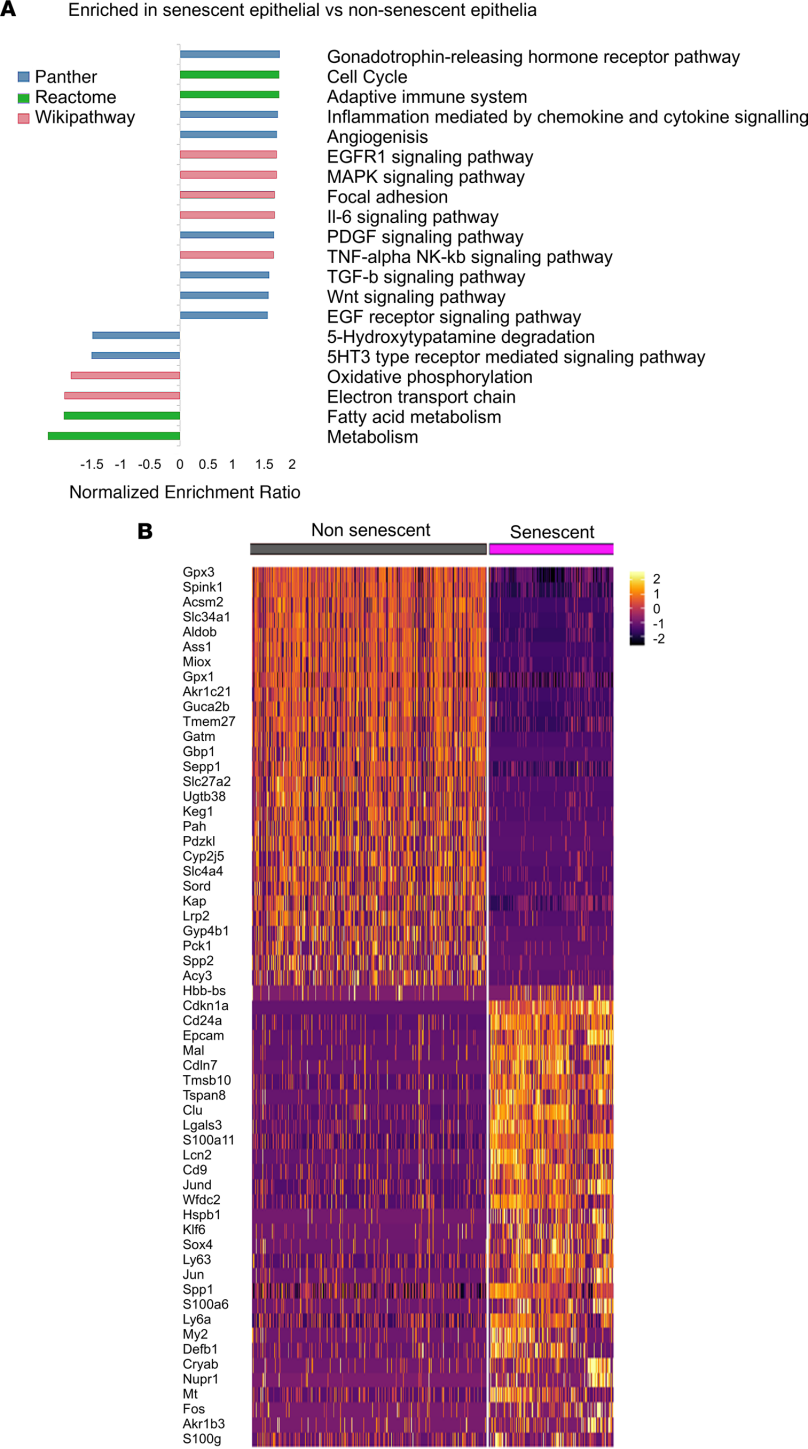
Senescent cells exhibit a proinflammatory, profibrotic transcriptome. (**A**) GSEA between senescent and nonsenescent epithelial cells. Selected pathways demonstrate consistency with known senescent signaling pathways. All FDR values for pathways shown are <0.05. GSEA scores generated with WebGestalt as described in Supplemental Methods. (**B**) Heatmap of top DEGs between non/senescent epithelial cells, calculated using Wilcoxon signed-rank test. The color scheme is based on *z* score distribution.

When compared with nonsenescent epithelial cells, the first notable pattern among the DEGs in senescent epithelial cells was the loss of normal tubular function (Figure 7, A and B). This apparent “dedifferentiation” remained when comparing senescent cells only with the distal tubular cell cluster, indicating that this phenomenon was not due to any senescent cell bias toward the distal tubule or transcriptomic differences along the tubule. Senescent cells expressed typical SASP components as well as being enriched for transcripts of *Notch* and *Tgf* signaling pathways (Supplemental Figure 4) (20, 21).

GSEA showed enrichment for multiple relevant ontologies (Figure 7A), including IL-6 signaling, an important component of the SASP (22, 23); TGF-β signaling, an inducer of senescence and renal fibrosis; WNT signaling, crucial in cell fate decisions by regulating p53 (24); and PDGF signaling, important in myofibroblast differentiation and underpinning senescence in healthy wound healing (5, 25–27).

To validate our transcriptomic signature in other data sets, we used a machine learning classifier, which we trained on our signature and then used to classify cells in 3 public data sets (28). Senescent cells were classified in publicly available data sets: 2 IRI models and “tabula muris senis,” which includes aged mice. We successfully identified senescent cells in all these data and found similar enriched pathways in all cases (Supplemental Figure 5). To corroborate our findings, we compared the DEGs in our senescent epithelial cells with the proteome of senescent human cortical renal epithelial cells in the SASP Atlas (29). We found 363/860 (42%) of the proteins found in the SASP Atlas appeared as DEGs in our senescent epithelial cell DEG list. These data support the hypothesis that senescent epithelial cells produce a profibrotic, inflammatory SASP. Next, we used 2 analytical approaches to identify candidate profibrotic molecules for further validation.

### Single-cell RNA-Seq reveals conserved senescent gene expression between organs and species.

**Figure 8:**
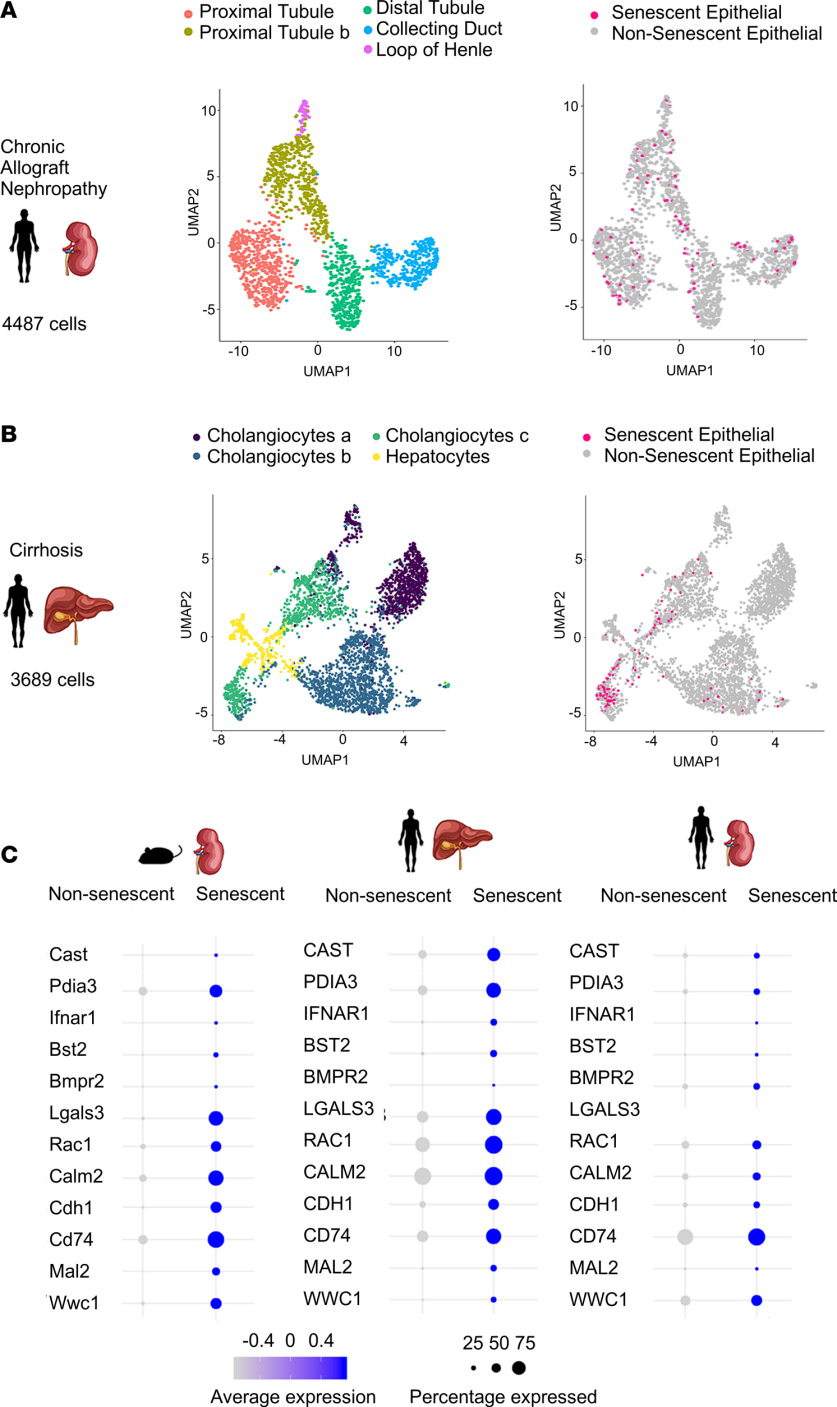
Conserved senescent transcriptome across species and organ. (**A**) UMAP of 3,869 epithelial cells from human cirrhotic livers, colored by classification from original paper (31), and senescent status. (**B**) UMAP of 1,692 epithelial cells from a patient with chronic allograft nephropathy colored by cell type classification and senescent status. (**C**) Expression of DEGs in senescent epithelial cells, which were consistent across species and organs. Size of dot represents the percentage of cells in each cluster expressing the gene; color indicates average gene expression per cell.

First, considering senescence is a highly conserved biological cell phenotype, we reasoned that DEGs in senescent epithelial cells that are observed across species and organs may be important pathogenic molecules representing good candidates for further interrogation as therapeutic targets. We therefore reanalyzed single-cell libraries from a human kidney transplant from a 70-year-old donor where the patient developed chronic allograft nephropathy, as we anticipated this would include chronic senescent cells because of both donor age and ongoing disease (30). Of the 4,487 epithelial cells in the single-cell RNA-Seq data set, 114 cells (2.5%) were classified as senescent (Figure 8A). Next, we explored recently published single-cell RNA-Seq from cirrhotic human livers and matched control tissue, and we used the same approach to senescent cell identification described above (31). Of the 3,689 epithelial cells in this data set, 134 senescent epithelial cells were identified (3.5% total), 130 of which were derived from the cirrhotic library and 4 from the healthy liver (Figure 8B). Importantly, several senescent epithelial DEGs were found to be shared across both human data sets and our murine data set (Figure 8C).

### Single-cell RNA-Seq identifies candidate ligand-receptor interactions between senescent epithelia and fibroblasts during repair.

**Figure 9:**
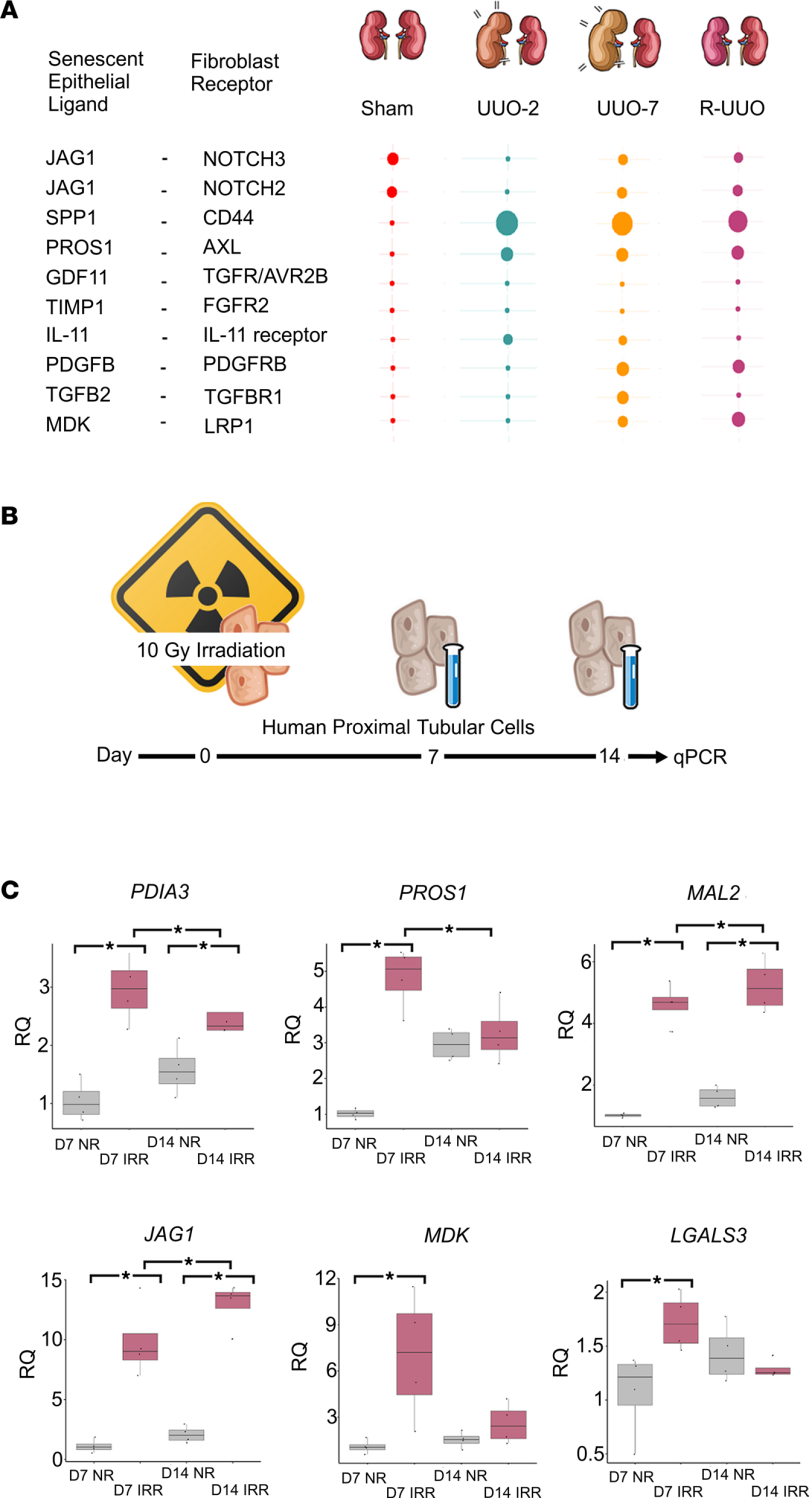
Ligand-receptor analysis allows identification of potential therapeutic targets. (**A**) Top senescent ligand–mesenchymal receptor pairs across the time course of the reversible ureteric obstruction model. Size of dot is proportional to mean expression values for all the interacting partners. All interactions are significant with *P* < 0.05. *P* value refers to the enrichment of the interacting ligand-receptor pair in each of the interacting pairs of cell types. (**B**) Experimental workflow for inducing senescence in irradiated human proximal tubular cells. (**C**) Irradiation-induced senescence in human proximal tubular cells increases key ligand transcription. ANOVA test used for significance testing, * denotes *P* < 0.05. For box plots, the center line represents the mean, the box limits the first and third quartiles, the whiskers ± 1.5 × IQR, and the points all the data. IRR, irradiated; NR, no irradiation.

Second, based on our earlier data, which demonstrated persistent mesenchymal activation during maladaptive repair with enriched pathways in senescent epithelia including TGF-β and PDGF-β (Figure 7A), we analyzed senescent epithelia-myofibroblast crosstalk using ligand-receptor pair analysis (32). We found that senescent epithelial cell ligand-fibroblast receptor interactions varied over the course of injury and repair (Figure 9A, myofibroblast classification described in Supplemental Figure 6).

Combining our data from a) conserved DEGs in multiorgan and species senescence and b) ligand-receptor analysis, we selected 7 candidate molecules for further analysis (Supplemental Table 3).

### Candidate profibrotic mediators are upregulated by senescent human proximal tubular epithelial cells in vitro and in CKD in vivo.

To confirm that these molecules were produced in excess by senescent epithelial cells as suggested by the in silico data, we induced senescence in human proximal tubular epithelial cells (hPTECs) using irradiation, an established model of senescence induction (Figure 9B). At day 7 following irradiation, compared to nonirradiated, time matched controls, hPTECs showed increased levels of all candidate molecules as measured by qPCR, with *PDIA3*, *MAL2*, and *JAG1* transcripts remaining elevated above nonirradiated controls at day 14 (Figure 9C).

Staining of human protein atlas samples (age range 26–70) demonstrated that our candidate molecules were found in human tubular epithelium and upregulated in human CKD (Supplemental Figure 7A), with PDIA3 showing the strongest correlation (fold change > 2, *P* = 4.4 × 10^–19^ vs. healthy control). PDIA3 is a thiol oxidoreductase known to be induced by endoplasmic reticulum (ER) stress and capable of mediating cellular responses to oxidative stress. PDIA3 enhancement was not restricted to senescent cells but enriched in other models of stress and injury, with upregulation also found in other tubular cells after murine UUO and in tubular cells of diabetic patients as compared with healthy controls as well as in a proteomic atlas of senescent cells’ supernatant (Supplemental Figure 7, B and C).

### Exogenous PDIA3 augments fibroblast proliferation and SMAD2 phosphorylation in response to TGF-β1 in vitro.

**Figure 10:**
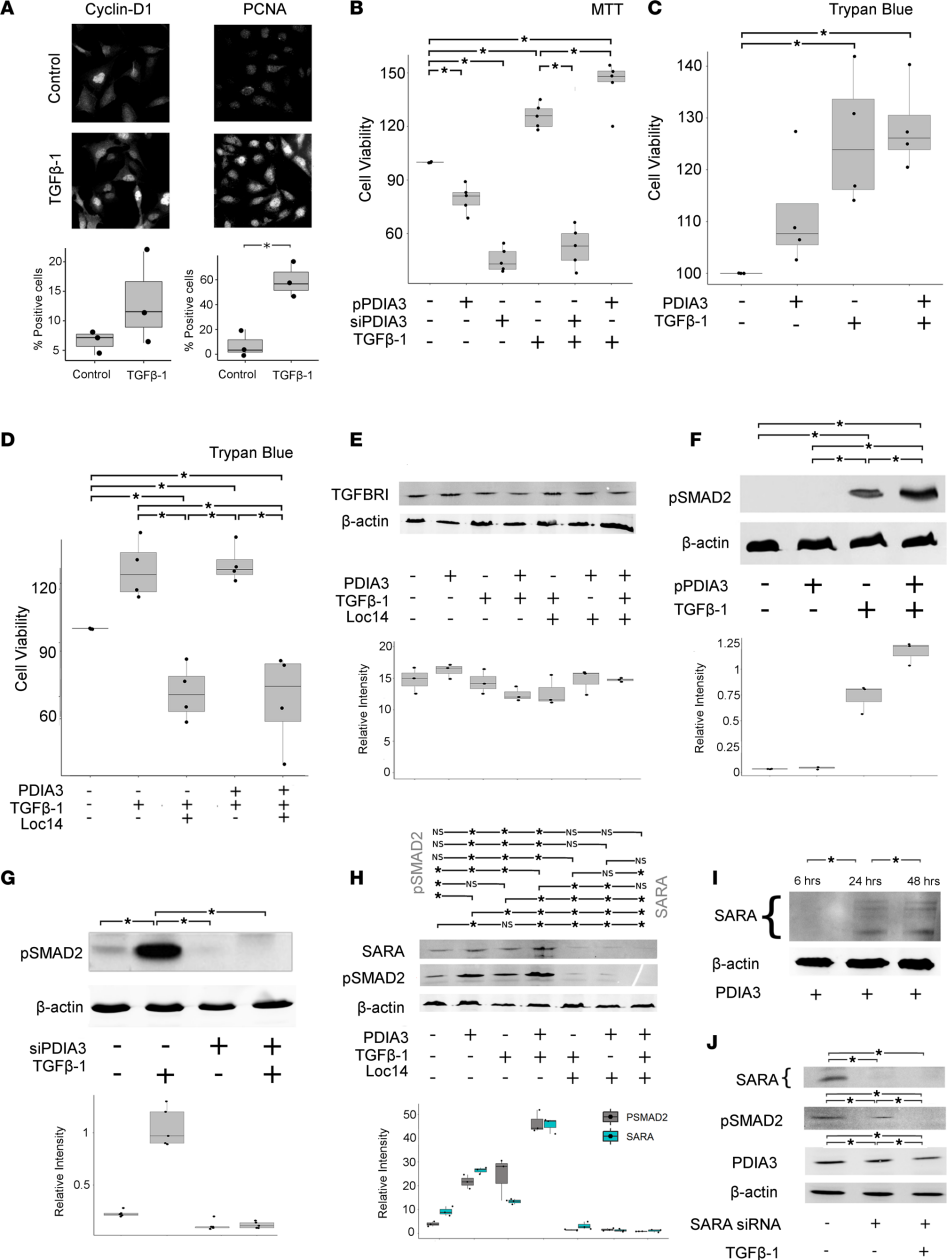
Increased expression of PDIA3 enhances TGF-β1–driven fibroblast proliferation in vitro. Full figure legend with more statistical data available in Supplemental Data 1. (**A**) Increased levels of cell cycle proteins cyclin D1 and PCNA in renal fibroblasts in response to TGFB1 administration. *P* values generated by *t* test. (**B**) MTT assay readout normalized to control group demonstrating that dual treatment with pPDIA3 and TGF-β1 results in additional proliferation compared with single TGF-β1. One-way ANOVA with Bonferroni’s correction. (**C**) Trypan blue assay readout normalized to control group demonstrating treatment with PDIA3 and/or TGF-β1 results in additional proliferation compared with control. One-way ANOVA with Bonferroni’s correction. (**D**) Trypan blue assay readout normalized to control group demonstrating treatment with Loc14 reduces cell viability. One-way ANOVA with Bonferroni’s correction. (**E**) Coadministration of PDIA3, TGFB1, and Loc14 to human renal fibroblasts does not alter TGFBRI synthesis. Comparisons tested using 1-way ANOVA with Bonferroni’s correction. No comparisons were statistically significant at *P* adj < 0.05. (**F**) Coadministration of pPDIA3 to TGFB1-treated fibroblasts increases p-SMAD2 synthesis. Comparisons tested using 1-way ANOVA with Bonferroni’s correction. (**G**) Coadministration of siRNA to TGFB1-treated fibroblasts reduces p-SMAD2 synthesis. Comparisons tested using 1-way ANOVA with Bonferroni’s correction. (**H**) Coadministration of PDIA3 and TGFB1 to human renal fibroblasts increases SARA and p-SMAD2 synthesis. Dual treatment with PDIA3 and TGFB1 results in a greater increase compared with either alone. This effect was abolished by Loc14 administration. Comparisons tested using 1-way ANOVA with Bonferroni’s correction. (**I**) Time course experiment showing increased SARA protein over time following PDIA3 administration, first detectable at 6 hours after administration. One-way ANOVA with Bonferroni’s correction. (**J**) siRNA to SARA results in reduced levels of p-SMAD2, such that in the presence of siRNA to SARA, TGF-β1 no longer induces p-SMAD2 production in fibroblasts. One-way ANOVA with Bonferroni’s correction. *P < 0.05.

PDIA3 was selected for further study on the basis of its upregulation in the proteomic SASP Atlas (29), in human CKD, our in vitro senescence model, and human and murine senescent cells in vivo. In the kidney, fibroblasts are the key cell responsible for matrix deposition and fibrosis and were therefore identified as the most important downstream target of epithelial PDIA3 during maladaptive repair. However, treatment of fibroblasts with recombinant PDIA3 alone did not result in activation (Supplemental Figure 7G). To test whether PDIA3 augmented the fibroblast response to known activating pathways, we first administered TGF-β1 to TK188 fibroblasts, which resulted in increased levels of cell cycle proteins cyclin D1 and proliferating cell nuclear antigen (PCNA) (Figure 10A). We next used siRNA (siPDIA3) and a *PDIA3* overexpression plasmid (pPDIA3) to knock down or overexpress PDIA3 in human TK188 fibroblasts prior to activating them with TGF-β1 (Figure 10, A and B, and Supplemental Figure 7D). Together, these results showed that TGF-β1 increased fibroblast viability, and this effect was augmented by pPDIA3. In combination, TGF-β1 and pPDIA3 resulted in increased numbers of viable fibroblasts in this assay and on further replication using trypan blue to assess cell counts (Figure 10, B and C). There was a loss of fibroblast response to TGF-β following the silencing of *PDIA3* by siPDIA3 (Figure 10B) and after administration of the pharmacological PDIA3 antagonist Loc14 (Figure 10D).

Next, to test whether this enhanced effect of TGF-β was due to PDIA3 converting latent TGF-β to its active form, an active TGF-β immunoassay was used to test supernatant from renal epithelial cells incubated with latent TGF-β with and without PDIA3 and Loc14, a PDIA3 inhibitor. PDIA3 did not increase free active TGF-β (Supplemental Figure 7E). Similarly, Western blotting demonstrated that total levels of TGF-β receptor 1 (TGFBR1) remained unaltered by Loc14 (Figure 10E). To test the impact of PDIA3 on canonical TGF-β signaling downstream of TGF-β receptor ligation, Western blot analysis of phosphorylated SMAD2 (p-SMAD2) was undertaken. In response to silencing of *PDIA3* during TGF-β treatment, there was diminished phosphorylation of SMAD2 protein, which is required for TGF-β1 signal transduction. Conversely there was increased phosphorylation of SMAD2 when pPDIA3 was given in combination with TGF-β1 (Figure 10, F and G). Further Western blotting demonstrated that PDIA3 administration significantly increased expression levels of Smad anchor for receptor activation (SARA), a cofactor essential for TGF-β–induced Smad2 activation, with levels of SARA potently inhibited by Loc14 treatment (Figure 10H). We next undertook a time course examining levels of SARA at 6, 24, and 48 hours after PDIA3 administration. This demonstrated marked induction from the 6- to 24-hour time point, with little further induction thereafter (Figure 10I).

Finally, we used an additional genetic strategy involving siRNA to inhibit SARA expression (Figure 10J), demonstrating that siRNA inhibition of SARA resulted in reduced levels of p-SMAD2, and furthermore, that in the presence of siRNA inhibition of SARA, TGF-β1 no longer induced p-SMAD2 production in fibroblasts.

Collectively, these data demonstrate that exogenous PDIA3 augments the canonical activation of fibroblasts by the prototypic profibrotic cytokine TGF-β, via intracellular effects on SARA levels, with inhibition of PDIA3 or SARA inhibiting TGF-β–induced activation in vitro.

### Targeted inhibition of PDIA3 inhibits fibroblast proliferation and fibrosis deposition in vivo.

We tested PDIA3 inhibition in vivo in mice during renal injury and repair. An orally bioavailable inhibitor of PDIA3, Loc14, was delivered by gavage to mice following IRI, UUO, and R-UUO.

**Figure 11:**
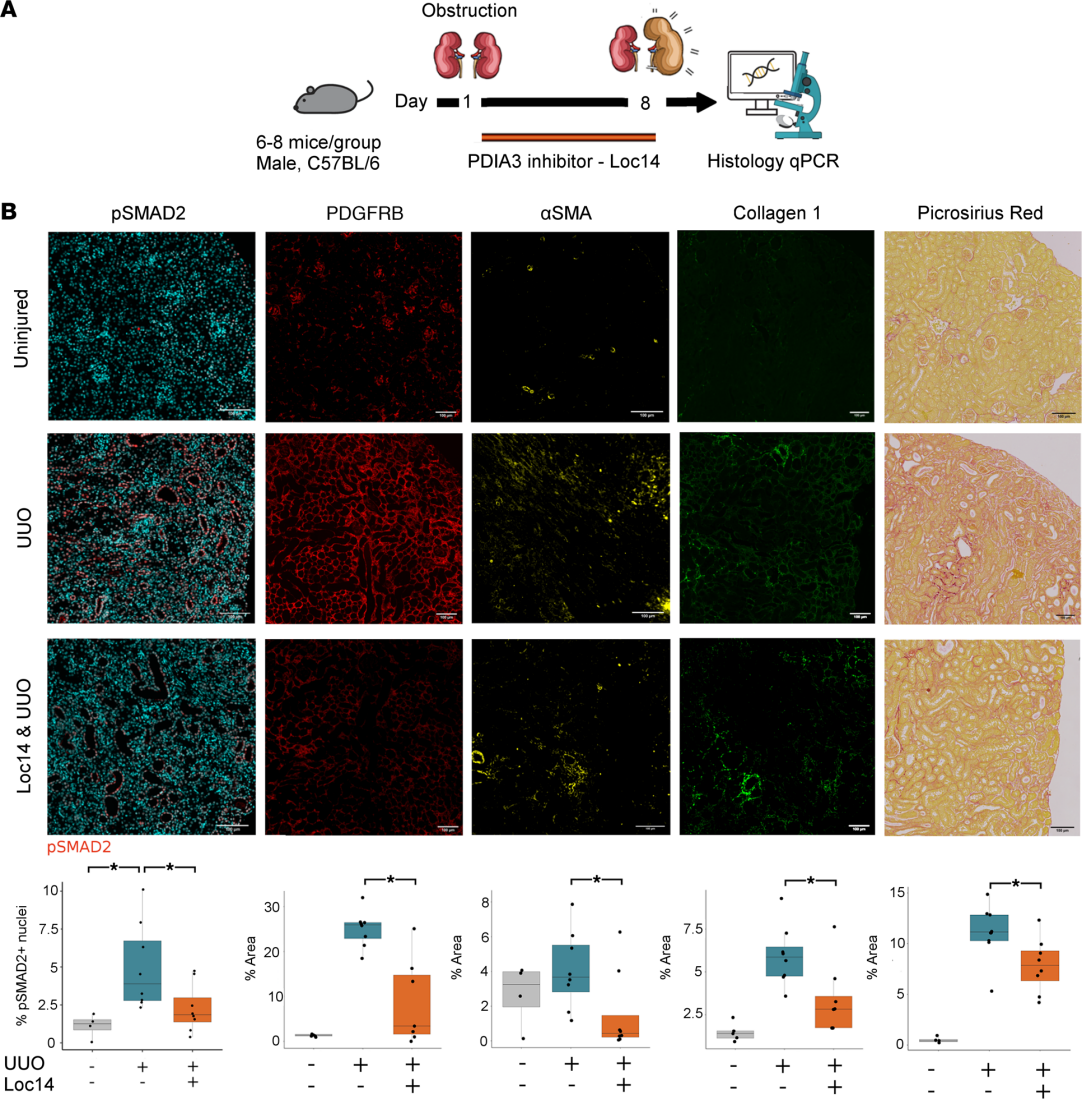
Inhibition of PDIA3 reduces SMAD2 phosphorylation, fibroblast proliferation, and fibrosis deposition after UUO in vivo. (**A**) Experimental workflow for inhibition of PDIA3 following UUO. (**B**) Representative images and quantification of immunofluorescence and of Picrosirius red staining of kidneys. Quantification of total staining per renal cortex. * denotes *P* < 0.05. Loc14 administration results in less p-SMAD2 activation in vivo after UUO in mice. Mean% staining in uninjured group 1.89% (s.d. 0.7) versus vehicle-treated after UUO 4.99% (s.d. 2.8), ANOVA, *P* adj = 0.02, CI: 0.5–7.2. vehicle-treated 4.99% (s.d. 2.8) versus Loc14-treated 2.27% (s.d. 1.6), ANOVA, *P* adj = 0.05, 95% CI: –5.44–0.010. Picrosirius red: 11% (s.d. 2.8) in vehicle-treated versus 7.8% (s.d. 2.6) in Loc14-treated mice, 2-sided *t* test, *P* = 0.03, 95% CI: 0.2–6.18. Collagen 1: 3.2% (s.d. 2) in vehicle-treated versus 5.9 % (s.d. 1.7) in Loc14-treated, Wilcoxon rank sum test, *P* = 0.01, CI 1:4.4. Pdgfrb: 25% (s.d. 4) in vehicle-treated versus 8.7% (s.d. 4) in Loc14-treated mice, 2-sided *t* test, *P* = 0.003, 95% CI: 7.2–25. α-SMA: 4% in vehicle-treated (s.d. 2.2) versus 1.5% in Loc14-treated mice (s.d. 2.3), 2-sample Wilcoxon test, *P* = 0.037, CI 0.6–5.2. For box plots, the center line represents the mean, the box limits the first and third quartiles, the whiskers ± 1.5 × IQR and the points all the data. Scale bar = 100 μm.

Following R-UUO, treatment with Loc14 resulted in less collagen 1 by immunofluorescence but a statistically nonsignificant decrease in fibrosis measured by Picrosirius red as compared with vehicle-treated animals (Supplemental Figure 8, A and B). However, mice treated with Loc14 during UUO showed significantly less renal fibrosis (Figure 11). Consistent with our in vitro findings, these mice showed significantly less p-SMAD2 staining and less fibroblast proliferation and activation as measured by PDGFR-β and α-SMA immunofluorescence (Figure 11). We quantified the nuclei in the dual α-SMA^+^PDGFR-β^+^ cells and found a statistically significant increase in the numbers of such cells following injury but no difference in the number of cells between Loc14-treated and vehicle-treated animals. This would suggest an effect of Loc14 is to reduce myofibroblast activity and expansion rather than number of cells during acute injury (Supplemental Figure 7H). This resulted in less fibrosis on Picrosirius red staining and collagen 1 by immunofluorescence (Figure 11B). No toxicity or alteration in levels of renal fibrosis was seen in uninjured control kidneys treated with Loc14 versus vehicle (Supplemental Figure 7F), and inhibition did not affect senescent cell number, as measured by *Cdkn1a* or *Cdkn2a* via qPCR or P21^CIP1^ expression on immunofluorescence (Supplemental Figure 8, C and D).

Collectively, these data indicate that PDIA3 produced in senescent cells (and other cells undergoing ER stress) facilitates TGF-β–mediated fibroblast proliferation in vitro and in vivo via phosphorylation of SMAD2. The effect of its inhibition is context and injury dependent and can reduce fibroblast numbers and collagen production.

## Discussion

The role of persistent senescence in the aftermath of acute injury in facilitating or opposing complete tissue repair remains poorly understood. We show that senescent cells persist in deobstructed kidneys in experimental and human renal disease and after experimental IRI where they oppose successful renal repair. The effect of experimental depletion of senescent cells is context dependent and during the repair phase reduces postinjury fibrosis. Using single-cell analysis of murine and human kidneys and human fibrotic liver disease, we identify factors generated by senescent epithelial cells not previously associated with the SASP to our knowledge. We characterize one such mediator, PDIA3, as a major contributor to renal fibrosis via its augmentation of TGF-β–driven activation of myofibroblasts. We then demonstrate the potential antifibrotic efficacy of its targeted inhibition in vivo during ongoing injury.

We provide in silico and in vitro evidence that senescent epithelial cells activate fibroblasts and identify new proteins, which may present future therapeutic targets, validating PDIA3 as an example. We hypothesized PDIA3 plays a central role in cell stress–associated fibrosis, which would unify past observations made of the association between PDIA3 and fibrosis in disease settings (33–36). PDIA3 has a number of functions that could be linked to a variety of stressors, including oxidative, nutritional (mTORC1 activator), proteostasis (chaperone function), mitogenic (role in cell division), and oxidative stress. PDIA3-knockout models have been associated with less fibrosis in murine models of lung injury (33, 34), muscle injury (35), and traumatic brain injury (38). PDIA3 is also described in the kidney, where it is upregulated in human CKD and a murine model of CKD (37). Intriguingly, it was demonstrated that ER stress alone is insufficient to induce a secretable form of PDIA3 – where it may have direct ECM stabilization function, instead requiring a more proinflammatory/SASP-like initiation, such as TGF-β. Based on our data, it appears the inhibition of PDIA3 inhibits p-SMAD–mediated TGF-β intracellular signal transduction. To our knowledge, this is the first study of inhibition of PDIA3 in kidneys in vivo and provides a connection between the cellular stress response (present in senescent and nonsenescent cells) and tissue fibrosis.

There are limitations to a single-cell RNA-Seq workflow in this setting. Conventional senescent markers such as p16*^INK4a^* (*CDKN2A*) and SA-β-gal (*GLB1*) are poorly sequenced and are often not present in RNA-Seq data of senescent cells. This is compounded by the high dropout intrinsic to single-cell workflows, necessitating a more involved classification process and requiring sensitivity to be weighed against specificity when setting thresholds and classifying cells. We optimized our workflow for specificity, at the natural expense of sensitivity, and as a result have probably underclassified and underquantified the true number of senescent cells in our data. The cells we have not captured may have a varied transcriptome worth investigating, but the risk of false positives obscuring ligands and DEGs outweighed this consideration. LOC14 is a PDI inhibitor and it is possible that LOC14 will react and oxidize both catalytic domains of PDIA1 and PDIA3 (38). It is therefore reassuring that consistent effects were noted with our siRNA studies, which are specific to PDIA3. The role of TGF-β during injury and repair is complex, and it is not surprising that inhibiting a partner molecule results in heterogenous outcomes across injuries. More work is required to understand the optimal clinical scenarios where inhibiting PDIA3 and where depleting senescent cells would be indicated. While fibrosis is often a feature of maladaptive postinjury repair and CKD development, these are heterogenous conditions, and prevention of fibrosis may not prevent the development of CKD when targeting this pathway alone.

In summary, our data shed light on the role of senescent epithelia in renal disease. We demonstrate increased senescence and persistent fibrosis following injury in humans, and attenuation of fibrosis following senolysis with ABT-263 in a relevant mouse model, and we use single-cell RNA-Seq to describe the in vivo senescent landscape. We identify PDIA3 as a SASP component and an upregulated protein in cells undergoing stress. Furthermore, we link this response with tissue fibrosis and demonstrate that its inhibition reduces fibrosis in a model of kidney disease. Our work demonstrates the ability of single-cell transcriptomics to reveal potentially novel senescence and stress-associated signaling pathways, leading to new evidence-based antifibrotic therapies.

## Methods

All reagents were purchased from Sigma-Aldrich, USA, unless otherwise stated.

### Human tissue samples.

Human kidney samples from 3 cohorts of patients were analyzed. Nontumor kidney was analyzed following nephrectomy for renal cell carcinoma in patients with no obstruction (*n* = 4), with obstruction (*n* = 7), and following deobstruction (*n* = 11).

### Mice.

Mice were purchased from Charles River Laboratories or Envigo or bred in-house before use. All mice were housed in a pathogen-free environment at the University of Edinburgh Animal Facility and were fed standard rodent chow. All procedures were approved in advance by the local Animal Welfare Ethical Review Body and performed in accordance with the Animals (Scientific Procedures) Act 1986 (amended in 2012).

### R-UUO.

The R-UUO model was performed as previously described and performed by a surgically trained animal technician (39). Briefly, 8-week-old male C57BL/6JOlaHsd mice (Envigo) underwent laparotomy, and the left ureter was isolated and ligated twice with 6/O black braided silk suture close to the bladder. In mice that required reversible ureteric obstruction, a silastic tube was placed around the ureter immediately proximal to the ligature to prevent excessive dilatation. Following 7 days of obstruction, the ureter was reanastomosed into the bladder, and the peritoneum and skin were sutured closed. For single-cell experiments, mice were culled at day 2 or day 7 post-UUO or 7 days following ureteric reanastomosis after 7 days of obstruction by CO_2_ narcosis and dislocation of the neck. For ABT-263 administration experiments, mice were culled at day 35 following reversal.

### UUO.

Surgery was performed as above, but without the placement of a silastic tube or reattachment of the ureter.

### IRI.

Surgery was performed as previously described (40). Anesthesia was induced with 2% isoflurane. Buprenorphine analgesia was administered subcutaneously. A posterior flank incision was made and the left renal pedicle identified and clipped using an atraumatic clamp for 15 minutes. During the ischemic period, body temperature was maintained at 37°C using a heating blanket with homeostatic control (Harvard Apparatus) via a rectal temperature probe. The clamp was then removed, the peritoneum closed with 5/0 suture, and the skin closed with clips. The other (right side) renal pedicle was not clamped, and the right kidney left completely intact. After surgery 1 mL sterile saline was administered subcutaneously. These animals were maintained for 28 days post-IRI before tissue harvest.

### ABT-263.

ABT-263 was administered as a dose of 50 mg/kg by gavage daily for 7 days, followed by a break of 2 weeks, then finally another 7 days. ABT-263 was reconstituted with 10% ethanol, 30% PEG 400, and 60% Phosal 50 PG (MedChemExpress). The vehicle was the above mixture without the drug.

### Loc14.

Loc14 was orally administered by gavage at 20 mg/kg, once daily following UUO. The drug was reconstituted, as per manufacturer’s guidelines, by adding each solvent one by one: 10% DMSO, 40% PEG 300, 5% Tween-80, and 45% saline.

### Immunohistochemistry.

Immunohistochemistry was carried out on-board Bond Rx using Leica Bond Intense R staining kit with protocol ImmPRESS Rat using a Bond Rx Autostainer (Leica). Sections were first warmed to 60°C for a 1 hour, then dewaxed in Bond dewax (Leica, AR9222) for 30 minutes before antigen retrieval in ER2 (Leica, AR9640) at 100°C for 20 minutes. Endogenous peroxidase was blocked for 5 minutes before incubation with 2.5% normal goat serum (ImmPRESS Rat kit – Vector Laboratories) for 20 minutes. Slides were then exposed to P21 1/150 Ab for 35 minutes (Hugo291, Ab107099, Abcam). Slides were then immersed in ImmPRESS Polymer Reagent RTU for 30 minutes, mixed with “DAB intense” for 5 minutes, and counterstained in hematoxylin before dehydration, clearing, and mounting. Slides were imaged on the bright-field microscope (Zeiss Axioskop), where 5 random fields were imaged from each organ’s cortex. Tubules were considered positive if they contained any cells with positive nuclear p21 staining.

### Picrosirius red staining.

Formalin-fixed, paraffin-embedded slides were immersed twice in xylene for 5 minutes to dewax. These were then moved through graded ethanol concentrations (100%, 75%, 65%) for 5 minutes each, followed by deionized water for 2 minutes, followed by tap water for 10 minutes. Slides were then lowered into 0.5% Direct Red in saturated picric acid solution (239801, Sigma-Aldrich) for 2–4 hours. Next slides were rinsed twice with methylated spirit (100% IMS), rapidly dehydrated in 3 changes of 100% ethanol for 20 seconds each (with agitation), before clearing in xylene and mounting.

Whole-slide images were acquired using the bright-field capabilities of an Axioscan Z1 slide scanner (Zeiss). The percentage of Sirius red staining per cortex per organ was then quantified in ImageJ version 1.52p (NIH). This was performed by importing.czi files, where each organ formed a separate region of interest. The cortex from each organ was selected using the polygon tool, taking care to exclude any major blood vessels, renal capsule that was incompletely removed during preparation, or other obvious artifacts. Following conversion to an RGB stack, the thresholding tool was used to “gate in” any Sirius red–staining areas, using the colored images as a guide. This threshold was then kept identical for all images, and the area of red stain gated in was expressed as a percentage of the selected cortex.

### SA-β-gal.

Frozen sections were cut, warmed to room temperature, and air-dried for 20 minutes before fixing with 500 μL of 4% paraformaldehyde for 5 minutes. These were then rinsed with PBS-MgCl_2_ at pH 5.5 before incubation with fresh SA-β-Gal staining solution — 1 mg/mL X-Gal (Teknova) in dimethylformamide, 40 mM citric acid/sodium phosphate (pH 6.0), 5 mM potassium ferrocyanide, 150 mM NaCl, and 2 mM MgCl_2_ — at ambient CO_2_ at 37.4°C for 14 hours. Slides were then rinsed and counterstained with nucleofast red (Sigma-Aldrich) before mounting.

Whole-slide images were acquired using the bright-field capabilities of an Axioscan Z1 slide scanner. Following a similar method to the Sirius red workflow above, thresholding in ImageJ allowed quantification of positive staining as a percentage of total renal cortex.

### Cystatin C measurement.

Tail vein blood was recovered from mice in an equal volume of 4% citrate buffer before centrifugation at 5,000*g* for 5 minutes at 4°C. A total of 2 μL of the clear phase was transferred into 250 μL PBS with 0.5% BSA. The mouse cystatin C ELISA (R&D Systems; Duoset ELISA kit) was carried out according to the manufacturer’s instructions.

### RNA extraction for qPCR.

Total RNA from cortical kidney tissue was isolated using the RNeasy Kit (QIAGEN) following the manufacturer’s instructions. For qPCR analysis of targeted gene expression, cDNA was synthesized from 1 μg of template RNA using the QuantiTect Reverse Transcription Kit (QIAGEN). VWR Perfecta qPCR mastermix with TaqMan (Thermo Fisher Scientific) gene expression assays on a Roche Lightcycler 480 used standard protocol. For hPTECs, the TaqMan gene expression assays used were *MDK* (Hs00171064_m1), *LAMA3* (Hs00165042_m1), *PROS1* (Hs00165590_m1), *NRP1* (Hs00826128_m1), *EGFR* (Hs01076090_m1), *JAG1* (Hs01070032_m1), *EPHB2* (Hs00362096_m1), *PDIA3* (Hs00607126_m1), *MAL2* (Hs00294541_m1), and *LGALS3* (Hs00173587_m1). Expression levels were normalized to GAPDH (Hs02758991_g1), *HPRT1*(Hs02800695) and *PPIA* (Hs04194521) average expression and presented as fold increases over day 7 nonirradiated cells analyzed in parallel. For murine kidneys, the following TaqMan gene expression assays were used: *Cdkn2a* (Mm00494449_m1), *Cdkn1a* (Mm04205640_g1), *Il6* (Mm00446190_m1), and *Il10* (Mm01288386_m1). mRNA expression levels were normalized to HPRT (Mm03024075_m1) expression and presented as fold increases as compared with uninjured animals. A relative quantification approach was taken using the ΔΔCt method. The Lightcycler 480 software was used to calculate the crossing point (equivalent to a CT on this platform) values of the target and the reference genes based on an assumption that the efficiency = 2. Data were then presented as fold change compared with uninjured control animals.

### Cell culture of hPTECs.

HPTECs (ATCC) were maintained in DMEM-F-12 + Glutamax-1 supplemented with hTERT immortalized RPTEC growth kit (ATCC) and 50 mg/mL gentamicin. Cells were grown at 37°C in 5% CO_2_. HPTECs were plated at 1 × 10^6^ cells per well of a 6-well plastic culture dish. After overnight culture, these cells were exposed to 10 Gy radiation. Cells were incubated for 7 days with media change every 3–4 days. To induce senescence, 100,000 hPTECs were seeded onto a 12-well plate. The following day, they were exposed to 10 Gy γ-radiation. Cells were analyzed on day 7 and day 14 alongside nonirradiated control groups.

### Cell culture of human renal fibroblasts.

Human primary kidney fibroblasts (Cell biologics) were plated at 3 × 10^6^ cells per well of a 6-well plastic culture dish plate precoated with gelatin-based coating solution (Cell biologics). Cells were maintained in fibroblast medium with added FCS (Cell biologics). Cells were grown at 37°C in 5% CO_2_. At 24 hours before being treated with the protein of interest, medium was swapped for serum-free medium to ensure a “serum-starved” phenotype to minimize baseline activation of cells. Cells were then treated with PDIA3 (10 ng/mL) ± TGF-β1 (10 ng/mL). Cells were collected for qPCR 72 hours later.

### siPDIA3 downregulation and pPDIA3 upregulation.

For siRNA-mediated gene knockdown, 4 × 10^6^ cells were transfected with siRNA oligonucleotide specific for the knockdown of PDIA3 expression in human cells (sense strand: 5′ ACCTCGTCCTTCACATCTCACTAACATCAAGAGTGTTAGTGAGATGTGAAGGACTT 3′) (antisense strand: 3′ CAAAAAGTCCTTCACATCTCACTAACACTCTTGATGTTAGTGAGATGTGAAGGACG 5′). The oligonucleotide was designed in our laboratory and synthesized by Eurofins MWG Operon. Nonspecific controls from Eurofins MWG were used as a control for knockdown efficiency. TK188 cells with 70% confluence were transfected with the siPDIA3ERP57 using transfection reagent, Lipofectamine 2000 (Invitrogen), according to the manufacturer’s protocol. Transfection reagent was removed after 6 hours and replaced with normal complete culture medium.

For the overexpression of PDIA3, 70% confluent TK188 cells were transfected with pPDIA3 (pcDNA3.1-ERP57, a gift from Neil Bulleid, University of Glasgow) using transfection reagent, or LTX reagent with PLUS reagent (Invitrogen), according to the manufacturer’s protocol. After 6 hours of transfection medium was replaced with normal complete media supplemented with 0.5 mg/mL G-418 (Invitrogen) as a selection factor for stable transfection.

Both overexpression and knockdown of PDIA3 were confirmed by performing Western blotting using mouse anti-PDIA3 antibody (Enzo Life Sciences catalog ADI-SPA-725-F).

The activation of TGF-β1 pathway in the siPDIA3- and pPDIA3-treated TK188 was investigated by monitoring the phosphorylation of SMAD2 using the rabbit anti–p-Smad2 antibody phospho-specific to Ser465/467 (Merck Millipore catalog AB3849-I).

### Measurement of cell viability.

A total of 6 × 10^3^ TK188 cells/well were seeded in 96-well plates and treated with either siPDIA3 or pPDIA3 for 24 hours. Medium was removed, and after washing in PBS, the cells were incubated for further 24 hours in 10 mL serum-free DMEM. Purified human *TGFβ1* (5 ng/mL) (R&D Systems) was added to the medium, and the cells were incubated for additional 48 hours. Cell viability was assessed using cell Proliferation Kit I (MTT), a colorimetric assay for the nonradioactive quantification of cell proliferation and viability (Roche Applied Bioscience).

For the trypan blue protocol to determine the impact of the different treatments on cell viability, fibroblast cell line TK188 was cultured in a 6-well plate and treated for 48 hours as follows: no treatment (control), PDIA3, TGFB1, PDIA3 and TGFB1, PDIA3 and Loc14, TGFB1 and Loc14, or TGFB1 and PDIA3 and Loc14. The cells were then suspended in PBS and mixed to equal volume (100 μL each) with the 0.4% trypan blue staining solution. To determine the percentage of viable cells, 10 μL of the stained cell suspension was counted on a Countess II FL Automated Cell Counter (Thermo Fisher Scientific).

To investigate whether ERp57 is involved in the TGF-β1 pathway regulation, TK188 cells were treated with human recombinant ERp57 (Novus Biologicals) with and without TGF-β1 and/or Loc14. The activation of TGF-β1 pathway was monitored by investigation the expression of the p-SMAD and SARA using rabbit anti–p-Smad2 antibody phospho-specific to Ser465/467 (Merck Millipore) and the rabbit anti-SARA antibody (Cell Signaling Technology catalog 13285).

### siSARA downregulation.

For siRNA-mediated gene knockdown, 4 × 10^6^ cells were transfected with silencer select predesigned siRNA (Life Technologies) specific for knockdown of SARA expression in human cells. As control for knockdown efficiency nonspecific controls from Eurofins MWG were used. TK188 cells with 70% confluence were transfected with the siSARA (50 nM) using transfection reagent, Lipofectamine 2000, according to the manufacturer’s protocol. Transfection reagent was removed after 6 hours and replaced with normal complete culture medium. The knockdown of SARA was confirmed by Western blotting using mouse anti-SARA antibody (Cell Signaling Technology).

The activation of TGF-β1 pathway in the siSARA-treated TK188 was investigated by monitoring the phosphorylation of SMAD2 using the rabbit anti–p-Smad2 antibody phospho-specific to Ser465/467 (Merck Millipore).

### Cell cycle monitoring by staining PCNA and cyclin D1 under TGF-β1 and PDIA3 treatments.

To monitor the effect of the TGF-β1 or PDIA3, and the combined treatments on cell cycle, TK188 cells were cultivated overnight in 8-well Chamber slides (30,000 cell/well, Thermo Fisher Scientific). The cell treatments were carried out in FCS-free medium. After the treatments, the medium was removed, and the cells were washed twice with PBS buffer. Fixation of the cells was carried out for 20 minutes at 25°C with 4% paraformaldehyde in PBS. Nonspecific binding sites were blocked with 10% goat serum in 1% BSA (BSA/PBS) (Sigma-Aldrich) for 60 minutes at 37°C. The incubation with the primary anti-PCNA (catalog ab29 Abcam) and anti-cyclin D1 (catalog b134175 Abcam) antibodies was carried out overnight at 4°C. Alexa Fluor 488–conjugated goat anti-mouse antibody rabbit (catalog AB_2536161, Thermo Fisher Scientific) or Alexa Fluor 555–conjugated goat anti-mouse antibody (catalog AB_2536164, Thermo Fisher Scientific) were used as secondary antibodies. The incubation with the secondary antibody was performed for 60 minutes at 37°C in the dark. The unbounded secondary antibody was removed with 3 successive wash steps with PBS buffer for 10 minutes each. Thereafter, the samples were counterstained with DAPI in PBS buffer for 5 minutes. The monitoring of the staining was performed on an immunofluorescence Laser Scanning Cytometer IX71 (Olympus) using the CellD 3.4 software (Olympus).

### Impact of PDIA3 treatment on SARA expression.

To investigate whither PDIA3 treatment had any effect on SARA expression, TK188 cells were treated with recombinant PDIA3 (200 ng/mL) (Abcam) for 6, 24, and 48 hours, and the expression of SARA was monitored using Western blot analysis.

### Measurement of free active TGF-β1.

For measuring free active TGF-β1 in media exposed to hPTECs, 80,000 cells were seeded into 24-well plates. For 48 hours, they were maintained at 37°C in 5% CO_2_ in in-house media composed of DMEM:12 + Glutamax-1 supplemented with the following: geneticin (Life Technologies) 50 mg in 1 mL, hydrocortisone (Merck) 12.5 μg, ascorbic acid (Sigma-Aldrich) 1.75 mg, insulin-transferrin-selenium-ethanolamine (ITS-X) (Thermo Fisher Scientific) 10 mL, triiodo-l-thyronine (Merck) 6 pM, prostaglandin E_1_ (Merck) 12.5 μg, recombinant human epidermal growth factor (Promega) 5 μg, and HEPES (Sigma-Aldrich) 1.13 g in 4 mL of 1N sodium hydroxide.

The cells were then maintained for a further 24 hours in serum-starved media composed of DMEM:12 + Glutamax-1 supplemented only with FBS 0.1% and penicillin-streptomycin. Recombinant Human Latent TGF-β1 (299-LT-005, R&D Systems) was then incubated at 5,000 pg/mL for 1 hour at 37°C at 5% CO_2_, in the presence or absence of PDIA3 at 2 μg/mL (ab92937, 100 μg, Abcam), with or without Loc14, a PDIA3 inhibitor (MedChemExpress), used at 100 μM.

LEGEND MAX Free Active TGF-β1 ELISA Kit (BioLegend) was used to measure active TGF-β per manufacturer’s instructions. The cytokine concentration in each supernatant was extrapolated from a standard curve.

### Bioinformatic methods and data availability.

For bioinformatic methods for bulk and single-cell RNA-Seq, complete workflow and methods are provided in Supplemental Methods. Data have been deposited in the National Center for Biotechnology Information Gene Expression Omnibus database, accession GSE157866 and GSE140023.

### Statistics.

Animal group size was determined from previous pilot experiments. Normality was assessed with a Shapiro-Wilk test and visualization of data distributions. Levene’s test was used to test for homogeneity of variance across groups. Comparisons between 2 unpaired, normally distributed data points were carried out via a *t* test and between 2 unpaired, non-normally distributed data points were carried out via Mann-Whitney test. Comparisons between multiple groups were performed with 1-way ANOVA with Tukey’s multiple-comparison test if parametric or Kruskal-Wallis test if a nonparametric distribution. All statistical analysis was performed using R version 4.

### Study approval.

Samples were obtained under local ethical approval, by East of Scotland Research Ethics Service (bioresource ethics project code SR1405; National Health Service Lothian Edinburgh, United Kingdom), from patients in the southeast Scotland. The requirement for written consent was waived by the board. All animal procedures were approved in advance by the University of Edinburgh Animal Welfare Ethical Review Body (Edinburgh, United Kingdom).

## Author contributions

EOS and DF conceived and designed the study. EOS and KJM performed flow cytometry sorting experiments for library preparation. KJM performed hPTEC work and animal experiments alongside DAF and EOS. EOS and C Carvalho performed fibroblast experiments. DPB and C Carvalho performed cell culture and measurement of free TGF-β1. MD and EOS produced immunofluorescence images. EOS and C Cairns prepared cDNA libraries, and C Cairns supported tissue staining and qPCR. KMG and AL provided human biopsy samples. NCH provided single-cell platform, resources, and expertise. KK supervised single-cell RNA-Seq workflow and analysis. TC, BC, and LD provided intellectual input, designed single-cell RNA-Seq experiments, and supervised analysis. RC performed staining, qPCR, and analysis of same. RB and LD performed R-UUO surgeries. HD performed animal experiments and confirmed independently the results of EOS and DAF. HD and GHD performed fibroblast experiments, and WB, HD, and MZ provided intellectual input and guidance. EOS performed statistical, microscopic, and bioinformatic analysis of data; generated figures; and wrote the manuscript. DAF performed animal surgeries. DAF and JH supervised the project and provided intellectual input, guidance, and manuscript editing. All authors critically reviewed and approved the manuscript before submission.

## Supplementary Material

Supplemental data

Supplemental data set 1

Supplemental data set 2

Supplemental data set 3

Supplemental data set 4

## Version 1 11/22/2022

Electronic publication

## Version 2 12/01/2022

Laura Denby's affiliation was updated
