# Supplementary material for: Single-cell analysis of senescent epithelia reveals targetable mechanisms promoting fibrosis
Source: JCI Insight. 2022 Nov 22;7(22):e154124. doi: 10.1172/jci.insight.154124 (PMC9746814; doi:10.1172/jci.insight.154124)
Supplement: Supplemental data set 2 [file jciinsight-7-154124-s290.pdf]

```
"","p_val","avg_logFC","pct.1","pct.2","p_val_adj","rank"
"Cdkn1a",0,1.4849092465621,1,0.065,0,Inf
"Tshz2",6.84644011262485e-89,0.855529590067071,0.226,0.013,1.227840569
79814e-84,165.298489748769
"Cd24a",8.60438031803446e-78,1.14823409465891,0.91,0.268,1.54310956623
63e-73,192.507086335567
"Epcam",8.64869528587093e-70,1.17850638591324,0.845,0.245,1.5510570125
6809e-65,175.867440717404
"Cldn7",9.45081753492414e-70,0.942722282772683,0.723,0.179,1.694909616
7133e-65,140.59797987702
"Tmsb10",1.04783158758521e-69,1.30484409816152,0.923,0.345,1.879181169
17532e-65,194.470304186116
"Lcn2",2.62542750367754e-67,1.36823477106204,0.748,0.207,4.70844168509
53e-63,196.360166095136
"Tspan8",4.57442658407974e-65,1.04413003526395,0.748,0.208,8.203776635
88861e-61,144.458623067004
"Mal",2.05054885908182e-63,1.11191384093746,0.839,0.262,3.677454323877
33e-59,149.60834057614
"Akap12",2.76566101876824e-62,0.619990804143933,0.239,0.022,4.95993647
105895e-58,81.8068829461728
"Anxa2",2.87355312233762e-61,1.09902546532218,0.639,0.168,5.1534301696
0029e-57,142.442149344655
"Tpm1",1.20334050247769e-60,0.980157840733443,0.716,0.213,2.1580708571
4349e-56,125.632270819501
"Dcdc2a",1.18354424082008e-59,0.79837231810489,0.548,0.119,2.122568241
48673e-55,100.506734512886
"Gpx3",1.54231737693256e-58,-2.94068439393819,0.845,0.974,2.7659919837
9085e-54,-362.651656281669
"Hoxb9",2.36110600346215e-57,0.769345892410465,0.594,0.147,4.234407506
60902e-53,92.7783189181267
"Hspb1",1.36258961930814e-55,1.17801521911601,0.555,0.135,2.4436682232
6722e-51,137.283937496897
"Krt18",4.34341581436435e-55,0.925064416259763,0.574,0.138,7.789481921
48103e-51,106.733067774163
"Spink1",5.13135245807576e-55,-2.72982066351682,0.523,0.885,9.20256749
831308e-51,-314.509073436202
"Aldob",5.09463485021935e-54,-2.55262896026956,0.29,0.81,9.13671814038
339e-50,-288.235085339747
"Clu",1.07413650525236e-53,1.41647970559913,0.903,0.386,1.926356408519
59e-49,158.888000595937
"Slc34a1",1.84129078841232e-53,-2.50078416059748,0.245,0.798,3.3021708
9993866e-49,-279.167761126108
"Adamts1",4.96064059929907e-53,0.951314205171829,0.516,0.119,8.8964128
5078295e-49,105.254375343662
"Tacstd2",1.49809958089223e-52,1.07063386398928,0.568,0.142,2.68669178
837212e-48,117.272708797153
"Acsm2",3.71647653997569e-52,-2.4187558464535,0.129,0.751,6.6651290267
924e-48,-262.742663356324
"Miox",1.54335166270813e-51,-2.53422900449528,0.232,0.779,2.7678468719
0075e-47,-271.678040813933
```

"Bst1",1.68715823493292e-51,0.842858426723539,0.148,0.01,3.02574957852
871e-47,90.2822245263212
"Ass1",1.84589804121414e-51,-2.46923559986927,0.135,0.745,3.3104335471
1344e-47,-264.268508435203
"Lgals3",3.05367610952385e-51,1.21917485172111,0.832,0.319,5.476462734
82007e-47,129.867773103925
"Klf6",4.02647948918784e-51,1.09768210754804,0.626,0.179,7.22108831590
946e-47,116.622679752196
"Pkhd1",8.74026435169766e-51,0.916709859142627,0.761,0.278,1.567479008
83346e-46,96.6848806892995
"Capg",2.51855624543178e-50,0.830039791968649,0.574,0.154,4.5167787705
5735e-46,86.665380339264
"Gpx1",1.48111131991949e-49,-1.68845488329163,0.813,0.943,2.6562250411
4361e-45,-173.302035366474
"Guca2b",1.84050589766072e-49,-2.08274597079848,0.31,0.796,3.300763276
86474e-45,-213.319373232622
"mt-
Nd1",2.79242036729499e-49,-0.993895055848863,0.994,1,5.00792668670684e
-45,-101.382570486761
"Foxc1",2.85273814804919e-49,0.439789222908494,0.226,0.025,5.116100594
71142e-45,44.8514362433698
"Marcksl1",1.02275613124092e-48,0.584836639676653,0.484,0.11,1.8342108
4576746e-44,58.897219360047
"Wfdc2",2.2711566095524e-48,1.11575471290484,0.871,0.377,4.07309226357
128e-44,111.474318765518
"Sox4",2.2790048152998e-48,1.06581704613191,0.639,0.19,4.0871672357586
6e-44,106.481402669617
"Sepp1",2.63355299015431e-48,-1.72123450660718,0.51,0.852,4.7230139325
4275e-44,-171.712586735763
"Akr1c21",3.22563040030869e-48,-2.32053205302646,0.2,0.743,5.784845559
91361e-44,-231.028696035702
"Txnip",4.2433470640537e-47,0.885973711063432,0.735,0.251,7.6100186246
7391e-43,85.9232307886326
"S100a11",9.94653985131307e-47,1.078708059551,0.942,0.533,1.7838124569
3449e-42,103.696012678219
"Jun",2.32904448965637e-46,1.39857239184127,0.677,0.224,4.176908387749
73e-42,133.254582492831
"Bcam",3.41209431602419e-46,0.915868461257703,0.632,0.199,6.1192499463
5778e-42,86.9132924373582
"Krt7",6.32432315448793e-46,0.697537887388049,0.71,0.214,1.13420411452
586e-41,65.7639122209898
"Ftl1",7.67882815708143e-46,-1.21113574514046,0.994,0.993,1.3771210416
9098e-41,-113.95091008036
"mt-
Nd2",8.80046087912286e-46,-0.871809488739383,0.968,0.997,1.57827465406
189e-41,-81.9062020471246
"Cldn3",1.02880369601306e-45,0.6186735463046,0.426,0.092,1.84505654842
982e-41,58.0275442027571
"Hoxb6",1.08804092720693e-45,0.687196984906275,0.497,0.125,1.951292598
8529e-41,64.4161251657708

"Apela",1.75793271817923e-45,1.05153776783224,0.406,0.082,3.15267653678263e-41,98.0640326259186
"mt-Nd4",2.36363957611973e-45,-0.904740961562711,0.994,0.999,4.23895121581313e-41,-84.1062333541443
"Cd9",1.92816107384552e-44,0.925369467820824,0.806,0.313,3.45796406983456e-40,84.0815902963046
"Neat1",2.35376131032729e-44,0.931964697447802,0.91,0.42,4.22123553394096e-40,84.49497245091
"Iqgap1",9.72807746482463e-44,0.760015415433421,0.703,0.24,1.74463341254165e-39,67.8270241098531
"Hes1",4.24779555597571e-43,0.848173373702518,0.652,0.21,7.61799655008683e-39,74.4444358822011
"Cldn4",6.73254560783487e-43,0.906351535658635,0.465,0.112,1.20741472930911e-38,79.1333278460197
"Hoxb4",8.29243501202826e-43,0.662809591479524,0.503,0.135,1.48716529505715e-38,57.7316179738937
"Klf5",8.3989077942198e-43,0.264504659526463,0.135,0.01,1.50626012381538e-38,23.0353414688049
"Tsc22d1",1.36028925727485e-42,1.05345630822065,0.877,0.445,2.43954275399671e-38,91.2360825447864
"Akr1a1",1.41692894619829e-42,-1.27265590390016,0.806,0.925,2.54112037211202e-38,-110.168258132681
"Gatm",1.77998241484414e-42,-2.39448222380224,0.123,0.678,3.19222046278147e-38,-206.733649199168
"Tmem27",2.22882466829102e-42,-1.89100683362113,0.368,0.777,3.99717416011311e-38,-162.839601824599
"Malat1",2.67568469709311e-42,1.10968260790471,1,0.902,4.79857293576678e-38,95.3549321355157
"Bicc1",4.56237015922597e-42,0.881872822123337,0.787,0.331,8.18215464355586e-38,75.308656691023
"Dbi",4.59320787329388e-42,-1.1424459283999,0.871,0.961,8.23745899996525e-38,-97.5529341416492
"Btg1",9.61909347552781e-42,0.896599498758208,0.755,0.3,1.72508822390116e-37,75.8974795334382
"Il1f6",1.0298221477999e-41,1.14205886450263,0.206,0.025,1.84688303986434e-37,96.597793119452
"mt-Cytb",1.93423691556711e-41,-0.813100854255971,0.994,1,3.46886048437805e-37,-68.2612984215008
"Fbp1",2.52881665339627e-41,-1.77860534820793,0.039,0.63,4.53517978620086e-37,-148.840425271734
"Jund",3.47277670420185e-41,1.07062904362604,0.877,0.439,6.2280777413156e-37,89.2546825698571
"Sh3bgrl3",6.87775894430097e-41,0.821625447389112,0.897,0.439,1.23345728907094e-36,67.9346559604532
"Arl4c",7.7308820346089e-41,0.538992638974481,0.394,0.086,1.38645638408676e-36,44.5026345137255
"Krt8",1.03423439419163e-40,0.921988117809431,0.697,0.259,1.85479596254327e-36,75.8568385255761

"Sh2d4a",1.0700442621538e-40,0.563727851979389,0.4,0.09,1.91901737974662e-36,46.3616832483906
"mt-Atp6",1.45176318409625e-40,-0.763981743756155,1,1,2.60359209435821e-36,-62.5977390123443
"Anxa3",3.74463906209651e-40,0.528804177780883,0.394,0.088,6.71563569396388e-36,42.8271231758082
"Sfn",4.89862665364453e-40,0.687271354152939,0.439,0.109,8.7851970406461e-36,55.4765404063874
"Rbms1",5.73258185879157e-40,0.570058960353846,0.542,0.159,1.02808123055568e-35,45.9255369317689
"Ptma",7.18621012113661e-40,0.881848831467068,0.961,0.826,1.28877492312464e-35,70.844900998697
"Fus",9.06051094263191e-40,0.786110163792934,0.806,0.361,1.62491203245161e-35,62.9713739887802
"Arpc1b",9.68016960109727e-40,0.665198244385753,0.735,0.268,1.73604161626078e-35,53.241716249868
"Slc38a2",1.37335674535708e-39,0.699383301536864,0.529,0.156,2.46297798712339e-35,55.7332308013217
"Sytl2",2.09053493251422e-39,0.666290277034527,0.439,0.107,3.749165347971e-35,52.8161273332509
"Rbm39",2.27763991725055e-39,0.777014381717734,0.955,0.512,4.08471942759713e-35,61.5265047808393
"Ly6e",3.05358615596981e-39,0.974218323843033,0.839,0.404,5.47630141211625e-35,76.8561306290468
"Tmsb4x",4.7278273669078e-39,1.01823982052989,0.968,0.613,8.47888559981245e-35,79.8838660307214
"Ttc36",8.69712320601366e-39,-1.59317469273082,0.394,0.774,1.55974207576649e-34,-124.018091366924
"Fth1",9.68680054930563e-39,-0.890478385981828,1,0.998,1.73723081051247e-34,-69.2218723377589
"Proser2",3.80721941586613e-38,0.241018348918384,0.116,0.008,6.82786730041433e-34,18.4058195204564
"Hrsp12",7.17845232558964e-38,-1.35329918058082,0.555,0.835,1.28738364007125e-33,-102.48899556986
"Runx1",7.88215769335629e-38,0.352548673775651,0.258,0.042,1.41358616072652e-33,26.6664919658551
"Keg1",9.40483921489042e-38,-1.68955718512485,0.103,0.633,1.68666386479845e-33,-127.498302912651
"mt-Co2",9.59532223113351e-38,-0.718154392302537,0.994,1,1.72082508893148e-33,-54.1793657504456
"Ephb2",1.03123790959731e-37,0.360398055306314,0.265,0.044,1.84942206707182e-33,27.1633581564318
"Slc27a2",1.66853406821878e-37,-1.8921136547468,0.11,0.63,2.99234899794357e-33,-141.698971330113
"Kcnk1",3.06118601030512e-37,0.593104279665345,0.477,0.131,5.4899309908812e-33,44.0572048263323
"Spint2",3.65849557535141e-37,0.946743555327658,0.903,0.577,6.56114596483522e-33,70.1576196107841

"Nfkbiz",3.7002187129776e-37,0.642910857687155,0.413,0.103,6.635972239
85403e-33,47.6350674478647
"Tcn2",4.7024667479914e-37,-1.41099190600549,0.355,0.75,8.433403865847
78e-33,-104.206137054461
"Mecom",5.27634692234582e-37,0.59331793954127,0.619,0.195,9.4626005705
35e-33,43.7500549807477
"Cyp2j5",5.83080274167331e-37,-1.53795762434244,0.09,0.624,1.045696163
69169e-32,-113.252185254715
"mt-
Co3",6.37115306151751e-37,-0.679545162787631,1,1,1.14260259005255e-32,
-49.9801487417639
"Pah",7.30477444522017e-37,-1.52458801123082,0.052,0.599,1.31003824900
579e-32,-111.924071433823
"Elf3",8.36140516156131e-37,0.560400447774836,0.419,0.105,1.4995344016
744e-32,41.0647820954475
"Tubb2b",9.47556338602242e-37,0.527879496258073,0.484,0.134,1.69934753
764926e-32,38.6156935532922
"Gprc5a",1.20246056593773e-36,0.270051280713776,0.155,0.016,2.15649277
895273e-32,19.6905838368081
"mt-
Nd5",1.40963684301996e-36,-0.797114110190074,0.929,0.98,2.528042714271
99e-32,-57.994258368306
"mt-
Nd3",1.44721247229499e-36,-0.738448373961366,0.968,0.992,2.59543084781
383e-32,-53.706589963508
"Khk",4.63026089969757e-36,-1.50412133627722,0.161,0.644,8.30390989751
762e-32,-107.643942287495
"Pdzk1",5.74664459570397e-36,-1.50482082778925,0.065,0.596,1.030603241
79355e-31,-107.368956402368
"S100a6",8.68069463373169e-36,1.58037193714828,0.606,0.227,1.556795775
61344e-31,112.107647193416
"Prdx5",1.68036219489053e-35,-0.771106497495515,0.91,0.966,3.013561560
31667e-31,-54.1910630922748
"Sord",2.14189054095535e-35,-1.4544338763155,0.174,0.65,3.841266496149
32e-31,-101.860310425708
"S100a10",2.36812721186059e-35,0.871340344251417,0.806,0.4,4.246999341
75078e-31,60.9362504468367
"Syne2",2.79980205366538e-35,0.853307891386645,0.755,0.332,5.021165003
04349e-31,59.5322844428779
"Tfap2b",2.98686084668134e-35,0.561780785835625,0.477,0.131,5.35663624
243832e-31,39.1571329171429
"Fam174b",3.03761042968898e-35,0.541286391393536,0.232,0.037,5.4476505
4460421e-31,37.7195169926928
"Sult1d1",3.61644474768668e-35,-1.33336815075008,0.194,0.681,6.4857320
1050129e-31,-92.6831311024432
"Slc4a4",5.53776941005762e-35,-1.59261620470751,0.142,0.634,9.93143565
999734e-31,-110.024987229493
"Ugt2b38",5.91769044822212e-35,-1.65690574945435,0.123,0.619,1.0612786
0498416e-30,-114.356450879958
"Ier3",1.40041122501471e-34,0.763392954008347,0.445,0.122,2.5114974909

4138e-30,52.0303243984302
"Flna",1.53307063053498e-34,0.559551663452147,0.4,0.103,2.749408868801
43e-30,38.0865368902327
"Pkm",3.34519755128084e-34,0.631132730706359,0.729,0.276,5.99927728846
706e-30,42.4663425018991
"Scp2",3.37723742998453e-34,-1.09634613794564,0.606,0.837,6.0567376069
3425e-30,-73.7582010142747
"Dock7",3.94332199872439e-34,0.485105949784572,0.413,0.109,7.071953672
51233e-30,32.5609982670069
"Cox6a1",5.50433233625739e-34,-0.721779037372095,0.923,0.964,9.8714696
11844e-30,-48.2061090937539
"Fxyd2",6.51541047528161e-34,-0.82113027798732,0.916,0.985,1.168473714
637e-29,-54.7030991336177
"Calml4",3.07249799230516e-33,-1.40870198707406,0.084,0.585,5.51021789
940008e-29,-91.6619329979368
"Junb",7.68810944876546e-33,1.14302000227902,0.639,0.249,1.37878554854
16e-28,73.3260813719115
"Chchd10",8.16086914672098e-33,-0.743082646831209,0.819,0.946,1.463570
27277294e-28,-47.6252842540487
"Anxa5",1.12404099605908e-32,0.777486683715797,0.71,0.293,2.0158551223
3236e-28,49.5813669686245
"Chpt1",2.51498058008647e-32,-1.04696350142684,0.665,0.844,4.510366172
32707e-28,-65.9231082934312
"Lrp2",2.69630255012417e-32,-1.52452426782066,0.116,0.593,4.8355489933
9269e-28,-95.8870697662183
"Hoxb8",2.93556048620052e-32,0.524743753570876,0.452,0.131,5.264634175
95202e-28,32.9598745555161
"Kap",3.04469632720605e-32,-2.84721543615538,0.671,0.871,5.46035839321
133e-28,-178.733573674603
"H3f3b",3.15953866485568e-32,0.849704313554345,0.981,0.755,5.666316641
55219e-28,53.308616091668
"Ly6a",6.60282915999376e-32,1.14615014725712,0.897,0.594,1.18415138155
328e-27,71.0621929567032
"Gls",8.19401052486018e-32,0.728129931802233,0.735,0.333,1.46951384752
843e-27,44.9874101801345
"Slc25a24",1.12801641474061e-31,0.300141670480813,0.226,0.038,2.022984
63819581e-27,18.4482748716506
"Btg2",1.18953696619286e-31,1.0474392355275,0.484,0.157,2.133315595170
28e-27,64.3254641547432
"Ttc3",1.30846015096753e-31,0.556750566863284,0.587,0.208,2.3465924347
4517e-27,34.1381816773024
"Cox7c",1.32635835576943e-31,-0.613514669187368,0.948,0.977,2.37869107
52369e-27,-37.6104413909459
"Arg2",1.6069965116148e-31,0.356627665426176,0.245,0.044,2.88198754392
998e-27,21.7939864324041
"Anxa6",1.75499637154359e-31,0.515505622442885,0.49,0.153,3.1474104927
2627e-27,31.4578117011562
"Dnm3",1.95722487197366e-31,0.479065196866961,0.271,0.054,3.5100870853
9755e-27,29.181852453752
"Cp",2.05286738606339e-31,0.81609996460245,0.381,0.098,3.6816123701660

9e-27,49.6731050059556
"Ddit4l",2.55584944067437e-31,0.407230821777285,0.297,0.063,4.58366038
690542e-27,24.6974496631506
"Rasd1",2.95398038074632e-31,0.472206152331249,0.219,0.036,5.297668414
83046e-27,28.5696672323081
"Zfp36l1",3.37192333457097e-31,0.892982267445492,0.716,0.333,6.0472073
0821958e-27,53.909518928073
"Cycs",3.94449133509606e-31,-0.823193489883641,0.819,0.913,7.074050760
36126e-27,-49.5672494500303
"Ktn1",6.12578462726445e-31,0.56650192570774,0.581,0.212,1.09859821505
361e-26,33.861620103148
"Chka",7.62331166367296e-31,0.631893758449762,0.626,0.237,1.3671647137
6311e-26,37.6321000130995
"Gabrp",9.61177973518368e-31,0.212452235417801,0.09,0.006,1.7237765777
0784e-26,12.6032391317416
"Klf7",1.02233537727962e-30,0.451699656899877,0.342,0.084,1.8334562656
1327e-26,26.7681776869451
"Ptbp3",1.12673553051993e-30,0.59547473635238,0.626,0.237,2.0206875004
3445e-26,35.2305330920193
"Cryab",2.17106418241193e-30,1.63764044536946,0.535,0.193,3.8935865047
3756e-26,95.8148734518956
"Ahnak",2.57629147922896e-30,0.650497870397883,0.497,0.163,4.620321138
84922e-26,37.9479304730259
"Uqcrq",2.62383855297221e-30,-0.719970896125371,0.871,0.934,4.70559206
090036e-26,-41.9875944944839
"H2-
D1",3.262167668796e-30,0.785099462927639,0.865,0.478,5.85037149721875e
-26,45.6148340614749
"Mal2",3.62481229213292e-30,0.542285375690078,0.458,0.136,6.5007383647
1119e-26,31.4500012336342
"Son",3.69875766845951e-30,0.683817373242948,0.865,0.454,6.63335200261
528e-26,39.6443820727631
"Uqcr10",3.73515244219202e-30,-0.696118040633396,0.865,0.945,6.6986223
8982717e-26,-40.3506983880991
"Lyz2",3.87106490588491e-30,0.698748219307399,0.839,0.426,6.9423678022
14e-26,40.478183547093
"Pnisr",5.0426772897711e-30,0.643759785851378,0.677,0.282,9.0435374514
7549e-26,37.1225123459855
"Maf",7.70142445840124e-30,-1.46594511327536,0.065,0.538,1.38117346236
968e-25,-83.913181377887
"Mt2",7.91985122717688e-30,1.21346676617523,0.542,0.196,1.420346119081
9e-25,69.4269559645106
"Cyb5a",9.91906468434145e-30,-1.07908172113724,0.703,0.87,1.7788850604
8979e-25,-61.4954003054816
"Nupr1",1.72654720049864e-29,1.42066500183691,0.387,0.111,3.0963897493
7426e-25,80.174363467729
"Uqcr11",2.94512221004287e-29,-0.662098671847891,0.897,0.947,5.2817821
7149088e-25,-37.0115565957516
"Rhob",3.08559487379876e-29,0.633508077530725,0.426,0.129,5.5337058466
707e-25,35.3838142013389

"Fut9",3.46816325316734e-29,-1.39517129285542,0.148,0.595,6.2198039782
303e-25,-77.7625073928819
"Scin",7.40381719109052e-29,0.537531567884073,0.348,0.09,1.32780057505
017e-24,29.5526891747839
"Igfbp4",7.56118395354825e-29,-1.23768319195502,0.135,0.581,1.35602273
022934e-24,-68.0199570405744
"Ogt",7.80511199985531e-29,0.556630739330589,0.729,0.321,1.39976878605
405e-24,30.5733525713296
"Pdgfa",8.83908069001626e-29,0.621806188128048,0.342,0.089,1.585200730
94752e-24,34.0758068506114
"Nfat5",9.36024351663262e-29,0.606697159173733,0.529,0.189,1.678666072
27289e-24,33.2130552232248
"Spp1",1.13200028451886e-28,0.843976951098133,0.961,0.847,2.0301293102
5613e-24,46.0422702650989
"Ccdc88a",1.15474072162131e-28,0.35042997591446,0.284,0.063,2.07091201
015566e-24,19.1103669273142
"Ddah2",1.17790037531775e-28,0.494185489044014,0.406,0.119,2.112446533
09485e-24,26.940124344084
"Itgb1",1.19275591345471e-28,0.647457672933312,0.658,0.291,2.139088455
18969e-24,35.2875195402827
"Gm21092",1.32717628183918e-28,0.551138615920209,0.445,0.141,2.3801579
4385038e-24,29.9791163319667
"Rsrp1",1.73131583862543e-28,0.623287111398656,0.865,0.474,3.104941824
99084e-24,33.7379375048094
"Glyat",1.73321618754528e-28,-1.30416678254431,0.103,0.553,3.108349910
7437e-24,-70.5918746864761
"Ndufa4",2.09309316703563e-28,-0.617203253861732,0.935,0.962,3.7537532
8576171e-24,-33.2915028401861
"Bclaf1",2.11864710882388e-28,0.489661300231843,0.613,0.236,3.79958172
496476e-24,26.4060389957213
"St14",2.77702095718449e-28,0.547670697657515,0.445,0.14,4.98030938461
466e-24,29.3861202914182
"Actn1",2.8848496981167e-28,0.432159641234956,0.374,0.102,5.1736894486
0249e-24,23.1717327826152
"Timp2",3.68705755880116e-28,0.328747752961784,0.252,0.051,6.612369025
954e-24,17.5462867731313
"Myof",3.73994820116968e-28,0.567516377971124,0.4,0.118,6.707223103977
71e-24,30.2820253770341
"Cdh1",3.80201531848667e-28,0.568349200213775,0.632,0.248,6.8185342721
7399e-24,30.3171090698271
"Crip2",4.31168978410067e-28,0.586680014042765,0.51,0.178,7.7325844588
0613e-24,31.2211150977716
"Ap2b1",5.91374135194186e-28,0.530376701007313,0.581,0.224,1.060570374
05725e-23,28.057272380214
"Vasp",8.19308535824345e-28,0.471267800940561,0.419,0.13,1.46934792814
738e-23,24.7767342347488
"Nfib",9.20390708826377e-28,0.550772737152728,0.581,0.223,1.6506286972
0922e-23,28.892601970272
"Dsp",1.05157612040279e-27,0.457814292421286,0.335,0.088,1.88589661433
036e-23,23.9551574692584

"Idh1",1.07331814664877e-27,-1.18098809979628,0.181,0.604,1.9248887641
999e-23,-61.7710971236898
"Cpm",1.14338625977212e-27,0.221122639792345,0.103,0.009,2.05054891827
532e-23,11.5517451335248
"Dync1i2",1.16979159616153e-27,0.560891349613427,0.748,0.34,2.09790424
855609e-23,29.2889151868935
"Efna5",1.24785295945373e-27,0.382814728233651,0.277,0.062,2.237899497
48433e-23,19.965288495699
"Hnrnpl",1.3959845635058e-27,0.480896079057856,0.568,0.212,2.503558716
19131e-23,25.0266706045726
"Oit1",1.49912271323906e-27,0.341203437449961,0.135,0.016,2.6885266739
2292e-23,17.7325008944819
"Hoxb3",1.76313382767021e-27,0.411818928353878,0.316,0.079,3.162004206
54376e-23,21.3356184354265
"Ehf",1.96547300960997e-27,0.467447923117837,0.348,0.094,3.52487929543
451e-23,24.1668757625279
"Calm2",2.06484935617503e-27,0.717015308565855,0.858,0.502,3.703100835
36429e-23,37.0340461887319
"Mier1",2.23970690391494e-27,0.484769177729977,0.51,0.181,4.0166903614
8106e-23,24.999061177835
"Cox7a2",2.7697893301619e-27,-0.615298155139403,0.929,0.949,4.96734018
471236e-23,-31.5996043849774
"Tubb5",3.25780261352864e-27,0.519553605096399,0.568,0.213,5.842543207
10227e-23,26.5981784266211
"Map3k1",4.67889970455168e-27,0.614743456493701,0.523,0.195,8.39113873
014297e-23,31.2488120637102
"Fubp1",4.84620704785449e-27,0.573643167871084,0.652,0.268,8.691187719
62224e-23,29.1394366292599
"Sdc4",4.96566373414595e-27,0.81905803897384,0.916,0.599,8.90542134081
734e-23,41.5858674688125
"Pnn",6.08082675854423e-27,0.501305324559742,0.581,0.225,1.09053547087
732e-22,25.3511121345295
"Cbx5",6.74440302446471e-27,0.533322329499085,0.574,0.224,1.2095412384
075e-22,26.9149811535397
"Cyp4b1",7.68888979724985e-27,-1.76715269505362,0.084,0.528,1.37892549
623879e-22,-88.9506337220908
"Grhl2",8.00538459080156e-27,0.263585544062577,0.206,0.037,1.435685672
51435e-22,13.2570954289291
"Acy3",8.00884856510408e-27,-1.61550730910395,0.148,0.571,1.4363069016
6577e-22,-81.2516119667191
"mt-
Nd4l",9.48718469875295e-27,-0.719391481334768,0.884,0.96,1.70143170387
435e-22,-36.0597871760521
"Sept4",1.03394037691142e-26,0.394346137247132,0.271,0.06,1.8542686719
5294e-22,19.7328368735626
"Cisd1",1.24156543308744e-26,-0.86421476715592,0.503,0.785,2.226623447
69902e-22,-43.086624808129
"Myl12a",1.30416882908005e-26,0.682321152754383,0.781,0.418,2.33889637
807216e-22,33.9845012484155
"Clic1",1.35345157943382e-26,0.650163585365002,0.787,0.425,2.427280062

55662e-22,32.3587071353994
"Irf2bpl",1.4700489214087e-26,0.493035466339312,0.51,0.181,2.636385735
65437e-22,24.4976815437957
"Pea15a",1.56355550242193e-26,0.517880769366758,0.452,0.149,2.80408043
804349e-22,25.7002456150035
"Camk2d",1.77565254451984e-26,0.431505297248441,0.477,0.16,3.184455273
34187e-22,21.3589041366408
"Ywhah",1.88067684756677e-26,0.62611946632179,0.645,0.285,3.3728058584
2625e-22,30.9560520822573
"Sema3c",1.89051683871976e-26,0.508176017161519,0.439,0.143,3.39045289
856001e-22,25.1221431040799
"Spp2",2.46736464591002e-26,-1.73056415692933,0.077,0.507,4.4249717559
7503e-22,-85.0911614751004
"Ralbp1",2.73395410656297e-26,0.489197970117553,0.665,0.272,4.90307329
471003e-22,24.0034816107069
"Slc22a12",2.78538222599321e-26,-1.32811862713164,0.039,0.482,4.995304
48409623e-22,-65.1420587120648
"Usmg5",2.98914110350012e-26,-0.626103652740166,0.871,0.934,5.36072565
501711e-22,-30.6651623883882
"Myh9",3.00670941409732e-26,0.557669241750296,0.548,0.208,5.3922326632
4214e-22,27.3101293240303
"4930523C07Rik",3.20856558682362e-26,0.4847978988675,0.303,0.077,5.754
24152340948e-22,23.7099792343618
"Lamb1",3.74696715635569e-26,0.602671339924626,0.516,0.191,6.719810898
20829e-22,29.3813198810051
"Msn",3.80815815886161e-26,0.623939055806411,0.619,0.259,6.82955084210
241e-22,30.4080524448164
"Upf3b",4.43722279345737e-26,0.425183538289568,0.452,0.152,7.957715357
78645e-22,20.6565770847748
"Etfb",5.66786766928521e-26,-0.903232215970416,0.555,0.8,1.01647538780
961e-21,-43.6603899110166
"Krt19",5.74810273396401e-26,0.654122126511586,0.219,0.042,1.030864744
30911e-21,31.6097249981983
"Pdlim7",6.30683469964997e-26,0.341511669794861,0.29,0.07,1.1310677350
3523e-21,16.4714919897636
"Slc4a11",6.3391441029129e-26,0.27272790162613,0.09,0.008,1.1368621034
164e-21,13.1525798819779
"Tsc22d4",6.3571472178785e-26,0.48497884905321,0.503,0.185,1.140090782
05433e-21,23.3872218783308
"Gsta2",7.86006628681438e-26,-1.37870693955207,0.065,0.498,1.409624287
87729e-21,-66.1930488918697
"Cald1",9.8567949153284e-26,0.650977288642301,0.206,0.039,1.7677176001
15e-21,31.1066878690468
"Atp5g1",1.13612374753437e-25,-0.612610737260504,0.884,0.926,2.0375243
2882814e-21,-29.1863385664797
"Pfkp",1.28256072695755e-25,0.384909879680784,0.406,0.127,2.3001444077
2567e-21,18.2914236566066
"Pck1",1.49014508844085e-25,-1.40680991073547,0.09,0.511,2.67242620160
982e-21,-66.6424146104697
"Nfe2l1",1.50973695118723e-25,0.513057982397357,0.574,0.228,2.70756224

825918e-21,24.2975221726974
"Tra2a",1.54341051954729e-25,0.568693777267542,0.548,0.219,2.767952425
75611e-21,26.9197904952102
"Set",1.58844661614915e-25,0.581871367596473,0.755,0.383,2.84872016140
188e-21,27.5268315003469
"Bok",1.62461254857642e-25,0.424497229451942,0.168,0.026,2.91358014461
695e-21,20.0723108862249
"2010107E04Rik",1.70192041293461e-25,-0.625323094601,0.923,0.943,3.052
22406855693e-21,-29.5392727430501
"Cox6c",2.6471418457593e-25,-0.501054886637009,0.968,0.981,4.747384186
18472e-21,-23.4477117983779
"Clk1",2.99764752124935e-25,0.565125115714172,0.587,0.248,5.3759810646
0858e-21,26.3757148614916
"Ophn1",3.52265913762912e-25,0.297056945962147,0.2,0.037,6.31753689742
407e-21,13.8164030042023
"Pecr",3.64009989607061e-25,-1.21349170493732,0.09,0.515,6.52815515361
304e-21,-56.4008647371072
"Egr1",3.80138768312955e-25,0.73045590747106,0.323,0.088,6.81740867092
454e-21,33.918579460516
"Calm1",3.87965374925812e-25,0.644393802456703,0.877,0.575,6.957771033
91952e-21,29.9091696083054
"Arglu1",4.18088335044175e-25,0.53513436984838,0.677,0.296,7.497996200
68223e-21,24.7979400124784
"Gm26699",4.89152263492211e-25,0.407090260629823,0.316,0.082,8.7724566
9346931e-21,18.8005151917281
"H2-
K1",5.0209270615664e-25,0.676534166644705,0.813,0.433,9.00453059221318
e-21,31.2264892479804
"Serpinh1",6.40705734020647e-25,0.592879026666185,0.574,0.241,1.149041
66339263e-20,27.2207205241673
"Tmc4",6.49787723973878e-25,0.440939405050256,0.355,0.104,1.1653293041
7475e-20,20.2385446540828
"Adgrg1",7.13097437889925e-25,0.585968531089815,0.677,0.31,1.278868945
11179e-20,26.8407138679302
"Litaf",9.104490913951e-25,0.473984733025512,0.516,0.185,1.63279940050
797e-20,21.5954108091826
"Emp2",9.12296851067352e-25,0.391491840885251,0.284,0.071,1.6361131727
0419e-20,17.8361249380479
"Gcnt1",1.10319722474349e-24,-1.13515631541709,0.071,0.495,1.978473902
85498e-20,-51.5013337706834
"Ier2",1.21830202130724e-24,0.734244610189145,0.381,0.119,2.1849028450
124e-20,33.2393493198693
"Ndrg1",1.21884933597456e-24,-0.85283776642569,0.581,0.83,2.1858843991
3678e-20,-38.6076939326594
"Luc7l2",1.24416088346019e-24,0.535206817571948,0.735,0.35,2.231278128
39751e-20,24.2176414131029
"Srrm2",1.47445981111123e-24,0.645926256914058,0.755,0.397,2.644296225
24688e-20,29.1179015157059
"Cox8a",1.61684154081453e-24,-0.506198279892014,0.961,0.976,2.89964361
929678e-20,-22.7723997344009

"Rbm25",1.86455268283854e-24,0.533217302955192,0.671,0.313,3.343888781
40263e-20,23.9118992942103
"Wsb1",2.3955620219035e-24,0.604991248144343,0.497,0.191,4.29620093008
174e-20,26.9789620130669
"Utrn",2.42747680959685e-24,0.449191746373017,0.342,0.1,4.353436910330
99e-20,20.0252987628318
"Sorbs2",3.04797524870113e-24,0.410748025645754,0.323,0.089,5.46623881
102061e-20,18.2179528086131
"Ctnna1",3.37229498080133e-24,0.601451057473542,0.69,0.336,6.047873818
56911e-20,26.6154092389185
"Tuba1a",3.92233618336808e-24,0.505585717157855,0.406,0.13,7.034317711
25231e-20,22.2967857397375
"Klf4",3.96572763980006e-24,0.537390177984643,0.206,0.04,7.11213594921
742e-20,23.6934788033386
"Paqr5",4.04618546951891e-24,0.570388371327958,0.671,0.296,7.256429021
0352e-20,25.1369094449815
"Kdm1a",4.39711517235417e-24,0.393294512727796,0.361,0.109,7.885786350
09998e-20,17.299704112727
"Wbp5",4.69141646768858e-24,0.558281882619892,0.787,0.396,8.4135862931
527e-20,24.5207753642806
"Atrx",4.99307409920994e-24,0.558940982454248,0.665,0.301,8.9545790895
2311e-20,24.5148926048083
"Cml1",5.28039306789846e-24,-1.22725345219171,0.142,0.547,9.4698569279
691e-20,-53.7581046659022
"Cox6b1",5.42090412692169e-24,-0.547661228151399,0.961,0.956,9.7218494
6122137e-20,-23.9751441145236
"F2rl1",5.58521317059523e-24,0.42989501474199,0.335,0.096,1.0016521300
1455e-19,18.8068175410539
"H3f3a",5.8385320471077e-24,0.579000775619468,0.955,0.687,1.0470823373
2829e-19,25.3041341219864
"Relb",6.91352823268685e-24,0.297114476789306,0.226,0.048,1.2398721532
5006e-19,12.9346138679223
"Prrc2c",7.10924954203138e-24,0.514968237846638,0.606,0.261,1.27497281
286791e-19,22.4043069842088
"Rtn4",8.03568372887179e-24,0.709660708144842,0.716,0.365,1.4411195199
3587e-19,30.7877047879034
"Pou3f3",1.24783267192898e-23,0.441275763637388,0.458,0.16,2.237863113
83743e-19,18.9499678196564
"Ildr1",1.34241063264868e-23,0.388679016753327,0.342,0.1,2.40747922859
214e-19,16.6628779861779
"Bmp3",1.62341280080537e-23,0.210050566234849,0.097,0.01,2.91142851696
434e-19,8.96505750125595
"Sfpq",1.76899189619353e-23,0.493395687365832,0.69,0.325,3.17251006663
348e-19,21.0159888234537
"Csrp1",2.34809180055834e-23,0.472330274123325,0.381,0.119,4.211067835
12132e-19,19.984955400867
"Mef2a",2.48238651356622e-23,0.438199853893205,0.432,0.151,4.451911973
42966e-19,18.5164779832072
"Cela1",2.57713685944186e-23,-1.23458259141321,0.168,0.538,4.621837243
72303e-19,-52.122008428114

"Scel",2.770111014425e-23,0.198382496257136,0.084,0.007,4.967917093269
8e-19,8.36105172155305
"Tpm3",2.93380180403867e-23,0.59519257185277,0.606,0.279,5.26148015536
294e-19,25.0508842066952
"Gm9844",3.10173939738613e-23,0.258882545690276,0.2,0.04,5.56265943527
228e-19,10.8816205166841
"Stap2",3.40971446874739e-23,0.325197745938699,0.258,0.062,6.114981928
25157e-19,13.6382650030693
"Mmp14",4.20433451546797e-23,0.255726335849184,0.194,0.037,7.540053520
04025e-19,10.6711754960478
"Srrm1",4.3370067139588e-23,0.542817553483538,0.613,0.275,7.7779878408
1371e-19,22.6343081464397
"Ndufv3",4.71670790910383e-23,-0.760408237723028,0.658,0.817,8.4589439
6418681e-19,-31.6435466059624
"Cd14",5.22191485275625e-23,0.481396032005516,0.297,0.081,9.3649820969
3305e-19,19.9837791596321
"Atp5k",5.22396932513728e-23,-0.617832956587866,0.813,0.903,9.36866658
770119e-19,-25.6473247564612
"Ccnl1",5.80151665850346e-23,0.564102234011911,0.471,0.176,1.040443997
53601e-18,23.3577158418465
"Capn2",6.02749288300009e-23,0.38872128670239,0.406,0.137,1.0809705736
3724e-18,16.0808835477956
"Pabpc1l",6.17339017430182e-23,0.14277188082002,0.058,0.004,1.10713579
385929e-18,5.90286848469703
"Arc",7.24378204675285e-23,0.0926787701810165,0.058,0.004,1.2990998722
6466e-18,3.81696217999314
"Ctsc",7.44373583646324e-23,0.543209466904593,0.49,0.187,1.33495958491
132e-18,22.3572146076875
"Nfe2l2",9.16654131890086e-23,0.507471503162264,0.574,0.241,1.64392752
013168e-18,20.7806756292182
"Kctd1",9.43320049893653e-23,0.46025471524829,0.387,0.125,1.6917501774
7928e-18,18.8339764571127
"Pdcd4",1.09523905877471e-22,0.512829405797172,0.471,0.177,1.964201728
00656e-18,20.9087963007467
"Fat1",1.34495101088983e-22,0.416449881108833,0.342,0.103,2.4120351429
2981e-18,16.8937312076652
"Cox4i1",1.43580731196168e-22,-0.451677123442638,0.981,0.983,2.5749768
3327209e-18,-18.2932356700206
"Agrn",1.44316659423202e-22,0.418357072120368,0.381,0.123,2.5881749700
9571e-18,16.9416117351168
"Slc44a2",1.55403449503082e-22,0.306175919593086,0.271,0.069,2.7870054
6338828e-18,12.3761095184002
"Pter",1.6043873783507e-22,-1.05035857013768,0.058,0.454,2.87730832433
414e-18,-42.4236427795265
"Wwc1",1.73466903120188e-22,0.585450038217607,0.632,0.298,3.1109554405
5746e-18,23.6004288022862
"Cldn8",1.76348461891173e-22,0.65745169866618,0.465,0.168,3.1626333155
563e-18,26.4920994742749
"Sphk1",1.92071433305791e-22,0.223056482932216,0.155,0.025,3.444609084
90605e-18,8.9690390014962

"Casp4",2.14710890720036e-22,0.22625579931267,0.148,0.023,3.8506251141
7313e-18,9.07247205134886
"Smagp",2.18287211808862e-22,0.497619803721675,0.555,0.231,3.914762856
58012e-18,19.9454859634063
"Col4a1",2.34984399495938e-22,0.576047868342081,0.497,0.197,4.21421022
056016e-18,23.0465631367078
"Cat",2.63270121983053e-22,-1.03052266195477,0.226,0.576,4.72148636764
407e-18,-41.1120915452112
"Dcxr",2.84628141661023e-22,-1.00182852245346,0.2,0.563,5.104521092548
79e-18,-39.889210228755
"Hnrnpdl",2.96716184726964e-22,0.426296792144274,0.484,0.185,5.3213080
5689337e-18,16.9558749877204
"Luc7l3",3.66002484956532e-22,0.435806812807421,0.587,0.251,6.56388856
521045e-18,17.2426740948552
"Tpm4",4.06490954512155e-22,0.491996569937975,0.51,0.205,7.29000877822
099e-18,19.4141978961341
"Aldh6a1",4.48833253736583e-22,-1.00233445987232,0.174,0.544,8.0493755
7251189e-18,-39.4528236824261
"Tnfrsf12a",4.6122398285961e-22,0.531278658400908,0.394,0.131,8.271590
90860425e-18,20.8971579226191
"Itm2c",4.66377576209021e-22,0.432172453585568,0.471,0.18,8.3640154517
3258e-18,16.9941415175693
"Zcchc7",5.15117979692127e-22,0.521369045985557,0.471,0.184,9.23812584
779861e-18,20.4497585267745
"Rbbp6",5.40740361336797e-22,0.390320946791342,0.516,0.205,9.697637640
21412e-18,15.2906862285275
"Ugt3a2",7.2589625312091e-22,-1.07657397617063,0.058,0.445,1.301822340
34704e-17,-41.8573915892889
"Plet1",7.7947347128232e-22,0.602629262393503,0.355,0.11,1.39790772339
771e-17,23.3874209324594
"Cd82",9.10785848362194e-22,0.444983227319663,0.484,0.182,1.6334033404
5276e-17,17.200061638598
"Hoxb5os",9.69553600840378e-22,0.366023739945992,0.381,0.122,1.7387974
2774713e-17,14.1251316818626
"Rsrc2",1.18705469481829e-21,0.515697932019877,0.6,0.271,2.12886388968
712e-17,19.7967969005581
"Gas2",1.25608312921348e-21,-1.01491047993571,0.039,0.43,2.25265948393
146e-17,-38.9033811275351
"Gpx8",1.3981960016346e-21,0.328077313210188,0.323,0.095,2.50752470933
149e-17,12.5406406298202
"Abcg2",1.4781102680076e-21,-1.02951940998371,0.103,0.484,2.6508429546
4483e-17,-39.2957972888095
"Cd2ap",1.62628638035347e-21,0.550532671004244,0.645,0.307,2.916581994
52591e-17,20.9607244777343
"Krit1",1.85427931757661e-21,0.515802469090783,0.51,0.212,3.3254645281
4188e-17,19.5707512153934
"Akap9",1.95831120768434e-21,0.48830159788383,0.574,0.256,3.5120353198
611e-17,18.5006492794339
"Ndufc1",1.96862261601434e-21,-0.64545063480255,0.735,0.852,3.53052779
956012e-17,-24.451283158381

"Gpx4",2.28779157180515e-21,-0.615096502235427,0.903,0.939,4.10292540487535e-17,-23.2089727502842
"Golgb1",2.30446343974278e-21,0.487558295662379,0.613,0.279,4.1328247328347e-17,18.3931296113697
"Dab2",2.54838444560546e-21,-0.931109950875656,0.194,0.56,4.57027266474883e-17,-35.0324287603032
"Tmem165",2.56750048974031e-21,0.401768718328339,0.419,0.15,4.60455537830027e-17,15.1132938884313
"Klf2",2.63715351512222e-21,0.392892795213749,0.284,0.077,4.7294711140202e-17,14.7688925053519
"Srsf11",2.65932450055656e-21,0.494098230156341,0.632,0.296,4.76923255929813e-17,18.5690816841975
"Dnase1",2.7317436061219e-21,-1.3568873460626,0.097,0.47,4.89910898321902e-17,-50.9577594098792
"Atp5l",2.77224027325994e-21,-0.500666919085758,0.942,0.952,4.97173570606437e-17,-18.7951250434642
"Pmepa1",2.79696639004248e-21,0.468457951148751,0.426,0.154,5.01607952390219e-17,17.5818349292647
"Atp2b1",3.55793985706782e-21,0.493534591961427,0.568,0.251,6.38080933966543e-17,18.4042265390703
"Ptpn18",4.89606829489451e-21,0.404999929961778,0.458,0.173,8.78060888006381e-17,14.9734147455763
"Ifitm3",5.49325771859716e-21,0.519771058830735,0.632,0.283,9.85160839253215e-17,19.1568442351109
"Fos",6.37208576176919e-21,1.44474941143234,0.387,0.14,1.14276986051569e-16,53.0337262678007
"Ptpn14",6.65555186405467e-21,0.313770769414051,0.226,0.054,1.19360667129957e-16,11.504211333986
"Hbegf",7.43583541951636e-21,0.628053543957956,0.335,0.104,1.33354272413606e-16,22.9575692778453
"Dnajc8",8.49801442508126e-21,0.423309814692526,0.561,0.245,1.52403390699407e-16,15.4169437822164
"Zmynd8",8.74004866021655e-21,0.499196498940048,0.458,0.18,1.56744032672324e-16,18.1667178377538
"Gnl2",9.9511446992418e-21,0.337211318378026,0.277,0.076,1.78463829036202e-16,12.2280059693891
"Vmp1",1.16578893123034e-20,0.601079627156021,0.652,0.329,2.09072586926849e-16,21.7012888029001
"Ppl",1.30105763482882e-20,0.239855035927272,0.148,0.026,2.33331676230201e-16,8.63335912797221
"Ifit3b",1.32867313799568e-20,0.331346805758185,0.11,0.014,2.38284240568145e-16,11.919560984503
"Psme1",1.4137551205336e-20,0.446417833610005,0.6,0.275,2.53542843316496e-16,16.031310312949
"Pinx1",1.53212282370624e-20,0.29137938136237,0.187,0.039,2.74770907203478e-16,10.4402963101082
"Hoxb5",1.59863107674267e-20,0.277308960771467,0.232,0.056,2.8669849730303e-16,9.9243609693905
"Gm6483",1.61705697096464e-20,0.416475410473529,0.452,0.174,2.90002997172799e-16,14.9000910025704

"Hsp90aa1",1.79325544387412e-20,0.593124827364977,0.903,0.592,3.216024
31304385e-16,21.1586698304189
"Nbl1",2.46440809042891e-20,0.376836600573166,0.142,0.024,4.4196694693
7521e-16,13.3231697736905
"Fosb",2.52361407600381e-20,0.649231636203864,0.161,0.03,4.52584948390
524e-16,22.9383640394865
"Slc17a1",2.7065160690114e-20,-1.01658779798098,0.058,0.429,4.85386591
816504e-16,-35.8464980690614
"Cda",2.85532910184502e-20,-0.981611768512268,0.084,0.462,5.1207472112
4886e-16,-34.5606471416992
"Hspe1",3.14880634958932e-20,-0.726061744337426,0.742,0.828,5.64706930
735349e-16,-25.4921910507388
"Cyba",3.16237675542831e-20,-0.784043606789222,0.806,0.853,5.671406473
18513e-16,-27.5245755436645
"Fmo1",3.53225090578432e-20,-0.95834901233212,0.026,0.397,6.3347387744
336e-16,-33.5377235638131
"RP24-64C6.3",3.64652166468184e-20,-1.039848129382,0.077,0.444,6.53967
195344041e-16,-36.3567035385897
"Atp5j2",4.06090236672668e-20,-0.483811407425014,0.929,0.945,7.2828223
0448764e-16,-16.8636544692833
"Jup",4.07360704783353e-20,0.397686653201838,0.516,0.214,7.30560687958
466e-16,13.8604613207869
"Mfge8",4.30429752933655e-20,0.319859311157745,0.355,0.115,7.719327189
11217e-16,11.1303473305074
"Tia1",5.02764115365323e-20,0.497352976354442,0.477,0.195,9.0165716449
6171e-16,17.2294496747783
"Kcnj15",6.28982665994784e-20,-0.954100114893105,0.045,0.413,1.1280175
1319505e-15,-32.8385180250325
"4931406C07Rik",8.07981356808902e-20,-1.0353541585802,0.271,0.579,1.44
903376530109e-15,-35.3758560570531
"Tnfaip2",8.45113079662873e-20,0.410368618371067,0.297,0.086,1.5156257
970674e-15,14.0029870147836
"Camkk2",8.61138898341655e-20,0.330481570921562,0.277,0.079,1.54436650
028592e-15,11.2707972312849
"Pycard",9.02659828726362e-20,0.439389547383133,0.49,0.196,1.618830136
83786e-15,14.9643217343681
"Ctsd",9.22748758068477e-20,0.811023384199181,0.897,0.627,1.6548576227
2001e-15,27.6032306929374
"Raph1",9.26032785240647e-20,0.31636575023742,0.252,0.067,1.6607471970
5058e-15,10.766403805349
"Ctss",9.33392113417415e-20,0.455424573559654,0.477,0.192,1.6739454162
0279e-15,15.4951803467385
"Hdgfrp3",1.06206457701422e-19,0.234981497333142,0.2,0.045,1.904706612
4173e-15,7.96456823805704
"Gapdh",1.14328521805877e-19,-0.558340622057045,0.884,0.919,2.05036771
006661e-15,-18.8835026397756
"Zscan21",1.30661428600961e-19,0.195376869946776,0.219,0.052,2.3432820
6052963e-15,6.58170435462743
"Clec2d",1.31928653143426e-19,0.390838647348682,0.374,0.13,2.366008465
47419e-15,13.1624967065539

"Wls",1.46195435320949e-19,0.471993005598015,0.781,0.427,2.62186893704591e-15,15.8471127701673
"mt-Co1",1.52596104647798e-19,-0.399403662062821,1,1,2.7366585407536e-15,-13.3928190649518
"Gk",1.60108215207645e-19,-0.934151634754816,0.052,0.417,2.87138073153391e-15,-31.279117821591
"Serinc2",1.66303730391487e-19,0.350275853465195,0.284,0.084,2.98249110084093e-15,11.7153323261263
"Plec",1.69870200019715e-19,0.411797992720099,0.394,0.144,3.04645216715358e-15,13.7642650138934
"Specc1",1.80658811846057e-19,0.306385577620544,0.29,0.086,3.23993513164719e-15,10.2220103766108
"Gnai2",1.98327318253322e-19,0.439951629615068,0.645,0.31,3.55680212555508e-15,14.6371531159825
"Mapk11",2.00617330035127e-19,0.104871828855387,0.065,0.005,3.59787119684997e-15,3.48787280458791
"Akr7a5",2.00861609270832e-19,-0.940889542047918,0.284,0.586,3.6022521006631e-15,-31.2913678248972
"Chrnb1",2.46516335773661e-19,0.237581544822869,0.168,0.034,4.42102396576483e-15,7.85264145407333
"Mpped2",2.6947850042916e-19,0.344257162590921,0.2,0.045,4.83282742669656e-15,11.3478675193605
"Ptrf",2.86029695730337e-19,0.29853482236349,0.206,0.049,5.12965656322786e-15,9.82291148479923
"Ndufa6",2.97649905116934e-19,-0.569955691385378,0.89,0.896,5.3380533983671e-15,-18.7309755895466
"Ptpn13",3.14763641655055e-19,0.50552298999681,0.387,0.146,5.64497114944175e-15,16.5852039723393
"Slc22a1",3.17799845317458e-19,-0.912370977422757,0.045,0.404,5.69942242592328e-15,-29.9243188074274
"2200002D01Rik",3.50374609070792e-19,0.575896159566852,0.445,0.176,6.28361823907558e-15,18.8322826579154
"Slc34a2",3.54423070824291e-19,0.367034866257743,0.181,0.038,6.35622335216284e-15,11.998128271405
"Zfhx3",4.4322962586227e-19,0.379211200466544,0.31,0.098,7.94888011021395e-15,12.3113742654869
"Eif4ebp1",4.54475381608648e-19,0.347910765983228,0.394,0.144,8.15056149376949e-15,11.2864651590937
"Selenbp1",5.05413302277694e-19,-0.900805421155363,0.174,0.52,9.06408216304816e-15,-29.1270539813722
"Itpkb",5.36463420123553e-19,0.295789118156596,0.239,0.062,9.62093497649581e-15,9.54654496675078
"Sf3b1",5.66066865580235e-19,0.477870595223161,0.606,0.297,1.01518431673159e-14,15.3975263250603
"Rab25",5.96768126880946e-19,0.325614947508834,0.316,0.101,1.07024395874829e-14,10.4744808400219
"Rock2",6.42423042716609e-19,0.374331538908646,0.361,0.126,1.15212148480797e-14,12.0140158764375
"Vgll4",7.01380108508925e-19,0.32830595092188,0.316,0.101,1.2578550865

9991e-14,10.5080174416222
"Ddt",7.61025428536032e-19,-0.819935533292869,0.342,0.635,1.3648230035
3652e-14,-26.1765784601674
"Shisa4",7.75243436355291e-19,0.335410891719669,0.348,0.117,1.39032157
875958e-14,10.7018400178658
"Cd47",8.44399859235072e-19,0.559527014677585,0.645,0.338,1.5143467075
5218e-14,17.8048250524381
"Cd74",8.81666267738682e-19,0.470023882895013,0.923,0.574,1.5811802845
6255e-14,14.9364282186669
"Rasl11b",1.0282904494919e-18,0.335312429683441,0.168,0.034,1.84413609
211877e-14,10.603980758905
"Fzd4",1.1397035436702e-18,0.407901674691219,0.381,0.137,2.04394433521
813e-14,12.8575950816623
"Ankrd1",1.18085932652871e-18,0.511764291288195,0.103,0.014,2.11775311
619659e-14,16.1133263548192
"Btbd9",1.19976822024897e-18,0.342009583063193,0.271,0.08,2.1516643261
945e-14,10.7630243601963
"Cttn",1.23601988359854e-18,0.397341198221626,0.477,0.202,2.2166780592
4562e-14,12.4924794278274
"Slc14a2",1.28691267468575e-18,0.107583033354261,0.052,0.004,2.3079491
9078143e-14,3.37808916992141
"Rbm6",1.31062663986157e-18,0.325045093692446,0.387,0.142,2.3504778159
2774e-14,10.2004262282495
"Cd59a",1.31688599567431e-18,0.344026044151575,0.394,0.143,2.361703344
64231e-14,10.7944391986817
"Dag1",1.49035671964211e-18,0.547285257905175,0.49,0.218,2.67280574100
616e-14,17.1043403935821
"Cpne2",1.49126263972286e-18,0.196287513871606,0.129,0.021,2.674430418
07897e-14,6.13446666922159
"Zfp644",1.51243044483875e-18,0.347066343907283,0.381,0.136,2.71239275
977381e-14,10.8417834362989
"Lactb2",1.54579530032719e-18,-0.886496063207622,0.135,0.478,2.7722292
9160678e-14,-27.673339379903
"Tapbp",1.5743934063016e-18,0.406483715640251,0.516,0.228,2.8235171348
613e-14,12.6815635023059
"Mt1",1.61365772234732e-18,0.972915960972204,0.729,0.445,2.89393375925
768e-14,30.329268252862
"Ddx5",1.77146449270162e-18,0.47879136347213,0.877,0.581,3.17694442121
109e-14,14.8809655402689
"Hsp90ab1",1.82604231679663e-18,0.487963145784003,0.961,0.763,3.274824
29094308e-14,15.1512200971944
"Dao",1.85056798661318e-18,-0.906811605023276,0.084,0.431,3.3188086271
9207e-14,-28.1443358487831
"Mpc1",2.02946096097158e-18,-0.641420318102055,0.677,0.833,3.639635287
40642e-14,-19.8483077660293
"Fndc3b",2.12451762737831e-18,0.287012616747808,0.342,0.114,3.81010991
294026e-14,8.86826888595737
"Slc22a18",2.39152444116338e-18,-0.967826088804582,0.097,0.439,4.28895
99327824e-14,-29.7898293208893
"Plp2",2.48422110669884e-18,0.393514772494065,0.342,0.119,4.4552021327

537e-14,12.0974779130791
"Nfkbia",3.11659234742445e-18,0.715534387809298,0.432,0.178,5.58929671
587101e-14,21.8347735756868
"Pip5k1a",3.33442990557119e-18,0.305846328612718,0.284,0.088,5.9799665
9265137e-14,9.31234060204094
"Tanc1",3.54217722302193e-18,0.48734005449187,0.452,0.192,6.3525406317
6754e-14,14.8089662109282
"Zfp329",3.63159321828537e-18,0.359712925901583,0.329,0.111,6.51289927
767299e-14,10.9217501109258
"Ndufa13",3.68263159895066e-18,-0.48522770578055,0.897,0.927,6.6044315
0955811e-14,-14.7259089797138
"Slc5a2",3.70718712125966e-18,-1.11906225686982,0.103,0.442,6.64846938
326707e-14,-33.9543683798118
"Hoxb2",3.71142932208381e-18,0.400599151123274,0.316,0.105,6.656077346
22511e-14,12.1544430322813
"Tspan12",4.09046558517812e-18,0.339353073019536,0.316,0.103,7.3358409
8045844e-14,10.263197259001
"B230219D22Rik",4.15657142542352e-18,0.470837385212087,0.529,0.247,7.4
5439519435454e-14,14.2321840849987
"Noxo1",4.36031513610814e-18,0.115059317294454,0.077,0.008,7.819789165
09633e-14,3.4724365447889
"Fam129a",5.54626930047955e-18,0.313865017752839,0.252,0.072,9.9466793
6348003e-14,9.39678987007954
"Plpp3",5.60655784118772e-18,0.459815583593296,0.316,0.106,1.005480083
23861e-13,13.7614256596465
"Csnk1a1",5.61425364733694e-18,0.487600473462617,0.69,0.373,1.00686024
911341e-13,14.5923069209656
"Hspa1a",5.78307488305113e-18,0.651103164134889,0.348,0.122,1.03713664
952639e-13,19.4661240897594
"Sumo2",6.02443056719342e-18,0.471700463818169,0.852,0.541,1.080421377
92047e-13,14.0832093687038
"Gpm6b",6.02623689127533e-18,0.238068476863266,0.194,0.045,1.080745324
08132e-13,7.10776180140616
"Mdh1",6.63305117449997e-18,-0.603091249494174,0.768,0.879,1.189571397
63482e-13,-17.948003507594
"Edn1",7.33494353735962e-18,0.347676884604955,0.245,0.068,1.3154487739
9007e-13,10.3118976404227
"Aoc1",9.09928377316099e-18,0.570286843269978,0.252,0.072,1.6318655518
7869e-13,16.7914587122656
"C1qb",9.16532485176556e-18,0.49996741446414,0.548,0.25,1.643709358915
64e-13,14.7173661494117
"Hoxd8",9.2653012206738e-18,0.39526936690764,0.484,0.2,1.6616391209156
4e-13,11.6311179974422
"4833439L19Rik",9.47104246879502e-18,-0.84380489457319,0.394,0.631,1.6
985367563537e-13,-24.8111033181203
"Dstn",1.1188534899539e-17,0.556880862131294,0.839,0.567,2.00655184888
332e-13,16.281630714826
"Adgra1",1.14462062738195e-17,0.199871445738438,0.071,0.007,2.05276263
314679e-13,5.83912830126484
"Wnt7b",1.17851330732962e-17,0.200037809139281,0.123,0.02,2.1135457653

6495e-13,5.83815131163533
"Arhgef1",1.26688519074195e-17,0.311907581101964,0.316,0.106,2.2720319
0107661e-13,9.08054409510169
"Cadps2",1.27289013214207e-17,0.243001806036853,0.226,0.06,2.282801162
98359e-13,7.07334588485559
"Gtpbp2",1.33455320867356e-17,0.433561978813799,0.342,0.122,2.39338772
443515e-13,12.5996997443883
"Nudcd3",1.47451930671165e-17,0.395374373540857,0.458,0.194,2.64440292
465668e-13,11.4505008504095
"Ehd4",1.54839872862639e-17,0.45352698075283,0.277,0.088,2.77689827991
857e-13,13.1124952594017
"Morf4l1",1.55962467319094e-17,0.478937885273894,0.845,0.559,2.7970308
8890063e-13,13.8437224436916
"Nrp1",1.62753451528985e-17,0.562479470814355,0.361,0.132,2.9188203997
2082e-13,16.2345224668
"Anp32b",1.72455158396779e-17,0.391952676851454,0.587,0.272,3.09281081
068783e-13,11.2900110256785
"Umod",1.83940225183613e-17,0.707597114540808,0.8,0.521,3.298783998442
91e-13,20.3363780747584
"Ppp4r2",1.86514132261463e-17,0.344236608856121,0.439,0.177,3.34494444
797707e-13,9.8885945692396
"Syne1",1.93330212858363e-17,0.300267500775085,0.219,0.057,3.467184037
40188e-13,8.61475370601939
"Slc47a1",2.0587372061798e-17,-0.842939282389981,0.013,0.348,3.6921393
0556285e-13,-24.1311601486229
"Ctxn1",2.19815723481396e-17,0.19550948283628,0.168,0.036,3.9421751849
1536e-13,5.58411712866063
"Sod2",2.29923823930889e-17,-0.683389599870027,0.516,0.732,4.123453858
37657e-13,-19.4881631871497
"Rbm5",2.42434262924002e-17,0.406274985894606,0.477,0.207,4.3478160712
7906e-13,11.5641837921979
"Cdo1",2.52431656400105e-17,0.471487842646544,0.381,0.143,4.5271093258
7949e-13,13.4013453468548
"Itga6",2.54911085636776e-17,0.579798147537849,0.555,0.281,4.571575409
80994e-13,16.4742386491226
"Itgav",2.61334307606474e-17,0.60691120879299,0.6,0.316,4.686769472614
5e-13,17.2295189627148
"Erbb4",2.72286709081555e-17,0.229595867530246,0.219,0.057,4.883189840
66861e-13,6.50853946627028
"Timp3",2.80163935486567e-17,-0.750525546492229,0.652,0.798,5.02446001
901609e-13,-21.2543492673266
"H2-
Aa",2.9127862234918e-17,0.525103975085841,0.729,0.408,5.22379081321019
e-13,14.8501414573463
"Cyr61",2.95577872269452e-17,0.799226779676079,0.258,0.076,5.300893561
28034e-13,22.5907290073561
"Scd2",3.0175221494595e-17,0.400870167560468,0.432,0.165,5.41162422284
066e-13,11.3226007063243
"Sephs2",3.19993415748833e-17,-0.838994273980964,0.206,0.516,5.7387619
1803957e-13,-23.6481969227202

"S100a13",3.29900584418349e-17,0.36265866745983,0.542,0.24,5.916437080
95867e-13,10.2109709429502
"Ankrd12",3.32314954903188e-17,0.364617908501305,0.465,0.197,5.9597364
0123377e-13,10.2634763511317
"Psmb8",3.39651616893009e-17,0.399492039877364,0.394,0.152,6.091312097
35923e-13,11.2364098160414
"Col18a1",3.63281899137691e-17,0.459753353437713,0.542,0.249,6.5150975
7913535e-13,12.9004418743163
"Csnk1e",3.80064573286277e-17,0.313481925049223,0.245,0.071,6.81607805
731609e-13,8.7819835792862
"Tjp1",3.94675223094998e-17,0.304241542683226,0.245,0.071,7.0781054509
857e-13,8.51164392133751
"Myo1b",4.15612243277857e-17,0.227373314424831,0.168,0.037,7.453589970
94508e-13,6.34937943659824
"S100a16",4.57082786722525e-17,0.417990276305877,0.51,0.231,8.19732269
708176e-13,11.6325852098291
"Pdzk1ip1",4.57703499493192e-17,-0.53288158003048,0.839,0.9,8.20845455
991091e-13,-14.829263898645
"Ube2i",4.64789263134747e-17,0.430782626912483,0.51,0.236,8.3355306450
5856e-13,11.981391247099
"Hnrnpa2b1",4.72171740515704e-17,0.389752106748082,0.929,0.626,8.46792
799440864e-13,10.83406417185
"Anxa1",5.67457383231005e-17,0.521301737770621,0.213,0.056,1.017678071
08648e-12,14.3949642245775
"Ndufb9",6.12700335735127e-17,-0.531579602706888,0.826,0.872,1.0988167
8210738e-12,-14.6379943824212
"Pax8",7.16154729164086e-17,0.547617391412461,0.394,0.16,1.28435189128
287e-12,14.9941841389426
"Tns3",7.57884406211389e-17,0.459730461156559,0.4,0.161,1.359189894099
51e-12,12.5617359244839
"Ucp2",7.71845401264308e-17,0.490712643305203,0.452,0.198,1.3842275426
2741e-12,13.3993399945529
"Slc17a3",7.80982338883421e-17,-0.889703010302296,0.006,0.329,1.400613
72655353e-12,-24.2836523636822
"RP23-306P12.2",7.8879325219387e-17,-0.861944331721511,0,0.322,1.41462
181848449e-12,-23.5174260981319
"Eif1a",8.15978346712945e-17,0.422085265500281,0.426,0.181,1.463375566
995e-12,11.5019396919856
"Nono",8.19707966577118e-17,0.442326424053285,0.548,0.27,1.47006426725
94e-12,12.0514996853402
"Mrps6",8.23250016029622e-17,0.553914566818552,0.529,0.255,1.476416578
74752e-12,15.0894100500926
"Sfxn3",8.25115519400703e-17,0.264817111757231,0.239,0.069,1.479762172
49322e-12,7.21339031146081
"Dip2b",8.39945165672308e-17,0.24008517063364,0.219,0.059,1.5063576601
1672e-12,6.53543682353978
"Hint2",8.96562575128908e-17,-0.709624393824409,0.555,0.745,1.60789532
223618e-12,-19.2706274852318
"Tmem123",9.58948633447264e-17,0.363581994437698,0.335,0.121,1.7197784
7922432e-12,9.84900924754751

"Defb1",9.92573725858768e-17,0.467460105981663,0.639,0.322,1.780081719
95512e-12,12.646834854204
"Msra",1.02187315574479e-16,-0.852460714157905,0.142,0.456,1.832627317
51271e-12,-23.0379813788629
"Crip1",1.12786907430601e-16,0.546821336461046,0.613,0.302,2.022720397
86039e-12,14.7240272473974
"Pak2",1.17796420434728e-16,0.408863947877391,0.445,0.196,2.1125610040
7641e-12,10.9915386392367
"Kif1b",1.18484523312136e-16,0.333038324919844,0.439,0.182,2.124901441
07984e-12,8.95116953673673
"G6pc",1.18608363134688e-16,-0.963492085365948,0.013,0.332,2.127122384
45749e-12,-25.8950551640786
"Slco4a1",1.21038341499961e-16,0.328695750291903,0.206,0.054,2.1707016
1646031e-12,8.82744343086175
"Ly6c1",1.21826225599527e-16,0.518200976057482,0.406,0.165,2.184831529
90192e-12,13.9134279828842
"Tmem174",1.2380952060859e-16,-0.832083121495793,0.071,0.4,2.220399942
59446e-12,-22.3275641334221
"Hif1a",1.25963042505342e-16,0.418265756599567,0.439,0.189,2.259021204
29081e-12,11.2162519603729
"Marcks",1.2619286440825e-16,0.397958465548578,0.271,0.085,2.263142830
29756e-12,10.6709644051949
"Pvrl2",1.28271767399878e-16,0.382633985802709,0.303,0.105,2.300425876
54941e-12,10.253797560494
"Rrbp1",1.42483111028875e-16,0.498956631709968,0.684,0.382,2.555292113
19185e-12,13.3185768063683
"Prmt1",1.48048435475597e-16,0.349734005607023,0.406,0.162,2.655100641
81935e-12,9.3219985365973
"H2-
Eb1",1.4820736226637e-16,0.521423371711933,0.697,0.383,2.6579508348850
8e-12,13.8977399285131
"Map4",1.56014569789696e-16,0.332453386836504,0.297,0.1,2.797965294608
4e-12,8.84396776456025
"Arhgef40",1.56725718307505e-16,0.192531771261718,0.155,0.032,2.810719
03212679e-12,5.12087933923439
"Pak6",1.57337692464051e-16,0.253192034193131,0.245,0.072,2.8216941766
503e-12,6.73330885737558
"Atxn7l3b",1.58730830660442e-16,0.413365876187189,0.671,0.333,2.846678
71706437e-12,10.9892773217858
"Trim2",1.61938425216796e-16,0.41263880907805,0.426,0.176,2.9042037178
3802e-12,10.9616929656303
"Scmh1",1.63319196561409e-16,0.261256997140102,0.245,0.072,2.928966471
13231e-12,6.93803787181343
"Ctnnal1",1.77963842679185e-16,0.280698067225624,0.245,0.072,3.1916035
5460851e-12,7.43021752709783
"Smpdl3a",1.844269482799e-16,-0.762973854496183,0.368,0.622,3.30751289
045174e-12,-20.1690800192051
"Cdkn2b",1.84594815296499e-16,0.185468074164042,0.11,0.018,3.310523417
52742e-12,4.90264727184424
"St3gal6",1.88005261897816e-16,0.357962172020326,0.31,0.107,3.37168636

687543e-12,9.45578844753659
"Slc22a8",1.9175205378902e-16,-0.937505696316112,0.026,0.342,3.4388813
3265229e-12,-24.74628306996
"Hnrnpr",2.13388727474065e-16,0.408905294598126,0.497,0.224,3.82691343
851988e-12,10.7496959673408
"Ifitm2",2.18535446466733e-16,0.510677243830254,0.703,0.408,3.91921469
693439e-12,13.4130041025689
"Sepw1",2.26126296595764e-16,0.404780756404565,0.742,0.414,4.055349003
14843e-12,10.6177976923811
"Ccdc80",2.3270066498357e-16,0.1983260226239,0.103,0.016,4.17325372581
535e-12,5.19660292343539
"Cyp2e1",2.4025400056431e-16,-1.10532506875721,0.071,0.388,4.308715246
12033e-12,-28.9267785573502
"Adgrg6",2.64346370547835e-16,0.130243056187983,0.103,0.016,4.74078780
940488e-12,3.39606395845139
"Ddit4",2.69441678207939e-16,0.445752716152175,0.258,0.081,4.832167056
98118e-12,11.6144106727443
"B4galt6",2.72424200682307e-16,0.228134931822291,0.271,0.084,4.8856556
1503649e-12,5.94170988076092
"Sec14l1",2.80559814006299e-16,0.317653744267268,0.316,0.111,5.0315597
0438897e-12,8.2638546259548
"Amn",3.19028035383672e-16,-0.816232743029846,0.032,0.353,5.7214487865
7078e-12,-21.1296530035716
"Pik3r1",3.2762146587699e-16,0.458622189664487,0.452,0.193,5.875563369
03794e-12,11.8600703399829
"Tmbim4",3.51452227503707e-16,-0.722389326539859,0.574,0.718,6.3029442
4805148e-12,-18.6304238021524
"Irx2",3.60261693027063e-16,0.273964143538572,0.168,0.038,6.4609332027
4735e-12,7.05875392328509
"Tgif1",3.64796115210278e-16,0.302579137219773,0.271,0.086,6.542253530
18113e-12,7.79224167878322
"Pbx1",3.69503117010685e-16,0.519737892997775,0.465,0.213,6.6266689004
6963e-12,13.3780111974916
"Lgmn",4.23512440508308e-16,-0.817351613267605,0.465,0.685,7.595272108
07599e-12,-20.9270580688046
"Hdac7",4.37395618486718e-16,0.327603019341263,0.258,0.081,7.844253021
9408e-12,8.37721541636727
"Hnf4a",4.39707209031091e-16,-0.770358424457836,0.019,0.335,7.88570908
676358e-12,-19.6949595762284
"Tmem213",5.1683157825618e-16,0.327748700221991,0.652,0.313,9.26885752
444633e-12,8.32624629389944
"0610011F06Rik",5.22201578656092e-16,-0.739892799002513,0.484,0.691,9.
36516311161835e-12,-18.7888557639027
"Cmbl",5.23235050294553e-16,-0.822006869886764,0.026,0.341,9.383697391
98251e-12,-20.8724372819034
"Arhgap29",5.24126298733903e-16,0.395606858496317,0.342,0.126,9.399681
04149382e-12,10.0445947636043
"Ccdc59",5.49567711229877e-16,0.295355467764544,0.394,0.155,9.85594733
319662e-12,7.4851776859505
"Pbxip1",5.75737059495185e-16,0.391774099193282,0.51,0.231,1.032526842

49867e-11,9.91048489283622
"Pafah1b1",5.99894832181887e-16,0.401733315048299,0.6,0.303,1.07585139
2035e-11,10.145904905478
"H2-
Q4",6.36077714084179e-16,0.245665875171479,0.2,0.052,1.14074177243857e
-11,6.1899834322488
"Cdv3",6.50156462280616e-16,0.404727793395948,0.49,0.226,1.16599059945
406e-11,10.1889675593805
"Plcb4",6.71814285338562e-16,0.253380069271079,0.213,0.059,1.204831739
32618e-11,6.37050605173099
"Hk2",6.78703895593208e-16,0.449529308490286,0.11,0.018,1.217187566356
86e-11,11.2975225258007
"Cited2",7.06523609909842e-16,0.508952998574126,0.432,0.187,1.26707944
201231e-11,12.7705068574612
"Ehd2",7.51029485013309e-16,0.187251483341965,0.123,0.022,1.3468962784
2287e-11,4.68702318265301
"Tgfbr1",7.89186833052306e-16,0.448454405878043,0.361,0.142,1.41532766
639601e-11,11.2028731259237
"Gm16136",8.04956180383246e-16,0.272844527637183,0.2,0.053,1.443608413
89931e-11,6.81055143403838
"H2-
Ab1",8.12165757636292e-16,0.561355176742149,0.781,0.503,1.456538069744
93e-11,14.0071440856457
"Efemp1",8.25865615132248e-16,0.309226813197068,0.265,0.085,1.48110739
417817e-11,7.71077034005756
"Mga",8.58456324298594e-16,0.387736760021895,0.335,0.126,1.53955557199
71e-11,9.65345971772569
"Ccl5",8.64865311473063e-16,0.258724961603152,0.348,0.129,1.5510494495
9579e-11,6.43953603317567
"Rrnad1",9.34385579829648e-16,0.227647419941348,0.232,0.067,1.67572709
886649e-11,5.64843077775287
"Chd6",9.47537663183295e-16,0.387456435325204,0.368,0.147,1.6993140451
5292e-11,9.60822657449594
"Sept1",9.53706921597224e-16,0.268600681756861,0.239,0.071,1.710377993
19246e-11,6.65907333126004
"Muc1",9.58437325850462e-16,0.403143015800689,0.329,0.12,1.71886150018
022e-11,9.99261476983032
"Sept2",9.61675996971873e-16,0.331130297111097,0.445,0.189,1.724669732
96936e-11,8.20653475440863
"Hp1bp3",1.0199918086576e-15,0.380910927181651,0.49,0.227,1.8292533096
4653e-11,9.41784294816848
"Nktr",1.03972375289767e-15,0.403584149814877,0.523,0.248,1.8646405784
4668e-11,9.97069482219338
"Smim24",1.08242015010742e-15,-0.795221587840619,0.052,0.36,1.94121229
720265e-11,-19.6142385543217
"Myo9a",1.15138225917427e-15,0.358657433170946,0.323,0.117,2.064888943
60313e-11,8.82417779373674
"Tra2b",1.17001260451789e-15,0.460149315935237,0.555,0.28,2.0983006049
4238e-11,11.3138330680403
"Hoxc8",1.17102327158124e-15,0.264437832422884,0.219,0.062,2.100113135

25379e-11,6.50158617141104
"Ccdc107",1.21284125141946e-15,-0.817124579300723,0.277,0.544,2.175109
50029565e-11,-20.0615172718966
"Tmem164",1.21519317459871e-15,0.230350310454565,0.213,0.059,2.1793274
3932533e-11,5.65496645117223
"Sec63",1.23058485811071e-15,0.320545403082953,0.439,0.188,2.206930884
53575e-11,7.86516909252072
"Trib1",1.27025912051535e-15,0.314517123498859,0.239,0.072,2.278082706
73222e-11,7.70727414548665
"Tmem52b",1.33078786231422e-15,0.571556430798615,0.439,0.183,2.3866349
5227432e-11,13.9794424657859
"Rassf6",1.37987530764659e-15,0.266934005137961,0.213,0.059,2.47466837
673339e-11,6.51915026162471
"Qdpr",1.42117691251048e-15,-0.809538067308412,0.213,0.499,2.548738674
89629e-11,-19.7469304639343
"Odc1",1.43001080115969e-15,-0.829310530868892,0.284,0.57,2.5645813707
9978e-11,-20.2240979996749
"Lrrfip1",1.43004621267232e-15,0.318139386715543,0.31,0.111,2.56464487
780655e-11,7.75834305929209
"Akr1b8",1.48935403078811e-15,0.269748520418641,0.174,0.042,2.67100751
88154e-11,6.56729215700684
"Lama5",1.49888912899523e-15,0.335661922950332,0.277,0.093,2.688107763
94004e-11,8.16987643981496
"Sprr1a",1.55362576778287e-15,0.50427243566426,0.103,0.017,2.786272451
94181e-11,12.255701808197
"Uhrf2",1.58308215971688e-15,0.36404554051778,0.374,0.15,2.83909954523
625e-11,8.84082741521188
"Acot13",1.58323418081239e-15,-0.759228604192606,0.297,0.569,2.8393721
7986894e-11,-18.4377550907231
"Pik3ip1",1.67810105601133e-15,0.258486618736969,0.213,0.059,3.0095064
3385072e-11,6.26226740939329
"Nedd4",1.67928925195982e-15,0.477067673407356,0.606,0.333,3.011637344
46474e-11,11.5574186157841
"Zc3h13",1.73504851852276e-15,0.367864707808078,0.374,0.15,3.111636013
11872e-11,8.89985659510317
"Gpatch2",1.76922243908867e-15,0.265306872462501,0.239,0.072,3.1729235
2226162e-11,6.4134706607186
"Alpl",1.82524115256947e-15,-0.792310321405834,0.032,0.337,3.273387483
01809e-11,-19.1284396010624
"Prpf4b",1.85445453161353e-15,0.331589241995056,0.51,0.238,3.325778756
99571e-11,8.00016482995246
"Cryl1",1.87193182921912e-15,-0.758737314946658,0.084,0.395,3.35712254
252158e-11,-18.29873474989
"Hint1",1.96906731347586e-15,-0.554602327422888,0.845,0.884,3.53132531
998761e-11,-13.3474826500444
"mt-
Atp8",2.22734391356522e-15,-0.559984906091314,0.787,0.868,3.9945185745
8786e-11,-13.408005855262
"Cirbp",2.23684063699385e-15,0.357751629662031,0.374,0.152,4.011549998
38476e-11,8.56430867796708

"Brd8",2.36267485921553e-15,0.369675645125903,0.381,0.154,4.2372210925
1713e-11,8.82952843417684
"Basp1",2.6178296876659e-15,0.188118829256375,0.116,0.021,4.6948157618
6002e-11,4.47383769931034
"Rnf216",2.624704309675e-15,0.25947703609827,0.245,0.075,4.70714470897
114e-11,6.17019643056549
"Rasl11a",2.724265201434e-15,0.21142329817504,0.245,0.073,4.8856972122
5174e-11,5.01963816651688
"Mycbp2",2.96678901819893e-15,0.350319574922472,0.477,0.221,5.32063942
523795e-11,8.28745502055012
"Car2",3.02157719130726e-15,-0.784744197018268,0.465,0.68,5.4188965348
9045e-11,-18.5502100883611
"F3",3.20663602539504e-15,0.770366580045192,0.258,0.084,5.750781047943
46e-11,18.1645508917215
"Srsf10",3.24384618555754e-15,0.361560366592668,0.458,0.208,5.81751374
91789e-11,8.52109673364073
"Elovl7",3.25515980946451e-15,0.470362265805158,0.497,0.241,5.83780360
229366e-11,11.0836547153152
"L1cam",3.26878480917211e-15,0.345747834324912,0.226,0.067,5.862238676
76926e-11,8.14578594586461
"Ptp4a2",3.40972280593406e-15,0.4052390836111,0.632,0.327,6.1149968801
6215e-11,9.53028785908752
"Prmt2",3.86964754311271e-15,0.202693790709144,0.194,0.052,6.939825903
81833e-11,4.74124275461888
"Atf7",3.95998895096909e-15,0.272263983856555,0.239,0.074,7.1018441846
6797e-11,6.3622869719785
"Slc22a6",3.98848956683845e-15,-1.00073545558824,0.045,0.341,7.1529571
8916807e-11,-23.3780910707666
"Mvp",4.11552416117355e-15,0.315713664922614,0.361,0.14,7.380781030648
64e-11,7.3654598023278
"Atp5b",4.47377988214754e-15,-0.448115767700073,0.923,0.934,8.02327684
06434e-11,-10.4169390634662
"Atp5d",4.71803684009113e-15,-0.490709600979909,0.806,0.873,8.46132726
901944e-11,-11.3809934131117
"Elf1",5.21060961040629e-15,0.349946033013847,0.387,0.161,9.3447072753
0264e-11,8.08152277396344
"Sept7",5.36519105072998e-15,0.455039174187555,0.458,0.211,9.621933630
37914e-11,10.4952013324762
"Aass",5.57380801186374e-15,-0.764690533739233,0.026,0.324,9.996067288
47643e-11,-17.6079510272934
"Slc5a3",5.62403943179504e-15,0.447218220733657,0.387,0.158,1.00861523
169812e-10,10.2937436972275
"Pdpn",5.62716708144166e-15,0.32360758195742,0.181,0.046,1.00917614438
575e-10,7.44838401342061
"Senp1",5.7499291087857e-15,0.236316237474492,0.174,0.043,1.0311922863
6963e-10,5.43412383956019
"BC021785",6.23658816632004e-15,-0.796855706909018,0.039,0.336,1.11846
972174784e-10,-18.2590636145755
"Me1",6.41669682103919e-15,-0.784065960880756,0.058,0.357,1.1507704078
8517e-10,-17.9436782673106

"Srsf2",6.45166742849886e-15,0.365611325562033,0.735,0.423,1.157042036
62699e-10,8.36518133390306
"Ifi27l2a",6.74751677611837e-15,0.413072547378416,0.432,0.191,1.210099
65862907e-10,9.43257284137234
"Phf14",7.13319575867258e-15,0.382723165968353,0.381,0.16,1.2792673273
6034e-10,8.71826663009855
"Ptprj",7.15945625382616e-15,0.343509614866751,0.394,0.163,1.283976884
56118e-10,7.82373676503595
"Rpl7",7.56419097016775e-15,0.414183842112043,0.929,0.793,1.3565620085
8988e-10,9.41062856714879
"Cx3cl1",7.73714652981705e-15,0.320194619029504,0.277,0.095,1.38757985
865739e-10,7.2678702580682
"Senp6",7.77063178352261e-15,0.420682820141119,0.484,0.235,1.393585104
05694e-10,9.54696384319562
"Mep1a",7.97167023034076e-15,-0.91713854789786,0.045,0.338,1.429639339
10931e-10,-20.7900898066251
"Gnb1",8.88552433030533e-15,0.417646469806699,0.671,0.375,1.5935299333
9696e-10,9.42206229789527
"Akr1b3",9.0936620284264e-15,1.33152352047871,0.381,0.161,1.6308573481
7799e-10,30.0082061396306
"Ubn2",9.1380212134087e-15,0.39453597801011,0.445,0.205,1.638812724412
72e-10,8.88963687774754
"Ncam1",9.54973024846987e-15,0.25755362895275,0.161,0.038,1.7126486227
6059e-10,5.79181703549336
"Rab2b",9.83710748942054e-15,0.152510787434505,0.135,0.028,1.764186857
15268e-10,3.42511182604733
"Gm5424",9.90868964111091e-15,-0.802340991292377,0.006,0.295,1.7770244
0023683e-10,-18.0132859061142
"Cox5b",9.92165317482398e-15,-0.396973242805296,0.923,0.941,1.77934928
037293e-10,-8.91189178409959
"2610524H06Rik",1.06121177462879e-14,0.215499751425365,0.187,0.049,1.9
0317719661926e-10,4.8233857392324
"Kmt2a",1.08075791287808e-14,0.353670891679712,0.342,0.136,1.938231240
95554e-10,7.90952238369849
"Atp5e",1.12643721406366e-14,-0.422360690179544,0.935,0.935,2.02015249
970177e-10,-9.42822165568169
"Nudt4",1.23644428853657e-14,0.526212851744664,0.645,0.35,2.2174391870
6148e-10,11.6974474929214
"Tspan15",1.25859990082125e-14,0.208468081333157,0.219,0.064,2.2571730
6213283e-10,4.6304383532968
"Ankrd11",1.27486400123807e-14,0.409714718460446,0.458,0.209,2.2863410
9982035e-10,9.09521531105903
"Hnrnpa3",1.2773993405454e-14,0.42182289933421,0.768,0.47,2.2908879773
3412e-10,9.36316553335017
"2410089E03Rik",1.29430219619702e-14,0.218128435443331,0.2,0.055,2.321
20155865974e-10,4.83891016221211
"Luzp1",1.29525752696138e-14,0.324743865117044,0.348,0.14,2.3229148488
5253e-10,7.20380229574988
"Ndufb4",1.29561261097529e-14,-0.52127527199629,0.832,0.86,2.323551656
52309e-10,-11.5633211398029

"Slc16a3",1.35463079487646e-14,0.0814283836004596,0.058,0.006,2.42939486753144e-10,1.80267855263707
"Laptm4b",1.36470800774374e-14,0.33107140775245,0.329,0.128,2.44746734108762e-10,7.32687417653398
"Siva1",1.39541246203325e-14,0.273806339501066,0.29,0.103,2.50253270941042e-10,6.05346049746811
"Irx3",1.43143731634625e-14,0.24622027446712,0.213,0.06,2.56713968313536e-10,5.43729675315981
"Glg1",1.56162580241656e-14,0.303663272809966,0.413,0.177,2.80061971405385e-10,6.67938044937315
"Uqcrb",1.57696297725991e-14,-0.426054811241605,0.942,0.945,2.82812540341793e-10,-9.36734198651923
"4931406P16Rik",1.60937793270221e-14,0.303532037686147,0.258,0.086,2.88625838450814e-10,6.66735133407184
"Ces1f",1.63099377622176e-14,-0.90689981145499,0.026,0.314,2.9250242382761e-10,-19.90876178971
"Serpinb6a",1.64303593691145e-14,0.355190480795518,0.335,0.132,2.94662064925699e-10,7.7947232830135
"Ept1",1.66998726718702e-14,0.248947798473242,0.213,0.062,2.99495516497321e-10,5.45915677225131
"Ell",1.73264635248046e-14,0.158003333770424,0.148,0.034,3.10732796853846e-10,3.45902284724504
"Matr3",1.77300147645909e-14,0.377391805124199,0.497,0.239,3.17970084788173e-10,8.25320546784606
"Tacc1",1.84522406094589e-14,0.297106497382992,0.342,0.132,3.30922483090035e-10,6.48557842249646
"Cnot6l",1.8592870091358e-14,0.289822116771643,0.335,0.129,3.33444532218415e-10,6.32436624273273
"Ccdc39",1.99601704644325e-14,0.138459893942338,0.084,0.012,3.57965697109132e-10,3.0115835472124
"Ndufa2",2.03663594378293e-14,-0.439117804405197,0.89,0.91,3.65250290158031e-10,-9.54222234227529
"Laptm4a",2.04169276943656e-14,0.421225796205263,0.8,0.513,3.66157181270752e-10,9.15237658440781
"Enpp2",2.05865614088787e-14,-0.725069896743001,0.103,0.406,3.6919939230683e-10,-15.7482892231624
"Skil",2.12734366284382e-14,0.392114625019738,0.284,0.103,3.8151781249441e-10,8.50373637880612
"Ppp1r14b",2.13354405042257e-14,0.415663062800098,0.465,0.217,3.82629790002783e-10,9.01321840070492
"Cdc42",2.32374695119126e-14,0.39837797010678,0.839,0.524,4.1674077822664e-10,8.60438918429509
"Atf3",2.33658596409524e-14,0.623310372105796,0.187,0.051,4.19043326800841e-10,13.4591700333748
"Sod3",2.38690352368273e-14,-0.72035921447923,0.039,0.333,4.2806727793726e-10,-15.5394022153221
"Laptm5",2.43094527883813e-14,0.357469447430917,0.297,0.107,4.3596572630683e-10,7.70470253740923
"Fgfr2",2.50359842443854e-14,0.196983032496505,0.148,0.034,4.48995341438808e-10,4.23986448622878

"Bnip2",2.52050545628379e-14,0.274035775479621,0.277,0.097,4.52027448529936e-10,5.89650402079062
"Parp8",2.67931180533468e-14,0.257404024495765,0.206,0.059,4.80507779168722e-10,5.52290648973883
"Rab11fip1",2.84628674106538e-14,0.250877708715525,0.194,0.054,5.10453064142664e-10,5.36770981792239
"Sbno1",2.94957803141265e-14,0.355490288568163,0.477,0.225,5.28977324153546e-10,7.59329942127904
"Cst3",2.95296148974676e-14,0.363994734574859,0.935,0.707,5.29584113571184e-10,7.77453773175685
"Gm1673",3.14888501877727e-14,0.301956767561624,0.135,0.029,5.64721039267515e-10,6.43007552990047
"Socs2",3.15691552193672e-14,0.418896847268816,0.323,0.123,5.66161229704131e-10,8.91921124762228
"Plpp2",3.39861276712228e-14,0.332223537650454,0.355,0.143,6.09507213655711e-10,7.04924205477849
"Pnrc1",3.54935164487719e-14,0.448229706846333,0.497,0.249,6.36540723992275e-10,9.49125181424611
"Zfp703",3.56690301604312e-14,0.17037226785111,0.123,0.025,6.39688386897174e-10,3.60678771739686
"Pura",3.60288927860321e-14,0.385451729926688,0.613,0.334,6.461421632247e-10,8.15615921019433
"Scx",3.6059075563135e-14,0.221073955234324,0.148,0.034,6.46683461149263e-10,4.67774011517318
"Pim1",3.6349633652949e-14,0.426470663471482,0.232,0.074,6.51894329931987e-10,9.02034012803697
"Leprot",3.65182141648281e-14,0.314116842652774,0.381,0.159,6.54917652832027e-10,6.64247545975941
"Capzb",3.91569585576786e-14,0.345075506622668,0.632,0.343,7.02240894773408e-10,7.27306832626035
"Kif5b",4.10670538870472e-14,0.381218622109858,0.677,0.386,7.36496544410305e-10,8.01669084374411
"Mprip",4.25642184060568e-14,0.334047789066699,0.439,0.2,7.63346692894223e-10,7.01276839203699
"Nox4",4.38279132347148e-14,-0.829346031528617,0.097,0.383,7.86009795951375e-10,-17.3864532152944
"Ppp1r15a",4.67548565297553e-14,0.374749984110278,0.206,0.062,8.38501597004632e-10,7.83205155303301
"Cldn19",4.73022304023149e-14,0.317923465176397,0.265,0.087,8.48318200035116e-10,6.64071072861839
"Serpine1",4.8842773800191e-14,0.17137510050661,0.077,0.011,8.75946305332626e-10,3.57415048084634
"Eif6",4.91993449049704e-14,0.307481984318744,0.574,0.282,8.82341051525739e-10,6.41052045599662
"Gapvd1",5.16972631946845e-14,0.325511716798444,0.316,0.123,9.27138718133471e-10,6.77029147954542
"Spaca6",5.24889414035432e-14,0.286241790789782,0.219,0.068,9.41336675131144e-10,5.94916930532879
"Stk39",5.58680889681571e-14,0.255856186185935,0.303,0.111,1.00193830755493e-09,5.30168031439003

"AI838599",5.59075320785897e-14,0.287577032334425,0.213,0.062,1.00264568029743e-09,5.95877545747189
"Cgn",5.70510016782431e-14,0.285866277107536,0.226,0.07,1.02315266409761e-09,5.91753974468753
"Map4k4",5.76332353513691e-14,0.331797315075919,0.232,0.076,1.03359444279145e-09,6.86496055899351
"Folr1",5.81474319523813e-14,-0.761770091608878,0.2,0.491,1.04281604463401e-09,-15.7544270646372
"Misp",5.874196029382e-14,0.26663848166216,0.129,0.027,1.05347831590937e-09,5.51172897390398
"Ddr1",6.0369326888542e-14,0.319195141840524,0.484,0.227,1.08266350841911e-09,6.58941395416326
"Gpr173",6.14844575002647e-14,0.0838894115881371,0.052,0.005,1.10266226080975e-09,1.73026427537504
"Wapl",6.24882373833735e-14,0.368756201602567,0.439,0.207,1.12066404923342e-09,7.59982356813728
"Hspa1b",6.41002829120299e-14,0.224642634433132,0.187,0.051,1.14957447374434e-09,4.62401567902715
"Mdm4",6.4498262823376e-14,0.280774223625,0.323,0.125,1.15671184547442e-09,5.7776833818873
"Aif1l",6.5289207301963e-14,0.47264242131623,0.568,0.301,1.1708966437534e-09,9.72012583420681
"Kcnq1ot1",6.64361124966536e-14,0.494563271722307,0.432,0.198,1.19146524151499e-09,10.1623266575099
"Cxadr",6.89185142846485e-14,0.366152046388498,0.381,0.159,1.23598463518089e-09,7.51029031869532
"Chmp4b",6.89821537994311e-14,0.360118634208154,0.626,0.334,1.237125946239e-09,7.38620421977587
"Rpl12",7.04827495720515e-14,0.431963865213587,0.858,0.587,1.26403763082517e-09,8.85048809682668
"Arpc3",7.14551089737561e-14,0.379037976594862,0.697,0.406,1.28147592433534e-09,7.76089860285521
"Morf4l2",7.4652943787651e-14,0.392089972228243,0.51,0.254,1.33882589388773e-09,8.0109756049538
"Hnrnph1",7.80819793342813e-14,0.357888245787679,0.529,0.272,1.400322217381e-09,7.29611143846673
"Sdcbp",7.95056402773355e-14,0.408128016839004,0.594,0.333,1.42585415273374e-09,8.31295338764632
"Fry",8.45277081017737e-14,0.233589240560304,0.148,0.035,1.51591991709721e-09,4.74355355813273
"Mif",8.57510754391363e-14,-0.550507351871399,0.761,0.852,1.53785978692547e-09,-11.1713764023958
"Tspo",8.85762776170551e-14,0.397449472043813,0.8,0.485,1.58852696278427e-09,8.05250860915914
"Tesc",8.89396285536372e-14,0.354148531283965,0.374,0.152,1.59504329848093e-09,7.17376189785724
"Vps36",8.97092856014208e-14,0.372324941698093,0.445,0.211,1.60884632797588e-09,7.53874177281065
"Cbx1",9.34463616823319e-14,0.411746376879045,0.523,0.267,1.67586705041094e-09,8.320132341427

"Hoxd4",9.51299817495021e-14,0.288440609669071,0.29,0.105,1.70606109269557e-09,5.82335013370326
"Fbl",9.58928167374375e-14,0.329250777046654,0.4,0.177,1.7197417753692e-09,6.64464011992083
"Cr1l",9.62570520708776e-14,0.295422099743739,0.361,0.149,1.72627397183912e-09,5.96082048815135
"Ezr",9.8352411973034e-14,0.471329463387133,0.794,0.495,1.76385215632439e-09,9.50000622893684
"Aldh2",9.92135831085489e-14,-0.701449597732012,0.303,0.55,1.77929639946872e-09,-14.1321385972328
"Trim12a",1.10576311562316e-13,0.267282780491759,0.226,0.071,1.98307557155858e-09,5.35597723647401
"Capns1",1.12678940504229e-13,0.375912054800594,0.748,0.437,2.02078411900285e-09,7.52567672033333
"C1qa",1.1297698461602e-13,0.404429042488267,0.523,0.258,2.0261292421037e-09,8.09551221409449
"Ddx39b",1.15781318148123e-13,0.358519664060733,0.477,0.227,2.07642215966843e-09,7.1677472411299
"Rnf122",1.23279077222031e-13,0.160853046034321,0.116,0.023,2.21088697089991e-09,3.20578062754493
"Rnf4",1.25366711471831e-13,0.28480469493417,0.303,0.114,2.24832660353581e-09,5.67133856899347
"Itga3",1.26817389006671e-13,0.353288830405204,0.213,0.066,2.27434305444564e-09,7.0310040113671
"Gm42418",1.34662071081945e-13,0.558649428357029,0.916,0.639,2.41502958278361e-09,11.0844729675871
"Pafah1b3",1.43193435158294e-13,0.369248495310674,0.29,0.111,2.56803106612885e-09,7.30378189319184
"Irx5",1.47404309380118e-13,0.114530336916521,0.052,0.005,2.64354888442303e-09,2.262105125052
"Elk3",1.51574479642834e-13,0.232542708926011,0.174,0.047,2.71833671791458e-09,4.5864969709731
"Aimp1",1.55361111583794e-13,0.360989229103035,0.445,0.21,2.78624617514377e-09,7.11097181676632
"Chd9",1.58715929230268e-13,0.302515018100307,0.271,0.096,2.84641147481562e-09,5.95265056148874
"Zc3h15",1.71793545108278e-13,0.373820387561089,0.548,0.285,3.08094543797186e-09,7.32614291833428
"BC005561",1.72914845637999e-13,0.333175554283519,0.452,0.215,3.10105484167187e-09,6.52741670531171
"Snrk",1.75702634629602e-13,0.265000804315835,0.284,0.103,3.15105104944729e-09,5.18753113482565
"Tcf4",1.76298008465053e-13,0.353275499441137,0.323,0.128,3.16172848381227e-09,6.91435999608329
"Erich4",1.7677990114456e-13,-0.687331846165135,0.019,0.291,3.17037074712654e-09,-13.4506837218492
"Cfap97",1.87183693319608e-13,0.254077916090363,0.258,0.089,3.35695235599386e-09,4.9576273239731
"Acsl5",1.89593754526677e-13,0.324338766773769,0.381,0.162,3.40017439368142e-09,6.32442403513835

"Rps9",1.92615195408275e-13,0.410355208482261,0.968,0.848,3.4543609144
5201e-09,7.99520838215045
"Ubtf",1.93474275256937e-13,0.333302012123823,0.406,0.182,3.4697676524
5791e-09,6.49244928997016
"Xbp1",1.93561360657547e-13,0.377705776313606,0.529,0.274,3.4713294420
3245e-09,7.35722815330671
"March7",1.98192184935558e-13,0.268127750931797,0.368,0.154,3.55437864
46343e-09,5.21644833873314
"Hnrnpc",1.98360950930482e-13,0.396154642091701,0.581,0.318,3.55740529
398726e-09,7.7068852690005
"Csf2ra",1.98964988395248e-13,0.272032720257704,0.226,0.073,3.56823810
188039e-09,5.29136118666416
"Tpr",2.05078007388998e-13,0.38335970067683,0.555,0.293,3.677868984514
29e-09,7.44520280596424
"Nfkb1",2.14770112295261e-13,0.267305044444888,0.258,0.089,3.851687193
90321e-09,5.17896962675405
"Knop1",2.20923762458432e-13,0.300806642685928,0.452,0.209,3.962046755
92952e-09,5.81955724312129
"Stx16",2.3890547599114e-13,0.326845909434519,0.374,0.162,4.2845308064
2511e-09,6.29775025928292
"Park7",2.42453680287376e-13,-0.522034113260106,0.8,0.841,4.3481643022
738e-09,-10.0509901060291
"Inmt",2.45159801805143e-13,-0.989557015150802,0.058,0.333,4.396695885
57344e-09,-19.0414642908746
"Nsd1",2.53231014530359e-13,0.369382103153309,0.381,0.169,4.5414450145
8745e-09,7.0958378072943
"Rell1",2.6364260421739e-13,0.19780219568177,0.194,0.056,4.72816646403
467e-09,3.79181435812118
"Cndp2",2.68091388269062e-13,-1.21260027274969,0.245,0.499,4.807950957
21736e-09,-23.2249267669713
"Slc25a36",2.83026402519357e-13,0.326536715967426,0.439,0.2,5.07579550
278215e-09,6.23645374101176
"Twsg1",2.8804061914003e-13,0.349868578972449,0.329,0.134,5.1657204636
5729e-09,6.6759197653803
"Lmna",2.89756448185898e-13,0.334603782170362,0.4,0.176,5.196492141765
9e-09,6.38266151223998
"Scarb1",2.97566619035896e-13,0.211094221443778,0.168,0.045,5.33655974
578977e-09,4.02106725233153
"Brd2",3.07756963932203e-13,0.383065261466428,0.432,0.204,5.5193133911
6014e-09,7.28399068532184
"Rbm4b",3.10437256514256e-13,0.316353439020502,0.342,0.141,5.567381758
32666e-09,6.01272132426693
"Pkn2",3.26146292961112e-13,0.42163484452354,0.49,0.255,5.849107617964
59e-09,7.99292193672806
"Ngfrap1",3.26761945495729e-13,0.428490891344731,0.465,0.228,5.8601487
305204e-09,8.12208377514148
"Chmp4c",3.30741368247125e-13,0.222615924996831,0.181,0.051,5.93151569
814394e-09,4.21701036356129
"Bcl10",3.37585581765936e-13,0.244992378826765,0.29,0.109,6.0542598233
903e-09,4.63586919718009

"Sdhb",3.58134982425904e-13,-0.590487478453313,0.555,0.709,6.42279277482617e-09,-11.1386090605829
"Ywhaq",3.64824153446357e-13,0.406634559884017,0.723,0.437,6.54275636790696e-09,7.66299058488665
"Map1b",3.68732703924756e-13,0.165337344692116,0.11,0.021,6.61285231218658e-09,3.11400501016452
"Gstm5",3.73969563720689e-13,-0.773667404164798,0.284,0.517,6.70677015576683e-09,-14.5605353197256
"Sostdc1",3.99164118296479e-13,0.555034558898485,0.394,0.167,7.15860929752906e-09,10.4096454598174
"Zbtb7a",4.1194199090681e-13,0.296928101006745,0.426,0.195,7.38776766492273e-09,5.55951554964915
"Klc3",4.24442002049148e-13,0.192179927404886,0.142,0.034,7.61194286474941e-09,3.5925245810198
"Jag1",4.26105309180261e-13,0.378500122481511,0.245,0.083,7.6417726148388e-09,7.07402960712206
"Arf5",4.28339937002720e-13,0.380533723295713,0.768,0.485,7.68184843020679e-09,7.11004644489256
"Actb",4.31997205933123e-13,0.437267379450098,0.981,0.863,7.74743789120462e-09,8.16636344480343
"Galnt11",4.35730526432055e-13,-0.757699798478907,0.206,0.463,7.81439126103247e-09,-14.1442085159993
"Ppp1r9b",4.39705392210729e-13,0.247177991318356,0.232,0.077,7.88567650390722e-09,4.61190080329228
"Efna4",4.44889970852531e-13,0.198386711861942,0.148,0.036,7.97865673726929e-09,3.69921698322394
"Cdkl1",4.48196229707439e-13,0.425471616654749,0.419,0.196,8.03795118357321e-09,7.93040444204559
"Snrnp70",4.66218605226002e-13,0.341095119364769,0.49,0.241,8.36116446612312e-09,6.34425602072358
"Capn1",4.67379785393658e-13,0.277082440487834,0.226,0.074,8.38198907124985e-09,5.15295218442484
"Sfxn1",4.69019681452062e-13,-0.685530568699979,0.194,0.456,8.41139896716128e-09,-12.7465346748955
"Hmgb1",4.71340289727416e-13,0.430170835727574,0.897,0.661,8.45301675597147e-09,7.99633487344314
"Irx1",4.7489845094506e-13,0.27298446924298,0.155,0.039,8.51682881924871e-09,5.07238496268873
"Tbc1d4",5.01641366365694e-13,0.23460058560927,0.148,0.037,8.99643626440236e-09,4.34631304186254
"Aktip",5.08688056928092e-13,0.413157266688215,0.548,0.289,9.1228116129484e-09,7.64856885469993
"Ctbp2",5.1576224314621e-13,0.302779438513459,0.265,0.096,9.24968006858413e-09,5.60101899690949
"Pcx",5.52690933052881e-13,-0.708916635806978,0.084,0.356,9.91195919337036e-09,-13.0649960185693
"Golga4",5.67127881387763e-13,0.349592706831653,0.574,0.299,1.01708714248082e-08,6.43381256392029
"Bdh2",5.76233085258404e-13,-0.724393868296582,0.148,0.425,1.03341641510242e-08,-13.3200171940066

"Slc2a2",5.76855130036608e-13,-0.657006638705416,0.026,0.292,1.0345319
9020765e-08,-12.0802047271836
"Rhobtb3",5.87136585486638e-13,0.140099194164247,0.123,0.026,1.0529707
5241174e-08,2.57349124421342
"Fermt2",5.90970326321309e-13,0.335313775625141,0.4,0.181,1.0598461832
2464e-08,6.15721830322353
"Hddc2",6.05842528811959e-13,0.193696131205739,0.239,0.079,1.086517991
17137e-08,3.55194206132311
"Rps3a1",6.11364780915556e-13,0.436072500337675,0.942,0.822,1.09642159
809396e-08,7.99261105817395
"Rplp2",6.11538067341595e-13,0.395926741143157,0.948,0.87,1.0967323699
7042e-08,7.25668212724966
"Nipal2",6.12108781930769e-13,0.229178677136965,0.187,0.055,1.09775588
951464e-08,4.20025220902807
"Cd52",6.21093358067158e-13,0.370502704648165,0.523,0.268,1.1138688283
5764e-08,6.78495725282529
"Hgd",6.29226886578426e-13,-0.668310336824637,0.026,0.29,1.12845549838
975e-08,-12.2299661270546
"Ash1l",6.35431539013193e-13,0.366615355770784,0.523,0.268,1.139582922
06626e-08,6.70540160508899
"Bzw2",6.36905658311145e-13,0.253092026585719,0.284,0.105,1.1422266076
1521e-08,4.62847136384913
"Prpf38b",6.68334404985097e-13,0.388380395797719,0.439,0.213,1.1985909
2190027e-08,7.08387747636444
"Rps8",6.92818221844165e-13,0.388848109319129,0.994,0.898,1.2425001990
5533e-08,7.07841798537182
"Akap8l",6.96690171827146e-13,0.263500941210252,0.239,0.081,1.24944415
41548e-08,4.79518537675113
"Psd3",6.98834017495411e-13,0.195434964814509,0.187,0.054,1.2532889269
7627e-08,3.55592149919631
"Chchd2",7.05622614706995e-13,-0.350900642105353,0.974,0.951,1.2654635
9721553e-08,-6.38121316794368
"Fnbp1l",7.06499444015402e-13,0.353940158236354,0.413,0.191,1.26703610
289722e-08,6.43604796062269
"Lypla2",7.27448737295767e-13,0.237082006975123,0.29,0.109,1.304606565
46623e-08,4.30417149628383
"Ephx2",7.35644390867348e-13,-0.707264313220968,0.077,0.342,1.31930465
05815e-08,-12.8323037666847
"Hmgn3",7.62326875945585e-13,0.300361243850917,0.284,0.108,1.367157019
32081e-08,5.43892558321619
"Dynll1",7.63192060774355e-13,0.472339096865091,0.845,0.607,1.36870864
179273e-08,8.55255572119036
"Cpsf6",7.63273199581307e-13,0.278249876263951,0.355,0.15,1.3688541561
2912e-08,5.03818890896841
"Lamp1",8.30359541278583e-13,-0.49983464656324,0.787,0.81,1.4891668013
2901e-08,-9.00825191071351
"BC026585",8.50864583009875e-13,-0.708135549310456,0.058,0.324,1.52594
054316991e-08,-12.7450730262122
"Ttc14",8.69142378477920e-13,0.293647476540053,0.477,0.233,1.558719941
5623e-08,5.2788465987752

"Npm1",8.78230761061939e-13,0.443782618969319,0.806,0.56,1.57501904688848e-08,7.97318198258964
"Ptprs",9.00375329195849e-13,0.185106324638268,0.148,0.037,1.61473311537984e-08,3.32108716976802
"Gsn",9.37825558241013e-13,0.34726803163294,0.335,0.138,1.68189635614943e-08,6.21636127450106
"Ncl",1.01439458263131e-12,0.419221839531911,0.729,0.46,1.81921524449099e-08,7.47148712822109
"Mllt4",1.03889328381969e-12,0.354320265382743,0.4,0.186,1.86315121520223e-08,6.30633788183587
"Serf1",1.0401627456504e-12,0.2869383952786,0.381,0.164,1.86542786804942e-08,5.10669722437618
"Slc13a1",1.04533102035408e-12,-0.892106868547091,0.084,0.362,1.87469665190301e-08,-15.8725740540618
"Smarca5",1.05462752342829e-12,0.329068837833089,0.445,0.218,1.8913690005163e-08,5.85195613609761
"Gadd45a",1.09069850660856e-12,0.402130666485259,0.426,0.198,1.95605870175179e-08,7.13771846032834
"Plxnb1",1.14829288446995e-12,0.226118434497728,0.232,0.078,2.0593484590084e-08,4.00190987528249
"Rnf19a",1.19580527308332e-12,0.2737793111185,0.271,0.101,2.14455717674762e-08,4.83432598951763
"Irf2",1.2069375805899e-12,0.284106851253603,0.277,0.104,2.16452185702993e-08,5.01405443965995
"Arid5b",1.21082475775187e-12,0.280313013719484,0.316,0.127,2.1714931205522e-08,4.94619761718786
"Rab11fip2",1.21521018867566e-12,0.379465514999861,0.329,0.138,2.17935795237094e-08,6.69439796502927
"Srsf5",1.25028227085831e-12,0.411326674048369,0.665,0.396,2.2422562245573e-08,7.24477821106612
"Top3b",1.26186790012066e-12,0.310204960884501,0.271,0.101,2.26303389207639e-08,5.4608401904118
"Enah",1.26847648082621e-12,0.312033445312031,0.303,0.12,2.27488572071372e-08,5.49139888446097
"Ccl20",1.29356323153636e-12,0.380646773079895,0.052,0.005,2.31987629943731e-08,6.69145314588767
"Ndufa3",1.42106940300045e-12,-0.428235442007355,0.858,0.898,2.548545867341e-08,-7.48776427714009
"Yeats2",1.42813673885161e-12,0.19190361633606,0.142,0.035,2.56122042745649e-08,3.35451299353965
"Mapre1",1.46454779882859e-12,0.282527074929355,0.477,0.232,2.62652002241919e-08,4.93151601447219
"Prkar1a",1.51214810404734e-12,0.337087797979441,0.626,0.349,2.7118864097985e-08,5.8730929266771
"Arih1",1.51930423289662e-12,0.253231061498904,0.342,0.142,2.7247202112768e-08,4.41085839592628
"Arrdc3",1.54066285507506e-12,0.450066250026399,0.342,0.147,2.76302476429161e-08,7.83311266981148
"Srsf3",1.580362446869e-12,0.291711853562425,0.652,0.363,2.83422201221486e-08,5.06963500199752

"Aldh8a1",1.60944008276103e-12,-0.685283029495835,0.045,0.303,2.886369
84442363e-08,-11.896980166135
"Hsd11b2",1.61552318430384e-12,0.676806462582477,0.271,0.099,2.8972792
7873051e-08,11.7472679292573
"Fut11",1.62598922374239e-12,0.15696540759193,0.129,0.03,2.91604907385
96e-08,2.72342062893741
"Epn3",1.65831695844632e-12,0.293957821894414,0.239,0.084,2.9740256332
7764e-08,5.09451371858147
"Foxp1",1.68017862496645e-12,0.277666186949543,0.374,0.16,3.0132323460
1484e-08,4.80853066611833
"Evl",1.69581765285405e-12,0.215761677525648,0.187,0.056,3.04127937862
846e-08,3.73448995196446
"Zfp868",1.78764777457925e-12,0.144393056140251,0.116,0.025,3.20596751
893043e-08,2.4915984680444
"Thrap3",1.80717662051267e-12,0.328929923283339,0.439,0.21,3.240990551
22743e-08,5.67233131179087
"Fsd1l",1.93305595084382e-12,0.128160425221407,0.09,0.016,3.4667425422
433e-08,2.20147126213978
"Kng2",1.93811189190402e-12,0.49308916965254,0.471,0.232,3.47580986694
068e-08,8.46873412146459
"Rif1",1.99766439234454e-12,0.276184461682117,0.277,0.105,3.5826113212
307e-08,4.73506902575463
"Snrnp40",2.10591659131259e-12,0.220146383556619,0.226,0.075,3.7767508
1485999e-08,3.76270162980005
"Samd14",2.10837771959809e-12,0.147798636539023,0.129,0.03,3.781164602
32722e-08,2.52597457334627
"Amotl1",2.13203253199785e-12,0.257933728955356,0.155,0.041,3.82358714
288495e-08,4.40537698782596
"Ythdc1",2.14321049486733e-12,0.306809492771567,0.4,0.183,3.8436337014
9507e-08,5.2385458319106
"Slco1a6",2.17362816025649e-12,-0.643578190042378,0.013,0.264,3.898184
74260398e-08,-10.9795531693836
"Slc5a12",2.21979463117254e-12,-0.766973736943689,0.032,0.289,3.980979
69154483e-08,-13.0685826983617
"Nfix",2.37163570999938e-12,0.229166725511257,0.277,0.104,4.2532914823
1288e-08,3.88964398913667
"Oxr1",2.37924287969689e-12,0.252619545791369,0.271,0.102,4.2669341804
484e-08,4.28689942058917
"Gm26917",2.4129871611792e-12,0.170350523319651,0.639,0.36,4.327451174
85878e-08,2.88841271209946
"Dnmt3a",2.50709007132976e-12,0.326324611793102,0.348,0.149,4.49621533
392278e-08,5.52057858664348
"Tmem43",2.5551699486252e-12,0.224413470278997,0.181,0.053,4.582441785
86443e-08,3.79223952561844
"Agk",2.59651076909603e-12,0.22310052806649,0.194,0.059,4.656582413296
82e-08,3.76647212134969
"Ywhab",2.62120679132476e-12,0.342566501661708,0.619,0.346,4.700872259
56183e-08,5.78010151593042
"Aig1",2.62901853476545e-12,0.307125702442789,0.439,0.213,4.7148818402
4835e-08,5.18119735958276

"Cideb",2.6946852298569e-12,-0.633007931778231,0.032,0.288,4.832648491
22536e-08,-10.6631997046909
"Rarg",2.87072614771488e-12,0.17161970030583,0.123,0.028,5.14836027331
186e-08,2.88012223503486
"Slc7a7",2.9048869909158e-12,-0.712778734332413,0.052,0.305,5.20962432
950839e-08,-11.9534226829434
"Nisch",2.97931666757005e-12,0.391311683361608,0.606,0.353,5.343106511
62014e-08,6.55246464337127
"Ctdspl2",2.98132181175292e-12,0.278691075115365,0.232,0.08,5.34670253
719768e-08,4.6664593009533
"Chkb",3.11768767601528e-12,0.291425800818497,0.303,0.122,5.5912610781
658e-08,4.86665813357434
"Ano6",3.12130463007454e-12,0.29514302933626,0.387,0.178,5.59774772357
568e-08,4.92839169281672
"Ift74",3.19383327744353e-12,0.21908357727028,0.206,0.067,5.7278205997
6723e-08,3.65329437385911
"Ppig",3.20500356778507e-12,0.319662540295016,0.535,0.273,5.7478533984
6575e-08,5.3293672908452
"Acin1",3.21547316100869e-12,0.388064197227819,0.465,0.24,5.7666295669
5299e-08,6.46848414773013
"Hivep2",3.25204678034044e-12,0.255757232124218,0.194,0.06,5.832220695
86254e-08,4.26022057119259
"Sim2",3.38188532564306e-12,0.168190522238863,0.123,0.027,6.0650731430
0827e-08,2.79501265994081
"Suco",3.38942385766063e-12,0.312172204434739,0.258,0.096,6.0785927463
2858e-08,5.18702448262368
"Kcnj1",3.4389512473588e-12,0.435966726669005,0.374,0.161,6.1674151670
1328e-08,7.23765842133967
"Kdm2a",3.49079562502345e-12,0.245894508755134,0.323,0.132,6.260392873
91706e-08,4.07851397517233
"Hoxb7",3.50657286149502e-12,0.415457563582911,0.348,0.156,6.288687769
80516e-08,6.88908753794486
"Nxnl2",3.55656110770216e-12,0.0884960320382958,0.065,0.009,6.37833669
055305e-08,1.46618220449417
"Pkp3",3.65053052722802e-12,0.209440683901508,0.181,0.053,6.5468614475
3073e-08,3.46450390896914
"Zfp36",3.68743814540813e-12,0.381540851920405,0.303,0.12,6.6130515699
7494e-08,6.30749430643146
"Stat3",3.71261129758696e-12,0.334817746625628,0.413,0.195,6.658197101
09245e-08,5.53280701413913
"Nsrp1",3.76903558579822e-12,0.280301806374918,0.335,0.143,6.759388419
57053e-08,4.62771227926484
"Plekhg2",3.78113036003672e-12,0.138743103627068,0.11,0.023,6.78107918
768986e-08,2.29016921165212
"Il17re",3.86140401877104e-12,0.343465379342778,0.252,0.093,6.92504196
726399e-08,5.66221109285638
"Numa1",3.92241345689733e-12,0.323167309387186,0.329,0.141,7.034456293
59967e-08,5.32252045279931
"Hn1",3.93799971116506e-12,0.362173075953404,0.439,0.215,7.06240868200
341e-08,5.96350368747402

"Chst14",4.10913595707354e-12,0.0862718252833816,0.058,0.007,7.3693244
2541568e-08,1.4168727878866
"Atg101",4.20739723408674e-12,0.224501110710912,0.271,0.102,7.54554619
961116e-08,3.68175608769112
"Nop14",4.23215392843373e-12,0.244725213586228,0.213,0.07,7.5899448552
5305e-08,4.01199001340161
"Trim47",4.24289444766915e-12,0.326600813331866,0.335,0.145,7.60920690
244986e-08,5.35341902993648
"Tspan14",4.39867841110885e-12,0.330135587371262,0.361,0.163,7.8885898
624826e-08,5.39945447257195
"Fermt1",4.41523632838385e-12,0.378805378396685,0.297,0.12,7.918284831
3236e-08,6.19403846438497
"Fosl2",4.53830978879228e-12,0.292930472820591,0.194,0.061,8.139004775
22008e-08,4.78180079805642
"Klf3",4.62036926487979e-12,0.324813839313596,0.342,0.149,8.2861702396
3541e-08,5.29644462445402
"Bola3",4.64747370187405e-12,-0.60466430013236,0.355,0.575,8.334779336
94093e-08,-9.85617545028185
"Foxj1",4.79058899835086e-12,0.0940524396170797,0.052,0.006,8.59144230
964244e-08,1.53022511500666
"Tcf12",4.85701477981891e-12,0.28785621546894,0.335,0.144,8.7105703061
2724e-08,4.67943193971565
"Neu1",4.94693912937547e-12,-0.690054305152676,0.232,0.469,8.871840634
62196e-08,-11.2049627313162
"Myl6",4.97245020753632e-12,0.298379286687111,0.955,0.854,8.9175922021
9564e-08,4.84348795020316
"Rfc1",4.99105401105484e-12,0.280178828129428,0.271,0.104,8.9509562634
2574e-08,4.54699989171616
"Tmem256",5.08509044097796e-12,-0.560686087499144,0.665,0.759,9.119601
19684987e-08,-9.08886426785968
"Cttnbp2nl",5.10285814761147e-12,0.205545848116743,0.194,0.06,9.151465
80192642e-08,3.33123360258654
"Ndufab1",5.20753121264221e-12,-0.519599525109689,0.723,0.765,9.339186
47675254e-08,-8.41047775874514
"Ivns1abp",5.29420043403922e-12,0.418333223957368,0.826,0.572,9.494619
05840593e-08,6.76442962423172
"Nrip1",5.37848655599854e-12,0.363543401953624,0.419,0.204,9.645777789
52777e-08,5.87273844901019
"2810474O19Rik",5.66140644907867e-12,0.493154976073703,0.413,0.204,1.0
1531663257777e-07,7.9412228644429
"Slc6a19",5.73310147386099e-12,-0.751386440267006,0.045,0.294,1.028174
41832223e-07,-12.0900413778608
"Slc13a3",5.74725598418569e-12,-0.733316136513437,0.032,0.278,1.030712
88820386e-07,-11.7974763135744
"Ccdc141",5.77819918672611e-12,0.387326918744665,0.394,0.185,1.0362622
4214746e-07,6.22917564621314
"Ier5",5.83759565544815e-12,0.407225800867602,0.258,0.097,1.0469144048
4807e-07,6.54503425704971
"Bag4",5.86358256075211e-12,0.322617252731834,0.323,0.134,1.0515748964
4528e-07,5.18375165792919

"Cgref1",5.93457896186098e-12,-0.667552193058325,0.052,0.304,1.0643073
9102015e-07,-10.7180654095989
"Pantr1",5.93929106336121e-12,0.285102040119246,0.245,0.087,1.06515245
93032e-07,4.57730689855344
"Arfgef1",6.00930756917999e-12,0.335071268613116,0.529,0.282,1.0777092
1945674e-07,5.37563479587767
"Strbp",6.07718550566975e-12,0.283510233801175,0.497,0.25,1.0898824485
8681e-07,4.54524338452759
"Hcfc1r1",6.33545917122339e-12,0.357579234583876,0.632,0.373,1.1362012
477672e-07,5.71783684910722
"Eif5b",6.67924093876325e-12,0.316823596175205,0.671,0.39,1.1978550699
578e-07,5.04939606677809
"Tnfsf10",6.81545795063353e-12,0.287035563047338,0.174,0.051,1.2222842
2886662e-07,4.56885247264227
"Rbms3",6.8783023856034e-12,0.20381149573368,0.116,0.026,1.23355474983
411e-07,3.24227314153435
"Tle6",6.88671627426154e-12,0.0654962459910158,0.058,0.007,1.235063696
62606e-07,1.04184702352259
"Hnrnpd",6.96010056153362e-12,0.393105598543408,0.413,0.206,1.24822443
470544e-07,6.24895344363319
"Fxyd6",7.0380971036693e-12,0.132399524888801,0.077,0.013,1.2622123345
7205e-07,2.10319685821855
"Hao2",7.04744314707519e-12,-0.714589460332045,0.077,0.333,1.263888453
99646e-07,-11.3504693922947
"Prkcdbp",7.09087365107173e-12,0.278222297537535,0.155,0.042,1.2716772
805832e-07,4.41754656882426
"Cish",7.1758752848154e-12,0.290450629536033,0.232,0.082,1.28692147357
879e-07,4.60824401174826
"Mbnl2",7.22309705833452e-12,0.346052078023417,0.426,0.208,1.295390226
44171e-07,5.4881380692877
"Tkfc",7.32828758568386e-12,-0.62366024801581,0.045,0.294,1.3142550956
1654e-07,-9.88178787159797
"Ifngr1",7.3712373166493e-12,0.32560993096328,0.316,0.134,1.3219577003
6789e-07,5.15732980518119
"Gar1",7.59870796923413e-12,0.221950251065608,0.226,0.078,1.3627522872
0245e-07,3.50872035427899
"R3hdm4",7.61552666189808e-12,0.325797948872187,0.465,0.239,1.36576855
15448e-07,5.14968564205636
"Fah",7.67693368925251e-12,-0.657146140201834,0.071,0.325,1.3767812878
3054e-07,-10.3818229341191
"Cd44",8.1195896549105e-12,0.205477986158004,0.129,0.031,1.45616720871
165e-07,3.2346936083546
"Efnb1",9.00091216471411e-12,0.263271309038415,0.2,0.065,1.61422358761
983e-07,4.11736359802218
"Mapk3",9.21461529354863e-12,0.268253795982488,0.329,0.141,1.652549106
74501e-07,4.18899136143591
"Gm26669",9.27380589812335e-12,0.0860888860422002,0.052,0.006,1.663164
34976944e-07,1.34379358748106
"Slit2",9.40883166847171e-12,0.332787176127141,0.29,0.116,1.6873798714
2372e-07,5.18978895511768

"Slc44a1",9.55661646011142e-12,0.283256606132473,0.297,0.122,1.7138835
9595638e-07,4.41294920291424
"Eva1b",9.86283041537275e-12,0.309939386771074,0.335,0.144,1.768800006
69295e-07,4.81887382993514
"Grb2",9.87581147233687e-12,0.294486816511822,0.342,0.151,1.7711280294
4889e-07,4.5782331094411
"Anapc2",1.02531298176951e-11,0.254908818191001,0.271,0.105,1.83879630
150545e-07,3.95337690446987
"Ifrd1",1.02734276582053e-11,0.324255778147934,0.232,0.083,1.842436516
22254e-07,5.02823654426334
"Aadac",1.03956819814254e-11,-0.633340785357053,0.013,0.252,1.86436160
654883e-07,-9.81372754419349
"Rpl22",1.05101871421612e-11,0.331528205596207,0.929,0.681,1.884896962
0752e-07,5.13345649834948
"Epha7",1.05408082337328e-11,0.286315939792786,0.071,0.011,1.890388548
63764e-07,4.43254675575759
"Tcf25",1.08509005525097e-11,0.319357526509078,0.639,0.362,1.946000505
08708e-07,4.93481450485312
"Atp5j",1.11274253106762e-11,-0.360338035094613,0.929,0.936,1.99559245
521667e-07,-5.55899060257125
"Lipa",1.12513891872018e-11,-0.615531825710974,0.052,0.3,2.01782413683
276e-07,-9.48908533176623
"Cyp2d26",1.13546002997144e-11,-0.674489042702302,0.006,0.242,2.036334
01775079e-07,-10.3918152534203
"Hk1",1.18564240943532e-11,0.329965675224344,0.342,0.148,2.12633109708
131e-07,5.06949286772525
"Lynx1",1.19003983007995e-11,0.201404581975675,0.155,0.043,2.134217431
26539e-07,3.09357349582691
"Efhd1",1.19795543971516e-11,0.325700426459288,0.387,0.173,2.148413285
58517e-07,5.00059788244206
"Mxd4",1.20995821015893e-11,0.362134334752065,0.439,0.218,2.1699390540
9903e-07,5.55637070952178
"Comt",1.21578766733633e-11,-0.761982893977105,0.432,0.587,2.180393602
60097e-07,-11.6877433796921
"Aplp2",1.21864226190641e-11,0.330711145222881,0.697,0.42,2.1855130325
0295e-07,5.07186716472679
"Mex3a",1.22073994025748e-11,0.189658811349286,0.116,0.026,2.189275008
85777e-07,2.90832782357798
"Tgfb1",1.22692846625974e-11,0.212208445959756,0.2,0.065,2.20037351139
021e-07,3.25304269352489
"Wnt10a",1.24069757451397e-11,0.0894099392629454,0.071,0.011,2.2250670
3013336e-07,1.36960904242143
"Ddx42",1.24848557255559e-11,0.244822213434354,0.303,0.124,2.239034025
8212e-07,3.74873024657964
"Tardbp",1.27381931884484e-11,0.321447802762212,0.51,0.272,2.284467566
41634e-07,4.91556779899524
"Gdi1",1.30865363802599e-11,0.270768490856152,0.348,0.155,2.3469394344
3581e-07,4.13327655605521
"Cd151",1.32729180667858e-11,0.299203922142617,0.419,0.203,2.380365126
09737e-07,4.56311167078237

"Parp14",1.40020136819752e-11,0.189088831866518,0.148,0.04,2.511121133
72544e-07,2.87365224692295
"Plk2",1.42745060780612e-11,0.148300595113344,0.11,0.024,2.55998992003
949e-07,2.25092012521822
"Prom1",1.44480797880456e-11,0.415763845647389,0.477,0.242,2.591118629
1881e-07,6.30547696325633
"Fam3c",1.52213612197317e-11,0.2661816319187,0.265,0.103,2.72979892114
669e-07,4.02303396891793
"Ppwd1",1.52663422262544e-11,0.162708332043689,0.155,0.043,2.737865814
85646e-07,2.4586720899405
"Prodh",1.53610417019276e-11,-0.594957302690127,0.045,0.292,2.75484921
88237e-07,-8.98667116775426
"Ccdc34",1.58807412658384e-11,0.274199455531933,0.29,0.118,2.848052138
61546e-07,4.1325862221722
"Cox7b",1.61209902908312e-11,-0.432935200972059,0.819,0.863,2.89113839
875767e-07,-6.51846517914984
"Dhcr24",1.63920961657089e-11,-0.564573097682787,0.077,0.332,2.9397585
2635823e-07,-8.49104852498519
"Chmp2a",1.69444135068193e-11,0.31318905424989,0.71,0.427,3.0388111183
1297e-07,4.69991203529425
"Isy1",1.71092438136404e-11,0.189158986973615,0.2,0.065,3.068371785538
27e-07,2.83680760356018
"N4bp2l2",1.72463659541314e-11,0.320623483810252,0.426,0.211,3.0929632
7021393e-07,4.80581450804047
"Slc22a28",1.74443165250343e-11,-0.7755600966769,0.013,0.248,3.1284637
2559965e-07,-11.6159929097717
"Ptgfrn",1.75267240888097e-11,0.151502891657696,0.116,0.027,3.14324269
808714e-07,2.26842865897356
"1700047I17Rik2",1.75748864905306e-11,0.0927751586477712,0.071,0.011,3
.15188014321175e-07,1.38885307825318
"Wfs1",1.76853057695228e-11,0.197911338793964,0.168,0.049,3.1716827367
0621e-07,2.96151229617042
"Rb1cc1",1.8512996206839e-11,0.289301057748051,0.406,0.194,3.320120739
7345e-07,4.31582051881185
"Kmt2e",1.88225477329179e-11,0.357015729776621,0.477,0.253,3.375635710
4215e-07,5.32007417635963
"Arrdc1",1.90495503885086e-11,0.255154842762393,0.213,0.074,3.41634636
667513e-07,3.79913415114227
"Cebpb",1.91165314393311e-11,0.562314688430976,0.426,0.211,3.428358748
32964e-07,8.37062432625993
"Cobl",1.962119667484e-11,0.25484866630201,0.252,0.094,3.5188654116658
1e-07,3.7870402328314
"Tmx3",1.97370848823788e-11,0.279397471797576,0.265,0.104,3.5396488028
0581e-07,4.15018908338144
"Cox5a",2.02181157959533e-11,-0.405329812626965,0.813,0.89,3.625916886
84626e-07,-6.01103644388632
"Cbx6",2.1060202357629e-11,0.294943243334411,0.342,0.149,3.77693669081
718e-07,4.36196941421604
"Taf15",2.14616234534888e-11,0.20605324668419,0.316,0.132,3.8489275501
4868e-07,3.04346849613048

"Vps37d",2.16656813083706e-11,0.136773789447365,0.129,0.032,3.88552328
584319e-07,2.01889574625754
"Npdc1",2.18801713626577e-11,0.245854220257406,0.29,0.117,3.9239899321
7904e-07,3.6265923270002
"Zrsr2",2.21328434179892e-11,0.22690269241863,0.323,0.137,3.9693041385
8219e-07,3.3444333357402
"Ppic",2.29701494238517e-11,0.306023711966522,0.239,0.088,4.1194665976
7356e-07,4.49927444269589
"Yap1",2.47560092211724e-11,0.263515318064346,0.297,0.122,4.4397426937
2505e-07,3.85457011156747
"Tgfb1i1",2.47982382359752e-11,0.118125329951921,0.077,0.013,4.4473160
4523979e-07,1.72767684513983
"Sema4b",2.52780484723582e-11,0.289971975380568,0.245,0.092,4.53336521
303273e-07,4.23551367796236
"Hnrnpu",2.55669904960148e-11,0.317301798278991,0.606,0.363,4.58518407
55553e-07,4.63110395737156
"Tagln2",2.58614291925367e-11,0.468801883098767,0.594,0.345,4.63798871
138953e-07,6.83691986260008
"Macf1",2.71547661826343e-11,0.370175004606277,0.413,0.208,4.869935767
19363e-07,5.38049920883275
"Chd7",2.71809851020985e-11,0.230234370211821,0.161,0.047,4.8746378682
1034e-07,3.3462378098616
"Apobec3",2.75568606842718e-11,0.279048527776778,0.206,0.069,4.9420473
951173e-07,4.051872789585
"Emb",2.78476232891247e-11,0.291050561603897,0.355,0.154,4.99419276067
163e-07,4.22309121942985
"Ogfrl1",2.78910028744555e-11,0.24768141882504,0.219,0.077,5.001972455
50484e-07,3.5934272450764
"Hnrnpa1",2.82900370301936e-11,0.281666995787776,0.503,0.256,5.0735352
4099492e-07,4.08249771452708
"Tnrc6a",2.84694931209057e-11,0.303704616849611,0.439,0.219,5.10571889
630322e-07,4.39999182110097
"Cblc",2.87043115911222e-11,0.21609194966449,0.194,0.063,5.14783124075
185e-07,3.1289077379673
"4732440D04Rik",2.89096099357767e-11,0.218077412253707,0.226,0.079,5.1
8464944588219e-07,3.15610210633494
"Mbnl1",2.91302691906419e-11,0.318884710195854,0.419,0.207,5.224222476
64972e-07,4.61260026275188
"Mnat1",2.9477214787543e-11,0.249232537300146,0.271,0.108,5.2864436999
9796e-07,3.60214537494751
"Plscr3",3.03520217492907e-11,0.195588574858534,0.194,0.063,5.44333158
051779e-07,2.8211117789079
"Arid3a",3.03638998245543e-11,0.210048300497858,0.168,0.05,5.445461794
53557e-07,3.02959240156094
"Gdf15",3.0693884985491e-11,0.37171162222356,0.168,0.049,5.50464133329
795e-07,5.3572952554523
"Plau",3.08475473643528e-11,0.379943719519934,0.168,0.051,5.5321991443
2304e-07,5.47404302929994
"Vsig10",3.14116901660276e-11,0.220875300131846,0.161,0.047,5.63337251
437539e-07,3.17826025240714

"Cytip",3.14624438751008e-11,0.211702967165526,0.226,0.08,5.6424746845
6058e-07,3.04593421548994
"Thnsl2",3.19500091308428e-11,-0.620228856264017,0.045,0.282,5.7299146
3752535e-07,-8.91417412366951
"Stk38",3.29291511348712e-11,0.280793960703324,0.329,0.145,5.905513964
52779e-07,4.02720571702028
"5930430L01Rik",3.40901568970259e-11,0.324788856876911,0.252,0.097,6.1
1372873791262e-07,4.64693566600066
"Irf1",3.44555866962243e-11,0.201243664639376,0.168,0.05,6.17926491810
087e-07,2.87715981079163
"Ddx17",3.46006943800504e-11,0.307003938494201,0.516,0.279,6.205288530
11824e-07,4.38791326795776
"Slc38a6",3.51611454411997e-11,0.185492468089103,0.206,0.069,6.3057998
2342476e-07,2.64820656196422
"Hmgn1",3.52701303250629e-11,0.344371878619719,0.755,0.486,6.325345172
49678e-07,4.91540270054038
"Il1rn",3.54683625575908e-11,0.228708302352498,0.09,0.017,6.3608961410
7833e-07,3.26319322065391
"Stox2",3.6347577200711e-11,0.194698138492612,0.174,0.053,6.5185744951
7552e-07,2.77317124100444
"Cnot4",3.768430364681e-11,0.293859303903648,0.355,0.165,6.75830301601
891e-07,4.17495428971008
"Fam177a",3.76850997882652e-11,0.266478780550529,0.335,0.149,6.7584457
9602747e-07,3.78594469818638
"Rprd2",3.79983653515996e-11,0.257474524707624,0.232,0.086,6.814626842
15587e-07,3.65588704394316
"Brd7",3.82862486840346e-11,0.289848712039245,0.381,0.181,6.8662558389
9477e-07,4.1133812419498
"Kdm5a",3.84512391488714e-11,0.251105998860596,0.329,0.144,6.895845228
95859e-07,3.56248514147336
"Aff1",3.91903256697931e-11,0.301752202575609,0.271,0.11,7.02839300562
07e-07,4.27526671579312
"Mical3",4.10264992311352e-11,0.246204968695252,0.258,0.1,7.3576923721
1178e-07,3.47699256824013
"Nme2",4.28846059556461e-11,-0.623828401737364,0.123,0.364,7.690925232
08558e-07,-8.78229027474568
"Rapgef6",4.31526347237676e-11,0.260140351978029,0.239,0.089,7.7389935
1136048e-07,3.66064925070125
"Phip",4.32560903679919e-11,0.358764295674055,0.348,0.162,7.7575472465
9567e-07,5.04760894472385
"Bpnt1",4.34178791781502e-11,-0.608091083739837,0.213,0.45,7.786562451
80947e-07,-8.55322442484226
"Phldb2",4.42960772774613e-11,0.397378531893117,0.542,0.303,7.94405849
893992e-07,5.58144826462021
"Arhgap5",4.43019239524325e-11,0.299297737967405,0.574,0.32,7.94510704
162925e-07,4.20379816443666
"Zfp451",4.59297087714163e-11,0.219755011470864,0.219,0.078,8.23703397
10658e-07,3.07864801604238
"Emsy",4.6789253057026e-11,0.275164368356363,0.245,0.093,8.39118464324
703e-07,3.84980099796303

"Tmem30b",4.75392460625437e-11,0.351799260192556,0.232,0.086,8.5256883
8885658e-07,4.91639884113709
"Jmjd1c",4.80592347626053e-11,0.228774863592272,0.323,0.138,8.61894316
232563e-07,3.1946426627016
"Atp5g3",4.81451174513834e-11,-0.397332195656627,0.832,0.853,8.6343453
637311e-07,-5.54769028969484
"Brd9",4.84006471026527e-11,0.262462819529681,0.194,0.064,8.6801720513
8974e-07,3.66320782414146
"Rpl21",4.84502520999089e-11,0.32926213885796,0.955,0.872,8.6890682115
9766e-07,4.5951922689297
"Crb3",4.89827362333065e-11,0.26945544368518,0.4,0.189,8.7845639160811
9e-07,3.75758299191014
"Pbrm1",5.08751909312443e-11,0.310276516371646,0.477,0.254,9.123956741
60935e-07,4.31507511348677
"Cubn",5.23444671878042e-11,-0.557242787883079,0.006,0.231,9.387456745
46081e-07,-7.7338173168949
"Nus1",5.29442054271765e-11,-0.670090514364975,0.297,0.501,9.495013801
30984e-07,-9.29236552408269
"Dstyk",5.42557573836465e-11,0.215843020432889,0.232,0.086,9.730227529
18316e-07,2.98788436216513
"Slc25a5",5.50504357355821e-11,-0.380083823452737,0.819,0.903,9.872745
14481929e-07,-5.25591986561937
"Cldn10",5.51682269048569e-11,-0.448865773487731,0.219,0.513,9.8938698
1311704e-07,-6.2060991131263
"Gpd1",5.65513828274478e-11,-0.585470846705195,0.052,0.287,1.014192499
62745e-06,-8.08032778206001
"Vangl2",5.77311199326807e-11,0.0944983573097527,0.084,0.016,1.0353499
048727e-06,1.30226023292744
"Actg1",5.81962774181219e-11,0.480902683881253,0.942,0.751,1.043692039
2166e-06,6.62335056083342
"Ppm1g",5.85966586544422e-11,0.294487244062721,0.323,0.143,1.050872476
30877e-06,4.05387895192588
"Kdm5b",5.89161200860584e-11,0.263844915645241,0.245,0.093,1.056601697
62337e-06,3.63062549388534
"Prpf40a",5.90174440837776e-11,0.262789090712535,0.439,0.222,1.0584188
4219847e-06,3.61564530805311
"Rgs19",5.90293379639825e-11,0.118964801688635,0.116,0.027,1.058632147
04606e-06,1.63678113922105
"Tbc1d9",6.10345460552528e-11,0.127642875341302,0.11,0.025,1.094593548
9549e-06,1.75191473237004
"Hspg2",6.23883174398226e-11,0.218851174381318,0.181,0.058,1.118872084
96578e-06,2.99895910326419
"Ctcf",6.38439501962149e-11,0.266781335732491,0.348,0.159,1.1449774028
1892e-06,3.64960219567798
"Notch1",6.51735634587512e-11,0.212568051623401,0.155,0.045,1.16882268
706924e-06,2.90357618487621
"Oxct1",6.5973103326697e-11,-0.570854105556269,0.503,0.673,1.183161635
06098e-06,-7.79062885209821
"Svbp",6.63117988566037e-11,0.246954999148507,0.387,0.183,1.1892358006
9433e-06,3.36900940083064

"Mpnd",6.82928126311968e-11,0.257156464067947,0.335,0.149,1.2247633017
2788e-06,3.50060998788728
"Nbeal1",6.95701834969144e-11,0.255234615761316,0.471,0.241,1.24767167
083366e-06,3.46971842970994
"Gnas",6.97030449967126e-11,0.392366670048884,0.89,0.673,1.25005440897
104e-06,5.33317470223985
"Atp5o",7.06894636195915e-11,-0.438731565514699,0.787,0.83,1.267744840
55375e-06,-5.95721607177385
"Ergic2",7.10789501221183e-11,0.323127996003556,0.445,0.234,1.27472989
149007e-06,4.38574399038706
"Zmym5",7.14866503662633e-11,0.269541121219804,0.323,0.143,1.282041587
66857e-06,3.65687969082745
"Adgre5",7.17330838420166e-11,0.170494788398093,0.155,0.045,1.28646112
562273e-06,2.31252574143054
"Dusp10",7.25246131017998e-11,0.10005687255669,0.058,0.008,1.300656411
36768e-06,1.35603492228246
"Lhfp",7.35755020184275e-11,0.16057375687731,0.077,0.014,1.31950305319
848e-06,2.17388852585966
"Scaf11",7.47558426676553e-11,0.29422776525409,0.49,0.258,1.3406712824
0173e-06,3.97864791228376
"Snrpb2",7.52811631091662e-11,0.295292466821697,0.419,0.213,1.35009237
919979e-06,3.99097736176551
"Smim6",7.65167459372505e-11,0.21119141355637,0.245,0.091,1.3722513216
3865e-06,2.85088511248401
"C1qc",7.72989343906357e-11,0.370528032691021,0.374,0.177,1.3862790893
6166e-06,4.99801087975534
"AU019823",7.9494496813137e-11,0.21079719793438,0.187,0.061,1.42565430
58468e-06,2.8375157198839
"Mknk2",8.02165096245349e-11,0.218831304944933,0.329,0.142,1.438602883
60641e-06,2.94368329300362
"Smtnl2",8.20277949191354e-11,0.320358269567975,0.316,0.138,1.47108647
407977e-06,4.3022543707973
"Ndufa1",8.24814165584218e-11,-0.386568885357345,0.832,0.852,1.4792217
2455874e-06,-5.18929857942189
"Adamts5",8.34640903131254e-11,0.219178465726294,0.077,0.014,1.4968449
9567559e-06,2.93965467853034
"Grhpr",8.41431207050667e-11,-0.630385284537959,0.142,0.385,1.50902272
672467e-06,-8.44971481259214
"Spns2",8.49826211626917e-11,0.347883229639513,0.297,0.126,1.524078327
93171e-06,4.65958996929427
"Kmt2d",8.54431422627086e-11,0.298853728071666,0.252,0.098,1.532337313
33942e-06,4.00126779005015
"Ndufs6",8.71092978584442e-11,-0.432987575579288,0.703,0.792,1.5622181
4779334e-06,-5.78878576299127
"Pcbd1",8.8155360438428e-11,-0.562333803693437,0.484,0.683,1.580978234
10277e-06,-7.51135509479727
"Clcf1",8.91796591066628e-11,0.188750374233884,0.129,0.033,1.599348006
41889e-06,2.51904635783233
"Gpatch8",9.05073279393948e-11,0.293673482441532,0.323,0.144,1.6231584
1926511e-06,3.91500132939755

"Cers4",9.12944607375059e-11,0.268411457685483,0.213,0.075,1.637274858
86643e-06,3.57590557077185
"Dap",9.1565027734448e-11,0.356978277521605,0.477,0.258,1.642127207389
59e-06,4.75477862123436
"Creb5",9.3232854036424e-11,0.197927090473261,0.116,0.028,1.6720380042
8923e-06,2.63272072443019
"Hnrnpm",9.38130378243422e-11,0.2621810765183,0.535,0.291,1.6824430203
4175e-06,3.48576653427208
"Arf3",9.52820824360156e-11,0.308887234979923,0.458,0.239,1.7087888664
075e-06,4.10193775389127
"Icam1",9.60955376369196e-11,0.300389519280522,0.194,0.065,1.723377371
98052e-06,3.98653677908878
"Atp2c1",9.63904857532919e-11,0.228633687170019,0.361,0.165,1.72866697
149954e-06,3.03354833891524
"Cep162",9.73884536889671e-11,0.23124300677019,0.206,0.072,1.746564528
45794e-06,3.06578736952112
"Zfp292",1.00760624633366e-10,0.366482309026487,0.361,0.174,1.80704104
217479e-06,4.84629602681627
"Cggbp1",1.0240366992505e-10,0.288514380698235,0.394,0.191,1.836507416
43585e-06,3.81059549842521
"Cxcl17",1.04565141611803e-10,0.203078546065054,0.161,0.048,1.87527124
966607e-06,2.67794748812308
"Cetn3",1.05287134596715e-10,0.284417072993708,0.477,0.257,1.888219471
85748e-06,3.7485818258168
"Ddx6",1.11659592214021e-10,0.344163923682858,0.439,0.228,2.0025031267
6625e-06,4.5158135943214
"Tsen34",1.14333120096153e-10,0.266286422170722,0.445,0.228,2.05045017
580441e-06,3.48767341719871
"Gsto1",1.14571056988441e-10,0.470748309205478,0.387,0.191,2.054717336
0307e-06,6.16462435397916
"Ptp4a1",1.14991274869674e-10,0.349100289659717,0.465,0.241,2.06225352
351274e-06,4.57032018240525
"Mapk13",1.15037469031597e-10,0.176968535219962,0.206,0.071,2.06308196
961266e-06,2.3167498825107
"Acat1",1.15200227768264e-10,-0.603668676641984,0.348,0.559,2.06600088
479605e-06,-7.90196005021804
"Sestd1",1.16964921457435e-10,0.302835482614184,0.368,0.174,2.09764890
141763e-06,3.95948108920594
"Dusp1",1.18094736027946e-10,0.633349301531133,0.284,0.123,2.117910995
92518e-06,8.27475950421901
"Gnb2",1.20540106994193e-10,0.358057327674105,0.697,0.437,2.1617662788
3386e-06,4.67070922736301
"Birc3",1.22601558365502e-10,0.240272360598845,0.174,0.056,2.198736347
72691e-06,3.13017887250045
"Dnajc12",1.2315649022557e-10,-0.685626276450389,0.155,0.388,2.2086884
9570537e-06,-8.92898755486473
"Rnh1",1.23498104043623e-10,0.273755612171473,0.523,0.278,2.2148149979
1834e-06,3.56439160738319
"Ptk7",1.23779163518953e-10,0.147499289728956,0.135,0.036,2.2198555185
4891e-06,1.92015584941802

"Actr3",1.24091275106902e-10,0.331799741105284,0.574,0.335,2.22545292776717e-06,4.31855615352975
"Cpn1",1.24286752211235e-10,-0.637752555068619,0.039,0.265,2.22895861415629e-06,-8.29969649071199
"Nat8",1.26264617876887e-10,-0.62365552203555,0.006,0.224,2.26442965700409e-06,-8.10639149676833
"Slc22a30",1.27980155110124e-10,-0.849129211888563,0.006,0.223,2.29519610174496e-06,-11.0256815144509
"Tyrobp",1.28319500674786e-10,0.424084952168022,0.49,0.264,2.30128192510162e-06,5.50548960698259
"Zfp512",1.29469932914892e-10,0.234223545806142,0.213,0.075,2.32191377689567e-06,3.03860988749073
"Amn1",1.29615868907428e-10,0.328346277882153,0.31,0.138,2.32453099298582e-06,4.25930537618047
"Slc3a1",1.30251363900299e-10,-0.560475473424112,0.026,0.25,2.33592796018796e-06,-7.26774225823464
"Txndc5",1.31045260046607e-10,0.235363944960864,0.316,0.136,2.35016569367585e-06,3.05055790985751
"Kpna4",1.32723999628965e-10,0.26737066553877,0.303,0.132,2.38027220934586e-06,3.46199443974303
"Mrps21",1.3566068814773e-10,-0.492592748326294,0.658,0.756,2.4329387812414e-06,-6.36745615119107
"Mcl1",1.40502369771217e-10,0.332851025540479,0.413,0.217,2.51976949947701e-06,4.29089678111046
"AI413582",1.42030455317215e-10,0.234239104521863,0.284,0.118,2.54717418565894e-06,3.0171228703295
"Emx2",1.43809168156105e-10,0.33273833590355,0.297,0.128,2.57907362171158e-06,4.28170362025786
"Safb2",1.44989444231541e-10,0.310486631377624,0.355,0.168,2.60024069284845e-06,3.99282906238238
"Dcaf8",1.4688603054581e-10,0.27116782904747,0.542,0.301,2.63425407180856e-06,3.48366883045543
"Dlgap4",1.47460640035532e-10,0.230144877347168,0.258,0.103,2.64455911839722e-06,2.95575208470328
"Tes",1.48956753386905e-10,0.2374172998558,0.265,0.107,2.67139041524076e-06,3.04675518962366
"Zfp950",1.50120032012238e-10,0.378908069272547,0.342,0.163,2.69225265410747e-06,4.85954611678543
"Snrpf",1.50137206582593e-10,0.283277033275259,0.626,0.372,2.69256066285223e-06,3.63303302180857
"Erc1",1.50400129368825e-10,0.276763631098011,0.245,0.096,2.69727592010052e-06,3.54901427310055
"Adss",1.51345110243492e-10,0.329042966704754,0.426,0.22,2.71422320710678e-06,4.21734526953821
"Ascc1",1.56903789732456e-10,-0.649166270104822,0.168,0.4,2.81391256506186e-06,-8.29695168893124
"Rps12",1.58436839688998e-10,0.425201449068461,0.852,0.706,2.84140628298248e-06,5.43033761842173
"Zfp608",1.60769314570718e-10,0.182341466242571,0.161,0.049,2.88323688751126e-06,2.3260565968085

"Hmgb2",1.65711829768168e-10,0.337617344207615,0.413,0.207,2.971875955
06233e-06,4.2966254044271
"Gsk3b",1.67358423749319e-10,0.334318997910362,0.51,0.278,3.0014059715
2029e-06,4.2513440416259
"Rgs2",1.7035202004192e-10,0.37109059824088,0.265,0.106,3.055093127431
8e-06,4.71236835881923
"Mypop",1.71223976527298e-10,0.126946977676788,0.097,0.021,3.070730795
04055e-06,1.61141351865387
"Rps11",1.73491721993445e-10,0.29838525156072,0.981,0.896,3.1114005422
3045e-06,3.78365556258558
"Ccnt2",1.76821656807344e-10,0.263934543541453,0.361,0.17,3.1711195931
829e-06,3.34178765189738
"Gpbp1",1.78158887140885e-10,0.346488051613396,0.355,0.17,3.1951014819
8464e-06,4.38442228319361
"Prr15",1.79059576610038e-10,0.308455852023834,0.206,0.073,3.211254446
92441e-06,3.90161146572011
"Etnk1",1.79842726015811e-10,0.414384900456174,0.477,0.26,3.2252994483
6756e-06,5.23968355851764
"Sema5a",1.85473656966967e-10,0.312904643301728,0.252,0.1,3.3262845640
4558e-06,3.94687110067034
"Hoxd9",1.873501283692e-10,0.230987459818283,0.323,0.14,3.359937202173
23e-06,2.91127084000519
"Rab19",1.8805171603392e-10,0.0998424406120361,0.09,0.018,3.3725194753
5232e-06,1.25799982268374
"Ankrd13c",1.89251769990016e-10,-0.586168681082626,0.206,0.445,3.39404
124300095e-06,-7.38190897773859
"Pdk1",1.90215291339934e-10,0.190356658829599,0.213,0.076,3.4113210348
9038e-06,2.39628784678417
"Cyth2",1.91702207400669e-10,0.213331274148882,0.297,0.125,3.437987387
52359e-06,2.68384061568032
"Rpl23a",1.92268534672305e-10,0.346895912651284,0.974,0.889,3.44814390
081311e-06,4.36314386480757
"Git2",1.95564966088739e-10,0.30438052623054,0.277,0.116,3.50726210183
544e-06,3.82322482151624
"Zc3hav1",1.96149056440381e-10,0.239087441663826,0.213,0.077,3.5177371
782018e-06,3.00238660223552
"Brd3",1.96510687857016e-10,0.271074597192106,0.316,0.14,3.52422267602
773e-06,3.40357216100071
"Clcnkb",2.00821944537112e-10,0.213128759613206,0.452,0.214,3.60154075
332856e-06,2.67138759018025
"Rbm22",2.00949183811355e-10,0.224379298512066,0.239,0.092,3.603822662
47283e-06,2.81226139899204
"Ube2n",2.0127404777496e-10,0.33789722440812,0.587,0.356,3.60964877279
613e-06,4.23449425467554
"Sept6",2.01997313422089e-10,0.138236288567413,0.103,0.023,3.622619818
91174e-06,1.73186750315666
"Krtcap3",2.0679992919944e-10,0.232713071921471,0.174,0.056,3.70874993
026276e-06,2.91003407166914
"Lasp1",2.08438589124993e-10,0.27131548827723,0.419,0.208,3.7381376573
6762e-06,3.39060877198426

"Rps20",2.10835313946049e-10,0.339538658517392,0.955,0.807,3.78112052030843e-06,4.23930657928693
"Nedd9",2.23528917183216e-10,0.230004794415047,0.258,0.103,4.00876760076379e-06,2.85827572025707
"Arid4b",2.23635835642612e-10,0.284721262837511,0.335,0.156,4.01068507641461e-06,3.53810257856769
"Cenpt",2.24283707669844e-10,0.104437968666442,0.09,0.019,4.02230401335099e-06,1.29750136282523
"Arl13b",2.25582829373046e-10,0.18436556887232,0.148,0.043,4.04560246197621e-06,2.28942952488382
"Nrg1",2.29195164783788e-10,0.262751026513443,0.155,0.046,4.11038608523245e-06,3.25863654812412
"Top1",2.32009976626531e-10,0.344078828079614,0.574,0.336,4.1608669208202e-06,4.26306342929994
"Igf2bp3",2.34034200921011e-10,0.160092685721604,0.148,0.043,4.19716935931741e-06,1.98212358672141
"Piezo1",2.42529349204358e-10,0.271957813936979,0.265,0.11,4.34952134863096e-06,3.35744016730019
"Fam73a",2.50451006723237e-10,0.223340995266654,0.213,0.077,4.49158835457454e-06,2.75006560722397
"Capn5",2.54522092905427e-10,0.163256612433062,0.135,0.037,4.56459921416592e-06,2.00759592367036
"Cuedc1",2.54996113141211e-10,0.192538175660743,0.232,0.087,4.57310029307448e-06,2.36731832446069
"Mboat1",2.56809429303806e-10,0.208279441911905,0.174,0.057,4.60562030513445e-06,2.55938635189672
"Fam199x",2.57931552736605e-10,0.210110633745665,0.206,0.074,4.62574446677827e-06,2.58097239008153
"Lrrcc1",2.59553967690343e-10,0.248192897999169,0.206,0.074,4.6548408565586e-06,3.04721382821433
"Fam49b",2.6060839819197e-10,0.215919700806453,0.239,0.093,4.67375101317479e-06,2.65010094022301
"Tcea3",2.69185681922027e-10,-0.580834481001681,0.039,0.259,4.82757601958963e-06,-7.11009134435997
"Enpp4",2.84213173175216e-10,0.231721348627668,0.213,0.078,5.09707904772433e-06,2.82395167648412
"Sgms1",2.87592942281324e-10,0.243688788872489,0.342,0.155,5.15769182687326e-06,2.96691621919989
"Hivep3",2.9041771595454e-10,0.113273739208776,0.071,0.012,5.20835131792873e-06,1.37800303851356
"Chd4",2.9488917994943e-10,0.345850428804139,0.542,0.312,5.28854255321308e-06,4.20207159428336
"Wnk1",2.95775676888089e-10,0.283277745516651,0.548,0.304,5.30444098931098e-06,3.44096519030234
"Ntn4",3.07146104867613e-10,0.293492194958314,0.265,0.107,5.50835824469578e-06,3.5539685827721
"Fam84a",3.1385534822638e-10,0.156387072071879,0.116,0.029,5.6286818150919e-06,1.89034988956958
"Rbfox2",3.16827432915904e-10,0.172520348744441,0.148,0.044,5.68198318191382e-06,2.0837370418517

"1110038F14Rik",3.27275116041952e-10,0.234287006044265,0.265,0.109,5.86935193109637e-06,2.82216652985018
"Sbds",3.31001146163593e-10,0.275619145204737,0.368,0.178,5.93617455529787e-06,3.31692362205038
"Ybx3",3.31694930428806e-10,0.266828009224155,0.323,0.148,5.94861688231021e-06,3.21056848299787
"Sltm",3.3379997800642e-10,0.272953220026381,0.355,0.168,5.98636880556714e-06,3.28254239501065
"Ube2q2",3.36916069802904e-10,0.169732056458466,0.245,0.094,6.04225279584529e-06,2.03962491197099
"Ran",3.47267057752161e-10,0.335920420719869,0.677,0.428,6.22788741372726e-06,4.02650118129194
"Cryz",3.50073644957165e-10,-0.556945046730436,0.194,0.438,6.2782207486618e-06,-6.67132387985276
"Metap2",3.50444933051447e-10,0.282648763462753,0.613,0.362,6.28487942934466e-06,3.38538709587312
"Frg1",3.51621784981189e-10,0.295828635609807,0.374,0.184,6.30598509185264e-06,3.54225544369068
"Ifngr2",3.62444300666454e-10,0.201360901363456,0.239,0.092,6.50007608815219e-06,2.4049935281424
"Kansl1l",3.68795386996699e-10,0.331812426294166,0.413,0.213,6.6139764703988e-06,3.95730300124612
"Slfn2",3.69731260393082e-10,0.266816230873036,0.219,0.082,6.63076042388953e-06,3.18146098993189
"Trmt1l",3.72930195103841e-10,0.229503142668839,0.297,0.129,6.68813011899228e-06,2.73457038949909
"Hykk",3.74289450022728e-10,-0.566092661851412,0.032,0.249,6.7125069967076e-06,-6.74303428474644
"Hn1l",3.79006911957283e-10,0.216857829808251,0.277,0.117,6.79710995904191e-06,2.58039414517988
"Trim56",3.81717139270157e-10,0.269504786769448,0.245,0.098,6.845715175671e-06,3.20492063817955
"Eif4a1",3.88892992200106e-10,0.33230687017397,0.774,0.516,6.97440692211671e-06,3.94556695366556
"Mast4",3.92089635930671e-10,0.301793344302383,0.284,0.124,7.03173553078066e-06,3.58080127055217
"Nfe2l3",3.93442449962593e-10,0.0904066160872904,0.052,0.007,7.05599689762914e-06,1.07237007177323
"Psme2",3.95630858002472e-10,0.321752722477867,0.49,0.274,7.09524380741634e-06,3.81472791132413
"Esrp1",3.98401533302065e-10,0.157655166615113,0.11,0.027,7.14493309823923e-06,1.86807295567742
"Smchd1",4.01294323206266e-10,0.29291357377482,0.297,0.131,7.19681239238118e-06,3.46864515117673
"Mettl7b",4.07650741187791e-10,-0.539956305828057,0.013,0.223,7.31080839246185e-06,-6.38560788555651
"Actn4",4.13102414523287e-10,0.315128187081453,0.574,0.335,7.40857870206064e-06,3.7225689202982
"Hoxd10",4.20534875692665e-10,0.206436779322815,0.2,0.071,7.54187246067226e-06,2.43493008399631

"Srrt",4.2335204239035e-10,0.256106692909803,0.29,0.125,7.592395528228
53e-06,3.0190787651893
"Bbc3",4.27617173919507e-10,0.16204356456426,0.11,0.027,7.668886397072
44e-06,1.90860405930653
"Arhgef28",4.28894383331785e-10,0.335017000388195,0.4,0.205,7.69179187
067223e-06,3.94494470978362
"Acaa1b",4.31679953661331e-10,-0.670211613014402,0.058,0.279,7.7417482
889623e-06,-7.88764207136896
"Dsg2",4.32031754032012e-10,0.184580409004241,0.161,0.05,7.74805747681
009e-06,2.17215487738579
"Bcl2",4.33530207168997e-10,0.286268380576134,0.232,0.09,7.77493073536
879e-06,3.36783471019042
"Rrad",4.55658447307752e-10,0.19159003942951,0.187,0.064,8.17177859401
723e-06,2.24444358720703
"Farp1",4.56537356683311e-10,0.172948390828652,0.174,0.057,8.187540954
7585e-06,2.02572668029307
"Ldb1",4.73682012206519e-10,0.1620015946662,0.187,0.064,8.495013206911
71e-06,1.89153568157637
"Swt1",4.76347974848028e-10,0.291295550028045,0.342,0.162,8.5428245809
2454e-06,3.39954108020199
"Papss1",4.83837476408977e-10,0.260258374067777,0.335,0.155,8.67714130
19186e-06,3.03326409377101
"Smox",4.84047110531131e-10,0.170080917830954,0.129,0.035,8.6809008802
6531e-06,1.98218853950213
"Hacd4",4.92703658388123e-10,0.155880876832074,0.116,0.029,8.836147409
5326e-06,1.8139327002404
"Ssh2",5.09246219464769e-10,0.215945651144526,0.213,0.079,9.1328216998
8117e-06,2.50575469993488
"Adhfe1",5.13782458908791e-10,-0.511205159421681,0.045,0.271,9.2141746
1807026e-06,-5.92730498886785
"AI661453",5.22434399143755e-10,0.148765295505096,0.174,0.057,9.369338
5142441e-06,1.72241471637427
"Trim24",5.29163981637112e-10,0.258833604519091,0.265,0.11,9.490026846
67996e-06,2.99348029263172
"Xylb",5.48088734839457e-10,-0.526088341394472,0.026,0.239,9.829423370
61082e-06,-6.06586711805948
"Nrbp2",5.49023398063235e-10,0.193548319295828,0.232,0.089,9.846185620
86606e-06,2.23130755856672
"Clint1",5.65735510696952e-10,0.379796607600311,0.465,0.251,1.01459006
488391e-05,4.36706881271776
"Pamr1",5.67989739078583e-10,0.194954802199617,0.168,0.054,1.018632798
06353e-05,2.24090098102261
"Dnajc5",5.70603034702964e-10,0.265907373076054,0.419,0.218,1.02331948
24363e-05,3.05524213977781
"Ddah1",5.70784444098774e-10,-0.57742645520494,0.129,0.357,1.023644822
04674e-05,-6.63437350749403
"Ces1d",5.82295076690651e-10,-0.590611750876115,0.052,0.277,1.04428799
053701e-05,-6.7740747265512
"Etl4",5.82613648640429e-10,0.393056370456697,0.232,0.093,1.0448593174
7174e-05,4.50798049793052

"Eif3c",5.84453531454075e-10,0.305480610619584,0.568,0.327,1.04815896330974e-05,3.50260714204506
"Peak1",5.90340821624349e-10,0.181970060558669,0.135,0.038,1.05871722950111e-05,2.08462489372506
"Usp34",6.05893850933773e-10,0.344795094212857,0.439,0.239,1.08661003226463e-05,3.94096057861339
"Ndufb10",6.23795625706088e-10,-0.444370615788048,0.703,0.768,1.1187150751413e-05,-5.06615593949359
"Ndufc2",6.25231051967574e-10,-0.451107167629665,0.742,0.794,1.12128936859865e-05,-5.14192078961469
"Sf3b2",6.25676674015082e-10,0.250300116176229,0.548,0.301,1.12208854717865e-05,2.85285407975071
"Fam195a",6.28493008374195e-10,-0.549648522040168,0.135,0.369,1.12713936121828e-05,-6.26227894575616
"Vwa5a",6.29150375820897e-10,0.256418969826551,0.265,0.111,1.1283182839972e-05,2.92117546624676
"Ntan1",6.30884027522529e-10,0.233774050779255,0.31,0.139,1.1314274149589e-05,2.66255679437799
"Myo18a",6.48717105697932e-10,0.261995206728131,0.419,0.215,1.16340925735867e-05,2.97667707880381
"Znrf1",6.53147186086998e-10,0.210960562687378,0.2,0.072,1.17135416352842e-05,2.39540760951391
"Chmp5",6.68518050300457e-10,0.283275720912837,0.574,0.33,1.19892027140884e-05,3.20993998928349
"Tst",6.74828657758366e-10,-0.551681508414144,0.032,0.245,1.21023771482385e-05,-6.24619798735664
"Mark2",6.84238486145667e-10,0.25755273798163,0.381,0.191,1.22711330105364e-05,2.9124735729879
"Rnd3",6.97939030215083e-10,0.189435483769957,0.142,0.042,1.25168385678773e-05,2.13843028441491
"Wdr47",7.01081453699415e-10,0.154267164786946,0.135,0.038,1.25731947906453e-05,1.74074195712214
"Gnb2l1",7.01174339384501e-10,0.344300631198152,0.813,0.6,1.25748606025216e-05,3.88502322459911
"Cct2",7.05519685500885e-10,0.275322372072682,0.497,0.276,1.26527900397729e-05,3.10498461678074
"Mrpl12",7.0721698940559e-10,-0.56745480468937,0.458,0.61,1.26832294879998e-05,-6.39818340904877
"Pax2",7.15590457852111e-10,0.24197494208994,0.252,0.102,1.28333992711198e-05,2.72547495234259
"Flot1",7.21540919938485e-10,0.258053211311794,0.31,0.139,1.29401148581768e-05,2.90443492812836
"Erh",7.21765211701402e-10,0.282350117860905,0.684,0.418,1.2944137306653e-05,3.17781319053388
"Itgb5",7.25304615578456e-10,0.251370250018401,0.316,0.145,1.3007612975784e-05,2.82790921908634
"Sim1",7.30607347011782e-10,0.281382834018906,0.187,0.065,1.31027121613093e-05,3.16350034392505
"Pipox",7.40494423743955e-10,-0.552817451625949,0.052,0.271,1.32800269954241e-05,-6.20772502654861

"Il1r1",7.49780131829822e-10,0.144239468066512,0.11,0.027,1.3446556884
236e-05,1.61790345930321
"Socs3",7.55748391203907e-10,0.40244526345794,0.226,0.087,1.3553591647
8509e-05,4.51095220506792
"Hdac1",7.56559762084064e-10,0.295717544857047,0.31,0.142,1.3568142773
2156e-05,3.31433894624485
"Tmem171",7.59725062434346e-10,0.207131322382417,0.206,0.075,1.3624909
2696976e-05,2.32061873631615
"Ier5l",7.68316912849454e-10,0.171118478995803,0.168,0.054,1.377899551
50421e-05,1.91522050758337
"Tspan6",7.68688977057015e-10,0.144844599013383,0.123,0.033,1.37856681
145405e-05,1.62108352258632
"Mpzl1",7.70159455390366e-10,0.234604417530214,0.213,0.081,1.381203967
29708e-05,2.62521637199778
"Lsr",8.01033382065979e-10,0.234663779588743,0.31,0.139,1.436573267397
13e-05,2.61665716161526
"Car12",8.11456711009709e-10,-0.683001508908597,0.097,0.318,1.45526646
552481e-05,-7.60709079659792
"Zranb2",8.18823756995512e-10,0.294155121653626,0.439,0.235,1.46847852
579575e-05,3.2735636954256
"Smarcd3",8.22892692175511e-10,0.14684280179449,0.129,0.036,1.47577575
414756e-05,1.63344139449711
"Apom",8.30954120240803e-10,-0.63269826951131,0.013,0.218,1.4902331192
3986e-05,-7.03180407606735
"Synpo",8.34628403067456e-10,0.128939697701821,0.084,0.017,1.496822578
06117e-05,1.4324660008911
"Hspb8",8.51217173994674e-10,0.185589419007958,0.116,0.03,1.5265728798
4205e-05,2.05816813308441
"Naaladl2",8.52600310585138e-10,0.172631353833104,0.123,0.033,1.529053
39700339e-05,1.91418420386246
"Rpl37",8.79727953159001e-10,0.292927058869484,0.968,0.933,1.577704111
19535e-05,3.23888124049996
"Shoc2",8.96984559410385e-10,0.214622415926054,0.31,0.139,1.6086521088
4658e-05,2.36890110465624
"Pabpc1",9.17445029666124e-10,0.322511023726539,0.748,0.468,1.64534591
620323e-05,3.55245080534388
"Prr13",9.18776786759855e-10,0.332912433342494,0.574,0.34,1.6477342893
7512e-05,3.66653916728452
"Snrpe",9.26174855085804e-10,0.308709050401276,0.684,0.438,1.661001985
11088e-05,3.39749882461549
"Bphl",9.28042900916492e-10,-0.64181792584526,0.245,0.453,1.6643521385
0364e-05,-7.06223682288072
"Dpep1",9.29443048887353e-10,-0.560843303863985,0.032,0.245,1.66686316
387458e-05,-6.170387905794
"Hspb2",9.31074729504738e-10,0.124012375915737,0.084,0.017,1.669789419
8938e-05,1.36416440274727
"Gm26724",9.39245776809269e-10,0.137358841377351,0.097,0.022,1.6844433
7612974e-05,1.50977837211239
"Gmds",9.42226504508641e-10,0.243018083556552,0.219,0.084,1.6897890131
858e-05,2.67036090244621

"Zc3h7a",9.53701679383679e-10,0.264696639780867,0.284,0.126,1.71036859
180669e-05,2.90536764277061
"Vav3",9.61019866518086e-10,0.203312015092464,0.181,0.061,1.7234930286
1354e-05,2.23004255792208
"Mfsd10",9.66729189216172e-10,0.201931172657032,0.155,0.048,1.73373212
794028e-05,2.21370058429372
"Zdhhc18",9.6932601223339e-10,0.176170052830779,0.135,0.039,1.73838927
033936e-05,1.93081787506942
"1190002N15Rik",9.81921401404402e-10,0.109411069809831,0.097,0.022,1.7
6097784127865e-05,1.19772913214194
"Arl2bp",9.85548842861515e-10,0.202998474244554,0.381,0.185,1.76748329
478784e-05,2.22148716899782
"St6gal1",1.02974479601654e-09,0.211148246281465,0.148,0.045,1.8467443
1717606e-05,2.30141056472586
"Camsap1",1.03691909507889e-09,0.176214072644494,0.148,0.045,1.8596107
0511447e-05,1.91942205911593
"Diaph1",1.04302487341203e-09,0.235321874890276,0.323,0.15,1.870560807
97713e-05,2.56187563895334
"Pcnp",1.06511152942243e-09,0.267990957524355,0.49,0.272,1.91017101686
619e-05,2.9119181076075
"Ifi27",1.07152337560624e-09,0.420207183907191,0.471,0.265,1.921670021
81224e-05,4.56333692013374
"Cfb",1.07593182358826e-09,-0.461612677999767,0,0.197,1.92957613242319
e-05,-5.01109417685591
"Nop58",1.09044319727585e-09,0.285348215526148,0.258,0.109,1.955600829
9945e-05,3.0938104089399
"Pdcd6",1.09279452201352e-09,0.24873949085246,0.555,0.32,1.95981769577
904e-05,2.69635448965522
"Snrpg",1.11722420969347e-09,0.292874767098045,0.729,0.461,2.003629897
66426e-05,3.16830897417028
"Appl1",1.11924856165235e-09,0.262660900982234,0.258,0.109,2.007260370
46733e-05,2.84098093141469
"Ddx23",1.12160825533557e-09,0.158218507555264,0.123,0.033,2.011492245
11881e-05,1.71098263136295
"Cdk11b",1.12584249900082e-09,0.267157387141817,0.361,0.177,2.01908593
770807e-05,2.88804630922832
"Thoc2",1.12736812932096e-09,0.278806792081172,0.406,0.209,2.021822003
1242e-05,3.01360208939031
"Ndufb6",1.13341973745058e-09,-0.43148603626985,0.703,0.775,2.03267495
714388e-05,-4.66159081644166
"Slc35g1",1.14126229384795e-09,0.241530764926539,0.213,0.08,2.04673979
778691e-05,2.60772972217583
"Ube2h",1.16054269410098e-09,0.241177873701548,0.323,0.148,2.081317267
6007e-05,2.5998792623323
"Tsc22d2",1.17814410145133e-09,0.265276088956607,0.271,0.117,2.1128836
3154282e-05,2.85566308935011
"Atp6v1g1",1.18465760455705e-09,-0.44074254724221,0.761,0.803,2.124564
94801262e-05,-4.74210703403461
"Osmr",1.22287956048917e-09,0.134520092608605,0.123,0.033,2.1931122037
8128e-05,1.44307826099324

"Cdc42ep3",1.27145371440362e-09,0.0924235256847109,0.071,0.013,2.28022
509141145e-05,0.987882838198324
"Eml2",1.27268532697105e-09,0.25145725070257,0.219,0.084,2.28243386538
988e-05,2.68749541035099
"C77080",1.2770068231962e-09,0.21069606757692,0.245,0.1,2.290184036720
07e-05,2.25113858004136
"Ino80",1.28280462385913e-09,0.159758550333149,0.161,0.052,2.300581812
42897e-05,1.70618352061794
"Cdk6",1.30721631267892e-09,0.315296608925909,0.232,0.093,2.3443617351
5838e-05,3.36134949034704
"Sptbn1",1.34509837609179e-09,0.354671599421003,0.723,0.486,2.41229942
768301e-05,3.77099082553629
"Lrrk1",1.37475465666559e-09,0.159779304970282,0.148,0.045,2.465485001
26407e-05,1.69534421471915
"2810402E24Rik",1.37498124109905e-09,0.168963151477323,0.161,0.052,2.4
6589135778703e-05,1.79276191127701
"Ing1",1.38347473571021e-09,0.188374279672084,0.219,0.084,2.4811235910
227e-05,1.99756116366026
"Pcnt",1.39066174539108e-09,0.170835947072408,0.187,0.065,2.4940127741
8437e-05,1.81069575446422
"Cisd3",1.3950342318274e-09,-0.585933988195115,0.187,0.404,2.501854391
35925e-05,-6.20849399135152
"Iah1",1.39539173168251e-09,-0.558396540141094,0.232,0.467,2.502495531
59942e-05,-5.91656705163153
"Sik2",1.42797066143073e-09,0.128312317014348,0.142,0.042,2.5609225842
0987e-05,1.35658939924219
"Vprbp",1.43902201518004e-09,0.201723745677832,0.194,0.069,2.580742082
02389e-05,2.13118080764261
"Cabin1",1.44704326335709e-09,0.183015064011153,0.187,0.066,2.59512738
850461e-05,1.93250910956239
"Sfr1",1.45351856140085e-09,0.271691693322078,0.419,0.218,2.6067401880
1629e-05,2.86765827543519
"Klf11",1.47375809933695e-09,0.160169742063552,0.116,0.031,2.643037775
35088e-05,1.68834869639954
"Atp5c1",1.482719473733e-09,-0.402700143900455,0.729,0.801,2.659109104
19276e-05,-4.24241956725462
"Setd2",1.49404138691194e-09,0.251956782664133,0.277,0.121,2.679413823
28787e-05,2.65243154604542
"Spink12",1.5283456287979e-09,0.17824295353364,0.097,0.022,2.740935050
68616e-05,1.87237562521694
"D130051D11Rik",1.5318814842381e-09,0.083973058415853,0.052,0.007,2.74
727625383261e-05,0.881911552753667
"Rpl37a",1.53916925670073e-09,0.27285194067541,1,0.965,2.7603461449671
e-05,2.86428217723349
"Ryk",1.54126054608008e-09,0.24160838519515,0.277,0.121,2.764096663340
02e-05,2.53597273230111
"Phf3",1.54427778619613e-09,0.295837682551449,0.374,0.192,2.7695077817
6414e-05,3.10459633274066
"Emp3",1.55798204728819e-09,0.216111355571587,0.206,0.077,2.7940850036
0665e-05,2.26601849871474

"Zfp131",1.57311166647732e-09,0.263117111242089,0.219,0.086,2.82121846
266043e-05,2.75635081318902
"Phxr4",1.583579709767e-09,0.126049567605282,0.116,0.03,2.839991851496
14e-05,1.31962858895115
"Kctd10",1.58534051486182e-09,0.205102752139416,0.148,0.046,2.84314967
935319e-05,2.14701827271702
"Arl6ip1",1.62057510280094e-09,-0.411365211285606,0.761,0.8,2.90633938
93632e-05,-4.29713379772968
"Fras1",1.63542733185049e-09,0.265353249451912,0.206,0.076,2.932975376
94067e-05,2.76946747065551
"Pnmal2",1.6424498794319e-09,0.0762405258263968,0.065,0.011,2.94556961
377318e-05,0.795388682277529
"Fcer1g",1.65746202683531e-09,0.479546290124581,0.484,0.272,2.97249239
892644e-05,4.99856258673009
"Ascc3",1.68086504788055e-09,0.23923369052778,0.239,0.096,3.0144633768
6898e-05,2.49030397118419
"Srd5a1",1.69471978022238e-09,0.294582180833878,0.213,0.081,3.03931045
385081e-05,3.0640361055834
"Bicd1",1.72783796445323e-09,0.17525048432322,0.265,0.11,3.09870460545
043e-05,1.81944024279631
"Sorl1",1.72966873577075e-09,0.258454809983866,0.432,0.226,3.101987910
73126e-05,2.68298896165832
"Ifit3",1.73578910774694e-09,0.375487677685675,0.116,0.031,3.112964185
83336e-05,3.89656708142775
"Atf7ip",1.76273141409668e-09,0.246055422250334,0.271,0.118,3.16128251
804098e-05,2.54961340671156
"Las1l",1.78457608419192e-09,0.300035213007557,0.31,0.146,3.2004587493
8979e-05,3.10525383352195
"Arhgef11",1.80402129012889e-09,0.105877074152933,0.11,0.027,3.2353317
8171715e-05,1.09464125710952
"Dek",1.80482073315292e-09,0.292492047124631,0.439,0.23,3.236765502836
44e-05,3.02388542898641
"Cbx3",1.80769520166569e-09,0.380550679017111,0.516,0.304,3.2419205746
6725e-05,3.93366086209782
"Arpc5",1.83582811461055e-09,0.297579510938439,0.581,0.35,3.2923741407
4255e-05,3.07141232735457
"Prdx1",1.83602348792065e-09,-0.399822013674019,0.935,0.942,3.29272452
323689e-05,-4.12664701386414
"Zfp664",1.84107098889843e-09,0.236778542417857,0.303,0.138,3.30177671
149044e-05,2.44319104206498
"Rbpms",1.85223742709518e-09,0.328254544373369,0.426,0.228,3.321802601
75249e-05,3.3850980316195
"Wdr70",1.853169831236e-09,0.180924727853107,0.155,0.049,3.32347477533
865e-05,1.8656803445108
"Ckmt1",1.85775499873682e-09,0.140759367669942,0.329,0.145,3.331697814
73461e-05,1.45115073916974
"Car14",1.87138180695699e-09,-0.52123025799091,0.026,0.231,3.356136132
59666e-05,-5.36978454293327
"Shisa5",1.88531700998721e-09,0.290507766409697,0.458,0.25,3.381127525
71106e-05,2.99069501594399

"Hmgb3",1.89571967241078e-09,0.34768716262043,0.335,0.164,3.3997836605
0149e-05,3.57742750465293
"Mme",1.95513176468332e-09,0.178267084943311,0.303,0.133,3.50633330678
307e-05,1.82872697361161
"Arf1",1.95682796814926e-09,0.362488565691185,0.723,0.496,3.5093752780
7888e-05,3.71822190493127
"Pdlim4",2.00629578719292e-09,0.118719287640291,0.084,0.018,3.59809086
475178e-05,1.21479773156951
"Crlf3",2.01775094507081e-09,0.212936043142443,0.155,0.049,3.618634544
88998e-05,2.17766043894164
"Tspan13",2.03478241893995e-09,0.372596626365185,0.187,0.068,3.6491787
901269e-05,3.80735005270928
"Bnip3l",2.0548603261972e-09,0.347245599537978,0.535,0.318,3.685186509
00206e-05,3.54489293017769
"Cdc42bpa",2.0571971330802e-09,0.243752954021766,0.284,0.126,3.6893773
3846603e-05,2.48810042058611
"Cldn2",2.06656798343417e-09,-0.525742228571676,0.103,0.324,3.70618302
149084e-05,-5.36410745133038
"Uqcrfs1",2.11075461558248e-09,-0.464890163596575,0.665,0.731,3.785427
32758563e-05,-4.73340317950443
"Itgb4",2.11493587434824e-09,0.156727548540009,0.116,0.031,3.792925997
05614e-05,1.59545317289314
"Gcnt2",2.11896692342464e-09,0.290447988841334,0.252,0.106,3.800155280
46975e-05,2.9561458036319
"Tax1bp3",2.13086282496766e-09,0.324936661944391,0.381,0.196,3.8214893
9029699e-05,3.3053484055502
"Serpinf2",2.15701864796490e-09,-0.533626523487179,0.019,0.217,3.86839
724326024e-05,-5.42169093973556
"2700094K13Rik",2.18511292394665e-09,0.253162110607692,0.303,0.14,3.91
878151780593e-05,2.56887256807903
"Nfatc1",2.18797857671093e-09,0.0797489815383609,0.084,0.018,3.9239207
7947338e-05,0.809119937304168
"Fam151a",2.229167369107e-09,-0.584571598922901,0.039,0.242,3.99778875
97565e-05,-5.92006418249653
"Ankhd1",2.24739297649124e-09,0.273938209854247,0.303,0.141,4.03047456
403939e-05,2.77199206960528
"1700016C15Rik",2.2994265103638e-09,-0.562570252820151,0.09,0.314,4.12
379150368644e-05,-5.67979504019499
"Prkci",2.3205769178442e-09,0.181521204004269,0.181,0.063,4.1617226444
6179e-05,1.83100372753641
"Smim1",2.33583685676079e-09,-0.585276920173194,0.245,0.45,4.189089818
9148e-05,-5.8998500369887
"Chst9",2.33592994231721e-09,0.0806840072861484,0.052,0.007,4.18925675
855168e-05,0.813327239014324
"Bcl7a",2.34775283000605e-09,0.340719272705259,0.413,0.222,4.210459925
33286e-05,3.43286714311298
"Casp1",2.35887226526047e-09,0.0780459313166532,0.052,0.007,4.23040152
051813e-05,0.785971584465092
"Shroom3",2.35948424112071e-09,0.224516850683574,0.239,0.097,4.2314990
3802588e-05,2.26096756726817

"Dhx30",2.37362205720827e-09,0.249328439804932,0.297,0.135,4.25685379739732e-05,2.50933993353805
"Tmpo",2.41764868926291e-09,0.236314741167541,0.323,0.152,4.3358111593241e-05,2.37402184889122
"Luc7l",2.41812554264023e-09,0.24700932437149,0.252,0.106,4.33666634817099e-05,2.48141109598871
"Rbm8a",2.42315505635827e-09,0.272057642843352,0.503,0.279,4.34568627807293e-05,2.73247671184566
"Dnajc19",2.43108230281693e-09,-0.615247786622324,0.439,0.574,4.35990300187188e-05,-6.17738042345643
"Tbrg1",2.44138114611691e-09,0.215575697436544,0.394,0.198,4.37837294744607e-05,2.16357122320682
"Ip6k2",2.46493555869946e-09,0.231854312418954,0.297,0.135,4.42061543097161e-05,2.32472123958436
"Rps27a",2.51436657430438e-09,0.384038382844404,0.935,0.883,4.50926501435747e-05,3.84299194552074
"Top2b",2.51610962954596e-09,0.257592547179851,0.329,0.158,4.51239100962773e-05,2.57749634703644
"Ubap2l",2.53142363744884e-09,0.295580586609278,0.445,0.247,4.53985515140074e-05,2.95581483944758
"Apbb1",2.53461525320255e-09,0.124321358364288,0.123,0.034,4.54557899509346e-05,1.24306071235633
"Yy1",2.60909846075435e-09,0.258894830376391,0.342,0.167,4.67915717951685e-05,2.58113161152822
"Ankrd27",2.61028259537945e-09,0.216641656691451,0.148,0.047,4.6812808065535e-05,2.159777305149
"Ets1",2.61399655050107e-09,0.187816641240438,0.103,0.026,4.68794141366863e-05,1.8721434914229
"Sox11",2.66473840899854e-09,0.0991530043973993,0.058,0.009,4.77894186269799e-05,0.986444120564218
"Cspp1",2.66837044745072e-09,0.33160331963772,0.381,0.195,4.78545556045813e-05,3.2985723711154
"Mdn1",2.7040340761905e-09,0.160418306614734,0.135,0.04,4.84941471224005e-05,1.5936062773542
"Sugt1",2.76306257890174e-09,0.27159324434049,0.355,0.176,4.95527642900237e-05,2.69216056865845
"Ubr4",2.7832708376457e-09,0.244398096707657,0.303,0.139,4.9915179202338e-05,2.42080846150396
"Sri",2.79085613768707e-09,0.270430517223909,0.684,0.423,5.005121397328e-05,2.67792840649131
"Prpf19",2.79649536697162e-09,0.272556167708435,0.361,0.181,5.01523479112691e-05,2.69842740964573
"Smim15",2.85034507920485e-09,0.224837877036591,0.31,0.144,5.11180886504598e-05,2.2217067337301
"Msrb1",2.85479220972619e-09,-0.633672965006198,0.374,0.549,5.11978434892296e-05,-6.26057049003242
"Gm12576",2.85846147812406e-09,0.269660169706925,0.174,0.06,5.1263648148677e-05,2.66384571762275
"Tspan17",2.86198343887819e-09,0.149685872284506,0.129,0.037,5.13268109928415e-05,1.47849186379133

"Fip1l1",2.87647639231657e-09,0.245160142442199,0.284,0.128,5.15867276198054e-05,2.42028126873819
"Srp14",2.95730267437656e-09,0.278093881247855,0.626,0.384,5.30362661622693e-05,2.73770483910845
"Pvr",2.96097735637689e-09,0.177108706726479,0.129,0.037,5.31021679092632e-05,1.74333285838156
"Skp2",2.97723698508542e-09,0.0888502919506156,0.077,0.016,5.33937680905218e-05,0.874092870068147
"Sardh",3.01619252314616e-09,-0.556894290941253,0.103,0.315,5.40923967101032e-05,-5.47138445417979
"H2afy",3.0327715468848e-09,0.257531231525062,0.535,0.308,5.4389724921832e-05,2.52878551309596
"Ywhae",3.03675053763739e-09,0.338722761159694,0.8,0.59,5.44610841419889e-05,3.32558825389239
"Rnpep",3.04104292785509e-09,0.239581781711438,0.323,0.154,5.45380638681531e-05,2.35188131677809
"Axl",3.07341678789899e-09,0.134358629031672,0.116,0.031,5.51186566741804e-05,1.31752371281639
"Ckb",3.0899627026706e-09,0.267128671993362,0.445,0.236,5.54153911096946e-05,2.61803546995806
"Hnrnpk",3.09139868567212e-09,0.32076619600565,0.787,0.532,5.54411440288437e-05,3.14356920704879
"Cxcl16",3.12362304197208e-09,0.299581379172954,0.516,0.289,5.60190556347273e-05,2.93284737179764
"Ssh3",3.13029520952085e-09,0.132114508819489,0.142,0.043,5.6138714287547e-05,1.29309518161514
"Gltscr2",3.13379516039189e-09,0.252216626362378,0.529,0.294,5.62014824064682e-05,2.46833501936769
"Pdgfb",3.15413486371516e-09,0.198811218253583,0.155,0.05,5.65662546458677e-05,1.94439318971465
"Slc18a1",3.16619501967627e-09,-0.496802638067753,0.006,0.197,5.67825414828743e-05,-4.85688251309244
"Hnrnph3",3.1753084080935e-09,0.273824380308455,0.316,0.15,5.69459809907488e-05,2.67619723614335
"Rock1",3.19116850579813e-09,0.268662441650146,0.4,0.207,5.72304159829836e-05,2.62440892580257
"Tmem176b",3.22006337553133e-09,0.505311690745369,0.929,0.779,5.77486165767789e-05,4.93154455607617
"Thbs1",3.22081028648879e-09,0.360190831739959,0.206,0.08,5.776201167789e-05,3.51516688603057
"Samd5",3.233049275361e-09,0.0975086762065213,0.097,0.023,5.79815057043242e-05,0.951234820127447
"Phactr1",3.31627687616392e-09,0.164580579623976,0.155,0.049,5.94741094971237e-05,1.60136401585545
"Nras",3.33882191356751e-09,0.199067126457866,0.297,0.133,5.98784321979197e-05,1.93556832563439
"Nipbl",3.39016915717639e-09,0.279509284696531,0.355,0.176,6.07992936648013e-05,2.71345723720911
"Arf4",3.40039218656196e-09,0.253345362000176,0.484,0.269,6.09826334738022e-05,2.45869683324047

"Ckap4",3.41660095998029e-09,0.233616699086928,0.206,0.079,6.127332161
62865e-05,2.26612076617084
"1110004F10Rik",3.4418168764972e-09,0.218379202485291,0.484,0.265,6.17
255438631007e-05,2.11670870982143
"Tead1",3.44295074640595e-09,0.324312432128444,0.297,0.14,6.1745878686
0443e-05,3.14339284101726
"Rpsa",3.46409233010284e-09,0.29354140073539,0.929,0.747,6.21250318480
644e-05,2.84334814061683
"Thap3",3.52978151623725e-09,0.192644509456032,0.239,0.098,6.330310171
21988e-05,1.86240548042283
"Slc7a1",3.53439150753678e-09,0.221807333096372,0.129,0.038,6.33857772
961647e-05,2.14404980359762
"Grn",3.53607167452122e-09,0.238379338486758,0.587,0.342,6.34159094108
636e-05,2.30412600658776
"Mif4gd",3.55275415531046e-09,0.234886696006978,0.387,0.195,6.37150930
213378e-05,2.26926129485001
"Pabpn1",3.57694587208413e-09,0.229505595907418,0.394,0.201,6.41489472
699568e-05,2.21571653496726
"Setd7",3.72168134563807e-09,0.208866662043824,0.187,0.068,6.674463325
26731e-05,2.00817706690444
"Sox6",3.72737338075955e-09,0.256231522655581,0.297,0.135,6.6846714210
5417e-05,2.46318140465541
"Rrp1",3.73059968185406e-09,0.250950597085483,0.477,0.26,6.69045746943
708e-05,2.41219817340391
"Ltbp1",3.73742548679221e-09,0.10034422036842,0.065,0.011,6.7026988680
1315e-05,0.964349621018099
"Minos1",3.85063116951708e-09,-0.383539919727526,0.794,0.856,6.9057219
3941193e-05,-3.67453301647139
"Grik5",3.85764144023938e-09,0.110661093410488,0.123,0.034,6.918294158
9253e-05,1.05999563842836
"Mtcl1",3.88665863553468e-09,0.116936413135121,0.11,0.028,6.9703335969
6789e-05,1.11922909183517
"Apoe",3.9131818291421e-09,0.455192880485114,0.852,0.659,7.01790029238
345e-05,4.3536747328253
"Anxa4",4.0593861659548e-09,0.183923176709638,0.406,0.201,7.2801031500
2335e-05,1.75237964439533
"Atp11b",4.06075474678942e-09,0.209821976060715,0.245,0.103,7.28255756
289215e-05,1.99906699068684
"Dnajb4",4.08780435457572e-09,0.299731378732494,0.252,0.107,7.33106832
949609e-05,2.85368377314926
"Psmb10",4.0885283196818e-09,0.249388715667638,0.271,0.119,7.332366688
51735e-05,2.37433697084738
"Paxbp1",4.10250518214677e-09,0.272244492533245,0.252,0.11,7.357432793
66202e-05,2.59100920419122
"Trpm6",4.10872784333102e-09,0.150153348452144,0.103,0.026,7.368592514
22986e-05,1.42881403047615
"Marveld3",4.16463289903719e-09,0.186490822512305,0.187,0.068,7.468852
6411333e-05,1.77207012347197
"Gltp",4.24878398286254e-09,0.228551535652413,0.271,0.119,7.6197691948
6568e-05,2.1671666598004

"Wdr1",4.26535797402903e-09,0.247338506889425,0.413,0.219,7.6494929906
2366e-05,2.34434513062383
"Stag2",4.28190846298873e-09,0.241357140770358,0.29,0.133,7.6791746375
2398e-05,2.28671732692838
"Pola1",4.33864364591615e-09,0.139598680875059,0.097,0.023,7.780923514
58602e-05,1.32077807947757
"Rab5a",4.35243124347809e-09,0.221629407563652,0.432,0.23,7.8056501920
5361e-05,2.096188137377
"Tmcc1",4.42232152214906e-09,0.259559098544617,0.252,0.109,7.930991417
82212e-05,2.45079527161586
"Lxn",4.43297203703514e-09,0.166410195576943,0.155,0.05,7.950092051218
82e-05,1.57086930536032
"Lilrb4a",4.50322857130101e-09,0.131443577521215,0.09,0.021,8.07609011
977123e-05,1.23872658870569
"1810011O10Rik",4.5650779401712e-09,0.186553081057908,0.135,0.041,8.18
701077790302e-05,1.75553475202395
"Hook3",4.63211744415233e-09,0.294214674427427,0.342,0.172,8.307239424
34279e-05,2.76438168207542
"Eif3a",4.70932584774723e-09,0.318388181836302,0.606,0.373,8.445704975
34989e-05,2.98624790710276
"Ttr",4.71242595677027e-09,-1.43579151087695,0.084,0.286,8.45126471087
179e-05,-13.4657277099185
"Sparc",4.73144282594417e-09,0.243418614653779,0.258,0.109,8.485369564
04827e-05,2.28194776637951
"Hprt",4.81509130841487e-09,0.23623124743734,0.523,0.303,8.63538475251
122e-05,2.21042929399885
"Ranbp2",4.83042386351706e-09,0.21759713774531,0.31,0.145,8.6628821568
315e-05,2.03537707639807
"Igsf11",4.89191887872163e-09,-0.515348539466398,0.026,0.219,8.7731673
1709937e-05,-4.8139879851172
"Ciz1",4.89698671845285e-09,0.165181018515616,0.187,0.068,8.7822559808
7333e-05,1.54282245162585
"Kctd12",4.92250317087545e-09,0.26732759798842,0.219,0.088,8.828017186
64803e-05,2.49550179853528
"Bmyc",4.92749608809821e-09,0.232917121459577,0.181,0.065,8.8369714843
9532e-05,2.17404404201121
"Fam204a",5.08118610748121e-09,0.200952481511283,0.232,0.095,9.1125991
651568e-05,1.86951468734892
"Fzd6",5.19200551564395e-09,0.14683415577999,0.142,0.044,9.31134269175
586e-05,1.36286943306636
"Sh3yl1",5.25336718393489e-09,0.23080715217392,0.284,0.128,9.421388707
66882e-05,2.13956913680892
"Wwc2",5.26789940098037e-09,0.310731994140448,0.394,0.208,9.4474507857
1819e-05,2.87960948241171
"Rcn2",5.30411964878462e-09,0.252496124240926,0.303,0.143,9.5124081781
3034e-05,2.33819702883166
"Hltf",5.39562775231099e-09,0.175375820720415,0.187,0.068,9.6765188109
9453e-05,1.6210378645049
"Plekhh2",5.40437408835680e-09,0.078384926847436,0.052,0.008,9.6922044
9005908e-05,0.724402419269174

"Ssb",5.5270546987463e-09,0.292478839312415,0.503,0.295,9.912219896731
61e-05,2.69640837857288
"Ccl12",5.5831333653758e-09,0.176326469036582,0.161,0.054,0.0001001279
1377465,1.6238013947026
"Slc7a9",5.60919184097226e-09,-0.500043159076844,0.026,0.218,0.0001005
95246475996,-4.60260003021892
"Tcf7l2",5.6137647720855e-09,0.286682466213638,0.29,0.136,0.0001006772
57422581,2.6385080594297
"Arid1b",5.69468330723102e-09,0.238400811564659,0.252,0.109,0.00010212
8450431881,2.19073162350294
"Cnep1r1",5.69759476191996e-09,0.185422008182501,0.245,0.102,0.0001021
80664460273,1.70379983220137
"Vat1",5.76006182305784e-09,0.182616994295363,0.219,0.087,0.0001033009
48734719,1.67603393729598
"Irf3",5.78555133961471e-09,0.23904455288287,0.29,0.134,0.000103758077
72465,2.19286290566554
"Tnrc6b",5.81559547528028e-09,0.292185390185347,0.342,0.172,0.00010429
6889253677,2.67883426133937
"Efna1",5.96719865824091e-09,0.180735586397403,0.206,0.08,0.0001070157
40736893,1.65238135654131
"Ezh1",5.97123320160781e-09,0.215797730914639,0.213,0.084,0.0001070880
96237635,1.97279237090812
"Eif4a2",6.09349749860671e-09,0.348813892785065,0.723,0.493,0.00010928
0784140013,3.1817373116344
"Lrrc19",6.11842509373514e-09,-0.443492051808321,0,0.182,0.00010972783
5631046,-4.04354209952865
"Ddx24",6.32326780003441e-09,0.270104725674195,0.413,0.22,0.0001134014
84725817,2.45378692831686
"Slc1a5",6.32628592713536e-09,0.213550093471114,0.245,0.103,0.00011345
5611817246,1.93991016553287
"Atp5a1",6.38809220880684e-09,-0.333035588638097,0.877,0.883,0.0001145
64045672742,-3.0220903326874
"Magoh",6.39508251169307e-09,0.251591829582714,0.361,0.182,0.000114689
409764704,2.28276383707022
"Efcab14",6.47274088600544e-09,0.274104312222041,0.303,0.145,0.0001160
82135049622,2.4837174357361
"Lman1",6.50720524554337e-09,0.256822022734604,0.458,0.252,0.000116700
218873575,2.32575510609047
"Aldh7a1",6.71185015760785e-09,-0.562404562272903,0.148,0.356,0.000120
370320726539,-5.07566605857418
"Ankrd10",6.72084268009172e-09,0.150559386051058,0.219,0.086,0.0001205
31592624765,1.35858747421141
"Tmbim1",6.7352725236537e-09,0.196465229223982,0.187,0.069,0.000120790
377439205,1.77240201480118
"Pik3r3",6.80687502198036e-09,0.147853917908037,0.123,0.035,0.00012207
4496644196,1.33229377449574
"Ptgs1",6.80702275374914e-09,0.291203739202253,0.148,0.048,0.000122077
146065737,2.62399535906004
"Bod1l",6.82189797196236e-09,0.278710709584087,0.329,0.16,0.0001223439
18229173,2.51081405597666

"Agtpbp1",6.84517488289169e-09,0.131524548674443,0.187,0.067,0.0001227
6136634978,1.1844138354372
"Ptov1",7.03736449672755e-09,0.270850279552494,0.419,0.227,0.000126208
094884312,2.43157963738724
"Tnk2",7.11728173624792e-09,0.108742997384405,0.077,0.016,0.0001276413
3065787,0.975020851096677
"Ccnl2",7.12838741902721e-09,0.224339313163751,0.419,0.226,0.000127840
499972834,2.0111407349027
"Ajuba",7.28010672988209e-09,0.181426075907261,0.174,0.062,0.000130561
434093705,1.62261435045302
"Atp11a",7.29407159711934e-09,0.255093854649378,0.542,0.305,0.00013081
1880022738,2.2809855506259
"Asap2",7.30212070456666e-09,0.167013211608572,0.174,0.061,0.000130956
232715699,1.49320623479677
"Gatad2b",7.31513642937685e-09,0.268442443861498,0.31,0.151,0.00013118
9656724444,2.39957117411575
"Ninl",7.33319382057933e-09,0.217667764888061,0.206,0.08,0.00013151349
797827,1.94516644604709
"Fam129b",7.35385004735474e-09,0.169353735737717,0.135,0.042,0.0001318
8394674926,1.51293653747948
"Prpf31",7.36716669991448e-09,0.212842890069228,0.239,0.1,0.0001321227
67596266,1.90106565796211
"Zfp652",7.40370141649556e-09,0.17846564908227,0.155,0.051,0.000132777
981203431,1.59313289191012
"Csnk2a1",7.50732001140581e-09,0.28251300985687,0.484,0.281,0.00013463
6277084552,2.51801971475791
"Klf13",7.54780480424547e-09,0.307214761531969,0.271,0.125,0.000135362
331359338,2.73653251998073
"Ppp1r1a",7.58529732188523e-09,0.249047303092661,0.374,0.18,0.00013603
472217069,2.21716861882058
"Colgalt1",7.68720510584587e-09,0.170687676633989,0.232,0.095,0.000137
86233636824,1.51728627156413
"Bag2",7.77205340734686e-09,0.279917454486584,0.206,0.083,0.0001393840
05807359,2.48518492261075
"N4bp3",7.7797742445143e-09,0.163162639739382,0.123,0.036,0.0001395224
7130112,1.44844123556813
"Tmem128",7.83344991031408e-09,0.18498117965606,0.31,0.143,0.000140485
090691573,1.64085875616379
"Baz2a",8.06867401290282e-09,0.207576907230943,0.245,0.105,0.000144703
599747399,1.8351507055391
"Slc35f5",8.15983222767996e-09,0.297916063291206,0.271,0.124,0.0001463
38431171212,2.63047627530009
"Hnrnpab",8.1877260112515e-09,0.276376193622232,0.581,0.348,0.00014683
8678285784,2.43934492723774
"Syf2",8.20714100271082e-09,0.223528066464058,0.439,0.237,0.0001471868
66742616,1.97236864634736
"Zfand6",8.24096645465269e-09,0.245178991442521,0.516,0.3,0.0001477934
92397741,2.16240382200567
"Rab11fip3",8.28764750784244e-09,-0.500319336315588,0.09,0.3,0.0001486
30670405646,-4.40983767061451

"Fgfbp3",8.31187759316426e-09,0.0848852884062829,0.077,0.016,0.000149065212755808,0.747935029268912
"Ssbp3",8.59566938869973e-09,0.229622998147501,0.31,0.147,0.000154154734816941,2.01552819434418
"Hnrnpa0",8.60570057534472e-09,0.30998342972164,0.406,0.221,0.000154334634118232,2.72053465571102
"Ilf2",8.74712513743223e-09,0.253303260625458,0.31,0.149,0.00015687094221471,2.21895862953511
"Efemp2",8.77898970626564e-09,0.143599552126838,0.142,0.045,0.000157442401392168,1.25742242344441
"Rbbp7",8.8228812525511e-09,0.24964627031425,0.458,0.253,0.000158229552383251,2.18477027652403
"U2surp",8.86252347309741e-09,0.221369561690669,0.439,0.237,0.000158940495966529,1.93631527592631
"Ndufb8",8.86394432599505e-09,-0.208525797665324,0.8,0.856,0.000158965977542395,-1.82393769184554
"Xrn1",8.87199701771928e-09,0.208397109427262,0.226,0.092,0.000159110394515778,1.82262284085956
"Fstl1",8.87509449651993e-09,0.121141661640141,0.097,0.024,0.000159165944700588,1.05945206076884
"Acer3",8.878020544145e-09,0.180728824208597,0.226,0.092,0.000159218420438696,1.58051578324192
"Zfos1",8.99606047551561e-09,0.32139391988733,0.348,0.179,0.000161335348567897,2.80641988756839
"Rbm3",9.02783092855934e-09,0.263944695117446,0.594,0.353,0.000161905119872783,2.30384129089276
"Pdlim1",9.11101530647521e-09,0.115916066656463,0.058,0.01,0.000163396948506326,1.01071021152505
"Prodh2",9.14789458081101e-09,-0.45208219332527,0.019,0.212,0.000164058341412265,-3.9400267193317
"Slk",9.17008992124379e-09,0.238460930279554,0.277,0.127,0.000164456392647586,2.07767792029149
"Arl15",9.19456763688075e-09,0.185600684972622,0.194,0.072,0.000164895375999819,1.61661896932568
"Ubl3",9.35102876382517e-09,0.288193968505884,0.523,0.314,0.000167701349850441,2.50536407333567
"Gldc",9.6656947973929e-09,-0.468804615840766,0.006,0.187,0.000173344570496444,-4.05995542665459
"Ccdc148",9.68398159748065e-09,0.212261236414557,0.187,0.07,0.000173672525969218,1.83782975558611
"Rpl30",9.71817241450653e-09,0.300417222059429,0.89,0.691,0.00017428570408176,2.60005536793487
"Gm26632",9.71978794077845e-09,0.237330352455192,0.11,0.03,0.000174314676929921,2.05401075609528
"Amacr",9.78011010129975e-09,-0.58571190572838,0.077,0.276,0.00017539649455671,-5.06550684549257
"Wasf2",9.84062911123225e-09,0.247064163687814,0.348,0.173,0.000176481842480839,2.13520078426134
"Sdhc",9.84662989591185e-09,-0.524207980539643,0.432,0.575,0.000176589460553283,-4.53003916838662

"Ssbp4",1.02016998765159e-08,0.249835928752777,0.31,0.147,0.000182957285585436,2.15015242165882
"Tap2",1.03076232348461e-08,0.187121357406505,0.194,0.073,0.000184856915093731,1.60848180236497
"Maff",1.03619175573072e-08,0.131752052657405,0.097,0.024,0.000185830629472747,1.13183905090819
"Raly",1.05619116266008e-08,0.273098898123991,0.445,0.251,0.000189417323111459,2.34088302269182
"Fitm1",1.06038033411269e-08,-0.489062365809244,0.013,0.196,0.00019016860911977,-4.19009046263438
"Lrpap1",1.06860639233051e-08,-0.612720640136776,0.4,0.55,0.000191643870400554,-5.24481009811758
"Tfcp2l1",1.07634002199295e-08,0.179402177373968,0.6,0.339,0.000193030819544216,1.53436595103279
"Slc39a6",1.09203036818615e-08,0.122525865210622,0.103,0.027,0.000195844726230503,1.0461489242037
"Il18r1",1.09328114605374e-08,0.101195430596306,0.084,0.019,0.000196069040733278,0.863909814467424
"Scai",1.10911578270804e-08,0.213147314214883,0.168,0.059,0.000198908824470859,1.81658294298805
"Ppp2r2d",1.11393109164024e-08,0.247685244892682,0.265,0.118,0.000199772401974761,2.10986510527795
"Pdcd6ip",1.11577321004489e-08,0.275760547932159,0.452,0.254,0.00020010276748945,2.34856420160491
"Ostf1",1.11677121765631e-08,0.3024980037605,0.568,0.342,0.000200281750174483,2.57600809360322
"Abcc2",1.12065965042951e-08,-0.430845892037916,0.006,0.187,0.000200979101708029,-3.66749363451004
"Rras",1.13259604549557e-08,0.197191216392281,0.194,0.074,0.000203119774799176,1.67646346888625
"Vps4b",1.13628096246804e-08,0.213942377042708,0.348,0.173,0.000203780627809018,1.81818212350154
"Smarce1",1.15665356147608e-08,0.221642520565213,0.316,0.152,0.00020743424971512,1.87968286802989
"Gbp3",1.15737525903504e-08,0.106018294786216,0.084,0.019,0.000207563678955344,0.899042813268498
"Lrrc31",1.17091854080107e-08,0.218060933290554,0.135,0.042,0.000209992531107264,1.84663562332906
"Pdcd2l",1.19894298849233e-08,0.150143306440909,0.181,0.065,0.000215018435556215,1.26792821034958
"Frzb",1.21985916516119e-08,0.245441228164621,0.058,0.01,0.000218769542680007,2.06845391288864
"Stag1",1.22924203183462e-08,0.289664262736465,0.265,0.121,0.000220452265989221,2.43892366604209
"Dpp9",1.24402920454098e-08,0.208263297154081,0.174,0.062,0.00022310419754238,1.75105107022785
"Anxa11",1.24954733146674e-08,0.307209290482428,0.368,0.19,0.000224093818425245,2.58161661001009
"Acox1",1.25193216020623e-08,-0.575021964868132,0.265,0.461,0.000224521513611386,-4.83106947885918

"Ddx58",1.25989343373796e-08,0.20220511484168,0.194,0.074,0.0002259492
88406566,1.69755237434636
"Supt20",1.27136650416324e-08,0.262288277253949,0.245,0.108,0.00022800
6868856636,2.19958485027188
"Rab3d",1.31221786927794e-08,0.13314039219125,0.116,0.033,0.0002353331
52676306,1.11232252185274
"Pard3",1.34888807559826e-08,0.163705950681888,0.232,0.096,0.000241909
587477792,1.36317069421862
"Snx2",1.36304881217707e-08,0.242899882476155,0.374,0.194,0.0002444491
73975835,2.02007763727593
"Cdk12",1.37201476762749e-08,0.308364289989213,0.329,0.167,0.000246057
128426314,2.56249085087954
"Hip1",1.38684537054317e-08,0.116183310048304,0.097,0.024,0.0002487168
48753211,0.96422799953055
"Inip",1.38979205413657e-08,0.18599954456172,0.135,0.043,0.00024924530
6988852,1.54325179507169
"Rpl27a",1.40791808326299e-08,0.291201393422738,0.981,0.882,0.00025249
6029052385,2.41234584172767
"Rps10",1.41783016918112e-08,0.336528287316433,0.91,0.765,0.0002542736
62540942,2.7854781050057
"Tmem72",1.42893498751532e-08,0.209498017789737,0.316,0.147,0.00025626
5200660998,1.73240147867825
"Efnb2",1.4758769837494e-08,0.223763127414084,0.123,0.036,0.0002646837
78265618,1.84313122692823
"Ccdc112",1.48708248122876e-08,0.14592968071661,0.135,0.042,0.00026669
3372183566,1.20091531654767
"Hs3st1",1.48797182876121e-08,0.114118413795806,0.065,0.012,0.00026685
2867770036,0.93905909936334
"Fam220a",1.49824142938298e-08,0.150006186704105,0.206,0.081,0.0002686
94617945543,1.23334112755732
"Tpm3-
rs7",1.50364058724491e-08,0.113209942162151,0.084,0.019,0.000269662902
916502,0.930397556824315
"Lsm12",1.50577176965809e-08,0.227426294348733,0.271,0.123,0.000270045
109170482,1.8687440172329
"Sfswap",1.53274738082892e-08,0.185500950335448,0.232,0.097,0.00027488
2915277859,1.5209529576277
"Ctgf",1.54855513862218e-08,0.139380743363507,0.084,0.019,0.0002777178
78560501,1.14137563624947
"Eci3",1.55470991111731e-08,-0.551656517202507,0.026,0.21,0.0002788216
75459779,-4.51527446592625
"Romo1",1.56033816864274e-08,-0.35676077738201,0.774,0.822,0.000279831
047164389,-2.91877570286931
"Rbm28",1.56541402676754e-08,0.241020324423072,0.277,0.128,0.000280741
351560491,1.97108271616371
"F2r",1.57144955794504e-08,0.169410199754629,0.077,0.017,0.00028182376
3721863,1.3847977056803
"Usp40",1.57845261668744e-08,0.193299240814283,0.232,0.098,0.000283079
692276726,1.57921267789826
"Srsf7",1.58439107048685e-08,0.286256089823599,0.361,0.19,0.0002841446

94581111,2.33757494741779
"Tmed9",1.62986785680526e-08,0.272911620989127,0.516,0.303,0.000292300501439455,2.22088058613982
"Rnf20",1.63109336941786e-08,0.201724315966923,0.252,0.109,0.0002925202848714,1.64142602731865
"Slc7a8",1.65567812274619e-08,-0.548168374121334,0.103,0.301,0.000296929314533302,-4.45223254962173
"Acvr2b",1.71791309228631e-08,0.107377873778474,0.116,0.033,0.000308090533970626,0.868162659472646
"Tspan1",1.73342432180255e-08,0.2475625329639,0.226,0.094,0.00031087231787207,1.99934677441733
"Gcdh",1.73990204983308e-08,-0.547665470948632,0.142,0.343,0.000312034033617065,-4.42097381336726
"Rps3",1.80125203000273e-08,0.302668212067529,0.948,0.856,0.00032303653906069,2.43276994365102
"Cldn16",1.82313968341e-08,0.204786178994832,0.174,0.062,0.00032696187082275,1.6435456781707
"Akap13",1.83990124279377e-08,0.279832778762695,0.316,0.155,0.000329967888882635,2.24328372851021
"Prkcq",1.85036405760395e-08,0.0734781759225991,0.071,0.014,0.000331844290090692,0.588622256509114
"Slx4ip",1.85569295925792e-08,0.120040127370294,0.103,0.027,0.000332799975313315,0.9612776095178
"Rassf1",1.8974329739691e-08,0.173372181464895,0.213,0.085,0.000340285629551618,1.38450259956302
"Zbtb12",1.98106480082792e-08,0.117641145289811,0.084,0.019,0.00035528416138048,0.93437569404463
"Raf1",1.99099775273859e-08,0.280365281168927,0.316,0.158,0.000357065536976139,2.22542499309715
"Oser1",2.01260670877943e-08,0.192560380777149,0.2,0.079,0.000360940887152503,1.5263869255284
"Fbxw2",2.01464092702803e-08,0.202117401471585,0.303,0.143,0.000361305703853207,1.60193929780275
"Nfic",2.0196300529811e-08,0.28402859014867,0.419,0.231,0.00036220045370163,2.25044735249806
"Slc37a4",2.03090984861949e-08,-0.572795017788624,0.103,0.298,0.000364223372251419,-4.53524386769132
"Ube3a",2.06330595147652e-08,0.210664939539152,0.542,0.308,0.000370033289337799,1.6646569903274
"Heatr6",2.06350242468753e-08,0.207004917602076,0.155,0.054,0.000370068524843461,1.63571608802728
"Zc2hc1a",2.09892872058663e-08,0.239060021143628,0.206,0.084,0.000376421876750007,1.88494046210671
"Wbp11",2.13724561797892e-08,0.228733001251029,0.335,0.17,0.00038329362912834,1.7993760068465
"Nsa2",2.17868219800288e-08,0.247101449679906,0.49,0.284,0.000390724865389836,1.93913033500779
"0610040J01Rik",2.18741031809923e-08,0.211826828565566,0.348,0.176,0.000392290166447916,1.66146558788701
"Tmem173",2.19230887224459e-08,0.183693407396245,0.135,0.044,0.0003931

68673148346,1.44038994342003
"Rin2",2.22946042768834e-08,0.204478654371705,0.232,0.097,0.0003998314
33101626,1.59993658903562
"Rps15a",2.24860421055969e-08,0.282910020591581,0.942,0.847,0.00040326
4679121775,2.2112013637747
"Pum1",2.26644400175668e-08,0.254652641513248,0.374,0.198,0.0004064640
67275044,1.98833165192431
"Acmsd",2.29349359709931e-08,-0.445087857912749,0.006,0.179,0.00041131
514170379,-3.46997209031405
"Bax",2.32102048469828e-08,0.255699679068877,0.542,0.323,0.00041625181
372579,1.99042259655493
"Shtn1",2.3238902657524e-08,0.193566784623291,0.181,0.068,0.0004167664
80260036,1.50652728296682
"Tmem50a",2.3597956787618e-08,0.235212107319488,0.445,0.246,0.00042320
575702914,1.82704581323759
"Bcl9",2.43671774965239e-08,0.1656273295597,0.161,0.057,0.000437000961
22266,1.28122265690189
"Adh1",2.56699671718091e-08,-0.577532147178878,0.084,0.278,0.000460365
191259224,-4.43746276017544
"Kpnb1",2.75185681270686e-08,0.231154687454254,0.303,0.148,0.000493518
000790848,1.76000051547318
"Gpc3",2.86786417439513e-08,0.412763670603522,0.174,0.064,0.0005143227
61036022,3.12571875201533
"Zfp553",2.88109902162408e-08,0.12699315966603,0.129,0.04,0.0005166962
98538062,0.961091253341358
"Spty2d1",2.94244607407045e-08,0.222598085031051,0.213,0.089,0.0005276
98278923794,1.67994460442737
"Oasl2",2.96768979350824e-08,0.179165485298311,0.11,0.031,0.0005322254
87567768,1.35062885396001
"Rbm33",2.98193487859284e-08,0.184885697120952,0.187,0.072,0.000534780
20112684,1.39286500745678
"Psma7",2.98329540246518e-08,0.281568666247832,0.768,0.522,0.000535024
197478106,2.12111267764042
"Them7",3.00318759337213e-08,-0.475761394326114,0.032,0.215,0.00053859
1662995357,-3.58084328158938
"Car3",3.03935791684081e-08,-0.795770061002817,0.026,0.204,0.000545078
448806232,-5.97987844621276
"Pan3",3.0746207476301e-08,0.251059300112636,0.29,0.14,0.0005514024848
79981,1.88370936522234
"Atl3",3.10978431430242e-08,0.172137573721202,0.187,0.071,0.0005577087
18926996,1.28959854088117
"Fkbp3",3.14212461483385e-08,0.158625988443826,0.581,0.333,0.000563508
628424302,1.18673303497721
"Pdap1",3.26099770853041e-08,0.257292880791665,0.529,0.318,0.000584827
329047844,1.91533809841703
"Stxbp1",3.3842545433278e-08,0.0970937150221487,0.065,0.013,0.00060693
2209800408,0.719182220952063
"Spi1",3.42082485089786e-08,0.162049306655778,0.181,0.067,0.0006134907
28760023,1.19857264482459
"Hibadh",3.42968513458491e-08,-0.528175729505646,0.439,0.592,0.0006150

79732036459,-3.90520387032284
"Sh3glb1",3.43873848658009e-08,0.26992091231082,0.51,0.295,0.000616703
360183273,1.99501850858597
"Clasp1",3.44249864858157e-08,0.191857243884621,0.194,0.077,0.00061737
7707636619,1.41783070233905
"Dusp3",3.44861730475653e-08,0.264435920779372,0.329,0.166,0.000618475
027435037,1.95371968147163
"Cast",3.47261776333473e-08,0.215395966477611,0.2,0.081,0.000622779269
67645,1.58990621156061
"Gm10116",3.51115127358167e-08,-0.49708218157351,0.11,0.304,0.00062968
9869404136,-3.66363641735858
"Igsf3",3.51241992670005e-08,0.134338696608719,0.148,0.05,0.0006299173
89654388,0.990065698756544
"Ccdc30",3.55814527548894e-08,0.102440172058001,0.071,0.015,0.00063811
7773706187,0.753651085137428
"Cdh3",3.63506941838662e-08,0.187670174151298,0.148,0.051,0.0006519133
49493456,1.37667312389647
"St3gal1",3.66669547301541e-08,0.125117631476027,0.084,0.02,0.00065758
5166130583,0.916728912336443
"Itpr3",3.68641666959625e-08,0.1664535477915,0.116,0.034,0.00066112196
5525391,1.21870167117332
"Slc25a15",3.70532769739188e-08,-0.43789273222099,0.039,0.223,0.000664
51346925026,-3.20382264977369
"Pros1",3.72736110972242e-08,0.201207039098023,0.226,0.094,0.000668464
941417618,1.47092941273133
"Kif2a",3.73781675992754e-08,0.20014600498653,0.187,0.073,0.0006703400
57725404,1.46261204945126
"Cryzl1",3.78684281565475e-08,0.233099705443986,0.29,0.139,0.000679132
390559523,1.70039113241908
"Znrd1",3.79499395774782e-08,0.188415735991342,0.271,0.125,0.000680594
216382494,1.37403010035738
"Zfp579",3.8964173199945e-08,0.120161320513998,0.155,0.053,0.000698783
482167814,0.873112536306459
"2410002F23Rik",3.92218426553224e-08,0.174653351605672,0.174,0.064,0.0
00703404526180552,1.26790969936779
"Parp2",3.92857888705303e-08,0.163200718430503,0.155,0.054,0.000704551
33760409,1.18450254960715
"Cactin",3.95405195116293e-08,0.144294970243112,0.129,0.04,0.000709119
676921561,1.04635299250196
"Maml3",3.96460563550105e-08,0.170290524870971,0.135,0.044,0.000711012
374670758,1.23440548573653
"Apc",4.0032250740557e-08,0.215229502041192,0.271,0.127,0.000717938384
781149,1.55807365802709
"Antxr1",4.01662932393123e-08,0.0986715336709874,0.052,0.009,0.0007203
42302953826,0.713965908357457
"Ankrd13a",4.02806605008234e-08,0.173932310945141,0.226,0.096,0.000722
393365421766,1.25804209783434
"Myo1e",4.07345973177747e-08,0.200084059741163,0.187,0.073,0.000730534
268296972,1.44495394055223
"Aco2",4.09411490129982e-08,-0.452187725240012,0.439,0.599,0.000734238

56639911,-3.2632925573761
"Capsl",4.09608695009094e-08,0.0623153320775049,0.052,0.009,0.00073459
2233629309,0.44967958756654
"Faim",4.1817365884602e-08,0.207075764803745,0.219,0.093,0.00074995263
9774453,1.49001376926506
"Slc2a1",4.18708925993258e-08,0.319196952020387,0.226,0.098,0.00075091
258787631,2.29637351344401
"Rps24",4.19150727686833e-08,0.287310694333069,0.942,0.86,0.0007517049
15033567,2.06667372226128
"Paqr9",4.19227404847244e-08,-0.405878892884921,0.013,0.185,0.00075184
2427853047,-2.91948029860434
"Col4a2",4.27178528104761e-08,0.354637052913272,0.284,0.138,0.00076610
1972303078,2.54423546898601
"Pcolce",4.39522210163811e-08,0.0988872021636433,0.361,0.175,0.0007882
39131707779,0.706619175183582
"Klf10",4.4124283447971e-08,0.128586905567024,0.148,0.051,0.0007913248
99355913,0.91834221028087
"Ldhd",4.42046161223017e-08,-0.575601994562742,0.135,0.322,0.000792765
585537358,-4.10978844710731
"Lage3",4.47501267122375e-08,0.214780973039706,0.419,0.232,0.000802548
772457268,1.53089819237737
"Hnrnpul2",4.50332621294234e-08,0.232143096712008,0.452,0.257,0.000807
626523029079,1.65318636309859
"Arid4a",4.74526640217466e-08,0.241979147072359,0.206,0.086,0.00085101
6076566004,1.71056983723726
"Fam98a",4.75461973406004e-08,0.173128822740503,0.194,0.076,0.00085269
3503106327,1.22352050224847
"Rgl3",4.79094214518167e-08,0.240848432767681,0.194,0.078,0.0008592075
6431688,1.70026951843113
"Ate1",4.7952067693839e-08,0.205008210071384,0.245,0.109,0.00085997238
2021309,1.44707305974232
"Blnk",4.82887907398805e-08,0.24841037613693,0.226,0.098,0.00086601117
3129017,1.75169377497559
"Zfp938",4.83637470595967e-08,0.158431971972781,0.232,0.099,0.00086735
5439766807,1.11695517767147
"Arhgef2",4.92693901890154e-08,0.233414664175983,0.297,0.147,0.0008835
97243649802,1.64125735994032
"Gcsh",5.13234147383925e-08,-0.512339196956354,0.148,0.342,0.000920434
119918331,-3.58159175876185
"Eps8l1",5.15764508425239e-08,0.165166444379352,0.11,0.032,0.000924972
069409824,1.15381099598957
"Ofd1",5.27770741407363e-08,0.093182625592267,0.097,0.026,0.0009465040
47639965,0.648805957573198
"Tmem8",5.31840587679176e-08,-0.406696621298496,0.052,0.238,0.00095380
2909943834,-2.82859675989222
"Ppp1r9a",5.36141858303842e-08,0.205325536387778,0.194,0.078,0.0009615
1680868211,1.42639619563
"Cic",5.4207538782736e-08,0.190362868284784,0.187,0.073,0.000972158000
529588,1.32035537239671
"Thra",5.48397364594284e-08,0.312497084984873,0.265,0.125,0.0009834958

3366339,2.16385392664586
"Ccdc174",5.54062147915581e-08,0.220183495642967,0.226,0.098,0.0009936
55056071802,1.52237520725163
"Sdc1",5.57048976445652e-08,0.303726026470861,0.161,0.059,0.0009990116
34357633,2.09836540353535
"Nrbp1",5.57083476995483e-08,0.171919940490523,0.245,0.108,0.000999073
507643698,1.18774023282909
"Ahctf1",5.60893683234942e-08,0.164099656427915,0.213,0.088,0.00100590
673151355,1.13259382680971
"Cdc42se2",5.64053996839697e-08,0.215994635665316,0.239,0.106,0.001011
57443793231,1.48955242570745
"Pcf11",5.64706301230086e-08,0.167979713737376,0.239,0.105,0.001012744
28062604,1.15823550027147
"Unc93b1",5.64962103071495e-08,0.216120742743299,0.239,0.106,0.0010132
0303564842,1.49007442463203
"Esf1",5.66887209870801e-08,0.203231015633551,0.31,0.152,0.00101665552
21823,1.4005130820598
"Ache",5.73829384611131e-08,0.0762911436412511,0.065,0.013,0.001029105
6183616,0.524811750195702
"Trim12c",5.73958385230596e-08,0.16724865087295,0.161,0.059,0.00102933
696807255,1.15047677719543
"Cdh16",5.74481507966829e-08,0.334169053040509,0.819,0.647,0.001030275
13638771,2.2983911510372
"Asxl2",5.76630413439313e-08,0.242272818409512,0.303,0.15,0.0010341289
8346206,1.66543078312093
"Ewsr1",5.79278323600732e-08,0.221331454538434,0.523,0.311,0.001038877
74554555,1.52046171169958
"Hist1h2bc",5.85971772299348e-08,-0.450938501859431,0.606,0.713,0.0010
5088177644165,-3.09259291977022
"Senp7",5.89766838432439e-08,0.199922670857336,0.187,0.074,0.001057687
84804474,1.36980417197767
"Ptger4",5.92203000881209e-08,0.104961875916299,0.09,0.023,0.001062056
86178036,0.718731464134051
"Bloc1s5",5.99427607959281e-08,0.146713567089401,0.142,0.048,0.0010750
1347211418,1.00284915669336
"D630023F18Rik",5.99432410801934e-08,-0.442778978251196,0,0.161,0.0010
7502208553219,-3.02657765913687
"Tuft1",6.01583936662818e-08,0.14273577989719,0.161,0.058,0.0010788806
320111,0.975146758336829
"Adamts9",6.03487612265204e-08,0.272258543366252,0.129,0.042,0.0010822
9468383642,1.85916423319825
"Mysm1",6.06700165842031e-08,0.235133990584768,0.226,0.098,0.001088056
0774211,1.60440447502392
"Spen",6.07484711452325e-08,0.214224248438465,0.226,0.098,0.0010894630
815186,1.46145288064051
"Ccdc136",6.15793446765782e-08,0.118611226029514,0.084,0.02,0.00110436
396742975,0.807562836852774
"Ugt3a1",6.17100218664281e-08,-0.465455698254054,0.006,0.17,0.00110670
753215252,-3.16806177336159
"Rnmt",6.20639338944082e-08,0.154454711513417,0.181,0.069,0.0011130545

9046232,1.05039199518027
"Rint1",6.21495093149212e-08,0.170091830142391,0.213,0.088,0.001114589
3000538,1.15650015592251
"Arpc2",6.24113823204606e-08,0.312498058056795,0.684,0.451,0.001119285
73053514,2.12344447240553
"Lrrfip2",6.36358384984825e-08,0.238288969688143,0.252,0.115,0.0011412
4512763179,1.61455917735003
"Asl",6.43697657281214e-08,-0.553131831449768,0.194,0.384,0.0011544073
7856813,-3.74147672193006
"Tmem109",6.46762450981152e-08,0.189642933892572,0.245,0.11,0.00115990
37795896,1.28187590377304
"Plscr1",6.4734050307493e-08,0.225905336360494,0.335,0.171,0.001160940
45821458,1.52678683236286
"Dgka",6.48093655866274e-08,0.107926632868254,0.097,0.026,0.0011622911
6243058,0.729299336566971
"Mgat4b",6.52440632316713e-08,0.201460453714976,0.342,0.174,0.00117008
702999679,1.35999448118512
"Ifit2",6.59232577125612e-08,0.126805013941899,0.071,0.015,0.001182267
70381707,0.85470648593463
"Plod3",6.63216777635307e-08,0.147327474218758,0.155,0.055,0.001189412
96901116,0.992146731471938
"Id4",6.65224839419593e-08,0.173296825043233,0.148,0.051,0.00119301422
70151,1.16650810258601
"Nsmf",6.66483897496121e-08,0.141027864231693,0.155,0.055,0.0011952722
2176954,0.949030275004741
"Alkbh1",6.67869545073605e-08,0.179167156245668,0.174,0.066,0.00119775
7242135,1.20531200495573
"Myo1c",6.69426102682234e-08,0.215974094575676,0.277,0.132,0.001200548
77255032,1.45242071414284
"Ehmt2",6.72712175361263e-08,0.191168427516368,0.284,0.136,0.001206442
01529289,1.28466707559398
"Cd72",6.76197611490728e-08,0.154326296140025,0.148,0.052,0.0012126927
9644747,1.03628748867046
"Cfl1",6.77639586220947e-08,0.288498195871626,0.794,0.587,0.0012152788
3392865,1.93662541999302
"Rap2b",6.78611544463432e-08,0.166207661567522,0.116,0.035,0.001217021
94384072,1.11547752918761
"Etf1",6.80532640043322e-08,0.223771866206322,0.361,0.194,0.0012204672
3665369,1.50117837838029
"Ttc12",6.80663578828003e-08,0.12378450556532,0.097,0.026,0.0012207020
6227014,0.830387203891957
"Smarcc1",6.84325075104993e-08,0.266709827951273,0.252,0.116,0.0012272
685896933,1.78774643860126
"Smc1a",6.94829666677412e-08,0.265810301771066,0.277,0.134,0.001246107
52421927,1.77766768012975
"Gpsm3",6.98420153015882e-08,0.159786916785759,0.123,0.038,0.001252546
70241868,1.06778828526754
"Anp32a",6.98783119071035e-08,0.250120632478366,0.652,0.42,0.001253197
64574199,1.67132029245301
"Rsf1",7.00143334828982e-08,0.246227172520563,0.265,0.125,0.0012556370

566823,1.64482514412575
"Rabac1",7.03177702610519e-08,0.291003743916421,0.748,0.516,0.00126107
88918617,1.9426792029481
"Rac1",7.05061081512471e-08,0.25530238128313,0.606,0.402,0.00126445654
358446,1.70366160345489
"Utp3",7.10248445464306e-08,0.227595904027084,0.265,0.124,0.0012737595
6209569,1.5171047864911
"Dbn1",7.12157423938431e-08,0.164980469554654,0.161,0.059,0.0012771831
2409118,1.09928108794169
"Abat",7.12698480249859e-08,-0.416660800664905,0.032,0.208,0.001278153
4544801,-2.77593544216978
"Snrpd1",7.14524461076346e-08,0.277557928524518,0.439,0.251,0.00128142
816849432,1.84847475966115
"Frmd4a",7.2751319565735e-08,0.117650646045147,0.11,0.032,0.0013047221
6509189,0.781407962093763
"Rnps1",7.49126703922697e-08,0.213488232956585,0.252,0.116,0.001343483
83081497,1.41168862730222
"Bst2",7.55819309191699e-08,0.30179229292418,0.297,0.147,0.00135548634
910439,1.99291406446718
"Zfp182",7.59047187787714e-08,0.136184736490741,0.097,0.026,0.00136127
522657849,0.898728473552193
"Rps19",7.62355835725212e-08,0.284577211127886,0.994,0.947,0.001367208
9557896,1.87678211878687
"Slc6a20b",7.6653116286092e-08,-0.385681110028822,0.013,0.181,0.001374
69698747477,-2.54145413826633
"Pon2",7.66590181733605e-08,0.206706882446779,0.181,0.07,0.00137480283
192105,1.36208362331248
"Safb",7.78367703515861e-08,0.260861633730396,0.31,0.157,0.00139592463
948534,1.71495609838221
"Nfx1",7.81074575303725e-08,0.212276053931178,0.265,0.124,0.0014007791
433497,1.39480792816189
"Smarcb1",7.8412122435153e-08,0.192246241479612,0.252,0.114,0.00140624
300375203,1.26244909092821
"Spin1",7.86158450884492e-08,0.235996289309363,0.335,0.175,0.001409896
56581625,1.549136030767
"Folh1",7.88836492294627e-08,-0.382927004002328,0.013,0.181,0.00141469
936528118,-2.51232212836756
"Atox1",7.89992993888952e-08,-0.363541716886637,0.877,0.851,0.00141677
343524045,-2.38460580264497
"Taf1d",7.90963307527115e-08,0.198243418425263,0.277,0.131,0.001418513
59571913,1.30010922590257
"Miip",7.91499109141715e-08,0.127804884397775,0.129,0.041,0.0014194745
0233475,0.838076509289715
"Tspan9",8.02638104033282e-08,0.172045420902151,0.219,0.094,0.00143945
117577329,1.12577807031997
"Hspa4",8.04588803495146e-08,0.218498978900611,0.432,0.24,0.0014429495
6018819,1.4292162320073
"Kansl1",8.08418264048679e-08,0.193580862891225,0.219,0.094,0.00144981
73147449,1.26530602443039
"Trim44",8.08675363222311e-08,0.249970243874153,0.387,0.213,0.00145027

839640289,1.633805449834
"Hoxc9",8.15225277087809e-08,0.220506288752313,0.2,0.082,0.00146202501
192928,1.43945023710863
"Nsf",8.16077508545524e-08,0.167517552275622,0.226,0.097,0.00146355340
382554,1.09336829537357
"Ing2",8.18545560153403e-08,0.235442082521938,0.245,0.109,0.0014679796
0757911,1.53599312456699
"Tcaf1",8.24834358974753e-08,0.206957917280243,0.174,0.068,0.001479257
93938532,1.34858222477778
"Tinagl1",8.28347790155772e-08,0.43714529765091,0.245,0.109,0.00148555
892686536,2.84667452667109
"Zak",8.30338727454225e-08,0.178221936212546,0.142,0.049,0.00148912947
381641,1.16014702432848
"Ccl9",8.36490863517084e-08,0.179483625470192,0.2,0.082,0.001500162714
63154,1.16703514504226
"Bbx",8.56211396191434e-08,0.27804395964692,0.342,0.18,0.0015355295179
2972,1.80141344777811
"Ice1",8.57101638340489e-08,0.189262516316111,0.161,0.06,0.00153712607
819983,1.22601244802746
"Zfp710",8.63460720870115e-08,0.118333231009609,0.129,0.041,0.00154853
045680846,0.765669123254753
"Tpi1",8.87581705208358e-08,-0.382110140853858,0.671,0.743,0.001591789
03012067,-2.46189617284589
"Zfp106",8.93956550305997e-08,0.259854367754081,0.406,0.234,0.00160322
167731877,1.67235518144102
"Gigyf2",9.0385253887869e-08,0.205746374087273,0.368,0.194,0.001620969
14322504,1.3218651225604
"Eif3f",9.25253408150856e-08,0.342252596480674,0.652,0.452,0.001659349
46217775,2.1908716914772
"Scnn1g",9.30831764057477e-08,0.327778353865603,0.142,0.048,0.00166935
368566068,2.09624704971085
"Nme1",9.39989696625119e-08,-0.448886931437175,0.639,0.701,0.001685777
52192749,-2.86638024201617
"Ptprc",9.40974926777118e-08,0.154062682765306,0.155,0.055,0.001687544
43368208,0.983610240803852
"Chd2",9.42447688757164e-08,0.234409210205846,0.258,0.122,0.0016901856
850171,1.49621450542193
"Zbtb4",9.56726980071976e-08,0.206001083310052,0.206,0.087,0.001715794
16606108,1.31179002078909
"Kras",9.6058010619302e-08,0.302029382841004,0.316,0.167,0.00172270436
244656,1.9220726838778
"Dhps",9.78572616854708e-08,0.158470963154781,0.181,0.07,0.00175497213
106723,1.00554616729417
"Zfp157",1.00117356812189e-07,0.155336043668614,0.168,0.063,0.00179550
46770698,0.982107342333973
"Arl6ip5",1.00337438475691e-07,0.230371942845429,0.381,0.211,0.0017994
5162162305,1.45601364292535
"Rpl13a",1.00454472142679e-07,0.272477410118505,0.981,0.928,0.00180155
05034068,1.7218140714958
"Samd4b",1.02140808351607e-07,0.192983161606372,0.206,0.087,0.00183179

325697772,1.21626863162784
"Dhx9",1.02530253855889e-07,0.245518363198534,0.342,0.183,0.0018387775
7265152,1.5464352918398
"Rps16",1.04655627130009e-07,0.286635798931123,0.974,0.893,0.001876894
01694959,1.79953881107842
"Haao",1.06982647705164e-07,-0.403045236292142,0.006,0.165,0.001918626
80394442,-2.52150966353469
"Akirin2",1.08289178028865e-07,0.217527379068384,0.329,0.172,0.0019420
5811876967,1.35824247374114
"Ak4",1.09220083418006e-07,-0.405212619567467,0,0.156,0.00195875297601
852,-2.52668191230071
"Ephx1",1.09478759671584e-07,0.121390935120764,0.103,0.029,0.001963392
07595019,0.756639610249869
"Hjurp",1.12081989841029e-07,0.165686029571056,0.168,0.063,0.002010078
40580901,1.02884091228517
"Susd4",1.12514923495263e-07,0.115364955390967,0.077,0.018,0.002017842
63796405,0.715923342301479
"Ltbp4",1.12979846081336e-07,0.119635438852997,0.058,0.011,0.002026180
55962268,0.741931467293024
"Atp8a1",1.13113154761858e-07,0.197148118169878,0.155,0.056,0.00202857
131749917,1.22240182846626
"Sub1",1.13160309145479e-07,0.267315926556196,0.794,0.54,0.00202941698
421502,1.65736054290198
"Hsd3b2",1.13759405897141e-07,-0.395241909929114,0.006,0.167,0.0020401
6118535932,-2.44841551825669
"Pigp",1.14433891359688e-07,0.223766632470163,0.432,0.247,0.0020522574
0764464,1.38485027300718
"Arfgap2",1.15025592894681e-07,0.163983843085412,0.232,0.102,0.0020628
689829732,1.01401993018005
"Ubr2",1.15869347038152e-07,0.204049136557531,0.252,0.117,0.0020780008
6978221,1.26027867394498
"Rps14",1.16153809217251e-07,0.274060304008708,0.987,0.927,0.002083102
41450218,1.69202007533257
"Pibf1",1.17781986971654e-07,0.108318212273183,0.097,0.026,0.002112302
15434964,0.667237680888068
"Dnajc17",1.17876747374697e-07,0.115472576716016,0.129,0.042,0.0021140
0158741782,0.711215535252011
"Ptbp2",1.17969991349374e-07,0.151156834380918,0.168,0.063,0.002115673
82485967,0.930881516526856
"Snw1",1.18112723475826e-07,0.215060180156852,0.323,0.164,0.0021182335
8281546,1.32416268157476
"Fbxo11",1.20188155007856e-07,0.20159504156197,0.194,0.079,0.002155454
37191089,1.23774390882653
"Rbm14",1.21117222037682e-07,0.190040774597128,0.148,0.053,0.002172116
26002379,1.16534016881439
"Tbpl1",1.2181109357056e-07,0.168921409548039,0.168,0.063,0.0021845601
5209443,1.03487011893059
"Acsm1",1.22539096266065e-07,-0.453128810313506,0.026,0.192,0.00219761
615243561,-2.77332144748227
"Atp1b1",1.23667754893137e-07,0.391903798051227,0.91,0.885,0.002217857

51625353,2.39500783302792
"Psma2",1.26702296762104e-07,0.222993277823371,0.684,0.449,0.002272278
99013157,1.35735383622739
"Sult2b1",1.27424741155764e-07,0.12058162490382,0.058,0.011,0.00228523
530788747,0.733291381214772
"Clmn",1.27621365959339e-07,0.265848349048962,0.432,0.249,0.0022887615
7711479,1.61629001236827
"Eif2s2",1.27737568281243e-07,0.239955346617476,0.71,0.47,0.0022908455
4955582,1.45864879003564
"B4galnt1",1.2954875514011e-07,0.187747754313953,0.174,0.069,0.0023233
2737468274,1.13864411436268
"Casp6",1.33202088504757e-07,0.190742883128344,0.239,0.108,0.002388846
25524431,1.15150425033221
"Ncbp3",1.34805205205053e-07,0.184747511098588,0.174,0.068,0.002417596
55014742,1.11310031684982
"F11r",1.35858306146683e-07,0.272041830151952,0.413,0.24,0.00243648286
243461,1.6369300267723
"Rab14",1.35929057028992e-07,0.223426121908962,0.658,0.423,0.002437751
70875794,1.34428327703188
"Mgst1",1.37013654887846e-07,0.389049691185281,0.606,0.402,0.002457202
88675863,2.33769517878703
"Ccdc12",1.37861643211779e-07,0.277152383820665,0.51,0.32,0.0024724107
0936004,1.66362425886056
"Azin2",1.38536244225379e-07,0.132220670033342,0.097,0.027,0.002484509
00393796,0.793017297715475
"Gpr107",1.40383663585523e-07,0.17530072062761,0.239,0.108,0.002517640
62274277,1.04907543091795
"Sema3f",1.40885699758758e-07,0.138929974399773,0.071,0.016,0.00252664
413947356,0.83092118290845
"Esyt2",1.41098700896142e-07,0.254063910023711,0.213,0.094,0.002530464
10187141,1.51913768917554
"Rrm1",1.41366223406187e-07,0.152834648381112,0.116,0.036,0.0025352618
5056657,0.913562745618619
"Nucks1",1.42191297544061e-07,0.201736207578033,0.535,0.317,0.00255005
873015519,1.20469578242046
"Tjp2",1.4251077363591e-07,0.177856765278036,0.194,0.079,0.00255578821
43864,1.06169721495714
"Aldh18a1",1.42984787271847e-07,0.0696834903500663,0.065,0.014,0.00256
42891749333,0.415736857466672
"Hist1h1e",1.43242894133924e-07,0.219615748406903,0.116,0.036,0.002568
9180633978,1.30984771983405
"Pdia6",1.44835431534262e-07,0.245336220310531,0.639,0.401,0.002597478
62913546,1.46053903679302
"Whsc1l1",1.45937886665998e-07,0.254212576378102,0.361,0.201,0.0026172
5005946801,1.51145420216946
"F13b",1.46583754937874e-07,-0.399795044156731,0.006,0.162,0.002628833
06105584,-2.37526840693296
"Eid1",1.49713873494799e-07,0.178639074580693,0.329,0.17,0.00268496860
725572,1.05755872788508
"Rsu1",1.50115044483696e-07,0.220639082204983,0.265,0.127,0.0026921632

077706,1.30561196484279
"R3hdm2",1.5039927922102e-07,0.224478698864037,0.329,0.173,0.002697260
67354977,1.32790791536844
"Adrm1",1.50417918765939e-07,0.166103993296956,0.348,0.179,0.002697594
95514835,0.982570675000726
"Eif4e3",1.50860101878399e-07,0.11571731861649,0.135,0.046,0.002705525
0670872,0.684173933812233
"Rab15",1.50978002091178e-07,0.156639508477365,0.09,0.024,0.0027076394
8950318,0.926002345711905
"Serbp1",1.51129077682885e-07,0.28654347583009,0.684,0.467,0.002710348
87916486,1.69366619418378
"Zcchc11",1.51570518610646e-07,0.222404639398811,0.213,0.093,0.0027182
6568076333,1.31391350365606
"Srsf1",1.51727601431667e-07,0.23848129830007,0.426,0.237,0.0027210828
0407552,1.40864353926973
"Phyh",1.51960332283939e-07,-0.457420153587756,0.413,0.562,0.002725256
59918016,-2.70115414743706
"Cnn2",1.5288045574977e-07,0.163162798652812,0.142,0.05,0.002741758093
41637,0.96252279031639
"Zfp207",1.53873312642205e-07,0.260761299750576,0.426,0.245,0.00275956
39889253,1.536583570341
"Nmt2",1.54072819307018e-07,0.229832592314059,0.194,0.081,0.0027631419
4145207,1.35403271485003
"Dus4l",1.58296703962927e-07,0.0610957588666971,0.058,0.011,0.00283889
308887113,0.358286367327513
"Ccdc90b",1.58795979180443e-07,0.207812720043367,0.245,0.113,0.0028478
4709062206,1.21803024742932
"Slc35a1",1.5957299662719e-07,0.242220228646755,0.297,0.151,0.00286178
212151203,1.41851692375611
"Rala",1.60498397781579e-07,0.209396982603286,0.413,0.228,0.0028783782
6581483,1.22508296126047
"Eif2b5",1.61494305665961e-07,0.145869138545012,0.232,0.102,0.00289623
887781335,0.852509179934673
"Bak1",1.61919445805976e-07,0.175719057622822,0.174,0.068,0.0029038633
4108437,1.02650034578386
"Eif3j1",1.62739527169817e-07,0.23946451513245,0.445,0.262,0.002918570
68026349,1.39767326235467
"Il13ra1",1.64308143214714e-07,0.249468063034268,0.31,0.159,0.00294670
224041268,1.45366752143378
"Setd5",1.66652610748521e-07,0.202017879227249,0.252,0.118,0.002988747
92116397,1.17430987859184
"Vcl",1.68174274779888e-07,0.180075027509167,0.206,0.088,0.00301603744
390251,1.04512150000282
"Cldn6",1.69055594600718e-07,0.134444388374489,0.065,0.014,0.003031843
03356927,0.779587157628338
"Ermp1",1.69186421022365e-07,0.174940985558543,0.252,0.115,0.003034189
27461509,1.01427477313161
"Tcerg1",1.71079286479864e-07,0.173518418359904,0.174,0.068,0.00306813
592372988,1.00409644484352
"Gstm2",1.72577431720968e-07,0.153396425525397,0.232,0.102,0.003095003

66048384,0.886319360373682
"Cdadc1",1.72693515831336e-07,0.121289936478354,0.135,0.046,0.00309708
551291918,0.700727593996669
"Il34",1.73522895972137e-07,0.205464614744799,0.174,0.068,0.0031119596
1636431,1.18604503299221
"Arhgap12",1.7525641767544e-07,0.18158207119124,0.194,0.08,0.003143048
59459134,1.04637795423622
"Sft2d2",1.79049619443431e-07,0.184338839890814,0.271,0.129,0.00321107
58750985,1.05831678951171
"Skap2",1.79608426716574e-07,0.203790445615828,0.348,0.183,0.003221097
52473504,1.16935632878798
"Rilpl1",1.79768249456627e-07,0.135262715669181,0.11,0.033,0.003223963
78575516,0.776021635302401
"Gal3st1",1.80772434713595e-07,0.129319921965512,0.161,0.06,0.00324197
284415362,0.741206603097626
"Cxxc5",1.82353395841455e-07,0.237919355336076,0.523,0.312,0.003270325
80102065,1.36158050985745
"Rai1",1.82507565326262e-07,0.194457450345905,0.168,0.065,0.0032730906
7656118,1.11268953270247
"Faap100",1.84008853731125e-07,0.150384798660694,0.129,0.043,0.0033000
14782814,0.859272923121027
"Lap3",1.85936540064341e-07,-0.49238286142904,0.2,0.388,0.003334585909
51389,-2.80825974648166
"Gmcl1",1.86658201703064e-07,0.21649956618197,0.232,0.106,0.0033475281
8934275,1.23394643480442
"Slc22a2",1.87287771679804e-07,-0.429706880384357,0.019,0.18,0.0033588
188973056,-2.44768167279421
"Phpt1",1.88961830787408e-07,0.229726764902016,0.548,0.335,0.003388841
47334137,1.30651748678257
"Ano1",1.89512172050933e-07,0.088721781843166,0.058,0.011,0.0033987112
9356143,0.504326454725929
"Exoc6b",1.90970730390536e-07,0.150064386035959,0.174,0.068,0.00342486
907882387,0.851869304671295
"Mdk",1.91272965487715e-07,-0.559052569616207,0.045,0.215,0.0034302893
6305668,-3.17268519674535
"Rap1b",1.92787723754881e-07,0.26134417818017,0.445,0.264,0.0034574550
3782003,1.48109560650149
"Arl1",1.96377660595154e-07,0.220376178797812,0.406,0.229,0.0035218369
651135,1.24485491176049
"Bptf",1.97372462269525e-07,0.266584158060327,0.368,0.204,0.0035396777
3834165,1.50452623532125
"Ppp1r10",1.98584863321202e-07,0.254837081169427,0.284,0.14,0.00356142
093880243,1.4366684262839
"Mrpl52",1.98605318750941e-07,0.290167153835235,0.69,0.507,0.003561787
78647938,1.63581520367941
"Wrnip1",1.98919344288922e-07,0.145249231930502,0.155,0.057,0.00356741
952047753,0.818612000771902
"2610037D02Rik",2.0088651026159e-07,0.195112373433432,0.168,0.066,0.00
360269867503136,1.09771627710475
"Smad1",2.03423661299589e-07,0.232107472190349,0.168,0.067,0.003648199

94174684,1.30294026216594
"N4bp1",2.06536834660352e-07,0.220539377193145,0.194,0.082,0.003704031
59279875,1.23465296860279
"Pgap1",2.08213226432476e-07,0.181388805544668,0.187,0.076,0.003734096
00284003,1.01400868380746
"Filip1l",2.10527134165647e-07,0.0985442015169827,0.084,0.021,0.003775
59362412671,0.549797578236122
"S100a4",2.11049753272316e-07,0.159556585621785,0.155,0.057,0.00378496
627518572,0.889802131282339
"Clock",2.1260031275458e-07,0.248506954319257,0.284,0.142,0.0038127740
0894063,1.38403420107939
"Sgpp2",2.14983097342306e-07,0.149797456020369,0.194,0.08,0.0038555068
6773692,0.832612128970085
"Aspdh",2.16452885658977e-07,-0.433853521789421,0.019,0.178,0.00388186
605140809,-2.40851149125205
"Smad7",2.17477775032812e-07,0.311712128139913,0.252,0.121,0.003900246
41743844,1.72897850637081
"Rnf10",2.17915884680542e-07,0.214584870933393,0.465,0.274,0.003908103
47586084,1.18980939203779
"Il17rc",2.18373956361975e-07,0.211944588994503,0.148,0.054,0.00391631
853339566,1.17472476059615
"Prkd1",2.18729720430762e-07,0.0798922771696224,0.071,0.016,0.00392269
880620529,0.442681141735534
"Agpat2",2.19286889640162e-07,0.163655132639042,0.258,0.121,0.00393269
107880666,0.906392714669683
"Borcs6",2.21089681445935e-07,0.112497820917766,0.135,0.046,0.00396502
234705139,0.622140375275189
"Itpa",2.2496287796622e-07,0.199425388174612,0.284,0.141,0.00403448425
344618,1.09940759652844
"Ptpn1",2.27089588370481e-07,0.217421287664568,0.232,0.106,0.004072624
6778362,1.19657101294857
"Exoc5",2.29409479101278e-07,0.225614283644961,0.252,0.12,0.0041142295
9820232,1.23936777467836
"Igkc",2.30013815962154e-07,-0.21151715134777,0.348,0.187,0.0041250677
7546528,-1.16137147575463
"Gpcpd1",2.30805288062744e-07,0.203755472952768,0.219,0.097,0.00413926
203611725,1.11805472482587
"Zcchc3",2.31500420205369e-07,0.111046109737738,0.097,0.027,0.00415172
853596309,0.609002463792049
"Pfkl",2.33572620488876e-07,0.287102719299864,0.226,0.103,0.0041888913
758475,1.57197902221367
"Lima1",2.3762355473984e-07,0.247326148181715,0.374,0.209,0.0042615408
3070429,1.34993690560396
"Ptpn3",2.37734442158316e-07,0.168830748429245,0.181,0.073,0.004263529
48566724,0.921420475351264
"Rpl5",2.38880018518561e-07,0.290260972494954,0.826,0.626,0.0042840742
5211187,1.58274977437147
"Ang",2.3946170109974e-07,0.0838881815392825,0.084,0.021,0.00429450614
752274,0.45722571448403
"Spint1",2.42066350728289e-07,0.225498960507745,0.368,0.202,0.00434121

793396114,1.22662422231389
"Rpl18",2.42493337796655e-07,0.254362271900545,0.929,0.793,0.004348875
52004521,1.38318081987887
"Zfp280d",2.44673130859152e-07,0.187536528477826,0.232,0.105,0.0043879
6792882802,1.01811500507157
"Rpl19",2.44942804686931e-07,0.251174810711236,0.968,0.896,0.004392804
25925542,1.3633234909611
"Ocln",2.46321162805089e-07,0.198779770492128,0.181,0.073,0.0044175237
3374647,1.07781889709499
"Gse1",2.46626432339499e-07,0.172487494212756,0.155,0.058,0.0044229984
3757658,0.93504391416193
"Ccl6",2.53746257063023e-07,0.237018270976366,0.245,0.115,0.0045506853
7416826,1.27811567571323
"Ggnbp2",2.54090075726883e-07,0.192635220044097,0.439,0.252,0.00455685
141808592,1.03852023713405
"Camta1",2.56958477201385e-07,0.238056785120937,0.29,0.147,0.004608293
33012965,1.28072115095634
"Gm20696",2.57249482391139e-07,0.116461370202811,0.09,0.025,0.00461351
221720268,0.626418440053412
"Rpl31",2.59332404439365e-07,0.299153954648442,0.935,0.822,0.004650867
34121558,1.60666660845268
"Slc1a1",2.61974430703735e-07,-0.417962874955201,0.084,0.262,0.0046982
4944024078,-2.24051728382603
"Aspa",2.61999428028813e-07,-0.427418573365534,0.013,0.167,0.004698697
74226872,-2.29116439071809
"Slc25a39",2.63157271850116e-07,-0.477275770945607,0.265,0.437,0.00471
946251335998,-2.55631783762513
"Prrc2a",2.64426913530572e-07,0.255703134029728,0.316,0.167,0.00474223
226725727,1.36833070856331
"Gnl3",2.65950416389131e-07,0.186594009522476,0.219,0.097,0.0047695547
6752267,0.997438710535601
"Ltbp3",2.66330340856163e-07,0.145543194079733,0.161,0.061,0.004776368
33291443,0.77779371349831
"Zranb1",2.67884226893582e-07,0.204146966586674,0.239,0.111,0.00480423
572510949,1.08978903617727
"Ccdc186",2.71061317645021e-07,0.173566905745633,0.252,0.118,0.0048612
1367064581,0.924498421048094
"Uqcrh",2.77901714144227e-07,-0.285914736398054,0.89,0.906,0.004983889
34146257,-1.51578975554589
"Rab11a",2.78079322623036e-07,0.267235736825153,0.613,0.414,0.00498707
457192153,1.41659146710789
"Gata3",2.79022385626449e-07,0.320945224886021,0.194,0.082,0.005003987
46382473,1.70021380923498
"Brd4",2.7947590896951e-07,0.2078239102279,0.29,0.145,0.00501212095145
918,1.10061383771294
"Plekha1",2.80155561041732e-07,0.271459178749771,0.297,0.15,0.00502430
983172241,1.43696025379923
"Rps18",2.84370640541947e-07,0.266557932681452,0.981,0.923,0.005099903
06747927,1.40703504287215
"Spred1",2.85600063376854e-07,0.178142191904783,0.194,0.081,0.00512195

15366005,0.939561067397838
"Nedd4l",2.86361598870456e-07,0.188600837643249,0.277,0.135,0.00513560891414276,0.994220039761778
"Zfand5",2.89999917097981e-07,0.254524203356457,0.432,0.252,0.00520085851323519,1.33852536793353
"Snapc2",2.90222918673711e-07,0.153690291170633,0.148,0.055,0.00520485782349434,0.808128585995409
"Zfp638",2.90261082591873e-07,0.297007610108623,0.329,0.182,0.00520554225520265,1.5616753407824
"Zfp800",2.90423258031942e-07,0.174160713303406,0.187,0.077,0.00520845070954484,0.915645219299593
"Dach1",2.9088927008685e-07,0.169116939232527,0.174,0.069,0.00521680816973756,0.888856567216025
"Spcs2",2.91177723097866e-07,0.250978701383932,0.535,0.34,0.00522198128603712,1.31886255485226
"Stard13",2.97967046688421e-07,0.11270758679868,0.052,0.01,0.00534374101531014,0.589666855692436
"Pdk4",2.98613124832453e-07,0.146448413551841,0.077,0.019,0.00535532778074521,0.765875903468153
"Strada",3.0082397570318e-07,0.103872944303509,0.097,0.028,0.00539497718026082,0.542454317347174
"Nkap",3.01894843986155e-07,0.169169918856498,0.187,0.078,0.0054141821320477,0.882852713833081
"Cyp2j13",3.0235416488071e-07,-0.405837152470911,0.019,0.178,0.00542241959297065,-2.11733892610499
"Tet1",3.04440873558134e-07,0.109102096510475,0.077,0.019,0.00545984262639158,0.568458506132232
"Akt1",3.05802301934929e-07,0.208994805463324,0.277,0.139,0.00548425848290103,1.08800049523616
"Khdrbs1",3.10589026981986e-07,0.19203506335442,0.361,0.197,0.00557010360989494,0.996727582557237
"2310035C23Rik",3.11544386259482e-07,0.18468955068088,0.252,0.118,0.00558723702317755,0.958034636731111
"Hdgfrp2",3.14745015164897e-07,0.184168031650251,0.187,0.078,0.00564463710196726,0.953446992243456
"Prr15l",3.23493746994415e-07,0.206516678535566,0.413,0.234,0.00580153685859784,1.06348498324525
"Hmg20b",3.24545205448206e-07,0.204266150525464,0.4,0.225,0.00582039371450813,1.05123273738416
"Srp54b",3.28314210148982e-07,0.2573601576761,0.477,0.297,0.00588798704481184,1.32150351457866
"Cep44",3.29059238618763e-07,0.107377513454535,0.116,0.037,0.0059013483853889,0.551123078226324
"Aqr",3.29829636374734e-07,0.189973119864647,0.232,0.106,0.00591516469874448,0.974606926724408
"Ttll7",3.3129714438469e-07,0.0678134864524534,0.052,0.01,0.00594148298739504,0.347598132568005
"Pnrc2",3.31789570285468e-07,0.193704768035457,0.335,0.18,0.00595031415349959,0.992603524329934
"Cdkl2",3.33008758456986e-07,0.122155319549586,0.142,0.051,0.005972179

07416759,0.625513832552197
"Timd2",3.34253142297366e-07,-0.435395403687213,0.045,0.211,0.00599449
585396096,-2.22788065747896
"Gtpbp4",3.36386943112722e-07,0.144925564705379,0.245,0.112,0.00603276
343778355,0.740649357989795
"Sertad2",3.41003759348494e-07,0.248682841073499,0.258,0.126,0.0061155
6142015589,1.26751622396406
"2410015M20Rik",3.41161245841244e-07,-0.396821857387466,0.761,0.76,0.0
0611838578291687,-2.0223855241887
"Ift43",3.4408773102926e-07,0.149754699387231,0.239,0.11,0.00617086936
827874,0.761939263475386
"Llgl2",3.44720066620119e-07,0.179780043806716,0.297,0.151,0.006182209
67476521,0.91437559870657
"Apaf1",3.46268913199645e-07,0.0894294838057029,0.071,0.017,0.00620998
668932244,0.454444554270652
"Actl6a",3.4848119218922e-07,0.186119600818771,0.284,0.143,0.006249661
70072147,0.944599399110128
"Rpl17",3.49554646053107e-07,0.28976201879032,0.942,0.873,0.0062689130
2231642,1.4697170903608
"Ptprf",3.52041187302209e-07,0.223986165505923,0.503,0.306,0.006313506
65307782,1.13450426951694
"Zswim8",3.52686184639084e-07,0.163968497638397,0.174,0.07,0.006325074
03531733,0.83021079662293
"Ddx54",3.57138192536014e-07,0.190603357453075,0.239,0.11,0.0064049163
4494088,0.962678357972843
"Gskip",3.68226564150947e-07,0.131691775814177,0.181,0.073,0.006603775
20148308,0.661107700222972
"Gtf2ird1",3.68678786430027e-07,0.141582413538278,0.168,0.066,0.006611
88535583611,0.710586055163237
"Caap1",3.71143639908674e-07,0.133695540657275,0.168,0.065,0.006656090
03812215,0.670111870322967
"Hadh",3.71372621873502e-07,-0.521601302979829,0.361,0.508,0.006660196
60067938,-2.61406036318916
"Ppil4",3.79903284486337e-07,0.197912515755975,0.187,0.078,0.006813185
50397798,0.987364859173048
"Defb29",3.81460453482903e-07,-0.490321768391324,0.206,0.397,0.0068411
1177276239,-2.44415841282494
"Gprc5c",3.8325710910191e-07,0.260336640601903,0.471,0.292,0.006873332
99463366,1.29650410189604
"Csrnp1",3.84866140029994e-07,0.136287793960289,0.084,0.022,0.00690218
935529792,0.678156699048055
"Tprgl",3.86437116570875e-07,0.22222665683904,0.4,0.228,0.006930363248
58207,1.10487605940352
"Abca3",3.88556523370108e-07,-0.498460801751356,0.219,0.382,0.00696837
269011953,-2.47554254397687
"Car4",3.88644001118834e-07,-0.547554292119492,0.135,0.311,0.006969941
51606517,-2.7192358963915
"Med12",3.89994382119118e-07,0.110723832827198,0.135,0.047,0.006994159
24892426,0.549486936437772
"Mpp1",3.90680137186051e-07,-0.427000550001721,0.065,0.231,0.007006457

58029464,-2.11831686817968
"Huwe1",3.90737044542288e-07,0.207825156362276,0.484,0.292,0.007007478
15682139,1.0309743371801
"Phf13",3.91334139830172e-07,0.102854681042417,0.077,0.019,0.007018186
4637143,0.510082121477355
"Tmem150a",3.93611822195242e-07,-0.421499449963291,0.039,0.199,0.00705
903441924947,-2.08787518787902
"Sept8",3.93701824726516e-07,0.133846244042796,0.09,0.025,0.0070606485
2464535,0.662969675107099
"Grb14",3.94972955819422e-07,0.171659422237954,0.194,0.082,0.007083444
98966552,0.849713266186734
"Gipc1",4.03851746352798e-07,0.198170752113757,0.342,0.186,0.007242677
21909108,0.976538769320751
"Ndufb2",4.04319788712384e-07,-0.367662455964599,0.735,0.78,0.00725107
109076789,-1.81132809254957
"Glul",4.05091534567776e-07,-0.485844037453393,0.239,0.422,0.007264911
58093849,-2.39263571953574
"Frk",4.11176402083256e-07,0.242313849016336,0.239,0.114,0.00737403759
496112,1.18971008389508
"Marveld1",4.12252263279326e-07,0.121616232839236,0.077,0.019,0.007393
33208965143,0.596792350691025
"Wbp4",4.20492774370095e-07,0.249753289969385,0.323,0.174,0.0075411174
1555328,1.22064046051765
"Timm13",4.20782630117035e-07,-0.406008980613052,0.671,0.7,0.007546315
68851891,-1.98404238970365
"Taz",4.25594823444978e-07,0.216725635204708,0.277,0.14,0.007632617563
66224,1.05660778401596
"Ctps2",4.3045426179706e-07,0.200757998303594,0.181,0.075,0.0077197667
3106848,0.976481108183432
"Cfdp1",4.31710306634853e-07,0.182834094753287,0.49,0.288,0.0077422926
3918946,0.888767034506829
"Rin1",4.36137229844848e-07,0.059385690444904,0.052,0.01,0.00782168508
003751,0.288071389116221
"Eps8l2",4.36349686605825e-07,0.273760030614944,0.361,0.202,0.00782549
527958887,1.32783696062547
"Nipsnap3b",4.42027628169626e-07,0.192951163362527,0.284,0.142,0.00792
732348359407,0.933389640606153
"Tmem35",4.43335010035824e-07,0.111627866987194,0.11,0.034,0.007950770
06998247,0.539663414952903
"Lamc2",4.47874268374468e-07,0.213915027631501,0.168,0.067,0.008032177
12902772,1.03199019579011
"Naa35",4.5401090849805e-07,0.209015088152399,0.252,0.122,0.0081422316
3300402,1.00550699929063
"Yes1",4.6154523986401e-07,0.260886765778032,0.219,0.1,0.0082773523317
2115,1.25075171437304
"Spry1",4.61992498215443e-07,0.113301458793316,0.084,0.022,0.008285373
46299575,0.543083752835282
"Ddit3",4.63958474957997e-07,0.20911072808286,0.181,0.075,0.0083206312
8989672,1.00143486321303
"Rbmxl1",4.6590795507983e-07,0.204573783969794,0.194,0.083,0.008355593

26640167,0.978849574243554
"BC029214",4.68228124745927e-07,0.135490772682725,0.181,0.074,0.008397
20318919345,0.647626461640536
"Cacna1a",4.69774439636016e-07,0.0880336003380377,0.052,0.01,0.0084249
348004323,0.420497733675593
"Fxyd5",4.7549669344992e-07,0.268276641841096,0.258,0.128,0.0085275577
0033086,1.27819125716926
"Syt17",4.7792309287276e-07,0.146757844944561,0.123,0.041,0.0085710727
4758008,0.698473766119673
"Slc12a1",4.78706639250125e-07,0.561440859928829,0.342,0.188,0.0085851
2486831174,2.67118078849657
"Psmc1",4.79489465331203e-07,0.192553772712531,0.426,0.246,0.008599164
0712498,0.915803127057388
"Cd81",4.82363443637326e-07,0.287346817414408,0.865,0.678,0.0086507059
981918,1.36493023883882
"Phactr4",4.82806586134336e-07,0.227677131901059,0.277,0.14,0.00865865
331573318,1.08128334113861
"Atp5f1",4.8477146365246e-07,-0.312942316761382,0.755,0.805,0.00869389
142914322,-1.48495342586973
"Hoxc6",4.87758843277599e-07,0.225227265806711,0.161,0.062,0.008747467
09534045,1.06735000708505
"Stx6",4.91406283571256e-07,0.18007524784249,0.226,0.103,0.00881288028
956691,0.852033410751551
"Irf8",4.9365237424839e-07,0.199435842418659,0.161,0.063,0.00885316167
977063,0.942729364401734
"Aida",4.94858341379731e-07,0.193405556998678,0.181,0.075,0.0088747894
9430409,0.913752418462104
"Fam133b",4.94896307231479e-07,0.171480182891735,0.361,0.195,0.0088754
7037388934,0.810151941379773
"Ica1",4.95925838648194e-07,0.137651693251559,0.116,0.038,0.0088939339
9031671,0.650044402760608
"Zfp91",5.02483582375995e-07,0.238277123945433,0.419,0.248,0.009011540
5663311,1.12210636435463
"Cd200",5.05547955613361e-07,0.181874678883014,0.174,0.07,0.0090664970
3597002,0.855387406859495
"Snrnp48",5.06753561306034e-07,0.137930278977378,0.194,0.081,0.0090881
1836846241,0.648380916445609
"Lmo4",5.11944600368578e-07,0.327752367010327,0.413,0.25,0.00918121446
301008,1.53735387242013
"Casp12",5.15090023907467e-07,0.0963559878530095,0.065,0.015,0.0092376
2448875651,0.451376784170551
"Pgm5",5.2269643424207e-07,0.117107487701334,0.058,0.012,0.00937403785
169729,0.546869874247495
"Ifi35",5.24689981620583e-07,0.191833178143689,0.206,0.092,0.009409790
13038353,0.895094497111044
"Sulf2",5.264884046265e-07,0.122749891758206,0.103,0.031,0.00944204304
857166,0.572331545994596
"Arhgef5",5.29638525972186e-07,0.199510564719812,0.277,0.138,0.0094985
3732478519,0.929044378738032
"Rwdd1",5.3578482027196e-07,0.239186747267036,0.49,0.299,0.00960876496

675734,1.11104147546129
"Upb1",5.43106486625723e-07,-0.392971879694269,0.026,0.18,0.0097400717
3114571,-1.82005193173987
"Arhgef17",5.52582794479021e-07,0.144848886933788,0.148,0.055,0.009910
01983618677,0.668363027446881
"Trip11",5.57101049119974e-07,0.254867546138553,0.316,0.17,0.009991050
21491761,1.17393662796313
"Arrdc4",5.57213461481549e-07,0.206432042048337,0.181,0.076,0.00999306
62182101,0.950797870593498
"Mkln1",5.58083900596484e-07,0.235912348290984,0.432,0.259,0.010008676
6732973,1.08621190817036
"Lcor",5.61689717416695e-07,0.134527549127551,0.168,0.067,0.0100733433
92151,0.618539188450807
"Unc13b",5.70304056969662e-07,0.110953494010956,0.11,0.035,0.010227832
9576939,0.508460203119738
"Trim26",5.71054812210725e-07,0.143261812852603,0.2,0.086,0.0102412970
021871,0.656329212296514
"Msantd3",5.73215921900184e-07,0.0815042775113085,0.071,0.017,0.010280
0543433579,0.37308988373174
"Dkk2",5.80918503240785e-07,0.0815618377029292,0.071,0.017,0.010418192
4371202,0.37226468060137
"Ppp2r3a",5.93073164085749e-07,0.251245607637507,0.432,0.256,0.0106361
741247138,1.14153301998757
"Fcgrt",5.97937796901433e-07,-0.452424168474108,0.181,0.361,0.01072341
64496303,-2.05189085699152
"Magi1",5.9950447869139e-07,0.211960481101378,0.174,0.072,0.0107515133
208514,0.960755129522126
"Rfc3",6.00072008840755e-07,0.123707426223713,0.116,0.038,0.0107616914
065501,0.560612680412576
"Ube2b",6.0339388107877e-07,0.199437330276784,0.613,0.393,0.0108212658
632667,0.902701624555428
"Rbm26",6.03740343395896e-07,0.195813942914921,0.29,0.148,0.0108274793
18462,0.886188894419114
"Eef1g",6.1031172757838e-07,0.287465400583263,0.781,0.593,0.0109453305
223907,1.29786096470751
"Gadd45b",6.10342355357692e-07,0.252146120130549,0.142,0.053,0.0109458
798009848,1.13838732820849
"Papss2",6.14064687507802e-07,-0.396089708713098,0.032,0.188,0.0110126
361057649,-1.78585439446625
"Dnah12",6.28728146722668e-07,0.127109029339508,0.077,0.02,0.011275610
5833243,0.570098390582979
"Rps4x",6.3896119383084e-07,0.299843058452417,0.948,0.824,0.0114591300
501623,1.33998917789933
"Fam171a2",6.43740459567533e-07,0.149802288992345,0.129,0.045,0.011544
8414018841,0.668345395110886
"Adrbk2",6.47480277885217e-07,0.121605326127102,0.129,0.045,0.01161191
13035935,0.541839754488268
"Arpc4",6.4911797457557e-07,0.223871171235155,0.452,0.272,0.0116412817
560383,0.996942590715768
"Fhl2",6.50838176849431e-07,0.109591360101603,0.077,0.02,0.01167213186

36177,0.48774195596528
"Ncor1",6.53873019590862e-07,0.272754134536846,0.613,0.393,0.011726558
7333425,1.21263734284109
"Gm26532",6.5928738675621e-07,0.160608370386573,0.09,0.026,0.011823659
9940859,0.712724163793084
"Lmo7",6.65329266035585e-07,0.303232406494899,0.355,0.203,0.0119320150
570822,1.34287385474791
"Cdc42se1",6.6841615140363e-07,0.232786996686752,0.361,0.2,0.011987375
2592727,1.02982668439874
"Ehhadh",6.68785515305148e-07,-0.415169028742627,0.026,0.176,0.0119939
994314825,-1.83643742576507
"Atp1a1",6.68903408053096e-07,0.352809339444073,0.968,0.866,0.01199611
37200242,1.56053658148891
"Dnajc7",6.7124507670094e-07,0.193541080789725,0.355,0.196,0.012038109
2055546,0.855389236378564
"Gm53",6.75139443461592e-07,0.194536763011271,0.168,0.068,0.0121079507
790402,0.858664447353156
"Zfp646",6.87284057192626e-07,0.203713323750034,0.155,0.061,0.01232575
22816925,0.895536915575521
"Pde2a",6.90888212762797e-07,0.104856060523933,0.058,0.012,0.012390389
207688,0.460405573562343
"Maged1",6.95530826564216e-07,0.216700176826239,0.387,0.223,0.01247364
98436026,0.95004323542999
"Mob1a",6.98624272183777e-07,0.215635966745436,0.258,0.128,0.012529127
6973439,0.944420655871675
"Ptpn6",7.01968190623516e-07,0.195001084317512,0.206,0.092,0.012589097
5306421,0.853114946219618
"Purb",7.02222715924974e-07,0.265453283855172,0.374,0.218,0.0125936621
873985,1.16124174017502
"Aldh9a1",7.05819423774748e-07,-0.492995547965232,0.155,0.322,0.012658
1655459763,-2.15412076479741
"Usp53",7.07455066694986e-07,0.286252039213205,0.206,0.094,0.012687499
1661079,1.25010218318864
"Msl1",7.09407305717596e-07,0.255109304927095,0.277,0.146,0.0127225106
207394,1.11339455149518
"Rtf1",7.10459962509239e-07,0.264012794380965,0.361,0.208,0.0127413889
676407,1.15186131780689
"Drap1",7.18130520685458e-07,0.234507177146774,0.548,0.347,0.012878952
757973,1.02061295992999
"Gm2a",7.22144018142968e-07,-0.513603893769229,0.303,0.461,0.012950930
821376,-2.23242432383806
"Rps6ka4",7.24419285725537e-07,0.132461012337818,0.148,0.056,0.0129917
354702018,0.575336705375021
"Taok3",7.25369099607947e-07,0.203485682854598,0.245,0.118,0.013008769
4323689,0.883561609035357
"Myo10",7.27023132475477e-07,0.163606928823085,0.297,0.15,0.0130384328
578152,0.710030169644898
"Cnksr1",7.31906281344068e-07,0.155000318504345,0.116,0.038,0.01312600
72496245,0.671641138376379
"Pygl",7.34440624123546e-07,0.156157138537041,0.129,0.046,0.0131714581

530317,0.676114039250709
"Nrde2",7.44497586464041e-07,0.125068764857492,0.11,0.035,0.0133518197
156461,0.539809620571991
"Lpcat2",7.45786201044279e-07,0.0929347539669584,0.071,0.017,0.0133749
297295281,0.40095521558855
"Ltc4s",7.46224273611546e-07,0.210290663278082,0.116,0.039,0.013382786
1229495,0.907148922448513
"Cnst",7.47268627788772e-07,0.122107455090033,0.103,0.032,0.0134015155
707638,0.526574660071761
"Fgf9",7.52193590011705e-07,0.175921041071097,0.174,0.071,0.0134898398
432699,0.757484069856294
"Pa2g4",7.52285065082317e-07,0.206113060336838,0.342,0.186,0.013491480
3571863,0.887460360285403
"Smad2",7.55117624900026e-07,0.180472412416749,0.187,0.08,0.0135422794
849571,0.77638125052067
"Sbno2",7.57814913405616e-07,0.108378696682761,0.084,0.023,0.013590652
6570163,0.465852066541386
"Birc2",7.65262707078627e-07,0.210028078196957,0.226,0.106,0.013724221
3887481,0.900724950708283
"Wfdc15b",7.73942997193843e-07,0.293225293436303,0.439,0.26,0.01387989
37116744,1.25421664737185
"Cdkn1b",7.76090936607518e-07,0.273526672130555,0.226,0.108,0.01391841
48571192,1.16920138648308
"Cyc1",7.78361946002128e-07,-0.373043036445543,0.619,0.716,0.013959143
1396022,-1.59349830544546
"Abi2",7.81632286769421e-07,0.153452935874017,0.155,0.06,0.01401779343
09228,0.6548493240801
"Fblim1",7.93725641820651e-07,0.0944922739796376,0.071,0.017,0.0142346
756604116,0.401788173816459
"Gtf3c6",7.9458040713121e-07,0.136185851579997,0.368,0.202,0.014250005
0214911,0.578925785402539
"Pak1ip1",7.94705211691382e-07,0.196548909580438,0.277,0.141,0.0142522
432664733,0.835498155969093
"Lamp2",7.97244337112146e-07,-0.404451072206172,0.729,0.746,0.01429777
99417692,-1.71796700235755
"Nr2f2",7.97736828779299e-07,0.235257835023402,0.406,0.238,0.014306612
287328,0.999147894924564
"Gm4450",7.98328496284537e-07,-0.344825110714135,0,0.137,0.01431722325
23669,-1.46422812392431
"2410022M11Rik",7.99118280021402e-07,0.109928994479486,0.11,0.035,0.01
43313872339038,0.466681915944923
"Patz1",7.99700828602225e-07,0.176613140657477,0.161,0.065,0.014341834
6601523,0.749647635311189
"Chfr",7.99777219640457e-07,0.170379224707911,0.174,0.072,0.0143432046
57032,0.723171039945532
"Slc16a2",8.0921053838889e-07,-0.429293719643806,0.097,0.268,0.0145123
817954664,-1.8170943127619
"Cyp4a31",8.12186862632631e-07,-0.362438804194532,0.013,0.158,0.014565
7591944536,-1.5327833378754
"Xaf1",8.12847520067559e-07,0.125861966492818,0.11,0.035,0.01457760742

48916,0.532178209121082
"Zfp316",8.20367057212742e-07,0.0836480088308821,0.084,0.023,0.0147124628040533,0.352915996125443
"Pigt",8.20418281612876e-07,0.224681972308839,0.329,0.178,0.0147133814624453,0.947932768373786
"Rhoc",8.22290470241339e-07,0.247537245044182,0.419,0.247,0.0147469572933082,1.04379488127912
"Rab13",8.24820396882949e-07,0.154689166576366,0.142,0.053,0.0147923289976988,0.651805472068814
"Mark3",8.32026479308698e-07,0.220212509597285,0.355,0.2,0.0149215628799222,0.92598213837917
"Scnn1a",8.32289310772221e-07,0.199272984609036,0.284,0.141,0.014926276499389,0.837869586905737
"Nmt1",8.33243480564333e-07,0.244793982892686,0.381,0.224,0.0149433885804407,1.02898815644297
"Kif13b",8.39343960917202e-07,0.137244954152509,0.103,0.032,0.0150527945950891,0.575906126178651
"Smarcd1",8.41159514705459e-07,0.141127180660068,0.168,0.068,0.0150853547367277,0.59189175521828
"Espn",8.47996622746355e-07,-0.383444986978996,0.026,0.175,0.0152079714323331,-1.60507600333159
"Tnk1",8.52353424259927e-07,0.112846747083408,0.097,0.029,0.0152861063106775,0.471790915586087
"2310033P09Rik",8.53801166139498e-07,0.173108081457665,0.2,0.089,0.0153120701135458,0.723438383413252
"Ccl4",8.61815478501901e-07,0.178580483373356,0.187,0.08,0.0154557987914531,0.744639724262818
"Fam96b",8.69023478495852e-07,0.198467184019365,0.4,0.229,0.0155850670633446,0.825909687644238
"Rexo1",8.76433259714897e-07,0.16714022995779,0.206,0.093,0.015717954079727,0.69412529345884
"Nrep",8.86606889752506e-07,0.13918587576139,0.129,0.046,0.0159004079608214,0.57642584902406
"Ppp2r2a",8.87931760905111e-07,0.181554930169619,0.232,0.11,0.0159241682000723,0.75162239834702
"Trim41",8.94982182941888e-07,0.173800740427851,0.181,0.076,0.0160506104688798,0.718146118450388
"Mxra8",9.02947452201873e-07,0.0801201535469462,0.058,0.012,0.0161934596077884,0.330347238532554
"G3bp1",9.04601589536376e-07,0.212590936404723,0.458,0.279,0.0162231249067454,0.876154765939096
"Brox",9.13247625200369e-07,0.16444457289754,0.194,0.085,0.0163781829103434,0.676164040109097
"Myl12b",9.13292150329211e-07,0.328608517169646,0.561,0.385,0.0163789814240041,1.35115816916919
"Dapk1",9.25060993045749e-07,0.152049832585573,0.213,0.095,0.0165900438492825,0.623245046216331
"Cep350",9.28744050989628e-07,0.158515302915074,0.194,0.084,0.016656095810448,0.649116838964701
"Fhl1",9.2917558660384e-07,-0.388117158745484,0.032,0.184,0.0166638349

701533,-1.58915132581743
"Zfp408",9.34452081484151e-07,0.099548283797707,0.065,0.015,0.01675846
36293368,0.40703818506981
"Btf3",9.38609581013302e-07,0.269275897470968,0.832,0.639,0.0168330242
258926,1.09983386698688
"Nasp",9.40630849491234e-07,0.187258086930574,0.206,0.092,0.0168692736
547758,0.764436467377948
"Pld2",9.42988538752277e-07,0.102631507616649,0.065,0.015,0.0169115564
539833,0.418711722143661
"N4bp2",9.64076865660714e-07,0.187144807573288,0.161,0.064,0.017289754
5087592,0.759366477428328
"Ik",9.73747495625453e-07,0.231079060963412,0.368,0.211,0.017463187586
5469,0.935329513525593
"Tiam1",9.74353422642016e-07,0.109441912352479,0.077,0.02,0.0174740542
816619,0.442915590196901
"Atxn10",9.79426578854055e-07,0.250605106597458,0.335,0.19,0.017565036
2651686,1.01290697919216
"Slc6a8",9.79859355968597e-07,-0.444522449619315,0.135,0.306,0.0175727
976899408,-1.7964944318653
"Rc3h2",9.95140563658007e-07,0.20663108950391,0.252,0.123,0.0178468508
686427,0.831881931934279
"Rela",9.98707022836609e-07,0.15798672603366,0.181,0.076,0.01791081174
75517,0.635478024408304
"Ttc19",1.00457115955443e-06,0.193569234461925,0.226,0.106,0.018015979
1754492,0.777470091346596
"Dleu7",1.01618492294015e-06,-0.322642363569097,0.006,0.147,0.01822426
04080087,-1.29218317686616
"Leo1",1.016984065825e-06,0.140808537888369,0.135,0.049,0.018238592236
5055,0.563827726752901
"Parp1",1.01875355988327e-06,0.181236856924385,0.245,0.119,0.018270326
3429466,0.725396359823553
"Acot9",1.02343710186748e-06,0.178613799767834,0.258,0.127,0.018354320
9848914,0.714078369712144
"Rpl36a",1.02630385558347e-06,0.301183806913774,0.916,0.795,0.01840573
33460339,1.20325733919798
"Nudcd1",1.03698421804744e-06,0.0887642775152304,0.097,0.029,0.0185972
749664627,0.353702586374713
"Phkg2",1.04010140751868e-06,0.138416773742331,0.187,0.08,0.0186531786
4244,0.551139426237073
"Mob3a",1.0414108943395e-06,0.0817489038479759,0.071,0.018,0.018676662
9790846,0.3253999179507
"Tmem191c",1.04159315078876e-06,0.0886010717453853,0.097,0.029,0.01867
99315662457,0.352659334030413
"Tbc1d2",1.07150878803508e-06,0.0932343134047351,0.084,0.023,0.0192164
386046212,0.368460998613301
"U2af1",1.07346451204332e-06,0.191799306478466,0.484,0.298,0.019251512
5589849,0.757639031539439
"Reep5",1.07542549385731e-06,0.236921489890759,0.548,0.347,0.019286680
806837,0.935446722135177
"Zcchc2",1.07575342942634e-06,0.101890901142188,0.11,0.036,0.019292562

0033319,0.402268909690096
"Dnm2",1.07693941149039e-06,0.177103048890009,0.284,0.146,0.0193138314
056686,0.699014007374043
"Tuba1b",1.07878675822185e-06,0.251286570309628,0.471,0.285,0.01934696
17219507,0.991380774970933
"Fam60a",1.08939752366244e-06,0.19135679809319,0.187,0.082,0.019537255
1893622,0.753071688373143
"B4galt1",1.09370017749446e-06,0.14776277143021,0.232,0.108,0.01961441
89831856,0.58092790565322
"Trip10",1.09859621354033e-06,0.161262307717902,0.135,0.05,0.019702224
4936324,0.633280909348942
"Cry1",1.10011796387251e-06,0.105124929977007,0.065,0.015,0.0197295155
640896,0.41268257886797
"Crem",1.11350209610301e-06,0.157617837640733,0.213,0.096,0.0199695465
915114,0.616844789842285
"Abi1",1.13007046894676e-06,0.183898725713266,0.252,0.125,0.0202666837
900912,0.716980109961069
"Ptpn23",1.13462588567565e-06,0.114376344820634,0.161,0.064,0.02034838
06337072,0.445467720319115
"Psmd7",1.13653366241272e-06,0.174722613650103,0.413,0.239,0.020382594
7017097,0.680208054491042
"Calu",1.1422419499906e-06,0.155152778545209,0.277,0.14,0.020484967131
1315,0.603243928497705
"Scarf2",1.15694125098978e-06,0.114681137038021,0.065,0.015,0.02074858
43952507,0.444421202118538
"Nol7",1.15806750991918e-06,0.170139414387939,0.523,0.324,0.0207687827
228906,0.659171856303267
"Nup93",1.17330389230565e-06,0.108060363170143,0.116,0.039,0.021042032
0046095,0.417246274585609
"Denr",1.17522599071154e-06,0.171256132366772,0.477,0.287,0.0210765029
174207,0.660979563063121
"Pdcd10",1.18369374293279e-06,0.184008698779271,0.4,0.233,0.0212283635
857567,0.708878255244296
"Dgat2",1.18847797042557e-06,-0.378612575183385,0.039,0.19,0.021314163
9216123,-1.45704636997017
"2610044O15Rik8",1.19144725747424e-06,0.114878631025871,0.103,0.032,0.
0213674151155431,0.441810368683304
"AW554918",1.19429131343554e-06,0.0807277112973993,0.077,0.02,0.021418
4204151529,0.310277278064482
"Cct7",1.19650687479791e-06,0.190689651122353,0.484,0.301,0.0214581542
926258,0.73256300356081
"Rsrc1",1.20065956001612e-06,0.198861523227966,0.226,0.108,0.021532628
549329,0.763267491981484
"2310001H17Rik",1.20259427026045e-06,0.187663906889167,0.194,0.085,0.0
21567325642851,0.719986805825083
"Pgm1",1.21013707269509e-06,0.116881183854332,0.123,0.043,0.0217025982
617138,0.447692720684768
"Habp2",1.22972721865693e-06,-0.385354182937318,0.077,0.242,0.02205392
79393934,-1.46984280114233
"Casp2",1.23963237154873e-06,0.117663022672848,0.103,0.033,0.022231566

951355,0.447853946433633
"Prkch",1.24691917628944e-06,0.0887419347769122,0.071,0.018,0.02236224
85075748,0.337253169607369
"Trpv5",1.25172790633934e-06,0.276012747081713,0.11,0.036,0.0224484882
722897,1.04789122797283
"Stard10",1.26205708221096e-06,-0.450843850499824,0.335,0.486,0.022633
7317123714,-1.70793803991946
"Cyp4a10",1.27564077111854e-06,-0.44060009892846,0.006,0.143,0.0228773
415892398,-1.66441459443918
"Dicer1",1.29084640740542e-06,0.17168016627801З,0.2,0.088,0.0231500394
704088,0.646506095825148
"Klc1",1.29294571392766e-06,0.162639248879227,0.181,0.077,0.0231876884
335786,0.612195895886047
"Nufip2",1.29550904018239e-06,0.287958158269967,0.265,0.138,0.02323365
91266309,1.08334271446705
"Rhpn2",1.30960362043073e-06,0.113875789817844,0.103,0.033,0.023486431
3288046,0.42718594161028
"Dusp18",1.31393946331017e-06,0.127575350425069,0.103,0.033,0.02356419
03350046,0.478155867877395
"Ppp1r12a",1.31418553523596e-06,0.21880608137498,0.213,0.101,0.0235686
033889216,0.820050144098749
"Eml4",1.315385737703e-06,0.15414243181437,0.187,0.081,0.0235901278199
656,0.577560434552133
"Gzma",1.32294915993025e-06,0.100388736469574,0.065,0.015,0.0237257702
341892,0.375573685334103
"2900060B14Rik",1.33240270841667e-06,0.0993937543168036,0.065,0.015,0.
0238953101727446,0.371143540960921
"Ncstn",1.33456179732187e-06,0.162367242151561,0.258,0.127,0.023934031
2731704,0.606028251102647
"Fktn",1.33464088986153e-06,0.151055841730753,0.206,0.093,0.0239354497
187768,0.563800018116882
"Lars",1.33851808156417e-06,0.195772247036018,0.2,0.09,0.0240049832747
718,0.730131388138023
"Rel",1.34110604434984e-06,0.131729557586664,0.084,0.024,0.02405139579
93701,0.491030125636997
"Lypd6b",1.35391133392968e-06,0.0813280308496766,0.065,0.015,0.0242810
458626949,0.30238243646175
"Lmnb1",1.35805784498485e-06,0.131446282650418,0.097,0.03,0.0243554093
919583,0.488323111255754
"Cdc42ep5",1.35828700355889e-06,0.161790298733914,0.174,0.073,0.024359
5191218252,0.601023872349966
"Ankrd17",1.37338505428638e-06,0.265290467389002,0.374,0.221,0.0246302
87563572,0.982577100607536
"Casz1",1.40784372334108e-06,0.195970362907683,0.226,0.106,0.025248269
3343989,0.720974507903577
"Hoxa9",1.4126060035273e-06,0.206648297217256,0.31,0.165,0.02533367606
72585,0.759560758558364
"Mfap1a",1.43522987359584e-06,0.143526959282807,0.194,0.085,0.02573941
25530678,0.525270191312715
"Mboat2",1.4365728535053e-06,0.0981632774746997,0.103,0.033,0.02576349

75547641,0.359159467078764
"Nfxl1",1.44377574375985e-06,0.135138815966246,0.129,0.046,0.025892674
1885891,0.493769557131106
"Aff4",1.44995354521438e-06,0.196647395152439,0.329,0.182,0.0260034668
798748,0.717669665088021
"Prkacb",1.45850905844305e-06,0.158671361105065,0.265,0.133,0.02615690
14541177,0.578141668103051
"1700011H14Rik",1.45980968058537e-06,0.253872206665478,0.619,0.4,0.026
180226811618,0.924793197889513
"Ssrp1",1.46115326420365e-06,0.240912408193206,0.284,0.153,0.026204322
6402283,0.877362251280712
"Dhx15",1.46246802349645e-06,0.209570063944564,0.361,0.205,0.026227901
5333854,0.763030245353822
"Tnfaip8",1.46312110135997e-06,-0.516618432749747,0.297,0.445,0.026239
6138317896,-1.8807416715727
"Ghr",1.4780043251196e-06,-0.503314477356585,0.219,0.385,0.02650652956
66948,-1.82721484866178
"Ing4",1.48314203134447e-06,0.184740329709569,0.232,0.113,0.0265986691
901317,0.67003361091208
"Afg3l1",1.48479847462354e-06,0.194355978177044,0.316,0.172,0.02662837
58438985,0.704691604718584
"Paf1",1.49263582087803e-06,0.121946859170827,0.194,0.084,0.0267689308
116266,0.441510233087373
"Stxbp3",1.50061387697646e-06,0.194958176463188,0.245,0.121,0.02691200
92696959,0.704809417170982
"Bcap31",1.5095866816647e-06,0.254921487254132,0.542,0.355,0.027072927
5489748,0.920067993776832
"Golm1",1.51568276395332e-06,0.109365204936808,0.084,0.024,0.027182254
6887389,0.394282443773218
"Phax",1.51963808859102e-06,0.197908797794274,0.258,0.13,0.02725318948
07914,0.712983210799246
"Gsk3a",1.52523690910749e-06,0.144035278207695,0.213,0.097,0.027353598
7279337,0.518369596659176
"Gm10804",1.52819285235794e-06,-0.317546913198773,0,0.131,0.0274066106
141873,-1.14220704788518
"Napg",1.55278036324147e-06,0.163069668207773,0.194,0.086,0.0278475630
343725,0.583954083145888
"Sdf4",1.5550325913622e-06,0.194263765669389,0.458,0.276,0.02788795449
34897,0.695378887012096
"Fbxl3",1.56491935802744e-06,0.162772149570481,0.284,0.146,0.028065263
7668641,0.581621128737864
"Eef1a1",1.56514666554633e-06,0.219750967679544,0.987,0.952,0.02806934
02999079,0.78518721423257
"Diaph2",1.5719744828098e-06,0.160262706882639,0.194,0.085,0.028191790
374711,0.571933442860974
"Suclg1",1.57765000923672e-06,-0.442096870363036,0.497,0.591,0.0282935
752656513,-1.57612862547383
"Spopl",1.58003876620809e-06,0.199089196060183,0.194,0.087,0.028336415
2331758,0.709475761036767
"Serf2",1.59109262655578e-06,-0.267255032593181,0.89,0.916,0.028534655

1646513,-0.950528859334805
"Zfp397",1.59517503377319e-06,0.179168204666473,0.206,0.094,0.02860786
90556884,0.636776960578144
"Sepn1",1.60115147820003e-06,0.102075132994566,0.071,0.018,0.028715050
6100393,0.362400803197879
"Smarcd2",1.61664953009875e-06,0.141425297396721,0.174,0.073,0.0289929
92672791,0.500744707498142
"Id3",1.6191789779137e-06,0.619243122896553,0.355,0.213,0.029038355789
9043,2.19158668755049
"Pds5b",1.6548678141984e-06,0.192958976765657,0.174,0.073,0.0296783993
79834,0.678701515247463
"Clcn4",1.6550468338015e-06,0.213827694392503,0.303,0.163,0.0296816099
173961,0.752080672564281
"Lst1",1.67364597232754e-06,0.27052506298778,0.194,0.087,0.03001516686
77222,0.948475063341792
"Tmem45b",1.69195916818405e-06,0.214207079111269,0.129,0.046,0.0303435
957222127,0.748690113392882
"Fcgr4",1.69530952175788e-06,0.0910773582227097,0.103,0.033,0.03040368
09632058,0.318150662109417
"Nudt19",1.70872362277386e-06,-0.667155940230307,0.284,0.426,0.0306442
494508265,-2.32524543955366
"Rnf149",1.75525054684184e-06,0.24253297075401,0.439,0.262,0.031478663
3070615,0.83878701716258
"Npl",1.76331379173365e-06,-0.384542807148196,0.026,0.169,0.0316232695
409512,-1.32815780878806
"Pttg1",1.76913063974065e-06,0.179633936010226,0.445,0.265,0.031727588
8931088,0.619839230680455
"D430042O09Rik",1.76953957923694e-06,0.140413895997596,0.161,0.066,0.0
317349228140352,0.484475336277677
"Crebzf",1.77047053673095e-06,0.171007259611255,0.174,0.074,0.03175161
86057328,0.589942823656081
"Fam45a",1.77254040344811e-06,0.171604314932414,0.168,0.07,0.031788739
5954384,0.591802045661473
"Nipal1",1.77507192767963e-06,0.101680215397074,0.052,0.011,0.03183413
99510065,0.350513663540459
"Zfp407",1.78318599002823e-06,0.150615134040112,0.103,0.033,0.03197965
75451662,0.518515986457119
"Mta2",1.78713234513336e-06,0.153400738408628,0.187,0.083,0.0320504314
776216,0.527766747166291
"Casp8ap2",1.79401753714218e-06,0.173304777273305,0.148,0.059,0.032173
9105111078,0.595579091414725
"Dnajc10",1.79758526509471e-06,0.15511950550241,0.239,0.115,0.03223789
41442084,0.532775420759727
"Fyttd1",1.8061794927346e-06,0.200611452500753,0.29,0.154,0.0323920230
227023,0.688065804034225
"Maea",1.81043387288833e-06,0.16582161752318,0.29,0.153,0.032468321076
3794,0.568352002359227
"Eml3",1.81325193041753e-06,0.0818365793406122,0.09,0.027,0.0325188601
201079,0.280366805254741
"Cdk17",1.85329951131114e-06,0.105702353879732,0.103,0.033,0.033237073

435854,0.359820257779645
"Vezt",1.85897092399157e-06,0.151921599504431,0.161,0.066,0.0333387845
508648,0.516690503753089
"Ccdc9",1.86634790992814e-06,0.140076432507071,0.155,0.062,0.033471083
4166513,0.475849922066158
"Fcgr1",1.87057124032085e-06,0.0979948329891955,0.103,0.033,0.03354682
46239142,0.332674139444625
"Stk25",1.88055755643208e-06,0.246134210409095,0.303,0.164,0.033725919
217053,0.834269105422692
"Pmm2",1.88330983536023e-06,0.157704974932844,0.232,0.111,0.0337752785
873503,0.534308578559183
"Rc3h1",1.89327194610396e-06,0.176497581747681,0.206,0.096,0.033953939
0814285,0.597047266270045
"Eif2ak3",1.90285493246724e-06,0.134246960746457,0.142,0.054,0.0341258
003588674,0.453446170351188
"S100pbp",1.91127627241364e-06,0.119405304136667,0.135,0.05,0.03427682
86694661,0.4027882051981
"Ncoa5",1.9279977120343e-06,0.101035885019059,0.123,0.043,0.0345767109
676231,0.339942804448872
"Rnase4",1.92950374298388e-06,0.14278305116565,0.103,0.033,0.034603720
1266728,0.480292782890179
"Celf2",1.93214873978832e-06,0.167471955886465,0.123,0.044,0.034651155
4993637,0.563111758325151
"Gas1",1.94187944904897e-06,0.379365611118895,0.161,0.067,0.0348256660
392442,1.27368234335661
"Atf4",1.96288244972293e-06,0.200549156852392,0.303,0.162,0.0352023338
533311,0.671166411063717
"Gbas",1.97628235744284e-06,0.217745034189676,0.31,0.169,0.03544264779
838,0.727233453898327
"Tmem150c",1.98105678351695e-06,0.0891452868202949,0.077,0.021,0.03552
8272355593,0.297515842145519
"Jrkl",1.9848784295743e-06,0.133427030628844,0.129,0.047,0.03559680975
59855,0.445045761757803
"Tiprl",1.99036772413324e-06,0.208247910125713,0.258,0.132,0.035695254
7646055,0.694035623285593
"Slc12a3",2.00264572373285e-06,0.374238876899771,0.213,0.097,0.0359154
484094249,1.24493846655106
"Cyfip2",2.00747560250139e-06,0.238716403117771,0.406,0.238,0.03600206
745526,0.793536033398419
"Sptan1",2.02746304449036e-06,0.253685493874406,0.4,0.245,0.0363605222
398901,0.84078263953787
"Slc16a1",2.03178932945787e-06,-0.329272538976584,0.013,0.149,0.036438
1098344975,-1.09059677311313
"Zfp952",2.03205306497473e-06,0.100702994233339,0.103,0.033,0.03644283
96672568,0.333529352371715
"Mid1ip1",2.04094603005945e-06,0.375296555736994,0.232,0.115,0.0366023
261030861,1.24134720785112
"Gpr137b",2.04723853314656e-06,-0.397260598974151,0.09,0.255,0.0367151
758534504,-1.31277351033967
"Tusc3",2.05062726797499e-06,0.171443357946129,0.297,0.156,0.036775949

4238635,0.566262186393402
"Ppp2r1a",2.05873266567186e-06,0.213676784268431,0.374,0.219,0.0369213116261591,0.704912519755005
"Rnaseh2a",2.07568566072008e-06,0.128477838606055,0.142,0.054,0.0372253466393539,0.422790424467835
"Riok1",2.1028303672199e-06,0.0805450449727025,0.103,0.033,0.0377121598057217,0.264008349150625
"Rabgap1",2.12818356161342e-06,0.204416798251693,0.226,0.11,0.038166843993975,0.667581946871295
"Vps72",2.12836400951342e-06,0.141712830716727,0.226,0.107,0.0381700801466136,0.462792060695269
"Dab2ip",2.13459547131668e-06,0.126709831649963,0.187,0.082,0.0382818351825934,0.413426275686588
"Zdhhc20",2.15529799886838e-06,0.143468439484527,0.2,0.091,0.0386531143117055,0.466721187549037
"Pls3",2.17971840965644e-06,0.200190589328244,0.232,0.113,0.0390910699587787,0.648990109729041
"Plaur",2.19152122863126e-06,0.150183460440947,0.084,0.024,0.039302741714273,0.486062912384702
"Mrpl23",2.19308297474896e-06,0.184872103051894,0.542,0.349,0.0393307500691478,0.598199652613115
"Ddx55",2.1948307877737e-06,0.159082776853683,0.161,0.066,0.0393620953479335,0.514625142813249
"G3bp2",2.19589527008895e-06,0.167751594794369,0.406,0.238,0.0393811857737753,0.542587013522762
"Rps27",2.23452712066106e-06,0.28785346606827,0.987,0.939,0.0400740093819354,0.926032458711121
"Armcx2",2.25829816466672e-06,0.133834358669945,0.097,0.031,0.040500319285133,0.429132566572166
"Tgfbi",2.26320363666641e-06,0.123160272204562,0.123,0.044,0.0405882940199754,0.394639452476238
"Med10",2.27176919493828e-06,0.201877279056775,0.284,0.151,0.040741908742023,0.646107831397195
"Fads3",2.27594243544512e-06,-0.35593004778898,0.026,0.166,0.0408167516372728,-1.13850016855871
"Nfatc2",2.28868071912463e-06,0.0901092657405237,0.077,0.021,0.0410452000167811,0.287726218609129
"Lbp",2.29072133673315e-06,0.093809206227857,0.084,0.024,0.0410817964529722,0.299456825439458
"Fbxl20",2.30118743845115e-06,0.171984062930011,0.161,0.067,0.0412694955211829,0.548221843865835
"Tpbg",2.31095435511505e-06,0.0853803819376334,0.071,0.018,0.0414446554046334,0.271799595922633
"Hagh",2.31529640262786e-06,-0.447079939289526,0.413,0.539,0.041522525684728,-1.42239341588903
"Mgl2",2.32158109145745e-06,0.126384494761956,0.129,0.048,0.0416352352941979,0.401752102167462
"Eftud2",2.33035041835265e-06,0.161707868287702,0.194,0.086,0.0417925044027364,0.513428671350284
"Cav1",2.33974879630544e-06,0.192328382610057,0.142,0.054,0.0419610549

129417,0.609875869773488
"Acer2",2.34360283110062e-06,0.128068980060234,0.103,0.034,0.042030173
1729586,0.405897664409846
"Rassf10",2.36477170643905e-06,0.0604781521584027,0.052,0.011,0.042409
8157832778,0.19113366690501
"Trim30a",2.3736691917134e-06,0.122669865238476,0.097,0.03,0.042569383
2841881,0.387222148334167
"Psip1",2.4018351844563e-06,0.151488009905137,0.219,0.104,0.0430745121
980392,0.476403102162511
"Lzts2",2.42442797731159e-06,0.158155501953914,0.219,0.104,0.043479691
345106,0.49589045821519
"H2afj",2.42943315263861e-06,-0.354972679233214,0.748,0.757,0.04356945
41594208,-1.11227102636248
"Ethe1",2.43087308684961e-06,-0.489789029122852,0.368,0.505,0.04359527
79395609,-1.53441422400689
"Sec61a2",2.44431582068131e-06,0.151241988300584,0.187,0.083,0.0438363
599280986,0.472977810261451
"Tor1aip1",2.44437173362324e-06,0.204800198882782,0.342,0.195,0.043837
3626707992,0.640465271423426
"Limch1",2.46002955638668e-06,0.208553006281284,0.271,0.139,0.04411817
00642387,0.650869649064906
"Acrbp",2.461402284854e-06,0.136591833885574,0.084,0.024,0.04414278857
65716,0.426211010287359
"Tmem106a",2.46455893026298e-06,-0.343186736097551,0.045,0.196,0.04419
93998553362,-1.07041455341535
"Klhdc7a",2.49024117232072e-06,0.231445860559719,0.277,0.147,0.0446599
851843997,0.719490507785765
"Fam53b",2.51531921304699e-06,0.175597762487539,0.2,0.092,0.0451097347
667847,0.544117272313898
"Higd2a",2.54067988027748e-06,-0.39904846813943,0.665,0.699,0.04556455
29728963,-1.2325111594409
"Sec23b",2.57041906660011e-06,0.177580992289503,0.284,0.15,0.046097895
5404063,0.546414578734269
"Ddx1",2.57641234510858e-06,0.147491450331904,0.31,0.166,0.04620537899
71773,0.453485923911841
"Unc119",2.5806225650942e-06,0.0995139627233229,0.103,0.034,0.04628088
50823994,0.305809019921949
"Mrps36",2.58611450715177e-06,-0.420950962431916,0.413,0.563,0.0463793
775712598,-1.29269846520718
"Wdr45b",2.59006158003723e-06,0.148432867040735,0.232,0.112,0.04645016
43763876,0.455596172084378
"Lca5",2.60231834471581e-06,0.0725088593619936,0.058,0.013,0.046669977
1941333,0.222214580938012
"Hspa8",2.6036188190251e-06,-0.265132082904662,0.942,0.938,0.046693299
9003961,-0.812405690295549
"Rab11b",2.62485094460409e-06,0.17936762069968,0.426,0.259,0.047074076
8405298,0.548153334811875
"Rps6ka6",2.63512957045246e-06,0.135871447896125,0.097,0.031,0.0472584
137164945,0.414696584868487
"Actr2",2.64074239526417e-06,0.221576941411494,0.471,0.302,0.047359074

1166676,0.675808970789319
"Cebpg",2.64135247927742e-06,0.16838563539819,0.258,0.131,0.0473700153
633613,0.513536758400128
"Adipor1",2.64521029261192e-06,0.201439477302423,0.51,0.322,0.04743920
13877021,0.614049239531828
"Lsg1",2.65320917097418e-06,0.203742040457289,0.161,0.068,0.0475826532
72251,0.620452989443631
"Flot2",2.69556742396722e-06,0.167894061913741,0.219,0.104,0.048342306
1814281,0.508626363206423
"Rrp9",2.70196997516202e-06,0.115945458266378,0.116,0.04,0.04845712953
45556,0.350975690728519
"Ndufa5",2.72394741830679e-06,-0.304009295461061,0.794,0.832,0.0488512
72999914,-0.917796414569732
"Rbbp8",2.73093080373121e-06,0.176510588947857,0.206,0.096,0.048976513
0341156,0.532429086070821
"Txlna",2.76492135805732e-06,0.180786411689044,0.174,0.075,0.049586099
6354,0.54309046797973
"Mbtps1",2.78335002835102e-06,0.203555771141421,0.232,0.115,0.04991659
94084473,0.610138409999247
"Srek1ip1",2.80416970636217e-06,0.206624660456896,0.323,0.178,0.050289
9795138991,0.617797287029367
"Pvrl4",2.81086815261935e-06,0.105070593455204,0.084,0.025,0.050410109
4490754,0.313905074102681
"Suclg2",2.81378556694562e-06,-0.416380620166828,0.419,0.547,0.0504624
303576028,-1.24353162001083
"Per1",2.88037041747685e-06,0.0902775359759392,0.077,0.021,0.051656563
0670298,0.267504799508756
"Ube2z",2.88262646814388e-06,0.183100110696909,0.226,0.11,0.0516970230
796923,0.542407543032948
"Fam192a",2.88329487714336e-06,0.165527525767051,0.219,0.105,0.0517090
103266891,0.490312929555845
"Mospd2",2.8979486161444e-06,0.184265133649757,0.206,0.097,0.051971810
4819337,0.544881916155948
"Trps1",2.89968591251329e-06,0.156667721229881,0.342,0.187,0.052002967
1550133,0.463180989647155
"Lncpint",2.90310851303218e-06,0.124206033351162,0.103,0.034,0.0520643
480727191,0.367062968132284
"Mrpl19",2.92827444763269e-06,0.148515180132636,0.232,0.113,0.05251567
39438447,0.437621305431394
"Isoc2a",2.93245449724135e-06,-0.299805437110744,0.013,0.145,0.0525906
389535263,-0.882992112565241
"Pck2",2.94504529130008e-06,0.134877245052389,0.135,0.052,0.0528164422
541757,0.396664904563144
"9930021J03Rik",2.96916859835249e-06,0.147822566696251,0.174,0.076,0.0
532490696428536,0.43353032006623
"Sptbn2",2.99817586426167e-06,0.179941516501738,0.161,0.067,0.05376928
59496688,0.525978565876334
"Lrif1",3.00765254759972e-06,0.193132303058857,0.213,0.101,0.053939240
7886534,0.563926439349728
"Ramp3",3.01648844962862e-06,0.155491974391998,0.148,0.058,0.054097703

8556396,0.453564419539266
"H1f0",3.02477432184085e-06,0.423029107692561,0.51,0.345,0.05424630268
78937,1.23280007324211
"Ranbp1",3.0435475862949e-06,0.236364966081214,0.516,0.346,0.054582982
4126127,0.687357150300702
"Fmo2",3.05068321789151e-06,-0.272839341147825,0.071,0.235,0.054710952
8296664,-0.792786914891445
"Myc",3.05820271416788e-06,0.109728892014326,0.077,0.022,0.05484580747
58868,0.318568159738934
"Plekha7",3.10219724196001e-06,0.181504324746415,0.206,0.097,0.0556348
053373107,0.524356243472154
"Uspl1",3.12046421005983e-06,0.138656225761282,0.135,0.052,0.055962405
143213,0.399756318943756
"Fam124a",3.13808703868049e-06,0.109040251799049,0.084,0.025,0.0562784
529516959,0.313757167652199
"Itga1",3.15779024285134e-06,0.177835799158421,0.168,0.071,0.056631810
2152959,0.510599378299654
"Fbxo6",3.16734159612824e-06,0.250461982440514,0.232,0.117,0.056803104
1849638,0.718366117445929
"Slc12a2",3.17316768930249e-06,0.170804934982274,0.206,0.095,0.0569075
893399509,0.489582722816823
"Suv420h1",3.204336558179e-06,0.229121637232387,0.226,0.112,0.05746657
18343822,0.654497838918969
"Hadha",3.21880446689441e-06,0.175182579445521,0.432,0.262,0.057726039
3092844,0.499628936042236
"Rps15",3.23012468882569e-06,0.233733068253026,0.961,0.87,0.0579290561
693999,0.665797102926409
"Mcc",3.24110245779544e-06,0.0871614015379202,0.058,0.013,0.0581259314
781035,0.247986685428989
"Srpk2",3.25495857721636e-06,0.162888102450246,0.277,0.146,0.058374427
1237983,0.462745125195298
"Lpcat4",3.25607516309823e-06,0.0770624999051378,0.071,0.019,0.0583944
519750037,0.218898681475009
"Pbld2",3.27172234280349e-06,-0.380436757653016,0.103,0.266,0.05867506
84958378,-1.07881987185867
"Dysf",3.28826710373125e-06,0.113198263067003,0.097,0.031,0.0589717822
383163,0.320429894992833
"Lcp1",3.3198107750296e-06,0.269838564647026,0.187,0.084,0.05953748643
93809,0.761254834760416
"Naa15",3.35137080732396e-06,0.148186585959614,0.303,0.162,0.060103484
058548,0.416654366682746
"Myo3b",3.35714128467317e-06,0.0750604195936038,0.058,0.013,0.06020697
17993286,0.210917311278058
"Bdp1",3.38799856697137e-06,0.229103013037891,0.206,0.099,0.0607603663
000646,0.641675744860016
"Tmprss2",3.3936876291313e-06,0.205738805381895,0.335,0.185,0.06086239
39408408,0.575891678564877
"Surf1",3.40407328357381e-06,0.162117842586545,0.271,0.141,0.061048650
2676127,0.453295136136798
"Btbd7",3.48198887030123e-06,0.138537875967146,0.116,0.042,0.062445988

3999823,0.384228326708094
"Hotairm1",3.49134261513819e-06,0.136797479164121,0.2,0.091,0.06261373
84598882,0.379034428051302
"Hebp2",3.49401803575059e-06,0.141638931416803,0.129,0.049,0.062661719
453151,0.392340484882965
"Foxq1",3.49572421188638e-06,0.23254351540715,0.174,0.075,0.0626923180
159703,0.644033069990387
"Prmt10",3.50130160412302e-06,0.0913804795136449,0.097,0.031,0.0627923
429683423,0.252934052427674
"Lilr4b",3.51440180672244e-06,0.0858065445794678,0.052,0.011,0.0630272
820017602,0.237185386412292
"Slc51b",3.52377807586635e-06,-0.353449217540394,0.019,0.153,0.0631954
360125871,-0.976058212643557
"Dpy19l3",3.52676160972302e-06,0.119766176548717,0.084,0.025,0.0632489
427087727,0.330635713116672
"Lsm5",3.53482357533485e-06,0.201557804245702,0.348,0.199,0.0633935260
000552,0.555975744425532
"Slc25a10",3.54531625618125e-06,-0.45246798890283,0.161,0.323,0.063581
7017383545,-1.24674367091987
"Kifc3",3.55101185116784e-06,0.236874851849827,0.226,0.112,0.063683846
538844,0.652311731392496
"Ikbkb",3.55282457503526e-06,0.213174433615567,0.258,0.136,0.063716355
9286824,0.586936149188381
"Erp29",3.55541396114721e-06,0.21252719354829,0.542,0.358,0.0637627939
79214,0.584999255421616
"Ccdc82",3.5642373184449e-06,0.0772152953394929,0.065,0.016,0.06392103
20689907,0.212350311201218
"Vasn",3.56569243684233e-06,0.149744488664592,0.123,0.045,0.0639471281
623303,0.41175221975053
"Mms19",3.5754161069745e-06,0.134704493732048,0.135,0.052,0.0641215124
624806,0.3700299256374
"Tradd",3.58011599867715e-06,0.114776430448399,0.168,0.071,0.064205800
320276,0.315137251979802
"Lad1",3.5847572615063e-06,0.231091592356816,0.361,0.212,0.06428903672
7854,0.634199946889434
"Smlr1",3.6127147224003e-06,-0.303892770543657,0,0.123,0.0647904258315
27,-0.831632176704939
"Taok1",3.71187041605618e-06,0.240535279504676,0.329,0.19,0.0665686840
415516,0.651735396255626
"Etfa",3.72863889050252e-06,-0.433837955659395,0.432,0.563,0.066869409
8622722,-1.17353759980114
"Stam2",3.72982449007146e-06,0.154877007362467,0.194,0.089,0.066890672
4049416,0.418895183199372
"Cds2",3.75197569093821e-06,0.232812262193894,0.226,0.112,0.0672879320
412858,0.628307767229442
"Iscu",3.78770906228127e-06,-0.404429050034628,0.529,0.627,0.067928774
3229523,-1.08762924826074
"Rps6kb1",3.78834606259103e-06,0.140585969009873,0.252,0.128,0.0679401
982865074,0.378053580996752
"Rpl35",3.81658666731673e-06,0.232555754295118,0.942,0.879,0.068446665

2916582,0.623644869982429
"Dalrd3",3.84299981493639e-06,0.144161129687139,0.329,0.18,0.068920358
6810693,0.385602717753068
"Smc3",3.84311249654003e-06,0.180091513153952,0.265,0.137,0.0689223795
129488,0.481704158631026
"Fbxw17",3.85285760160426e-06,0.171077802243263,0.174,0.076,0.06909714
82271708,0.457161257506541
"Hectd1",3.85439968028415e-06,0.175698577269224,0.406,0.242,0.06912480
38662159,0.469438777739578
"Dennd5a",3.85553978096804e-06,0.1749798835346,0.2,0.093,0.06914525043
18808,0.467466791880408
"Clec2h",3.86294703337992e-06,-0.401013124232589,0.026,0.161,0.0692780
920966355,-1.07055528500515
"4930426D05Rik",3.87013815516494e-06,0.11103107412357,0.09,0.028,0.069
407057674728,0.296205004548049
"Aldh4a1",3.87390155631147e-06,-0.466035193504026,0.194,0.345,0.069474
5505108899,-1.24282021815026
"Syap1",3.87918052285649e-06,0.206937224280616,0.297,0.164,0.069569223
4969084,0.551577306644628
"Armc9",3.88818927587329e-06,0.121113796146328,0.11,0.038,0.0697307864
735115,0.322539768473201
"Ccl17",3.92111432445275e-06,0.12572180919405,0.129,0.049,0.0703212642
947356,0.333751304012201
"Lamb2",3.93015391077767e-06,0.136635001024538,0.155,0.064,0.070483380
2358868,0.362407716969745
"Nrd1",3.96450200304857e-06,0.221960361546352,0.335,0.193,0.0710993789
226731,0.586791431144206
"Trp53bp1",3.97352318178658e-06,0.168634353599763,0.148,0.06,0.0712611
647421604,0.445431418020022
"Hipk2",3.9846863820524e-06,0.242532323162196,0.258,0.134,0.0714613655
757278,0.639945379693867
"Nudt22",4.01189606991439e-06,0.176853142028402,0.213,0.102,0.07194934
41178446,0.465440854408643
"Zcchc9",4.03588573007638e-06,0.197821313935581,0.271,0.144,0.07237957
46831899,0.519445365699966
"Atf6b",4.08213281528477e-06,0.157153732607409,0.187,0.084,0.073208969
9093171,0.410868584414064
"Nfkb2",4.09663020072156e-06,0.181974295105113,0.135,0.053,0.073468966
0197404,0.475115266407864
"Hnrnpf",4.1458653061866e-06,0.220338788675045,0.697,0.478,0.074351948
4011505,0.572648481405281
"Ist1",4.15003699594828e-06,0.194649875413636,0.29,0.158,0.07442676348
53365,0.505688634569796
"Pcdhga12",4.16590977105442e-06,0.0908455751589653,0.065,0.016,0.07471
14258340899,0.235664527130805
"Rhbdf2",4.16643287087966e-06,0.0938979048733784,0.084,0.025,0.0747208
071063558,0.243570853817433
"Slc23a1",4.16936528356819e-06,-0.278526685842593,0.019,0.154,0.074773
3969955119,-0.72230133601581
"Glis3",4.18041286896446e-06,0.135541747278668,0.252,0.126,0.074971524

3920087,0.351140809077092
"Taf3",4.20852141754841e-06,0.125391502404181,0.168,0.072,0.0754756231023132,0.324004814354045
"Cep290",4.24255179814489e-06,0.202876939828404,0.174,0.077,0.0760859239479305,0.522589086096925
"Prdm11",4.24477590840152e-06,0.0980662152126826,0.058,0.014,0.0761258111412729,0.252556582500098
"Pde4d",4.26225107998577e-06,0.115225308766517,0.265,0.133,0.0764392108684649,0.296274167871149
"Car11",4.27174280768566e-06,0.063804479593029,0.052,0.011,0.0766094355130347,0.163915943196466
"Pfdn5",4.27828163518821e-06,0.224815847813637,0.897,0.745,0.0767267028454654,0.577215922282388
"Smim4",4.28028404930446e-06,-0.440956267672935,0.284,0.436,0.0767626141402261,-1.13195129808294
"Calml3",4.31717994709367e-06,0.0893189959071676,0.097,0.031,0.0774243051711778,0.228518589459823
"Myo5a",4.33177696091292e-06,0.0807190129029914,0.084,0.025,0.0776860880170124,0.20624346163347
"Ikzf2",4.34771821391451e-06,0.192348089578768,0.161,0.069,0.0779719784483427,0.490758025119028
"Elf2",4.37081178421972e-06,0.19688229947093,0.174,0.078,0.0783861385381965,0.501283631529728
"Sae1",4.39376319152454e-06,0.152976967676435,0.206,0.097,0.0787977490768011,0.388694717514353
"Glyctk",4.39451288302986e-06,-0.289171696676187,0.013,0.144,0.0788111940442575,-0.734698597934232
"Xndc1",4.4099297849022e-06,0.127550801797381,0.103,0.034,0.079087680762436,0.323621659487957
"Nop56",4.42564030971774e-06,0.144485499816151,0.206,0.097,0.0793694333144779,0.366074524324449
"Tmem87a",4.4376708623091e-06,0.110401861384735,0.11,0.038,0.0795851892446514,0.279419081439731
"Manbal",4.45603735306303e-06,0.16746296482601,0.194,0.09,0.0799145738898324,0.423144924035977
"Arsb",4.48401591934708e-06,-0.397089032593711,0.097,0.251,0.0804163414975706,-1.00087794480836
"Rap2a",4.51742746653084e-06,0.106420559283353,0.123,0.045,0.081015544184764,0.267447022879177
"Rps23",4.53423522552893e-06,0.260487877115766,0.948,0.924,0.0813169745346358,0.653668407869011
"Zyx",4.54600998230993e-06,0.20215907803928,0.206,0.099,0.0815281430227463,0.506773792989529
"Zcchc10",4.55528283207574e-06,0.130138711422595,0.135,0.052,0.0816944423104463,0.325967449764344
"Smg6",4.56161412654868e-06,0.174503196381426,0.226,0.11,0.0818079877455241,0.436847879890376
"Atxn2",4.59666138748924e-06,0.188889114879795,0.284,0.153,0.0824365253232321,0.471415602017319
"Naa20",4.60089991558852e-06,0.180420501528354,0.381,0.223,0.082512539

0861645,0.450113970807061
"Zmat2",4.61503592502992e-06,0.167390235055949,0.471,0.295,0.082766054
2794865,0.41709248801305
"Gtf2f2",4.65385871476482e-06,0.152267155327296,0.168,0.073,0.08346230
21905924,0.378134196328049
"Rbpms2",4.73652760505747e-06,-0.366224291452179,0.065,0.209,0.0849448
860691007,-0.903018510543417
"Noct",4.81228436255682e-06,0.138908180424142,0.11,0.038,0.08630350775
80941,0.34030907254668
"Ostc",4.81643586395481e-06,0.213532348687382,0.381,0.231,0.0863779607
841656,0.522945573225528
"Scyl2",4.828987575869e-06,0.149686720712814,0.187,0.085,0.08660306318
56347,0.366196601114408
"Hbb-
bs",4.84510208659734e-06,-1.78718081724469,0.297,0.163,0.0868920608210
367,-4.36624110009792
"Phlda3",4.86245083464988e-06,0.111773795702521,0.084,0.025,0.08720319
3268611,0.27267377618779
"Parp4",4.87293391302778e-06,0.150081873329124,0.155,0.065,0.087391196
7962402,0.365803663519102
"Fbxo4",4.88505924118728e-06,0.111989718335796,0.148,0.06,0.0876086524
314527,0.272681022980902
"Ppp1r18",4.91392674547577e-06,0.125425116088662,0.077,0.022,0.0881263
622533624,0.304655544962231
"Gem",4.91428228470885e-06,0.147226947249744,0.071,0.019,0.08813273849
39686,0.357601182442859
"Pdcl3",4.96023383856211e-06,0.166812614710775,0.316,0.177,0.088956833
6607729,0.40362047680333
"Tnrc18",5.02950073300875e-06,0.165710076935451,0.219,0.108,0.09019906
6145779,0.39865473163653
"Hsp90b1",5.04071604780729e-06,0.299950468173261,0.787,0.587,0.0904002
016013759,0.720933584264304
"2010111I01Rik",5.09373999976477e-06,0.19751142649485,0.258,0.134,0.09
13511331557814,0.4726536509692
"Tmcc3",5.11971958252512e-06,0.170380480047787,0.2,0.094,0.09181705099
30055,0.406861303941212
"Ddx50",5.13747923377028e-06,0.14340728910344,0.252,0.128,0.0921355525
784362,0.341953876169647
"Gm5914",5.14334867101156e-06,0.0855183208650115,0.077,0.022,0.0922408
150659214,0.203820309533462
"Cracr2b",5.15086622996445e-06,0.0963961994333714,0.084,0.025,0.092375
6349681824,0.229605338760071
"Ddc",5.17156857365541e-06,-0.325711739895716,0.026,0.16,0.09274691079
99361,-0.774503720249229
"Bet1l",5.20059236247777e-06,0.138418008607801,0.2,0.093,0.09326742342
86763,0.328366881321651
"Dynlt3",5.20512092185848e-06,0.144613931181126,0.458,0.281,0.09334863
861261,0.342939499955289
"Atxn7",5.21087315186724e-06,0.181509542714139,0.226,0.111,0.093451799
105587,0.430233792201656

"Fabp4",5.23630517820088e-06,0.127893408332177,0.187,0.084,0.093907897
0658545,0.302524285544007
"Baz1b",5.25479867278137e-06,0.199485986598207,0.239,0.123,0.094239559
3976612,0.471168990797959
"Trmt2a",5.27339314321915e-06,0.143765291373885,0.135,0.053,0.09457303
26304923,0.339053606355964
"Apob",5.28010722526153e-06,-0.39917116884055,0.013,0.139,0.0946934429
778403,-0.940890561804075
"Prpf39",5.30629387179264e-06,0.135321362003868,0.148,0.061,0.09516307
42967292,0.318297939714875
"Lsm1",5.33862820329974e-06,0.185121188300892,0.265,0.14,0.09574295819
79776,0.434310634933636
"Slc16a4",5.34153255701448e-06,-0.351102475834456,0.013,0.139,0.095795
0448774977,-0.823526417566288
"Tceanc2",5.39047550381657e-06,0.109121916474971,0.135,0.053,0.0966727
876854464,0.254954991007082
"Cntrl",5.40386276705804e-06,0.105217668984449,0.123,0.045,0.096912874
8644189,0.245572031625338
"Fam193a",5.43270301152064e-06,0.186790368227637,0.194,0.092,0.0974300
958086112,0.434963810459166
"Syt11",5.45143809351657e-06,0.1145470870395,0.142,0.056,0.09776609076
91262,0.266342307437541
"Nrf1",5.4757089545818e-06,0.153479912583002,0.129,0.049,0.09820136439
147,0.356186230975605
"Gpr108",5.49865440040844e-06,0.131680785999911,0.194,0.089,0.09861286
80169249,0.305045588142455
"P3h3",5.50094504929109e-06,0.0518149526698982,0.052,0.011,0.098653948
5139865,0.120010530163152
"Ifitm1",5.50119295193912e-06,0.0915280012367572,0.123,0.045,0.0986583
944000763,0.21198726753959
"Kdm6b",5.53880555803967e-06,0.205460294252478,0.226,0.111,0.099332938
8778835,0.474464948111846
"Zc3h6",5.54096573764024e-06,0.104204825579452,0.11,0.038,0.0993716795
3884,0.240597283828269
"Srp72",5.58932451881671e-06,0.209647253981129,0.335,0.194,0.100238945
920459,0.482230295783768
"Faf2",5.63373598571295e-06,0.202135778100829,0.232,0.119,0.1010354211
67776,0.463352634020606
"Rpl6",5.69848417047964e-06,0.230371657995291,0.961,0.86,0.10219661511
3382,0.525444744692774
"Met",5.72922300522272e-06,0.269255891335032,0.413,0.256,0.10274788537
5664,0.612685589490654
"Iyd",5.73185886494177e-06,-0.342036590784785,0.026,0.156,0.1027951568
83866,-0.778139072029532
"Mtf2",5.73533092754272e-06,0.166772716176742,0.181,0.082,0.1028574248
54551,0.379309779287509
"Borcs7",5.76224149299518e-06,0.139819745068313,0.239,0.12,0.103340038
935376,0.317353123015014
"Tmem234",5.76741509284867e-06,0.20920391400474,0.548,0.362,0.10343282
2275148,0.474648730674846

"Csgalnact2",5.79216912161939e-06,0.105179042593748,0.11,0.038,0.103876761027122,0.238183208543831
"Clstn1",5.82972267764122e-06,0.185121084796451,0.277,0.15,0.104550246500818,0.418019606709838
"Phf7",5.85106457848584e-06,0.07493165625441 8,0.071,0.019,0.104932992150565,0.168928421274168
"Sik1",5.88471255215591e-06,0.164990049391722,0.11,0.039,0.105536434910364,0.371012964206093
"Flnb",5.96309526359773e-06,0.198771689201837,0.303,0.169,0.106942150457362,0.444347599580423
"Gm26782",5.97301993304247e-06,0.130247586192592,0.135,0.053,0.107120139479184,0.290947614912725
"Cyth4",5.98836567747315e-06,0.144875999681225,0.11,0.039,0.107395350059803,0.323252892767654
"Rmnd5b",5.99000791222171e-06,0.14397407336867,0.168,0.073,0.107424801897784,0.321201002466422
"Itpripl2",6.088388091899e-06,0.143899054749529,0.097,0.032,0.109189152040117,0.318689431991977
"Usp42",6.11048007845252e-06,0.131400743672384,0.103,0.036,0.109585349726968,0.290533822406337
"Id1",6.16413136826771e-06,0.361003599396383,0.148,0.062,0.110547531958513,0.795041727618118
"Cxcl2",6.16693098581297e-06,0.528609680661458,0.168,0.074,0.11059774029957,1.16392219688583
"Ugt2b37",6.21642555372725e-06,-0.507795029762004,0.032,0.166,0.111485375880544,-1.11403214580413
"Scn2b",6.24563294352551e-06,0.0640861508273671,0.052,0.011,0.112009181209187,0.140295763090656
"Rpl35a",6.24627047665941e-06,0.207163084185157,0.974,0.929,0.11202061472841,0.453494982544452
"Smyd2",6.27352111010445e-06,0.113553086166223,0.116,0.042,0.112509327588613,0.248081601761301
"Pla1a",6.28171875773194e-06,0.0908727423577389,0.097,0.031,0.112656344201165,0.198412753897041
"Rnf121",6.28339813992764e-06,0.15823773236023,0.181,0.082,0.112686462241462,0.345456070431042
"Zfp322a",6.31940326475026e-06,0.134774732247567,0.213,0.102,0.113332178150031,0.293462834044684
"Ifnar1",6.34342271876333e-06,0.154091910984882,0.181,0.082,0.113762943038302,0.33494010142153
"Trim16",6.36731628169187e-06,0.0691337749360999,0.065,0.017,0.114191450195862,0.150011916158156
"A230050P20Rik",6.38222605861266e-06,0.0930252642611711,0.09,0.028,0.114458842135159,0.201635979244798
"Gabpb2",6.39293020456579e-06,0.188590200188083,0.155,0.066,0.114650810288683,0.40846076347402
"Tmed8",6.41345212307877e-06,0.0996775066405809,0.11,0.038,0.115018850375295,0.215568481528395
"Spata7",6.41371159514473e-06,0.131810794899013,0.116,0.042,0.115023503747326,0.285056501917141

"2810405F17Rik",6.44066548596589e-06,0.082302170694787,0.058,0.014,0.1
15506894825312,0.177643067336888
"Atpaf2",6.44846935931317e-06,0.118340020722194,0.135,0.053,0.11564684
9489922,0.255284765135203
"Zfyve9",6.47802420497159e-06,0.127210153868321,0.187,0.084,0.11617688
6091961,0.273837839802015
"Cul7",6.47851302978855e-06,0.13056160398166,0.129,0.05,0.116185652676
228,0.281042458294783
"Tirap",6.48347463963106e-06,0.102767721339667,0.103,0.035,0.116274634
187143,0.221135618745097
"Zmym2",6.51513849379106e-06,0.149217773425431,0.174,0.077,0.116842493
747649,0.320359884269202
"Gm12927",6.5386392203031e-06,0.0516406209676733,0.071,0.019,0.1172639
55776916,0.110682781262246
"Rpa2",6.57212918575809e-06,0.088881456517422,0.097,0.032,0.1178645648
17386,0.190048025248482
"Pds5a",6.59943947022773e-06,0.192998145055693,0.284,0.155,0.118354347
459064,0.411871977902429
"Upf2",6.60526453896496e-06,0.21394140657765,0.252,0.132,0.11845881424
1798,0.456377655824576
"Shkbp1",6.6442275840136e-06,0.148033084666124,0.135,0.053,0.119157577
4917,0.314912036511704
"Nde1",6.66925413926315e-06,0.125041101641209,0.116,0.042,0.1196064037
33545,0.265530893341856
"Yaf2",6.67293534422283e-06,0.141354517408253,0.245,0.124,0.1196724224
63292,0.300095227948434
"Emd",6.67473234749256e-06,0.181610761603656,0.206,0.099,0.11970464991
9932,0.385510216198924
"2900093K20Rik",6.73368738956067e-06,0.142428023697462,0.116,0.042,0.1
20761949644381,0.301083446046736
"Zfp62",6.74460568701197e-06,0.18192669140594,0.148,0.062,0.1209577583
90873,0.384286278772913
"D130017N08Rik",6.76949418852607e-06,0.0519373803849992,0.052,0.011,0.
121404108777026,0.109516747277297
"Ampd3",6.79612820387667e-06,0.194655797130719,0.187,0.087,0.121881763
208324,0.40969280724444
"Hip1r",6.8153954476654e-06,0.129768262263873,0.213,0.102,0.1222273019
58431,0.272756385546417
"Gmps",6.82624491487968e-06,0.179464362344855,0.271,0.145,0.1224218763
03452,0.376925805211713
"Vrk2",6.85768841975128e-06,0.0736786422056933,0.084,0.025,0.122985784
119819,0.154407336291456
"Dhrs4",6.92844958573588e-06,-0.48150481427235,0.258,0.402,0.124254814
870587,-1.00414018545461
"Itga4",6.995856661793e-06,0.0726034412204814,0.058,0.014,0.1254636933
72596,0.150705784173155
"Usp22",7.10188357167551e-06,0.177440806979612,0.194,0.092,0.127365179
974429,0.365651718357324
"Bcap29",7.12436852359655e-06,0.163852517404279,0.194,0.09,0.127768425
102181,0.337132425803395

"Serpinb8",7.14551484624638e-06,0.092591573217923,0.103,0.035,0.128147663252583,0.190236059399894
"Slc30a7",7.16914246680568e-06,0.140265957046755,0.129,0.05,0.128571400999693,0.287723473027515
"Ptpn11",7.19189944207244e-06,0.174471705238449,0.277,0.152,0.128979524594127,0.357335780624254
"Dcaf7",7.19519092506367e-06,0.135559530558079,0.206,0.098,0.129038554050092,0.27757766625852
"Cct3",7.21313999556922e-06,0.199472615226064,0.419,0.262,0.129360452680538,0.40795193054393
"Zfp398",7.22526223222515e-06,0.188091311280304,0.161,0.07,0.129577852872726,0.384359590995255
"Hdac2",7.27353482730993e-06,0.190031455460001,0.284,0.155,0.130443573592976,0.38705883001538
"Aif1",7.29099701394198e-06,0.175701280869729,0.206,0.1,0.130756740448035,0.357449606867157
"Chpf",7.31620827840504e-06,0.118218625573174,0.103,0.036,0.131208879264916,0.240097858665328
"Lrch1",7.35288162480864e-06,0.115889506118677,0.103,0.036,0.131866579059318,0.234788040647099
"Pomgnt1",7.37652626431489e-06,0.134769374300797,0.213,0.103,0.132290622024223,0.272605303686106
"Srbd1",7.37874975226868e-06,0.093768324639004,0.084,0.026,0.132330498057187,0.18964200250834
"Rasgrp3",7.39418086102412e-06,0.110027181346422,0.052,0.012,0.132607239561606,0.222294912832839
"Inppl1",7.41120398777164e-06,0.165571038425356,0.187,0.087,0.132912532316697,0.334132955211881
"Cldnd1",7.42276380583134e-06,0.175532114187477,0.277,0.151,0.133119846093779,0.353961466323227
"Unc5b",7.42337756664728e-06,0.106289840224429,0.065,0.017,0.133130853280252,0.214325254599065
"Mgp",7.55617631270891e-06,0.170283257330026,0.277,0.148,0.135512465992122,0.340343723360213
"Rpl23",7.57424762252711e-06,0.217754410555908,0.968,0.906,0.135836556862401,0.434703762096819
"Tnrc6c",7.60707119425151e-06,0.23206600309117,0.271,0.15,0.136425214797707,0.462270533164428
"Neurl3",7.65838337162421e-06,0.148062921386515,0.09,0.029,0.137345447386709,0.29394280499446
"Plxnb2",7.66930138645958e-06,0.243063519784623,0.426,0.271,0.137541251064766,0.482197042530595
"Uvssa",7.78404301589967e-06,0.186371316551154,0.181,0.082,0.139599027447145,0.366961591557152
"Smyd5",7.78888888652609e-06,0.0910199757732366,0.097,0.032,0.139685933290959,0.17915996210191
"Sbsn",7.82381843749883e-06,0.134008449690757,0.09,0.029,0.140312359858104,0.263177076991414
"Cdc5l",7.83627090732606e-06,0.165897394154111,0.271,0.144,0.140535682451986,0.325539437079739

"Bhlhe40",7.89219205846093e-06,0.352396444859196,0.277,0.154,0.1415385
72376438,0.688999539217012
"Mrpl42",7.90363961816791e-06,-0.383788360286439,0.503,0.609,0.1417438
72912223,-0.74982020016341
"Nit2",7.92731399215269e-06,-0.405966250001911,0.142,0.293,0.142168449
135266,-0.791935683583222
"Sart1",7.93548969443852e-06,0.141310971537607,0.213,0.103,0.142315072
18006,0.275515677317973
"Tmem106c",8.05257244533176e-06,0.21319492806451,0.194,0.093,0.1444148
3423458,0.412546113327066
"Gm13111",8.08941295639899e-06,0.0891771379004407,0.11,0.039,0.1450755
3196006,0.172156532680369
"Lysmd3",8.09163146819031e-06,0.100354464404131,0.123,0.046,0.14511531
8750525,0.193706851696075
"Abca13",8.11228650830096e-06,0.082395471323961,0.135,0.052,0.14548574
6239869,0.158831868253927
"Gla",8.11585959696415e-06,0.101013231525131,0.065,0.017,0.14554982601
1955,0.194676417503838
"Ep400",8.1230178009241e-06,0.152662589168167,0.206,0.1,0.145678201241
773,0.294082371110023
"Trappc4",8.17929346251019e-06,0.15419579730863,0.355,0.205,0.14668744
8956658,0.295971300984896
"Slc38a1",8.19504130176998e-06,0.104983306945876,0.09,0.029,0.14696987
0705943,0.201308396418209
"Mau2",8.19517843924065e-06,0.132606600425265,0.187,0.087,0.1469723301
29342,0.254274607075892
"Pdrg1",8.23484910946279e-06,0.15787698836012,0.284,0.153,0.1476837839
29106,0.301968455863262
"Max",8.29809173469354e-06,0.166604882233178,0.219,0.109,0.14881797716
9994,0.317387523644978
"Gars",8.37720680036356e-06,0.137835089636289,0.232,0.117,0.1502368267
5772,0.261272254532066
"Nat14",8.48957181102288e-06,0.0942472405346368,0.09,0.029,0.152251980
858884,0.177393886693584
"Pacsin3",8.4960827462869e-06,0.142674891533698,0.148,0.063,0.15236874
7971909,0.268435920442077
"5730409E04Rik",8.50105897887196e-06,0.123896506423372,0.065,0.017,0.1
5245799172709,0.233032749367208
"Bicd2",8.50459651663874e-06,0.146316601179528,0.142,0.059,0.152521433
929399,0.275141073531462
"Ccdc171",8.56877318226284e-06,0.0922674924433628,0.065,0.017,0.153672
378250702,0.172810772067332
"Orc6",8.63718051171176e-06,0.118267984282215,0.123,0.046,0.1548991952
97039,0.220567511255008
"Dync1h1",8.65568192215186e-06,0.187259955541663,0.445,0.279,0.1552309
99591871,0.348835513766566
"Rap1a",8.74919455667204e-06,0.21142346597664,0.458,0.292,0.1569080551
79356,0.391576403624982
"Mri1",8.77972838703041e-06,0.188611572808548,0.194,0.092,0.1574556488
93003,0.348669513983188

"Csnk1g1",8.78964985153753e-06,0.179497277300945,0.155,0.067,0.1576335
80437474,0.331617997911595
"1110046J04Rik",8.79823476342048e-06,0.0696865216719461,0.058,0.014,0.
157787542247183,0.128676567853666
"Smu1",8.8086216712048e-06,0.174657566524715,0.239,0.124,0.15797382105
1387,0.322300139737663
"Emc3",8.87677418314121e-06,0.144090210700662,0.335,0.191,0.1591960682
00454,0.264782866118621
"Ccdc25",8.88099808832252e-06,0.163487496409216,0.252,0.132,0.15927181
9715976,0.300349906089404
"Crebbp",8.95717057430405e-06,0.1830478863099,0.284,0.154,0.1606378970
79569,0.334721828649608
"Rhoa",8.95958924179103e-06,0.242466509334141,0.735,0.532,0.1606812734
6228,0.443309409861789
"Topors",8.99178260088764e-06,0.167420846257805,0.226,0.114,0.16125862
9164319,0.305500487806119
"Lyrm9",9.1057042827701e-06,0.143375642327817,0.2,0.094,0.163301700607
199,0.259819011150212
"Add1",9.13776776641621e-06,0.200838519222088,0.297,0.167,0.1638767271
22908,0.363244739611427
"Adipor2",9.14079304197986e-06,0.162572011709878,0.252,0.132,0.1639309
82414867,0.293980558512901
"Hoxa5",9.18321339790884e-06,0.123570492286953,0.155,0.065,0.164691749
078097,0.222881593365892
"Ptbp1",9.19162246606478e-06,0.188870582424367,0.335,0.197,0.164842557
306406,0.340489173300215
"Pdia4",9.19269881866125e-06,0.2344150812152,0.368,0.225,0.16486186061
3871,0.422567728266546
"Myh10",9.20168086954585e-06,0.235180518669659,0.187,0.088,0.165022944
714435,0.4237178628384
"Slc26a1",9.21518072674872e-06,-0.335495303061859,0.032,0.163,0.165265
051153512,-0.603960229212718
"Crim1",9.22581451383905e-06,0.198244319844031,0.297,0.165,0.165455757
491189,0.356651730324329
"Pfdn6",9.22859770528478e-06,0.148107493395853,0.51,0.324,0.1655056712
46577,0.266408326701541
"Stat6",9.26258846353495e-06,0.173107881518827,0.174,0.079,0.166115261
505036,0.310741350875299
"Napsa",9.2742122766537e-06,-0.840886951663305,0.265,0.39,0.1663237229
69507,-1.50839920206596
"Tstd1",9.33271419167694e-06,-0.309788579718888,0.006,0.124,0.16737289
6313534,-0.55375670324723
"Ankrd44",9.38855418539316e-06,0.193095073525181,0.135,0.055,0.1683743
30760841,0.344011544181827
"Asf1a",9.40191497909387e-06,0.147993629867601,0.206,0.1,0.16861394323
5069,0.263449903688163
"Sort1",9.40535087327899e-06,0.164207084148749,0.181,0.084,0.168675562
561385,0.292252181641014
"Itch",9.44160840673195e-06,0.195249770650144,0.387,0.235,0.1693258051
66331,0.346750038189403

"Polr2f",9.46966799644477e-06,0.147209843249262,0.671,0.467,0.169829025848241,0.260997616846573
"Col4a4",9.49772806456508e-06,0.289860535014169,0.503,0.336,0.17033225510991,0.513054395562489
"Dopey1",9.56807167284617e-06,0.14744320081944,0.194,0.092,0.171593797380823,0.259887106992016
"Itsn2",9.61796043551485e-06,0.204346211173415,0.206,0.101,0.172488502450523,0.359123078293941
"Fam208a",9.63228531947284e-06,0.275607503892169,0.245,0.133,0.172745404919426,0.483949252686012
"Fam110a",9.68030697647039e-06,0.127798224703347,0.097,0.033,0.17360662531602,0.223770002950378
"Il15ra",9.69074506090635e-06,0.122946763599859,0.129,0.05,0.173793821922295,0.215142772890972
"Pes1",9.73024448862944e-06,0.118924817440926,0.181,0.082,0.17450220465908,0.207621075431485
"Zc3h3",9.76294735855107e-06,0.102328522536017,0.077,0.023,0.175088697928255,0.17830362222039
"Pcgf2",9.96859604682408e-06,0.128994871698909,0.129,0.051,0.178776801503743,0.222079785980099
"Usp36",1.01505767310215e-05,0.0970119036943193,0.116,0.043,0.18204044309414,0.165262339231029
"Llph",1.01676056047296e-05,0.163517217416971,0.477,0.297,0.182345838915221,0.278281806091679
"Rpl11",1.03689215684522e-05,0.207902709505446,0.974,0.893,0.185956239408622,0.349743065887377
"Eif3g",1.03938543919199e-05,0.158146973591468,0.413,0.25,0.186403384664692,0.265661963018053
"Acaa2",1.04002636791782e-05,-0.501139842751242,0.303,0.425,0.186518328822382,-0.841526936747662
"Prkrir",1.04564523626019e-05,0.120603112831184,0.2,0.095,0.187526016670902,0.201870035501025
"Bcorl1",1.06356244263827e-05,0.0878139269699882,0.084,0.026,0.190739288462747,0.145494308657375
"Nfyb",1.06574819071601e-05,0.110070016815881,0.148,0.062,0.19113128052301,0.182143286450152
"Rpap3",1.07302188015698e-05,0.168349963249974,0.258,0.137,0.192435743987352,0.277439557357494
"Ap4m1",1.0731835613058e-05,0.104947621268854,0.129,0.051,0.192464739884582,0.172937130814171
"Gnpnat1",1.07435461210872e-05,0.170103518867665,0.31,0.175,0.192674756135578,0.280118261082049
"Map3k12",1.07741439777937e-05,0.0616013477255066,0.071,0.02,0.193223498097752,0.101266932248612
"Rtp4",1.0806149434837e-05,0.124043880346968,0.116,0.043,0.193797483964367,0.203548758804076
"Bag5",1.08263540608591e-05,0.110565311426482,0.155,0.066,0.194159833727447,0.181224680152806
"Slc10a7",1.09180810090028e-05,0.0892688999011436,0.071,0.02,0.195804864815456,0.14556514464688

"Saraf",1.09679694973721e-05,0.176022251881428,0.465,0.297,0.196699564
965871,0.286225869974093
"Gnaq",1.09751597338311e-05,0.137537130756241,0.245,0.125,0.1968285146
66527,0.223555934986879
"Lrrc8c",1.10559826145359e-05,0.0741092645364071,0.052,0.012,0.1982779
92209087,0.119915106494778
"Prmt5",1.10695515220908e-05,0.12938505425081,0.174,0.078,0.1985213369
97177,0.209197349769475
"Arl6ip6",1.10883630632134e-05,0.156187115266204,0.155,0.067,0.1988587
0317567,0.252267296788107
"Psmc6",1.10980294754693e-05,0.227459719812685,0.387,0.237,0.199032060
613066,0.367185805209639
"Vopp1",1.11499202780639e-05,0.113794302324743,0.09,0.03,0.19996267026
6798,0.183166105917571
"Dph6",1.14256770223113e-05,0.122369613181939,0.129,0.051,0.2049080917
18131,0.193979543981609
"Slc35e4",1.15979347695051e-05,0.107578167894366,0.09,0.03,0.207997362
156305,0.168922453803465
"Tet3",1.16353051428373e-05,0.192715931524911,0.187,0.09,0.20866756243
1645,0.301988351458884
"9830147E19Rik",1.16400602651834e-05,0.107051430522196,0.071,0.02,0.20
8752840795799,0.167707232098871
"Rgs11",1.164393258241e-05,0.0895388598261971,0.09,0.029,0.20882228693
2941,0.140242181344094
"Rhou",1.16738000757881e-05,0.126591540581245,0.11,0.04,0.209357930559
183,0.197952445948472
"Arhgap21",1.17063134753717e-05,0.12689397810574,0.135,0.055,0.2099410
25867315,0.198072441738525
"Mtdh",1.17370207588665e-05,0.176548118235315,0.458,0.285,0.2104917302
89511,0.275116506160677
"Uty",1.18039903232856e-05,0.137200626962207,0.181,0.083,0.21169276245
7803,0.213020339905623
"Egfl6",1.18551665969452e-05,0.0894936443952918,0.097,0.032,0.21261055
7749615,0.138562396957093
"Tmem216",1.18973897152981e-05,0.129188793633029,0.135,0.055,0.2133677
87154155,0.199562826045252
"Sp3",1.1949977660547e-05,0.182179374337821,0.213,0.107,0.214310899364
249,0.280615903411075
"Maml2",1.19685454820815e-05,0.14027916855461,0.097,0.033,0.2146438946
75649,0.215858067475173
"Nipal3",1.20492140551893e-05,0.109099787729532,0.174,0.078,0.21609060
4865764,0.167147147197025
"Atp10b",1.21129276590621e-05,0.0771781588831601,0.071,0.02,0.21723324
4637619,0.117834350549486
"Src",1.21406639096972e-05,0.0855478685144862,0.135,0.054,0.2177306665
56509,0.130417422243129
"Xpc",1.22027520001344e-05,0.184284131089022,0.194,0.093,0.21884415437
0411,0.280000465929137
"Zfp113",1.22981170789357e-05,0.105496333077349,0.123,0.047,0.22055443
1693632,0.159469392094267

"Atp9a",1.23452553034408e-05,0.12760831896141,0.161,0.07,0.22139980861
1908,0.192405924920555
"Orai1",1.239905334328e-05,0.145016563597835,0.174,0.08,0.222364622658
384,0.218023238328247
"Ctnnb1",1.24644372594678e-05,0.196413687437019,0.652,0.45,0.223537217
811296,0.29426253895533
"Ube2m",1.25202691682789e-05,0.153636474854139,0.465,0.288,0.224538507
263913,0.22948804109884
"Psenen",1.25612095941979e-05,0.191566584597735,0.626,0.437,0.22527273
2862345,0.28551916408368
"Tsg101",1.25627425654673e-05,0.138642567194561,0.361,0.211,0.22530022
5169091,0.206621989294952
"Dennd1b",1.26405664404347e-05,0.166825212476111,0.181,0.085,0.2266959
18542756,0.24759292598739
"Fam168a",1.26495596236358e-05,0.159395321124215,0.174,0.081,0.2268572
02290285,0.236452522358862
"Smurf1",1.27553769057301e-05,0.117368155763113,0.09,0.03,0.2287549294
27364,0.173130239049219
"Mlxip",1.28131206987485e-05,0.107346193531263,0.11,0.04,0.22979050661
1356,0.157861940961077
"Mea1",1.28282096656214e-05,0.129372064158358,0.439,0.269,0.2300611121
43254,0.190100643655919
"Chic2",1.28554683789027e-05,0.151422245745532,0.245,0.129,0.230549969
90724,0.222179991247209
"Srek1",1.29239495776506e-05,0.198434776957217,0.252,0.134,0.231778111
725586,0.290106639376135
"Rpl4",1.30205499987968e-05,0.244178670717889,0.91,0.761,0.23351054367
8421,0.355164725271033
"Slc25a40",1.30631524812247e-05,0.100177112960896,0.11,0.04,0.23427457
6598283,0.145383181937857
"Dnajb6",1.30875961574897e-05,0.164267481103547,0.51,0.335,0.234712949
48842,0.238087973465333
"Chmp2b",1.30893192552404e-05,0.179009803377092,0.329,0.191,0.23474385
1523482,0.259431810802319
"AI987944",1.31309964264947e-05,0.111646242517922,0.142,0.059,0.235491
289912756,0.161449549294565
"Akap17b",1.32137557979645e-05,0.0824172744993459,0.09,0.03,0.23697549
6480695,0.11866427096491
"Zfp560",1.32965433774384e-05,0.177133066232664,0.239,0.122,0.23846020
8930981,0.25392960686757
"Lsm14a",1.33552353339347e-05,0.127383346144198,0.271,0.145,0.23951279
0478785,0.182049712797788
"Bbs4",1.33562634177137e-05,0.121010972180954,0.09,0.03,0.239531228133
277,0.172933329324531
"Fam172a",1.33610528126096e-05,0.113851846217566,0.123,0.048,0.2396171
21141341,0.162661607995732
"Elavl1",1.34114951150535e-05,0.152634077240815,0.361,0.216,0.24052175
3393369,0.217495125940498
"Rrp1b",1.3435860600784e-05,0.107055207757205,0.097,0.033,0.2409587240
1446,0.152353438184674

"Tmem246",1.35207609066106e-05,0.105396045918578,0.161,0.07,0.242481326099154,0.149328340588845
"Btbd3",1.36217312719283e-05,0.0906545763926978,0.129,0.051,0.244292128630762,0.127767700683294
"Ranbp3",1.3675214158437e-05,0.122573362430214,0.129,0.051,0.24525129071741,0.17227341875207
"Zc3h14",1.37228188383846e-05,0.162932940364466,0.29,0.163,0.246105033047589,0.228431472502888
"Wdr60",1.37481014562774e-05,0.147616734650512,0.135,0.056,0.246558451516879,0.206686484440815
"Rgl1",1.37740834244332e-05,-0.306733746477267,0.052,0.19,0.247024412133785,-0.428896016784559
"Pecam1",1.38367121927429e-05,0.141961853081654,0.103,0.037,0.248147596464651,0.197856715355185
"Dzip1l",1.38538159818397e-05,0.147140845796588,0.135,0.056,0.248454335818313,0.204893070051103
"St8sia1",1.38997178474478e-05,-0.251698439932902,0,0.109,0.24927753987613,-0.349656549344465
"Aggf1",1.39064206478923e-05,0.137222548268087,0.213,0.106,0.249397747899301,0.190561813968289
"Pigr",1.39376553419206e-05,0.214054493044022,0.219,0.11,0.249957910902005,0.296778577155348
"Gm4944",1.39838090843322e-05,0.119492089041394,0.103,0.037,0.250785632118414,0.16527629073231
"Mycbp",1.39924572334841e-05,0.149530110808221,0.31,0.174,0.250940728025304,0.206731136755742
"Arhgap17",1.40777205099992e-05,0.162532535367384,0.174,0.08,0.252469839626325,0.223720099964307
"Gm15883",1.40982998707839e-05,-0.281441728708549,0,0.109,0.252838909882639,-0.386983140491397
"Tm9sf4",1.41247310058126e-05,0.211340364823374,0.252,0.136,0.253312925858242,0.290197730360866
"Med19",1.41723241556201e-05,0.150000920808543,0.187,0.089,0.254166461406892,0.205466141304379
"Eif2b4",1.42941571861789e-05,0.122839643446431,0.194,0.091,0.256351414976933,0.167210067197791
"Cbfa2t3",1.43839243492182e-05,0.0987753543068508,0.071,0.02,0.25796129927888,0.133835242384826
"Polr2h",1.44042805518439e-05,0.148292110434608,0.206,0.101,0.258326367416768,0.200718043193562
"Clec4a2",1.44053936128282e-05,0.138979509987556,0.103,0.037,0.258346329052461,0.188102406169734
"Tfdp2",1.44876229667893e-05,0.190271744344133,0.245,0.129,0.259821030286399,0.256441070470306
"Gm15638",1.45038831778424e-05,-0.28447905431741,0,0.109,0.260112640911425,-0.383091018082181
"Agxt2",1.45090777864786e-05,-0.277209629406434,0.019,0.141,0.260205801022706,-0.373202450357587
"Spr",1.45647366607698e-05,-0.431601011140141,0.29,0.425,0.261203987274245,-0.579404338281955

"Rab43",1.46044425984606e-05,0.0653974670434301,0.058,0.015,0.26191607
3560792,0.0876150241515362
"1110034G24Rik",1.48322892239425e-05,0.0953805868625253,0.11,0.04,0.26
6002274942184,0.126307782004882
"Baz2b",1.48678194769906e-05,0.171023532944143,0.297,0.167,0.266639474
500349,0.226068793708862
"Rhbdl2",1.48793525190157e-05,0.102355815505357,0.071,0.02,0.266846308
076027,0.135220467578978
"Cuedc2",1.4879983229602e-05,0.163950806154173,0.342,0.204,0.266857619
239682,0.216585576911623
"Pde7b",1.49781535593891e-05,0.0969764055893977,0.052,0.012,0.26861820
5934084,0.127472014977343
"Pde4a",1.49994367141498e-05,0.0927422847543447,0.084,0.027,0.26899989
8031563,0.121774726366627
"Cotl1",1.50292486148696e-05,-0.407973419589393,0.206,0.358,0.26953454
4659071,-0.534877107769094
"Nenf",1.50581787961578e-05,0.167685269609937,0.619,0.424,0.2700533785
30294,0.219522762961187
"Gart",1.5065534275206e-05,0.121179634768716,0.174,0.079,0.27018529169
1545,0.158581400622915
"Atp9b",1.50926616075327e-05,0.109910806154833,0.123,0.047,0.270671793
269491,0.143636748748114
"Wnt4",1.50979367686773e-05,0.136844874824425,0.097,0.033,0.2707663980
09458,0.178787668822669
"Slc34a3",1.51338952452836e-05,-0.30031262673877,0,0.108,0.27141127732
8916,-0.391643696781359
"Herc2",1.51608609739983e-05,0.160983772843753,0.226,0.116,0.271894880
707686,0.209655567343967
"Tet2",1.51898539992821e-05,0.189916758472805,0.213,0.107,0.2724148416
23125,0.24697330237527
"Rdh16",1.52251904285371e-05,-0.297017907830929,0.013,0.129,0.27304856
5145384,-0.385560611033686
"Kcnj10",1.52287093914551e-05,0.10268219803428,0.155,0.065,0.273111674
226356,0.133268606878331
"Hdac4",1.52351118364409e-05,0.120470803747767,0.135,0.055,0.273226495
674732,0.156305347081421
"Pcm1",1.52765367243952e-05,0.191481026055304,0.329,0.192,0.2739694096
15304,0.247917918218661
"Akirin1",1.53051418558131e-05,0.160655042026221,0.194,0.094,0.2744824
14042152,0.207705776611172
"Ext1",1.5332301439606e-05,0.182935664568253,0.213,0.106,0.27496949401
7894,0.236187343489393
"Gps2",1.53656967171858e-05,0.135253639933799,0.232,0.119,0.2755684049
2601,0.174331038736105
"Cacybp",1.5468545250263e-05,0.207281218769912,0.361,0.221,0.277412890
518217,0.265785991035858
"4930550C14Rik",1.55688364243813e-05,0.0757143820582739,0.077,0.023,0.
279211512434854,0.0965953240437444
"Syce2",1.55703375237369e-05,0.0874045392644524,0.077,0.024,0.27923843
3150697,0.111501032278542

"Pla2g4a",1.55757646144553e-05,0.122336978087099,0.11,0.041,0.279335762595641,0.156021335901935
"Gtpbp6",1.55762904868098e-05,0.125126980626435,0.123,0.048,0.279345193590448,0.159575315397286
"Dync2h1",1.5721271234248e-05,0.14338133371625,0.174,0.08,0.281945278315003,0.181526829988328
"Yipf2",1.57272355545888e-05,0.110191978676483,0.155,0.067,0.282052242435996,0.139465906818574
"Hspa4l",1.57366764489754e-05,0.187940622299768,0.226,0.117,0.282221555435925,0.237756700956977
"Cir1",1.58095080902352e-05,0.156451679743381,0.316,0.182,0.283527718090277,0.197198798420221
"Fbxo2",1.59495257596768e-05,0.0884090633149243,0.077,0.024,0.286038794974044,0.110655244122156
"Nck1",1.61561122331108e-05,0.147965952602373,0.187,0.089,0.289743716788609,0.18329407882415
"Zfp81",1.61784483382602e-05,0.0856944505929765,0.084,0.027,0.290144292498358,0.106036335284803
"Dlg1",1.6289144639632e-05,0.136156125998981,0.213,0.105,0.292129519967161,0.167548011938813
"Slc7a6os",1.62903004673711e-05,0.114276862827493,0.135,0.055,0.292150248581834,0.140616200865089
"Golga1",1.63069863424567e-05,0.151185214495825,0.174,0.081,0.292449493065619,0.185876672800823
"Rpgrip1",1.63997128853261e-05,0.146199465822907,0.219,0.11,0.294112450885438,0.178917897317596
"Camk2g",1.64266053193491e-05,0.0960084853672552,0.11,0.04,0.294594739797206,0.117337214960769
"Fmnl3",1.65866362106606e-05,0.0796728862288947,0.071,0.021,0.297464733801987,0.0966001559962062
"Rnf213",1.68471192228891e-05,0.127726928026178,0.142,0.06,0.302136236143293,0.152873454401522
"Rchy1",1.69668294511459e-05,0.213610816370822,0.323,0.189,0.30428311937685,0.254153443812712
"Pla2g7",1.70372524847081e-05,0.234792571765473,0.135,0.056,0.305546086060756,0.278382906192484
"Efhd2",1.70480216115142e-05,0.184236405008501,0.219,0.113,0.305739219580895,0.218324333887177
"Rpl13",1.7299931270939e-05,0.250204691199387,0.961,0.929,0.31025696741302,0.292828160679013
"Gm4631",1.74015248708385e-05,0.110728615417715,0.142,0.059,0.312078947033619,0.128943371649117
"Tuba1c",1.74064647756851e-05,0.177161132382648,0.31,0.178,0.312167539287136,0.206253692098505
"Unk",1.74296891539082e-05,0.0842217792098396,0.09,0.03,0.312584045286189,0.097939982717274
"Ccnd2",1.7488932680973e-05,0.147997429503613,0.084,0.027,0.313646518700569,0.171601341783005
"Ccl25",1.75620632059439e-05,0.0899571193994631,0.129,0.051,0.314958041535398,0.103928885907839

"Dcaf11",1.77115395030708e-05,0.251576547832009,0.387,0.244,0.317638749448073,0.288518186475995
"Ulk3",1.79543316272457e-05,0.0964168266203096,0.097,0.033,0.321992983403025,0.109262008902866
"Epc1",1.80360277821447e-05,0.137361979105527,0.187,0.089,0.323458122244983,0.155038491309784
"Zfp142",1.80416261418471e-05,0.101682119369336,0.084,0.027,0.323558523227886,0.114735589403886
"Med9",1.81274959229592e-05,0.17439247010644,0.219,0.111,0.325098511882351,0.195952093082736
"Vangl1",1.81412960989628e-05,0.112086193290017,0.11,0.04,0.325346004238799,0.125857779386274
"Hoxa10",1.81651643604147e-05,0.253217439502119,0.226,0.117,0.325774057639678,0.283996326332073
"Esd",1.82659951291995e-05,-0.360658821253653,0.639,0.692,0.327582356647064,-0.402500937461667
"Ppip5k1",1.82770515799656e-05,0.166851561461451,0.168,0.078,0.327780643035102,0.186108011219387
"Gpat4",1.83084459132176e-05,0.160081659381789,0.258,0.138,0.328343669007644,0.178282055242109
"Taf6l",1.83762529510614e-05,0.204190777758089,0.329,0.198,0.329559720424335,0.226651292828401
"Nudc",1.8464024916094e-05,0.2006807451072,0.465,0.301,0.33113382284523,0.221798919080913
"Trp53",1.85941199400381e-05,0.154097995288993,0.226,0.116,0.333466947004644,0.169232194863358
"Polr2a",1.86234744343935e-05,0.123637120389748,0.232,0.12,0.333993390506413,0.135584679167158
"Fam189b",1.8699590363841e-05,0.0547917083433527,0.065,0.018,0.335358453585125,0.0598629718127862
"Akr1b10",1.8706639924929e-05,0.163431659153472,0.213,0.107,0.335484880413676,0.178496526273982
"Dock1",1.87859164996763e-05,0.212420990874664,0.206,0.103,0.336906626505194,0.231103302252386
"Rbm42",1.90920556134056e-05,0.137403230797168,0.297,0.166,0.342396925370816,0.147266668736731
"Mesdc2",1.91260995691154e-05,0.141826376470885,0.245,0.131,0.343007469672516,0.151754656044205
"Usp9x",1.92075416160283e-05,0.215868710324561,0.452,0.295,0.344468051341852,0.230062926832772
"Eif3l",1.92194471280531e-05,0.247654015393769,0.4,0.254,0.344681564794504,0.263784783548408
"Kansl2",1.94255027484209e-05,0.127488465310054,0.2,0.098,0.34837696629018,0.134432781013684
"Dst",1.94876644620607e-05,0.149427958768484,0.497,0.319,0.349491774462597,0.157089915121106
"Uqcc2",1.94930591434112e-05,0.208069064583892,0.71,0.501,0.349588522677936,0.218680267618219
"2210013O21Rik",1.9509557489204e-05,0.198256239517227,0.2,0.1,0.349884404011385,0.208199276311923

"Dph5",1.95187176409484e-05,0.149972245162502,0.148,0.064,0.350048682172769,0.157423322555067
"Zcchc6",1.96051932050721e-05,0.162575751805416,0.206,0.104,0.351599534939764,0.169934326345349
"Hspd1",1.97877347783854e-05,-0.407771754378958,0.548,0.615,0.354873235515563,-0.422449350383804
"Foxn3",1.97962131584395e-05,0.187896123121346,0.252,0.137,0.355025286783454,0.1945788858026
"Rnf138",1.97968426378357e-05,0.142502145424432,0.161,0.072,0.355036575866945,0.147565882809853
"Taf7",1.98008270769649e-05,0.118507035921087,0.103,0.037,0.355108032798288,0.122694270862968
"Fam229b",1.98839217006881e-05,0.118385869294678,0.129,0.052,0.35659825178014,0.122073053549379
"Snrpd3",1.99539688237896e-05,0.173236237048646,0.548,0.366,0.357854476885843,0.17802255754538
"Rbm27",2.00960445193583e-05,0.187778185930899,0.2,0.1,0.360402462410171,0.191634008335276
"Tmco4",2.01358656631032e-05,0.109054963493723,0.116,0.045,0.361116614802093,0.111078406378193
"Slc29a1",2.01544665975887e-05,0.129484679083053,0.129,0.052,0.361450203961155,0.13176762284492
"Nt5c3b",2.01668473441247e-05,0.105343813245406,0.103,0.038,0.361672240269532,0.107136437423545
"Pick1",2.03782126646936e-05,0.211943383820448,0.187,0.091,0.365462865928615,0.213340218513262
"Rnf43",2.03991030107969e-05,0.0851219154700535,0.058,0.015,0.365837513395631,0.0855957037645116
"Mmgt2",2.04470026557206e-05,0.131973624954878,0.123,0.049,0.366696545627693,0.132398662393058
"Sfrp1",2.08136361980221e-05,0.0911059257594748,0.213,0.103,0.373271751575328,0.0897802040855144
"Anxa7",2.10043700426835e-05,0.137782052870352,0.387,0.234,0.376692372345485,0.134520257469492
"Slc25a28",2.1053159171758e-05,0.157574240622825,0.168,0.077,0.377567356586308,0.153478302795396
"Flywch2",2.12443878851276e-05,0.13794209836252,0.129,0.053,0.380996852331878,0.133109181831733
"Cab39",2.13246017194933e-05,0.197512302767425,0.342,0.206,0.382435407237392,0.189847938578608
"Ahr",2.13758483683654e-05,0.124413380971859,0.103,0.038,0.383354464638265,0.119286955311914
"Zfp948",2.14985932846085e-05,0.0901277845266607,0.077,0.024,0.385555771966168,0.085898035512362
"Nemf",2.15508180130025e-05,0.16781114714609,0.271,0.151,0.386492370245187,0.159528517827266
"Mcf2l",2.15665534421509e-05,0.078052581257738,0.077,0.024,0.386774569431535,0.0741431821953216
"Hs6st1",2.18573559497342e-05,0.163671141647398,0.29,0.162,0.391989821602533,0.153281200163212

"Mitd1",2.1929609357317e-05,0.146465818511451,0.161,0.073,0.3932856142
14124,0.136684710660415
"Ccdc64",2.2089445563761e-05,0.0886572727353842,0.116,0.044,0.39615211
674049,0.0820928230265246
"Serpinb1a",2.21028416171431e-05,0.159923769822284,0.206,0.101,0.39639
2361561845,0.147985579745452
"Rad21",2.22103296586528e-05,0.187921566494572,0.406,0.258,0.398320052
09828,0.172981697822115
"Srgap3",2.25097604300875e-05,0.0762217592348232,0.077,0.024,0.4036900
43553188,0.069141361052299
"Cpsf4",2.29032335025247e-05,0.0728895775296756,0.09,0.03,0.4107465896
34277,0.0648556026400919
"Cdk14",2.29153315039955e-05,0.106757491956843,0.084,0.027,0.410963555
192656,0.0949341789275874
"Lamtor4",2.29461316712549e-05,-0.384562462655703,0.503,0.588,0.411515
925392285,-0.341455917415847
"Lrp10",2.30902729077603e-05,0.169057703682521,0.413,0.262,0.414100954
327773,0.149048960961811
"Gpd2",2.32574623336479e-05,0.158982047313907,0.187,0.091,0.4170993294
91642,0.139018812384075
"Ibtk",2.34564344267961e-05,0.142522774831472,0.181,0.086,0.4206676950
1016,0.123412192401041
"Dido1",2.35104285995879e-05,0.142857899995318,0.226,0.117,0.421636026
505009,0.123373915809075
"Cyp20a1",2.38100491817399e-05,0.150158109547803,0.142,0.061,0.4270094
22025322,0.127776923247855
"Irf9",2.38960316894091e-05,0.137553582589559,0.142,0.061,0.4285514323
17863,0.116555274331195
"Haus4",2.39550705688139e-05,0.0812934616595485,0.103,0.037,0.42961023
5581108,0.0686829687088808
"Setd1a",2.41613649559143e-05,0.100103745457839,0.103,0.038,0.43330991
9119367,0.0837169683861369
"Cap1",2.41996570341406e-05,0.152791122141376,0.129,0.053,0.4339966492
50278,0.127537571020917
"Rnf38",2.43684638062689e-05,0.150042698938364,0.135,0.057,0.437024029
901627,0.124200409336024
"Lats2",2.4393474823627e-05,0.144784952669751,0.142,0.061,0.4374725774
86927,0.119699693501196
"Ggh",2.44377061077514e-05,0.157408564183724,0.277,0.154,0.43826582133
6414,0.129850992493468
"Macc1",2.44492694994094e-05,0.0679642949816837,0.052,0.013,0.43847319
9202408,0.0560336107669841
"Megf9",2.45394015722049e-05,0.123466453748374,0.123,0.049,0.440089627
795922,0.101338409862261
"Aco1",2.45757425516119e-05,-0.371558727946654,0.155,0.3,0.44074136692
0609,-0.304416967904014
"Ppp3ca",2.46589504131907e-05,0.14762993539438,0.342,0.201,0.442233616
710162,0.120453772807546
"Cdk9",2.4725213445918e-05,0.124722108448343,0.226,0.117,0.44342197793
9094,0.101428186327027

"B3gat2",2.5018179531008e-05,-0.260166651648917,0.006,0.114,0.448676031709098,-0.208511651660499
"Psmd9",2.50192370519847e-05,0.142165213183955,0.252,0.135,0.448694997290294,0.113932895801606
"Igsf6",2.50698255133087e-05,0.0711401630226187,0.052,0.013,0.449602250755678,0.0568688753696797
"Fam92a",2.50743546784589e-05,0.182223578892735,0.232,0.124,0.449683476803481,0.145635148587311
"Kdm5c",2.51967381107517e-05,0.169735994885482,0.168,0.079,0.451878301278221,0.134828494210535
"5730508B09Rik",2.51993823766687e-05,0.171070782772444,0.129,0.053,0.451925723543177,0.13587082075575
"Madd",2.53640887092689e-05,0.183967807449855,0.116,0.046,0.454879566912028,0.144915596505017
"Epb41l1",2.57359106449099e-05,0.18300023433134,0.2,0.101,0.461547821505814,0.141490219535173
"Ccdc71l",2.5745293901829e-05,0.0805918105945226,0.11,0.041,0.461716100835402,0.0622817604033038
"Mapkap1",2.58442145606194e-05,0.138017266738458,0.284,0.158,0.463490143930148,0.106131159499821
"Pard6b",2.5857343962075e-05,0.149979179342988,0.2,0.1,0.463725606615852,0.115253340120699
"Pyroxd1",2.58591294687115e-05,0.104732775361681,0.155,0.068,0.463757627891872,0.0804759541762217
"Unc50",2.59042587949267e-05,0.185612359589975,0.303,0.174,0.464566977228216,0.142299629917504
"Arl2",2.59116121483225e-05,0.145653237756743,0.316,0.178,0.464698852268015,0.111623647364663
"Cdipt",2.59287098890306e-05,0.139446291711612,0.29,0.163,0.465005483149876,0.106774873640249
"Tgfb2",2.59611378970449e-05,0.167852201134457,0.065,0.018,0.465587047045603,0.128315656322808
"Slc51a",2.59785722638388e-05,-0.293189820836938,0.013,0.124,0.465899714979686,-0.223933949756901
"Plgrkt",2.60211527119848e-05,0.217059582169585,0.252,0.138,0.466663352736735,0.165431342658006
"Pkd2",2.60341528689007e-05,0.239484060558423,0.419,0.274,0.466896497550866,0.182402478769447
"Erich1",2.6138241736723e-05,0.207103608657692,0.135,0.058,0.46876322730639,0.156913599030187
"Scaper",2.61555483721638e-05,0.169170491463004,0.174,0.083,0.469073604506386,0.128061314913529
"Cxxc1",2.61570378086834e-05,0.137984171539133,0.168,0.078,0.469100316060929,0.104445551130864
"Rps17",2.62278572275365e-05,0.189683302087576,0.961,0.897,0.470370391518639,0.143065752644619
"Rab6a",2.63704575317752e-05,0.160510370472865,0.368,0.225,0.472927785374856,0.120192183951921
"Ensa",2.65554279766312e-05,0.204108783686528,0.239,0.13,0.476245045332904,0.151412540457703

"Snrpb",2.66903919342775e-05,0.156700153596072,0.594,0.399,0.478665488949334,0.115449351896678
"Csf2rb",2.67501026240468e-05,0.0500062243520345,0.058,0.015,0.479736340459655,0.0367305027376792
"Sgpp1",2.68392306459365e-05,-0.322470925553063,0.071,0.202,0.481334762404225,-0.235788251036694
"Flii",2.69943603563861e-05,0.146639285450808,0.213,0.109,0.484116858631428,0.106376384039834
"Slc30a2",2.70986787327186e-05,-0.253580110190194,0.006,0.114,0.485987704392575,-0.182976295860433
"Kansl3",2.71574210853318e-05,0.164338043956612,0.239,0.127,0.487041189744341,0.11822587032212
"Tpgs1",2.73841770509009e-05,0.148297545029816,0.31,0.178,0.491107831230856,0.105453132603552
"Fam175b",2.75129996946854e-05,0.100268023339677,0.206,0.102,0.493418136524489,0.070829162966593
"Wiz",2.7513799551609e-05,0.156046270338239,0.129,0.053,0.493432481158555,0.110226286283033
"Cdcp1",2.75454884180612e-05,0.123470435792476,0.174,0.081,0.49400078928951,0.0870735940432138
"Npepps",2.75957388135762e-05,0.194154016539864,0.297,0.172,0.494901979882675,0.13656707250322
"S100a8",2.77168284250437e-05,0.912878549512123,0.277,0.159,0.497073600974734,0.638117783298567
"Slc18b1",2.77461850729926e-05,0.109110578997714,0.116,0.045,0.497600083099049,0.0761546637499442
"Ugcg",2.7865345035451e-05,0.110704813434937,0.116,0.045,0.499737097865779,0.0767929536789362
"Ptprb",2.79624983872523e-05,0.212558226553534,0.071,0.021,0.501479446076983,0.146706127218384
"Psma5",2.79785790534591e-05,0.150701348292172,0.4,0.249,0.501767836744736,0.103926323656684
"Lurap1",2.80120078990534e-05,0.121667655159675,0.116,0.046,0.502367349661624,0.0837588918202563
"R3hdm1",2.80851916051543e-05,0.217118605887403,0.329,0.203,0.503679826246837,0.148903083395622
"Tln1",2.81486726431805e-05,0.13959073572687,0.4,0.245,0.504818295182799,0.0954181862897167
"Tax1bp1",2.83084540298136e-05,0.177025543964301,0.645,0.448,0.507683814570677,0.120004985590168
"Susd3",2.83739742434614e-05,-0.253666216987453,0.006,0.113,0.508858854082238,-0.171372990070378
"Cnpy4",2.8612389582922e-05,0.101364087919519,0.084,0.028,0.513134594780123,0.0676318528092607
"Slc7a13",2.87847140067516e-05,-0.576194277601056,0.006,0.113,0.516225060997084,-0.380986826436346
"Mettl7a1",2.88075041310311e-05,-0.274357939389577,0.232,0.393,0.516633779085912,-0.181191748296045
"Psmb2",2.88823235899504e-05,0.197740321465945,0.561,0.389,0.51797559126217,0.130078953879484

"Cops8",2.91967548403939e-05,0.169228213259182,0.245,0.132,0.523614601
307624,0.109490545496131
"Phf20l1",2.92633550471239e-05,0.155628722729223,0.226,0.118,0.5248090
0941512,0.100337086163786
"Ube2w",2.9270777094543e-05,0.143492829490733,0.284,0.161,0.5249421164
13535,0.0924764330793515
"Vezf1",2.95740406105051e-05,0.238567648298843,0.277,0.162,0.530380844
308799,0.151290049424376
"Adgrg3",2.95811092621595e-05,0.12877391474465,0.084,0.028,0.530507613
507569,0.0816324848763039
"Phgdh",2.96202897837097e-05,0.671302547391076,0.142,0.063,0.531210276
98105,0.424664201975427
"Dnajc1",2.96387369190139e-05,0.199426772736445,0.271,0.155,0.53154110
7905596,0.126032683048731
"Nbea",2.98137534630981e-05,0.14596488563989,0.174,0.082,0.53467985460
7201,0.0913867339669788
"Cdc37",2.9933872009332e-05,0.181780179166639,0.452,0.298,0.5368340606
15359,0.11307931330906
"Haghl",3.00479127224159e-05,0.166109932309702,0.219,0.114,0.538879266
763806,0.102699746013618
"Cdc40",3.005746084222e-05,0.161136779039495,0.174,0.084,0.53905050274
4374,0.0995738305335594
"Rsbn1",3.01438377794055e-05,0.140627047995038,0.161,0.074,0.540599586
735859,0.0864963797726831
"Cyp2s1",3.01518412324778e-05,0.12275247163291,0.123,0.049,0.540743120
663257,0.0754695619899228
"Nup214",3.02144245950985e-05,0.0928700062087691,0.161,0.072,0.5418654
90688496,0.0569049336187282
"Samhd1",3.02181310443105e-05,0.142426895463838,0.168,0.078,0.54193196
2148664,0.0872528264311243
"Lpar6",3.0244895646685e-05,0.183012943553729,0.142,0.061,0.5424119585
27649,0.111954415568968
"Col4a3",3.02597407728036e-05,0.223232372598345,0.381,0.24,0.542678191
01946,0.136448284153003
"Uvrag",3.03363030939718e-05,0.167565959812102,0.148,0.066,0.544051259
687289,0.101999378557387
"Tmx4",3.05957157851089e-05,0.129841387425233,0.187,0.092,0.5487035668
90143,0.0779304026802764
"Ube2d3",3.0664894731026e-05,0.175319360514069,0.813,0.599,0.549944222
10622,0.104830181465151
"Ankrd16",3.11377709801778e-05,0.109912399808062,0.148,0.065,0.5584247
84758509,0.0640388487848155
"Sowahc",3.12050982600774e-05,0.130175267435662,0.148,0.066,0.55963223
2196229,0.0755635455395678
"Arid5a",3.14383829450289e-05,0.071124456218329,0.065,0.018,0.56381595
9736149,0.040756261743835
"D630029K05Rik",3.14906609269069e-05,-0.279017248527287,0,0.101,0.5647
53513063148,-0.159420942182488
"Taf1",3.15397701559392e-05,0.178054308574093,0.181,0.088,0.5656342379
76614,0.10145670399422

"Gm26518",3.17733071451781e-05,0.124085445462264,0.09,0.031,0.569822490341624,0.0697894251258408
"Nop9",3.18393803270555e-05,0.110609537139968,0.123,0.049,0.571007446785413,0.0619803890350107
"Hoga1",3.19744230910889e-05,-0.374690432845106,0.084,0.217,0.573429303715589,-0.208373076439872
"Otud5",3.20470413635502e-05,0.141085859412556,0.181,0.087,0.57473163981391,0.0781406939425612
"Mterf1a",3.2049285084292e-05,0.0996390115768434,0.065,0.018,0.574771878701693,0.0551782960508166
"Slc9a3r2",3.21829012903073e-05,0.153407277606652,0.142,0.061,0.577168151740371,0.0843159580901115
"A130014A01Rik",3.2245475956438e-05,0.0534921217104016,0.058,0.015,0.578290365802758,0.0292965210055761
"Psmc3",3.23148075469138e-05,0.168104958144849,0.574,0.389,0.579533758546353,0.0917065269924518
"Fam81a",3.25453769230003e-05,0.147844011218494,0.129,0.053,0.583668789737087,0.0796024087602474
"Cks1b",3.25559974867817e-05,0.103157410193598,0.11,0.042,0.583859258927943,0.0555085196675038
"Thsd7a",3.26268340927664e-05,0.174068362870282,0.065,0.019,0.585129642619672,0.0932870381832664
"Map3k14",3.26710997396481e-05,0.114843298509939,0.103,0.038,0.585923502730848,0.0613913272293521
"Aqp11",3.28283178017689e-05,-0.294152997564681,0.019,0.131,0.588743051456924,-0.155832090981282
"Tmem252",3.30015486958839e-05,-0.361323498515054,0.058,0.184,0.591849774311982,-0.189515055122195
"Adtrp",3.31408729796216e-05,-0.287931497349688,0.013,0.123,0.594348416016533,-0.149807755702083
"Angel2",3.31484593904059e-05,0.158566169521797,0.232,0.123,0.594484470707539,0.0824640307067618
"Kdelc1",3.3284390725389e-05,0.115343367365588,0.084,0.028,0.596922263269126,0.059513531135499
"Rhbdf1",3.33063329302819e-05,0.122712346512293,0.148,0.065,0.597315774771675,0.0632348219063174
"Ssh1",3.33792115622039e-05,0.145552240254333,0.135,0.058,0.598622780156565,0.0746862936648268
"Rad50",3.34579638197975e-05,0.121106219985605,0.155,0.07,0.600035123144248,0.0618570711881911
"Gdf11",3.3710225291769e-05,0.0669046696463765,0.058,0.016,0.604559180382585,0.0336701572925606
"Mgat4a",3.38794696235662e-05,0.116793821166366,0.103,0.038,0.607594408229035,0.0581922540958721
"Ephb6",3.40589853875246e-05,0.079115528837183,0.065,0.018,0.610813843939867,0.0390010316571071
"Tmod3",3.46220243468708e-05,0.203670608160474,0.187,0.093,0.620911384636781,0.0970626713864602
"Nav2",3.47224806002549e-05,0.200595013042318,0.277,0.16,0.622712967084972,0.095015758256272

"Ehbp1l1",3.49585397075024e-05,0.0997090154420545,0.071,0.022,0.626946
451114347,0.0465535557048238
"Fam161b",3.50948317909625e-05,0.0601132420134444,0.052,0.013,0.629390
713339121,0.0278326143521921
"Ddx21",3.51870000295742e-05,0.245848361183733,0.232,0.129,0.631043658
530384,0.113183724920442
"Kif26b",3.53044036206682e-05,0.127744374087076,0.071,0.022,0.63314917
4533063,0.0583854667699929
"Chd1",3.53483199119271e-05,0.189085804281858,0.265,0.15,0.63393676930
05,0.0861864559211607
"Zfp326",3.53599943603816e-05,0.200306161353649,0.194,0.098,0.63414613
8859083,0.0912346187370959
"Cd302",3.55069624825866e-05,-0.322414519633877,0.065,0.196,0.63678186
5162708,-0.145514739952093
"Trnt1",3.55484746779243e-05,0.175906712083743,0.187,0.092,0.637526344
873894,0.0791861087836452
"Elmsan1",3.55600995105402e-05,0.0937049100141576,0.071,0.022,0.637734
824622027,0.04215153430786
"Map7d1",3.5894663602807e-05,0.145175686418085,0.135,0.058,0.643734897
052741,0.0639452860802389
"Slc4a3",3.59063481763867e-05,0.110625677374112,0.11,0.042,0.643944448
19532,0.0486910972901962
"Susd2",3.59127095630244e-05,-0.275648685174233,0.013,0.121,0.64405853
330328,-0.121275957571246
"Mfsd7b",3.59339808133976e-05,0.147363115653989,0.135,0.058,0.64444001
1907473,0.0647474534782237
"Smo",3.60055478012385e-05,0.120587295073246,0.194,0.095,0.64572349426
7411,0.0527429407197387
"Ift57",3.60363883738575e-05,0.158474060318082,0.206,0.106,0.646276589
09676,0.0691783186982765
"Sh3gl1",3.60612953378846e-05,0.139547217197409,0.135,0.058,0.64672327
0589622,0.0608198108595969
"Nup188",3.60959683213281e-05,0.0791900419092592,0.077,0.025,0.6473450
95874698,0.0344378287252668
"Rps6",3.61012565387788e-05,0.234482417095942,0.935,0.844,0.6474399347
66459,0.101936366389392
"Srgn",3.61696995376603e-05,0.181612353467258,0.181,0.088,0.6486673915
084,0.0786082170601628
"Arhgap36",3.6207037299562e-05,0.0707773881791889,0.058,0.016,0.649337
006930344,0.0305619186972305
"Gon4l",3.6213040097128e-05,0.171232306206805,0.2,0.102,0.649444661101
893,0.0739103100510698
"Nr2c2ap",3.62931263942017e-05,0.136554416424957,0.155,0.07,0.65088092
8753613,0.0586403662217235
"C3",3.64901923937451e-05,0.179251966591575,0.226,0.115,0.654415110389
424,0.0760052364406762
"Ercc1",3.66406571300045e-05,0.0865514047048222,0.135,0.057,0.65711354
4969502,0.0363428008571232
"Uckl1",3.66881154361501e-05,0.185864816547198,0.148,0.067,0.657964662
231915,0.0778037656865821

"Ube2e3",3.70932426523815e-05,0.173458115170995,0.381,0.239,0.66523021372781,0.0707053632804145
"Dnajc9",3.71327089451117e-05,0.196039338305276,0.181,0.089,0.665938002221634,0.0797014990266589
"C1qtnf4",3.72439956407223e-05,0.142131123706402,0.168,0.078,0.667933817820714,0.0573593154311051
"Wrn",3.7542307809937e-05,0.13060165579524,0.123,0.05,0.673283748263411,0.0516645028168679
"Spop",3.77192296513165e-05,0.190151012574394,0.387,0.245,0.676456664566711,0.0743275382613353
"Rorc",3.78698068662785e-05,0.13479126560256,0.148,0.066,0.679157116339839,0.0521511159831931
"Cers5",3.79090059398484e-05,0.100097538081292,0.181,0.086,0.679860112525241,0.038624458730623
"Traf2",3.80187699288111e-05,0.115585756252646,0.129,0.054,0.681828619903299,0.0442666796049932
"Eif4g1",3.80676955361801e-05,0.177518318111837,0.535,0.353,0.682706051745854,0.0677571248612216
"Rpl15",3.8134465217271e-05,0.208197043219914,0.858,0.727,0.683903499206538,0.0791020627999882
"Rsbn1l",3.81420233835999e-05,0.155361408717276,0.239,0.131,0.68403904736148,0.0589969842576142
"Zfp266",3.81435515429898e-05,0.171523548754493,0.174,0.084,0.684066453371979,0.0651275278295562
"Fbxl8",3.82102318750835e-05,0.067147119321552,0.058,0.016,0.685262298447748,0.0253784952470377
"Plekha3",3.82723646269804e-05,0.146887163003128,0.2,0.101,0.686376587220266,0.0552778757986583
"Avl9",3.83992372074485e-05,0.193501962644409,0.174,0.084,0.688651920078382,0.0721799727389634
"Stk24",3.85863723859735e-05,0.148168013351495,0.168,0.079,0.692008002370049,0.05454920379731
"Sp4",3.87245144848493e-05,0.0867443345846272,0.071,0.022,0.694485442771288,0.031625603223698
"Tmsb15b1",3.90466152296446e-05,0.110985765786201,0.103,0.039,0.700261997528447,0.0395443095669358
"Wdr44",3.93835706087343e-05,0.143542493887475,0.09,0.032,0.706304955297042,0.0499109002695756
"Proc",3.94039683370349e-05,-0.357790483146679,0.065,0.192,0.706670768156384,-0.124221419530927
"Lockd",3.94206405655149e-05,0.0747240495078498,0.084,0.028,0.706969767901944,0.0259118625047056
"Tm9sf3",3.94603787037727e-05,0.189823630328107,0.581,0.403,0.70768243167346,0.065633385914559
"Pigu",3.98671006641917e-05,0.140343884101281,0.155,0.071,0.714976583311615,0.0470861432442818
"Snapin",3.99361221339401e-05,0.177029261855216,0.187,0.093,0.716214414350082,0.0590880649939654
"Tfpi2",3.99462729542377e-05,-0.232965058440994,0,0.099,0.716396459161299,-0.0776988677020603

"2700029M09Rik",3.99648989395428e-05,0.110431363765375,0.252,0.136,0.71673049758176,0.0367797602757362
"Tox4",4.00587828317258e-05,0.122966352827272,0.213,0.11,0.718414211304171,0.0406660771556526
"Hpcal1",4.00945469809356e-05,0.138944067366474,0.271,0.151,0.719055605556099,0.045826058068738
"1700123O20Rik",4.02130262478147e-05,0.114418459567511,0.271,0.151,0.721180412728309,0.0373994981751807
"Nt5c",4.0489535707621e-05,0.117358580377549,0.303,0.174,0.726139333380475,0.0375563140204502
"Mbtd1",4.06435394599346e-05,0.2514319549693,0.2,0.102,0.728901236674467,0.0795070670442196
"Prkd2",4.07691763595301e-05,0.097980576539174,0.065,0.019,0.731154408831813,0.0306807178905437
"Nub1",4.12997463885149e-05,0.177878724357224,0.181,0.089,0.740669651731626,0.0533992939480661
"Lrfn4",4.13439931725733e-05,0.0881699479101797,0.071,0.022,0.74146317355693,0.0263742574233376
"Capza2",4.14193201153794e-05,0.217058684459594,0.51,0.342,0.742814086949214,0.0645336056966099
"Man1a2",4.156540789533e-05,0.199292685397404,0.277,0.162,0.745434025194848,0.0585499282174848
"Zer1",4.15875900123321e-05,0.0944949731434017,0.148,0.065,0.745831839281163,0.0277111347548706
"Epha4",4.16189286318286e-05,0.0931851131562103,0.058,0.016,0.746393866083214,0.0272568177562806
"Lemd2",4.16411143076538e-05,0.116512227353435,0.161,0.074,0.746791743993464,0.0340179494846503
"Klhl14",4.16876020284744e-05,0.10154933100389,0.065,0.019,0.747625454778659,0.0295359433430941
"Sart3",4.20804667643075e-05,0.0934654210752352,0.116,0.046,0.754671090951091,0.0263080173072369
"Abca1",4.21533356181855e-05,0.12290142715688,0.161,0.074,0.755977920976539,0.0343808272457207
"Nudt21",4.26821962566073e-05,0.205164424312399,0.232,0.126,0.765462507665996,0.0548353302602643
"Zfp282",4.27816138431256e-05,0.117217605142906,0.09,0.032,0.767245462662614,0.031056628570206
"Grina",4.28967817532461e-05,-0.407762289080864,0.419,0.518,0.769310883962716,-0.106939787167202
"St5",4.31072527880712e-05,0.118749291712581,0.161,0.075,0.773085471501269,0.0305619904719547
"Eftud1",4.32582970460966e-05,0.105630265345019,0.116,0.046,0.775794299224697,0.0268161307090902
"Jagn1",4.32868852497864e-05,0.169984289089674,0.29,0.171,0.77630700006967,0.0430412490102041
"Trim34a",4.35120959952838e-05,0.111676586579938,0.077,0.025,0.78034592957942,0.027697798977223
"Tnip1",4.36840254558101e-05,0.11504336940413,0.103,0.039,0.783429312524499,0.0280791461230282

"Atg2a",4.40116746481472e-05,0.161433401468212,0.135,0.059,0.789305373
139873,0.0381954648173329
"Cyp24a1",4.40459258049717e-05,-0.30137827274572,0,0.098,0.78991963338
6363,-0.0710722504624936
"Kifap3",4.40475042729809e-05,0.135213043181044,0.206,0.107,0.78994794
1631639,0.0318816444438319
"Rhog",4.41551698185821e-05,0.181566289468652,0.142,0.063,0.7918788155
26451,0.0423679325303223
"Brix1",4.43508353067586e-05,0.122975214961542,0.181,0.088,0.795387880
391409,0.0281521482397741
"Chd8",4.4499095766763e-05,0.122786410299627,0.206,0.105,0.79804678348
1127,0.0276991477665773
"Atp1b3",4.45166550121148e-05,0.197938794387689,0.31,0.183,0.798361690
987267,0.0445745372902128
"Sdk1",4.47478681271366e-05,0.0991280235039165,0.065,0.019,0.802508266
992068,0.0218094659766925
"Slc25a3",4.47492170406474e-05,-0.236146350945176,0.884,0.845,0.802532
45840697,-0.051948177567935
"Oraov1",4.50245973507849e-05,0.0970861601935362,0.129,0.054,0.8074711
28888976,0.0207616790707606
"Capza1",4.51363477681042e-05,0.153794245019883,0.232,0.124,0.80947526
0873182,0.0325073461302868
"Shfm1",4.52610685589657e-05,0.17898699765825,0.89,0.711,0.81171200353
6492,0.0373384198009662
"Eif4a3",4.5310298940586e-05,0.162188314788342,0.265,0.15,0.8125949012
00469,0.0336577359285525
"Zfp503",4.57782364772295e-05,0.223785441026337,0.361,0.223,0.82098689
2982634,0.0441412607383724
"Stx5a",4.59920194798629e-05,0.174763451836069,0.258,0.145,0.824820877
351861,0.0336575244694033
"Sppl2a",4.62100030442792e-05,0.184937951728495,0.323,0.197,0.82873019
4596103,0.0347425611750842
"Smc6",4.62668350227494e-05,0.175125305507251,0.31,0.186,0.82974941929
7988,0.0326839033992775
"Chrna4",4.65719399205245e-05,-0.239257952214511,0,0.097,0.83522117053
4687,-0.0430804792648108
"Csf1r",4.66011287346404e-05,0.17720051352288,0.142,0.063,0.8357446427
27041,0.0317954715750802
"Tgs1",4.66437422238546e-05,0.154681277993505,0.174,0.085,0.8365088730
42608,0.0276134157832171
"Rpl26",4.67701255030032e-05,0.190329435526168,0.974,0.911,0.838775430
770859,0.0334622503569776
"Gdap10",4.68039659482396e-05,0.0923554292497589,0.077,0.025,0.8393823
25315729,0.0161704183329039
"Rnpc3",4.68978163421935e-05,0.131361343741863,0.194,0.097,0.841065438
280899,0.0227367848406573
"Pyroxd2",4.72254315109224e-05,-0.232413583194668,0,0.097,0.8469408887
16882,-0.0386095614276578
"Fxyd4",4.7535530332896e-05,0.616899579723594,0.271,0.159,0.8525022009
90156,0.098444519092762

"Amz2",4.77756330700622e-05,0.15305640575155,0.213,0.111,0.856808203478495,0.0236535183686789
"Sash1",4.78636512179897e-05,0.247230200694569,0.219,0.119,0.858386720943427,0.0377521894346972
"Cstf3",4.80106028785802e-05,0.142654562989552,0.174,0.084,0.861022152024457,0.0213461221854902
"Itgb6",4.8027945752734e-05,0.197036472024471,0.432,0.283,0.861333179129531,0.0294123990307287
"Tubgcp5",4.80292590777946e-05,0.108429494327609,0.129,0.054,0.861356732301169,0.0161827265514143
"Stk11",4.80906070352085e-05,0.134243641935232,0.342,0.206,0.862456946569429,0.0198640381869599
"Crbn",4.83119745370863e-05,0.169507124766515,0.187,0.093,0.866426951348107,0.0243035037975144
"Eef1d",4.84642116853092e-05,0.215782821333913,0.645,0.455,0.869157172364335,0.0302595064788679
"Lpar3",4.8559647634741e-05,-0.234341775779382,0,0.097,0.870868720681446,-0.0324010397258486
"Sra1",4.87641681502903e-05,0.129660136460837,0.49,0.318,0.874536591607306,0.0173823860317955
"Ppib",4.89375940897463e-05,0.177789663396033,0.774,0.578,0.87764681240551,0.0232035121023867
"Ttc1",4.9036109579474e-05,0.14662834886071,0.252,0.14,0.879413589198287,0.0188417383634801
"Dynlrb1",4.90647622837505e-05,0.185234677740872,0.69,0.507,0.879927446796782,0.0236944460162357
"Eif5",4.9073682216255e-05,0.202414116256728,0.658,0.483,0.880087416866318,0.0258551726394026
"Serinc3",4.93830455910832e-05,0.280844116698063,0.555,0.387,0.885635539630486,0.034108452768144
"Myef2",4.94813038098695e-05,0.211839697247775,0.181,0.089,0.8873977025262,0.0253068000634366
"Tpd52l2",4.9499138492152e-05,0.206263841246287,0.31,0.189,0.887717549718254,0.0245663661437096
"Acat2",4.96837701961402e-05,-0.328169527845624,0.058,0.181,0.891028734697578,-0.0378637413714229
"Esyt1",4.97626429707243e-05,0.100950762409232,0.097,0.036,0.892443239036969,0.0114874260100092
"Scrib",5.01421304819731e-05,0.148836646467897,0.148,0.067,0.899248968063706,0.0158057588653978
"S100a9",5.03301307024756e-05,0.289475086023544,0.213,0.113,0.902620564018197,0.0296575935234418
"Garem",5.10205988338855e-05,0.0772503617561429,0.097,0.035,0.915003419486902,0.0068619546988748
"Rrm2",5.12617742937822e-05,0.214057715460451,0.071,0.022,0.919328660184689,0.0180047353348872
"Slc39a8",5.13560726817356e-05,0.219660486477487,0.077,0.025,0.921019807474247,0.0180722889776273
"Rasa1",5.14175699240647e-05,0.15481405396785,0.194,0.099,0.922122699018177,0.0125518567444029

"Cog7",5.14301074492648e-05,0.148040764343535,0.213,0.111,0.922347546995115,0.0119666053717071
"Ctps",5.15191584575987e-05,0.124953862493757,0.129,0.055,0.923944587778575,0.00988424776099765
"Thbd",5.15355922223319e-05,0.088416119252596,0.065,0.019,0.9242393109153,0.00696579732286468
"AY036118",5.16090478637199e-05,0.292057594027285,0.477,0.343,0.925556664387952,0.0225935530264012
"Klhl9",5.18985835234338e-05,0.131459619027172,0.297,0.172,0.930749196909262,0.00943425596516224
"Acadm",5.19739586037744e-05,-0.45017093641563,0.49,0.564,0.93210097360009,-0.0316533774718062
"Paip2",5.24459930936754e-05,0.144973377555219,0.639,0.442,0.940566440141975,0.00888295221322924
"Trp53rkb",5.288419447085e-05,0.0767514278990816,0.097,0.035,0.948425143640223,0.00406417334693662
"Cdc42bpg",5.37824384105697e-05,0.173867748865221,0.161,0.076,0.964534250455156,0.00627835331314141
"Slc7a4",5.40219910794287e-05,0.0598649402870033,0.071,0.022,0.968830388018474,0.00189566647148508
"Dedd",5.46492720427507e-05,0.119248496153878,0.168,0.08,0.980080044814691,0.00239940283934214
"Gm15261",5.48332805037036e-05,0.0747495879246309,0.077,0.025,0.983380052553421,0.00125277381971385
"Sectm1b",5.50381755982006e-05,-0.274999035125853,0,0.095,0.98705464117813,-0.00358320448598963
"Gtf2a1",5.57658753545366e-05,0.151266340347297,0.123,0.051,1,0
"Brcc3",5.64588430387874e-05,0.15885264709583,0.252,0.14,1,0
"Tmem65",5.64969558548256e-05,0.155010089603794,0.161,0.076,1,0
"D3Ertd254e",5.65800675801508e-05,0.119084429869148,0.097,0.036,1,0
"Pdzd3",5.67461904717946e-05,-0.275484112268009,0.019,0.127,1,0
"Rpl8",5.68642235817258e-05,0.209328017761448,0.948,0.848,1,0
"Mbp",5.71893859667988e-05,0.0898710811444986,0.097,0.036,1,0
"Btc",5.72127232364485e-05,0.0927455915483145,0.084,0.029,1,0
"Fchsd2",5.72129814318198e-05,0.0806166803851235,0.11,0.043,1,0
"Msi2",5.73995323138649e-05,0.203753630589539,0.503,0.334,1,0
"Tfap2a",5.75648869772612e-05,0.0941609448675166,0.084,0.029,1,0
"Pdpr",5.79861746648832e-05,0.0748693403896228,0.084,0.029,1,0
"Dubr",5.8165650383053e-05,0.158895906215197,0.103,0.04,1,0
"H2afz",5.85804252971068e-05,0.210934639264066,0.742,0.596,1,0
"9330160F10Rik",5.86366830555072e-05,0.0657848088622865,0.058,0.016,1,0
"Sin3b",5.88126628362727e-05,0.129717579277669,0.387,0.241,1,0
"Ctr9",5.90234848472026e-05,0.162178200200687,0.219,0.118,1,0
"Ptprm",5.9113216070288e-05,0.0580371748456958,0.058,0.016,1,0
"Hsf1",5.93153225878341e-05,0.0766965375543991,0.11,0.043,1,0
"Fam132a",5.97042611726788e-05,-0.361649059262285,0.09,0.217,1,0
"Ssbp2",5.99340687104564e-05,0.163815476276353,0.194,0.099,1,0
"Hist1h4d",6.0212912825506e-05,0.0869630152910277,0.052,0.013,1,0
"Nifk",6.0265214782749e-05,0.122724119790297,0.194,0.097,1,0

"Vdac1",6.0577562450505e-05,-0.347092073197084,0.632,0.682,1,0
"Sh3bgrl",6.06539651994742e-05,0.151231945183265,0.323,0.194,1,0
"Grtp1",6.06595542737255e-05,-0.221875422659605,0,0.094,1,0
"Atp5h",6.09463129125977e-05,-0.236352343955383,0.91,0.881,1,0
"Fbxl6",6.11517222740577e-05,0.0912872932324736,0.123,0.051,1,0
"Sssca1",6.11580018847793e-05,0.158121729594313,0.29,0.169,1,0
"Ccl7",6.15956565726215e-05,0.0933856330753564,0.097,0.036,1,0
"Zfyve16",6.16721391611374e-05,0.159140468882772,0.181,0.09,1,0
"Snx15",6.17611692205308e-05,0.129898696933337,0.148,0.068,1,0
"Ociad1",6.18074521630837e-05,0.143414582240515,0.439,0.28,1,0
"Cib1",6.19202174822889e-05,-0.432148408513268,0.355,0.458,1,0
"Pomp",6.22355597044476e-05,0.184824434635232,0.723,0.518,1,0
"Ms4a7",6.24062396155055e-05,0.148594237154302,0.129,0.056,1,0
"Adra2b",6.25406779495303e-05,-0.238588476790337,0.006,0.105,1,0
"Elk4",6.257499310755e-05,0.138202498776454,0.187,0.094,1,0
"Tbcc",6.26107048336349e-05,0.119104186019353,0.187,0.093,1,0
"Dapk3",6.2745332144585e-05,0.137157581840168,0.219,0.116,1,0
"Ube2r2",6.27840460890458e-05,0.206796235889359,0.471,0.317,1,0
"Rab28",6.28842178811237e-05,0.155134091484446,0.213,0.112,1,0
"Nek8",6.30898319052483e-05,0.118077728388548,0.097,0.036,1,0
"Spata13",6.30947876826373e-05,0.158641381714168,0.168,0.081,1,0
"Gadd45g",6.32685927207245e-05,0.282393546998115,0.277,0.161,1,0
"Nae1",6.33969326456632e-05,0.169623084536411,0.232,0.127,1,0
"4933427D14Rik",6.39545867981323e-05,0.0870044053181408,0.084,0.029,1,
0
"Sh3bp4",6.409728840019e-05,0.118396068653967,0.142,0.064,1,0
"Ppp1r11",6.42126712099857e-05,0.187278633658009,0.381,0.24,1,0
"Blcap",6.43709131873846e-05,0.123312310495955,0.168,0.081,1,0
"Erich2",6.47016070584756e-05,0.0857628826199213,0.065,0.019,1,0
"Polr2m",6.49652931801823e-05,0.166035002945622,0.471,0.307,1,0
"Rwdd4a",6.5054279485011e-05,0.130380852558009,0.232,0.127,1,0
"Cbs",6.53937746878304e-05,-0.278880501006629,0.019,0.125,1,0
"Atp6v0e",6.58374471925836e-05,0.124022721607355,0.787,0.622,1,0
"Mcm7",6.58486235836201e-05,0.153968205220754,0.129,0.055,1,0
"Tmem222",6.58687167605977e-05,0.142242256728506,0.174,0.085,1,0
"Gngt2",6.63754708143564e-05,0.131821784789857,0.148,0.068,1,0
"Depdc5",6.65833816170279e-05,0.0809925178541378,0.084,0.029,1,0
"Ahsa1",6.68349482361007e-05,0.109584448882854,0.361,0.219,1,0
"Igbp1",6.6976389580321e-05,0.132260420332722,0.342,0.208,1,0
"Btf3l4",6.73050678883553e-05,0.138438622152133,0.439,0.282,1,0
"Synrg",6.75439278879806e-05,0.153258227780904,0.232,0.128,1,0
"Gas8",6.7685537610653e-05,0.105593827838673,0.11,0.044,1,0
"Tmem64",6.84513234619345e-05,-0.360321310610488,0.135,0.269,1,0
"Gjb2",6.88099678420235e-05,-0.254629092179013,0.019,0.126,1,0
"Rybp",6.95626796153505e-05,0.13581930696559,0.142,0.065,1,0
"Rbm17",6.98136470413504e-05,0.149270595807072,0.265,0.152,1,0
"Foxj3",7.02616828913236e-05,0.119839126497819,0.161,0.076,1,0
"Gca",7.06090621318511e-05,0.116226715660758,0.161,0.077,1,0
"Lin7c",7.06505117583371e-05,0.182224649357013,0.271,0.159,1,0
"Rusc1",7.07033225392543e-05,0.147478386389987,0.097,0.037,1,0

"Dcun1d2",7.08986398803292e-05,0.0897290040971635,0.103,0.039,1,0
"Zfp84",7.12404139054041e-05,0.0958251981522562,0.11,0.043,1,0
"1700029J07Rik",7.15496562523873e-05,0.0801264162589799,0.09,0.033,1,0
"B3gat3",7.18869362231388e-05,0.196332926755917,0.213,0.114,1,0
"Ndufb5",7.22292850816914e-05,-0.310024865875684,0.69,0.721,1,0
"Tmem229a",7.23749238179624e-05,-0.286563857142974,0.142,0.287,1,0
"Abcc3",7.24795741173077e-05,0.0968454602335876,0.097,0.036,1,0
"Rps5",7.26535436089647e-05,0.270267394217675,0.923,0.868,1,0
"Ighm",7.27106224126066e-05,0.166156324859038,0.11,0.044,1,0
"Adnp2",7.32161085143262e-05,0.0878695210070362,0.077,0.026,1,0
"Srcap",7.36469876502875e-05,0.149851717326586,0.129,0.056,1,0
"Myo19",7.36629454504145e-05,0.109622621002637,0.148,0.068,1,0
"Dnttip2",7.41696485813414e-05,0.174897189835057,0.181,0.092,1,0
"Qprt",7.46890013366531e-05,-0.237596200914892,0.019,0.126,1,0
"Kctd15",7.51441244522637e-05,0.077370880550562,0.071,0.023,1,0
"Dnajb11",7.5259393208053e-05,0.182780273069615,0.4,0.261,1,0
"Lsm11",7.52846705495809e-05,0.0868672006968688,0.065,0.019,1,0
"Isg20l2",7.54391784617796e-05,0.0979885273426867,0.09,0.033,1,0
"Ambra1",7.54959856977557e-05,0.12610593010416,0.123,0.052,1,0
"Ten1",7.57176897176823e-05,0.183742948708507,0.194,0.102,1,0
"Slc4a2",7.57557349512515e-05,0.17331852768271,0.213,0.113,1,0
"Mat2a",7.67106298866781e-05,0.277821612254548,0.574,0.401,1,0
"Setbp1",7.68853229723332e-05,0.105454532497406,0.116,0.048,1,0
"Fam65a",7.69859657893994e-05,0.109917522820733,0.161,0.077,1,0
"Acbd3",7.73322439926743e-05,0.197194489190158,0.2,0.106,1,0
"Mapk1ip1l",7.75651438813775e-05,0.138977148974663,0.265,0.148,1,0
"Afap1",7.814246855722e-05,0.0926557359576429,0.142,0.063,1,0
"Ppp1ca",7.82459803151508e-05,0.158990493664916,0.697,0.504,1,0
"Ppm1j",7.84928911416911e-05,0.0618068246717931,0.065,0.019,1,0
"Kri1",7.89459200055735e-05,0.0980535616873037,0.097,0.037,1,0
"Map2k3",7.94452759958792e-05,0.10241198876836,0.213,0.112,1,0
"Rab22a",7.96365564627051e-05,0.173642936132042,0.277,0.163,1,0
"Slc5a8",7.98909891039525e-05,-0.369525021082613,0.013,0.112,1,0
"Smco4",8.02659490489661e-05,0.148413963851413,0.252,0.14,1,0
"Cd36",8.05420581485365e-05,-0.411116721529386,0.045,0.158,1,0
"Pogz",8.1419638532679e-05,0.1233438282112,0.148,0.069,1,0
"Rpl3",8.1708715192369e-05,0.247542134168102,0.91,0.792,1,0
"Zfp40",8.19180841290562e-05,0.0852557040594097,0.065,0.02,1,0
"Taf1a",8.19922786453104e-05,0.0583441823253125,0.065,0.019,1,0
"Fhod3",8.22624638098496e-05,0.152165473508049,0.155,0.073,1,0
"Snhg20",8.27491133668563e-05,0.126568043782171,0.213,0.112,1,0
"4930402H24Rik",8.30691739959102e-05,0.155571002325132,0.342,0.209,1,0
"Lrrc16a",8.32985278676664e-05,0.175552593634007,0.168,0.083,1,0
"BC089597",8.34192058959632e-05,-0.284703990116099,0,0.091,1,0
"Pigyl",8.3735834737552e-05,0.164496533521258,0.348,0.217,1,0
"Pde3b",8.4126834912013e-05,0.110520188678939,0.123,0.052,1,0
"Ccng2",8.48782449557491e-05,0.128271999133398,0.142,0.064,1,0
"Larp7",8.51061124478286e-05,0.167429607804977,0.187,0.095,1,0
"Cldn34c1",8.51201800391882e-05,0.0652677198781104,0.052,0.014,1,0
"Dnajb14",8.55199346930988e-05,0.132800506674124,0.297,0.176,1,0

"Dcp1a",8.60650284603904e-05,0.0756114224732937,0.077,0.026,1,0
"Rfwd2",8.71409060075497e-05,0.175283441512126,0.271,0.158,1,0
"Pik3c2a",8.75749851711995e-05,0.148536676539368,0.29,0.171,1,0
"Adprhl2",8.77891457920725e-05,0.129184897157987,0.168,0.082,1,0
"Nek9",8.82098215526575e-05,0.10761969552896 7,0.187,0.094,1,0
"Kdm7a",8.84605356957243e-05,0.162808469241567,0.187,0.096,1,0
"Dusp6",8.93484795274986e-05,0.147351169735746,0.148,0.069,1,0
"Stub1",8.95317654370694e-05,0.140221239335628,0.503,0.345,1,0
"Limd1",8.97646908548671e-05,0.1066479377487,0.277,0.158,1,0
"Psmb3",8.99191287824402e-05,0.14898273803434,0.69,0.502,1,0
"Ces1e",9.07038274701345e-05,-0.250480515499483,0.006,0.101,1,0
"Snrpa",9.12677826709159e-05,0.133234674960769,0.245,0.138,1,0
"Prkab1",9.16793930423049e-05,0.159656993324133,0.226,0.124,1,0
"Podn",9.18036294505137e-05,0.13539325784549,0.052,0.014,1,0
"Tm9sf1",9.25101644646389e-05,0.0616344009121026,0.058,0.017,1,0
"Pmaip1",9.34603714409725e-05,0.226129171582766,0.129,0.056,1,0
"Tmem178",9.40351174923155e-05,0.156459488856226,0.213,0.113,1,0
"Fmnl2",9.43257985401817e-05,0.109543415178297,0.071,0.023,1,0
"Kdm4a",9.46028138823966e-05,0.126391550201691,0.148,0.069,1,0
"Mrpl34",9.46931510175774e-05,-0.388989850988945,0.4,0.509,1,0
"Rpl18a",9.52171816776582e-05,0.210651528610815,0.981,0.919,1,0
"Parn",9.56691513755884e-05,0.094672429630049,0.097,0.037,1,0
"Polr3d",9.64081319819033e-05,0.11425128722411,0.129,0.056,1,0
"P3h4",9.64762436587468e-05,0.0688426209813382,0.065,0.02,1,0
"Zmiz1",9.64826673797447e-05,0.17772215472335,0.323,0.199,1,0
"Plac8",9.66012055163512e-05,0.105665398056356,0.342,0.208,1,0
"Clip1",9.69648258539238e-05,0.180973206009093,0.232,0.127,1,0
"Rcc2",9.73633492216915e-05,0.111636884748009,0.142,0.065,1,0
"Smad6",9.79241738749182e-05,0.13074248205195,0.09,0.034,1,0
"Cav2",9.8289725896871e-05,0.212480572775524,0.213,0.113,1,0
"Pum2",9.84380322854404e-05,0.169177495405136,0.406,0.271,1,0
"Adamts10",9.85073300274719e-05,0.0898393647878194,0.058,0.017,1,0
"Vps37a",9.8570604163693e-05,0.155044294012971,0.226,0.125,1,0
"Trac",9.93865727557602e-05,0.120178731631003,0.065,0.02,1,0
"Ggt1",9.99535298001886e-05,-0.508506806915813,0.419,0.503,1,0
"Leng8",0.000100405508798982,0.127044532724764,0.2,0.104,1,0
"Galnt18",0.00010040707192898,0.154381800086517,0.142,0.064,1,0
"Bfar",0.000100511616740897,0.104144657698086,0.2,0.104,1,0
"Uxs1",0.000100628047459569,0.159814051738302,0.206,0.11,1,0
"Hmha1",0.000101708208324553,0.0995054293041786,0.077,0.026,1,0
"Lsm2",0.000102479663193438,0.110843368690013,0.258,0.146,1,0
"Ruvbl1",0.000102577622741464,0.102311943579706,0.155,0.073,1,0
"Fam195b",0.000102600294014667,0.133961594453147,0.335,0.203,1,0
"Herc6",0.000102634860064056,0.140837501685017,0.09,0.034,1,0
"1110008P14Rik",0.00010268273213713,0.128519751456094,0.445,0.285,1,0
"Fem1b",0.000102732302042949,0.137933235131416,0.155,0.074,1,0
"Dcaf5",0.000102988174645445,0.125692129443409,0.123,0.053,1,0
"Anp32e",0.0001035223249476,0.145482005143878,0.316,0.193,1,0
"Decr1",0.00010405043291978,-0.341224572566408,0.148,0.281,1,0
"Fahd1",0.000104606029870338,-0.40521853255157,0.245,0.369,1,0

"Ephb4",0.000105267663839264,0.176410726364898,0.135,0.062,1,0
"Macrod2",0.000105609714295555,-0.322137330481117,0.103,0.23,1,0
"Ackr3",0.000105896461750833,0.201293583824815,0.161,0.079,1,0
"Crtc1",0.0001060229253492,0.0671820701877464,0.071,0.023,1,0
"Eef1b2",0.000106189982257857,0.17671789610708,0.658,0.482,1,0
"Mgea5",0.000106214944740357,0.161133630601586,0.316,0.192,1,0
"Fnta",0.000106407037304797,0.168841425165881,0.265,0.156,1,0
"Nmi",0.000106650575464184,0.155411773690448,0.168,0.083,1,0
"Slc22a22",0.000106937068990299,-0.224958693016921,0,0.089,1,0
"Zfp706",0.000107120147575839,-0.38087216844072,0.561,0.605,1,0
"Fiz1",0.000107167803718479,0.130436130587523,0.123,0.053,1,0
"Taf6",0.000107361926336972,0.115617784433333,0.168,0.082,1,0
"PISD",0.000107518353417322,0.1289777054803,0.239,0.131,1,0
"Dnm1l",0.000107813616897131,0.198255073684756,0.265,0.157,1,0
"Eif1",0.000109836156059279,0.210336334613256,0.948,0.886,1,0
"Il10rb",0.000110423006900892,0.205181485388856,0.245,0.139,1,0
"Marf1",0.000110469432965563,0.170924563521669,0.226,0.125,1,0
"Nck2",0.000110664569008597,0.0597879261705963,0.077,0.026,1,0
"Gm28875",0.000111467860992721,0.0733598669886459,0.052,0.014,1,0
"U2af2",0.000111576543557441,0.173642641872689,0.284,0.166,1,0
"H2-DMa",0.000112476570430223,0.173522871095379,0.252,0.145,1,0
"Clk3",0.000112943475054604,0.097884884786168,0.155,0.073,1,0
"Enpp1",0.000113040541474059,0.129354336616259,0.135,0.06,1,0
"Cyb561",0.000113087040630736,0.124804118867178,0.2,0.105,1,0
"Strn4",0.000113119013397111,0.148336299229113,0.213,0.115,1,0
"Zfp865",0.000113153954787768,0.101226556611061,0.142,0.065,1,0
"Adamts16",0.000113755593843457,0.0892613139132146,0.071,0.023,1,0
"Slbp",0.000114112942863523,0.139262024459465,0.219,0.12,1,0
"Dusp16",0.000114464306660817,0.121429645045355,0.116,0.049,1,0
"Pfdn2",0.000115114571352697,0.117388614802583,0.587,0.396,1,0
"Gprasp1",0.000115354223417007,0.130376241154455,0.116,0.049,1,0
"Mrpl24",0.000115383532586332,0.141464251864792,0.458,0.307,1,0
"Man2a2",0.000115594732841271,0.108679415102035,0.116,0.049,1,0
"Mbd2",0.000116191530655022,0.129848226753328,0.277,0.163,1,0
"Rnf113a2",0.000116507980921871,0.12388909220433,0.129,0.057,1,0
"Hist3h2a",0.000117364479643222,0.252626713131312,0.219,0.12,1,0
"Ip6k1",0.000117545837733766,0.135465646686483,0.206,0.11,1,0
"Dhfr",0.000117687365647959,-0.297288195430671,0.045,0.157,1,0
"Dnajc22",0.00011775415687666,-0.198628737910447,0,0.088,1,0
"Anapc10",0.00011829579164149,0.143980018479723,0.148,0.07,1,0
"Zfp958",0.000118473420513519,0.0789862040186877,0.065,0.02,1,0
"Hoxd3os1",0.00011881433380336,0.156964968776331,0.213,0.115,1,0
"Mpc2",0.000118978687941077,-0.23138748690965,0.865,0.861,1,0
"Zfyve27",0.000119015399958871,0.131838651237178,0.142,0.066,1,0
"Tmigd1",0.000119679602261722,-0.433200986002071,0.045,0.155,1,0
"Ilk",0.000119743237759146,0.14986446186546,0.31,0.188,1,0
"Dpyd",0.000120035075917914,-0.204723186488451,0.006,0.098,1,0
"Nckap1",0.000120072288572989,0.112019011761387,0.394,0.249,1,0
"Tmem5",0.000120495468335576,0.156590064621366,0.239,0.134,1,0
"Ift81",0.000121355186754843,0.130311188199326,0.142,0.066,1,0

"Rplp1",0.000121369626419101,0.187462621424885,0.987,0.905,1,0
"Smc5",0.000121432935234591,0.169166803028319,0.129,0.058,1,0
"Atp6v1b2",0.000121550150859239,-0.367503738236574,0.271,0.398,1,0
"Swap70",0.000121812923881961,0.0981593681454251,0.097,0.037,1,0
"Igsf8",0.000121999319046686,0.126578893961539,0.135,0.062,1,0
"Ubqln4",0.000122450751180331,0.131326105854062,0.187,0.097,1,0
"Lifr",0.000122764548270689,0.209497188722517,0.206,0.113,1,0
"Zfp622",0.000123517267253558,0.124177307026016,0.194,0.102,1,0
"Ifrd2",0.000123908864743439,-0.255776061732986,0.032,0.138,1,0
"Phldb1",0.00012410544801547,0.0873157720409338,0.097,0.038,1,0
"Myo1d",0.000124169723909807,0.109483360606591,0.142,0.065,1,0
"Cox7a1",0.000124208215883206,-0.32159331172224,0.103,0.229,1,0
"Gng5",0.000124244670577213,0.19191606363546,0.884,0.774,1,0
"Gspt2",0.000124274919587915,0.0595434325586861,0.052,0.014,1,0
"Atat1",0.000124574357735043,0.126820817555704,0.174,0.087,1,0
"Aadat",0.000125173994474148,-0.247717024529993,0.006,0.099,1,0
"Anpep",0.000125289665781205,-0.214233402300194,0.013,0.109,1,0
"Ppfibp1",0.000125955884824301,0.154303036531981,0.226,0.125,1,0
"Ino80b",0.00012612512896741,0.152519707738186,0.213,0.115,1,0
"Cxcl10",0.000126676689027958,0.219853412570663,0.084,0.03,1,0
"Ppp4c",0.000127191280307198,0.194302325443307,0.303,0.188,1,0
"Exosc1",0.000127973712630528,0.0876222017510299,0.142,0.065,1,0
"Camk2b",0.000128355583978964,0.0624898278494543,0.058,0.017,1,0
"Shroom4",0.000128476012029155,0.101815991659059,0.155,0.073,1,0
"Rad52",0.000129266263301618,0.111308225900806,0.077,0.027,1,0
"Ftx",0.000129504956693378,0.078006708273299,0.058,0.017,1,0
"Ick",0.000129733185745325,0.110849624063083,0.097,0.038,1,0
"9530068E07Rik",0.000129827170738727,0.184396724690869,0.432,0.288,1,0
"D10Wsu102e",0.000130257329427801,0.0842437522532888,0.123,0.052,1,0
"Sept10",0.000130575054093395,0.12461042650879,0.129,0.057,1,0
"Morc3",0.00013171288842177,0.110411239288383,0.135,0.062,1,0
"Dgcr8",0.000132353892089799,0.140174275858778,0.116,0.05,1,0
"Fam103a1",0.000132405969207199,0.16017744202529,0.445,0.301,1,0
"Prss23",0.000133493865934685,0.224235240565528,0.265,0.155,1,0
"Arhgdia",0.000133980786374272,0.18885615170791,0.497,0.348,1,0
"Tbca",0.000134108815597496,0.175770687683541,0.787,0.637,1,0
"E2f5",0.000134192282851447,0.125684311508568,0.206,0.11,1,0
"Terf2ip",0.000134693060509183,0.126299203569161,0.161,0.079,1,0
"Uba2",0.000135390781134082,0.145415658507892,0.2,0.108,1,0
"Sdhd",0.000136018183434633,-0.385298980523683,0.361,0.454,1,0
"Cd59b",0.000136163111314521,0.113474505204909,0.09,0.034,1,0
"Hmgn5",0.00013634494167424,0.0985521021185558,0.258,0.145,1,0
"Hspa14",0.000136918942972752,0.109657102164622,0.168,0.083,1,0
"Dpysl2",0.000137009971698366,0.105217006402692,0.116,0.049,1,0
"Gpihbp1",0.000137372299997761,0.0967793961004509,0.103,0.041,1,0
"Pfn1",0.000138034645180385,0.222668571307219,0.877,0.765,1,0
"Brpf1",0.000138297661817977,0.0980863919581641,0.103,0.041,1,0
"Galnt7",0.000138456388985195,0.12883638301731,0.116,0.05,1,0
"Abcf1",0.000140079944020947,0.159063217157039,0.342,0.213,1,0
"Spast",0.000140121486573045,0.0875856453851549,0.123,0.053,1,0

"Fbxo3",0.000140261241723857,0.132330476914225,0.265,0.155,1,0
"Asxl1",0.000140447645715035,0.132860221989227,0.148,0.071,1,0
"Tmem107",0.000140911607274483,0.129175145755593,0.174,0.087,1,0
"Hlf",0.000140928109044752,-0.21188802375375,0.013,0.109,1,0
"Vps13a",0.000140976035862445,0.139968940996597,0.161,0.08,1,0
"Dsel",0.000141294719433228,0.0551214616330252,0.052,0.014,1,0
"Ppip5k2",0.000141327651506396,0.123771643236451,0.123,0.053,1,0
"Trp53i11",0.000141505072557383,0.0593537581978801,0.052,0.014,1,0
"Acp5",0.000141889455453642,-0.247897785623757,0.019,0.117,1,0
"BC067074",0.00014202328765987,0.0587591337662693,0.058,0.017,1,0
"Phlda1",0.000142160936123766,0.428572909069581,0.155,0.075,1,0
"Alyref2",0.000142486748495967,0.08867062733445,0.077,0.027,1,0
"Tpgs2",0.000142615145191306,0.0841242051189707,0.123,0.053,1,0
"Trpm4",0.0001428958602421,0.087867366629698,0.065,0.02,1,0
"Copb2",0.000143004703871452,0.137776657266936,0.342,0.216,1,0
"Ythdc2",0.000143100039563339,0.0730383756769891,0.103,0.041,1,0
"Ogfr",0.000143220172081987,0.135877859585684,0.187,0.098,1,0
"Znhit1",0.000143519266111706,0.150665689489148,0.445,0.3,1,0
"Sp1",0.000143626958184569,0.163164516783985,0.168,0.084,1,0
"Rev3l",0.000143929732531757,0.191533253470771,0.174,0.09,1,0
"Fgfr1op2",0.000144086326254884,0.152918688650054,0.406,0.266,1,0
"Ssr2",0.000144130606064066,0.148824648402078,0.452,0.302,1,0
"Rnf152",0.000144622932176903,-0.287718598007402,0.032,0.136,1,0
"Slc31a2",0.000144745467640438,0.220857022677999,0.148,0.071,1,0
"Ndufb11",0.000145237626092301,-0.247796438707106,0.768,0.771,1,0
"Tle1",0.000147348680480796,0.0995866501374964,0.097,0.038,1,0
"Zfp653",0.000147844838696732,0.0544581353474173,0.052,0.014,1,0
"Schip1.1",0.000148248710809825,0.0944186388065251,0.084,0.03,1,0
"Casp7",0.000148353342156164,0.0644090521063655,0.09,0.034,1,0
"Ccdc120",0.000148664147642703,0.0904482201509734,0.058,0.017,1,0
"Mettl9",0.000148718740651622,0.153040126026787,0.394,0.259,1,0
"Atp13a2",0.00015031598838398,0.0792425726213573,0.11,0.045,1,0
"Trim63",0.000150529057410573,-0.2207330007186,0.006,0.096,1,0
"Spag7",0.000150605364753168,0.169749442324066,0.387,0.255,1,0
"Zbtb11",0.000150985136607555,0.0914254281941829,0.116,0.049,1,0
"2510046G10Rik",0.000151412972603614,0.0779992083395817,0.077,0.027,1,
0
"Arhgef38",0.000151816218035379,0.118828364924541,0.11,0.046,1,0
"Cops4",0.000151845973300547,0.112298833309757,0.277,0.161,1,0
"Zfp788",0.00015193108056338,0.0802908349700427,0.071,0.024,1,0
"Ildr2",0.000152472360251668,-0.244727416615721,0.032,0.136,1,0
"Spred3",0.000154581322853822,0.0542394219966778,0.052,0.014,1,0
"Gt(ROSA)26Sor",0.000155253304053316,0.174114722731162,0.181,0.093,1,0
"Srsf9",0.000155967184270435,0.124020612217928,0.323,0.198,1,0
"Atxn2l",0.0001563481017645,0.128055835486011,0.168,0.084,1,0
"Sdha",0.000156660302721717,-0.349082916057144,0.626,0.646,1,0
"Clrn3",0.00015710759271242,-0.200019156835732,0,0.085,1,0
"Maged2",0.000157319955820222,0.117452948168422,0.155,0.076,1,0
"Nhsl2",0.000157655902232254,0.106333099214687,0.084,0.031,1,0
"Commd7",0.000159162258833298,0.152955494494766,0.206,0.113,1,0

"Thsd4",0.000161077810155973,0.0511089349259067,0.058,0.017,1,0
"Ubb",0.000161446273625904,0.214654027049268,0.961,0.943,1,0
"2010107G23Rik",0.000161841633439438,0.068135955352618,0.09,0.034,1,0
"Shroom2",0.000162207099280783,0.0975598394984236,0.116,0.05,1,0
"Pold4",0.000162656001160543,0.129172203879792,0.219,0.121,1,0
"Dapp1",0.000163781891031092,0.0898375214480556,0.077,0.027,1,0
"Zfp74",0.0001651651308002,0.0956497535950124,0.084,0.031,1,0
"Lta4h",0.000166463383639577,0.074280113538037,0.142,0.066,1,0
"Cables2",0.000167378669225296,0.0551965831321122,0.077,0.027,1,0
"Sgpl1",0.000167436469881253,0.0995825533627836,0.2,0.106,1,0
"Ablim1",0.00016794754420873,0.134554237093519,0.232,0.131,1,0
"Ppia",0.000168006073690928,0.170861993993566,0.974,0.936,1,0
"Heatr1",0.000169148831787592,0.0861825058683128,0.084,0.031,1,0
"Ipo5",0.000169421836579363,0.10523870350006,0.2,0.106,1,0
"Cdk20",0.000169697345706596,0.057568128662706,0.052,0.014,1,0
"Ncoa6",0.000169750886324357,0.127790009391152,0.155,0.076,1,0
"Arl10",0.000169866556073071,0.0767956609033441,0.058,0.017,1,0
"Pnpla6",0.000171067891291682,0.118262523110756,0.155,0.074,1,0
"Klf16",0.000171346087088892,0.0872395765225644,0.058,0.017,1,0
"Cfap36",0.00017145422726183,0.143318367836312,0.284,0.17,1,0
"Sypl2",0.000171661305383217,-0.210694633696835,0.006,0.095,1,0
"Peli1",0.000171819870698783,0.2172842147244,0.174,0.091,1,0
"Stx4a",0.000171979103207562,0.181640956949251,0.252,0.149,1,0
"Whsc1",0.000172054363692432,0.147399204720261,0.161,0.081,1,0
"Lactb",0.00017333753101318,0.12640405194133,0.29,0.172,1,0
"Bhlhb9",0.00017451064127729,0.0654237121807745,0.09,0.034,1,0
"Mon2",0.000174630505582739,0.135718215594604,0.181,0.094,1,0
"Plcd1",0.000174632194271553,0.0680682763111759,0.058,0.017,1,0
"Bahd1",0.000174694632250688,0.0892649490235018,0.11,0.046,1,0
"Mphosph8",0.000175996830891357,0.161789803928932,0.213,0.118,1,0
"Rab27b",0.000176016383282313,0.0865534207041916,0.065,0.021,1,0
"Clasp2",0.000177178491075334,0.087313364230567,0.103,0.042,1,0
"Scarb2",0.000177218854092069,0.13042213155184,0.135,0.063,1,0
"Armc8",0.000178030916507911,0.0702839818493366,0.174,0.087,1,0
"Bach1",0.000178114351047724,0.112475134086129,0.123,0.054,1,0
"Ikzf5",0.00017878126632563,0.130082383067269,0.155,0.076,1,0
"Phf6",0.000179059143962326,0.0968168522538487,0.11,0.046,1,0
"Eapp",0.000179111226414088,0.161787856194976,0.316,0.196,1,0
"BC005624",0.000179196878238666,0.177241207884393,0.265,0.157,1,0
"Gtf2a2",0.000179256314482187,0.0981468081686812,0.394,0.251,1,0
"Eif3m",0.000179612932273188,0.117145594918281,0.497,0.324,1,0
"Ddost",0.000179677781138285,0.159981924763726,0.387,0.256,1,0
"Zkscan3",0.000179722820390159,0.134107177400443,0.265,0.156,1,0
"Setd4",0.00017988188588144,0.0758754002317689,0.058,0.017,1,0
"Ephb3",0.000180278195652542,0.0920829869795491,0.065,0.021,1,0
"Atf2",0.000180321132221994,0.146298914339682,0.232,0.132,1,0
"Rab3gap2",0.000180930880976348,0.0976739123798925,0.11,0.046,1,0
"Dcaf17",0.000181098258417926,0.132786156356123,0.168,0.085,1,0
"Ctbp1",0.000181243041855986,0.18737408028321,0.374,0.25,1,0
"Clk4",0.000182575006411902,0.143320653626965,0.226,0.128,1,0

"Foxj2",0.000182933477749657,0.100143299081967,0.103,0.042,1,0
"Spryd7",0.000183023822562846,0.131111955988246,0.148,0.071,1,0
"Fgd3",0.000183504353818075,0.0639892833318716,0.058,0.017,1,0
"Glt28d2",0.000183568371233023,0.0717305348992858,0.058,0.018,1,0
"1700020I14Rik",0.000184366307317802,0.216433624995244,0.4,0.274,1,0
"Armcx5",0.000184863060614566,0.0688309069242573,0.065,0.021,1,0
"D1Ertd622e",0.000185342464478739,0.119661727561801,0.2,0.107,1,0
"Trove2",0.000185968211810306,0.157017476417888,0.206,0.115,1,0
"AW112010",0.000186105823663183,0.148574500588434,0.194,0.103,1,0
"Kl",0.000187783295135239,-0.266391699943125,0.31,0.467,1,0
"Gm42726",0.00018782025210161,0.0807488874738889,0.11,0.046,1,0
"Zfp949",0.000187850454919296,0.0821923996949156,0.09,0.034,1,0
"Gripap1",0.000188143631083202,0.129400451527145,0.181,0.093,1,0
"Ash2l",0.000188409853652508,0.161461185243784,0.174,0.091,1,0
"Dbnl",0.000188745649722892,0.14583316721523,0.245,0.142,1,0
"Amotl2",0.000188961863808371,0.178221979068858,0.174,0.09,1,0
"Sec22a",0.000189548363652824,0.163382454312268,0.181,0.095,1,0
"Ank2",0.000189568903258014,0.0602178276286725,0.058,0.017,1,0
"Impad1",0.000189648410998445,0.169424666300418,0.31,0.194,1,0
"Gstz1",0.000190574355622353,-0.375194378940957,0.155,0.272,1,0
"Tbc1d17",0.000190698646697138,0.154030881012088,0.194,0.105,1,0
"Gm20186",0.000192168100318926,0.115252998809405,0.077,0.028,1,0
"Scnn1b",0.000192177823391598,0.236808258189489,0.161,0.08,1,0
"Cnbp",0.000192295728717726,0.169703743027943,0.568,0.407,1,0
"Ap3s1",0.000192474404276954,0.152003248828566,0.31,0.191,1,0
"Alkbh6",0.000193273935464784,0.104379537297845,0.219,0.121,1,0
"Cks2",0.000193718712025938,-0.390893033547591,0.219,0.34,1,0
"Ccdc166",0.000194043379580588,0.0679459546370423,0.058,0.018,1,0
"Srpr",0.000194872298589409,0.152783109965143,0.342,0.217,1,0
"Cbl",0.000195143443558583,0.156397535990984,0.206,0.115,1,0
"Smc4",0.000195234178501536,0.13129723124155,0.155,0.076,1,0
"Exoc8",0.000196597263316075,0.0939243787459329,0.123,0.054,1,0
"Jkamp",0.000196875682856179,0.114320115564631,0.265,0.155,1,0
"Rnf146",0.000197317772909852,0.132318697664524,0.2,0.108,1,0
"Bspry",0.000198672139925156,0.106897058170683,0.265,0.152,1,0
"Ndnl2",0.000199244834077302,0.146624320881258,0.187,0.099,1,0
"Slc9a3r1",0.000199362555394626,-0.365188409339206,0.323,0.435,1,0
"Ghitm",0.000200235967260835,-0.341980483079664,0.471,0.563,1,0
"Lrch3",0.00020052981363528,0.12606012592743,0.123,0.055,1,0
"Terf1",0.000201455266610398,0.126788790253214,0.135,0.063,1,0
"Pqbp1",0.000202878920844043,0.154301384512876,0.181,0.095,1,0
"Fam210b",0.000202997800395808,0.0938726437211325,0.116,0.05,1,0
"Mettl3",0.000204623474902325,0.108079710293633,0.116,0.05,1,0
"Nsun6",0.000204950056369063,0.114805487345328,0.09,0.035,1,0
"Crtc3",0.000205053625863016,0.112882235241003,0.135,0.063,1,0
"Vwa2",0.000205445570325665,0.0670188458464271,0.071,0.024,1,0
"Adgrl4",0.000207384634775196,0.110653494032309,0.065,0.021,1,0
"Qtrtd1",0.000207450090560761,0.0664717868837922,0.058,0.018,1,0
"Cpne8",0.000208931898718296,0.127332649262199,0.116,0.051,1,0
"Baz1a",0.000210013890851063,0.134779578748924,0.135,0.063,1,0

"Sike1",0.000210381562558217,0.178259958338067,0.213,0.121,1,0
"Ndufb3",0.000210566428774791,-0.314353876733933,0.677,0.696,1,0
"Fra10ac1",0.000211412138839836,0.0757891861867565,0.155,0.075,1,0
"Dnpep",0.000211444485578287,0.0983962699664599,0.232,0.129,1,0
"Exoc4",0.000211999341899996,0.126004601162132,0.194,0.103,1,0
"Lyn",0.000212976299210475,0.108355719987545,0.116,0.05,1,0
"Ube2e2",0.000213323166348269,0.111449274566433,0.142,0.068,1,0
"Gchfr",0.000213475948069977,-0.295514756035402,0.045,0.152,1,0
"Cnppd1",0.000213949089251199,0.114621993561742,0.219,0.12,1,0
"Pitpna",0.000214646603364415,0.148559632520655,0.348,0.222,1,0
"Rnf8",0.000215154605160703,0.101735054925849,0.11,0.047,1,0
"Net1",0.00021673194054246,0.212054586023317,0.323,0.21,1,0
"Egln3",0.000217407294205791,0.0802138824625763,0.065,0.021,1,0
"Tceb1",0.000217588910668721,0.133182062054273,0.594,0.432,1,0
"Cdc25b",0.000217695357878937,0.0775068778883454,0.084,0.031,1,0
"Clptm1l",0.00021813466946124,0.14579019879381,0.335,0.215,1,0
"Timm8b",0.000218857788250244,-0.298450425789196,0.639,0.662,1,0
"Midn",0.000220343400527394,0.144117280943275,0.258,0.154,1,0
"Cct4",0.000220597285447693,0.167164571070019,0.477,0.329,1,0
"Rbx1",0.000220849407612641,0.132665630734782,0.8,0.604,1,0
"Neurl1b",0.000220987285518632,0.179849883324338,0.135,0.065,1,0
"Smim5",0.00022100515825716,0.186498807168863,0.232,0.13,1,0
"Hnrnpul1",0.000222785598232534,0.141735977425404,0.29,0.177,1,0
"Wnt5a",0.000223425566849377,0.103541120323471,0.071,0.024,1,0
"Syncrip",0.000224214951744158,0.199294491659027,0.303,0.187,1,0
"Tubb2a",0.000224546737245092,0.170225592873815,0.226,0.128,1,0
"Rgs3",0.000224946521927232,0.0648025037091802,0.058,0.018,1,0
"Fam19a5",0.000225093067178308,0.0598651194935108,0.058,0.018,1,0
"B9d1",0.00022700052706114,0.157060416444126,0.206,0.115,1,0
"Acot12",0.000227381767426208,-0.214868245254462,0.006,0.093,1,0
"Pkp2",0.000227868691647751,0.11299151630816,0.181,0.095,1,0
"S100a1",0.000228386705373337,0.257833373490804,0.871,0.767,1,0
"Tmem68",0.00022978754679491,0.130362560631756,0.116,0.051,1,0
"Tbc1d32",0.000229798351824672,0.116265586501537,0.103,0.043,1,0
"Triobp",0.000230053215358327,0.0883014185461534,0.187,0.098,1,0
"2210404O09Rik",0.000230222782818612,0.0780763829863236,0.058,0.018,1,
0
"Tmed5",0.000230510235074624,0.144030612729733,0.258,0.152,1,0
"Dync1li2",0.000231113358950995,0.10481535850808,0.181,0.094,1,0
"Tank",0.000232025265418427,0.0982815843770183,0.116,0.051,1,0
"Cchcr1",0.000233001545746567,0.102969124097486,0.09,0.035,1,0
"Aldoa",0.000233553057627068,0.220850900140797,0.935,0.807,1,0
"Nosip",0.000235218092989589,0.0917520185374174,0.155,0.076,1,0
"Lims1",0.000235906150856143,0.129203952185112,0.245,0.142,1,0
"B3galt6",0.00023627775227907,0.0913763066380308,0.084,0.032,1,0
"Sh3gl2",0.000237142969146241,0.092481712704685,0.116,0.051,1,0
"Pdk2",0.000237229170864923,-0.299177097553285,0.103,0.221,1,0
"Def6",0.000238045647218833,0.0533397107131544,0.065,0.021,1,0
"Fam21",0.000238638282355289,0.14897652768039,0.271,0.163,1,0
"Zfp511",0.000239909522088805,0.0882741900660206,0.123,0.055,1,0

"Hpn",0.000242238901331605,-0.357206511439984,0.284,0.41,1,0
"Asb13",0.000242877051224395,-0.239235667871651,0.052,0.161,1,0
"Rnf40",0.000243236099836142,0.0877794142330959,0.116,0.05,1,0
"Fam25c",0.000243281922679381,-0.21840445874724,0,0.08,1,0
"Snrpc",0.000243522661689095,0.160461023685862,0.271,0.163,1,0
"Fem1a",0.000244209981242135,0.0982683194040154,0.155,0.076,1,0
"Nom1",0.000246050784962387,0.113426949370814,0.155,0.076,1,0
"Fbxw9",0.000246593893504851,0.10369740174108,0.123,0.055,1,0
"Exosc8",0.000246983391612383,0.141534201883275,0.129,0.06,1,0
"Hmgn2",0.000247455387157364,0.174877822823101,0.355,0.231,1,0
"Fcgr3",0.000247576714471339,0.145561382800505,0.123,0.056,1,0
"Lrp6",0.000247897610627186,0.164338910229566,0.361,0.234,1,0
"Slc24a5",0.000250862604130882,0.157742605523079,0.11,0.047,1,0
"Bcl2l11",0.000251183717929712,0.102305208562575,0.097,0.039,1,0
"Ddx3y",0.00025201019833584,0.120085242306015,0.265,0.157,1,0
"Med30",0.000252409869066471,0.12530619811308,0.206,0.114,1,0
"Tdp1",0.00025267157045614,0.0686188464731611,0.058,0.018,1,0
"Slc25a53",0.000253071801807l7,0.0648533923522416,0.052,0.015,1,0
"Armc10",0.000253250590493053,0.110228928785371,0.135,0.064,1,0
"Dnajc11",0.000253521074086874,0.107319896503855,0.206,0.113,1,0
"Dctn4",0.000253698207031975,0.117617202970977,0.181,0.095,1,0
"Slc4a1ap",0.000253949291759003,0.118539134544019,0.135,0.064,1,0
"Arap2",0.000254255624899453,0.192834436438655,0.194,0.106,1,0
"Med14",0.000256174708674351,0.125610357412889,0.142,0.068,1,0
"Enpp6",0.000256234103036069,-0.238767870166585,0.026,0.122,1,0
"Zc3h8",0.000258251071793047,0.0658430441359757,0.071,0.025,1,0
"Pank1",0.000259284537034763,-0.368834354165398,0.155,0.269,1,0
"Skap1",0.000259548979112126,0.0588971932760402,0.052,0.015,1,0
"Zfp626",0.000260489222904944,0.0938776078991891,0.071,0.025,1,0
"Pxmp2",0.000260717878911546,-0.274624415796312,0.039,0.137,1,0
"Slain1",0.000261205022654187,0.0704005846905666,0.058,0.018,1,0
"Zxdc",0.000261572828534833,0.100749488805707,0.116,0.051,1,0
"Bbip1",0.00026210703764476,0.0946183507650369,0.355,0.224,1,0
"Zfp428",0.000262419955009242,0.10335904746358,0.103,0.043,1,0
"Creld1",0.000262788016013045,0.140686862125059,0.148,0.073,1,0
"Exoc1",0.000262955340093672,0.119337142891535,0.168,0.086,1,0
"Kif13a",0.000263607300397058,0.0777298222696769,0.09,0.035,1,0
"Rad23b",0.00026565213073074,0.163611585158331,0.355,0.231,1,0
"Txndc16",0.000267103746218698,0.0714685090146254,0.09,0.035,1,0
"Mospd3",0.000267414517171449,0.102098364625312,0.174,0.09,1,0
"Adgrl2",0.000267656394176867,0.14488554453755,0.168,0.087,1,0
"Fam20b",0.000267744227345744,0.146412299415784,0.239,0.139,1,0
"Snrpd2",0.000268111234085105,0.154502830010946,0.658,0.489,1,0
"Cyb5rl",0.00027043699697078,0.140487969565906,0.123,0.055,1,0
"Nucb1",0.00027046537054099,0.201519481277983,0.31,0.201,1,0
"Fbxo38",0.000270599919535407,0.106006188165315,0.116,0.051,1,0
"Rab10",0.000271225673816994,0.139052388557393,0.445,0.306,1,0
"Rnf111",0.000271442363240336,0.103489662838715,0.148,0.073,1,0
"Mapkapk2",0.000271964479403618,0.143862906730774,0.297,0.185,1,0
"Pllp",0.00027270827657281,-0.206695898235024,0.013,0.101,1,0

"Vil1",0.000273018902714486,-0.259445254655647,0.039,0.139,1,0
"G0s2",0.000274845769304497,-0.161189947130641,0.065,0.172,1,0
"Zscan26",0.000275085434951033,0.123231918618282,0.174,0.09,1,0
"Cln3",0.00027526962224042,0.124919782687868,0.168,0.086,1,0
"Gm15706",0.000275464303259015,0.0667619383022277,0.052,0.015,1,0
"Apbb2",0.000276958004169061,0.146746209911597,0.213,0.118,1,0
"Esrrb",0.000277538339149438,0.0631089334455314,0.065,0.021,1,0
"Csf1",0.000278227405252587,0.104084975550871,0.065,0.021,1,0
"Arid1a",0.000278828310850811,0.161503080014325,0.271,0.165,1,0
"Krba1",0.000280942473840166,0.109518000011544,0.097,0.04,1,0
"Gle1",0.000281094863814451,0.126836502993322,0.181,0.096,1,0
"Obfc1",0.000281351168264405,0.0754192927429677,0.065,0.021,1,0
"Acss2",0.000281805383227431,-0.289321390351514,0.065,0.172,1,0
"Atp8b1",0.000282600103527214,0.0806867942472273,0.103,0.043,1,0
"Tnfaip1",0.000283822303806668,0.141190991111861,0.194,0.106,1,0
"Dpp7",0.00028402828174595,-0.234166328444205,0.032,0.13,1,0
"Nsmce1",0.000284042716578988,0.149358671086319,0.239,0.138,1,0
"Spns3",0.000284186889527242,-0.262478400347807,0.026,0.12,1,0
"Azi2",0.000284377176311389,0.115481961898723,0.277,0.168,1,0
"Slco1a1",0.000286456143177157,-0.333341555751712,0,0.079,1,0
"Irak1bp1",0.000287009699428925,0.0976116176448447,0.181,0.094,1,0
"Ddb2",0.000287791074721783,0.061945931328951,0.052,0.015,1,0
"Med15",0.000287904501549896,0.12905504327691,0.116,0.052,1,0
"Sf3b6",0.000289015699476531,0.137750940308311,0.51,0.358,1,0
"Ndufv2",0.000290339507287014,-0.338307140626543,0.606,0.639,1,0
"Bcas2",0.000290598695738097,0.124137635188769,0.413,0.269,1,0
"Ddx18",0.00029208795974935,0.115113033188186,0.206,0.114,1,0
"Pphln1",0.000292447596775279,0.115806246868381,0.135,0.064,1,0
"Klhl18",0.0002925730001078,0.0819259952081669,0.09,0.036,1,0
"Ccl8",0.00029389766488464,0.124319128827137,0.097,0.04,1,0
"Yme1l1",0.000294609953443149,0.162827823252856,0.316,0.202,1,0
"Gbp7",0.00029707480222005,0.116951689633801,0.097,0.04,1,0
"Tbl1x",0.000297158984291913,0.183384007511479,0.29,0.182,1,0
"Ppie",0.000297302876702495,0.107254848570555,0.142,0.068,1,0
"Gclm",0.000298606468216643,-0.292024133826458,0.329,0.445,1,0
"Hspb11",0.000301298180377944,0.112392837208835,0.213,0.119,1,0
"Poglut1",0.000302957198058158,0.10616780646023,0.123,0.056,1,0
"Usp32",0.000302982527379816,0.11643753682902,0.187,0.1,1,0
"Arpin",0.000303817505084074,0.106030387810428,0.123,0.056,1,0
"Arrb1",0.000304973698025375,0.114235589742139,0.103,0.044,1,0
"Mogat1",0.000306604201083507,-0.22554663524934,0,0.078,1,0
"Mtch1",0.000306995327197897,0.111273442885385,0.497,0.332,1,0
"Atp5s",0.000308311093620597,0.0929818290299037,0.103,0.043,1,0
"Smg1",0.000308322551960568,0.217149406176758,0.2,0.113,1,0
"2510039O18Rik",0.000311294356811664,0.131671492595633,0.219,0.125,1,0
"B3glct",0.000312897602423451,0.103977469356539,0.09,0.036,1,0
"Bzw1",0.000312938943779169,0.131351817922773,0.445,0.305,1,0
"Naa16",0.000314954098975295,0.0795559284126033,0.103,0.043,1,0
"Ahdc1",0.000315924672029007,0.140634792992272,0.155,0.079,1,0
"Fndc4",0.000316783678365803,0.0677546022720109,0.052,0.015,1,0

"Tle3",0.000317246515231533,0.141446442909102,0.194,0.106,1,0
"Papd4",0.0003178226633417,0.0752164265702244,0.084,0.032,1,0
"A430033K04Rik",0.000318528653913677,0.0711777189444681,0.052,0.015,1,
0
"Apex1",0.000318531850939864,0.15749318479231,0.187,0.101,1,0
"Marc2",0.000318999269935988,-0.328205958981,0.484,0.551,1,0
"Idh3g",0.000319131109114986,-0.328512396129893,0.348,0.468,1,0
"Tmem37",0.000320012358595331,-0.348099489563743,0.155,0.27,1,0
"Ehmt1",0.000321027993402427,0.150996047779727,0.174,0.092,1,0
"Tmem38a",0.000321157414435936,0.0982022697457338,0.097,0.04,1,0
"Rps12-ps3",0.000321298447487282,0.195394013991653,0.548,0.404,1,0
"Fryl",0.00032131470643405,0.117767274838463,0.181,0.095,1,0
"Mxd1",0.000321875765545589,0.110041698825751,0.077,0.029,1,0
"Slc50a1",0.000323364635521619,0.163355461765292,0.174,0.094,1,0
"Mier3",0.000323410355420744,0.0820357078018307,0.11,0.048,1,0
"Zmynd10",0.000324962980980973,-0.292835222494233,0.045,0.144,1,0
"Ctdsp2",0.00032524282010851,0.141314268136075,0.271,0.166,1,0
"Prkcd",0.000326497845029773,0.13949392268815,0.258,0.153,1,0
"Zfp236",0.000326870923143603,0.126012667692581,0.09,0.036,1,0
"Agpat5",0.00032736548064603,0.106357454795476,0.187,0.1,1,0
"Nsdhl",0.000327497466283432,-0.237060910435776,0.039,0.138,1,0
"Wdr20",0.000327837400841935,0.146604876074577,0.142,0.069,1,0
"Tbc1d23",0.000328121048480099,0.132594031229096,0.11,0.048,1,0
"Fem1c",0.000328212109599769,0.129276072837105,0.161,0.083,1,0
"Gm16586",0.000328570904670561,0.0950512510700609,0.155,0.077,1,0
"Ubp1",0.000328682663157333,0.154962977663563,0.2,0.112,1,0
"Rassf9",0.000329123590728959,0.10598036701472,0.071,0.025,1,0
"Fundc1",0.00032913396439191,0.0826766222465954,0.252,0.145,1,0
"Ints8",0.000330654870889846,0.114324163251936,0.097,0.04,1,0
"Gmfg",0.000332140258481771,0.138753724274923,0.168,0.087,1,0
"Armcx3",0.000336220202007236,0.126663629044478,0.148,0.073,1,0
"Krcc1",0.000338921042870638,0.114945514569192,0.471,0.321,1,0
"Klc2",0.000341431234660019,0.108007109815084,0.135,0.065,1,0
"Ar",0.000341556969409095,-0.221711290856406,0.026,0.118,1,0
"Ywhaz",0.000346281207873429,0.184233055646807,0.555,0.412,1,0
"Zdhhc13",0.000347723924676361,0.0981164592077144,0.097,0.04,1,0
"Kmo",0.000348297346378472,-0.211331870885558,0.013,0.098,1,0
"Ube2g1",0.000350393868599818,0.145879087698804,0.252,0.15,1,0
"Errfi1",0.000351038180056546,-0.319916641649955,0.271,0.393,1,0
"Rars",0.000351183873711971,0.104527915827747,0.174,0.091,1,0
"Bhmt2",0.000351192879758684,-0.213912492374617,0,0.076,1,0
"Slc16a14",0.000351192881212898,-0.185939734167717,0,0.076,1,0
"Micu2",0.00035265487770163,0.104578726685112,0.181,0.095,1,0
"Igf1r",0.000359544843291515,0.14350850646172,0.497,0.347,1,0
"Seh1l",0.000360873545924606,0.116108646661283,0.187,0.101,1,0
"Dok4",0.000363680991981851,0.0970352818638112,0.097,0.04,1,0
"Celsr2",0.000363946985110311,0.223953341915145,0.284,0.179,1,0
"Slc38a10",0.000365197083415705,0.118202443174274,0.258,0.154,1,0
"Cisd2",0.000366665488791923,0.154625111589326,0.523,0.367,1,0
"Nfil3",0.000367494603781608,0.0523492839893037,0.084,0.032,1,0

"Vnn1",0.000368036940842621,-0.188789238361292,0.006,0.088,1,0
"Polr1d",0.000370796692418197,0.148361682555355,0.626,0.457,1,0
"Aldh1l1",0.000371100041729111,-0.352607731359329,0.213,0.33,1,0
"Cul1",0.000372489949094578,0.106811013324678,0.277,0.168,1,0
"Cbfa2t2",0.000374085147765896,0.0725744798071266,0.11,0.048,1,0
"Zfp763",0.000374574922489748,0.0663502894064563,0.052,0.015,1,0
"Bre",0.000375525344489289,0.128163225113004,0.245,0.145,1,0
"Tcirg1",0.000376053811110007,0.0947952048388019,0.103,0.044,1,0
"Kank2",0.000378240156844287,0.0801046057333362,0.077,0.029,1,0
"Fam32a",0.000379736789923938,0.107065829126135,0.406,0.266,1,0
"Tnfaip8l2",0.00038004048019357,0.0921051156122649,0.077,0.029,1,0
"Ebna1bp2",0.000380121046885481,0.153066063930735,0.271,0.167,1,0
"Bckdha",0.000381238552573169,-0.391732209719205,0.284,0.384,1,0
"Tcf20",0.000382881140122628,0.09720216185346,0.123,0.057,1,0
"Sh3rf1",0.000383574963966898,0.13493003666801,0.174,0.092,1,0
"Cyfip1",0.000384016778537398,0.126639841273765,0.213,0.121,1,0
"Itm2b",0.000384383141485427,0.206239209996821,0.987,0.918,1,0
"Cldn1",0.000385665063094052,0.109422350654409,0.084,0.033,1,0
"Ppp2ca",0.000385981037564015,0.12252991250017,0.523,0.358,1,0
"Gm11128",0.000386192964211504,-0.264175993995706,0.013,0.097,1,0
"Isg15",0.000387346065260751,0.134541095065503,0.097,0.04,1,0
"Hdgf",0.000387486172878435,0.131255204055011,0.587,0.412,1,0
"Sft2d3",0.000387816609730539,0.0852326624870515,0.116,0.052,1,0
"Veph1",0.00038826257629541,0.15719472503613,0.168,0.088,1,0
"Bub3",0.000389604360308187,0.106736665555652,0.277,0.169,1,0
"Alyref",0.00039120222224361,0.127381836636229,0.265,0.161,1,0
"Harbi1",0.00039181362070899,0.0729094694522394,0.09,0.036,1,0
"Vamp2",0.000392853873227976,0.156986437195191,0.206,0.117,1,0
"Arhgdib",0.000394287792947321,0.167227090054477,0.213,0.122,1,0
"Gm13075",0.000394750671980388,0.069410112875032,0.058,0.019,1,0
"Exosc10",0.000394769149174749,0.0970345049459747,0.135,0.065,1,0
"Mrfap1",0.000395305796079352,0.175569696822774,0.677,0.516,1,0
"Zfp263",0.000395594238636822,0.100875643030797,0.129,0.06,1,0
"Zfp422",0.000398465551611809,0.107198611653062,0.181,0.096,1,0
"Ccdc173",0.000401025975226663,0.0866535761994869,0.071,0.025,1,0
"Il6st",0.000401340567123821,0.113554370513889,0.342,0.22,1,0
"Zfp780b",0.000401536996883279,0.128091061339318,0.135,0.066,1,0
"Ube2f",0.000402608648851419,0.148583626672896,0.239,0.142,1,0
"Ech1",0.000407292680802967,-0.371120708686203,0.523,0.591,1,0
"Rfx5",0.000407720370423173,0.0609036978992868,0.077,0.029,1,0
"Alg9",0.000408163736595359,0.0854863362207251,0.103,0.044,1,0
"2810403A07Rik",0.000409958162325186,0.138265926127182,0.219,0.126,1,0
"Gmnn",0.000410767887649028,0.0812961645290337,0.161,0.082,1,0
"Cdk19",0.000411206987722149,0.084362436239933,0.11,0.048,1,0
"Igfbp3",0.000411782738060165,0.0751106907468294,0.071,0.025,1,0
"Tmem219",0.000412006759326378,0.118877763172976,0.271,0.165,1,0
"Uap1l1",0.000412627699881678,-0.338832547470691,0.142,0.254,1,0
"Acvr2a",0.000413043874478793,0.0911639305702553,0.097,0.04,1,0
"Gm10263",0.000413782330471879,0.120220719561087,0.129,0.06,1,0
"Fyn",0.000415679674831652,0.0827022588396619,0.084,0.033,1,0

"Slc27a1",0.000417629654872256,0.113325378504051,0.161,0.084,1,0
"Npc1",0.000419127811037119,0.132054116420921,0.142,0.071,1,0
"3010003L21Rik",0.000419816972245489,0.0513691772026358,0.065,0.022,1,
0
"Txndc9",0.000421416933836548,0.119336813756787,0.387,0.255,1,0
"Usp4",0.000422122796363575,0.170021374968141,0.239,0.143,1,0
"Ngdn",0.000422537505059305,0.111382890617487,0.213,0.121,1,0
"Gm15446",0.000422732153056567,0.0717661686653428,0.084,0.033,1,0
"Dpy19l1",0.000422840374792658,0.0741228138852725,0.071,0.026,1,0
"Mettl2",0.000422854184561865,0.082749157693451,0.077,0.029,1,0
"Reep3",0.00042368112622687,0.161228416514071,0.406,0.271,1,0
"Ganc",0.000425604213826653,0.118967751186576,0.161,0.083,1,0
"Lat2",0.00042579500302745,0.0702362024056736,0.077,0.029,1,0
"Kmt2b",0.000426012258019382,0.121038110337938,0.123,0.058,1,0
"Becn1",0.000428610257120699,0.129685635613205,0.31,0.192,1,0
"Kif1bp",0.000429619922416593,0.112971599748945,0.174,0.092,1,0
"Rufy2",0.00042971861130173,0.0913438375496779,0.09,0.037,1,0
"Cdkl3",0.00042977885890776,0.0789095756147956,0.065,0.022,1,0
"Zfp110",0.00042999047869674,0.0962257452641077,0.11,0.048,1,0
"Wipi1",0.000430732874804983,0.116587957514724,0.168,0.088,1,0
"Eed",0.000431110778582222,0.149500351200262,0.174,0.094,1,0
"Meaf6",0.000431415328002598,0.107806906347335,0.155,0.079,1,0
"Stxbp6",0.00043377375261196,0.151113928662961,0.155,0.079,1,0
"Ubxn4",0.000434086892558548,0.165138436945983,0.419,0.286,1,0
"Btbd1",0.000435594610366703,0.156434650572344,0.303,0.192,1,0
"Slc16a9",0.000436212704707907,-0.167369712667903,0,0.074,1,0
"Srsf6",0.000436480888385893,0.14984361123546,0.439,0.307,1,0
"Usp12",0.000437629931037081,0.0901315866936775,0.187,0.1,1,0
"Vim",0.000439410579658573,0.129115100926434,0.148,0.075,1,0
"Mecp2",0.00043975875382176,0.104667991640151,0.174,0.092,1,0
"Stk17b",0.000440579025631114,0.128574618666129,0.103,0.045,1,0
"Kif3b",0.000442636603391887,0.130580713349034,0.129,0.062,1,0
"Sec61b",0.00044320859083566,0.15099405717672,0.716,0.543,1,0
"Tmem237",0.00044333594076439,0.108318549797201,0.161,0.083,1,0
"Daam1",0.000444736715086548,0.127021519261698,0.155,0.08,1,0
"Wdr82",0.000446270646516597,0.0991321605238434,0.168,0.088,1,0
"Trpc4ap",0.000446968360525637,0.154630717040373,0.219,0.129,1,0
"Stat5b",0.000447112634605891,0.0957043400982514,0.097,0.04,1,0
"Unc45a",0.000448435044818104,0.10921247494404,0.161,0.083,1,0
"Erdr1",0.000449002831627444,0.0822411386846009,0.194,0.105,1,0
"Rest",0.000451312396754811,0.174276210292324,0.226,0.135,1,0
"Phtf1",0.000454723723249279,0.103022194215512,0.116,0.053,1,0
"Fam110b",0.000454882448126381,0.0531820526495819,0.065,0.022,1,0
"Celf1",0.000455133927014815,0.184717548783226,0.361,0.246,1,0
"Rnf220",0.000455654295756343,0.107008149964879,0.174,0.093,1,0
"Ddx47",0.000455708547503355,0.0987065773023009,0.168,0.088,1,0
"Slc39a5",0.000457121468169856,-0.204535815867668,0.006,0.085,1,0
"Rnf135",0.000457644432047115,0.0789941305957223,0.071,0.026,1,0
"Erbb2",0.000461104134433927,0.174752577700044,0.187,0.104,1,0
"Rhod",0.000462355930777874,0.09546094049932,0.142,0.069,1,0

```
"Gpi1",0.000462710916033276,0.144076059178337,0.626,0.462,1,0
"Ube3c",0.000462914872977117,0.109079136400368,0.194,0.106,1,0
"Tspan33",0.000464038105482288,0.235643016575644,0.284,0.18,1,0
"Ctso",0.00046432385788818,0.136561035657464,0.174,0.094,1,0
"Ccdc157",0.000465843463810231,0.106676902938486,0.071,0.026,1,0
"Arhgef12",0.000466312324097784,0.162661597964954,0.394,0.262,1,0
"Sipa1",0.00046883988383283,0.0892583376247366,0.071,0.026,1,0
"Aqp1",0.000471955126258796,1.2218763946877,0.123,0.26,1,0
"Map4k5",0.000472333588707025,0.154843887360766,0.129,0.063,1,0
"Ypel3",0.000472987469365251,0.188710225388543,0.439,0.312,1,0
"Nhsl1",0.000473448913037286,0.0946126405281269,0.116,0.053,1,0
"Nvl",0.000475041707351233,0.163820550313382,0.174,0.095,1,0
"Golim4",0.000475407043814114,0.0821552131174401,0.103,0.044,1,0
"BC025446",0.000475625367682991,-0.208326831886498,0.013,0.095,1,0
"Psmc3ip",0.000477060040061493,0.0587344335207675,0.071,0.026,1,0
"Lix1",0.000477676491949695,0.100592007199886,0.084,0.033,1,0
"Ginm1",0.000477681695637353,0.183054078952707,0.342,0.229,1,0
"Prdm2",0.000477870803569839,0.174397014543513,0.148,0.077,1,0
"Pi4k2b",0.000478501561731458,0.0749034361732168,0.123,0.056,1,0
"Bmpr1a",0.000479090279767879,0.121663604039343,0.206,0.117,1,0
"Man1b1",0.000479166197877718,0.113129277313508,0.155,0.08,1,0
"Tmem158",0.000479893670397047,0.156027219676617,0.129,0.061,1,0
"Uba3",0.000480698748763028,0.121305988672944,0.206,0.116,1,0
"Inafm1",0.000481075332007551,0.0747558763047489,0.097,0.04,1,0
"Tnpo3",0.000481496828597282,0.152319999313585,0.271,0.166,1,0
"Acot4",0.000485794564517325,-0.18858379775183,0.006,0.084,1,0
"Kitl",0.000488302879501829,0.094770202812869,0.09,0.037,1,0
"Pard6a",0.000489430493585764,0.0577702495308038,0.097,0.04,1,0
"Tnip2",0.000490135657994505,0.0794110766879958,0.077,0.029,1,0
"Tspyl1",0.000490612172053974,0.099007563272504,0.226,0.13,1,0
"Sdpr",0.000490988145941188,0.0921466565374214,0.123,0.057,1,0
"Zbtb20",0.000491116968675807,0.0840937093402732,0.768,0.572,1,0
"Ppil2",0.00049139422093307,0.170561268532746,0.213,0.123,1,0
"A330017A19Rik",0.000491634752341166,0.117292942837739,0.077,0.029,1,0
"Fyco1",0.000492031457347491,0.172192370737694,0.181,0.1,1,0
"Ccl3",0.000493446252201973,0.195256422202642,0.11,0.049,1,0
"Rpp38",0.000494182211614827,0.12035892539613,0.084,0.033,1,0
"Stim2",0.000494940049721223,0.134945948616488,0.129,0.063,1,0
"Psma3",0.000496126885474818,0.195986752709843,0.632,0.471,1,0
"Pycr2",0.000497238342004006,0.103050437769711,0.155,0.079,1,0
"Neil1",0.000500100200114059,0.0835198822574208,0.058,0.019,1,0
"Prkx",0.000505475143858882,0.111097383206343,0.116,0.054,1,0
"Stx7",0.000506880483880554,0.0989414164859705,0.548,0.385,1,0
"Shprh",0.000510192938525872,0.119401849099555,0.135,0.067,1,0
"Msh3",0.00051426844238578,0.161107724322986,0.168,0.09,1,0
"Ctf1",0.000516833305178036,0.0931528797817364,0.11,0.05,1,0
"Lsm8",0.000517392279300396,0.156501073115053,0.258,0.159,1,0
"Asun",0.000519529836683371,0.0987861111794251,0.135,0.066,1,0
"Hoxa7",0.000519677608502286,0.161930607713066,0.29,0.179,1,0
"Map3k2",0.000519784084444242,0.247610394059873,0.226,0.138,1,0
```

"Trio",0.000520522583605928,0.0965975015708524,0.168,0.088,1,0
"Trmt1",0.000521211407324675,0.111466806899328,0.181,0.098,1,0
"Adgrl1",0.000521781291911221,0.0890504187311606,0.103,0.044,1,0
"Ugt2b34",0.000523197151670258,-0.220078871603683,0.013,0.094,1,0
"Fam120b",0.000523584395997153,0.104682088039908,0.11,0.049,1,0
"Prr14",0.000525594066317197,0.100151678620443,0.135,0.066,1,0
"Cdon",0.000525877318110298,0.0527564012646115,0.058,0.019,1,0
"Neo1",0.000526139417511676,0.102869037051483,0.135,0.066,1,0
"Fam20c",0.000526562914774846,0.184213248039455,0.226,0.132,1,0
"Hsd3b3",0.000527097853533184,-0.173791601335941,0,0.072,1,0
"Golga5",0.000528954221774236,0.101516034357732,0.148,0.074,1,0
"Gtf2i",0.000534509278420338,0.159724036246651,0.368,0.251,1,0
"0610030E20Rik",0.000538483007616057,0.150083559178364,0.174,0.095,1,0
"Eif4e2",0.000538807881768209,0.0837818191473619,0.329,0.21,1,0
"Tigd2",0.000539411027148992,0.118220424474192,0.181,0.099,1,0
"Psmb9",0.000541990191473821,0.133621348754853,0.135,0.066,1,0
"Eprs",0.000542505866937693,0.158434086548378,0.316,0.205,1,0
"Celsr1",0.000543042926848638,0.13062095143056,0.116,0.054,1,0
"Aebp2",0.000543829367882364,0.165540830188502,0.2,0.115,1,0
"Sergef",0.000544643577816297,0.064239282824578,0.11,0.049,1,0
"Ctsa",0.00054512711244246,-0.304446660219844,0.258,0.379,1,0
"Hdac3",0.000547417708170187,0.079010840906251,0.187,0.101,1,0
"Fam122a",0.000547605640436369,0.0962015275850869,0.135,0.067,1,0
"Naf1",0.000548061039932589,0.0790171984938225,0.123,0.057,1,0
"Tspan18",0.000548237697597938,0.057786129333925,0.077,0.029,1,0
"Dnal4",0.000548680367233781,0.136190196140335,0.155,0.08,1,0
"Samd9l",0.000550665008260939,0.21669012958668,0.471,0.346,1,0
"B4galt3",0.000551410529960561,0.13925444174929,0.148,0.076,1,0
"Eif3b",0.000554038942678876,0.12530050608019,0.297,0.19,1,0
"Lsm7",0.00055566417984279,0.129400496121735,0.29,0.183,1,0
"Cct8",0.000556709323288982,0.105776791898755,0.465,0.319,1,0
"Nkg7",0.000558381497793481,0.0851459249984562,0.097,0.041,1,0
"Ap2s1",0.000558818909684159,0.129191424280698,0.529,0.366,1,0
"Chtop",0.000560477279267425,0.149175117364521,0.252,0.155,1,0
"Irf2bp1",0.00056057655680793,0.107054475345774,0.129,0.063,1,0
"Zzz3",0.000561325821040081,0.103363762530185,0.213,0.121,1,0
"Mtrf1l",0.000561837853053658,0.0999824416291585,0.084,0.033,1,0
"Hcfc2",0.000563626985208634,0.0696577509622932,0.103,0.045,1,0
"Snx13",0.000565678277679881,0.116022362270052,0.187,0.103,1,0
"Nme7",0.000566172390533109,0.121115761406301,0.161,0.085,1,0
"Rasa2",0.000566589153553353,0.157072641027134,0.213,0.123,1,0
"Cdk4",0.000567697015528459,0.132063803190371,0.335,0.216,1,0
"Mgat3",0.000569104856846842,-0.181899024258393,0.013,0.094,1,0
"Zfp618",0.000571783871993258,0.125357463465656,0.161,0.085,1,0
"Agap3",0.000573516379038149,0.0814442043835611,0.103,0.045,1,0
"Pard3b",0.000575327520372799,0.099768152313077,0.116,0.054,1,0
"Hdac6",0.000575399123335595,0.105568398909565,0.174,0.093,1,0
"Uchl1",0.000575630928417641,0.0789690009242572,0.065,0.023,1,0
"Dda1",0.000578767886881813,0.101579764042739,0.206,0.117,1,0
"Spcs3",0.000578820443469987,0.119801243362944,0.232,0.136,1,0

"Suz12",0.000579770415816148,0.114352834218327,0.206,0.119,1,0
"Pstk",0.000582786730464381,0.130694228927852,0.161,0.086,1,0
"Ppp1r8",0.000585527412205383,0.0945445727934481,0.11,0.05,1,0
"Gstt2",0.000587138950553121,-0.393696591002351,0.239,0.343,1,0
"Gpr160",0.000588579585227175,0.132357396787333,0.155,0.081,1,0
"Copb1",0.000589187593158591,0.165356775891592,0.303,0.197,1,0
"Bgn",0.000590598114401755,0.0822359314397183,0.084,0.033,1,0
"Tfeb",0.000590843298568403,0.104836290600072,0.142,0.071,1,0
"Caskin2",0.000591458365406157,0.0849658423070646,0.116,0.054,1,0
"Kdm6a",0.000595405604527117,0.108009589929922,0.129,0.062,1,0
"Ufl1",0.000596767994364587,0.136134777015847,0.187,0.105,1,0
"Ndufs8",0.00059740755037511,-0.348049521498148,0.587,0.611,1,0
"Mrpl50",0.000597475008416947,-0.344638457716775,0.181,0.29,1,0
"Clec12a",0.000597570063675417,0.0791995218110098,0.09,0.037,1,0
"Tchp",0.000597857954273768,0.0767520314208752,0.077,0.03,1,0
"Nr1h4",0.000598324228546969,-0.280864437696397,0.058,0.155,1,0
"Arhgap32",0.000599432987474329,0.106536018496703,0.194,0.107,1,0
"Ints3",0.000602428294845723,0.1032842812207 4,0.11,0.049,1,0
"Limk2",0.000602932819582249,0.0868783069412885,0.116,0.054,1,0
"Crtc2",0.000604939259543004,0.0729362745339077,0.084,0.033,1,0
"Ddx10",0.000606350761478548,0.126135016841905,0.116,0.055,1,0
"Sertad1",0.000607738260708188,0.0926167546144658,0.103,0.046,1,0
"Smn1",0.000608381794714535,0.0842737638145933,0.129,0.062,1,0
"Rps13",0.000608709496166412,0.179904684729443,0.89,0.813,1,0
"BC003965",0.000609450563826605,0.138924411866417,0.271,0.17,1,0
"Cadm1",0.000612681092696504,0.141253316893668,0.168,0.089,1,0
"Mlh3",0.000615281641575962,0.118267540426762,0.116,0.054,1,0
"Fuca2",0.000617065348319608,-0.334229833045517,0.355,0.453,1,0
"Snupn",0.000617845982915703,0.120948124377248,0.084,0.034,1,0
"Mettl16",0.00061855506269416,0.101748472344031,0.155,0.081,1,0
"Supt4a",0.000619158471967564,0.106375532805669,0.426,0.286,1,0
"Amer1",0.000621284792012217,0.0824412869886353,0.084,0.033,1,0
"Slc25a17",0.000623795489342326,0.07760427628939,0.329,0.209,1,0
"Ccar1",0.000624677636394689,0.117069218442415,0.342,0.222,1,0
"Zbtb21",0.000627140803697086,0.100392765277268,0.084,0.034,1,0
"Gpalpp1",0.000629000596256648,0.0838923865837132,0.116,0.054,1,0
"Serpine2",0.000630388101619934,0.0719878878274865,0.071,0.026,1,0
"Anks4b",0.000634870006554111,-0.159067365099668,0.006,0.082,1,0
"Mylip",0.000635925355612461,0.14858846392043,0.161,0.087,1,0
"Rcor3",0.000637122862133878,0.11662091208863,0.161,0.085,1,0
"Zbtb38",0.000637352254983227,0.110464862390995,0.245,0.147,1,0
"Ncapd2",0.000637814668697983,0.0717918309625931,0.058,0.019,1,0
"Ccs",0.000639545907310693,-0.316181082931375,0.239,0.353,1,0
"Dctn2",0.000640280635983377,0.121630302272486,0.368,0.242,1,0
"Coro1a",0.000642201690224445,0.182736557714672,0.213,0.125,1,0
"Wfdc17",0.00064334325831213,0.324105953873866,0.245,0.153,1,0
"Dtnb",0.000644348210942974,0.134585498852484,0.142,0.072,1,0
"Slco3a1",0.000645353455743242,0.0852931592960316,0.323,0.201,1,0
"Sap30",0.000649454475579247,-0.346420627265929,0.123,0.227,1,0
"Zfp362",0.000653549514125213,0.106335956630467,0.09,0.038,1,0

"Ddx3x",0.000653723316466684,0.126108766369352,0.471,0.325,1,0
"Snrnp200",0.000654552347993058,0.117716687628972,0.2,0.114,1,0
"Rexo2",0.000658812612091826,0.0914166495648318,0.497,0.341,1,0
"Mark4",0.000658925467943536,0.0765532002000153,0.097,0.041,1,0
"Bcl2l1",0.000660426156876603,0.160201090828032,0.303,0.198,1,0
"Bmf",0.000660768322082077,0.0774611118289529,0.077,0.03,1,0
"Sec61a1",0.000661102603648312,0.14133703841327,0.303,0.196,1,0
"Rhobtb1",0.000662419168407947,-0.206703786132853,0.039,0.131,1,0
"Pdgfd",0.00066269904557216,0.0968466640170863,0.077,0.03,1,0
"Il4ra",0.000664575338739966,0.0872326368809534,0.09,0.038,1,0
"Foxo1",0.000664808272552527,0.114261727752678,0.174,0.094,1,0
"Ebpl",0.000667791205496497,0.138845566623135,0.187,0.104,1,0
"Rsph3a",0.00067019798218911,0.0658695854007208,0.065,0.023,1,0
"Cenpa",0.00067164045863603\6,0.102089804377462,0.058,0.019,1,0
"Proser1",0.000672797655846003,0.0877726398411921,0.129,0.062,1,0
"Txnrd1",0.000673057875278856,0.0981205760258955,0.335,0.214,1,0
"Alkbh2",0.000674925224853656,0.073294444796056,0.09,0.038,1,0
"Scg5",0.000676490816208082,0.130013118321723,0.123,0.059,1,0
"Acap2",0.000678391098279631,0.153390482230707,0.129,0.064,1,0
"Gfod2",0.000679126403405943,0.114711592548888,0.084,0.034,1,0
"Uap1",0.000680849336089855,0.208465738320775,0.31,0.206,1,0
"Col12a1",0.000680909210727031,0.054185293961482,0.052,0.016,1,0
"Zfp26",0.00068152093784635,0.0887377765258262,0.077,0.03,1,0
"Stau2",0.000683800856860082,0.0893742775918165,0.148,0.076,1,0
"Rxrb",0.000687909646601675,0.0633842486981236,0.142,0.071,1,0
"Slc10a2",0.000690112097844664,-0.186194001474084,0.006,0.081,1,0
"Psmd11",0.000690219269938324,0.132765342063597,0.394,0.26,1,0
"Fam234b",0.000691593083160288,0.0825378315305999,0.097,0.042,1,0
"C130074G19Rik",0.000691629189590398,-0.282691516420876,0.181,0.299,1,
0
"Cstb",0.000692648239447022,0.207942267869764,0.716,0.56,1,0
"Mcur1",0.000693611512609925,-0.304241826121507,0.148,0.261,1,0
"Tmtc4",0.000698952099652747,0.0968051403367355,0.103,0.046,1,0
"Gm14305",0.000700332006517368,0.0974154372478045,0.097,0.042,1,0
"Rpgr",0.000700630399848199,0.063162915250043\6,0.071,0.026,1,0
"Mipol1",0.000701148774897994,0.078044285225204,0.071,0.027,1,0
"Vcpip1",0.000702800660613807,0.108917722278585,0.181,0.1,1,0
"Hba-a1",0.00070527435957173,-1.59117527889487,0.155,0.081,1,0
"Ganab",0.000706814758116938,0.157730002580833,0.323,0.214,1,0
"Mat2b",0.000707068646527372,0.101843150986609,0.387,0.256,1,0
"Med13l",0.000712703679901663,0.142376834806191,0.181,0.101,1,0
"Plekhj1",0.000714695581354385,0.123427319084512,0.316,0.205,1,0
"Hsph1",0.00071640321525665,0.166164716969796,0.252,0.157,1,0
"Zbtb5",0.00071914157153899,0.062910104812202,0.077,0.03,1,0
"Ptpn12",0.000719430495619046,0.096720318003776,0.123,0.059,1,0
"Lpp",0.000721441950575344,0.142057772616796,0.381,0.263,1,0
"Use1",0.00072176426964065,0.139726150594595,0.445,0.308,1,0
"Lhx1os",0.000725685996498798,-0.214308726103777,0.026,0.112,1,0
"Trim27",0.000725854837074986,0.131602334943216,0.155,0.082,1,0
"Rtn3",0.000728722917567579,0.104776757838616,0.639,0.454,1,0

"Pitpnb",0.000732804553505064,0.161308031626375,0.194,0.112,1,0
"Slc8b1",0.000733365261062692,0.124264149524803,0.084,0.034,1,0
"Arl16",0.000734239080495921,0.11486913437852,0.097,0.042,1,0
"Prox1",0.00073454046953221,0.0673795287698349,0.052,0.016,1,0
"Rdh10",0.000735852248844635,0.116089212845965,0.258,0.156,1,0
"Mdc1",0.000756250172527356,0.0808035714229787,0.071,0.027,1,0
"Dlg5",0.000758202132756061,0.0649921474989974,0.097,0.042,1,0
"Eif3k",0.000759760985923451,0.152382998085156,0.671,0.51,1,0
"Gpc1",0.000762282739154363,0.0564249445727716,0.052,0.016,1,0
"Dpf2",0.00076245861724362,0.102865568857155,0.174,0.096,1,0
"Setdb1",0.000763369315788072,0.112603204465083,0.116,0.055,1,0
"Mta3",0.000763741693866099,0.123102735050392,0.129,0.064,1,0
"Crebrf",0.000765565345076753,0.156051630361087,0.213,0.127,1,0
"Kpna1",0.000766453260280844,0.121869622820023,0.161,0.086,1,0
"Plcb3",0.000766828105539199,0.0692127901738781,0.103,0.046,1,0
"Hps4",0.00076892002664614,0.100348571649991,0.129,0.063,1,0
"Ccdc50",0.000770070312401874,0.216605909194648,0.297,0.199,1,0
"Myo15b",0.000770129518904928,-0.189702197653688,0.019,0.101,1,0
"Cd34",0.000770905130663263,0.110607766219409,0.065,0.023,1,0
"Zkscan1",0.000771368240340711,0.158785431603275,0.245,0.153,1,0
"Entpd5",0.0007747964533426,-0.298813682594495,0.142,0.253,1,0
"Acnat2",0.000776667906773295,-0.198327680570854,0.013,0.09,1,0
"Fabp7",0.000780090998892468,-0.177687532352687,0.006,0.08,1,0
"Xiap",0.0007804044571219,0.156283189388925,0.265,0.166,1,0
"Tmem205",0.000780625587323597,-0.439341178654042,0.387,0.463,1,0
"Rgl2",0.000783311580897141,0.0894420497619507,0.097,0.042,1,0
"Wdr26",0.000784198300727121,0.151746879798404,0.297,0.192,1,0
"Agtr1a",0.000785321340252386,-0.175652990676133,0.006,0.079,1,0
"Zfr",0.000785492050763915,0.179605098795492,0.271,0.177,1,0
"Abhd3",0.000785833474847954,-0.253922332968066,0.045,0.136,1,0
"Aars",0.000788096192794003,0.137538596443258,0.284,0.181,1,0
"Cyb5b",0.000789333374479385,-0.31247678450521,0.361,0.459,1,0
"Epha1",0.000790419306101446,0.0631852267652961,0.071,0.027,1,0
"Glrx3",0.000791170129641008,0.121588079436984,0.4,0.271,1,0
"Dctn6",0.000791232425393843,0.169129252278682,0.194,0.112,1,0
"Dnase2a",0.0007916654972212,0.156874411863474,0.168,0.092,1,0
"Kif3a",0.000791918241212814,0.112034896546817,0.123,0.06,1,0
"Bcl2a1b",0.000792724362810939,0.10373482631479,0.103,0.046,1,0
"Zbtb6",0.000795720107687213,0.0735692005630125,0.09,0.038,1,0
"Mapk1",0.000804134499977683,0.116410815469535,0.348,0.231,1,0
"Nap1l4",0.00080491444325104,0.0698215298691733,0.265,0.159,1,0
"Stat2",0.000805409177912697,0.072125011710926,0.077,0.03,1,0
"Mndal",0.000807177973896878,0.0863678165272377,0.071,0.027,1,0
"Ylpm1",0.000807339263021522,0.126699895417542,0.194,0.111,1,0
"Sgms2",0.000808031558719612,0.0961479938991374,0.252,0.152,1,0
"Rab6b",0.000808559486879074,0.0944914179917086,0.116,0.055,1,0
"Lztfl1",0.000809057831481051,0.111886376205731,0.245,0.15,1,0
"Dcaf6",0.000813039964401797,0.157532860631692,0.161,0.087,1,0
"Carkd",0.000814581982370325,0.0856578079656984,0.297,0.187,1,0
"Fam178a",0.000816410370992771,0.0942425096991423,0.135,0.068,1,0

"Btrc",0.000820764284959569,0.0713066512425229,0.11,0.05,1,0
"Wrb",0.000825114907685881,0.104028433775853,0.097,0.042,1,0
"Tbl1xr1",0.000829602211591037,0.0862582387847597,0.194,0.109,1,0
"Ddx19b",0.00083054655984805,0.0713455546493469,0.071,0.027,1,0
"Mlxipl",0.00083278244624153,-0.261480759919854,0.032,0.12,1,0
"Gpn3",0.000832788500501774,0.0812298142831066,0.11,0.05,1,0
"Uchl3",0.000833509199363059,0.118601102747183,0.329,0.217,1,0
"Nudt8",0.000838970766318107,-0.242562622036876,0.084,0.189,1,0
"Glod5",0.000839179072622213,-0.291132738959379,0.142,0.248,1,0
"Fam120a",0.000847053524374225,0.0674485285893821,0.406,0.265,1,0
"Zfp839",0.000847923558462711,0.0702493984144931,0.084,0.034,1,0
"Extl3",0.000848680878418018,0.0680998215533472,0.11,0.05,1,0
"Slc12a4",0.000851656492806735,0.0970053870668549,0.084,0.035,1,0
"Pik3c3",0.000855510561595661,0.0960672184538478,0.103,0.047,1,0
"Tmed10",0.000856273260409677,0.148651204075643,0.613,0.452,1,0
"Msl3",0.000857861191247126,0.127789712062438,0.2,0.115,1,0
"Mpzl2",0.000860406306887311,0.0815419480421523,0.097,0.042,1,0
"Cwc25",0.000860735560593471,0.0966970648451805,0.123,0.06,1,0
"Gemin7",0.000865846951572084,0.0893274391652183,0.419,0.284,1,0
"Rpf1",0.00086603511254363,0.0923443325667616,0.206,0.118,1,0
"Nin",0.000868227305987353,0.0706621750951003,0.065,0.023,1,0
"Utp11l",0.000871567068776902,0.110356677924776,0.271,0.17,1,0
"M6pr",0.000872755130514479,0.105779555212136,0.439,0.307,1,0
"Stip1",0.000877225637972807,0.117094297858979,0.265,0.166,1,0
"Fmo5",0.000879324754316764,-0.158181696650599,0,0.067,1,0
"Rplp0",0.000879715431015698,0.224494466632943,0.929,0.902,1,0
"AA986860",0.000881103252236133,0.0874228358644734,0.09,0.038,1,0
"Carf",0.00088469513284357,0.142000757508541,0.09,0.039,1,0
"Dguok",0.000886821390548793,0.110919633195578,0.219,0.131,1,0
"Pdxdc1",0.000887730704061693,0.0823091841632468,0.226,0.131,1,0
"Selplg",0.000889483671714451,0.0651814688607051,0.084,0.034,1,0
"Wasl",0.00089016456330786,0.167750157346983,0.4,0.28,1,0
"Znrf3",0.000901310839118064,0.0784489834131014,0.11,0.051,1,0
"Rapgef3",0.000902040146834216,0.141132130404985,0.232,0.142,1,0
"Fxr2",0.000903030332386094,0.087430428788275,0.161,0.086,1,0
"Fam168b",0.000905570122962402,0.116133578772584,0.239,0.145,1,0
"Col1a2",0.000907888175644666,0.0747375263270974,0.071,0.027,1,0
"Stx3",0.000914121046852742,0.0802705261914037,0.232,0.138,1,0
"Frmd4b",0.000924636760067652,0.109296799549849,0.206,0.118,1,0
"Rnaseh1",0.000927841281885702,0.0723550765402435,0.09,0.039,1,0
"Tmem181a",0.000929984381811689,0.0521186704521931,0.071,0.027,1,0
"Araf",0.000934243914583508,0.0992002178970017,0.271,0.167,1,0
"Ahi1",0.000939665041007465,0.107591609770761,0.11,0.051,1,0
"Vcam1",0.00094045689322031,0.125364507386659,0.097,0.042,1,0
"Bace1",0.000942180158213833,0.129205284930792,0.142,0.074,1,0
"Fto",0.000948605951878925,0.137687094128098,0.213,0.127,1,0
"Pfdn4",0.000949080663254877,0.150381653878335,0.29,0.19,1,0
"Tox3",0.00095021875758184,0.114629960126639,0.11,0.051,1,0
"Lig4",0.000951517050722012,0.0831060379556431,0.071,0.027,1,0
"Arl6ip4",0.000953154225923448,0.109141842500007,0.258,0.159,1,0

"Zfp748",0.000955343182389563,0.064311090724515,0.052,0.017,1,0
"Ssr1",0.000955703121109689,0.119801662341573,0.361,0.244,1,0
"Ecscr",0.000957689842731533,0.0686205124535682,0.065,0.024,1,0
"Rpl10a",0.000961885698332661,0.177640503108563,0.89,0.793,1,0
"Gramd4",0.000963813858581551,0.0760269446318208,0.077,0.031,1,0
"Atp2b4",0.000964185848051609,0.0781395836883384,0.09,0.038,1,0
"BC017643",0.000964691622804845,0.0985702163716306,0.097,0.043,1,0
"Adprh",0.000965532890360906,0.072109142731781,0.148,0.077,1,0
"Mtif2",0.000972363602146504,0.113871380422656,0.187,0.106,1,0
"Tjp3",0.000972548832918903,0.0708316724657777,0.09,0.038,1,0
"Vsir",0.000973880113097885,0.0888203504567051,0.09,0.039,1,0
"Xylt2",0.000975734368350036,0.0646853819881196,0.077,0.031,1,0
"Fdft1",0.00097895381752289,0.086360877915358,0.168,0.091,1,0
"Slc36a4",0.000980069299193818,0.06305717272445,0.052,0.017,1,0
"Pgrmc2",0.000981921901029468,0.157599937519588,0.226,0.139,1,0
"Rfc2",0.000982345797009454,0.109092088336319,0.232,0.141,1,0
"Mapk15",0.000984015242976765,0.13059836755009,0.123,0.06,1,0
"Zc3h18",0.000984584311576636,0.0938828628392211,0.168,0.092,1,0
"Arrb2",0.000985044648479057,0.0928144190480752,0.103,0.047,1,0
"Rnf219",0.000986401632600087,0.062894932591541,0.058,0.02,1,0
"Jmjd6",0.000988954337558204,0.0856738371541965,0.155,0.082,1,0
"Havcr1",0.000991845680949299,0.0938136086952874,0.116,0.054,1,0
"Cab39l",0.000991945982152356,0.167766756913389,0.2,0.117,1,0
"Ptges3",0.000994440098104072,0.16550835684696,0.439,0.313,1,0
"Cmtm8",0.00100158443020371,0.119612092911346,0.116,0.056,1,0
"Zfp688",0.00100463273061011,0.084676804711161,0.103,0.047,1,0
"Ttc8",0.00100568351561656,0.100268453024769,0.123,0.06,1,0
"Wtip",0.00100866083408517,0.0781452650843195,0.077,0.031,1,0
"Ptpn2",0.00101207306468988,0.118524191294702,0.148,0.078,1,0
"Dtx3",0.00101408389837964,0.101843861257617,0.219,0.131,1,0
"Csnk1d",0.00101900576862441,0.107994396598608,0.206,0.12,1,0
"Mitf",0.00102133483083869,0.139165153264871,0.155,0.084,1,0
"Rps29",0.00102142820515843,0.132787197087755,0.994,0.97,1,0
"Nagk",0.00102230196718525,0.148318110789785,0.258,0.164,1,0
"Mfsd1",0.00102319463849991,-0.333578082438331,0.284,0.389,1,0
"Slc2a5",0.00102922192911543,-0.187642378753332,0.013,0.088,1,0
"Pear1",0.00102943683989931,0.0571895661024474,0.052,0.017,1,0
"Rnf31",0.00103425890193888,0.0912806412858546,0.097,0.043,1,0
"Spg20",0.00104449524908399,0.0727920912670769,0.103,0.047,1,0
"Cux1",0.00105562031971949,0.11896650070621,0.465,0.332,1,0
"Coa4",0.00105825935089158,0.078487546674911,0.116,0.055,1,0
"Smim14",0.0010590159098477,0.065130702465846,0.477,0.327,1,0
"Zswim6",0.00105904395525126,0.104898406932844,0.097,0.043,1,0
"Fam109a",0.00106167181010938,0.0649813890936003,0.084,0.035,1,0
"Pygb",0.00106757244302319,0.174810081630437,0.155,0.084,1,0
"Slc19a1",0.00106967830880365,-0.224658985599859,0.052,0.144,1,0
"Trrap",0.00107123921557824,0.110536829609513,0.161,0.088,1,0
"Mkrn2os",0.00107172280598826,0.068545088856097,0.129,0.063,1,0
"Ppp1r12c",0.00107254834123069,0.100354495427254,0.123,0.06,1,0
"Cds1",0.00107890253439863,0.107293850525717,0.194,0.111,1,0

"P3h2",0.00108467445411182,0.0970822213862239,0.303,0.194,1,0
"Cdk5rap3",0.00108895571123482,0.115705216739402,0.219,0.132,1,0
"Dclk3",0.00109012233806813,0.0930036737448236,0.103,0.047,1,0
"Rpl9",0.00109013629218922,0.200365367746116,0.948,0.824,1,0
"Lig1",0.00109035823558457,0.0933009971883235,0.077,0.031,1,0
"Upf3a",0.00109061935363105,0.0769007167290722,0.155,0.082,1,0
"Katnal1",0.00109166069594887,0.0613309004395009,0.084,0.035,1,0
"Cdyl",0.00109829769354236,0.0795731860564696,0.071,0.028,1,0
"Pold3",0.00110048338214864,0.0918929172536117,0.11,0.051,1,0
"Mob3c",0.0011004896386613,0.0589372508348047,0.097,0.043,1,0
"Apeh",0.00110226996115592,-0.282978411252831,0.129,0.233,1,0
"Abcc5",0.00110365247617418,0.113620602208163,0.129,0.064,1,0
"Pla2g15",0.00110666881552486,0.0811925633404991,0.071,0.028,1,0
"Zufsp",0.00110786108551731,0.0668543434663846,0.084,0.035,1,0
"Zfp687",0.00110926989765484,0.0880393228322043,0.11,0.052,1,0
"Gemin8",0.00111066214529084,0.0688454637247606,0.077,0.031,1,0
"Arhgap8",0.00111103823487243,0.0579066585561034,0.058,0.02,1,0
"Atm",0.001113103728899,0.0826154272184853,0.09,0.039,1,0
"1810037I17Rik",0.00111328455654396,0.192284960935495,0.613,0.456,1,0
"Mbd5",0.00111593097134357,0.111047181071677,0.097,0.044,1,0
"Saal1",0.00111615463894423,0.0533845330981761,0.071,0.027,1,0
"Gm8186",0.00111897632262457,0.114926829073691,0.29,0.183,1,0
"Zfc3h1",0.00112248557283907,0.111351668656324,0.161,0.088,1,0
"Prrc2b",0.00112507916325031,0.109122433048608,0.258,0.162,1,0
"Necap2",0.00112552339273464,0.0769998004308508,0.245,0.149,1,0
"Agbl3",0.00112836021294484,0.0638401180245034,0.129,0.063,1,0
"Xpo6",0.00113024039648662,0.099676025751112,0.148,0.078,1,0
"Tmed6",0.00113263746992212,-0.205139772185727,0.039,0.126,1,0
"A1cf",0.00113463798426801,-0.138889753361404,0,0.064,1,0
"Pxmp4",0.00113492902864899,-0.250794764625859,0.097,0.2,1,0
"Ndufv1",0.00113671548490688,-0.29514381540708,0.374,0.47,1,0
"Sav1",0.00114227907754261,0.129683660580505,0.148,0.079,1,0
"Slc5a10",0.00114692996694502,-0.210347754714127,0.006,0.075,1,0
"Ppat",0.00114718261719676,0.143594444409476,0.187,0.109,1,0
"Phf21a",0.00114823524732466,0.109570839742013,0.161,0.088,1,0
"Sde2",0.00114866478879743,0.104397471073212,0.135,0.07,1,0
"Pls1",0.00115119084057209,-0.186087236730961,0.032,0.116,1,0
"Sec11a",0.0011604162877002,0.150054946467158,0.432,0.313,1,0
"Pcbp1",0.00116957997541523,0.165259853520287,0.574,0.428,1,0
"Sec24c",0.00117480846679185,0.102093182199309,0.2,0.116,1,0
"Ttll10",0.00117831713348438,0.0646370417530321,0.084,0.035,1,0
"Arsa",0.00118021267055795,0.059240854123887,0.058,0.02,1,0
"Elp5",0.0011849586266688,0.0854402589152075,0.206,0.121,1,0
"Ppp2cb",0.00118614560192734,0.130196149436089,0.206,0.123,1,0
"Xrn2",0.00118714971270549,0.144341430561185,0.265,0.168,1,0
"Gnpda1",0.00118930406042921,-0.24773268074733,0.097,0.2,1,0
"Fam102a",0.00119237243036922,0.104120562485865,0.206,0.121,1,0
"Ap1m2",0.00119308370377628,0.11477689828947,0.129,0.066,1,0
"Klhl12",0.00119356916516887,0.0780363422938997,0.123,0.06,1,0
"Pex3",0.00119643322250916,0.122590585423135,0.168,0.094,1,0

"Tmem11",0.0012016133595983,0.134691426685249,0.239,0.147,1,0
"Anks3",0.00120306528202375,0.0777131903118941,0.155,0.082,1,0
"Ypel5",0.00120640107992818,0.101670897021352,0.161,0.089,1,0
"Rab12",0.00120758514318028,0.169450356946593,0.168,0.095,1,0
"Zfp414",0.00121050792582755,0.0785754329260899,0.103,0.047,1,0
"Trim28",0.00121224926888018,0.0938481432531289,0.219,0.131,1,0
"Rac3",0.00122360360318578,0.0698895948661615,0.084,0.036,1,0
"Hnrnph2",0.00122585813671646,0.137211134749899,0.284,0.182,1,0
"Cdca4",0.00122744235406151,0.0693387351700664,0.065,0.024,1,0
"Hesx1",0.00123040157843847,0.0645438395078625,0.065,0.024,1,0
"Srgap2",0.0012349647585696,0.0800134011548688,0.11,0.052,1,0
"Chn2",0.00123629933800168,0.123773138784062,0.077,0.032,1,0
"Mpv17l",0.00123775844152747,-0.33568878419044,0.11,0.211,1,0
"Ube2d2a",0.00123970828125574,0.0754054340954847,0.471,0.322,1,0
"Smek1",0.00124011284439189,0.109769233267417,0.213,0.126,1,0
"Ube2d1",0.00124756936313041,0.129579726220504,0.174,0.099,1,0
"Cables1",0.00124818442972258,0.0612493597162697,0.052,0.017,1,0
"Zfp593",0.00124868204338438,0.102260577478914,0.142,0.074,1,0
"Prdx3",0.00125117350323977,-0.303992862896977,0.29,0.393,1,0
"Pold2",0.00125769618023457,0.0968323494061515,0.084,0.035,1,0
"Sccpdh",0.00125981613395764,0.0562183540024718,0.123,0.06,1,0
"Ap1s2",0.00126492552178883,0.070081859880757,0.077,0.032,1,0
"Mxra7",0.00127181683487304,0.055600672812289,0.09,0.039,1,0
"Ankzf1",0.00127679213584995,0.0981121987957273,0.103,0.048,1,0
"Cd3g",0.00128458139117926,0.139475265143723,0.071,0.028,1,0
"Cetn2",0.00128860174214784,0.129967707103186,0.413,0.293,1,0
"Tap1",0.00128993535025659,0.115517389622415,0.103,0.048,1,0
"Ppp1cb",0.0012916233384697,0.119093914059044,0.445,0.308,1,0
"Qrich1",0.00129197328128798,0.140154682927798,0.213,0.13,1,0
"Snx14",0.00129299356297589,0.106380139164798,0.155,0.083,1,0
"Pym1",0.00129522301963905,0.100473405685397,0.116,0.057,1,0
"1810022K09Rik",0.00129972886423985,0.158973320403171,0.387,0.271,1,0
"Zfp281",0.00130025422311925,0.10229041254801,0.142,0.074,1,0
"Smdt1",0.00130265239500379,-0.238185487452606,0.794,0.773,1,0
"Os9",0.00130468233966109,0.104183051623203,0.348,0.232,1,0
"Ccp110",0.00130891400173112,0.0831872739373111,0.065,0.024,1,0
"H2afy2",0.00130923247192579,0.0736802844932347,0.09,0.039,1,0
"Gpatch11",0.00131443849276567,0.0778674303643167,0.168,0.092,1,0
"Adam10",0.00131887343243841,0.141948863310711,0.245,0.154,1,0
"Akap10",0.00132052043444729,0.080697058703521,0.103,0.048,1,0
"Manf",0.00132294060359019,0.129954594926091,0.548,0.408,1,0
"Ncoa2",0.00132629204650815,0.118307244702913,0.161,0.089,1,0
"Timm9",0.00133060628627547,-0.261922921519242,0.097,0.195,1,0
"Ppp1r3e",0.00133066468587339,0.0553417560525088,0.058,0.021,1,0
"Plxna1",0.00133072688583934,0.115677982341674,0.103,0.048,1,0
"Slco2a1",0.00133490553550145,0.139591911055067,0.077,0.032,1,0
"Leprotl1",0.00133817937212293,0.122735703099441,0.226,0.136,1,0
"Kdm4b",0.00134006622233313,0.0644199194273616,0.077,0.032,1,0
"Magi3",0.00134156444962941,0.104656269717907,0.11,0.053,1,0
"Tor3a",0.00134647045163667,0.0997842265865083,0.097,0.044,1,0

"Tinag",0.00134741938562715,-0.358425282721954,0.239,0.334,1,0
"Atp7a",0.00134808272574096,0.0951966075023434,0.116,0.057,1,0
"Ap5s1",0.00134843532493966,0.11753999435667,0.123,0.062,1,0
"Krr1",0.0013499125449241,0.0865469884434842,0.135,0.07,1,0
"Cenpb",0.00135559252188088,0.1534118073925,0.439,0.313,1,0
"Fbxl19",0.00136001286292973,0.0645257592294202,0.084,0.036,1,0
"Nos1ap",0.00136639842009387,0.103177357177378,0.116,0.057,1,0
"Mut",0.00137068135356374,-0.332411652026292,0.2,0.297,1,0
"Ccdc47",0.00137580915470515,0.12114178625403,0.323,0.219,1,0
"Pex11b",0.00137638683082468,0.0551156129272296,0.148,0.077,1,0
"Pcnxl4",0.00137665035551803,0.082027355309001,0.09,0.04,1,0
"Tyms",0.00137732477104662,0.104777362480658,0.142,0.073,1,0
"Pld3",0.00137743141856975,0.101977475602233,0.239,0.146,1,0
"Trappc5",0.00138150031814933,0.0843717436923283,0.277,0.176,1,0
"Bcl9l",0.00138995093746033,0.0817813847588648,0.09,0.04,1,0
"Zbtb2",0.00139068140265401,0.0561780029828434,0.058,0.021,1,0
"RP23-306P12.3",0.00139203988735194,-0.305418315590101,0.006,0.073,1,0
"Eif3e",0.00139912277521267,0.11342640340055,0.49,0.35,1,0
"Hspa9",0.00140384158887445,-0.309232364722357,0.594,0.608,1,0
"Phc2",0.00140404925383771,0.109384481209022,0.161,0.09,1,0
"Pop4",0.00140410552021577,0.0930063901779663,0.129,0.066,1,0
"Ccdc86",0.00140431034071473,0.0911124925466352,0.097,0.044,1,0
"Ahcy",0.00140569069586972,-0.14724920849411,0,0.062,1,0
"Sh3kbp1",0.00141166328170781,0.0780991384476586,0.058,0.021,1,0
"Specc1l",0.00141219716133185,0.0810792341082707,0.116,0.057,1,0
"Emp1",0.00141315564405851,0.149774808494617,0.097,0.044,1,0
"Qtrt1",0.00141368793804607,0.0787368176640314,0.084,0.036,1,0
"Kcnk5",0.00141580148202318,-0.195436488976738,0.026,0.103,1,0
"Rnf217",0.00142161647958651,0.0997382678859752,0.077,0.032,1,0
"Mthfd1",0.0014287572822335,-0.239736645753435,0.123,0.228,1,0
"Jade1",0.00143116980241461,0.117375250751404,0.239,0.146,1,0
"Nr2c2",0.00143738737709215,0.150778029029462,0.161,0.091,1,0
"Rpl22l1",0.00143960117547542,0.205893865728304,0.858,0.706,1,0
"Wash1",0.00143997310942621,0.100534349987002,0.161,0.089,1,0
"Wdr43",0.00144349576518588,0.112486807614409,0.181,0.105,1,0
"Mboat7",0.00145287895079226,0.117857960672182,0.161,0.09,1,0
"C5ar1",0.00145458730188288,0.0575407262796775,0.065,0.024,1,0
"Stx2",0.00146030106027919,0.0736047023141919,0.065,0.024,1,0
"Klhdc3",0.00146074876388651,0.083609519398416,0.155,0.084,1,0
"Mab21l3",0.00146191017494238,0.0627181250695046,0.058,0.021,1,0
"Ywhag",0.00146283443638004,0.12645359677219,0.252,0.162,1,0
"Slc5a11",0.00146323733991399,-0.147852030042796,0,0.061,1,0
"Rpl38",0.00146979251051783,0.157600731972504,0.981,0.931,1,0
"Rpl39",0.0014700457166706,0.182085993056028,0.942,0.9,1,0
"Nol12",0.00147329417930202,0.116428586606487,0.155,0.085,1,0
"Dars",0.00147546168173768,0.147186860268564,0.29,0.193,1,0
"Klhdc8b",0.00147963175462635,0.0812590031303649,0.065,0.025,1,0
"Tmtc3",0.0014808716203293,0.0620342424983093,0.116,0.056,1,0
"Rictor",0.00148211195989715,0.0905713264544785,0.116,0.057,1,0
"Scpep1",0.00148312334425786,-0.247063844094661,0.11,0.21,1,0

"Esrp2",0.00148700446364321,0.081141282033966,0.09,0.04,1,0
"Sf3a3",0.00148790056415198,0.113109831317226,0.148,0.081,1,0
"Tprkb",0.00149018368288834,-0.242142339705954,0.097,0.196,1,0
"Spata5",0.00149501313342946,0.0742605031822934,0.058,0.021,1,0
"Slain2",0.00149910538778554,0.118240721680488,0.219,0.134,1,0
"Ago1",0.00150061872016706,0.133074507980368,0.129,0.067,1,0
"Pxk",0.00150267682341194,0.124758436516691,0.213,0.129,1,0
"AW549877",0.00150733889943661,0.0929671133248307,0.2,0.117,1,0
"Nup88",0.0015083089302959,0.086704741953535,0.155,0.083,1,0
"Thap11",0.00151299994861611,0.103591785744619,0.142,0.075,1,0
"Ppp1r1b",0.00151641510094259,0.269647906738906,0.103,0.049,1,0
"Adap2os",0.00151929468367544,-0.196156251877055,0.019,0.094,1,0
"Pdha1",0.00153872265435519,-0.335973551218436,0.452,0.506,1,0
"Lcp2",0.00154015464205409,0.0606150266399078,0.052,0.017,1,0
"Tbc1d12",0.00154120662993115,0.0855210109894866,0.097,0.044,1,0
"Eif2s1",0.00154134554746396,0.127356678335798,0.239,0.151,1,0
"Zfpl1",0.00154207495276556,0.06692172928242,0.155,0.083,1,0
"Npat",0.00154257776136025,0.112739802702304,0.084,0.036,1,0
"Creb1",0.00154626137627482,0.135425130549782,0.168,0.096,1,0
"Ssna1",0.00154667462380309,0.0907205289347282,0.297,0.19,1,0
"B2m",0.00155099177045863,0.2305546473392,0.852,0.715,1,0
"Eif4h",0.00155139241433973,0.178481521130625,0.374,0.265,1,0
"Rpa3",0.00155232737559171,0.119776207896262,0.168,0.094,1,0
"Camk2n1",0.00155832973046586,0.243913419774503,0.587,0.444,1,0
"Asb3",0.00155876816688296,0.0693218307862619,0.071,0.028,1,0
"Gm16685",0.00155938375990967,0.0875945466536893,0.097,0.044,1,0
"Rsph9",0.00155992979624753,0.0870500881899767,0.135,0.07,1,0
"Dcaf15",0.00156093130293999,0.0534169585169075,0.077,0.032,1,0
"Sec16a",0.00156118304071193,0.116801690178468,0.161,0.09,1,0
"Mthfd2l",0.00156195920759967,0.107707216523908,0.258,0.164,1,0
"Tomm22",0.00156381642240429,0.095878830436064,0.51,0.368,1,0
"Wdr33",0.00157261786180577,0.102324878737946,0.155,0.085,1,0
"Ddx52",0.00157386315005557,0.0549853337940406,0.181,0.101,1,0
"Cd93",0.00158434324540134,0.0841700945915342,0.058,0.021,1,0
"Slu7",0.00158459069181743,0.135945088930851,0.181,0.105,1,0
"Galnt4",0.00159045762289617,0.0577332984993524,0.071,0.028,1,0
"Sun1",0.00159290268690215,0.0718061792262869,0.148,0.079,1,0
"A230046K03Rik",0.00159376206979023,0.152994781013621,0.194,0.114,1,0
"Hyou1",0.00159544053575617,0.090936947539021,0.11,0.052,1,0
"Syngr2",0.00159626824792937,0.0828725518477661,0.361,0.245,1,0
"Med13",0.00159905488997511,0.132410677439898,0.2,0.12,1,0
"Dimt1",0.0016175634936709,0.0544422744415668,0.071,0.028,1,0
"Kalrn",0.00161921273574382,0.0748417397637531,0.09,0.04,1,0
"Rab21",0.00162062927744547,0.125621492920242,0.174,0.099,1,0
"Eif4b",0.00162142975560217,0.12534439675945,0.432,0.309,1,0
"Cx3cr1",0.00162445268047296,0.134647369972429,0.097,0.045,1,0
"Appbp2",0.00162679290836834,0.101897952260262,0.226,0.138,1,0
"Eif2ak2",0.00162991744786315,0.102211196993376,0.129,0.067,1,0
"Ftsj3",0.00163962605929062,0.095345347377963,0.129,0.066,1,0
"Ola1",0.00164315475659836,0.102225251339948,0.226,0.138,1,0

"Prelid1",0.00164524748444158,-0.321517029075927,0.419,0.493,1,0
"Zfp51",0.00165236914226985,0.054010536308568,0.058,0.021,1,0
"Cbx4",0.00165511544812291,0.0734642842192932,0.097,0.044,1,0
"Ankra2",0.00165780477699677,0.103557900750865,0.142,0.076,1,0
"Tomm34",0.00165951631381731,0.103537337314049,0.135,0.071,1,0
"Slc39a13",0.00166259802638911,0.0735579019569727,0.116,0.057,1,0
"Znhit6",0.00166537682129162,0.115898814430644,0.123,0.062,1,0
"Eif2b3",0.00167381274687696,0.0932424691762165,0.103,0.049,1,0
"Acvr1b",0.00167756858478362,0.0560826723641933,0.129,0.065,1,0
"Zmynd11",0.00168475526416507,0.144629004115429,0.29,0.195,1,0
"Fam98c",0.00169274153819295,0.100985510515999,0.148,0.08,1,0
"Zfp945",0.00169388471677737,0.0815573590692249,0.148,0.08,1,0
"Nln",0.0016962886563321,0.0857915899944082,0.103,0.049,1,0
"Cnih4",0.00169833783761865,0.086487429172142,0.413,0.285,1,0
"Sult1c2",0.001698882109318,-0.23393987840182,0.058,0.146,1,0
"Nrros",0.00172082640647437,0.0744558849320917,0.065,0.025,1,0
"Raver1",0.0017424173174093,0.0842146309113544,0.116,0.057,1,0
"Lypla1",0.00176153587048886,-0.368377647453879,0.284,0.369,1,0
"Fbp2",0.00176424526323208,-0.138029563126027,0,0.06,1,0
"Taf11",0.00177073804064267,0.0976544121710766,0.181,0.104,1,0
"Pdcl",0.00177172888099475,0.0848857064406066,0.155,0.085,1,0
"Psmd13",0.00178544339587382,0.123130428821285,0.323,0.218,1,0
"Treh",0.00179591867895506,-0.188114481546562,0.013,0.081,1,0
"Rcbtb2",0.00179692980576936,0.0909388709007822,0.174,0.098,1,0
"Abtb1",0.00180028344482516,0.0875734077513439,0.11,0.053,1,0
"Optn",0.00180174538241773,0.0933275325284607,0.077,0.033,1,0
"Lyrm2",0.00180907775860653,0.10434271455185,0.29,0.189,1,0
"Kat8",0.00180914486722386,0.0897841514946383,0.077,0.033,1,0
"Ube2s",0.00181026304694175,0.11106906497886,0.535,0.388,1,0
"Smap2",0.00181052613405325,0.0933705019654625,0.168,0.094,1,0
"Klhl26",0.00182046218992408,0.0662675354385109,0.058,0.021,1,0
"Sod1",0.00182227096100425,-0.334610941097306,0.4,0.476,1,0
"Supt6",0.00182477824740643,0.107085905773493,0.174,0.1,1,0
"Arhgap6",0.0018294064468764,0.0974990068401229,0.103,0.049,1,0
"Ilkap",0.00183133656413347,0.102308795782336,0.265,0.17,1,0
"Atf1",0.00183555772374751,0.105136589939374,0.168,0.095,1,0
"Dhx37",0.00183745649277182,0.057773986871155,0.058,0.021,1,0
"Fdx1",0.00184071354614473,-0.3564531691674,0.355,0.429,1,0
"Fam208b",0.001841957222902,0.0513520772024477,0.103,0.048,1,0
"N6amt1",0.00185239098398981,0.127702651517422,0.245,0.155,1,0
"Ilf3",0.00185328554583728,0.0956041694546071,0.135,0.071,1,0
"Ndufa12",0.0018601090997965,-0.24933314588603,0.787,0.783,1,0
"Ralgapa1",0.0018611032785335,0.168430758712361,0.181,0.107,1,0
"Med23",0.00186215483791133,0.0593856160704293,0.09,0.04,1,0
"Carhsp1",0.00186339302238485,0.0982182320374448,0.219,0.131,1,0
"Cops7b",0.00186384614243942,0.109745808834123,0.09,0.041,1,0
"Lrrc41",0.0018763789021087,0.101409834130172,0.155,0.086,1,0
"Atg4b",0.00187924432284047,0.109290109014771,0.155,0.086,1,0
"Tulp4",0.00187967485876735,0.199260121737935,0.277,0.187,1,0
"Gm27216",0.00188602403570162,-0.153564752658276,0,0.059,1,0

"Fbxo32",0.00188676006983471,0.0633429307048879,0.052,0.018,1,0
"Nap1l1",0.00189147534033977,0.0901721585356455,0.419,0.295,1,0
"Abhd4",0.00189240607649509,0.132778495615105,0.142,0.077,1,0
"Ift122",0.00189336965565817,0.0949233164391637,0.155,0.085,1,0
"Rlim",0.00189551285957883,0.115041831921371,0.181,0.105,1,0
"Tmem127",0.00191017110809413,0.0970478348644578,0.2,0.12,1,0
"2610021A01Rik",0.00191337474519467,0.0567252792373597,0.052,0.018,1,0
"Slc15a3",0.00192411682114127,0.0805735158720244,0.052,0.018,1,0
"Tbl3",0.00192566528430023,0.0841533971562654,0.116,0.058,1,0
"Gm4131",0.00193704040660163,-0.141676939092309,0,0.059,1,0
"Tbc1d14",0.0019405223944528,0.100886860011908,0.174,0.099,1,0
"Papolg",0.0019502633396709,0.0615442441599001,0.058,0.021,1,0
"Naca",0.00195406904763245,0.172925426100604,0.845,0.742,1,0
"Cramp1l",0.00195426109948718,0.102158807991489,0.103,0.05,1,0
"Gnpda2",0.00195641247292578,0.113830163162872,0.129,0.067,1,0
"2900026A02Rik",0.0019589513021812,0.0886965177085505,0.097,0.045,1,0
"Wdr13",0.00197018587214727,0.111035270880527,0.148,0.081,1,0
"Prpf8",0.00197132282881683,0.0669041869739028,0.245,0.152,1,0
"Tada2a",0.00197344862265435,0.0630411268718503,0.077,0.033,1,0
"Ube4a",0.00198657100711813,0.0742232717318586,0.226,0.138,1,0
"Rnf181",0.00198783109212008,-0.281702981037381,0.226,0.328,1,0
"Wdr89",0.00199064419988713,0.116152077681509,0.645,0.485,1,0
"Zfp30",0.0019972008985455,0.105645352395973,0.116,0.059,1,0
"Psmd8",0.00199787672772008,0.129440822793999,0.497,0.368,1,0
"Ocrl",0.00200093684438126,0.101178065469924,0.116,0.058,1,0
"Ndufa8",0.00200605679150166,-0.264148211819062,0.677,0.681,1,0
"Mapk8ip3",0.00201669112361257,0.0931988759106593,0.129,0.067,1,0
"Llgl1",0.00201774132895031,0.0811066855538928,0.077,0.033,1,0
"Psma6",0.00201908632224814,0.108884316072155,0.445,0.307,1,0
"Abhd14b",0.00201953215023277,-0.324426558837693,0.245,0.345,1,0
"Fbxo44",0.00202920654904012,0.119707218774917,0.135,0.073,1,0
"Eif1b",0.00203688183887919,0.111938850499399,0.355,0.244,1,0
"Nob1",0.00203958860125668,0.0872246546055179,0.116,0.058,1,0
"Nup43",0.0020402014528741,0.0712122783216439,0.084,0.036,1,0
"Rpl34",0.00204873708044252,0.175418975645529,0.935,0.839,1,0
"Zcchc8",0.00205382821606226,0.0901225014548535,0.148,0.08,1,0
"Tdrd3",0.00205615447345906,0.115412839199149,0.103,0.05,1,0
"Acsm3",0.00205742194740398,-0.252587147609793,0.013,0.08,1,0
"Stx12",0.00205767820704264,0.108351298960446,0.206,0.126,1,0
"Pebp1",0.00206797030136736,-0.270596895722337,0.748,0.742,1,0
"Adrbk1",0.00207218786378308,0.10803880209594,0.148,0.082,1,0
"Flt1",0.00207480627043201,0.127650818036192,0.065,0.025,1,0
"Mkrn2",0.00207860963716001,0.10412628949405,0.11,0.054,1,0
"Tfe3",0.00208719770921272,0.0899049708235525,0.11,0.054,1,0
"1700049G17Rik",0.00208826046482025,0.0658888281022932,0.071,0.029,1,0
"Tsn",0.00208923049843512,0.141479589209774,0.361,0.258,1,0
"Tmem245",0.00209741541679843,0.112416942091801,0.245,0.155,1,0
"Brwd1",0.00209836570002597,0.104498211312845,0.213,0.132,1,0
"Tpp2",0.0021028473936153,0.114728366912007,0.168,0.097,1,0
"Wwox",0.00211006693782659,0.0996967719174447,0.116,0.058,1,0

"Gm20045",0.00211224131994996,0.0786945086820194,0.077,0.033,1,0
"Grpel2",0.00211372701037721,0.131039732866439,0.206,0.125,1,0
"Mrpl3",0.00211697915339002,0.0761120851650398,0.2,0.119,1,0
"Orc5",0.00212087079095162,0.0557407889837258,0.071,0.029,1,0
"Tmem214",0.00212689354105354,0.0956376872401324,0.174,0.1,1,0
"Vstm5",0.00212924022289664,0.0728406138110337,0.052,0.018,1,0
"Zfp64",0.00213626278823417,0.0700710561830901,0.084,0.037,1,0
"H2-M3",0.00213998015178314,0.0585952786351116,0.058,0.022,1,0
"Fmr1",0.00214384287383502,0.127015383072172,0.219,0.138,1,0
"Htra1",0.00214773556222639,-0.225030620320224,0.071,0.162,1,0
"Dopey2",0.00214907969424614,0.0597383483596335,0.077,0.033,1,0
"Tmem87b",0.00216668717175082,0.103296600028018,0.194,0.115,1,0
"Iws1",0.00216935026739305,0.128041627485262,0.161,0.092,1,0
"Nfatc2ip",0.00216962585380101,0.0503675047522585,0.052,0.018,1,0
"Tm6sf1",0.00217299812919231,0.0728204453097653,0.065,0.025,1,0
"2700060E02Rik",0.00217344862214926,0.136515878156179,0.6,0.462,1,0
"Cdk13",0.00218933761820196,0.102485585790082,0.174,0.101,1,0
"Rbm10",0.00219139945639099,0.0869480499051699,0.148,0.081,1,0
"Myo5c",0.002192705947496 7,0.0806904380231808,0.071,0.029,1,0
"Gm15217",0.00219620592180965,-0.162005546432925,0.013,0.08,1,0
"Chchd4",0.00219879537173957,-0.261399723773971,0.155,0.255,1,0
"Gtf3c5",0.00220819274911082,0.0833291370278031,0.077,0.033,1,0
"Ift46",0.00221262190921163,0.0559341356210109,0.168,0.094,1,0
"Adck3",0.0022196876123327,-0.234773145836854,0.084,0.173,1,0
"Desi2",0.00222138181808729,0.112190418120518,0.161,0.091,1,0
"Bcl7b",0.00222452490918316,0.123896700760207,0.265,0.172,1,0
"Zfp580",0.00223456266193985,0.0590671969651989,0.065,0.025,1,0
"Gorasp2",0.00223870716834079,0.0820244833088269,0.239,0.15,1,0
"Nipa1",0.0022488604801184,0.0551884553944548,0.058,0.022,1,0
"Amd1",0.00225412912916891,0.115010826356183,0.232,0.148,1,0
"Poldip3",0.0022556102724826,0.118557411701582,0.155,0.087,1,0
"Slc43a2",0.00226139877462378,-0.239942566770582,0.2,0.305,1,0
"C1d",0.00227135653117809,-0.308741304845886,0.258,0.357,1,0
"Jarid2",0.00227551519958368,0.151369230202708,0.2,0.121,1,0
"Zfp612",0.00228322373033683,0.0665880269024087,0.077,0.033,1,0
"Twistnb",0.00229120161352375,0.129181572467581,0.155,0.088,1,0
"Osgep",0.00230036316235335,0.155729535599209,0.181,0.108,1,0
"Hap1",0.00231014049154439,0.0507622332833808,0.052,0.018,1,0
"Nlrp6",0.0023106677682641 2,-0.152365955162335,0.013,0.079,1,0
"Sgta",0.00231095657950743,0.0880889695170505,0.271,0.177,1,0
"Zfp866",0.00232472943429755,0.0677982361871658,0.077,0.033,1,0
"Them6",0.00233187104054101,0.139720351215446,0.116,0.059,1,0
"Gcat",0.0023362860662424,-0.247663275197247,0.097,0.189,1,0
"Sdf2",0.00234118544057756,0.0907114741861209,0.348,0.238,1,0
"Trpm7",0.00234203737552403,0.206113945219147,0.31,0.211,1,0
"Hdac8",0.00234673658139206,0.0695591400328009,0.071,0.029,1,0
"Mafg",0.00235841900188016,0.123477805461869,0.31,0.209,1,0
"Hilpda",0.00236210906926313,0.0576896768688774,0.065,0.025,1,0
"Zmym4",0.00236297474690566,0.110171824796634,0.142,0.078,1,0
"Tbcb",0.00236889227196611,0.127903187706342,0.471,0.341,1,0

"Pdcd11",0.00237165802078727,0.117241581122951,0.155,0.088,1,0
"Ptprg",0.0023733903480091,0.122706732993411,0.148,0.082,1,0
"Tmbim6",0.0023789874757068,-0.167074891478368,0.916,0.877,1,0
"Tvp23b",0.0023795590760991,0.101482946999444,0.265,0.171,1,0
"Cirh1a",0.0023822296888558,0.0763526666577193,0.097,0.045,1,0
"Cacnb3",0.00238358477732804,0.0773849134596622,0.077,0.033,1,0
"Vps33b",0.00238837757883275,0.0851902641636354,0.071,0.029,1,0
"Tcea1",0.00239866712097897,0.095197293844866,0.348,0.237,1,0
"Usp14",0.00240841259763069,0.0911655769162603,0.194,0.115,1,0
"Arid2",0.00241427088031362,0.101999568939813,0.148,0.082,1,0
"Lyst",0.00242054230261351,0.0590879924977052,0.071,0.029,1,0
"Rab23",0.0024254079284434,0.102657406608815,0.077,0.033,1,0
"Igfbp5",0.0024438396713412,-0.0532370347907381,0.11,0.209,1,0
"Fam104a",0.00244476577442514,0.128919787923743,0.432,0.311,1,0
"Rsl24d1",0.00244697017764717,0.0791635145448015,0.187,0.111,1,0
"Rsl1d1",0.00244724023685744,0.126976825361102,0.252,0.165,1,0
"Haus8",0.00244750603886994,0.0562875204110999,0.071,0.029,1,0
"Pla2g16",0.0024476360837387,0.0911574732391024,0.174,0.101,1,0
"Tle2",0.00245227291571315,0.0881910355354743,0.09,0.042,1,0
"Rcan3",0.00245568270066649,0.0792752713924832,0.103,0.05,1,0
"Tor1aip2",0.00246258690695965,0.111564832016702,0.297,0.202,1,0
"Rnaset2a",0.00247775399424923,0.0829547791152078,0.477,0.339,1,0
"Lhx1",0.00248637949117233,0.125960206156574,0.116,0.059,1,0
"Ncor2",0.00248771244345428,0.108220741581411,0.148,0.083,1,0
"Bsnd",0.00249097874880158,0.0848962764509845,0.155,0.085,1,0
"Bcl2a1a",0.00249240246054801,0.0544417384439709,0.052,0.018,1,0
"Wdr19",0.00249240246405226,0.0672958584513747,0.052,0.018,1,0
"Med31",0.00249897322136134,0.109859358605908,0.168,0.097,1,0
"1110008L16Rik",0.00250245510651432,0.0681116153603262,0.077,0.033,1,0
"Pbdc1",0.00250577192878808,0.104533047737411,0.226,0.142,1,0
"Golga7",0.00251643123201826,0.17593533865987,0.232,0.149,1,0
"Zfp729b",0.00251954932931371,0.0852431066775946,0.084,0.038,1,0
"Utp14a",0.0025212694965205,0.0843696712838685,0.11,0.054,1,0
"Pdzd8",0.00252434647501741,0.112178883588003,0.174,0.102,1,0
"Cebpz",0.00252700751964197,0.127065846210881,0.245,0.16,1,0
"Ndufaf2",0.00252930205992245,0.0860726598120941,0.303,0.203,1,0
"Dcun1d1",0.002533420566687,0.103658700427565,0.265,0.172,1,0
"Als2cl",0.00253772106880521,0.0746460988350391,0.071,0.03,1,0
"Yipf5",0.00255044622514559,0.113764217612071,0.232,0.147,1,0
"Cops7a",0.00255224899484181,0.12716817828201,0.258,0.17,1,0
"Endod1",0.00255505628186438,0.115426650822864,0.123,0.064,1,0
"G2e3",0.00255672690672939,0.117380046447309,0.097,0.046,1,0
"Uqcrc1",0.00256322809086189,-0.278015389938152,0.613,0.647,1,0
"l7Rn6",0.0025655758684753,0.0671099744450376,0.271,0.174,1,0
"Fam13b",0.00256991508301808,0.0905471498995171,0.084,0.038,1,0
"Pawr",0.00257750019216744,0.0841812221089049,0.09,0.042,1,0
"Sox9",0.00258410125149495,0.141983140164922,0.09,0.042,1,0
"Rac2",0.00258501364975505,0.168993169996655,0.135,0.074,1,0
"Rpp30",0.00258952069221432,0.106371166847414,0.103,0.051,1,0
"Lamc1",0.00259083920631552,0.0909906227192244,0.116,0.059,1,0

"Ccdc160",0.00259267915519012,0.0698740729088594,0.084,0.038,1,0
"Vps13c",0.00260447919324623,0.0729279611017585,0.09,0.042,1,0
"Cers2",0.00260465880190187,0.0930327926573996,0.4,0.285,1,0
"Cog1",0.00260636066570614,0.0956657997752134,0.11,0.054,1,0
"Polr3f",0.00260722686809077,0.0868855499018883,0.11,0.055,1,0
"Ddx39",0.00262139067440552,0.0905582295981178,0.181,0.106,1,0
"Ift52",0.00262242931609271,0.0751921090798515,0.155,0.086,1,0
"Cep295",0.00262296624359927,0.054985548277252,0.097,0.046,1,0
"Wdr48",0.0026253008234436,0.0894463206767754,0.11,0.055,1,0
"Aqp2",0.00263261161913642,0.33888086714166,0.187,0.11,1,0
"Gtf3a",0.00263284446657255,0.100192795295487,0.265,0.173,1,0
"Pgs1",0.00263775892289121,0.0636038118620857,0.129,0.068,1,0
"Ino80e",0.00263919127231929,0.0866533675967008,0.174,0.102,1,0
"Fam76b",0.00264698118128279,0.108605681903377,0.097,0.046,1,0
"Erf",0.00265110674546257,0.0889238274053179,0.155,0.087,1,0
"Rpp21",0.00265862830625365,0.114136564179747,0.406,0.296,1,0
"Atp6v1g2",0.00266440222373719,0.0604490183000981,0.052,0.018,1,0
"Sgsm2",0.00267111126950642,0.056331316546147,0.142,0.076,1,0
"Cd63",0.00267185048635274,-0.216811196868484,0.987,0.949,1,0
"Cdc16",0.00267806129832483,0.101386898172185,0.161,0.092,1,0
"Slpi",0.00267807510007441,0.605496574901906,0.052,0.018,1,0
"Zfp148",0.00267825200260885,0.115571820379495,0.213,0.133,1,0
"Ccrl2",0.00268111009512176,0.250119395242215,0.058,0.022,1,0
"Banp",0.00268682341128258,0.0693488011819546,0.09,0.042,1,0
"Txnrd3",0.00269688487068454,0.0841014177951112,0.09,0.042,1,0
"Trmt112",0.00270074954958117,0.100400699175806,0.406,0.288,1,0
"Snx24",0.00270660818194366,0.0887650944141907,0.123,0.064,1,0
"Adam15",0.00270957204664509,0.0575543862555851,0.155,0.085,1,0
"Trappc6b",0.00271692192012676,0.0730878803644984,0.413,0.285,1,0
"Rab2a",0.00272848958327713,0.115740612184975,0.606,0.464,1,0
"Psmc5",0.00273024758924176,0.0683061079030076,0.4,0.278,1,0
"Odf2l",0.00273289656734856,0.105845637172935,0.123,0.064,1,0
"Polr3a",0.00273291236050389,0.0688995211897012,0.071,0.03,1,0
"Manea",0.00273315556086166,0.0934613338229942,0.116,0.06,1,0
"Gss",0.00273839959327596,-0.330023901814996,0.174,0.264,1,0
"Ago4",0.00275805858488775,0.0547527674919809,0.058,0.022,1,0
"Rai14",0.00275974413475262,0.067980022735059,0.09,0.042,1,0
"Thumpd1",0.00276048475336485,0.121813572710304,0.123,0.065,1,0
"E2f4",0.00276065792303168,0.106699240456585,0.129,0.069,1,0
"Aoah",0.00276332733189146,-0.1497931920367,0.006,0.066,1,0
"Ifit1",0.00276689559430728,0.0640959024840222,0.058,0.022,1,0
"Rnf214",0.00276766954043917,0.11884357276603,0.135,0.074,1,0
"Wwtr1",0.0027713588090198,0.14822220972488,0.213,0.135,1,0
"Gimap5",0.0027735463353277,0.0880076325644897,0.058,0.022,1,0
"Ndufs5",0.00277417720393962,-0.243720172684513,0.761,0.745,1,0
"Ttll5",0.00279202534882121,0.0640197022622744,0.065,0.026,1,0
"Plcd3",0.00279353872554155,0.0830462688775057,0.058,0.022,1,0
"Hpcal4",0.00279402245378743,0.0549916257262776,0.058,0.022,1,0
"Zfp111",0.00279752610729344,0.0500728401610506,0.052,0.018,1,0
"Smc2",0.00279781839367321,0.0609113120169123,0.084,0.038,1,0

"Sp2",0.00280007272440399,0.0688475432841081,0.071,0.03,1,0
"Nedd1",0.0028079867747946,0.116198432620265,0.11,0.056,1,0
"Stat1",0.00280869300999216,0.0961527808854793,0.161,0.091,1,0
"Bnip3",0.00282090948284312,-0.282773368791266,0.232,0.333,1,0
"Zfp513",0.00282203652640431,0.05728549877222,0.09,0.042,1,0
"Gm26890",0.00282402351191274,0.0671170264471058,0.065,0.026,1,0
"Sema4d",0.00282426862174088,0.119136878256082,0.187,0.111,1,0
"Nans",0.00282475827839561,0.0843677657851264,0.135,0.073,1,0
"1810030O07Rik",0.00282494505399848,0.0710915171153071,0.103,0.05,1,0
"Ift20",0.00282837448566025,0.0927854165108888,0.439,0.307,1,0
"Smg9",0.00282936621420161,0.0700870883402428,0.09,0.042,1,0
"Kazn",0.00283030237635124,0.154489991643734,0.181,0.11,1,0
"Fgfr1op",0.00283603459132863,0.0782730026430084,0.116,0.059,1,0
"Lgals9",0.00284447916062776,0.0835009242050092,0.058,0.022,1,0
"Tnpo1",0.00284606877182508,0.164850314366681,0.271,0.183,1,0
"Pus3",0.00284653804594977,0.0687617348511475,0.065,0.026,1,0
"Gabrb3",0.00285809736481309,-0.13914785379231,0.006,0.066,1,0
"Lrrc1",0.00285826199837443,0.0710283832289563,0.123,0.063,1,0
"Phf8",0.00286648428871739,0.0997760726122741,0.077,0.034,1,0
"2210011C24Rik",0.0028665263575591,-0.165159666267811,0.019,0.087,1,0
"Tollip",0.00288148186309824,0.0800931745676329,0.161,0.091,1,0
"Cdkn2aip",0.00288735757027657,0.0732249819982747,0.077,0.034,1,0
"H2afv",0.00289007500651147,0.0818784076631064,0.516,0.366,1,0
"Stk4",0.00290387449263371,0.0693220440176311,0.135,0.073,1,0
"1600012H06Rik",0.00290857437330364,0.103985226014097,0.142,0.078,1,0
"Eif2a",0.00290994446447318,0.0980045372289837,0.258,0.168,1,0
"Sf3b4",0.0029136830223892,0.127476403828239,0.2,0.124,1,0
"Fnbp4",0.0029407827384895,0.0757450485887566,0.142,0.078,1,0
"Tep1",0.00294402141918607,0.111394146311379,0.116,0.06,1,0
"Tgfbr2",0.00296010478128148,0.121426603480159,0.194,0.117,1,0
"Ube2j2",0.00296447260684523,0.107982787113716,0.252,0.164,1,0
"Cpne4",0.00296712192786709,-0.133987748085023,0,0.054,1,0
"Mob4",0.00297009404936719,0.112860797306979,0.277,0.188,1,0
"Gulp1",0.00298923567906684,0.063822535607291,0.065,0.026,1,0
"Mff",0.00299273294758521,0.0881648358636106,0.406,0.288,1,0
"Pdk3",0.00300678435202545,0.0668946920450857,0.161,0.092,1,0
"Nf1",0.00300696674293422,0.113992675916861,0.174,0.103,1,0
"Cyp2d12",0.00301408145964897,-0.288845720134644,0.006,0.065,1,0
"Pacs2",0.00301724893063821,0.0863923047898139,0.116,0.06,1,0
"Gemin5",0.00302307896899831,0.0736249007850383,0.065,0.026,1,0
"Cdkn2d",0.00302610558129284,0.0929790875088507,0.103,0.051,1,0
"Trafd1",0.00303207354475458,0.101460477299382,0.103,0.051,1,0
"H13",0.00303468950457307,0.0968171839233145,0.426,0.298,1,0
"Sucnr1",0.00304110455665126,-0.154052542327664,0.006,0.065,1,0
"Tesk1",0.00304858814072374,0.10948870454306,0.129,0.07,1,0
"Plekhm3",0.00305424313017595,0.0872147766295359,0.065,0.026,1,0
"Rab3gap1",0.00306383725102049,0.133835330768601,0.181,0.107,1,0
"Rpl14",0.00307469089077106,0.193309037422801,0.955,0.882,1,0
"Arf2",0.00307485262541453,0.170899093692159,0.09,0.043,1,0
"Wdr11",0.00309150073723337,0.0620735415908572,0.142,0.077,1,0

```
"Slc22a15",0.00310666226425028,0.110531528516092,0.065,0.027,1,0
"Stard3nl",0.00310820146776364,0.0760305321084701,0.258,0.166,1,0
"Tm9sf2",0.00314486765296864,0.0983299342646556,0.426,0.303,1,0
"Wdr72",0.00315676890721943,0.102604952810774,0.194,0.118,1,0
"Tmem126a",0.00316131804428067,-0.284315300003666,0.2,0.296,1,0
"Fbxw4",0.00318835188005091,0.0503718894966227,0.071,0.03,1,0
"Wbscr27",0.00319394524901997,0.114744246323841,0.161,0.094,1,0
"Lnp",0.00319631495995731,0.089395668581708,0.097,0.047,1,0
"Tmem263",0.00321270732974292,0.0811444228792835,0.077,0.034,1,0
"Nup210",0.00321555895177356,0.053870336111414,0.09,0.042,1,0
"Hoxc10",0.00322227547243536,0.0799077682049745,0.181,0.107,1,0
"Mzt1",0.00322264521639731,0.106255948243473,0.187,0.113,1,0
"Tceb3",0.00322693190707629,0.133196510956175,0.135,0.075,1,0
"Anapc13",0.00322870255959549,-0.262952151643149,0.71,0.694,1,0
"Anapc1",0.0032304533856177,0.0928591683759322,0.174,0.102,1,0
"Hmgcl",0.00323373768438912,-0.26768929555812,0.116,0.208,1,0
"Dtd1",0.00323639530532043,0.0571441670043805,0.148,0.082,1,0
"Defb42",0.00323773613133649,0.069507504626227,0.071,0.03,1,0
"Gm20342",0.00324601646231682,0.0840725813834794,0.116,0.06,1,0
"Dpp3",0.00325507252558089,0.12509280552109,0.181,0.11,1,0
"Rexo4",0.0032555323500332,0.0769569009508749,0.116,0.06,1,0
"Sun2",0.00326096320241146,0.107470538764808,0.09,0.043,1,0
"Epb41l3",0.00326652126228753,-0.199485550420863,0.045,0.122,1,0
"Tmed1",0.00326998027949113,0.0711644882214183,0.174,0.102,1,0
"Hist1h4h",0.00327596023186152,-0.173154236537135,0.026,0.095,1,0
"Tnfrsf1a",0.0032918612795851 5,0.116380068967675,0.219,0.141,1,0
"Tfec",0.00329948807089163,-0.108247516252832,0.006,0.065,1,0
"Gm6710",0.00330223893645601,0.0564893893313429,0.065,0.026,1,0
"Atp4a",0.00330377013254569,0.0844277437295601,0.103,0.05,1,0
"Map2",0.00330455605137413,0.0672330728763028,0.065,0.026,1,0
"Inpp5f",0.00331921669588128,0.132433160309461,0.181,0.108,1,0
"Akap8",0.00332077963643475,0.130465388560227,0.161,0.094,1,0
"Lyrm4",0.00333442791770116,-0.184583246116475,0.032,0.103,1,0
"Tnpo2",0.00333874753531501,0.0832705717096182,0.116,0.06,1,0
"Traf1",0.00334192284678186,0.0932870608920297,0.058,0.023,1,0
"C1qtnf3",0.00334460321524576,-0.133666737732806,0,0.053,1,0
"Rprd1a",0.00335775176515329,0.0657139259009174,0.123,0.064,1,0
"Mia3",0.00336278245221477,0.0930454502722763,0.219,0.138,1,0
"Xrcc4",0.00336527111600989,0.0859855016382851,0.123,0.065,1,0
"Psmd4",0.00336566598837231,0.13857581988294,0.426,0.318,1,0
"Slc6a13",0.00337112358545679,-0.182586989373053,0.026,0.095,1,0
"Wbp2",0.00337219427307732,0.124713120852437,0.4,0.289,1,0
"Nab1",0.00337431988471722,0.102246613172592,0.174,0.104,1,0
"Thoc6",0.00337501747451594,0.0569520352005154,0.103,0.051,1,0
"Fga",0.00339243567478598,-0.241061419311388,0.026,0.094,1,0
"Zdhhc4",0.00340159606360812,0.0670575443559016,0.168,0.097,1,0
"Zfp467",0.0034107057000364,0.103552851316,0.252,0.162,1,0
"Cep104",0.00341776085983772,0.0816198242891576,0.123,0.065,1,0
"Ap1s1",0.00342043931701173,0.119930615715664,0.316,0.221,1,0
"2410004B18Rik",0.003444529250906,0.0679035405693208,0.148,0.083,1,0
```

"Ndufa11",0.00344562019635982,-0.236855780139411,0.735,0.722,1,0
"Rdx",0.00344629581273743,0.138554446728341,0.581,0.43,1,0
"Pofut1",0.00344731460949608,0.0840850250369308,0.084,0.038,1,0
"Dmtf1",0.00345544557433623,0.169093648621007,0.135,0.076,1,0
"Smad5",0.00345917581876185,0.103261372188511,0.168,0.1,1,0
"Ormdl3",0.00346341583444657,0.0624468282677663,0.123,0.065,1,0
"Fggy",0.00346791981543053,-0.265461885582058,0.097,0.182,1,0
"Ubap2",0.00347253599818889,0.179124189712715,0.187,0.115,1,0
"Arhgap42",0.00347796605339162,-0.140044534269004,0.026,0.096,1,0
"Ttyh3",0.00348304569038503,0.0901937175697491,0.123,0.065,1,0
"Acacb",0.0034967577431171,0.0583329896845315,0.077,0.034,1,0
"Bend7",0.00349849965485329,0.0895685626025759,0.123,0.065,1,0
"St3gal2",0.00350426878744009,0.0516558986956543,0.084,0.038,1,0
"Rbm19",0.003509790240055З,0.0529572246088321,0.077,0.034,1,0
"Psmg2",0.00352036579922464,0.0684613263476687,0.161,0.092,1,0
"1110002L01Rik",0.00352151095154637,0.0793063889004564,0.058,0.023,1,0
"Snx11",0.00352488282929774,0.0633362477579461,0.077,0.034,1,0
"Aim1",0.00352493569879128,0.0539500054245921,0.077,0.034,1,0
"Tcp1",0.00352501244685637,0.0627766570096973,0.452,0.318,1,0
"Ppp2r4",0.00352507474405645,0.0880454261622126,0.29,0.195,1,0
"Snx8",0.0035310611546473,-0.197420302659439,0.039,0.111,1,0
"Eif3h",0.00353573820228519,0.17292286677058,0.548,0.449,1,0
"Klhl24",0.00354774208111513,0.117412027845236,0.284,0.194,1,0
"Ski",0.00355856609593041,0.168438989379079,0.2,0.127,1,0
"Fgfr1",0.00356023263385295,0.0665018728286813,0.123,0.064,1,0
"Rasal2",0.00356042031389618,0.0686862761613191,0.09,0.043,1,0
"Psmb6",0.00357031441304419,0.102039851210807,0.716,0.536,1,0
"Cluap1",0.00357158927985392,0.0611499898760094,0.123,0.064,1,0
"Cyld",0.00358339524069663,0.0987336404909012,0.174,0.104,1,0
"Ep300",0.00358458400039344,0.114045549254075,0.181,0.11,1,0
"Zfp346",0.00358816563292049,0.078593686691714,0.052,0.019,1,0
"Tns1",0.00359828376438024,0.258245767373971,0.703,0.579,1,0
"Aak1",0.00360119332635755,0.112638346840302,0.206,0.13,1,0
"Lancl2",0.00360261321680904,0.0667271233780923,0.077,0.034,1,0
"Rptor",0.00360712965057725,0.0846227763949846,0.11,0.056,1,0
"Tagap1",0.00361698571623796,0.116462660816649,0.11,0.056,1,0
"Metap1",0.00362272864952784,0.0798131920556472,0.187,0.113,1,0
"Ier3ip1",0.00365308548406127,0.0728452402266895,0.606,0.451,1,0
"Micall1",0.00366027604739995,0.0625671333943085,0.129,0.069,1,0
"Agap1",0.00366924070360442,0.0897073233165233,0.258,0.169,1,0
"Kank3",0.00367075785479323,0.0815113074802582,0.09,0.043,1,0
"Rpl24",0.00368176761883049,0.13682196549053,0.968,0.897,1,0
"Arf6",0.0036943085056169,0.116442270592954,0.361,0.256,1,0
"Foxi1",0.00370871577080252,-0.223012614395504,0.006,0.064,1,0
"Emc10",0.00371040082309993,0.091213860927334,0.426,0.304,1,0
"Phrf1",0.00371926072250671,0.0861988880347919,0.116,0.061,1,0
"Slc25a21",0.0037200814577871,-0.11770215947053,0,0.052,1,0
"Golph3l",0.00372091467428417,0.0949956160672542,0.116,0.061,1,0
"AI314180",0.00372291699699988,0.171982802675065,0.219,0.143,1,0
"Gm15895",0.00372596045569147,0.07925092795588,0.058,0.023,1,0

"Casc3",0.00375516737567692,0.0672920997086344,0.116,0.061,1,0
"Cdc14a",0.00375520456458339,0.0520622060870848,0.065,0.027,1,0
"Xpo7",0.00375629426312695,0.0867333784401839,0.174,0.103,1,0
"Entpd7",0.00377029412695995,0.0735020474944541,0.09,0.043,1,0
"Fam43a",0.00379761945090535,0.0562223803491307,0.071,0.03,1,0
"Zdhhc21",0.00379930675118365,0.159687282642443,0.161,0.096,1,0
"Zfp160",0.0038016070363771,0.0729415053918281,0.071,0.03,1,0
"Ltn1",0.00382462999953118,0.0940821818481783,0.142,0.08,1,0
"Piga",0.00382693313782066,0.0773465460055804,0.084,0.039,1,0
"Hexim1",0.00383488699942698,0.0918378742952728,0.232,0.148,1,0
"Rora",0.00384038576401104,0.122235651141228,0.2,0.125,1,0
"Coq10b",0.00384422706489282,0.0751101528828932,0.194,0.118,1,0
"Rp9",0.00384766377028817,0.107347507020144,0.329,0.23,1,0
"Gm14698",0.00384910931388497,0.112033821398473,0.135,0.075,1,0
"Cnksr3",0.00385435984514823,0.0738861950795923,0.09,0.043,1,0
"Smim3",0.00386255500075171,0.0838940979795975,0.071,0.031,1,0
"Arsk",0.00386370223976004,0.0900766230251412,0.103,0.052,1,0
"Itgb2",0.00386901902269968,0.0866099888264622,0.077,0.035,1,0
"D630024D03Rik",0.00389060230376174,-0.168870564259184,0.019,0.082,1,0
"Lamtor5",0.00389398878622452,-0.280247401298272,0.477,0.547,1,0
"Mrpl33",0.00389456020203531,0.0949473804239587,0.71,0.549,1,0
"Vta1",0.0039007375151124,0.0976688539951316,0.161,0.094,1,0
"Tcof1",0.00390674945388279,0.10746180005344,0.103,0.053,1,0
"Gtf3c3",0.0039076935208942,0.0790740744918383,0.11,0.056,1,0
"Gsta3",0.00391818210241975,-0.261207439826917,0.123,0.214,1,0
"Ndfip2",0.00391825662304621,0.129135733809297,0.374,0.27,1,0
"Chtf8",0.00395505829144775,0.0512111755181618,0.213,0.131,1,0
"Prosc",0.00395992480185735,-0.219549738098198,0.097,0.183,1,0
"Pld4",0.00396034379189334,0.0816953138755397,0.11,0.056,1,0
"Cep89",0.0040023289531376,0.0895059883387122,0.116,0.061,1,0
"Ttl",0.00401661995308134,0.0537330391589542,0.071,0.031,1,0
"Stmn1",0.00402589588796634,0.150661585033132,0.29,0.197,1,0
"Ubald2",0.00402674211070987,0.120390072049445,0.342,0.242,1,0
"Dcun1d3",0.00403084141995116,0.0813765264674959,0.09,0.043,1,0
"Exd2",0.0040329389273876,0.068308472986127,0.084,0.039,1,0
"Gm10138",0.00403398976606966,0.145154312294727,0.129,0.071,1,0
"Supt5",0.00403490517880759,0.11818166962876,0.232,0.15,1,0
"Cdkal1",0.00403962545712707,0.100101923041406,0.181,0.107,1,0
"Baiap2",0.00405301732020504,0.0840816770037068,0.232,0.148,1,0
"Ppp2r5b",0.00405549132600804,0.113508106844445,0.103,0.052,1,0
"Lcmt1",0.00406054859145213,0.132572245729429,0.2,0.124,1,0
"Usp19",0.00408366989530168,0.0642360111582685,0.2,0.122,1,0
"Cox7a2l",0.00410987456240731,0.134154080551163,0.735,0.555,1,0
"Mapk8",0.00410992480953021,0.120231992886184,0.129,0.071,1,0
"Mcrs1",0.00412644312108256,0.0658644413099134,0.219,0.137,1,0
"Pwwp2a",0.00412678132012435,0.1231631574515,0.155,0.091,1,0
"Crym",0.00412864854095616,-0.165929795014018,0.032,0.102,1,0
"Cndp1",0.00412979847699327,-0.12909622960949,0.006,0.062,1,0
"Vps45",0.00413455227736541,0.0978264043180005,0.103,0.052,1,0
"Gm17354",0.00414297828799692,0.0738409596170287,0.058,0.023,1,0

"Bms1",0.00414379055092955,0.105714554792404,0.116,0.061,1,0
"Trim11",0.00414457521047885,0.0635318726991784,0.097,0.047,1,0
"Gm26853",0.00414828750372158,0.0946727114713257,0.077,0.035,1,0
"Hs2st1",0.00415478845586738,0.0723629430162472,0.116,0.061,1,0
"Spag9",0.0041704064633912,0.0817530347934329,0.355,0.249,1,0
"Mpzl3",0.00418945628317317,0.063968573110048,0.058,0.023,1,0
"Smarcad1",0.0042139525907605,0.104080189431147,0.168,0.101,1,0
"Prdm16",0.0042241600102402,0.0616731897074735,0.097,0.047,1,0
"Spryd3",0.0042330687063202,0.0841832153263149,0.097,0.048,1,0
"Ndufb7",0.00423824826689773,-0.214644133503846,0.781,0.762,1,0
"Oaz1",0.00424189820644066,-0.143526806603883,0.897,0.868,1,0
"Mtap",0.00424672765820726,0.0638787068775853,0.135,0.074,1,0
"Rbpj",0.00425084495427648,0.112993987443486,0.148,0.086,1,0
"Pclo",0.00426638752809055,0.0919806643993267,0.116,0.061,1,0
"Anapc4",0.00427521966553569,0.0803885893198854,0.135,0.075,1,0
"Sys1",0.00427972791341251,0.0543822230640709,0.368,0.253,1,0
"Borcs8",0.00429925845942006,0.0715909312420966,0.252,0.163,1,0
"Rheb",0.00430437241079461,0.109564016768131,0.516,0.375,1,0
"Edrf1",0.00430498168433006,0.103939978372771,0.071,0.031,1,0
"Ndufs1",0.00430828282869464,-0.232826249173331,0.232,0.321,1,0
"Ccm2",0.00431673285433093,0.0648886010862367,0.142,0.079,1,0
"Snd1",0.00432173499352314,0.101187567565999,0.168,0.1,1,0
"Taf10",0.00432682410068677,0.085077634877084,0.439,0.314,1,0
"Mia2",0.00433133558371612,0.100341954377049,0.09,0.043,1,0
"Snapc3",0.00433205751676938,0.127586564734659,0.129,0.072,1,0
"Txnl4a",0.00433277572447404,0.0533905007307168,0.29,0.193,1,0
"Cdc123",0.0043377638764769,0.0537358468753142,0.277,0.184,1,0
"Ap1m1",0.0043708271268307,0.0803702924607649,0.148,0.084,1,0
"Atg2b",0.00437279929332418,0.102378581746225,0.103,0.052,1,0
"Mdfic",0.00437922000914618,0.0950776876109147,0.103,0.053,1,0
"Arcn1",0.00438331169258627,0.102128723655585,0.297,0.206,1,0
"Abhd2",0.00438498731944339,0.092358494313533,0.245,0.158,1,0
"Lyrm1",0.00439102948102518,0.0653140665046705,0.084,0.039,1,0
"Braf",0.00439443731538048,0.130965225951691,0.148,0.086,1,0
"Rpp25l",0.00439447239747866,0.0764593890383362,0.206,0.13,1,0
"Ehd3",0.00440325032514353,0.223402825123507,0.084,0.04,1,0
"Dctn1",0.00440378720488777,0.101037484031186,0.219,0.141,1,0
"Zfand1",0.00441042847735512,0.0786309184480972,0.194,0.119,1,0
"Stard3",0.00441179749374483,0.101008915017481,0.129,0.071,1,0
"Zfp68",0.00441355497569028,0.0624093932058463,0.116,0.061,1,0
"BC030336",0.00442542113457885,0.0733847943734581,0.135,0.075,1,0
"Pofut2",0.00443638069911495,0.0793764453360502,0.194,0.119,1,0
"Ap2m1",0.00444722499916207,0.10084382286986,0.594,0.444,1,0
"Mtpn",0.00445400684803104,0.094818766323479,0.368,0.262,1,0
"Cmc1",0.00447033151196346,0.0903617369317146,0.265,0.177,1,0
"Prpsap1",0.00447459451618612,0.0935693746418856,0.206,0.131,1,0
"Sar1a",0.00448517723123047,0.112512695526046,0.329,0.233,1,0
"Polr3e",0.00449694235917478,-0.284416086397617,0.097,0.18,1,0
"Psmd12",0.00451336383876273,0.0880935950945275,0.271,0.183,1,0
"Ttyh2",0.0045147841119582,-0.176880384574677,0.039,0.11,1,0

"Zzef1",0.00451666327847475,0.0946906156329065,0.168,0.1,1,0
"Nr1h2",0.00452192556704794,0.103892677164123,0.219,0.141,1,0
"Cwf19l2",0.00452438344121252,0.0948894598883513,0.135,0.076,1,0
"Helz",0.00454359668199644,0.114187630936882,0.161,0.096,1,0
"Pcmtd2",0.00454861607071341,0.10706113786941,0.206,0.132,1,0
"Dnajb2",0.00455180432063091,0.110425237578938,0.161,0.096,1,0
"Tubgcp3",0.00455416631487154,0.0983544458081672,0.097,0.048,1,0
"Ubtd1",0.00455616821427002,0.0668085009323784,0.084,0.039,1,0
"Cpne3",0.00456310567332084,0.121340734693597,0.29,0.198,1,0
"Dynlt1f",0.00456848196531546,0.0863733073757786,0.168,0.101,1,0
"Evi5",0.0046265447418476,-0.267045198251033,0.181,0.275,1,0
"Pik3r2",0.00463118076558152,0.0689527841826014,0.103,0.052,1,0
"Hras",0.00466052819693941,0.07058429525376,0.368,0.258,1,0
"Glis2",0.00467065117162156,0.151281603318125,0.245,0.166,1,0
"Phf10",0.0046797267836167,0.0737369446346944,0.148,0.085,1,0
"Adk",0.00468286071941174,-0.300841151045355,0.206,0.292,1,0
"Ccdc189",0.00471931982947771,0.105535761716349,0.077,0.035,1,0
"Mbd1",0.00472325080298146,0.0624705273322353,0.103,0.052,1,0
"Pja2",0.0047277956219457,0.122238872074895,0.219,0.142,1,0
"Sft2d1",0.00472901793140285,0.0926280722071617,0.239,0.156,1,0
"Tsc22d3",0.00473051535367997,0.0857447189080321,0.161,0.095,1,0
"Rnf215",0.0047512662858391,0.0932679460803292,0.084,0.04,1,0
"Scrn2",0.00476010689920162,-0.266999929235574,0.148,0.235,1,0
"Gm10053",0.00476223487771684,-0.28608956744376,0.187,0.276,1,0
"Fn3k",0.00476866404964536,-0.123076727284727,0.013,0.072,1,0
"Rap1gap",0.00476917083747995,0.110123153617719,0.181,0.11,1,0
"Ift22",0.00478144651744286,0.0526040917245666,0.206,0.128,1,0
"Fads1",0.00479754929356732,-0.184804689336001,0.052,0.125,1,0
"Blvrb",0.00480478998931214,0.122667878563396,0.187,0.114,1,0
"Sf3b3",0.00482043412495669,0.0893339804458933,0.135,0.076,1,0
"Thnsl1",0.00482066237881829,0.0634679332208344,0.065,0.027,1,0
"Fance",0.00482156757273036,0.073863621775785,0.09,0.044,1,0
"Vps4a",0.00482324157967252,0.121380310249124,0.168,0.102,1,0
"Rprd1b",0.00482397011659638,0.0701010066132711,0.148,0.084,1,0
"Mcm2",0.00484165465297493,0.0787570241105216,0.077,0.036,1,0
"Plcg1",0.00485425242458342,0.0536085088814093,0.09,0.043,1,0
"Slc16a13",0.00485544514605983,-0.129033883435877,0.019,0.081,1,0
"Ric1",0.00485707421066377,0.0830905722077389,0.116,0.061,1,0
"Fam98b",0.00486761638500748,0.10830012028067,0.155,0.091,1,0
"Sec23ip",0.00486931024167702,0.068453022309676,0.129,0.071,1,0
"Arhgap15",0.00487359836288529,-0.128133517846532,0.006,0.06,1,0
"Kctd3",0.00487493048500688,0.0757455037505436,0.097,0.048,1,0
"Taok2",0.00487875496087043,0.129691177369212,0.135,0.077,1,0
"Wdr6",0.00488223034715269,0.114051360565406,0.11,0.058,1,0
"Hemk1",0.00489003048942292,0.0639142302091297,0.084,0.039,1,0
"Map2k1",0.00489008980796952,0.0816729187634962,0.174,0.105,1,0
"Nuak1",0.00490281444442222,0.0723863843518651,0.052,0.02,1,0
"Fn1",0.00490511991805602,0.0851827039417192,0.077,0.036,1,0
"Tmem260",0.00491813768771586,0.097071578115329,0.116,0.062,1,0
"Lcorl",0.00492507099185188,0.0627881281048187,0.135,0.075,1,0

"Lpin1",0.00492773712790041,-0.134531974057549,0.032,0.101,1,0
"Birc6",0.00494839083315814,0.122568817339075,0.4,0.291,1,0
"Ifi204",0.00496353857030159,0.0648108112329573,0.052,0.02,1,0
"Shmt2",0.00497344565179533,-0.264591187780462,0.103,0.185,1,0
"Apip",0.00498520033560944,0.105305224372623,0.174,0.106,1,0
"Sdhaf2",0.00499504951694729,0.081179405654655,0.155,0.091,1,0
"Usp10",0.00499749650194247,0.103531363705904,0.2,0.126,1,0
"Paip1",0.00500737038039113,0.0958257893099537,0.239,0.156,1,0
"Slc20a1",0.00500979340345734,0.0927335522771906,0.123,0.066,1,0
"Pdzd2",0.00501231630375513,-0.12213367677 2475,0.013,0.071,1,0
"Txndc17",0.00501509127668964,-0.261011613687353,0.606,0.619,1,0
"Rbms2",0.00502487965246205,0.0919219804587394,0.161,0.096,1,0
"Dio1",0.00503367025857478,-0.111534241101088,0.019,0.082,1,0
"Mob2",0.00504840083537811,0.0675147454933334,0.129,0.071,1,0
"Zfp609",0.00505111547801218,0.0896818954143746,0.071,0.032,1,0
"Evc2",0.00505455726321655,0.0922619959382825,0.116,0.062,1,0
"Bex1",0.00506255458340186,0.110345417445313,0.071,0.031,1,0
"Creg1",0.00506988792595486,-0.278882521391029,0.194,0.28,1,0
"Pcdh1",0.00507176367666803,0.159702825654125,0.148,0.087,1,0
"Gfpt1",0.0050892962556411,0.113562800469469,0.187,0.115,1,0
"Sp3os",0.0051107341814506,0.0844187159210005,0.155,0.091,1,0
"D3Ertd751e",0.005112142498976,0.114143137117804,0.142,0.081,1,0
"Mettl10",0.00511932779124542,0.0697251971899958,0.142,0.081,1,0
"Ino80d",0.00512212346859693,0.130387222127012,0.2,0.128,1,0
"Metap1d",0.00512992395230139,0.0649928113617373,0.168,0.099,1,0
"Rpusd3",0.00513694359425875,-0.144561809318347,0.013,0.07,1,0
"Palm",0.00513883708145554,-0.167354725462554,0.045,0.117,1,0
"Slc31a1",0.00514356084405142,-0.298447923987331,0.252,0.332,1,0
"Gabpb1",0.00514690511865951,0.0676259948306616,0.09,0.044,1,0
"D5Ertd579e",0.00514711888585056,0.11360518917863,0.226,0.148,1,0
"Ptpn21",0.00515206542734989,0.0710506365026154,0.09,0.044,1,0
"Cdc42ep1",0.00515687129198397,0.0725462050576944,0.084,0.04,1,0
"Phykpl",0.00515765625413827,0.0810088317561093,0.103,0.053,1,0
"Ddx46",0.00515766970207206,0.110086722966011,0.239,0.157,1,0
"Rpl23a-ps3",0.00516480385523686,0.128327033746028,0.703,0.58,1,0
"Tcf3",0.00517493687442046,0.0651389002457344,0.129,0.071,1,0
"Prps2",0.0051964716654246,0.106101362543641,0.2,0.126,1,0
"Ccdc61",0.0052120831430729,0.08100921074918,0.09,0.044,1,0
"Pip4k2c",0.00522242824466596,0.134858226865898,0.297,0.208,1,0
"Txndc12",0.00524902293798648,0.103425580980207,0.148,0.086,1,0
"Sigmar1",0.00525582786986805,0.0899639407938222,0.135,0.076,1,0
"Ank",0.00527148361361369,0.0759413355207495,0.052,0.02,1,0
"Sipa1l3",0.00527660000522407,0.0953133279783702,0.135,0.077,1,0
"Ppan",0.00527778176423348,0.0749227091057475,0.103,0.053,1,0
"Napa",0.0052933146882216,0.0997741520321877,0.316,0.223,1,0
"Smad4",0.00531479270680478,0.10412463695378,0.181,0.113,1,0
"Sp100",0.00533487249759256,0.0845933016797135,0.071,0.032,1,0
"Rit1",0.0053352650505488,0.121183344643794,0.148,0.087,1,0
"Copa",0.00533775013413731,0.0979028444810851,0.413,0.307,1,0
"Zfp276",0.005340362663759,0.0769674762144967,0.058,0.024,1,0

"Trmt13",0.00536458001491511,0.127777238037657,0.097,0.049,1,0
"Gins2",0.0053673738687943,0.0656088653637247,0.052,0.02,1,0
"Stradb",0.00537775272616182,0.0762134330232113,0.135,0.076,1,0
"Ap1g2",0.00538671962367792,0.0674845430321931,0.077,0.036,1,0
"Nav1",0.00539186495919927,0.10977663840779,0.097,0.049,1,0
"Stk3",0.00540009052950929,0.0692076416191761,0.09,0.044,1,0
"Gcc1",0.00541113999377309,0.0538910844947509,0.071,0.031,1,0
"Mapk1ip1",0.00542332940831617,0.0815368456053633,0.09,0.045,1,0
"Plaa",0.00543192509391292,0.0735710946393824,0.239,0.155,1,0
"Nudt5",0.00544432313915212,0.10657128090538,0.239,0.158,1,0
"A830080D01Rik",0.00544518778579571,0.0919194250129101,0.077,0.036,1,0
"Irak1",0.00545504093547915,0.109142435428915,0.232,0.155,1,0
"Noc2l",0.00546115399314498,0.0702468194982423,0.129,0.071,1,0
"Pnpt1",0.00546919441212148,0.0748237921644158,0.11,0.058,1,0
"Gpt2",0.00548126277729609,-0.148841389155784,0.032,0.1,1,0
"Hmces",0.00548128480735471,0.0578254965552772,0.123,0.066,1,0
"Lrrc42",0.00548686617767674,0.090006692795418,0.103,0.054,1,0
"Brd1",0.00549739618203269,0.106738214921206,0.129,0.072,1,0
"Ttf1",0.00552157553344085,0.0929218429765237,0.123,0.067,1,0
"Trim39",0.00552625596920145,0.0805769457855164,0.084,0.04,1,0
"E130308A19Rik",0.00553624087561517,0.0681544829889351,0.103,0.053,1,0
"Napb",0.00555331209974214,0.0603385361633505,0.058,0.024,1,0
"Elovl6",0.00556087582797765,0.088536793929048,0.161,0.095,1,0
"Repin1",0.00556214777397148,0.0824909578676545,0.129,0.071,1,0
"Meis2",0.00556557788226593,0.188741098268662,0.071,0.032,1,0
"Polb",0.00557456760478653,0.0871734831799052,0.155,0.092,1,0
"Lpin2",0.00558494901039868,0.115638471733493,0.181,0.111,1,0
"Abhd14a",0.00558858309315395,-0.269330939792823,0.103,0.182,1,0
"Larp1b",0.00559012022973047,0.0694942457358259,0.271,0.184,1,0
"Rnf103",0.00559945853440062,0.0683495090235695,0.174,0.105,1,0
"Klk1",0.0056252750076671,0.458056736844583,0.458,0.352,1,0
"Smad3",0.00563326367200863,0.0825195223311558,0.103,0.053,1,0
"Cd48",0.00564495895106155,0.0717999079500017,0.071,0.032,1,0
"Keap1",0.00564894352707779,0.0792638685513637,0.174,0.106,1,0
"Grb7",0.00565280689472665,0.0755954231806436,0.219,0.138,1,0
"Gm16286",0.0056529961208296,0.0904850190174389,0.232,0.153,1,0
"Kin",0.00565938835810133,0.0699024374423721,0.116,0.062,1,0
"Pcbp2",0.0056619513501653,0.14929701622819,0.723,0.556,1,0
"Mus81",0.00567255962048186,0.0554437396554592,0.077,0.036,1,0
"Stk32b",0.00568719728040825,0.088304928642092,0.065,0.028,1,0
"Tbce",0.00568789979717713,0.124182791819824,0.135,0.078,1,0
"Lyar",0.00569308133572609,0.106777968217651,0.174,0.108,1,0
"Gm3716",0.00569820391052431,-0.130493418123574,0.006,0.059,1,0
"Adap2",0.00572041753075436,-0.121409434675244,0.013,0.07,1,0
"Urm1",0.00574055939781202,0.0683647750364824,0.161,0.095,1,0
"Ythdf2",0.00574847724123869,0.0768872674237371,0.213,0.137,1,0
"Ttll12",0.00575914224078419,0.054309956859938,0.077,0.036,1,0
"Stat5a",0.00576241567280077,0.0852673559544815,0.084,0.04,1,0
"Eif4g3",0.00577599080109873,0.1086233997943,0.329,0.236,1,0
"Ngrn",0.00582029616499678,0.102488015046864,0.161,0.097,1,0

"Slc36a1",0.00582073991090316,-0.176834534332025,0.045,0.116,1,0
"Cobll1",0.00584942818925545,0.186336669676409,0.245,0.168,1,0
"Acadsb",0.00585370225085445,0.106874683715779,0.277,0.19,1,0
"Larp4b",0.00585509379259439,0.0992993808993183,0.368,0.266,1,0
"Gm11837",0.00586369602209613,-0.135321533689891,0.006,0.058,1,0
"Kars",0.00589922632082308,0.0582903207664378,0.194,0.12,1,0
"Klhl7",0.00590327336652069,0.0834552031699041,0.123,0.067,1,0
"Wdr4",0.00590376724299799,0.070596806790054,0.077,0.036,1,0
"Rab5c",0.00594782698827661,-0.298635391721339,0.419,0.474,1,0
"Mbd3",0.00597751455447638,0.069306183634724,0.348,0.247,1,0
"Ppp2r3d",0.00599986195341548,0.0783532499225225,0.097,0.049,1,0
"Pygo2",0.00600159628258241,0.0540895903201926,0.077,0.036,1,0
"Otud7b",0.00600211848192155,0.103768720783333,0.168,0.102,1,0
"Ecm1",0.00600307045910409,0.0903736257926374,0.058,0.024,1,0
"Wdr31",0.00603832969263069,0.0599080713682031,0.077,0.036,1,0
"Col3a1",0.0060386998594709,0.0563373587483905,0.077,0.036,1,0
"Btbd2",0.00604068094334703,0.0909484154439651,0.155,0.092,1,0
"Cpsf7",0.00604288771094744,0.103558548037254,0.142,0.082,1,0
"Fam174a",0.00604346247275764,0.0992386860448818,0.284,0.199,1,0
"Anapc5",0.00604607290001924,0.0880492765806776,0.406,0.298,1,0
"Idnk",0.00604960264846947,0.0502798138529473,0.29,0.195,1,0
"Nim1k",0.00605044470951954,0.0552496330985425,0.052,0.02,1,0
"Mpp5",0.00606029301479966,0.142524695087857,0.226,0.15,1,0
"Ring1",0.00607345654688454,0.0954603259600623,0.181,0.111,1,0
"Rabl2",0.00608998829361123,0.0846956333789804,0.103,0.054,1,0
"Pcyt2",0.0060945840058263,-0.309143518678839,0.174,0.258,1,0
"Ndufs7",0.00612268475871957,-0.288562256485492,0.671,0.642,1,0
"Sf3b5",0.00612823658666403,0.105283708252077,0.439,0.327,1,0
"Atg16l1",0.00614840583070454,0.104110452852585,0.097,0.049,1,0
"Tmem248",0.0061514858297 4074,0.11152760973793,0.174,0.108,1,0
"Fam206a",0.00615691678299781,0.0707592533397203,0.065,0.028,1,0
"Cnot7",0.00616296094175035,0.0719290366279783,0.226,0.146,1,0
"Snrnp27",0.00617138050543107,0.069464412399234,0.49,0.354,1,0
"Ipo7",0.00618776272272734,0.0741857500072767,0.226,0.146,1,0
"Zfp738",0.00620946960763889,0.0592849855857403,0.065,0.028,1,0
"Tex2",0.00621973707222063,-0.153543593052043,0.039,0.106,1,0
"Tmem30a",0.00622529991333341,0.0987045960842686,0.458,0.339,1,0
"Fahd2a",0.00623336296096689,-0.275846478717446,0.168,0.254,1,0
"Hipk1",0.00626688239493714,0.0760825456662415,0.265,0.177,1,0
"Snx16",0.00627987442027839,0.0605011773575355,0.116,0.062,1,0
"Gm10250",0.00629887245605559,-0.198576741302503,0.071,0.146,1,0
"Pabpc4",0.00630286117224905,0.0830237519642207,0.194,0.123,1,0
"Mrpl9",0.00631809526727109,0.0574389611162798,0.194,0.12,1,0
"Evi2a",0.00632569892174724,0.0516252607859459,0.058,0.024,1,0
"Zcchc17",0.00632618713649661,0.0813136970737775,0.252,0.167,1,0
"Trub2",0.00632852549316038,0.0547515402862279,0.09,0.045,1,0
"Arhgef16",0.00633256778725153,0.0978150058042684,0.181,0.112,1,0
"Cdk5rap2",0.00635770676796008,0.111436958114965,0.071,0.032,1,0
"Vps29",0.00635789266732142,-0.276424374522885,0.406,0.473,1,0
"Ddb1",0.00636931664232808,0.09903116584701,0.374,0.276,1,0

"Rpl32",0.00637733933416813,0.12768198038027,0.974,0.927,1,0
"Rbm18",0.00639688938967828,0.131010208427509,0.135,0.079,1,0
"Fam219a",0.00641288491210527,0.0622531586852126,0.052,0.02,1,0
"Mtmr3",0.00641294529397631,0.121436429086872,0.155,0.093,1,0
"Wars",0.00641834727712605,0.0778093199122509,0.123,0.067,1,0
"Plpp1",0.00647178228445831,0.156264576750797,0.116,0.063,1,0
"Tmem25",0.00648298885707242,-0.106321704404816,0.006,0.057,1,0
"Kat5",0.00651232702214052,0.0601982995480398,0.103,0.054,1,0
"Fbxo36",0.00651558975017526,-0.189192657808796,0.052,0.122,1,0
"Fmn1",0.00651842135264056,0.11513112637747,0.142,0.083,1,0
"Oat",0.00653926326776006,0.117577574761277,0.568,0.424,1,0
"Prpf6",0.00656324211187419,0.0827830747696141,0.148,0.087,1,0
"Lman2",0.00657281896921941,0.0563080116932593,0.394,0.281,1,0
"Peg3",0.00657981743059159,0.0881916581211628,0.065,0.028,1,0
"Esco1",0.00659651692639949,0.0807835674564945,0.129,0.073,1,0
"Puf60",0.00661447870709299,0.10685591714955,0.265,0.182,1,0
"Stx17",0.00661885709831482,0.0726430397417219,0.116,0.063,1,0
"Pum3",0.00662831584425668,0.100620470649779,0.155,0.092,1,0
"Mllt3",0.00663212955691174,0.0753077579023613,0.097,0.049,1,0
"Gm10020",0.00663535153322206,0.0658126247887966,0.161,0.096,1,0
"Yeats4",0.0066426251592982,0.11650487626786,0.2,0.13,1,0
"Cnot10",0.00664279252296709,0.101620981733828,0.11,0.059,1,0
"0610010K14Rik",0.00666761074379105,0.0604806372897437,0.187,0.115,1,0
"Cyth3",0.00666932142777513,0.130034558821259,0.103,0.055,1,0
"Hat1",0.00670224818802071,0.099426232718169,0.142,0.083,1,0
"Lysmd4",0.00671794273666652,0.0614509798988227,0.065,0.028,1,0
"Rassf7",0.0067364297742243,0.118727131877255,0.129,0.074,1,0
"Elmo3",0.00676839273776689,0.0638499056602404,0.097,0.049,1,0
"Tuba4a",0.00677145326129691,-0.267950777073087,0.226,0.306,1,0
"Prkcsh",0.00677980982007421,0.112846204361625,0.323,0.232,1,0
"Ezh2",0.00681546998788951,0.0849311741973165,0.097,0.049,1,0
"AK010878",0.00683227934888242,0.0872491878498176,0.097,0.049,1,0
"Nek1",0.00688407777446089,0.0871531519180029,0.084,0.041,1,0
"Uggt1",0.00689073590367953,0.0922358976833327,0.155,0.093,1,0
"Galc",0.00689422204173016,-0.186733580304926,0.065,0.138,1,0
"Rab9",0.0069483309859029,0.0635864437124446,0.258,0.172,1,0
"Rbmx",0.00698529820148301,0.123089368576494,0.187,0.12,1,0
"Ssu72",0.00699007484382096,0.0831579152936159,0.381,0.275,1,0
"Wdr73",0.00699653660036893,0.0556985923806127,0.103,0.054,1,0
"Zfp617",0.00700954536096307,0.0642358460171667,0.097,0.049,1,0
"Gm20721",0.00702357755044782,0.0613723796039953,0.052,0.02,1,0
"Stra13",0.0070320892160754,-0.322907527256628,0.426,0.465,1,0
"Snhg9",0.00703514403205588,0.0508428893116673,0.161,0.095,1,0
"Vti1b",0.00708182362390567,0.0860270918882151,0.419,0.302,1,0
"Paip2b",0.00709679083036981,0.0732436439353313,0.103,0.054,1,0
"Slf1",0.00711273730071897,0.0970422074788258,0.116,0.064,1,0
"Ap1g1",0.00711819120109321,0.108798784205549,0.252,0.172,1,0
"Dcbld2",0.00713060121873163,0.0789310872886264,0.065,0.028,1,0
"Nlk",0.00716473505201307,0.124072447033131,0.116,0.064,1,0
"Htatsf1",0.00716744691216442,0.0814233449505039,0.194,0.122,1,0

"Acp2",0.00716791903883607,0.0879728942995447,0.129,0.074,1,0
"Fam216a",0.00720606268848645,0.0854525065553638,0.123,0.068,1,0
"Eef2kmt",0.00721544565502649,0.0889083083817437,0.161,0.098,1,0
"Lmf2",0.00722194186481474,0.0854313025756656,0.129,0.072,1,0
"Tpd52l1",0.00722901005286948,0.132939467930987,0.135,0.078,1,0
"Gabarapl2",0.00723285783711313,0.0891660966702219,0.632,0.493,1,0
"Kif16b",0.00724802672244192,0.108387953405143,0.161,0.098,1,0
"Katna1",0.00727747245821725,0.0774539932274144,0.148,0.088,1,0
"Thumpd3",0.00728361435728301,0.126876282877815,0.155,0.093,1,0
"Wdr45",0.00729225247095588,0.0846644507039194,0.161,0.097,1,0
"Pigh",0.00730190654304713,0.0821919127574589,0.09,0.045,1,0
"Snx21",0.0073169460645484,0.0714752050217437,0.123,0.068,1,0
"Prkce",0.00731784897403606,0.0953829886697154,0.11,0.059,1,0
"Gatb",0.00732680641699377,-0.152973247554066,0.026,0.085,1,0
"Alad",0.00733256426458993,-0.261083701441809,0.168,0.251,1,0
"Samd4",0.00733667079105546,0.060750566790932,0.052,0.021,1,0
"Pithd1",0.00734748972084053,0.0918781646261187,0.11,0.059,1,0
"Abhd6",0.00734879790121093,0.0705310733286465,0.11,0.059,1,0
"Gtf3c2",0.00736348144032211,0.0926216665231941,0.2,0.128,1,0
"Nf2",0.00737655770690281,0.0766336861695256,0.142,0.082,1,0
"Slc27a4",0.00739389638503353,0.0606813496286467,0.142,0.082,1,0
"2310039H08Rik",0.00739499770634593,-0.291288199312318,0.226,0.308,1,0
"Naa25",0.00740224585744937,0.103717416874147,0.097,0.05,1,0
"Haus1",0.00740579208830072,0.0723762814254297,0.065,0.028,1,0
"Ppa2",0.00740659427887923,-0.311520846811911,0.355,0.416,1,0
"Scamp2",0.00741593701614405,0.0835392245265884,0.284,0.196,1,0
"Gimap6",0.00743222128440088,0.105538490807964,0.097,0.05,1,0
"Armcx1",0.00744348835068346,0.0869780367423573,0.09,0.046,1,0
"Sfi1",0.00744697804597456,0.0815812612710825,0.084,0.041,1,0
"Clcn5",0.00745568039875631,-0.271909391267782,0.129,0.212,1,0
"Mrpl13",0.00746985867661817,0.0787729383523754,0.323,0.226,1,0
"Supt16",0.00748849043961623,0.105698741973063,0.219,0.146,1,0
"Rps28",0.00749466513734029,0.186475021472567,0.961,0.932,1,0
"Itfg2",0.00752405914366045,0.0624095321486515,0.11,0.059,1,0
"Plcl1",0.00753947533465312,0.0735731433185726,0.116,0.063,1,0
"Tsnax",0.00755558859488127,0.120595816660303,0.168,0.105,1,0
"Asb7",0.00756171392325019,0.0688919846302802,0.103,0.054,1,0
"Mroh1",0.00757215936987268,0.064879352453174,0.174,0.106,1,0
"Vimp",0.00758169954650063,0.0695008536688128,0.439,0.323,1,0
"Gtf2b",0.00759205352210799,0.107904962621265,0.181,0.116,1,0
"Tmem60",0.00759424909630138,0.0694819998665614,0.219,0.143,1,0
"Mrpl4",0.00760836214180877,-0.227994699851396,0.2,0.291,1,0
"Nfya",0.00762514025967158,0.0770173909807537,0.071,0.033,1,0
"Pias1",0.00764724762522535,0.0971949040686077,0.123,0.069,1,0
"Ubr5",0.00766975344297323,0.0735471322917984,0.284,0.194,1,0
"Ncbp2",0.00769553959841962,0.0943883624901029,0.245,0.164,1,0
"Zbtb1",0.00770834208863517,0.0706495857625129,0.116,0.064,1,0
"Cep57l1",0.00771140859252016,0.0664688456300224,0.071,0.033,1,0
"2700081O15Rik",0.00773745023342079,0.0617958932101796,0.052,0.021,1,0
"Gxylt1",0.00776322850733623,0.0865211019439261,0.103,0.055,1,0

```
"Lrfn3",0.00776722891297281,0.0794571767659775,0.058,0.025,1,0
"Uxt",0.00777915492859384,0.0775628397374971,0.239,0.16,1,0
"Prr5",0.0077813028517232,-0.157509793866111,0.032,0.095,1,0
"Zpr1",0.00779725525434488,0.0615787906904268,0.148,0.088,1,0
"Dpy30",0.00780231593372018,0.0823514673463527,0.419,0.311,1,0
"Swi5",0.00782089972331806,0.104695933617507,0.755,0.616,1,0
"Fbxo33",0.00784017491493861,0.0914707785091376,0.116,0.064,1,0
"Metrnl",0.00784644741108352,0.100818677507348,0.161,0.098,1,0
"C230035I16Rik",0.00785068551609469,0.0641572154426044,0.052,0.021,1,0
"Opa1",0.00786578027371291,0.0598335713373989,0.226,0.147,1,0
"Htatip2",0.00787335823851516,-0.114960061268383,0.013,0.066,1,0
"Glt8d1",0.00787764731271987,0.0819665234308818,0.135,0.078,1,0
"Sirt7",0.00788520811330754,0.0523078868608012,0.2,0.127,1,0
"Cog2",0.00788612171044656,0.061254416655609,0.116,0.064,1,0
"Otub1",0.00788693739543581,0.124831570159509,0.284,0.199,1,0
"Lrrc45",0.00788707851967499,0.0585257732220299,0.097,0.05,1,0
"Megf8",0.0078916597975 2059,0.0583434865708945,0.09,0.046,1,0
"Cdh5",0.0079046455869565,0.109362856162813,0.052,0.021,1,0
"Setd1b",0.00790702959775932,0.11197721925909,0.142,0.085,1,0
"Sel1l",0.00792522194036272,0.102126286109164,0.252,0.173,1,0
"Arl4a",0.007929259948203,0.082452907090984,0.226,0.149,1,0
"Eps8",0.00795834088795233,-0.148160454376754,0.129,0.217,1,0
"Tmem82",0.00796238886301444,-0.109902438152802,0.006,0.055,1,0
"Cdc42bpb",0.00798141169426599,0.112358631967086,0.174,0.11,1,0
"Fndc3a",0.00799405450935171,0.132926186573219,0.226,0.154,1,0
"Lias",0.00800020307939035,0.0930578441650302,0.168,0.103,1,0
"Tbc1d15",0.00801901286522456,0.118337536686084,0.148,0.088,1,0
"Kdelr1",0.00803551120234492,0.0651791040195574,0.387,0.28,1,0
"Nsun2",0.0080404362671137,0.0783399016959032,0.213,0.138,1,0
"Fam58b",0.00804318098993882,0.1004585287935,0.135,0.08,1,0
"Usp7",0.00805936164983282,0.100512092784336,0.213,0.14,1,0
"Rfk",0.008060947573893,0.0833967722364019,0.355,0.257,1,0
"2700062C07Rik",0.00806376503252494,0.0613650038822305,0.11,0.059,1,0
"Cct5",0.00808855994268124,0.0778388363625975,0.413,0.3,1,0
"2510002D24Rik",0.00810243144619016,0.148160945421817,0.245,0.169,1,0
"Nsmce4a",0.00811575609259833,0.0689904853232147,0.277,0.191,1,0
"Slc29a3",0.0081391825081782,-0.181979716785551,0.065,0.137,1,0
"Rnf114",0.00814391496392476,0.10621458906407,0.174,0.11,1,0
"Bin2",0.0081878861369499,0.0646175835932154,0.052,0.021,1,0
"Col4a3bp",0.00818917417134873,0.0985706117826045,0.174,0.108,1,0
"Ccdc191",0.00819563738352025,0.0923640910466604,0.103,0.055,1,0
"Fbxo45",0.00820396690004698,0.0735650595529557,0.058,0.025,1,0
"Sumo3",0.00820937032074395,0.122562446906455,0.355,0.26,1,0
"Zbtb22",0.00821450738204297,0.0725548331331376,0.09,0.046,1,0
"Vps9d1",0.00822847419485125,0.0641865117992395,0.11,0.059,1,0
"Cckar",0.00826120983119458,-0.137264282226196,0.013,0.065,1,0
"Prkag1",0.00830019093983018,0.0521477935681665,0.2,0.128,1,0
"Tmem29",0.0083028801424253,0.114896167959342,0.161,0.099,1,0
"Tmem98",0.00834525683497112,0.0972158658130131,0.09,0.047,1,0
"Smim11",0.00836488814204022,0.0852258233368378,0.348,0.248,1,0
```

```
"Rpn1",0.00837200756654166,0.062893860992125,0.529,0.407,1,0
"Lekr1",0.00838475524352484,0.06005468620801,0.09,0.046,1,0
"Osbpl8",0.0083905561561059,-0.192224646056217,0.084,0.16,1,0
"Isyna1",0.00841843051266042,0.092716313075524,0.161,0.099,1,0
"Cep164",0.00841858071820132,0.0799140336457951,0.142,0.084,1,0
"Axin1",0.00843249601197997,0.0876811666283679,0.103,0.055,1,0
"Gm44386",0.00844055758594039,-0.249839266034394,0.142,0.224,1,0
"5031425E22Rik",0.00846779467697772,0.0842892511965351,0.194,0.124,1,0
"Pot1a",0.00849066524582729,0.0791085459360502,0.071,0.033,1,0
"Sppl3",0.0085128125031809,0.0766348230166663,0.161,0.098,1,0
"Cd99l2",0.00852317439035184,0.0855011623357208,0.155,0.094,1,0
"Zfyve26",0.00854223821007391,0.0897769697519599,0.058,0.025,1,0
"Vcp",0.0085742382764661,0.0823640948438588,0.6,0.451,1,0
"Abcb10",0.00858006114479132,0.0750415131824337,0.116,0.064,1,0
"Rdh13",0.00859176717449017,0.110655508409735,0.116,0.066,1,0
"Kctd13",0.0085973193111986,0.0781499148811889,0.09,0.046,1,0
"Eng",0.00861020631653786,0.187783715087258,0.084,0.042,1,0
"Slc22a5",0.00861623953483391,-0.18867516040971,0.058,0.126,1,0
"Cybb",0.00861765496422454,0.118426885503084,0.071,0.033,1,0
"Dgcr2",0.00863049357738874,0.07900527762534,0.174,0.108,1,0
"Agpat4",0.00863986618082899,0.0650065804651635,0.077,0.037,1,0
"Cml2",0.00866120872813433,-0.131241891426711,0.013,0.065,1,0
"Mex3c",0.00866716039551316,0.0652500593294225,0.09,0.046,1,0
"Arhgap1",0.00867131210885487,0.0771559113164277,0.161,0.099,1,0
"Gda",0.00867340105385287,0.0578649929758304,0.058,0.025,1,0
"Acsm5",0.00870145011942244,-0.124171064440682,0.006,0.054,1,0
"Actr10",0.00870977741281908,0.0906481604710678,0.252,0.173,1,0
"Tfam",0.00872511057706631,0.0611014822977754,0.2,0.128,1,0
"Fdxacb1",0.00873382643431387,0.0587314171642508,0.052,0.021,1,0
"Zdhhc5",0.00875090034186904,0.0794893105751664,0.174,0.108,1,0
"Api5",0.00875549743486473,0.132758974232651,0.181,0.116,1,0
"Tex261",0.00877131136296899,0.0637210869989836,0.323,0.227,1,0
"Emcn",0.00877354550260555,0.246733107083942,0.135,0.08,1,0
"Fam179b",0.00877397796583425,0.110495885620673,0.123,0.07,1,0
"Hspa5",0.00878076265875434,0.199543153783157,0.729,0.582,1,0
"Rabif",0.00879203707294443,0.058787195454616,0.116,0.064,1,0
"Igsf9",0.00880775336219522,0.0628087890060413,0.052,0.021,1,0
"Ndufa7",0.00885788203594521,-0.185322988181068,0.819,0.802,1,0
"Dnal1",0.00885891070981089,0.0700505830354615,0.077,0.037,1,0
"Klhdc2",0.00888091441611504,0.0650245541567481,0.277,0.191,1,0
"Osbpl3",0.00888463979462016,0.120403944575273,0.265,0.185,1,0
"Ripk1",0.00893211629747744,0.101470413927068,0.123,0.07,1,0
"Lgalsl",0.00904311406728616,0.0740547501235699,0.065,0.029,1,0
"Klhl20",0.00905593190576768,0.0523928700115151,0.084,0.041,1,0
"Itgb8",0.00907560855983661,-0.126545284475597,0.123,0.212,1,0
"Hmox2",0.00907673176824958,0.119400676465193,0.297,0.216,1,0
"Mapkapk3",0.00909113485677376,0.130390790936694,0.11,0.061,1,0
"Gtf2f1",0.00909548108212408,0.0740132944578858,0.155,0.094,1,0
"4932438A13Rik",0.00910717891069013,0.0906352075195389,0.265,0.183,1,0
"Dzip3",0.00914708377153343,0.0847063460376657,0.084,0.042,1,0
```

"Bin3",0.00915772962243558,0.0740049820209425,0.116,0.065,1,0
"Mrpl48",0.00916668781572973,0.0894036636971978,0.329,0.237,1,0
"Ddx41",0.00918315980973737,0.0899248715665047,0.2,0.131,1,0
"Phactr2",0.00918342344525012,0.0930159474503678,0.2,0.13,1,0
"Psmb5",0.00918554022818376,0.0928765865308421,0.639,0.507,1,0
"Dgcr14",0.00920127803147044,0.0570652047221426,0.065,0.029,1,0
"Hibch",0.00920572210149261,-0.150837671275678,0.039,0.101,1,0
"Nup54",0.00921079695822168,0.0674367201130363,0.097,0.051,1,0
"Tecpr1",0.00923121440003129,0.184759727555184,0.135,0.08,1,0
"Hgsnat",0.00923681098869853,0.10032063438979,0.142,0.084,1,0
"Polr1a",0.00924373571406759,0.0561034772760882,0.077,0.037,1,0
"Bckdhb",0.00924793251364241,0.0605652062380052,0.135,0.078,1,0
"Txnl1",0.00928315320894339,0.0673055452679185,0.381,0.279,1,0
"Gtf2e1",0.00931131281295004,0.0545758077217204,0.065,0.029,1,0
"Letm1",0.0093150478936994,-0.220450490611947,0.181,0.266,1,0
"Ppox",0.00933329790093284,0.0713998514865162,0.11,0.06,1,0
"Acaca",0.0093488476091591,0.0548961129892518,0.071,0.033,1,0
"Prr14l",0.00937946449008632,0.0618010516686107,0.103,0.055,1,0
"Mpst",0.00938443942341535,-0.244309974511153,0.116,0.192,1,0
"Pqlc1",0.00939867821782022,0.110381555477669,0.155,0.096,1,0
"Elovl2",0.00940856284103198,-0.117295028535136,0.013,0.064,1,0
"Emg1",0.00942578091348926,0.0579141548708517,0.381,0.279,1,0
"Ints1",0.00946596363347701,0.0835233678120316,0.103,0.055,1,0
"Fam175a",0.00949397881917279,0.0607899253192524,0.077,0.037,1,0
"Acox3",0.00952707101035949,-0.220652720072544,0.103,0.181,1,0
"Tex10",0.00955746221464867,0.087726635294001,0.09,0.047,1,0
"Qser1",0.00957474679049803,0.105468594635635,0.135,0.081,1,0
"Herc1",0.00959223343879799,0.0773617608193223,0.252,0.169,1,0
"Tango2",0.00961234785730773,-0.272177292626249,0.142,0.217,1,0
"Eea1",0.00965204668480847,0.105942322867379,0.252,0.171,1,0
"Dhx38",0.00965779802053831,0.0548182557866175,0.077,0.038,1,0
"Tti2",0.0096880673541231,0.0581771736013728,0.065,0.029,1,0
"Ndufs2",0.00969610378163062,-0.249972960875583,0.574,0.591,1,0
"Zgpat",0.00970785774097504,0.0675410474315177,0.103,0.055,1,0
"Ly86",0.00971025832460732,0.076979820450963,0.052,0.021,1,0
"Coq9",0.00975726875651246,-0.280241432277201,0.219,0.293,1,0
"Bnip1",0.00977481365677424,0.0551556064244318,0.116,0.065,1,0
"Mgam",0.00979097139098343,-0.115012128515888,0.013,0.064,1,0
"Myo6",0.0098212428788794,0.0687169461376396,0.568,0.431,1,0
"Rnf139",0.00982316116822153,0.0631829423562522,0.161,0.098,1,0
"Thap2",0.0098533088361561,0.121198888627467,0.123,0.071,1,0
"Polr2d",0.00987522099743392,0.0779850476312294,0.129,0.075,1,0
"Sh3bp1",0.0098998009633502,0.092842468011584,0.077,0.038,1,0
"Ppp1r16b",0.00990450391518141,-0.196761654001571,0.045,0.108,1,0
"Slc35a2",0.0099171526471055,0.0732595253719166,0.116,0.065,1,0
"Dtymk",0.00992509184841147,0.0522397697002654,0.258,0.176,1,0
"Fkbp15",0.00993428606305494,0.0864052592374382,0.148,0.089,1,0
"4833420G17Rik",0.00995044422005386,0.0688501362085424,0.123,0.069,1,0
"Tada3",0.00997372439879034,0.0701807266150798,0.116,0.065,1,0
"Fzd7",0.00998396884659442,0.0585121970244048,0.065,0.029,1,0

"Cluh",0.0100130121836402,-0.223191680303228,0.155,0.236,1,0
"Klhdc1",0.0100172943957516,0.0661945486819729,0.084,0.042,1,0
"C2",0.0100179087457193,-0.119837634290533,0.013,0.063,1,0
"Rpap1",0.0100296858748852,0.0512281859235638,0.071,0.033,1,0
"Selt",0.0101242884693273,-0.289489263197423,0.465,0.499,1,0
"Trappc12",0.01014959369977,0.0575612606637437,0.09,0.046,1,0
"Pcna",0.0101515448526821,0.0795305981266577,0.161,0.1,1,0
"Ctnnd1",0.0101640108714476,0.180418279609664,0.232,0.164,1,0
"Ctdsp1",0.0101725690902853,0.0965938430873664,0.277,0.196,1,0
"1110008F13Rik",0.0101870612875435,-0.275364000281595,0.316,0.394,1,0
"Slc52a2",0.0102061561553128,-0.123254948432151,0.039,0.102,1,0
"RP23-218K15.3",0.0102087248236367,0.0657417977694893,0.071,0.033,1,0
"Recql",0.0102487763351823,0.0591697015167243,0.052,0.021,1,0
"Nfrkb",0.0102582409287413,0.0579684839988222,0.084,0.042,1,0
"Bud31",0.0102583942374756,0.0672603755384963,0.406,0.299,1,0
"Slc35c1",0.010280208056627,0.0642745604160189,0.065,0.029,1,0
"Caml",0.0103017763092072,0.0572552659172757,0.168,0.105,1,0
"Usp16",0.0103110301354978,0.0899019134429487,0.239,0.162,1,0
"Fuca1",0.0103314710163793,-0.322822742416546,0.406,0.446,1,0
"Gm17491",0.0103584427154801,0.0800231781761228,0.077,0.038,1,0
"Itpr1",0.0103808810308482,0.0879131048785333,0.477,0.361,1,0
"A430005L14Rik",0.0104114273943984,0.0583557750999904,0.187,0.119,1,0
"Cth",0.0104545944074454,-0.258881079371837,0.103,0.176,1,0
"Msantd2",0.0104797302067503,0.0651104794532206,0.065,0.029,1,0
"Nme4",0.0105311078360973,-0.123073057696853,0.013,0.063,1,0
"Pfkfb3",0.0105995779861639,0.0711593626379435,0.065,0.029,1,0
"Clk2",0.0106113103501406,0.0660060279128566,0.09,0.047,1,0
"Tial1",0.0106282035298955,0.117789032155693,0.194,0.128,1,0
"Ttc4",0.0106508358457329,0.0863349718501631,0.135,0.08,1,0
"Vps37b",0.0106811262758559,0.0837000452830665,0.116,0.066,1,0
"5430403G16Rik",0.0106945077774804,0.0542014459589415,0.052,0.021,1,0
"Nab2",0.0106951061200659,0.0515964523872495,0.077,0.038,1,0
"Arpc5l",0.010792538625872,0.0814427722080555,0.413,0.31,1,0
"Cnot8",0.0107976508587183,0.117676090128468,0.123,0.071,1,0
"P4hb",0.0108096685002377,0.0632042946798438,0.581,0.434,1,0
"Tmem55b",0.0108427593991827,0.142039086361298,0.181,0.118,1,0
"Elp4",0.0108485220227654,0.0705665341672282,0.065,0.03,1,0
"Rnf183",0.010867931739614,-0.151564127258912,0.052,0.116,1,0
"Mta1",0.0108747584114637,0.0816573340309067,0.11,0.061,1,0
"Pmp22",0.010878420967678,0.0524669825081043,0.065,0.029,1,0
"Dut",0.0108983262415873,0.122399662213501,0.194,0.125,1,0
"Rad9b",0.0109022461834103,0.076872944627162,0.052,0.021,1,0
"Mllt10",0.0109063551288463,0.0970631553804945,0.239,0.163,1,0
"Arl6",0.0109153607503398,0.0566601378914002,0.206,0.136,1,0
"Prpsap2",0.0109288388026534,0.0659384684296456,0.103,0.056,1,0
"Phlpp2",0.0109586356283184,0.0783308407067126,0.065,0.03,1,0
"Mtmr6",0.0109588399419337,0.110262316670375,0.142,0.085,1,0
"Map2k7",0.0109815979775193,0.102596346170506,0.129,0.077,1,0
"Lhfpl2",0.0110102378547546,0.0773891189034917,0.058,0.026,1,0
"Fes",0.0110115923294271,0.0584519329701883,0.052,0.022,1,0

"Xpnpep1",0.011025246021542,-0.191433332327112,0.135,0.216,1,0
"Nfu1",0.0110383737778369,0.0583220230986593,0.29,0.201,1,0
"Ppid",0.0110814428605299,0.0976277459814046,0.174,0.111,1,0
"Sin3a",0.0111152643262003,0.0954450422810375,0.11,0.061,1,0
"Fbxl17",0.011116024933564,0.0916428240579522,0.123,0.071,1,0
"Selm",0.0111248474265563,0.0789826304837853,0.361,0.262,1,0
"Ncoa1",0.0111288185810567,0.0955708540605857,0.148,0.091,1,0
"Dcaf13",0.0111637034349812,0.0578680399288428,0.116,0.065,1,0
"Kdelc2",0.0112051371561928,0.0726259102401017,0.071,0.034,1,0
"Clic4",0.0112232835982476,0.206784541772674,0.31,0.227,1,0
"Alkbh8",0.0112493300590484,0.0521537177813839,0.084,0.042,1,0
"Cbx7",0.0112758260305222,0.0529974170539411,0.11,0.06,1,0
"Coa6",0.0113130243806477,-0.271078249744265,0.206,0.287,1,0
"B230354K17Rik",0.0113297382603774,0.0706481543353001,0.103,0.056,1,0
"Ccdc181",0.0113336200660806,0.074602813496022,0.155,0.095,1,0
"Gm37494",0.0113498294451286,0.0854847773367273,0.11,0.061,1,0
"Scaf4",0.0113513271205108,0.111919144697725,0.103,0.057,1,0
"Hmgcr",0.0113593352114764,0.0645562531969962,0.116,0.066,1,0
"Sptssa",0.0113660269434256,0.0641036832462448,0.4,0.294,1,0
"Fam134c",0.0113688426536638,0.0918232566378238,0.161,0.1,1,0
"Kdelr2",0.011390109080352,0.139695113886854,0.323,0.239,1,0
"Tmed2",0.0114239626850978,0.149305680016751,0.594,0.475,1,0
"Qk",0.0115049955557371,0.105815063416811,0.239,0.162,1,0
"Slc25a4",0.0115076413301999,-0.263146315419272,0.742,0.704,1,0
"Ccdc28b",0.0115135383420613,0.0827810228323845,0.135,0.081,1,0
"Cklf",0.011539129802804,0.067592553262228,0.071,0.034,1,0
"Rtfdc1",0.0115600082944761,0.069225993238581,0.219,0.147,1,0
"Nsmce2",0.0115852321630564,0.086743546987051,0.168,0.106,1,0
"Cfp",0.0116010853500842,0.0599145537423431,0.103,0.056,1,0
"Lsm6",0.0116016764846828,0.0774029774751273,0.387,0.285,1,0
"Tapbpl",0.0116221292677596,0.0866418990564545,0.097,0.052,1,0
"Mettl6",0.0116383076313235,0.0628944522345464,0.084,0.043,1,0
"Pccb",0.0116439720156448,-0.215741160111465,0.174,0.259,1,0
"Rlf",0.0116637919579274,0.0609699929948668,0.103,0.056,1,0
"Iqcg",0.0116760245717291,-0.150363672685401,0.026,0.081,1,0
"Abhd13",0.0116790992681997,0.0692664502260859,0.123,0.071,1,0
"Orc4",0.0116902055465107,0.0729675785072417,0.135,0.08,1,0
"Ccnd3",0.0117145576572589,0.0880374195310102,0.271,0.187,1,0
"Fgd4",0.0117644082893408,0.0900432136321862,0.187,0.12,1,0
"AI480526",0.011766472261272,0.0516379200892979,0.11,0.06,1,0
"Wbp1",0.0117665838528223,0.0812749170238553,0.284,0.202,1,0
"Cys1",0.0117792134912966,0.0840924540458742,0.148,0.091,1,0
"Maf1",0.0118017512116798,0.0521138626788067,0.316,0.226,1,0
"Chac2",0.0118168950695613,-0.201838496792072,0.065,0.13,1,0
"Ttc5",0.0118177420988507,0.0713117472561912,0.129,0.076,1,0
"Serhl",0.0118183026042794,0.0782523290906546,0.2,0.132,1,0
"Vamp4",0.0118812008223382,0.0826384659303306,0.168,0.106,1,0
"Acp1",0.0118831810402791,0.0760108311570838,0.368,0.265,1,0
"Pdia3",0.0118859815619041,0.124576626838537,0.735,0.592,1,0
"Smtn",0.0119269052645502,0.147863940730921,0.161,0.103,1,0

"Eif3d",0.0119456375189699,0.121476251496771,0.232,0.162,1,0
"Zfp516",0.0119866566334669,0.0594792622155762,0.065,0.03,1,0
"Cyp2a4",0.012061235577572,-0.164467103453437,0.006,0.051,1,0
"Tmem97",0.0120713482574107,-0.109536066823827,0.006,0.051,1,0
"Chmp3",0.0120764925457233,0.0609098556947567,0.368,0.269,1,0
"Nepn",0.0121526368148062,-0.115201431543743,0.006,0.051,1,0
"Rarres2",0.0121895384485619,-0.216543682771894,0.045,0.106,1,0
"Kpna3",0.0121982354413677,0.0689154028461759,0.135,0.081,1,0
"Smg5",0.0122241149843232,0.0811318391196339,0.135,0.081,1,0
"Tpst2",0.0122351538529314,0.0808619626824783,0.142,0.086,1,0
"Prkab2",0.0122564800773477,0.0537486832165291,0.058,0.026,1,0
"Abcc1",0.012262405721831,0.0762795861073832,0.103,0.057,1,0
"Zfp7",0.0122897039715567,0.0767219954887479,0.065,0.03,1,0
"Hacl1",0.0123189488027742,-0.128237983411613,0.026,0.081,1,0
"Slc25a23",0.0123312178596571,0.0656276587491679,0.219,0.147,1,0
"Rbm15b",0.0123709760966943,0.0745043836388983,0.084,0.043,1,0
"Prr12",0.012392566634746,0.0529898609943544,0.077,0.038,1,0
"Ublcp1",0.0124299268225279,0.0722773701198768,0.174,0.111,1,0
"Zc3h4",0.0124330490474191,0.0569710996711158,0.11,0.061,1,0
"2310036O22Rik",0.0125599329816889,0.0757659759946853,0.516,0.402,1,0
"Glb1l2",0.0126585262124089,-0.137224280530298,0.065,0.132,1,0
"Zfp704",0.0126826877984227,0.121650289140888,0.194,0.129,1,0
"Pelo",0.0126867738922134,0.0807527333953137,0.142,0.086,1,0
"Map9",0.0127324092390556,0.0827776746990948,0.071,0.035,1,0
"Exosc2",0.0127796725962052,0.0659701444967235,0.084,0.043,1,0
"Dyrk1a",0.0128373880409101,0.0983087030839934,0.148,0.092,1,0
"Dhx32",0.0128483311420246,0.120613443822697,0.129,0.076,1,0
"4632415L05Rik",0.0128621729566939,0.0920064165516113,0.097,0.053,1,0
"Usp39",0.0128720277404576,0.121683435799651,0.097,0.053,1,0
"Caprin1",0.0128761044778633,0.0547121469887073,0.477,0.36,1,0
"Gmeb1",0.0129002448266045,0.0550288492910501,0.071,0.034,1,0
"Bloc1s2",0.0129205326035295,0.0693945381077163,0.187,0.122,1,0
"Rbm12",0.0129205466442796,0.0659620483030882,0.077,0.039,1,0
"Htt",0.012922760389889,0.0786125892694328,0.181,0.116,1,0
"Mybbp1a",0.0129314099838482,0.114718465049543,0.148,0.093,1,0
"Gusb",0.0129402977146568,0.0997626261475808,0.155,0.098,1,0
"Tmem57",0.0129468938636914,0.137947939771334,0.174,0.115,1,0
"Cdkn2c",0.0129726029726329,-0.13327911414103,0.032,0.089,1,0
"Ranbp10",0.0130208027055302,0.0580766159125068,0.084,0.043,1,0
"Mpp7",0.0130297771348602,0.0704047474906032,0.09,0.048,1,0
"Gm26526",0.0130367221170939,0.0543855523481823,0.058,0.026,1,0
"Abce1",0.0130766359993192,0.0979346061528785,0.181,0.115,1,0
"Slc23a2",0.0131134124090312,0.0663171979277993,0.065,0.03,1,0
"Glrx5",0.0131212282367268,-0.254985649114448,0.394,0.448,1,0
"Ssbp1",0.0131675799560734,0.0949415381374201,0.323,0.237,1,0
"Ccni",0.0131828442577367,0.0639833501555263,0.439,0.333,1,0
"Prss8",0.0132111002791082,-0.274601454793735,0.271,0.344,1,0
"Per3",0.0132196173440734,0.121266211258802,0.097,0.053,1,0
"Dixdc1",0.0132481486359524,0.0657335062071678,0.071,0.034,1,0
"Dazap1",0.0132741727203108,0.0659621386816633,0.258,0.181,1,0

"Fam214b",0.0132842737599152,0.0620069270778264,0.084,0.043,1,0
"Sar1b",0.0132955559751406,-0.243070227816141,0.29,0.368,1,0
"Ivd",0.0133095166939471,-0.228207998335137,0.148,0.226,1,0
"Cnot11",0.013312071354569,0.055714886890729,0.129,0.076,1,0
"Gdap2",0.0133159962120461,0.0671887720906229,0.071,0.034,1,0
"Tmem59",0.0133192374101373,0.11162682577932,0.748,0.588,1,0
"Nr3c2",0.0133452356701716,0.0670445631088554,0.071,0.035,1,0
"Cant1",0.013345288702525,0.123748722022314,0.123,0.073,1,0
"Tmed4",0.0133641801427144,0.0502905040457626,0.361,0.267,1,0
"Yipf4",0.013370282562108,0.0529915197003629,0.368,0.263,1,0
"Acss1",0.0133736319592357,-0.303278052754915,0.265,0.329,1,0
"Mrpl20",0.0133775483646662,-0.28894432299362,0.477,0.507,1,0
"Cse1l",0.0134469906389732,0.0923303086664221,0.135,0.082,1,0
"Adar",0.0134619464531249,0.0794784678570009,0.084,0.044,1,0
"Cpsf3l",0.0134973322227902,0.0530556092442056,0.103,0.057,1,0
"Dnajc25",0.0134990179827336,0.0962943672629515,0.129,0.077,1,0
"Samd1",0.0135461858963404,0.0601074817751794,0.077,0.039,1,0
"Pgp",0.0135700570943594,0.0619374381281065,0.271,0.189,1,0
"Kcnq1",0.0135903287062333,0.104108318735312,0.129,0.077,1,0
"Cfap20",0.0136244740143369,0.0710008990189121,0.142,0.087,1,0
"Trak2",0.0136596737120068,0.072582114782513,0.2,0.132,1,0
"Cnot2",0.0137013683975465,0.149695835741901,0.161,0.104,1,0
"Tmf1",0.0137071749565293,0.0681942405821956,0.277,0.194,1,0
"Psmc2",0.0137238971999515,0.0836461025247819,0.297,0.215,1,0
"Gpatch4",0.0137338333208443,0.112123387307423,0.116,0.068,1,0
"St7",0.0137732928161154,0.0602486758267588,0.065,0.03,1,0
"Acad11",0.0137966860595494,-0.170111255947794,0.103,0.179,1,0
"Rab4a",0.0138056973649761,-0.136756646144403,0.058,0.123,1,0
"Taf4a",0.0138748261876219,0.0535593170511738,0.103,0.057,1,0
"Cep170b",0.0138893278380284,0.0857528357773128,0.181,0.118,1,0
"Igha",0.0139038714120702,-0.0638787095449097,0.071,0.035,1,0
"Ints4",0.0139095428242709,0.0577392954594886,0.09,0.048,1,0
"Rad17",0.0139645873429948,0.0556210005148287,0.11,0.062,1,0
"Gpbp1l1",0.0139817972579629,0.0901902156107067,0.232,0.162,1,0
"Npnt",0.014024858825511,0.203597864562721,0.31,0.234,1,0
"Exosc9",0.0140410248855216,0.0566716235641526,0.103,0.057,1,0
"Ncapd3",0.0140520234867109,0.0606616751281053,0.084,0.043,1,0
"Lrba",0.0140781765382975,0.0501546696849108,0.206,0.136,1,0
"Tipin",0.0140929246879319,0.0640765223448523,0.103,0.057,1,0
"Mief1",0.0141545148475165,0.05578326031686,0.084,0.043,1,0
"Ankmy2",0.014160906061942,0.068124973156189,0.116,0.067,1,0
"Ddx27",0.0141655387250692,0.0910301764707327,0.135,0.082,1,0
"F8a",0.0142382384828215,0.0775384039483624,0.11,0.062,1,0
"Socs6",0.0142605588337857,0.0654710331309911,0.142,0.087,1,0
"Tbc1d13",0.014303460281708,-0.144965619304573,0.045,0.104,1,0
"Rrp8",0.014310968072637,0.0902183822368817,0.097,0.053,1,0
"Tmem259",0.014436896183848,0.0620881859900287,0.252,0.175,1,0
"Phf23",0.0144530826795363,0.080360024263156,0.123,0.072,1,0
"Fgg",0.0144694299885649,-0.380532596579735,0.071,0.137,1,0
"Mrps18c",0.0145060395296982,0.0788201474355347,0.413,0.312,1,0

"Rab20",0.0145097989046657,0.111613967254178,0.213,0.146,1,0
"Asap1",0.0146368331567028,0.13013102675882З,0.103,0.059,1,0
"Rpn2",0.0146433284432277,0.0654102240064948,0.387,0.289,1,0
"Tmub1",0.0146737080298647,0.0944631623452307,0.11,0.062,1,0
"Cyp27a1",0.0147474348337511,-0.126597901505065,0.013,0.059,1,0
"Mid2",0.0147657237413697,0.0635120644036066,0.071,0.035,1,0
"Ints7",0.0147833139451304,0.0549587821728862,0.09,0.048,1,0
"Rnf25",0.0147835722771732,0.106222282826713,0.09,0.049,1,0
"Bad",0.0147969734190026,0.0691849809543451,0.277,0.199,1,0
"Acy1",0.0148005783683982,-0.149278112213516,0.019,0.068,1,0
"Slmap",0.0148070471748193,0.0649692676089457,0.252,0.176,1,0
"Hmgcs1",0.0148156905935898,-0.30465256898527,0.452,0.483,1,0
"Pfas",0.0148648035357989,0.0718215540205609,0.116,0.067,1,0
"Gm26601",0.0148924902494632,0.0524230246722031,0.052,0.022,1,0
"Sat1",0.0148962710316917,0.178066425300509,0.8,0.659,1,0
"Clcnka",0.0149764531814844,0.0534133792139283,0.116,0.067,1,0
"Adck4",0.0150252286822942,0.0546746216858187,0.103,0.058,1,0
"Jam3",0.0150286888749614,-0.121336032540178,0.026,0.079,1,0
"Tmem94",0.0150416227047458,0.070986525338509,0.135,0.082,1,0
"Tmem218",0.0150609128835432,0.0710139449238145,0.11,0.062,1,0
"Senp2",0.0150811953660388,0.0741341072773413,0.11,0.063,1,0
"Yif1a",0.0150926977874334,-0.221888060766884,0.194,0.273,1,0
"Rap1gds1",0.0151225775229444,0.0657878941708282,0.123,0.072,1,0
"Eif2s3y",0.0151940097733883,0.0589397395889243,0.239,0.164,1,0
"Hdac5",0.0151992786367421,0.0792006905486692,0.155,0.098,1,0
"Rbm48",0.0152640987044891,0.0710461215861542,0.071,0.035,1,0
"Ctdnep1",0.0153371944012165,0.105075546796009,0.187,0.126,1,0
"C2cd3",0.015355815713824,0.0873944605053887,0.09,0.049,1,0
"Scoc",0.0153587954432212,-0.253294520667726,0.258,0.332,1,0
"Psmd10",0.0154164145254764,0.134624184514411,0.219,0.156,1,0
"Tspyl2",0.0154442044265787,0.0730570153638261,0.071,0.035,1,0
"Nelfb",0.015504360664595,0.109473042481206,0.181,0.121,1,0
"Zdhhc16",0.0155053654379406,0.0533877896354926,0.123,0.072,1,0
"Nr1h3",0.0155314399996723,-0.11317041283034,0.026,0.079,1,0
"Tubgcp4",0.0155390114288351,0.0729956799544527,0.142,0.088,1,0
"Hyal1",0.0155595368412165,0.0854713351235124,0.058,0.027,1,0
"Phf1",0.0155746192930606,0.0783257330906961,0.077,0.039,1,0
"Ptges",0.0157165748336104,0.0811628025888559,0.123,0.072,1,0
"Etv3",0.0157281236246811,0.0785854903657055,0.084,0.044,1,0
"Dpp8",0.0157432573308196,0.072632144972376,0.116,0.068,1,0
"Gm43672",0.0157653061320675,0.0558704183139924,0.058,0.027,1,0
"Rcn1",0.0157717663600124,0.0786908739181605,0.123,0.073,1,0
"Zfp101",0.0157813427726819,0.0540794825610693,0.09,0.048,1,0
"Ugp2",0.0157889329827766,-0.213934035015471,0.206,0.287,1,0
"Mtus1",0.015831408341409,-0.16129769661142,0.065,0.127,1,0
"Dnajc13",0.015849413867221,-0.185407951946551,0.11,0.182,1,0
"Abcc4",0.0158632131752452,0.0928949657500377,0.142,0.088,1,0
"Fam114a2",0.0159039306523522,0.138649886324371,0.2,0.138,1,0
"Atg7",0.0159069470024129,0.0592647995198965,0.084,0.044,1,0
"Pappa",0.0159193053561487,0.0626256088714316,0.077,0.039,1,0

"Synj2bp",0.015957701865648,0.0601824317307888,0.329,0.242,1,0
"Strn3",0.0159741938378553,0.107498285266695,0.31,0.231,1,0
"Tex9",0.0160498080364237,0.0915995819226487,0.103,0.058,1,0
"Mvd",0.0160913191558669,-0.122721574495661,0.039,0.096,1,0
"Pml",0.0160973459779765,0.0552987278582294,0.084,0.044,1,0
"Slc16a12",0.0161101714141939,-0.242169904071421,0.161,0.233,1,0
"Taf1b",0.0161594417154515,0.0686321471494086,0.065,0.031,1,0
"Mrs2",0.0161911107037898,0.0625731277629963,0.09,0.049,1,0
"1700088E04Rik",0.0162344817399464,0.0910372568590133,0.103,0.059,1,0
"Pigk",0.0162907128361596,0.101256776125235,0.181,0.119,1,0
"Tpd52",0.0162936393528875,0.086815868227254,0.497,0.397,1,0
"Parg",0.0163729944537927,0.0727837415402051,0.116,0.068,1,0
"Cnn3",0.0163803295851398,0.105726685496495,0.439,0.336,1,0
"Zfp637",0.0163850733385431,0.0839624142192961,0.161,0.103,1,0
"Capn15",0.016429718206789,0.0618128979073277,0.058,0.027,1,0
"Cops5",0.016484536807514,0.0978637057493842,0.29,0.213,1,0
"Dtna",0.016521615523052‌8,0.0637398935070411,0.071,0.036,1,0
"Cyp39a1",0.0165277941969768,0.10799358340192,0.077,0.041,1,0
"Gpd1l",0.0166302698322898,0.0594046702344561,0.103,0.058,1,0
"Shank2",0.0166422862278578,0.0782351373378265,0.097,0.054,1,0
"Telo2",0.0166843977716237,0.0650755039599741,0.077,0.04,1,0
"Ptdss1",0.0167027468278092,0.0647418339775748,0.168,0.108,1,0
"Vbp1",0.0167067693432055,0.122431859441832,0.245,0.171,1,0
"Trim7",0.0167694779219239,0.16109976068407,0.213,0.146,1,0
"Slc35b1",0.0168355891552792,0.0753062866024217,0.213,0.145,1,0
"Mkl2",0.0168503448930681,0.0744810643664261,0.11,0.064,1,0
"Rev1",0.0168532588274883,0.0839194148249399,0.084,0.045,1,0
"Atp6v1h",0.0168901144863117,0.0731413758382283,0.316,0.231,1,0
"Gamt",0.0169196800518922,-0.133219836751256,0.019,0.067,1,0
"Acbd4",0.0169427825677074,-0.234467055033145,0.116,0.185,1,0
"Zmat5",0.0169732471668829,0.0733780222997256,0.245,0.173,1,0
"Iqsec1",0.017015238161886,0.0824579053604093,0.181,0.119,1,0
"Ptms",0.0170306600603029,0.0660452422014144,0.768,0.601,1,0
"Tomm7",0.0170862228607969,-0.204098282989182,0.755,0.733,1,0
"Foxp4",0.0171161217975094,0.106747871354554,0.084,0.045,1,0
"Rrp36",0.0171823110488968,0.0731629616247614,0.097,0.054,1,0
"Sec62",0.0172601227779353,0.0965704899822477,0.871,0.702,1,0
"Socs4",0.0173371514704479,0.0896607971809276,0.155,0.1,1,0
"Ldah",0.0173431209026483,0.0707157388048451,0.11,0.063,1,0
"Tm7sf3",0.0173742435860308,-0.186489983548906,0.097,0.165,1,0
"Plagl1",0.0174231881977674,0.0638781192339452,0.084,0.044,1,0
"Slc45a4",0.0174632878223681,0.0939065581324707,0.135,0.084,1,0
"Tshz1",0.0174638361520611,0.0849787047747225,0.181,0.119,1,0
"Mafb",0.0174697708985971,0.106131958962734,0.11,0.064,1,0
"Wac",0.017483411649566,0.125201671736816,0.245,0.176,1,0
"Usp15",0.0175756156667191,0.0631278446214959,0.142,0.089,1,0
"Mrps12",0.0176044760804561,-0.248782071095084,0.271,0.343,1,0
"Mrps24",0.0176233197315282,-0.264249570042505,0.51,0.523,1,0
"Mphosph10",0.0176565881458821,0.0834569133945997,0.11,0.064,1,0
"Ercc5",0.0176842725914887,0.0566641773257175,0.084,0.044,1,0

"Nuak2",0.0177092101838406,0.135134891659247,0.271,0.195,1,0
"March6",0.0177202865111137,-0.233709252798205,0.181,0.253,1,0
"Pepd",0.0177921320293424,-0.213225874070899,0.252,0.339,1,0
"Pex10",0.0178115870587095,0.0533504628382184,0.065,0.031,1,0
"Zfp120",0.0178684354305202,0.0591357392695507,0.084,0.044,1,0
"Zfp217",0.0179363326856183,0.0535419835717073,0.084,0.044,1,0
"Mcmbp",0.0179814791169316,0.0991049314570021,0.155,0.1,1,0
"Upf1",0.0179859398614817,0.0663810709461052,0.174,0.113,1,0
"R3hcc1",0.0179974684751992,0.0798193376465738,0.09,0.049,1,0
"Abr",0.0180104900606061,0.1097276396445,0.123,0.075,1,0
"Sec11c",0.018031959825509,0.074278439369789,0.29,0.211,1,0
"Cbwd1",0.0180572928421091,0.0658482529569937,0.065,0.031,1,0
"Larp1",0.0180993015902899,0.129657005095129,0.213,0.15,1,0
"L3hypdh",0.0182182056875667,-0.167683325288646,0.052,0.11,1,0
"Pcmt1",0.0183488063248683,0.0737264049197296,0.31,0.229,1,0
"Lrrc47",0.0183581282559088,0.0709654710493598,0.11,0.064,1,0
"Ubash3b",0.0183635272074092,0.0772546544995029,0.065,0.031,1,0
"Tom1l1",0.0183759196114366,0.0818920360274346,0.103,0.059,1,0
"Ptprd",0.0183890144505649,-0.208205987769319,0.155,0.229,1,0
"Kat6b",0.0184547912423579,0.0860851027717495,0.103,0.059,1,0
"Rps21",0.0184913075818791,0.134744755618092,0.865,0.812,1,0
"Erlec1",0.0185245895361665,0.0677939982116434,0.252,0.178,1,0
"Sh3pxd2a",0.0185278445793376,0.0633659699593534,0.065,0.032,1,0
"Cox17",0.0185654420888145,-0.172782075360798,0.716,0.718,1,0
"Avpr2",0.0185829560782504,0.0609666357434105,0.065,0.031,1,0
"Rraga",0.018635232642494,0.104736408654635,0.239,0.17,1,0
"Myd88",0.0187263264316139,0.0945767059711063,0.084,0.045,1,0
"Ctu2",0.0187721728629246,0.0640506207530989,0.071,0.036,1,0
"Mterf4",0.0188685030376065,0.05425987939233,0.097,0.054,1,0
"Cep126",0.0188922013628525,0.0696383101657626,0.071,0.036,1,0
"Tubg1",0.0189502924431495,0.0772312874785607,0.097,0.054,1,0
"Epn1",0.0190977110762997,-0.24715012615166,0.277,0.348,1,0
"Mttp",0.0191068790816702,0.080758557659759,0.097,0.054,1,0
"Ipo8",0.0191277330395115,0.13783295540042,0.194,0.135,1,0
"Acot8",0.0191496285013432,0.0685916139454055,0.187,0.125,1,0
"AI317395",0.0191745135871978,-0.18557772563684,0.11,0.18,1,0
"Ago3",0.0191943018506587,0.0836796003444467,0.11,0.064,1,0
"Cox16",0.0192455418146017,0.0522480156240312,0.116,0.068,1,0
"Mib2",0.0192532932965787,0.0522474521163927,0.097,0.054,1,0
"Usp20",0.0193768664455471,0.102840217231029,0.11,0.064,1,0
"Tmed3",0.019394008574128,0.0872756469615991,0.277,0.203,1,0
"Pdcd5",0.0194058033566269,0.112033813173966,0.523,0.411,1,0
"Sema6a",0.0194118811023686,0.103018177313141,0.065,0.032,1,0
"Wdr46",0.0194892473001372,0.0506761229056132,0.097,0.054,1,0
"Gclc",0.01949157125535,0.0717333766892875,0.348,0.257,1,0
"Snhg6",0.0195161135859842,0.0752072978431578,0.226,0.157,1,0
"Slc41a1",0.0195519817734342,0.0839040678882475,0.084,0.046,1,0
"Ak6",0.0195579760333832,0.0653613955783688,0.232,0.161,1,0
"Hypk",0.0196132582500582,0.0914614579617523,0.555,0.436,1,0
"Atp6v1e1",0.0196537811373117,-0.292292406084115,0.677,0.657,1,0

"Fcho2",0.0197902305277306,0.0951284201022816,0.219,0.154,1,0
"Kdm3b",0.0198047317234918,0.0666771166957082,0.123,0.074,1,0
"Gm10073",0.0198428239742728,0.0990853773728743,0.535,0.434,1,0
"Gpr137",0.0199363337634991,0.0904292291860591,0.116,0.07,1,0
"Hiat1",0.0199439512741289,0.111929673604083,0.213,0.147,1,0
"Tssc4",0.0199589254265211,0.056919353123549,0.148,0.094,1,0
"Atpaf1",0.0199906906536504,0.0573444734493838,0.142,0.088,1,0
"Gps1",0.0201007227482921,0.0569267917800964,0.265,0.191,1,0
"Fgf1",0.0201168803391506,-0.122417474836582,0.071,0.135,1,0
"Hspa13",0.0201172048442048,0.0958551875992057,0.116,0.07,1,0
"Tonsl",0.0202359895552838,0.0970978981877764,0.439,0.336,1,0
"Tmem176a",0.0202879892954827,0.102935020860184,0.929,0.784,1,0
"9330151L19Rik",0.0203258658829935,0.0534601741247991,0.052,0.023,1,0
"Tmem135",0.0203449665338644,0.111859996205728,0.258,0.189,1,0
"Cfi",0.0204500120773531,-0.122034908378532,0.032,0.083,1,0
"Snapc1",0.0205065550206822,0.0516178817420397,0.09,0.05,1,0
"Mcm6",0.0206254015836151,0.0531984248550884,0.097,0.055,1,0
"Fam117b",0.0207471450944752,0.108137369808092,0.11,0.065,1,0
"Brk1",0.0207944748617304,0.0566771147041474,0.497,0.378,1,0
"Whrn",0.0207984728304632,0.0878666940664177,0.077,0.041,1,0
"Smarca4",0.0208749929145338,0.121824707824801,0.213,0.152,1,0
"BC005537",0.0209534703999446,0.0832852279236421,0.471,0.366,1,0
"Sirt3",0.0209548647844704,-0.205414814629246,0.116,0.183,1,0
"Vamp3",0.020960346293312,0.0643466009742953,0.181,0.121,1,0
"Bcdin3d",0.0209623671365304,0.0604757980922707,0.058,0.027,1,0
"Sigirr",0.0209792217739685,-0.162648416605695,0.103,0.174,1,0
"Ubqln2",0.0209981896813944,0.112447794347351,0.129,0.081,1,0
"Ercc3",0.0210134094746317,0.0601812443951674,0.077,0.04,1,0
"Cyth1",0.021234251750799,0.0884314082154698,0.097,0.055,1,0
"Rdh11",0.0213445874287383,0.0528646230542796,0.084,0.045,1,0
"Zfp697",0.0214545432004419,-0.118732440775691,0.039,0.092,1,0
"Sdf2l1",0.021493349575759,0.10563916030397,0.219,0.153,1,0
"Nr2f6",0.0215165163392115,-0.205394851772076,0.277,0.356,1,0
"Rpl7a",0.0215345290499063,0.116195386800959,0.865,0.755,1,0
"Snx1",0.021541946706468,0.0907755194475485,0.206,0.143,1,0
"Iqce",0.0215992916525876,0.0806855913510478,0.084,0.046,1,0
"Pja1",0.021627588844255,0.0811795058545076,0.181,0.121,1,0
"Gbf1",0.0217142724808986,0.085017688478961,0.142,0.09,1,0
"Micu3",0.0218357780590804,0.0862065879526817,0.123,0.074,1,0
"Il1b",0.0218508641231568,0.539918251891536,0.135,0.087,1,0
"Gm11273",0.0221013847281304,-0.238793096776759,0.206,0.278,1,0
"Nsun5",0.022142483683769,0.0656057277978263,0.058,0.028,1,0
"Nxt1",0.0221694490715457,0.0637469339885732,0.123,0.075,1,0
"Cnnm2",0.022173109901093,0.0703515165150564,0.11,0.065,1,0
"Dhx40",0.0221765410957467,0.0610361288122526,0.194,0.13,1,0
"Ankrd49",0.0222377823736476,0.0564492527319218,0.071,0.036,1,0
"Tatdn3",0.0222845518084575,0.0512201398467204,0.058,0.028,1,0
"L3mbtl3",0.022296808302756,0.0590206510918085,0.065,0.032,1,0
"Lrrc59",0.0223628521550759,0.070263700323837,0.187,0.126,1,0
"Uhrf1bp1",0.0223787598813805,0.0724373119608874,0.058,0.028,1,0

"Gcn1l1",0.0225117054434209,0.086838604760935,0.194,0.134,1,0
"Tor2a",0.022594667134576,0.073992259883944,0.181,0.121,1,0
"Aftph",0.0226787195503529,0.0811637623116568,0.271,0.196,1,0
"Mdh2",0.02281900261578,-0.249074590417297,0.677,0.65,1,0
"Elovl5",0.0228260749101642,-0.136359153704254,0.071,0.134,1,0
"H2-Q7",0.0228612597341654,0.112854796261616,0.09,0.051,1,0
"Dusp11",0.0228969972043892,0.0649596926136239,0.29,0.213,1,0
"Dlc1",0.0229122413892179,0.0735754571117124,0.052,0.024,1,0
"4921524J17Rik",0.0229526222635873,0.0922448740204406,0.148,0.095,1,0
"Slc26a2",0.0231199289124464,0.0611723733901264,0.077,0.041,1,0
"Man2c1",0.0231245766095438,0.0901101875550351,0.11,0.065,1,0
"Tle4",0.0232243970880431,0.0753762941307498,0.103,0.06,1,0
"Ipo9",0.0232747453390963,0.08430144237914,0.11,0.065,1,0
"Kif1c",0.023287563694522,0.0897699790434633,0.271,0.201,1,0
"Evpl",0.0234336531163747,0.0754075976198316,0.065,0.032,1,0
"Atp6v0a1",0.0235326533857859,0.0960374802192486,0.11,0.064,1,0
"Selo",0.0235527810993597,0.0500811541885675,0.135,0.084,1,0
"Nudt12",0.023564244308859,-0.0814088629212338,0.013,0.054,1,0
"Reps1",0.0235689957957045,0.0937786949719278,0.123,0.076,1,0
"Lgals1",0.0235811235845142,-0.246476003124608,0.49,0.521,1,0
"Kmt2c",0.0236513754186638,0.124536355173297,0.194,0.137,1,0
"Dock10",0.0236655011366134,0.083496347755419,0.065,0.032,1,0
"Maml1",0.0237168212386129,0.0504951547461693,0.058,0.028,1,0
"Mesdc1",0.0237218921052617,0.0781438723321939,0.084,0.047,1,0
"Cenpc1",0.0238417535661313,0.0746810491115028,0.09,0.051,1,0
"Aste1",0.0238831231522717,0.0630562782625144,0.065,0.032,1,0
"Plagl2",0.0239505686152129,0.0695930094672405,0.065,0.033,1,0
"Kat2b",0.0240730484588731,0.0594874914527957,0.187,0.126,1,0
"Banf1",0.0240959529983356,0.0607468000275771,0.348,0.261,1,0
"Myadm",0.0242101270287674,0.0706360972820419,0.077,0.042,1,0
"Stt3b",0.0244085492869446,0.0965639916426971,0.303,0.23,1,0
"Pbx3",0.0244088584636544,0.0565127498653526,0.065,0.032,1,0
"Ptrhd1",0.02450030506454,-0.166687747439509,0.129,0.202,1,0
"Rrs1",0.0245265276436986,0.0875127886455583,0.161,0.107,1,0
"Mbip",0.0245362345809513,0.0610311043330629,0.084,0.046,1,0
"Sbf2",0.0245627118428153,0.109993155494559,0.181,0.125,1,0
"Tnfrsf21",0.0246063960466661,-0.231453616879382,0.187,0.257,1,0
"Cyb5r3",0.0246445266815062,0.0510117021596497,0.413,0.311,1,0
"D11Wsu47e",0.0246916463019228,0.0905760780315842,0.052,0.024,1,0
"Mterf3",0.0247435743170136,0.0782171905780794,0.123,0.076,1,0
"Serpina10",0.0247515905873573,0.0874898969922682,0.103,0.06,1,0
"1810013L24Rik",0.024805545249551,0.0683218003337015,0.129,0.081,1,0
"Pgls",0.0248114010928745,0.0773171082661814,0.465,0.365,1,0
"Mllt6",0.0248730490520542,0.0694289106986622,0.161,0.106,1,0
"Sdccag8",0.0249210346446527,-0.124341213398659,0.039,0.091,1,0
"Fbxl12",0.025117719934591,0.0671630549665198,0.077,0.042,1,0
"Mmaa",0.0251484686676713,0.0544710995024721,0.103,0.06,1,0
"Ddhd2",0.0252766472481334,0.0613429890255796,0.11,0.065,1,0
"Xpo1",0.025336174717204,0.10324139331435,0.148,0.097,1,0
"Nt5c2",0.0253433230755114,0.12227113524494,0.174,0.12,1,0

"Khsrp",0.0254279405601636,0.072044239921239,0.097,0.056,1,0
"Enpep",0.0255139374324824,-0.124021555859493,0.065,0.124,1,0
"Fads6",0.0255270375271267,0.0907532776142339,0.071,0.037,1,0
"6330408A02Rik",0.0255499167133861,0.0713186150769076,0.052,0.024,1,0
"Gm15417",0.0255557323146559,-0.0962061547070009,0.032,0.082,1,0
"Endog",0.0255570909416059,-0.234331816127236,0.161,0.227,1,0
"Pusl1",0.0255585253003475,0.0503396611648414,0.065,0.032,1,0
"Svil",0.0256149699351278,0.106110902277769,0.123,0.076,1,0
"Cops3",0.0257965247960921,0.0819694189790626,0.206,0.145,1,0
"Fam118b",0.0258386740217163,0.0740124553846193,0.09,0.051,1,0
"Smarcc2",0.0258780541858703,0.088811466611103,0.206,0.146,1,0
"Desi1",0.0259305953495739,0.0796694771746921,0.161,0.108,1,0
"Katnbl1",0.0260906955897436,0.0649753820221924,0.11,0.066,1,0
"Bag3",0.0261270269622326,0.0570989849016208,0.097,0.056,1,0
"Ccl27a",0.0261274625688241,0.0554374922102165,0.181,0.122,1,0
"Gpm6a",0.0261977925259192,-0.13850403621887,0.032,0.08,1,0
"Prim1",0.0262094673796613,0.0583401742603464,0.052,0.024,1,0
"Tob2",0.0262459207282578,0.0915820500241822,0.168,0.113,1,0
"Scaf8",0.026288125750235,0.0502286713759282,0.116,0.071,1,0
"Gm29107",0.0263306032827613,0.0589705980830326,0.065,0.033,1,0
"Mre11a",0.026339718810412,0.0595965486142584,0.058,0.028,1,0
"Pop5",0.0264362124757652,-0.219137340269137,0.31,0.38,1,0
"Rrm2b",0.0264763598296541,0.0501140842727078,0.11,0.066,1,0
"Slc30a5",0.0265400330437369,0.0681774404954921,0.187,0.129,1,0
"S100g",0.0266228361747349,0.828556068942794,0.548,0.47,1,0
"Usp21",0.0266444795379424,0.0503801214706608,0.065,0.033,1,0
"0610037L13Rik",0.0266813616986889,0.0502035826207565,0.206,0.143,1,0
"Epb41l5",0.0267052947156611,0.0646952503941938,0.265,0.191,1,0
"Slc39a14",0.0267566955082768,0.0892187481702602,0.09,0.052,1,0
"Vps13d",0.0268197916304493,0.0660620445427448,0.155,0.102,1,0
"Lancl1",0.0268893791709625,0.0548059276721318,0.123,0.076,1,0
"Myo7b",0.0268937411068117,-0.101068031883031,0.019,0.062,1,0
"Abl2",0.0269500746043381,0.0651918332771458,0.084,0.047,1,0
"Pik3ca",0.0270362591351741,0.12304468999233,0.219,0.158,1,0
"Abcf2",0.0270427521227311,0.0627609886876047,0.129,0.081,1,0
"Cyp4f13",0.0271221006946656,0.0831472676340571,0.123,0.076,1,0
"Dgcr6",0.0272104653041893,-0.254435981131587,0.316,0.368,1,0
"Tob1",0.0272676630185175,0.0721172077779808,0.31,0.224,1,0
"Glud1",0.0272928707541122,-0.252796608954221,0.439,0.484,1,0
"Reep4",0.0272963650007895,0.0640278096359004,0.071,0.038,1,0
"Smim12",0.0273513114430474,0.105771640272322,0.245,0.178,1,0
"Gm12216",0.0275088033749559,0.0734019997224728,0.097,0.056,1,0
"Pak4",0.0275667825557757,0.0853444693299848,0.129,0.082,1,0
"Ncoa3",0.0276190451909242,0.0995462024583773,0.168,0.113,1,0
"Cacnb4",0.027692988407079,0.0545438656853545,0.077,0.041,1,0
"Smyd4",0.0277355440604648,0.0546234631033444,0.084,0.047,1,0
"Bnc2",0.0277483847144878,-0.114239244083359,0.026,0.071,1,0
"Wsb2",0.0277597581919936,0.0548474604260125,0.297,0.22,1,0
"Dazap2",0.0277796583507747,0.0829584218289994,0.49,0.399,1,0
"Uba6",0.0277883863778762,0.0595315651007807,0.097,0.056,1,0

"Cmtm4",0.0278538555780239,0.0860187603690935,0.206,0.144,1,0
"Casd1",0.0278639431736284,0.0776730698575014,0.123,0.077,1,0
"Srpk1",0.0279409297298163,0.122262491720947,0.252,0.189,1,0
"Hiatl1",0.0280520436541409,0.0562640667902036,0.148,0.097,1,0
"Ift80",0.028061052116474,0.0746446722454549,0.077,0.042,1,0
"Prickle3",0.0281287810242973,0.0504356504238589,0.058,0.029,1,0
"Cwc15",0.0281338649476609,0.0714860570757984,0.374,0.286,1,0
"1700040L02Rik",0.0281832105859288,0.0557563873575184,0.09,0.051,1,0
"Stt3a",0.0282257763209881,0.107953070456509,0.258,0.191,1,0
"Trpc1",0.0282841608029827,-0.145270505126682,0.052,0.106,1,0
"Prkca",0.0282938052399199,0.109984230165232,0.103,0.062,1,0
"Aimp2",0.0284962936912963,0.070545767748284,0.129,0.082,1,0
"Wdr5",0.0285724581906263,0.0625697070303439,0.11,0.066,1,0
"Gm10269",0.0286263763241536,0.0672122216235219,0.09,0.051,1,0
"Mrgbp",0.0286288226201285,0.0720151155295846,0.071,0.038,1,0
"Prdx2",0.0287241488818867,0.080758047439708,0.761,0.614,1,0
"Htra2",0.0287267668424149,0.0601894559503642,0.116,0.071,1,0
"Ppp2r5c",0.0287985043278343,0.106320162811725,0.161,0.11,1,0
"Asnsd1",0.0289584046774461,0.0614051770342369,0.148,0.097,1,0
"Etv6",0.0290944182515347,0.0606493801007224,0.103,0.061,1,0
"Col27a1",0.0291085837680567,-0.122099419274657,0.032,0.08,1,0
"Eif1ax",0.0292393947285055,0.0730486416070171,0.394,0.3,1,0
"Srr",0.0293499859892823,-0.164374725066549,0.077,0.135,1,0
"Pkn1",0.0293640715479529,0.0849243247924395,0.148,0.098,1,0
"Ctbs",0.02940058417655,-0.103713788768259,0.026,0.071,1,0
"Eif4enif1",0.0294655193661656,0.0864781318936867,0.116,0.072,1,0
"Perp",0.0294721160491536,0.0852509435002817,0.232,0.168,1,0
"Rere",0.0295723386959265,0.076721913459373,0.271,0.202,1,0
"Pmpcb",0.0295896222568158,-0.265937520792464,0.219,0.28,1,0
"Otulin",0.0297372634404635,0.120382787716968,0.168,0.115,1,0
"Tmem168",0.0297779428704196,0.058291224127897,0.148,0.096,1,0
"Spg11",0.0297958572364541,0.0643651182673765,0.065,0.033,1,0
"Sympk",0.0297972668498555,0.0561333368663213,0.161,0.107,1,0
"Iqgap2",0.0298010179954877,-0.165306564504235,0.084,0.143,1,0
"Phc3",0.0298645524428321,0.123051662349922,0.168,0.116,1,0
"Sarnp",0.0299632416488419,0.0584104360609815,0.348,0.261,1,0
"Nfyc",0.0299695343959643,0.0756866928623043,0.168,0.114,1,0
"Ptgds",0.0300282388152902,0.0508634795811776,0.09,0.051,1,0
"Dnajc18",0.0301470031726063,0.0529581369374773,0.11,0.066,1,0
"Psma1",0.0302727241925282,0.0617276464267128,0.465,0.364,1,0
"Dynll2",0.0303678888439807,-0.246449492241739,0.387,0.432,1,0
"Bop1",0.03041329575627,0.0507880383762336,0.103,0.061,1,0
"Setx",0.0304133364114759,0.0743760035724465,0.116,0.072,1,0
"Col4a5",0.0304193873029473,0.0580413410464583,0.123,0.076,1,0
"Cyp4a12a",0.0304884377834917,-0.135520824781877,0.013,0.051,1,0
"Dym",0.0305137849670426,0.0537556438669668,0.097,0.056,1,0
"Hexb",0.030601662407564,-0.357246851215764,0.232,0.293,1,0
"Ltb",0.0306169393851315,0.153798423195082,0.071,0.038,1,0
"Mthfsd",0.0307247735544321,0.110402222362047,0.129,0.083,1,0
"Palm3",0.0307425383182104,-0.08995745358365,0.013,0.051,1,0

"C4b",0.0307747499382363,-0.0910699501959525,0.013,0.051,1,0
"Rreb1",0.0308037347064693,0.0572587339175728,0.123,0.076,1,0
"Ndufa10",0.030874315357411,-0.259828065918713,0.535,0.54,1,0
"Gcc2",0.0308990845861257,0.055572058386073,0.258,0.185,1,0
"Camsap2",0.0309372335324721,0.0800329873944592,0.103,0.062,1,0
"Sgk3",0.0310844817317954,0.0697473102642382,0.11,0.067,1,0
"Cope",0.0311290774030682,0.0903651787641538,0.49,0.375,1,0
"Crnkl1",0.0311391415767061,0.116963759142517,0.11,0.068,1,0
"Dclre1a",0.0311938939644566,0.0671346308034929,0.065,0.033,1,0
"Bcl7c",0.0312955922918528,0.105148119147726,0.258,0.194,1,0
"Taf13",0.0313070472092296,0.0743335955927587,0.168,0.114,1,0
"Slc15a4",0.0313152436240377,0.0689525374270936,0.077,0.043,1,0
"Ramp2",0.0313449313202279,0.0849718240610142,0.103,0.062,1,0
"Gid4",0.0314375363599391,0.0533053414984415,0.103,0.061,1,0
"Edem2",0.0316140452584431,0.0676635609942842,0.142,0.092,1,0
"Mettl17",0.0316261851804766,0.055445689630015,0.065,0.033,1,0
"Osbpl1a",0.031633205560034,0.060514464927258,0.213,0.149,1,0
"Dgkz",0.0316478471835916,0.091775269918591,0.135,0.087,1,0
"Tmem167b",0.0316823286928474,0.0593281769524613,0.129,0.082,1,0
"Tsta3",0.0317063450294126,0.0515725802906074,0.148,0.098,1,0
"Ap1s3",0.031781683291983,0.0594529248674134,0.084,0.047,1,0
"Wdr75",0.0319929509929028,0.0685771090042073,0.077,0.043,1,0
"Copg1",0.0320534739350006,0.068929079924324,0.187,0.13,1,0
"Glb1l",0.0323036290336708,-0.141088016522015,0.084,0.144,1,0
"Pfn2",0.0323834950211981,0.0707964650660826,0.239,0.173,1,0
"Srsf4",0.0323969812497789,0.0810630298084373,0.148,0.098,1,0
"Gtf3c4",0.0324623721787673,0.064153818322701,0.071,0.038,1,0
"Txndc15",0.0325443605368933,0.0539341369320387,0.31,0.23,1,0
"Zbtb18",0.0326604973116748,0.0783738288140467,0.103,0.062,1,0
"Dtd2",0.0326816802566886,0.0558367611615881,0.155,0.103,1,0
"Rad54l2",0.0327753664698952,0.0716357693304836,0.097,0.057,1,0
"Sos1",0.0327763309493217,0.0875126059380297,0.11,0.068,1,0
"Pgpep1",0.0328447270962552,-0.235859684951599,0.232,0.297,1,0
"Rps27l",0.0328976459521697,-0.132504052441468,0.839,0.844,1,0
"Skiv2l2",0.0329008903027996,0.11332529810661,0.123,0.079,1,0
"Gm21887",0.032917055464657,0.0564677130978124,0.071,0.038,1,0
"Sgk2",0.0330471680372564,-0.206896222378267,0.097,0.154,1,0
"Pole4",0.0331445838572384,0.107750067829855,0.258,0.194,1,0
"Gab1",0.0333456043614184,0.0689824647181514,0.084,0.047,1,0
"Rps27rt",0.0334342531307478,0.0637123927594532,0.632,0.496,1,0
"Agl",0.033634004998484,0.0807500525542337,0.168,0.114,1,0
"Lmo2",0.0336790625660879,0.0903146077243278,0.058,0.029,1,0
"Sv2a",0.0338401316966375,0.0510351235735328,0.09,0.052,1,0
"Zfp395",0.0338539220792956,0.0587648855250254,0.103,0.062,1,0
"Trem2",0.033864705541003,0.0540587746355412,0.052,0.025,1,0
"Impa2",0.0338812069889946,0.0902726168986411,0.084,0.048,1,0
"Snn",0.0339328953819204,-0.122141162118447,0.058,0.112,1,0
"Slc10a3",0.0340110812316616,0.0928347192207641,0.052,0.025,1,0
"Echs1",0.0340196403458039,-0.271136961190055,0.361,0.405,1,0
"Csde1",0.0342061637463593,0.109396532697034,0.516,0.425,1,0

"Ormdl2",0.0343571466740398,0.0571913497702456,0.297,0.223,1,0
"Pwwp2b",0.034371176109742З,0.0574558055454912,0.052,0.025,1,0
"Elp2",0.0344742416252786,0.0766088689106825,0.116,0.073,1,0
"Mbtps2",0.0344943585452218,0.0661236995909435,0.097,0.058,1,0
"Lbh",0.0346463477792372,0.0897874782220307,0.065,0.034,1,0
"G6pc3",0.0346597001416986,0.0723826504999356,0.206,0.148,1,0
"Sgf29",0.0346792162413868,0.0507124397316603,0.09,0.053,1,0
"Ace2",0.0346977675544068,-0.11558866266623,0.032,0.077,1,0
"Prpf40b",0.0347624075869682,0.0729937029007813,0.084,0.048,1,0
"Gspt1",0.0347923889581155,0.073343146661295В,0.284,0.216,1,0
"Tmem106b",0.0348127606872029,0.058750256857361,0.426,0.331,1,0
"Rbsn",0.0348738608233513,0.0615881753278549,0.077,0.043,1,0
"Arhgef10",0.0349052052006239,0.0505098867066181,0.052,0.025,1,0
"Fam76a",0.0349146067858833,0.0652615866404018,0.155,0.104,1,0
"Tnks2",0.0350228128346615,0.0895301274730026,0.258,0.193,1,0
"Atp6v1a",0.0351086912420087,-0.298779337867258,0.574,0.558,1,0
"Igsf5",0.0351511616112249,0.0578604599607911,0.245,0.178,1,0
"Alg11",0.035214816244726,0.0565730590844665,0.077,0.043,1,0
"Pias4",0.0353310286194367,0.094513480051592,0.129,0.083,1,0
"Iars2",0.0354083154822663,0.060027967618906,0.174,0.12,1,0
"Fbxo46",0.0354221496485414,0.0518680023252296,0.058,0.029,1,0
"Pafah1b2",0.0354337295163522,0.0836212938448052,0.226,0.166,1,0
"Naaa",0.0354555030482593,0.0832872708172708,0.103,0.063,1,0
"Rbck1",0.0355196774509756,0.119878336158989,0.161,0.111,1,0
"Vars",0.0355342042047845,0.0785357007312629,0.142,0.094,1,0
"Ifi30",0.0357253825908985,0.10686938919005,0.252,0.191,1,0
"Mrpl28",0.0358172871165231,-0.18612800272808,0.29,0.36,1,0
"Gpank1",0.0361025910336174,0.0744296757487429,0.077,0.043,1,0
"Fam219b",0.0361357284417662,0.0679734496856206,0.065,0.034,1,0
"Cdc42ep2",0.0361837297546606,0.0678300882451983,0.071,0.039,1,0
"Rce1",0.0362005548343945,0.0615594285376109,0.155,0.103,1,0
"Cnpy2",0.0362137560781182,0.0566033323486383,0.535,0.437,1,0
"Champ1",0.0362991770755889,0.057279590439172,0.071,0.038,1,0
"Dhodh",0.0364641833872587,0.0528570230994709,0.071,0.038,1,0
"Kdm4c",0.0364680617556078,0.0626417642540004,0.077,0.044,1,0
"Amt",0.0365436625606902,-0.175658727184744,0.103,0.165,1,0
"Ndel1",0.0365901653490609,0.0866462206828405,0.232,0.17,1,0
"Eif2s3x",0.036617854747049,0.0848805464066811,0.239,0.179,1,0
"Marveld2",0.0366336002773565,0.0653900044684565,0.084,0.048,1,0
"Rrp7a",0.0368448481864989,0.0655541754751727,0.174,0.12,1,0
"C330007P06Rik",0.0370577156742071,0.0618169419989252,0.168,0.115,1,0
"Ppp1r2",0.0370833996528985,0.0524570239433789,0.31,0.234,1,0
"Gm17634",0.0371817173451529,-0.0933778891329514,0.019,0.059,1,0
"Ogfod2",0.0372324569299091,0.0598339408218846,0.161,0.108,1,0
"Parp12",0.0372564267470275,0.0690067884731467,0.077,0.044,1,0
"Elof1",0.0372809150403564,0.076094617669721,0.387,0.306,1,0
"Synj1",0.037328012804181,0.0804904688595917,0.123,0.079,1,0
"Cnpy3",0.0373774461193569,0.0670834573739385,0.142,0.094,1,0
"Eef2",0.0374360594753994,0.10682119002034,0.903,0.792,1,0
"Atg4d",0.0374384742320122,0.0593504118305189,0.181,0.123,1,0

"Bsdc1",0.0374607161681402,0.0774188477630421,0.194,0.138,1,0
"Clybl",0.0375001415748868,-0.217778175394858,0.155,0.216,1,0
"Clns1a",0.0378517615079884,0.0709704791822689,0.155,0.105,1,0
"Mtfr1",0.0378837666045342,-0.189331962626187,0.135,0.198,1,0
"Ccdc6",0.0380468384382965,-0.158806259773658,0.097,0.156,1,0
"Uck2",0.0381023154163658,0.0575339997314771,0.058,0.03,1,0
"Pde7a",0.0381335836877543,0.0870132761925259,0.135,0.089,1,0
"C2cd2l",0.0383402440850582,-0.12574082968701,0.045,0.093,1,0
"Epb41l4a",0.0383972009560773,0.0509373716533297,0.065,0.034,1,0
"Sh3bp5",0.0384057054072025,-0.131407159806111,0.058,0.109,1,0
"Tdg",0.0384784477069381,0.0615596387715165,0.116,0.074,1,0
"Zbed3",0.0385575607091365,0.0804085619072118,0.155,0.105,1,0
"Mettl14",0.0386400487707716,0.0556101260465678,0.058,0.03,1,0
"Nckap5",0.0387041874992539,0.0694639355770683,0.097,0.058,1,0
"Snx4",0.038833640866322,0.0987582591485729,0.161,0.112,1,0
"Cdk8",0.0388461143487375,0.107235849185939,0.161,0.112,1,0
"Pts",0.0389101319923835,0.0700436517177325,0.406,0.314,1,0
"Chmp6",0.0389492766919994,-0.210130936312176,0.206,0.271,1,0
"Sclt1",0.0389604039715003,0.0767822869912355,0.103,0.063,1,0
"St6galnac2",0.0390048307492943,0.0929154055120283,0.297,0.227,1,0
"Myo9b",0.0392466314792904,0.0783915635693383,0.077,0.044,1,0
"Ttc39b",0.0392668351589582,0.0694465148274184,0.077,0.044,1,0
"Hist1h1c",0.0396005228296257,0.352598071456186,0.252,0.195,1,0
"Olfm1",0.0397431755367614,0.0746016559352847,0.084,0.049,1,0
"Trim33",0.039749713054209,0.0504860922227011,0.116,0.073,1,0
"Nars",0.0398375269952374,0.0916748935214171,0.342,0.268,1,0
"Zhx1",0.0398437393647295,0.0576421154122862,0.155,0.105,1,0
"Pelp1",0.0399629637819646,0.059154919070375,0.084,0.049,1,0
"Them4",0.0400466800247033,0.113567010804522,0.142,0.094,1,0
"Arap1",0.0401577696870221,0.0675521027424614,0.077,0.044,1,0
"Rassf4",0.040269039540876,0.0731285290524628,0.052,0.026,1,0
"Cdk7",0.0402862239450865,0.0595200906496053,0.11,0.069,1,0
"Hmbox1",0.0404540088875508,0.0936338660807913,0.116,0.074,1,0
"AA467197",0.0405291983350391,0.0582693122270368,0.052,0.026,1,0
"Fam53a",0.0405708173669337,0.0537927568639486,0.11,0.068,1,0
"Mynn",0.040579550027498,0.0704029970279084,0.116,0.074,1,0
"Paqr7",0.0405913782441388,-0.126613647743677,0.026,0.066,1,0
"Zdhhc8",0.0406263043197433,0.0638671035791599,0.065,0.035,1,0
"Prmt7",0.0408204029409322,0.0665768254132227,0.097,0.059,1,0
"Zfp260",0.0410097319937289,0.0693552662134281,0.155,0.106,1,0
"Tufm",0.0412314771091254,-0.209844459973525,0.161,0.222,1,0
"Tmem102",0.0413809499633124,0.0558784952528428,0.071,0.039,1,0
"Gnl3l",0.0414818795655056,0.0692620714775192,0.148,0.1,1,0
"0610012G03Rik",0.0417195446248074,-0.221448090503013,0.426,0.474,1,0
"Rfxap",0.0418124377195545,0.0792593861314024,0.11,0.069,1,0
"Arpp19",0.04190959158335,-0.202326452791489,0.787,0.732,1,0
"Vdac3",0.0423893521109485,0.0548749795606391,0.458,0.37,1,0
"Entpd6",0.0424360923943083,0.0538175961495853,0.071,0.039,1,0
"Syvn1",0.0424854963440573,0.063505234666349,0.11,0.069,1,0
"Susd6",0.0426369582900196,0.0554491692234564,0.213,0.153,1,0

"Dvl1",0.0427204030595397,0.0814660366785316,0.135,0.09,1,0
"Jak1",0.042803145048336,0.0805918732575688,0.335,0.259,1,0
"Psat1",0.0428192647538184,-0.128455696547291,0.052,0.1,1,0
"Cacfd1",0.0428274517204182,0.0987560994658948,0.174,0.125,1,0
"Ndor1",0.0428720805117689,0.050506997837019,0.058,0.03,1,0
"Polg",0.0429018829242659,0.0784582919900723,0.116,0.075,1,0
"Golga2",0.0429403115318782,0.050971036013128,0.123,0.079,1,0
"Emc1",0.0430024513597705,0.0517294008913344,0.148,0.099,1,0
"Fam171a1",0.0431069476861687,0.0617445222350468,0.116,0.074,1,0
"Slirp",0.043290920703521,-0.250619062157724,0.535,0.539,1,0
"Trappc3",0.0433355904291212,0.0878227248096955,0.258,0.198,1,0
"Mink1",0.0433421036856693,0.0576706812133337,0.168,0.116,1,0
"Prkdc",0.0433539146955991,0.0623898261389757,0.065,0.035,1,0
"Adcy6",0.0434494492171914,0.0577088524986876,0.155,0.105,1,0
"Prkra",0.043450823396834,0.0641849283532209,0.11,0.069,1,0
"Dmd",0.0435871273131508,0.0720287243155355,0.077,0.045,1,0
"Ascc2",0.0436341106773918,0.090625890250375,0.097,0.059,1,0
"Tmem230",0.043747177975424,0.0649578251860936,0.161,0.111,1,0
"Pnkp",0.0439285677423408,0.0508735592537907,0.103,0.064,1,0
"Akap11",0.044001562116883,0.0738744496607155,0.2,0.146,1,0
"Btg3",0.0440609628177055,0.0513780674506793,0.097,0.059,1,0
"Psma4",0.0441761726353426,0.0580866499689037,0.574,0.459,1,0
"Plekha5",0.0442348206272888,0.0505373208086974,0.123,0.079,1,0
"Actr1a",0.0443139905607369,0.0767960195840816,0.168,0.117,1,0
"Ninj1",0.0443630261950144,-0.243925659851583,0.419,0.459,1,0
"Atrn",0.0446937546384817,0.0887717274615348,0.129,0.085,1,0
"Ap3d1",0.044734892212251,0.0783858035518,0.168,0.118,1,0
"Rps6ka3",0.0449320626742196,0.10514749077415,0.161,0.114,1,0
"Ccdc58",0.0449674063960955,0.0576561907138116,0.219,0.16,1,0
"Rnf208",0.0451139238614014,0.0525515734920301,0.097,0.059,1,0
"Rbbp4",0.045332139524817,0.112359928082372,0.284,0.223,1,0
"Plscr2",0.0454209621499204,-0.180285363266815,0.626,0.618,1,0
"Aptx",0.04544658984629,0.0672485622794301,0.097,0.059,1,0
"Ddx19a",0.0455256974521007,0.0572821214472033,0.097,0.059,1,0
"Brap",0.0456924841605366,-0.124861690036969,0.103,0.166,1,0
"Atg12",0.0459244947109337,0.0701182305169453,0.155,0.107,1,0
"Abcb7",0.0461624155847489,-0.0966435072112248,0.058,0.109,1,0
"Atf6",0.0462013520777016,0.0684545199818793,0.123,0.08,1,0
"Lims2",0.0462593170501984,-0.0948904240870227,0.032,0.075,1,0
"Lpl",0.046274721613837,-0.389167431417404,0.252,0.298,1,0
"Tpt1",0.0463114169261723,-0.0793712705307885,0.948,0.944,1,0
"Blvra",0.0463198410201999,-0.190409045601396,0.129,0.188,1,0
"Sall1",0.0464920560547957,0.0751717617076909,0.116,0.075,1,0
"Alg13",0.0467325226872323,0.0514600719339003,0.052,0.026,1,0
"Usp1",0.0467531502307145,0.0942494907094384,0.135,0.092,1,0
"Strn",0.0467604078104884,0.0855912220217576,0.071,0.04,1,0
"Mvb12a",0.0468174083365794,0.0557357570242287,0.29,0.218,1,0
"Calm3",0.0469528136867194,-0.259733386182476,0.581,0.565,1,0
"Cgnl1",0.0469584665336715,0.0554288608182258,0.516,0.409,1,0
"Idh3b",0.047100103884354,-0.22532506631759,0.361,0.407,1,0

```
"Phf20",0.0471021350276084,0.075946325600398,0.129,0.086,1,0
"Rab35",0.0471656465016683,0.0919840679669305,0.148,0.102,1,0
"Ralgps1",0.047188046387045,0.0543877431168355,0.071,0.04,1,0
"Creld2",0.0472352776407575,0.0522752101601832,0.187,0.133,1,0
"Slc44a3",0.0472938410849285,0.0647940886264057,0.097,0.059,1,0
"Pou2f1",0.0475433158792874,0.0724138273716909,0.09,0.055,1,0
"Farsa",0.047563137825275,0.0673248572909902,0.129,0.085,1,0
"Foxk1",0.0475952739061837,0.074753069403116,0.09,0.055,1,0
"Rnf128",0.0477218501824897,0.055012193667736,0.381,0.299,1,0
"Stab1",0.0477857784989308,0.0831584056790109,0.058,0.031,1,0
"Tor1b",0.0478201546155708,0.0732694648820072,0.135,0.091,1,0
"Pcyox1",0.0478208570687719,0.0724501125231918,0.245,0.184,1,0
"Tec",0.0479809429920203,0.0602023342215867,0.058,0.03,1,0
"Cops2",0.0480555514591035,0.0575384109763816,0.219,0.159,1,0
"Smim10l1",0.0481130750667765,0.0812042175195561,0.394,0.315,1,0
"Uba5",0.048150479494723,0.0618313742497892,0.181,0.129,1,0
"1810043H04Rik",0.0481977415903725,-0.238345516587319,0.29,0.343,1,0
"Pdss1",0.0482196693244099,-0.0937366622010171,0.026,0.065,1,0
"Nme5",0.0485888449167289,0.0582297645552949,0.077,0.045,1,0
"Ms4a6d",0.0490078138802784,0.055382276927001 2,0.052,0.026,1,0
"Rnf14",0.0490131282709057,0.101376776062147,0.194,0.142,1,0
"H2-Ke6",0.0491970681485448,-0.23639103588677,0.258,0.313,1,0
"Upp2",0.0493850991499583,0.0658248526091512,0.09,0.055,1,0
"Dnajb1",0.0503261923488218,0.176573090136434,0.148,0.104,1,0
"Ehd1",0.0504024155566019,-0.195839948073594,0.135,0.192,1,0
"Imp3",0.050471471651067 9,0.0791479685601446,0.258,0.198,1,0
"Rras2",0.0508859051176864,0.0695825156531955,0.129,0.086,1,0
"Wtap",0.0509122531832728,0.0918640030297218,0.239,0.181,1,0
"Bmpr2",0.0510851596181472,0.115557785404364,0.181,0.132,1,0
"Arhgap10",0.05114624687842,-0.10447824961812,0.045,0.091,1,0
"Ypel2",0.0517192930411435,0.0918306314582225,0.155,0.108,1,0
"Hfe",0.0517228173779266,0.0580979298884197,0.123,0.08,1,0
"Slc5a1",0.0519368849414947,0.105987302420926,0.129,0.086,1,0
"Arhgap35",0.0521785300397062,0.0844388449265199,0.129,0.087,1,0
"Rilpl2",0.0522807012472551,-0.132396582632108,0.071,0.12,1,0
"Rnf11",0.0523305179176493,-0.199774568171595,0.303,0.359,1,0
"Rcor1",0.0523555181461065,0.0950928101165805,0.135,0.093,1,0
"Smcr8",0.0523909361725798,0.0646712256762689,0.065,0.036,1,0
"Cdhr5",0.052408434241348,-0.249570707839135,0.226,0.273,1,0
"Tmem70",0.0527673890643913,-0.192760113375058,0.155,0.213,1,0
"Fbxo7",0.0527849801753942,-0.14462563485603,0.097,0.151,1,0
"Acads",0.0527864033884689,-0.218820414627413,0.239,0.3,1,0
"Bid",0.0528522583532354,-0.152412043569432,0.071,0.12,1,0
"Cpeb3",0.0531833906950636,-0.134144691852056,0.045,0.087,1,0
"Ino80c",0.0531891282212614,0.0560171306701466,0.084,0.05,1,0
"Znhit2",0.0533394230777544,0.0698110057617433,0.135,0.091,1,0
"Gjb1",0.0536299636351231,-0.114962446405011,0.052,0.098,1,0
"Osgin1",0.0536579247196044,-0.101693357660472,0.019,0.054,1,0
"Fbxl14",0.0537299894800275,0.0564985399560526,0.084,0.05,1,0
"Map4k3",0.0537375814918958,0.0761322737154458,0.142,0.098,1,0
```

"Pcgf5",0.0537639328795436,0.0815041210062472,0.084,0.051,1,0
"Ppp2r5e",0.0538382557865574,0.0718283197629855,0.2,0.147,1,0
"Adgre1",0.0540008489911471,0.0651588257167245,0.058,0.031,1,0
"Anapc16",0.0540272093022961,0.0532809110132892,0.265,0.203,1,0
"Creb3",0.0541771878473518,0.0546483236404555,0.181,0.129,1,0
"Ermard",0.0543678653334549,0.0705678330928809,0.097,0.06,1,0
"Dhrs3",0.0547934192516253,-0.138328954213885,0.168,0.231,1,0
"Lyplal1",0.0548472219766456,-0.142384804825993,0.077,0.127,1,0
"Ammecr1l",0.0548845298593455,0.0793994171192795,0.181,0.13,1,0
"Igf2bp2",0.0548933862931203,0.0574313750468048,0.052,0.027,1,0
"Cep57",0.0549003649290829,0.0525684763831321,0.129,0.086,1,0
"Rnf170",0.054954585856447,0.0652183434613664,0.11,0.071,1,0
"Sidt2",0.0551668996275459,0.0517224154234985,0.168,0.118,1,0
"Pgd",0.0551763526344488,0.0661089962627829,0.219,0.163,1,0
"Tab2",0.0552237396378751,0.0718031807763985,0.213,0.16,1,0
"Plod2",0.0552625507677034,0.0635675706888938,0.123,0.081,1,0
"Vamp8",0.0557157089322235,-0.172287196995582,0.768,0.713,1,0
"Tfcp2",0.0558525914161855,0.0662053621184464,0.09,0.055,1,0
"Got2",0.0559354015198081,-0.22013017223342,0.335,0.381,1,0
"Fibp",0.0559778713903961,0.0661372664260249,0.174,0.124,1,0
"Cmtm6",0.0561698979087348,-0.180887454605105,0.213,0.279,1,0
"Emc8",0.0564055540807623,0.0742829895202033,0.181,0.13,1,0
"Nt5e",0.056436610198094,-0.0552012054712043,0.039,0.082,1,0
"Gigyf1",0.0565554149206991,0.105928181358784,0.155,0.109,1,0
"Rpl28",0.0567833058592393,0.083328633154137,0.955,0.881,1,0
"Plbd1",0.0575369141669893,0.0853091404106299,0.097,0.062,1,0
"Slc35e3",0.0578266783674471,-0.117430945181772,0.058,0.104,1,0
"Irf7",0.0578569867571219,0.0773292674911834,0.09,0.056,1,0
"Zdhhc9",0.0580107040571381,-0.107541788468752,0.045,0.088,1,0
"Nploc4",0.0580490052860284,0.0684315319588674,0.09,0.056,1,0
"Med6",0.0580872424365459,0.0761014793410403,0.084,0.051,1,0
"Tmed7",0.0582407447641362,0.0955436191334115,0.335,0.27,1,0
"Coprs",0.0584002382260438,-0.0812630404219251,0.058,0.105,1,0
"Kdr",0.0585212177116828,0.293133645234454,0.052,0.027,1,0
"Ubxn7",0.0585499686373463,0.0901760919777911,0.097,0.062,1,0
"Dhx29",0.0585540991581611,0.0628401953644106,0.097,0.06,1,0
"Zfp524",0.0588058811005395,0.0544847844153044,0.097,0.061,1,0
"Mmgt1",0.0591817686963919,-0.0957794302303147,0.052,0.097,1,0
"Dmtn",0.0594846619868885,-0.105313545232746,0.097,0.152,1,0
"Cep83",0.0597526867238776,0.0529486569141315,0.071,0.041,1,0
"Plod1",0.0597992348514138,0.0543323209125642,0.084,0.051,1,0
"Nol3",0.0599679438024763,0.0589634801694065,0.065,0.036,1,0
"Cnot3",0.0600621479029916,0.0588279108262253,0.123,0.081,1,0
"Clasrp",0.0601766115313155,0.0705157806473145,0.065,0.037,1,0
"Sec22b",0.0602067928091041,0.0734225105506355,0.206,0.153,1,0
"Kcne1",0.060209441906962,0.15649205461455,0.071,0.041,1,0
"Lars2",0.0603607369978192,0.0866006254742377,0.148,0.104,1,0
"Fam134b",0.0603906771125165,-0.154656075000709,0.252,0.314,1,0
"AI846148",0.0606343029472686,0.0717058859734339,0.052,0.027,1,0
"Atxn3",0.060638354678514,0.0552137020316048,0.071,0.041,1,0

"Arvcf",0.0610103348298032,-0.0602022135996928,0.026,0.063,1,0
"Birc5",0.0610744271139103,0.0695166777939689,0.052,0.027,1,0
"Ndst1",0.0615520215614136,0.0981605618188277,0.129,0.088,1,0
"Canx",0.0616583757660588,0.0527815535085332,0.729,0.61,1,0
"Usf3",0.0620185928170103,0.0671873323895867,0.116,0.077,1,0
"Mki67",0.0620502617767927,0.0525509482835107,0.052,0.027,1,0
"Asph",0.0621368626492791,0.0859254487514627,0.213,0.16,1,0
"Zfp821",0.0621921929985433,0.0589794795211632,0.065,0.037,1,0
"Mthfd2",0.0622056007117029,0.087757398566009,0.071,0.042,1,0
"Gm14964",0.0623098973096759,-0.0897438436736755,0.026,0.063,1,0
"Pcnxl3",0.062318780923278,0.0824430597489007,0.123,0.083,1,0
"Pcdh17",0.0624275527641601,-0.0871731328404529,0.032,0.071,1,0
"Rab8a",0.0624671247201173,0.0855675308995534,0.239,0.184,1,0
"Gtf2h1",0.0629669894836178,0.0632957634166281,0.135,0.093,1,0
"Alox5ap",0.0629771339014858,0.1458093590217,0.11,0.073,1,0
"Apoa2",0.0629960026544277,-0.101266763002169,0.026,0.061,1,0
"Vapa",0.0630746123379431,0.0567532051230546,0.484,0.39,1,0
"Wdr18",0.0632223902581701,-0.202186818090126,0.174,0.23,1,0
"Ap3s2",0.0635499548614809,0.0520094728075504,0.148,0.104,1,0
"Ercc6l2",0.0642063804778468,0.0537140763395123,0.077,0.046,1,0
"Ppfia1",0.0642397253412847,0.0647964754751235,0.155,0.109,1,0
"Fcgr2b",0.0642446776406779,-0.0786749022098019,0.071,0.121,1,0
"Ankib1",0.064298233235463,-0.132772103613431,0.103,0.157,1,0
"Grk6",0.0643724323700316,0.067282111380447,0.071,0.042,1,0
"Pank4",0.0644350158192492,0.0769991304105773,0.052,0.027,1,0
"Tmem79",0.064523263029424,0.059796617483708,0.052,0.027,1,0
"B3galnt1",0.0646442604892761,-0.100609213326443,0.026,0.061,1,0
"Snhg11",0.0646524649529076,-0.255433668844383,0.052,0.094,1,0
"Capn10",0.0649461795660329,0.0790213720218722,0.084,0.052,1,0
"Scamp1",0.0650478715138272,0.0519093862089117,0.161,0.114,1,0
"Ldlrap1",0.0651040948947434,0.0614235636145222,0.065,0.037,1,0
"4930453N24Rik",0.0654031457777246,0.0871526817721328,0.142,0.1,1,0
"Plvap",0.0657763701012216,0.0590456444067438,0.103,0.066,1,0
"2700097O09Rik",0.065788585402919,0.0559513213084618,0.103,0.067,1,0
"Fzd1",0.06580217781509,-0.112648646662095,0.045,0.086,1,0
"Gbp2",0.0661219225027432,0.0718560029672825,0.058,0.032,1,0
"Cc2d2a",0.0662322987885582,0.0879885280008613,0.065,0.037,1,0
"Ppcdc",0.066273665861241,0.0530610072100435,0.09,0.057,1,0
"Idh2",0.0663835450197632,-0.232796428556626,0.574,0.556,1,0
"Hdlbp",0.0667262706871842,0.0644725673625353,0.394,0.32,1,0
"Bmp4",0.0669654269410548,0.139245754298133,0.097,0.061,1,0
"Mzt2",0.0671904213481102,0.0934960842735709,0.123,0.083,1,0
"Erlin1",0.0672945188899669,-0.0920241957564983,0.071,0.12,1,0
"Ctsh",0.0674092247542517,0.120281667079316,0.439,0.371,1,0
"Parva",0.0674425435788202,0.088471118864629,0.219,0.168,1,0
"Gm7932",0.0675236549000009,0.0846043528662529,0.071,0.042,1,0
"Mtg2",0.0678073205885728,0.055374264791358,0.123,0.083,1,0
"Cdc34",0.0678123785164752,-0.149530713595491,0.123,0.178,1,0
"Dcp2",0.0678626242401433,0.0632256931500964,0.11,0.073,1,0
"Sat2",0.06790361314733,-0.0952004527853226,0.032,0.07,1,0

"Abcd4",0.0679738147723805,-0.0727659503033582,0.032,0.071,1,0
"Tmem26",0.0679827151473889,-0.0860500342280826,0.019,0.052,1,0
"Atg5",0.0683272701643252,0.0768198886603446,0.148,0.105,1,0
"Lmbr1",0.068348876173577,0.0901054181742567,0.071,0.042,1,0
"Rab8b",0.068420788833793,0.0547589736405044,0.065,0.037,1,0
"Ccdc91",0.0687706162178313,-0.105990591178655,0.065,0.111,1,0
"Idi1",0.068880328289745,-0.191231251296405,0.097,0.144,1,0
"Rnf44",0.0689620928555572,0.0713755387071171,0.194,0.145,1,0
"Acsl1",0.0689938031621573,-0.190635538829594,0.239,0.291,1,0
"Cmpk2",0.0690229393302293,0.087404033855417,0.052,0.027,1,0
"Ctla2a",0.069030654185092,0.121510079634586,0.077,0.047,1,0
"Pcgf3",0.0694579743301891,0.0538235205268948,0.129,0.088,1,0
"Txn2",0.070115497026593,-0.245485353219724,0.4,0.433,1,0
"Creb3l2",0.0702253372059525,0.077750747748292,0.194,0.145,1,0
"Sept9",0.0702406171295776,0.0547522647682835,0.123,0.083,1,0
"Ccdc127",0.0710168021813904,0.0754414338057277,0.142,0.101,1,0
"Slc6a18",0.0710982478734038,-0.137580400204696,0.065,0.109,1,0
"Atad2b",0.071360635203206,0.0582073799769192,0.077,0.047,1,0
"Ckap5",0.0713874666435992,0.0595773127744852,0.103,0.068,1,0
"Gm11808",0.0715909100847839,-0.179564425479142,0.529,0.558,1,0
"Med28",0.0716233077878427,-0.233530268712174,0.484,0.49,1,0
"Nol11",0.0716583844475852,0.0968693706169062,0.09,0.058,1,0
"Abhd8",0.0723605503489993,0.064121503277081,0.077,0.047,1,0
"Vkorc1l1",0.0724992781115434,0.0551647385612679,0.129,0.088,1,0
"Csnk2a2",0.0727771500609147,0.0627017743105768,0.052,0.028,1,0
"Pop7",0.072794238373707,0.0572044486465919,0.168,0.121,1,0
"Rundc1",0.073164966902482,0.0762159412452053,0.077,0.048,1,0
"Wdr36",0.0737116477026276,0.0585502501204416,0.09,0.057,1,0
"Zmiz2",0.0738266970388189,0.0575269254853626,0.161,0.115,1,0
"Morn2",0.0741694826483733,-0.174941892825258,0.2,0.255,1,0
"Mavs",0.0742087956276683,0.069737313900258,0.155,0.112,1,0
"Atpif1",0.0742348757675325,-0.0907340393283276,0.942,0.926,1,0
"Lamtor3",0.0744584078785439,0.077351692379411,0.245,0.19,1,0
"Oxgr1",0.0744751470014506,-0.100089226097952,0.019,0.051,1,0
"Pnkd",0.0744774658422184,-0.17065274868437,0.4,0.451,1,0
"Sucla2",0.0749020763845365,-0.204574844190996,0.303,0.355,1,0
"Nhs",0.0749296223604311,-0.0713465158009866,0.039,0.078,1,0
"Blmh",0.0749696477408171,0.0743223388684682,0.219,0.168,1,0
"BC037034",0.0754283926635381,0.0547955809157503,0.052,0.028,1,0
"Gm11992",0.0756284128305744,-0.0875412245389266,0.019,0.051,1,0
"Qars",0.0759079374360363,0.0518541780853937,0.187,0.139,1,0
"Mettl21a",0.0760936147429673,0.0556088499735326,0.052,0.028,1,0
"Rpl27",0.0762991160279155,0.116356064451892,0.884,0.814,1,0
"Tmem159",0.0764900420107417,0.0820903210220468,0.129,0.09,1,0
"Hirip3",0.0765395556312912,0.0714743789846224,0.058,0.033,1,0
"Cpd",0.0766822156050802,0.0505652602992387,0.232,0.175,1,0
"Zscan29",0.076743441896785,0.0611192770792133,0.097,0.063,1,0
"Sumf1",0.0768273034035779,0.0541911838489738,0.123,0.084,1,0
"Mlec",0.0768619594095177,-0.220461383875396,0.51,0.505,1,0
"Mapk6",0.07700340568171,0.0758042461700886,0.103,0.069,1,0

"Fam213b",0.0772189099162869,-0.139288389175878,0.077,0.124,1,0
"Aprt",0.0775171365640556,-0.204455720861703,0.329,0.373,1,0
"Otud4",0.0775637827038507,0.0723391541412916,0.116,0.08,1,0
"Ppil1",0.0776084493547667,0.0675069544256979,0.142,0.101,1,0
"Rapgef2",0.0777235295812636,0.0557078652777723,0.065,0.038,1,0
"St8sia6",0.0777729815800062,-0.0632117135166546,0.026,0.059,1,0
"Dtx3l",0.0778209890136874,0.0547419349539811,0.084,0.053,1,0
"Deptor",0.0786989158757838,0.109132172645298,0.226,0.174,1,0
"Hus1",0.0788228282928782,0.0599029174368741,0.077,0.047,1,0
"Sox12",0.0792502091484797,0.0520121633796949,0.071,0.043,1,0
"Trib2",0.0792507797267959,-0.0934597384346858,0.032,0.067,1,0
"Uri1",0.0797345273470009,0.0676171956044616,0.148,0.107,1,0
"Orai3",0.0802444381531666,0.0511139433465726,0.097,0.063,1,0
"Vwa9",0.0803747567630073,0.0508113825326821,0.084,0.053,1,0
"Cdip1",0.081532502058949,0.0623328829291332,0.129,0.09,1,0
"Rnf145",0.0816990536619847,0.0725755910911678,0.206,0.154,1,0
"Rps25",0.0818995920588824,0.0856949029869605,0.942,0.865,1,0
"Usf2",0.0819714320025677,0.0665801069364376,0.219,0.169,1,0
"Hebp1",0.0821087682752273,-0.226610904727174,0.245,0.292,1,0
"Wipf2",0.0822478606492083,0.0607272600167704,0.103,0.069,1,0
"Fbrsl1",0.082317654865875,0.0810803138377316,0.148,0.107,1,0
"Pigc",0.082566065557031,0.0762661953237585,0.084,0.053,1,0
"Pou6f1",0.0826896345698003,0.0844966716488944,0.065,0.038,1,0
"Cln8",0.0828816930995123,0.067211436488552,0.084,0.053,1,0
"Pdlim5",0.0832109014699528,0.0708580067931663,0.194,0.146,1,0
"Mpi",0.0833597493895772,-0.0705944372581925,0.032,0.068,1,0
"Iqsec2",0.0834086851248379,-0.078707065071728,0.039,0.076,1,0
"Cept1",0.0839323219644266,0.0548488800272689,0.168,0.122,1,0
"Sfxn2",0.0845184737903376,-0.124544506843155,0.077,0.121,1,0
"Erbb2ip",0.0848037503940951,0.0503558939706881,0.355,0.289,1,0
"Tspan3",0.0849808787065186,-0.193610754315982,0.413,0.46,1,0
"Dus2",0.0850383513037912,0.0521296488683264,0.058,0.033,1,0
"Epas1",0.0854662499287848,0.0597654595216665,0.103,0.069,1,0
"Hba-a2",0.0856515670137494,-1.55542951687502,0.097,0.063,1,0
"BC004004",0.0857602443380486,-0.242188960608252,0.348,0.375,1,0
"Plekha2",0.0857759010570786,0.0661651342025349,0.065,0.038,1,0
"Hook2",0.0858968840389519,-0.108780981022761,0.065,0.107,1,0
"Ppm1h",0.0861724834258535,0.087721266687677,0.303,0.244,1,0
"Asb9",0.0862965561910351,-0.158355994056694,0.09,0.134,1,0
"Irf2bp2",0.0864884610908415,0.0553360064395165,0.258,0.199,1,0
"D630045J12Rik",0.0867262604324881,-0.0585823901820526,0.084,0.132,1,0
"Fyb",0.0869812747803822,0.0701015631924667,0.097,0.064,1,0
"Mlycd",0.0870142319914566,-0.0949892821483528,0.052,0.092,1,0
"Lgals8",0.087120475197182,0.0556933980538363,0.187,0.14,1,0
"Pcyox1l",0.0873190973396807,0.0674692629757964,0.052,0.029,1,0
"Gc",0.0876323043187311,-0.149034695302529,0.045,0.081,1,0
"B3gnt7",0.0877380322407784,0.060619014900472,0.052,0.029,1,0
"Hbp1",0.0879520432503428,0.0664853396223634,0.219,0.171,1,0
"Rb1",0.0881034608132929,0.051488778251681,0.129,0.09,1,0
"Gabbr1",0.0882448951143225,0.093519833298195,0.071,0.043,1,0

"Dctpp1",0.0884379868804203,0.0551119062818979,0.284,0.218,1,0
"Chchd3",0.0888699020878474,-0.141968124877742,0.297,0.355,1,0
"Trp53i13",0.0889429189275477,0.0556458438352449,0.071,0.043,1,0
"Scyl1",0.0892808844002248,0.0695509623780578,0.135,0.096,1,0
"Zmat3",0.0892840591855398,0.0749876141173518,0.084,0.054,1,0
"Zfp462",0.0893475693344739,0.0705568589566005,0.052,0.029,1,0
"0610010F05Rik",0.0894355226589949,0.0587388315701314,0.077,0.049,1,0
"Sirt5",0.0894381078306043,0.0820253496106863,0.065,0.039,1,0
"Pddc1",0.0895428180106078,0.0588453844722619,0.129,0.091,1,0
"Ddrgk1",0.0895537591796436,-0.230580229662027,0.426,0.446,1,0
"Pkp4",0.089726746380231,0.0681018220432759,0.432,0.354,1,0
"Zfp691",0.0898486594207136,0.063796868104581,0.052,0.029,1,0
"Rgs6",0.0899248162796877,0.0651177452795694,0.09,0.059,1,0
"Acot1",0.0899332500546723,-0.135283538396336,0.058,0.097,1,0
"Lsm14b",0.0902362202815378,0.0879242525933635,0.129,0.092,1,0
"Srebf1",0.090254466896956,0.0631611486732177,0.103,0.069,1,0
"Dnttip1",0.0906654555977699,0.0501437071172178,0.123,0.085,1,0
"9530034E10Rik",0.0907288535159973,0.0529103263125656,0.058,0.034,1,0
"Hcls1",0.0910614380519705,0.0538472011697031,0.071,0.043,1,0
"Thrb",0.091153005177448,-0.0700834986615641,0.026,0.058,1,0
"Tomm20",0.0913627150830924,-0.196960567740991,0.716,0.636,1,0
"Crk",0.0914297029012993,0.0615559784137374,0.29,0.234,1,0
"Ap3b1",0.0914533288877577,0.0616674499212355,0.161,0.12,1,0
"Ppp1r13b",0.0915221880291944,0.0620894726043964,0.058,0.034,1,0
"Josd2",0.0916472997725308,0.0527943106036374,0.181,0.135,1,0
"Fubp3",0.0916886690489564,0.0508916931030408,0.116,0.08,1,0
"Zfp426",0.092196057244626,0.0570913141624659,0.058,0.034,1,0
"Zfp442",0.0926796514369602,0.0534623443468323,0.052,0.029,1,0
"Usp48",0.0927961312169689,0.0564389424064317,0.129,0.091,1,0
"Cnot6",0.0929722865114918,0.0815517792540694,0.239,0.189,1,0
"Tmem201",0.0929940253377675,0.0923505718624533,0.065,0.039,1,0
"Xrcc5",0.0933578490193433,0.0536267725353533,0.142,0.101,1,0
"Tnks1bp1",0.0937095384057406,0.0505892727692078,0.071,0.044,1,0
"Zbtb17",0.0944569612015789,0.0525257718610545,0.071,0.044,1,0
"Ptch1",0.0944704715440209,0.0910191910982802,0.097,0.065,1,0
"Ntn1",0.0948371690958709,-0.0861745829704992,0.032,0.065,1,0
"Arhgap27os2",0.0951826233733586,-0.0922501483968103,0.026,0.056,1,0
"Slc6a6",0.095372215728482,-0.097213849581361,0.284,0.343,1,0
"Dip2c",0.0955429259480602,0.0633825954775994,0.077,0.049,1,0
"Tlr3",0.0962321041481195,0.053358855818222,0.058,0.034,1,0
"Rps7",0.096305706556074,0.123188094166075,0.89,0.81,1,0
"Pvrl1",0.0963462917687927,-0.083086293024973,0.032,0.065,1,0
"Ush1c",0.0964654986172988,-0.110998752228554,0.084,0.129,1,0
"Dld",0.096644251567782,-0.177051945434639,0.29,0.339,1,0
"Mgat1",0.096928052449024,0.117224077105725,0.2,0.157,1,0
"Ptger3",0.097361771960887,0.0711618086820333,0.123,0.086,1,0
"Alkbh7",0.0974618565397986,0.0614210975084469,0.187,0.142,1,0
"Tceal1",0.0979851696609911,0.0843730821430068,0.077,0.05,1,0
"D730003I15Rik",0.0981453662057296,0.0572085032374997,0.071,0.044,1,0
"Cplx2",0.098216453869618,0.0670737259681197,0.065,0.039,1,0

"Arhgap24",0.0988936720673037,0.0621713717078207,0.368,0.29,1,0
"Galm",0.0990072939869924,-0.166826895122882,0.11,0.154,1,0
"Pigs",0.0990873113655258,0.051041758793612,0.097,0.065,1,0
"Nubpl",0.0991980905071427,-0.0893177460971295,0.039,0.073,1,0
"Btd",0.0995731952170914,-0.144442670208143,0.077,0.117,1,0
"Fam213a",0.0995755672808784,-0.201445727165728,0.323,0.364,1,0
"Ddx26b",0.1008431003606,0.0556879632580857,0.077,0.049,1,0
"Ahsa2",0.100889359899265,0.0738749464705237,0.11,0.076,1,0
"Ankrd54",0.101719559855382,0.0517011758241279,0.097,0.065,1,0
"Nup153",0.101824934999824,0.0819786122136361,0.103,0.072,1,0
"Retsat",0.102045144482053,-0.160275672994656,0.135,0.183,1,0
"Timp1",0.102114804080359,0.0518660985971692,0.077,0.049,1,0
"Hsd17b10",0.102331574944903,-0.248873898776594,0.381,0.403,1,0
"Glb1",0.102463823662351,-0.148762100566637,0.123,0.169,1,0
"2610528J11Rik",0.103022704593382,-0.180759345272927,0.206,0.253,1,0
"Tmem55a",0.103053101189123,0.0598466534901083,0.168,0.126,1,0
"Bloc1s1",0.103280746426701,-0.202830142352041,0.323,0.364,1,0
"Mocs2",0.103680182426259,-0.24517370683862,0.297,0.332,1,0
"Gramd1a",0.103684803644326,0.0546494924282512,0.09,0.06,1,0
"Tbc1d22b",0.103685899235056,-0.0685131099840841,0.026,0.056,1,0
"Mageh1",0.103763899211161,0.0580811025624221,0.09,0.06,1,0
"9130401M01Rik",0.103901707732508,0.0859532245890883,0.097,0.065,1,0
"Sfxn5",0.103939796897587,-0.0837317646358503,0.032,0.064,1,0
"Coasy",0.104156604938695,-0.165732093912139,0.168,0.215,1,0
"Tars",0.104381121044232,0.052930779416406,0.11,0.076,1,0
"Mrpl51",0.105262283920328,-0.215997402549693,0.516,0.505,1,0
"Slc22a23",0.105629285487065,-0.109565503806017,0.065,0.104,1,0
"Cwc27",0.105688515414861,0.0506908883618091,0.09,0.06,1,0
"Mum1",0.106440856844719,0.0575639126202383,0.09,0.06,1,0
"Eaf2",0.106762089223172,-0.11101136239742,0.058,0.096,1,0
"Slc37a3",0.1073465056956,0.0504380943394994,0.071,0.045,1,0
"Prrc1",0.107603214690031,0.080928436656114,0.103,0.072,1,0
"Slc25a48",0.108116954064416,-0.0921495409947456,0.026,0.055,1,0
"Hook1",0.109428378106522,0.061197530696746,0.194,0.149,1,0
"Znfx1",0.11022637682342,0.0801789234900442,0.135,0.099,1,0
"Casc4",0.110311263553299,0.0606954601594399,0.155,0.115,1,0
"Gak",0.110574781973725,-0.103369326915446,0.097,0.142,1,0
"Slc3a2",0.110943777452775,-0.237946372891298,0.387,0.417,1,0
"Rap1gap2",0.111054213783512,0.0511908150750819,0.09,0.06,1,0
"Zfp386",0.111277941745055,0.0555053093083459,0.097,0.066,1,0
"Galnt1",0.111582010203647,-0.179747157630024,0.206,0.252,1,0
"Slc25a42",0.111644869827445,-0.0658080984902282,0.026,0.055,1,0
"Cox14",0.111862797067288,-0.20174905774514,0.6,0.587,1,0
"Acyp2",0.11188801937912,-0.128984052474464,0.123,0.169,1,0
"Chp1",0.112403837315598,0.0673113844690884,0.271,0.217,1,0
"Txlng",0.112617930850153,0.0601891218434943,0.084,0.056,1,0
"Uso1",0.113149485263491,0.0566211282519414,0.194,0.15,1,0
"Mtfp1",0.113231344550561,-0.100652583562476,0.045,0.079,1,0
"Hes6",0.113323728342689,-0.170204742024907,0.142,0.186,1,0
"Tgm2",0.113450558351807,-0.104162757933605,0.213,0.256,1,0

"Cd53",0.114275028173653,0.182878015949162,0.077,0.051,1,0
"Zfp512b",0.114548385055752,0.0976782725803072,0.077,0.05,1,0
"Plekhg3",0.115273417918995,-0.107290335075341,0.09,0.133,1,0
"Slc39a10",0.115366141463151,0.0849391659374447,0.116,0.084,1,0
"Man1a",0.115431942348348,-0.163300212382642,0.187,0.235,1,0
"Sqrdl",0.116180569067249,-0.166532252827174,0.155,0.201,1,0
"Herpud1",0.116325828240196,-0.182888014166749,0.284,0.322,1,0
"Tmem132a",0.116708995148755,0.093634402048999,0.077,0.051,1,0
"Lipe",0.116872922218411,0.0559639152419334,0.065,0.04,1,0
"Nubp1",0.117022158382242,-0.0897040895671573,0.084,0.126,1,0
"Rpa1",0.117326726061527,0.0576463382878562,0.097,0.066,1,0
"Gabarap",0.117423591754278,-0.133393601777767,0.852,0.768,1,0
"Mrpl57",0.117729502550417,-0.220588402423798,0.348,0.378,1,0
"Fbxo8",0.117760185777631,-0.134089895247151,0.103,0.146,1,0
"Ccnt1",0.118138358245875,0.0580367662798082,0.142,0.105,1,0
"Dexi",0.118199525526769,0.0550368935731195,0.09,0.061,1,0
"Slc25a16",0.11860648483403,-0.162424314050165,0.194,0.24,1,0
"Ankfy1",0.118812301795887,0.0500522204456687,0.116,0.082,1,0
"Sirpa",0.11919976760849,-0.0967911800417158,0.084,0.126,1,0
"Pisd",0.119529991894384,0.054040669579707,0.097,0.066,1,0
"Gas6",0.119844538734379,-0.142546371686157,0.497,0.516,1,0
"Clec4n",0.120214018025333,0.0815365231356001,0.052,0.03,1,0
"Lamtor2",0.120985933714843,-0.19365358059454,0.6,0.58,1,0
"Uqcrc2",0.120999437095768,-0.176414037297902,0.548,0.543,1,0
"Gng12",0.12116850688784,0.105150365967804,0.561,0.491,1,0
"Dot1l",0.121891027488397,0.0511933144242706,0.103,0.072,1,0
"Ets2",0.121988833482277,0.0538425978738747,0.123,0.088,1,0
"Arpc1a",0.122039159060217,0.0598012691132633,0.348,0.29,1,0
"Nr4a1",0.122063592545392,0.126678511638106,0.052,0.031,1,0
"Gimap1",0.122268282229572,0.0917016790763475,0.065,0.04,1,0
"Slc25a13",0.122706975890999,-0.135605009793338,0.148,0.196,1,0
"Pdp2",0.122710552261068,-0.0938863523323199,0.039,0.07,1,0
"Cand1",0.123710650957683,0.117542892489104,0.155,0.117,1,0
"Hacd2",0.123761655269486,-0.158923146437156,0.181,0.229,1,0
"Ttc23",0.1242672079474,0.0670722268900289,0.065,0.041,1,0
"1500026H17Rik",0.124287093732707,-0.103745085480941,0.052,0.085,1,0
"Parm1",0.124919245397393,0.10512093517757,0.071,0.046,1,0
"Gm561",0.12528803231124,-0.151893685853266,0.219,0.265,1,0
"Hnmt",0.12556476768932,0.0692838602138463,0.077,0.051,1,0
"AI464131",0.12578487527173,-0.076880234142525,0.026,0.053,1,0
"Ncoa7",0.125916208996046,-0.154997698384807,0.258,0.301,1,0
"Slc35b4",0.126218087119807,0.0556808284735393,0.187,0.143,1,0
"Hivep1",0.126480569740647,0.0618146697422261,0.084,0.057,1,0
"Med1",0.12679584691851,0.062001989974164,0.155,0.117,1,0
"Cpsf2",0.126956440166949,0.0614169735296081,0.129,0.095,1,0
"Macrod1",0.12732700498456,-0.0892067362746971,0.077,0.117,1,0
"Wdr35",0.127853345316492,0.0625620948315743,0.084,0.056,1,0
"Ebag9",0.128514548902732,0.0505942433828691,0.142,0.105,1,0
"Fars2",0.128553112290777,-0.130193992958832,0.103,0.144,1,0
"Cyp51",0.128681798043997,-0.134288533189105,0.103,0.144,1,0

"Rab3ip",0.128756031997232,0.0805116923244387,0.51,0.427,1,0
"Fau",0.129414669839242,0.0761088128326719,0.942,0.901,1,0
"Fkbp2",0.129681491171988,-0.172416390315814,0.561,0.556,1,0
"Zfp518a",0.129789247057341,0.0570499564140726,0.071,0.046,1,0
"Rps26",0.129792130900401,0.0500974112168411,0.897,0.816,1,0
"Nmrk1",0.130301103648755,-0.108397311902125,0.161,0.209,1,0
"Mrps34",0.130317522804805,-0.239151206211901,0.284,0.313,1,0
"Ogdh",0.130959375791567,-0.159882546628257,0.419,0.46,1,0
"Ptgr2",0.132415877450335,-0.146261889340411,0.181,0.228,1,0
"Aph1c",0.132639516831051,-0.0841743298632365,0.071,0.109,1,0
"Map3k4",0.133631815139415,0.0533835774770162,0.123,0.09,1,0
"Plekhb2",0.133825922722825,-0.201187916506759,0.277,0.31,1,0
"Tmem192",0.134323813439357,-0.111391234781818,0.09,0.13,1,0
"Mina",0.134800642394594,0.0623863767876025,0.09,0.062,1,0
"Smek2",0.136292750009194,0.0687843003975434,0.206,0.165,1,0
"Klf15",0.136879257388808,-0.112818286606763,0.045,0.076,1,0
"Dennd5b",0.137008380138929,0.0610995951794885,0.168,0.13,1,0
"Gatsl2",0.137197923315828,-0.102724908970936,0.084,0.122,1,0
"Tmem2",0.137385815053332,0.0702583945468211,0.097,0.068,1,0
"Epn2",0.137394249122182,0.0857681412626048,0.09,0.063,1,0
"Aatf",0.137476869603193,-0.147188098612878,0.116,0.156,1,0
"Tcaim",0.137687016831326,-0.0829261211615795,0.032,0.06,1,0
"Amdhd2",0.138157297643988,-0.123996815406356,0.142,0.187,1,0
"Fam46a",0.13834925938748,0.0518248236846984,0.052,0.031,1,0
"As3mt",0.138446865748008,-0.138014668232507,0.135,0.18,1,0
"Psme4",0.13880521541354,-0.151584698831244,0.206,0.252,1,0
"Akt2",0.13888371873615,0.11829728055011,0.161,0.124,1,0
"Tpk1",0.140046397114787,-0.105819052472825,0.077,0.115,1,0
"Gzf1",0.140155444091938,0.0724707006410125,0.097,0.068,1,0
"Faah",0.140650046159157,0.0650759052159786,0.155,0.117,1,0
"Fnbp1",0.140896179073234,0.0509283612924853,0.11,0.079,1,0
"Vps39",0.141122489682098,0.0514167141784745,0.052,0.031,1,0
"Tceb2",0.141179897032441,0.0568502672519537,0.955,0.814,1,0
"Phlda2",0.141398909113291,-0.107977730467457,0.039,0.067,1,0
"Fastkd1",0.141932954082258,0.0577856051597556,0.09,0.062,1,0
"Plat",0.142466191613212,0.109680235778187,0.077,0.052,1,0
"Insig1",0.143595790369094,-0.115909504712178,0.071,0.106,1,0
"Tmx1",0.143954701173306,0.0646239533681501,0.213,0.169,1,0
"Srp68",0.144277346578027,0.0576266541862928,0.135,0.101,1,0
"1700037C18Rik",0.14433555952211,0.0671530094604089,0.097,0.069,1,0
"Def8",0.145641744817085,-0.105196719411303,0.071,0.107,1,0
"Zfp639",0.147401882713337,0.0687355180372231,0.11,0.08,1,0
"H6pd",0.147903980691623,-0.118107832997726,0.135,0.179,1,0
"Tnfsf12",0.148219507563496,0.063026521647018,0.071,0.047,1,0
"Ndufaf1",0.148597949436497,-0.0752640021007497,0.058,0.092,1,0
"Ubr7",0.148770269472733,0.0555991708138964,0.071,0.047,1,0
"Pctp",0.1496956165483,-0.0627895342836781,0.032,0.06,1,0
"Edem3",0.150617285050035,0.0522310374018213,0.11,0.079,1,0
"Flrt1",0.150694787947917,0.0919680168165605,0.213,0.171,1,0
"Usp33",0.151417060152669,0.0982949540092553,0.142,0.109,1,0

"Atp6v1c2",0.152557036057379,-0.172107547210228,0.039,0.067,1,0
"Galnt3",0.152844024954167,-0.145836649568405,0.116,0.154,1,0
"1700021F05Rik",0.153478287707856,-0.120792623187722,0.148,0.189,1,0
"Epc2",0.15392056367932,0.0549828980729778,0.09,0.064,1,0
"Tpmt",0.154181634477883,-0.142942417965594,0.077,0.112,1,0
"Crebl2",0.154672551159417,0.0719438872444435,0.103,0.074,1,0
"Rnf168",0.154924360083766,0.0781661076265955,0.071,0.048,1,0
"Galt",0.156037177599913,0.0617827487841671,0.181,0.139,1,0
"Bex4",0.157613613795713,-0.101484187067452,0.09,0.128,1,0
"Gde1",0.158132758594573,-0.194108133805614,0.29,0.321,1,0
"Ppp1r35",0.1586034030714,0.0750790548841825,0.168,0.132,1,0
"Ak3",0.15904490698095,-0.194324225908785,0.29,0.325,1,0
"Gstk1",0.15905291753966,-0.116860110079395,0.071,0.104,1,0
"Psph",0.159659760200148,-0.113661876450212,0.071,0.105,1,0
"Tcta",0.159742193402358,0.0653552790020528,0.103,0.075,1,0
"Setd8",0.159887588176601,-0.149641714522751,0.181,0.222,1,0
"Tmem33",0.161831489453155,-0.159101575782283,0.297,0.335,1,0
"Ranbp3l",0.161980059567492,0.127925929527103,0.071,0.048,1,0
"Atraid",0.162602551856692,-0.210911598647169,0.394,0.415,1,0
"Rhbg",0.162628175443399,-0.21789089649674,0.052,0.081,1,0
"Rgs10",0.162764879819541,0.063081630393557,0.084,0.058,1,0
"Cmc4",0.162833800754104,0.0637334098373398,0.103,0.075,1,0
"Gpc4",0.163063858816891,-0.171975746390781,0.174,0.212,1,0
"Spsb4",0.163561500613408,-0.105070785663338,0.103,0.142,1,0
"Dgkd",0.163591261273693,0.0552708676985279,0.103,0.075,1,0
"Nipsnap1",0.163880367719597,-0.195238943578048,0.194,0.226,1,0
"Uaca",0.165049326566393,0.0578081933419879,0.09,0.063,1,0
"Klc4",0.166062075540921,-0.144591412166745,0.103,0.137,1,0
"Lman2l",0.167014573565497,0.0671694095933153,0.174,0.139,1,0
"Hepacam2",0.169717843942787,-0.119011080451275,0.032,0.057,1,0
"Notch2",0.169759842227354,0.0852668470532317,0.103,0.074,1,0
"Nop10",0.170712439875082,-0.13774411159881,0.748,0.704,1,0
"4930556M19Rik.1",0.170861133468838,0.0519792778064229,0.052,0.032,1,0
"Eva1a",0.170890146232118,-0.104675766298851,0.142,0.184,1,0
"Whamm",0.171697591912498,0.0544505516706996,0.071,0.048,1,0
"Rtca",0.174494548851632,-0.05609022659509,0.052,0.083,1,0
"Abhd17b",0.174958722327972,0.0738462277777074,0.135,0.105,1,0
"Renbp",0.175283159152232,-0.162468360256461,0.135,0.172,1,0
"Zfp36l2",0.175548476384747,0.106922153048486,0.206,0.169,1,0
"Klhl5",0.176016978858152,0.0543499004136758,0.071,0.048,1,0
"Atp2a2",0.176966511459155,-0.152761031817939,0.361,0.398,1,0
"Ctsz",0.177225646298207,-0.184385143018939,0.4,0.417,1,0
"Ulk1",0.177534144326953,0.115759158253667,0.116,0.087,1,0
"Cd68",0.177607032640699,0.0668644930677262,0.077,0.054,1,0
"Urod",0.179112073316696,-0.125053013789316,0.129,0.168,1,0
"Fnip2",0.180361204089282,-0.0865652753758997,0.045,0.072,1,0
"Clic5",0.18097028037191,-0.0904564305212365,0.058,0.088,1,0
"Sdhaf1",0.182113677458819,-0.132040829867945,0.123,0.158,1,0
"Ak2",0.183193859222371,-0.23902376699032,0.335,0.344,1,0
"Pi4ka",0.183244299529138,-0.0914426922410306,0.142,0.182,1,0

"Ube4b",0.183704424763703,-0.0854455421238224,0.09,0.127,1,0
"Acsf3",0.183959091630673,-0.0819999033069186,0.052,0.081,1,0
"Snrnp35",0.184650134032858,0.0628462280702584,0.065,0.043,1,0
"Smim13",0.186713137047407,0.0714186581262869,0.058,0.038,1,0
"Bik",0.187352031706086,0.0680159447404153,0.116,0.088,1,0
"Mtmr10",0.188985085720935,0.0524311347663195,0.071,0.049,1,0
"Gadd45gip1",0.18924459774269,-0.196131743455329,0.31,0.335,1,0
"Dock9",0.189368218775423,-0.0694480752597924,0.058,0.088,1,0
"Psen2",0.189504271438095,0.0615482479940683,0.084,0.06,1,0
"Bsg",0.190480698143477,-0.107546433153511,0.806,0.759,1,0
"Fech",0.191018632885261,-0.126284069518214,0.142,0.182,1,0
"Fam136a",0.191035889728442,-0.120954290113391,0.11,0.145,1,0
"Gna12",0.191157264570444,-0.0856309974042365,0.135,0.173,1,0
"Eci1",0.19120423966008,-0.172998177806687,0.252,0.285,1,0
"Sos2",0.193204439917505,0.0871585125363045,0.097,0.071,1,0
"Ralgapa2",0.193423570275839,-0.0732570158521378,0.071,0.103,1,0
"Rnf34",0.1935927793549,0.0754217290323077,0.11,0.084,1,0
"Tbxas1",0.19447522153936,-0.0749788438799454,0.032,0.056,1,0
"Ecsit",0.196860955191707,-0.152434340473059,0.123,0.155,1,0
"Zbtb7b",0.196865833547029,0.0725757643192999,0.103,0.076,1,0
"Pqlc2",0.197954920139761,-0.0551852151560194,0.032,0.057,1,0
"Mfsd6",0.199963597825875,0.0747492672801254,0.103,0.078,1,0
"Oxa1l",0.200357385887989,-0.124220939551915,0.116,0.15,1,0
"Ggact",0.2006679542621,-0.0723675655048541,0.09,0.126,1,0
"Srebf2",0.200985006079083,0.0763854371164978,0.129,0.1,1,0
"Spry2",0.201142788170308,0.0806551711612158,0.084,0.06,1,0
"Gpr146",0.201165158769026,-0.0580511666082825,0.065,0.095,1,0
"Cul2",0.204765520893751,0.0597593511388982,0.097,0.071,1,0
"Hmox1",0.205524413915887,-0.0699286557045448,0.058,0.087,1,0
"Txnrd2",0.205575935654249,-0.0809845159570914,0.071,0.102,1,0
"Dbp",0.205635506566171,0.113145906110497,0.077,0.055,1,0
"Fam101b",0.205752468645194,0.0862742191297962,0.052,0.034,1,0
"2310030G06Rik",0.205995749215212,-0.134026716653029,0.135,0.171,1,0
"C1qbp",0.206381666489365,-0.177563667346226,0.323,0.349,1,0
"Paxip1",0.206385282985527,0.0573199917595205,0.065,0.044,1,0
"2300009A05Rik",0.207429062806053,-0.199442909525611,0.271,0.298,1,0
"Lonp1",0.20795864470251,-0.0883259890510948,0.135,0.174,1,0
"Rnf186",0.208354061818601,0.0524041227662603,0.11,0.082,1,0
"Enpp5",0.208406286096755,-0.134788021247744,0.181,0.218,1,0
"Cdc23",0.208760165383768,0.0650596614170395,0.077,0.055,1,0
"Rab3il1",0.209116188692541,-0.0691467083463472,0.045,0.071,1,0
"Kat6a",0.209277795282993,0.0623685963571632,0.11,0.083,1,0
"Rpl29",0.209431642028499,0.0586148991829931,0.871,0.791,1,0
"Polr1c",0.209991387515585,0.0722022075220952,0.187,0.156,1,0
"Smim8",0.21075310383918,-0.119582868139955,0.174,0.211,1,0
"Ankrd40",0.211519711301052,0.0572628148631766,0.174,0.142,1,0
"Slc16a5",0.211815267435927,0.0539044983321571,0.071,0.05,1,0
"Fez2",0.212107962685443,0.0501318006437951,0.129,0.1,1,0
"Dis3l2",0.212156144475295,0.0925557190089217,0.077,0.055,1,0
"Snap23",0.212982948059807,-0.147764501157369,0.174,0.209,1,0

"Sbk1",0.213643046794791,-0.071484333734513,0.045,0.071,1,0
"Pgrmc1",0.214392653451509,-0.0998323741557238,0.619,0.481,1,0
"Slc25a25",0.214839173242993,-0.0519735969145677,0.032,0.055,1,0
"Ak1",0.216219858570544,0.0545613674331828,0.071,0.049,1,0
"Gnpat",0.217933714036411,-0.0925850629338493,0.084,0.114,1,0
"Klhdc4",0.218173899503675,0.0542683677446213,0.077,0.055,1,0
"Arnt2",0.219149153032659,0.0676244026162067,0.077,0.055,1,0
"Tbc1d20",0.219418475441447,0.0750865210217874,0.123,0.096,1,0
"Usp50",0.219440942988423,-0.139699922865228,0.452,0.467,1,0
"Tgfbr3",0.220047239575107,0.0668963828777685,0.052,0.034,1,0
"Ppp1r21",0.22026399837149,-0.0810544034898226,0.077,0.108,1,0
"Pex5",0.22213563568469,-0.100353750150564,0.084,0.114,1,0
"Tmem38b",0.22240610495358,-0.100091323626202,0.09,0.121,1,0
"Lrrc24",0.222584754117302,-0.0735819415805037,0.052,0.078,1,0
"Mgrn1",0.222660792471985,-0.0940242701020952,0.142,0.181,1,0
"Kcmf1",0.224158216841718,0.0506521754205566,0.187,0.156,1,0
"Tacc2",0.224172019895634,0.0830508119309628,0.174,0.144,1,0
"Parl",0.224209992215109,0.0644609586199594,0.213,0.178,1,0
"Vwa8",0.225494192523142,-0.0687943513112555,0.084,0.114,1,0
"Oplah",0.226743254590513,-0.0813408510046104,0.097,0.13,1,0
"Frrs1",0.227698293137289,-0.0504244017633736,0.252,0.201,1,0
"Smim22",0.228667415451931,-0.156088605490854,0.348,0.375,1,0
"Scrn3",0.229117057213686,-0.0679957709144991,0.039,0.062,1,0
"Galnt14",0.229505791172004,-0.0543673674456416,0.071,0.1,1,0
"Scand1",0.22990408328462,-0.203995625741124,0.639,0.583,1,0
"Dnph1",0.230089293506724,-0.0942727059701262,0.045,0.069,1,0
"Arfgef2",0.231518577017325,0.0609543222321021,0.103,0.079,1,0
"Ccbl1",0.231960738148161,-0.125962592383613,0.084,0.111,1,0
"Tomm6",0.232021615062193,-0.127983674879472,0.161,0.197,1,0
"Pex7",0.232840661294429,-0.1416791449999,0.155,0.186,1,0
"4430402I18Rik",0.234110238662764,-0.08918199969182,0.065,0.092,1,0
"Nop16",0.235180306601396,0.0544524540374099,0.11,0.085,1,0
"Auh",0.235577602271935,-0.170013089562553,0.226,0.257,1,0
"Fastk",0.236463071129323,-0.145899591120405,0.161,0.193,1,0
"Gsta4",0.236713443248553,0.189648982302616,0.123,0.095,1,0
"Oxld1",0.236854411674408,-0.0804943925738605,0.058,0.084,1,0
"Gata2",0.237158757017645,0.063991419993861,0.077,0.056,1,0
"Smpd2",0.238205868043047,-0.099320807798645,0.103,0.135,1,0
"Ugdh",0.238949960357329,-0.050805480287747,0.097,0.13,1,0
"Sec24b",0.239285659990048,0.0525266536791767,0.09,0.068,1,0
"Mpv17",0.240153412441744,-0.13730582544472,0.187,0.222,1,0
"Ncald",0.240463160471096,-0.0582634734537918,0.039,0.061,1,0
"Ugt8a",0.241255245888863,-0.117465245854195,0.11,0.14,1,0
"Hyal2",0.241605672757554,-0.0789036564030514,0.071,0.099,1,0
"Tubb4b",0.24466512587065,-0.0547762685100975,0.387,0.316,1,0
"Rxra",0.24491962207672,-0.078375031810601,0.084,0.113,1,0
"Mthfsl",0.24524680985441,-0.0513307124992392,0.252,0.202,1,0
"Fbxo28",0.245713806410855,0.0769326757406584,0.071,0.051,1,0
"Prnp",0.246213243665935,-0.129305676171484,0.174,0.205,1,0
"Tarbp2",0.246419981124401,0.0611008647906233,0.097,0.073,1,0

"Tbcel",0.246529571256746,0.0509278763329227,0.129,0.103,1,0
"Ppt2",0.249154848342145,-0.156373392460646,0.161,0.189,1,0
"Gm17750",0.24922663486375,-0.113041670813056,0.135,0.169,1,0
"Ankrd33b",0.249952447695599,-0.0619662718306612,0.065,0.092,1,0
"Ost4",0.250197936162788,-0.166972597880582,0.639,0.62,1,0
"Guf1",0.251080127307891,0.0521488169829336,0.123,0.097,1,0
"Phyhipl",0.252981659720649,0.0903319037364431,0.11,0.085,1,0
"Tmem53",0.254159722889228,-0.102214059202353,0.097,0.126,1,0
"Pcyt1a",0.254196832741206,-0.114977118364355,0.116,0.145,1,0
"Atg4a",0.254272463735751,-0.051593889992012,0.032,0.053,1,0
"Ubc",0.25605500050486,0.0791558179054626,0.832,0.731,1,0
"Smg7",0.256582171426961,0.0694507738416866,0.135,0.109,1,0
"Lemd3",0.257930466816617,0.0506729476412483,0.052,0.035,1,0
"Pcca",0.25838900087126,-0.144439756439934,0.187,0.221,1,0
"Psmb7",0.258509121466539,-0.0577665581517773,0.432,0.354,1,0
"Cpt1a",0.259833586229386,-0.107529116928553,0.239,0.277,1,0
"H2afx",0.26036331982124,0.0595714419058671,0.071,0.051,1,0
"Clpx",0.261775147564575,-0.131912821041364,0.174,0.206,1,0
"Osbpl6",0.263491114977647,-0.131093888019058,0.135,0.164,1,0
"L2hgdh",0.266120905048889,-0.12235892758537,0.084,0.109,1,0
"Acyp1",0.266311806723211,-0.1169614801385,0.148,0.179,1,0
"Med20",0.266601745515102,0.0501339033539439,0.065,0.047,1,0
"Ube2q1",0.267317569571295,0.0518663663672241,0.155,0.128,1,0
"Cpe",0.268190095457497,0.105539204280031,0.11,0.086,1,0
"Lrpprc",0.26865754005148,-0.106947928958551,0.129,0.159,1,0
"Cflar",0.270066545251882,-0.0586893824620038,0.335,0.272,1,0
"Mccc1",0.270238289306601,-0.0976370575105805,0.129,0.161,1,0
"Pitpnc1",0.271285388552144,0.0996782533644506,0.187,0.155,1,0
"Rpl36al",0.271508782387597,-0.126753404464535,0.787,0.753,1,0
"Acadvl",0.272777990987902,-0.154484305786527,0.323,0.351,1,0
"Fam120aos",0.275200145693893,-0.0656583890410331,0.045,0.067,1,0
"Steap2",0.276364504058536,-0.136581194411994,0.277,0.299,1,0
"Sc5d",0.276521242350592,-0.0702955147456574,0.058,0.082,1,0
"Phb2",0.276892623866237,-0.148423359412823,0.465,0.469,1,0
"Sf3a1",0.277461467969324,0.0698114226000609,0.103,0.081,1,0
"Fabp3",0.277646217401385,-0.130421111705978,0.29,0.322,1,0
"Magi2",0.277992803687716,-0.0617568608821298,0.039,0.059,1,0
"Tbx2",0.278485523237234,-0.081855341248553,0.11,0.14,1,0
"Atp6v1d",0.278865653119978,-0.0871613277219491,0.484,0.389,1,0
"Mrpl41",0.279070745366336,-0.0770993480871567,0.31,0.25,1,0
"Spc25",0.279946829073883,-0.104377164664604,0.077,0.102,1,0
"Uba52",0.281022690320566,0.0508432453291912,0.787,0.7,1,0
"Aldh5a1",0.281345650857855,-0.0860850267090553,0.065,0.088,1,0
"Pam",0.282930953329566,-0.272887944986039,0.239,0.265,1,0
"Ttc38",0.284514970590951,-0.0517924556833149,0.045,0.067,1,0
"Egln1",0.28535998869461,-0.0916869752771633,0.084,0.109,1,0
"Fastkd2",0.287414580632247,-0.0595652184954683,0.032,0.051,1,0
"Cmtm7",0.289820617959921,0.0603812678359005,0.09,0.07,1,0
"Spg7",0.292177452017228,-0.0644573677891807,0.142,0.173,1,0
"Dlst",0.294443982845183,-0.144098110058925,0.265,0.293,1,0

"Dera",0.295091496698879,-0.131541401037297,0.142,0.17,1,0
"Mgat5",0.295142773313718,0.0512964363024551,0.097,0.075,1,0
"Ndufaf6",0.297943648076672,-0.0848012882637594,0.071,0.095,1,0
"Mrps18a",0.298236155333153,-0.169543057763074,0.31,0.328,1,0
"Ldhb",0.298685218650354,0.235876462922345,0.845,0.888,1,0
"Slc35d1",0.2999930441385,-0.0527591684512275,0.065,0.088,1,0
"Tpp1",0.300101910155178,-0.201681789002346,0.355,0.352,1,0
"Coq2",0.300735997926076,-0.125298023490416,0.168,0.195,1,0
"Irgq",0.30288844083433,0.0602659225337158,0.058,0.042,1,0
"Degs1",0.303000875621092,-0.0748637338250093,0.297,0.324,1,0
"1110019D14Rik",0.303047386535831,-0.0585499600458635,0.052,0.073,1,0
"Neurl4",0.307144301407354,-0.074816060670505,0.084,0.109,1,0
"Cpt2",0.308778102710984,-0.135403155558572,0.123,0.147,1,0
"Nxt2",0.3091220873603,-0.118448791301197,0.11,0.136,1,0
"Slc39a1",0.309251687552064,0.0904862601138505,0.265,0.238,1,0
"Tgfbrap1",0.309317302050727,-0.0665097410547805,0.052,0.073,1,0
"Naa38",0.309878478536941,-0.168432704380567,0.484,0.474,1,0
"Tmem147",0.310876435508875,-0.139904761499099,0.523,0.526,1,0
"Npc2",0.311439227144292,-0.0589068051713694,0.581,0.478,1,0
"Mapk14",0.31236669292317,-0.07859253195835,0.097,0.124,1,0
"B4gat1",0.314975572081065,-0.0532296009393611,0.071,0.096,1,0
"Dus1l",0.315711173068387,-0.0970284006397653,0.084,0.108,1,0
"Fh1",0.317888260196392,-0.170524735939861,0.239,0.259,1,0
"Slc12a7",0.317925791860482,-0.0696270365192649,0.103,0.13,1,0
"Etv1",0.320706139865632,-0.116436644933675,0.071,0.092,1,0
"Vegfb",0.321591252059464,-0.0847026293020172,0.116,0.143,1,0
"Zdhhc12",0.322220242678861,0.064237551001022,0.097,0.077,1,0
"Atp6v0a4",0.323566097864981,-0.174266045401311,0.381,0.383,1,0
"Slc39a3",0.323830315134264,-0.0576898516654079,0.045,0.065,1,0
"Unkl",0.324388783951571,-0.0597056802226015,0.039,0.057,1,0
"Tmem120a",0.32547272565663,-0.16013994097953,0.168,0.191,1,0
"Svip",0.326141759539028,-0.131083708501062,0.142,0.167,1,0
"Grpel1",0.326457532340292,-0.167928977280979,0.355,0.368,1,0
"Ccr2",0.32685941163361,0.0574757297474681,0.065,0.049,1,0
"Eif2b2",0.327483240692879,0.0585671014110387,0.11,0.089,1,0
"Slc35a3",0.328441798642572,-0.114560202702188,0.148,0.174,1,0
"Hdhd3",0.330650214734403,-0.0823732839422162,0.039,0.056,1,0
"Csad",0.330712291731102,-0.125262931288564,0.161,0.188,1,0
"Mlx",0.330904461771185,-0.077908790815538,0.084,0.107,1,0
"Mrpl39",0.331040248968042,-0.0611495949851524,0.09,0.116,1,0
"Pcbd2",0.331593430723125,-0.0581272906062188,0.394,0.33,1,0
"Rab17",0.331756524833069,-0.0900621723208839,0.103,0.129,1,0
"Polr2l",0.332166493809041,-0.0555984916155016,0.458,0.496,1,0
"D10Jhu81e",0.333335776493655,-0.203491602711084,0.258,0.271,1,0
"Dnajb9",0.334551492539599,0.0801763968659104,0.168,0.144,1,0
"Slc12a6",0.33464328482816,-0.071165831680004,0.065,0.086,1,0
"Tomm5",0.336470515524609,-0.132223561221225,0.419,0.44,1,0
"Pgm3",0.336807305718947,-0.0607407647859337,0.039,0.056,1,0
"Msrb2",0.337002624781438,-0.0718838278117892,0.077,0.101,1,0
"Gbe1",0.338274076761411,-0.0570563993939152,0.065,0.087,1,0

"Socs7",0.340501516728866,0.0595624497066654,0.084,0.066,1,0
"Sema3b",0.340508507679799,-0.051988732473819,0.09,0.114,1,0
"Csnk1g3",0.341003620012803,-0.101129103838219,0.123,0.149,1,0
"Snta1",0.34366526233575,-0.0962553659023918,0.09,0.113,1,0
"Baiap2l2",0.344597723152015,0.0942698987661537,0.135,0.113,1,0
"Stard7",0.344813996283964,-0.0855423496761474,0.181,0.212,1,0
"Myl6b",0.345098748816141,-0.0713251119002241,0.039,0.056,1,0
"Atp6ap1",0.3463023184941,-0.102743428285077,0.394,0.32,1,0
"Pyurf",0.346335783922624,-0.173415530067631,0.265,0.28,1,0
"Pdhx",0.347938637156576,-0.0560544950558945,0.065,0.086,1,0
"Ndufa9",0.3488088668692,-0.162720847738441,0.381,0.392,1,0
"Deb1",0.348825156011852,-0.169682815510551,0.284,0.3,1,0
"Nkiras1",0.349662494943047,-0.103339736631107,0.103,0.127,1,0
"Gns",0.349686433508629,-0.137330320853924,0.245,0.265,1,0
"Pttg1ip",0.349801429767988,-0.12824631165515,0.323,0.339,1,0
"Ppih",0.350144373695652,-0.0569663414299891,0.045,0.063,1,0
"Mapt",0.351077059883446,-0.137789942498326,0.181,0.202,1,0
"Cyp2j11",0.352277309388736,-0.103252997504037,0.065,0.084,1,0
"Mtmr4",0.352753841134594,-0.0583538660878696,0.058,0.078,1,0
"Gmfb",0.353184426990353,-0.116002124653278,0.181,0.208,1,0
"Crocc",0.354843978863389,-0.052069089240643,0.039,0.056,1,0
"Nudt2",0.356364964475929,-0.0739449778647357,0.097,0.121,1,0
"Pex11a",0.356870539738145,-0.0789905816491697,0.065,0.085,1,0
"Psmd3",0.357481679555843,-0.0610345025047923,0.335,0.279,1,0
"Esrra",0.358968137954166,-0.131049096582138,0.2,0.224,1,0
"Galns",0.35917090152218,-0.0543375126978594,0.071,0.092,1,0
"Eci2",0.359994057939575,-0.102016166687656,0.187,0.214,1,0
"Samd8",0.360730998396058,-0.0715356597079845,0.11,0.135,1,0
"Tysnd1",0.360986073725646,-0.0863490266006283,0.065,0.084,1,0
"Eny2",0.364225605387451,-0.144562575099644,0.535,0.521,1,0
"Pde4dip",0.364834972365907,-0.0758974434632306,0.148,0.175,1,0
"Rnf5",0.365857833652844,-0.159597521420753,0.29,0.307,1,0
"Mcfd2",0.36763038705488,-0.124441653513532,0.213,0.237,1,0
"Aldh3a2",0.369183507547176,-0.110304818146945,0.11,0.131,1,0
"Mpeg1",0.369883450719137,0.0508028945867042,0.065,0.049,1,0
"Tmem189",0.369974586316335,-0.147824693570072,0.2,0.221,1,0
"Fnip1",0.371016418291782,-0.0797368115466169,0.11,0.134,1,0
"Cdk18",0.375181731958028,-0.0626715111654007,0.045,0.062,1,0
"Polr3k",0.375638881556092,0.0573649466033583,0.135,0.115,1,0
"Tm4sf1",0.376249498862933,0.087961903062678,0.071,0.056,1,0
"AU040320",0.376340359439283,0.0613854305288718,0.129,0.109,1,0
"Mrps11",0.376452024985468,-0.103236199620196,0.155,0.179,1,0
"Inpp5b",0.378344069378217,-0.0609213566858319,0.045,0.062,1,0
"Rufy3",0.378771572991202,-0.128740225363401,0.161,0.183,1,0
"Mrps7",0.380225659434704,-0.0754968984881826,0.381,0.319,1,0
"Aacs",0.384032930408057,-0.0721218668155095,0.045,0.061,1,0
"Lpcat3",0.384484459169889,-0.109698464464354,0.168,0.194,1,0
"Ergic3",0.386062505419249,-0.0510650832632631,0.406,0.348,1,0
"2010315B03Rik",0.390538742073346,0.05443398435079,0.084,0.068,1,0
"Bcar3",0.396010380530599,-0.0583458910012469,0.052,0.068,1,0

"Glrx",0.396511776479874,-0.0899044264773303,0.129,0.151,1,0
"Atp6v0d2",0.397814895261056,-0.214355881655978,0.077,0.095,1,0
"Bscl2",0.40288162528652,-0.0949200292604419,0.161,0.184,1,0
"Fundc2",0.405519202965318,-0.0736200818115559,0.465,0.388,1,0
"Ahcyl1",0.408492597704987,-0.11349927018131,0.239,0.257,1,0
"Fam107b",0.409695985268127,-0.0972549746772684,0.206,0.228,1,0
"Diablo",0.411042575732119,0.0541215288389756,0.187,0.167,1,0
"Atp6v1b1",0.411157311541321,-0.105904668078665,0.168,0.139,1,0
"Twf1",0.41152847190387,-0.0568101032876076,0.252,0.284,1,0
"Ptdss2",0.411911340739098,0.0547010016032138,0.174,0.154,1,0
"Gm9843",0.415635660932067,-0.0651523659214578,0.477,0.41,1,0
"Mrps26",0.417177279625187,-0.113670094473137,0.271,0.292,1,0
"Anapc7",0.420231644649478,-0.0667734898237977,0.077,0.096,1,0
"Chd3",0.421685868829605,-0.108995921900404,0.4,0.415,1,0
"Tmem140",0.425949496107774,-0.0654596301065162,0.065,0.082,1,0
"Mtch2",0.426724523311622,-0.185834889747582,0.477,0.445,1,0
"Mrps16",0.426869096599544,-0.0509828904175817,0.432,0.371,1,0
"Flad1",0.428985976183946,-0.0500069634723818,0.052,0.067,1,0
"Pus1",0.430933826445326,-0.054547160752635,0.065,0.082,1,0
"Mrpl14",0.434922628165682,-0.153439818517773,0.465,0.446,1,0
"Wwp1",0.437233459093028,-0.0520238962003321,0.329,0.284,1,0
"Vps8",0.438018970653648,-0.0753276889043528,0.058,0.074,1,0
"Chdh",0.439384788967905,-0.125754919632958,0.174,0.192,1,0
"Ddx49",0.43964656469301,0.0564027096721296,0.09,0.076,1,0
"Cpox",0.44000244174672,-0.137251776884567,0.187,0.204,1,0
"Pla2g12a",0.440003312003067,-0.123868348279626,0.129,0.146,1,0
"Hmgxb4",0.440249616602603,0.0635277660459834,0.071,0.058,1,0
"Pafah2",0.441263114202528,-0.108692649595287,0.135,0.153,1,0
"Stk16",0.441778343826656,-0.128265083160209,0.232,0.247,1,0
"Myeov2",0.444811239950397,-0.089075989599549,0.819,0.727,1,0
"Slmo2",0.445326996251357,-0.0531258880101592,0.135,0.162,1,0
"Clpp",0.44680506076133,-0.0689953831887898,0.342,0.294,1,0
"Edf1",0.446903676515046,-0.105882065569788,0.858,0.748,1,0
"Aifm1",0.447051649597286,-0.139834990498,0.226,0.241,1,0
"Atp6ap2",0.447582271547354,-0.148294225671189,0.31,0.319,1,0
"Sep15",0.447765137916049,-0.081675068868164,0.652,0.62,1,0
"1700037H04Rik",0.449455384263824,-0.0731425955224905,0.071,0.087,1,0
"Atp6v1c1",0.450168776551058,-0.079573860359246,0.303,0.26,1,0
"Cdpf1",0.450698230685596,-0.0830067686909593,0.11,0.129,1,0
"Tbc1d10a",0.450769643866726,-0.0775228075489401,0.071,0.087,1,0
"Mto1",0.450819418588777,0.0680694385112161,0.077,0.064,1,0
"Dfna5",0.45463909355305,-0.0556562029545675,0.071,0.089,1,0
"Auts2",0.454693570000537,0.0528363802574324,0.077,0.064,1,0
"Zbtb8os",0.455831575269964,-0.10364631268956,0.142,0.161,1,0
"Gm9493",0.456563572453773,-0.132103397935537,0.374,0.379,1,0
"Egfl7",0.457763357105546,0.0760425684042037,0.103,0.088,1,0
"Nt5c3",0.458830625093237,-0.103750620247334,0.155,0.175,1,0
"Cltb",0.461944242169638,-0.162269832184788,0.381,0.379,1,0
"Ctnnbip1",0.464524039529903,-0.0990858235989778,0.148,0.168,1,0
"Mrpl16",0.464973377776841,-0.079714880415255,0.135,0.157,1,0

"Pfdn1",0.46671134490183,-0.154353176143512,0.613,0.574,1,0
"Cers6",0.466971670445364,-0.0613584641639611,0.077,0.095,1,0
"Zfp871",0.467150312150485,-0.0682508480861388,0.103,0.123,1,0
"Klf9",0.468046408911457,-0.136573355257583,0.271,0.285,1,0
"Tef",0.469675468970661,-0.121841341025891,0.123,0.138,1,0
"Dlat",0.471089187498894,-0.139858831596067,0.226,0.24,1,0
"Nomo1",0.474696076137706,-0.0540237081900758,0.077,0.094,1,0
"1110001J03Rik",0.474849905591259,-0.0828426472670352,0.542,0.538,1,0
"Stoml2",0.475582948725111,-0.051782474554651 7,0.232,0.203,1,0
"Klhdc8a",0.475660108728123,0.067969036698861,0.116,0.1,1,0
"Ergic1",0.4767256829089,-0.104852415384361,0.135,0.153,1,0
"Adgra3",0.478294697756281,-0.0602639967590658,0.09,0.108,1,0
"Pgap2",0.479021719184532,0.0533567051498601,0.09,0.077,1,0
"Alas1",0.480182180242376,-0.129925448050693,0.168,0.184,1,0
"Slc25a19",0.481398171171916,-0.0891656394682454,0.135,0.154,1,0
"Grb10",0.481635704787861,-0.0542035573440319,0.071,0.087,1,0
"Mrps2",0.488151216713603,-0.0761994897314941,0.058,0.071,1,0
"Airn",0.488355002717136,-0.0636211565110213,0.045,0.057,1,0
"Msmo1",0.491175626358731,-0.0654965642726093,0.116,0.136,1,0
"Ppcs",0.49159044192928,-0.0931040928489844,0.11,0.126,1,0
"Asna1",0.492110958032059,-0.126887649915163,0.213,0.227,1,0
"Spcs1",0.497908912889084,-0.148767940167211,0.594,0.533,1,0
"Unc119b",0.498568548920472,-0.10861929579205,0.123,0.138,1,0
"Acad8",0.500008225984344,-0.0722162218578694,0.11,0.127,1,0
"Por",0.500541944531143,-0.161228554267988,0.258,0.265,1,0
"App",0.505156321094808,0.100611103483281,0.852,0.722,1,0
"Tmem161b",0.506366484185299,0.0542932514571222,0.065,0.053,1,0
"Tmem19",0.506393934995796,-0.119548176217345,0.168,0.183,1,0
"Vill",0.507644572424063,-0.0534615890861662,0.058,0.072,1,0
"Nhp2",0.508762740727799,-0.139237382635937,0.297,0.303,1,0
"Nnt",0.509361964043928,-0.0658328302690044,0.239,0.258,1,0
"Prcp",0.510415095049411,-0.089344302978476,0.168,0.185,1,0
"Mrps28",0.512704148952617,-0.0708769973343186,0.252,0.222,1,0
"Zeb2",0.516279741861203,-0.0542292863879453,0.065,0.079,1,0
"Get4",0.518976623296252,-0.0557342501183034,0.123,0.142,1,0
"Acly",0.519837621662848,-0.0763417354044316,0.148,0.167,1,0
"Serp1",0.520886219849693,-0.140020023927013,0.439,0.42,1,0
"Ufc1",0.524095997122941,-0.0559465398830858,0.361,0.321,1,0
"Sec61g",0.524261373282391,-0.0811740051502403,0.781,0.74,1,0
"Syne4",0.528564984521121,-0.069176593033114,0.135,0.154,1,0
"Prkaa2",0.531933421289057,-0.140516114075399,0.252,0.259,1,0
"Agtrap",0.539244704262175,-0.0674116690415459,0.258,0.225,1,0
"Cmip",0.53944929826183,-0.0955506668271944,0.258,0.27,1,0
"Slc30a9",0.540653046008811,-0.119839464120029,0.219,0.231,1,0
"Aamdc",0.543137309730502,-0.128538961326265,0.271,0.281,1,0
"2810428I15Rik",0.543521855225887,-0.111058839551358,0.329,0.342,1,0
"Rmnd1",0.543638560136045,-0.0955608762799413,0.11,0.123,1,0
"Fhit",0.544583367083133,-0.0688422609365104,0.045,0.055,1,0
"Abracl",0.545449778505789,-0.07908858620173,0.413,0.361,1,0
"Mrpl35",0.54644359761647,-0.123607165087904,0.239,0.251,1,0

"Prdx6",0.547127854563598,-0.0651323074831345,0.516,0.438,1,0
"Mtfr1l",0.547655220693528,-0.118721598181822,0.219,0.233,1,0
"Pon3",0.548399282302199,0.0655764263119462,0.052,0.043,1,0
"1110065P20Rik",0.548944017272529,-0.0504022001522491,0.219,0.195,1,0
"Slc39a7",0.552135977773099,-0.0532254727922692,0.129,0.148,1,0
"Tmem238",0.554800647843446,-0.122148079708372,0.368,0.368,1,0
"Immt",0.55799839598429,-0.0657731674636016,0.484,0.413,1,0
"Lrrc28",0.558578519633552,-0.0599486179629344,0.052,0.062,1,0
"Naprt",0.561260683703808,-0.0520933526482535,0.084,0.098,1,0
"Lppos",0.566974777255755,-0.0800196663901593,0.077,0.089,1,0
"Ggnbp1",0.56779992209415,-0.113876008951801,0.161,0.174,1,0
"Egln2",0.568128985335308,-0.0774509180622824,0.135,0.151,1,0
"Rpgrip1l",0.569358133351223,0.0865299558585148,0.052,0.043,1,0
"Ubr3",0.572832896952733,-0.0646899758548953,0.155,0.172,1,0
"Tusc2",0.573472922393748,-0.140542450418356,0.187,0.195,1,0
"Snx27",0.574732503640798,-0.0568328513268012,0.135,0.152,1,0
"Isca2",0.57475924679524З,-0.0729748006393348,0.181,0.198,1,0
"Rab1a",0.577234109604568,-0.129571397693643,0.529,0.483,1,0
"B4galt5",0.577434406582889,-0.115940237341271,0.116,0.126,1,0
"Ccser2",0.57818683800501,-0.134064993216591,0.335,0.327,1,0
"Gdi2",0.578459577762315,-0.150238938620818,0.574,0.506,1,0
"Sms",0.583907695130404,-0.113181501530387,0.181,0.192,1,0
"Tmem160",0.585374485609356,-0.0696014527261274,0.439,0.381,1,0
"0610009B22Rik",0.587323335699152,-0.0813052807023713,0.239,0.255,1,0
"Slc33a1",0.588026518815388,-0.140099674440567,0.213,0.217,1,0
"Rab1b",0.589871497710106,-0.0715083515060054,0.174,0.19,1,0
"Gucd1",0.591187975413096,-0.0612838948788103,0.129,0.143,1,0
"Dtnbp1",0.593007560977995,-0.12178118382218,0.232,0.241,1,0
"Rhcg",0.593882967071386,-0.091726102026724,0.129,0.112,1,0
"Ccpg1os",0.594566538433148,-0.0660182617619527,0.09,0.102,1,0
"Add3",0.596031180453776,-0.093377793272999,0.181,0.192,1,0
"1810024B03Rik",0.598875938468678,-0.0554158885832854,0.058,0.068,1,0
"Igfbp7",0.599488733122044,0.122260251824576,0.955,0.942,1,0
"Hint3",0.602212731012797,-0.113717810872966,0.206,0.217,1,0
"Sgk1",0.605096073834208,0.0686471609976406,0.071,0.085,1,0
"Cuta",0.607101277601034,-0.12887991915557,0.432,0.413,1,0
"Atp6v0b",0.608151235299873,-0.156728135863152,0.768,0.666,1,0
"Mgst3",0.609000919231001,-0.100105682907861,0.6,0.562,1,0
"Skp1a",0.609606562782693,-0.0911126711724919,0.606,0.523,1,0
"Hdhd2",0.609703431885586,-0.091000500290911,0.142,0.154,1,0
"Got1",0.610037621747151,-0.0634965283099543,0.245,0.217,1,0
"Fam210a",0.611989925685353,-0.0837069684341362,0.135,0.148,1,0
"Ggct",0.615055450829384,-0.135626346910599,0.116,0.125,1,0
"Sqle",0.615608616538862,-0.051692573144894,0.103,0.115,1,0
"Osr2",0.615610408468553,0.091226042688913,0.058,0.05,1,0
"Gfm1",0.618625808877051,-0.0685975887298976,0.116,0.128,1,0
"Slc11a2",0.620373297028843,-0.0516253136199636,0.09,0.102,1,0
"Pgm2",0.620433601820105,-0.0953718002267654,0.097,0.106,1,0
"Mrpl30",0.627121741829982,-0.126291214400282,0.477,0.458,1,0
"Rabggta",0.627819652849486,-0.0545706267003003,0.077,0.088,1,0

```
"1190007I07Rik",0.627901544878756,-0.0791282442338491,0.135,0.148,1,0
"Tomm70a",0.628956999180891,-0.126286777143034,0.187,0.194,1,0
"DHRSX",0.629876909108699,-0.0717045423572442,0.116,0.128,1,0
"Rnf7",0.630722346034208,-0.0594471719203156,0.413,0.378,1,0
"March8",0.632694821405339,-0.113183089080626,0.232,0.241,1,0
"Akr1e1",0.633277016725138,-0.129170736755481,0.194,0.2,1,0
"Nfs1",0.63378882526268,-0.106299395663956,0.206,0.216,1,0
"Rnpepl1",0.637233945373491,-0.0853235258228978,0.2,0.211,1,0
"Rdh14",0.637897306668117,-0.0983370459562787,0.148,0.159,1,0
"Atp5g2",0.638498527224727,-0.0785966871503687,0.832,0.743,1,0
"Trf",0.640622999684735,0.187187019335609,0.077,0.069,1,0
"Nadk2",0.640729976889701,-0.0684771407973651,0.123,0.135,1,0
"Adi1",0.642643111619275,-0.0692337607238456,0.077,0.086,1,0
"Rpl6l",0.643308199378611,-0.0976350958761125,0.4,0.401,1,0
"Chchd7",0.646015706752449,-0.107110623871027,0.29,0.298,1,0
"Rpain",0.646457402846702,-0.0990257228724565,0.129,0.137,1,0
"Gipc2",0.647656753815353,-0.064314237728103,0.381,0.372,1,0
"Ubap1",0.650910801803163,-0.050755188918342,0.071,0.081,1,0
"Commd8",0.6517424500181,-0.0552528279813621,0.181,0.163,1,0
"Cs",0.65596623578529,-0.0528819656983864,0.316,0.285,1,0
"Tprn",0.656677991345772,-0.0543741344361021,0.071,0.08,1,0
"Gsr",0.658900434248357,-0.158389077639856,0.426,0.401,1,0
"Tfg",0.659146142458677,-0.0713271875873869,0.148,0.161,1,0
"Calr",0.662146610566272,-0.0674200419175897,0.813,0.714,1,0
"Jtb",0.664370214812709,-0.0630320079290588,0.348,0.315,1,0
"Furin",0.664527181825501,-0.120449574315163,0.284,0.283,1,0
"Ahcyl2",0.665582027206839,-0.119739304492301,0.29,0.291,1,0
"Gtf2h5",0.669020675617728,-0.0616959268894204,0.484,0.431,1,0
"Nr3c1",0.669411898258721,-0.0624319094365848,0.181,0.193,1,0
"Tmem242",0.669729230997295,-0.0916219346866796,0.232,0.243,1,0
"Rnaseh2b",0.676774909083286,-0.0639180605772335,0.097,0.106,1,0
"Gabarapl1",0.67779518672479,-0.0531616256527248,0.619,0.579,1,0
"Ttc32",0.679638002638938,-0.0559904350957249,0.161,0.147,1,0
"Adh5",0.680509218882613,-0.0970445961070802,0.335,0.301,1,0
"Evc",0.687220825718742,-0.0785220663311496,0.226,0.204,1,0
"Zcrb1",0.687610403835918,-0.060141763321214,0.316,0.292,1,0
"Far1",0.687718269703923,-0.0938361499122838,0.323,0.288,1,0
"2210016F16Rik",0.688204472925047,-0.0753230875483496,0.181,0.191,1,0
"Tfrc",0.690577576286234,-0.0557064339650466,0.097,0.105,1,0
"Dmxl1",0.695778389171269,-0.069184957236494,0.232,0.211,1,0
"Dync1li1",0.696533106653444,-0.0626570077918299,0.135,0.146,1,0
"Fuom",0.697868117423454,-0.1272797609163,0.297,0.294,1,0
"Pi4k2a",0.701028677101096,-0.0637604630129205,0.116,0.125,1,0
"Kti12",0.701384752691636,-0.052126183478667,0.058,0.065,1,0
"Chchd1",0.706406454350234,-0.0542421114236425,0.381,0.356,1,0
"Pet100",0.707593021914528,-0.0691548880891086,0.581,0.503,1,0
"Polr2k",0.70898598420237,-0.0841945029967972,0.503,0.441,1,0
"Aga",0.713911330546365,-0.0670672774886138,0.148,0.158,1,0
"Mrpl22",0.71556377171059,-0.0621431865300722,0.194,0.179,1,0
"Slc44a4",0.715930455469227,-0.0897573381411675,0.11,0.116,1,0
```

"Zswim7",0.716301766070164,-0.0745642607295459,0.077,0.084,1,0
"Rnf187",0.717943867277976,-0.0738394265703725,0.413,0.368,1,0
"Cox20",0.717961931905283,-0.11585710534337,0.561,0.524,1,0
"Pnpo",0.720346208468867,-0.0687908126942602,0.11,0.117,1,0
"Il11ra1",0.720424794517455,-0.0835129340004148,0.194,0.175,1,0
"Sec31a",0.722936782803091,-0.0943950327355317,0.213,0.217,1,0
"Nedd8",0.72892219760571,-0.0719697731137943,0.684,0.631,1,0
"Fbxo22",0.729048605913673,-0.0869679260979421,0.168,0.174,1,0
"Chuk",0.731064339132016,-0.105817039410062,0.245,0.245,1,0
"Pink1",0.732167000107906,-0.113501366205775,0.284,0.286,1,0
"Mrps35",0.733727601814123,-0.0746816620215122,0.213,0.222,1,0
"Smim20",0.734983869544269,-0.0533119008680895,0.297,0.276,1,0
"Mrpl11",0.736309374694525,-0.0784336777392742,0.239,0.246,1,0
"Dnajc2",0.738678710945903,-0.0715586948440843,0.226,0.206,1,0
"Rhot2",0.743128708097589,-0.0621684176434063,0.116,0.124,1,0
"Glmp",0.743274251488664,-0.0611356747255856,0.335,0.317,1,0
"Tmco1",0.746692897036985,-0.0915140857628947,0.645,0.553,1,0
"Trappc2l",0.747419125951543,-0.11691756441303 3,0.387,0.366,1,0
"Thap7",0.749112100848865,-0.0552700784511739,0.09,0.097,1,0
"Amfr",0.751797047218824,-0.0819054879511321,0.258,0.264,1,0
"Pex16",0.752028227015355,-0.0863539033052562,0.123,0.128,1,0
"Man2b1",0.752330216294277,-0.066417418514007,0.194,0.201,1,0
"Lgr4",0.753061160627651,-0.0778743008082969,0.303,0.312,1,0
"Nfia",0.754066082837282,-0.0625833484680656,0.226,0.229,1,0
"Commd3",0.754904186128032,-0.104843875503116,0.374,0.367,1,0
"Commd6",0.756683484907736,-0.128615231347276,0.348,0.339,1,0
"1810058I24Rik",0.758079123118368,-0.138170185645049,0.387,0.367,1,0
"Hmbs",0.758490324514402,-0.0513419182549036,0.116,0.125,1,0
"Dpm3",0.75893753854714,-0.128371397562356,0.51,0.467,1,0
"Apoo",0.759258674447456,-0.0811921872944114,0.265,0.272,1,0
"Abcd3",0.761560680428916,-0.14605083672488,0.419,0.392,1,0
"Slc25a45",0.762209001735684,-0.0526934950105664,0.077,0.083,1,0
"Prkcz",0.762581503982185,-0.0603561345949939,0.232,0.216,1,0
"Crat",0.763701689233084,-0.0888130672061858,0.129,0.134,1,0
"Rars2",0.765407183155623,-0.0506880950026637,0.084,0.09,1,0
"Dram2",0.76847387349109,-0.0897387257686044,0.316,0.288,1,0
"Coa3",0.768787231328395,-0.0557563620711361,0.606,0.536,1,0
"Rab18",0.76891876680098,-0.102972008905772,0.381,0.338,1,0
"Itfg1",0.769353675949578,-0.107414794977449,0.245,0.245,1,0
"Bola1",0.769527932007795,-0.0932399353872754,0.245,0.249,1,0
"Mrps33",0.771146554988483,-0.0709215623778745,0.516,0.463,1,0
"Zfyve21",0.773395099648899,0.0998334015417373,0.116,0.129,1,0
"Rogdi",0.774780882149152,-0.0780929534254186,0.245,0.248,1,0
"Scamp3",0.776110582012049,-0.0549632396564552,0.142,0.15,1,0
"Dnajc15",0.779434992700062,-0.103218738515885,0.458,0.428,1,0
"Slc25a11",0.78001078529544,-0.114889365393064,0.335,0.326,1,0
"Atp6v0d1",0.780378277837336,-0.0852995529266704,0.284,0.286,1,0
"Atp6v1g3",0.780451806379869,-0.840275667163263,0.219,0.194,1,0
"Cyhr1",0.782142325997182,-0.0524242354101613,0.239,0.224,1,0
"Lamtor1",0.784152047887423,-0.113195778104303,0.284,0.278,1,0

"Rnft1",0.786059500635915,-0.0572621495081007,0.129,0.136,1,0
"Bccip",0.786643742061713,-0.118503613507425,0.232,0.232,1,0
"Ctsl",0.788528472323868,0.230438387327812,0.735,0.723,1,0
"Timm17a",0.789948201541337,-0.0993336633333705,0.445,0.419,1,0
"Cd164",0.794229731006618,-0.0618728400644869,0.394,0.384,1,0
"Polr2i",0.801360062436675,-0.106696907914483,0.413,0.367,1,0
"Etfdh",0.807009415800917,-0.0531718056641911,0.161,0.168,1,0
"Tmem51",0.808690842100059,-0.0587566815557852,0.142,0.147,1,0
"Avpi1",0.808711868311768,-0.0605787813231169,0.135,0.126,1,0
"Tmem9b",0.809317879842684,-0.0883508568879923,0.258,0.259,1,0
"Mtor",0.810175460123146,-0.072078052380272,0.123,0.127,1,0
"Thoc7",0.811809936938331,-0.0986297955225386,0.439,0.394,1,0
"Gm5617",0.811976752301791,-0.118498153565417,0.232,0.231,1,0
"Mrpl37",0.813239271449776,-0.0803962391140408,0.161,0.165,1,0
"Eif5a",0.813891683764208,-0.0861486665600313,0.723,0.668,1,0
"Mrpl2",0.817432562415941,-0.130363335418066,0.265,0.258,1,0
"Slc25a46",0.818657240734031,-0.0607911659826424,0.116,0.12,1,0
"Snrpn",0.8254268621245,-0.0583781798194994,0.077,0.081,1,0
"Toporsos",0.829732062241025,-0.0862877802988055,0.252,0.233,1,0
"Poldip2",0.829738625508876,-0.0618233896585177,0.219,0.224,1,0
"Slc48a1",0.830996931393988,-0.050580770285594,0.245,0.252,1,0
"Lrrk2",0.832316480364424,-0.0581249111106306,0.129,0.121,1,0
"Trap1",0.834410847533946,-0.0832270511645821,0.232,0.235,1,0
"Hsd17b4",0.834856879186183,-0.0707862125443325,0.29,0.271,1,0
"Isca1",0.834981876887105,-0.069267064612808,0.123,0.126,1,0
"2310061I04Rik",0.836919304818107,-0.0800249898903454,0.161,0.164,1,0
"Aspscr1",0.83814076207944,-0.0723881966531731,0.148,0.152,1,0
"Mgat2",0.839410643277242,-0.0732417550540694,0.252,0.252,1,0
"Ppdpf",0.840433751108601,-0.124759462677117,0.426,0.398,1,0
"Mpp6",0.841667128977862,-0.0575856323906133,0.245,0.233,1,0
"Mettl23",0.842292114148987,-0.0793966556299605,0.187,0.188,1,0
"Szrd1",0.843307264800048,-0.0575342574107023,0.213,0.203,1,0
"Lrrc58",0.8448779212743,-0.0931964141626083,0.342,0.336,1,0
"Fam96a",0.845139237067859,-0.124350173281783,0.31,0.297,1,0
"March5",0.845459573399146,-0.0848079156419391,0.245,0.229,1,0
"Vldlr",0.845787362092902,-0.0737361672136405,0.161,0.163,1,0
"Snx3",0.846045431264241,-0.099088033897631,0.458,0.416,1,0
"Dbt",0.846730378148174,-0.0774381611804464,0.194,0.195,1,0
"Cetn4",0.850603946953836,-0.0517616282790286,0.09,0.094,1,0
"Dhrs1",0.851040776380689,-0.0593659259641802,0.277,0.263,1,0
"Tkt",0.85127354801994,-0.0568341870953242,0.458,0.425,1,0
"Ykt6",0.854759452345304,-0.0537819395370744,0.116,0.109,1,0
"Egf",0.857733234026332,-0.187396092208944,0.2,0.206,1,0
"Tmem14c",0.860932828603476,-0.0684495492946982,0.432,0.399,1,0
"Cmc2",0.863602080325257,-0.0641918726527181,0.2,0.19,1,0
"Snx6",0.868500205309974,-0.0660233312713404,0.335,0.309,1,0
"Nadk",0.870696920630291,-0.110947473677093,0.245,0.239,1,0
"Babam1",0.876476892313061,-0.0790250549493372,0.213,0.215,1,0
"C330006A16Rik",0.876974303066743,-0.0670043800515539,0.187,0.188,1,0
"Acaa1a",0.877078866958223,-0.11679128109305,0.413,0.375,1,0

"Pam16",0.877937983123353,-0.0731109107652117,0.316,0.303,1,0
"Gstm1",0.883573442153498,0.0867970824776209,0.394,0.379,1,0
"Sdc2",0.883878209510396,-0.0684674805016012,0.219,0.206,1,0
"Guca2a",0.88467102293009,-0.168060541123706,0.065,0.06,1,0
"Idh3a",0.888615168695744,-0.112899650945314,0.335,0.318,1,0
"Itpr2",0.89325345837931,-0.073520380424376,0.226,0.225,1,0
"Sema4a",0.894762857510645,-0.0616880990029832,0.174,0.176,1,0
"Snf8",0.897925934222722,-0.068683252497527,0.329,0.311,1,0
"Pnpla2",0.899176784580811,-0.0526485444471756,0.142,0.136,1,0
"Akt1s1",0.901666518559613,-0.0693583069336238,0.168,0.159,1,0
"Dnaja3",0.903461315850338,-0.062126962103023,0.194,0.187,1,0
"Mrpl54",0.909951365945028,-0.108080858328594,0.555,0.502,1,0
"Fam63b",0.918449117539904,-0.0819975454381705,0.252,0.246,1,0
"Dnajc30",0.920556003327807,-0.0727123329602863,0.155,0.147,1,0
"Rab7",0.925297236712642,-0.115390971864087,0.497,0.435,1,0
"Rer1",0.926078754987369,-0.106683729369219,0.548,0.488,1,0
"Dnaja1",0.928855669201625,-0.0658297254853064,0.652,0.583,1,0
"Trabd2b",0.929460746117757,-0.0870282194600788,0.226,0.209,1,0
"1500011K16Rik",0.929723299269674,-0.138460285183639,0.4,0.357,1,0
"Gm8797",0.930241824871823,-0.0623558076883853,0.11,0.105,1,0
"Grcc10",0.931832654042221,-0.0857932445304745,0.542,0.491,1,0
"Mcee",0.937905360526942,-0.1180116032761,0.49,0.445,1,0
"Dhrs7b",0.940186686284594,-0.0594972865861857,0.123,0.119,1,0
"Mkks",0.940411817225367,-0.0586598445676131,0.297,0.287,1,0
"Ndufs3",0.941139351966844,-0.124414344828557,0.639,0.54,1,0
"Mrpl18",0.949534213610248,-0.0752878339835176,0.387,0.364,1,0
"Phb",0.949846920872589,-0.0999579224863127,0.374,0.356,1,0
"Eno1",0.952020167383706,-0.0553761144795326,0.665,0.61,1,0
"Tmem116",0.956501116016303,-0.0684459515647491,0.2,0.193,1,0
"Emc7",0.958008446737231,-0.0552489573279312,0.348,0.334,1,0
"Glyr1",0.966338711094118,-0.0682270459074132,0.194,0.19,1,0
"Znrf2",0.966533459385696,-0.0793638089818018,0.168,0.162,1,0
"Nit1",0.969808484348686,-0.0644972677775728,0.245,0.239,1,0
"Snx7",0.969902565642915,-0.076961160417809,0.2,0.197,1,0
"Tm2d2",0.971308219207548,-0.0735634184862641,0.361,0.34,1,0
"Alkbh5",0.971615332855477,-0.0856572223493011,0.316,0.3,1,0
"Nudcd2",0.975055773254302,-0.0635777592972763,0.148,0.145,1,0
"Ebp",0.976090130089711,-0.0895216507498609,0.219,0.213,1,0
"Zdhhc6",0.980972835233707,-0.0573515617422244,0.155,0.152,1,0
"2610507B11Rik",0.984150026134879,-0.0710658878540007,0.277,0.269,1,0
"Slc16a10",0.984212878393799,-0.0540369815362828,0.11,0.107,1,0
"Taf12",0.985166329461244,-0.0558361675227375,0.135,0.133,1,0
"Arhgap18",0.985774938568847,-0.0512531880647807,0.11,0.107,1,0
"Mccc2",0.992621503013698,-0.0775090692497111,0.129,0.126,1,0
