# Supplementary material for: Single-cell analysis of senescent epithelia reveals targetable mechanisms promoting fibrosis
Source: JCI Insight. 2022 Nov 22;7(22):e154124. doi: 10.1172/jci.insight.154124 (PMC9746814; doi:10.1172/jci.insight.154124)
Supplement: Supplemental data set 3 [file jciinsight-7-154124-s291.pdf]

Gene	Gene Alias	Protein symbol	Protein name
PDIA3	Protein Disulphide Isomerase Family A Member 3, P58, ER protein 57/60	P30101-PDIA3_HUMAN	Protein disulfide-isomerase A3
MAL2	Mal, T Cell Differentiation Protein 2	Q969L2-MAL2_HUMAN	Protein MAL
LGALS3	Galectin 3	P17931-LEG3_HUMAN	Galectin-3
LAMA3	Laminin Subunit Alpha 3, Epiligrin	Q16787-LAMA3_HUMAN	Laminin subunit alpha-3
PROS1	Protein S, THPH5/6	P07225-PROS_HUMAN	Vitamin K-dependent protein S
JAG1	Jagged Canonical Notch Ligand 1, HJ1, CD339, JAGL1	P78504-JAG1_HUMAN	Protein jagged-1
MDK	Midkine, NEGF2, ARAP	P21741-MK_HUMAN	Midkine
