# Supplementary material for: Single-cell analysis of senescent epithelia reveals targetable mechanisms promoting fibrosis
Source: JCI Insight. 2022 Nov 22;7(22):e154124. doi: 10.1172/jci.insight.154124 (PMC9746814; doi:10.1172/jci.insight.154124)
Supplement: Supplemental data set 4 [file jciinsight-7-154124-s292.pdf]

```
group,Sample Name,% Aligned,M Aligned
uninjured,EOS02_1,85.40%,59.3
Late injury and Vehicle,EOS02_10,80.10%,49.4
Late injury and Vehicle,EOS02_11,78.30%,50
Late injury and Vehicle,EOS02_12,83.80%,62.6
Late injury and Vehicle,EOS02_13,80.90%,49.9
Late injury and Vehicle,EOS02_14,75.70%,44.5
Late injury and ABT263,EOS02_15,75.00%,39.8
Late injury and ABT263,EOS02_17,77.20%,47.1
Late injury and ABT263,EOS02_19,77.50%,58
uninjured,EOS02_2,86.40%,54.4
uninjured,EOS02_3,86.70%,54.8
uninjured,EOS02_4,86.80%,52.5
Early injury,EOS02_5,69.10%,37.2
Early injury,EOS02_6,49.30%,24.6
Early injury,EOS02_7,71.60%,47.8
Early injury,EOS02_8,75.50%,42.5
Late injury and Vehicle,EOS02_9,74.40%,49.3
```
