# Supplementary material for: Single-cell analysis of senescent epithelia reveals targetable mechanisms promoting fibrosis
Source: JCI Insight. 2022 Nov 22;7(22):e154124. doi: 10.1172/jci.insight.154124 (PMC9746814; doi:10.1172/jci.insight.154124)
Supplement: Supplemental data set 1 [file jciinsight-7-154124-s289.pdf]

"","baseMean","log2FoldChange","lfcSE","stat","pvalue","padj","rank"
"Gnai3",3994.05361649944,-0.281617175200882,0.19195856399579,-1.467072
73350439,0.14235627056793,0.257506500003109,-0.382072926889167
"Cdc45",138.247738139596,-0.840024416134431,0.313266445201051,-2.68150
14151781,0.00732926044327393,0.026681463801896,-3.04406888206389
"Scml2",55.5789031140869,-0.342670953195824,0.396927199659877,-0.86330
9325965706,0.387967418006837,0.532267711532318,-0.216091283995595
"Apoh",190.979122501932,1.59343229532112,0.659446105490739,2.416319214
04,0.0156783097233428,0.0477183846420054,4.84791981513854
"Narf",963.699609766635,0.209772011356659,0.220128022342157,0.95295459
9440317,0.340613074290821,0.485295851115997,0.151664445053549
"Cav2",3036.02344596266,-1.0400450463536,0.409613861062238,-2.53908655
253142,0.0111142323564362,0.0368358494791421,-3.43348379766423
"Klf6",8978.05304761691,-0.376970810072955,0.191528835131471,-1.968219
61462978,0.0490427743566786,0.114670345893575,-0.816403355395693
"Scmh1",1640.56386433039,0.172102948737793,0.154320475503971,1.1152308
089756,0.26475152680456,0.406550425585528,0.154900795969626
"Cox5a",9812.69561344126,0.294146353788565,0.548402801670785,0.5363691
6677378,0.591703436527986,0.714677173104297,0.0988109210527677
"Tbx2",4490.2515090332,-0.168297657600532,0.154590939802689,-1.0886644
3153355,0.276301892283404,0.419868622534628,-0.146050965841667
"Tbx4",3.17477510445459,-1.29668634477755,1.06883973506469,-1.21317191
178252,0.225064100641818,0.361051289463199,-1.32098009366858
"Ngfr",280.790729569748,-2.30731173907824,0.486063170458671,-4.7469380
0993183,2.06519211805942e-06,4.88086253550576e-05,-22.9060761233315
"Wnt3",1.95078912944271,0.10646280284053,1.15212567942898,0.0924055463
231196,0.926375832956093,0.954827720620495,0.004921174076705
"Wnt9a",141.939329953991,-0.525748221639215,0.262551035238763,-2.00246
104975782,0.045235168203067,0.107573522334846,-1.1721981072111
"Fer",454.597879204284,-0.710924466922441,0.21292083375144,-3.33891453
643452,0.00084106436050873,0.005160186564254,-3.74428457268803
"Xpo6",2470.21160042978,0.150349963772181,0.164256583499634,0.91533599
7917645,0.360015266597002,0.505187309687539,0.102662865596269
"Tfe3",2092.84870026928,-0.501275204740469,0.0890614811026883,-5.62841
756654031,1.8187038200431e-08,1.27557867205757e-06,-6.80336253074323
"Axin2",820.147332501563,-1.6775264111466,0.278809550059865,-6.0167465
9561125,1.77957324179185e-09,2.42644189289913e-07,-25.5515282214686
"Brat1",1006.12220298906,-0.0533760548824274,0.0984243674894628,-0.542
305287236332,0.587608213893553,0.711096384961302,-0.0181984215744075
"Gna12",4888.6413643398,0.233509166741751,0.108831807554948,2.14559669
629537,0.0319051692570497,0.0823847159547021,0.582921856562509
"Slc22a18",12140.5619296865,2.28270628583318,0.680568913838162,3.35411
482866525,0.000796193145231502,0.00494715549576923,12.1187564498754
"Itgb2l",1.20651734405811,-0.0109343648007026,1.85922588786426,-0.0058
8113842006751,0.995307557504901,NA,NA
"Igsf5",1783.76324110783,0.854197716634677,0.245069190297159,3.4855369
4407249,0.000491150396963428,0.00341205340562352,4.85221972927109
"Pih1d2",116.541251562828,0.876020816525491,0.213861583949909,4.096204
6588539,4.1997868207654e-05,0.000506729229153984,6.64683752144562
"Dlat",6262.87826308798,-1.0936417805528,0.554857536287446,-1.97103167

755521,0.048720252588121,0.114148940755009,-2.37347737071588
"Sdhd",8224.43720760952,0.0882072880413053,0.473226467580533,0.1863955
08459793,0.852134614440915,0.905988369724028,0.00870860057906509
"Fgf23",0.473898998762002,-2.0686958498418,3.6726186301166,-0.56327543
3195774,0.573247344022778,NA,NA
"Fgf6",0.639788793777739,-0.545076952324287,3.11189966431838,-0.175158
909708511,0.860954770425797,NA,NA
"Ccnd2",4174.77206247162,-1.14856298982031,0.238356319647538,-4.818680
66900309,1.4451063095761e-06,3.66883890938995e-05,-11.7303315032461
"Gpr107",2872.40013520276,0.134130850112663,0.12037325005153,1.1142911
7395471,0.265154292159452,0.407020814716186,0.120569007689627
"Nalcn",28.9807315908501,-1.34917784703676,0.366259956855272,-3.683661
89583179,0.000229907148652997,0.00188905587207591,-8.4616091742174
"Btbd17",52.2070494435933,-1.05427860875801,0.588948723099481,-1.79010
254612595,0.0734374275728188,0.155825765895617,-1.95992162573951
"Slfn4",108.493149264541,-0.2452175230876,0.422265825331513,-0.5807183
73065317,0.561430276768073,0.689516725650283,-0.0911631267997682
"Th",6.84565442332041,-0.445406850408982,4.57146600873375,-0.097431950
6167247,NA,NA,NA
"Ins2",0.592160316975129,1.47341162443337,3.49023380387325,0.422152700
1424,0.672913563602859,NA,NA
"Scnn1g",1367.47701181577,0.995289599628335,0.386198359863824,2.577146
10693655,0.00996198324401017,0.0336635614023761,3.37536472478088
"Drp2",35.1325927915819,-1.57723761906294,0.455337610307513,-3.4638860
998057,0.000532431901474753,0.0036362021768668,-8.85905270595783
"Tspan32",369.340280841097,-1.26229461200585,0.428653219392613,-2.9447
920950984,0.00323171740114696,0.0142852019695224,-5.36289792256091
"Lhx2",6.78440634004687,-1.41457802734792,0.781230027706203,-1.8107061
6486838,0.0701863480617487,0.150368367397393,-2.68015460828284
"Clec2g",4.93393117725118,-1.64424445043489,0.874571923801313,-1.88005
62946135,0.0601004060969481,0.133756160036331,-3.3077871314857
"Gmpr",3092.4021453868,1.42062669367278,0.327734960660349,4.3346815695
5358,1.45971264371959e-05,0.000226396504312416,11.9236361454706
"Mid2",858.765760359241,-0.111552985672782,0.105227812448987,-1.060109
32924089,0.289094864045374,0.433054503829519,-0.093357766035621
"Trim25",4164.56756190698,-0.119740606210616,0.103712022633451,-1.1545
4894399095,0.248275184701419,0.38767274375817,-0.113465448770805
"Dgke",652.820684556902,-0.824666827215542,0.262679245203488,-3.139444
17868531,0.0016926866052947,0.00883877970809749,-3.89952488151478
"Scpep1",4664.32516190261,0.2930350517823,0.180518312492728,1.62329820
025381,0.104525674103156,0.203946922211882,0.465895111409853
"Mnt",1440.32572724291,-0.122122194221025,0.176411713407194,-0.6922567
207266,0.488776124023889,0.625379412442928,-0.0573237615005008
"Itgb2",1677.31094664322,-1.46881997705236,0.39078908884207,-3.7586002
7567443,0.000170866558974749,0.00150058588251565,-9.55012010762065
"Hddc2",470.70263466457,0.340292355413485,0.217028089907283,1.56796456
882085,0.11688940137358,0.222092140711563,0.512025294243721
"Tpd52l1",548.566424233774,1.22592050978175,0.39951480719906,3.0685233
380372,0.00215119525079874,0.0105705657762283,5.57754842030205
"Pemt",105.699190064301,-0.609322611058977,0.446298239265298,-1.365281

23449927,0.172164674673616,0.297436366009761,-0.738837161982115
"Cdh1",7290.49591938831,0.265510266238952,0.256918430656591,1.03344188
09908,0.301397155655982,0.446108190813638,0.2143182343935
"Cdh4",145.453986132641,0.759028018861017,0.440764834328435,1.72207027
363582,0.0850567892398801,0.174315458965649,1.32593742258478
"Ckmt1",2787.92013157938,0.86435834057346,0.26961507759193,3.205897638
5649,0.00134641874987437,0.00739923133738739,4.24086974679507
"Bcl6b",784.825908607622,-1.18566341722796,0.383469172368372,-3.091939
33349348,0.00198853484261184,0.00995186148902611,-5.46590320881013
"Clec10a",986.217265552283,-1.42458423935259,0.254128910796143,-5.6057
5431929253,2.07349910700748e-08,1.40378769404277e-06,-19.1981765520021
"Alox12",353.396215978732,-0.265363894356786,0.180619688897789,-1.4691
8586769881,0.141782382390081,0.256671124924648,-0.360884197680169
"Arvcf",3324.14906632318,0.0225918408652783,0.331540426981509,0.068142
0394820757,0.945672565441473,0.966491439405485,0.000769994045273786
"Comt",11024.1880502375,-0.672780352361872,0.415707149994413,-1.618399
76139673,0.105576473409368,0.205401125377754,-1.06487035201776
"Rtca",1213.82215112459,0.0714725028717535,0.173804151329589,0.4112243
71368545,0.680908019232415,0.785257263795684,0.0172780408943538
"Dbt",4164.25816025346,0.769913753010114,0.324801689026765,2.370411789
78188,0.0177682832462937,0.0524600222192964,2.26947775778019
"Dazap2",11227.0745748787,0.0690100436455933,0.244337578059517,0.28243
7291036681,0.777608224294335,0.856260527322017,0.0107090196200029
"Mcts1",1262.753044552,0.239565514851586,0.170938337014556,1.401473297
53879,0.161072587100007,0.282715530699263,0.302646487697026
"Rem1",1000.41286130837,-0.863976221726946,0.34528999724535,-2.5021756
4545618,0.0123432670238733,0.0397493998767156,-2.78646201386961
"Rnf17",16.9636971347427,0.755721035984204,0.81204264854561,0.93064205
1052023,0.352038754686777,0.497106868401129,0.528211406303244
"Trappc10",2406.167682534,0.0359626048427267,0.153618779246609,0.23410
2920353212,0.81490507880934,0.88218417781492,0.00450807170157208
"Ccm2",1656.86136234056,-0.466847319891991,0.148454847499436,-3.144709
16750471,0.00166252015810729,0.00871627266544124,-2.21405309630447
"Tbrg4",1677.28236977523,0.0678686238832798,0.166697392365668,0.407136
685944091,0.683907600238171,0.787458981306476,0.0162168002526567
"Tmprss2",8673.12391957368,-0.12110479297101,0.310205548890464,-0.3904
01762328798,0.69623948489878,0.797419964553361,-0.0274149530654486
"Mx1",576.865580275499,-1.09196369761182,0.490558430729409,-2.22596051
603514,0.0260168262543008,0.0705983245254027,-2.89452153392138
"Fap",123.170125963902,-1.75519784799303,0.495485699888368,-3.54237841
453037,0.000396536177331341,0.00287487747858919,-10.2709706202784
"Gcg",0.347925958796048,-0.59079951739922,4.78471990595204,-0.12347630
1437057,0.901729939384687,NA,NA
"Ndufa9",10103.1804268579,0.651257167266767,0.38359999794465,1.6977507
0582961,0.0895548231763917,0.181360608879652,1.11187046640747
"Egfl6",645.790774096417,-0.0218704936881918,0.348500544374298,-0.0627
5598142166,0.949960818474189,0.968944132591009,-0.000689977409067959
"Lck",895.887055450477,-1.16067199643476,0.521433038083769,-2.22592722
68212,0.0260190563154962,0.0705983245254027,-3.0766499791591
"Tssk3",1.16463105007857,-2.59585487840236,1.56280971662927,-1.6610178

7746828,0.0967098562721466,NA,NA
"Cttnbp2",1067.46269839506,0.819193834469551,0.434267058027706,1.88638
262867566,0.059243411740069,0.132301917547574,1.65695773970903
"Galnt1",6378.29435212564,0.260425804921388,0.15657721926532З,1.663241
98464715,0.0962640065707017,0.191604287476066,0.430307556763378
"Myf5",0.0983679749722721,0.897902326501968,4.78471990596623,0.1876603
73887789,0.851142895370425,NA,NA
"Mkrn2",1140.45359415393,-0.418839713258024,0.252612294585485,-1.65803
376255025,0.0973106518839586,0.193174820854554,-0.688639374172507
"Pparg",1083.69150316011,-3.22157250260131,0.765207417781482,-4.210064
39266025,2.55297913955808e-05,0.000346505365622777,-25.6682405496855
"Raf1",5057.97084454449,0.277768332371949,0.137582790963872,2.01891770
348581,0.0434957738567925,0.10461090399158,0.627064090293122
"Sept1",941.463176508939,-0.189643594371988,0.287202920290718,-0.66031
2207758972,0.509053497648301,0.643386601228143,-0.0836346247110744
"Pdgfb",3887.81271234643,-0.799693900012487,0.256102969557893,-3.12254
833043517,0.00179292665286437,0.00918515849937256,-3.75069738346163
"Acvrl1",2054.77761255546,-0.91912530867463,0.168401571514658,-5.45793
783518599,4.81696233612982e-08,2.64566567971434e-06,-11.8039475214158
"Grasp",710.55257702956,-0.0086408797853068,0.183358910951167,-0.04712
54968764952,0.962413206339977,0.977401567645027,-0.000197510553657702
"Acvr1b",2427.00728218392,0.196709996282547,0.13728335760947,1.4328757
6664702,0.151893343789468,0.27071448054909,0.257039101261614
"Tom1l2",4977.21390407285,0.39425065596951,0.125042169125494,3.1529415
9343824,0.00161634143278218,0.00852459433497079,1.87852546338446
"Gpa33",242.488838730676,-4.11137251254171,1.15615615759771,-3.5560702
4667363,0.000376443462650573,0.00275418185169263,-24.2350396897056
"Zfp385a",739.687398501864,-1.27330845272708,0.205477915233185,-6.1968
1415047487,5.76174664683532e-10,1.03031818478804e-07,-20.4852766752544
"Itga5",1475.63812921336,-0.898124308627381,0.23163540730687,-3.877318
75307624,0.000105613951264373,0.0010342038328554,-6.17381731877171
"Gabra2",155.965278454582,0.555819459345041,0.433179082900012,1.283117
03239221,0.199451077399064,0.330885485163529,0.614726834052118
"Wdr77",1868.3507809085,-0.486074541905496,0.161157044895931,-3.016154
47354091,0.0025600280332564,0.012045228424815,-2.14800553304323
"Adora3",58.1449293751973,-1.28656379478384,0.48203867980022,-2.669005
30745179,0.00760762480954118,0.0274133188941848,-4.62741782788789
"Atp5pb",18553.0823428912,0.750142785575678,0.405579156917619,1.849559
50714215,0.0643770635897064,0.140704403752056,1.47110052829371
"Sox9",965.155782808098,-1.19511677221095,0.404257984273317,-2.9563219
9908002,0.00311331876878693,0.0139038813552843,-5.10982602591459
"Hnrnpd",5038.18066056903,-0.127162271341342,0.107163571519374,-1.1866
1845194616,0.235378154268205,0.373243595634471,-0.125321470440083
"Dynlt1c",694.341487419074,-0.263894629194679,0.161421505393654,-1.634
81704963119,0.102087405526726,0.200493577050775,-0.4240715617495
"C1d",2833.40203798203,0.209971030529733,0.261085649155662,0.804222795
12018,0.421268316242766,0.564334567509547,0.120126106031177
"Gm2a",10390.3589926822,0.0871213816303156,0.243391054120008,0.3579481
66769346,0.720382103738685,0.815112015941091,0.0178102007254395
"Krit1",2983.42185507163,0.171222648884748,0.173195892236278,0.9886068

69793205,0.322855523749834,0.467997695492846,0.130007971633906
"Clcn4",7086.99218622655,0.313419539300713,0.145639433968013,2.1520238
7678566,0.0313954738644381,0.0814027858256402,0.78616457960726
"Vmn2r88",10.8067639058463,1.63862966896021,0.571652838838066,2.866476
92031211,0.00415068423153827,0.0172475467431484,6.65297633499011
"Grm6",2.07108040914748,-3.87790324472109,2.46624860558016,-1.57238943
225225,0.115860263586862,0.220372980140146,-5.86507195543065
"Sema4f",75.0664377332502,-1.7277170184952,0.389633427207674,-4.434211
48661953,9.24098797780817e-06,0.000160732188752278,-15.0929402296671
"Hk2",2764.46357715153,-2.43884514055359,0.802127863783798,-3.04046929
506858,0.00236209775158254,0.0113410938094943,-10.9243739839711
"Myo18a",9015.8919013267,0.313555217271815,0.175629619650258,1.7853208
2399436,0.0742092930203953,0.157019294119552,0.580511924074978
"Sez6",12.9656243065672,-3.356537141052,1.08246903681306,-3.1008158449
8167,0.00192988272613629,0.00971819560800759,-15.5533718166019
"Haao",5051.2808454667,2.07945881587368,0.730285688647589,2.8474593548
7879,0.00440697213426528,0.0180291948539455,8.3506135621058
"Cd52",2774.91199693763,-1.55532987717544,0.472043312703101,-3.2948880
6497232,0.000984608912992978,0.00580347780699428,-8.00885690026443
"Abhd15",149.298848524204,-2.05517895729648,0.531068229121218,-3.86989
626680789,0.000108881671551922,0.00106042698897072,-14.0760925576294
"Hoxb6",4281.83580414695,0.398280890696409,0.326271550322423,1.2207037
0616999,0.222198224017164,0.358021731417789,0.40909883380643
"Loxl3",756.227869010941,-1.02710102240916,0.331042708495074,-3.102623
90939941,0.001918132307214,0.00966901337281762,-4.76454601322166
"Btn1a1",0.186490667865646,0.895501585821939,4.78471990596623,0.187158
622327152,0.851536266129833,NA,NA
"Kat2b",2861.11732997819,-0.0879026884823841,0.217560708209084,-0.4040
37517647286,0.686185130525489,0.789382127263318,-0.0207894039723735
"Rab5b",4947.78591578389,-0.120487058712475,0.184573938183215,-0.65278
4785861127,0.513895033259919,0.647700411021454,-0.0523307849431582
"Dnmt3l",104.153058333975,-2.81465091096293,0.765573359712679,-3.67652
671720353,0.000236431103523312,0.00192642442812267,-17.5974497505996
"Aire",20.4253345418433,-1.96235110538171,0.629592744927788,-3.1168578
7549343,0.00182789670992044,0.00931704778119934,-9.17577610169149
"Icosl",1905.84469026996,-0.864430884791239,0.169945345255555,-5.08652
28670509,3.64687767596144e-07,1.24452665318564e-05,-9.76302954380248
"Spg7",4024.9324702297,0.823453721313967,0.187074175679522,4.401749831
70649,1.07381327799037e-05,0.000180961203926156,7.09588837559059
"Sult5a1",680.723612093953,-0.824652413391311,0.394698597771952,-2.089
32187255395,0.0366787587663693,0.0917370979391868,-1.9699531249072
"Rpl13",24673.5041541236,-0.337480042519338,0.181545181038484,-1.85893
142736628,0.0630368587703868,0.138570739138956,-0.666986893542913
"Chmp1a",4374.70989280986,0.0179269202328803,0.119001491987434,0.15064
4499774619,0.88025615619302,0.925249003311348,0.00139278518728066
"Rpa1",2187.59537461526,-0.468528471648509,0.287811818776439,-1.627898
65141863,0.103546377431746,0.202603840156969,-0.748006985460754
"Serpinf1",764.786461559001,-2.30360796936184,0.383720377899928,-6.003
35062205794,1.9328659335481e-09,2.50697739369696e-07,-35.0125786436496
"Tubgcp3",2552.06771071473,0.266921035344853,0.181968790859711,1.46685

062907648,0.14241669345933,0.257591900655846,0.36204601407709
"Polr3d",932.621618663783,-0.0466030610890037,0.131872114691099,-0.353
395872949849,0.723791679394151,0.817925707941762,-0.00936645882224342
"Tcf7",463.709948964805,-1.66457578670866,0.396148783691485,-4.2018954
8784531,2.64689335272928e-05,0.00035460820897817,-13.2242173681248
"Ddx3x",9345.89113139302,-0.491267296146387,0.178095704356785,-2.75844
55106352,0.00580769866461278,0.0222516228262173,-1.86943925828451
"Il12rb1",81.4702187578055,-1.34950960209105,0.522480985615049,-2.5828
8749111597,0.00979772683421214,0.0332467939111501,-4.59345665444113
"Slc5a5",22.095445050554,-0.894349177746159,0.475068711247313,-1.88256
805083628,0.0597589295339545,0.133163383777491,-1.80316754362646
"Kcnn3",93.0099389773433,-0.582471877211722,0.348410073109087,-1.67179
976174039,0.0945638138314576,0.18921382494549,-0.969744354495799
"Usp32",1971.43044879878,0.0513545944999302,0.158349264996414,0.324312
174742921,0.745701703278409,0.834750936417638,0.00927576332124447
"Car4",4756.48999164366,1.83136214696052,0.552854604928248,3.312556557
61102,0.00092447431772232,0.00553265814823505,9.51774822520531
"Txnrd3",973.968527741737,0.302515386594053,0.157351199402568,1.922548
97161665,0.0545367177675258,0.124122437613076,0.631194361055617
"Fasl",45.2395501922457,-0.343230008258306,0.442774466651034,-0.775180
219524304,0.438233174814121,0.580362048667101,-0.186752528100599
"Zfp512b",3127.06167664411,0.0976129028766363,0.169396450967222,0.5762
39362272853,0.564453402805635,0.69222670909518,0.0359061022582527
"Dnajc5",5550.41654091996,-0.0213299857279327,0.12934737639561,-0.1649
04664650442,0.869019020808784,0.9178836873093,-0.00182765126666029
"Tpd52l2",3245.89486965746,-0.158161744970349,0.119763642707181,-1.320
61568431958,0.186629540469452,0.315356857619671,-0.182526625055642
"Fmr1",2321.95556974363,-0.173742267311008,0.103462472110667,-1.679278
13599087,0.0930978500511321,0.186841921725811,-0.291451325674447
"Bcl11a",104.486330080205,-1.20916492950178,0.494703823767505,-2.44421
989766153,0.0145165755054728,0.0451795319765662,-3.74491815763529
"Il4",1.21821802328777,0.819097540275203,1.59931944211602,0.5121538066
16316,0.608543369456985,NA,NA
"Pxmp4",4732.18191308243,1.3102530913756,0.341339980262888,3.838557353
77523,0.000123759308514734,0.00117366682753905,8.8410930964688
"Dlg3",1228.17646785754,0.0518791781819874,0.146851359916614,0.3532767
97786863,0.723880938217725,0.817960092462874,0.0104246918298964
"Gnb1l",237.046910723533,0.361176117748386,0.248605778361301,1.4528066
0863597,0.146277456559384,0.2629407069231,0.482468709141579
"Dbh",11.1368597916436,-0.652204723857303,2.82382462652228,-0.23096502
4432319,NA,NA,NA
"Mmp11",1208.17584973949,-0.415424471489009,0.363425337857154,-1.14308
065017826,0.253005107348233,0.393357274784774,-0.387606395935483
"Smarcb1",3090.61004023555,-0.204735046742042,0.083310972653822,-2.457
47997196922,0.0139915612093904,0.043944771345058,-0.639760502516738
"Vpreb3",10.0511757072653,-0.900541999314855,0.836253463902363,-1.0768
7685395345,0.281535284852128,0.425466980626785,-0.769574317029749
"Hip1r",4823.56767338792,-0.275838143283694,0.151249829202702,-1.82372
532080034,0.0681936088413768,0.147132785790546,-0.528621678071112
"Nsun5",808.822785019863,0.154933311226495,0.208589023161187,0.7427682

86070214,0.457621979138212,0.597444061650789,0.0798053151067688
"Top1mt",1201.52495076398,0.457088608796092,0.148257803664665,3.083066
1017342,0.00204879640614268,0.0101776897648331,2.09692015305508
"Hoxa10",1931.75580459621,-0.04246310671731,0.182555176154454,-0.23260
4232932751,0.816068740059785,0.882553161880229,-0.00530518139721126
"Hoxa4",122.412126281276,0.235141832578588,0.176355872851396,1.3333371
2553325,0.182421195536899,0.310451991845897,0.275051516476621
"Gm38393",81.7637428599818,0.0824122987184885,0.337597584324789,0.2441
14005979388,0.807142525434427,0.877166132549578,0.0108008628898811
"Mmp14",4584.78648978734,-1.37585206612278,0.281117462297692,-4.894224
8371103,9.8693956371029e-07,2.75589540336503e-05,-14.4453227124081
"Slc7a7",3413.24169902484,1.54753712865381,0.408733735813872,3.7861742
0843021,0.000152984442392942,0.00138032885598222,10.1912028600011
"Oxa1l",2751.00872781474,0.295785978953865,0.149737335778485,1.9753655
7877215,0.0482266760753123,0.113278367680573,0.644194371439946
"Heatr6",1053.80967946087,0.168856148422899,0.207446824752051,0.813973
164567463,0.415660324456282,0.558972688202537,0.098215966500599
"Ccl3",41.976155756262,-0.904004362780091,0.562642064131751,-1.6067130
7818963,0.108117323374665,0.209009673920208,-1.41510559560138
"Wfdc18",31.0974115561494,-2.06988286008994,0.827217266460537,-2.50222
395495499,0.0123415827493318,0.0397493998767156,-6.67570451333931
"Icam4",14.3937273423548,0.286572645814346,0.432965726355989,0.6618829
81422699,0.508046219437822,0.642655065952942,0.126707276765168
"Ilf2",3310.20660097119,-0.00308350984014851,0.122948996914996,-0.0250
795851736827,0.979991483731973,0.987078931181217,-4.0101884014707e-05
"Chtop",6413.87896957186,-0.192167271149622,0.0813632699486267,-2.3618
430192267,0.0181843404712281,0.0534295163514174,-0.562933256209399
"Snapin",1849.78042485094,-0.380939511522025,0.161323349089267,-2.3613
4145288067,0.0182089562006124,0.0534535121950526,-1.11575009045979
"S100a4",1068.28395810464,-1.16043205975441,0.424026587260182,-2.73669
645871138,0.00620595267041274,0.0234821783752586,-4.35337655256262
"S100a3",18.3462434429296,-1.69984341617343,0.539741027621554,-3.14936
854747551,0.00163623702041804,0.00860856703294952,-8.08275103271808
"S100a5",9.44328753301004,-3.49648949477938,1.40159899562363,-2.494643
26508286,0.0126083817141992,0.0403807862456399,-11.2216375632767
"S100a6",12719.2056180976,-1.66833235585261,0.461772932435162,-3.61288
468567149,0.000302809390109121,0.00232265046748531,-10.1185129862277
"Scn4a",12.3942116711062,-0.21085896239773,0.509061885339784,-0.414210
862117457,0.678719687562422,0.783570906194209,-0.0514271771541834
"Icam2",679.048066967263,-0.634152528692341,0.209114064581721,-3.03256
75604881,0.00242482811111657,0.0115597306871762,-2.82846468272127
"Mapk7",1101.61089029212,-0.446639075271495,0.141645454508909,-3.15321
855417113,0.00161480858479041,0.00852111983389536,-2.12832787613383
"Epn2",2268.75693184136,-0.401812654192615,0.107646596888865,-3.732701
87637653,0.000189436760087318,0.00162429109418756,-2.58071562797893
"B9d1",539.542332117059,0.574551776270262,0.292995732586996,1.96095612
450487,0.0498841413235174,0.116177853263968,1.2367991392384
"Sec24b",2718.04338613845,-0.378073441653981,0.14632249093977,-2.58383
683346128,0.00977080067416747,0.033166969280104,-1.28779405923305
"N4bp3",1029.28800820778,-0.170180489642998,0.166178535049127,-1.02408

225943675,0.305796423021364,0.450367751043928,-0.135751411850411
"Rmnd5b",1858.96219223906,0.339901822491627,0.17878619978271,1.9011636
4073251,0.0572805818832266,0.12907162666811,0.695910838864583
"Nhp2",2858.62486296234,0.760522937982889,0.271691131353524,2.79921885
633175,0.0051226404029331,0.0202434622165362,2.96598119527896
"Vps9d1",1869.47839622225,0.17074179193854,0.187062390183393,0.9127531
8235348,0.361372366438189,0.506253657192974,0.116226914912262
"Zfp276",2267.15915097122,0.341649575370551,0.198549417149012,1.720728
17073113,0.0853001619727631,0.174722403418945,0.596027100414465
"C1ql4",25.3043882020659,2.71338632644731,0.571420829484243,4.74849040
574243,2.04940608302667e-06,4.84943201539489e-05,26.9549530901953
"Mfsd10",1471.7332311885,-0.0658022293296196,0.239075775124902,-0.2752
35871535885,0.783135029314093,0.859821318856139,-0.00993815544731669
"Luzp1",2763.92246888364,-0.210833849408941,0.206107550925334,-1.02293
122431657,0.306340364979009,0.450760277440248,-0.167996547010968
"Slc13a2",1218.04530314303,2.05783313307384,0.598835937829687,3.436388
83887276,0.000589524216039322,0.00390970855929752,11.4092286928275
"Kctd10",3552.09818749627,-1.06671116164101,0.150344273677879,-7.09512
33162794,1.29236195942449e-12,8.99945481602099e-10,-22.2181928310232
"Poldip2",3703.45258729168,0.185541310866728,0.278823917896957,0.66544
259282411,0.505767449978533,0.640562113652578,0.0826418041549994
"Ift20",2968.29399951477,0.44337594417954,0.159956118235623,2.77185986
425618,0.00557370258472868,0.0215303185579815,1.70180685904507
"Col6a1",3993.24749026653,-1.78184282837884,0.303761633281449,-5.86592
457095423,4.46636818141209e-09,4.48564132124303e-07,-26.045578245388
"Pcbp3",1074.43730238531,0.50588120612412,0.149547362536214,3.38274910
065109,0.000717641624590269,0.00455784247435214,2.72715798335268
"Lgals9",1525.88943516462,-1.2850425146553,0.233591311481453,-5.501242
77527903,3.77123499471165e-08,2.16994144780817e-06,-16.7579957553162
"Araf",5715.84752675683,0.0927295966504538,0.112541646904712,0.8239580
56424813,0.409963390771012,0.553513680875572,0.054846664192812
"Cfp",1533.13144371843,-0.921497847109572,0.302695057582249,-3.0443108
4692944,0.00233214034038357,0.0112443304542035,-4.13558211294672
"Timp1",1102.19386474181,-1.61128757871914,0.553643300115741,-2.910335
18943027,0.00361041325463203,0.0155639702937907,-6.70746250669791
"Uxt",617.869889993146,0.402162252033266,0.213128840053121,1.886944309
99122,0.0591678164642066,0.132148234297721,0.813908432441443
"Cnnm3",1438.53469974037,0.168374029038452,0.0955846443282845,1.761517
55568785,0.0781508442878525,0.163389737169669,0.305029237629648
"Lman2l",2302.43508254467,0.215568591175698,0.191848666140038,1.123638
72792499,0.261166319541355,0.402674434478676,0.196086988987422
"Mcm3ap",1772.32949940005,0.482863561455257,0.23159120601806,2.0849822
8303886,0.0370709087679843,0.0924899013638078,1.14953194536683
"Pcnt",1817.83320244949,0.00581719340522996,0.198793342164803,0.029262
516248695,0.976655221791381,0.98525471005355,8.64148870895199e-05
"Ftcd",989.199226756608,2.01021268051152,0.835448628031915,2.406147563
19251,0.016121751261634,0.0488108549843695,6.07044538398702
"Mxd1",1072.01686397006,0.0560182628462827,0.130058126940679,0.4307171
27518168,0.666674063594731,0.77429180914761,0.01432983362844
"Gmcl1",1601.53047792275,0.292636878554235,0.122907920379721,2.3809440

2419421,0.0172683357279389,0.0514121255189118,0.868511498863534
"Snrnp27",2469.64282035595,0.609530962399967,0.189191774266444,3.22176249344514,0.00127404698852644,0.00707181383926476,3.01817684481829
"Oas1c",248.300271154822,-0.00228695051620246,0.161339629677743,-0.0141747599196203,0.988690556631074,0.992458878882467,-1.73115273455919e-05
"Ocrl",1694.0665233435,0.0429279263876031,0.188566542976918,0.227653992643105,0.819915241794421,0.884533551143454,0.00526703479240913
"Calm1",18110.2427602744,-0.437486809166624,0.174247182135676,-2.51072530301224,0.0120483410895286,0.0390879458508533,-1.4183064891239
"Agpat3",11905.2559705021,0.425751940326123,0.365824950655643,1.16381329256814,0.244499684071207,0.383620732278136,0.407913313297835
"Slc26a3",42.0164670300753,-2.51063233668783,0.907552829719152,-2.76637596674649,0.00566831430291013,0.0218296615189979,-9.60187725334021
"Sema6b",543.764043861513,-0.640930748057531,0.230431095317734,-2.78144209302036,0.00541179843685539,0.0211029881450346,-2.47292914642545
"Uhrf1",482.633202214672,-1.05524336487172,0.405157518177612,-2.60452618432994,0.00920013907888601,0.0317157552616901,-3.64158334220371
"Dpp9",2045.16577635707,-0.0597626871090272,0.159681481861066,-0.374261851859722,0.708209510882422,0.806418509783654,-0.0128580872548958
"Ramp2",1619.65030742146,-0.423879018727231,0.174736066404551,-2.42582443023446,0.0152736572675276,0.046765981375982,-1.29817155808365
"Lsr",2778.69896217204,0.146591894986226,0.242209552892811,0.605227552899616,0.545027810823476,0.67502713939123,0.0576109639719267
"Gramd1a",5243.358286677,-0.177297372453652,0.224046064919412,-0.791343389661516,0.428743635011356,0.571404196544867,-0.0992259717507951
"Hpn",10692.2390363735,1.8961905626603,0.562161571708867,3.37303483213239,0.000743445471240282,0.00468056176888699,10.1718054601173
"Gabrg1",14.1253195890252,2.80150168235951,0.661826192675027,4.23298701889714,2.30607820546048e-05,0.000320941562098989,22.5359841132152
"Ckb",6535.92529796126,-0.0764062322052544,0.189473776805339,-0.403254917348021,0.686760703247706,0.789805024885296,-0.0180295150377459
"Sp1",2984.92740529005,-0.429224228940702,0.154280938485598,-2.78209500897461,0.0054009226487342 6,0.0210701770453112,-1.65676116273519
"Itgb7",1253.05350775374,-0.949242255059971,0.512439016837201,-1.85240042984771,0.063968343634244,0.139983699683557,-1.86642792898556
"Myg1",2068.19691639041,0.4995473830335,0.182233828937123,2.74124396083376,0.00612070432375791,0.0232136730022625,1.87980370628206
"Rarg",2581.44631592321,-0.996141786833678,0.1785564231369,-5.57886280052739,2.42096093860405e-08,1.58402336848663e-06,-13.3040139818858
"Pfdn5",8556.39475790493,0.554437626250603,0.133707524973227,4.14664489797132,3.37382698944347e-05,0.000426331034609001,4.30259922660925
"Efnb2",2184.14545004103,-0.914678076755601,0.152932652300699,-5.98092077130229,2.21879782981533e-09,2.73810886618604e-07,-13.8215432429347
"Rrp15",463.338711371809,-0.461453915856861,0.189794210167974,-2.4313382133652,0.0150431649588933,0.0462782629170876,-1.41808614329403
"Rnd2",533.598567189821,0.224432826063579,0.175637897130176,1.2778154927307,0.201314490398389,0.333126532613748,0.246703942513586
"Srr",2831.70510351417,0.894799259129837,0.280302210620083,3.19226615141696,0.00141161195721436,0.00767315582430043,4.35769681806752

"Sync",89.4240174770499,-1.13940403300232,0.479575494876364,-2.3758595
7826319,0.0175081242652609,0.0519041214724126,-3.37076398819912
"Fndc5",285.034629097389,-0.310880242003483,0.329439480643309,-0.94366
4194092387,0.345341276812678,0.489991574391907,-0.221771731445082
"Acp5",4094.10762982287,-0.694401686173877,0.492721910488139,-1.409317
65239734,0.158741261404568,0.279760113951345,-0.884544685561019
"Cnn1",620.560751558695,-2.58027595768542,0.597571630549742,-4.3179358
3525989,1.57495198231051e-05,0.000240284927629815,-21.5032074065074
"Fbxo9",1549.83824199307,-0.0442454120628994,0.13695804929803,-0.32305
8135609237,0.746651214621576,0.835286326392306,-0.00796332054684393
"Vps50",1674.02490289988,0.469062859517617,0.122623357052385,3.8252325
7226788,0.000130648689379025,0.00122487520347775,3.14502721948726
"Hars",2362.33131432386,-0.0866029204582806,0.158475902256991,-0.54647
3749162454,0.584740327560085,0.708681433787079,-0.0298216438722337
"Zmat2",4452.03608913633,0.328007877275191,0.103468953094095,3.1701091
7252541,0.00152381672864616,0.00814903416762009,1.57767061253818
"Ube2c",232.038259392124,-0.595366110645377,0.415456555764304,-1.43304
059686842,0.151846235920513,0.270680006214861,-0.778035117032579
"Smg5",4044.8745358234,0.012095194712645,0.202539947207589,0.059717576
1098047,0.952380573006062,0.970244861929682,0.000365357180405199
"Cct3",6359.19759113156,0.261618711938604,0.158017698835755,1.65562917
234058,0.0977969386916178,0.19397296989317,0.429064226216849
"Glmp",5760.95917008629,0.830197743596968,0.152684628939298,5.43733674
675939,5.40828647118984e-08,2.89699916525438e-06,10.5865447494204
"Mef2d",4025.73790798407,-0.477107635889017,0.178690436090239,-2.67002
334499915,0.00758459734479712,0.0273657437137036,-1.71685430702015
"Tmem79",751.027874674376,-1.91779324426575,0.521184668325098,-3.67968
085175807,0.000233526027545407,0.00190993728401021,-12.0066991540302
"Snd1",3827.36526406118,-0.664797927437427,0.250685859888928,-2.651916
33757078,0.0080036370841103,0.0285171949891639,-2.36485112482928
"Col18a1",13911.0050255341,-0.672940087876277,0.326188268932293,-2.063
04196677275,0.0391086410994,0.0963170272018828,-1.57475393783152
"Slc19a1",2219.09782462406,1.26384652852306,0.297559833499295,4.247369
39008286,2.16295181668188e-05,0.000305381857506614,10.2295075417036
"Kpnb1",7614.21252814498,-0.509929278651929,0.138787881199713,-3.67416
286093562,0.000238630496662756,0.00193948204415607,-3.18467882937954
"Npepps",7285.33534278976,0.518098581149413,0.2526442372955,2.05070413
121448,0.0402957684184264,0.0984200040864935,1.20121736414731
"Tbx21",108.630294536965,-1.70117466611229,0.359337378298908,-4.734199
0253438,2.19921846555656e-06,5.1538896203632e-05,-16.7959930904142
"Mrpl10",3056.80119790014,0.445343379152969,0.191386875103488,2.326927
48085343,0.0199691252952422,0.057393110326336,1.27271611994051
"Cyp51",7764.97015321947,0.634245842597055,0.398571135757605,1.5912989
8202859,0.111542305808681,0.214102370413237,0.97756375900345
"Tcf25",14170.0466153477,0.00199520855328345,0.143154887639007,0.01393
74113325055,0.988879914696821,0.992596168687702,1.48271443824898e-05
"Tubb6",838.797094869698,-1.73367637566186,0.275895686007239,-6.283811
10539137,3.30372092820629e-10,6.54259992164739e-08,-28.6790746592367
"Def8",4897.66197742964,1.31263000439881,0.236518822101557,5.549790890
78664,2.86011488645919e-08,1.77036571606925e-06,17.3848976131166

"Meox1",417.598802308209,-0.594849707261567,0.158830417186473,-3.74518
758937207,0.000180259034587664,0.0015606974495163,-3.84428908714987
"Sost",19.9855381284495,-0.132189239516528,0.405569264387649,-0.325935
05258865,0.744473494428669,0.833806892663001,-0.0240258499388718
"Nkx2-1",8.09128512495222,1.11532390866868,0.633258168333181,1.7612467
7807845,0.0781966437771029,0.163435501683454,2.02022729723214
"Pax9",4.26723890429503,-0.986252375307709,0.867612957501368,-1.136742
33052951,0.255646017835639,0.396451607075422,-0.912481975430287
"Irx2",1185.61447463097,1.56231164050899,0.575137380130126,2.716414711
48252,0.00659931960423708,0.0246029701039033,5.78818981697556
"Col1a1",16411.4105119455,-1.94187037127631,0.425351965399995,-4.56532
596352342,4.98718797528734e-06,9.92246848389312e-05,-17.9004013607781
"Itga3",4915.67420730406,-0.455191642804099,0.296243360610523,-1.53654
631066162,0.124404448795687,0.232764412495759,-0.663545804320169
"Sgca",27.5220731735356,-1.48343170900213,0.565763403551283,-2.6220001
1469577,0.0087415401579721,0.0304797120887232,-5.17820615729548
"Dlx3",3.30603040868842,-1.78143305894205,1.62039673990267,-1.09938079
673565,0.271602003334725,0.41412809043458,-1.57047567740784
"Foxm1",264.411424808933,-0.265098531446807,0.399930896442467,-0.66286
0843723143,0.507419681572017,0.642070799616535,-0.117453680940768
"Itfg2",1041.03803588602,0.423027330484469,0.268994134748811,1.5726265
9603897,0.115805305605987,0.220332928249955,0.639877725062295
"Nrip2",449.338420965807,-0.134182943577138,0.298972910990045,-0.44881
3048422324,0.653566525077716,0.764029024879508,-0.0361152721531617
"Tulp3",2194.3462396646,0.812202078443623,0.209531691556311,3.87627319
004078,0.00010606858943448,0.00103717420099975,5.58084789977851
"Gtf2h4",798.306751189549,0.309354186744487,0.211524798290564,1.462496
07253868,0.143605319814231,0.259284797271773,0.417575010635794
"Tubb5",8678.07666145165,-1.07464642752266,0.204672919245362,-5.250555
03915678,1.51641584816045e-07,6.30993576912789e-06,-12.8671554808151
"Ell2",1785.97735949917,-0.186302365471846,0.299831118650111,-0.621357
670646762,0.534364316505377,0.66647703212575,-0.0755921103407264
"Jup",7429.27231723551,0.104102192987534,0.119231202829514,0.873111991
802913,0.382602038235791,0.527429638902544,0.0665983170057918
"Fkbp10",1071.39425254778,-1.31322133635843,0.251852951773161,-5.21423
84161581,1.84573955221553e-07,7.2304801684033e-06,-15.5448703141765
"Klhl10",15.6382803916264,-1.00744401182332,0.473470853520599,-2.12778
464467716,0.0333549430717318,0.0852365242480507,-2.48065482691388
"Nom1",1755.84137447102,-0.292356762246741,0.15150920211123,-1.9296303
9982289,0.0536526493447071,0.122640018396612,-0.613511219419413
"Ergic1",3769.0650404124,-0.472232170292089,0.281630305563674,-1.67678
037825841,0.093585436071477,0.187497824961124,-0.790511002266223
"Tnk1",776.050745746607,0.0655181924660997,0.219804700861182,0.2980745
73516413,0.765646247368693,0.84835884134766,0.0107745696625342
"Acap1",688.672497543357,-0.893335710273714,0.405992570765141,-2.20037
452554887,0.027780333689097,0.0741290469782418,-2.32441290966682
"Tcea3",1959.97969627843,2.22602282564834,0.493156796333024,4.51382368
082611,6.36691558160217e-06,0.000119482309923079,20.106199976759
"Csn3",7.39322482437066,-0.53963564655123,0.920583964382192,-0.5861884
05870596,0.557748912126612,0.686782595104151,-0.202761344588071

"Ifrd1",2165.0935638099,-0.78637452250791,0.293319780793309,-2.6809461
0046786,0.00734143396576496,0.026695874573757,-2.84922852865304
"Stk38l",1151.0218745765,-0.150221221458198,0.126315999033817,-1.18924
93635583,0.234341554722973,0.372114311750836,-0.148501816736707
"Brpf1",2102.32755742913,0.000793467703015886,0.164521005855464,0.0048
2289601191089,0.996151900651752,0.997430789241917,2.04120903113396e-06
"Akr1b3",7730.09915487297,-1.28674408072251,0.511025438493211,-2.51796
482875012,0.0118035106979639,0.0385375074765853,-4.18979737177725
"Hoxc13",1.20222737581642,-1.07281190395929,2.17887322916254,-0.492370
042277142,0.622457773299917,NA,NA
"Hoxc11",109.539970516601,-1.05447334316227,0.506540952120783,-2.08171
390436924,0.0373686098240993,0.0930184034661418,-2.504329816373
"Hoxc8",1923.03632365839,-0.423381721528558,0.257742685529039,-1.64265
271256692,0.100454807808882,0.198008330262216,-0.685643910400889
"Hoxc6",1689.48017292182,-0.365307711823718,0.209239873319877,-1.74588
000856439,0.0808318043796989,0.167576663667769,-0.652554403737012
"Gstt1",3768.14034452949,0.823264336975059,0.22642743062186,3.63588605
282517,0.000277026774761433,0.0021692642788347,5.04938249033017
"Gstt3",1954.82908823293,0.472291475488641,0.262438730610204,1.7996256
6649558,0.0719197655210918,0.153122034082796,0.886264431286955
"Ddt",3819.3167094406,0.663440888038373,0.483458008699542,1.3722823411
7575,0.169975560722829,0.294412485406923,0.811237925667762
"Tat",8.54718548236804,0.882744684866886,1.00275085953013,0.8803230398
43136,0.378684334718215,0.523505611815485,0.571319006167595
"Marveld3",784.722045637748,0.678748714434433,0.217375640765828,3.1224
6906802969,0.00179340949877482,0.00918515849937256,3.18344434941597
"Ddx18",2305.33825221798,0.147437376824791,0.290896062800383,0.5068386
81161402,0.612268013096429,0.73173191136267,0.0460507485384132
"Ubl3",5277.06009894882,-0.192559148596935,0.156584229774146,-1.229748
03321305,0.218791473686425,0.354259430217546,-0.199823592776456
"Gramd3",1296.14029197691,0.287204503946884,0.147596669307048,1.945873
88248855,0.0516698909286641,0.119240091528831,0.610773763032941
"Eef1e1",767.537448246654,-0.0263181558435461,0.21148246385989,-0.1244
46043247265,0.900962119420778,0.939378270034268,-0.00164585949645934
"Akt1",5179.97297508702,-0.491300827632412,0.104712722961949,-4.691892
3864767,2.70689507967775e-06,6.07868198490612e-05,-4.76961602354429
"Cldn15",849.729951890923,-0.777691031732352,0.278706549756877,-2.7903
5793170542,0.00526498007285724,0.0207052403107242,-3.01539467467448
"Il16",724.854541024213,-1.01713270900996,0.295259578485561,-3.4448762
4830669,0.000571320893610247,0.00382148020021015,-5.66249724765136
"Tcirg1",6094.18714850258,-0.444815717955255,0.301802707024202,-1.4738
6258506815,0.140518593351437,0.255010380972293,-0.607818893716968
"Naglu",1728.00255238362,0.344018983919519,0.127075568326978,2.7072000
4205941,0.00678533575426982,0.0251809053172351,1.26656413074712
"Coasy",6164.0657761175,0.627121085522205,0.430740071641887,1.45591535
779747,0.145416019592097,0.261802543860269,0.840445546927212
"Smo",2701.95729198841,0.0734901693849754,0.170949783171565,0.42989331
7332968,0.667273246844626,0.774716532526774,0.0187589596109345
"Tspan33",2152.58747820992,0.732948706311823,0.464427043538625,1.57817
835224935,0.114524642001352,0.218504221385041,1.11477829440115

"Crnkl1",1239.56079387802,-0.116251391430517,0.0847102913671377,-1.372
34082842047,0.169957361155947,0.294412485406923,-0.142149118844525
"Rin2",3924.49587164456,-0.232798787997707,0.12163101279118,-1.9139755
779012,0.0556232763422313,0.126094947345753,-0.482061130829861
"Folh1",1699.86393419131,1.45584915765988,0.603710543369285,2.41150195
843001,0.0158869665291511,0.0482048044483953,4.41456642983398
"Chordc1",3616.72362876342,0.0490967502308793,0.189285980313018,0.2593
78693285626,0.795343067610921,0.868821734118311,0.00690385313696752
"Rtcb",5433.43855054558,-0.26167750676573,0.20745768968131,-1.26135361
464649,0.207181472474512,0.340293517842475,-0.282074416924355
"Pwp1",784.020067876004,-0.408932730510094,0.121480531397297,-3.366240
87667758,0.000762001366059801,0.0047681330665706,-2.18607277124384
"Fbxo7",2569.21142137596,1.08384512012394,0.224116227132623,4.83608498
139928,1.32421326462552e-06,3.42450882859351e-05,11.1440602039566
"Capns1",12257.5489289093,-0.196086550261459,0.132831458445666,-1.4762
0565606954,0.139888691617366,0.254174793509961,-0.268586235458682
"Lrp3",1036.28711977445,-0.0333830719391684,0.153405029499951,-0.21761
3933832457,0.827729927033207,0.890186327484472,-0.00388326853232774
"Dsg4",0.0837058623428615,-0.544048378937457,4.78471990596623,-0.11370
5376621746,0.90947135024012,NA,NA
"Evx2",0.580171029588956,0.801017442018288,1.94540961265277,0.41174744
7328595,0.680524544012272,NA,NA
"Hoxd13",0.709405946815883,-3.11408841894463,4.72902282883555,-0.65850
568535137,0.510213244983034,NA,NA
"Hoxd12",0.212952144137468,-0.539944266952227,4.78471990596146,-0.1128
47622758333,0.910151361951238,NA,NA
"Folr1",7404.08304283277,1.84734752898847,0.665674991086616,2.77514936
52675,0.00551763606127046,0.0213825046630005,7.10338789190226
"Clpb",2481.10523081061,0.402804932810123,0.360843997310515,1.11628553
006938,0.264299933431795,0.406221039102407,0.362869980199186
"Sept7",5375.12129924478,-0.511854066600727,0.126357325625938,-4.05084
599618699,5.10327882492971e-05,0.000588068177193943,-3.80751229656027
"Zdhhc4",1346.52222105745,0.110101975367218,0.109879677737389,1.002023
10048961,0.316332436050339,0.461596305800741,0.0851159358967632
"Rac1",11505.3760342588,-0.213547441813539,0.143244956833112,-1.490785
06172007,0.136017934974236,0.248531318163964,-0.297297846929241
"Nup214",1490.29678419714,0.495631715623904,0.211927075705346,2.338689
91951272,0.019351487244592,0.0560230583957024,1.42840659770638
"Aif1l",6886.90349282946,0.66645717880684,0.475545850975884,1.40145724
631848,0.161077383861838,0.282715530699263,0.841944736876322
"Cpa3",3.51264940624352,-2.2662747920958,1.29007371445392,-1.756701781
22737,0.0789686537785688,0.164624271503746,-4.08856265651038
"Ltbp1",1564.87801259584,-0.16627403550753,0.171100002872407,-0.971794
463565992,0.331152810732664,0.475955882623137,-0.123446850906131
"Ugp2",5322.70650172454,0.140292685041449,0.342225964086534,0.40994167
5278544,0.681848732932779,0.78601670774053,0.0337792841091865
"Kcnh6",1.71273075447124,-1.09152020035399,2.07985453303944,-0.5248060
29948102,0.599718039290149,0.721497027832377,-0.356301686495272
"Trmt1",2717.8278201142,0.535003661998455,0.23629780229854,2.264107650
57615,0.0235674945528662,0.0653096942569335,1.459818908306

"Nacc1",3865.02543329007,-0.0814193910212388,0.0853493880154593,-0.953
954010853497,0.340106921993704,0.484787247973772,-0.0589513150430036
"Nfix",4097.47173859899,-0.689618815578165,0.279541276521422,-2.466965
96710045,0.0136263307880683,0.0430261048916203,-2.1695051108561
"Slc1a5",13165.4133855455,-1.70951320677273,0.499562416198413,-3.42202
12556858,0.000621574541572496,0.00407925291537547,-9.40547056274967
"Uba1",7296.23484205156,-0.165388437983845,0.134330176115653,-1.231208
37600519,0.218244939498668,0.353668536431602,-0.171903939024763
"Vwf",735.069387954568,-1.12118917071403,0.343391620037082,-3.26504522
909718,0.00109446598713314,0.00630425341717043,-5.68053938511075
"Siae",1087.8427527896,0.106655592742171,0.129156460316942,0.825785969
052148,0.408925514727334,0.552662769614041,0.0632475433052309
"Vsig2",841.408078843317,-2.48839807399098,0.557525814181399,-4.463287
63744263,8.07115884273862e-06,0.00014371822384997,-22.016490035768
"Esam",2533.82171836424,-0.517168607155751,0.218812234349976,-2.363526
92385825,0.018101911281994,0.0532274266590738,-1.51694937887855
"Spa17",517.316321593189,1.46620286596952,0.25001991115899,5.864344400
30318,4.50910708259121e-09,4.48564132124303e-07,21.4318013132312
"Fam50a",2188.10692031699,0.157457287334628,0.0696419154629613,2.26095
572311435,0.0237619989894004,0.0657272600787811,0.42863676553338
"Emd",1251.55065653473,-0.471271592043031,0.123793033418258,-3.8069314
486442,0.000140701725490338,0.00129720639021244,-3.13279782281116
"Taco1",418.697483372348,-0.122460415341922,0.296477815539635,-0.41305
085548821,0.679569354433389,0.784328100419405,-0.0297490455001822
"Grik3",10.4567392077301,-2.45588845824712,0.816612210491171,-3.007410
89429702,0.0026348335621443 5,0.0122876278810099,-10.8038521301161
"Gria3",889.118481794447,0.73400341546017,0.263344566435818,2.78723584
615542,0.0053159774324701,0.0208427363720696,2.84114354927751
"Npas1",0.589521515515549,-0.572067124206988,3.13121267767108,-0.18269
826520774,0.855034777014864,NA,NA
"Sipa1l2",881.695715056393,-0.691465536088126,0.173562893958096,-3.983
94795292518,6.77797235871168e-05,0.000732983389074656,-4.9912661947544
5
"Ap4e1",509.451249011365,-0.0986737663288005,0.183392944904031,-0.5380
45595922112,0.590545570473064,0.713853072541044,-0.0332607675066395
"Blvra",2151.01281154853,0.898025137306752,0.208230873395165,4.3126416
4945679,1.61315465301445e-05,0.000243553280906977,7.4717261410898
"Pdzd4",419.456158048608,-1.10743202218024,0.199253753217614,-5.557897
92812968,2.7304297155714e-08,1.73264480341006e-06,-14.6910398587612
"Srpk3",131.117561003583,-0.46291989278411,0.382534465626033,-1.210139
04466496,0.22622554311428,0.362384623697193,-0.469886436654573
"Idh3g",9159.02062004321,0.121863418412351,0.392221109383704,0.3107008
15169879,0.756028077491571,0.841767671021623,0.0209911234657382
"Pnck",152.958114420038,-1.16758650172117,0.374320587552751,-3.1192152
9444765,0.00181333410071322,0.0092610301077574,-5.46656993859749
"Ssr4",3848.17165348212,0.201573245876779,0.157894667222771,1.27663112
011493,0.201732509643425,0.333591762617886,0.221294628246447
"Bcap31",9257.40861614053,-0.0333520175159508,0.301435824490825,-0.110
643841263021,0.911898780597225,0.945755448089612,-0.00186008386975454
"Fam98a",2321.16938238215,0.134003347585261,0.123812433708369,1.082309

29294949,0.279115118078773,0.422858319526023,0.115339106467302
"Ltbp2",1965.5614465793,-2.08011471284473,0.395445683109524,-5.2601780
7676665,1.43915957265363e-07,6.10015942339143e-06,-24.9763443360306
"Kmt2a",5683.03367175837,-0.0929793553932524,0.210196298322436,-0.4423
45351156585,0.658239318118751,0.767283447401173,-0.024630137572246
"Ift46",1738.22830764612,0.259366829715739,0.0982533184507296,2.639776
79131318,0.00829606469702952,0.0292666309865536,0.915903975560344
"Tmem25",3141.82875956731,1.79394489700143,0.616206137641802,2.9112739
835192,0.00359958224351785,0.015524143902701,7.47242415595258
"Cd3g",509.105927864499,-1.28234270775578,0.51531266579889,-2.48847504
217223,0.0128292241417573,0.0409401329486063,-4.09791135877277
"Trappc6a",1160.22463774631,0.205712468874694,0.25710586158401,0.80010
8047351835,0.423648199062128,0.566821696652259,0.116785127058407
"Supt6",3831.97800100253,-0.115806347119484,0.0945302752799774,-1.2250
7151043929,0.220548278252917,0.356482660148833,-0.119450735740963
"Spag5",258.898427230234,0.491430635426155,0.384217640861443,1.2790423
5298601,0.200882141897253,0.332552216226239,0.541044681143021
"Foxn1",18.5335419300792,-1.56854692668901,0.676725769283019,-2.317847
19584546,0.0204576270804559,0.0584723037706221,-4.45342169161098
"Unc119",872.297796495439,-1.13720997666035,0.311609811344184,-3.64946
781282269,0.000262784162685407,0.00208622378014661,-7.01931446305403
"Rab34",3075.14295585033,0.298680095486143,0.180075256123684,1.6586404
0354881,0.0971882750711712,0.193010489645314,0.49133196521563
"Sdf2",3038.88335341801,0.164153206629527,0.140976929270983,1.16439766
051362,0.244262895248566,0.383409712931,0.157365654568926
"Ccne1",87.3653858369512,-0.669098086230178,0.351223629901035,-1.90504
860512578,0.0567737532544486,0.128196252571539,-1.37445658232087
"Hsf2bp",340.4234759205,1.91013374676663,0.576172839213413,3.315209632
88435,0.000915744227391086,0.00550236700945189,9.93761773352201
"Bbc3",528.385572045324,-1.09940316139782,0.2004531639535,-5.484588717
45645,4.14432462815694e-08,2.32092203512231e-06,-14.2631575114655
"Mybpc3",10.6057882504706,-1.51820780922322,0.793562507967115,-1.91315
465887173,0.0557282560918998,0.12628888159924,-3.14145022227041
"Psmc3",8209.07974814775,0.346659852675923,0.161740174476653,2.1433132
1081865,0.0320879567391124,0.0827252387279139,0.863956271614974
"Acp2",2842.00450701761,-0.121008828292243,0.132424836801475,-0.913792
5423586,0.360825865839919,0.505973439875808,-0.0824398177341787
"Rapsn",139.881533067322,0.517599798217135,0.483203440287537,1.0711840
0876685,0.284086700412609,0.428095098898296,0.4391368004958
"Slc39a13",2353.36275320726,0.0131963615179296,0.159366264698889,0.082
8052382532978,0.934006403911106,0.958802786298719,0.000555169218798936
"Celf2",3325.17933753452,-1.29384592783162,0.295034257947093,-4.385409
12785675,1.15767980605428e-05,0.000191129897192603,-11.0786298569866
"Nr1h3",2158.89764592634,0.0639731945195192,0.311927103984111,0.205090
207623567,0.837501647008378,0.896266988844728,0.00700614797947395
"Ddb2",1007.7462151127,-0.0059390653953473,0.141115468983792,-0.042086
5652654241,0.9664296900473,0.979592747792798,-0.000122453771545703
"Spi1",1332.3358066706,-1.15688060086731,0.38089093218511,-3.037301503
16384,0.00238706562462548,0.0114300111858909,-5.17300644091761
"Sf3a1",1791.98758637326,-0.0456119682186147,0.100293793436754,-0.4547

83557941476,0.649264960197096,0.760916523046401,-0.0124626320088202
"Stat6",5566.30283937142,-0.24633850410762,0.101199051431329,-2.434197
75801733,0.0149248384337,0.0460231693468739,-0.758380262891407
"Clgn",5.80287675760738,-2.17465118926409,1.07696879331178,-2.01923324
312566,0.043462982659894,0.104557832930612,-4.91039486483327
"Napsa",55611.0447809509,2.39375977368857,0.73689357584293,3.248447065
03291,0.00116036797149968,0.0065846492166184,12.0238893711824
"Vrk3",1933.32349108405,0.481993955031429,0.166699893877595,2.89138729
377566,0.00383545136141772,0.0162573110097658,1.98543555489962
"Smg9",1502.80360304899,0.0358556238118649,0.210807659014534,0.1700869
12304229,0.864941786013697,0.915014646177368,0.00318452465395887
"Paxip1",1082.11092308665,0.118145814092836,0.142365664723832,0.829875
759172841,0.406609031670445,0.550521692903989,0.0705199271135958
"Rmnd5a",5055.1243086348,0.0216510612921606,0.157996577403974,0.137035
001946922,0.891003128524607,0.932561768321665,0.0015116747215013
"Mov10",1554.43595207219,0.163622385203067,0.165625917462999,0.9879032
68458092,0.323200024585593,0.468114253407361,0.12419640676655
"Ppm1j",428.675426065116,-0.0847045281219489,0.308278610349448,-0.2747
66153986267,0.78349590279054,0.860120663960922,-0.0127634979062184
"Rhoc",5361.21106281145,-0.137870034546062,0.182529279407759,-0.755331
062465154,0.450050365243188,0.590795261665096,-0.0725590343348374
"Tead3",1662.56377959775,-0.188924118476675,0.175959928546681,-1.07367
694472866,0.282967498067723,0.426903456926994,-0.160811716084374
"Ppard",1871.2318146472,-0.764259635973084,0.211075427146492,-3.620789
24252356,0.000293705698735584,0.00227429456471264,-4.6513493787613
"Def6",618.821799889642,-0.780977502001077,0.150023198427649,-5.205711
58451683,1.93254606071655e-07,7.4763458515578e-06,-9.218476003417
"Peg3",2063.65104754282,-0.296469702408975,0.226187951967249,-1.310722
78532281,0.189951437741563,0.319419847614057,-0.338345723187991
"Zim1",172.630708244435,-1.09845582030324,0.38846808667625,-2.82766038
698746,0.0046889520643819,0.0189012171493319,-4.35925373366467
"Metrn",650.176678576963,0.683027840189049,0.271445425546379,2.5162621
1351404,0.0118606938112331,0.0386722086841845,2.2216396160905
"Lmf1",1202.26763731416,0.689500705019593,0.142176525383842,4.84961004
046278,1.23704428583061e-06,3.25944452501691e-05,7.1234859853739
"Ciao3",2000.60795238585,0.576713939908619,0.126282465031439,4.5668568
4560434,4.95091873964631e-06,9.8704512868736e-05,5.31925175533744
"Angptl4",5232.32609591511,-0.536946833949793,0.548542486601938,-0.978
860976250033,0.327648676193846,0.472416911072071,-0.402652885369806
"Dbf4",425.843026841348,-0.095657011682132,0.250208597912553,-0.382309
051248366,0.702232141774282,0.802022159109357,-0.0211037582477774
"Daxx",1028.601343496,-0.324441769246885,0.114884628477532,-2.82406596
553808,0.00474186243637362,0.0190632646978171,-1.28478684641226
"Cd320",879.070139560806,-0.044927194265351,0.270984935957182,-0.16579
2220540443,0.868320468505232,0.917493767675446,-0.00386865786622481
"Ipo4",2098.31642621612,0.126044177023538,0.0932781236328126,1.3512726
4694676,0.176608110758685,0.302992075985293,0.150502876349728
"Tm9sf1",3745.27867335941,0.170198671546657,0.0799955194969047,2.12760
255345604,0.0333700503465365,0.0852639551378285,0.41902972156843
"Rec8",1228.27328634103,1.00020416818163,0.469208365525512,2.131684432

05698,0.0330327974777488,0.0847081983246778,2.46904688742211
"Irf9",2617.44556907115,-0.462485855242609,0.253736466091194,-1.822701
57051998,0.0683486023170573,0.147418257519688,-0.885420587570903
"Gmpr2",1259.47271363864,0.0943458720718807,0.172809294898883,0.545953
689164035,0.58509777134774,0.708963498163417,0.0324503794356121
"Mdp1",3318.57932476288,0.195931952256158,0.126744881100064,1.54587665
044612,0.122134360664941,0.229685162446472,0.288224868933676
"Dhrs1",2618.28330913492,0.87641893653465,0.216807010400483,4.04239205
603058,5.29086714017552e-05,0.000602930026295396,6.4975153174136
"Ncan",77.6474036005855,-5.52435417483783,1.69546340762785,-3.25831518
980822,0.00112075849965128,0.00641966781028222,-27.8890883266667
"Tmem161a",1998.58109324743,0.0996153772338037,0.102566027412524,0.971
231700659981,0.331432911164954,0.476253143333648,0.0738952598582732
"Armc6",421.013300898027,-0.157244353293705,0.129102162750499,-1.21798
388147527,0.223230098032444,0.359268712458654,-0.160968633566769
"Borcs8",1033.44959451498,0.0778469761080042,0.171075245822941,0.45504
5238915362,0.649076693192016,0.760787337773244,0.0212834731251815
"Slc25a42",3365.84562235878,1.88425898057903,0.360916223451388,5.22076
553544793,1.78185017491757e-07,7.07586857648529e-06,22.3450887529366
"Snx9",3895.31784385012,0.116225789193889,0.112632978927824,1.03189838
624767,0.302119722034853,0.446722005254968,0.0936569248170426
"Ranbp3",3300.05562928782,0.0889618276032014,0.120931093627296,0.73564
0644062787,0.461949420821426,0.601234217153472,0.0452611720113898
"Ndufa11",4035.24673285188,0.544860239658902,0.288303593287154,1.88988
362387914,0.0587735261888636,0.131493541437804,1.10541111555934
"Bmp8b",10.5113721386531,-1.71676422501179,0.838386085994485,-2.047701
23656738,0.040589282616861,0.0990179302266306,-3.96993886888726
"Nr2f6",4612.81856004246,0.247477438271423,0.13323619423463,1.85743400
802648,0.0632494318504976,0.13889372927368,0.488531890631033
"Use1",4222.91134218797,0.135341429991549,0.161608487449961,0.83746486
4173391,0.402331318814246,0.546171137940554,0.0818575979232085
"Ocel1",673.81537844348,0.369876642294412,0.172454176633434,2.14478216
483337,0.0319702678512882,0.0825014353013835,0.922819877610935
"Dyrk1b",847.432245892072,0.205698104091977,0.194521228061281,1.057458
38714927,0.290302436533962,0.434106672869023,0.171647865358467
"Braf",2145.04910443544,-0.0391592230140593,0.120179246627735,-0.32584
0143892383,0.744545304348176,0.8338394224113,-0.00711579605514452
"Ndufb2",3555.1743369128,0.793585739567127,0.348848654091902,2.2748711
5188371,0.0229136603591862,0.0639065297787745,2.18262563143063
"Hltf",1850.8624180177,0.23554596487745,0.0997857199634267,2.360517767
10918,0.0182494441173271,0.0535159665212278,0.689625641484123
"Prpf6",3164.3502018912,-0.0929567053906646,0.113841166141692,-0.81654
737509423,0.414187139348856,0.557645411063665,-0.0542896889487403
"Rgs19",637.18139133953,-1.21135250145846,0.229676094452451,-5.2741775
5141793,1.33352756648525e-07,5.70200010774768e-06,-14.6267102344135
"Rgs20",7.10226084128429,-1.65440702946615,0.675122306640853,-2.450529
35326317,0.0142646331479457,0.044600996972201,-5.14520431736519
"Abhd3",2327.47074211501,1.68866847657137,0.440785412499404,3.83104437
825209,0.000127600485838993,0.00120307266580691,11.3527095160859
"Snrpd1",1832.34163300535,-0.0228961248798849,0.139648523605895,-0.163

955366578027,0.869766280549246,0.918324738081399,-0.00195084613464001
"Tchp",1282.03726334841,-0.00644426782982483,0.186375064965957,-0.0345768777116292,0.972417139385582,0.983164907824719,-0.000109413441759126
"Tiam1",755.029596402744,-1.51225656843858,0.26879794949671,-5.62599741281543,1.84439109856025e-08,1.28682027797923e-06,-20.511244384288
"Tsc2",3514.8771956691,0.354532301067369,0.145645281937789,2.43421754793818,0.0149240224019712,0.0460231693468739,1.09146680362037
"Rpl3l",66.7994552019207,1.55257396429844,0.519652007189368,2.98771859401798,0.00281068217302647,0.0128765697861067,6.75733895083062
"Slc9a3r2",3940.25947175276,-0.15883362741756,0.175771363692984,-0.903637680680403,0.366187554079897,0.511190957602132,-0.106579280268827
"Puf60",4848.70463714218,0.0265147580276936,0.0833307506406952,0.318186957681681,0.750343125339199,0.837638283399959,0.00469759089548838
"Golga2",3049.74357749965,-0.134470760034345,0.170207341509127,-0.790040892725735,0.429503887052089,0.572143662618134,-0.0750837875379138
"Uck1",3755.99347078378,-0.821281810300726,0.469321293414955,-1.74993511230819,0.0801295110355089,0.166504464078745,-1.47233913391178
"Scin",2378.52027210771,-0.509060075611343,0.53646986865403,-0.948907115489159,0.342667855296332,0.487155511671009,-0.366101692149968
"Ikzf4",427.150397138181,-0.45137746663086,0.319321746661492,-1.41355066277198,0.157493876092849,0.278184948733826,-0.577523522470567
"Mien1",4826.60893604025,1.01622065797589,0.298157159087721,3.40833894810791,0.000653596540014241,0.00424087365630538,5.55159920705625
"Pon1",97.0687422197876,-3.72057413871643,1.25887408477785,-2.95547758406114,0.00312185381858329,0.0139295754550897,-15.9007701236303
"Axl",5980.83612133953,-1.28299453952652,0.281971788226543,-4.55008122477748,5.36252114341592e-06,0.000104873056423594,-11.7557709516173
"Tgfb1",2131.02913239166,-0.99992611660935,0.195692319899577,-5.10968502556707,3.22696332006695e-07,1.13984294954104e-05,-11.3811940322781
"Ccdc97",2274.2383611371,-0.133500667077968,0.0954784281071249,-1.39822858131035,0.162044436562459,0.284080419357564,-0.168010311055667
"Zfp40",607.249749164694,0.524134865870205,0.22957565716501,2.28305941641486,0.0224268686969662,0.0629132292404499,1.44975641821297
"Akap8l",6043.73947050706,0.58467695625897,0.262592905843828,2.22655274856014,0.0259771799099751,0.0705141380878039,1.55052940539437
"Shh",408.418534714087,-2.79036606457558,0.721839956279507,-3.86562982597642,0.000110802888808597,0.00107324129458024,-19.0779335842774
"Pdcd2l",619.241934434882,0.0351402657097204,0.153397405709376,0.229079921835813,0.818806798603948,0.883807294020138,0.0043403932575679
"Gtf2f1",2761.41424524381,-0.00659366494269923,0.116904120419773,-0.0564023314064812,0.95502129972007,0.972274712925121,-0.00018539333861676
"Clpp",2989.39216191064,0.55810590807273,0.227508450762426,2.45312165856876,0.0141622431805633,0.0443448558751603,1.73892326394673
"Alkbh7",796.995598440942,0.572255441053179,0.188130454878308,3.04180118749695,0.00235167163813494,0.011313321885111,2.56471974005947
"Pspn",0.675443762612156,-1.14420503969377,2.05803779104854,-0.555968915959902,0.578232104494061,NA,NA
"Dennd1c",929.331647499248,-1.27993267943954,0.48032719971653,-2.66470997310772,0.00770547456410993,0.0276805829021989,-4.59114935453455
"Med6",876.583717235735,0.020047960796706,0.0928563580686525,0.2159029

40990683,0.829063404587422,0.891010752359036,0.00231351029443426
"Prkd1",505.564051409592,-0.13801499479278,0.319417726685389,-0.432083078872822,0.665681032490022,0.773553177870579,-0.0354368489851449
"Lcp2",644.849264300721,-1.41890067903458,0.416647649609737,-3.40551706067135,0.000660388871118564,0.00427569085520574,-7.73983302571163
"Cse1l",1979.64793449925,-0.183046607045956,0.144684331726499,-1.26514464186747,0.205819482507896,0.33879850316074,-0.198120446496234
"Naa20",2246.76910117456,0.132535935087313,0.132029299903553,1.00383729357143,0.315457045418669,0.460732694499866,0.102707032075596
"Prkra",902.372698518442,-0.116590952919427,0.139412150190806,-0.836304100896911,0.402983846701037,0.546828522720915,-0.0703766324593662
"Fkbp7",591.129712641675,-1.57153940137893,0.292816031178726,-5.36698552689457,8.00634786190074e-08,3.87065895599706e-06,-19.5846588463568
"Plekha3",1885.42529756792,0.100242753578778,0.10974956456919,0.913377232723157,0.361044175458596,0.506106294483983,0.0682661736265669
"Ykt6",4065.01094237714,-0.385511538608771,0.183851638634902,-2.09686213009138,0.0360057817281691,0.0903992701694362,-0.926584340519839
"Baz1b",6341.4123049025,-0.0199945980953565,0.130214670516423,-0.153551040109837,0.877963743310505,0.923773558241121,-0.00158533777639832
"Pex6",4310.21799958483,-0.0799734550947776,0.242719620011381,-0.329489042093209,0.741786075139145,0.831876263309317,-0.0147208395339989
"Mrpl2",2636.17316732323,0.572529755003593,0.241001649021884,2.37562588192749,0.0175192154925337,0.0519133227467206,1.6936459684199
"Mea1",2633.9828632278,0.276368188606874,0.187873988670743,1.4710295478488,0.141283126180412,0.256016579392721,0.376555270034042
"Gnmt",365.961859258923,1.25415989308061,0.441908284320287,2.83805472216859,0.00453894002513264,0.0184311502551563,5.00875479090328
"Grin2d",115.849264200023,-0.966023483461857,0.329299065396494,-2.9335749322541,0.00335082760082681,0.0146984109248417,-4.07663445166652
"Kdelr1",5418.48380824716,0.260045902795885,0.175397093680291,1.48261238165034,0.138177425581733,0.251703644410745,0.358734076457841
"Tmem143",1392.43882072097,-0.365376945832576,0.464426835391531,-0.786726601456068,0.431441928332206,0.573588893946636,-0.20309198090231
"Pdxk-
ps",17.0425525829712,0.852447977138646,0.518493844098014,1.64408504911295,0.100158635771884,0.197601242566042,1.38224797981372
"Ggct",1979.88453969885,1.73938140489232,0.382351460460657,4.54916898394141,5.38581813752672e-06,0.000105197868167418,15.9321553635183
"Jag2",1659.98171257353,-1.70335217693102,0.417891575098753,-4.07606249666195,4.58047128917172e-05,0.000540945058729681,-12.812943548122
"Btbd6",934.258919674433,0.552546022771119,0.106581617916996,5.18425253406672,2.16882901820696e-07,8.22720392937728e-06,6.46924438666791
"Nudt14",951.286353465295,0.728341017102068,0.212040611511262,3.43491283066491,0.000592744462395808,0.0039230589028491,4.03565279477149
"Epdr1",531.862323395182,-0.163518036531733,0.320765389933416,-0.509774563164922,0.610209408258225,0.72996256066487,-0.0514692695970568
"Flii",4909.64155523323,0.143234123043416,0.210503006502609,0.680437421883759,0.496227532771569,0.632001674114596,0.0657248731943361
"Top3a",544.195678870172,0.095704820714622,0.119740500445974,0.799268587972897,0.424134688904396,0.567239053725078,0.0542621883358032

"Atg4d",2064.11539419632,0.624669786049263,0.14057417133977,4.44370242
481763,8.84238836032373e-06,0.000155183517776411,5.47891757309076
"Qtrt1",489.909389079625,-0.16324530065363,0.120939814379571,-1.349806
11216487,0.177078183766817,0.303584843672328,-0.194603769023593
"Plin4",5781.71482549265,-3.72344824775972,1.17823476502261,-3.1601921
4361603,0.00157665125619569,0.00836049665306055,-17.8138605329012
"Hdgfl2",3289.55776652535,0.24269517136587,0.213106417493973,1.1388449
6872429,0.254767821991796,0.395447243697575,0.225157506701275
"Chaf1a",432.534494869049,-0.513007599992083,0.338917119453701,-1.5136
6682455875,0.130110363197585,0.240235971744935,-0.731617387242292
"Adprh",1549.46344455652,-0.583895173027562,0.152382179771014,-3.83178
120896411,0.000127218852460597,0.0012006356172685,-3.92663909057049
"Tmem39a",983.323598588491,-0.141414497925606,0.174507696804372,-0.810
362525637682,0.417731849151109,0.560907347617129,-0.0817657980546232
"Timmdc1",1569.85545119322,0.0480020630363747,0.213119427842972,0.2252
35510071578,0.821796066042093,0.885758966041389,0.00582315007960213
"Pla1a",781.853084848243,0.314340899617139,0.183562014848431,1.7124506
9344381,0.0868136437544098,0.176930325694939,0.544438206796603
"Mcm2",785.399809140925,-0.403427083788146,0.231339308279457,-1.743876
07012643,0.081180702180704,0.16814778800463,-0.719274987106835
"Tpra1",2663.17134772277,0.451077138782455,0.170097517582492,2.6518737
3215895,0.00800464704973299,0.0285171949891639,1.60459326813239
"Nab1",2142.11552112788,-0.496686187161195,0.188456176540608,-2.635552
71192808,0.0084000377775364,0.0295532184385429,-1.7491115135826
"Adgre5",2733.95036916777,-0.637023800144013,0.173688052406112,-3.6676
3166101111,0.000244807501077065,0.00197568570198701,-3.96664512043685
"Il17ra",1735.35162654391,0.391878254240051,0.222609039567416,1.760387
87553986,0.078342063665407,0.163615752176731,0.709391564917903
"Lamb1",7778.89929056249,-0.056251359108184,0.134498986727606,-0.41822
8869055401,0.675779790417376,0.781330310339066,-0.0138804327062666
"Kcnn1",227.789135952636,-0.63433767996272,0.307375207102944,-2.063724
28648831,0.0390438652988014,0.0962405919191881,-1.4849236484603
"Arrdc2",1433.66105737773,0.794418764316227,0.327772015847893,2.423693
07294643,0.0153635840984041,0.0469749353537217,2.42944468160623
"Ppp1r17",0.23819938385719,-0.541377195517438,4.78471990595033,-0.1131
4710289398,0.909913932025132,NA,NA
"Cd36",19276.9125506976,-1.08670884387926,0.735581944382011,-1.4773457
2902309,0.139582985872279,0.253737559065607,-1.49037203638624
"Map2k7",2660.04928025577,-0.123805942313448,0.145752918751076,-0.8494
23417207099,0.395645727397635,0.540065835396183,-0.0762724124532595
"Timm44",3179.32689130347,0.0897049927547805,0.169641268358103,0.52879
2278099561,0.596949555034569,0.719305337063229,0.029555044967069
"Ap2a2",8309.90134801208,0.164786999680744,0.150969926267045,1.0915220
2531554,0.275043245674407,0.418445162621691,0.14356398819513 9
"Pnkp",1469.37592810488,0.0923582240933007,0.124032709098046,0.7446279
67613713,0.456496655817094,0.596486516225821,0.0477213690143878
"Med25",3600.95280743027,0.379251343823711,0.103540167272538,3.6628426
804203,0.000249431739156636,0.00200718945525221,2.35553761188058
"Bcam",14507.0624087469,-0.246598742571379,0.254601907582466,-0.968565
966032698,0.332761791100214,0.477498416487162,-0.182284418266684

"Clptm1",7626.96064507797,0.499229031119385,0.152572762027007,3.272071
79372565,0.0010676246161929,0.00619355690762545,2.5382030726214
"Relb",1619.22890453238,-0.991727943857009,0.267506498183776,-3.707304
12378878,0.00020947732524075,0.00175521653955485,-6.29267550036653
"Tomm40",3238.70213939905,0.317425179281132,0.168312060709308,1.885932
46344572,0.0593040561958383,0.132422181368124,0.641757567449626
"Apoe",69047.616478261,-1.80870711564784,0.230276557751056,-7.85449953
443879,4.01371564354223e-15,7.11449341979877e-12,-46.4274987228933
"Apoc2",36.4347012132377,-1.59607374651927,0.438474747436529,-3.640058
53438643,0.000272576069566551,0.00214215566481605,-9.8093776904449
"Hbp1",3757.49536846981,-0.15873372363212,0.16348990630874,-0.97090840
172336,0.331593893963916,0.476406456773638,-0.117698498608103
"Prkar2b",3110.23040487678,-3.15724800826246,1.03886820534828,-3.03912
276072016,0.00237268149036013,0.0113834999259453,-14.1305487087307
"Cdkn1b",3844.6376283276,-0.256213249349437,0.11326079906562,-2.262152
93784918,0.0236879558430657,0.0655782710532579,-0.698055783363636
"Klf4",1932.77438610767,-0.667403911926687,0.209211244409154,-3.190095
79916004,0.00142225639205093,0.00772455574713343,-3.24581945999019
"Ap1m1",2358.31573102556,-0.204753101125791,0.106870723557625,-1.91589
515172869,0.0553784427396548,0.125627559806607,-0.42474671755397
"Rab8a",4113.2512961644,-0.00156145308065094,0.184891753904729,-0.0084
4522834401542,0.993261762789243,0.995815397514637,-6.54776992404e-06
"Hmgn2",6963.30650865387,0.251053867332094,0.146989483711483,1.7079716
2486041,0.0876416076509371,0.178356754616217,0.432809208902715
"Fam32a",3724.50626571145,-0.0981055568698078,0.165163040248809,-0.593
992195360518,0.552517333047011,0.682395829464155,-0.0374905867222599
"Elf3",3465.4381542118,-0.556811096853278,0.218170429748373,-2.5521840
7689565,0.0107049944146054,0.0357775079012642,-1.8544236408289
"Cyp2c29",1.66377110143723,0.276291081744976,1.20537909579297,0.229215
093168026,0.818701742192496,0.883742820642711,0.03414658976667 5
"Stard3nl",1689.79868502254,0.2747190033889,0.190471755803794,1.442308
34765806,0.149215406308304,0.26694210406453,0.362827838948349
"Stk11",7741.67503085321,0.232658378987823,0.163935579380069,1.4192061
2881983,0.155838929682679,0.27610608368495,0.299424383305899
"Efna2",128.235284786576,-0.931959430706539,0.302489971538163,-3.08095
976196342,0.00206334530595877,0.0102317158635768,-4.27048320236697
"Atp5d",14188.7666056569,0.940447938362452,0.316604323871468,2.9704203
8738626,0.00297392477063336,0.0134474919243528,4.05235505416737
"Ppp5c",3328.80740569287,0.344978415002252,0.110199815997422,3.1304808
6223958,0.00174520370227237,0.0090116477189901,1.62458523597213
"Cdk12",1172.15636651827,-0.0834232388295424,0.113598057175287,-0.7343
72056212341,0.462722012810333,0.601934460326666,-0.0423461957572067
"Lipe",8073.47578429748,-2.69343648165551,0.785182914976209,-3.4303299
6551781,0.000602847669532383,0.00397986797491561,-14.8852945797304
"Pafah1b2",3251.78943853017,-0.378047514615069,0.130887558122456,-2.88
833805166855,0.00387283401208859,0.0163979408398922,-1.55400192855204
"Tbc1d8",1013.919348842,-0.784772529828027,0.229074352815761,-3.425841
95996486,0.000612896886033314,0.0040263690983415,-4.32793439463504
"Cnot11",1944.32419509559,0.68184276011536,0.110055777478661,6.1954290
4276481,5.81264746777541e-10,1.03031818478804e-07,10.9696417706701

"Slc2a3",285.163168110672,-1.02609224453526,0.257474307856585,-3.98522
187738747,6.74171004874717e-05,0.000729974562027417,-7.41095212984815
"Foxj2",1972.79506222622,-0.342991765872747,0.117003225097564,-2.93147
274860792,0.00337358963372863,0.0147849518270265,-1.4454171668738
"Sri",5943.354679922,0.057391711155458,0.119366352723406,0.48080308936
3426,0.630656454360448,0.746285097233721,0.0167955456062372
"Dgcr2",3336.61238532917,0.0175982082639305,0.139935670368584,0.125759
273654656,0.899922480667068,0.938676966139543,0.00111368286448895
"Irf3",5081.88175376442,0.26378465710095,0.163583234127326,1.612540909
26966,0.106844285243455,0.207103079200406,0.415339135906576
"Bcl2l12",589.282054487524,-0.385454737336063,0.125212931655894,-3.078
39399843586,0.00208119555600396,0.01029319142675,-1.7639459641316
"Zfp959",514.001744921417,0.062941717319068,0.125590034616636,0.501168
086394735,0.616252832339186,0.735162804677183,0.019364856542098
"Mpnd",2571.29240013235,-0.0842759167353804,0.123262559987476,-0.68371
0582872391,0.494157939465547,0.630197625985953,-0.0389120293830303
"Sh3gl1",1763.11922362077,-0.190073427135174,0.187605899260945,-1.0131
527200581,0.310987219749877,0.456148591690802,-0.149195601730166
"Ebi3",185.517737678051,-1.05840390894984,0.420664342279277,-2.5160295
3370164,0.0118685236913707,0.0386848002230601,-3.44225604558848
"Yju2",484.197063243132,-0.277601865515296,0.115670651985173,-2.399933
43817999,0.0163980533287166,0.049409557070517,-0.834918538309294
"Ranbp2",4183.78564204297,-0.142926918469252,0.12494697666859,-1.14390
057510837,0.252664872519216,0.392984978013694,-0.133491439544482
"Edar",44.2244185634663,0.311464280029845,0.3169985382019,0.9825416918
21523,0.325833053929115,0.470704073906044,0.234696332040273
"Grk5",1147.92029090315,-1.50751526579612,0.25473187950701,-5.91804711
963675,3.25786644209725e-09,3.52899332711179e-07,-22.3972794233873
"Dvl3",2633.83434920881,0.233302534501834,0.140706314836881,1.65808147
823571,0.0973010218062938,0.193174820854554,0.383586623394589
"Abcf3",2587.482659724,0.397648352957996,0.173284101866428,2.294776893
40776,0.0217459135062495,0.0614584463755402,1.10919793033884
"Eif2b5",2404.08242991871,0.385168695830994,0.157659726932047,2.443037
94841028,0.0145642057544625,0.0452984237485801,1.19189799791864
"Cyth2",3342.30428992212,0.343752165198101,0.132664848880446,2.5911322
2604943,0.00956607284048318,0.0326830227307134,1.17594161639159
"Sult2b1",131.345872138688,0.331852513779473,0.292356000246454,1.13509
73248359,0.25633454231832,0.397171877473188,0.306428015480881
"Car11",286.267556505051,0.0986998610425871,0.249387470240804,0.395769
125639248,0.692275355205809,0.794456118602909,0.0227105938417867
"Dlgap1",60.1745119315778,-0.168883514268381,0.539152096416966,-0.3132
3909410856,0.75409901901695,0.840195581302428,-0.0294060953492312
"Plag1",45.123031140888,-0.550159023251989,0.413404302143579,-1.330801
39804862,0.183254367822943,0.311407849382233,-0.641844021997225
"Hck",642.898905326191,-1.11313745138456,0.407270357709897,-2.73316589
413428,0.00627287286721029,0.0236652486969609,-4.16730566945644
"Mrpl4",3689.83865295611,0.731200918724552,0.154807750672014,4.7232836
5699028,2.32066810026719e-06,5.34850905662054e-05,7.19217093127407
"Keap1",2900.23372976049,-0.0788800671314993,0.166937460280274,-0.4725
12682288722,0.63656089156265,0.751087639341473,-0.0225780732989844

"Ap1m2",1192.51869010006,0.392885004750304,0.185169470256249,2.1217590
7943467,0.0338579745763588,0.0863183562094461,0.962455504113189
"Glg1",10437.9688125505,0.556324121523444,0.125309011353494,4.43961783
366136,9.01187625591431e-06,0.000157732103445078,4.87040211491717
"Atp8b3",1.32315321844066,-1.02572411098206,1.97442055134091,-0.519506
399123252,0.603407651655236,0.724784859087627,-0.330160501750479
"Btbd2",2184.39541692484,0.2605811290944,0.11683600858159,2.2303152277
958,0.0257265229384593,0.0700340186755305,0.692826376243022
"Csnk1g2",5893.50998074921,0.0849228359926632,0.0871696722557733,0.974
224564519213,0.32994505040199,0.474569828322367,0.063296939414348
"Abhd17a",3001.39730878588,0.4734635015767,0.135318686394969,3.4988774
5876243,0.000467221256161423,0.00326987798012758,2.7096328551282
"Mob3a",1146.58608825392,-1.10531605263014,0.217796959217827,-5.074983
85927728,3.87528895646249e-07,1.30501526896555e-05,-12.4311699024223
"Cacnb3",1637.15230396268,-0.284652171662297,0.381319589548154,-0.7464
92389755264,0.455370027080608,0.595533220740337,-0.147534681283369
"Ccdc65",41.9133186698522,0.337519877264846,0.43796034171308,0.7706631
06035214,0.440906645572912,0.582596758971309,0.182348488052369
"Fkbp11",220.112475081492,0.702659144544436,0.248175558446237,2.831298
73442656,0.00463593997035761,0.0187068620740962,2.79578578342265
"Ddx23",3972.25245918919,-0.319063589900689,0.0906324394806451,-3.5204
1268809527,0.000430875825020785,0.00307174290173867,-1.84594564043091
"Pld3",2997.67989983241,0.0654008120025239,0.228708192934312,0.2859574
51560591,0.774910710068706,0.854443956493554,0.0102878331955235
"Grik5",2173.49701676766,0.129391547845726,0.216926489147321,0.5964764
76221641,0.55085696424127,0.680905863368599,0.0497292108365368
"Cd79a",197.674109649276,-2.14183392869398,0.743495493898546,-2.880762
48783056,0.00396714465869885,0.0167065629709093,-8.76428515041306
"Rabac1",8318.09070782284,0.407369941056503,0.16941720473986,2.4045370
2256521,0.0161929659138348,0.0489808329798248,1.2287606348857
"Etv3",1805.58038976868,-1.35190756812481,0.255584646171567,-5.2894709
7713104,1.22670644497492e-07,5.32702054880198e-06,-16.415833000657
"Prkcsh",5856.21339671702,0.270358692644217,0.166401798286311,1.624734
20016194,0.104219206771549,0.203634241269832,0.430256888090337
"Elavl3",41.4882080955303,1.59698604321911,0.495666143430167,3.2218985
7908683,0.00127344203457776,0.00707181383926476,7.90769722043924
"Rab3b",541.842674384536,-0.45730666236979,0.51680958895092,-0.8848648
94434495,0.37622950393638,0.521410396456858,-0.297806256053097
"St8sia6",1536.90906159906,-0.0733552070886362,0.176817035276047,-0.41
4865043824053,0.678240700816336,0.783299184068916,-0.0179163165844801
"Fcgrt",9709.35901922208,0.232114701948004,0.239649300998225,0.9685598
95568914,0.332764821172816,0.477498416487162,0.171577896036877
"Nosip",2046.56753632243,0.095132535590361,0.0983014030888571,0.967763
761259525,0.333162366073217,0.477857864770898,0.070249854589863
"Pih1d1",2120.62582657884,0.299241924531961,0.153216931147869,1.953060
42413266,0.0508124357374984,0.117860180240172,0.639855921467122
"Rps11",17450.9687913331,-0.35521506147375,0.195127033740196,-1.820429
77164664,0.0686935820432621,0.148014969906014,-0.678617723565857
"Supt5",5198.52282179895,-0.018443001489535,0.15506638171471,-0.118936
169694514,0.905325925858505,0.942296743841837,-0.00109616051867965

"Dll3",0.883111623907397,0.539587025924548,1.83307576301766,0.29436155
1666726,0.768481643376847,NA,NA
"Paf1",4033.60135746637,0.0370258505770929,0.128420718538611,0.2883167
99644448,0.773104248648436,0.853228428151197,0.00587703822900006
"Timm50",2496.18052232534,0.4041322106366,0.215112278987785,1.87870358
929881,0.0602849774563124,0.134035265255713,0.812165242817734
"Med29",903.791693982809,0.572198960707163,0.159859234673872,3.5793925
9420642,0.000344393756963912,0.00257773108379361,3.41078970981562
"Bicd1",815.840599227167,0.49344009133878,0.343938336618639,1.43467604
161239,0.15137943448006,0.269995994648024,0.646084873064601
"Ncstn",4118.5585240822,-0.11079928505165,0.0948869227024968,-1.167698
16004091,0.242928539253186,0.381765583985378,-0.106694006807109
"Pex19",4422.5569454233,-0.50262187319011,0.357104050263524,-1.4074941
822116,0.159280901177488,0.280395360342963,-0.639111190362581
"Phyhip",151.718226157414,-0.41766391417605,0.71915894004832,-0.580767
18638579,0.561397373210156,0.689516725650283,-0.155272542876624
"Crhr2",26.3253691108374,-0.92307756945293,0.388070845192273,-2.378631
58464167,0.017377033687098,0.0516175202362946,-2.73590418470884
"Inmt",26679.4172364515,2.14933222962816,0.607708790329277,3.536779891
67078,0.000405037072566451,0.00292064084352835,12.5434001966281
"Cyp4f18",398.8598153583,-1.18008464004603,0.441730477894551,-2.671503
77685222,0.00755122227109435,0.0272907751328633,-4.2497285248041
"Impdh1",976.694270751456,-1.17225839345211,0.289763391972427,-4.04557
106221221,5.21957288306227e-05,0.000596898721841338,-8.70256860980464
"Dusp3",3433.81767556296,-0.332372933681386,0.199655450195681,-1.66473
258483868,0.0959661190106153,0.191206559214079,-0.549878105027691
"Prodh",6202.43136926448,1.50419060110441,0.369522640593756,4.07063177
154029,4.68858110247353e-05,0.00055071056828933,11.2878992896254
"Ess2",806.114084929695,0.0114767687767013,0.115249005100103,0.0995823
674723514,0.920675892951443,0.951226658634901,0.000573871809594777
"Slc25a1",14156.7787772765,-2.58087203999398,0.731955876005718,-3.5259
9401766921,0.000421896498965619,0.00302072628954978,-14.9748853978813
"Dgcr6",3051.61714001165,0.514325350951535,0.316204203278809,1.6265607
6553807,0.103830417823965,0.203016996262703,0.820074067991727
"Ddr1",6117.29637791413,0.356677386368851,0.275383030641865,1.29520466
652395,0.195249635453688,0.325807900162954,0.39999490425655
"Ier3",2170.44547492598,1.22317212683934,0.212716446439005,5.750247088
62873,8.91131162902504e-09,7.24367910523317e-07,17.2931664428122
"Fosb",104.993627081206,0.317084508141386,0.501259715771824,0.63257528
6153105,0.527011030135024,0.659499458672274,0.131994077014859
"Klc4",4061.56572908129,0.956185810760072,0.188973880798847,5.05988344
37754,4.19512839361961e-07,1.37864223250853e-05,10.7014655424399
"Ercc1",813.563984152945,-0.411388319148648,0.260044007874021,-1.58199
499581604,0.113650712880511,0.217333272222541,-0.627911571478629
"Cyp17a1",4.92528699178849,2.31460944945408,0.747339029680241,3.097134
44304443,0.00195401231772451,0.00981688538288909,10.7019471698296
"As3mt",1981.97484790589,0.667348790869558,0.24869050325017,2.68345104
516612,0.00728666423891664,0.0265733039396239,2.42104009534149
"Homer3",1033.11506433465,-0.934337502364081,0.174232655553943,-5.3625
8544297285,8.20391407140692e-08,3.93338483275419e-06,-11.6287741053833

"Crtc1",1196.85984142126,-0.0445033738719254,0.16277780740027,-0.27339
9516695122,0.784546124260388,0.860788944394184,-0.00667131978482655
"Rnf215",1553.74527560872,0.161068572540096,0.0987035579705711,1.63184
160583268,0.102712858033735,0.20133661465183,0.258157015072327
"Sec14l2",210.482068722135,-0.048987672387954,0.355507482462225,-0.137
796459440651,0.890401282623824,0.932247972198407,-0.00343680045833505
"Aven",806.09142575271,0.106910169133471,0.286057716361677,0.373736358
149134,0.708600472423213,0.806696561645811,0.0229651263936681
"Cp",9350.80630249752,-1.22700978075834,0.270230540709661,-4.540603654
70185,5.60933928598276e-06,0.00010818090741651,-11.2046921641595
"Crot",10809.5960859487,1.63948126133408,0.321228327912544,5.103787925
51706,3.32921350818416e-07,1.16750008961465e-05,18.6213276461052
"Rps6ka1",1893.73996551459,-0.35625115049533,0.232792196796486,-1.5303
3974247331,0.125932655829886,0.234869431232412,-0.51611013717939
"Calb2",0.304877989334812,-1.80107325170135,4.75461944527084,-0.378804
922756284,0.704832731513718,NA,NA
"Snrnp200",3651.7343958521,0.254479922936007,0.12406465633023,2.051187
90849381,0.0402486509635333,0.098341879259019,0.59021663588404
"Ciao1",2561.774127885,0.541816939702419,0.173062565049488,3.130757593
63374,0.00174356013731055,0.00900554584298838,2.55191156177782
"Has1",8.18015152183774,-2.2115068409759,0.753623156543165,-2.93449958
613265,0.00334085998838172,0.0146744960697154,-9.3361951461839
"Taf6l",495.5310190039,0.0475951602411868,0.104048859956709,0.45743086
7200174,0.647361382200246,0.759735899250053,0.0130784078867993
"Insig2",1743.10741428968,-0.286167608280985,0.229112152943254,-1.2490
2849807301,0.211654650725836,0.345718554063194,-0.303947276902237
"Kpna6",3354.57363147832,0.066238476391587,0.177747289687679,0.3726553
38418802,0.709404980579133,0.807368060385607,0.0141734197125989
"Man1a",5250.60011810682,0.87729320683829,0.223991776928056,3.91663131
062203,8.97949153780271e-05,0.000908102313299156,6.14469629034883
"Itpkc",613.341923052226,-0.117659977791601,0.117201632106618,-1.00391
074489957,0.315421636982245,0.460715490140071,-0.0911834924076171
"Coq8b",1505.92037692593,0.279601813810185,0.167494040060439,1.6693239
5749301,0.0950531947750631,0.18996138891611,0.464400283079413
"Brd8",2588.45760817863,-0.0599795191186462,0.123923771977543,-0.48400
3336579484,0.628383501167956,0.7445895779868,-0.0176892866013254
"Kif20a",169.092483552086,-0.637141119251668,0.403402783328615,-1.5794
1676553245,0.114240494320629,0.218164658008191,-0.970050660398354
"Farsa",1781.23160170728,0.117117116757007,0.100476078526874,1.1656218
9203754,0.243767352621246,0.382748900097362,0.112476481957989
"Gcdh",9960.1718651663,1.69830344494498,0.394472090364679,4.3052562815
6594,1.66792528691994e-05,0.000249395761076419,14.0899227689875
"Mast2",3145.78110893732,0.338174359293341,0.175662755796366,1.9251340
8867024,0.0542125852779286,0.123581354386139,0.707073752843788
"Dnase2a",1350.74849204863,-0.114895298131583,0.16904338425423,-0.6796
79353548605,0.496707511824215,0.632456283259228,-0.0526386119353215
"Rad23a",2560.75297635118,-0.098322025331156,0.198380034069821,-0.4956
24601498713,0.620159292259872,0.738263820774365,-0.0298362155657121
"Calr",38086.9893516389,0.287909193193153,0.136105964510368,2.11533119
969346,0.0344017291080303,0.0873050779836489,0.702022416950177

"Syce2",117.372660894126,-0.33020205801476,0.302547436039017,-1.091405
90427009,0.275094315559804,0.41849020556956,-0.28763960478348
"Nfat5",9288.32868695214,-0.221388890172441,0.263579937612226,-0.83993
0732885081,0.400947224864674,0.545013175572463,-0.13437094840996
"Nob1",1289.84365325556,0.130403484714463,0.150314022218151,0.86754038
5056077,0.385645994199423,0.530052558501364,0.0827774080160619
"Nqo1",1519.16778320905,0.852129431344424,0.438358090785769,1.94391172
253022,0.0519060973884331,0.119657730773193,1.809172914849
"Ppfia3",97.8392125545018,0.39847734213021,0.273905253707981,1.4547999
23462,0.145724659398169,0.262212939179172,0.533401116816391
"Gys1",2156.33420251026,-0.656091943127026,0.460736370179767,-1.424007
27529072,0.154444366050617,0.274158434928527,-0.849015195106806
"Ruvbl2",1306.91542798869,-0.0999402436092408,0.115949574513681,-0.861
92850666691,0.38872686097384,0.532897162009979,-0.0629050692504621
"Lin7b",43.4386873478154,1.3809231795863,0.374571365976963,3.686675771
3435,0.000227202530286891,0.00187156524526143,8.67355110544928
"Bax",3574.23014810253,0.232577465762035,0.165207819864689,1.407787270
3152,0.159194070871988,0.280318431668204,0.295798781062242
"Il7r",383.02876936882,-0.917476242598322,0.397719262205206,-2.3068438
7150689,0.0210635255083929,0.0598945049384053,-2.58285206667322
"Tfam",2072.38398759378,0.0287800798805956,0.266343591044388,0.1080562
13283537,0.913951101448806,0.947077943029806,0.00156488472428316
"Zfp81",569.200306707907,-0.0317566658014674,0.15603030304224,-0.20352
8834990921,0.838721710240332,0.896904662858871,-0.00345530647481539
"Efnb3",147.320547906985,0.0576344619003191,0.24654824526353,0.2337654
51620696,0.815167072101977,0.882271860774041,0.00721900673266718
"Mmd",5274.15697717484,-2.79664271978355,0.671382225315695,-4.16550008
971197,3.10670801283704e-05,0.000401954829690091,-21.8674272033491
"Hlf",2360.19785899004,2.18223583531758,0.416933162955659,5.2340183732
2704,1.65863954542362e-07,6.66807296013809e-06,26.0082787268267
"Fam162a",2122.87589804331,0.439834089456854,0.240323242361815,1.83017
707789856,0.0672234642239531,0.145458118459509,0.847941661481628
"Rpl8",25139.5718602491,-0.115206127449294,0.222922800270885,-0.516798
314525484,0.605296956748402,0.726237158493653,-0.0368519808553191
"Grm3",0.816001034473348,1.20482612527588,1.9048877507936,0.6324919275
55276,0.527065481669071,NA,NA
"Ssbp2",1132.48555182935,0.145772337590904,0.180654395741234,0.8069127
62862992,0.419716745158618,0.562720009427404,0.0838151718918329
"Fancl",384.385412555286,-0.212989241997279,0.200991467095367,-1.05969
295649859,0.289284308204472,0.43316368687141,-0.178195232671691
"Brinp2",15.6664873223417,-0.729865849324573,0.557255299382065,-1.3097
5129376771,0.190279985004238,0.319861983585881,-0.831949030838803
"Gstm5",4667.74228708085,0.854144302547724,0.289633878494456,2.9490483
1916641,0.00318754135184806,0.0141412244091771,3.63751105558366
"Gstm7",355.566613919087,-0.96438282003388,0.278370460884275,-3.464386
33240901,0.000531442680859235,0.00363326416248014,-5.41753989799356
"Gstm3",2.55093141855818,-0.258904692313072,1.45232152660209,-0.178269
541262544,0.85851129818889,0.910434748835363,-0.0242938163131678
"Stat3",6037.03563409143,-0.457009271380808,0.180576811554294,-2.53083
03289174,0.0113792879212633,0.0375102884004722,-1.50042543125548

"Stat5a",1533.91710470428,-0.0569470585268013,0.245668120818792,-0.231
804836284828,0.816689600549372,0.882743712595579,-0.00710245802302434
"Cavin1",9953.57502385867,-2.03984240344987,0.457700609601216,-4.45671
768981723,8.3224085169388e-06,0.000147518473875703,-17.9945863255883
"Map3k11",2715.54556486037,-0.058616043407306,0.0965240420720047,-0.60
7268843585931,0.543672511414748,0.673999221371535,-0.0231255720922064
"Akt2",6483.07155702469,-1.26125330310082,0.423884409353458,-2.9754651
8642803,0.00292544553131442,0.0132765409462539,-5.45082983711652
"Dnaja3",4135.85254913817,0.364961181579885,0.327162566485877,1.115534
65758632,0.264621375336157,0.406499985717858,0.328527612340969
"Hmox2",3056.20508249979,0.39087624870534,0.188266652362264,2.07618419
832108,0.0378769181671985,0.0940451061651094,0.924023931083413
"Cdip1",3746.27721112701,0.26932237338022,0.204239679494514,1.31865842
155052,0.187283332967415,0.316050755253476,0.310219638419353
"Map3k20",1195.32615307442,-0.493147395437385,0.168204153014294,-2.931
83840351123,0.00336962030703071,0.0147708760671054,-2.07866453622426
"Cwc15",2816.72302797359,0.312814069295589,0.151450542713816,2.0654536
0412929,0.0388801011165642,0.0959236000975286,0.733299753719997
"Col5a3",1412.46680462046,-1.94243837102244,0.519463158570059,-3.73931
883132856,0.000184519575292484,0.0015905228466193,-12.5164755289844
"Dnmt1",1461.75633629129,-0.408271522978483,0.179865351259288,-2.26987
310296318,0.0232152834932792,0.0646009364516576,-1.11847060419453
"Ppan",1944.86470869297,-0.00513573083939601,0.277374428742049,-0.0185
155165985834,0.985227599231984,0.990459795299058,-4.92311362472301e-05
"Angptl2",3155.69917796809,-0.777218290050663,0.193741458340272,-4.011
62609546182,6.03019539200824e-05,0.000669190379928154,-5.6810319848234
1
"Cacna1e",89.1721352478548,-0.121246603983585,0.43318888161124,-0.2798
93157766675,0.779559476820315,0.857497366018682,-0.0186401099680086
"Cacna1b",36.2327888290092,-3.65798722124269,0.718655944097683,-5.0900
3960975446,3.5798872751915e-07,1.23105188874222e-05,-41.3537520915545
"Trmt10a",490.566231337181,0.667015801540157,0.299367971844897,2.22808
003618282,0.0258751783076779,0.0703155716575755,1.7707682044896
"Etv1",878.491252043145,1.25624467046438,0.407022024286351,3.086429223
74288,0.00202576166455728,0.0100915434173577,5.7737727285486
"Kifc2",1299.48201458457,0.637802972521676,0.221730397496119,2.8764796
33483,0.00402138145522417,0.0168585026045927,2.60408584825069
"Psap",82613.0041801403,-0.238184889448169,0.213152949389712,-1.117436
5174403,0.263807728664198,0.405702492150625,-0.214874964768517
"Ikbkg",1789.23387988973,-0.165471906968778,0.193135448895076,-0.85676
6108528702,0.391574159577488,0.536046687035165,-0.103177363188305
"Pax2",1735.85850886568,0.262577079773779,0.416275226597668,0.63077757
9343103,0.52818596518191,0.660672950289767,0.108837238999298
"Wars2",474.203643998636,0.117034681561487,0.132918447841798,0.8804999
11500499,0.378588553095801,0.52346960515371,0.0757537769624797
"Atn1",4978.53398909376,0.061499991578977,0.174928451262142,0.35157226
3604022,0.725159069254359,0.819091155852247,0.012272932164318
"Phb2",7584.54217396145,0.416724289712024,0.203046498886235,2.05235890
29994,0.0401347954239703,0.0981460882231814,0.967341349497782
"Ptpn6",1889.25635528105,-0.430015368956774,0.352113277125605,-1.22124

156313305,0.221994571697824,0.357841448327065,-0.441911861296869
"Eno2",530.738826825469,-1.04995901502651,0.326467022061442,-3.2161258
0773596,0.00129933857651381,0.00718505489644535,-5.18233743333955
"Emg1",3024.60882726189,0.204849569361959,0.1254745389052,1.6325987020
899,0.102553424395707,0.20116566085186,0.328502203480136
"Lpcat3",4095.20994311012,0.322092749208366,0.23772681174877,1.3548860
8474148,0.175453853776934,0.30179638259343,0.385867989265599
"Atp6v1f",6927.22136448606,0.884985988938997,0.189274769060515,4.67566
804245341,2.92998236707596e-06,6.4992942199371e-05,8.53235512594033
"Il12b",36.8466848537654,-1.47933894268079,0.572271603203911,-2.585029
44126283,0.00973706825791977,0.0330985629171757,-5.04197975383523
"Clcn5",3676.38022552378,0.46233350448496,0.352111755084026,1.31303058
704936,0.189172641611503,0.318467291153607,0.52901841134272
"Clcn3",4591.32258459289,0.688180616952822,0.157257797826669,4.3761303
1890057,1.20804804908028e-05,0.000196944154355914,5.87196329115948
"Hif3a",68.2569445564269,1.26928357129483,0.804010350000889,1.57869058
687295,0.114407044975402,0.218355983660476,1.93137817780371
"Gpx6",663.547602219002,3.21650664249855,1.09618957391372,2.9342612984
492,0.0033434261033587,0.0146787043654688,13.5780244975673
"Gpx5",1.06814846203625,-0.304119529372838,2.76875262628507,-0.1098399
06420563,0.912536341757142,NA,NA
"Pde1c",226.111381225608,0.232963218591104,0.466410251544804,0.4994813
42486583,0.617440327447345,0.736134655881272,0.0713664697076842
"Utp20",2065.7078988497,1.65564119249502,0.257817547170365,6.421755270
99004,1.34711874858727e-10,3.05420015813426e-08,28.6494850638512
"Spic",123.277797254326,-0.36949796449121,0.277514045919562,-1.3314568
0344522,0.183038749398921,0.311177045582018,-0.431349429776781
"9330159F19Rik",198.408784135249,-4.10562857310519,1.27652279067731,-3
.21625951615544,0.00129873330461961,0.00718374524070161,-20.2651133513
437
"Cul3",3958.37258213917,0.361549587703699,0.220090575813412,1.64273089
09865,0.1004386244398,0.198008330262216,0.585510097653736
"Sst",0.108439792482169,0.86337900794553,4.78471990596623,0.1804450469
23009,0.856803192346404,NA,NA
"Il11",100.040400499586,-0.373779750042358,0.442739631076825,-0.844242
809556615,0.39853373694384,0.542867877807112,-0.228338052702261
"Large1",733.983906386381,-0.624096170544559,0.247852781202018,-2.5180
115692786,0.0118019444420569,0.0385375074765853,-2.03213407720951
"Ddx56",1349.98159363776,0.145184591898126,0.136446378927676,1.0640413
6950443,0.287309959124599,0.431341609805307,0.122079176178929
"Tmed4",5645.96724864588,0.493724891829214,0.162839686970261,3.0319690
5505832,0.00242964110260459,0.0115741857362776,2.20150844466271
"Col26a1",226.800487959512,0.891436556096476,0.271699009130852,3.28097
094997926,0.00103450398554648,0.00603192545161045,4.55585500783241
"Bid",1814.19059653956,0.974054352354088,0.274963710784401,3.542483295
61512,0.000396378526456959,0.00287487747858919,5.69991790214576
"Ralb",3120.59223204525,-0.164560668521866,0.100376877377322,-1.639428
05177406,0.101124143460772,0.199103155528439,-0.265589764919969
"Ppp1cc",8463.14073431057,0.310016700608236,0.106208331543613,2.918948
97605968,0.00351213705494993,0.015261789234993,1.29661478177401

"Dnajb11",4826.21233084604,0.154553225540017,0.132123685907967,1.16976
168563504,0.242096886368062,0.38076995163382,0.149230395096556
"Tbccd1",950.486449608319,0.338867046429135,0.139297034337783,2.432693
90507921,0.0149869644922197,0.0461636388103159,1.04220689214321
"Clec11a",453.332820030222,-1.61002775613974,0.301019047489895,-5.3485
9095982552,8.86416236890302e-08,4.17472072147031e-06,-19.942549383286
"Zfp324",304.065318740592,-0.457228188156929,0.164013061710203,-2.7877
5472751562,0.00530747103094866,0.0208219457065266,-1.77027221624917
"Gab2",1331.48462164588,-0.971364680588066,0.177316495006139,-5.478140
54498672,4.29818682142857e-08,2.39445847554898e-06,-12.5717450336718
"Coro1c",3931.80919687477,-0.404584519498249,0.14991516381763,-2.69875
647796657,0.00695990848105183,0.0256481375096482,-1.48210812708505
"Tax1bp1",9556.59410100446,0.537452669759215,0.128797394374064,4.17285
359204008,3.00808250150635e-05,0.00039179420584082,4.21619464457079
"Psg17",4.23671711590237,-1.42330373435142,1.01008813948207,-1.4090886
5149256,0.158808956101322,0.279766626857931,-1.81300358353402
"Psg19",0.74570968381,-0.465305985687075,2.11969210044024,-0.219515837
036159,0.826248244527519,NA,NA
"Ctse",1245.00708150926,-3.42656014413704,0.820092802420437,-4.1782590
1413087,2.93749017550209e-05,0.000384217140640084,-26.9475061753552
"Ndrg2",5945.11158940079,-0.339197690061377,0.381684355236246,-0.88868
6385511997,0.374171649960686,0.519296663885932,-0.222269446199138
"Mettl17",1297.10764194445,0.0543199359233985,0.106575565518504,0.5096
84707363504,0.610272368451602,0.72996256066487,0.0170978534590811
"Arhgef40",2186.60940284234,-0.655183440558224,0.186316010265664,-3.51
651712391228,0.000437248453325513,0.00310355673204982,-3.7838196595299
2
"Pnpla6",3568.67447645243,0.443209459840815,0.161104112550601,2.751074
77285291,0.00594000830404568,0.0226576300801261,1.67854884498772
"Mcoln1",3101.01711197459,0.554626500852726,0.0989325896275778,5.60610
515645617,2.0693026846194e-08,1.40378769404277e-06,7.47433334558632
"Arhgef18",3357.59618488937,0.0856407748082876,0.109465506677486,0.782
353979876126,0.43400657146728,0.576095045984685,0.0472293989993265
"Pkn2",3004.15875748523,-0.0358686246530627,0.167722175617009,-0.21385
7377661068,0.830658278941064,0.892056351773125,-0.00409712857857194
"Cd33",625.857241540419,-1.25405702971276,0.372723612357574,-3.3645762
9228403,0.000766612865798484,0.00479237501036833,-6.69757895145665
"Etfb",21948.4000487071,0.668735623939495,0.472109038436853,1.41648553
510789,0.156633396301084,0.277186237164506,0.858031716285465
"Nkg7",316.357667739804,-1.05352843844267,0.499702260339901,-2.1083123
3328033,0.035003981833437,0.0884077251021185,-2.5556449929761
"Stxbp2",6289.22878534063,0.630777344227457,0.150575631589695,4.189106
41494945,2.8005505705187e-05,0.000369722125301025,4.98488123769251
"Pcp2",1.01507566517912,0.0446872662996229,2.68002654520492,0.01667418
79402564,0.986696539338366,NA,NA
"Sgce",729.890658079857,-1.35437149771695,0.253325561858097,-5.3463672
9030769,8.97370210252123e-08,4.20599143257113e-06,-16.7657654713149
"Chn2",821.292056266398,-0.0308822151027859,0.256669044897983,-0.12031
9203724238,0.904230292248503,0.941457907740766,-0.00186298937653454
"Wwox",507.760866107168,0.292208396925904,0.16174790527547,1.806566807

94876,0.0708298487008163,0.151452769193035,0.551537930818152
"Slbp",1507.44490784041,-0.188930588056198,0.102702904962287,-1.839583
68193746,0.0658293818966998,0.143108628411401,-0.367309647975196
"Tyr",59.001457805781,0.201267856019144,0.361052536894555,0.5574475608
18341,0.577221677927037,0.702407057119226,0.0710962981482864
"Ghrhr",9.8702507536689,1.47776594706777,0.570150525581021,2.591887371
43025,0.0095451014835156 2,0.0326337697221791,5.05751747582285
"Aqp1",20864.6023870633,0.487643874803308,0.479015976035641,1.01801171
401228,0.308672389243101,0.453575570537492,0.385528021586345
"Arid3b",429.833577463978,-0.364085269197864,0.181108165530256,-2.0103
1945816402,0.04439738875365,0.106215986002291,-0.816381355592054
"Cnn2",4967.16861191472,-0.97708194359801,0.259498953857703,-3.7652635
1676082,0.000166373523719178,0.00147051267700659,-6.37266931096654
"Polr2e",2700.75579133859,0.222560381692763,0.15688983758401,1.4185774
2426171,0.156022251499951,0.276369208425674,0.286216566921852
"Abca13",1767.41068538001,2.35658271620857,0.987793974256108,2.3857026
6434686,0.0170465270485406,0.0508604719804811,7.01948044601789
"Myo9b",3476.98761817153,-0.43457410344799,0.203149680702674,-2.139181
8187695,0.032420946061366 9,0.0834623192902736,-1.07920395204036
"Hdac9",188.040043266016,-0.81786101950786,0.247403896479636,-3.305772
58945951,0.000947149252595681,0.00563377551162617,-4.2356821461343
"Ly9",384.011367478078,-1.26496733938421,0.450580232700676,-2.80741862
953525,0.00499402949238521,0.0198762169917385,-4.956434734005
"Cd244a",147.8652876549,-1.0061748123354,0.349282047248064,-2.88069432
787309,0.00396800258920951,0.0167065675846269,-4.11722041246334
"Adgre1",1136.03441766138,-1.6389543227655,0.396955934104648,-4.128806
70612024,3.64650787577634e-05,0.000454601090549151,-12.613540379744
"Mtfp1",672.319657012107,-0.0358553152371684,0.45587009776375,-0.07865
24832689291,0.937309041189745,0.961067084829494,-0.00142385275876508
"Rab23",944.746693974225,0.190598285401296,0.153579691188469,1.2410383
4254621,0.214591584862595,0.349315972754289,0.200467159722771
"Rab11a",5329.85980751578,0.276150397436978,0.126776400009835,2.178247
66609207,0.0293876030919444,0.0774114408385209,0.706564121775723
"Eif2b2",1407.97019189822,0.35076812005002,0.170889109134212,2.0526066
3963398,0.0401107430881139,0.0981278881721511,0.814302462551414
"Dlst",14572.4893943864,0.351656917959554,0.291858840214053,1.20488698
475484,0.228246933727585,0.364723710360633,0.354686502072931
"Pgf",120.220521844315,-0.830901347529806,0.330565357352106,-2.5135766
0156978,0.011951382008293,0.0388639170109569,-2.69850920143469
"Ulk2",1870.9711906586,-0.10755785010271,0.132966955179014,-0.80890661
8624943,0.418568861570679,0.561563635193733,-0.0620641256605677
"Ccl24",19.5277757213276,-1.46028033487184,0.789467648701367,-1.849702
56510691,0.0643564306036365,0.140679547129433,-2.86400502074938
"Dgkq",2920.27330202273,0.602332005709806,0.210965909186669,2.85511535
030452,0.00430212005245274,0.0177229530493817,2.4291415516891
"Grap",961.438970984421,-0.468989856139939,0.204925449523253,-2.288587
66556821,0.0221033212299515,0.062269983722236,-1.30204517468246
"Pou1f1",82.3396319125408,0.760019226936549,0.404015306502861,1.881164
43784084,0.0599495536437651,0.133481374551345,1.53052164785833
"Chmp2b",3001.37477832383,-0.309353066967548,0.190882020115456,-1.6206

5063425269,0.105092586407278,0.204689579374993,-0.490714610830624
"Plod3",2620.94575405583,-0.553803336675058,0.135012352003718,-4.10187
163215856,4.09821627751343e-05,0.000497243441063827,-4.2124667742501
"Ap1s1",3865.66394246571,0.089824233930685,0.154700891775065,0.5806316
49242781,0.561488736929946,0.689545089920016,0.0333897506672457
"Mapk13",1267.0349510007,0.42389072412142,0.256836467212394,1.65043044
207145,0.0988549290743604,0.195603146650282,0.691648690268438
"Srpk1",5019.79670557556,0.375634133083525,0.115438637052475,3.2539723
4994011,0.00113803355941038,0.00649001998870535,1.89225306448678
"Pax3",0.25517397124712,-0.547356146356674,4.03474573159759,-0.1356606
29632773,0.892089572912501,NA,NA
"Lbr",1854.32711253011,0.0320645520336343,0.115527318025553,0.27754952
3191926,0.7813581847762,0.858789283357742,0.00488124099902008
"Crabp2",32.071449627496,-1.85951343039073,0.857457309409718,-2.168636
74725781,0.0301102730122832,0.0789205677098398,-4.72188737939452
"Nes",5831.3833707729,-1.0880363537727,0.229763174231546,-4.7354688470
4953,2.18549275243245e-06,5.13406478155758e-05,-10.74656527495
"Bcan",0.838096099454567,1.20539779973231,2.27842458692599,0.529048802
689848,0.596771595764382,NA,NA
"Hapln2",2.26595323308512,-3.47230290657867,1.72842833722197,-2.008936
57654304,0.0445438603074509,0.106396690956104,-7.77997523327437
"Prcc",2298.71804942149,-0.02048761690606,0.117277108519043,-0.1746940
8279906,0.861320017520975,0.912222580207712,-0.00188222320905167
"Rrnad1",2358.18352309429,0.197589507526471,0.22117300509568,0.8933708
13680417,0.371658627167326,0.516875885342975,0.130399689530925
"Hdgf",11715.4970863892,0.203816124717646,0.173355301624148,1.17571324
792558,0.23970948286926,0.377942407541418,0.198315831894379
"Slc25a18",4.63927655473556,-0.457916905275888,0.790187405228413,-0.57
9504181218278,0.562249026162783,0.690261397312803,-0.169742889771453
"Thop1",1039.20057403503,0.1401023845785,0.144942062778479,0.966609567
249119,0.333739250055019,0.478439549798653,0.103287037741792
"Apba3",2003.26500507333,0.585784220744904,0.218241682919511,2.6841078
7943266,0.00727236359755893,0.0265486885274675,2.12567909395102
"Matk",346.068448556123,-0.538589965446866,0.444540804848362,-1.211564
74180269,0.225679037967275,0.361863846560386,-0.547469834136109
"Pias4",2102.90116972558,0.470988351532443,0.174370434370393,2.7010791
8944551,0.00691148802210372,0.025517930970456,1.72776137531458
"Map2k1",2210.22031782057,-0.500293439049387,0.132017987475148,-3.7895
8540890925,0.000150898922962411,0.00136784156202747,-3.2991957319169
"Sgta",4538.08280842994,-0.212843998409313,0.262553091760649,-0.810670
318075507,0.417555023210281,0.560824401911831,-0.123097779795926
"Nmrk2",1.65805535393933,-0.391807520043772,1.30030196398064,-0.301320
409333478,0.763170179308619,0.846433000919195,-0.0653238066542981
"Tmem242",3295.53105508455,0.320945679207307,0.278806004101123,1.15114
335590456,0.249673263938277,0.38924551713642,0.302826686074689
"Dtx2",1346.95845643212,-0.979588228543422,0.305795956495335,-3.203404
77935118,0.00135812943836925,0.00745308409494052,-4.79912749177393
"Zp3",0.141717866057875,-1.11443143702664,4.7787435296438,-0.233205952
59267,0.815601483774631,NA,NA
"Hspb1",5029.8222314438,-1.44079975124259,0.231860614044543,-6.2140771

8244805,5.16271487381881e-10,9.62463073877169e-08,-23.2780725953875
"Rasa4",2146.32337358557,-0.701957910913989,0.266620558870211,-2.63279
738775019,0.00846848492057704,0.0297296577208158,-2.46781035996432
"Syt5",31.1853584595691,-1.06369352355417,0.469037090156576,-2.2678239
0108869,0.0233399420280242,0.0648819774254941,-2.90939898659611
"Hnrnpa2b1",37015.9855208123,-0.167692884333905,0.109131878807367,-1.5
3660769123114,0.124389407980243,0.232758606218692,-0.244454870159427
"Fxyd4",1775.20303521894,1.3139007999802,0.579598273307021,2.266916346
18485,0.0233953364673257,0.0649803804045465,3.59177106136966
"Ccdc130",627.968306349569,-0.0954512586387128,0.132016150612218,-0.72
3027131120417,0.469663215784955,0.607698276793034,-0.04754205511694
"Mri1",1043.17041106029,-0.15818886093596,0.114776145049697,-1.3782381
4232013,0.168129774497168,0.291914010965787,-0.194777312201651
"Prkacb",3028.67891814068,-0.386823557375192,0.150650285149106,-2.5676
9216860316,0.0102378026001533,0.0344706734493842,-1.3026849454919
"Sgsh",780.206662317697,-0.15421118431586,0.111144096752392,-1.3874887
5398586,0.165292778010324,0.288065658352279,-0.191926127041332
"Chd5",83.4469547950323,1.6785927254896,0.509960449729411,3.2916135484
2376,0.000996143938772471,0.005857302327559,8.62807762282598
"Cstb",3811.84191464758,-0.453892711411308,0.368253054479821,-1.232556
54200196,0.217741259110203,0.353145821837527,-0.472445219377689
"Sh2b2",790.136551611732,-3.28149613685326,0.651522954327306,-5.036654
6797133,4.73738307533096e-07,1.51177569890021e-05,-36.4234275705366
"Pex5",3912.37378904913,0.128001527014962,0.297924696196783,0.42964389
5417168,0.667454701179074,0.774783101958063,0.0326624257784835
"Jkamp",2452.69186320972,0.393535725672295,0.118054410208119,3.3335114
2899726,0.000857571483021057,0.00523674562353416,2.06687129057165
"Cd44",2218.16625905357,-1.58313723525393,0.261544301048165,-6.0530366
3245329,1.42140527460156e-09,2.06825074956576e-07,-24.3666864891381
"Slc1a2",203.341345851405,0.959972716454319,0.658789421715647,1.457176
88355456,0.145067559884592,0.261344108161302,1.28820401296064
"Eif2ak4",875.85397808252,-0.12347569880664,0.174592098608626,-0.70722
3865172898,0.479427370015101,0.616817872685875,-0.0596611709779034
"Wdr1",6855.40412430222,-0.554707189673811,0.141301735573111,-3.925692
68469316,8.64805102033107e-05,0.000883078642617973,-3.90075437516705
"Slc2a9",655.566220921479,1.57373364705053,0.392844242386399,4.0059990
1246005,6.17559067746642e-05,0.000679907775433317,11.4781103816819
"Ccn4",621.12357266224,-1.0467055477833,0.4791531159463,-2.18449074617
019,0.028926206533831,0.0765250932379314,-2.69017621240154
"Ndrg1",67385.5550768124,0.976773353397267,0.482210879341997,2.0256145
0859451,0.0428043035838303,0.103227991500003,2.21807180446384
"4930550C14Rik",177.82206166611,1.13666689487731,0.364027670206485,3.1
2247388840681,0.00179338013088113,0.00918515849937256,5.33115676938042
"Man2b1",7223.34402558601,0.138655746903554,0.152893688635077,0.906876
851107271,0.364471923898824,0.509424628830055,0.0935196087315352
"Klf5",1134.46463689543,-1.3486351757399,0.418319521295104,-3.22393554
946844,0.00126441853765337,0.00704188307545427,-6.6836736392718
"Wdr83",1610.62554684137,0.510496536070168,0.154754014811056,3.2987611
7717171,0.000971124934888066,0.00574135657381671,2.6341926332276
"Prdx2",10403.9139296965,0.638086838903734,0.187605355305245,3.4012186

7984808,0.000670861384402846,0.00432126041396984,3.47387757309846
"Polr2a",4086.5894317513,0.0502877209098323,0.161042968415399,0.312262
754497413,0.754840843396066,0.840733849225208,0.00872392078865761
"Shbg",3.88925357819084,0.227095455608036,1.44227315566177,0.157456619
584544,0.874884993921785,0.92193199002794,0.01845918651648
"Senp3",2770.27781210303,-0.168405027080512,0.115128611207683,-1.46275
565486256,0.143534251233639,0.259228494864162,-0.227354429899858
"Corin",130.154852978982,1.06438279154509,0.427862418888105,2.48767534
739585,0.012858105022886,0.0410153434016352,3.39943539546857
"Plekha8",2096.49433627726,1.30980360286499,0.248838623620124,5.263666
80465383,1.41210269813747e-07,6.00125080604588e-06,15.7484794727249
"G6pc2",8.44245868802677,-0.0529880517198168,0.798386668844301,-0.0663
689084344548,0.947084123082857,0.967325627651626,-0.00176026838430208
"Spc25",396.962117692636,1.57332194063155,0.313585770702569,5.01719812
447682,5.24304918802567e-07,1.63828482480969e-05,17.3368680854043
"Dnah2",97.8683614204146,0.509638159113179,0.4889396505867,1.042333462
83461,0.297257103189486,0.441546881315835,0.416614455386272
"Ripk4",930.182246401904,-0.407080438478783,0.345997330447071,-1.17654
213676385,0.239378304689237,0.377617744910325,-0.396444587565377
"Ufd1",2338.8124377296,-0.215054725848516,0.213236691427049,-1.0085258
9865891,0.313202060980179,0.458331866180692,-0.167777473116447
"Zfp287",202.601054691194,-0.21167717748836,0.227617385912848,-0.92996
9284373599,0.352386988073971,0.497382473828456,-0.147834490620342
"Prlr",1198.09780869063,1.55423652084378,0.619941085096104,2.507071330
17783,0.0121736142345787,0.03938950055523,5.02679876429962
"Letm1",5570.07501616027,0.349956836467811,0.318670771646017,1.0981767
6299647,0.272127310886717,0.414753162172851,0.307987116974671
"Ubqln1",5967.66750667123,-0.257049125356887,0.30450080747326,-0.84416
5660806861,0.398576840419052,0.542888664651811,-0.157018719783282
"Fgfr4",1532.04697793762,1.23564178066579,0.540834227397482,2.28469597
165059,0.0223306610059247,0.0627020778072465,3.42193777803004
"Cadm3",136.206682863243,-1.86578661563463,0.540535533374296,-3.451737
21325487,0.00055698991218181,0.00375773821588713,-10.4184368592536
"Fcer1a",0.563895552866122,-2.73001756393547,4.46079526022734,-0.61200
2435591796,0.540536146127009,NA,NA
"Txn2",8048.89259367771,0.638663677262851,0.252711190293443,2.52724731
548789,0.0114960516309166,0.0377993279425989,2.09191985476016
"Casp14",2.34956105621583,-1.14618403005991,1.19050725778015,-0.962769
460303092,0.335663229930528,0.480260704334544,-0.840641384479309
"Slc1a6",7.60445067688432,-0.697238751315212,0.73703393524312,-0.94600
6307138651,0.34414536928977,0.48868592312373,-0.499247544427607
"Slc1a3",824.323258064237,-2.75490868261337,0.815094897191959,-3.37986
250693528,0.000725220995790744,0.00459486064863505,-14.8291688780106
"Crbn",1241.27003600246,-0.552168340340372,0.252201972361841,-2.189389
4610315,0.0285685450018579,0.0758379156495882,-1.42412878767615
"Il5ra",157.144056562968,-0.0246196468321531,0.611887768736738,-0.0402
355596729464,0.967905328054052,0.980359381846977,-0.000488356751637848
"Msh6",1102.52882882919,-0.292023283535697,0.10571680847441,-2.7623164
9204946,0.00573928144830307,0.0220327839494021,-1.1141341684523
"Fbxo11",2823.64121683748,-0.306748844259631,0.138481667601102,-2.2150

8629678858,0.0267541418186375,0.0721510729156009,-0.806440602122759
"Mlxipl",10877.1089674466,0.509549210287608,0.41955182095377,1.2145083
9881769,0.224553644584471,0.360765618938972,0.519499068939991
"Tbl2",1209.47074221012,0.0499441727981427,0.186781120386902,0.2673941
17214242,0.789165728381001,0.864081834914865,0.007296234243331
"Bud23",1744.35794889956,0.409823452829904,0.182636483642647,2.2439298
2527948,0.0248369168057367,0.0680784703095776,1.10123424934482
"Nid1",4519.67593037298,-1.91352627896326,0.301736979715194,-6.3417028
9889364,2.27239223753817e-10,4.76420471478701e-08,-32.2611922102985
"Mcm5",493.012859778121,-1.12436773184137,0.31603748642831,-3.55770369
062349,0.000374110979555054,0.00274639152084504,-6.63092204774022
"Hmox1",1582.97414187358,0.280966082344506,0.265993350730367,1.0562898
7180704,0.290835801791135,0.434471074419518,0.234220605680134
"Mprip",20910.1969514652,-0.0404291891422449,0.174471301365598,-0.2317
24007477466,0.816752383572352,0.882761056939136,-0.00504154894212832
"Cic",4792.88096977151,-0.010235246733568,0.242362871415076,-0.0422310
838034217,0.966314483364937,0.979577820351957,-0.000211189942947329
"Pafah1b3",842.253079485756,-0.0860218456815576,0.250694926725756,-0.3
43133571967573,0.731497975839571,0.824055207587241,-0.0166467541835259
"Il27ra",312.559374983144,-1.27074089818922,0.40032446329704,-3.174277
40419234,0.0015021005565052 4,0.00805277884265582,-6.12717973956666
"Prkaca",3821.50652883864,-0.883565003116618,0.438020665552624,-2.0171
7652294299,0.0436770951566555,0.104943438245776,-1.99185041333019
"Asf1b",110.609636950242,-0.929172969833769,0.408306251973205,-2.27567
656714388,0.022865374865011,0.0638104910192588,-2.55693317672076
"Myl10",0.934884730918883,-2.44566854576847,2.66406176971369,-0.918022
462381307,0.358607106161109,NA,NA
"Ddx39",1620.49965310941,0.0424803182977454,0.0939853682031689,0.45198
8635144945,0.651277169746751,0.762404073950657,0.0115240005561544
"Dnajb1",2410.42046740547,0.0822880074767493,0.153840735359776,0.53489
088754229,0.59272530986666,0.715645432644754,0.0275311349077008
"Msh4",274.376427064911,0.392995265788629,0.203301232011971,1.93306878
615221,0.053227724670079,0.121954662234689,0.826903666985089
"Usp40",1583.33202777064,0.258203806533638,0.174743280164411,1.4776179
4496876,0.13951006841852,0.25366924415809,0.354184370940125
"Evx1",0.368088626729778,3.58761329124359,4.68223370736823,0.766218329
853531,0.44354640360024,NA,NA
"Kbtbd4",853.431619435379,-0.0407878719374645,0.108999986275955,-0.374
20070709185,0.708254997966455,0.806418509783654,-0.00877560969366476
"Celf1",6965.81828472269,-0.0210690984036863,0.187606739239789,-0.1123
04592516567,0.91058190054023,0.945143779437498,-0.00118868093431425
"Ndufs3",8128.43670351836,0.768274551207259,0.300265812767497,2.558648
09958286,0.0105080050593246,0.0352642138806731,2.569791251717
"Por",8296.2615974604,0.49683419314495,0.404753287777672,1.22749884472
305,0.219635155762393,0.355437560372348,0.51392811342525
"Igf1r",10878.4452131153,1.04682031230814,0.282815052088839,3.70143068
615495,0.000214387224222619,0.00178714070025336,6.62337690216411
"Insr",4156.89205379955,0.00239004665101772,0.17389632258394,0.0137440
896708097,0.989034148292024,0.992699877658699,1.75116295268322e-05
"Fcer2a",37.9378289311232,-1.8209063117441,0.591521518276603,-3.078343

31547111,0.00208154958435098,0.01029319142675,-8.33296371413481
"Cyp2a5",1285.66327506081,1.77065256897752,1.05199269710469,1.68314150
264611,0.0923477018205737,0.185628401040984,2.98179388372218
"Atp4a",756.920329047407,2.1381106709649,0.713471977753361,2.996768951
87609,0.00272857429475299,0.0126087597024852,9.35073515044894
"Trim28",6228.64460540205,0.17198094246927,0.204291952507172,0.8418390
46318934,0.399878052629417,0.544052911183336,0.104686308581863
"Ube2m",5494.57149837992,0.506768101003062,0.16747483801174,3.02593575
858564,0.00247864959137259,0.0117502333412553,2.25201773331777
"Adcy9",196.538942990947,-0.178233631533205,0.314832684231658,-0.56612
175437941,0.571311015793835,0.697652795512507,-0.0641701187464459
"Mef2c",1715.03631462382,0.13963087379381,0.320600032301388,0.43552981
8233289,0.663177900110637,0.771518060641837,0.0362195781096099
"Ctr9",3041.02082000495,0.138216100364082,0.11714396745296,1.179882356
46521,0.238047008078727,0.376221169126936,0.135117031782671
"Eif4g2",30795.7859561249,-0.0391104318869808,0.217018110170644,-0.180
217364607165,0.856981926634537,0.90916204940204,-0.00372456185399559
"Mrvi1",1590.2790423795,-1.24651783742837,0.240108646503088,-5.1914741
7463928,2.08635530043856e-07,7.99209344753457e-06,-14.630451936609
"Pcyt1a",2999.93472968006,0.181077918560463,0.172320146022517,1.050822
68521756,0.293340034835648,0.437340877750839,0.14975910681775
"Zfp592",2608.91112922945,0.383862787332406,0.162556374406838,2.361413
31727596,0.0182054274706736,0.053451200846739,1.12432879720628
"Psmd4",5990.87885582103,0.0136710426654051,0.211078559183402,0.064767
5572464308,0.948359072761074,0.968070006842334,0.000443637272080929
"Tmod4",42.3720354380674,-0.336440335214969,0.319128733406258,-1.05424
645290925,0.291770097351728,0.435599797715466,-0.279592466743587
"Insrr",1363.14657056631,1.85993002170482,0.642061991528101,2.89680754
544932,0.00376980939215205,0.0160558635928748,7.68463783561221
"Cabp5",0.844712349308926,-0.509287207772745,2.0927946776744,-0.243352
686819085,0.807732192639427,NA,NA
"Snx6",4319.91536650757,0.457029844681688,0.158699727469119,2.87984013
564623,0.00397876861552498,0.0167206020153349,1.86976104347139
"Mthfd2",546.042292322314,-0.689206564355404,0.185447428678172,-3.7164
5252386575,0.000202039584423563,0.00170683180983129,-4.39239363740302
"Kit",1770.41689714236,-0.225539047870489,0.202717257462003,-1.1125794
1575479,0.265889105058049,0.407635301967436,-0.202394765766301
"Tomm40l",2681.44320160637,-0.349810507941808,0.2545439939774,-1.37426
345236363,0.169359908186496,0.293709818537783,-0.428574895132258
"Nr1i3",13.6710483514615,2.03809911769526,0.563062454253902,3.61966794
677489,0.000294981305497145,0.00227874227202192,12.4000632933906
"Apoa2",116.749438810734,1.53996241499135,0.448073066314537,3.43685557
281484,0.000588509324214011,0.00390696452282083,8.53908318811401
"Pan2",3208.13327671675,0.206743591608454,0.174341178346042,1.18585633
967724,0.235679037711138,0.373547375204309,0.203582573310061
"Cs",12357.5627186041,-0.954990391721098,0.509211967556296,-1.87542801
930616,0.060733863446048,0.134842731663749,-1.91346280281228
"Ampd3",1511.86793555733,0.0157234268916476,0.183653548729026,0.085614
6096847112,0.931772784255269,0.957426856800689,0.000684062633759731
"Bcas2",2659.9894254202,-0.0472628607629501,0.171029495179568,-0.27634

3333138695,0.78228437615324,0.859226045867275,-0.00717087444203933
"Sh2d1a",45.512256150394,-1.39180039774668,0.519405753311082,-2.679601
42696592,0.00737098686648343,0.026783358539451,-5.03828199674832
"Ctcf",3158.63919168242,-0.246906932835181,0.11544968872414,-2.1386539
5016482,0.0324637046991593,0.0835393050315702,-0.612931162147506
"Pard6a",362.564501648658,0.163674820483988,0.209653683944359,0.780691
363989707,0.434984041110033,0.577061585576321,0.0899894448459034
"Agrp",9.29330895520619,-0.0477386401133757,0.655139929938553,-0.07286
78530063848,0.941911275499327,0.963859874550535,-0.00175722846938413
"Pvalb",451.695807467871,2.36751563459729,1.1618939422038,2.037625328
29778,0.0415874170384403,0.100804507386314,5.43243557765087
"Tfap4",391.303096126504,-0.0783855929663292,0.155596120323246,-0.5037
76011917815,0.614418786363628,0.733795019999878,-0.0242623440630072
"Ranbp1",2410.53789111109,-0.142338658416987,0.14956525466114,-0.95168
2653430931,0.341257949326892,0.48578867605868,-0.102765888441268
"Cd247",191.379591078322,-1.32086988241805,0.452687120840204,-2.917842
85792462,0.00352461925527721,0.015305796489843,-5.5206069343112
"Rfx5",1253.48953262574,-0.466400855599387,0.191238283558912,-2.438846
69387188,0.0147342175719901,0.0456374542047123,-1.43979181739576
"Psmb4",7609.09951873991,0.291812409252185,0.226268305715034,1.2896742
5786844,0.197163780995339,0.328218360386058,0.325101248111098
"Mmp8",9.51441072624963,-1.37308847070201,0.688627862133937,-1.9939484
6796796,0.0461576864107839,0.109240636651976,-3.04029544998565
"Slc30a4",1163.98984544284,-0.304428190800334,0.221809499200717,-1.372
47589439285,0.169915338048843,0.294412485406923,-0.372246719296819
"Sqor",3268.30798406351,0.279919075947062,0.407758094411539,0.68648318
6436877,0.492408463315245,0.628830247427342,0.129852761793142
"Bloc1s6",1245.53349520422,-0.469819826608736,0.19891011725964,-2.3619
7048738086,0.0181780892711558,0.0534191988860581,-1.37637714865034
"Metap1",2086.73919983654,-0.083454321367002,0.127912339184588,-0.6524
33704981114,0.514121427035993,0.647901830040152,-0.0362205183332828
"Gpr108",2481.97878951289,-0.345122138371297,0.157742126809819,-2.1878
8820305049,0.0286777468212572,0.0760760146286903,-0.88904230605649
"Tnfsf14",73.0224662625241,-0.967294449427689,0.409569430523043,-2.361
73497663729,0.0181896404907819,0.0534338828499583,-2.83350552026284
"Gata6",366.675178216462,-1.33484538168417,0.393742830531911,-3.390145
23738987,0.000698556038922809,0.00445403716380541,-7.22677863400381
"Rsl1d1",2549.63299269928,0.0893808586779726,0.138738149932617,0.64424
1390860291,0.519418914531765,0.652763776702569,0.0381245084208075
"Cnga2",2.68523012301978,0.144116495994778,1.27215496122844,0.11328533
1101184,0.909804346457308,0.944789366596963,0.00818484689880302
"Apc",2562.4666108545,-0.225175958192107,0.196312804605129,-1.14702634
21942,0.251370734013291,0.391377990241248,-0.211233391396318
"Reep5",7087.65219454965,0.327765639560229,0.288752765934483,1.1351082
2484934,0.256329975834447,0.397171877473188,0.302654252424937
"Ergic3",5586.59356529027,0.289234165240691,0.1424878948799,2.02988587
545967,0.0423681411379761,0.102416813278981,0.659079161400127
"Uqcc1",2463.2694972,-0.0111822519802644,0.310019822265195,-0.03606947
41986495,0.971226962542899,0.982455677427184,-0.000197926400038271
"Spo11",31.7102885844178,-0.191830795105066,0.606359065097023,-0.31636

501562712,0.75172546779547,0.838578454495305,-0.0337712622295153
"Ncoa2",2276.30485556556,0.0814007323873271,0.148282575069617,0.548956
830221693,0.583035076072482,0.707274181127434,0.0281920756059092
"Trh",2.09303786160215,-2.83992320799143,1.54306942737483,-1.840437738
96998,0.0657039971495863,0.142931667569188,-5.52475426805096
"Nr2c2",2541.18113447906,-0.0335254447385188,0.119987916329059,-0.2794
068416571,0.779932621199678,0.857560832806142,-0.00515162579 26827
"Nr2c1",586.431454376818,-0.440536661861285,0.174560786276394,-2.52368
628291898,0.0116131513698573,0.0380944188104775,-1.43953614127073
"Smpd4",1815.64172989826,-0.00262859003404084,0.176900158000542,-0.014
8591729015459,0.988144531628556,0.992215577180636,-2.05421146354075e-0
5
"Pex1",1065.70807372016,0.566202857609125,0.14578402171736,3.883847152
37762,0.000102816556202106,0.00101197234367928,3.90445226133147
"Otx1",1.12617497935245,-1.16999530366026,1.45782972044046,-0.80255964
5516601,0.42222930077683,NA,NA
"Kctd20",2877.51546313495,-0.270535221906668,0.100719341754794,-2.6860
3048027556,0.00723064923449504,0.0264211392005593,-0.983014310154878
"Itgae",218.164636963363,-1.12555127815375,0.314780162151987,-3.575674
11637044,0.000349326586029701,0.00260067574433261,-6.69926316054596
"Ctns",1363.05235553412,0.324065422667813,0.135448310582441,2.39253942
167533,0.0167322305005624,0.0501529639200561,0.969823352488938
"P2rx5",600.711273881353,0.0360833454928548,0.418484807211098,0.086223
7884651642,0.931288520665122,0.957159009853384,0.00157993623069439
"Shpk",808.625422571217,0.564207783152024,0.296307458849053,1.90412953
26941,0.0568933163124392,0.128391884428234,1.1581313154897
"Trpv1",25.1533838601387,-2.95356657419427,1.1654976922768,-2.53416767
254551,0.0112714783961257,0.0372367478427073,-9.71859018892521
"Ephb3",632.540208010509,-0.0649246031670734,0.168234409610401,-0.3859
17502355353,0.699557767854714,0.799811033049795,-0.0145028440851883
"Tuft1",1389.21139725339,0.398279770015816,0.24941946966969,1.59682710
633322,0.110304251937773,0.21222738348951,0.617372282821911
"Rcn1",2353.20114715678,-1.14944266689846,0.208230670459073,-5.5200449
7879374,3.38912900509801e-08,1.9992397084915e-06,-15.0838413953584
"Dnase1",15113.3215882893,2.1578551994282,1.11079206842521,1.942627482
46613,0.0520611838684192,0.119944341612482,4.57622278410184
"Trap1",7658.93232460543,0.666341686764338,0.220789585541342,3.0179941
917576,0.00254453781535688,0.0119897047665124,2.94770033985675
"Naa60",2193.55609615941,-0.0271934742390873,0.146934383268028,-0.1850
72231796712,0.853172381164339,0.90649856072924,-0.00266947014632954
"1700037C18Rik",392.823051958222,1.08045296808921,0.219676782127954,4.
91837579567188,8.72652600092833e-07,2.48393874403066e-05,11.4561292151
993
"Ankrd13d",328.362388641161,-0.494851023309733,0.251081229576959,-1.97
088019739069,0.0487375806920075,0.114175819816504,-1.0738345093326
"Tpr",4654.41743103202,0.0923173110016588,0.119916131584602,0.76984897
5127487,0.441389484264244,0.583076704890531,0.0497993300908195
"Odr4",3385.68254349025,0.453534697844628,0.155506753196472,2.91649519
086556,0.00353988180243723,0.0153459361383661,1.89437115964882
"Prg4",23.5263459198785,-2.04850832114598,0.702952034451567,-2.9141509

2459929,0.00356657433476318,0.0154158870270921,-8.54710736995649
"Dhx34",1134.97628963748,0.112478683846594,0.132825745458909,0.8468138
72250317,0.397098874776257,0.541632309226125,0.0689683188210452
"Kptn",1087.6843449911,0.253092469298363,0.133949974801149,1.889455146
77463,0.0588308674686224,0.131564182111945,0.513337450615676
"Napa",5683.8548209008,-0.078001906456742,0.147686529407341,-0.5281585
71873547,0.597389280671077,0.719583381387821,-0.0256690913521118
"Sra1",3095.66480387156,0.543182984441283,0.138641149339469,3.91790595
381804,8.93215395188997e-05,0.000905193023669182,3.80627999497774
"Calcoco2",0.298287120533201,1.99349616271065,3.17705116310352,0.62746
7440833812,0.530352872256066,NA,NA
"Atp5g1",9800.74340600864,1.64905235943001,0.392885579124982,4.1972840
1104135,2.70135081974592e-05,0.000359528588965228,13.0781673494966
"Snf8",2992.69527096122,0.650073827707314,0.135778171321531,4.78776390
47583,1.68649885467216e-06,4.10528772389486e-05,6.56616796954037
"Tbcb",3246.02348355156,0.622044574651323,0.223588929981914,2.78209021
663836,0.00540100240396274,0.0210701770453112,2.40102776890262
"Inpp5k",2769.12345340968,0.174865484311662,0.104080923978906,1.680091
58284473,0.0929394983242521,0.186632904748982,0.293531242242817
"Crkl",1858.5831383958,-0.168882525477014,0.0835614243200895,-2.021058
4830399,0.0432737114161678,0.104218234337011,-0.381888679555865
"Upk3bl",28.8948914266259,-0.369347143739109,0.802333809603271,-0.4603
40994382051,0.645271480554548,0.758044543758034,-0.102314008422794
"Eps8l1",993.016972633263,-0.225518077551972,0.218548949357521,-1.0318
8817981024,0.302124503874881,0.446722005254968,-0.181726704379991
"Clint1",5185.9717814568,-0.0454589509906999,0.188202094374301,-0.2415
4327900459,0.809134077449628,0.87822551595972,-0.00590292963243225
"Prss16",12.404636444543,-1.34513574047552,0.774736320142768,-1.736249
74782083,0.0825196844467903,0.170225222952128,-2.38174156727636
"Cdkal1",673.769428378952,0.350481633548126,0.107423470854803,3.262616
91936809,0.00110388626973139,0.00634913701687985,1.77323952665269
"Rhox6",38.1236969082448,3.29379109400224,1.05837942699347,3.112108011
54639,0.00185756521585473,0.00943198087987904,15.361086012447
"Cdcp3",0.67236877356297,-0.582213968803036,2.67747673524742,-0.217448
749839179,0.827858643064142,NA,NA
"Htra1",4729.15523698929,-0.0938004024797457,0.275338874398555,-0.3406
7257187944,0.733350089259897,0.825547164060294,-0.0179823704146145
"Zbtb17",1465.59413403867,0.231898137581788,0.177451726671163,1.306823
78769703,0.191272564321491,0.321032319802051,0.263485789021215
"Clcnkb",7705.02247542943,1.52920188746785,0.518701294481949,2.9481358
6111278,0.00319696529291175,0.0141701362312329,6.50922921457811
"Fam131c",149.527665236861,0.576312665485208,0.48731425766501,1.182630
4205558,0.236955650789815,0.375073979469055,0.565150638255616
"Fblim1",2532.12479986659,-1.41685127872183,0.335634622876858,-4.22140
977762494,2.42779127451369e-05,0.000333829860863666,-11.3417231794789
"Hspb7",346.583533122426,0.536621501044426,0.26693040019835,2.01034239
878888,0.0443949623679681,0.106215986002291,1.20325601040566
"Epor",226.724046550314,-0.241470358447344,0.386030552949899,-0.625521
36508916,0.531628930512356,0.663914743299168,-0.0989066298139671
"Ccdc159",161.558243683315,-0.329745402997277,0.200674440815971,-1.643

18585693567,0.100344485183889,0.197867796532713,-0.534239047735699
"Mob1b",2255.38251135487,-0.181970441661651,0.246621642612928,-0.73785
2686948701,0.46060397260912,0.600003758547076,-0.0929540244649037
"Atp6v1b1",4457.4380342443,0.966483699440483,0.507091311112928,1.90593
622540941,0.0566584803839813,0.128010086967192,1.98674855739262
"Vax1",0.200706703775918,0,4.7847199059619,0,1,NA,NA
"Atp6v1b2",10665.4378854212,0.525085452039922,0.26999127905778,1.94482
375087215,0.0517961945309053,0.119475003071524,1.11562181263995
"Eps15l1",2445.96442304643,0.0120055413939508,0.174106866031217,0.0689
550140532548,0.945025427805743,0.966083248118092,0.000414254450976134
"Tep1",2131.55766025849,-0.0725679660409027,0.206535297913668,-0.35135
8662533493,0.725319290361154,0.819137146850748,-0.0144775815492332
"Ttc5",2217.94627336704,-0.0841799517267792,0.105593950542803,-0.79720
4302841726,0.425332387471471,0.568334079695637,-0.0475655336126868
"Osgep",1624.40324507211,-0.425024254582659,0.223369644524722,-1.90278
430843641,0.0570686955556849,0.128728068711794,-0.871322288153639
"Aamp",9337.85446218574,0.428411756237388,0.174666752374141,2.45273785
888981,0.014177361348944,0.0443846303478797,1.33444351391259
"Tmbim1",3606.22054499155,-1.10114978062736,0.213313865902238,-5.16211
065778547,2.44180866531679e-07,9.08595140388295e-06,-12.7830068046737
"Arpc2",9832.18832442229,-0.149192727773322,0.141263606421818,-1.05612
996547623,0.290908841785766,0.434513604806103,-0.124356319480648
"Kmt2b",3673.45219705164,-0.0954116530039527,0.140675363415178,-0.6782
39961053893,0.497619559183334,0.633286741397862,-0.0435870935378306
"Zbtb32",61.9353903091003,-0.156213963707883,0.474213314984665,-0.3294
17076180019,0.741840462487895,0.831876263309317,-0.0287545497438917
"Etv2",0.20907454716973,-0.379137285725014,4.77099592825755,-0.0794671
157607719,0.936661086691445,NA,NA
"Upk1a",2436.79002314025,-2.61859010891638,0.715207538405399,-3.661301
04662305,0.000250937687382032,0.00201681081144883,-16.2515929727279
"Tmem147",3574.30197319378,0.513495299683354,0.265764595004769,1.93214
336798376,0.0533418128126105,0.122129951411494,1.07971096955399
"Rps9",21129.8839215684,-0.370468077255249,0.239648156455373,-1.545883
27627814,0.122132760171201,0.229685162446472,-0.544975496755145
"Tfpt",904.240614542147,0.120546960726653,0.125053988631871,0.96395934
3044343,0.335066309870042,0.479741732254816,0.0885426320115573
"Susd2",6520.00091919272,1.79088878526537,0.547979050173287,3.26817016
946001,0.00108245261793084,0.00625306630637711,9.08819345824273
"Ggt5",1063.09426857073,-0.651296367047759,0.197124456794844,-3.303985
6019773,0.000953207275897906,0.00566017275552232,-3.37000335542289
"Ggt1",24904.8497490876,2.24942671041528,0.775596202577342,2.900254930
25925,0.00372859276112864,0.0159255425315413,9.31224645092581
"Crip2",4486.65268319288,-0.328361537338158,0.128479818236327,-2.55574
409931191,0.0105961012345063,0.0354860956929263,-1.09627253392728
"Crip1",3169.58137524555,-1.08279400292068,0.262376338179871,-4.126873
67478381,3.67728259972097e-05,0.000457268215110711,-8.32694622432706
"Cbfa2t3",1557.72006006171,-0.889420224485589,0.231063463015447,-3.849
24649219046,0.000118481733921842,0.0011368882126024,-6.029789179207
"Fbln1",3158.19804914361,-2.07347855115519,0.377564536649004,-5.491719
56020531,3.98039084484788e-08,2.26182406888254e-06,-26.9538505129495

"Pgrmc1",10518.9104340437,-0.0625665993790892,0.230346209471645,-0.271
619834867701,0.785914346638618,0.861562910759012,-0.00932287384979068
"Gcat",1522.87672447168,0.483927092488458,0.448169815580684,1.07978510
748531,0.280237884422584,0.424080541025407,0.415128090697574
"Tek",2172.45613261828,-0.563207263533664,0.194506284739188,-2.8955736
0210167,0.00378466265393985,0.0161120856826461,-2.32502412926047
"Mpl",4.87869479956514,-0.627953532835766,0.708365590964286,-0.8864822
64025477,0.375357707302487,0.520471667594186,-0.41006610641252
"Elovl1",3233.14979596318,0.238400931282859,0.11531218478875,2.0674392
0184676,0.0386927875421019,0.0956187543087328,0.559619080808088
"Med8",1798.20706516685,-0.154317285836289,0.216014873746266,-0.714382
686525339,0.474990574902907,0.612564735065605,-0.075631002441045
"Hyi",4443.73789284844,1.40524453639269,0.331500717978266,4.2390391941
3176,2.24478468085589e-05,0.000313979997900489,11.3349571663014
"Cdc20",213.000447436731,-0.802701793277965,0.379286845278313,-2.11634
493331547,0.0343154817965797,0.0871542613090676,-1.95865306944721
"Adamts4",164.104514532019,-2.41629045377276,0.361958066427646,-6.6756
088008216,2.46207924392882e-11,8.2768312238145e-09,-44.9666957247347
"Nectin4",685.346805612729,-1.52784654030849,0.333668053078805,-4.5789
4163439028,4.67334568148353e-06,9.41331550594689e-05,-14.1643600647208
"Pfdn2",4287.00040534468,0.566496394873329,0.148108647245005,3.8248704
9480789,0.000130840846341418,0.00122551055809493,3.79801714933507
"Rnf114",3942.49252140136,0.0334214853884321,0.120771963713428,0.27673
2151741243,0.781985782091607,0.859027182131512,0.0050785522394039
"C330007P06Rik",2108.34489262559,-0.511241317793621,0.260790627827563,
-1.96035157418179,0.0499547117978352,0.116300533807068,-1.099975382668
18
"Neurl1a",332.893122796398,-0.758304563074309,0.246206362037764,-3.079
95519205144,0.00207031739327281,0.0102532508341461,-3.47315658335366
"Srm",896.762142244363,-0.0451351545556325,0.180317877653652,-0.250308
816535244,0.802348538840378,0.874026024376205,-0.0060772286441973
"Epha2",828.320174076069,-0.979591775272274,0.169927122449284,-5.76477
587069504,8.17664292452396e-09,6.81317024540035e-07,-13.9094568688274
"Rbm14",1592.92296267098,-0.0916032875352228,0.11466970999688,-0.79884
4677794296,0.424380480617661,0.567489926005292,-0.0518962194634362
"Actn3",52.8991795808222,0.168465802762964,0.378950873511893,0.4445584
23105671,0.656638899442185,0.766196604507704,0.0448652193101779
"Zdhhc24",681.032787541873,0.155589857609342,0.0934836100859247,1.6643
5439823444,0.0960416274081811,0.191318372205868,0.257317053276643
"Bbs1",790.948627159953,0.449481944549184,0.198692265554636,2.26220151
697644,0.0236849556408182,0.0655782710532579,1.22461844462292
"Slc34a3",1346.98694478188,1.46314171028932,1.11493840847974,1.3123072
0832765,0.189416500992175,0.318716006395577,1.67303646262606
"Ndor1",1325.14861959588,-0.152479565233182,0.178942804013156,-0.85211
3422912337,0.394151142621852,0.538591280316832,-0.0943540939624438
"Nsmf",3597.70013643847,0.133821653545497,0.190728276328843,0.70163510
1628926,0.482906755227858,0.620516403943111,0.0638601263909808
"Pdk1",2344.46935165414,-0.546246938490542,0.431968433455142,-1.264552
90753848,0.206031643463303,0.339061865652218,-0.590805772689947
"Ptbp1",8905.76147925995,-0.0656131004549713,0.0849212457015413,-0.772

634691271144,0.439738607503107,0.581565717228216,-0.0355643541444335
"Mvd",2051.03393097076,0.303172633766475,0.398851284644557,0.760114472
331842,0.4471861626093,0.588144067356231,0.160918985749472
"Cyba",14937.4078460609,1.25351804996933,0.432606688442859,2.897592856
18374,0.00376038403322528,0.0160262224873938,5.18145320257172
"Itih3",127.324983789607,-0.490005807703935,0.423962337315843,-1.15577
673905239,0.247772489874602,0.387073792771012,-0.465084075639545
"Stimate",1342.0995276179,-0.943619005058364,0.318203431104077,-2.9654
5829749311,0.00302232318095941,0.0136063859114169,-4.05493472808775
"Sfmbt1",1057.99867900348,-0.195369877334085,0.158429777981184,-1.2331
6386492247,0.217514633999276,0.352925050654729,-0.203477642391103
"Itih1",129.98847683274,1.74246911027055,0.537762520939865,3.240220436
38366,0.00119437326942039,0.00673449682104072,8.71323801435048
"Ihh",695.654412419079,1.14870980778798,0.51397695862318,2.23494417116
499,0.0254210141870597,0.0693714394148761,3.06507944266277
"Prkag3",400.853203314729,2.67616724213646,0.482182387471483,5.5501140
4744585,2.85483298976314e-08,1.77036571606925e-06,35.4440271395651
"Cryba2",0.79578460614054,0.101226521582596,1.97068543305052,0.0513661
489981699,0.959033758334948,NA,NA
"Atp7b",102.878437126036,1.18038332190653,0.398942396714083,2.95878134
695343,0.00308858161903737,0.013815362332643,5.05437080476805
"Defb2",20.3661398181944,1.07608109583528,0.676180176078053,1.59141177
73708,0.111516935546148,0.214102370413237,1.65856488192323
"Slc4a1",1436.8185476133,1.30348623556983,0.579226382183781,2.25039168
736663,0.0244240922225138,0.0671665624176732,3.5201685170096
"Rundc3a",392.829393952541,0.961552567294491,0.460778649516058,2.08679
930874484,0.0369062796592462,0.0921499091811989,2.29266688330291
"Slc4a3",2260.31415012987,0.458978327417615,0.266909530627403,1.719602
61718168,0.085504700119983,0.174998482457496,0.799989116502566
"Cdt1",275.816628864439,-0.356396355888469,0.281243849970214,-1.267214
75305581,0.205078511987531,0.337778410773179,-0.386820197782999
"Runx1t1",256.031281009059,-0.274163565218557,0.243627927167265,-1.125
33718283589,0.260446179036163,0.401890694055215,-0.249920688337759
"Snai3",32.9365752426737,-0.781296375604568,0.407506585594375,-1.91726
073448604,0.0552048156400013,0.125321166066916,-1.622655307159
"Aprt",3211.45302604481,0.863569216149353,0.249702877228548,3.45838712
686897,0.000543419923445399,0.00369178517040818,4.83740831297134
"Gtf2h1",2371.92578494498,0.245942221657086,0.10771088703722,2.2833552
709681,0.0224094497462541,0.0628780329763221,0.68041352256857
"Hfe",1436.60598469451,-1.00180444556985,0.169882271402992,-5.89705115
95844,3.7005515377099e-09,3.8584681220464e-07,-14.7944731429545
"Abhd1",282.035330750956,0.605347452383397,0.189978878908896,3.1863934
341548,0.00144058547625593,0.00779580021267317,2.93845951834226
"Slc5a6",749.582489114925,-0.0559093539732508,0.409805129911812,-0.136
429121776202,0.891482053056915,0.932817273290959,-0.00388826890551844
"Tcf23",47.247554479964,-0.776871610818075,0.308456731304631,-2.518575
64440971,0.0117830570459521,0.0385027729649695,-2.53029027004712
"Nphs1",2522.54597200942,-0.239678657530772,0.496872127807483,-0.48237
49293171,0.629539627549683,0.745550640027836,-0.0703773761731278
"Aplp1",893.277179646623,-0.641330547095222,0.359513263354822,-1.78388

563779429,0.0744422501316901,0.157359711955742,-1.18596187418882
"Qrich1",2944.31373518361,-0.0504211691370107,0.120266345276312,-0.419
245874821988,0.67503644845469,0.780702335368026,-0.0124823319177698
"P4htm",302.008183793169,-0.00606709012712123,0.243942041898355,-0.024
8710311675149,0.980157833863415,0.987144496108929,-7.85011749409489e-0
5
"Usp19",7478.61905811862,-0.156933897617121,0.112880107207778,-1.39027
062871454,0.164446713262607,0.287053000465024,-0.195867378699043
"Pola1",301.052486499885,-0.379546324104199,0.209224409600151,-1.81406
330566089,0.0696679800475979,0.149536137711147,-0.721220450049183
"Cdc42",14686.3877370842,-0.592868557187491,0.212895704207173,-2.78478
403026188,0.0053563386298756,0.0209546329464916,-2.29167146136308
"Pknox1",1476.41554102967,0.0530041911772829,0.131554135712252,0.40290
7828707253,0.687016031898694,0.789937666544033,0.0124984539911731
"D130043K22Rik",58.2894077379557,1.62550373633659,0.652269938956096,2.
49207213034848,0.0127000243290632,0.0405903875923665,5.20847808278326
"Gmnn",532.121241019625,0.605341676056455,0.192863883602029,3.13869898
682309,0.00169699672367343,0.00885417236237209,2.8613692760976
"Acot13",3772.27958261757,1.36370551694681,0.303529822044327,4.4928221
8057526,7.02854701773984e-06,0.000128557795264438,12.217617716567
"Zfp184",89.6284740068262,-0.416213345800723,0.288519306673953,-1.4425
840357057,0.149137683161929,0.26682754141047,-0.54988140289241
"Cyp27b1",211.344440161635,0.808953184642276,0.524945795551437,1.54102
231410864,0.123311355274003,0.23116285021945,1.18481939825684
"Cdk4",4544.51473715128,0.0410193740460181,0.203418175689987,0.2016504
86279714,0.840189973530155,0.897743539231201,0.00442479443820844
"B4galnt1",2491.37665150401,-0.557951153368868,0.340578655863911,-1.63
824462796581,0.10137067341884,0.199402496612887,-0.899657122937214
"Mettl1",1859.59249668215,0.843388717604322,0.200826512299291,4.199588
53016092,2.67400465577038e-05,0.000356695249868367,6.69534994846814
"Tspan31",4914.17377676164,0.295795316032491,0.0643425035050563,4.5971
9936152696,4.28207851371746e-06,8.81646957343854e-05,2.76163497752579
"Kif5b",11061.6158096673,1.06966741494478,0.292625950441569,3.65540859
698417,0.000256772683787204,0.0020485080967606,6.62192963853239
"Saal1",544.968319747668,-0.0637910073198723,0.107528840182717,-0.5932
4556287854,0.553016824265612,0.682710018964854,-0.0243480756788441
"Tph2",1.17641198223534,-4.13141531381602,2.18224417317878,-1.89319571
319921,0.0583318472874831,NA,NA
"Krt23",159.830737514504,-0.941536408961742,0.46108410796144,-2.042005
77010666,0.041150958971107,0.1000374783652,-2.1676148934415
"Cnp",1144.73751784579,-1.02728467465138,0.236856447849603,-4.33716153
382353,1.4433455150276e-05,0.000225021719382461,-8.6284861695147
"Ttc25",375.208369151217,-1.94477133206516,0.65642424599702,-2.9626744
3490195,0.00304978951707226,0.0136994265323637,-8.3438494629165
"Sulf2",1737.25007274829,-1.5438525986321,0.314005519977636,-4.9166415
8879134,8.80415270663555e-07,2.50238147921254e-05,-16.3581721083224
"Sod2",12521.0690607329,0.668057410009941,0.408786141273665,1.63424671
865944,0.102207056419322,0.200647723123634,1.0730368381485
"Tmco6",896.53432127605,-0.0631010970519403,0.106295429424379,-0.59363
8855345438,0.552753687125629,0.682514811701318,-0.0241027922991031

"Stambp",1003.09238671534,0.0513918176035395,0.139644139377575,0.36801
9867017722,0.712858420467321,0.80951155982946,0.0108603360653165
"Ezh1",3178.37613366503,-0.155011831895186,0.131852645748743,-1.175644
45533065,0.239736983099408,0.377942407541418,-0.150828598268918
"Hap1",839.462317023448,-0.108536983639736,0.314766599293963,-0.344817
346831556,0.73023169111352,0.823104261378854,-0.0211291552877464
"P3h4",472.871845469495,-0.971079424475127,0.200635394277099,-4.840020
51569207,1.29825725626383e-06,3.37512266435095e-05,-9.99871326983566
"Ctnnb1",21585.7516909112,-0.22870312085297,0.150705107996064,-1.51755
387653444,0.129126901788197,0.238963205302416,-0.327376097003228
"Eif1b",2245.93514253354,0.268207686267764,0.163027387165,1.6451695076
0126,0.0999348598190363,0.197226582445181,0.43540890676888
"Chrd",569.082106285244,0.0889561451061094,0.234895804543824,0.3787046
99638486,0.704907163064536,0.804041176168967,0.0194017619679634
"Psmd2",7917.73204997305,0.144697326295424,0.178764973578701,0.8094277
27360258,0.418269158682396,0.561310141756832,0.0835600563628261
"Syngr3",57.2381804547006,0.238098201477604,0.403210468607429,0.590506
001245271,0.554851463561592,0.684411579459981,0.0902858422656291
"Vars",4295.78847460473,-0.360496763175828,0.21085485383484,-1.7096915
561555,0.0873229249926744,0.177805056299309,-0.622602250089223
"Vwa7",148.951217623299,-1.72929347975264,0.374566503748034,-4.6167862
3808793,3.89728343878499e-06,8.21505216101943e-05,-16.2673900339042
"Hspa1l",30.2365786685121,-0.465083052503695,0.383906201268468,-1.2114
4970038778,0.22572310121136,0.361863846560386,-0.472750994167626
"Slc44a4",3073.14584922632,1.59920408274393,0.471145499247269,3.394289
20641058,0.000688069875348435,0.00440590687341339,8.67537696819128
"Msh5",121.618722325856,-0.0312790862148618,0.469847656099214,-0.06657
2825912442,0.946921779519692,0.967309892988783,-0.00103960317073041
"Abhd16a",3304.62071047955,-0.184909433949587,0.147471505504099,-1.253
86550654321,0.209890894340642,0.343643686107467,-0.19751100348124
"Neu1",13869.9518748998,1.60862167567761,0.368859033993517,4.361074360
20094,1.29425368478589e-05,0.000207186850130996,13.6441513232635
"Ddah2",2312.033282108,-0.237833428925038,0.198612976514369,-1.1974717
5184111,0.231122731468178,0.36805219031089,-0.237721773115787
"Clic1",7711.34464787892,-0.208893625752741,0.248478173653887,-0.84069
2052267397,0.400520474174268,0.544660915431013,-0.126922064831438
"Lsm2",792.174190472324,0.115801765521964,0.118068056312721,0.98080521
6402018,0.326688805381339,0.471602570453051,0.0870387678086785
"Pole",198.975559543456,-0.361772665027337,0.376022162612672,-0.962104
63371008,0.335997046735881,0.480533833473155,-0.265127861506708
"Atp1a2",7149.20931869272,-2.45200916940941,0.674758252214923,-3.63390
764227126,0.000279160825958378,0.00218246903950941,-15.0241919981706
"Atp1a4",5.90876566292041,-1.3225215251789,0.814878817258898,-1.622967
11752505,0.104596434333221,0.203982924247763,-2.10243758763712
"Casq1",47.3045564861276,-1.08745461629089,0.379513316008737,-2.865392
51831114,0.00416492632710835,0.0172855967488205,-4.41276216758236
"Stx1a",568.531696062731,-0.542318572472524,0.327262019601205,-1.65713
874507461,0.0974914273704518,0.193474590419244,-0.890817417251842
"Zfp775",361.692154471427,0.194613780303127,0.13711215837282,1.4193765
3533215,0.155789269595407,0.276068270341839,0.250488775961401

"Scube2",125.512990448099,-2.14624079697139,0.642254152496221,-3.34173
128913171,0.000832576190765465,0.00511615838876301,-11.3221744708465
"Mrpl49",1808.44823088943,0.564255744933013,0.172494238859263,3.271157
04654569,0.00107108412373372,0.0062099310866964,2.86732508571339
"Dennd2c",194.969870911691,-0.617895666595196,0.222622522844711,-2.775
53078951588,0.00551116807587441,0.0213674199927221,-2.3763574968322
"Mark3",5103.78193321273,0.764104708855682,0.220090715035665,3.4717716
6802272,0.000517035894476224,0.00355220784725067,4.30969263558277
"Gatad1",3572.7320067908,0.479051495432325,0.227655415036305,2.1042833
3257932,0.0353537390862152,0.0890948952698751,1.15837201983861
"Pcdha11",6.59913695018835,-4.16025760933107,1.96679228272698,-2.11525
011861589,0.0344086353868635,0.0873068293858363,-10.1440667511779
"M6pr",8944.42102341302,0.0990486461942884,0.139941987265613,0.7077836
18981283,0.479079640498125,0.616492531047548,0.0479107286879592
"Lrrc8a",1957.73584243127,-0.307589691693454,0.127280735824292,-2.4166
2408455961,0.0156651859123838,0.0476883387922014,-0.936016465034104
"Mc5r",60.5383581409514,-2.20352980272062,1.09171405312007,-2.01841296
850858,0.0435482698740296,0.104697153111922,-4.97266902228036
"Ppp2r1a",6719.72790750497,0.20195679945122,0.108314159146726,1.864546
62107141,0.0622449809169863,0.137241520614378,0.40108882532919
"Fance",789.938999174693,-0.000852143812690462,0.14051999208491,-0.006
06421762517284,0.995161484039402,0.996930467424169,-2.61970588870379e-
06
"Tinf2",979.062897944583,-0.176531772428672,0.110794168513388,-1.59333
090177341,0.111085977837214,0.213415547922949,-0.272655808903933
"Tssk4",68.2810618174108,0.222037755232383,0.279423486473602,0.7946281
03866849,0.42682986536954,0.569827368365305,0.12487887985923
"Hapln4",15.1361989603254,-2.75392574486657,0.888482819491763,-3.09958
243924389,0.00193793638372728,0.0097523021685984,-12.7513699181023
"Dus3l",2426.02236417277,0.20683179570835,0.148232431351296,1.39532080
6809,0.162919120407235,0.285152334802537,0.259518417558191
"Gtpbp3",1038.20030465148,-0.374056585153388,0.124368183421977,-3.0076
5497140235,0.00263271853916567,0.012286439941755,-1.6455718243688
"Tgfbr1",3320.04443058406,-0.245593949790084,0.209235112210488,-1.1737
7024914929,0.240487063573886,0.378817175930462,-0.238398434041837
"Homer1",782.413793251117,0.482111991131122,0.170105588125239,2.834192
55325211,0.00459416407701957,0.0185882986457206,1.92132379625896
"Rad51c",219.006414296289,0.530206107031484,0.261182118758795,2.030024
52675995,0.0423540462561455,0.102395436317709,1.20829366134844
"Gabrb2",2.80321311128421,-2.3765492937055,1.23278045979763,-1.9277960
441518,0.0538804999668663,0.123059855728471,-4.97907137492885
"Cav1",6223.28998497539,-2.41994467501986,0.649410257820776,-3.7263727
2952919,0.000194255086613132,0.00165541332114635,-15.4966107514837
"Arpp19",7580.01352024218,1.10548044658999,0.18286829963500З,6.0452273
5103064,1.4919946009688e-09,2.12342414085326e-07,16.9857796968552
"Bcl2l1",4912.96021113439,0.313886659668857,0.174946531484316,1.794186
24082293,0.0727834475261552,0.154690610406036,0.585815530427878
"Khsrp",3883.4760620913,-0.294700851976408,0.120895134085525,-2.437656
85199479,0.0147827993627917,0.0457298146875635,-0.909153541036113
"Dio2",1097.2250100684,-4.26733980578,1.23116996381304,-3.466085050161

28,0.000528096198490802,0.00361545634767334,-23.9932767777959
"Ccdc124",3764.8709002761,0.232688492539283,0.184279844385285,1.262690
95415984,0.206700266187201,0.339746182543081,0.251200365774833
"Cct4",6496.1130163169,-0.0182314217711745,0.119970463292432,-0.151965
919534168,0.879213815680883,0.924555139904302,-0.00143011988817736
"0610009B22Rik",1263.42142635723,0.492477214106184,0.193454635737948,2
.54569869689393,0.0109059276872741,0.0362563986438995,1.63361556999371
"Cpt1c",517.374229901308,-0.859642703138823,0.2365737545821,-3.6337196
6031209,0.000279364395793167,0.00218318516600207,-5.26700538227045
"Twist2",63.5921620386976,-3.25646004394289,0.624544315555663,-5.21413
767259347,1.84674280637237e-07,7.2304801684033e-06,-38.5473854748373
"Zfp655",1404.98437431128,0.122138114395938,0.19292628414006,0.6330817
74939845,0.526680243073025,0.65912781639524,0.0509117839037353
"Rhoa",13247.5862125003,-0.377866719154551,0.195483458855121,-1.932985
64168849,0.0532379666112057,0.121963796614415,-0.795043301304036
"Zmiz1",4991.30516010363,-0.212519694240345,0.149648463118736,-1.42012
613969663,0.155570961511453,0.275756600686392,-0.273775666176374
"Ankrd26",740.706716003197,0.749879104101219,0.224925200362328,3.33390
435083865,0.000856361005073045,0.00523099212935909,3.93923065459447
"Aldh16a1",3289.31676667564,1.02501366788875,0.2528379059269,4.0540347
9407514,5.03417237442815e-05,0.000581514980048513,7.63622249496984
"Hnrnpa0",7859.09735445067,-0.13323705426747,0.146186777512272,-0.9114
16590028365,0.362075915650751,0.506875086398503,-0.0905333370929411
"Prrg2",1130.60488104265,0.127485391212085,0.16613557510381,0.76735757
0059425,0.442868948296591,0.584224964460668,0.0685194657715826
"Hnrnph1",25487.9468216403,0.0152704905699833,0.174829747106986,0.0873
449216890915,0.930397348092364,0.956679177759194,0.000676286982657088
"Ift43",1038.42278840683,0.185458121098614,0.141858205330395,1.3073485
6448151,0.191094357548989,0.320966772951494,0.210757886281397
"Id3",5224.18779957844,-0.487494623444344,0.123149308001884,-3.9585656
7409122,7.54012091094616e-05,0.000792973449415471,-3.48057551170815
"Tcap",21.6333357107984,-0.260683550225906,0.463764729430908,-0.562103
009743312,0.574045837852811,0.699885591447864,-0.0930219004698273
"Arid1a",6529.49473736182,-0.108118924912295,0.152193151535821,-0.7104
05979646513,0.477452416137554,0.614942691429232,-0.0525702540282461
"Crlf1",533.258664261421,-1.59772809475192,0.530749586657375,-3.010323
76645699,0.00260969344008045,0.0122199334041039,-7.03749180899899
"Ctsd",37697.025492822,-0.849140695911814,0.328409754503631,-2.5856135
0345769,0.00972058619579427,0.0330598272537235,-2.89509120741531
"Rplp1",29112.4216701348,-0.145951142526704,0.257148698845281,-0.56757
4882478868,0.570323666470347,0.697122074563815,-0.0526584046244127
"Hmgcll1",118.487246483234,0.521430348696151,0.217546485465124,2.39686
863973605,0.0165358506364809,0.0497171959460454,1.56502334851816
"Ttc9b",0.49865073136556,-1.0994170777488,3.68750161963593,-0.29814687
3182213,0.765591067777938,NA,NA
"Phox2a",3.72980754124512,-1.99508062675099,1.15064196717863,-1.733884
80835869,0.0829385238620475,0.170892188105375,-3.52475363354753
"Abhd8",888.106190453941,-0.151006693505888,0.190790197292976,-0.79148
035721145,0.428663734176112,0.571375819590226,-0.0845196707045123
"Ift22",928.13204646232,0.524141327301372,0.175280464773837,2.99030087

567183,0.00278702775506792,0.0127952595169094,2.28456459664918
"Fzd3",368.231437397273,-0.342544447143683,0.132063921680393,-2.593777
64029052,0.00949278593353272,0.0324890890173812,-1.17384877046781
"1700008O03Rik",33.2996686971757,0.431838616443762,0.379629801947714,1
.13752559527252,0.255318630958342,0.396038398283671,0.399987973684434
"Mid1ip1",3481.45874242216,-1.23690048686917,0.286532023644228,-4.3167
9667472339,1.58309858502824e-05,0.000240774229414045,-10.3054230187175
"Ap2s1",4675.88647680901,0.343134827497634,0.162338849093889,2.1136950
8539008,0.0345413185423174,0.0875453826775127,0.835738486168443
"Fgfrl1",1626.9878515959,-1.04672810666137,0.471950368841709,-2.217877
50527735,0.0265631856649505,0.0718049347144328,-2.75687466104027
"4930432K21Rik",26.5022264122609,-0.21985614197888,0.29839411099479,-0
.73679785852985,0.461245285677444,0.600558302513443,-0.112103668924945
"Fhl2",2262.67521429417,-0.772854034009268,0.331443504110233,-2.331782
11195905,0.0197121565393313,0.0567973442003666,-2.21675072565357
"Emc10",3614.88083181159,-0.371291676651072,0.30904532076527,-1.201414
9760678,0.229590269670832,0.366269929474872,-0.37291968119003
"Clstn3",1509.63844793456,-4.12134765743294,1.35569637235641,-3.040022
63447043,0.00236560369613274,0.0113523359259651,-18.4567620579013
"Fbxw9",1445.42032169432,0.193806334854948,0.14102372051497,1.37428181
689743,0.169354209034995,0.293709818537783,0.237444352730091
"Spib",323.938526546445,-2.09516627543584,0.548506817225465,-3.8197634
1886489,0.000133579735100759,0.00124499888862074,-14.0137723977576
"Fnbp4",4387.0235358113,0.1021525882733,0.18372604602462,0.55600493497
5908,0.578207481150012,0.703255534119078,0.0359612825611273
"Cers4",2205.48630622496,-0.169222328821624,0.185200339487795,-0.91372
5802499277,0.360860942399887,0.505973439875808,-0.115286282343933
"Scrn3",1505.16720398544,0.62796044576121,0.24136729052193,2.601679972
47396,0.00927683756684063,0.0319519128914076,2.16239638158636
"Phax",1731.70973161004,0.341000102923315,0.123370514924612,2.76403241
999671,0.00570918665386467,0.0219431739359459,1.30238141014782
"Tle1",1150.32975034259,-0.807609520629553,0.154133191754343,-5.239685
95886032,1.60850087510737e-07,6.55427933297315e-06,-9.63913652529554
"1700109H08Rik",50.5577081952208,0.0920372436548091,0.319408038381432,
0.288149428302427,0.773232358028539,0.853273219584609,0.01460405367282
22
"Relt",238.053822587048,-1.76766091901664,0.45898796140588,-3.85121412
248438,0.000117533676902134,0.0011294586654696,-11.9953767252316
"Snrpb2",1847.56145497577,0.046702527948259,0.177786584634841,0.262688
706485825,0.792790522094296,0.866858995053532,0.00667280819721559
"Ubc",57767.9272845157,0.28098820880944,0.26335957292021,1.06693751699
913,0.286000052020884,0.430139743298953,0.237054336386899
"Prpf31",1643.47219482791,-0.289848360031425,0.117199624812627,-2.4731
1679107181,0.0133940363231457,0.0424300439039308,-0.91589142273671
"Sertad1",1082.49752898896,0.0245143389025769,0.190620008327946,0.1286
03178216224,0.897671651341917,0.937131330399138,0.00159176127973596
"Carhsp1",3346.04289651803,-0.982894240331831,0.301533656030975,-3.259
65019384392,0.00111549700646165,0.00640373612531635,-4.96447465791849
"Elk3",2598.4786194526,-0.847099042071938,0.248824641112678,-3.4044017
4366949,0.000663091510600282,0.00428191464783333,-4.61953181503984

"Herpud2",1742.5666128181,-0.179616538107636,0.140072705550446,-1.2823
093364392,0.199734154074839,0.331242369324761,-0.198459199892209
"Rdh13",1264.60275489736,0.381139928409333,0.143220843778315,2.6612043
2162292,0.00778616927537241,0.0278763732154262,1.36447171519542
"Adam21",12.1231920374259,0.280633893747098,0.419620820875177,0.668779
716797172,0.503636007237707,0.638567750625622,0.125872018803778
"Nutf2",178.943680941746,-0.362088312886732,0.320341139904757,-1.13032
098529208,0.258340995081877,0.399392064867303,-0.332328899353798
"Fut1",15.9903122982769,-1.08948758016102,0.748997390355953,-1.4545946
2768389,0.145781519183276,0.262291045587849,-1.45806180677313
"Arpc5",7317.65650771498,-0.166775903054713,0.125055077037206,-1.33361
960990272,0.182328552889462,0.310348504953185,-0.195137713634173
"Rnf151",2.63896703508726,-0.611315172273854,1.19140352592311,-0.51310
5055485044,0.607877835359311,0.728079245275253,-0.193998047668147
"Elavl2",6.8277472727126,-0.803803472215304,1.27582257194136,-0.630027
630716858,0.528676506551374,0.661116888451687,-0.332633665626213
"Pou2f2",1689.40409973463,-0.683131431382526,0.442087347064312,-1.5452
4085776006,0.122288014966566,0.229863271552888,-1.0043880720238
"Mgst1",8226.12716086268,-1.90036451812861,0.614683201763316,-3.091616
15719629,0.0019907007986615,0.00995758957729656,-8.75957868651665
"Nfib",9320.41307498803,0.379526014714237,0.203680671754754,1.86333838
868723,0.0624146723517427,0.137509749323647,0.753002559418296
"Htr3b",2.24362070338,-0.433658140396327,3.43057209194471,-0.126409860
738562,0.899407497027562,0.938343658844678,-0.0275975803880149
"Rab25",753.943967587407,-0.225227019559673,0.299572173941288,-0.75182
8905190019,0.452153956851361,0.592718693739938,-0.117801697670697
"Ubqln4",3735.63113816914,-0.0776553597227601,0.109143513867185,-0.711
497705830301,0.476775871966579,0.614217109455193,-0.0378497515404406
"Rbfox1",108.898902187252,1.08781637780069,0.459810096594218,2.3657948
9197403,0.0179914087280652,0.0529744015976767,3.1959462880703
"Rps18",23240.3943862119,-0.0157634105385488,0.159862279701009,-0.0986
061913293816,0.921450954012274,0.951630324229648,-0.000781528369396185
"Rpl10",4841.99836331743,-0.559123440918997,0.240889946453488,-2.32107
420484215,0.0202828392995386,0.0580897180761463,-1.5911348135644
"Rps15a",11724.1366777057,-0.341878930168672,0.215181686962863,-1.5887
9194133131,0.112107373252696,0.214970837529262,-0.525554376663719
"Ncaph2",4063.42232188325,0.0956783678155883,0.0983392897656129,0.9729
41415823046,0.330582419538278,0.475241172023693,0.0711782830743238
"Hipk1",5115.4425767277,-0.145540595280368,0.10743741528588,-1.3546546
600465,0.175527609736322,0.301846452710381,-0.174333884652082
"Gprc5b",1346.47751300998,-1.25764900480769,0.520352650891285,-2.41691
668650774,0.0156525993185888,0.0476642794805317,-3.82774657899903
"Man1a2",3034.27895262477,0.660814244742067,0.393621854951034,1.678804
76256653,0.093190100129071,0.186897816531231,1.10831318889518
"Tppp2",0.380866608768923,-0.360412436339999,3.18779817189627,-0.11305
9992165566,0.909982993185256,NA,NA
"Acyp1",635.351832170542,0.00397597985561286,0.263645722654418,0.01508
07675375204,0.987967744500225,0.992123643519782,3.14402150531036e-05
"Cldn13",0.586692608390066,2.54827289308836,2.73464194468181,0.9318488
28708311,0.35141465594435,NA,NA

"Cd163",62.8194261157627,-1.24894754651426,0.45098586746923,-2.7693718
0653333,0.00561645013840629,0.0216678956863169,-4.78587154790038
"Hdac5",4864.22302474704,0.124912117065515,0.174352931602043,0.7164325
59623513,0.473724296235153,0.611456249529657,0.0614457534116101
"Rala",2720.8128194841,-0.057016393797188,0.154362091162641,-0.3693678
50407738,0.711853557991994,0.80874727151427,-0.0121028018839269
"Vdac3",5944.27188331875,0.164974355921264,0.218841214514081,0.7538541
41632217,0.450936809542916,0.591530095506957,0.0866185840484432
"Slc1a7",5.62670552660073,1.50384826410301,0.674857478874349,2.2283938
6267364,0.0258542620033047,0.0702881205438419,3.99294644309123
"Vps72",2080.79860983218,0.0405860509831707,0.169602358670392,0.239301
217868358,0.810872021117248,0.879527295713401,0.00521005895495191
"Gabpa",1965.7338407372,-0.219498195194216,0.140891265347077,-1.557926
21106423,0.119250740985179,0.225414536861756,-0.327011524965949
"Bmp7",1494.90448556628,0.61381565830401,0.18518028171894,3.3146923236
4404,0.000917440442909814,0.00550577216246708,3.19304342952425
"Dynll1",6217.27675793993,0.063322202042375,0.221515976189509,0.285858
39780785,0.774986578421336,0.854443956493554,0.00996085877588266
"Pdcl",1476.78168450294,-0.0494839286150015,0.11282217844089,-0.438601
073821022,0.660950618815432,0.769738346085997,-0.0129501733360666
"Tmem184b",2921.59084535914,-1.29552093185822,0.271009278392819,-4.780
35637577104,1.74984730513766e-06,4.25417989470998e-05,-13.039448573006
9
"Rsph14",0.0784399670938122,-0.00670295202529588,4.78471990596623,-0.0
0140090792293562,0.998882237562793,NA,NA
"Nf2",3530.48865262341,-0.155176329913855,0.177835269160992,-0.8725846
71454432,0.382889498539119,0.52766029642258,-0.0992046281184911
"Cabp7",1.58822204878087,-3.78024896734766,1.49918408164493,-2.5215375
5741584,0.0116843201602689,0.0382570738009947,-12.3365657900887
"Zmat5",1029.99834083547,0.137425690013828,0.168004073799293,0.8179902
24320423,0.413362769281445,0.556881591615395,0.080449362291989
"Ewsr1",16732.8718801389,0.233862765853323,0.144094015571611,1.6229873
5950696,0.104592107050279,0.203982924247763,0.371776080704845
"Ap1b1",4242.78974806861,0.223633262234664,0.108615380983576,2.0589465
3417898,0.0394993605938356,0.0969972963297994,0.521752541245946
"Derl3",86.7188140182705,-0.419187602467383,0.561188414043035,-0.74696
4106844939,0.455085227267177,0.595320480459941,-0.217413911880497
"Gstt4",5.8129016344883,-1.22368631781913,0.800414806036811,-1.5288151
9505881,0.126310264361459,0.235269156908648,-1.77070478178272
"Tbx1",55.4261150302235,-1.15595350655251,0.424782695097061,-2.7212820
1053102,0.00650292586979834,0.0243179993496985,-4.29614569842294
"Gnat2",13.8571650377563,0.193078381849373,0.537312192228193,0.3593411
51461111,0.719339892875785,0.81414043269295,0.0397012414463867
"Bcl2l13",1660.37609897681,-0.813678596342476,0.422919476172294,-1.923
95631363875,0.0543600595979397,0.123831376708811,-1.69963992727518
"2610028H24Rik",2.91471010068386,1.88343995232459,0.93489899354151,2.0
1459191349634,0.0439474254717656,0.105411078454253,4.23752808055398
"Spatc1l",5.55280671684622,-0.451013078700067,0.705571169850752,-0.639
21698897571,0.522681781578247,0.655934181451545,-0.190189882749603
"Dqx1",656.346426628864,0.562083776121037,0.214968611323865,2.61472487

848107,0.00892993538747286,0.0310090614755024,1.95238440042032
"Ccl8",635.275523401682,-1.64594333618726,0.582966847143942,-2.8233909
7712165,0.00475185841694883,0.0190995125569302,-6.51479591650783
"Lnpk",405.847783672109,-0.192197392505094,0.11172696426388,-1.7202417
8560116,0.0853885003949387,0.174848244139941,-0.335160896988214
"Prr29",3.61265762863628,-1.11850258632117,0.820371695806163,-1.363409
52770444,0.172753480010696,0.298208440644914,-1.35334626583982
"Mymk",4.63448303915381,-0.517315315521669,0.919855884626314,-0.562387
352375123,0.573852134324756,0.699791663960479,-0.184667398970082
"Fam163b",4.30431331945294,1.18918884060527,0.922747331675586,1.288748
06762742,0.19748568572056,0.328599277923746,1.32346760977966
"Trpm5",7.37095030748189,-1.74693418098837,0.939971161464913,-1.858497
63546558,0.0630983788574002,0.138655718580141,-3.45152488389998
"Ascl2",15.1653505427426,-1.20757687865508,0.57310759379002,-2.1070683
6157806,0.0351116543207317,0.0885993342231072,-2.92672068047881
"Rarres2",4394.49260259722,-0.727783719786427,0.319334112044972,-2.279
0666337705,0.0226631045890641,0.0633940843275661,-2.00750749869445
"Pttg1ip",12922.0942251192,-0.0516090658608587,0.227448704291413,-0.22
6904198120803,0.820498237564142,0.884849260842126,-0.00631374862007834
"Trpm2",608.530654650789,-1.07979399806428,0.448750553779929,-2.406223
21013073,0.0161184130956239,0.0488083271530477,-3.26082059384172
"Ube2g2",1951.96192862514,-0.803864037185045,0.226049191159877,-3.5561
4648767533,0.000376334292250208,0.00275418185169263,-4.73848496745986
"Mpo",0.793586978571083,-0.558212666915032,2.94354097034593,-0.1896398
49602443,0.849591358332994,NA,NA
"Lpo",11.3729317004978,2.11046212072114,0.751942534416638,2.8066800641
3342,0.00500549284759712,0.019901529270483,8.26659296993152
"Met",5138.35098977621,0.60485868287321,0.221176977416922,2.7347271399
4568,0.00624320047956393,0.0235910703392515,2.26633714416766
"Slc16a12",3503.18933486739,1.03884994823799,0.300770101956677,3.45396
680547597,0.000552405364736156,0.0037346739950158,5.80726963960987
"Syn2",314.872402060861,0.184995923429484,0.258575404985135,0.71544284
5154277,0.474335445800627,0.611962715689845,0.0908485233359639
"Elk1",455.753759020045,-0.526579524617464,0.231282654744967,-2.276779
14367646,0.0227994175852246,0.0636789921324608,-1.45014765017026
"Nav1",3424.32257571127,-0.989470921503041,0.29087850266095,-3.4016639
6777824,0.000669769365374906,0.00431565204430929,-5.38817068565335
"Tnpo1",5417.37442443135,0.0341058747178155,0.167716963935658,0.203353
756933614,0.838858541212173,0.896910717073642,0.00371068359387784
"Otog",1.02121442350651,0.319106559841026,2.21530653829503,0.144046232
123985,0.885463962337975,NA,NA
"Rnmt",1168.91539314986,-0.467176661978863,0.154859396541034,-3.016779
55883725,0.00255475523993624,0.0120262234834082,-2.06523185628148
"Kcnq1",939.897870576827,0.788599082781976,0.323918009033256,2.4345638
7354188,0.0149097481266394,0.0459984602805719,2.42821278007288
"Srp14",3426.28338313897,-0.143193899658858,0.17166883814102,-0.834128
670115596,0.404208480779259,0.548100444892579,-0.0861020215059522
"6330409D20Rik",2.36613850110265,-1.02733991786357,1.42673290388566,-0
.720064642138442,0.471485196157118,0.609414541244381,-0.50879682456880
7

"Cdk9",4490.42587102911,0.0130921747058288,0.148539059691139,0.0881396
09426986,0.929765715095927,0.956462801665501,0.000582776959618435
"Tor2a",1807.63183787592,0.303535086071822,0.137576798406526,2.2062956
0788954,0.0273633087153325,0.0734181633867556,0.792707348844927
"Fpgs",2814.08812468716,1.25746984850608,0.304193283930159,4.133785704
46936,3.56836215669137e-05,0.000447723103121004,9.69677199616955
"Mrtfb",1447.58502496695,-0.445639896177361,0.147080072486554,-3.02991
349299274,0.00244623794045239,0.0116333530153514,-1.98482619590411
"Cbx5",4418.29131035393,-0.122308203633085,0.102769528001867,-1.190121
29384172,0.233998722034185,0.371660726802096,-0.121057463502325
"Odam",0.419510291884049,2.30453208788503,3.63537310403797,0.633919001
415642,0.526133684285244,NA,NA
"Apobec3",3122.77276493224,-0.944356565590775,0.242288045847385,-3.897
6605811812,9.71264169516139e-05,0.000967196566763314,-6.55488167563179
"St6galnac1",0.470166085995672,0.365681046456519,3.08534196968258,0.11
852204716683,0.905654027102129,NA,NA
"Taf7l",0.289932122451383,-0.474970018094316,3.93860283167104,-0.12059
3529836264,0.904012993883157,NA,NA
"Sardh",9493.91490786409,2.13281605906661,0.52731325722422,4.044685070
68032,5.23935060583371e-05,0.000598809250366621,15.8267054404532
"Vav2",2774.96686831262,-0.251730732741367,0.146305224948243,-1.720586
07497045,0.0853259620989547,0.17475689170225,-0.439108885319842
"Tex15",20.8691961046249,-1.91072034278394,0.861941999463463,-2.216762
08372874,0.0266393537845957,0.0719502191504199,-5.02859711189237
"Ppp2cb",1994.28162185693,-0.500324225445269,0.24686458098936,-2.02671
530861218,0.0426915359719687,0.10300700017095,-1.13721611604526
"G0s2",3340.35174916624,-1.23111969444973,0.520456695505711,-2.3654603
8331487,0.018007669805664,0.0529982710748432,-3.61640916219847
"Dmap1",1127.98491545326,0.0797268635523972,0.139463834070431,0.571666
942069971,0.567547634400833,0.694623298948431,0.0290513207136873
"Pla2g12b",53.3406883530141,0.340751408051532,0.700672254281268,0.4863
20681273538,0.626739818274894,0.743364740965015,0.101056117490006
"Mcu",1461.63966722702,-0.328087126900658,0.205247545538522,-1.5984947
6416311,0.109932909734163,0.2116732191412,-0.509424691904251
"Oit3",15.3857661413786,0.520764921609929,0.448590034898567,1.16089275
529199,0.245685515426246,0.384774960622724,0.497380823450878
"Tex11",2.53991180032195,1.73025090117996,1.35790767495965,1.274203639
2359,0.202591255808955,0.334614511288692,1.89423737221058
"Bcr",2416.88309273733,0.137479933406165,0.172587945611994,0.796579001
613719,0.425695576699273,0.568655542503614,0.0776047280243753
"Fxyd5",3083.2007801237,-0.958090296682737,0.330722032196801,-2.896965
43746626,0.0037679126375O524,0.0160547990834959,-3.95858715530988
"Kcnd1",224.413190893677,-0.501164776849126,0.273399981463713,-1.83308
270236896,0.0667902730855839,0.144745664624065,-0.968639810917242
"Tfcp2",1981.25276048705,0.460917291385846,0.165335031832765,2.7877775
5855191,0.00530709702760292,0.0208219457065266,1.78455549343589
"Pou6f2",1.47939658334817,0.676422590772239,1.83061545119828,0.3695055
61820462,0.711750928309892,0.808724918425773,0.143602112294821
"Pou6f1",2492.34424750776,0.200011880906823,0.138741905498618,1.441611
17139057,0.149412095144538,0.26722042112715,0.2639519617851

"Ubp1",5671.85624577247,-0.0265976549029509,0.158545127751711,-0.16776
0783823039,0.866771475155677,0.916273245901195,-0.00232571637697747
"Nuak2",4318.0998160586,1.10991883154079,0.442668433577929,2.507336749
923,0.0121644758362685,0.0393664647063174,3.59041091485661
"Ick",1171.43087240627,-0.29615236080046,0.174277108995155,-1.69931876
026641,0.0892591396725661,0.18093795879938,-0.506302394547956
"Sdhb",11890.8528998025,0.862378975202713,0.392200779538799,2.19882014
568357,0.0278907134463647,0.0743655716730393,2.24111787470219
"Cox4i2",86.1071231860088,-0.286666404157362,0.363325444157349,-0.7890
07235158606,0.430107777820177,0.572791575161383,-0.159740086916451
"Snap47",1705.01734223776,0.199951069228282,0.15995492359415,1.2500463
5515699,0.211282614370249,0.345284419997578,0.212625326822746
"Wnt3a",1.76808834535056,-0.662677408446103,1.93040563450337,-0.343284
021037676,0.731384800631727,0.823975318814203,-0.128304089234527
"Kdsr",1390.54275686804,-0.0850678791300729,0.200499646480614,-0.42427
9446987943,0.671362026560469,0.777744447381381,-0.0213824309317825
"Vps4b",3071.93372131597,-0.293371287451388,0.140596497275096,-2.08661
875037589,0.0369226109911278,0.0921788820877092,-0.699404254518471
"Rps25",15018.5365457686,0.138592897379211,0.143675585601258,0.9646238
55884934,0.334733246189912,0.479414717179974,0.101892009428137
"Nxf2",3.93401077238672,-1.64453153395648,0.829868296406201,-1.9816777
4462313,0.047515319625052,0.111931098471579,-3.60131321268324
"Taz",3554.32011462796,0.0886130156417955,0.132055244128466,0.67102988
7730856,0.502201489827889,0.637174124362563,0.0399389769862402
"Kif19a",97.7712256969902,-1.21979094984187,0.387674178148541,-3.14643
331590296,0.00165274944887648,0.00867204218358277,-5.79114168615743
"Aldh3a2",5432.05692955557,1.28276211158954,0.256686502529557,4.997388
24966786,5.81119943010104e-07,1.77318883392974e-05,14.0336047048229
"Zmynd10",682.124781703352,2.007169856075,0.539138557335652,3.72292025
63329,0.000196931735010123,0.00167456387668006,12.830236211547
"Tmem115",1657.12739199734,0.234644983680624,0.118435356704513,1.98120
721893922,0.0475680400033682,0.112028221281033,0.513638916662657
"Hyal2",2437.80723525359,0.243115648731792,0.137320490612806,1.7704251
3937188,0.0766563449424083,0.160818314362715,0.444288993872297
"Ifrd2",2651.78593354113,1.22867942392762,0.240928054270992,5.09977730
756758,3.40053327124109e-07,1.18278652441857e-05,13.9394323845846
"Hyal1",1183.4489416565,-0.67908606110512,0.280417567936184,-2.4216958
5202188,0.0154482740689304,0.0471525434871643,-2.07417827710647
"Tusc2",2061.28534354364,0.366910800828596,0.163291730692762,2.2469649
8268457,0.024642265118462,0.0676527108351782,0.988225756125134
"Nprl2",1352.06563215424,0.543354408866966,0.127912787311594,4.2478505
885683,2.15831251653854e-05,0.000305304920043773,4.39801900789103
"Slc38a3",954.167907283139,2.20181487714563,0.662618431317939,3.322900
13841941,0.000890868004014341,0.00539445476468063,11.4987222315223
"Cacna2d2",26.7752870170146,-0.265467307120391,0.487680217570675,-0.54
4347089662129,0.586202648255373,0.709791916766023,-0.0909977932590866
"Rassf1",2110.60008429929,-0.145493665296123,0.128023886793718,-1.1364
571795149,0.255765276890967,0.396573468693446,-0.134566212545138
"Epn3",2247.75780518841,0.186118661162645,0.185912165772784,1.00111071
477761,0.316773285460122,0.461928466075946,0.143747861134881

"Rnf112",2.42969677947373,-1.52216006588169,1.04466316671274,-1.457082
16235048,0.145093701625558,0.261367054166216,-2.04247931334267
"Slc3a2",6848.15694005767,0.344050516389693,0.122506304870199,2.808431
09874411,0.00497835342298344,0.0198300173731013,1.34887016281901
"Nxf1",9785.78865646898,0.496259029821659,0.3235038318826,1.5340128335
8446,0.125026495579461,0.233637253305616,0.721553466345757
"Stx5a",3814.64454679815,0.0304289617566893,0.102929934886822,0.295627
912231246,0.767514255035616,0.849837719005984,0.0049510923064298
"Slc47a1",16988.0260610134,1.89239410988164,0.555063729207076,3.409327
63267557,0.000651232168554356,0.00423257494082428,10.3418292331549
"Pifo",1.80228903696963,-0.308858308745278,1.33741008541576,-0.2309376
2497631,0.817363260167262,0.882982372804105,-0.0384374293183292
"Tnfrsf13b",272.0426834743,-1.45775723681043,0.426544679775188,-3.4175
9563752792,0.000631768933051832,0.00412947725666933,-8.0025106175017
"Spire2",344.978508007684,0.319548764053916,0.270391866180629,1.181798
72999748,0.237285571863223,0.375443810775714,0.313044814668272
"Prox1",293.006981679864,1.11328593239513,0.391640992781458,2.84261850
24415,0.004474459366577,0.0182173749696217,4.45913261968679
"Raver1",1902.09557221239,-0.0330743745443576,0.0932952774269172,-0.35
4512848415788,0.722954576837578,0.817121809702574,-0.00667993555576497
"2610507B11Rik",12141.8169913448,-0.074159875885803,0.220088619678091,
-0.336954613983548,0.736151114134817,0.827623503626862,-0.014030821126
4728
"AI597479",852.334417611252,-0.338989566779915,0.121410132680122,-2.79
210276190907,0.00523667223818893,0.0206188681947108,-1.31580450906641
"Tmem86a",1888.09329728132,0.570959515694298,0.174616141821858,3.26979
802518365,0.00107624299531711,0.00622646311848861,2.89987301951566
"Optc",24.1278383492861,-1.89231562917049,0.808265999335664,-2.3412040
4758562,0.0192216586321475,0.0557465268495629,-5.46300200274261
"Tex14",29.7035917661799,0.520572652732213,0.770828609136151,0.6753416
34394715,0.499458737899563,0.634828199671814,0.236548665463212
"Ifi35",2019.92174289899,-0.478982893053072,0.149623133161709,-3.20126
22843247,0.00136826922263135,0.00749256557370926,-2.34406756057473
"Rdm1",921.609866604244,0.207414038297014,0.210116470643069,0.98713840
7865964,0.323574791221704,0.468483053333391,0.15727280423254
"Nedd8",5468.82133396185,0.388023430042866,0.188141170067566,2.0624057
451302,0.0391691227997963,0.0963805598624973,0.907761707876771
"Gosr1",2301.35873593342,-0.0782220409872624,0.149390320827947,-0.5236
08494537947,0.600550872068754,0.722231598322123,-0.0254541888501723
"Mrpl52",2139.45258306349,0.123965886552125,0.166994329235453,0.742335
905175194,0.457883842733027,0.597698277137883,0.0638014243585472
"Spaca7",53.4318607227334,-1.29452364215602,0.530073163383453,-2.44216
031215972,0.0145996618705654,0.0453503595909327,-4.00439810388646
"Kansl3",5019.62107661652,0.62449492475636,0.170101853240327,3.6712999
4682685,0.000241319929241689,0.00195522171231303,3.89513202117488
"Eya4",177.553917614978,-1.22630382176962,0.318224008505369,-3.8535867
4705063,0.000116399996455768,0.00112049493542002,-8.33148950647576
"Ebf3",262.980257611468,-3.42399835632306,0.793502352053816,-4.3150449
9950221,1.59570395144168e-05,0.000241936513570838,-28.5110700615975
"Myt1",1.38082700092576,-0.342203159565819,1.67931201473819,-0.2037758

06141165,0.838528700039751,0.896803937983383,-0.0372720892375025
"Faf1",1684.68623911115,0.0189011596476459,0.214461487862416,0.0881331
181464693,0.929770874311026,0.956462801665501,0.000841354519032934
"Gm266",157.095476045499,-0.091128456632901,0.474076019580179,-0.19222
3299363677,0.847567295957505,0.902756863136645,-0.0093226248627675
"Tsacc",90.8364894822045,0.490064128957127,0.216222757301327,2.2664780
2975788,0.0234221308347598,0.0650150074568486,1.33941247760967
"Mettl16",1505.46446761619,-0.89563097148423,0.278021742625859,-3.2214
4219018691,0.00127547190912525,0.00707716314289245,-4.43416337860686
"Dazl",6.35661289047142,-1.32013002986867,0.878485046810868,-1.5027347
7580647,0.132907451251876,0.244152014745533,-1.86133612954904
"Apol7a",15.0678452997277,-2.45254952255552,0.918641579204625,-2.66975
671260055,0.00759062237931253,0.0273759258655944,-8.82449702596508
"Pigyl",1088.37622170464,0.215620651717606,0.240141604570525,0.8978896
10187402,0.369244404303899,0.514251956794102,0.143396777668288
"Rbm25",16520.7773689647,0.428213880866429,0.253272997091502,1.6907206
2866506,0.0908901725132526,0.183398176928842,0.72629170472615
"Psen2",1519.73775950456,-0.0717324067323387,0.146858488067388,-0.4884
45766236021,0.625234131558026,0.742283794261141,-0.021377952737136
"Acaa1b",10067.9443823756,1.56999713750996,0.637068201758783,2.4644098
2798325,0.0137239090622383,0.0432711479455891,4.93021364758591
"Plcd1",1020.12930073378,-0.535783064791088,0.172003276517302,-3.11495
848009147,0.00183970803679038,0.00936325432037034,-2.50262253150803
"Fads1",4345.71374002879,-0.520897440360447,0.212981003508148,-2.44574
60138717,0.0144552791942186,0.045059797558573,-1.61465956915439
"Lmbr1",754.26260627986,-0.158096726469559,0.188118474400093,-0.840410
422069002,0.400678307616258,0.544799556617977,-0.0960180449990846
"Tnfrsf22",455.982518650203,-0.521567891872909,0.140777233637004,-3.70
491647262921,0.000211460403441729,0.0017657622896389,-3.30630898813607
"Cars",1286.07037724644,0.0549265276430806,0.173638696680499,0.3163265
37189733,0.751754670733997,0.838578454495305,0.00966965792627429
"Phlda2",319.777539271922,1.70503506072212,0.518579199595309,3.2878971
2748352,0.00100938715686871,0.00591554877806613,8.74712143698998
"Asz1",9.24285514588387,-3.43737903509324,1.13406853131546,-3.03101526
951467,0.00243732921615936,0.0115966434984566,-15.32053439823
"Wnt2",49.0278491635329,-1.40415608688748,0.501170292396173,-2.8017544
2996431,0.00508255457167677,0.0201422050891369,-5.48314233474506
"Gabra1",0.810899549368993,0.0462763950852629,2.49537125943239,0.01854
48938350717,0.985204163612424,NA,NA
"Grid2ip",3.45011908550919,-2.97316814353492,1.40637083669841,-2.11407
124348136,0.0345091828551643,0.0875043587896175,-7.24283582507668
"Kdelr3",419.333554890934,-1.48612776021508,0.321129550807364,-4.62781
377945055,3.69546073407942e-06,7.8747643052547e-05,-14.0428108841264
"Apip",1005.25870700823,-0.292799197441439,0.279862237350167,-1.046226
17261179,0.295456621829093,0.439823882457143,-0.240499668120761
"Pdhx",1951.17418974387,-0.50611074964112,0.420012133126353,-1.2049907
8413258,0.2282068596466,0.364689562280912,-0.510518367742824
"Vac14",1434.55640773464,-0.313817522866081,0.267807542513045,-1.17180
240676304,0.24127639619776,0.379830393693235,-0.303784920477564
"Mcoln2",90.9285515239506,0.367600041389564,0.440888253676081,0.833771

456428135,0.404409782817654,0.548267413807441,0.220924731737041
"Slc5a1",2028.95093768744,1.78481762034766,0.474732686399951,3.7596265
6770591,0.000170167171259097,0.00149854117883885,11.6071387340294
"Akt1s1",3147.34644590242,0.192868475696999,0.167175815888708,1.153686
46279194,0.248628735851651,0.388069411754362,0.182563717304572
"Tbrg1",1961.15894323301,-0.24525837067001,0.133508844384567,-1.837019
65064991,0.0662069945001794,0.143753227033909,-0.475717152420749
"Adssl1",353.096706532316,-0.863866353105558,0.313101322421999,-2.7590
6325282535,0.00579673137593883,0.0222183346506891,-3.28859878116307
"Cfap161",6.02722129316117,0.288748674418744,0.930311612385889,0.31037
8448010786,0.75627318142867,0.841774546120265,0.0497349554105096
"Brf1",3097.97957727126,0.230939463686243,0.169997366512711,1.35848847
793166,0.174308736208193,0.300342147277072,0.277781599441263
"Vipr2",32.3749046256383,-0.437660719237711,0.364592892849934,-1.20040
935470964,0.229980397537159,0.366682295459934,-0.439087268133137
"Odc1",23696.4048967985,1.1792349420205,0.431348410487261,2.7338339804
9017,0.00626015999227285,0.0236276808999876,4.41663147560598
"Thg1l",429.229998376267,-0.0774120096380692,0.183577469660051,-0.4216
85786286415,0.673254380540743,0.77924218875599,-0.0193091395602359
"Adam19",817.511920776797,-1.57434255765187,0.251455531388379,-6.26091
8377732,3.82716817177413e-10,7.31589460914236e-08,-25.8674422921142
"Pabpc4",4924.35448684361,0.018724338335324,0.165257033640992,0.113304
335209121,0.909789280375931,0.944789366596963,0.00106341638060343
"Exoc3l2",1689.65742613471,0.308855716528717,0.312570035401833,0.98811
6842779438,0.323095427928334,0.468111873190333,0.23443630352755
"Zfp296",138.801421934172,-0.142160390741746,0.200117308851967,-0.7103
85281299711,0.477465248002212,0.614942691429232,-0.0691221066072443
"Plin5",409.118993764181,-1.38887174680487,0.818673763823371,-1.696489
86956485,0.0897931470634273,0.181711134555548,-2.36849441359458
"Sugp1",1702.11211610513,0.375031013515058,0.0914383780801428,4.101461
78649796,4.10548314050645e-05,0.000497815362397977,2.85221635697803
"Dmrtc2",0.339298802794277,-2.39089727048973,4.74033101555884,-0.50437
3484181223,0.613998947566137,NA,NA
"Dhdh",1929.21695628448,-1.75976584557846,0.607964375541691,-2.8945213
1798104,0.00379737123606901,0.0161485592935384,-7.26066091292593
"Zfp790",464.424710170756,-0.503279793904259,0.134951513193152,-3.7293
3790808206,0.000191983557408982,0.00164107645872877,-3.22723279745858
"Cpb1",6.23358915778619,0.095009355665704,1.03330264852206,0.091947268
113168,0.926739935972119,0.955102028203625,0.00436445506952955
"Slc25a41",0.587226725782629,0.617778155136654,2.50660494627753,0.2464
60119714554,0.805326072268551,NA,NA
"Fsd1",17.0520817526781,-1.08143687073894,1.11605003133061,-0.96898601
3511958,0.33255216762663,0.4774040763057,-0.799605799452171
"Pinlyp",2.36817900434545,0.552965233939707,0.975290915697048,0.566974
658576101,0.570731398864049,0.697376796331572,0.199304936874881
"Fuz",1583.57495561349,0.283256585883702,0.128616269646825,2.202338682
82382,0.0276413936619311,0.073930300908139,0.737779312050449
"Sptbn4",30.3680264368056,-0.213531697255068,0.327800341019599,-0.6514
07794729233,0.51478328027512,0.648568389687535,-0.0924566246327956
"Pgam1",11968.7462556138,0.0719726298963621,0.349119493702596,0.206154

71549026,0.836670059796503,0.895848040961681,0.00791587209571284
"Evi5",3831.25857656646,1.10134209269824,0.18071857634555,6.0942384284
4122,1.09959668193513e-09,1.674995008154e-07,17.1834537258812
"Evi5l",1265.81217903267,-0.123877536508243,0.127169883979649,-0.97411
0635565785,0.330001609293868,0.474616167146997,-0.09231959419723
"Snapc2",934.072550574477,0.245584084706871,0.124078872879636,1.979257
8624171,0.0477869812165372,0.112462348794212,0.53663481871305
"Git1",2800.22694871119,0.0247075234341547,0.117592313247385,0.2101117
22032171,0.833580476826982,0.893668671972975,0.00277762436791628
"Gltp",3155.46288797012,-0.145949662391101,0.12902574096523,-1.1311670
1597111,0.257984801881701,0.398936288927702,-0.134120960625894
"Bnip2",3512.42479051315,-0.686667278858385,0.137773996672483,-4.98401
218983822,6.22791533407349e-07,1.87975066847933e-05,-7.47216660152285
"Ccnt1",2862.84723139319,0.482946214967946,0.254440344057348,1.8980724
804361,0.0576865347648397,0.129791374895551,0.986092585582921
"Scarf2",3368.76468853363,-0.757376908944232,0.349189021182357,-2.1689
5968372587,0.03008574486261,0.078877484648537,-1.9236309050939
"4930579F01Rik",24.6680708955411,1.82074958788112,0.60297907874856,3.0
1958998587473,0.00253117088943838,0.0119411492867819,8.06185115419035
"Brms1l",840.147601318026,0.455986567476034,0.175636523319619,2.596194
45237048,0.00942627020886256,0.0323313009777889,1.56481797139691
"Med15",1908.97630410058,-0.243596714127743,0.190337278261366,-1.27981
610514175,0.200609818397415,0.332178995590018,-0.268463386306687
"Dhdds",2288.17304555363,0.274126360883696,0.161432920391858,1.6980821
5213036,0.0894922576541236,0.181271560330366,0.468141770565607
"Crybg2",258.101550950979,0.218466407869865,0.348243888003696,0.627337
37876125,0.530438106525193,0.662682270841815,0.0898901080405635
"Ubxn11",292.435876740857,0.0717728584734732,0.43170926771129,0.166252
762777083,0.867958038460555,0.917232035449201,0.00620080183700243
"Mogat1",3273.92539488321,2.14196647801339,0.634808017435321,3.3741956
925294,0.000740317182921786,0.00466736301593826,11.4962790114995
"Tex22",0.565752516713439,-1.51823250845711,2.35982564459928,-0.643366
391043235,0.519986386061911,NA,NA
"Wnt8a",0.154367723061889,-0.541723841893663,4.78471990596623,-0.11321
9551518192,0.909856495461738,NA,NA
"Tjap1",1453.54010189641,-0.22176054896551,0.135051266166645,-1.642047
16668018,0.100580229596057,0.198212382925401,-0.35890086100128
"Ehf",2187.00868808527,-0.087668305358946,0.228872677072969,-0.3830439
98436719,0.701687141329453,0.801540563691,-0.0193939560788662
"Nanog",7.54206242570197,0.704310476103136,0.644688171514643,1.0924823
9260915,0.274621125498149,0.417900780844681,0.614518807577661
"Rpl15",16589.8975808296,0.281501060312397,0.173826973361084,1.6194325
5910948,0.105354226142367,0.205065551941611,0.446017482273096
"Tmem167",2374.57814236964,-0.152289453200793,0.130347520595785,-1.168
33410029371,0.242672025461961,0.381495737256774,-0.146754584696204
"Steap4",1355.185009478,-3.39715087434567,0.811313938303163,-4.1872211
408702,2.82390652256071e-05,0.000372030603897896,-26.8257188721606
"Mplkip",869.627574474675,0.312929732668326,0.167240649861444,1.871134
39781287,0.0613264548671396,0.135839267217332,0.624696290151694
"Kif11",206.793256217329,-0.856920319092819,0.400244838824693,-2.14099

030385786,0.0322748199878473,0.0831520137583307,-2.13123354777694
"Rpa3",635.531448600682,-0.0882045490592144,0.146333674051495,-0.60276
3168702886,0.546666251932,0.67654069058522,-0.0344670463750869
"Mlkl",290.259270365444,-0.93128157502899,0.33450271303199,-2.78407779
293535,0.00536801576158625,0.0209834746029288,-3.59849070112491
"Phox2b",2.69547191976165,-0.951970288176452,1.46917883188371,-0.64796
0797907688,0.517010305876795,0.650617461209871,-0.409188651061908
"Tnpo3",4082.46524457557,0.45077453261396,0.19005896800469,2.371761445
12601,0.0177035168001636,0.0523242641457617,1.32991788300934
"Ttll5",424.67840125927,0.134648638513526,0.159814016379316,0.84253334
9477555,0.399489480606943,0.543753176824292,0.0820360098637052
"Zfp715",1363.51137610103,0.0843134184017901,0.137822200768356,0.61175
4985276281,0.540699876447009,0.671287245524596,0.0336037998799322
"Retn",5895.27062357039,-3.61830146788352,1.56988546508194,-2.30481875
803256,0.0211767260979215,0.0601199483775878,-10.1725418571233
"Cdh23",48.4359163681,-4.59850913406841,1.05958386856867,-4.3399199161
8396,1.4253465121974e-05,0.000223859249642613,-38.6481380733364
"Rps5",18047.6008109272,-0.359286904993277,0.228627456854084,-1.571494
99861945,0.116067715223801,0.220724501261453,-0.542825011639515
"Podnl1",297.556323897942,-1.59778082025507,0.3193394804254,-5.0033926
8457075,5.6330030338211e-07,1.73785273976968e-05,-17.5121174536184
"Adgrl1",3527.02404091511,-0.0325816120125978,0.283637043919072,-0.114
870792483277,0.908547536036251,0.943836105153968,-0.00188330662165491
"Amotl1",3402.8210095476,-1.07997758889723,0.238199758420848,-4.533915
55078381,5.79001861057407e-06,0.000110897625608029,-9.83525122422711
"2200002J24Rik",2.83402418175693,0.518287804604956,1.73567741984364,0.
298608369665629,0.765238878599208,0.847955652132721,0.0854796224670802
"Etv5",665.263565470689,-0.00501402701178931,0.254598648546305,-0.0196
938477105756,0.984287598645118,0.989769963815499,-5.15578490493494e-05
"Tmem190",1.87008671873307,-0.597364519640158,1.2723156095237,-0.46950
9699612808,0.638705353345967,0.752786575809673,-0.169635707286633
"Gfod2",1160.26235349029,-0.288049575404521,0.115086371454435,-2.50289
909886129,0.0123180656564838,0.0397381434736702,-0.929087705492131
"Enkd1",626.431843692469,0.479823219214593,0.26941495238575,1.78098214
284551,0.0749153680094688,0.158067292008292,0.885146416559289
"4933405L10Rik",3.16157787229646,-0.847164487964381,1.17914461837236,-
0.718456815868583,0.472475665584672,0.610371067883783,-0.4182351101105
54
"Atp6v0d1",4240.11163732374,0.232108725250991,0.170265918618226,1.3632
1306773923,0.17281537000184,0.298221859819639,0.280832425629322
"Ptprs",4654.5109095341,-0.983989106957544,0.187987664766334,-5.234328
05115501,1.65586136182437e-07,6.66807296013809e-06,-11.7273589516455
"Slc41a1",1105.37571329767,-0.275177571961155,0.129970985452054,-2.117
22309409331,0.0342409182919069,0.0870556037104708,-0.671765756512001
"Fer1l4",1021.79379933638,-3.27530902098234,0.770272819265385,-4.25214
149982084,2.11736000510423e-05,0.000300461900807526,-26.5633771522894
"4931406B18Rik",1.69174801777026,-1.00735587072103,1.38491603289743,-0
.727376856641268,0.466995127942129,0.605537740547691,-0.50532837550644
1
"Iglon5",170.149732425501,-1.28804502662911,0.441750685350518,-2.91577

369168556,0.00354807758755428,0.01535977282463,-5.37888033512207
"Igf2bp1",234.96213755088,1.06301805303403,0.382536449468972,2.7788673
5893975,0.0054548794295042,0.0212215114009021,4.09553209182689
"B4galnt2",6.2965127204864,-1.71583631812918,0.908128669751904,-1.8894
1983144077,0.0588355956417235,0.131564182111945,-3.48016297625935
"Zfp651",1295.30180775774,-0.275157809001607,0.156548396203374,-1.7576
5332430583,0.0788065142567904,0.16439172087075,-0.496798292906809
"Nelfb",3841.52091887558,0.0286502995641721,0.120121960978743,0.238510
088669316,0.811485491482108,0.879810136779931,0.0036686464619501
"Card14",224.06279984055,0.137500827547357,0.196076826484322,0.7012599
60255173,0.483140796453229,0.620735322169548,0.0655673383925363
"Tmem175",1194.09189933634,0.272676854810605,0.0954839211268169,2.8557
3583062691,0.00429372220848485,0.0176958350498917,1.10009458583121
"Bcas1",69.3310187621699,-1.99657761820699,0.419902352673199,-4.754861
70890994,1.98582302489366e-06,4.74093718717351e-05,-19.8793056632574
"Tango2",2472.94550867125,0.934445811035078,0.19885768174515,4.6990682
1217315,2.61351158840056e-06,5.89794548039748e-05,9.09993363721444
"Aldh1a2",1712.07956381731,-1.5939884682859,0.482449356645675,-3.30394
982671016,0.000953328922409236,0.00566017275552232,-8.24777591034065
"Ndufs2",15934.0989284476,0.650560183151357,0.33103250327576,1.9652456
3815844,0.0493858140992354,0.115249872075022,1.40563462084712
"Snx31",1564.47099714442,-1.37964233059695,0.543700984177646,-2.537501
99235647,0.0111646732620736,0.0369411970186858,-4.55065075572005
"Atraid",4041.97041255492,0.602224694269166,0.183321531629618,3.285073
4385413,0.00101955793368531,0.00596260965536775,3.08474365287722
"Cad",915.778689713282,-0.614244983314692,0.174518094914216,-3.5196635
8340449,0.0004320944868835,0.00307705562646256,-3.55265914237102
"Lypd8",6.28545583235673,-1.18097228442444,0.811116846979008,-1.455982
93121756,0.145397338172908,0.261793083359069,-1.58274005399827
"Sh3bp5l",1668.28710792264,-0.0376296279042443,0.147133197509572,-0.25
5752124885318,0.798142243427474,0.871098654483565,-0.00519289026043572
"1810065E05Rik",0.340746372225417,1.08565208692275,3.15975245357748,0.
343587702794114,0.731156374051315,NA,NA
"Atad1",3622.01674484288,0.0645695114131391,0.200464483949113,0.322099
506811041,0.747377311896472,0.835782134929573,0.0115829505737639
"Pten",6767.47829567976,-0.285791883064279,0.182280957037756,-1.567864
7276637,0.116912705046179,0.22211477374943,-0.429991330473666
"Pea15a",7879.52653559086,-0.934643918589764,0.165834270162264,-5.6360
1189112023,1.74033367564766e-08,1.2429679856329e-06,-12.7092959508525
"Timm23",5296.97140669292,0.338211334531749,0.298397097939806,1.133427
02347586,0.25703497109459,0.397909318491649,0.311672277566081
"Tnfaip8l2",382.459022958602,-1.24098108067678,0.410141260876475,-3.02
574063878577,0.00248024954394445,0.0117549600407946,-5.51427460781474
"Trnt1",1282.71758908729,-0.0909528259613176,0.137657068970687,-0.6607
20343978017,0.508791674634149,0.64318076193054,-0.0401401626128082
"Ly6g6e",38.2590086690415,-0.732602660838039,0.373490876485375,-1.9615
0082093565,0.0498206293305832,0.116043797716845,-1.57787052122717
"Ehmt2",7791.63253239609,0.187049907446428,0.110560741964371,1.6918293
430656,0.0906785170817916,0.1830468709039,0.317613180810861
"Elof1",2590.16428417814,0.40474734308872,0.145131333176455,2.78883501

053916,0.00528980059101786,0.0207818923884075,1.56785894770705
"Med16",1664.08369537304,0.17869977237763,0.128684281632313,1.38866822
047641,0.164933662085663,0.287671217760645,0.222648668298926
"St3gal1",1548.21192804647,-1.07006299781781,0.222604540742198,-4.8070
1334415756,1.53201768649858e-06,3.85435881952896e-05,-10.8758215441273
"Tmem259",8721.62947932127,0.5026105702197,0.180819496453076,2.7796259
8104088,0.0054421539461642,0.0211883222129212,1.93721447812303
"Rnf170",1196.37344611544,0.0597645823296455,0.110010286246071,0.54326
3583515856,0.586948333796296,0.710341916228675,0.0204400162353959
"Clip3",323.780547936241,-1.24669097036353,0.307800135684086,-4.050326
25340713,5.11462736451365e-05,0.000588068177193943,-9.27371981469974
"Thap8",6.62126614909461,-0.467702712805266,0.741161362604492,-0.63104
033265,0.528014153125235,0.660525341387034,-0.193965570266751
"Myl2",4.60731679512612,-0.748283649933364,0.717612201812276,-1.042740
97910212,0.297068273107878,0.441380567633727,-0.611982144745763
"Dedd",2196.34400944085,0.113513937378831,0.101121485719602,1.12255013
433636,0.261628603793409,0.403259645593983,0.103090479511976
"Mcemp1",155.742038614691,-1.51484197748264,0.501509749282649,-3.02056
336820858,0.00252304906877501,0.0119149011471998,-6.710697645129
"Nit1",5591.88492811231,1.14477200065963,0.22448127194072,5.0996325473
5094,3.40313488664898e-07,1.18278652441857e-05,12.9874982751405
"Pax5",136.016480314734,-1.72135966556253,0.897926825180538,-1.9170378
0006398,0.0552331296193639,0.125370845322277,-3.5743674917823
"Prdm15",576.292697503994,0.0470022167790151,0.245849055465015,0.19118
3231068804,0.848382040965873,0.903082002224851,0.00479149616668128
"Rnf168",1817.68315431408,0.262008976950857,0.20985067710678,1.2485495
9041918,0.211829859700688,0.345929290935308,0.278128001422969
"Tctex1d2",676.313032724942,0.514649146702583,0.209050057648597,2.4618
4647108628,0.013822382107687,0.0435042463818695,1.61337192590011
"Chp1",6566.41460377192,-0.494354742088964,0.381126451236954,-1.297088
51349605,0.194600733272265,0.325051409007336,-0.555541981435887
"Trpv4",4659.92239595912,0.704344212185094,0.167219718889066,4.2120882
445231,2.53020654895941e-05,0.000345234200781039,5.61453023429099
"Klhl3",1821.80161353928,1.66430066021971,0.427771566383071,3.89062946
444019,9.99845136698783e-05,0.000991102210236546,11.5114565441459
"Tvp23b",1798.08796521369,-0.0706361059766005,0.19920942453276,-0.3545
82149626081,0.72290265086743,0.817110486180472,-0.0142671484437144
"Dnajc7",7564.85065349899,-0.0290219077768388,0.103747705076174,-0.279
735419260891,0.779680502077835,0.857497366018682,-0.00446174601735583
"Zfp385c",17.3150135932834,-0.900155023627203,0.63801215320107,-1.4108
7441533973,0.158281647296348,0.279165586520506,-1.14855295824888
"Cacybp",2904.26767506201,-0.254707315603153,0.220109446771312,-1.1571
8484299217,0.247196848922041,0.386484176111134,-0.24214116546623
"Cluap1",1409.8169363914,0.493874707351709,0.133663742446693,3.6949040
8027946,0.000219969812188462,0.00182431790644433,3.11464509694155
"Zswim7",535.685297862578,0.531757272417244,0.325515575404052,1.633584
72711234,0.102346076886785,0.200819543840045,0.853655774745784
"Pigl",507.49871027283,-0.192634141041093,0.243033283823375,-0.7926245
24553149,0.427996615782771,0.570903493450632,-0.107978196901576
"Ndufa2",7519.94564397401,0.797647594804494,0.310370391791366,2.569986

10660221,0.0101702593667713,0.0342901119026988,2.6903841759084
"Pam16",412.428683908515,0.192766908576112,0.300087632628234,0.6423687
20389495,0.520633804286483,0.654079762627439,0.0818345595715096
"Glis2",5591.8475408016,0.733840050988324,0.36291600118616,2.022065846
06309,0.0431695498397698,0.104018769497755,1.66081500338425
"Cox6c",19868.8702935465,0.950881396920893,0.316815773317293,3.0013701
2423488,0.00268767662599232,0.0124594196038037,4.16987960092148
"Bicc1",16931.4360154342,0.139076042674792,0.367736915557606,0.3781944
01462004,0.705286184143761,0.804213269037301,0.0303033881105645
"Ube2z",3699.0181152177,-0.422719910439818,0.241424000620376,-1.750944
02111462,0.0799555530108261,0.166201852090095,-0.758592967616936
"Gip",2.54720992999642,2.27494895413671,1.07683001028982,2.11263517212
381,0.0346320058797795,0.0877523281133641,5.53548970847591
"Tmem87b",2934.56958320203,0.129225215787404,0.131564240427657,0.98222
1425573925,0.325990774441447,0.470897030675606,0.0973215553072991
"Anapc1",3130.21615405635,-0.139208420882393,0.138952277763324,-1.0018
4338913469,0.316419237690193,0.461683562224443,-0.107590785247014
"Mertk",3216.89496924526,0.305510969692939,0.260495467157625,1.1728072
3932166,0.240873114402137,0.379242892814346,0.296216837493926
"Tsg101",2793.09193200927,-0.0821749216168696,0.189206057963953,-0.434
314432112556,0.664060125061746,0.772267942171891,-0.0212359484272539
"Hps5",3993.08401589915,0.764847164796814,0.235074652428612,3.25363520
437017,0.00113938490925529,0.00649582659668409,3.85222568923274
"Map3k4",1913.74543704555,0.121813558520912,0.146397808709537,0.832072
280279811,0.405368147125306,0.549184834119596,0.0730053286873693
"Piezo1",5631.09540737001,-0.191661667129955,0.14760585573617,-1.29846
926583002,0.194126130835842,0.324564508578052,-0.215671309858097
"Blk",48.2310465599777,-1.48678981666786,0.615081734946803,-2.41722316
270121,0.0156394254497217,0.0476434965580068,-4.52580179381888
"Rnf166",1763.31009719451,-0.0891510536294858,0.17423238792677,-0.5116
78997747284,0.608875687270645,0.728826702093624,-0.0282001984566166
"Ankrd28",1671.86310912273,0.384139908790147,0.137801830734159,2.78762
558337278,0.00530958703539992,0.0208260567322928,1.48721699785266
"Ankrd52",1442.2900849292,-0.941027626169348,0.27623900242338,-3.40657
046222269,0.000657845674337759,0.00426418715366942,-5.13566175304078
"Pkd2l2",124.182997683599,-0.979114815426892,0.551873528997907,-1.7741
6520992549,0.0760358269772002,0.159809598618809,-1.79547351053798
"Srp19",1686.90917895931,0.280005655803837,0.184706785887983,1.5159467
7184004,0.129532812221219,0.239533106835844,0.400145901297454
"Tmbim7",0.177617859711189,0.0542912073317471,4.78471990596491,0.01134
67890281445,0.990946766486904,NA,NA
"Clec4f",0.994735386562561,0.100254903476089,1.5999307599472,0.0626620
263738145,0.950035636503603,NA,NA
"Klra17",7.85925785029349,0.482499681951345,0.693567802373817,0.695677
740950393,0.486630668375187,0.623575497632716,0.227877571874622
"Wdfy2",1505.43584957023,-0.0956387898534868,0.127434947151847,-0.7504
91070079288,0.452959000057994,0.593533876105432,-0.0498910256094328
"Rbsn",1446.60244525571,0.0551505243385019,0.121092407117925,0.4554416
38754393,0.648791545077809,0.760544552752186,0.0150958338124654
"Mrps25",1781.66981434567,0.449055980964406,0.166212638875232,2.701695

7555285,0.00689868569299121,0.02549480167588,1.64771244572017
"Dguok",1158.78011265016,-0.203817591138063,0.118537468474733,-1.71943
600416528,0.0855350110743068,0.175015737269115,-0.355229477252813
"Camta1",1233.55199396779,0.615294497497045,0.19949597505811,3.0842451
69948,0.0020406934993855,0.010140302595446,2.82496313322947
"Csf1",2355.21589890273,-1.06371351284624,0.329225161198779,-3.2309605
6502192,0.00123374960313945,0.0069073230774127,-5.29215887075898
"Strip1",2595.17936405725,0.338656092366405,0.155063926036202,2.183977
28616351,0.0289639171523317,0.0765539455925395,0.87026474971357
"Kif1a",358.667244435533,-1.27521492066222,0.418375895259564,-3.048012
4096802,0.00230360414155908,0.0111397999881248,-5.73493607162967
"Alx3",0.363908822403663,-2.30232936345835,3.70917969742878,-0.6207111
95268953,0.534789662041733,NA,NA
"Slc25a11",5033.20865479598,0.599996489410645,0.193291323392829,3.1041
0461721172,0.00190855831704213,0.0096282199393758,2.78581784949268
"Chrne",19.0364650638977,2.40190302391836,0.564435117825111,4.25541031
743987,2.08665992334376e-05,0.000296975877265377,19.5079192382571
"Cmc2",618.90984290226,0.566487138369716,0.331829765751749,1.707161915
04508,0.0877919616479144,0.17858817613052,0.975871993774292
"Chfr",1898.10291926253,-0.115647737698809,0.115760524837375,-0.999025
685666813,0.317782248653106,0.462845916503941,-0.089090515420714
"Ceacam16",12.4465401581146,-0.374731458367823,0.877510210554016,-0.42
7039427987095,0.669350610052,0.776313695387516,-0.094881478330534
"Hoxa2",235.275203095267,0.170419272407399,0.303648891134184,0.5612379
21109647,0.574635354959472,0.700352553506675,0.0606984747361213
"Adam28",232.708527535004,-2.23196048177185,0.640719659046318,-3.48352
114728933,0.00049486402394011,0.00343375755828621,-12.6643672360771
"Ankrd53",5.4672142011239,1.82801063275292,0.946308011175221,1.9317290
0489631,0.0533929628163218,0.122218359825387,3.84237963648513
"Tex261",4251.40586415459,0.163884541976554,0.108108964442352,1.515920
00554166,0.129539581030574,0.239533106835844,0.234201439859023
"Fam120b",2122.59389042537,0.161863505463167,0.119399470066183,1.35564
676604884,0.175211584550335,0.301444937400727,0.194101521283961
"Tbp",1101.99263633275,0.160853428543764,0.141131576677807,1.139740888
1145,0.254394267117996,0.395076019137131,0.149380892181006
"Psmb1",6440.01077441869,0.265290948476951,0.212014198178282,1.2512885
9650177,0.210829204422046,0.344687894333478,0.282565614114628
"Pdcd2",986.313844382736,0.672502957766843,0.167726554989154,4.0095198
8676051,6.08423290818052e-05,0.00067327113078152,4.91153284290096
"Dll1",800.454537944964,0.643743038812688,0.153236690799367,4.20097194
382475,2.65771528949906e-05,0.000355175686872191,5.11318529441136
"Nol3",676.769186952011,0.0207158795090698,0.450410967579642,0.0459932
839122236,0.96331560290811,0.978046740493088,0.000459847326658771
"Fhod1",2655.57827102024,-0.442757855101993,0.195730710501497,-2.26207
65743279,0.0236926726504797,0.0655787329485482,-1.20629547130234
"Plekhg4",22.1517204012992,-0.0981822268482989,0.542113897539883,-0.18
1109961013452,0.856281266134402,0.908613449093255,-0.00940934513498279
"Slc9a5",391.828126798362,-0.685937135425043,0.307959939143167,-2.2273
5832892264,0.0259233350884849,0.0703996128093726,-1.8201805003763
"Elmo3",1680.39373480128,0.922059338403118,0.190911797898405,4.8297661

4621689,1.36693494512775e-06,3.50229928516438e-05,9.45987310589896
"Stc1",616.490626554806,0.0797577131037501,0.255257474791394,0.3124598
5320873,0.754691069240529,0.840663075185777,0.013843093504114
"4931428F04Rik",375.374703076466,-0.263507871618348,0.173536253905661,
-1.51846006634209,0.128898459206733,0.238709236470596,-0.3774774112333
35
"Tppp3",586.002816792848,-0.823152550271278,0.248192520956094,-3.31658
88605358,0.000911236043078407,0.00548477384594356,-4.28515060364305
"Msh3",1189.09183548856,0.911444828911098,0.21882141136159,4.165245179
80092,3.11018137996436e-05,0.000401990692825569,7.12666151101291
"Adamts13",412.787097631842,0.858262901337324,0.249386465090456,3.4414
9752082985,0.00057850377263659,0.00385497831813678,4.77055983918919
"Tmem208",1882.01278801741,0.227615811439555,0.191921794240296,1.18598
209411573,0.235629370868634,0.373547375204309,0.224135666108972
"E2f4",1744.29609281411,0.0449770791936048,0.124706134067252,0.3606645
29696264,0.718350243636584,0.813426624683554,0.00928774676946153
"Surf4",9371.96410435958,0.257517019579609,0.163571189809575,1.5743421
5572682,0.115408368858116,0.219770717452441,0.390182183457827
"Surf2",1620.63281727912,0.43986272469771,0.143558458864496,3.06399726
060653,0.00218400909945144,0.0106994496032925,1.99590481489461
"Dnajb9",2394.70499850118,-0.65047615398205,0.301509497833684,-2.15739
855180569,0.0309746239749032,0.0805850942630228,-1.63818619381561
"Naf1",621.773595808331,0.0472914245456284,0.156214936510136,0.3027330
52306812,0.762093306926066,0.84576784668703,0.00792180402032109
"Yes1",1502.25868494379,-0.0983621306060117,0.14391038325926,-0.683495
717114508,0.494293655600873,0.630247037464415,-0.0454082297318251
"Ppp1cb",8843.27835963325,-0.152817324028337,0.223364084443915,-0.6841
6247136951,0.493872577189272,0.629916105843948,-0.0706273738657599
"Gorasp2",3489.06865553785,0.128024334060116,0.101555292100518,1.26063
675670786,0.207439749285042,0.340516941535591,0.137919386017783
"Tsen15",831.021194565032,0.359988462789364,0.12991995982869,2.7708480
1491655,0.00559105171831905,0.0215827215212403,1.38086614188196
"Oc90",2.01982942014078,2.25345478893168,1.51234553424572,1.4900396357
2094,0.136213814549188,0.248842589345083,3.13440858187727
"Efr3a",4169.51582896111,0.314547990953814,0.158358477143624,1.9863034
5926182,0.0469996387755931,0.110984492775404,0.691491228756113
"Trappc2l",1537.27442270569,0.220174463934665,0.226466845320336,0.9722
14999609455,0.330943599957099,0.475725325270092,0.163570832403977
"Acsf3",2197.36397475755,0.418709583339402,0.409162230589488,1.0233339
05322,0.306149998086416,0.45065020856779,0.333738264641736
"Ddx19a",1173.20579903141,-0.281445553307193,0.132826469928095,-2.1188
9658333579,0.0340992077689101,0.0867858442864128,-0.68794067430563
"Galns",3464.51735990197,1.45388720639934,0.366461189694829,3.96737020
804322,7.26700645257522e-05,0.000773006501976605,10.417426193264
"Gata2",1773.89720843522,0.104988611980293,0.307756081417766,0.3411422
82214647,0.732996471732148,0.825266497622903,0.0201629492688716
"C8g",447.796202109386,1.72774562805374,0.397738364001899,4.3439250130
9098,1.39959306534647e-05,0.000220965713264174,14.5433040282565
"Entpd2",764.866858472309,-0.720394706619097,0.259759367372662,-2.7733
1560322746,0.00554882774594661,0.0214537067996167,-2.7676540496225

"Rabl6",5622.9366429292,0.510992450745539,0.213884576850742,2.38910377
863352,0.0168895307498558,0.0504770187861264,1.52594462436426
"Ptgds",705.008177797218,-0.661539165185763,0.413787818161366,-1.59874
006954884,0.109878370314586,0.211595897717906,-1.02742123965766
"Edf1",9292.21839658996,0.672828954252386,0.191178445005858,3.51937664
432707,0.000432562138200391,0.00307926125251231,3.89101395569578
"Clic3",563.737252606899,-0.300725716457002,0.53490518221144,-0.562203
782011836,0.573977184935224,0.699881038727666,-0.107312439135463
"Npdc1",3435.41058146075,-0.175375070145975,0.159703643746997,-1.09812
817059957,0.272148525840571,0.414753162172851,-0.154342640620075
"Fbxw5",3030.77083261661,-0.00969331780632922,0.106631155858728,-0.090
9051180048311,0.92756798345394,0.955515781951002,-0.000441084718751439
"Slc25a13",1983.68117467644,1.77712241174046,0.434799692806308,4.08722
094597274,4.36571308168811e-05,0.000521585010212958,13.4326255000296
"Ube2i",4449.07884422729,-0.296303276687964,0.087506609387798,-3.38606
739263378,0.000709019691463341,0.00451337445124134,-1.60024812570597
"Tsr3",1142.25599468117,0.463432154464843,0.147234854258745,3.14757097
969768,0.00164633129829875,0.00865000475727,2.20139329208861
"Unkl",2249.91890537182,0.282087744833227,0.152385216383873,1.85114902
565497,0.0641481167411691,0.140305906795297,0.554000638753193
"Lrrk1",2538.18176978375,-0.528164577452854,0.232871671391225,-2.26804
992765967,0.0233261637637834,0.0648715485999973,-1.44471293805246
"Aldh1a3",584.475577852637,-2.78114380881417,0.865822583622,-3.2121405
255795,0.00131749913649982,0.00726076827684386,-13.6978831451333
"Actn1",6046.06163064227,-0.890674660040863,0.308664470334199,-2.88557
558657952,0.0039069863092322,0.0165066996874127,-3.65531895290106
"Sirt2",3691.96112136209,0.159404504317426,0.149930158460043,1.0631917
284334,0.287695012730469,0.431697503326049,0.133904591233252
"Hnrnpl",12938.1520086495,0.00585741725510665,0.108319710714863,0.0540
752667861672,0.956875197653751,0.973609630735731,0.0001566557193783
"Nolc1",2017.44880931629,-0.0733419300645932,0.130347696540762,-0.5626
63798524878,0.573663839833725,0.699642597594282,-0.0261966849030995
"Casd1",1684.11101389692,-0.167834037985262,0.0992800694182323,-1.6905
1088469968,0.0909302576418166,0.183460074865495,-0.284605947969491
"Cnksr3",974.019572497565,-0.181496000038044,0.158502407155929,-1.1450
6778347848,0.252181079671308,0.392357699603508,-0.169804273874488
"Mtmr1",1466.29423177254,-0.0911983656884885,0.148821287046015,-0.6128
04575868841,0.540005564876179,0.670766930238628,-0.036418567054966
"Hmgb3",2627.67509044198,-0.124996023507566,0.124474810888314,-1.00418
729392343,0.315288345338532,0.460555300974729,-0.0969122096263373
"Map2",323.108533137625,0.840514010671468,0.264279355071715,3.18039981
005473,0.00147071985966006,0.00792376231656586,4.06631360927257
"Cyp2j9",658.654571595127,1.5263749981544,0.299572114982141,5.09518383
66044,3.48403005485873e-07,1.20446131222758e-05,17.289086345736
"Abca1",3363.36288109914,-1.43346852618172,0.263419857927283,-5.441763
34108199,5.27557320539508e-08,2.84152282759097e-06,-18.30707087197
"Nipsnap3b",1014.60319694416,-0.375871099652435,0.215150624616906,-1.7
4701375058384,0.0806349529327467,0.167293074301202,-0.672060558981296
"Lage3",1613.98502274974,0.0427898492791767,0.251024877640078,0.170460
592119197,0.864647923685158,0.914852681572238,0.00380796412648836

"Ubl4a",2136.7034273481,0.273813590023102,0.128204613833713,2.13575457
103479,0.0326994237824946,0.0840239015433684,0.678141517079003
"Gdi1",9205.78333039555,-0.217430362461169,0.112985453910635,-1.924410
22216138,0.0543031841511971,0.123749822882193,-0.454319287490261
"Sash1",3896.90048139577,-0.757904543166568,0.145097793131969,-5.22340
503468059,1.75662572455548e-07,7.004230752021e-06,-8.99556614154488
"Gadd45b",1057.11558808621,-0.693707876879138,0.328756924617687,-2.110
09358262447,0.0348502959471158,0.0881223019552411,-1.68503699057634
"Slamf6",166.598256695521,-1.65951731973769,0.648959122486186,-2.55719
853876161,0.0105518973716514,0.0353749819381806,-5.54569272280068
"Slamf1",79.1531146294252,-0.545865878810587,0.47609601008883,-1.14654
57958968,0.251569389694837,0.391562222421165,-0.511809766683241
"Zdhhc12",732.352925835911,0.434131166393046,0.144917102769184,2.99572
07127202,0.00273797076869256,0.0126384834393863,1.89759118274753
"Endog",1553.12551297856,0.985071627308923,0.325844703424746,3.0231322
3739856,0.00250172897732405,0.0118337485686231,4.37056558078475
"Cybb",2208.00582819724,-1.08099338835217,0.426747760996661,-2.5330968
0132247,0.0113059725747261,0.0373190880755054,-3.55457687524893
"Golga7",4106.14191962926,-0.298202803178031,0.0870749505956102,-3.424
66807202604,0.000615550970720481,0.00404244285183831,-1.64336764809955
"Xk",101.40656335022,-1.6561861949659,0.414721897876946,-3.99348624570
897,6.51088571917787e-05,0.00071040430751276,-12.0068138106711
"Pcolce2",229.749032492615,-0.610292082524431,0.326923408056929,-1.866
77389102146,0.0619331700085334,0.136727009604437,-1.2143402494863
"Cd48",624.579440452189,-1.21523662458076,0.406345482496425,-2.9906487
8761498,0.00278385472103319,0.0127867136279635,-5.29764013267765
"Clpx",5052.72253866277,0.132295065930441,0.283986728607432,0.46584946
6202764,0.641323254822597,0.754830425119582,0.0372095956636718
"Trabd",3582.49783477543,0.147419795615948,0.129109798258558,1.1418172
5635355,0.253529987919209,0.39392979750482,0.137333711255277
"Mov10l1",0.126703529248624,-0.545046838331149,4.78471990596623,-0.113
914053286904,0.909305925174374,NA,NA
"Dennd6b",1628.26090472482,0.52142732489074,0.254511997982265,2.048733
76903463,0.0404881552187402,0.098827998304331,1.20677801606667
"Cd83",863.877099016092,-1.28909045562039,0.429410667494401,-3.0019991
4255088,0.00268212931205276,0.0124415604964143,-5.65486954750485
"Cltrn",37523.0711484794,2.03699583475447,0.916674406117699,2.22215851
250997,0.0262725951058647,0.0712069862905408,5.38207776207901
"Ace2",1601.58475543186,1.70343797469563,0.41503595062446,4.1043142699
6301,4.05515902476756e-05,0.00049386315218562,12.968702736403
"Gzmb",28.4383692879208,-2.00783555352541,0.768785172476243,-2.6116991
1362652,0.00900935073042146,0.0311959368747572,-6.96210436861567
"Ager",526.774647158077,-0.146194466993186,0.345559500989264,-0.423065
974382596,0.672247126676551,0.778475373929642,-0.0366097144752647
"Atf6b",2123.55811419072,-0.196100186281461,0.0811674009958639,-2.4159
9686420235,0.0156921964885252,0.0477401228168614,-0.596533449722805
"Egfl8",409.593734134716,0.0441155034641803,0.247896988640087,0.177959
013161834,0.858755164627908,0.910643835313807,0.0041293619245172
"Notch4",1586.02227935916,-0.0714824698969621,0.225139803791852,-0.317
502585917902,0.750862277626501,0.83797794569066,-0.0126354913273039

"Ppt2",4828.03526225677,1.01817889947159,0.201089982109357,5.063299965
47456,4.12060716992358e-07,1.36175590846051e-05,11.4078296142257
"Prrt1",412.384012495814,-0.868812317388889,0.278799925473161,-3.11625
735162732,0.00183162349324985,0.00933185128596433,-4.06110840816677
"Rnf5",4584.1550444859,0.804790385834089,0.16641395628495З,4.836075073
2711,1.32427923723433e-06,3.42450882859351e-05,8.27482852003328
"Fam163a",260.218896201987,1.14451807071172,0.645987928191691,1.771732
9082535,0.0764389042432687,0.160431189982266,2.09434234545654
"Cacfd1",2362.46136264752,0.212257626351471,0.116783581966405,1.817529
67992136,0.0691360479789024,0.14874714669126,0.4044584812528
"Hivep2",1603.66429823566,-0.569627564713686,0.188501883037836,-3.0218
6670782143,0.0025122114397007,0.0118746905821295,-2.52535607733481
"2310057J18Rik",0.222265746865732,3.19052280584978,4.69750997731396,0.
679194471381224,0.497014649864957,NA,NA
"Arnt",2421.34310201524,-0.362528480143661,0.131113231545949,-2.765003
01204621,0.00569222701981948,0.0218952539815427,-1.38539728627373
"Itga2",338.747739917435,-1.57288011535515,0.375241529955128,-4.191647
2186414,2.76936392177623e-05,0.00036657880344055,-12.4435218064542
"Mocs2",4575.56323993436,0.556371143820242,0.252186648247128,2.2061879
4725021,0.027370842829857,0.0734282737337029,1.45293331866649
"Nat9",1328.0752655073,0.550481541451102,0.206102816976711,2.670907411
77644,0.00756465094275814,0.0273139933484997,1.98192959290109
"Lpl",68580.8910637485,-0.337731056562513,0.57539280462055,-0.58695738
6068174,0.557232327062496,0.686379430794964,-0.127096537882303
"Atp6v0e",5778.1015203784,0.0791463369427525,0.28924424317547,0.273631
502822127,0.784367821884372,0.860690179025352,0.0118735871168785
"Nkx2-5",0.182624878335611,-0.546804771982973,4.78471990596623,-0.1142
81459046567,0.909014679736066,NA,NA
"Zfp318",2231.7707874425,0.671915493437193,0.3081968893601,2.180150146
33104,0.0292463336533231,0.0771122398340087,1.72177892208137
"Ttbk1",332.921759504543,1.58607035869014,0.548106846782341,2.89372476
917805,0.0038070170414561,0.0161754670460473,6.54136584671782
"Srf",1256.30817089929,-0.670394097935763,0.147388400826464,-4.5484861
3714922,5.40332020017441e-06,0.000105248688574426,-6.1402632612612
"Gata3",2777.72085886163,-0.623937886774808,0.365243965866269,-1.70827
705611777,0.0875849464885124,0.178260050796766,-1.07598827024817
"Gata5",47.537597707678,-0.0896170235348902,0.297282288716519,-0.30145
4297603134,0.763068095194481,0.846396995439143,-0.0149451413454839
"Lama5",11944.0527964655,0.0389557768543096,0.236619600997608,0.164634
614757479,0.869231584328072,0.918012909442399,0.00333242619252193
"Steap1",286.339628611824,0.14988393490169,0.384302797213899,0.3900152
06728424,0.696525302264414,0.797506851713595,0.033913466478054
"Steap2",3681.9540332008,0.982094445274097,0.375110825381342,2.6181447
6901776,0.00884092885858315,0.0307492741499562,3.41954373782029
"Hspa8",95996.5806957782,0.572217188626038,0.184164047874166,3.1071058
397729,0.00188928744029936,0.0095582061522981,2.66101318466396
"Serac1",143.46088167923,-0.672894536380415,0.354729225997155,-1.89692
443437353,0.0578379120492011,0.130041929097708,-1.3726364538188
"Awat1",0.405013008858982,-0.0576317831671303,3.78970840355944,-0.0152
074452781117,0.987866681876593,NA,NA

"Pdzd11",1398.0207036926,0.353737921396356,0.136774312563823,2.5862891
5595019,0.0097015505085154,0.0330181238985919,1.20649345422448
"Psma2",6118.26073497186,0.416821385135493,0.151260132442033,2.7556592
6332392,0.00585739806011454,0.0224068172211327,1.58325019200307
"Mrpl32",1388.30271999318,0.335980361361383,0.261005809887346,1.287252
42363914,0.198006320369882,0.32904860091809,0.373458908841272
"Setdb1",2295.24509921204,0.0946686035691503,0.12240246625921 8,0.77342
0719878845,0.439273431465923,0.58122647711201,0.0513685846709763
"Anxa9",433.529358675637,0.106229959698982,0.283251537668772,0.3750375
39330872,0.707632548468254,0.80620658116361,0.0228835549346438
"Arnt2",1511.47856169417,0.536336735124568,0.433984667358697,1.2358425
8952926,0.21651708051839,0.351862813464541,0.560211182333619
"Prune1",1301.36957463992,0.022448690459205,0.182785607409182,0.122814
322075981,0.902254132744902,0.940153435242631,0.00138535780725108
"Cers2",8205.32849935825,0.339535203111151,0.122820596964509,2.7644809
7064099,0.00570134325116046,0.0219216704222297,1.29711941482081
"Nlrp5",21.8134415200509,0.0280469436976451,0.53416485763646,0.0525061
566605963,0.958125389797813,0.974604489440235,0.00072146673808861 2
"Capza2",5215.66567684694,-0.457817187876433,0.191428961026116,-2.3915
7745736276,0.0167761436757504,0.0502266721768623,-1.36942692712183
"Plekho1",2920.27699720393,-0.612692714638042,0.307514428644551,-1.992
40314458948,0.0463268426059212,0.109621453535225,-1.3544933735842
"Vps45",728.455914618374,0.519931142394002,0.207755299973791,2.5026131
341034,0.0123280217338479,0.0397429311221036,1.67694602125304
"Prpf3",1386.68633703115,0.370644955778953,0.166349726748694,2.2281067
9057436,0.0258733945768394,0.0703155716575755,0.983974144738925
"Anp32e",4229.11885693712,0.00651573687256652,0.143872839913751,0.0452
881647187376,0.963877620946479,0.978277333465954,0.000143099115527323
"Aph1a",5692.27271266961,0.259855141149012,0.198190945717815,1.3111352
8021101,0.189812063179308,0.319295626595648,0.296660471462482
"Tab2",4428.98813493974,0.0092028235281 0705,0.0953768775775139,0.09648
90418081448,0.923132176688388,0.95268858636282,0.000446035094453892
"Ppil4",1474.65042015955,-0.152271118250669,0.140392537492236,-1.08460
977321598,0.278094524581209,0.421705322778381,-0.131478268055632
"Cnih1",2971.35448100602,-0.0796172979682662,0.38825314114838,-0.20506
5431622197,0.837521004038924,0.896266988844728,-0.00871944218951498
"Eps8",4241.20393237156,0.746322377142265,0.230595569981208,3.23649919
728764,0.00121005566765189,0.00680128723201974,3.72462883522556
"Med22",1876.7674131143,-0.231081590469952,0.236234455233767,-0.978187
496998623,0.327981599114602,0.472637279873301,-0.173178791150239
"Abo",0.873368776491107,-1.02439714539426,2.14612016315716,-0.47732515
7733604,0.63313059915716,NA,NA
"Surf1",1783.18847058731,0.0121066781728307,0.176039159096082,0.068772
6426040405,0.945170594553977,0.966180992587472,0.000416519359175568
"Med28",3703.716286495,0.430954427017714,0.27230348949607,1.5826254294
987,0.113506863924073,0.21714647103528,0.658146348586086
"Qdpr",10340.6863536361,1.19857544077575,0.378326347404766,3.168099311
60679,0.00153439096092827,0.00820108414368955,5.7573438505167
"Gnrh1",88.142252307156,0.467500289888776,0.319976734384523,1.46104463
1223,0.144003193594047,0.259786664387188,0.630141072465798

"Tnr",1.58332643734244,-1.21076332224796,1.91877085780555,-0.631009855
774382,0.528034080223597,0.660525341387034,-0.50212750926607
"Sqstm1",16259.8037763102,0.562559693658393,0.154298219618906,3.645924
72322645,0.000266431967058159,0.00211346236521562,3.4650457853209
"Nfe2l2",5040.57369322163,-0.561180282250614,0.23333215831696,-2.40507
046391909,0.0161693477204804,0.0489245719822976,-1.69334776073403
"Rxrg",117.819177922047,-3.61940754560257,0.849295346214086,-4.2616594
5891126,2.02914488865487e-05,0.000291556868378723,-29.4629745043665
"Rxra",4914.49352611731,-0.256332345201233,0.335255503391988,-0.764588
030942847,0.444516898594778,0.585700127638936,-0.137124308262016
"Adamtsl4",735.722408138731,-0.83958262070046,0.314191675166363,-2.672
19881066519,0.00753559879596593,0.0272494631535133,-3.02478232407964
"Fcrls",908.686238825219,-1.57801547641101,0.356129764095856,-4.431012
61254385,9.37916025081052e-06,0.000162700059226249,-13.765979249648
"Cd5l",24.4236609202477,-2.28902014861795,0.622018129965544,-3.6799894
3816114,0.000233243613989218,0.00190842802583373,-14.3326433481961
"Prpsap1",3222.64451689424,0.171409727970952,0.110274401684048,1.55439
272717221,0.120090766983497,0.226606965512845,0.254464287587385
"Fam184b",75.6575110656225,-0.0102708088708309,0.473015317148889,-0.02
17134805120866,0.982676510414864,0.988843275184532,-0.0001152325993941
2
"Ncapg",131.232227886142,0.0978737272429322,0.580822271336983,0.168508
908960462,0.866182935551408,0.915880416343891,0.00860011280140619
"Lcorl",692.326434015501,-0.182434788493335,0.180322880339263,-1.01171
181466322,0.311675864849184,0.456784126039491,-0.142945751707172
"Lta4h",2092.79496389442,-0.0180801871368884,0.130657472345294,-0.1383
78516072216,0.889941275348876,0.931963645610656,-0.00127395659812498
"Amdhd1",94.9780216886828,0.852419706252143,0.350064293797406,2.435037
56697182,0.0148902436938957,0.0459746589934408,2.62516707135127
"Dstn",14662.1840297756,-0.28334294052925,0.256019764093586,-1.1067229
1856997,0.268413743336444,0.410666287474418,-0.252167948450482
"Macroh2a1",6378.48937283663,-0.0843520092086039,0.131457921759357,-0.
641665470438643,0.521090413557031,0.654569055761821,-0.035746541786341
6
"Gtf2ird2",1007.40598337625,0.370863504490181,0.256672365573078,1.4448
9066309163,0.148488599348165,0.265944997826071,0.491196015804997
"Bola1",1093.08751667888,0.668407221597984,0.213073303595849,3.1369824
8592324,0.00170696323521339,0.00888714797334867,3.15698696299624
"Castor2",4617.26769547363,1.10054998999331,0.241126600103289,4.564199
84158481,5.0140300307894e-06,9.9630809135096e-05,10.1405106531043
"Fcgr1",653.517018419202,-1.41973445930777,0.488997162371117,-2.903359
30053983,0.00369182815747178,0.0158140610840868,-5.8874340666507
"Ncf1",1245.84546620948,-1.33250048904302,0.369805341886018,-3.6032483
5289622,0.000314264994082864,0.00239544130360738,-8.04055820075918
"Wnt11",144.749839500136,-0.404968546493327,0.337896965932681,-1.19849
713765707,0.230723536664282,0.367536561918314,-0.405346170374885
"Adss",3983.653445375,0.319319068411099,0.0858620727083746,3.718976939
86082,0.000200031310388993,0.00169500673184033,2.03727755107619
"1700016C15Rik",711.625193002531,1.39190206092173,0.598704030087353,2.
32485834564809,0.0200795359385336,0.0576259628686383,3.9721851426888

"Il17rb",587.188397501883,0.173448093487693,0.358731333389726,0.483504
164101713,0.628737803812784,0.744876911680844,0.0510867585658441
"Cacna1d",836.530427574094,0.403148507139311,0.297245118732418,1.35628
301941008,0.175009136134581,0.30122953181074,0.483730935648294
"Chdh",9164.37931360743,1.0185308951359,0.212432500330575,4.7946095515
0893,1.62991974200147e-06,4.00758828871937e-05,10.3123562467623
"Actr8",1630.57953105881,-0.0141744105710006,0.161695866222448,-0.0876
609334681481,0.930146170620011,0.956640470236786,-0.000628318201594055
"Lrrc27",186.190478678023,-2.47313364564636,0.583719380297724,-4.23685
3750487,2.26673674526125e-05,0.000316596225351747,-19.9282223836349
"Stk32c",222.363247891462,-0.0779602878068116,0.306835627665591,-0.254
078342857165,0.799435034370926,0.872022897947082,-0.0106758503400315
"Fnta",3575.96204213766,-0.096155262436886,0.167947541021695,-0.572531
528904403,0.566961932122192,0.694124309463676,-0.0351066915380977
"Mtrex",2090.08803757168,0.373734899130506,0.11969036552771,3.12251447
710703,0.00179313286389347,0.00918515849937256,1.75287883058155
"Lbp",1004.91194732674,-1.57211826233944,0.270575091653398,-5.81028450
450754,6.23667642306229e-09,5.60381183856537e-07,-22.6300899429505
"Celsr1",1140.62658195676,0.361289329240529,0.489827893539936,0.737584
229084074,0.460767141916482,0.600136121114734,0.18447389108848
"Fli1",968.901832492065,-1.16678836154558,0.219745432729818,-5.3097274
7442798,1.09789277420402e-07,4.88261201992845e-06,-14.2696235775697
"Stard13",923.458437955333,-0.992185668276383,0.192764010339556,-5.147
15203594611,2.64470970451903e-07,9.60270946344729e-06,-11.463182653409
2
"Tenm1",2.90634745671459,0.460342088442205,1.28684508878742,0.35772921
8888327,0.720545964671237,0.815250114266801,0.0940296242055757
"Camk1g",69.4896835953416,-0.265790523644767,0.449757345904446,-0.5909
6427454736,0.554544358356745,0.684119322950953,-0.100900176771588
"Utp25",635.129165730767,-0.129482605233861,0.178131405878821,-0.72689
3747877035,0.467291047478838,0.605720040137108,-0.0649144700156378
"Hsd11b1",3816.64597337096,-0.265858741148856,0.3815651161343,-0.69675
8508330937,0.485953936335495,0.623024378237074,-0.125796282331527
"Syt14",361.372093145314,1.06318526111731,0.299891072961118,3.54523811
135638,0.000392258565044907,0.00285169929203788,6.23009575394223
"H2-
M3",663.635656102028,-1.06556950764897,0.241481573288384,-4.4126327865
8714,1.02121122214853e-05,0.000174204518017952,-9.2228030428296
"Lonrf3",110.411391195483,0.411113712701849,0.24657248980668,1.6673137
9086197,0.0954520258328928,0.190591254448514,0.681472028081963
"Atp5e",10049.0727919437,0.876767805907367,0.313157738592106,2.7997641
3755297,0.00511399580961869,0.0202194897788058,3.42036616409616
"Nelfcd",1862.90380976365,0.276909999639568,0.116554709607996,2.375794
17057354,0.0175112278934475,0.0519054304448827,0.819191674683564
"Tubb1",7.4643136014221,-1.58715022383416,0.880394734110956,-1.8027711
4610062,0.0714241634254256,0.15238301110285,-2.98599795753494
"Ctsz",10251.0509310308,-0.212449408863067,0.160509409599768,-1.323594
73125477,0.185637675933313,0.314116411121039,-0.246014636353712
"Prelid3b",2029.87052973411,-0.390105330158039,0.250202206923262,-1.55
916022866132,0.118958461431904,0.224971103879657,-0.581952621540458

"Sertad4",442.592316207483,-1.35510306316918,0.278298020497898,-4.8692
515338226,1.12021739998064e-06,3.01607735157191e-05,-14.105224920017
"H2-
M2",275.376695824805,-1.56326092675,0.641884415420041,-2.4354243368364
3,0.0148743350335846,0.0459400894162574,-4.81549574578795
"Ube2a",1718.63405861186,0.5368783818817,0.179677376314018,2.988013254
06383,0.00280797376886559,0.012870209813197З,2.33694567163323
"Slc25a5",21963.3649855369,0.986176534273313,0.327174264501695,3.01422
404288221,0.00257637471897644,0.0121069429068337,4.35295974830564
"Atp1b4",0.186468003080665,-0.0694338828847404,4.78471990596172,-0.014
5115877730327,0.988421834531527,NA,NA
"Ppdpf",3569.07955756787,0.210096384963729,0.248138957500245,0.8466884
32482522,0.397168807982895,0.541651914251275,0.128816755459483
"Kcnq2",2.60558910471425,-0.803503195071691,1.20680745159726,-0.665808
944093126,0.505533227263356,0.640402958209011,-0.358087368627768
"Eef1a2",4.76098170343518,0.249837902119928,1.72735908155899,0.1446357
64958866,0.884998457779544,0.928224848294005,0.0186082472730582
"Col20a1",345.2764986536,-0.65545079376416,0.248486699115227,-2.637770
13457053,0.00834531286939365,0.0294031279955615,-2.31154830513691
"Pls3",4397.54668592196,-0.635773596202278,0.124508052001269,-5.106284
98304509,3.28554071793239e-07,1.15634427650263e-05,-7.22725930057113
"Mpped2",447.029079740851,0.755184150100094,0.31472391762776,2.3995130
582776,0.016416894259645,0.0494434050470433,2.27078330573324
"Nkap",1645.57801231167,1.22920373376385,0.332166472425263,3.700565336
37787,0.000215119681470544,0.00179248014927891,7.77367501249766
"Ndufa1",5712.9220453002,0.650951828047649,0.441869409399715,1.4731769
4821186,0.140703329123035,0.255226859358167,0.888941433854627
"Wt1",1431.76267029623,-0.563312510564342,0.407694364712551,-1.3817029
6016113,0.167062925118815,0.290371983773102,-0.696588011803964
"E2f3",536.741206116856,-0.520171030630828,0.104532087199708,-4.976185
25149168,6.4849587066013e-07,1.93635106985164e-05,-5.64495852546347
"Cr1l",2939.55156812516,-0.119126456949576,0.19379408500673,-0.6147063
61886326,0.538748657652089,0.669812533151446,-0.047740809994032
"Ppfibp1",4114.99760122935,-0.626621302410933,0.209349904389598,-2.993
17692185231,0.002760896493881,0.0127172123405839,-2.73507597192016
"Cd46",404.359193652326,-0.169992729481413,0.208929729822041,-0.813635
903450442,0.415853563346037,0.559155422255088,-0.0988215008457307
"Cd34",2623.84754879209,-0.989444824063823,0.189090424049234,-5.232654
31889982,1.67093049366995e-07,6.70236711058241e-06,-11.7873054573654
"Plgrkt",2395.9137675695,-0.294818729656719,0.129137797127994,-2.28297
784392676,0.0224316734885428,0.0629132292404499,-0.815468257047923
"Cd274",579.50230129712,-1.31748616065064,0.401167432607734,-3.2841303
9933601,0.00102297578466126,0.00597721961322303,-6.7452653380124
"Pdcd1lg2",24.3805667588294,-1.59866124785658,0.706138878222634,-2.263
9473581747,0.0235773526980723,0.0653184459941765,-4.36191652339272
"Gtf3a",1974.81153989985,0.519139620579353,0.117017826274125,4.4364148
3617395,9.14694985237894e-06,0.000159431318768499,4.53930354321957
"Mtif3",1784.7107908931,0.488501738915754,0.202642520405208,2.41065763
463135,0.0159237883993095,0.0483014975435185,1.4803032600967
"Lnx2",869.491970355238,-0.586270555091344,0.230040917363637,-2.548549

02254019,0.0108172082451226,0.0360413408003078,-1.94822902382882
"Il19",1.37161199061384,-0.156110194282093,1.56597513138542,-0.0996888
080489392,0.920591386161556,0.951226658634901,-0.0078059636324403 2
"Dyrk3",99.7037371843668,-0.273274720865042,0.453637202341652,-0.60240
8090549919,0.54690252585862,0.676747188499801,-0.10670216566425
"Mapkapk2",6399.5762185999,-0.330580161013411,0.131074125450426,-2.522
08557468836,0.0116661324022265,0.0382103560521773,-1.07922809265639
"Il10",6.78760060112954,-2.11179877964243,1.1686484946694,-1.807043597
17661,0.0707554818215394,0.151353854586547,-3.98736071535039
"Lamp2",26134.556024499,0.691737773213862,0.240198408692298,2.87985993
321047,0.00397851879201018,0.0167206020153349,2.82997785747174
"Atxn10",3141.59683502777,-0.573698725232219,0.286741863949771,-2.0007
4979401234,0.0454193603732564,0.10793256411429,-1.27719601440794
"Foxred2",614.026314154056,0.0460381780034174,0.225028439910985,0.2045
88264583929,0.837893824972694,0.896388779904753,0.00503570443303602
"Eif3d",4034.07317810174,-0.0706534016893863,0.13606090367491,-0.51927
7762980308,0.603567057976681,0.724786357979485,-0.0227418000893771
"H3f3b",19656.115165641,-0.31567530517949,0.176458207671314,-1.7889522
3603027,0.0736225081829293,0.156133528883049,-0.586222831512338
"Nup50",1973.70714022213,0.0257041053897295,0.112972846896987,0.227524
631765432,0.820015818049738,0.884573633213488,0.00315259619946881
"Phf21b",291.409968692149,0.424027476079095,0.261121729277513,1.623868
97962233,0.104403774281567,0.20389270742608,0.674272111213052
"Ift27",657.180572132442,0.182325403525137,0.154519957277942,1.1799472
8148403,0.238021183099647,0.376210848579516,0.178242321882321
"Pacsin2",5353.7330497585,0.475333998725729,0.169613670667168,2.802450
9867396,0.00507159220389329,0.0201151148884279,1.85678948075687
"Cmah",1609.9180327649,1.55902691784396,0.305948399762122,5.0957184906
2168,3.47421067590567e-07,1.20319999571596e-05,17.6605646220834
"Ttll12",969.353469330566,-0.122304836825647,0.133844660389231,-0.9137
81965376688,0.360831424656258,0.505973439875808,-0.0833227508952019
"Bik",880.883254346211,0.414718878393878,0.246436900389434,1.682860309
22526,0.0924021367150974,0.185718674535715,0.698188460412307
"Scube1",492.256463881249,-1.25124553134729,0.531499522553578,-2.35417
997242162,0.0185636208194573,0.0542503687061573,-3.6463115222558
"Tox4",2217.96925735692,-0.268294080137142,0.139572916585568,-1.922250
29540498,0.054574270966404,0.124178916478346,-0.559670003977677
"Mrps18c",1742.68725494179,0.138266638415503,0.306437391834166,0.45120
6811244264,0.651840501040976,0.762880317484811,0.037422435044178
"Sulf1",866.484948063224,-1.23712404388694,0.244812920827968,-5.053344
56899961,4.34139639461475e-07,1.41316438901834e-05,-13.8150805219043
"Srsf6",16009.9398465739,0.196354216273369,0.137959771064146,1.4232715
4328251,0.154657452941261,0.274461729084255,0.253874897459174
"Plcg1",4033.45473312603,-0.248254305998288,0.156587036863205,-1.58540
777685936,0.112873715381329,0.216168520037831,-0.38025037328291
"Kctd2",2954.89627391146,0.274778546057912,0.178661025977002,1.5379881
7932056,0.124051505042329,0.232337812407423,0.401056565612605
"Tmprss6",55.0213615355822,-1.64176766362233,0.398199373894528,-4.1229
7901818699,3.74003777582747e-05,0.00046329896158249,-12.6040769288851
"Kctd5",946.617674941801,-0.207111520897047,0.0937137546086711,-2.2100

4399793714,0.0271021089018309,0.0728677494991588,-0.542447675631496
"Etaa1",498.476881547422,-0.179525079069573,0.146625494366221,-1.22437833778879,0.220809537236754,0.356597604435433,-0.185116799015126
"Matn4",169.440803405343,1.10182696912036,0.259013057359055,4.25394372142777,2.10038111470899e-05,0.000298710656269845,8.94246641928705
"Slpi",138.375752618433,-1.90742298040378,0.709760050966172,-2.68741947057638,0.00720064627698905,0.0263460688888596,-6.93622198114318
"Rbpjl",2.71815698905934,1.35481408255179,1.01542494419721,1.33423360366916,0.182127309847989,0.310249719326935,1.58564427056441
"Sdc4",18381.0930987114,0.6426297990294,0.185991503432001,3.45515675270805,0.000549972973084793,0.00372468670691466,3.59408229096432
"Il13ra1",2676.482028758,-0.0343120274895947,0.292131260798957,-0.117454145084487,0.90650017937688,0.942925053407193,-0.0020164655734949
"Nbr1",7145.01865671205,0.391983469316813,0.128678522137144,3.0462229656247,0.00231735920379008,0.0111868952105717,1.76118653188104
"Cyth1",2291.27527880365,-0.546187706106246,0.22349387105101,-2.44385988545334,0.0145310687278945,0.0452165301717981,-1.69115692380802
"Rnd3",1119.45860172519,-0.410244297675894,0.177264961368896,-2.31429998634732,0.0206512725503119,0.0588689613763647,-1.16199289131869
"Brca1",111.950141575916,-0.956255879266042,0.379665832279351,-2.51867773701176,0.0117796414611935,0.0385027729649695,-3.11454931971872
"Gast",0.193414357714749,0,4.78471990595412,0,1,NA,NA
"Cntnap1",613.30526074302,-1.08806080095256,0.334375757649451,-3.25400623717838,0.00113789781425684,0.00649001998870535,-5.48109504333199
"Nt5c3b",780.953712885513,-0.377389564588344,0.162322239462439,-2.32494059863973,0.0200751367080641,0.0576218188626283,-1.07701471215946
"Coa3",2945.47220572223,0.324067256061465,0.23054574725171,1.40565271719217,0.15982727373942,0.281128749063708,0.411222724723692
"Zpbp2",10.44582953486,-1.94612617396927,0.827877985046686,-2.3507403374901,0.0187361035489071,0.0546635563364642,-5.65652867338497
"Gsdma",6.22885053522355,-1.82049062137481,0.939359755452304,-1.93801215222195,0.052621739957791,0.120900721018984,-3.84630629261583
"Med24",1848.19340498774,-0.125707069481593,0.142771659313196,-0.880476350042494,0.378601311512854,0.52346960515371,-0.0813672082246196
"Psmd3",6482.06142002881,0.198934566749566,0.189723720711146,1.04854873182907,0.294385862424966,0.438592979947089,0.163958577293084
"Exosc10",2191.72061768262,0.0189484661220656,0.160456251080371,0.118091168118932,0.905995421305908,0.94269979257559,0.0011180997314536
"Glod4",1972.88322437049,0.16984096857268,0.227370980251111,0.74697733362941,0.455077243024618,0.595320480459941,0.088088934781491
"Vps53",2139.3348213827,0.528564752951893,0.128068774461872,4.12719458878913,3.67215649154702e-05,0.000456922190632954,4.06519016328776
"Taok1",3568.45713718317,-0.207839418718794,0.121084786221208,-1.71647838845001,0.086074520154627,0.175798598953227,-0.361311425658095
"Dnttip1",1204.32196607575,0.0870892085516332,0.114233722046244,0.762377404777005,0.445834787893318,0.586970151192932,0.0463995026367196
"Tnnc2",5.71967511439029,-0.0720923896138314,1.08448445284143,-0.0664761854583938,0.946998717013584,0.967309892988783,-0.00239608907732288
"Acot8",1251.12133689419,0.753948332340274,0.142578261369688,5.28796132802726,1.23687118458443e-07,5.34734242949604e-06,9.15211160130251

"Cd300lg",2207.24761161481,-0.985359341183274,0.499838471941484,-1.971
35554083286,0.0486832226034238,0.114103314619733,-2.13887121210973
"Spint4",0.468824111290567,-1.00637860337629,4.78162849227705,-0.21046
7752775382,0.833302617535465,NA,NA
"Pyy",0.190187088227992,0.864256293530331,4.78471990596623,0.180628398
425718,0.856659263798371,NA,NA
"Mpp2",353.707872798835,-0.8766794291908,0.406576591906981,-2.15624668
670392,0.0310644077857556,0.0807699457269854,-2.20585724512111
"Ppy",1.96653610703159,1.0109545872672,1.443380194526,0.70040768960335
6,0.483672735415246,0.621295934852525,0.481161576229511
"Vtn",107.476624556296,-1.31342867341161,0.690689212435781,-1.90162036
667646,0.0572208033619638,0.128966734938339,-2.69016562885012
"Nlk",1380.68587899277,0.245683776715229,0.191092007336566,1.285683164
5633,0.198553659111621,0.329733348552797,0.272579021958639
"Traf4",3165.77009772405,0.655919059711576,0.14680275711205,4.46802956
98461,7.89433954349169e-06,0.000141344198731865,5.81427153083557
"Aldoc",285.927094543913,1.47188450705171,0.389377390666037,3.78009751
550808,0.000156766947639649,0.00140729371320344,9.66452139055571
"Stac2",43.7111334542924,-2.28968705126026,0.3838211853544,-5.96550461
15969,2.43879159970309e-09,2.93528139574142e-07,-34.4398522739385
"Rpl19",25157.3445225739,0.0687583575526075,0.165909015097415,0.414434
125307992,0.678556201395072,0.783564632206166,0.0167702822861807
"Nek8",1363.81746164098,0.812926806593819,0.214699316723195,3.78635022
691712,0.000152876168703625,0.00138023382824381,5.35353139797376
"Cacnb4",706.495675468992,1.89973108346519,0.476329853719704,3.9882679
3792162,6.65574665782356e-05,0.000722571430079824,13.7401741365516
"Plxdc1",139.22186444259,-1.17714472355784,0.284048318564049,-4.144170
71542147,3.41045617554438e-05,0.000430123379759148,-9.12456488406774
"Arl5b",835.085923988675,0.194838086201181,0.170643543215363,1.1417841
1049097,0.253543768661713,0.39392979750482,0.181507764002009
"Zfp207",6030.15259246603,-0.187381840950989,0.104052092198989,-1.8008
4645095497,0.0717270851353912,0.152828620475342,-0.351985191728997
"Psmd11",4890.58231596613,0.0207555478521003,0.197744006995633,0.10496
1703605807,0.916406196906243,0.948764829144477,0.00109162392971459
"C1qtnf1",767.036606253871,-1.48069717408299,0.246348487786607,-6.0105
7951435693,1.8486131150136e-09,2.45452644100394e-07,-22.5364505723279
"Pipox",9811.48590736508,1.9674560693837,0.65219267016726,3.0166792105
7613,0.00255560103964986,0.0120273012481518,8.69728929643834
"Timp2",9513.41186528609,-1.17363535472618,0.224823367716328,-5.220255
20143892,1.78676743008337e-07,7.08097385198488e-06,-13.9170843553458
"Zc3h18",2386.58868734852,0.0066309448875947,0.110916738735499,0.05978
30856116983,0.952328397205566,0.970244861929682,0.000200299654954796
"Top2b",3568.85597725338,-0.545202235028305,0.0999381293335478,-5.4553
9764116125,4.88633730970228e-08,2.66873402982003e-06,-6.99707442296348
"Rarb",589.286823821224,-0.88980284754495,0.195612734413392,-4.5487981
6599523,5.39531583585077e-06,0.000105197868167418,-8.150298244027
"Igfbp4",29979.058090898,1.00223142108847,0.182537739803855,5.49054361
123027,4.00698531978752e-08,2.26182406888254e-06,13.028345959185
"Cdc6",112.788415757016,-0.994534701222316,0.301214137953128,-3.301753
05840749,0.000960826195900927,0.0056942824217861,-5.14004642773477

"Suz12",2763.06815677024,0.171099249906459,0.0870646845880289,1.965196
91900411,0.049391450431669,0.115249872075022,0.369686057490452
"Atad5",214.993692013123,-0.243934001709569,0.317136700623545,-0.76917
6198244963,0.441788718210725,0.583445978574418,-0.131432472728287
"Crlf3",1195.02838690174,-0.204384894128459,0.212160091823529,-0.96335
221375405,0.335370798633196,0.479895775117426,-0.150056596110301
"Tns4",311.672218798486,-2.66895749743579,0.587789646061735,-4.5406677
6323494,5.60763379150405e-06,0.00010818090741651,-24.3721342950593
"Tnfaip1",4834.96582821,-0.0738467419741465,0.137266980813923,-0.53797
8919156473,0.590591602463709,0.713853072541044,-0.0248921208474028
"Abr",3377.69365443391,0.123384685015249,0.246808992108244,0.499919731
292186,0.617131598243809,0.735953021563168,0.0378283861686052
"Rab11fip4",2352.37784544527,0.777749072725355,0.470900078487124,1.651
62230429872,0.0986115702574947,0.195200852475191,1.27062906655486
"Cd40",551.172039354509,-0.992134511471149,0.434156906984246,-2.285198
03672535,0.0223012182757051,0.0626599269455372,-2.74824549530614
"Slc35c2",1803.90287514267,0.229052632723268,0.153911121604735,1.48821
365431607,0.136694557447191,0.249580530115678,0.317919018654071
"Zfp334",323.086732655504,-0.699682194032106,0.201787948713911,-3.4674
1318543306,0.000525493455408759,0.00360128334134162,-3.93673738517811
"Elmo2",2269.77129393064,-0.369416337476153,0.156713463234587,-2.35727
250136236,0.0184097317720677,0.053913029452054,-1.07883722752734
"Wsb1",9685.88308356707,-0.00383823827442036,0.292537403948729,-0.0131
205043273477,0.989531652518332,0.992994758661988,-2.69824052869901e-05
"Ttpal",1489.51688309268,-0.435962153837754,0.125203463892511,-3.48202
949250696,0.000497628881420887,0.00344969205366041,-2.47167448822159
"Rhot1",2383.32954383357,0.245712923134596,0.159182973145083,1.5435879
7476817,0.122688180049965,0.230329095236163,0.360767051897502
"Hnf4g",147.632893383962,1.59538695125309,0.422736743391127,3.77394909
762315,0.000160683560001478,0.00143518463257389,10.4441396914877
"Rhbdl3",583.472052558805,-0.747176458268019,0.263479169523741,-2.8358
0846113413,0.00457098505355038,0.0185389040804907,-2.97965291733612
"Ada",122.050052027099,-0.800309833235038,0.305701064564612,-2.6179491
2089973,0.00884599936898361,0.0307614224534408,-2.78627379595845
"Serinc3",22528.2207092494,-0.328318979215601,0.166040984973188,-1.977
33697658211,0.0480035526600886,0.112849441736967,-0.716293754223708
"Tha1",1075.97935689258,0.214527274522237,0.155518080840165,1.37943622
608563,0.167760296419713,0.29140225029769,0.264522998056349
"Pgs1",2040.17460652124,-0.297335295942572,0.111931626782516,-2.656401
09493179,0.00789796147183912,0.0282247897320233,-1.06075990833682
"Birc5",270.197071868823,-0.559505437411161,0.408856617032066,-1.36846
369632628,0.171166978114448,0.29600121856102,-0.681137280032861
"Afmid",1574.5568829088,1.10408185629234,0.319522594592747,3.455410900
43903,0.000549454760003951,0.00372247008705943,6.17553593573499
"Pigt",3445.83001743898,-0.0380645862932534,0.179647479969123,-0.21188
4888670833,0.832196840034931,0.892673419561928,-0.00432164288070433
"Wfdc2",9061.87257307063,0.114786839794842,0.221617961914077,0.5179491
71644062,0.604493733192422,0.725586502318834,0.0368207465207566
"Etv4",168.505155537555,-0.33004771061535,0.434728861489278,-0.7592035
86080495,0.447730781213329,0.588564335853014,-0.174948071965457

"Eppin",4.01019795424579,-1.78570487203253,1.22851000409438,-1.4535533
8261889,0.146070169374919,0.262689186724975,-2.38710124666631
"Dbndd2",2167.34067713743,0.225455376143656,0.193197739046175,1.166966
94928594,0.243223717009172,0.382018369119126,0.216952683941385
"Mmp9",132.445465066624,-2.26061261182785,0.389275784541895,-5.8072263
9731666,6.35162253858337e-09,5.65497425832413e-07,-32.5201785753951
"Slc12a5",293.058913809162,0.382273310323183,0.312183862372802,1.22451
336022834,0.2207586294541,0.356586178203633,0.394192337640309
"Ghdc",1972.06288308643,0.924956258711575,0.166989858009756,5.53899661
773195,3.04209518665583e-08,1.87112845266294e-06,12.1992192916022
"Pltp",3742.60192471612,-1.52372933581716,0.262959315480598,-5.7945440
4584342,6.85070432811396e-09,5.96319630519015e-07,-21.838833976297
"Slc12a7",5944.97216855624,-0.0198698203523294,0.299702713682987,-0.06
62984332312281,0.947140230582657,0.967332262107834,-0.0006599411440558
08
"Ctsa",11927.8790833625,0.509419904567678,0.182697337433538,2.78832692
213151,0.00529810488128232,0.0208016907922234,1.97284116605325
"Zswim1",306.205320662211,-0.304032041919372,0.294471204816915,-1.0324
6781670351,0.301853016727317,0.446651750789195,-0.245042550001815
"Slc12a4",1982.60045868481,-0.644984663967072,0.208803741252041,-3.088
95166389058,0.00200864116079005,0.0100293176320319,-2.96837596910799
"Spata25",3.21979517139964,-0.143744988987211,0.994886510515732,-0.144
483805406806,0.885118443682586,0.928287868314782,-0.0106965521234855
"Myo1c",6741.01025365366,-0.312224163410333,0.13420096983669,-2.326541
78125747,0.0199896662455721,0.0574187555179972,-0.892144415691539
"Crk",6428.52269618106,-0.0251015342430726,0.132541350167391,-0.189386
438355811,0.849789952757798,0.904333833581025,-0.00252412749420803
"Cox7c",16195.3492333519,0.820994733731465,0.325844261147934,2.5195924
2995147,0.0117490788784908,0.0384368355659084,2.67540757374199
"Pitpna",6395.09914813704,-0.166697631717353,0.122542153424714,-1.3603
2889139464,0.173725869625175,0.299549611421264,-0.200949865545077
"Mlx",1891.08508176657,0.452656277503589,0.169526698472151,2.670117931
76606,0.00758246101859825,0.0273641978418399,1.62889256275355
"Retreg3",4112.34729876999,-0.20619149155116,0.164926430656107,-1.2502
0283729474,0.211225460634096,0.34524889189736,-0.219282526721928
"Jph2",482.872294665675,-1.45150688955143,0.288361862367949,-5.0336298
9000018,4.81278450533855e-07,1.52584832983888e-05,-16.0977556152892
"Dhx58",472.93073403028,-0.86521794444595,0.289330779704802,-2.9904109
9370317,0.00278602309710716,0.0127936595259998,-3.77131680443876
"Rab5a",3621.48286603613,-0.102070804722814,0.199538026461214,-0.51153
5603178144,0.608976064731353,0.728902106208221,-0.0322764053597533
"Hspb9",1.87579826627656,-3.16842740075553,1.75701816950313,-1.8032980
2830186,0.0713414221307389,0.152306476371964,-5.96253836057013
"Nkiras2",1073.72311401682,-0.24326047474175,0.162559356552857,-1.4964
4093025586,0.134538790175508,0.246544861921246,-0.340616067142113
"Ppp2r5c",4018.75177087171,0.0485323600234422,0.119248557663755,0.4069
84880775572,0.684019092737548,0.787540848550145,0.0115914707342052
"Ift52",1773.96490421658,0.15587117340029,0.151257545512482,1.03050180
321369,0.302774506209556,0.447370212342674,0.125377910513508
"Mybl2",270.411084389358,0.200548549153485,0.25134491162282,0.79790176

7171785,0.424927497809325,0.567909819198452,0.113468893451339
"Sgk2",3610.77517045228,2.22992774769744,0.747892886022646,2.981613797
07244,0.00286733460429247,0.0130593996997184,9.67397763146365
"Eya2",389.63987484832,-1.25954695509305,0.302795856697887,-4.15972321
691891,3.18633486895534e-05,0.000407926180399811,-9.83003890151198
"B4galt5",4625.93418193494,0.857029505302934,0.380930101459786,2.24983
40299668,0.0244594829396032,0.0672269205800662,2.31370670710815
"Gdap1l1",22.3478584597968,0.0995081283323117,0.807109190913635,0.1232
89549236418,0.901877816197397,0.939912002791001,0.00616642140895724
"Hnf4a",15663.8626288651,2.01359600704391,0.687214423125274,2.93008403
095877,0.00338870352568427,0.0148378489431376,8.47839499713062
"Ptgis",522.11740498879,-1.12819943572263,0.29787483684769,-3.78749493
465775,0.000152173786311867,0.00137683734826394,-7.432539734251
"Cadps2",777.997255431946,-0.020230544603898,0.157220836756501,-0.1286
75975915524,0.897614045791272,0.937121367789581,-0.00131382167914462
"Ddx27",1914.77693545828,-0.161579413927286,0.212679048914352,-0.75973
357390909,0.447413855811169,0.588364157321519,-0.0857032089068331
"Cyth3",1814.47434946707,-0.778969761638945,0.123109766722694,-6.32744
080649249,2.49260540524432e-10,5.11587580962671e-08,-13.0776030818494
"Cyth4",3838.35072260945,-1.16138615144079,0.413748282616226,-2.806987
2437828,0.00500072219147392,0.0198866166203056,-4.54997217098052
"Rac3",236.384093984739,-0.44522280335819,0.204625667770473,-2.1757915
7204067,0.029570849173857,0.0778205449037474,-1.13680956007115
"Rrp7a",3294.9718256543,-0.142366255241032,0.121570055954994,-1.171063
50015777,0.24157325381086,0.380086965814643,-0.137718736605605
"Cyb5r3",12713.4548721278,-0.153660703434335,0.195289722976827,-0.7868
34560938815,0.431378718946213,0.5735439660425,-0.085423163154144
"Ints2",1768.97700803257,0.622269246465255,0.151374729581035,4.1107868
4128806,3.94313095230676e-05,0.000482328527654186,4.7521987262195
"Med13l",2108.68047104795,-0.0772835788820287,0.212820947332183,-0.363
138966585842,0.716501077429228,0.812087310801319,-0.0160863774741048
"H2bc4",7290.05020796514,0.841310115559009,0.344833339966583,2.4397586
2554514,0.0146970782925838,0.0455368874223422,2.59900260952673
"Baiap2l2",3762.77874352333,1.61637093510263,0.430651053882843,3.75331
935340475,0.000174508303556331,0.00152239950905652,10.4861540136179
"Mafk",1918.18319124857,-0.313780738145798,0.106425707300932,-2.948354
73593371,0.00319470242393925,0.0141661575987661,-1.33573304428164
"Med1",2219.49825089611,0.162041791804178,0.0861686394422673,1.8805193
2643947,0.0600373349544604,0.133631045313022,0.326137085725969
"Erbb3",2702.54367100745,0.455728450533676,0.22167134351431,2.05587444
596445,0.0397946198384908,0.0975258292623044,1.06077086850918
"Stard3",1435.35597291737,-0.203123376160641,0.0963768475443946,-2.107
59514692649,0.0350660237326465,0.0885185565431306,-0.492481374125679
"Ikzf3",244.382461330527,-2.11755943926122,0.371564829619888,-5.699030
88359437,1.20490398836964e-08,9.25538046058966e-07,-29.4190216225637
"Mfng",1080.36969165421,-1.73869935437624,0.397547366827487,-4.3735652
6406257,1.22233679663963e-05,0.000198443987184676,-14.8224183911454
"Vmp1",8340.0952722222,-0.00864451916649425,0.179783976905226,-0.04808
28120241841,0.961650244378101,0.976728471369704,-0.000203548917227657
"Uchl5",1223.43655343789,0.236911590942189,0.155372094779676,1.5248014

212471,0.127308635324703,0.236428590490624,0.341652351622288
"Glrx2",1763.78808881565,0.253887649048569,0.228090289272101,1.1131015
2597375,0.265664828391938,0.407579890155481,0.22786881524126
"Ro60",2145.49882714312,0.255050939949544,0.090031616619142,2.83290414
553454,0.00461272156582606,0.0186404832747525,1.01571984779304
"Stk4",1604.33033162003,-0.249800060323674,0.144369931635108,-1.730277
61040323,0.0835806878050998,0.171940942268816,-0.439799040370128
"Wfdc15b",2896.5046014376,2.50818540139349,0.514712904551952,4.8729794
4001778,1.09927667018299e-06,2.98935795191464e-05,26.1299415027547
"Pmp22",2554.43960001949,-1.17338716606786,0.233386216234736,-5.027662
66576636,4.9649427773077e-07,1.56644748012857e-05,-12.9824907338272
"Gdf9",36.9833272850141,-0.723315310708917,0.401250963338444,-1.802650
65207786,0.0714430967865312,0.152393878719638,-1.36076354802349
"Zcchc10",481.889396440923,0.0206542977355572,0.147384316697519,0.1401
39047344818,0.888550129666596,0.930848400399704,0.00148006322732385
"Tbx5",29.2575643830873,-3.53421655508166,1.36181284639021,-2.59522926
696563,0.00945278410698917,0.0323941902536924,-12.1215717747363
"Psmb6",6600.5212024636,0.475793328847188,0.154123825165948,3.08708487
045979,0.00202129877531363,0.0100821907191264,2.18721465626311
"Spag7",2558.98364136585,0.324771395332662,0.11828441981752,2.74568194
047614,0.00603852744033359,0.0229645422335916,1.22562355656872
"Pfn1",16902.6135706025,-0.213857082173836,0.164995291080318,-1.296140
51876021,0.194927077592319,0.325512860474052,-0.240023203661747
"Tomm34",2084.9795544956,-0.405263183310864,0.0971625011758852,-4.1709
834391485,3.03287905422748e-05,0.000394496836553218,-3.17641207614167
"Ywhab",15291.4828915583,-0.214273778751888,0.178617409868913,-1.19962
426344187,0.23028529894255,0.367078381195377,-0.214740870089173
"Ksr1",861.776847732688,-1.04554197183977,0.15144395851062,-6.90382093
892804,5.06221023791846e-12,2.44907581740543e-09,-20.7305410569982
"Gpx3",493127.008994356,1.9239481687402,0.58667411133334,3.27941549076
986,0.00104022360310221,0.00606164967522023,9.82324317574886
"Anxa6",6262.14309998305,-0.99131988220631,0.2018267497729,-4.91173684
024425,9.02731594427803e-07,2.55463869784518e-05,-10.4832222882303
"Il12rb2",16.841683849714,-1.55904722735552,0.587120274547601,-2.65541
371153777,0.00792111958625405,0.0282712776300167,-5.55942040607901
"Zkscan6",497.202128311436,-0.209489892901069,0.127024270424718,-1.649
21154201964,0.0991043041859737,0.195977253855793,-0.341417551979783
"Kpna2",1166.5103468253,-0.0613994981691306,0.180999801554568,-0.33922
4118710538,0.734440901221966,0.826236908055152,-0.0117195514173041
"Smurf2",2136.70568705656,0.230728962370379,0.23835282476992,0.9680143
82011625,0.333037187053762,0.47774860750252,0.170432703509173
"Cep95",914.841823839382,0.127206921211095,0.180695165684469,0.7039863
0051467,0.481441305096415,0.618959683948958,0.0610230859359975
"Vezf1",3021.98689579961,-0.0766013237941204,0.0967397194971297,-0.791
829087290182,0.428460339510857,0.571182804579699,-0.0429002628742423
"Cuedc1",4313.94340599372,-0.192972601602806,0.172512713335156,-1.1185
9930710092,0.263311119209273,0.405276302679382,-0.174290193765758
"Srsf1",11258.1776000566,0.130817850772109,0.110669397143585,1.1820598
4805703,0.237181954712169,0.375371246183269,0.128180530576291
"Abi3",752.65704784446,-0.282142468571284,0.229333990165508,-1.2302688

6842053,0.218596438116869,0.354120417946388,-0.292897248545482
"Shroom1",306.309831725786,-0.247100849628913,0.437195966634966,-0.565
194714696964,0.571941331223371,0.698160150015231,-0.0887850059834801
"Kif3a",1791.18973724579,0.353169915358869,0.236692588982717,1.4921038
1650208,0.135671931539501,0.248061826815191,0.492346148699664
"Sept8",2976.92081093228,-1.14146595017517,0.162104893762188,-7.041526
77740701,1.90144663534723e-12,1.15857520300001e-09,-23.4868888410021
"Mtmr4",2590.09481501875,0.0936654495037458,0.161571621959514,0.579714
731880431,0.562107007087076,0.690130497083547,0.0347381335956337
"Mrm1",614.865769316988,-0.0548782695790939,0.136325932325184,-0.40255
1947696865,0.687277865553291,0.79005030153336,-0.0129325396214551
"Mapt",9439.03380004084,1.23029566191196,0.358019332742297,3.436394488
779,0.000589511920838102,0.00390970855929752,6.82111894348795
"Kansl1",2503.52947035274,-0.129367557924057,0.186148241726344,-0.6949
70614410853,0.487073717392021,0.623856226874441,-0.0610401860416033
"Gid4",1783.05855627381,-0.522637395941896,0.292493086755569,-1.786836
74796955,0.0739638780440191,0.156689376227368,-0.968703149796889
"Myo1b",1563.38591348942,-1.00544023384399,0.15757658716607,-6.3806448
1485664,1.76343879334737e-10,3.82039217696521e-08,-17.1732486390923
"Dhx40",2295.53526780498,0.0742537952253178,0.181415025973141,0.409303
44566007,0.682316987190616,0.786277577792117,0.0178539832337861
"Ypel2",2156.15263234128,-0.603607796362387,0.163470077049634,-3.69246
658016265,0.000222089532960616,0.00183876930516607,-3.80191955047966
"Akap1",4159.98970409194,-0.158245074275381,0.328126461982916,-0.48226
8553773698,0.629615183028944,0.745550640027836,-0.0464658523815089
"Nol11",801.895346922009,-0.248968646379873,0.179118189564294,-1.38996
852852013,0.164538434244327,0.287161689124229,-0.310640631214402
"Derl2",3389.68170551285,0.345895612859474,0.181196393218087,1.9089541
8344865,0.0562680023251235,0.127275349110819,0.713030059030792
"C1qbp",3482.77181408163,0.801333572127417,0.190441953747264,4.2077575
6790896,2.57917349668925e-05,0.000348742890696582,6.37955729000386
"Rpain",1003.34052229679,0.583950372087667,0.180300042508265,3.2387700
1893052,0.00120046329028199,0.0067571112107154,2.91809333390797
"6330403K07Rik",44.9278336522263,-0.956891964837859,0.355728747315793,
-2.68994837234336,0.00714630739139646,0.0261767239371497,-3.4858470623
0182
"Slc13a3",15190.263561864,2.5835181245221,0.799680294944471,3.23068874
005642,0.00123492341101513,0.00690919273112568,12.8527507098333
"Kcnab3",80.3609028375851,-0.234030470232659,0.22392149701143,-1.04514
516630225,0.295955881851311,0.440298167582547,-0.191975926773698
"Chd3",18283.9877433136,0.690008261070115,0.18498390445299,3.730098913
79748,0.000191404613617829,0.00163845738808658,4.42571305331321
"Kdm6b",2766.86461817458,0.00967281210265028,0.17885975427108,0.054080
4282219362,0.956871085441143,0.973609630735731,0.000258697865007095
"1700125H20Rik",0.459714718176847,-2.07048984337634,2.65714980750915,-
0.77921456950794,0.435853329056085,NA,NA
"Appbp2",2332.07075916765,-0.1771898343983,0.132467371083978,-1.337611
16377836,0.18102322334348,0.308813632286264,-0.208201123601946
"Wnt9b",272.560064946954,0.111625932628334,0.800891940274519,0.1393770
2081266,0.889152229846806,0.931329045262048,0.00794136265469751

"Adora2b",201.706289918754,-0.977065599396749,0.295490871784933,-3.306
58471273484,0.000944407897306163,0.00562182116910664,-5.06227420196579
"Ncor1",11251.5531026616,0.741222331710902,0.275321893817726,2.6922026
4844474,0.00709818004976285,0.0260395700113407,2.70408133578743
"Trpv2",631.354571595857,-1.04724471089513,0.257174648751789,-4.072114
86815667,4.65881925177788e-05,0.000548205538751751,-7.86361420326031
"Cenpv",660.274272249503,0.442941321941049,0.304918327786558,1.4526556
181664,0.146319395364042,0.262991848341453,0.591606710980131
"Pcgf2",1863.71815120634,0.216192731431553,0.188012986530479,1.1498819
0667619,0.250192513974062,0.389886000436881,0.203632125965403
"Cwc25",1272.38299439619,0.15955121826183,0.165899767798324,0.96173261
951631,0.336183932386099,0.48063604001057,0.116894397337091
"1700001P01Rik",3.60733357686182,-0.437454577694477,0.745337507323434,
-0.586921459602122,0.557256456533197,0.686379430794964,-0.164624961860
568
"Pip4k2b",1324.90662898855,-0.585952554080177,0.147645646534234,-3.968
64091718625,7.22837077431346e-05,0.000770157231462097,-4.2006445624056
4
"Trim37",932.895110764417,-0.0825329775823149,0.156030844865465,-0.528
95296217538,0.596838080553246,0.719305337063229,-0.0271920858449813
"Ybx2",1182.48276029672,0.900958767192714,0.212805838407731,4.23371263
652315,2.29864633915118e-05,0.00032013575943407,7.24980383819291
"Ctdnep1",4178.80490722546,0.0589383415103819,0.14998675880394,0.39295
6964870646,0.694351266858741,0.796145898336474,0.0134363399535903
"Elp5",1462.0011792557,0.267306734358039,0.207619098304498,1.287486250
25816,0.197924859022674,0.328941263316066,0.297211962648359
"Slc2a4",2762.46285920481,-2.06363817911495,0.6813685825906,-3.0286664
6898935,0.00245635704804164,0.0116672471918918,-9.18519110625196
"Gabarap",13414.4673040993,0.453069098532201,0.232620081082169,1.94767
836217959,0.0514534607575757,0.118881334026687,0.964869307103454
"Cldn7",2688.45703737458,-0.158129238395762,0.235866617990749,-0.67041
8051281695,0.502591330578514,0.637450449724834,-0.071202232781794
"2810408A11Rik",188.081271642065,0.165386527259779,0.277577421242744,0
.595821254190364,0.551294643369661,0.681274113095554,0.063473784318224
8
"Phf23",2070.37294904823,-0.00124997553518172,0.111126617542209,-0.011
2482100402897,0.991025416119977,0.994184492339468,-7.2904617754761e-06
"Acadvl",13118.5695409549,0.259696268821661,0.4629703230516,0.56093502
3026771,0.57484183340335,0.700516629231158,0.0924355561262992
"Dnah11",88.4774022702793,2.32589183859289,0.799315950321517,2.9098529
0316967,0.00361598898256633,0.0155827258775188,9.6794135516938
"G3bp1",6330.74613167027,-0.115891276818781,0.0595687258762081,-1.9455
0538246561,0.0517141825428914,0.119328181209621,-0.246370687550147
"Atox1",4024.42692313333,0.408236678527153,0.193954552092469,2.1048058
6365678,0.0353082105561395,0.0890221628932947,0.987471421512689
"Glra2",0.0821000670474453,-0.544048378937457,4.78471990596623,-0.1137
05376621746,0.90947135024012,NA,NA
"Sparc",36143.4366621254,-1.45268307196323,0.271689487251818,-5.346850
50443925,8.94978769931686e-08,4.20489061593446e-06,-17.9831447542588
"Glra4",0.585331304985444,-1.55588854782207,4.55838683620985,-0.341324

377181588,0.732859398283889,NA,NA
"Mief2",991.760809687054,0.185728522522922,0.24362208537172,0.76236324
0752727,0.445843239103443,0.586970151192932,0.0989526855719111
"Tbx3",1153.63980310196,-1.75406873919186,0.469851176445462,-3.7332432
632431,0.000189029864707876,0.0016222900619462,-11.2680571489954
"Mmp20",4.30637767032433,-0.483336134521447,0.948308129945988,-0.50968
2580227353,0.610273858929021,0.72996256066487,-0.152135864283424
"Mmp7",665.721682706686,-1.15929239213396,0.706507992929071,-1.6408765
4171854,0.100823045431007,0.198570478769069,-1.87412504792683
"Crhr1",0.133590265425185,-1.03635685866904,4.78109845466094,-0.216761
246081166,0.828394415030285,NA,NA
"Dusp14",435.490316236545,0.791608004131145,0.230230352081233,3.438330
33731295,0.000585313194674015,0.00388971938301089,4.39296391446863
"Tada2a",837.534171658956,0.431148792667786,0.190946327138699,2.257958
03003119,0.0239482761723684,0.0661018528891336,1.1712409178795
"Ikzf1",730.518248658503,-1.37310059579729,0.365814455245075,-3.753543
84199331,0.000174352021184021,0.00152239843665295,-8.90794654521408
"Pnpo",2487.66639618265,1.00594852571128,0.231511818212323,4.345128181
70132,1.39194358208422e-05,0.000220293149054205,8.47064126941881
"Cog1",1532.13144821459,0.264316770517528,0.101230908296037,2.61102834
071752,0.00902704125464578,0.0312494511533941,0.916056763555038
"Cbx1",5277.51816315543,0.097176900037837,0.140063011697392,0.69380844
2787088,0.487802347227565,0.624543316451708,0.0457445281977288
"Cdk5rap3",2632.57315210604,0.357865392278506,0.142885460959548,2.5045
6127499089,0.0122603366491643,0.0395912626673411,1.15559989517473
"Copz2",1313.00005423533,-0.296744059254719,0.217923085593033,-1.36169
171084923,0.173295198324984,0.298912754506417,-0.358349176168582
"Slc25a39",17837.9255120044,1.29053450658018,0.307067417046392,4.20277
253442753,2.63665507000237e-05,0.000353572906154789,10.2564208491138
"Sp2",548.085828579255,-0.72803762107846,0.189115042719955,-3.84970762
033221,0.00011825890524419,0.00113552583127476,-4.93657517376793
"Aatf",1072.8230713089,0.671019141532097,0.187994714190042,3.569351108
74325,0.000357866537483929,0.00265061521628455,3.98113208044293
"Lhx1",729.927750384686,2.11389107497359,0.704570599385134,3.000254448
33825,0.00269754157094419,0.01249975528679,9.26316837750442
"Dync1h1",14290.8572831504,-0.139728252606754,0.232120407301735,-0.601
964533110268,0.547197746133688,0.676940654407375,-0.0545180056065616
"Cpsf4l",204.568082866144,0.723296770372107,0.424743590694191,1.702902
14194867,0.0885863831300036,0.1798839551727,1.24077461997008
"Pex12",544.622347316172,-0.0949375270435178,0.242857488639126,-0.3909
18672409523,0.695857351457822,0.797356995693736,-0.0214988665595031
"Ndel1",3476.29060656927,0.497813419079962,0.119215294102217,4.1757512
9792602,2.97004172091456e-05,0.000387605096854007,3.9105850262642
"Slc25a35",1080.88223912111,-1.40590596922959,0.555768221066683,-2.529
66239511004,0.0114172325181109,0.0375972301364849,-4.6125313079303
"Zbtb4",4040.32533347407,-0.373607129219278,0.170427522569874,-2.19217
602641675,0.0283667988096214,0.0754050229297883,-0.96573011514693
"Tnfsfm13",425.309571203488,-0.718472479864112,0.256279965318661,-2.80
346721200292,0.00505563724943324,0.0200640779746488,-2.80838263661859
"Mpdu1",3494.82047053511,0.0791815500685024,0.281725630647496,0.281059

092445788,0.778665076455052,0.856940320636711,0.0122246019972886
"Fxr2",4674.34482777072,-0.0389160447854969,0.169482452914635,-0.22961
6955125721,0.818389430586758,0.883454607329233,-0.0048222959631 2275
"Atp5g3",29229.0610789473,1.09061477354732,0.406475707033882,2.6830995
1781796,0.00729432807822625,0.0265940181131742,3.95573500493609
"Cd68",1303.52417454768,-1.29503197994302,0.402596309319481,-3.2167010
7242673,0.00129673631381824,0.00717880881511301,-6.39308312055489
"Slc35g3",0.794692955964361,-0.605008654123072,2.07950396886799,-0.290
938927350265,0.771098028707842,NA,NA
"Acsl1",14299.0471604249,-1.57094812752677,0.732103556255853,-2.145800
4323336,0.0318889041406741,0.0823645321148315,-3.92202967679591
"Abca5",545.540976078897,-0.0228455230397185,0.126769051366932,-0.1802
13725616613,0.85698478336035,0.90916204940204,-0.00217562321720711
"Smyd4",687.045101450216,0.649319196596279,0.279613793791256,2.3222001
5969965,0.0202221604015631,0.0579755452888806,1.84908834670035
"Lsp1",3937.90229323666,-1.09418273422506,0.336058794753229,-3.2559264
9651835,0.00113023006882658,0.00646063496979791,-5.51689962580637
"Zfyve27",2359.32085101671,-0.240844433775549,0.129100798721083,-1.865
55339828596,0.0621038747429764,0.137010788610382,-0.478725423051971
"Avpi1",587.512963849556,0.248687551726607,0.274367959229134,0.9064015
79926902,0.364723336862131,0.509702954568364,0.167597296698897
"Sfrp5",95.0254754138792,-1.10253391979397,0.444571588925854,-2.479991
85566006,0.0131385383655335,0.0417631596105594,-3.50136181696949
"Myh11",4494.44876787104,-1.67584735196638,0.445844793089855,-3.758813
3313214,0.000170721145836723,0.00150009955003354,-10.8967345478266
"Rad51d",795.199321611424,-0.294542311180678,0.274422974675112,-1.0733
1505873145,0.283129780958667,0.4270821962813,-0.250590350265591
"Fndc8",7.46378778482221,-0.221890616922396,0.706213604523935,-0.31419
759616777,0.753370968291449,0.839720296103966,-0.0387612782614773
"Unc45b",864.901092844156,-0.946429292189597,0.31912139298982,-2.96573
43975676,0.00301961143901421,0.013600458267013,-4.06742354235328
"Pank3",3902.17678678182,-0.650551753240494,0.44405771268079,-1.465016
22348387,0.142916491542695,0.258376054900276,-0.880417175082418
"Rars",2260.37689269532,0.0581262471157481,0.0644559295300685,0.901798
291321396,0.367164027631983,0.512052702795014,0.0389055086920845
"Wwc1",10238.5415180909,0.88672557228098,0.349425818091993,2.537664724
1548,0.0111594837097296,0.0369382476711158,2.92487116564286
"Mrpl58",2487.20150085995,0.686402350297628,0.180859410326458,3.795226
07675567,0.000147508978700809,0.0013471335207065,4.53696596115267
"Fdxr",1061.83019949846,0.617129766000391,0.184174865207491,3.35078168
948371,0.000805838038286993,0.00498759665537849,3.27128215321384
"Otop3",4.36179233810672,2.0823421446507,1.05364289249422,1.9763262861
493,0.048117834319162,0.113090830949255,4.53859791053708
"Sult4a1",194.379053823632,0.234224804046782,0.55650854283311,0.420882
674782268,0.673840756989632,0.779596931097362,0.0583168813182696
"Pnpla5",2.47246032684702,-5.21141774515041,2.3573917183827,-2.2106711
0082398,0.0270586211607567,0.072780934665807,-13.6554844034169
"Mrpl45",2386.70312366984,0.623118635634983,0.170931825422275,3.645421
99263135,0.000266953384717392,0.00211587686797549,3.83734274376044
"Mb",7.23503905695162,-0.448995284757058,0.878289441920499,-0.51121562

3604981,0.609200079794349,0.729080724025916,-0.141869408551377
"Irf1",3600.87113439692,-1.00291135545898,0.263464826228894,-3.8066233
3494062,0.000140877053017187,0.00129750627289991,-6.66666379381378
"Slc22a5",6949.48379696329,1.80086966659344,0.279440575600503,6.444553
23899709,1.15941431385201e-10,2.72364581825138e-08,31.3688253658713
"P4ha2",1390.3276330617,0.038054139511042,0.187017787891298,0.20347871
6864946,0.838760879196259,0.896904662858871,0.00414050755416382
"Alox12e",3.18085787507413,-1.43560049917859,1.0302327271685,-1.393472
03920051,0.163477094553314,0.285872321937266,-1.7976732933718
"Arrb1",4639.68483934173,-0.598067696413734,0.150499752808906,-3.97387
826392725,7.07117143939335e-05,0.0007563011559259,-4.2983549411367
"Csf2",6.78432894794211,-1.45371431747312,0.704673830059371,-2.0629605
5772447,0.0391163757318821,0.0963170272018828,-3.40185164647784
"Tm4sf5",19.1491773698643,-1.10422950393531,0.676568409292251,-1.63210
325632914,0.10265773590343,0.201263999396767,-1.77023206081727
"Cxcl16",6672.14862681796,-0.807701056740009,0.425990601648195,-1.8960
5370075054,0.0579529439443276,0.130210474882058,-1.6465818783055
"Pelp1",1573.11392793326,0.123080171027642,0.208106180325741,0.5914296
77076329,0.554232560714699,0.683821202861178,0.0467777014714491
"Med11",852.345658134938,0.0641749126902654,0.177463532438217,0.361623
099735196,0.717633700236469,0.81285127728655,0.0132974986381085
"Alox15",5.03117948219854,-2.95579383214261,1.10715828196373,-2.669712
07305608,0.00759163150863005,0.0273759258655944,-10.6352159828887
"Heatr9",10.8459535246922,-1.75742772130269,0.636214485559316,-2.76231
956548063,0.00573922741729732,0.0220327839494021,-6.70497999057408
"Ccl6",1398.20083358331,-1.43660700968926,0.428431774230883,-3.3531756
8886726,0.000798899777375863,0.00495793668379305,-7.62373668267239
"Ccl4",65.779608650085,-0.88460319438156,0.611689288825296,-1.44616427
088396,0.148131132428479,0.265376687547981,-1.17351902056939
"Natd1",1086.23163787095,-0.617802522916322,0.264654088026737,-2.33437
740381289,0.0195759689085438,0.0565135092950529,-1.77511689422937
"Map2k3",2342.90811043588,0.234365250542756,0.170609608397219,1.373693
15095723,0.169536964082064,0.293964582096227,0.286932435956944
"Ywhah",6439.01293697616,-0.452210772317384,0.18581000466212,-2.433726
71530627,0.0149442733523721,0.0460612459412824,-1.39180677874629
"Hoxb1",1.78500117194758,1.11497307089916,1.28839936583851,0.865393992
315044,0.386822577279716,0.53122035581067,0.705307837890585
"Sart3",2175.67239288642,-0.0737129925880115,0.16499282318232,-0.44676
4842047448,0.655044858679898,0.765114997576268,-0.0197351155834471
"E2f2",328.491773690099,-0.95407497050343,0.267163687211755,-3.5711251
7970013,0.000355450950911198,0.00263720800641801,-5.66533008545147
"Slfn3",95.0132784080866,-0.555344568509332,0.276801828533056,-2.00628
937840638,0.044825382604174,0.106911964527974,-1.24161136613804
"Nars2",581.828805867786,-0.0131674841592691,0.170245540526912,-0.0773
440767876534,0.938349827693423,0.961780210291035,-0.000513127983473806
"Slc35b4",3733.9056291598,0.182304337426596,0.156888505562664,1.161999
32412372,0.245235739472665,0.384373508700805,0.174308564027554
"Dnah1",68.8021574996828,-1.54232227091329,0.426655992582181,-3.614908
25800652,0.000300453999537296,0.00231185954340103,-9.36143823987564
"Dalrd3",6426.43798595767,0.594321445452315,0.26290894677416,2.2605599

8757181,0.0237865178293659,0.0657670908447216,1.61752646613088
"Fis1",6812.35403669025,0.663202506679293,0.18440405713267,3.596463749
17962,0.000322572512636245,0.00244823622085695,3.98743041041835
"Plod1",2674.6529546626,-0.585176817143363,0.127198482781835,-4.600501
54959028,4.21474885039236e-06,8.72390372451701e-05,-5.46956499649793
"Rab3d",1476.06374550305,-1.40682055168555,0.337765004040189,-4.165086
77588801,3.11234162837653e-05,0.000401990692825569,-11.0000447208388
"Mfsd3",391.561729410917,-0.0314179958765628,0.357822970272247,-0.0878
03183380482,0.93003310756792,0.956625107161812,-0.00139319024128882
"Slc25a22",1936.26092248609,-0.708414192418234,0.331483111397725,-2.13
710493252929,0.0325894578640668,0.0837965514220723,-1.75641623830667
"Atp6ap1",10086.124906866,0.172253030376942,0.119287464338133,1.444016
19510221,0.148734419829018,0.266350451673971,0.227880828772111
"Dnase1l1",928.996644534383,-0.460019817054821,0.146702197764979,-3.13
573909636844,0.00171421631481824,0.00891229260464894,-2.17144247420672
"Aldh3a1",909.474475681807,-3.65185807461085,1.14014754319762,-3.20296
973527565,0.00136018274557986,0.00746225187769162,-17.8864285426474
"Ccl9",1391.66851418842,-1.44397832082382,0.411878759945711,-3.5058334
1810136,0.000455180002218723,0.00320980097043785,-8.29066845269971
"Scrn1",464.174397045188,-0.537370998920011,0.312499242033101,-1.71959
136740271,0.0855067464546396,0.174998482457496,-0.936625816471225
"BC005537",13226.4906046577,-0.14456101428805,0.0960358431313291,-1.50
52818778335,0.132251622309188,0.243199295650717,-0.204391063139265
"Isyna1",4481.70313487344,-0.271388461375626,0.311800207663564,-0.8703
92176481349,0.384086120692144,0.528728549933312,-0.172950470403401
"Hars2",2061.28112886272,0.190125147928607,0.114919079150559,1.6544263
0879001,0.0980409246378986,0.194333559818486,0.31145906574775
"Cacng2",0.237219538624863,2.40478170765777,4.73929969109492,0.5074128
8046762,0.61186514994978,NA,NA
"Tmem160",1458.17912237514,0.30235418327248,0.264347649577609,1.143774
81228072,0.252717038163654,0.393018633548902,0.282368044037168
"Rab5c",11748.7053048495,0.494131625055296,0.191654149721253,2.5782464
1821726,0.0099303155095468,0.0335740058618248,1.67708429021678
"Styxl1",10.591562603136,0.322394908061339,0.484702197940027,0.6651401
81809593,0.505960836211747,0.640682191483092,0.143537223910459
"Mdh2",13984.6452011109,0.404462589958394,0.376123543453368,1.07534504
818505,0.282220274573282,0.426106782065061,0.345033001450039
"H13",8462.79838322729,0.505485611849399,0.154595377064042,3.269733037
61858,0.00107649026425403,0.00622646311848861,2.56733454345347
"Rnf145",4195.43655468341,0.112030135192803,0.192254041157405,0.582719
273511035,0.560082296405711,0.688466842649681,0.041819518638766
"Scn1b",1409.95147319752,-1.21651113003563,0.236075063324119,-5.153069
16751907,2.56257392480565e-07,9.35675400484282e-06,-14.0864837256536
"Atp6v1e1",9508.56993956761,0.826939361831863,0.275141629989032,3.0055
0433558465,0.00265140816117199,0.0123394484712482,3.63436041956578
"Chtf18",148.20076961585,-0.640080906765902,0.336961979661453,-1.89956
418053156,0.0574903366154172,0.129442676000501,-1.30865639268539
"Lhx9",2.50833788618998,1.16417374701382,1.25144575549985,0.9302630512
7291,0.352234903435576,0.497275805313653,0.813303964533779
"Etnppl",334.439785136472,2.42376692165107,0.862436209258584,2.8103724

0277136,0.00494842059403063,0.0197228750495522,9.51565111991808
"Rps6kl1",51.0021609941354,-0.710657097509813,0.292172606165057,-2.432
31939789845,0.015002471241217,0.046196807369117,-2.18516013009299
"Ppp1r12c",4593.46579773709,-0.122899136440724,0.165403254005645,-0.74
3027319381092,0.457465140618321,0.597431702061354,-0.0633072265062657
"Ahr",886.814122048646,-1.15522386627504,0.262909101105617,-4.39400485
345296,1.11281329410185e-05,0.000185926594759193,-9.9235562459565
"Map1s",1609.1110741755,-0.363898110234219,0.166398422684895,-2.186908
41152578,0.0287492107579496,0.0761723211521267,-0.936949250917059
"Dpep1",4496.2701242526,0.443727016274173,0.19369607616086,2.290841534
16545,0.0219725804653677,0.0620001988297742,1.23383518965629
"Tmem129",1156.70380065719,0.196706164984379,0.0618776654698948,3.1789
5259122344,0.00147808264289491,0.00795244353508968,0.950931899249499
"Nop9",1105.90061829579,0.158317636398079,0.115293639163278,1.37316887
164842,0.169699854364535,0.294168541998552,0.193717840460183
"Hsd17b1",47.7491871119695,0.0247522315598225,0.337758858534127,0.0732
837375968379,0.941580332418536,0.963774125754455,0.000913315790750429
"Atp6v0a1",3838.58724768931,-0.16680506277081,0.179659035625757,-0.928
453513010481,0.353172371046174,0.498058360383214,-0.11626947056283
"Psmc3ip",154.307164678125,0.0971863009348427,0.233847686625486,0.4155
96589118666,0.677705223005784,0.78297292368848,0.0237773247477383
"Grb7",4484.12100826464,0.868375750799705,0.278804787604186,3.11463715
620451,0.00184171309996518,0.00937101305405037,4.05543118094765
"Noxo1",486.878943271602,-0.839439969456346,0.355843363254762,-2.35901
53874961,0.0183234959630772,0.053692744858443,-2.45492321232393
"Aoc3",2402.82253892576,-3.1691660054809,0.78814575309762,-4.021040515
69807,5.79416372924601e-05,0.000649136580996668,-23.2612582964321
"Zfp687",1299.43892567893,-0.028531891267156,0.111800883540762,-0.2552
0273510856,0.798566518687367,0.871366611526459,-0.00392862692275717
"Gdpd2",58.1415810456411,-0.479004156906112,0.401048571253369,-1.19437
941247145,0.232329586346821,0.369491213261853,-0.476910103511839
"D8Ertd738e",6480.17955902714,0.316869007789777,0.182416841342314,1.73
706005135325,0.0823765717382778,0.170055944494753,0.561373946582651
"Sec14l4",33.1097947641034,-2.31929086810656,0.903743645918368,-2.5663
1499273197,0.0102785440341373,0.0345953826303485,-7.80217578493889
"Calm3",9681.75625622144,0.166153835723465,0.121310945514435,1.3696524
6638601,0.170795413353392,0.295463487673182,0.202576419870787
"Cops3",2032.71928483243,0.253963144788139,0.119998143551595,2.1163922
8134344,0.0343114580004113,0.0871542613090676,0.619689275932018
"Fkbp8",12245.1970088634,0.159007378935617,0.107549532016805,1.4784571
9041131,0.139285447457979,0.253338400609672,0.218321763507533
"Ffar3",0.246343069730164,0.697171411930329,4.74828381414899,0.1468259
77388481,0.883269379902654,NA,NA
"Ddx39b",10438.3319669116,-0.105980272608025,0.142660978827423,-0.7428
81995336853,0.457553127160444,0.597444061650789,-0.0545898682706158
"Gipc1",2095.10452516978,0.0138979077342878,0.112422796690312,0.123621
79329671,0.901614736291258,0.939775885413669,0.00086325258296775
"Tlcd1",2668.10614056485,0.0602070782583048,0.395966273166114,0.152051
026409128,0.879146690236147,0.924555139904302,0.00472279897349473
"Plscr3",2348.91791638481,-0.748746119510165,0.115948393021589,-6.4575

8082538284,1.06389934362007e-10,2.52974504901269e-08,-13.0974880805923
"Ptger1",887.222772620412,0.280890506335974,0.387114193275345,0.725601
156494366,0.4680833119305,0.606350538775183,0.140528681391225
"Arhgef25",2182.50643307492,-0.766351975567456,0.236161798908065,-3.24
502937863285,0.00117438506936253,0.00664292430589807,-3.84264437872434
"Xab2",2561.15370406984,0.0169201494826999,0.135988772096153,0.1244231
3598314,0.900980255816537,0.939378270034268,0.00105813602112408
"Cdc37",6048.71834411069,-0.656661563942746,0.221530823719818,-2.96419
953176927,0.00303471443433447,0.0136558647682099,-2.81943302657026
"Rab4a",889.917580170275,1.15913476025258,0.260836330972374,4.44391606
004972,8.83360803281923e-06,0.000155169089571089,10.1667653432002
"Trip10",2770.76448641163,-0.249359266971667,0.151191722981894,-1.6492
9178696858,0.0990878714253737,0.19596463303093,-0.406410989742252
"Cd70",2.08392143511821,-1.03256139838511,1.34566263590827,-0.76732560
6605828,0.442887947466055,0.584224964460668,-0.554969904559537
"Cops6",4141.7380289456,0.207637522911059,0.291660807883518,0.71191437
8958948,0.476517797558007,0.614087509371185,0.101247759923324
"Ubb",57406.3195709234,0.305136509795071,0.197595557820331,1.544247821
97039,0.122528307692592,0.230159628457626,0.44824009353127
"Ap4m1",2068.46310785372,-0.280508719028207,0.186217839136458,-1.50634
719170301,0.131978069483921,0.242787847796725,-0.39707896197341
"Gyg",1614.5858634761,-0.897359371038913,0.397919986046866,-2.25512515
707925,0.0241254739837495,0.0664780231395065,-2.43263704009699
"Rcn3",3250.28710343259,-1.30847344510182,0.304259692343404,-4.3005152
4414547,1.70401448320551e-05,0.000253431536182616,-10.8347055568937
"Slc6a8",6870.45301574455,0.969484600410173,0.218416552881545,4.438695
63739502,9.05056955361545e-06,0.000158125452649099,8.48504734080275
"Arid3a",991.947700229098,-0.0687614804810522,0.245700809553243,-0.279
858583315541,0.779586003696074,0.857497366018682,-0.010571195527286
"Pdk4",6240.73216560477,-2.56200640170429,0.797174948064492,-3.2138571
4381108,0.00130964813953043,0.00722567046535493,-12.6309868350118
"Ubxn6",3457.05817806597,0.260610030668009,0.135026405885963,1.9300671
5211029,0.0535985178211711,0.122573762664461,0.547031474531491
"Mydgf",2513.58707081421,0.307771497199966,0.11953777573467,2.57467980
568005,0.0100332921434632,0.0338693092474456,1.0418822318384
"Cyb561",2046.89202849662,0.438098410787116,0.215695363415153,2.031097
95153037,0.0422450592287514,0.102195305811687,0.999245317909156
"Sema6a",924.731336427389,-0.611584044415096,0.208259867323562,-2.9366
3898030296,0.00331790111361684,0.0145867950199101,-2.58555631956453
"Ccdc12",3380.08975049828,0.0504101620651454,0.172116021028829,0.29288
4774838607,0.769610226731033,0.850961789769859,0.00813559784685607
"Fmc1",1734.56652822903,0.822594437702048,0.298805806297353,2.75293993
746377,0.00590627309906848,0.0225539586536696,3.11915034107264
"Akt3",624.835604786145,-0.946391286958881,0.213975018094344,-4.422905
51199582,9.73823376952881e-06,0.000167663873137123,-8.22749934611814
"Mrpl24",5530.65378975008,0.417410359211385,0.103008067744385,4.052210
35935935,5.07360151436882e-05,0.000585355516728777,3.10690684282795
"Gle1",1991.65366267852,0.258512868060331,0.160556916202524,1.61010110
417322,0.107375786087878,0.207864682003717,0.406089586409649
"L3hypdh",1030.97702457013,0.545553999688967,0.230280860786604,2.36908

094674233,0.017832350152564,0.0526117681720081,1.6065558496882
"Lyst",1179.09169299489,-0.42620748215871,0.250903341961575,-1.6986919
298348,0.0893772448022123,0.181076217700908,-0.72831921921861
"Slc35e1",1308.9095952,-0.352484224804954,0.225240209433534,-1.5649258
4379775,0.117600298137347,0.222986542164932,-0.528953287129135
"Calr3",75.4472542540622,-0.660610570425248,0.506354198666083,-1.30464
124157661,0.192015038064273,0.321973616458307,-0.74866049506678
"Tmc4",3278.45157907638,-0.0282773364095717,0.238293416892201,-0.11866
6041128462,0.905539941651055,0.942441117052384,-0.00167633244464853
"Syne4",1965.04051612433,1.6250156201738,0.451745594263564,3.597191961
1588,0.000321671092844902,0.00244234539263625,9.77413822382859
"Polr2i",1271.9106403125,0.773387768559368,0.180048794997441,4.2954342
9363335,1.74351673598683e-05,0.000257929357498265,6.39036764851683
"Prl8a1",558.127593550975,0.308387983734448,0.181301651005065,1.700966
21859132,0.0889493307932351,0.180413915555006,0.52811489617016
"Krt10",443.194341205308,0.928707161852533,0.324622764473832,2.8608811
9346109,0.00422465335048157,0.0174776768571376,3.758320765801
"Iyd",1502.68583803592,1.76643231193619,0.565092397153934,3.1259176744
0646,0.00177251164720856,0.00911884751906925,8.29766007679236
"Rmnd1",2820.56529738624,0.722549945557431,0.221351957900486,3.2642582
0855973,0.00109751094729496,0.00631805977276561,3.65924084270445
"Ccdc170",45.3858978906381,-1.29894865835912,0.584136932872518,-2.2237
0575332652,0.0261682478942197,0.0709735008264705,-3.43630203884851
"Esr1",804.109435631075,-0.309722513529882,0.229886424112074,-1.347284
92439765,0.177888485559937,0.30472636635231,-0.368055981447477
"Vip",2.34429859947268,-0.0534337171408483,1.23964458165243,-0.0431040
621898431,0.965618581151535,0.979228745789391,-0.00112157432404405
"Fbxo5",231.124979443314,-0.0731940547009656,0.22759157988889,-0.32160
264776359,0.747753737700172,0.835944176232897,-0.0131158946666547
"Mtrf1l",466.874049755505,0.649305296242398,0.168710703074552,3.848631
3221958,0.000118779615921028,0.00113862583639538,4.40094789208841
"Rgs17",51.2269786084997,-0.747369839625624,0.493955488526106,-1.51303
074261948,0.130271850059516,0.240443064413143,-1.06520527308159
"Hdac2",3592.30171666,0.102192660462489,0.104378704616911,0.9790566077
39625,0.32755201024356,0.472312460858521,0.0766561983556302
"Frk",1130.08058567328,-0.11672059664908,0.305479421171131,-0.38208988
4161761,0.702394694891011,0.802126628446948,-0.0257355834439189
"Rwdd1",2526.82039956021,0.320564510193255,0.172145956559469,1.8621669
4600384,0.0625795619864689,0.137737734888922,0.635488326820233
"Clvs2",4.37636551808226,0.0847434934636937,0.979166268702727,0.086546
5816913487,0.931031928121199,0.957120165603645,0.00371399635376463
"Trdn",16.9563046199428,2.05462438540891,0.728722203346968,2.819489204
48996,0.00481001489175495,0.0192736683845948,8.11374343581315
"Hey2",164.631528111694,-0.415339532380345,0.32854604908776,-1.2641744
8492707,0.206167406700202,0.339228024965447,-0.44901640705028
"Stxbp5",1653.79846383693,0.140581362181462,0.137224903813213,1.024459
54250999,0.305618270691253,0.450139374674274,0.112211765317946
"Hint3",841.369985944673,0.267849810874181,0.34791639443852,0.76986832
2263016,0.441378006497336,0.583076704890531,0.144487973076331
"Trmt11",401.964001134685,0.0777647258579026,0.138120221163816,0.56302

2019532323,0.573419889974612,0.699527060922542,0.0277892869606453
"Katna1",1329.8127189105,0.0367372508283668,0.181251829055985,0.202686
235055975,0.839380282702206,0.897124821651609,0.00398820594362999
"Pcmt1",2742.61244249153,0.0885086079516032,0.153356488530296,0.577142
896266289,0.563842928607685,0.691782621570139,0.0326139619384296
"Lrp11",396.69896826843,0.659451569537412,0.30554582209303,2.158273888
41412,0.0309065436201279,0.0804667896254845,1.66175908659706
"1700021F05Rik",1763.00892322549,0.634955485833924,0.224141547385268,2
.83283261510872,0.00461375383546802,0.0186404832747525,2.5286591359129
"Sec63",4472.57088562681,0.420667359290776,0.116779212975832,3.6022451
9904785,0.000315480573862894,0.00240376718608,2.53692626254615
"Nr2e1",0.273070954020578,-0.0591317168524064,4.78471990595626,-0.0123
584489823106,0.990139635359838,NA,NA
"Snx3",4970.90454734805,0.278599409691524,0.146216608127224,1.90538826
785745,0.0567296191346778,0.128111433158206,0.572481340478307
"Aig1",1484.26712379147,0.751705461550696,0.20543455014,3.659099509008
69,0.000253103053435331,0.002025427025393,4.66205827236832
"Adat2",127.436146961078,-0.443487783956561,0.311658488540594,-1.42299
279584293,0.154738243668902,0.27453014331722,-0.573294091564638
"Pex3",1208.82241000181,-0.317769507706435,0.322949482712804,-0.983960
417081777,0.325134975562253,0.470009026802552,-0.239917051902509
"Fuca2",12976.211613761,1.64346016796216,0.374863230334666,4.384159434
61546,1.16434478514265e-05,0.000191581389204316,14.0683441646881
"Cep57l1",331.891901594857,0.111074126049917,0.236924633524584,0.46881
6283041297,0.639200960015621,0.75319636903273,0.0314816621201137
"Ltv1",1011.49648533325,-0.208876549007841,0.138501981787113,-1.508112
34837706,0.131525775328177,0.242129018627802,-0.296246384417628
"Plagl1",1263.03185361598,-0.131560222058719,0.228900214110866,-0.5747
49231099448,0.565460906714545,0.692939272146326,-0.0482579884214879
"Cd164",12838.5457988188,0.256964390881553,0.236459288400415,1.0867172
6376176,0.27716178201099,0.420650768712562,0.222518909810975
"Utrn",3285.43152663648,-0.644013927094657,0.190749691359092,-3.376225
26414619,0.000734877245442763,0.00463860036634607,-3.46050747166131
"Smpd2",3750.76499137887,1.18254086331886,0.24064546071184,4.914037687
72886,8.92195876403123e-07,2.53217397352374e-05,12.5158318679468
"Mical1",1457.70642085687,-0.776654045251831,0.353703825423689,-2.1957
7507911176,0.0281080462357002,0.0747963798883041,-2.01385290493872
"Zbtb24",913.415330858729,0.162016666960871,0.0983974690158274,1.64655
319472506,0.0996499177098268,0.196796727996172,0.263371688670738
"Grm1",6.64102295793139,0.452277527831248,0.668160696806077,0.67689932
974689,0.498469829400315,0.633996394888933,0.20610830167302
"Wasf1",102.717001982754,-0.222230793055603,0.286137727670377,-0.77665
6734031266,0.437361319578199,0.579364835188241,-0.121298653103486
"Rab32",896.836984027207,-0.621720319757117,0.189751016739675,-3.27650
586773969,0.00105100128803211,0.0061134913824732,-3.16906840609266
"Gtf3c6",1707.95466338691,0.426884349756863,0.190618874917526,2.239465
26775777,0.0251256587706809,0.0687193287572922,1.14307879690493
"Slc16a10",1473.88111466017,0.379360534261359,0.217905491861147,1.7409
4067580038,0.0816939778076954,0.16901006399737,0.674426025627988
"Rev3l",1254.88250071925,-0.0986593106808036,0.158129771803409,-0.6239

13571464956,0.532684346978277,0.664849532542725,-0.0402721910305803
"Traf3ip2",505.294152067023,-0.359989190887731,0.116922508853967,-3.07886988071173,0.00207787413760025,0.0102802308893504,-1.64786224223202
"Fyn",1396.65006859746,-0.815621809524172,0.228094058589636,-3.57581348048857,0.000349140523621968,0.0026002833955 6193,-4.85469099003891
"Tube1",99.6971800135977,-0.416872121841371,0.267386094086313,-1.55906433079801,0.118981154892392,0.224992198437771,-0.621843788249291
"Lama4",1120.88824743714,-2.04973850144638,0.603147091216401,-3.39840568129502,0.000677798298292742,0.00435300106064291,-11.1442022953341
"Popdc3",8.5378840198936,-1.02785890658752,0.924057324152956,-1.11233240592484,0.26599525553745,0.407765979437785,-0.922053011200203
"Prep",1151.28004853586,-0.510488726448327,0.262996594636403,-1.94104690653537,0.0522525881145825,0.120250292129076,-1.08130698029152
"Tnfaip3",851.406242827646,-1.15361024412603,0.310695083832016,-3.71299806195118,0.000204818456155428,0.00172582120056981,-7.33932868788482
"Perp",3327.08380383514,-0.150204035464346,0.393127312811796,-0.382074789945348,0.70240589055817,0.802126628446948,-0.0331183064453305
"Arfgef3",669.429904840327,0.434377709993346,0.265301625138238,1.63729758446451,0.101568305403929,0.199715492009461,0.699722314351572
"Hebp2",362.400387247797,-0.861724520775344,0.342648509307514,-2.51489353482633,0.0119068333932882,0.0387902151215262,-2.80024901722942
"Reps1",1649.39000009482,0.204158638368421,0.15026853956631,1.35862529147913,0.174265356608603,0.300293939297795,0.245601512140325
"Fam184a",500.724302567768,0.959391208311532,0.395871281803803,2.4234928180191,0.0153720572155622,0.046987781673444,2.93369136401969
"Asf1a",873.328322940159,0.207373773773652,0.161483773310549,1.28417716233848,0.199079974079651,0.330438523419174,0.22963216475023
"Gopc",2136.16245828545,0.132585397458345,0.0998308265156381,1.32810076893008,0.184144819486014,0.312295006552865,0.154303814201401
"Qrsl1",666.695581793815,0.403685236219106,0.108790439182022,3.71066832025269,0.000206712797872243,0.00173727850556595,2.56559546795784
"Rtn4ip1",1652.86618424168,0.800214587541996,0.28173852304907,2.84027394934069,0.0045074806408135,0.0183365027195038,3.19994732326453
"Nmbr",3.0104763859534,-0.736055763336751,1.09621106395114,-0.671454419264612,0.501931087239553,0.637027425567715,-0.331918877634171
"Crybg1",1243.16733540005,-0.337221125898676,0.202290948972299,-1.66701045010597,0.0955123269522713,0.190660404493114,-0.558863562088104
"Gje1",0.815289496993794,0.572368818226939,2.11296666337875,0.270883979452704,0.786480267065799,NA,NA
"Vta1",1276.91489218517,-0.152708186228535,0.107036385387488,-1.42669416269718,0.153668068936224,0.273153433140532,-0.19817271413546
"Smpdl3a",8472.76935137726,1.10170023343887,0.378221845940338,2.91284135293617,0.0035815651050148,0.0154703935351304,4.59279787309933
"Reep3",5391.37728017966,0.121075561615635,0.207687870123279,0.582968863534335,0.559914260999832,0.688348566265745,0.0452168710480131
"Fabp7",332.221060209876,1.22426356118655,0.453233742868963,2.70117479214364,0.00690950153822212,0.0255154282182301,4.49117657231604
"Pkib",297.916725874967,-0.387318259532159,0.308563663121811,-1.25522965216827,0.209395404191284,0.343024920198014,-0.414412016456609
"Serinc1",7705.24841254919,-0.0286693650199537,0.142848748470343,-0.20

0697348257872,0.840935233313819,0.898239455056056,-0.00307675590147962
"Hsf2",1127.86085602406,0.384678078877245,0.118539320320576,3.24515171
705833,0.00117388062942254,0.00664249418790033,1.92887888709164
"Rspo3",385.007953129595,-1.31077585692675,0.500810721890579,-2.617307
89624176,0.00886263586968916,0.0308027939727628,-4.56169644081752
"Echdc1",5598.18879368375,-2.54633595608299,0.744817580780275,-3.41873
771751659,0.000629123369936436,0.00412130382311354,-13.9834221334042
"Mgat4c",32.0114464377298,2.29777889248207,0.496342693593373,4.6294202
012864,3.66690998928575e-06,7.83104172739251e-05,21.725108623855
"Ptprk",3880.00335583405,0.66950209527979,0.27307468691834,2.451717890
20488,0.0142176079395599,0.0444753601164027,2.08403947768968
"Nts",4.25080026085889,-3.37616375528992,1.41348560146352,-2.388537776
25626,0.0169155692087961,0.0505221716225503,-10.0790068236505
"Dcbld1",801.027048841094,0.806004960136097,0.197165924201722,4.087952
63887216,4.35196970269209e-05,0.000520261834844208,6.09434725019723
"Lrriq1",68.5388768372923,-0.0785367496126881,0.537762072753294,-0.146
043675431752,0.883886910021733,0.92780764315498,-0.00588482530769354
"Ros1",45.3365828237004,-1.21486500406794,1.03678177411985,-1.17176539
402351,0.241291260047272,0.379830393693235,-1.17602632664093
"Slc6a15",1261.06161800125,1.8656366259745,0.670553922051464,2.7822320
6907336,0.0053986421111898,0.0210701770453112,7.20116455988293
"Ccdc59",1528.11338633063,0.113821581049374,0.12153600825952,0.9365255
83482443,0.349002646707494,0.49398817470247,0.0802719529511407
"Lama2",1382.56565701621,-1.04732920599118,0.263146351789682,-3.980025
55942044,6.89078590556915e-05,0.000742711683730168,-7.54621915156413
"Lin7a",49.3162285993215,1.9026707048247,0.425325921713489,4.473441677
75975,7.69705589773262e-06,0.000138447597688183,16.9052647161525
"Ppp1r12a",6342.63051491988,-0.0909384355534218,0.233695279518562,-0.3
89132530792941,0.69717811012797,0.797943472534031,-0.0205263981880724
"Fam162b",0.0825764842869715,0,4.78471990596623,0,1,NA,NA
"Sim1",652.774009582998,1.19681478046614,0.49779293401865,2.4042422032
8788,0.0162060320846381,0.0490051509904272,3.60938975677735
"P4ha1",2607.95956960249,-0.0573963897089604,0.158617179455814,-0.3618
54812359398,0.717460529326743,0.812749558494819,-0.011900123367658
"Sept10",881.562888061468,-1.23969196391863,0.268341548788026,-4.61982
860841991,3.84057164209049e-06,8.12185096285036e-05,-11.6758743612653
"Lims1",2849.59300137075,-0.635669754463551,0.127248461099363,-4.99550
052685652,5.8683363696585e-07,1.7850362330047e-05,-6.95008683865642
"Zwint",3338.04811948802,-0.321459947842522,0.25028437465525,-1.284378
81224232,0.199009442770084,0.33036185054892,-0.35603833632793
"Ube2d1",1670.25641051752,0.0213297440081422,0.0797772023548309,0.2673
66407677125,0.789187061040307,0.864081834914865,0.0031160153410225
"Dcn",14693.7527814593,-1.69505496894872,0.39486895362286,-4.292702562
19655,1.76511392865003e-05,0.000260926394092633,-13.9863599518881
"Mrln",17.3685440388228,1.62330098996105,0.519484068137929,3.124833059
42319,0.00177905993646907,0.0091428863050274,7.62103965007445
"Slc17a8",23.515215813102,0.286660395063299,0.384471008596093,0.745596
90757971,0.455910951541524,0.596080717035918,0.148312122950888
"Epyc",38.1935727744808,-5.83652722489871,1.44581209158476,-4.03685047
238833,5.41735724088025e-05,0.000614187169781651,-43.162349398564

"Cdk1",248.638186971673,-0.733503471769622,0.351323682371185,-2.087828
14417461,0.0368133398296732,0.0919885300524117,-1.75020631342588
"Atp2b1",7657.70002286835,0.541970207455098,0.281854294367222,1.922873
69142929,0.0544959145098767,0.124102001998783,1.13090292182277
"Rhobtb1",1919.65835631782,0.566762110156609,0.275754169510862,2.05531
655663428,0.0398484391776439,0.0976209155780501,1.31866472030623
"Cabcoco1",342.147068461224,1.96467359443308,0.448086511032665,4.38458
54451304,1.16206864257404e-05,0.000191420081543051,16.819649553462
"Arid5b",2747.89659114527,-0.369340021872007,0.147293568843464,-2.5075
0949122919,0.0121585316215128,0.0393537598864969,-1.19487545344797
"Actr6",488.744472770628,0.0257773329144534,0.25710078271879,0.1002615
88634088,0.920136651615194,0.950948988554825,0.00129646729005879
"Uhrf1bp1l",4154.07345588154,0.562403775437795,0.200351475699556,2.807
0857650241,0.00499919297751019,0.0198848084546076,2.20338508006127
"Poc1b",732.041913505507,-0.0344595394035692,0.120694752690136,-0.2855
09838957444,0.77525356757074,0.854681333285893,-0.00541106393539575
"Dusp6",2315.83205108691,-0.513929819624939,0.25027515015844,-2.053459
23995886,0.0400280589730824,0.0979706534452973,-1.1939038369576
"Tmpo",4159.92513092248,-0.318146844872485,0.149818033250528,-2.123555
07524568,0.033707365512132,0.0859793580266288,-0.780620380598953
"Kitl",1415.78497546626,-0.522661200079235,0.269099475818158,-1.942260
19389357,0.0521056093169796,0.120001462763557,-1.10817311327211
"Psen1",2756.43872011621,0.243763096805025,0.12469690168547,1.95484485
58881,0.0506013849379265,0.117484395634833,0.522006424497873
"Sgk1",5225.66268498313,1.0064954938899,0.282830239217191,3.5586558802
0448,0.000372757536780104,0.00273995300565272,5.93813671905178
"Cep290",939.71190741501,0.241004672987619,0.145581095733649,1.6554668
1575027,0.0978298423602432,0.194007960368188,0.395212980219512
"Ikbip",1523.34917333235,-0.0849247191845358,0.151094521319626,-0.5620
63524493293,0.574072738948902,0.699885591447864,-0.0303044007516622
"Hbs1l",2603.37307464168,0.111828079208054,0.0964404734742034,1.159555
47686072,0.246229837151938,0.385312148056861,0.106650606773307
"Epb41l2",3934.60870050772,-1.34447011854846,0.272071151252499,-4.9416
1219357179,7.74792103752206e-07,2.25476066253142e-05,-14.385671128916
"Apaf1",897.734027886504,-0.443213382531767,0.438991245312013,-1.00961
781644815,0.312678430361112,0.457737370311657,-0.346353391013468
"Myb",37.8204740513367,-0.754089930914188,0.448690489051709,-1.6806461
231392,0.092831671290717,0.186469748840123,-1.26648367601427
"Med23",1117.38608353004,-0.312352653096673,0.144020830355565,-2.16880
19179276,0.0300977255782972,0.0788982862766387,-0.793249477040136
"Ahi1",749.890815030738,0.238231064201415,0.191396009797501,1.24470235
535979,0.213241179867442,0.347669247015251,0.251691998585605
"Arg1",16.8425525120395,-0.119523188042559,0.458682421874797,-0.260579
39511618,0.794416881161874,0.868150451120626,-0.0168994132657461
"Nedd1",870.200891519689,-0.0632361765527445,0.117719707589179,-0.5371
75786856587,0.591146194836473,0.714358134919216,-0.0212708066395259
"Enpp3",1606.25551088308,1.24039496948901,0.315284427509766,3.93420943
522681,8.34709775866617e-05,0.000858788192022722,8.75717389934055
"Pde7b",497.637940251351,-0.157327445962752,0.230259835040361,-0.68326
0482381457,0.49444226029372,0.630395291388672,-0.0725921751799548

"Mtfr2",59.1648288055146,0.164836207357645,0.253264387613059,0.6508463
70116133,0.515145663725493,0.64885724491729,0.0712986731462842
"Map7",3608.43088975925,1.08478148620264,0.325599130142288,3.331647371
81142,0.000863335750339946,0.00526370245782623,5.69176233787348
"Ccn2",8405.67150006053,-0.153565959448693,0.374903440172885,-0.409614
698061659,0.68208861323492,0.786153796822987,-0.0369484052697449
"Stx7",6429.90256488478,0.293129236580567,0.194679793981262,1.50569933
62587,0.132144374446254,0.243024996505666,0.414657972473942
"Moxd1",51.5523746613859,-1.75952963533747,0.612333513753685,-2.873482
49902463,0.0040597357009529,0.0169827776651319,-7.17106073014684
"Pex7",2166.52098775507,0.373006800271594,0.358675630625223,1.03995579
410118,0.298360438929845,0.44269323782468,0.303955115293112
"Il20ra",4.64050495835498,-0.944603440847644,0.749089592323766,-1.2610
0195561037,0.207308142566577,0.340358215204035,-1.01805263229936
"Ifngr1",3965.83617026443,-1.19610946138763,0.176654423325469,-6.77090
014997195,1.27983583933086e-11,4.79889215293712e-09,-22.9113340934092
"Vnn3",168.225118322767,-3.51936988807218,0.83184248283801,-4.23081287
705467,2.32848317725334e-05,0.000323828566263094,-28.279179835296
"Cfap54",40.4129029913575,0.238406866810013,0.554101546730359,0.430258
439480641,0.667007655730552,0.774569897397634,0.0609004082566383
"Cdk17",1363.94423168451,-0.813730857233254,0.180198409424893,-4.51574
938885583,6.3093245312101e-06,0.000118744410916539,-7.35493660934371
"Hal",14.0306207674343,0.873120297034063,0.818646143845158,1.066541757
50837,0.286178812821289,0.430250172903809,0.736379567013635
"Snrpf",1136.94982320609,-0.143395100208544,0.200292672527498,-0.71592
7838991997,0.4740359075653,0.611697691972748,-0.0704811417834967
"Ntn4",4008.88496008652,0.981078624500264,0.308430551022117,3.18087368
857929,0.00146831633882091,0.00791518716459221,4.74741193401471
"Usp44",0.476823137666417,-0.0831927920379075,2.49932039745733,-0.0332
861653602088,0.973446386090589,NA,NA
"Fgd6",660.675572726745,-0.655874442564926,0.261521748888651,-2.507915
48065159,0.0121445712203478,0.0393151003908917,-2.12250584736022
"Ndufa12",4712.68314314497,0.471267588809135,0.405252316290087,1.16289
918617465,0.244870407666493,0.384022325100759,0.451028808894303
"Tmcc3",2604.51747457516,-0.348227021816,0.261052372067923,-1.33393548
220813,0.182225001768352,0.310312656117172,-0.407486527968609
"Cep83",887.434304913525,0.0395055012400728,0.158330671529394,0.249512
623539519,0.802964277458341,0.874452495636881,0.00529995168731354
"Socs2",2301.74479436256,0.594358962502691,0.355374405069232,1.6724866
9016245,0.0944283902785013,0.188992481384748,0.990230587949738
"Nudt4",18482.3292859623,0.827774838264331,0.266407515955169,3.1071752
4352289,0.00188884391996265,0.0095582061522981,3.84944703224217
"Nuak1",964.516389719483,-0.523267938023764,0.242897615095854,-2.15427
367542192,0.0312187160378463,0.0810738579256695,-1.31465560124954
"Tcp11l2",1121.08670561117,-0.348022947530742,0.151699304256984,-2.294
16310928611,0.021781131449131,0.0615312229781449,-0.970361251267342
"Rfx4",2.73968309601108,-0.175599725141846,1.42679864635961,-0.1230725
34158816,0.902049660473301,0.940001935145635,-0.0108649423852009
"Cry1",350.876515499085,-0.903848891909155,0.218066438968188,-4.144832
63076081,3.40061997285427e-05,0.000429160441622735,-7.00815482237803

"Btbd11",253.407822934952,-0.255570482232064,0.27685377327365,-0.92312
4432114754,0.355942363652134,0.500986371651578,-0.176644280887009
"Timp3",58257.077358388,1.06725936848604,0.260049692635463,4.104059334
46774,4.05963272015602e-05,0.000493903626034222,8.12522716519385
"Hsp90b1",31075.1253518324,0.135460316801884,0.134311717403602,1.00855
174381272,0.313189660236061,0.458331866180692,0.105680958978704
"Pah",17796.9946693161,2.46561243975321,0.916366453949898,2.6906402227
2472,0.0071315057691143,0.0261323246544241,8.98612730481613
"Ascl1",9.69450073384086,-2.2877274961835,1.07875660886791,-2.12070774
57299,0.0339464042904445,0.0864759590874166,-5.600102591539
"Igf1",3352.7765810068,-1.88185532230348,0.445957137947859,-4.21981208
993116,2.44506030869573e-05,0.000335967483431638,-15.0520126033418
"Washc3",1185.78811266362,0.0786773028475025,0.255533416134596,0.30789
4380459662,0.758162701963684,0.843181403313251,0.013420235878419
"Dram1",1346.82096338611,-1.72013190595719,0.412540296623819,-4.169609
4176364,3.05122101001397e-05,0.000396516438679571,-13.4734372523066
"Sycp3",77.2393606229345,-3.18757285988758,0.91005250669231,-3.5026252
18267,0.000460697184021967,0.00323933418465933,-18.27240271809
"Mybpc1",5.53045026738124,-2.22097415269265,1.26959239251938,-1.749360
00387128,0.080228809886107,0.166627751934311,-3.9799701012358
"Slc5a8",6901.68271613059,2.14983996191889,0.807486168403525,2.6623861
1389384,0.0077588820256592,0.0278195442692724,7.70076216011604
"Sirt1",1515.57249424259,0.361893329208786,0.101356674563468,3.5704933
1745958,0.000356309544916245,0.00264257265377594,2.14819965128081
"Herc4",2602.23535719522,0.806093721027524,0.186666418005568,4.3183649
7234055,1.57189342322785e-05,0.00024000609213858,6.71866722868806
"Mypn",0.881196202938074,-3.31922066815453,1.92955976111496,-1.7201958
3691805,0.0853968495259048,NA,NA
"Hnrnph3",4129.77601018415,0.0356974839143319,0.141728506380757,0.2518
72293202821,0.801139776117769,0.873096823691072,0.00484446342394221
"Rufy2",873.805236159182,0.0446535508848905,0.156121111750744,0.286018
658105525,0.774863831144808,0.854443956493554,0.00702419846215129
"Pbld2",718.781731341479,1.55250195544475,0.499542410411433,3.10784814
880098,0.00188454871655388,0.00954165953657949,7.22237548873632
"Ccar1",8561.6261316634,0.853090566673217,0.278655391375149,3.06145365
594135,0.00220265087023031,0.0107691290541,3.86541448908991
"Ddx21",2780.92884484718,-0.272210941520589,0.155354867585235,-1.75218
804374598,0.0797414788580564,0.165951473452277,-0.488907146052197
"Ddx50",4053.53722294141,0.124273290902358,0.143864581153789,0.8638213
09635007,0.387686059735509,0.531956565286626,0.0784404856332897
"Srgn",2438.82438193223,-0.996307434741079,0.265872492589167,-3.747312
95080081,0.000178739030979614,0.00155196078648198,-6.44435173998442
"Vps26a",2868.4363268434,0.0713256624286686,0.16103060346268,0.4429323
42641309,0.657814672758246,0.767155361531209,0.0189060038091187
"Supv3l1",1028.35818506198,0.808586536775082,0.22522376901151,3.590147
43569876,0.000330490974004215,0.00249764070198999,4.84538100729226
"Hkdc1",151.155206893769,1.18591219276991,0.493365970967944,2.40371704
283383,0.0162293297587227,0.0490527781174354,3.57535740255833
"Tacr2",0.792339290665846,0.309101467168361,2.49846685248265,0.1237164
57098967,0.901539780888116,NA,NA

"Fam241b",323.277551677549,0.1015176929032З,0.195935322076384,0.518118
386350237,0.604375672656677,0.725533608266216,0.0325717369006622
"Aifm2",2294.892986328,-1.94192103286756,0.630979974436387,-3.07762704
292185,0.0020865587691984,0.010306752365993,-8.88420333720692
"Macroh2a2",586.555829648282,-0.0230588428823005,0.285791122129102,-0.
0806842518777892,0.935693061160815,0.959820915915004,-0.00094560989215
4088
"Tysnd1",2601.18757959084,0.374746771584055,0.307379486590359,1.219166
49592,0.222781005555725,0.358753224836928,0.384160606424929
"Sar1a",7447.15981045031,-0.358564281336482,0.16287608851316,-2.201454
39769393,0.0277038716398589,0.0740364568469885,-0.933413693473616
"Ppa1",2612.70737133551,-1.60801907948484,0.54382842187224,-2.95685001
888815,0.00310799254703952,0.013889442741732,-6.87689659361512
"Npffr1",8.16724879652471,-1.97315146484747,0.910263987404491,-2.16766
948066755,0.0301838431329613,0.0790284105554558,-5.00775554532381
"Eif4ebp2",9190.81027545635,-0.224543328427513,0.258503019993328,-0.86
8629420396359,0.385049857197014,0.529643290731635,-0.142708857141895
"Pald1",1267.56037046727,-0.798431314984797,0.2111399520259,-3.7815264
5827473,0.000155869651867007,0.00140182032846075,-5.24568068694977
"Tbata",1.96901944969045,-1.79439898464115,1.38988880048392,-1.2910378
0389941,0.196690569077795,0.327643974017842,-2.00224010122324
"Sgpl1",4639.27427491163,-0.676124630679696,0.206704957827045,-3.27096
474988967,0.00107181269358473,0.00621046178291679,-3.43574084993983
"Pcbd1",6460.26313934828,1.88119761411863,0.561022341798039,3.35315989
037,0.000798945382114974,0.00495793668379305,9.98307481543901
"Unc5b",1624.08599973466,-1.35373209834534,0.177773535107479,-7.614924
78352812,2.63845069944692e-14,3.42963411585441e-11,-32.619504869123
"Slc29a3",3162.77377087626,0.449398732624478,0.293863377727137,1.52927
777561232,0.126195596728728,0.235177928866578,0.650465526086231
"Vsir",1711.81907530698,-1.51484078123754,0.252174080486286,-6.0071232
472281,1.88843965970486e-09,2.4756458072675e-07,-23.0431434792405
"Slc16a7",3783.17191492554,1.08772877079342,0.386427366549933,2.814833
69178738,0.00488024821741057,0.0195149876421393,4.28192317918085
"Lrig3",463.49102624995,0.443061756119737,0.139401176673706,3.17832149
406317,0.00148130399732935,0.00796757664549728,2.141040505857
"Anapc16",2496.56485823178,0.389764892348273,0.146860546162024,2.65397
959175683,0.00795486378644556,0.0283657523972413,1.38856578945548
"Ddit4",3281.15716483881,-0.190690978334628,0.252574942706946,-0.75498
7712917668,0.450256354663953,0.590906535857694,-0.100322031688472
"Dnajb12",2656.69741862979,0.652747157019673,0.156891220715207,4.16050
786044017,3.17540687110983e-05,0.000407329494558549,5.09527128559301
"Micu1",2375.87954662438,-0.200820423611063,0.146205402121322,-1.37354
995572887,0.169581442295995,0.294015557699387,-0.245828818220081
"Cand1",2754.81189985564,-0.212098917591405,0.182389367778499,-1.16289
079881557,0.244873811056402,0.384022325100759,-0.202990242572709
"Tbk1",2117.36724494139,-0.0118941254498833,0.265519078336886,-0.04479
57469737533,0.964270115114977,0.978482952401665,-0.000258719999515495
"Pno1",1743.99711991424,0.217320571518774,0.175983692190076,1.23489039
70264,0.216871297867093,0.352262293053364,0.226747770607281
"Plek",974.553246433903,-1.39106513978762,0.389730628488753,-3.5692989

9295241,0.000357937730253217,0.00265061521628455,-8.25313871277904
"Srgap1",1062.76844005477,-0.748991459535995,0.312782826614633,-2.3946
0544443124,0.0166382586141878,0.0499534670756096,-2.24447526776898
"Egfr",2045.8512467421,0.514076328734156,0.164063283365338,3.133402661
39502,0.00172792219414061,0.00895561588021094,2.42411380420123
"Avpr1a",652.694298938241,-1.30858253269187,0.302785520925939,-4.32181
343642237,1.54752016795753e-05,0.000237586993975086,-10.9200907599132
"Usp15",2644.29309762966,0.00664462306407063,0.145727989361526,0.04559
60662957235,0.96363220455447,0.978160984057015,0.00014672003562058
"Elane",3.44564089653987,-1.02940204351824,1.49581199316388,-0.6881894
57112783,0.491333482224558,0.627745248438138,-0.479311054667241
"Vps54",3004.27801075855,0.00757856832706495,0.093814303283805,0.08078
26531966926,0.935614803720466,0.959820915915004,0.000310786157615005
"Tbc1d15",1649.03648177436,-0.410373793468491,0.180058419106899,-2.279
11472012234,0.0226602467096616,0.0633940843275661,-1.13196880509699
"Pcsk4",387.616136793588,0.99794863982775,0.318118896054709,3.13703037
51341,0.00170668444781157,0.00888714797334867,4.71346021388578
"Rab21",4435.22784440215,0.158694962781701,0.132258871611339,1.1998814
2079458,0.230185396576219,0.366979138384555,0.159083809269215
"2310011J03Rik",1443.46096630617,-0.0949653381503064,0.199940886382772
,-0.474967075861123,0.634810440631731,0.749472235630487,-0.02738667457
57904
"Peli1",2057.05084939743,-0.192922227913734,0.155861762921241,-1.23777
778653268,0.215798467191432,0.35092898359454,-0.202022639867577
"Apc2",132.637664011076,0.0696321595124852,0.27257649268523,0.25545915
1398269,0.79836848959423,0.871248044445531,0.00959730037617695
"Thap2",1642.28478184678,0.412606658164989,0.38750165526896,1.06478682
75004,0.286972407401207,0.431011246303246,0.347258467741155
"Lgr5",86.3095850965161,-0.539973041110653,0.307041332707963,-1.758633
06854598,0.0786398524684135,0.164114293420649,-0.975835058810459
"Slc1a4",871.760280439621,0.739283663729592,0.290545196265758,2.544470
44119559,0.0109443574246338,0.0363655557371354,2.45008455140494
"Dock2",1086.04458287618,-1.45215302770649,0.422730640795921,-3.435173
34104849,0.000592174912622914,0.00392062018550818,-8.04711388402236
"Rab1a",9630.42052201129,0.219901137725894,0.195260530703077,1.1261934
8587292,0.260083630198351,0.401513113349759,0.200663105434698
"Gamt",710.234650559434,1.03415468189034,0.184593161749641,5.602345569
51215,2.11470419785813e-08,1.4267301885757e-06,13.9198516116081
"Ptprr",177.906881544686,-1.00238259640117,0.303648817589749,-3.301124
6490525,0.000962980895574102,0.00570471671332131,-5.17877154641595
"Actr2",9524.22119966596,-0.121714462919273,0.131233789731445,-0.92746
2836883306,0.353686278460373,0.498638832785275,-0.0846978361183712
"Ndufs7",5574.35355535001,0.62851163119713,0.322580240723321,1.9483884
995182,0.0513684948602357,0.118770599889683,1.33908260727665
"Ptprb",3778.0131597574,-0.695107195359024,0.229147025975306,-3.033455
01605564,0.00241770753502151,0.0115427182952618,-3.10136011514975
"Kcnmb1",118.217024477653,-1.21149917579201,0.266019192256546,-4.55417
95895074,5.25904144594432e-06,0.000103263635561956,-11.1194123513996
"Pwwp3a",3373.77858483167,0.00459189639073881,0.238671389973001,0.0192
394085912779,0.984650119903959,0.990083442725357,4.57630865850839e-05

"Gabrp",688.103565834474,-0.400415125429044,0.673241592858584,-0.59475
6963438461,0.55200593886661,0.681893803599922,-0.153311482367154
"Meis1",894.364826289196,-0.979210929528355,0.17947429710653,-5.455995
34482158,4.86992687313448e-08,2.66724253293191e-06,-12.5676487731991
"Uqcr11",8230.59218289078,0.600791955714506,0.415443455286165,1.446146
15555965,0.148136212292144,0.265376687547981,0.797013612333792
"Cnot2",2359.98142782369,-0.186344295818164,0.0808004140936424,-2.3062
2946563371,0.0210978139649695,0.0599482915606203,-0.524423700980502
"Tcf3",3124.71156762952,-0.154159461860155,0.172160777437899,-0.895438
927230467,0.370552499377409,0.515716403431358,-0.102084129000704
"Best3",8.10724940277305,0.18522306352235,0.694943364083339,0.26652972
471601,0.789831270037944,0.864592976824603,0.0269492944620526
"Frs2",1581.6346914161,-0.124116011131099,0.10679930993477,-1.16214244
461791,0.245177608932752,0.384344188693584,-0.11868181538111
"Yeats4",1583.25862716725,0.15396272847802,0.111291171612214,1.3834226
5830836,0.166535306949702,0.289587263650086,0.190805793631206
"Cobl",1520.98187277388,0.762263696247411,0.427979520803734,1.78107516
643764,0.0749001720395825,0.158052332730279,1.40624623421592
"Rab36",561.377322512199,0.318368813375793,0.265141761419908,1.2007494
0918715,0.229848421673339,0.366561796645408,0.319511171273704
"Grb10",2398.89581136404,-0.451993366900764,0.184020456636802,-2.45621
261440979,0.0140410057416177,0.0440643054804541,-1.41117085192662
"Adora2a",745.091098430726,-1.7568169147561,0.287247694476546,-6.11603
4866555,9.59323444865566e-10,1.52072264455194e-07,-27.5801106400722
"Snrpd3",3463.64699069022,0.370961295592671,0.204971180102731,1.809821
72911697,0.0703234371255474,0.150578341431355,0.702330555814209
"Nav3",446.216909311531,-0.988684162837774,0.454480801088372,-2.175414
58400468,0.0295990625936826,0.0778527616958889,-2.52404729974702
"Ddc",1832.69311227291,1.47338056235468,0.690314058335303,2.1343626782
1019,0.0328131041951132,0.0842383022509963,3.64529904879523
"Cpm",1741.88583392118,-0.205005198389509,0.431551831324367,-0.4750418
91863555,0.634757114535821,0.749472235630487,-0.0591206303688682
"Mdm2",3213.58079182029,0.114938386208048,0.140323718819209,0.81909449
9313637,0.412732500027422,0.55618621090156,0.0674288489852467
"E2f7",76.2353500971066,-0.137496519144603,0.418255143559714,-0.328738
381970365,0.742353440624983,0.832101603064439,-0.0252719601167392
"Csrp2",5593.36119340574,1.42867766089979,0.23388737095103,6.108400188
90513,1.00634762554276e-09,1.56974128022662e-07,22.3833569827268
"Osbpl8",4731.91919000265,0.77963160637234,0.239398262284465,3.2566301
815756,0.00112743217919774,0.00645031473884905,3.93217051525386
"Mknk2",10089.0215248477,-0.946844012532097,0.415647476572082,-2.27799
774063561,0.0227267122120547,0.0635032150631473,-2.61013150689348
"Spata48",16.3942736827443,-0.884431972685696,0.644517392805344,-1.372
23910876337,0.169989014358764,0.294412485406923,-1.08145996403268
"Zpbp",33.3917008199443,-0.384630648969617,0.403723778208347,-0.952707
444373323,0.340738320454502,0.485374959860049,-0.278023946522008
"Cabin1",3210.15578275643,-0.0337609004610678,0.18015773471094,-0.1873
96341962428,0.851349890492321,0.905400903502742,-0.00335507206505602
"Ap3d1",7711.35240197909,-0.0256761412800455,0.170117319831389,-0.1509
31964514221,0.880029385281767,0.925103135336634,-0.00199888888307196

"Phlda1",1433.1017464221,-0.342038006856421,0.277309750690076,-1.23341
500255678,0.21742097051843,0.352802437014677,-0.356351287485639
"Sf3a2",1720.88658148184,-0.101850085019922,0.150215808803687,-0.67802
5074930877,0.497755794684954,0.63337743814966,-0.04651378961793
"Mdm1",349.163096979937,0.171945866409655,0.125909538764642,1.36563018
256358,0.172055068127066,0.297299691451749,0.208572844097163
"Jsrp1",35.9001671407441,-1.491370400991,0.626423215469134,-2.38077128
07612,0.0172764350026834,0.0514253065148257,-4.42582791180496
"Wif1",87.7066252203659,0.138548031124065,0.540224751061962,0.25646368
6367036,0.79759281702843,0.870693955905063,0.0191840163897017
"Timm13",4063.80663206274,0.624095960786556,0.256740502790213,2.430843
41583812,0.0150637230803117,0.0462830873967723,1.91783357405058
"Vps13d",3204.06114691627,0.209399667031429,0.132896809716697,1.575656
08593477,0.115105083019681,0.219343130249975,0.317683997876224
"Llph",2518.06244633669,0.294702461991108,0.137448783462532,2.14408927
141537,0.0320257345398321,0.082597588896514,0.734921569968503
"Tmbim4",8562.33316233588,1.42088442137773,0.261587272310383,5.4317796
4595999,5.57947941476066e-08,2.97236856909845e-06,18.0823903038329
"Slc5a4b",4.47901767348701,2.53633056626031,0.687032131525846,3.691720
44490454,0.000222742217463069,0.00184182686857291,15.9712668204003
"Irak3",308.84019992766,-1.38180986885032,0.232856964584228,-5.9341573
5414046,2.95358462272876e-09,3.25361542225792e-07,-20.6419302087476
"Helb",818.695794079076,0.219932339061005,0.104974204652704,2.09510841
057218,0.0361613564599436,0.0906731132016435,0.527946348821169
"Prmt2",1055.18167185358,-0.672676270937672,0.149193128693332,-4.50876
17427768,6.52070870176302e-06,0.000121549501211257,-6.06430369193528
"Dip2a",1422.34519092315,-0.233440768297111,0.174759986764674,-1.33577
927429953,0.181621429189199,0.309604356210089,-0.273699978819378
"Hmg20b",4502.87555414134,0.537638915553015,0.244743698251582,2.196742
63073508,0.0280388323779897,0.0746655495364713,1.39503130589403
"4930404N11Rik",24.9570920155612,-0.701912066351259,0.516715857570194,
-1.35841015147461,0.174333574880766,0.300358402670776,-0.8442449822210
16
"Fzr1",1345.15137220325,-0.296064430578385,0.100543870571307,-2.944629
3334054,0.0032334177529717,0.0142894785465644,-1.25775032246643
"Ncln",3165.3602486975,-0.0511676238393806,0.0833292401479435,-0.61404
1646708131,0.53918780700759,0.670178100403773,-0.0204778858367847
"Col6a2",2782.71934420822,-2.0449508864107,0.350175498012852,-5.839788
61460962,5.22670966974482e-09,4.94710607479051e-07,-29.6912408382576
"Hcfc2",861.453192265237,-0.0335581006232084,0.117388551365534,-0.2858
72005684033,0.774976155598067,0.854443956493554,-0.00527883570553893
"Nfyb",960.322439823721,-0.0165629685500713,0.084744452349026,-0.19544
6050932697,0.845043768242724,0.90075789378945,-0.00173114103113821
"Txnrd1",5448.70665224274,0.117324673032712,0.161441983558937,0.726729
630337336,0.467391598362826,0.605769834145066,0.0588095997124388
"Glt8d2",29.0303871443468,-1.62016777498643,0.859877131927079,-1.88418
521068871,0.0595399273131652,0.132795263079857,-3.27103240244893
"Ppm1m",2954.94449764547,-1.09086815170203,0.284561642835875,-3.833503
84412563,0.000126330825841661,0.00119456762476271,-7.34151100325585
"D10Wsu102e",6580.00589529916,0.449475135535362,0.136510290926558,3.29

260990130904,0.000992620946354847,0.00583834787693117,2.31178879968246
"Aldh1l2",105.259137871768,0.49841683337038,0.248015960032741,2.009615
96707156,0.0444718500011215,0.106322536162412,1.11709068885227
"Wdr82",3267.29104154518,-0.170625953606582,0.0646702504837962,-2.6383
9945461993,0.00832983976983518,0.0293771536893514,-0.601889533789106
"Glyctk",5297.70223940503,1.63933543654657,0.546035386927594,3.0022512
7490492,0.00267990869535689,0.012435437468539,7.19210065983555
"Pofut2",2339.40793515391,-0.248810845873851,0.106900725499694,-2.3274
9445535393,0.0199389637555837,0.0573322393904101,-0.711323379432806
"Slc36a1",4280.47089226137,1.00358293602534,0.256176740465038,3.917541
20301295,8.94567590186447e-05,0.000906092409010667,7.03147263325208
"Adarb1",840.329567657053,-0.569047157369254,0.247865489385525,-2.2957
901835385,0.0216878809922516,0.0613155240548429,-1.58862159395489
"Appl2",3318.77578708128,0.0491613713484433,0.191575607637534,0.256616
027242142,0.797475201284114,0.870614304291022,0.00681161376519814
"Slc36a2",2279.97237488021,-3.63210955117652,1.16067598446317,-3.12930
533568023,0.00175220128811657,0.00903581611100153,-17.0947380107302
"Sumo3",4460.86878976882,-0.474761339878937,0.198868825058982,-2.38730
902009467,0.0169722183909958,0.0506542651442885,-1.41608576659242
"Hint1",10552.8638575895,0.812280255422087,0.286102388548806,2.8391243
412619,0.00452375254222722,0.0183873519008435,3.24594683142934
"Lyrm7",205.967145445328,-0.452220304946726,0.361853101455451,-1.24973
450034779,0.211396549965329,0.345325899063672,-0.480830779045163
"Fbxw11",3085.43309792434,-0.186935254363077,0.22171626664722,-0.84312
8279173651,0.399156704473142,0.543412751278964,-0.114009216326577
"Stk10",868.228674679265,-1.06289383949226,0.219805203106225,-4.835617
28508582,1.32733083889386e-06,3.42785386711952e-05,-10.927602098047
"Papolg",533.049232310359,-0.0672842135551669,0.120095003598214,-0.560
258225065472,0.575303317292159,0.700967946056096,-0.0239056180947258
"Rel",498.722644971526,-1.13070523049397,0.246530798142591,-4.58646643
345543,4.50810842325673e-06,9.16990218915506e-05,-10.5121652221121
"Pfkl",4346.25782104933,-1.57285302238161,0.546832552623011,-2.8762973
5069182,0.00402370469474668,0.0168646161088071,-6.42123156118956
"Il9r",84.9299189134189,-1.76304009625573,0.641212366188535,-2.7495416
327285,0.00596786810893631,0.0227401783052648,-6.67067720630591
"Pus10",915.088144883274,-0.345479758981293,0.245642851705964,-1.40643
115230902,0.159596135180034,0.28082352168038,-0.438769258810641
"Rhbdf1",3951.04858072352,0.0773701775437383,0.241832126836778,0.31993
3412304472,0.749018808629658,0.836582999401546,0.0138051252847948
"Pex13",2295.67429677125,-0.552956085599968,0.452629258741229,-1.22165
342810085,0.221838714964972,0.357719110422667,-0.568442841872948
"Cfap410",1300.09236608562,0.510478414900898,0.12084012942603 8,4.22441
135511482,2.39566122911914e-05,0.000330794246877897,4.09098115682623
"1700093K21Rik",13.540317254264,1.16551728287463,0.55948203649109,2.08
320769364539,0.0372322961336899,0.0927619869683983,2.77127182641355
"Mpg",1008.8166605056,0.37829449663052,0.123713788114675,3.05782000854
962,0.00222953447704036,0.0108704111887052,1.71053829235119
"Ahsa2",2209.79756088279,0.128715419717951,0.151647409132773,0.8487808
69083335,0.396003237325704,0.540364694616599,0.0792257576039236
"Nprl3",1211.25737401971,0.158985428989918,0.132868096062808,1.1965658

7022037,0.231475809922075,0.368446451693814,0.158740574286768
"Xpo1",4614.71346882998,-0.0592083893357016,0.114047515381296,-0.51915
5451460293,0.603652341982962,0.724800090992299,-0.0190567765903022
"Hbq1a",1.0140565730765,1.36805835846127,1.92625786124179,0.7102155874
28846,0.477570456213082,NA,NA
"Nsg2",122.770331588853,0.425999574513445,0.427118543903224,0.99738019
0100026,0.318580003203029,0.463661484097384,0.327423508612414
"4930524B15Rik",1.06208007786409,2.24749965058903,1.67708480521561,1.3
4012283910716,0.180205411979967,NA,NA
"Cpeb4",4203.55954909745,0.412291005839948,0.225799456227428,1.8259167
3482456,0.0678628050218498,0.146576151260015,0.791685387259298
"Stc2",1028.06264281888,-0.801681417151198,0.431299921931421,-1.858756
23060898,0.0630616990901514,0.138590735894925,-1.58430689511182
"Asb3",482.268901111598,-0.393503306930416,0.21301297449218,-1.8473208
3981514,0.0647006543523074,0.141284954481049,-0.770076714189623
"Cdc34",2458.06689275396,-0.021247908566072,0.302411083474727,-0.07026
16726937778,0.94398538784263,0.965526259883314,-0.000745418687664363
"Tpgs1",1545.27211406459,0.465731598295793,0.163771267616219,2.8437930
845549,0.00445799880721749,0.0181773578262783,1.86645595634694
"Chac2",1146.56239537464,1.31128615760575,0.25403919578785,5.161747397
04662,2.44655282529604e-07,9.08626418811851e-06,15.2223888740789
"Madcam1",21.027778143611,-1.32544435734742,0.634409077292127,-2.08925
818496303,0.036684488287611,0.0917370979391868,-3.16625915506559
"Erlec1",1876.38783389148,0.276944816673621,0.187090339439193,1.480273
20653631,0.138800350611738,0.252573890455219,0.381090313122351
"Shc2",198.165317931405,-1.51428129824471,0.254488152894758,-5.9503017
3711438,2.67648606138617e-09,3.09850777959792e-07,-22.6947988414285
"Sptbn1",20953.9915082458,0.031375857715637,0.0913570659319232,0.34344
2046825556,0.731265932137199,0.823936616284953,0.00607630112888533
"Theg",0.626665662477643,3.36054900158077,2.43572739091944,1.379690113
97135,0.167682078079119,NA,NA
"Wdpcp",425.262535391902,0.419941298621542,0.128988167264136,3.2556575
3455203,0.00113130117062041,0.00646296812914058,2.1172040208977
"Mdh1",37443.8622319938,0.798804609883023,0.435029421171175,1.83620824
479527,0.0663268637641779,0.143965400164081,1.54822894398413
"Prss57",7.1228618614429,-1.2466541857002,0.751593639932068,-1.6586811
2696227,0.0971800644048751,0.193010489645314,-2.05075952586465
"Fstl3",420.656209517659,-0.924677855479482,0.298849580219707,-3.09412
465896616,0.00197394527186037,0.00989662764482733,-4.26790725241741
"Ccng1",6296.30989732737,0.144330684634195,0.264342294550028,0.5459992
12422212,0.585066478584358,0.708963498163417,0.0496427175638693
"Fgf22",0.546878087091903,-0.767355092244241,3.80167138707703,-0.20184
6770568519,0.840036516368224,NA,NA
"Nudcd2",1285.76632805021,0.0539827811768811,0.18989544052463,0.284276
34190553,0.776198614983593,0.855191308976103,0.00844453087993158
"Polrmt",2016.85737121319,1.04511724813848,0.269688202696905,3.8752798
1456816,0.000106502245379801,0.00104089262176208,7.17752731506626
"Hmmr",126.393658439561,-0.256751145645135,0.443959159932878,-0.578321
54129662,0.563047053615289,0.691036714169779,-0.0948855502506717
"Hcn2",65.1042639572134,0.71251659604527,0.255525758948517,2.788433537

88385,0.00529636135830057,0.0208016907922234,2.75937798969042
"Meikin",0.196458446039387,0,4.78471990595362,0,1,NA,NA
"Acsl6",21.4076154427661,0.25350182375409,0.494026786863509,0.51313376
2166076,0.607857755992365,0.728079245275253,0.0804476334288939
"Slc22a4",1376.10402420662,1.19673809695878,0.325109293694205,3.681033
17921271,0.000232290771651677,0.0019037446862807,7.49628659367401
"Zfp354b",119.554377362354,0.221759915034666,0.244648248787306,0.90644
3909302047,0.364700940717166,0.509702954568364,0.149450030843705
"Cyfip2",4582.12976550677,1.00914800436748,0.366930143209834,2.7502455
7955269,0.00595506159196449,0.0227002523793008,3.82000790231403
"Mgat1",6243.30624108864,0.346224644715872,0.133352251099191,2.5963164
615672,0.00942292331109653,0.0323313009777889,1.18814584646812
"Ppp2ca",9052.67324591413,-0.318659814149202,0.246014453454965,-1.2952
8899491076,0.195220554163595,0.325807900162954,-0.357360199222743
"Sgcd",87.3932528718214,-2.19552919353983,0.75082111372948,-2.92417082
230705,0.00345375156541183,0.0150617866299262,-9.21155005426781
"Flt4",587.65947433626,-0.680026478728713,0.17123461313198,-3.97131436
390479,7.14771859543717e-05,0.000763232295585071,-4.88119478638203
"Hnrnpab",11982.7350216751,-0.3149921214772,0.1932071321448,-1.6303338
1832472,0.10303096378263,0.201838413727893,-0.504078059723105
"Phykpl",728.891724909755,0.569845791706482,0.134858411105884,4.225511
68320579,2.38398455200783e-05,0.000329603900453367,4.56880640454387
"Hspa4",7399.58990542058,-0.152864031643592,0.171689957434688,-0.89034
9289659192,0.373278362466845,0.518462851644006,-0.100414379821391
"Cnot6",3832.18639039071,0.010615532966655,0.121461738724387,0.0873981
64048541,0.93035502867315,0.956679177759194,0.000470132031869872
"Gfpt2",232.747268020601,-2.14510678572808,0.388646487277868,-5.519429
23954542,3.40102508917002e-08,1.9992397084915e-06,-28.1496863339316
"Zfp354a",460.636834520311,0.558942661716722,0.27880636954275,2.004770
05827881,0.044987634889668,0.107141676447874,1.24845614096031
"Mapk9",2349.61426504783,-0.052834687256957,0.12242092963547,-0.431582
143790949,0.666045138642327,0.773745716947575,-0.0135527307928767
"Canx",19151.9577265094,0.0165241834952032,0.218648023846644,0.0755743
555532567,0.939757739405451,0.962574372938369,0.000630297550047867
"Rack1",20786.8944775031,0.0538801678787383,0.138165876629827,0.389967
256698943,0.696560759292365,0.797506851713595,0.0121911882576744
"Rasgef1c",12.4267819809443,-1.7866702821147,0.549694192032332,-3.2502
9863515388,0.00115283878256031,0.00655336751672329,-8.98297838734376
"Rufy1",2208.66092191033,0.432253763635629,0.0950217581213758,4.548997
74726848,5.39020200302292e-06,0.000105197868167418,3.95930076022325
"Rnf130",3177.41302764752,0.116878148930489,0.280325083947426,0.416937
889698122,0.676723838454524,0.782282646717632,0.0286981628526666
"Ltc4s",678.405370031987,-0.860770990640271,0.51678208596812,-1.665636
2788345,0.0957858807015411,0.19092548578191,-1.4253266281669
"Rad50",998.842010538382,0.0358622503611175,0.176871171247606,0.202759
161417624,0.839323279320831,0.897124821651609,0.00389321565487742
"Mrnip",600.946373816372,1.10726014774289,0.253323128369498,4.37093981
457484,1.23712892684644e-05,0.000200345015080165,9.42884012988633
"Il13",0.718952545052701,-1.9738870190167,4.28078811078874,-0.46110364
9125257,0.644724243801788,NA,NA

"Clk4",5970.43989652589,0.297387703337673,0.178468183302295,1.66633456
919292,0.095646795046111,0.19075163624039,0.492707637319475
"Sar1b",3634.71728030476,0.521633092418556,0.262642334820539,1.9860967
6834842,0.0470225800510984,0.111025219888147,1.14654844028645
"Jade2",1561.6767520819,-0.123969836914677,0.155521847390199,-0.797121
684155671,0.425380363913843,0.568359236318242,-0.0700431564405642
"Pdlim4",701.870805213852,-1.45166452794428,0.315275397722808,-4.60443
326193372,4.13590619707621e-06,8.57892542878638e-05,-13.5928303628595
"Cdkl3",239.657842197616,-0.145491368576532,0.191769135209427,-0.75867
9797025962,0.448044125151893,0.588756190336407,-0.0770730514260457
"Ube2b",5586.33022804592,-0.138768929792622,0.168542487507374,-0.82334
6871432348,0.410310766377861,0.553803082018243,-0.0820049582842357
"Cdkn2aipnl",1119.91643138913,-0.467541356577911,0.14355285325975,-3.2
5692834354135,0.00112624860368975,0.00644543448040588,-2.3584577790615
1
"Kremen1",1721.901990485,-0.667304424246251,0.129460757686229,-5.15449
187979866,2.54319580243411e-07,9.30842440287515e-06,-7.73044863924873
"Itk",251.210652490032,-1.05433044832537,0.433016378448957,-2.43485119
916695,0.0148979147457661,0.0459765023287349,-3.24694222973961
"Nefh",38.279196724003,-1.04359368512093,0.569859402219074,-1.83131783
218299,0.0670531175484215,0.145202319631177,-2.01374676191187
"Med7",1246.62869562708,0.376785069620294,0.128666788821344,2.92837858
993642,0.00340734875945169,0.0148996541937024,1.58491522572863
"Havcr2",245.756833888351,-1.17635232067942,0.451059472220951,-2.60797
609434347,0.00910793148909543,0.031453497728371,-4.06929098529279
"Tnip1",1530.59377365148,-0.736273094738016,0.164657873399793,-4.47153
2879271,7.76609167232784e-06,0.000139344045066026,-6.537048189665
"Fam71b",14.73695289779,0.014333742186445,0.770616555630724,0.01860035
58860909,0.985159918929802,0.99044282248599,0.000137648938958968
"Vdac1",24849.0496143184,0.953751012095,0.236939099400452,4.0253002333
0198,5.69026688440479e-05,0.00063947448825432,7.01470882264706
"Fabp6",2.94710416388412,-2.26170929316465,1.3031506236549,-1.73557012
682181,0.0826398718621689,0.17043708711324,-4.00184363700575
"Upp1",456.759730851735,0.344985612551334,0.1999093383914,1.7257103411
342,0.084399536689503,0.173259861694244,0.604746918006564
"Slu7",2864.81364972676,0.197752572416528,0.207869757983965,0.95132920
8897152,0.34143728394677,0.485937530101761,0.142713127689951
"Nipal4",214.438162232941,-3.19718336153655,0.812684935061119,-3.93409
945675455,8.3509201562985e-05,0.000858788192022722,-22.5720769381933
"Ascc2",3051.51098626249,0.0509924430509317,0.136289941104914,0.374146
783229427,0.70829511410649,0.806418509783654,0.0109711479487699
"Hus1",1182.21072936501,0.722176614057027,0.195367390760243,3.69650539
553599,0.000218587615757173,0.00181671838535096,4.55745692253263
"Pttg1",2771.48290657315,0.347154709666643,0.182329310424251,1.9039983
6898889,0.0569103966010517,0.12841556682413,0.71252936843255
"Hormad2",3.80163450152568,-1.69332456517353,1.07601055428721,-1.57370
627864822,0.115555369628302,0.219943244534619,-2.56434658366559
"Zfp607a",145.278469305046,-0.189192453506426,0.203582023108212,-0.929
318073462029,0.352724271475393,0.497750441139699,-0.131991335436687
"Tns3",8574.06674047697,-0.580951608282841,0.276172480486507,-2.103582

5410969,0.0354148782232375,0.0892374380455782,-1.40384319691257
"Btg2",3344.87391702263,-0.757824086341113,0.174684879387821,-4.338235
16378114,1.43631424831639e-05,0.000224581036196255,-6.36668775745872
"Castor1",377.032150698665,0.0707456198841567,0.218764152087203,0.3233
87626396653,0.746401699250162,0.835055102821876,0.0127524331680252
"Igfbp3",7395.05948598346,0.238662355179668,0.18029537685144,1.3237297
5584571,0.185592812426546,0.314070691728827,0.276403748316911
"Igfbp1",203.372333800954,1.04565685328451,0.598757106738306,1.7463790
2668189,0.0807451119186826,0.167485977892604,1.86843785133308
"Pes1",2573.09683733017,-0.10990221131527,0.143691753320368,-0.7648470
33846383,0.444362636747393,0.585576013199558,-0.0588151880844916
"Adcy1",869.414835345106,0.189499507541096,0.515349859944625,0.3677104
08539662,0.713089178616568,0.809726446023286,0.0399955404274262
"Tcn2",29893.3940341809,1.69375209260453,0.41289597665964,4.1021278683
9706,4.09367922431251e-05,0.000497002226124815,12.8842299826179
"4921536K21Rik",55.7153435461468,0.720513917031088,0.247105216150797,2
.91581832328215,0.00354757009968887,0.01535977282463,3.00886852507234
"Osbp2",605.560453841452,1.85085866080087,0.578927727758776,3.19704614
592596,0.00138842712840746,0.00757670085353728,9.03714578639228
"Gabrg2",0.221621608581856,-0.722128730926801,4.78471990595555,-0.1509
2392974309,0.880035723499026,NA,NA
"Myo1g",1174.42076710651,-1.15613748340271,0.417935900071302,-2.766303
35705898,0.00566957667105373,0.0218296615189979,-4.42163117291146
"Smtn",5124.39790893426,-0.0829791542366364,0.144864270538177,-0.57280
6213211615,0.566775911732116,0.694027301824581,-0.0303076365043074
"Arf5",5341.30031104442,0.191724648533198,0.121744324497031,1.57481385
128449,0.115299418266771,0.219627594506204,0.290620424697202
"2310033P09Rik",1517.17486760224,0.579934591025026,0.250859627015315,2
.3117892581002,0.0207892996728667,0.0591837881474018,1.63953750339332
"Guk1",2251.06471573548,0.544674587888803,0.20987721943948,2.595205851
03742,0.00945342817435172,0.0323941902536924,1.86811193034448
"Npc1l1",10.6328576173728,0.119837006738416,0.540030437128902,0.221907
874999668,0.824385600001991,0.887529718683341,0.0142981454229275
"Rnf185",1678.97114236855,0.119081767981686,0.211622926484638,0.562707
311347621,0.573634204812816,0.699642597594282,0.0425342986034426
"Limk2",3491.96201444928,-0.35386980738363,0.291323101038608,-1.214698
75242311,0.224481008549452,0.36071622751749,-0.360828198221162
"Patz1",1460.11773014291,0.0755824116663545,0.195973195498252,0.385677
293643093,0.699735681271429,0.799940154769115,0.01687138202621
"Eif4enif1",1761.32383118637,-0.116940961095264,0.171441421684084,-0.6
82104475957696,0.495172890559952,0.631161732374841,-0.0538154278554591
"Trim11",2627.40995510168,0.206563856920173,0.148218417869646,1.393644
99965071,0.163424832584551,0.285806849497834,0.258708640254667
"Ogdh",18142.0540033061,0.213423678935882,0.369690107555109,0.57730427
3428705,0.563733927356361,0.691715746616738,0.0786637376866284
"Drg1",2722.37156028321,0.277387618623169,0.194468861768724,1.42638577
765246,0.15375701673398,0.273236813003932,0.359887249139985
"Rtn4",9987.45472975587,-1.48223486953485,0.38044296689001,-3.89607641
232432,9.77635563946244e-05,0.000972051923805398,-10.2809312579241
"Mtif2",1998.26353348917,0.117957393419727,0.169901432142546,0.6942695

65195672,0.487513173653719,0.624255064024445,0.0555810799860848
"Rps27a",16698.1914298251,-0.116101958197338,0.23981940806042,-0.48412
2445036171,0.628298973012076,0.744534664871123,-0.0342495977798673
"Clhc1",18.005823841748,0.804571592560105,0.646157828015433,1.24516264
862288,0.213071969283213,0.347480533379398,0.850469694209222
"Cfap36",2005.85046459105,0.603718249830849,0.131384008616876,4.595066
44824126,4.32611387449983e-06,8.86967069663489e-05,5.63286501792994
"Ppp4r3b",2404.87142057862,-0.155959125557672,0.134919858824851,-1.155
93899160637,0.247706112314985,0.387060076347524,-0.148032559452186
"Pnpt1",1447.13490349401,-0.154129981843494,0.16162364950495,-0.953635
079492333,0.340268392506169,0.484911059573548,-0.11155770675715
"Efemp1",1678.33635372548,-0.0293131011398528,0.161633436320198,-0.181
355428723195,0.856088601427122,0.908458449473496,-0.00281423732542103
"Myl7",90.6267291642728,-0.781737669612299,0.614841433616634,-1.271445
98081809,0.203570033505326,0.335868172967103,-0.852904360963439
"Pold2",651.496252197446,0.0458784191325509,0.188032180943814,0.243992
378869763,0.807236722360779,0.877194538961738,0.00601128813354464
"Zkscan17",1293.36748688715,-0.1543117532331,0.121599095730336,-1.2690
2056554194,0.204433729242028,0.336966323704451,-0.167856048151842
"Aebp1",7478.04339828366,-1.12252528832331,0.350779445111142,-3.200088
55697816,0.00137385368036286,0.00751188980923027,-5.49057225300091
"Polm",939.061963645403,-0.114773045392781,0.153144578416811,-0.749442
432629939,0.45359058377178,0.59400290163088,-0.0597819595038825
"Pgam2",1596.97948680565,2.19996443412981,0.840487761499463,2.61748538
753853,0.00885802808295833,0.0307977588376465,7.65656560428243
"Dbnl",2911.61366794914,-0.654913263783848,0.140653590173536,-4.656214
34174434,3.22076627969421e-06,7.00876126355779e-05,-6.26474604237001
"Mrps24",2284.87820806599,0.594426609471931,0.1929638627619,3.08050741
192613,0.00206648211263951,0.0102394582547002,2.72336941405392
"Ankrd36",0.126027965541212,-0.799378235893475,4.77967760337759,-0.167
245220750577,0.867177104442843,NA,NA
"Ccdc117",1768.282714187,-0.0240562406004811,0.135405988417241,-0.1776
60093779265,0.858989927145236,0.910744187029789,-0.00224909553274298
"Dynll2",11910.4307000977,0.301925451945107,0.267187299511833,1.130014
2353201,0.25847022617574,0.399560173628365,0.276983662928971
"Xbp1",7177.14174069704,-0.401696141039593,0.142401535228759,-2.820869
45477304,0.00478936885038273,0.0192106796636006,-1.58761920828478
"Supt4a",1926.86083582726,0.397802111990725,0.156130129493788,2.547888
18327697,0.0108377202508787,0.0360831084286402,1.32147094241674
"Sept4",3124.3942036218,-0.418039234092276,0.232249470660883,-1.799957
74760095,0.0718673101467478,0.153060492981026,-0.784627059063204
"Btnl10",6.87023236957242,2.48445767968673,1.06031534525684,2.34313092
873792,0.0191226711834229,0.0555172487692644,7.18271999054161
"2810021J22Rik",358.575717491724,0.13392496964023,0.183766338439064,0.
728778571624195,0.466137121349049,0.604907926260484,0.0673212725635639
"Ska2",638.839575419046,-0.0395531960191525,0.147359578073739,-0.26841
2793631644,0.788381592823691,0.863526862140962,-0.00580365126714854
"Prr11",116.058773971195,-0.687524626148734,0.289282331893066,-2.37665
612569412,0.0174703663640491,0.0518315890697245,-2.03490478536864
"Smg8",452.184319137805,0.050783644771757,0.189446080318608,0.26806384

5323956,0.788650174896922,0.863639489477124,0.00744487494995042
"Rnf187",7335.59388695481,0.20069669095106,0.186585317459189,1.0756296
0303647,0.282092942530018,0.426012562423537,0.171251765722208
"Tubd1",237.214881071805,0.208209940059436,0.231645194740073,0.8988312
50495251,0.36874255179393,0.513773208151926,0.13866226923874
"Mrpl22",1085.54826736537,0.449208763813657,0.20249483032l384,2.218371
51645159,0.0265295115765566,0.0717636538179384,1.18338530087376
"Cnot8",2086.54602697244,-0.0318672558422,0.112239375138584,-0.2839222
49236089,0.77646996541616,0.855393603349584,-0.00497746017506883
"Rps6kb1",2684.60607963916,0.0142159245623358,0.187508819397481,0.0758
14698252677,0.939566522266904,0.962511041050371,0.00054318680505352
"Sap30l",1188.48987813785,0.713864462724884,0.140447166904473,5.082797
17176799,3.71916786369156e-07,1.26335078408115e-05,8.05179000953688
"Galnt10",1438.62793333485,-0.838666744379512,0.225988690967547,-3.711
10050148459,0.000206360147369638,0.00173506259310617,-5.33116278147209
"Rnft1",1900.74111515744,0.0104017761114094,0.17108979962243,0.0607971
727967686,0.95152074193947,0.969930765084564,0.000317572322551895
"Mfap3",1835.406331846,-0.188616930011127,0.160011563681922,-1.1787706
1926641,0.238489526591279,0.376663950077401,-0.184165922828291
"Fam114a2",3851.62113747408,0.343098387472194,0.141351055122048,2.4272
7857373225,0.0152125698857621,0.0466448636000297,1.05166259127437
"Gria1",7.20375087209566,0.159900373114898,0.798963939490048,0.2001346
559107,0.841375270120034,0.898419223263988,0.0171282846900607
"Ppm1d",787.133978591618,0.0673693035381985,0.131423065233117,0.512614
002866998,0.608221356096026,0.728311736237813,0.0213578284579882
"Znhit3",381.836905642397,-0.078204327366283,0.14360199559255,-0.54459
0811872676,0.586034975445225,0.709677035664306,-0.0268198058672307
"Myo19",1772.07054052638,0.946397610002851,0.217277579982617,4.3557076
1639818,1.32637728795973e-05,0.000211526275052137,8.00762286554719
"Prpsap2",1202.02419083221,-0.028457288402955,0.0951962740368927,-0.29
8932796381574,0.764991322706281,0.847825885870919,-0.00469772881490708
"Ggnbp2",7899.18765802793,0.75480058762205,0.276171007810198,2.7330913
3209521,0.00627429313318323,0.0236652486969609,2.82578289338299
"Acaca",16340.7668841392,-3.64821073359534,1.07500743169224,-3.3936609
4228059,0.000689650209139534,0.00441312759363395,-19.7848729725291
"Shmt1",1611.46547464616,1.3533380948858,0.298128866509114,4.539439976
85454,5.64038332517667e-06,0.00010856485101115,12.353490532549
"Llgl1",1594.64205968479,-0.3321078226851,0.12228376052046,-2.71587839
032424,0.00661001930567766,0.0246381488094252,-1.22994779543503
"Drg2",2043.21185810819,0.246486663766685,0.0988234281616834,2.4942128
4357199,0.0126236822525625,0.0404231493776424,0.790816141802995
"Srebf1",4958.33935248983,-1.86764875174758,0.497826340968248,-3.75160
693207816,0.000175704783726718,0.00153009909473137,-12.1068888094018
"Tom1l1",767.766061313979,0.238975625694926,0.164303263875948,1.454478
8707019,0.145813587325437,0.262324536415517,0.319790694870439
"Myocd",21.3127702935378,-2.10087325397044,0.806027501064569,-2.606453
56541271,0.00914852274307995,0.0315679537233973,-7.25980449843158
"Cox11",772.408147225665,0.394482828172435,0.215246082602767,1.8327061
9099,0.0668462761216511,0.144834836295139,0.762204432735072
"Lrrc72",1.47299175281349,-0.0804231793532443,1.90983030864562,-0.0421

101178409286,0.966410914456024,0.979592747792798,-0.00165819383622487
"Stxbp4",930.706423907363,0.873208462056095,0.226930982970967,3.847903
22865614,0.000119133091093103,0.00114081192133737,5.91687368799579
"Bzw2",2856.18831643379,0.209114127161893,0.0974462971670773,2.1459422
5990295,0.0318775856384374,0.0823571173682593,0.522093233333706
"1700086D15Rik",0.175348947559291,-0.0714235131930082,4.78471990596623
,-0.0149274178210406,0.988090086099572,NA,NA
"Elac2",1193.81787795006,-0.26422488530388,0.220270177075728,-1.199549
06656765,0.230314517797778,0.367094945477076,-0.264788941323654
"Pctp",1524.32972059829,0.900066618427438,0.275552241523071,3.26641007
691486,0.00108920394569143,0.00628136602575909,4.56348878852349
"Twistnb",1353.23851953588,0.340137117573597,0.175045553737545,1.94313
48601038,0.0519998660406993,0.119845554144392,0.721618382727005
"Efcab10",4.46777041601293,2.4301461531467,0.908505816426614,2.6748823
2789206,0.0074755483776047,0.0270825422271528,8.77007172248317
"Atxn7l1",956.07602979757,-0.367582071196903,0.124322401186136,-2.9566
8413487734,0.00310966495034475,0.0138937321727364,-1.57189763027973
"Atp6v1c2",1194.06699401881,1.63802905031641,0.575018879512004,2.84865
264199071,0.00439047866267999,0.0179730323252014,6.58304685594231
"Sypl",7095.77485915414,-0.00711525585607174,0.306680857661062,-0.0232
008476509981,0.981490062462759,0.988128942938962,-8.49709596507308e-05
"Pdia6",9459.38113737838,-0.448807304204212,0.198459977397513,-2.26144
994113979,0.0237314091982097,0.0656612766491688,-1.22221266173047
"Nampt",5140.50690144407,0.0898865770119644,0.298897873603751,0.300726
719558825,0.763622892122213,0.84679060175163,0.0149483029981517
"Pik3cg",319.072987933499,-1.18468539397376,0.386701490055302,-3.06356
562992383,0.00218716224537932,0.0107122053404687,-5.37417305955724
"Nbas",928.8518781729,-0.147957432231559,0.166453066422829,-0.88888378
815271,0.374065539069971,0.519244233824454,-0.0969684782199926
"Tspan13",1967.82195861501,-0.736049370943174,0.278391003001997,-2.643
94094279654,0.0081946952596553,0.0289929537602539,-2.60613181271452
"Rock2",4829.24781306165,-0.233189228214394,0.182018333427196,-1.28113
044342132,0.200147852349589,0.331646369092571,-0.257367693778608
"Agr2",4.03442510853462,-1.33803513861762,1.03723794974182,-1.28999824
866673,0.197051266170963,0.328160709583314,-1.49090809157493
"Matn3",1.91734620953509,-1.12162102213809,1.14204784856771,-0.9821138
61117786,0.326043757502908,0.470938675738329,-0.844611354877815
"Laptm4a",18580.0042660628,-0.402566848894128,0.139461269654965,-2.886
58528557857,0.00389447182938559,0.016460960704392,-1.65324692968548
"Fam49a",736.612589825166,-1.59975449242993,0.270978884992743,-5.90361
308953197,3.55625646505093e-09,3.74810208408449e-07,-23.6713209422883
"Snx13",2280.05639666035,0.0165829847268466,0.182969770141768,0.090632
3744845817,0.927784706668177,0.955572669727754,0.000753604853375875
"Ntsr2",20.9138952384062,-2.78452672705508,1.48009875004194,-1.8813114
5099351,0.0599295642343396,0.133466252487735,-5.60777681879743
"Sdc1",3069.24212732494,-1.62231348244606,0.413418019112821,-3.9241479
7479674,8.70372271652679e-05,0.000887579422211502,-11.4000172024852
"Lpin1",5321.5488869075,-0.753763870068822,0.604364607629264,-1.247200
54840009,0.212323971891952,0.346637595574754,-0.798594306352175
"Pum2",7158.50661714907,0.0404823514461201,0.0775187034784066,0.522226

890151701,0.601512365597537,0.723209477981179,0.0131185636309597
"Nrcam",74.8529561893011,-0.641274148070404,0.440936123586156,-1.45434
704431764,0.145850113748155,0.262364560506072,-0.858037821622274
"Rgs9",97.0684875394944,-0.297863681967346,0.442495166085397,-0.673145
617843565,0.500854654645047,0.636075298395696,-0.13476494652303
"Slc7a15",14.595874551148,-1.33445603245944,0.811389324819317,-1.64465
564389401,0.100040844868832,0.197408804093967,-2.16512629219182
"Trib2",2521.41786935652,0.602134313665364,0.210575684492846,2.8594674
4096098,0.00424352976962629,0.0175408826474822,2.43456208456732
"Arsg",1387.57940793658,1.39360664343684,0.382254451825025,3.645756476
56487,0.000266606361253483,0.00211398569813762,8.58347438473221
"Hs1bp3",1048.69928565202,-0.133211700368178,0.090474171578812,-1.4723
7270088887,0.140920260848424,0.255501510695793,-0.18177095609385
"Lratd1",554.495992664383,-0.558897423811611,0.413057594630045,-1.3530
7383541074,0.176032043451841,0.302385494236688,-0.668470719466023
"Smc6",2978.53836890437,0.304357473865322,0.220947509411501,1.37751031
761338,0.168354527088284,0.292278209346217,0.374374654635309
"Apob",2288.02858171508,2.09849916664538,0.794638819530009,2.640821358
16941,0.00827053156501203,0.029192401240877,7.41577463840652
"Amz2",2235.63947765854,0.231306165788069,0.159479647093675,1.45038047
176142,0.146952449696557,0.263716416399766,0.308303578176411
"Gna13",3045.22271185317,0.0546328565673813,0.173580569447412,0.314740
622993133,0.752958597902951,0.839500614244724,0.00955791285796792
"Prkar1a",12931.6771403279,-0.261033622909948,0.106464700218989,-2.451
83260153858,0.014213076559451,0.0444683194409783,-0.812591994819618
"Fam20a",471.297333247398,-0.741759602643468,0.215610541922804,-3.4402
7521116775,0.000581122942296623,0.00386714509518756,-4.12065168458372
"1700012B07Rik",1.16605875541074,-3.24647281364455,2.13689625691911,-1
.51924680626526,0.128700383655805,NA,NA
"Abca8b",273.145680202547,-0.673818269687371,0.259941255911957,-2.5921
9440686089,0.0095365864450244,0.0326103757462444,-2.3065640073049
"Rdh14",1212.53485622148,0.594521577846067,0.229996066734554,2.5849206
3054376,0.00974014161517478,0.0330986372479555,2.02628599647486
"Map2k6",516.828121759291,1.01923906798285,0.187971211199182,5.4223147
3362602,5.88321772727053e-08,3.08362847436346e-06,12.9335358763782
"Klhl29",147.088761741192,-0.610383933836516,0.206124809997859,-2.9612
3467060008,0.00306408375669276,0.0137436174575559,-2.61682624975271
"Trappc12",1592.89084977018,-0.0701926259961506,0.097889796263214,-0.7
17057637012656,0.473338533565019,0.611080893031235,-0.0345716886848048
"Adi1",2161.64624529378,0.564484984857241,0.218719916822642,2.58085771
546346,0.0098555192238673,0.0333848008733434,1.91905394505681
"Rnaseh1",636.206823668504,-0.133191432896109,0.1224021701133,-1.08814
60089541,0.276530655250158,0.420104643957148,-0.115510462835107
"Dcdc2c",76.3462227669618,-0.0482639282283996,0.295714561812146,-0.163
21119911254,0.870352148824007,0.918545396361054,-0.00410069403761386
"Ubxn2a",1522.74157857095,0.280745175718919,0.228829489495937,1.226874
98161772,0.219869583118413,0.355650998145247,0.290235870615946
"Fkbp1b",96.5017730361861,-0.671188914349372,0.413187501069515,-1.6244
1727451105,0.10428678275852,0.203745860744051,-1.0677822566815
"Allc",0.850686718700135,-3.65018339540816,4.72365323903982,-0.7727458

41129976,0.439672811249954,NA,NA
"Cmpk2",1104.63611985623,-0.398635146676875,0.220018291184691,-1.81182
730095039,0.0700128852929956,0.150094693506633,-0.756007128439285
"Pfn4",41.1703619678548,0.79039118652973,0.42327283176385,1.8673326687
093,0.0618551460139199,0.136647590865557,1.57315509552207
"Itsn2",4596.57571377839,0.365588152892363,0.207753954037626,1.7597169
4298608,0.0784558116182812,0.163782806738035,0.661427232978102
"Rsad2",2838.10675153258,0.108533783068733,0.251290229604909,0.4319061
00127233,0.665809661018692,0.773579226723292,0.0278635729237011
"Rnf144a",1130.95849946846,-1.21997519093326,0.303412017930674,-4.0208
5322543817,5.79877282455756e-05,0.000649136580996668,-8.9544561510691
"Id2",9455.13977271081,0.646856218001134,0.241772745293395,2.675472031
45732,0.00746240993813593,0.0270500221181956,2.33519458132475
"Mboat2",597.072750778937,-0.352661034430782,0.266612549794843,-1.3227
4731516634,0.185919422992483,0.314483986250319,-0.407966086288978
"Ncoa1",2086.36221065317,0.308299340595614,0.175463016609892,1.7570616
6776477,0.0789072985268809,0.164541727190586,0.556354240073504
"Dus4l",308.073785007804,-0.0781222721104075,0.293998774038635,-0.2657
23122029554,0.790452454574797,0.865030137469798,-0.0113270210160313
"Rrm2",354.474491398142,-0.733777818125154,0.381118048381706,-1.925329
49106163,0.054188150415337,0.123574334128449,-1.53426514478259
"Bcap29",921.901215554454,-0.179272082319036,0.177059257043447,-1.0124
9765368125,0.311300167982988,0.456421067407232,-0.140610175785879
"Slc26a4",2107.52885361095,1.93548296185006,0.746034192522507,2.594362
27085753,0.00947665740923169,0.0324566777033549,6.63454347584148
"Cenpo",305.048676162425,-0.335020739803886,0.12232190973315,-2.738844
90958935,0.00616554506271298,0.0233564790427002,-1.25863300450078
"Klf11",1271.3112677099,-0.216533075626046,0.224971724910878,-0.962490
178318301,0.335803434495176,0.480377715850604,-0.158758278286684
"Adcy3",966.498075238901,-2.40345223859907,0.562154988844618,-4.275426
32600098,1.90771881216337e-05,0.00027862697677574,-19.6737867825468
"Grhl1",281.273934051776,-2.09942832871713,0.499245500244307,-4.205202
30565879,2.60848724950329e-05,0.000351002652800656,-16.7003577479052
"Dnajc27",375.691909450223,-0.438617801869962,0.133186107136229,-3.293
26993108465,0.000990293510596253,0.00582993444130608,-2.25657872727523
"Efr3b",349.23479765767,-1.10691662285316,0.252592787580795,-4.3822178
5132762,1.17477252345481e-05,0.00019280904597914,-9.46835476321198
"Cbll1",624.833662996809,-0.333788930789543,0.181015115132932,-1.84398
374988973,0.0651855084390426,0.142073221947737,-0.65135996091925
"Pomc",46.5156466456356,0.0625524651896632,0.294634328399448,0.2123054
21196061,0.831868766331639,0.892654640167043,0.00710317733930712
"Dnmt3a",3673.00986583646,-0.169673453313886,0.175898272790038,-0.9646
11252984946,0.33473956096462,0.479414717179974,-0.124742100292914
"Dld",11373.9302722341,-0.089994264479396,0.425491041141347,-0.2115068
37460063,0.832491797375346,0.892796054409795,-0.0102050892123431
"Kif3c",682.821705396795,-0.512884754714282,0.167916743966433,-3.05439
911827263,0.00225511844728829,0.0109678971028254,-2.31453745757805
"Sh3yl1",1150.61195439846,0.863168315544006,0.22463101302302,3.8426052
748805,0.000121735169686728,0.00115935691974418,5.83492269807478
"Rab10",6239.68085039997,-0.219687045067588,0.152558087303564,-1.44002

228233531,0.149861095053035,0.267802367458902,-0.289439001177429
"Sntg2",142.568953764378,-0.193170877918814,0.283786989467416,-0.68068
9690113485,0.496067861333125,0.63193069125005,-0.0886607112102685
"Tpo",4.51983990758056,0.459397065876641,0.990187485462673,0.463949577
85392,0.642683871270203,0.755968588474202,0.12851883441044
"Pxdn",3481.65183057065,-1.29418187816452,0.21792231506818,-5.93873040
381209,2.87237760543657e-09,3.21189920567974e-07,-19.3496151101313
"Ccl11",156.527260685501,-2.98509050490493,0.756074027753598,-3.948145
81023773,7.87588320438449e-05,0.000820320356405389,-21.2115027233083
"Ddx52",1830.64129985531,0.229612422479958,0.105028839734804,2.1861845
0950926,0.0288021091035794,0.0762606631316664,0.590930077910472
"Hnf1b",6393.63837142334,1.24194642587027,0.434608524721203,2.85762095
133078,0.00426829928860559,0.0176245869396933,5.01555141832283
"Taf15",11392.3477944879,0.131972793458931,0.207461441799835,0.6361316
70126263,0.524690604909871,0.657439587078765,0.0553497065649839
"Ace",3838.27713482133,1.74114902689734,0.403294752324927,4.3173113879
1146,1.57941269839572e-05,0.00024058897494781,14.5079859821865
"Mmp28",332.309194144208,-1.65835239181894,0.404238365819202,-4.102412
17074543,4.08865079581824e-05,0.000496701016927502,-12.6159527829257
"Rasl10b",37.1416970256033,-0.0836152206209872,0.544736053884628,-0.15
3496762376401,0.878006543303131,0.923773558241121,-0.00662970904942149
"Gas2l2",0.428425425755728,-0.534002986488199,2.99194804565996,-0.1784
80033188681,0.85834600064273,NA,NA
"Cdc27",1696.58026795504,-0.367396767847392,0.193023452623488,-1.90337
89047595,0.0569911216105681,0.12858283836645,-0.753597609806052
"Itgb3",252.500638523764,-1.14857869940083,0.233458805318259,-4.919834
56282597,8.66173931378702e-07,2.47271732269721e-05,-12.1836723573362
"Efcab3",4.08075851554331,0.745950767667441,1.34444107751311,0.5548408
03471484,0.579003554973564,0.703828635590683,0.26199310298724
"Mettl2",588.174734628954,-0.446587295087376,0.163501041654549,-2.7314
0336335558,0.0063065232972438,0.0237566830080486,-1.67018798347466
"Nle1",409.922426567976,-0.404151562249674,0.210774139399227,-1.917462
75611247,0.0551791681586479,0.125292118406582,-0.839466168678361
"Tlk2",1692.13150344363,-0.0592580968830206,0.118276105270474,-0.50101
4949279138,0.616360601979862,0.735213447779479,-0.0182274597506144
"Mrc2",5569.59083699527,-1.08868217731848,0.386831637452646,-2.8143566
1386861,0.00488749759492495,0.0195359631213298,-4.28450680292931
"Rffl",1692.29861197897,0.54010063337781,0.130943284265943,4.124691360
88627,3.7123223217909e-05,0.000460158045964901,4.15010115751762
"Lig3",1393.98458663723,0.360265785085316,0.171845399133721,2.09645289
836928,0.036042034065538,0.0904669902432879,0.865635908156394
"Cct6b",73.5237073822137,1.21067622592528,0.409819349303052,2.95417048
507882,0.00313510758941858,0.013967906729413,5.17078963456366
"Map3k3",1391.09158540061,-0.438833373499455,0.176231054317047,-2.4901
0241242712,0.0127706290644969,0.0407932053570708,-1.40393317072463
"Tmem132e",42.1623180973539,-0.513512450064274,0.385588573848555,-1.33
176262185083,0.182938204144687,0.31108748512237,-0.599618784463367
"Ccl1",17.5761455434416,-1.73178365747208,0.649915687556182,-2.6646281
8274775,0.00770734868068713,0.0276805829021989,-6.21194969775908
"5530401A14Rik",0.551270222265527,-0.960327045511802,3.06501398566404,

-0.313318976684456,0.754038334139858,NA,NA
"Asic2",182.631164350124,-0.381994625054057,0.15388568106285,-2.482327
28617578,0.0130527327083286,0.041537813342091,-1.21518262234739
"Ddx42",4102.14288172746,-0.0449617657189098,0.123780033069905,-0.3632
39244681066,0.716426173768642,0.812049618424659,-0.00936076239980815
"Ftsj3",2199.86173965749,0.10465940783504,0.157412028332532,0.66487554
3144312,0.506130099646271,0.640813291097597,0.046575230561287
"Rnf135",763.756270246345,0.182335131488244,0.16753000565157,1.0883729
7998703,0.276430484454549,0.420031295658883,0.158162244160733
"Psmc5",5533.47664002421,0.154240685287401,0.145135814021811,1.0627334
5643151,0.287902844054893,0.431942878838281,0.129479182490886
"Adap2",1280.23808554378,0.357571032618229,0.362809816629433,0.9855605
22976271,0.324348818071275,0.469185640978836,0.270594298213152
"Tcam1",6.38273869836534,0.0323055397785912,0.760085396736049,0.042502
5134245669,0.966098108139592,0.979460321989693,0.000670454662571031
"Gh",2.18414388616935,-1.63230569547277,2.1101061475707,-0.77356567931
5228,0.439187674549886,0.581152445088135,-0.885921139466107
"Ern1",1167.14681322028,-0.257506326466645,0.138403987542215,-1.860541
2390167,0.0628089895744806,0.138159936679064,-0.509693422890287
"Nf1",1800.09055689783,0.10797811438044,0.133045071955769,0.8115904842
85939,0.417026653266922,0.560384954196998,0.0625335071788346
"Pecam1",5164.60482592045,-0.947669892269587,0.227327809497623,-4.1687
3718338229,3.06291920289983e-05,0.00039707977804615,-7.42155673437616
"Polg2",441.070319330387,0.171858693530486,0.14441233114037,1.19005553
177753,0.234024566502136,0.371671499361297,0.170096262189991
"Ddx5",59128.9260338451,-0.0127055074066171,0.087738407242464,-0.14481
1238383956,0.88485990881364,0.928179390039184,-0.000946944727828835
"Psmd12",3149.08084129647,0.191148895998726,0.22813456834832,0.8378778
25279318,0.402099323907357,0.545970237990643,0.115681555945088
"Helz",2806.20599833172,0.251270580947009,0.200466907334746,1.25342673
405656,0.210050447216852,0.34384716814996,0.268245918594202
"Cacng1",7.42288676395908,0.473616847815528,0.650974222695166,0.727550
84810372,0.466888578081538,0.605469827505788,0.23763751290479
"Cacng4",89.3110139057437,0.00449359794070778,0.370509214839739,0.0121
281678315382,0.990323359363776,0.993584689764068,2.8920691979117e-05
"Cep112",214.555554751804,-0.203545446080017,0.334729126882458,-0.6080
90033800651,0.54312776180972,0.673578906872149,-0.0804310095954924
"Rab37",98.6870489491192,-1.17049918723107,0.31049161853863,-3.7698253
9090808,0.000163361797835246,0.00145245250077138,-7.64862901247662
"Slc9a3r1",10531.7529297906,1.44492145461198,0.343887154414177,4.20173
139957337,2.6488130457164e-05,0.00035460820897817,11.4791741825332
"Grin2c",14.2280745335133,0.848044064255298,0.658126909328494,1.288572
23771716,0.197546840209153,0.328599277923746,0.943802037476686
"Nt5c",1983.23379853222,0.222508606901454,0.178600259137478,1.24584705
51836,0.212820550597979,0.347215722162111,0.235371610183349
"Jpt1",3382.85609665018,-0.764971526082122,0.227643361761105,-3.360394
61095687,0.000778312184697882,0.00484996196140598,-4.07636834769064
"Sumo2",7526.78764161655,-0.107062392104038,0.148104875160106,-0.72288
2295321478,0.469752201652713,0.607698276793034,-0.0533252910328545
"Nup85",1440.88311013965,-0.0261114415532725,0.134279063613403,-0.1944

56535893406,0.845818425411423,0.901337249749791,-0.00271234649823392
"Gga3",2111.44289849228,-0.356619524986579,0.180203012932841,-1.978987
58285183,0.0478174043147895,0.112520365596158,-0.779078519771442
"Cluh",9809.15743450282,-0.0149030624426763,0.365122542839352,-0.04081
66045481159,0.967442101874606,0.980058507941553,-0.000300192492869321
"Mif4gd",2881.2408572813,-0.262366921403054,0.185111078964604,-1.41734
856104005,0.156381043917684,0.276865304123037,-0.336937938664135
"Slc25a19",4259.39252760756,-0.277769518543433,0.356838737268881,-0.77
8417502173064,0.436322924040888,0.578460894332578,-0.15204668264l521
"Pafah1b1",6589.26939890936,-0.261211168537428,0.162537153468589,-1.60
708590597969,0.108035524597706,0.208872251720979,-0.409065166149192
"Tmem94",3231.5929103783,0.372788716709277,0.146863602921406,2.5383329
1090356,0.0111381975422388,0.0368901946116141,1.2301317458818
"Recql5",1493.50293983415,0.152613170135878,0.155291216169214,0.982754
6843962,0.325728189619933,0.470587451186237,0.115035758608433
"Sap30bp",1731.49632268012,0.052866693769675,0.200820554200396,0.26325
3400430914,0.792355274243062,0.866577470113935,0.00757070975144991
"Itgb4",1435.75510673183,-1.39888209023927,0.373329628206498,-3.747042
78618229,0.000178931574931351,0.00155196078648198,-9.04829966927759
"Galk1",1928.29087870874,0.839536867527524,0.259135963078092,3.2397543
6506482,0.00119632709292045,0.00673771971628047,4.19771265717063
"Unk",1758.45936878175,0.0577557997683625,0.241846972552356,0.23881134
0736793,0.8112518760004,0.879792484468313,0.00739673978915751
"Trim47",3139.31902572626,-0.0105807926251303,0.328899375206972,-0.032
1703032073928,0.974336238523446,0.984077077375299,-0.00016983287729753
"Aspa",2937.31186557101,1.23174390736216,0.525564843175338,2.343657349
52942,0.0190957055955385,0.0554802663838191,3.56186820053621
"Mrpl38",2997.28083432515,0.213882158926509,0.191729192615053,1.115543
00109078,0.264617802075478,0.406499985717858,0.192530599255191
"Fbf1",2729.92429707976,-0.0382221821581798,0.164150868873725,-0.23284
7882075986,0.815879530120397,0.882553069530513,-0.0047753401777521
"Acox1",26049.2615717045,0.747907241367415,0.467246519270102,1.6006694
7643771,0.109450145154588,0.211063092693518,1.1634431380188
"Ten1",572.169576873671,-0.0537908172419496,0.221089449546451,-0.24329
888808488,0.807773865699013,0.877557139446744,-0.00702579128608817
"Srp68",3096.64790975074,0.00934299151101204,0.105252092440361,0.08876
77507818296,0.929266487631335,0.956303244594089,0.00041744686730846
"Tsen54",1049.20735513956,0.451876526652132,0.201573332502139,2.241747
56175813,0.0249776920595581,0.0684298215227293,1.21190869719086
"Llgl2",4265.9874358278,1.09204435637376,0.334457143836849,3.265124924
06642,0.0010941580855758,0.00630425341717043,5.53287628769948
"Ncbp3",1447.36908411247,-0.0415109392591899,0.134137663144098,-0.3094
65203777977,0.756967677286199,0.842236690922524,-0.00712718745064673
"Camkk1",241.182183214434,-0.71852002469767,0.235340637047437,-3.05310
648306284,0.00226485555725035,0.0110042745216216,-3.24014556550008
"P2rx1",233.620235640945,-0.600210806749782,0.309597382861347,-1.93868
17847184,0.0525400980045328,0.12074809416459,-1.26887491768408
"Atp2a3",1556.13884714187,-0.731927933305368,0.228599272995639,-3.2017
9466764679,0.00136574311350197,0.00748433367820727,-3.58274572298414
"Ankfy1",3376.48292807328,-0.208586573595487,0.075711167346812,-2.7550

3047839705,0.00586866683196306,0.0224367188018855,-0.792014990475907
"Exoc7",1843.03248689118,0.356552767497029,0.151006369946467,2.3611769
9950955,0.0182170335450322,0.0534691735753483,1.04421825753755
"Galr2",10.8876080609588,-0.167026426885954,0.649777694873986,-0.25705
1647361251,0.797138903373088,0.870344626384168,-0.0231942957191574
"Ube2g1",2419.03039491517,0.103563251032718,0.167091461146021,0.619799
781044553,0.535389612856621,0.667158348020605,0.0419149327489764
"Spns3",358.776997072303,0.492465871782888,0.472263665050698,1.0427773
8946955,0.297051405605363,0.441380567633727,0.402762134993358
"Tekt1",35.3679484458651,-0.670664984776659,0.414334292775081,-1.61865
671384513,0.105521145201531,0.205334459993957,-1.06173987594191
"Med31",538.793914056692,0.125972050653741,0.139195787108121,0.9049990
18080131,0.365465907388193,0.510448013055515,0.0847119817220686
"Ube2o",1253.26417426869,-0.227122470054901,0.177209869121628,-1.28165
813326692,0.199962597639177,0.33142389737918,-0.250824304700602
"Txndc17",5558.97059999223,0.990080116540166,0.270566624949205,3.65928
398125984,0.000252920941617529,0.002025427025393,6.14045185743562
"Aanat",2.0252976988733,0.743681225469777,1.63003433442096,0.456236540
40021,0.648219891954439,0.760194361441577,0.203903365941874
"Slc13a5",41.4546008056541,1.7095513966323,0.571425388705396,2.9917316
0735019,0.00277400026816853,0.0127564757614976,7.45657807861531
"Rhbdf2",1466.17482115423,-0.470167028382069,0.205735577112215,-2.2852
9763778107,0.0222953813597094,0.0626599269455372,-1.30237825904927
"4933427D14Rik",733.99758782808,0.254820153353523,0.370029815258791,0.
688647624719935,0.49104504321142,0.627500082090462,0.118749116073076
"Pimreg",97.8435530883937,-1.12382422964485,0.380755130262594,-2.95156
687414767,0.0031616607474809,0.0140561913731867,-4.7927645534834
"Cygb",3899.73128692857,-1.20506122176246,0.248037659969699,-4.8583800
6176025,1.18350065970284e-06,3.13957766841986e-05,-12.4950835938786
"Wscd1",313.263273122651,-0.0851763216780373,0.272561906943712,-0.3125
02662727655,0.754658539757064,0.840663075185777,-0.0147835706346264
"Mxra7",1722.06992335392,-1.02427347607959,0.300732814567812,-3.405925
21488414,0.000659402393298959,0.00427143782875186,-5.58823612720822
"Rabep1",2271.27325075786,0.221944627970562,0.118614736351037,1.871138
73704295,0.0613258535726398,0.135839267217332,0.443064276858795
"Mfsd11",2449.03664200292,0.339556149857659,0.132188025266184,2.568736
08009426,0.0102070160782583,0.0343843122829787,1.14435680763488
"Kif1c",11266.7789779115,-0.305188029485213,0.200232092991645,-1.52417
139992613,0.127465900425717,0.236675566755607,-0.439795785363936
"Sec14l1",4174.31102474296,-0.345344271671266,0.120478993263158,-2.866
4272693327,0.00415133536083677,0.0172475467431484,-1.40212722274902
"Nos2",146.900515093659,-2.28987424902036,0.523919857173466,-4.3706574
921099,1.23872970261932e-05,0.000200437773789805,-19.4982946058854
"Mink1",6514.18179798569,-0.0101049827286582,0.169180044181773,-0.0597
291647341163,0.952371343097323,0.970244861929682,-0.000305239236365997
"Pld2",1630.97482212457,-0.575920800917318,0.207644127406108,-2.773595
42074088,0.00554405787817531,0.0214430649026237,-2.21289159334618
"Slc46a1",982.208033428461,0.941193133163687,0.282274166614982,3.33432
260008214,0.000855074241872157,0.00522640676113584,4.94505816290479
"Vmo1",3.88638604310886,-1.1771973048732,1.00131979515753,-1.175645693

3801,0.23973648816246,0.377942407541418,-1.14542881797584
"0610010K14Rik",1222.24317806233,0.343176448040379,0.121183989722207,2
.8318629286513,0.00462776822676617,0.018681620059107,1.36591608835086
"Eral1",1240.22885955644,0.0965389574028511,0.188905592237332,0.511043
406706373,0.609320662597088,0.729135527146067,0.0304962372743368
"Dhrs13",293.385581524879,0.688720831975274,0.215204662651795,3.200306
2735199,0.00137281622508471,0.00751084002638148,3.36881456324242
"Coro6",414.934747119017,-0.16874217472075,0.384760045989905,-0.438564
701505357,0.660976978588462,0.769738346085997,-0.0441606087653292
"Slc6a4",115.761222126249,1.69326041325947,0.35943106178351,4.71094625
171641,2.46569263878714e-06,5.63611665545975e-05,16.5664029957436
"Tmigd1",7955.31018613379,2.16053708369478,0.756446949970695,2.8561647
1026618,0.00428792626051812,0.0176794219132126,8.71853178896403
"Blmh",3637.26167187396,0.0324732676903529,0.222947545559086,0.1456542
95538081,0.884194303352535,0.92793048747337,0.00242895024364271
"Cpd",6439.74396368434,-0.226638482564581,0.126831877994314,-1.7869204
9781634,0.0739503390308403,0.156689376227368,-0.420072144913841
"Timm22",1454.98562435318,0.509813051683083,0.24744428616302,2.0603145
0387669,0.0393684842535855,0.0967819150352776,1.19056393695997
"Nxn",1152.16430522537,-0.720707536954703,0.189321979212138,-3.8067821
8109658,0.000140786638290692,0.0012972863295803,-4.79088939543093
"Rflnb",2955.10977945053,-0.897955921509066,0.360040835434306,-2.49403
910094223,0.0126298630761928,0.0404258080326176,-2.88090020653746
"Rph3al",454.303388274557,0.768305482390844,0.219129994816444,3.506163
0108394,0.000454616703538673,0.00320814928903259,4.41165698768783
"Doc2b",72.1010167263477,-0.815086127633991,0.34193279446249,-2.383761
19762156,0.0171367185498849,0.0510982930548488,-2.42406955581531
"Ywhae",16540.5842644262,0.0309011841785391,0.155977915397605,0.198112
56035682,0.842956997858058,0.899467823796662,0.00327404218701742
"Prpf8",5601.84508719701,-0.338459506597673,0.175692397300655,-1.92643
228618756,0.0540504192861485,0.123332366909459,-0.708352559169162
"Nme2",16363.6977421309,1.36321710551762,0.255901941119344,5.327107326
95015,9.97892043748689e-08,4.56734719929857e-06,16.762905473235
"Spag9",6448.19923646154,0.112601682175484,0.113789709713052,0.9895594
46626905,0.322389499967136,0.467565491695866,0.0856015836250989
"Luc7l3",14171.3716409943,0.352576737034944,0.239944349783498,1.469410
45852121,0.141721492309363,0.256620139027485,0.479560191395869
"Ankrd40",3983.01970006676,-0.113630962346524,0.211104962246837,-0.538
267604594062,0.590392313078293,0.713756778298646,-0.038317840127956
"Abcc3",1882.56003771729,-1.98071978058959,0.44105376288347,-4.4908805
8480734,7.09293120034002e-06,0.000129613844933674,-17.7293256048419
"Cacna1g",627.086429683228,-0.803531746913352,0.549365452903041,-1.462
65430901635,0.143561994540121,0.2592545864169,-1.08472345591756
"Spata20",7.53055903480227,-1.15169588061909,1.00056145535911,-1.15104
961764266,0.249711823478156,0.389261363461552,-1.0866299874081
"Xylt2",1455.06568928629,-0.302024705706083,0.118122081310264,-2.55688
608222855,0.0105613798086096,0.0353976225937466,-1.0090979515758
"Lrrc59",2915.8145904742,-0.715688505701542,0.248976103881786,-2.87452
689050573,0.00404633312177237,0.0169451707506882,-2.91841483355343
"Cdc34b",3.29209077407674,-2.67327700803058,1.1736528110714,-2.2777408

9817091,0.0227420194725782,0.0635277787501905,-7.36829462681699
"Dlx4",2.80670262653731,-1.62833307024433,1.1299176182,-1.441107779909
06,0.149554235963688,0.267387043824754,-2.14786587353609
"Tac4",3.11000749975659,-1.11296183982127,1.05289683072924,-1.05704738
331335,0.290489962883067,0.434253875357972,-0.928350351559781
"Slc35b1",2002.65328649722,-0.00430751534398453,0.104792290535414,-0.0
411052695000385,0.967211973702355,0.980031236663284,-8.68861755744644e
-05
"Hoxb9",2955.47286811081,0.217357324829548,0.32090287260923,0.67733056
7539755,0.498196240674559,0.633855158282059,0.0991007700795536
"Snx11",1192.41960488495,-0.50035239119766,0.130769878261878,-3.826205
22285463,0.000130133813000182,0.00122193349506861,-3.35602402219211
"Scrn2",3337.00515013419,1.61384180149765,0.387365377163526,4.16620043
152789,3.0971842169693e-05,0.000400988697625946,12.6227884270561
"Lrrc46",39.9028396155893,0.797945181454937,0.305784163644661,2.609504
59940167,0.00906734271353346,0.0313466397568219,2.76300344820922
"Cacnb1",780.265557299043,-0.127132375596477,0.24555960250699,-0.51772
512375222,0.604650066579245,0.725639625663945,-0.040771577977176
"Fbxl20",2869.63613536481,-0.127250371100438,0.165498095545752,-0.7688
93265392649,0.441956675721462,0.583509700922065,-0.068549037113102
"Asgr1",25.7450760271161,-0.172870567249309,0.478283385266357,-0.36143
962465482,0.717770831012449,0.812959376304429,-0.0357970237554197
"Dlg4",1166.1430476733,-0.440010317900339,0.325212485056258,-1.3529933
1396893,0.176057766366801,0.302385494236688,-0.526275486784955
"Dvl2",722.306915446496,-0.220482485992486,0.101117105701961,-2.180466
74162482,0.0292228815428422,0.0770712490629429,-0.565102117588895
"Nr1d1",3089.78070875378,0.600236545057098,0.443347420254549,1.3538739
9956556,0.175776579899541,0.30212374425963,0.718434282974194
"Gucy2e",3.3145967667997,0.647845113077852,0.78949780985515,0.82057873
371012,0.411886265162879,0.555389196420526,0.38098874235527
"Alox8",85.6861396559177,-0.164275883209417,0.297966037062526,-0.55132
4187242671,0.58141146074448,0.705787614344159,-0.0572404392695146
"Aloxe3",12.843867719358,-2.50093370946276,0.626274332920156,-3.993351
75976564,6.51458131156147e-05,0.000710409991123185,-18.1309398396561
"Per1",3521.86089285853,0.751640897884112,0.279980889109541,2.68461501
166973,0.00726133951370332,0.0265233416706983,2.72825360340717
"Vamp2",6314.91842343379,-0.0307456023610354,0.162522154315576,-0.1891
77915407985,0.849953376074785,0.904359668578781,-0.00309079804157948
"Tmem107",451.78376201397,0.325511990411297,0.256151733797067,1.270778
0095261,0.203807634079302,0.336196383187668,0.354827540368448
"Aurkb",169.489694535259,-0.743600236769269,0.448182300445855,-1.65914
681599325,0.09708621147543,0.192858174950506,-1.22381742213082
"Ctc1",1207.02255297376,0.123897367645268,0.168986072306938,0.73318094
1801093,0.463448077614474,0.60251166438802,0.0627723849709413
"Pfas",1981.4203774416,0.443400799936982,0.155918207762322,2.843803852
67698,0.00445784815723157,0.0181773578262783,1.77696352817737
"Myh10",2730.96775932432,-0.655894075106254,0.195959990506479,-3.34708
158237316,0.000816671720133909,0.00503907126556043,-3.47002956800261
"Pik3r5",630.123059922069,-1.17664541135729,0.345185480127038,-3.40873
379414525,0.000652651336929691,0.00423756102812358,-6.42891684518396

"Ntn1",2429.39127041759,0.231834166281402,0.262751696141464,0.88233175
917001,0.377597426699182,0.522785956527774,0.150363736076628
"Stx8",1296.81264921536,0.436786748104174,0.188574454548319,2.31625619
254942,0.0205442846986415,0.0586232201162173,1.23899995302036
"Cfap52",39.4631105060164,1.08694774342245,0.865245993092063,1.2562297
3362535,0.209032689383016,0.342612590584233,1.16428944887515
"Usp43",418.198755549526,-0.196762015163901,0.249909253488859,-0.78733
3851856238,0.431086458075541,0.573350870365409,-0.109450329032731
"Rcvrn",1.18549149430415,0.915717380940997,1.55021462724196,0.59070361
2808881,0.554719026843616,NA,NA
"Myh3",2.86400732362286,-0.467017123792325,0.95212274052687,-0.4905009
65803941,0.623779446558401,0.741386872843384,-0.139746792248492
"Adprm",728.862630364406,0.01316706229014,0.130985728500625,0.10052287
7116931,0.919929221510203,0.950847114133054,0.000663646176474472
"Krt19",4941.26724416312,-2.09489376872164,0.584229061995932,-3.585740
43126979,0.000336123161393729,0.00252844498489774,-12.5277806378871
"Krt12",12.1356542648275,-1.43987377463228,0.581203050540079,-2.477402
30078678,0.0132342635819469,0.0420126459330512,-4.5640897345907
"Krt24",0.15580818916143,-0.0620942904651072,4.78471990595397,-0.01297
76228672945,0.989645645723552,NA,NA
"Top2a",705.997537624955,-1.04724178424267,0.415019998049053,-2.523352
58340707,0.011624178674425,0.0381113562794583,-3.4215933639337
"Krt36",9.67802626641924,-4.32866952713787,1.38764115865836,-3.1194444
6165249,0.00181192415512789,0.00925809674441392,-20.2679422704322
"Acly",26030.5433574471,-3.31961857768615,0.722273086827299,-4.5960712
620044,4.30531506572453e-06,8.84563046907238e-05,-30.982006360648
"Kat2a",2137.3593290074,0.172883187446003,0.150745586356554,1.14685405
804909,0.251441942916569,0.391426347543893,0.162157153193238
"Stat5b",2120.78785036788,0.0235339786513129,0.100071970710709,0.23517
0532609431,0.814076376244062,0.881747956198947,0.00296172833260207
"Tmem101",671.950183503041,-0.0619818956566579,0.145453347195135,-0.42
6129043104833,0.670013818287611,0.776644041910221,-0.0156673591384218
"Lsm12",2039.40895659274,0.134735316576327,0.187662354700432,0.7179666
73664554,0.472777835698212,0.610478294069122,0.0664935683670381
"Ubtf",8278.89183128595,0.164605725335348,0.083696173009056,1.96670551
851322,0.0492171703196864,0.115009154709162,0.355999970370443
"Ccdc43",949.277674929204,-0.220819758003491,0.134155729047481,-1.6459
9573623379,0.0997646368528995,0.196983381200793,-0.358751709194271
"Adam11",1056.10831169958,-0.0152366080934258,0.293444903478513,-0.051
9232329913048,0.958589861992803,0.974775160952669,-0.00038927154830694
1
"Higd1b",76.0267209763683,-1.37959676290499,0.524144406321058,-2.63209
288560057,0.00848606587979502,0.0297645822133915,-4.84850467892385
"Eftud2",2622.68240788661,0.263964711725191,0.236967518151545,1.113927
82345967,0.265310151911978,0.407180975321621,0.237171642959944
"Ccdc103",74.4188549727925,1.05754153941035,0.288923099468498,3.660287
2575983,0.000251932652466418,0.00202230665203385,6.56047640196526
"Gfap",18.7860937562489,-0.715225239249719,0.490699788121024,-1.457561
74460242,0.144961380672146,0.261201090504158,-0.960164640970113
"Dcakd",1004.33688297601,-0.243821920456294,0.139021619952894,-1.75384

174446328,0.0794576279330187,0.165484386823115,-0.438605992995194
"Nmt1",7359.14577770919,0.267164423379032,0.192836250247634,1.38544709
843688,0.165915797906074,0.288790057808662,0.331832987608943
"Plcd3",1119.41506886447,-0.149132371321389,0.208397205113206,-0.71561
5985542499,0.474228500207183,0.611865226114323,-0.073260269200591
"Efcab15",4.08988029800232,0.211644569095503,0.904689504835099,0.23394
1665029131,0.815030266533283,0.882212657080868,0.026523751214894
"Map3k14",1208.49929934351,-0.84539928017802,0.220741875588905,-3.8298
0926442989,0.000128242620726705,0.00120629101447548,-5.68125638870294
"Lyzl6",0.273975376445809,-0.981078679014927,4.78175924886694,-0.20517
1073647716,0.83743846856737,NA,NA
"Gosr2",3803.04633392857,-0.250554094379179,0.144961234136724,-1.72842
136638312,0.0839127075659261,0.172496570597831,-0.440318234054551
"Klhl28",800.272064898574,-0.178489674868576,0.0940063832908699,-1.898
69739288131,0.0576042745513487,0.129655709976868,-0.364631688749989
"Fkbp3",2230.27848432393,-0.0352858845205264,0.190488998020747,-0.1852
3843837261,0.853042021295868,0.906427978408292,-0.00346661592813881
"Foxg1",1.57496002795412,0.694827051377595,1.12197909956034,0.61928698
2841186,0.535727318298425,0.667408552308651,0.280955332097217
"Scfd1",1576.41108771824,-0.00436825406853058,0.122772210836502,-0.035
5801531858692,0.971617133763788,0.982603261106138,-7.66621617245377e-0
5
"Coch",181.047797673706,-0.602139647476148,0.558867762926022,-1.077427
769896,0.281289203460623,0.425259896803538,-0.514862380833664
"Strn3",1784.97816548405,-0.0945276757223225,0.152575527468601,-0.6195
46773264641,0.535556218799293,0.667323309953261,-0.0382346136336791
"Ap4s1",1115.69632508624,0.201466419461451,0.213150727956601,0.9451828
8252091,0.344565516891419,0.489068824950782,0.144099270148591
"Dtd2",837.191462600342,0.306346348022753,0.276254963208966,1.10892613
281675,0.267462049200099,0.409563734807471,0.273463972634238
"Ston2",890.302092044717,0.0811528809920537,0.194771111693826,0.416657
687509756,0.67692880768861,0.782369095919354,0.0199172428469376
"Gtf2a1",2727.36608558846,-0.0909128511189227,0.0980321674626644,-0.92
7377752343861,0.353730437594309,0.498665033057178,-0.0632590423477854
"Tshr",336.677913239639,-3.24071930312238,0.712713494062568,-4.5470154
9798058,5.44119929395247e-06,0.000105880742348788,-29.6629427521671
"Sel1l",5398.47856369594,0.250975254960138,0.141467464344139,1.7740846
3581142,0.0760491517705231,0.159809598618809,0.460231440666101
"Dnaaf2",238.967151525287,0.0863556185777381,0.209185020689155,0.41281
9322785358,0.679738993336763,0.784419442002853,0.0209681285704533
"Pole2",140.249352792902,-0.488504000747211,0.227507517243947,-2.14719
938340942,0.0317774117863449,0.0821418500610039,-1.2209217848736
"Klhdc2",2612.81253177849,0.101194879680925,0.108455051516286,0.933058
241789029,0.350789897987708,0.495702379400227,0.0710164995419221
"Nemf",5005.83382978393,0.610650938063533,0.217033914569477,2.81361988
643507,0.00489871158215812,0.0195692834632513,2.40217506481487
"Sec23a",1355.26505113238,-0.401045171541962,0.105622872730302,-3.7969
5383372117,0.000146485056922101,0.00134044651593,-2.6528145509425
"L2hgdh",2249.92691995579,1.07624803017255,0.313757854273654,3.4301867
3640553,0.000603165994665063,0.00397987497935005,5.94788998266247

"Cdkl1",1746.94605661848,1.20869764993991,0.549906623697953,2.19800525
735041,0.0279487312915974,0.0744866542815153,3.13915134191054
"Trappc6b",2678.36185212296,-0.219026247990188,0.21969266251313,-0.996
966605460016,0.318780719698054,0.463849736766616,-0.168254785866087
"Pnn",7686.5176447825,0.0459107868612252,0.100431485635306,0.457135395
048715,0.647573730808724,0.759802178559905,0.0126115632669581
"Mia2",6040.63238841395,0.616205381753278,0.162462396164909,3.79291082
920994,0.000148891634052394,0.00135531703116414,4.0692477300653
"Galc",3325.43513426601,0.791340797229544,0.172038777431123,4.59978156
695727,4.2293418257731e-06,8.73556217361482e-05,7.39549375169095
"Spata7",599.902391429825,0.482137295188733,0.171145284138437,2.817122
87671766,0.00484559833197453,0.0193963203196139,1.90090916816563
"Ptpn21",1550.53599410419,0.251681797989905,0.123823364030963,2.032587
30660047,0.0420942349913354,0.101844123451827,0.57491971006452
"Npas3",26.5185365645066,-0.365118510386505,0.67150137217517,-0.543734
570792893,0.586624139149087,0.710169965548795,-0.12496215685931
"Zc3h14",3184.73217526227,0.129936712244269,0.0955343527474563,1.36010
459596408,0.173796826899339,0.299618968247862,0.156605406096491
"Ttc8",870.855979113677,0.0446255917302513,0.147141030811336,0.3032844
84852292,0.761673069017809,0.845664505366508,0.00748070235236726
"Polr2h",836.362950593389,0.283189187757315,0.159685990804379,1.773412
84812036,0.0761603215683428,0.160018744605555,0.518934081476094
"Ppp2r3c",1026.42450812807,0.146145759611256,0.158238791158675,0.92357
7326021829,0.35570642351409,0.500762732539908,0.101077750843536
"Prorp",513.836968949524,0.647358699258342,0.172637937610737,3.7498055
6543719,0.000176971733974377,0.0015383838025111,4.19295711477736
"Psma6",3986.42877098674,-0.219685208814197,0.316260212759487,-0.69463
4354721267,0.487284476780904,0.624085176581323,-0.103574638076547
"Nfkbia",6349.80790747698,-0.397696129404274,0.229186223633023,-1.7352
5320632305,0.0826959660823086,0.17049867258886,-0.703535496019573
"Ralgapa1",2351.54152815012,-0.662554720633565,0.208829172376145,-3.17
271151867693,0.00151022504517458,0.00808743969536225,-3.19181965119309
"Mbip",553.348475482155,-0.078334563026376,0.17106213650983,-0.4579304
60969512,0.647002401671023,0.759497461034413,-0.0215497051116047
"Ngb",87.8822693591232,-0.751953780894647,0.268670259303776,-2.7987979
8695709,0.00512932166657196,0.0202534454951033,-2.93219139651931
"Gstz1",9078.38989805241,0.0770438482233418,0.54722989723121,0.1407888
14012459,0.888036780167606,0.930610616989572,0.00554055679335086
"Sptlc2",4647.51394630932,0.684289361624247,0.167929550294012,4.074859
72794061,4.60420910975015e-05,0.000543419305217364,5.14423377849774
"Ahsa1",4311.67589752277,-0.0777486421983423,0.149362319980456,-0.5205
37189088356,0.602689214650527,0.724267137581262,-0.0250813216820796
"Vipas39",1630.36031069051,0.356419025576271,0.08912164917689,3.999241
81013353,6.35457291303075e-05,0.000696466906454601,2.59098464201131
"Snw1",3340.13363617757,0.18150736348199,0.168884432742109,1.074743009
37586,0.282489802154721,0.426413730929221,0.154706930873876
"Slirp",1669.10392805461,0.629224860482363,0.190102693705684,3.3099208
0236656,0.000933223723386408,0.0055662270292408,3.26633005316343
"Adck1",767.517018068679,0.159558433962663,0.219618419143703,0.7265257
37617023,0.46751653525231,0.605851216559188,0.0799580565021158

"Nova1",317.262636823586,-0.700265147444561,0.486209508768037,-1.44025
391280171,0.149795575208665,0.267709819011783,-0.922845579973544
"Mthfd1",5247.85044796628,1.46128553980032,0.31813773408914,4.59324809
106385,4.36399734603992e-06,8.93794330389563e-05,13.6230097373056
"Ppp2r5e",2163.07997539929,0.191531765981744,0.104909784875996,1.82568
066656638,0.0678983770355913,0.146609363836097,0.367737853049677
"Sgpp1",5233.3479905064,1.04550306025766,0.287700235365831,3.634001407
50053,0.000279059337479487,0.00218246903950941,6.40610928700949
"Esr2",8.01930623325753,-0.387050244371341,0.863366810311626,-0.448303
362775363,0.65393427404967,0.764374907157495,-0.103999198485562
"Tex21",2.96004523372668,1.34379308222084,0.844656630813267,1.59093415
383123,0.11162439523682,0.214196679296085,2.07059784707414
"Akap5",84.578645171319,-0.629224373159952,0.374026987591121,-1.682296
71664176,0.0925113174979129,0.185918951615575,-1.05863505080035
"Sptb",237.883113372433,0.453651433059488,0.416162404658054,1.09008268
882971,0.275676721149273,0.419081920237683,0.394535599685669
"Rab15",906.527341762871,-1.26590048819587,0.338306738266256,-3.741871
93161838,0.000182654597824347,0.00157793502365047,-8.16713198983068
"Fut8",2077.60398290797,0.6517155691835,0.148420545553282,4.390979392
29977,1.12841236090156e-05,0.000187795454709123,5.59178760370784
"Atl1",235.389696787273,-0.147327016675414,0.159744713487771,-0.922265
366150552,0.356390174717887,0.501508056195825,-0.101675620155941
"Sav1",3064.7325528906,-0.385413138821781,0.170653173405011,-2.2584586
6872384,0.0239170785812485,0.0660425269890038,-1.04734339924792
"Nin",1089.02188736906,-0.553525747041881,0.197495376872848,-2.8027276
1725583,0.00506724454284363,0.0201036203220238,-2.16254503428165
"Pygl",3750.84472464122,-2.87496124931719,0.733919316489298,-3.9172715
3751663,8.9556853036544e-05,0.000906635265060506,-20.1413181975578
"Bdkrb2",115.775616872004,-0.34544630665881,0.262249655275959,-1.31724
217633501,0.187757460645163,0.316659023238421,-0.397238974860053
"Trim9",13.5713090356298,-1.01060129486019,0.743214850195151,-1.359770
05114312,0.173902702492328,0.299748487729438,-1.21758409247591
"Tmx1",2823.01109287026,-0.0107852356966845,0.16532999147284,-0.065234
5990016959,0.947987213767233,0.967842428214133,-0.000352526060036737
"Actr10",2797.06987188368,0.228707493193066,0.125607335760643,1.820813
18585477,0.0686352592334794,0.147921994532373,0.437076076618624
"Tomm20l",0.323393249422347,0.869709338651851,4.78471990595042,0.18176
8077493994,0.855764737612356,NA,NA
"Timm9",1820.72088011016,0.963810080354408,0.197890036346409,4.8704325
8038138,1.11354192303282e-06,3.01135095912537e-05,10.0337801779597
"Serpina1f",3954.79800279208,2.30560998741446,0.733220451296203,3.1444
9765188431,0.00166372246510938,0.00872023135072653,10.9334550485143
"Rtn1",438.719313095385,-0.788383230517893,0.53251903156803,-1.4804789
7592779,0.138745467370256,0.25254814651244,-1.08493623888735
"Lrrc9",13.6710862693947,-1.7936661907534,0.788164386394146,-2.2757513
8095166,0.0228608941883904,0.0638104910192588,-4.93587818414499
"Serpina3n",837.80368291551,-2.82944306126094,0.614238214129798,-4.606
42629548776,4.09648164765835e-06,8.51526643561221e-05,-26.514894448139
7
"Dhrs7",2174.56724893414,-2.29939087368655,0.579966384337229,-3.964696

80965084,7.34892931244656e-05,0.000776698269304374,-16.4646931811044
"Gsc",1.6361265371173,-3.15618367432073,1.86748921733797,-1.6900679506
0361,0.0910149555154339,0.183573973584352,-5.35016551444257
"Ppm1a",5258.84070693933,-0.0679560274778449,0.244363402793007,-0.2780
94128257857,0.780940103006437,0.858523349595145,-0.0103661076299712
"Clmn",5652.87656653578,0.614151809934857,0.170854838151324,3.59458249
224942,0.000324912188880842,0.00246311736345204,3.68879691155474
"4930447C04Rik",5.77146638987535,-3.51644153247413,1.86832551236736,-1
.88213537159187,0.0598176378486038,0.133263745746352,-7.08712439953277
"Glrx5",3867.13818415045,0.449671468675204,0.442903414085382,1.0152811
0729017,0.309971847591642,0.455210596094136,0.353889250800393
"Mnat1",777.566849039397,-0.295622027447979,0.199117920350874,-1.48465
807058979,0.137634424117683,0.251061465192869,-0.408566636824386
"Prkch",807.353451741636,-0.841471655837899,0.160349649344879,-5.24772
994063783,1.53984797275916e-07,6.36100757899534e-06,-10.0684808043601
"Hif1a",3248.60374055698,-0.200470731703165,0.197528514323228,-1.01489
515268222,0.310155809084598,0.45532955287252,-0.15771710659112 8
"Papola",5721.88523229051,-0.0701901489831798,0.166570355959413,-0.421
384396874812,0.673474410976777,0.779418888440524,-0.0174918520160234
"Mpp5",3006.12536576408,0.771720219439506,0.203669797992236,3.78907539
088798,0.000151209023851207,0.00136874352230773,5.08861670442847
"Snapc1",1134.17263361339,-0.104543636520049,0.105565005837696,-0.9903
247358389,0.322015419924542,0.467132656324739,-0.0795725526925068
"Atp6v1d",5025.4048049261,0.695346664784836,0.188913292576574,3.680771
50792861,0.000232529310457728,0.00190418164439512,4.35544495249223
"Vrk1",792.07750626899,-0.430659414454778,0.160244150344446,-2.6875203
46478,0.00719847166020034,0.0263430556363713,-1.5661147442535
"Eif2s1",2049.84951481032,-0.747576516715438,0.273469187750331,-2.7336
7732162189,0.00626313893754401,0.0236343493331204,-2.79971453256873
"Plek2",187.890712389985,-0.478891687141876,0.275829438361545,-1.73618
773248621,0.0825306456557051,0.17022982428805,-0.847928421505207
"Pigh",892.820692937816,-0.314470565880472,0.203348104564692,-1.546464
2099992,0.121992497243269,0.229506919263726,-0.462844728863249
"Rdh12",194.522875067919,-0.812352889722015,0.266965781681726,-3.04291
016101267,0.00234302271213035,0.0112856365714223,-3.64277269960029
"Vti1b",3815.66172874962,-0.0509715602647671,0.277377840406987,-0.1837
62193079225,0.85420001654523,0.907244357914745,-0.00496174761956567
"Arg2",439.660463411926,-0.2019401448129,0.292235073286498,-0.69101953
6231111,0.489553263991391,0.62612722474937,-0.0945487179097021
"Zfp36l1",10346.6173336591,-0.906217834419957,0.167763614137893,-5.401
75436179559,6.59922775885295e-08,3.34212319070428e-06,-11.426413919338
8
"Galnt16",356.299362391159,-3.14667798295826,0.675643182094587,-4.6573
0738701918,3.20371923269976e-06,6.99461449050479e-05,-30.1067383124217
"Erh",2985.6688963595,0.00583273483887375,0.120258886174249,0.04850148
72865398,0.961316579155268,0.976507253256082,0.000138662193750559
"Susd6",3537.59353021747,0.0922341153100869,0.18955857901091,0.4865731
5216938,0.626560853442044,0.743242898364238,0.027368847814438
"Srsf5",28595.581071746,-0.205466530201138,0.0996604663154449,-2.06166
535033858,0.0392396075916175,0.0965174553830399,-0.480387199710625

"Slc10a1",15.0900633548053,2.26684690579198,0.861804907457409,2.630348
10567496,0.00852974772000573,0.029874801696546,7.95830989414663
"Smoc1",969.088955862469,-3.30200703359352,0.815913579851674,-4.047005
85347997,5.18769445102337e-05,0.000593949890816522,-24.5296711652488
"Gm20498",189.102606001571,0.533745492779194,0.32893613533699,1.622641
70895174,0.104666018921787,0.204077803693701,0.848257141870959
"Pcnx",2087.70790381427,-0.262374537978608,0.188492152303292,-1.391965
31405953,0.163932901735457,0.286463857146257,-0.328005666805529
"Pacs2",2867.01207113921,-0.279167127586296,0.135609404671142,-2.05861
18511713,0.0395314365467345,0.0970336600864708,-0.6512124185804
"Mta1",2149.55807706894,-0.0761891975172772,0.145559233593054,-0.52342
4008471234,0.600679220406333,0.722341398759263,-0.0247811003216004
"Wdr37",1470.03779640684,0.0495571271952172,0.109635341451288,0.452017
812314981,0.651256150432409,0.762404073950657,0.0134437872465143
"Gm9745",0.0983898136351889,-0.544048378937457,4.78471990596623,-0.113
705376621746,0.90947135024012,NA,NA
"Gtpbp4",2324.51876099758,0.155026904012957,0.150214195806391,1.032038
97062278,0.302053861670188,0.446722005254968,0.124923592211872
"Zmynd11",6180.23427845364,0.001543100005687,0.209852321369972,0.00735
326631420241,0.994132995208479,0.996250456664318,5.79679481352251e-06
"Esyt2",4182.1478053792,-0.586449740047314,0.109482224495889,-5.356574
93942619,8.48143316627542e-08,4.02362491182574e-06,-7.28565709040184
"Cdca7l",313.796252307168,-0.740107231923766,0.231682715078425,-3.1944
8618198919,0.00140079990874684,0.00762516117774309,-3.60898624102395
"Efcab11",28.3897692060736,0.449451128475892,0.461504333297515,0.97388
2791661996,0.330114738955635,0.474743854562396,0.334832045038581
"Tdp1",471.147459799878,0.20480687117545,0.165623600481141,1.236580237
23962,0.216242960596043,0.35153453774401,0.214114793440272
"Psmc1",6046.79736001865,0.11425489044476,0.175688631202833,0.65032603
2268146,0.515481645107095,0.649238493398239,0.0493529885128356
"Nrde2",1022.06774390194,0.327164845755795,0.140769136461721,2.3241234
1212848,0.0201188805831343,0.0577303802222154,0.933064889089295
"Rps6ka5",493.178092137662,-0.326787482096166,0.168779423928212,-1.936
18081215374,0.0528455596756645,0.121299604840142,-0.689355487924781
"Ccdc88c",2000.85706775566,-0.0532679032041864,0.286147380095388,-0.18
6155481089603,0.852322834597689,0.906089669537415,-0.00525312106796046
"Dglucy",3541.48575006037,1.29044665278066,0.346114687736465,3.7283787
6722301,0.000192715579077346,0.00164589065302238,8.27108376724168
"Fbln5",5725.92372465688,-0.703627530869042,0.340107155886821,-2.06884
071296398,0.0385610365421965,0.095348239825753,-1.65367907852032
"Tc2n",321.421252546633,-0.116720249532093,0.434922726349508,-0.268370
086134096,0.788414463013304,0.863526862140962,-0.0171263941293344
"Trip11",2509.72827936798,-0.0290418245733159,0.151315615814557,-0.191
928800057938,0.84779797762218,0.902791705035749,-0.00296991636556002
"Atxn3",1334.82726254609,-0.175465312304764,0.11362057698224,-1.544309
28767586,0.122513423646536,0.230153842784483,-0.257759826776117
"Lgmn",18495.3585346162,0.859669928876992,0.34653428345358,2.480764443
59119,0.0131100977934008,0.0416923380695072,2.73154781900584
"Golga5",1811.38543306914,-0.0802509632686701,0.112716238463477,-0.711
973397645573,0.47648124940274,0.614080996817676,-0.0391326522594503

"Pitrm1",1936.11454957333,0.0525605937432692,0.181205975713454,0.29005
9936138005,0.771770385108574,0.852431822854301,0.00839193194726929
"Chga",2.22871236743544,-1.34320742248464,1.45068427489231,-0.92591299
5496108,0.354491197145498,0.499593014957928,-0.932134213671953
"Pfkp",3730.30346536011,-0.495643543604049,0.180941054078705,-2.739254
20699968,0.0061578740050055,0.0233364873371423,-1.86249816347328
"Unc79",13.6221163784892,1.33204535615651,1.06422343031737,1.251659490
11221,0.210693967222921,0.344495679070232,1.41952574484811
"Asb2",452.810911056438,-1.32706348875602,0.289415048250443,-4.5853299
5011252,4.53270058781475e-06,9.20610375637625e-05,-12.3324789648245
"Otub2",288.104456993481,-0.370032535889513,0.172530522582676,-2.14473
665500082,0.0319739084224681,0.0825014353013835,-0.923208822712897
"Akr1c21",32849.2937239914,2.35761866868257,1.04604863752623,2.2538327
4171461,0.0242066921859597,0.0666829731904271,6.38397311189001
"Ifi27l2b",5.61565839551009,-1.6978064722952,0.850290165658042,-1.9967
3775008472,0.045853678997062,0.108712917447071,-3.76750837953332
"Ppp4r4",251.311315771369,0.268108034429195,0.338152961540094,0.792860
228720507,0.427859261183301,0.570849861403586,0.150309150936755
"Akr1c6",11.435041731672,1.87436795872765,0.504792351935902,3.71314650
774594,0.000204698307362594,0.00172555451662596,11.9251166125346
"Akr1c12",123.371043106111,0.629287842111365,0.481369127127043,1.30728
749861286,0.191115088369888,0.320966772951494,0.715133824716419
"Akr1c13",419.038091139418,-0.400448249782594,0.461782362632995,-0.867
179611406798,0.385843605646486,0.530107436237116,-0.254154726431138
"Akr1c18",571.188659029245,3.35416795276357,0.85578833013948,3.9193896
8391505,8.87734824560276e-05,0.000900904965479376,23.5197972683403
"Net1",6861.69256304541,0.439701509158732,0.307675661076692,1.42910722
16113,0.152973418196338,0.272216455963512,0.572121019811669
"Tubal3",0.366389463895641,-0.388130839671804,3.12953098703514,-0.1240
22047162892,0.901297818718233,NA,NA
"Tshz3",327.233391567284,-0.569708232979831,0.358314250606177,-1.58996
81132303,0.111841992767304,0.214529776190546,-0.876955719161411
"Gdi2",15862.8865973349,-0.136829349931524,0.179792507425098,-0.761040
334167027,0.44663297640771,0.587796616110073,-0.0727075975609087
"Rgs6",415.499508554722,1.28559366314876,0.366986303394755,3.503110746
25009,0.000459858227477898,0.00323576893517288,7.37093672347699
"Dpf3",71.9518447508901,0.320279733697629,0.374051420020344,0.85624520
2010487,0.39186216470206,0.536365636178362,0.199514798980139
"Dcaf4",655.218658510723,-0.403270401709445,0.168460804891448,-2.39385
299131927,0.0166724297282854,0.0500275522994935,-1.20786799803676
"Papln",160.826426683897,-0.793190557295636,0.385023948567282,-2.06010
706670894,0.039388306461104,0.096802738698987,-1.85216340078316
"Numb",2093.28979242859,-0.0919260031310711,0.162111360172729,-0.56705
4665589903,0.57067704204142,0.697376796331572,-0.0331328357131327
"Acot2",1472.54056167355,0.629925941129974,0.290493591662139,2.1684675
9863335,0.0301231272964189,0.0789330380359596,1.59947985863973
"Acot3",487.545740996715,2.27074498151337,0.656014625708883,3.46142432
275748,0.000537325165701579,0.00365936642712168,12.7399358192428
"Fam161b",233.913314716937,0.186599988497842,0.196628045894236,0.94899
9862401172,0.342620682060585,0.487123965204703,0.134209548679686

"Coq6",1202.72956042547,-0.386131826175689,0.338909436563945,-1.139336
30792495,0.254562910193399,0.395223739363557,-0.358447427496435
"Entpd5",10746.3011548659,1.03982167638969,0.331662006459789,3.1351847
8492277,0.00171745891221691,0.00892035531976702,4.90735469517046
"Aldh6a1",20892.1511911655,0.746003737847735,0.502585695512036,1.48433
141752613,0.137721019368223,0.251195924755997,1.03062062636723
"Vsx2",0.144352309981214,0,4.78471990596382,0,1,NA,NA
"Abcd4",2094.03139868358,0.410937505112986,0.190733493634681,2.1545114
9812246,0.0312000812455436,0.0810362573765298,1.0326278448254
"Isca2",1924.63270635662,0.0596260382689677,0.27545884522537,0.2164607
86438657,0.828628588443125,0.890759742941011,0.00689757210518513
"Npc2",13070.7499777434,-1.33198372211531,0.407278833675778,-3.2704467
1114841,0.00107377771038986,0.00621815200391489,-6.7668536228585
"Fcf1",1921.89624102639,0.223947826729637,0.172070347357435,1.30148994
390323,0.193090804417674,0.323332575106133,0.252853632951369
"Ylpm1",2478.48156627622,0.106978422584374,0.139341553287658,0.7677424
29019193,0.442640223602107,0.58408570699955,0.057523105335373
"Mlh3",809.048229927601,0.504025826477455,0.136467373153265,3.69337970
557541,0.000221293216293014,0.00183373358745482,3.1760689371159
"Tmed10",7783.40826740575,-0.159349276149525,0.180801463255985,-0.8813
49482907189,0.37812869108202,0.523177036858622,-0.103232097209588
"Fos",976.958343362158,0.196210559188107,0.385975836707443,0.508349333
113378,0.611208378580208,0.730856185793996,0.0615195790978501
"Erg28",1424.29407154968,0.144439957749811,0.187945860650898,0.7685189
62054198,0.442178929668655,0.583643559123388,0.0777758367831237
"Tgfb3",547.714277878934,-1.96030089749601,0.280350589317151,-6.992319
5177535,2.70378227172565e-12,1.50623847811733e-09,-39.8208670480096
"Gpatch2l",831.689388258447,-0.294504683535504,0.140564251634947,-2.09
516061238919,0.0361567173077156,0.0906731132016435,-0.706956844305535
"Esrrb",509.576649004723,2.27991519523726,0.577333586799036,3.94904306
170374,7.84642370326184e-05,0.000818563774029959,16.2055442976737
"Vash1",81.4143978288804,-1.44186717471521,0.396322270649989,-3.638117
9698796,0.000274637629834217,0.00215488310121029,-8.85309156313572
"Angel1",386.121524619924,0.154663062885376,0.151886671022265,1.018279
36476864,0.308545212034074,0.45347736660006,0.122309084223596
"Ccnk",1400.44997511884,-0.222422245325373,0.136644262128599,-1.627746
68954665,0.103578608633206,0.202626237697427,-0.355073135459694
"Cyp46a1",259.205516263718,0.666613776718984,0.311773156274175,2.13813
717859905,0.0325056112359204,0.0836311216837734,1.65409513489501
"Hhipl1",149.227028994544,-0.869828065354554,0.329692814307106,-2.6382
9852398396,0.0083323196232798,0.0293771536893514,-3.06835154715136
"Evl",1994.89489179758,-0.538879460988312,0.256545412057519,-2.1005226
9758576,0.0356828861150777,0.0897967105668282,-1.29881101400516
"Degs2",1153.20458418901,1.18335598385365,0.502426432653927,2.35528210
09096,0.0185086485795955,0.0541292302887229,3.45111683412499
"Yy1",3894.45209478332,-0.0864730052180692,0.0881827264278643,-0.98061
1608655651,0.326784307491041,0.471658925713252,-0.0649843916161803
"Slc25a29",235.900193185849,0.0835047905625116,0.188525688787729,0.442
935873086952,0.657812119073173,0.767155361531209,0.0221342758650628
"Wars",2205.73504336701,-0.31897067372845,0.154470972530745,-2.0649230

6290726,0.0389302805333457,0.0960231005489151,-0.747401392615484
"Hsp90aa1",33543.0994179292,0.739024872551518,0.241122369539197,3.0649
3700258442,0.0021771585086169,0.0106712510309231,3.3553221844783
"Zfp839",1221.63832355521,0.343721126843159,0.128462675000858,2.675649
77018315,0.0074584540298575,0.0270407096828126,1.24097480624378
"Fdft1",2389.65907987101,-1.09264779974138,0.405548679285407,-2.694245
73559619,0.00705481305279822,0.0259146090624453,-3.99138604584923
"Tecpr2",715.326638241692,0.0439616393380189,0.172542857021762,0.25478
6782233902,0.798887784690097,0.871619608633402,0.00604042503217907
"Cinp",831.65862128332,-0.0735581300133853,0.154841064688266,-0.475055
697669585,0.634747274471978,0.749472235630487,-0.0212131353219824
"Traf3",1430.30768892398,-0.593107095879597,0.153270406034503,-3.86967
785383228,0.000108979256734322,0.00106084750264893,-4.06200495176591
"Amn",4570.48397328874,1.94113148228181,0.729013767249902,2.6626815150
617,0.00775207469658727,0.0278053628465892,6.95415472046446
"Cdc42bpb",4571.50598833218,-0.0753644144972396,0.116705354551845,-0.6
45766552757094,0.518430552189027,0.651731715446914,-0.0322651850492474
"Exoc3l4",1120.89971322308,0.136833177391802,0.305973313122671,0.44720
6248137538,0.654726150498249,0.764834370763577,0.0366844244339856
"Tnfaip2",3541.96380243307,-0.821788735439418,0.33670053016383,-2.4407
1114185522,0.0146583742938502,0.0454708124840472,-2.53988982419205
"Eif5",15346.0393659727,0.421965985928185,0.239397707899135,1.76261497
919593,0.0779654489635027,0.163090904826776,0.765213177165072
"Ppp1r13b",1088.08036726582,-0.0891814665816951,0.134381699508929,-0.6
63642943254854,0.506918865402409,0.641478714798557,-0.039594722951086
"Zfyve21",1498.96464204804,0.204774642305786,0.226195507484699,0.90529
9334115368,0.365306829093236,0.510262379372442,0.137778566854885
"Xrcc3",193.4860141711,-1.12684369086693,0.530442611417167,-2.12434609
628434,0.0336412138340226,0.0858443119141177,-2.76664911026942
"Klc1",4585.84302495816,0.0362475264920115,0.195043591483828,0.1858432
06722416,0.852567720635088,0.90617957840098,0.0035710259283695
"Atp5mpl",6757.47824296727,0.791109710383814,0.367757769889056,2.15117
062141875,0.031462734978791,0.0815013161440769,1.98341972176307
"Kif26a",432.479128029078,-1.25297348044624,0.412719548885197,-3.03589
56434961,0.00239822352872268,0.0114693554974331,-5.59838141285364
"Gpr132",758.127668084539,-1.32237597889738,0.384105467236105,-3.44274
18813191,0.000575848646021983,0.00384122370856538,-7.35500797131517
"Hecw1",0.821279713962594,1.67849260364871,3.4826017414323,0.481965130
746874,0.629830716944854,NA,NA
"Ggps1",1437.80489625719,-0.348464234826014,0.115952399540271,-3.00523
521900029,0.00265375537637921,0.0123394484712482,-1.53148426733548
"Gng4",83.1900223771122,0.918363499928521,0.429671946296045,2.13735969
463495,0.032568747010979,0.0837764418496133,2.27717728856947
"Gpr137b",5674.01784093297,1.34884900795424,0.322242744283428,4.185816
53701366,2.84142788394201e-05,0.000374086163950717,10.6438004366357
"Mtr",1501.2825762378,0.0449737145251035,0.128962976172239,0.348733534
693229,0.727289365839181,0.82078416710843,0.00888208799281721
"Ryr2",52.7039886577076,0.40948934070022,0.563335625400298,0.726901197
504139,0.467286483555396,0.605720040137108,0.205292313053153
"Amph",98.4971380479473,-0.83931516957554,0.435957513987557,-1.9252223

9586744,0.054201541417803,0.123581354386139,-1.75488682232031
"Gli3",154.749921500098,-1.2412603134632,0.29554707906535,-4.19987339
204527,2.67064273232801e-05,0.000356658849280353,-9.85403152763452
"Sfrp4",423.330162367065,-2.05248690868216,0.553731193102038,-3.706648
52233444,0.00021002009243594,0.0017582532255543,-13.019816161215
"Aoah",1720.25483427731,1.27319388730469,0.454415886860175,2.801825209
28555,0.00508143967421586,0.020141880619609,4.97176358682999
"Trim27",1773.09965695778,-0.11466193604371,0.121833054690472,-0.94113
9794409817,0.346633224795075,0.491394687026469,-0.0814681805288631
"Zkscan3",4903.93735367269,0.145947460723391,0.153333577865368,0.95182
9747633865,0.341183332806739,0.485753386131128,0.10538197988661
"Slc17a1",13246.5869065232,2.36568629920632,1.02183083382691,2.3151447
5869403,0.0206050112333592,0.0587621045821322,6.70496558335699
"Slc17a4",118.300648375792,1.16923430147637,0.501262504321449,2.332578
82126879,0.0196702615689106,0.0567017681949467,3.35564384746838
"Scgn",1.32113291824061,-1.39343550794618,2.44215705388094,-0.57057571
532175,0.568287284050235,0.695266704173401,-0.506457735317717
"Carmil1",1126.49091795552,0.293832402390945,0.199441476806017,1.47327
630689746,0.140676546676598,0.255223631923683,0.401261974453065
"Mrs2",3310.65009755557,-0.0382559710604618,0.178113261044569,-0.21478
4518772519,0.829935323451643,0.891437692897741,-0.0043963660300007
"Gpld1",418.230062568832,-1.39381203861223,0.437763612793348,-3.183937
62724679,0.00145286314277961,0.00784707079166669,-6.75666409591395
"Prl7d1",0.586338482333666,-1.66206842211623,2.78709062415825,-0.59634
530994779,0.550944567785344,NA,NA
"Irf4",481.171906247828,-2.49183529078283,0.630421251347871,-3.9526511
6056156,7.72900730332249e-05,0.000808477384121147,-17.7427589785605
"Exoc2",1244.92664296195,-0.201705199258344,0.118585866950791,-1.70092
106626876,0.0889578102444263,0.180413915555006,-0.345420463772119
"Tfap2a",590.11885089794,0.578562951183421,0.264061676836652,2.1910144
5584366,0.0284507463698879,0.0756075579555778,1.49396469008789
"Gcnt2",2333.47427336498,0.511287829844396,0.123179184527692,4.1507648
5369533,3.31366071793255e-05,0.000419839241111228,3.97558942595094
"Tmem14c",4038.16000189886,0.0241448076908401,0.326861444878233,0.0738
686317067308,0.941114915907269,0.963500059352058,0.000897769853855804
"Gcm2",0.269028506467732,-1.57852747769588,4.76142694245881,-0.3315240
361287,0.740248688333899,NA,NA
"Mak",29.5256805250392,-0.497125974587043,0.554448513659046,-0.8966134
13761889,0.369925237874665,0.514979457952321,-0.329906848789652
"Elovl2",643.10914316039,1.93097882276282,0.630536719697482,3.06243675
021696,0.00219542866895024,0.0107384496840819,8.75491229011988
"Nedd9",1970.73904828309,-0.47647531313562,0.150807770316692,-3.159487
81773669,0.0015804670763655,0.00837189478915665,-2.27892188307187
"Hivep1",1307.70855794184,-0.971090102414391,0.202742543310166,-4.7897
6975704978,1.66972790748634e-06,4.07463763957055e-05,-9.81591824747742
"Edn1",858.552265653325,-0.692546148786955,0.394695913483277,-1.754632
17410863,0.0793222444049439,0.165237726646111,-1.24683936144408
"Tbc1d7",490.380314462412,0.0133152368396544,0.171977388837479,0.07742
43458960611,0.938285973686634,0.961765319889706,0.00051909195560612
"Mcur1",5857.98340225378,0.722019309032006,0.238144450123545,3.0318544

4236654,0.00243056377851956,0.0115757529442048,3.21937047620305
"Cap2",185.3300673812,-1.72675910222296,0.376721037497858,-4.583654562
25093,4.56918852145055e-06,9.27055544133639e-05,-16.0348269093598
"Nup153",1829.80160294601,-0.319330108575737,0.206808885167991,-1.5440
8311962199,0.122568197667786,0.230190205945529,-0.469047815854216
"Kif13a",1393.39056191713,-0.252794558443411,0.162360205212176,-1.5569
982688372,0.119470896428974,0.22578690884677,-0.376199661597249
"Tpmt",2913.78481109265,1.64061946921718,0.346774293329063,4.731087340
60733,2.23320404786169e-06,5.21473203894698e-05,16.1788667844973
"Dek",5570.05985039771,0.357171733545927,0.183305103552627,1.948509488
40812,0.0513540306023845,0.118770599889683,0.760976300933705
"Id4",2115.73753234499,0.49512877675318,0.238742163638549,2.0739058790
7545,0.0380880529442087,0.094442484103661,1.16838719326158
"Barx1",0.190062432325283,0.43663188201043,4.78471990596623,0.09125547
37981588,0.927289597068987,NA,NA
"Susd3",1552.20389535671,2.08123078088636,0.500292153278927,4.16003082
847877,3.18204640638544e-05,0.000407912825980955,16.2428762784449
"Ippk",592.152019120932,0.0794171682843833,0.136734378866525,0.5808134
64343942,0.561366179523356,0.689516725650283,0.0295244699123783
"Aspn",250.591083973892,-1.7932148065481,0.577159191563974,-3.10696742
382095,0.00189017226443977,0.0095602020264712б,-8.3387114123618
"Ogn",1996.10279317096,-1.63677446786955,0.27509077087212,-5.949943223
03321,2.68235520401241e-09,3.09850777959792e-07,-24.530625412956
"Cenpp",29.6569034241026,0.244352137370154,0.38101783276727,0.64131417
575777,0.521318580933204,0.65472555947877,0.103492690285332
"Nol8",1344.58120000232,0.28274360304907,0.12518039245229,2.2586892204
9299,0.0239027234715263,0.0660134989019576,0.768467715966736
"Spin1",3959.52807399021,-0.192463883520034,0.135515509956515,-1.42023
509767844,0.155539248898938,0.275750525098336,-0.24794327557446
"Nxnl2",72.0581058245672,-0.0746661570157446,0.36995240576937,-0.20182
638591163,0.840052453028768,0.897645789946562,-0.00806243160609371
"Wrnip1",1751.14965416919,0.0169313907292862,0.108286010551275,0.15635
8061794778,0.875750792367545,0.922395686559118,0.00136773444358903
"Serpinb9b",16.7539872357485,-1.73627479534439,0.606412246199528,-2.86
319217038553,0.00419396114011583,0.01738759394216,-7.03538255186845
"Serpinb9c",0.239211507279182,-0.0926397567699961,4.78471990596078,-0.
0193615840824007,0.984552656120711,NA,NA
"Ripk1",1535.48309100791,0.0353151567377692,0.152165618116554,0.232083
680761044,0.816473019731989,0.882692059184005,0.00440658581245434
"Pxdc1",564.899147747745,-0.839595075317351,0.266441390521546,-3.15114
357297823,0.00162632524718593,0.00857029450530573,-3.9960134530484
"Prpf4b",5783.23447732241,0.235628462319111,0.20993433934783,1.1223912
3456935,0.261696129747982,0.403277737435626,0.2139812235602
"Fam217a",246.726840844026,1.23632554679773,0.229418735637493,5.388947
60866987,7.08714605894925e-08,3.52513198615797e-06,15.5227999848763
"4933417A18Rik",0.142425137392067,2.57912268336951,4.72883040948399,0.
545403928674817,0.585475739019112,NA,NA
"Eci3",6920.08234128948,2.21357936702172,0.711108365227634,3.112858004
85995,0.00185285139922802,0.00941294856231057,10.3278287003058
"Eci2",5025.15983084517,0.625478770212885,0.211432858264916,2.95828555

384324,0.00309355404260873,0.0138344304410057,2.67742119290649
"Rpp40",368.567816369769,0.976091226063154,0.289645334781113,3.3699532
1122915,0.00075180947963377,0.00471646757847466,5.22862314047783
"Fars2",1401.22667997004,1.06381648750098,0.258276577311682,4.11890423
271792,3.80678303884193e-05,0.000468294357674069,8.15565707106188
"Ly86",1270.26894681928,-1.26681228129268,0.416546035552222,-3.0412299
5580367,0.002356138093756,0.0113215439518149,-5.67664641650872
"Ssr1",6386.56629561696,-0.165352718348359,0.108340138746264,-1.526236
90777819,0.12695087491438,0.236056471398111,-0.238717109716835
"Riok1",1247.29970279711,0.193662228039808,0.263888219675507,0.7338797
77876963,0.463022012815088,0.602204477271011,0.0982173924235229
"Snrnp48",2831.47398333859,0.409736309282411,0.22622986154356,1.811150
42234828,0.070117570171692,0.150286070485616,0.776538261967486
"Slc35b3",1225.65664344041,0.205753108106218,0.101487501468167,2.02737
386505426,0.0426241926289999,0.102897212847041,0.467887654573716
"Shc3",11.0765702353576,0.111601423297504,0.556862005197637,0.20041127
2911132,0.841158943824857,0.898286618835418,0.0119710479758037
"Sema4d",2874.52447333893,0.540828801935151,0.37155820625348,1.4555695
254008,0.145511657558545,0.261878004345256,0.724643810852236
"Gadd45g",3395.56639624912,0.731279662954889,0.402780636906592,1.81557
799940735,0.0694351309914054,0.14920059335138,1.39123295067444
"Fbp2",479.340181705204,0.878015169440878,0.302448842331385,2.90302043
371309,0.00369582522178247,0.0158271909014528,3.64027361109625
"Syk",1574.28667632055,-1.30286875779926,0.28596488594012,-4.556044542
0284,5.21258874103214e-06,0.000102558077974414,-11.9669554707385
"Aopep",6601.51402515251,0.139139199359634,0.242608784170892,0.5735126
19648701,0.566297656983776,0.693790242907299,0.0508672887680295
"Auh",3246.21854943811,0.743053188101828,0.277690159975292,2.675835500
14139,0.00745432227131852,0.0270307561179409,2.68300155025955
"Fancc",864.815362452752,-0.0289447717648646,0.150935725625264,-0.1917
6885820079,0.847923265738561,0.902821162359775,-0.00295904698541143
"Ror2",214.959029486952,-0.371118150807197,0.400938677757788,-0.925623
222191084,0.354641820783464,0.499710421278564,-0.257454497990741
"Ptch1",1473.44482467509,-0.441235593436646,0.190031753479955,-2.32190
455203682,0.0202380756584751,0.0580041157120311,-1.25630420925213
"Sptlc1",2826.80186715471,0.11539194312307,0.176905716162577,0.6522793
36282296,0.514220987787022,0.647901830040152,0.0500819600823557
"Msx2",129.074854410823,-2.8110200081948,0.751291375283719,-3.74158429
162485,0.000182863822570833,0.001579042875326,-18.1337112655946
"Ercc6l2",916.712552931807,-0.262405791675791,0.164927945385019,-1.591
03292691371,0.111602165754102,0.21417510116865,-0.404357187913304
"Sfxn1",9293.19856319996,0.55302516889253,0.349405238326112,1.58276152
796648,0.11347582853997,0.217108400046349,0.844667698386785
"Habp4",3459.34961144349,0.370957668831634,0.179474768375143,2.0669070
766329,0.0387429107994165,0.0956865787173977,0.870517916093446
"Ctsl",23684.1924375303,0.309510361033788,0.231101847202412,1.33928120
774691,0.180479144115035,0.308196037130405,0.364299641958322
"Drd1",25.8819402712671,-0.127904664573782,0.504427343926607,-0.253564
098207157,0.799832335758786,0.872162120833555,-0.0174947941325324
"Zfp346",1074.5279152637,-0.225908363000498,0.129473858529685,-1.74481

834067457,0.0810164952219498,0.16787613438279,-0.403139995038414
"Prxl2c",2616.63727156069,-0.0194960578341335,0.167425018001418,-0.116446502839667,0.907298682106649,0.943127331475567,-0.00114157172957999
"Cdk20",479.079584545844,-0.322901039299457,0.200624172597086,-1.60948222300182,0.107510939072773,0.208070169916272,-0.506915851545474
"Lman2",6872.47299579907,-0.027874405178774,0.167959206350473,-0.165959376591777,0.86818891954925,0.91740448479142,-0.0024029635021107l
"Mxd3",67.1060275140862,-0.707224047305566,0.449316700754633,-1.5739990214425,0.115487675668297,0.219878791151299,-1.07121729518717
"Prelid1",10636.4759937575,0.861122629850901,0.160115748561535,5.37812574707452,7.52652430992437e-08,3.66880427487263e-06,10.7775049297069
"Nsd1",5100.43331360728,-0.0106259802193724,0.124788051261501,-0.0851522250083465,0.932140371485622,0.957529791013469,-0.000461151018985964
"Slc34a1",124078.55760226,2.22777586776242,0.881554543296556,2.52709929828243,0.0115008980238872,0.0378088871471509,7.29643623765929
"F12",4.04582781456314,0.803290807723905,0.943573390627343,0.851328381769891,0.394586963147304,0.538908617016278,0.496601819150075
"Pdlim7",2654.52417083647,-0.889987151576864,0.27693928872571,-3.21365435605758,0.00131057334034892,0.00722668523476335,-4.38761424890691
"Ddx41",2403.33498313192,0.213921068804261,0.139112310540943,1.53775800267009,0.124107795717168,0.232410084603664,0.312164717897202
"Fam193b",10834.9978370708,0.480228395970228,0.273632267197335,1.75501376679345,0.0792569529871169,0.165137002494422,0.864881655635307
"Pcbd2",1106.47053893298,0.583229696631662,0.245209835617112,2.37849226220419,0.0173836017646542,0.05162916484497,1.72849951282795
"Txndc15",2568.76888598113,-0.00518806332170352,0.160241447337984,-0.0323765380798189,0.974171772577136,0.984077077375299,-8.32738862525278e-05
"Catsper3",14.2177978279739,0.183233544612483,0.545542719007187,0.335873870603466,0.736965981804211,0.827995797237524,0.0345848185667796
"Ddx46",5273.40907983437,0.325352795094285,0.157746079525933,2.06250954744518,0.039159249529355,0.0963805598624973,0.761146843386859
"Caml",1805.33823277151,0.385280035795812,0.199183436191112,1.9342975659188,0.0530765523462841,0.121636885007974,0.811675240161829
"B4galt7",864.84490445536,-0.183122096596833,0.0984626198709013,-1.85981336711264,0.0629119355127232,0.138355167902896,-0.362202911859884
"Pitx1",4.73571519338605,-1.97568237352926,1.44058893700498,-1.37144075091729,0.170237601437059,0.294681529902323,-2.41400739420855
"Cxcl14",3008.1609396531,-0.804358319084658,0.41282975247713,-1.94840200895941,0.0513668796333968,0.118770599889683,-1.71373476900178
"Slc25a48",1040.28887835768,0.119449055364333,0.419417276944019,0.284797651242859,0.775799171778959,0.854965357073935,0.0187169895690608
"Zfp729a",961.029709864443,0.506639580702342,0.278464567560123,1.81940411716099,0.0688497990511522,0.148269646774836,0.967034481205518
"Zfp369",1660.51268942141,0.368023979303209,0.129937159221691,2.83232280517469,0.00462111706373133,0.0186624980340997,1.46519150083841
"Ptdss1",3109.35902731008,0.119242223943493,0.144721792617154,0.823941037400879,0.40997306140619,0.553513680875572,0.0705280563107123
"Mterf3",1875.87600485589,0.26543151686056,0.184334412988843,1.43994554547244,0.149882805943851,0.267816619345052,0.349693492138372

"Uqcrb",9557.90986860069,0.760810389417396,0.318531450567698,2.3884937
8314591,0.0169175945493707,0.0505221716225503,2.27127996810795
"Fastkd3",476.384122374011,0.564063198205857,0.246348727183261,2.28969
398240992,0.0220390624977721,0.062115877505285,1.56739266876309
"1700001L19Rik",13.6641468694104,0.676510176494809,0.632217217172727,1
.07005971700701,0.284592429759181,0.428724653901299,0.572963798479706
"Adcy2",49.1664302832389,-1.88645982670489,0.367972365933112,-5.126634
50126523,2.94967354169667e-07,1.05527953607342e-05,-21.6171690955726
"Cetn3",2345.74699759512,0.0442682396344902,0.194317765781306,0.227813
650782254,0.819791113930046,0.884526984638815,0.00543181293843749
"Lect2",8.54776257160669,-0.65952117586,0.824710742614485,-0.799699993
926593,0.423884636474977,0.567060215573866,-0.374139623145833
"Smad5",3075.81318803604,-0.34533729603153,0.0870784928472981,-3.96581
618192587,7.314521852432e-05,0.000776698269304374,-2.47277341500246
"Trpc7",0.63713753980554,-1.11484908613566,2.53449216226137,-0.4398707
96499505,0.660030688169835,NA,NA
"1700067P10Rik",0.120217854293175,-0.0675307986870953,4.78471990596051
,-0.0141138457452796,0.988739154248813,NA,NA
"Hnrnpk",17126.5202214069,-0.19797401444772,0.125767875648712,-1.57412
227428163,0.115459183962083,0.219846012587176,-0.299896564616829
"Ccnh",1181.12582514754,0.162630177298469,0.180632723345561,0.90033618
6524455,0.367941369209404,0.512986829949586,0.108556473859005
"Rasa1",2282.33382066495,0.122422601452334,0.269151331205126,0.4548467
24718717,0.649219512655576,0.760908996018178,0.0334509368467566
"2210016F16Rik",2333.11385424331,0.175866089127987,0.212181660184318,0
.828846795595886,0.407191104692922,0.55108018041942,0.104794199248924
"Gkap1",1963.9527546279,0.770857573323073,0.15042569446539,5.124507326
10999,2.98316750921718e-07,1.05948634052307e-05,8.83028239276022
"Slc28a3",20.7125600580422,0.320677217331514,0.432634257862531,0.74122
0121855004,0.458559984845778,0.598340532993574,0.164698290455223
"Naa35",1989.07488960625,0.511009203023764,0.290141828091222,1.7612393
4417032,0.0781979014609348,0.163435501683454,0.925609800939113
"Golm1",1141.98861558638,-0.602179824986652,0.310501219573427,-1.93937
990264237,0.0524550958646691,0.120609606034118,-1.27372856534848
"Agtpbp1",2110.85574332442,0.250410115512928,0.268131256487795,0.93390
8708716046,0.350350986193401,0.495306507749544,0.175932763459222
"Dapk1",3777.40825778456,-0.234602602890034,0.172081082280642,-1.36332
593787054,0.172779810953205,0.298208440644914,-0.283860368729055
"Slc6a19",5202.66118908871,1.92041440294057,0.839451995040626,2.287700
0880171,0.0221549925807628,0.0623614905932891,5.3287799658401
"Nkd2",1057.00366519811,-1.90028233431347,0.391984169908645,-4.8478547
864735,1.24803699945967e-06,3.27954520424053e-05,-19.6208345120207
"Trip13",110.737128320784,-0.648884303452107,0.37431455807383,-1.73352
676099795,0.0830020852421212,0.170908323241829,-1.14633717121667
"Cep72",229.162284226631,0.446560385958653,0.302242749391774,1.4774891
6014462,0.13954456186606,0.253691362915099,0.612518684452497
"Tppp",413.868610046934,-2.50015836180752,0.629844091343986,-3.9694876
8142381,7.2027330243307e-05,0.000767845207809732,-17.9309417522495
"Ahrr",43.276524571061,-2.27030005983008,0.636752715254237,-3.56543443
858517,0.000363253963183509,0.00268488467556939,-13.4404431219761

"Pdcd6",3123.77978256432,0.256381173969333,0.179505392342556,1.4282644
6951562,0.153215750489451,0.272498467850344,0.333326878363251
"Sdha",23675.7813993269,-0.0349537316914928,0.446107361483419,-0.07835
27345867212,0.937547470222967,0.961159922940607,-0.00138467358370306
"Ccdc127",1870.79721163843,-1.21188818709103,0.264563026440663,-4.5807
1637369493,4.63385919436367e-06,9.35310419996925e-05,-11.2429499056088
"Lrrc14b",76.3490324526629,0.0575929555116201,0.242260279200673,0.2377
31730936848,0.812089171368829,0.880220524196152,0.00734787094063143
"Erap1",2088.18111180301,-0.761861758943923,0.213853174156412,-3.56254
59474672,0.00036727560088344,0.00270947395612006,-4.50336544761033
"Cast",5135.53045378464,-0.736191863145538,0.219974889329008,-3.346708
64202343,0.000817771130216073,0.00504266334501992,-3.89432310039822
"Pcsk1",3.35241917954593,-0.235945881796085,0.901476602767645,-0.26173
267400585,0.793527548172769,0.867421659150791,-0.0335586010992988
"Rhobtb3",469.018252966899,-0.662523789874965,0.173278956373603,-3.823
4521013995,0.000131596165479197,0.00123052468075979,-4.43911784257177
"Spata9",6.19054827692065,-0.434269415320409,0.733071034832127,-0.5923
97454934032,0.553584470204851,0.683281002725466,-0.165391107222075
"Glrx",1811.34489460192,0.561952265766705,0.138607246497449,4.05427768
004212,5.02894515470225e-05,0.000581514980048513,4.1864734757983
"Arsk",891.656105638278,0.0827122200503622,0.122895935550463,0.6730264
89280433,0.500930438820343,0.636088680958584,0.0374204384361989
"Srd5a1",2469.7945553051,-0.345388504529178,0.233650067599079,-1.47822
985064071,0.139346266598629,0.253425380667855,-0.474109914861657
"Nsun2",3040.64828891051,-0.109476977789261,0.0995824400133419,-1.0993
6026647463,0.271610954652955,0.41412809043458,-0.0965127092433427
"Mctp1",84.4124857864242,-1.42348729376442,0.362409793639611,-3.927838
92363562,8.57125836355561e-05,0.000877743674225879,-10.0187255659985
"Slf1",544.026263138419,0.27157229989682,0.143495142396107,1.892553959
4028,0.0584172114059626,0.130910336859408,0.552172380422091
"Med10",1753.92215745016,0.0814509603535478,0.12821632290934,0.6352620
20508421,0.525257539412505,0.65797014208407,0.0340950238384691
"Irx4",35.3821628177564,1.18805891736721,0.639783837730806,1.856969256
3367,0.0633155281644503,0.138991912649229,2.34444362551914
"Ndufs6",5285.90034447451,0.634759840737066,0.395885058325577,1.603394
28677057,0.108847631601909,0.210129813957825,0.9902442531389
"Mrpl36",2149.2493721054,0.357745487270615,0.167550268750206,2.1351531
6889144,0.0327485008034427,0.0841025214176236,0.885677203862486
"Lpcat1",1373.30890152598,-0.847882307119945,0.224901404976467,-3.7700
178316301,0.000163235884023654,0.00145199510341843,-5.54075548978165
"Slc6a3",2.18619118578542,-0.181285901425897,1.02279676172878,-0.17724
5282943094,0.859315728062625,0.910990542940684,-0.0168999725769657
"Clptm1l",4815.50439781658,0.0548504832569099,0.0968835054790936,0.566
148829830957,0.571292611545362,0.697652795512507,0.0197480239482194
"Tert",180.669748603598,-0.355281417799006,0.403684470571486,-0.880096
817437745,0.378806863076287,0.523605289682507,-0.229873167717581
"Slc6a18",18270.1605638733,2.08233107365837,0.734704776329406,2.834241
91695298,0.00459345441728137,0.0185882986457206,8.29855369106795
"Hapln1",59.1180051925904,-0.712164792366006,0.547729222625894,-1.3002
1324944429,0.193527890787749,0.323730852314648,-0.80320994142206

"Vcan",286.656207173816,-1.87288301386697,0.549617881814312,-3.4076093
1519277,0.000655346523924032,0.00425081388006346,-10.2271489153814
"Xrcc4",469.766694524402,-0.053720323568061,0.24873253624111,-0.215976
262614824,0.829006250593914,0.891010752359036,-0.00619926000730552
"Atg10",554.734351856614,0.0365976227434948,0.295231622054015,0.123962
407850738,0.901345039617113,0.939654974297799,0.00227792825518104
"Acot12",2836.67795854994,2.17341635933257,0.83749591097818,2.59513668
167533,0.00945533094244463,0.0323950171702369,7.45427619081743
"Zcchc9",1221.22440047567,0.134053885557594,0.104430375964781,1.283667
55667722,0.19925830083206,0.330678215135204,0.148345308118834
"Ckmt2",0.118149636523694,0,4.78471990596588,0,1,NA,NA
"Cd180",327.45342017721,-1.50129222248206,0.504148625388256,-2.9778762
5886292,0.00290253141446248,0.0131919714496945,-6.49781321558663
"Slc30a5",3426.00285534168,0.672586304420633,0.1353686083278,4.9685544
7307208,6.74538521270177e-07,2.00796215079785e-05,7.2745623899654
"Rad17",861.895687174821,-0.0295227883721389,0.116950907479844,-0.2524
37445833646,0.800702959515092,0.872738410179888,-0.00401862461570872
"Marveld2",878.400647692264,0.289230047630979,0.175554384749093,1.6475
239171289,0.0994504043395976,0.196482316730517,0.470630171160781
"Ocln",984.127757284441,0.363069984080969,0.33704234626662,1.077223642
97144,0.281380365200334,0.425364735670346,0.31035522735016
"Gtf2h2",1004.51899665383,0.719157258395286,0.15140084711461,4.7500213
6448343,2.03395159039122e-06,4.81871058438007e-05,7.14872453741084
"Naip1",60.2257499980512,-1.25151731550253,0.512919504260037,-2.439987
76632218,0.0146877592992294,0.0455204433586897,-3.86668007517456
"Serf1",468.993589067154,0.170635836732532,0.15770394943832,1.08200103
637398,0.279252067438627,0.422979087640195,0.14682062630573
"Smn1",838.585341381352,0.228024330649299,0.0908338582131163,2.5103450
9746689,0.0120613226146056,0.0391040353075458,0.739147620174641
"Mccc2",2977.59263695088,0.73838604914218,0.388715555711979,1.89955364
093865,0.0574917209028792,0.129442676000501,1.50964294242419
"Cartpt",2.7745221043703,1.53150743460721,1.34534890596362,1.138371932
97471,0.254965208744154,0.395679054448705,1.41993997410237
"Ptcd2",1187.77451127993,0.140231704847107,0.213701214513406,0.6562045
29143236,0.51169253514046,0.645884705779031,0.0613000832496592
"Btf3",12382.993681891,-0.103849282103308,0.231984173927675,-0.4476567
53239059,0.654400937428157,0.764637710671433,-0.0278682807472626
"Ankra2",1011.92724865799,0.0778275678666823,0.102343417067176,0.76045
5045345982,0.44698263091824,0.587981074053183,0.04133114855156
"Arhgef28",3206.49406202122,0.711844281019747,0.1685435084225,4.223504
58752357,2.40532472153779e-05,0.000331676247670041,5.70283455093708
"Hexb",9519.07581277806,0.662856674743076,0.284683443289258,2.32839910
563245,0.0198909211070232,0.0572194127684771,1.8963415080859
"Gfm2",2547.79351576184,0.463743952119972,0.174929587746144,2.65103209
865762,0.00802462146447958,0.0285663086450813,1.64885427036464
"Polk",698.043079041307,-0.0680620924621635,0.118153485159672,-0.57604
8115467646,0.564582659797835,0.69234167929171,-0.0250247767421729
"Col4a3bp",3261.45965121371,0.162366483680754,0.126849580879557,1.2799
9227553554,0.200547852576767,0.332111604343622,0.178974011014691
"Hmgcr",1615.33522377291,0.209168408290434,0.192635769518842,1.0858233

0692211,0.277557172787375,0.421086971289157,0.180913078761484
"Poc5",574.453888931374,0.0736611789132351,0.147645398978843,0.4989060
23639723,0.617845590299311,0.736323602054599,0.022546624684262
"F2rl2",29.6723504311004,-1.27792214593102,0.56564486963778,-2.2592305
0756096,0.0238690500574356,0.065948524588335,-3.47451795035191
"Iqgap2",1843.80259631113,0.505222377553368,0.432089448442274,1.169254
14257336,0.242301253047508,0.381039595232131,0.487464813728255
"F2rl1",1346.6907216727,0.0702512598445632,0.332817885371722,0.2110801
82082462,0.83282470424349,0.893005723896809,0.00794979332610251
"S100z",1.45773071062102,1.1362689642311,1.32040792456333,0.8605438842
74906,0.389489303762824,0.533679722049722,0.713530767244418
"Crhbp",2.16147189364719,-2.14734615778181,1.23483448910287,-1.7389748
8022213,0.0820391815347554,0.169471964717614,-3.81168494655938
"Aggf1",2119.50991269781,-0.308868137982256,0.141397898024308,-2.18438
988342781,0.0289336109715389,0.0765250932379314,-0.793833298512947
"Pde8b",644.080246646217,0.384302000021413,0.449604845587694,0.8547550
22755769,0.392686782577944,0.537192653245265,0.238804682686609
"Ap3b1",2584.42987600338,0.382124566113544,0.134761225257384,2.8355676
1511861,0.00457443308381125,0.0185469528525997,1.52370251330977
"Scamp1",2417.85539756366,-0.134264266909935,0.184765332565263,-0.7266
74560892046,0.4674253407798,0.605773299735762,-0.0672998907150471
"Jmy",1629.4849468501,0.139916671823262,0.141162195448892,0.9911766488
06936,0.321599330770023,0.466870951630847,0.10657486393563
"Dimt1",487.645982931837,-0.29188306576576,0.165778218984959,-1.760684
04856154,0.0782918940770011,0.163580727680387,-0.528439306231375
"Kif2a",1254.05318080875,-0.455557644076041,0.151177660217059,-3.01339
260987342,0.00258344456919927,0.0121290638599199,-2.0099889939667
"Ercc8",282.180226819048,-0.0778539482099226,0.127007801553095,-0.6129
8555882314,0.5398858884902,0.670726059780292,-0.0310944373193827
"Elovl7",2396.62517333257,1.09484088225093,0.443082115060738,2.4709660
9191921,0.0134748594738956,0.0426370062150508,3.45425879827151
"Depdc1b",424.852787027945,1.36327222128726,0.581028149727432,2.346310
1089453,0.0189603248308476,0.0551691409568523,3.94987884614304
"Pde4d",804.262634908252,0.818088935839478,0.347350136390329,2.3552284
8599133,0.0185113194976513,0.0541292302887229,2.38585897803359
"Rab3c",262.683049350198,1.12238564565324,0.483131241354142,2.32314855
588177,0.0201711732814271,0.0578634010065126,3.19842892871275
"Plk2",1238.33535174086,-0.684357346745103,0.196826687970556,-3.476954
03403566,0.000507144754383426,0.00350152564481871,-3.86973728811197
"Thbs4",154.304088385402,-2.69450346530176,0.731866536846951,-3.681686
93285295,0.000231695814848981,0.00189974978886687,-16.8838478788816
"Serinc5",1387.58663242794,-1.10818168191747,0.241345517787725,-4.5916
8122149329,4.39689611290287e-06,8.9958741248038e-05,-10.3239972187491
"Mtx3",1424.57901279137,-0.0841181628162112,0.173364727986169,-0.48520
9210623987,0.62752794913608,0.743892263634198,-0.0248871207124583
"Zfyve16",1171.23789875486,-0.0366550442072209,0.106770372800155,-0.34
3307260674543,0.731367319152242,0.823975318814203,-0.00709695547625012
"Dhfr",1933.35055160239,1.75993913319491,0.360816713431343,4.877654132
08829,1.07354953321551e-06,2.9440321798363e-05,18.3617007547583
"Rasgrf2",196.319572345443,-1.01442819101776,0.331833444439844,-3.0570

4023514018,0.00223534268893807,0.0108906675428905,-4.58506229060734
"Erbin",6512.91345502045,0.287965156059504,0.167598165182224,1.7181879
9893429,0.0857623298126476,0.175327009006883,0.501376831780095
"Nln",1407.90561537332,0.145194305571558,0.145871839214963,0.995355281
409687,0.319563499822936,0.464665729102311,0.111282287114808
"Trappc13",1563.94606128583,-0.128038217763957,0.175289333734706,-0.73
0439297337613,0.465121705046635,0.603906352529865,-0.0645743003337023
"Trim23",968.030867526815,-0.0910771424107979,0.177523645476257,-0.513
042317075329,0.607921719816134,0.728087081878069,-0.0289019303753147
"Ppwd1",740.097418555226,-0.0680118295256717,0.201773788635635,-0.3370
6969565055,0.736064361388619,0.827573689214352,-0.0128717233414415
"Cenpk",79.2766307195777,-0.933906126577992,0.448552946845647,-2.08204
211597647,0.0373386229672079,0.0929674972052892,-2.21849899012073
"Cwc27",1045.26892215185,-0.0447333690689697,0.112380514145297,-0.3980
52717672512,0.690591326468027,0.792907177215498,-0.0103803387879765
"Srek1ip1",1818.45049622312,0.646496711595734,0.133000186044185,4.8608
7073127067,1.16870554397675e-06,3.10951183951186e-05,6.70964007774944
"Rgs7bp",228.335853521424,-0.222732078852325,0.209732400858726,-1.0619
8221133394,0.288243760877962,0.432321296122962,-0.186780053447527
"Rnf180",286.347781384945,-0.953162751999492,0.430186174807744,-2.2156
9824373243,0.0267121752700611,0.0720725602674638,-2.50689608839076
"Htr1a",0.39425997266837,-1.93147266646656,4.75105661583361,-0.4065353
92575546,0.684349257344212,NA,NA
"Parp8",2223.70482904937,0.22612331877876,0.386989774787233,0.58431341
9916801,0.559009464988003,0.687686419683884,0.084665619950888
"Emb",1315.0587036475,0.311085929702214,0.336528769340118,0.9243962419
97993,0.355280050603578,0.500379256477071,0.215392461822587
"Hcn1",0.649899978678065,0.845435098454681,1.74096377741864,0.48561326
1700495,0.627241392011203,NA,NA
"Mrps30",1449.99037756669,0.228577022420683,0.272223165191635,0.839667
786023181,0.401094680774428,0.545137605307389,0.138681572028922
"Fgf10",168.603383381852,-3.06197664403484,0.891802538397935,-3.433469
30760646,0.000595909666077083,0.00393999547954254,-16.9528654905081
"Slc4a7",1002.32416146768,-0.270190809951418,0.215464658929879,-1.2539
9131019138,0.209845163964419,0.343597666021014,-0.28864048723194
"Psmd6",3419.04630558,0.305901663091619,0.12375522782713,2.47182820849
333,0.0134424095737221,0.0425487178357846,0.965763840605997
"Atxn7",2079.80462551471,0.23322413827857,0.196505621678187,1.18685733
408948,0.235283899111197,0.373202466609375,0.229873687111692
"Fezf2",2.7790433839095,-1.6037221915402,1.54268759000592,-1.039563811
83701,0.29854259703475,0.442873894919284,-1.30618395179737
"Ptprg",2686.88072209535,-0.271028237061678,0.200774240256177,-1.34991
539111721,0.177043124133096,0.303551427571852,-0.323121011892688
"4930452B06Rik",159.259244324781,-0.489196591261455,0.258504188071711,
-1.8924126332752,0.0584360241086647,0.130933762131779,-0.9945678398010
15
"Pdhb",11033.2002486746,-1.13738899018337,0.54158212967839,-2.10012282
13693,0.0357180382679131,0.0898735720928855,-2.74036969984695
"Oit1",710.88935786016,-1.17013453531518,0.340856437579852,-3.43292485
136372,0.000597107572354576,0.0039465774392439,-6.47658530009384

"Fam107a",1836.84033792596,1.59084756939409,0.509752302972709,3.120824
6831192,0.0018034536621125,0.00922690619361572,7.45412544617759
"Acox2",2126.1988000239,1.94449219746486,0.721336796082062,2.695678645
57356,NA,NA,NA
"Kctd6",1111.32263060265,0.478618864329278,0.150764905708128,3.1746039
4434138,0.0015004114075934 8,0.00804593554049989,2.30818169548069
"Map3k1",4953.530374478,-0.283592837965268,0.220160511481958,-1.288118
54612951,0.197704700336884,0.328715464071678,-0.31551482861202
"Il6st",8302.79186392582,-0.945669169152193,0.166032400853038,-5.69569
050554923,1.2287359728306e-08,9.32213774251015e-07,-13.131281859368
"Ddx4",25.0302688668897,0.561179151808666,0.366893996531563,1.52954029
532721,0.126130557615176,0.235091636782402,0.812463894655381
"Plpp1",2530.04965219901,-0.569454132366067,0.210554518996799,-2.70454
481375782,0.00683980431269962,0.025330010349291,-2.09317980027715
"Gpx8",2211.84234220662,-1.17173732929195,0.327299834153866,-3.5800119
860162,0.000343578447365136,0.002574593607504,-6.9859724558882
"BC067074",625.178149529011,0.196919587513863,0.186163952405216,1.0577
7506853333,0.290158001726376,0.433923969754631,0.164405395935247
"Ndufs4",4952.40527785832,0.631644394882864,0.397203659999852,1.590228
03285121,0.111783413833322,0.214446013575439,0.97254114236166
"Fst",532.406630798098,0.291243446859274,0.608686334150176,0.478478701
622071,0.632309529785557,0.747606040371038,0.0847166386204525
"Kat6b",1467.72924272439,-0.088885861555584,0.186764460759903,-0.47592
4922728485,0.634127865852523,0.748983833801339,-0.0256913809333504
"Dusp13",6.65678599149251,0.303326364848227,0.621586318065261,0.487987
51843888,0.625558682001972,0.742551027741047,0.0902892439540507
"Samd8",2335.54031128677,0.668651952547572,0.147252300895139,4.5408591
1379904,5.60254619858969e-06,0.00010818090741651,6.10593282201006
"Vdac2",10258.8306453059,0.347145256657744,0.150616893211434,2.3048228
4726472,0.0211764969834677,0.0601199483775878,0.975968886284736
"Nkiras1",1454.09078789615,0.598580032925345,0.181987590545238,3.28912
554494506,0.00100499184774685,0.00589510560991821,3.07289010839893
"Comtd1",671.025702779904,0.33074051079174,0.216722912308244,1.5260984
9724301,0.126985336280772,0.236098034404739,0.477426626233665
"Ube2e1",2869.31726047023,0.176395024446555,0.129281952359702,1.364421
10617087,0.17243506887857,0.297718849995072,0.21372121604873 7
"Nr1d2",4866.97194027822,1.34437817014309,0.123612488066364,10.8757471
932879,1.50418262830345e-27,7.33213822166516e-24,71.6147227059498
"Thrb",1233.80913171432,0.511464448431403,0.277020495367992,1.84630544
303943,0.0648478678779702,0.141524067312189,1.00005901433636
"Dlg5",1998.20665230855,0.426058214163478,0.225243263538962,1.89154697
667475,0.0585513662935872,0.131162056524858,0.865461273574939
"Ngly1",2113.75352470853,0.0811201983141385,0.214614586454855,0.377980
824389131,0.705444839025617,0.804325096270481,0.0176640645701291
"Oxsm",1085.37202687665,0.56132447300662,0.310688729406352,1.806710124
56477,0.0708074882534358,0.151431875174453,1.05956697840238
"Sftpa1",1.39535968377157,1.64562667088031,1.4284864955204,1.152007160
05425,0.249318131091902,0.388927507803016,1.55406780100599
"Dydc2",1.32976218936424,2.12583171965916,1.76269016053282,1.206015536
51186,0.227811499934671,0.364237436612871,2.14698231163545

"Prxl2a",8210.31760772519,-0.283006854876765,0.58921447607705,-0.48031
212125168,0.631005471144812,0.746535289358386,-0.082726410145216
"Glud1",15524.0845651386,0.303500398564918,0.169951646570229,1.7858044
0195678,0.0741309337490594,0.15693247852973,0.562064418615617
"Sftpd",45.761420257003,-0.404482512373894,0.807142732149491,-0.501128
853996767,0.616280441196459,0.735162804677183,-0.12444425089351
"Bmpr1a",3209.81535925512,-0.452075673980318,0.143066066033544,-3.1599
0847105786,0.00157818708521628,0.00836636535822377,-2.16252014146435
"9230112D13Rik",2.04837263902616,0.411598802916198,1.15871464111778,0.
355220162333619,0.722424661055557,0.816753891388125,0.0833147858854914
"Ldb3",214.460440887325,-0.956309647174158,0.23854642245336,-4.0089037
4854032,6.10012721040223e-05,0.000674264627825525,-6.98286584252251
"Opn4",0.875217721624989,-1.2172759199356,3.52779741313066,-0.34505267
0939898,0.73005477382736,NA,NA
"Cdhr1",12.3433645283157,-1.39796843470965,0.600747085644667,-2.327049
88191409,0.0199626105147067,0.0573917693623932,-3.99519020981516
"Nid2",1425.48898163042,-0.147368846205541,0.215865856225219,-0.682687
150170646,0.494804551145231,0.630815949930019,-0.0678988900497197
"Rtraf",3708.6427499897,0.181983651380313,0.269116959727446,0.67622513
1127451,0.498897718978052,0.634416469355902,0.0828115965888616
"Nudt13",1541.0162618036,-0.276321220511621,0.252458924848746,-1.09451
95170944,0.273727192310255,0.416670528196218,-0.241908029183123
"Ecd",1933.9370922479,0.284530613911762,0.114676623124067,2.4811561952
2501,0.0130956974121947,0.0416541448846609,0.904339128172355
"Dnajc9",1088.21951036801,-0.250715167898666,0.160181144947325,-1.5651
9775146515,0.117536548596144,0.222952390750814,-0.376272578031022
"Anxa7",7220.20688879149,0.0366297699236047,0.127579795356329,0.287112
624858021,0.774026085724542,0.85386507588042,0.00578684755189222
"Mss51",101.678548177028,0.360297079615077,0.291788895023889,1.2347868
125193,0.216909856623985,0.352266232255077,0.375922460525671
"Ppp3cb",2643.73269366381,-0.483968342119233,0.141796900508069,-3.4131
0945715413,0.000642261404568086,0.00418263622787861,-2.65060437617028
"Zswim8",5625.11081829507,-0.0987234210389863,0.169185981472325,-0.583
520101251031,0.559543229219668,0.688109358771687,-0.0369035554052312
"Camk2g",2082.19398936335,-0.119424417027859,0.187329205998744,-0.6375
10933712384,0.523792090343994,0.656724094241685,-0.0502169279596047
"Plau",21627.7775689398,1.17468526080188,0.355378764294295,3.305445847
7128,0.000948254260658903,0.0056369090165632,6.0830129556629
"Vcl",4284.29552690581,-1.0374117301534,0.145244308077535,-7.142529327
89357,9.16289581595504e-13,7.76774533128223e-10,-21.7606146088037
"Ap3m1",2017.71679069554,-0.728981994467232,0.262834349532265,-2.77354
15700593,0.00554497554821155,0.0214430649026237,-2.80100688269612
"Txndc16",1373.31554183121,-0.163790368890128,0.139868025327654,-1.171
0351133251,0.241584663418857,0.380086965814643,-0.158443464243156
"Ero1l",1532.65916651263,0.0578496000881934,0.144620068577933,0.400010
874403778,0.689148507360361,0.791576883446971,0.0135210868328587
"Psmc6",4214.90561447356,0.0137680616114044,0.15701729288263,0.0876850
018150296,0.930127040521318,0.956640470236786,0.0006103057102644
"Bmp4",2086.67143835463,0.779493983363323,0.373502169459323,2.08698649
459442,0.0368893553732416,0.0921265440038622,1.85877524830344

"Samd4",713.726677198554,-0.585173398537076,0.403442578667331,-1.45045
027341944,0.146932996448827,0.263705777315835,-0.779990073758326
"Mapk1ip1l",3613.41355918857,-0.304323232951442,0.157295763502944,-1.9
3471983080934,0.0530246855129986,0.12156088396619,-0.641312533629708
"Ktn1",4362.05662551435,0.104976283080536,0.308679053037143,0.34008230
2468072,0.733794548919713,0.825926578239137,0.0200766509163781
"Peli2",1710.85287526581,0.730057998736613,0.217562106863526,3.3556303
0373928,0.000791843436389462,0.00492251315541957,3.87948153628994
"Otx2",0.350131091224499,0.00799630514901372,3.86535327726682,0.002068
71263127284,0.998349407308075,NA,NA
"ccdc198",3586.1211681245,1.36433872336201,0.645443262632589,2.1138011
6944322,0.0345322530531046,0.0875337844552046,3.32316137544429
"Anxa11",5562.87630869518,0.190326223529047,0.129390155191485,1.470948
25914215,0.141305110106808,0.256032621212019,0.259310042424993
"Tmem254b",6045.15311256515,0.994624667364702,0.243187502222032,4.0899
4976418074,4.31466629948207e-05,0.000517706852352624,7.52543108623169
"Ppif",2014.45606965589,1.02776415823341,0.194276753314265,5.290206577
47393,1.22178284757767e-07,5.31748258081905e-06,12.4816926787122
"Slmap",3649.94919870837,0.0121455908906812,0.169201689656364,0.071781
7352495002,0.942775608478315,0.964592475946382,0.000437843347923884
"Gm49342",332.789662775073,-4.95326126034127,1.23522817669232,-4.00999
698177633,6.07195240016265e-05,0.000672573859719555,-36.1805947340507
"Rnase10",3.73632631256686,-0.120481753109403,0.834135443889255,-0.144
43907640184,0.885153761806771,0.928287868314782,-0.00896545584749598
"Rnase4",2210.13107975879,-1.8303227156576,0.351345004021829,-5.209474
14850358,1.89376590714788e-07,7.35550750150784e-06,-21.6345265247187
"Arf4",7376.1926925375,-0.175976346670687,0.239902322035556,-0.7335333
19634168,0.463233213394478,0.602342193715608,-0.089207613232042
"Dnah12",107.764411460387,0.73692249110682,0.616555060862606,1.1952257
5984667,0.23199883663407,0.369115733716124,0.734450147549213
"Rnase6",505.168391994864,-1.28391887017903,0.486802824788289,-2.63745
156108618,0.00835315543752703,0.0294156892672905,-4.52738951221088
"Hacl1",1124.81058743381,0.46991023675784,0.386989962620279,1.21426983
164141,0.224644701897257,0.360860306277206,0.478962756143683
"Gpr65",599.823510863045,-0.909003750820153,0.438782555486901,-2.07164
970314616,0.0382981211050035,0.0948478045605688,-2.14114172692636
"Eaf1",2098.83785100567,-0.114692435991796,0.111643898523634,-1.027305
90304061,0.304276443640457,0.449010981465347,-0.0918351434606653
"Mettl6",1738.36137291338,0.139931658350178,0.130210213995522,1.074659
61429869,0.282527151167273,0.426437095019314,0.119262411039645
"Sh3bp5",3511.27586904071,0.235126958710323,0.161954411968228,1.451809
52993396,0.146554573347456,0.263294221616431,0.313772966561721
"Capn7",2351.31160492701,0.0806402518125133,0.0805151032250244,1.00155
434921494,0.316558878452917,0.461719405451449,0.0623186188623977
"Arhgef3",1992.06901434203,-0.297341909528141,0.17663224242043,-1.6833
9542913344,0.0922985675339343,0.185628401040984,-0.500726287437219
"Asb14",17.3116317731383,0.6361332174365,0.658846526303513,0.965525645
259987,0.334281597342376,0.479040319343058,0.468175289177021
"Btd",3155.59898517367,0.641501950013099,0.160002325879983,4.009328904
97033,6.08915540038221e-05,0.000673433647173297,4.68496641809664

"Bap1",3072.71702291995,0.191986655391234,0.0930647156455549,2.0629371
0843573,0.0391186038779213,0.0963170272018828,0.449269923185248
"Phf7",466.859005357166,0.52982788440722,0.185823872075697,2.851236918
53429,0.00435495074202575,0.0178801494141963,2.13206139678266
"Galnt15",702.559792439682,-1.16958666349126,0.36787093481645,-3.17933
968899942,0.00147610994730666,0.00794402201285822,-5.65534400932166
"Sema3g",5179.09872604304,-0.334952696923458,0.224584182219747,-1.4914
349426253,0.135847339812739,0.248289410542631,-0.466642778030849
"Dph3",1584.25040793281,0.517169733180564,0.151677132688305,3.40967503
811759,0.000650403262503527,0.00422859713647675,2.82679028040193
"Oxnad1",1384.29912072978,0.315667525941782,0.244470753926361,1.291228
1770803,0.196624567232242,0.327590003579788,0.352282573000762
"Gm6768",0.906227186984328,2.32597184027891,2.04412869125515,1.1378793
5673008,0.255170862286299,NA,NA
"Nisch",17725.5341331109,-0.021861784333429,0.188482666470005,-0.11598
8301433056,0.907661813319305,0.943217504455568,-0.00127800430689561
"Parg",1222.55200611566,0.00400719368734569,0.123903138259322,0.032341
341338416,0.974199840814704,0.984077077375299,6.43196835928946e-05
"Ogdhl",225.903987408802,0.273856883836788,0.354684813369202,0.7721133
62383301,0.440047288672323,0.581868060108743,0.148296667779427
"Glt8d1",2278.44304588772,0.120003236988905,0.173833613930172,0.690333
901917899,0.48998423287274,0.626431878077023,0.0561273433498039
"Spcs1",3480.34525382363,-0.0122643417040885,0.414485688182759,-0.0295
893007979586,0.976394598305268,0.98508443949892,-0.000184307498207736
"Nek4",1179.10776788493,0.371176207164743,0.0911551222816739,4.0719182
6278057,4.66275427961649e-05,0.000548337653461776,2.78702083460344
"Chat",0.190492544179401,-0.093074062650293,4.78471990595139,-0.019452
3534250196,0.984480246300821,NA,NA
"Itih4",124.512118654378,-1.54074001399139,0.478681773581485,-3.218714
60127595,0.00128766576769939,0.00713669901608946,-7.61511511840772
"Hnrnpa1l2-
ps2",0.189257774950513,1.54478541448517,4.76381577441217,0.32427480146
9582,0.745729995342555,NA,NA
"Ebpl",846.609486763759,-0.180456796700378,0.319003137999449,-0.565689
722778493,0.571604722809597,0.697880330954385,-0.0649116878390938
"Kpna3",1871.17604959962,-0.538239864753975,0.156145116598074,-3.44704
897905602,0.000566745825051369,0.003803927744183,-2.9989224270133
"Spryd7",1046.37703010448,-0.264160931289846,0.292601576746301,-0.9028
00778544286,0.366631637885092,0.511542075905442,-0.177073791741891
"Rnaseh2b",898.184266900769,1.05464586430119,0.273958127831244,3.84966
079542945,0.00011828151417166,0.00113552583127476,7.15119444392721
"Gucy1b2",3.41128422898187,1.88065651120192,1.01377982229756,1.8550936
503547,0.0635828543828567,0.139406105336438,3.70557785663626
"Mapk8",1418.22910614118,0.0953194616350202,0.156049736361936,0.610827
44423188,0.541313822582147,0.671766059337886,0.0379223831342831
"Pspc1",686.352423687072,-0.736939339293042,0.188664892362233,-3.90607
563530225,9.38071605309142e-05,0.000941839349140971,-5.13475443011831
"Ctsb",38841.7714975697,-0.898042346174063,0.309307720697542,-2.903394
53586488,0.00369141276894389,0.0158140610840868,-3.72405210530567
"Ptpn20",0.539681850234574,-1.96359776848959,2.4443424060125,-0.803323

529330265,0.421787761603662,NA,NA
"Gdf10",1149.71086050609,-1.54577142209135,0.31742649827177,-4.8696987
5075747,1.11768513582753e-06,3.01607735157191e-05,-16.0898858368317
"Gata4",1.91529805612336,-1.6015802148719,1.15235096491235,-1.38983717
950349,0.164578325283071,0.28717989854746,-1.9982058460722
"Zmym2",2032.49663037448,-0.041450292520822,0.134362306203507,-0.30849
6435436592,0.757704612182408,0.842854396336032,-0.00708638581126485
"Cryl1",7557.87408945225,1.76549609520164,0.466210174386651,3.78691026
536334,0.000152532153715869,0.00137943967214843,11.6276948097458
"Prkcd",6530.78875399155,-0.262456599322333,0.244515655581764,-1.07337
339483594,0.28310361665772,0.427075769252784,-0.223296456904754
"Anxa8",602.671621556694,-2.81644702318714,0.750278717195137,-3.753867
67429073,0.000174126810507226,0.0015211315021053,-18.2739894864859
"Eef1akmt1",650.007214714937,0.22030796689351,0.282134899788638,0.7808
60386497927,0.434884612981785,0.576985586820825,0.121155721000965
"Xpo4",1224.82273354334,0.482373527545115,0.231809149589064,2.08090805
906598,0.0374423223563503,0.0931662074150227,1.14485095767799
"Tdh",1.63868703795132,-0.721426763824422,1.6061165550762,-0.449174601
646636,0.653305707773557,0.76376991786598,-0.194416353367863
"Tkt",28452.9858857102,-1.81727988453632,0.592977947688254,-3.06466689
296128,0.00217912555097104,0.0106782080906844,-8.24963333835312
"Pinx1",506.128832360431,-0.11238929334779,0.260887422395899,-0.430796
135419812,0.666616609810183,0.774277403998468,-0.0287519984451104
"Lats2",2246.24230584428,-0.371490345542256,0.125911010485759,-2.95041
985692166,0.00317342362128288,0.014085001995851,-1.58353136588319
"Dcp1a",1331.010736806,0.0338104140154453,0.166921660970326,0.20255258
5559617,0.83948475291734,0.897124821651609,0.00367046774302639
"Sap18",1664.7920233074,0.489874808016337,0.156896733316761,3.12227538
241552,0.00179458988394855,0.00918879032490253,2.29740065884904
"Ska3",52.323579772084,-0.752899493339217,0.494856398410846,-1.5214504
5665174,0.128146835105725,0.237645709614936,-1.08189724459353
"Mrpl57",1386.00982852338,0.865971901526182,0.218493441062071,3.963377
1032979,7.38969129862838e-05,0.000780098543262892,6.19697313215614
"Zdhhc20",2988.35561977352,-0.306260583556189,0.156374617577498,-1.958
50572363132,0.050170700086826,0.116691913431103,-0.657914509231677
"Hmbox1",1977.45324341926,-0.304929159676772,0.0839280121371959,-3.633
22270969922,0.000279903224798575,0.00218564400365343,-1.86794869355841
"Micu2",2992.83631221455,0.357191703754227,0.0966588243361725,3.695386
38823022,0.000219552640562618,0.00182163293008082,2.25317311329016
"Fgf9",955.361179296712,1.32888971799731,0.384109654383385,3.459662372
0135,0.000540853079798768,0.00367825369721534,7.44884836426438
"Ints9",741.766839875465,-0.131399107746012,0.0819041471781807,-1.6043
034726942,0.108647176017772,0.209805153807519,-0.20518969544087
"Extl3",3001.65100579829,-0.811278253973741,0.186945598012134,-4.33964
887432698,1.42710558675643e-05,0.000223859249642613,-6.81838244990771
"Cab39l",2440.30095390692,0.128096274021398,0.256336333644975,0.499719
53721867,0.617272573738159,0.736020317180965,0.0392611948906523
"Cdadc1",2311.54426506703,0.333049376789753,0.18307999361091,1.8191467
5776953,0.0688890431314904,0.148337776143324,0.635545914274161
"Atp8a2",87.1248127303944,-1.83022116588849,0.594667326109248,-3.07772

276284818,0.00208588872142122,0.010306752365993,-8.3731813573298
"Amer2",81.9038291291157,-1.41111006635446,0.632513040701065,-2.230958
06023289,0.0256839072315641,0.0699464302568552,-3.75358891779107
"Mtmr6",2742.0270662528,-0.375242085268759,0.14465780603631,-2.5939981
7784164,0.0094866989697666,0.0324739565506512,-1.28607352084204
"Spata13",2330.19255304989,-0.157997517517556,0.240501214942904,-0.656
951016048152,0.511212413483752,0.645404016971393,-0.0691837594838043
"Cacna2d3",21.6059714383035,-0.304068342501622,0.3901954209486,-0.7792
71939589665,0.435819540520348,0.578029345015016,-0.166669175688873
"Mipep",1377.24548095289,0.356806590638708,0.313110608434547,1.1395544
6103416,0.254471966741293,0.395165212449962,0.331277557970074
"Wnt5a",1362.96450159766,-1.5799393366858,0.435929629158038,-3.6242990
4050643,0.000289746207262303,0.00224929072014609,-9.63311187702604
"Esd",13266.179393085,1.00612791676805,0.288481628739798,3.48766720835
297,0.000487254144967855,0.00339181767889441,5.72123503324272
"Lrrc63",1.21706099360028,-3.67066886723376,1.77462040997853,-2.068424
80036515,0.0386000951416782,NA,NA
"Lcp1",5012.13824586669,-1.26409217381009,0.336523327713014,-3.7563285
2082133,0.000172424320687012,0.00151030072091436,-8.21084307980781
"Cpb2",24.2138899054068,2.09965702486055,0.498731231054138,4.209997076
8276,2.55373992409442e-05,0.000346505365622777,16.729253041004
"Zc3h13",3954.94847892385,0.534973845374044,0.178981118948991,2.988995
98189744,0.00279895812523851,0.0128418928777393,2.32983387108304
"Erich6b",0.185691316495273,2.57351138408779,4.72851649690914,0.544253
443076702,0.586267079921631,NA,NA
"Slc25a30",4524.88518461734,1.10478432441706,0.332324217794336,3.32441
713622199,0.000886035596600765,0.00536852767635852,5.77493037032715
"Gpalpp1",1159.93812110914,-0.336946040841324,0.143421604291843,-2.349
33950505592,0.0188067499477546,0.0548368491821923,-0.978286736150723
"Nufip1",671.902128721247,0.179172726274774,0.109438944141315,1.637193
48428119,0.101590048057091,0.1997322620552,0.288607395918551
"Tsc22d1",32766.1891113532,0.0672271415222775,0.264976975377237,0.2537
09370131382,0.79972009455333,0.872162120833555,0.00919532532273223
"Enox1",101.203149816668,1.35447999493838,0.301838132852953,4.48743829
063587,7.20846817343919e-06,0.000131478683298145,12.1045350796286
"Dnajc15",2394.4269262648,0.457096235883816,0.397440286535728,1.150100
40846155,0.250102518174093,0.389808065496281,0.430630729846908
"Epsti1",1043.22903602173,-1.18154743977087,0.332752287882855,-3.55083
190348142,0.000384015598786992,0.00280327073947913,-6.94391699296064
"Tnfsf11",39.6835102354343,-1.63036749003882,0.670643168669842,-2.4310
5061857635,0.0150551110966562,0.0462785048340853,-5.01024595057644
"Akap11",2616.37179921291,-0.0145508876188697,0.148795877444264,-0.097
7909325768794,0.922098307627879,0.952029272021625,-0.00071531431248727
5
"Rgcc",1623.30128180399,-1.84151609305859,0.612766897719131,-3.0052473
4595351,0.00265364956512805,0.0123394484712482,-8.09337843802667
"Tdrd3",1725.74105915219,1.01003214818819,0.265502879082326,3.80422295
863315,0.000142250014426614,0.00130891495105716,6.70515575185801
"Naa16",1196.52051899622,0.443193414516585,0.251643764416937,1.7611937
07873,0.0782056226642025,0.163435501683454,0.802772564096356

"Diaph3",62.8714685239614,-1.05559114122892,0.585848216271021,-1.80181
677081456,0.071574238242771,0.152619695675585,-1.98430666757406
"Mtrf1",265.956574671637,-0.133776131264019,0.350553368463371,-0.38161
4165770018,0.702747575301831,0.802469822737049,-0.0294389130682888
"Wbp4",2465.28955673729,0.428336918490038,0.196528994235949,2.17951005
222052,0.0292937987338453,0.0772060675469742,1.09708959604198
"Sugt1",2554.08950556502,0.323746152137471,0.161407134980627,2.0057734
8811952,0.0448804204880449,0.106960551807331,0.723668215745885
"Cnmd",4.28179023541701,-0.867921235271054,1.05339975094255,-0.8239239
04001744,0.409982797169198,0.553513680875572,-0.513348340294857
"Olfm4",2424.82041730429,0.80074631060882,0.592745105055944,1.35091172
205166,0.176723712786323,0.303110393376823,0.955817403981498
"Elp3",2077.60394058824,0.0318455949837408,0.126999675513532,0.2507533
57085133,0.802004805218105,0.873846867401096,0.00429438254707794
"Scara5",374.318691461263,-2.44628433092059,0.626583052601248,-3.90416
61289192,9.45508187815557e-05,0.000947841575631246,-17.0293758274627
"Pbk",102.603728330335,-0.621696396257913,0.467076221386202,-1.3310384
2112284,0.183176369116517,0.311329571568501,-0.725459521838119
"Esco2",77.7598474204663,-0.852290398694535,0.3827976968625,-2.2264773
4215777,0.0259822250035828,0.0705180154676863,-2.26017788243839
"Ccdc25",2013.98416217614,0.327221353540942,0.181478520820368,1.803085
85314531,0.071374732548966,0.152310882701077,0.615775472145316
"Clu",24395.4279620001,-0.124469567913984,0.325550578640957,-0.3823355
7573018,0.702212469866439,0.802022159109357,-0.0274603567921481
"Adam2",2.8274163936332,-2.52597189995775,1.67599300717999,-1.50714942
671982,0.131772359969159,0.242500941451502,-3.57866989696349
"Ephx2",12866.9419516585,0.903523814357698,0.439186635634676,2.0572661
8491476,0.0396606281911903,0.0972585748298112,2.10555574858122
"Chrna2",29.8439040190865,-1.05516244783607,0.506055281725303,-2.08507
348098153,0.0370626310249356,0.0924839605742762,-2.51204639419464
"Trim35",2241.90104562178,-0.865971798177015,0.13179540437562,-6.57057
658633508,5.0120796233772e-11,1.37641589431843e-08,-15.6751268091766
"Stmn4",8.14379215836258,-0.307532307332614,0.714486034327352,-0.43042
4518545192,0.666886863046694,0.774492825998239,-0.0785889222286814
"Dpysl2",2216.72600801152,-0.644912584518348,0.239565913812571,-2.6920
0477753653,0.00710239278567115,0.0260501231254733,-2.35246877754764
"Bnip3l",5589.59613302426,0.0225013596651117,0.207323912388977,0.10853
2389755867,0.913573388944409,0.946837502665077,0.00122919960032591
"Ppp2r2a",2013.19625585173,-0.559562823964512,0.245595949887077,-2.278
38783262425,0.0227034806899698,0.0634655865939831,-1.54285877261159
"Ebf2",117.924272937694,-3.20474338649802,1.28047386624222,-2.50277922
180708,0.0123222383985459,0.0397385055069215,-10.336690361043
"Nefm",37.0749061100613,0.93836585018422,0.537806434661049,1.744802199
65316,0.0810193058027518,0.16787613438279,1.67454094732495
"Nefl",35.71771993759,0.277347802634425,0.605137784497081,0.4583217405
02328,0.646721307117407,0.759213200444049,0.0764017326462396
"Adam7",6.9542373451905,-3.94892155610678,1.43781886799128,-2.74646664
056068,0.00602410120571523,0.0229230923710062,-14.9095895962793
"Adamdec1",89.7490425377767,-2.61318488708009,0.817296460988052,-3.197
35250525976,0.0013869532082484,0.00757128476665695,-12.7612074989291

"Nkx3-1",98.743356141599,-0.0122269362070126,0.466677207657902,-0.0261
999857854115,0.979097827221238,0.986669833155619,-0.000164083173715677
"Pibf1",329.84231217483,0.0941321698572537,0.144092328879145,0.6532767
60737246,0.51357787157322,0.647463094731714,0.0409186408327725
"Bora",343.717634161385,-0.148864066225626,0.248189248175583,-0.599800
62520803,0.548639116618813,0.678505127626474,-0.0577388994087901
"Tnfrsf10b",496.833296372273,-0.820294606081918,0.213559227039185,-3.8
4106375292043,0.000122502290701556,0.00116457809073571,-5.541415238243
59
"Rhobtb2",925.233695674144,0.00790811010218094,0.103158868149705,0.076
6595276201034,0.938894402032998,0.962136072467515,0.000305248232701139
"Klhl1",0.504957397744252,-2.56651751724077,2.52163309205463,-1.017799
74466848,0.308773133406742,NA,NA
"Pebp4",0.927011348153509,-2.04876255156336,3.09021026841563,-0.662984
837149537,0.507340265254927,NA,NA
"Bin3",1336.25547220426,-0.0390941435500546,0.16781445447055,-0.232960
525798541,0.815792058325018,0.882553069530513,-0.00488427985709423
"Pdlim2",1411.50212041062,-0.476429968345363,0.253241170242131,-1.8813
29042548,0.0599271726772868,0.133466252487735,-0.95948546886211
"Sorbs3",2055.83297058066,0.00631587151940382,0.138825835675985,0.0454
949288700472,0.96371281689072,0.978160984057015,0.000139460867135215
"Ppp3cc",620.469309852263,0.398213178793632,0.102058309696123,3.901820
24353828,9.54720466823549e-05,0.000956093459790733,2.76863880416637
"Slc39a14",1775.5818883486,0.75341494006765,0.161317856298653,4.670375
35307205,3.00649869374194e-06,6.63130220934167e-05,7.24869876405494
"Fam160b2",2171.47497879082,0.118258495950751,0.15062682089682,0.78510
9154177516,0.432389572974218,0.574486571927306,0.065548148306593
"Hr",996.847912501933,-2.3559002435942,0.41043331802293,-5.74003166931
633,9.46588475889232e-09,7.62668681937531e-07,-33.1862524471334
"Sftpc",5.14370532779221,-1.93475024853745,1.17041715080419,-1.6530433
1640055,0.0983220481729223,0.194746372945514,-3.16536211023286
"Bmp1",2841.488099737,-0.990390905433695,0.240699955019268,-4.11462854
388324,3.87803403018068e-05,0.000475260260970854,-7.57812257706145
"Dmtn",4796.14199058565,1.24362250247619,0.324114183668827,3.836988830
29105,0.000124552134834211,0.00117946455803664,8.38536686602492
"Xpo7",2603.71953581093,-0.169145009669472,0.198099721482868,-0.853837
695496709,0.393194925012861,0.537812321845021,-0.104911452040996
"Fgf17",21.7135724244833,0.24081674427437,0.489549569333144,0.49191493
4380202,0.622779480254867,0.740559511252631,0.0723291362829537
"Dok2",560.296938564273,-0.382200523406073,0.263781769273303,-1.448926
98407098,0.147357974496228,0.264395489714499,-0.508444883890366
"Gfra2",150.541241315221,-1.96358015655194,0.320635321355016,-6.124029
46828746,9.12380110193128e-10,1.4702138337641e-07,-30.8923935646691
"Rb1",1534.41418860761,-0.173531935975354,0.209258505517862,-0.8292706
45634723,0.406951277370474,0.550832072625443,-0.103481481117309
"Rcbtb2",1941.85287797604,-0.424276017358776,0.286169813280562,-1.4826
0227902799,0.138180111281804,0.251703644410745,-0.585289995396998
"Itm2b",62134.4471024088,0.0144170448455344,0.168570448513346,0.085525
3395401206,0.931843751231238,0.957426856800689,0.000627227240984422
"Med4",1034.59785932117,-0.0350036076857763,0.10044069859419,-0.348500

241194073,0.727464532714354,0.820839320536139,-0.00691068877800362
"Sucla2",7230.93907465081,-0.237016573315987,0.498624412667552,-0.4753
40892452478,0.634544016833281,0.749339182376313,-0.0683943440430481
"Uchl3",1084.25174414165,0.231511915771771,0.202520617157675,1.1431523
319501,0.252975349661431,0.393342373819664,0.216017949824901
"Gpc5",35.5743765516894,0.136533674121689,0.640116222174352,0.21329513
2027604,0.831096770276075,0.892267355417859,0.0155634005367234
"Trim52",24.954444550199,-0.016513053014355,0.360350745441844,-0.04582
49447884658,0.963449776625846,0.978046740493088,-0.000366553748311827
"Spry2",976.632432120174,-0.0578792982134151,0.208980078107447,-0.2769
60841136525,0.781810174592758,0.858947133837245,-0.00880042590521977
"Rbm26",2828.23563540499,0.00976653287984358,0.0834237323022252,0.1170
71396955265,0.906803475766025,0.943050351856775,0.000572666538572882
"Rnf219",278.180851164336,-0.536735719054261,0.20106264581886,-2.66949
49569987,0.00759654138731086,0.0273885658228157,-1.93097634160355
"Ednrb",1335.36862102919,-0.961029047833732,0.355145128714928,-2.70601
782237915,0.00680953912948528,0.0252563047264036,-3.53531782093378
"Scel",264.836332912249,0.230212418268524,0.556317988278287,0.41381444
2673323,0.679010005963897,0.783856548033396,0.0560634575203645
"Fbxl3",4444.95902128914,-0.0174482666190595,0.109928791261215,-0.1587
23355536573,0.873886836386736,0.921235285353051,-0.00143145244485891
"Cln5",1445.20835859444,-0.234426138274337,0.156315784814619,-1.499695
87877739,0.133693198606891,0.245318086433009,-0.329415515391355
"Acod1",22.6000458713147,-1.69955514473714,1.22794911087654,-1.3840599
172094,0.16634010889697,0.289399432789606,-2.10735902491986
"Dct",19.3547293180563,-3.34950229204306,0.86246590669937,-3.883634432
41658,0.000102906593071411,0.00101234750338364,-23.0964374003541
"Tgds",928.658615850365,0.128614146580421,0.149282911786123,0.86154634
205344,0.388937209203243,0.533073084847803,0.0809107409009807
"Gpr180",1146.31666994785,0.257879535867613,0.118071402284873,2.184098
1886996,0.028955033764787,0.0765408416954741,0.662732562493481
"Cldn10",5329.95793381029,1.67144548867051,0.569093794143772,2.9370298
9888561,0.00331372153788052,0.0145749926789069,7.06762041566316
"Dnajc3",6775.25564166853,0.169091928351227,0.120437990114156,1.403975
00980342,0.160326291285116,0.281828527540316,0.214147563532327
"Mbnl2",4294.67829379738,-0.705451565039786,0.181422402714292,-3.88844
792310874,0.000100887310958391,0.000998021709318477,-4.87448374299011
"Nipbl",5013.20533890824,0.191372193965616,0.185681418824661,1.0306480
5933182,0.30270589021794,0.447302724325077,0.153962695860206
"Nup155",940.348297619756,-0.164453235547981,0.162389784191722,-1.0127
0678058062,0.311200238150553,0.456361480404594,-0.129008640103328
"Gdnf",20.3052943254906,-0.187746401049624,0.470495323724594,-0.399039
887502734,0.689863814007135,0.792258490134946,-0.0437200470591705
"Osmr",3057.28729596066,-1.00803432321575,0.265076600636898,-3.8028038
7176292,0.000143067617355004,0.00131519679546811,-6.68706681424635
"Fyb",1478.6320169618,0.211700713847194,0.464800795907184,0.4554654718
99641,0.648774402471766,0.760544552752186,0.0579468433446329
"C9",13.506056406635,1.12809767036981,0.518737464163166,2.174698664168
53,0.029652705072425,0.0779517923017586,2.87852699322922
"Dab2",11961.2278112893,0.936472772626909,0.291340451505685,3.21435889

793915,0.00130736152174708,0.00721713900085634,4.61802506040456
"Ttc33",1197.12999034191,-0.280442639026348,0.294287102759982,-0.952955927718909,0.340612401263536,0.485295851115997,-0.202759066579993
"Mcpt8",0.266234037804189,-1.081821048545,4.7800018809208,-0.226322306035705,0.820950750730983,NA,NA
"Rab2b",593.009007270949,-0.151264368727632,0.16850828366615,-0.897667256687025,0.369362970811494,0.514332416315035,-0.100573485163607
"Mettl3",1265.86328753934,0.131149026393176,0.142934171195038,0.917548444131103,0.358855319695203,0.503886001974439,0.0898902276882586
"Dad1",4377.13661142571,0.302492319118892,0.249067130215168,1.21450116222711,0.224556406288516,0.360765618938972,0.308399022059254
"Lrp10",6984.2897382107,0.223685823660138,0.0777064042044766,2.8786021686389,0.00399441872159041,0.0167716166735872,0.9144427023138
"Rem2",44.6117806196991,-0.42475065192945,0.550574225430209,-0.771468463852549,0.440429307930465,0.582202755662929,-0.229763137183054
"Haus4",672.465371864136,-0.0713079953041212,0.221910869168336,-0.321336199400079,0.747955626654754,0.836121936046003,-0.0127627634734501
"Ajuba",2523.15674407322,-0.14483166291921,0.214895897915535,-0.673961971001121,0.500335491422697,0.63558156308292,-0.0656398606526971
"4931414P19Rik",367.292855132192,-0.0146029017775461,0.221973617543714,-0.0657866549148358,0.947547681054237,0.967596348863282,-0.000481023580608559
"Slc7a8",4444.50793470715,0.719733518942245,0.346059653189818,2.07979610540574,0.0375442384870757,0.093372138012883,1.70660497641939
"C6",219.563446314531,-1.52805728611691,0.353264759625784,-4.32552991624636,1.52165614703229e-05,0.00023453953798289,-12.7713291776924
"Fbxo4",796.168380151934,0.0776755005402252,0.140104115263465,0.554412697972197,0.579296437877722,0.703965219429095,0.027266145750747
"Acin1",14564.2845109374,0.0638630619036439,0.203845390858197,0.313291664995602,0.754059082004012,0.840195581302428,0.0111198733385429
"Oxct1",34201.8398425105,1.81287719124151,0.350852498307462,5.16706365206737,2.37800296078561e-07,8.88243328149384e-06,21.0863632497279
"Drosha",2160.12267503279,0.126786302792858,0.0901372910994747,1.40659100408218,0.159548702145836,0.28079841562872,0.161033596925078
"Psmb5",6744.24526912077,0.446382898996404,0.259075645203335,1.72298287106865,0.0848916226376719,0.174125174594849,0.780268756617181
"Pabpn1",11770.5438032451,0.256402798997501,0.114664509638109,2.23611298567211,0.0253443710446693,0.0692107208163812,0.684749187638725
"6030458C11Rik",1132.17165703286,-0.279446555712166,0.124203312617137,-2.24991225937407,0.0244545155737323,0.0672231981751915,-0.754432172468659
"Pdzd2",2013.03934198846,0.0886964158139366,0.422577978605032,0.209893606161711,0.833750712625051,0.89375155694601,0.00996304158614002
"Slc22a17",2655.09918965032,0.32783344410815,0.144825187797463,2.26364936302808,0.0235956892117298,0.065359958552395,0.894278972990382
"Golph3",4203.04043465705,-0.111081153546404,0.201849741873819,-0.550316054482963,0.582102614534889,0.706362756920666,-0.0386147363441615
"Zfr",3797.78130801526,-0.417732317187858,0.138901139410428,-3.00740741912516,0.0026348636870706,0.0122876278810099,-1.83767229725474
"Efs",651.463626937673,-2.64780711838301,0.491560671725466,-5.38653165

455393,7.1830283521618e-08,3.53839172465398e-06,-33.2348466648133
"Ngdn",1706.13108465034,0.0829324627066353,0.14417441770489,0.57522315
0034767,0.565140388096289,0.692695283132211,0.030449904583892
"Sub1",4425.95647530522,-0.230240709169252,0.233050496459973,-0.987943
44001235,0.323180349138655,0.468114253407361,-0.17476257136036
"Npr3",1897.69187803629,-1.31112959831346,0.290763678250603,-4.5092619
7591787,6.50535365152105e-06,0.000121495579978312,-11.8206628472225
"Jph4",14.9691965545155,-2.57038931549246,0.648373949991529,-3.9643624
1080635,7.35923778167399e-05,0.000777304540991763,-18.4031607068267
"Dhrs2",0.734427747304919,-2.06039011276931,1.71366407997053,-1.202330
22145434,0.229235612359744,NA,NA
"Dhrs4",6707.99765776619,1.48303210724973,0.306719385992992,4.83514304
92353,1.33049921196212e-06,3.43149122153933e-05,15.2454670508297
"Carmil3",398.454155791532,0.569917125140104,0.375714103874309,1.51689
042083649,0.129294352529848,0.239250382995823,0.815120917939359
"Cpne6",5.39195204387769,-2.17429002552731,0.802476158576993,-2.709476
16609934,0.00673895467207761,0.0250326039619298,-8.01786003112597
"Dcaf11",7470.3988490854,0.510164757505511,0.13011702707643,3.92081473
861098,8.82500908546259e-05,0.000897132571159278,3.57947251479948
"Fitm1",745.464618146764,2.76778962522818,0.952095768912666,2.90704960
11017,0.0036485533952954,0.015687908576838,11.4997922901876
"Psme1",7568.48259250831,-0.279007144721839,0.139321469932686,-2.00261
413303092,0.0452187217096268,0.107547528164711,-0.622136382918799
"Emc9",759.482707489655,-0.176599872915184,0.297858229748699,-0.592899
088483069,0.553248688075632,0.682909782229595,-0.067353870311304
"Tgm1",811.694640809945,1.89592171205606,0.55410356293659,3.4216017345
3535,0.000622534299403906,0.00408417690773128,10.4287734448019
"Cideb",5791.61864889426,1.87529375168007,0.668282399157586,2.80613967
095947,0.00501389541693994,0.0199227497125525,7.34344967247223
"Adcy4",721.151053122726,-0.856405532187969,0.266265662150242,-3.21635
739761568,0.0012982903793597,0.00718374524070161,-4.22716153579887
"Ripk3",317.849094060992,-0.923492333129286,0.331448410394341,-2.78623
250004597,0.00533246095187926,0.0208989595255762,-3.57211997212056
"Sdr39u1",2708.24097847817,0.431977743922831,0.287619884138936,1.50190
500638044,0.133121643510837,0.244453362702421,0.608540324205365
"Cma1",7.51662117351149,-4.09128757279434,1.2126692379603,-3.373786886
58406,0.000741417436513156,0.00467081007338724,-21.9555787446349
"Mcpt2",0.137986538933697,0,4.78471990596107,0,1,NA,NA
"Zscan26",4670.1137913351,-0.175703145263378,0.216608313290141,-0.8111
56056730048,0.417276057387133,0.560565562004569,-0.101698574587449
"Atp12a",46.8912225266374,1.88688555074349,0.577872217525044,3.2652297
4027855,0.00109375325004765,0.00630387255968936,9.56007761815011
"Sema5a",3264.25495574717,-0.259838203602436,0.331605485548958,-0.7835
76312594125,0.433288759400648,0.575377254702298,-0.143620203509553
"Cct5",6263.98080304115,-0.323033226456665,0.235386772078263,-1.372350
8063115,0.169954256465939,0.294412485406923,-0.394996463554282
"Cmbl",5290.85512807308,1.57107161748197,0.465273247016501,3.376664417
21343,0.000733705063553565,0.00463620638581795,8.44271709424746
"Ropn1l",245.860564243134,0.87475599254545,0.406237295955852,2.1533128
5742044,0.0312940989844143,0.0812155386660602,2.19620498433915

"Ankrd33b",2127.26799051735,1.09789483937785,0.436136314601864,2.51732
039415266,0.0118251242368694,0.0385949568748709,3.57324550152804
"Ctnnd2",184.371063865394,-0.872296996212644,0.249974805610996,-3.4895
3965212834,0.000483853269051591,0.00337304519979324,-4.96506157365888
"Tars",1955.39750858608,-0.554771320792605,0.177165851049077,-3.131367
12017333,0.00173994505171427,0.00899903017798692,-2.61332712325619
"Slc45a2",5.01455472072196,0.746944772109503,0.827148004442336,0.90303
6419235629,0.366506566356877,0.511425250900049,0.500866686189916
"Amacr",8259.98893878669,1.90963978744892,0.521899394834335,3.65901897
252648,0.000253182597961537,0.002025427025393,11.8435376935458
"Skor1",3.74858852173343,-2.01644169606828,1.04840639504418,-1.9233397
5221823,0.054437395303528,0.123983218505804,-4.20954194795986
"Rai14",3508.58481614439,-0.229834776259709,0.153186717921505,-1.50035
707650241,0.133521931926082,0.245049946225033,-0.323215089199518
"Brix1",1405.55426475303,0.0334745638545982,0.140236331514696,0.238701
080476355,0.811337378871716,0.879810136779931,0.00428638939622351
"Rad1",589.728468270623,0.000266292767167054,0.18604771440638,0.001431
31437016956,0.998857976752323,0.999473100211269,1.40346580300481e-07
"Ttc23l",1.01330985088951,-1.47312789601635,2.14353208427169,-0.687243
22198185,0.491929471597816,NA,NA
"Nadk2",2741.13088491976,0.911892129085318,0.249210045654466,3.6591307
0113422,0.000253072251795595,0.002025427025393,5.65553193552666
"Mtdh",7972.72763614934,0.460745917703204,0.232405292390009,1.98251043
668148,0.0474221404609243,0.111752102333467,1.00971188504002
"Laptm4b",2569.50333892597,-0.472715002919661,0.239401305421932,-1.974
5715341298,0.0483167921004582,0.113462701719226,-1.02876073376438
"Sdc2",4932.00855223129,0.361625395381654,0.192194265808055,1.88156183
464292,0.0598955322406591,0.13342203674493,0.728399600902815
"Dnah5",44.853364879933,0.56084172859247,0.540009837513496,1.038576873
29345,0.299001566110082,0.443408315790568,0.456112515082685
"Trio",3435.29338812042,0.0736982437945626,0.184813967759385,0.3987698
80264205,0.690062772228464,0.79243147378423,0.017145840804664
"Ank",1139.74240346746,-1.81377925095039,0.317148686934498,-5.71901863
596552,1.07141044923564e-08,8.38970318843231e-07,-25.3767496614536
"March11",1.01666418967249,-0.915303664787881,1.97282078414585,-0.4639
56823723433,0.642678679815511,NA,NA
"Retreg1",3823.43786878553,1.05935689799981,0.293213222358935,3.612923
3513999,0.000302764222517039,0.00232265046748531,6.42504863725599
"Myo10",3896.49824924623,0.609053239920606,0.3170398297428,1.921062222
41762,0.0547238635294775,0.124432173888951,1.26926360193913
"Rnf19a",2218.57051869533,-0.0553607198602417,0.107628537046007,-0.514
368413616709,0.606994438520525,0.72746804119941,-0.0176149623035038
"Pabpc1",19300.6095072867,0.0709511491431195,0.137423557641235,0.51629
5388948874,0.605648116240863,0.726519153348486,0.0226682133228111
"Ywhaz",20280.122013618,0.180975302596933,0.15923642393477,1.136519510
5805,0.255739204792589,0.396564578896604,0.167387021529232
"Grhl2",779.675185203692,-0.198840177506202,0.195890672419207,-1.01505
689398361,0.310078707900588,0.45527401630614,-0.156458548467144
"Rrm2b",1123.8202656613,0.173711525683252,0.159463393854221,1.08935048
655778,0.275999357014276,0.419474274149532,0.150912422966435

"Atp6v1c1",3668.51960494676,0.191799832944451,0.168061253447863,1.1412
4956829477,0.253766082256936,0.394226043490218,0.178533195728218
"Baalc",28.4165049877348,-0.821664882951577,0.525326933811386,-1.56410
195264534,0.117793627493693,0.223252712441978,-1.23204615604862
"Fzd6",1322.73136903705,-0.21991619612261,0.123958825463321,-1.7741068
0764866,0.0760454849544772,0.159809598618809,-0.403276202601699
"Slc25a32",1534.18287345411,-0.175509223057671,0.264223756998699,-0.66
4244672966843,0.506533725631459,0.641199414553151,-0.0779988912254946
"Dcaf13",1750.29653581011,-0.0865893452224874,0.16200310332652,-0.5344
91892096443,0.593001257525086,0.715801307449027,-0.0289513773915338
"Dcstamp",58.9211754242321,-1.97002258637543,0.496876946722692,-3.9648
0979721303,7.34544934755137e-05,0.000776698269304374,-14.1062651921001
"Dpys",515.781515660655,2.60971273984351,1.02494133375092,2.5462069426
8704,0.0108900607742159,0.0362098234951673,8.66013555426554
"Lrp12",531.598925396934,-0.361898564513877,0.180440020377767,-2.00564
466661116,0.0448941727317317,0.106971352795222,-0.808913576461941
"Zfpm2",100.995087143027,-1.01819477231171,0.249773597292291,-4.076470
78534161,4.57243973102415e-05,0.000540945058729681,-7.65905742529903
"Oxr1",2268.29523394691,-0.746735743050676,0.163894706213875,-4.556191
96190644,5.20893357320333e-06,0.000102558077974414,-6.85882851361848
"Angpt1",499.605859041948,-0.493928204711235,0.37256947600317,-1.32573
449121482,0.184927658475122,0.313186787539992,-0.573428666423809
"Csmd3",1.27851181291386,1.3005205577713,1.97139393634119,0.6596959307
81345,0.509448978533049,NA,NA
"Eif3h",6077.20206575999,0.1458702721479,0.15967290259214,0.9135568388
86455,0.360949754401904,0.506061574122983,0.0993517944080784
"Utp23",581.256333286625,0.295942791210955,0.267949354414708,1.1044728
6524498,0.269388067962623,0.411607910754269,0.262703697153569
"Rad21",6999.02326799473,-0.0406390974570224,0.135039530958338,-0.3009
42229054099,0.76345854793596,0.846656510502522,-0.00676479241971007
"Slc30a8",0.628879332206672,1.33705672712663,2.95151781591514,0.453006
490395202,0.650544067936487,NA,NA
"Cdh10",1.85452580107119,-1.20624391273286,1.17325971516009,-1.0281132
9592806,0.303896537000722,0.44865409846597,-0.966808199432055
"Shcbp1",122.887611572209,0.106854603330157,0.320744921169286,0.333145
114007152,0.739024746452896,0.82981166316243,0.0199344224936506
"Rida",30611.0749114453,2.04325590669697,0.581222779029196,3.515443613
73753,0.000439019975763515,0.00311499690226965,11.792706881052
"Matn2",2411.47634606091,-1.69074878543331,0.265417598934539,-6.370145
73344215,1.88848670764491e-10,4.04634217864401e-08,-28.7813924431941
"Pop1",572.559342338675,0.573403681625877,0.259672864847718,2.20817713
072231,0.0272319279426329,0.0731562594413689,1.49954098740466
"Stk3",1012.14390621672,-0.249624119330647,0.130663397220988,-1.910436
46988959,0.0560770389156038,0.126931751192993,-0.515250574844819
"Osr2",282.864184239331,0.420418661271562,0.243228536550866,1.72849233
578166,0.0838999939202096,0.172488620988639,0.738853849557995
"Khdrbs3",1430.63698311303,-0.27398410154278,0.18527900667956,-1.47876
495266749,0.139203145829795,0.253235952359521,-0.376298972801671
"Zfat",170.835485682514,-0.448104345848831,0.168564430348948,-2.658356
48079018,0.00785227869168839,0.0280832284580741,-1.60088984849697

"Eif3e",4919.86004613475,0.0984059968105417,0.124946240811994,0.787586
694653842,0.430938499883503,0.57330895304314,0.0547462596236153
"Emc2",2301.73589689595,-0.0654769505075531,0.253240246418921,-0.25855
6652954911,0.795977332042004,0.869368475249552,-0.00916600184669377
"Eny2",4293.66097485725,0.915238007633687,0.17224735434146,5.313509813
44733,1.07533641982552e-07,4.81867240885228e-06,11.2052700033847
"Ebag9",1345.97464278928,0.570708539505994,0.166373793970043,3.4302790
4748481,0.000602960816386332,0.00397986797491561,3.15402452872879
"Sybu",107.956171409452,-0.748887471142421,0.309682224308025,-2.418244
93742186,0.0155955749459933,0.0475275027996427,-2.28144578699526
"Kcnv1",6.91490645686535,-6.29920212165862,2.07394273823221,-3.0373076
3898908,0.00238701703067829,0.0114300111858909,-28.1669630587222
"Myc",872.741740988313,-0.640306155447184,0.245162736311,-2.6117597032
9735,0.00900775430370887,0.0311959368747572,-2.22024073349188
"Washc5",1888.23933187353,0.112659402074293,0.0770350939579956,1.46244
25866959,0.14361996654823,0.259287232199758,0.152069781974406
"Sqle",2515.11459799913,-0.389314891792547,0.26039335234196,-1.4951030
3658321,0.134887553520146,0.24702146318548,-0.544371246611204
"Mtss1",5524.2382504194,0.240007767405259,0.164265031169991,1.46110079
361252,0.143987782475704,0.259782898372469,0.323508630484661
"Ndufb9",11593.2470223499,0.555786981648856,0.44731152908974,1.2425053
8048921,0.21405015655589,0.348725764749894,0.585504600326804
"Klhl38",17.1200345031101,-1.28384450407717,0.509874237074854,-2.51796
307937145,0.0118035693225032,0.0385375074765853,-4.18035599272609
"Fbxo32",745.728647241441,-0.996974153766998,0.302321362515229,-3.2977
2975840164,0.000974698944415257,0.00575376325104713,-5.14229414401291
"Wdyhv1",670.664502358661,-0.356054283831905,0.160865629390847,-2.2133
6456507323,0.0268725215919657,0.0723802218538675,-0.934935241368635
"Atad2",678.791451595177,-0.518713702538861,0.234015290549087,-2.21658
038379358,0.0266517792694286,0.0719546375235833,-1.36510891034269
"Zhx1",2014.17278551223,-0.395272232107959,0.214581412981915,-1.842061
83851195,0.065466105020703,0.142525470711664,-0.770083020596069
"Gm29394",13.3748133772433,-0.272069510576482,0.956699192928948,-0.284
383547710056,0.776116465769145,0.855191308976103,-0.0425598558181532
"Tbc1d31",292.846068294789,-0.919685564987537,0.324370688049198,-2.835
29184008158,0.00457838406223217,0.018559112774512,-3.666597219662
"Derl1",6486.35429135119,-0.548230727388868,0.370753978838989,-1.47869
141986189,0.139222806530685,0.253248090468821,-0.752932228130787
"Slc22a22",2101.86375111593,2.41178942795708,1.00241114395274,2.405988
2439523,0.0161287837264861,0.0488245653002679,7.28245052676202
"Has2",15.8710086835833,-1.34343878097923,0.663813933962949,-2.0238182
9040397,0.0429888515747884,0.103634597923494,-3.04541994222413
"Mtbp",342.142403137069,0.0851441296121177,0.314099273838542,0.2710739
46054026,0.786334159643399,0.861835909821113,0.0126601135211147
"Mrpl13",2469.89199653598,0.604510634105113,0.252455467908714,2.394523
83072843,0.0166419619571735,0.0499534670756096,1.81151487120137
"Col14a1",2253.26708324022,-1.12300519990682,0.325212621764122,-3.4531
4149806075,0.000554098271041638,0.003743523246282,-6.27504767439049
"Sla",719.250820608295,-0.969851084094201,0.417878673435539,-2.3208915
5476801,0.0202926974645644,0.0581094162379315,-2.75964100257144

"Lrrc6",6.87158785978782,-0.382662308370882,0.877979045804384,-0.43584
4465992119,0.662949579509106,0.771344486291237,-0.0993468652911687
"Adcy8",51.7531788620989,-2.9945709663326,0.830953984619187,-3.6037747
2370503,0.000313628915390742,0.0023933998404261,-18.0723567519953
"Asap1",1492.4359844638,-0.400353197642491,0.19881336517606,-2.0137137
0223504,0.0440396005838463,0.105541314181887,-0.900255338508591
"Fam49b",1716.61055461691,-1.05261391457805,0.209042458578127,-5.03540
726481006,4.76833933511749e-07,1.51440601952762e-05,-11.6818062459316
"Wnt7b",681.450609186214,-0.79838886884116,0.433066372937787,-1.843571
60641483,0.0652455971586304,0.142124751804153,-1.55769666102158
"Ppara",548.393116642519,1.39284011119581,0.501755407801337,2.77593442
848809,0.00550433083834673,0.0213536495595075,5.35760491430437
"Gtse1",107.108429644829,-0.929542635643654,0.24525694040857,-3.790076
78272077,0.000150600724466501,0.00136768184706467,-6.12999723854227
"Trmu",1226.99805662258,0.349379944957291,0.17347814579209,2.013970943
49865,0.0440125842235737,0.10552845144998,0.785676774071148
"Brd1",3121.54017418822,0.130208111425681,0.11753908992975,1.107785601
39868,0.26795441989841,0.410156639910441,0.116043570941134
"Ttll8",6.23372114722878,0.0982949180863851,0.832434354658298,0.118081
284771979,0.90600325228608,0.94269979257559,0.005800127609678
"Tef",8962.20719969685,2.00865910068715,0.233704923541763,8.5948514487
6806,8.33725812671592e-18,1.8439802875533e-14,63.5223674274123
"Zc3h7b",4610.25848613667,0.125907830887093,0.098382424361501,1.279779
71374695,0.20062262037481,0.332178995590018,0.1387606674561
"Rangap1",3385.55895154384,0.123350825667138,0.0968727747970528,1.2733
2809373487,0.202901641611423,0.334978554733704,0.134907412176551
"L3mbtl2",926.819279245059,-0.0421487164518492,0.13893250415282,-0.303
375489478599,0.761603722602869,0.845664505366508,-0.00706549739926178
"Rbx1",4389.82439319685,0.417810650624939,0.137046070530127,3.04868756
184505,0.0022984338551675,0.011125834982139,1.87951511145484
"Xpnpep3",952.584540452144,-0.0244918012164847,0.223485294140218,-0.10
9590214025976,0.912734371985237,0.946353538447837,-0.00135045490796312
"St13",9843.29531858904,0.069483484487076,0.192050965145915,0.36179711
1690037,0.717503650684241,0.812751186954124,0.014406028861466
"Slc25a17",4029.75758717529,0.733900449170031,0.159506811338624,4.6010
6024947111,4.20345809607213e-06,8.70977959162747e-05,6.8608529222109
"Adsl",1514.34585532458,-0.000432809979072616,0.140051378431742,-0.003
09036572091691,0.997534248828849,0.998367315585527,-7.07219597696623e-
07
"Fam83f",242.625820666496,-1.49356936053618,0.397288031343337,-3.75941
191957387,0.000170313224551589,0.00149854117883885,-9.71307464639562
"Mief1",1556.7574069809,0.0949792780369907,0.109051635771589,0.8709569
31227764,0.383777669656095,0.528420950454034,0.0605836777813653
"Tab1",1548.94853342286,0.318979395407377,0.147382808413247,2.16429174
366789,0.0304419659827536,0.0795333582650046,0.8075214573506
"Syngr1",757.668836658358,-0.191692778103524,0.312922011316372,-0.6125
89626716027,0.540147718919084,0.670900765924595,-0.0765111134955091
"Cacna1i",12.727380385396,-2.29320147575447,1.04928399350566,-2.185491
71620629,0.0288528126717217,0.0763638036477847,-5.89867939944839
"Deptor",7835.1254574172,0.0686497237056612,0.375190494326751,0.182972

982374854,0.854819217660728,0.907625280140461,0.00665378334655757
"Dnal4",1184.98517795451,0.253722392404139,0.12203914931351,2.07902459
031686,0.0376150903501525,0.0935244875857272,0.601203322251158
"Nptxr",326.204795964313,-1.10819336735764,0.161351230396994,-6.868205
24783733,6.50146371837917e-12,2.8810349904763e-09,-21.7927513806799
"Dscc1",42.4914888572657,-0.743187163627021,0.402457549981012,-1.84662
249139589,0.0648018721568696,0.141474292163771,-1.45340450776765
"Enpp2",6468.90613145267,0.933349500199577,0.380338985856207,2.4539937
6584667,0.0141279430718261,0.0442658900874924,2.90975521656795
"Josd1",1508.56784393845,-0.327845496200603,0.206322169178183,-1.58899
791285865,0.11206086391757,0.214907320218823,-0.504078322241947
"Tomm22",3598.95487089979,0.12633813563355,0.22934850729794,0.55085658
5560541,0.581731990515383,0.706082404815144,0.043968618365828
"Cby1",888.003737190265,0.425910239288453,0.134542217836168,3.16562523
004551,0.00154750032970973,0.0082426806908815,2.04370032529768
"Dmc1",45.5112927274724,-0.489401351341829,0.542613667946004,-0.901933
327987105,0.367092285799347,0.512022704665259,-0.327598564623283
"Ribc2",10.7885643471202,0.087758705195839,0.52007676833523,0.16874182
9166404,0.865999715605976,0.915814650629735,0.00771761297636905
"Smc1b",2.70595363728305,-0.904448862330847,1.34149277645792,-0.674210
758494692,0.500177330735313,0.635504860854759,-0.410018812968687
"Csnk1e",3505.59563722615,-0.506959136389478,0.23684590247333,-2.14045
981414673,0.0323176250925164,0.0832402977614115,-1.26031242815788
"Fam118a",540.074135746066,-0.808230395270261,0.214364840436532,-3.770
34962274776,0.000163019007771175,0.00145072780169893,-5.28234324439021
"Upk3a",2680.62512398563,-2.46459349858594,0.71370703438636,-3.4532285
3753989,0.000553919504209099,0.003743523246282,-13.7714782646623
"Sh3bp1",1190.39118204706,-0.735139469644145,0.269955618080956,-2.7231
8640697333,0.00646555653355429,0.0242247158515068,-2.73499954069291
"Samm50",8134.25494588914,0.0797772244416399,0.353755052745752,0.22551
5434542717,0.821578319570134,0.885647443420974,0.00968785311104928
"Parvb",835.241664331141,-0.583755848238411,0.218710178512961,-2.66908
404632763,0.00760584154461226,0.0274119590456284,-2.09963896601377
"Parvg",436.566201592267,-1.29417375937448,0.38769600744463,-3.3381147
4589228,0.000843489087129496,0.0051718082455506,-6.81322032710571
"C1qtnf6",709.594462909718,-1.38768085405828,0.32587314255472,-4.25834
680078082,2.05944282332118e-05,0.000294607602121176,-11.2816597403147
"Efcab6",4.95126263421543,-3.83471707092847,1.41291603923618,-2.714044
54648382,0.00664672277031271,0.0247560268530195,-14.183415534493
"Ttll1",371.918524464457,-0.0759700827893785,0.132025444934545,-0.5754
20009582568,0.565007274655183,0.692644567194389,-0.0278991241620584
"Myh9",19454.4224063638,-1.00989861333417,0.175343399316686,-5.7595473
6402823,8.43397918011224e-09,6.968039239569e-07,-14.3170920632189
"Cyp2d26",2961.57825841334,1.90803580598987,0.970935288441043,1.965152
39347564,0.0493966020743498,0.115249872075022,4.12260272942544
"Adamts20",16.5186704285897,-1.43682058638723,0.712896559135767,-2.015
46853884253,0.0438555793083269,0.105269479488122,-3.23461611633609
"Ndufa6",6650.16238221409,0.772060680586221,0.291660346178439,2.647122
55437656,0.0081179926912104,0.02881045167332,2.73851239948891
"Twf1",4279.75380240032,-0.0087012120085015,0.163722854078388,-0.05314

5982932447,0.957615594115367,0.974254571044165,-0.000226950605743511
"Smdt1",6504.70722327323,0.540485200717018,0.25724009249233,2.10109238
991637,0.0356328568082737,0.0897171283636002,1.30316039563655
"Naga",3566.31221238252,0.188534817947642,0.168724071093302,1.11741505
954645,0.263816899065431,0.405702492150625,0.170083889276083
"Nell2",27.2759874334862,-0.611952082294809,0.493977248956101,-1.23882
645119389,0.215409775879668,0.350364007070326,-0.641804723325792
"Wbp2nl",0.601426376193038,-0.78109076708091,1.9580138527976,-0.398919
939184746,0.689952196784794,NA,NA
"Sept3",226.104441625789,-1.25260941577989,0.466950827456209,-2.682529
59868105,0.00730676856529459,0.0266294156048811,-4.54163459058762
"Slc38a2",7971.86330102677,-0.277057888303985,0.195310490238494,-1.418
55098497612,0.156029964424547,0.276369208425674,-0.356301319335683
"Srebf2",4869.19784086405,-0.168500606713577,0.0799323978110809,-2.108
03893449845,0.0350276217786563,0.0884330660935182,-0.40869979343267
"Slc38a4",104.376913400192,-0.0672191897658128,0.330317948038754,-0.20
3498447979964,0.83874545863635,0.896904662858871,-0.00731383146712225
"Rpap3",1046.50847542087,0.450250936901225,0.127977432481013,3.5182057
3496836,0.00043447538086932,0.00308862728392595,2.60246326160682
"Endou",254.079357671902,0.576044856558584,0.208261716836686,2.7659661
3774343,0.00567544277954412,0.0218479335272559,2.20259330363159
"Rapgef3",5480.66401527199,0.729194210674722,0.173014715892312,4.21463
692792808,2.50180330339559e-05,0.000342076864022491,5.8193162395673
"Xrcc6",1139.42308358643,-0.424408376920829,0.309419606960682,-1.37162
728984643,0.170179493874685,0.294617451775549,-0.51865994429889
"Desi1",1540.85741148847,0.0432222721890554,0.15948669031815,0.2710086
47197042,0.786384381558489,0.861835909821113,0.00642673634749429
"Pmm1",1743.46450553871,-0.363090041439871,0.367404495190186,-0.988256
938042956,0.323026829156789,0.468073440522315,-0.275632569004023
"Hdac7",4446.8506885378,-0.103999727788871,0.211358855162088,-0.492052
853471011,0.622681980312631,0.740533897659999,-0.0312398406709395
"Polr3h",767.322782358366,0.161293053723042,0.183603521324171,0.878485
622496656,0.37968023673418,0.524440723706649,0.104102226030654
"Aco2",26829.569807267,-0.193648619245583,0.525709219223622,-0.3683569
01808887,0.71260712900675,0.809438708777626,-0.0409400724976026
"Vdr",6537.82772515227,1.67210702688035,0.524777540539564,3.1863159104
7347,0.00144097158510997,0.00779580021267317,8.11669197496123
"Col2a1",9.37939444676961,-0.9178797853019,0.651544616235293,-1.408775
02849387,0.158901701288508,0.279904722352817,-1.16874184802941
"Hoxc10",1040.3926157532,-0.178656222719132,0.117782541320281,-1.51683
110855386,0.129309330669316,0.239255392806066,-0.25551836539304
"Hoxc5",354.914166128117,-0.578780991356861,0.278579598523102,-2.07761
442124723,0.0377448868479088,0.0937754591947663,-1.36988899687742
"Gtsf1",0.408927586926845,0.124646693396322,3.81533113091902,0.0326699
542239463,0.973937784151395,NA,NA
"Nckap1l",1772.94085133421,-1.20123243870482,0.413600213184466,-2.9043
3225228795,0.00368037365039123,0.0157852893610489,-4.98352518078968
"Pde1b",667.404081235077,-0.916279940407146,0.258971874993916,-3.53814
459747289,0.000402949328414882,0.00290989111312347,-5.35074463996109
"Ppp1r1a",4452.35847320858,1.72128413736868,0.555851332778569,3.096662

78708804,0.0019571237029117,0.00982492223464788,7.95720917509862
"Glycam1",0.536461702703298,-1.53282331635614,3.70754842849241,-0.4134
33120543062,0.67928931276694,NA,NA
"Shisa9",48.4154860263194,-2.82137380689806,0.522018780536798,-5.40473
621274086,6.49038391280888e-08,3.29556003989447e-06,-35.6140165891935
"Tnfrsf17",10.5768622080906,-1.07573431894344,0.638204381190911,-1.685
56398333726,0.091879811276568,0.18503124976973,-1.81501180713685
"Txndc11",1674.72935544049,0.338267811436095,0.117179207342221,2.88675
626937965,0.00389235621895822,0.016455585766966,1.38929644684915
"Litaf",4116.13677302743,-0.898045320116042,0.154414574718071,-5.81580
671225941,6.03421603314034e-09,5.52371569080612e-07,-12.9399753260728
"Prm1",0.144806447373948,-1.12082276384184,4.77798187819239,-0.2345807
90052278,0.814534120870031,NA,NA
"Nubp1",1417.50772157502,0.298638879847691,0.115812350440135,2.5786444
9441481,0.00991888070611666,0.0335469793595599,1.01382179388357
"Ciita",2038.40607098064,-1.04707170639028,0.481572559560938,-2.174276
09942086,0.0296844062450737,0.078024609458944,-2.67079825670246
"Emp2",3032.24233241563,-0.900379287134968,0.232100744761184,-3.879260
65494273,0.00010477443677675,0.0010276116540609,-6.1950758485114
"1810013L24Rik",2815.05653075093,-0.0124891336644746,0.130566786426598
,-0.0956532209015939,0.923795994778254,0.953075522841759,-0.0006002418
89060643
"Bcl6",3546.17474085961,-0.0722390696101149,0.26754421060891,-0.270007
971563669,0.787154120884729,0.862534059177838,-0.010682759988l357
"Trp63",487.578769720513,-2.79403194353317,0.65542181879594,-4.2629522
9027624,2.01743604653222e-05,0.000290783946044037,-22.7516065898485
"Cldn1",2551.26368899533,-0.880235451591257,0.460730035846807,-1.91052
326331061,0.0560658740392372,0.126921213516434,-1.81697211744348
"Il1rap",350.601920922822,-0.194869406592077,0.163218495055685,-1.1939
1743273698,0.232510267600398,0.369748405323593,-0.193881903974361
"Anks3",2504.22377205711,0.26720092661118,0.159326367662464,1.67706658
057535,0.0935294629591587,0.187443464773119,0.447368633071075
"Nudt16l1",2214.31082103661,0.229600258254249,0.163753039609999,1.4021
1295497827,0.160881519085484,0.282498906621827,0.290233231931398
"Mgrn1",4856.88164463189,0.291868836053662,0.124568276227152,2.3430430
6757392,0.0191271750586831,0.0555220573610544,0.843785481240917
"4930562C15Rik",21.4815157287255,0.689402971575117,0.516928448417844,1
.33365260450487,0.182317734363937,0.310348504953185,0.806642429641887
"Srl",145.579938980043,-0.901045946905924,0.416470933557148,-2.1635266
0967223,0.0305006993646081,0.0796547865270732,-2.27969413620083
"Crebbp",6266.16340208687,1.0368234127149,0.515962362816238,2.00949427
213196,0.0444847415268868,0.106322536162412,2.3238095160144
"Fgf12",139.979498101403,-0.653969509203832,0.617775862845685,-1.05858
701923288,0.289787901180882,0.433503490657114,-0.546623965668024
"Plaat1",44.8839750120848,0.122050775604226,0.483163913600355,0.252607
390926093,0.800571618086256,0.872675429610657,0.0166222879282878
"Zfp251",810.79959428021,-0.0842343209028633,0.1115289322765,-0.755268
7825795,0.450087725525571,0.590804528901143,-0.0443300013383957
"Hes1",3509.39228333429,0.293150037609234,0.216368113328786,1.35486709
709287,0.17545990434434,0.30179638259343,0.351194542079657

"Zfp263",1525.19541652997,-0.12278285619038,0.13265148825262,-0.925604
814599243,0.354651390381207,0.499710421278564,-0.0851777217945508
"Atp13a3",4246.20702487561,0.201403572101242,0.167076280266781,1.20545
879869751,0.228026234355653,0.364490532666545,0.203267502012008
"Mefv",50.6047231451446,-1.46872944480753,0.554764765386202,-2.6474814
8485073,0.00810938002967096,0.0287876964389303,-5.2107688846008
"Glyr1",5120.09643119709,0.0391857624799744,0.112756016335438,0.347527
021204799,0.728195422019809,0.821473868233177,0.0077060820700887
"Tmem44",713.507414663792,-0.209422662053029,0.202330993037775,-1.0350
4984040646,0.300645635811699,0.445270687964793,-0.169438199295475
"Lsg1",1344.92364771022,0.12468823270576,0.134356799139466,0.928038130
592331,0.353387790716158,0.498290074008074,0.0868544430542258
"Rogdi",3871.31692471871,0.740967173290244,0.169257193230714,4.3777588
3640133,1.19905920471878e-05,0.000195898824286807,6.32631269429437
"Sept12",0.136930709172513,-0.671103770306161,4.78471990596623,-0.1402
59781867135,0.888454739565252,NA,NA
"4930451G09Rik",107.675447324123,0.278262762222534,0.288549142116369,0
.964351375927201,0.334869791367105,0.479566007497306,0.204487995418887
"Eef2kmt",1601.61891876307,0.627331711396282,0.196763080286169,3.18825
925312767,0.00143132143171569,0.0077629778235306,3.04782166055438
"Ercc4",979.547132053459,0.353285301153294,0.147342179129129,2.3977200
77451,0.0164974672385772,0.0496477259172369,1.0608460532804
"Gpt",2032.97597678277,-1.26559982002265,0.578587017905938,-2.18739754
06555,0.028713515561081,0.0761278324574352,-3.25935154357029
"Apod",239.666275700349,-2.66266055004859,0.641367309877573,-4.1515376
7434273,3.30248886114543e-05,0.000419551884686937,-20.7057094284244
"Adck5",1735.51055221504,0.649508031914292,0.243025906482167,2.6725876
3197721,0.00752687123620574,0.0272400714308675,2.34022042045771
"Cyc1",13268.4386822226,0.512291699203601,0.393947036936424,1.30040754
510428,0.193461325309182,0.323675040404876,0.577872837985091
"Sharpin",2712.56661475904,0.642983814784025,0.231979333143881,2.77172
886942142,0.00557594587697413,0.0215329357712897,2.46788222980845
"Maf1",4984.90705652449,0.546213350983048,0.131556130069181,4.15194146
176095,3.29666597074904e-05,0.000419551884686937,4.24753163942531
"Hgh1",599.477127780038,-0.030603965031987,0.135110028789432,-0.226511
424105186,0.820803674964271,0.885129697165718,-0.00373432930134514
"Dgat1",3909.81155546145,-1.55986672594824,0.557160986355071,-2.799669
689999,0.00511549218526322,0.0202194897788058,-6.0852090302418
"Hsf1",2360.64006917961,0.143182845428363,0.198067086853147,0.72290074
8949387,0.46974086338467,0.607698276793034,0.0713160499530039
"Bop1",1471.75540913244,-0.310226890072164,0.125599928058495,-2.469960
73061191,0.0135127884892079,0.0427231812196955,-0.978149609796211
"Mroh1",4771.96062820629,0.96268319991324,0.284446119487615,3.38441319
448253,0.000713305698706547,0.00453473573960882,5.19462744547635
"Fbxl6",2114.02373461808,0.287834448380643,0.172960174110748,1.6641660
4204122,0.0960792521712122,0.19134670512103,0.475982699014907
"Slc52a2",1771.75775043498,0.851045723611042,0.171355608432821,4.96654
723702661,6.81554576347335e-07,2.02267140481284e-05,9.19853310747568
"Gpaa1",2421.80887743233,0.225956730113827,0.119465648313068,1.8913950
0186441,0.0585716352846415,0.131162253908343,0.458990457266696

"Oplah",5572.02049447018,0.876990249891267,0.251520982106726,3.4867478
750506,0.000488932055950452,0.00340106929251585,4.98451913857201
"Grina",16842.5769904334,0.95830883834039,0.188211450968711,5.09166064
768134,3.54940954834205e-07,1.2227276921126e-05,10.8402374848928
"Plec",8244.78445783343,-0.622411695913041,0.178843922978368,-3.480194
82880794,0.000501049275398375,0.00346803648268283,-3.52544360187688
"Scrib",3718.02403542057,0.295477080808396,0.257621922530297,1.1469407
4908803,0.251406109806021,0.39140181483534,0.277163584859342
"Tsta3",1447.89508351053,0.172612972127404,0.171525509958538,1.0063399
4423995,0.314252071982911,0.459420176915558,0.134256655595264
"Pycrl",1485.39836444171,0.582203691685909,0.200617906071064,2.9020524
7920231,0.00370726429636843,0.0158692072998006,2.41228804363834
"Naprt",1897.96081619926,1.01270309630635,0.249374284423324,4.06097645
011079,4.88679011877672e-05,0.000569491712762559,7.56566819109429
"Gsdmd",1709.35373439475,-1.1444437878177,0.238580708524649,-4.7968831
8009778,1.61153521487642e-06,3.98247320908244e-05,-11.5943856835131
"Ly6h",45.005569535618,-3.70444128618545,1.09567238193952,-3.380975323
68935,0.000722290291225257,0.00458139755963242,-19.9511990408293
"Gpihbp1",1236.12903717289,-2.3534951476909,0.650087924614903,-3.62027
205640692,0.000294293415872598,0.00227571126228481,-14.3221067774649
"Rhpn1",2486.38172711588,0.115396489607065,0.535463528016625,0.2155076
55646496,0.829371543947287,0.891116237608652,0.013302953774405
"Ly6g",0.526039523285424,0.899765651198382,4.37588020018041,0.20561935
200175,0.837088259816394,NA,NA
"Ly6f",13.9847010060125,0.853662228565457,0.6782209695345,1.2586786120
0365,0.208146439728379,0.341354022868744,0.917546169501837
"Ly6c2",348.543059255455,-1.8886443559915,0.439506062153075,-4.2971975
1017611,1.72971063035308e-05,0.000256081229085985,-15.6191187830427
"Ly6i",50.0522789585921,-1.25554198358849,0.748020330129332,-1.6784864
4350536,0.0932521747572501,0.187003075533977,-2.10507962658488
"Ly6e",24271.7108338047,-0.933146758219274,0.300501027397902,-3.105303
05436749,0.00190084159377138,0.00959922543262223,-4.33546783509906
"Gm9747",3.40821583400612,-2.37080378705034,1.38136727161297,-1.716273
3154102,0.0861120299393784,0.175849639689778,-4.12075562974139
"Lynx1",1452.83285195579,-0.361420556930587,0.226493795452516,-1.59571
945981345,0.110551441949786,0.212598088089253,-0.559606172088676
"Lypd2",142.922328559708,0.384044441638436,0.584175656305675,0.6574126
07822674,0.510915647002573,0.645112892452801,0.168338181071364
"Slurp1",125.020140362942,0.0737959986361152,0.914724033886008,0.08067
56966061215,0.93569986509962,0.959820915915004,0.00302626747872347
"Psca",3045.16694860871,-1.74906013151525,0.690790321569846,-2.5319696
5403401,0.0113423806957759,0.0374076012865761,-5.74720407012059
"Zbtb11",1073.39182698665,0.057631810174766,0.134738538190822,0.427730
706808957,0.668847191024274,0.776058869034312,0.014611214147258
"Arc",191.237129765969,-0.969677647464475,0.366843613317962,-2.6432997
9386613,0.00821023042513215,0.029032113316871,-3.43203120541153
"Mroh4",0.672425060946421,1.25756108794567,3.40860059281003,0.36893764
8663888,0.712174200248152,NA,NA
"Cep97",1099.26558402321,0.90034789711997,0.199971689648145,4.50237680
495752,6.71977073464493e-06,0.000124073948659192,8.09829873581064

"Ptk2",2333.95432914733,0.0829838186190501,0.138374008739953,0.5997066
88956318,0.548701729611123,0.678505127626474,0.0321863729594793
"Mapk12",1146.40271418717,0.114390160402154,0.199409947934033,0.573643
198783619,0.566209272867026,0.693725549978716,0.041830063201303
"Miox",52814.3653414503,2.51714342155521,1.10309848054452,2.2818845877
7741,0.0224961550003819,0.0630487322405413,6.95700010974242
"Lmf2",2398.43370595137,0.166787331873228,0.126638654723007,1.31703335
161004,0.187827445437607,0.316749656732612,0.19174597922541
"Tymp",52.6121069044872,0.0326306927168596,0.384931135301941,0.0847702
088095939,0.932444078190753,0.957752251771138,0.00140854119038589
"Chkb",4731.76251894586,0.286039618793065,0.2448288342716,1.1683248815
2008,0.242675742599024,0.381495737256774,0.27564368103213
"Mapk8ip2",13.6377487637499,0.363420340409177,0.687310628091174,0.5287
57050387075,0.596973995505035,0.719305337063229,0.119735860545715
"Arsa",1202.76438035927,-0.881504581247871,0.181604202808579,-4.853987
78010126,1.21003195665375e-06,3.19258499199389e-05,-9.12541875723709
"Rabl2",778.107203878859,0.318019068687023,0.191203214201083,1.6632516
8756092,0.0962620651114979,0.191604287476066,0.525470233228945
"Acr",15.9992663144848,-0.644491383494438,0.488747071167183,-1.3186603
4911538,0.187282688264176,0.316050755253476,-0.742358985786054
"Shank3",1960.86033180674,-0.702955604682548,0.304565948512957,-2.3080
571157568,0.0209959599561371,0.0597372285458575,-1.98078820390143
"Kif21a",3661.91935266928,1.28926702269833,0.283265923132704,4.5514370
6817971,5.32807352001633e-06,0.000104303993467147,11.8202590886922
"Yaf2",1303.44947585186,-0.00278909243693219,0.155048484197828,-0.0179
88517923036,0.985648013300511,0.990729196996256,-2.59777306821217e-05
"Zcrb1",3066.88398031741,0.536982228719025,0.249919269738441,2.1486227
5038262,0.0316643167574935,0.0819146673925445,1.34357099939986
"Alcam",2463.1036459808,-0.737048729312479,0.185211041197082,-3.979507
50964242,6.90581787906288e-05,0.000743509867509487,-5.3097937718025
"Cblb",1049.15149208877,-0.46826334783803,0.148378924668351,-3.1558615
8131735,0.00160024782945635,0.00846490292423761,-2.23447156053033
"Bbx",2743.54163600604,0.803575109600717,0.306809316168829,2.619135297
56877,0.00881529767380859,0.0306984593755885,2.79928829442347
"Retnlg",3.51730271750802,-0.228042687519765,0.910907153959294,-0.2503
46796079675,0.802319170291011,0.874026024376205,-0.0307048367583777
"Morc1",18.2688114934012,-0.2358461241889,0.417581840947569,-0.5647901
82575283,0.572216485391891,0.698408577913683,-0.0846572003067911
"Nectin3",3700.04232309849,0.962242291001328,0.3182744897522,3.0233095
0793607,0.00250026383342577,0.0118296879942091,4.26960662220278
"Cd96",63.0334442085193,-1.26202155173363,0.484057459412604,-2.6071730
2707219,0.00912932151520517,0.0315162023554303,-4.36312843298881
"Tagln3",62.8951766549482,0.241922769396975,0.369163723177667,0.655326
496641018,0.512257564693768,0.646514207806271,0.105517060384312
"Gcsam",133.29305546292,-1.39021132457852,0.539568452628499,-2.5765244
7582162,0.00997991396561935,0.0337183092187916,-4.71241919151038
"Cd200",1871.60909165928,-0.0172043759275489,0.201720638060764,-0.0852
881296278989,0.932032328331756,0.957507809105404,-0.000747038127385533
"Atg3",2896.78304990202,0.18135428735919,0.148470865008151,1.221480640
99468,0.221904091007851,0.357784335274215,0.186400450372869

"Slc35a5",1437.90595434566,0.22835044036397,0.138048634513459,1.654130
38070802,0.0981010244485688,0.194427096432018,0.373969062161883
"Ccdc80",3838.25968413524,-1.9685143267058,0.461696288746583,-4.263656
37906673,2.01108638357324e-05,0.000290783946044037,-16.0294744057421
"Cd200r1",183.2790764312,-1.77192681909301,0.40010736324657,-4.4286283
6793395,9.48342686089269e-06,0.000164070857971327,-15.4427181650864
"Gtpbp8",869.227034601681,-0.21954448992875,0.20270459860828,-1.083076
02015982,0.278774681776335,0.422562290119642,-0.189119665617862
"Mzt2",847.831301104738,0.435383306090306,0.206172636207663,2.11174147
112217,0.0347086298748074,0.087866910769301,1.05882258865327
"Prkdc",970.72835353528,0.0518902450038951,0.16526976317776,0.31397300
9981766,0.753541537885925,0.839812582812826,0.00905881879624399
"Mcm4",783.850389679472,-0.772178307864634,0.222897129090195,-3.464281
08345968,0.000531650670526389,0.00363341211844498,-4.33777580833478
"Ube2v2",1400.76231322753,0.117718298651567,0.15388049674758,0.7649981
71566001,0.444272633403456,0.58553655577265,0.063005972132647
"Snai2",339.700080893412,-1.16070520179786,0.252384721722733,-4.598951
92496239,4.24621747229945e-06,8.76113738358674e-05,-10.8440041050902
"Fopnl",1388.9875109394,-0.0850076226916248,0.192793899901867,-0.44092
485672469,0.659267397928175,0.768252194884268,-0.0224111734088263
"Nde1",1429.78332555554,-0.567788472499382,0.132552577239563,-4.283496
28746345,1.839791699219e-05,0.000270734026802808,-4.66402667859155
"Mpv17l",12162.6362641039,2.35140590916595,0.684635364285892,3.4345376
1203028,0.00059356569320225,0.00392716114219799,13.0264089137134
"Pdxdc1",4013.25014157393,0.203278823110502,0.151030187179498,1.345948
29620986,0.178319191681218,0.305302600300028,0.241180538201147
"Ntan1",1531.38686282565,-0.317203747586665,0.156502544163476,-2.02682
805753833,0.0426799999974068,0.102991910884831,-0.721037370395612
"Rrn3",1340.99002914544,-0.122518514088313,0.154517899825623,-0.792908
227633028,0.427831293370602,0.570849861403586,-0.0686874373826737
"Bfar",3496.6696622781,0.273159128019634,0.161258547761623,1.693920302
59026,0.0902804284208409,0.182451056524622,0.464718338076742
"Parn",1081.63538272738,-0.146611464350101,0.123353706434635,-1.188545
27024521,0.234618655466525,0.372493652331757,-0.144783993795491
"B3gnt5",71.6179476292587,-1.13014885375131,0.401847504313719,-2.81238
241277969,0.00491759999317404,0.0196241024696904,-4.44261147155115
"Boc",685.991555374009,-0.815011614480743,0.144687672594627,-5.6329029
2714964,1.77201211075358e-08,1.26097416552823e-06,-11.0708129473742
"Sidt1",244.103014484704,-2.56185785883559,0.614485261668473,-4.169111
96841327,3.05788746475363e-05,0.000396955324818684,-20.0636684163613
"Naa50",4142.74399729922,-0.298849956650352,0.245851922481615,-1.21556
892308906,0.224149178512571,0.360301787521691,-0.305070010464072
"Ccdc191",1425.91915812648,0.617002785846031,0.25122023373856,2.456023
45266558,0.0140483988864444,0.0440804122124063,1.92612213028462
"Hira",1594.43423329368,0.324812314555987,0.132299511819418,2.45512859
487599,0.0140834198493465,0.0441618720203534,1.01337982440341
"Qtrt2",800.027682236032,-0.287763451618958,0.177661588964444,-1.61972
800815459,0.105290716935116,0.204981322839246,-0.456057996196024
"Drd3",0.510567350266936,-0.0814421193884972,3.06028907875025,-0.02661
25576024851,0.978768757289289,NA,NA

"Mrpl40",1264.58781480373,0.0606547436246994,0.22469488352395,0.269942
700400805,0.787204335977528,0.862540609322273,0.00896920181015405
"Gbe1",2080.86733634817,-1.84647884920976,0.701227402617204,-2.6332097
7234791,0.00845820891967897,0.0297096302496668,-6.49274409825846
"Zbtb20",36902.9474452588,0.914671448455843,0.242883388043681,3.765887
22605988,0.000165958693388866,0.00146817722490749,5.96707288929836
"Usp7",5550.87666680638,0.0297674159396817,0.113469880351026,0.2623375
98731879,0.793061177171558,0.867106310351093,0.00424464571304853
"Pmm2",2500.36893700829,0.135686267988482,0.129756178225562,1.04570179
119033,0.29569873557637,0.440083500974586,0.111370039790838
"Tmem114",0.620744376399948,0.0676153978943893,2.27699606259992,0.0296
949999189655,0.976310299651507,NA,NA
"Dgcr8",1497.80111642877,-0.34172310662696,0.145175345061285,-2.353864
60753858,0.0185793769341221,0.0542793963832054,-0.995648053603648
"Trmt2a",2243.16706312636,0.207087974598662,0.213054775551763,0.971994
05205704,0.331053507599286,0.475848233775959,0.153795191515383
"Arl6",868.444995771884,0.649331667919121,0.200941411488224,3.23144772
95148,0.00123164848331191,0.00689902586318475,3.23131791568945
"Crybg3",826.29984959961,-0.688941730604334,0.214378037193674,-3.21367
682820013,0.00131047078351823,0.00722668523476335,-3.39646538549506
"Riox2",1164.70855996669,-0.0392592642370749,0.122890099477939,-0.3194
66453390922,0.749372825967863,0.836746727793002,-0.0069973297940307
"Gsc2",0.223225755326959,-0.743285423935834,4.78007376619995,-0.155496
642999869,0.876429800251749,NA,NA
"Cpox",3240.25682256846,1.03265667630308,0.248763167363161,4.151163885
10819,3.30788786450894e-05,0.000419630433195805,8.03007891630965
"Cldnd1",1387.75782240505,-0.579133404309982,0.178587234974278,-3.2428
6001960552,0.00118336335480191,0.00668788947592104,-2.89998557826548
"St3gal6",1724.74810004269,-0.434515278622288,0.235383234242656,-1.845
99077338851,0.0648935451388288,0.14159515925659,-0.849383203444622
"Cmss1",329.388592735972,-0.0801859630302531,0.243656233015805,-0.3290
9465125422,0.742084146962721,0.831943232375755,-0.0147535012015726
"Tbc1d23",2066.08312718638,-0.108063743440084,0.128912381735084,-0.838
272801926474,0.401877507472056,0.545859118125402,-0.0654211810090676
"Klhl22",1786.05767382381,0.0339479930813353,0.0846706270643264,0.4009
41793610955,0.688462978593569,0.791162324312925,0.00795238928857982
"Nit2",3466.27629029998,1.40153755010895,0.270441134678037,5.182412622
90774,2.19033985671171e-07,8.2456588118601e-06,16.4061514715353
"Tomm70a",3852.57304856217,0.294015548965073,0.163604933138342,1.79710
686789901,0.072318655621698,0.153786579486516,0.550452819267544
"Tmem30c",6.66923528220499,0.412559061654087,0.727843212926836,0.56682
4082888795,0.570833706781245,0.697419363044095,0.148673240912485
"Tmem45a",316.537978365031,-1.25398892846552,0.422856776063564,-2.9655
1693019818,0.00302174712881713,0.0136063859114169,-5.38866134257001
"Adgrg7",1.22566776228978,-2.08434980977541,1.54359244580419,-1.350323
92484241,0.176912101303836,NA,NA
"Slc7a4",328.097863120067,0.269556028030934,0.201631980911892,1.336871
39714569,0.181264618759527,0.309125886333083,0.316460579136386
"Tfg",2038.72788031775,-0.042019215052774,0.182843185043536,-0.2298101
24138721,0.818239317336424,0.883341465557035,-0.00521220808299009

"P2rx6",170.498256175648,-1.59037150733365,0.441623213672179,-3.601195
44919165,0.000316757328901622,0.00241161046424202,-9.58590146022933
"Lrrc74b",8.41192723660891,-0.103999126971738,0.821203872825342,-0.126
642275338924,0.899223535422171,0.938249076559186,-0.00662888617243116
"Thap7",1026.08859971847,0.501882917174433,0.116595757427545,4.3044698
0445505,1.67386119484956e-05,0.00025009153698986,4.16245809879205
"Lztr1",6083.54909509945,0.834190202678054,0.18394904545334,4.53489824
109827,5.76312642369883e-06,0.000110599841544567,7.59913231468198
"Ncam2",20.1298872624028,2.30770816210951,0.864863297843487,2.66829239
703397,0.00762378775330034,0.0274512675187276,8.29700224025233
"Aifm3",859.579985079997,1.50158469257505,0.414825218305488,3.61980088
556054,0.000294829801186531,0.00227847461891993,9.13601529440784
"Snap29",1940.04155362074,0.0542187547976201,0.224209736955903,0.24182
1588722009,0.808918409915106,0.878121647011892,0.00704681941283125
"Serpind1",10.844501654008,-0.727002939313815,0.565302243341657,-1.286
04290514805,0.19842808828531,0.329552884615585,-0.806986667355673
"Ccdc116",75.6251836008576,-0.352288990592747,0.318102244514786,-1.107
47093636547,0.26809037125419,0.410235917337482,-0.313897549802102
"Sdf2l1",1090.86879007731,-0.639497395862961,0.341659795346391,-1.8717
3733805761,0.0612429511893261,0.135701368838274,-1.27726726941851
"Dlg1",3552.1569960715,0.123263578408339,0.131986072947849,0.933913523
262745,0.350348502478065,0.495306507749544,0.0866023400805146
"Ppil2",3041.43272587286,0.0323137597729379,0.0887227870739905,0.36421
0377498509,0.71570091828263,0.811559273102535,0.00674704367221979
"Senp5",1509.69377135211,-0.00425661753733707,0.0952442966233805,-0.04
46915740705062,0.964353149906854,0.978482952401665,-9.25895806158912e-
05
"Ypel1",155.721404391447,-0.329204932229154,0.250511651109881,-1.31413
022416572,0.188802384576721,0.31800871583249,-0.377162350687104
"Ncbp2",2825.59325282579,-0.157492172462163,0.131549782195096,-1.19720
587776111,0.231226319452653,0.368157012631703,-0.157373386676115
"Top3b",3170.32174055837,0.480480678598037,0.145076663584455,3.3119087
9860792,0.000926617485239898,0.00554378267174211,2.49613470778833
"Meltf",64.9667351643844,-1.05360489528122,0.484649828428364,-2.173950
82692566,0.0297088283159175,0.0780580286257599,-2.68701148518721
"Pak2",4150.52347597305,-0.116138963413487,0.0802700083103595,-1.44685
376092702,0.147937885893169,0.265118832642004,-0.154183431548965
"Spag6l",48.1634998664081,1.31279058056221,0.512161615325161,2.5632350
0488951,0.010370182982668,0.0348737198682407,4.40575373604988
"Wdr53",640.355054042483,0.123042855108119,0.134043324688308,0.9179334
77062226,0.358653693941009,0.503675433913986,0.084385644465531
"Fgd4",2863.9422454362,0.587509412219169,0.241033099646058,2.437463622
55595,0.0147907023197267,0.0457397484266506,1.81234174410146
"Dnm1l",4534.90772175431,0.0239895650634903,0.111220749424322,0.215693
251373149,0.829226862029293,0.891058989024368,0.00276706794483651
"Igsf11",2717.40270989299,1.56907726053838,0.368506893787646,4.2579319
0572594,2.06326771133271e-05,0.000294652042427947,12.7561515923181
"Tnk2",2379.28651617479,-1.00649905029934,0.213406535399888,-4.7163459
5638474,2.40118011019576e-06,5.49509504560997e-05,-9.87281921711345
"Yars2",744.252448235747,0.46451036772997,0.226355132809674,2.05213092

349244,0.0401569403811895,0.0981546976999415,1.07822634151701
"B4galt4",718.617143958404,-0.342139564611413,0.124949835699724,-2.738
21540216855,0.00617736009554132,0.0233876052704592,-1.28492192924638
"Tfrc",2230.52178943544,0.809105059539132,0.255197096740097,3.17051044
026241,0.00152171364335743,0.00814225373715235,3.89235221540358
"Arhgap31",1291.5109882137,-0.621760547518525,0.254726084741409,-2.440
89861527028,0.0146507671980013,0.0454584116528692,-1.92183536576113
"Fyttd1",2547.19816275962,-0.0890492999443957,0.125529095135165,-0.709
391713917083,0.47808142833616,0.615532995886056,-0.0432126622839126
"Lrch3",2313.66579254162,-0.393240427996517,0.145730376151087,-2.69841
084873631,0.00696713963331681,0.0256695146896677,-1.44022387347875
"Lmln",440.119218698346,0.912486652479443,0.288362826500415,3.16436991
394981,0.00155419122113694,0.0082683821090663,4.37566223023296
"Popdc2",51.6202985054296,-2.26227709270019,0.574484347571488,-3.93792
642438997,8.21887858972506e-05,0.000849690852292997,-15.9957423279997
"Maats1",74.39053916936,0.324951643014353,0.261323450256974,1.24348443
545656,0.213689372972748,0.348312606104552,0.34271184550175
"Osbpl11",1758.11923887438,-0.440450833237863,0.317384957233376,-1.387
74955523174,0.165213320390303,0.287971420785887,-0.548314615956659
"Snx4",1836.15229428461,-0.184731360106969,0.116825398576553,-1.581260
25982201,0.113818542106005,0.217589437453355,-0.281742166792377
"Nr1i2",300.371963736699,1.33472033698991,0.657961275423222,2.02856974
543278,0.0425021327271556,0.102664343894212,3.0382111022407
"Zfp148",2271.43957120675,-0.0426976446549152,0.148543910959035,-0.287
441231210683,0.773774494621053,0.853786279311295,-0.00674940348985669
"Gsk3b",3438.52754103377,-0.00540364781219187,0.189015593861965,-0.028
5883704184643,0.977192887356466,0.985481892917987,-7.90257882262355e-0
5
"Umps",1120.61993645807,0.313183406542631,0.129720535244757,2.41429320
309014,0.015765769721329,0.047901231264627,0.951643505610145
"Fstl1",9191.03766831157,-0.885044729809498,0.28184702972401,-3.140159
86145446,0.00168855664628166,0.00882666956815008,-4.18624166545213
"Itgb5",9948.64627479024,-0.415314501041335,0.248288727878899,-1.67270
783732035,0.094384825621584,0.188924682267698,-0.692082929384557
"Cyp2ab1",16.480896590225,-2.3647059833993,0.705442617012373,-3.352088
3575394,0.000802044167840409,0.00497083826590982,-12.5427949403493
"Ndufb4",2455.97925876329,0.904519412700593,0.266952693799489,3.388313
48665838,0.000703238387194981,0.00448242630713558,4.89127084797979
"Hgd",7898.03662265207,2.29886827008704,0.983713839375002,2.3369278524
6126,0.0194429358286328,0.0562043532671137,6.61789246906164
"Abcc5",4457.61803086582,-0.541857567690904,0.211480162440745,-2.56221
463723685,0.0104007019362568,0.034970320116078,-1.81698690913502
"Muc13",5.45409843118074,-1.52445569935768,0.802749747006036,-1.899042
26696221,0.057558918903303,0.129579009441935,-3.1151710613305
"Rabl3",417.633751034017,0.139223124843134,0.115556271795067,1.2048080
3577706,0.228277417109469,0.364742528788038,0.140415372499484
"Gtf2e1",503.474191628115,-0.749073727513394,0.237754269785527,-3.1506
2155640409,0.00162923444570164,0.008583305382948,-3.56404500228381
"Stxbp5l",65.5647188036736,1.63298799997751,0.638293144367722,2.558366
81685671,0.0105165095025995,0.0352866808263095,5.46111962894002

"Hcls1",1784.30386371204,-1.34034147517719,0.297653425704412,-4.503027
21027046,6.69923026008725e-06,0.000123901469933686,-12.0577439522646
"Ccdc14",268.832532747128,0.0491102403593415,0.14969540305859,0.328067
792035805,0.742860405723081,0.832478429265397,0.0090042627904267
"Mylk",19417.7807795189,-0.334588647272382,0.272812227179683,-1.226443
00342158,0.220032011572839,0.355799705406477,-0.34575966943055
"Iqcb1",918.975807573179,0.160148337201603,0.260469767556321,0.6148442
43553044,0.538657587497395,0.669812533151446,0.0641806323547265
"Eaf2",488.289339317098,1.38886830473573,0.321335953875045,4.322169019
64168,1.54502754849216e-05,0.000237391230421593,11.5912185095389
"Adcy5",772.534488494487,-1.5839821763732,0.404152299944325,-3.9192704
7450035,8.88173981405142e-05,0.000900904965479376,-11.1070584984473
"Ap2m1",13401.0570848392,0.0960670602181736,0.0969348866439446,0.99104
7326140085,0.321662471606829,0.466927849269652,0.0731628027903272
"Ece2",27.7356078753837,-2.28105836557029,0.634619578574632,-3.5943712
4630411,0.000325175900205819,0.0024632011275109,-13.7007060424841
"Clcn2",975.383838903438,0.835139731976035,0.218934390801831,3.8145662
2195077,0.000136422351773691,0.00126725250828176,5.5711370631999
"Pdia5",804.455306437009,0.256271280740216,0.235017555939845,1.0904346
2610858,0.275521735945486,0.418944303787341,0.222960446306477
"Thpo",434.326766175476,1.00567954860859,0.266370530298763,3.775491033
0381,0.000159692769488659,0.00142895347383657,6.58801859962472
"Slc49a4",1483.08301693141,-0.12796750286229,0.224301192178283,-0.5705
16374075161,0.568327519638125,0.695272303796221,-0.0465100072443873
"Hspbap1",392.979229297842,0.270622846341366,0.175547900426361,1.54158
976372883,0.123173314585844,0.231015129645516,0.39653609941272
"Ehhadh",13774.066495656,1.91560651441484,0.592533531215521,3.23290820
434276,0.00122536930475769,0.0068715130091038,9.54043106277665
"Senp2",2611.88611000183,-0.404308569883736,0.133319874019518,-3.03262
040155053,0.00242440359949331,0.0115597306871762,-1.80330828798523
"Tmem41a",639.23531818221,0.433423690223746,0.251638011926893,1.722409
45199355,0.0849953726690332,0.174244535411714,0.757319302726555
"Tra2b",6075.17369890127,0.0452956098693426,0.161976259109859,0.279643
511452017,0.779751021033312,0.857497366018682,0.00696361894925215
"Chodl",45.7818622041059,0.0554779083285909,0.416873015482379,0.133081
073296134,0.89412926331702,0.934584130811368,0.00375328180103208
"Dgkg",162.654229158727,0.583084634884143,0.419992548029479,1.38832138
241466,0.165039203954518,0.287778071785476,0.726270209733563
"Btg3",847.785484892363,-0.502590086285905,0.153335793570911,-3.277708
84137029,0.00104653282403835,0.00608931572757378,-2.56382238062176
"D16Ertd472e",500.487572778312,-1.34565028229283,0.20558142689666,-6.5
4558294786645,5.92637530726887e-11,1.60489535751567e-08,-24.1512228628
326
"Cxadr",1862.12168067149,-0.100510616431699,0.192589412524244,-0.52189
0664259884,0.601746458765931,0.72340171730798,-0.0325443913461251
"Usp25",1940.13428917924,-0.56105417862349,0.155532797990089,-3.607304
60632003,0.000309394367211167,0.0023638602554401 7,-3.39295230739714
"Ahsg",25.5294112688829,0.819123218724093,0.741421229943253,1.10480140
794833,0.269245650322149,0.411519298422925,0.727298983482743
"Fetub",109.759704553464,0.845406306406062,0.347530700380333,2.4326089

910355,0.0149904791832088,0.046167171554921,2.60003551825219
"Kng1",293.556177564135,1.73920586103283,0.417124168468151,4.169515919
9715,3.05247294316358e-05,0.000396516438679571,13.622839595217
"Samsn1",168.681557097827,-1.09356491108336,0.386703693261429,-2.82791
431822208,0.00468523445244045,0.0188901367563449,-4.34048528341853
"Hrg",76.3793530416127,2.47999770178056,0.767147987870926,3.2327500573
4334,0.00122604781312966,0.00687334107544625,12.3506488620314
"Adipoq",7592.80993078841,-3.73876695398592,1.61610724872086,-2.313439
8765584,0.0206984667876273,0.058971721559066,-10.5833173185296
"Rfc4",244.061522586727,0.408011481184597,0.228992025050654,1.78177157
52081,0.0747864893798661,0.157863697080073,0.753198698887392
"Robo1",287.129703394696,-0.798648273086115,0.235496737315513,-3.39133
476832889,0.000695530876685374,0.00444201147514295,-4.32599654730386
"Eif4a2",23609.0921063226,-0.157921939945735,0.0996318202105125,-1.585
05525254944,0.112953781178538,0.216266770631009,-0.241816799412736
"St6gal1",1719.04767519908,-0.904317554264641,0.302523022005028,-2.989
25201880871,0.00279661356197495,0.0128362455817767,-3.93873921299847
"Masp1",153.545503054279,-1.02049242644345,0.343352673855287,-2.972140
61269713,0.00295731209056088,0.0133910058387729,-4.40155936346522
"Mrpl39",1418.46224019742,0.0435114920623635,0.277118333330094,0.15701
4122954233,0.875233717653033,0.922100125725339,0.00352884575452266
"Atp5j",11477.2943987305,1.12490104968683,0.202416520591001,5.55735789
945615,2.73888796897382e-08,1.73264480341006e-06,14.9227815587058
"App",30484.783492425,0.740084808010058,0.230491785120448,3.2108945124
5871,0.00132322498696916,0.00728820361466797,3.64232603645788
"Adamts1",5274.3166643952,-0.714607435082759,0.239235570165124,-2.9870
4508944688,0.00281688173379578,0.012898910297217,-3.10897999983643
"Adamts5",3784.40840124265,-0.527571972409897,0.232616062793651,-2.267
99459192074,0.0233295363002965,0.0648715485999973,-1.44309195813586
"Ets2",3995.20876384727,-0.803697489819866,0.150033078027385,-5.356801
9825146,8.47078680303481e-08,4.02362491182574e-06,-9.98459700019964
"Dyrk1a",1905.99236359199,-0.234205365689431,0.127014385750355,-1.8439
2786931835,0.0651936529067934,0.142075091581162,-0.457028244558601
"Vps26c",1491.13795032732,-0.4617575541027,0.167044786760564,-2.764273
95944159,0.0057049618618683,0.0219312591448557,-1.76384103827002
"Slc15a2",4362.42188137509,2.05355164166159,0.676286919792428,3.036509
47779958,0.00239334584329652,0.0114516459515572,9.17859920160025
"Ildr1",1111.48919959096,0.0527421688023214,0.207678301946657,0.253960
901586476,0.799525764006564,0.872022897947082,0.0072224913040029
"Cd86",616.245250567691,-1.0401759695166,0.421028266910646,-2.47056088
929381,0.01349013512028,0.0426720724489325,-3.2809340832006
"Stfa2",0.251703376073965,-0.551680863350111,4.78471990596017,-0.11530
0555558728,0.908206898028326,NA,NA
"Kpna1",1511.67891482465,-0.185634713153171,0.207314843434539,-0.89542
4129202724,0.370560406814716,0.515716403431358,-0.12292698596553
"Parp9",1601.52204632464,-0.795737593602377,0.220191814779446,-3.61383
820919694,0.000301697365054559,0.00231776801569495,-4.8278598332546
"Arl13b",768.431745893057,-0.356376182423178,0.151776080169787,-2.3480
3917734937,0.0188725361252919,0.0549711247939859,-1.03382850114799
"Pros1",2994.35783543452,-0.83863259129972,0.234773660712426,-3.572089

76831076,0.000354143968693445,0.0026285112682089,-4.98259938985927
"Psmg1",1244.32648915379,0.368280983543173,0.239748658522064,1.5361128
0168761,0.124510717082346,0.232873857234684,0.536680541094561
"Brwd1",4194.48638479716,0.0989855986547826,0.159004795860912,0.622532
15771787,0.53359200523401,0.665769304284426,0.0402685351418897
"Grik1",11.8637019174573,-1.02606525608477,0.676937466719095,-1.515745
99801336,0.129583591712203,0.239558250801605,-1.46620484708044
"Fam3b",21.9423655220658,-1.7705004550299,0.562364735790219,-3.1483134
3850542,0.00164215507981791,0.00863038807177616,-8.41424287405002
"Pigp",1534.08547747769,0.375102262199039,0.165958475592026,2.26021756
864741,0.0238077510054037,0.065807134831778,1.02066338861938
"Ripply3",277.597586661205,-0.265697947911524,0.161357532547868,-1.646
64111873861,0.0996318334758317,0.19678094500727,-0.431935621528105
"Chaf1b",236.854639948855,-0.291181519508527,0.37015217828634,-0.78665
3534923349,0.43148471123044,0.573606661183004,-0.16184200126347
"Dop1b",1178.96645318525,0.00109775498891821,0.184130743339817,0.00596
182347937563,0.995243181270461,0.996930467424169,3.37477684634392e-06
"Cbr3",226.044284099012,-1.29815147711313,0.250146023021375,-5.1895747
189321,2.10774915171053e-07,8.05821430589255e-06,-15.2257831593938
"Setd4",344.858511492378,0.00781703809117145,0.259429994412757,0.03013
15894828045,0.975962107398849,0.984902912378384,0.000118914415494452
"Clic6",93.185008588416,-1.58964207195265,0.553842353856647,-2.8702067
6711211,0.00410203466932159,0.0171156584597544,-6.46628487481734
"Rcan1",3325.69021733116,0.49147208967543,0.197881084073278,2.48367392
960831,0.0130034815218966,0.0414081141130066,1.56498397129282
"Runx1",1060.19836473113,-1.39087902768774,0.351852235573723,-3.953020
29393049,7.71708892285927e-05,0.000807663981846001,-9.90495646201295
"Atp5o",12306.8269425078,0.79770088569146,0.304633554627392,2.61855883
429242,0.00883020630601265,0.0307284245144806,2.77804691822331
"Itsn1",2082.74561911366,-0.42327325538591,0.279208479413769,-1.515975
64756852,0.129525510275496,0.239533106835844,-0.604884418442455
"Donson",436.473985173865,-0.439016377283597,0.245978443360005,-1.7847
7581729009,0.0742976873671535,0.157138117831084,-0.812456934909006
"Son",26477.1664948773,-0.159631077526576,0.141853751865258,-1.1253215
0491306,0.260452820146141,0.401890694055215,-0.145515720674761
"Gart",2564.57655341823,0.582365165794505,0.209781589914972,2.77605468
635521,0.00550229527198652,0.0213500006394414,2.24018612698904
"Tmem50b",2066.99308780368,-0.518821745834908,0.226727718520641,-2.288
30312067765,0.022119874911575,0.0623032719938642,-1.4401149652044
"Ifngr2",2182.05528520851,-0.779664534685667,0.242895913217417,-3.2098
7094578156,0.00132794579023751,0.00731213979611719,-3.83456118251243
"Ifnar1",2928.2080863277,-0.442920370553073,0.186652776055642,-2.37296
428112613,0.0176459702878786,0.0522095794648038,-1.30771764873262
"Il10rb",2779.33255783299,-0.327273610979493,0.157105924596907,-2.0831
3984223823,0.0372384786310879,0.0927655367764088,-0.778151945244048
"Ifnar2",3113.05017768199,-0.824902401363758,0.239448741827484,-3.4450
0620495252,0.000571046282814298,0.00382095416002512,-4.59244210713374
"Cfap298",1290.55046656686,0.531167885806482,0.132709816516214,4.00247
622783493,6.26829676970469e-05,0.000688559157271561,3.8673852110624
"Synj1",2109.45564886339,-0.0450272426820255,0.121665408676213,-0.3700

90752761583,0.711314871956661,0.808370774226904,-0.00957884555929312
"Paxbp1",4221.8596208658,0.234412067173266,0.16709262714773,1.40288695
662208,0.160650550985459,0.282245849983283,0.296525821784613
"Mis18a",388.37354208925,-0.715278139774534,0.234381601645913,-3.05176
743716909,0.00227498287186471,0.0110369783616865,-3.22340377992299
"Sod1",21124.3177934395,0.809219193951443,0.385891917397022,2.09700995
918731,0.0359926937369083,0.0903780479693802,1.94516377178492
"Scaf4",2355.36425008295,0.0152834367989991,0.170517195199779,0.089629
8861888562,0.928581334238002,0.956091596063947,0.000686250135149079
"Krt75",1.40563516846807,-1.75363023016981,1.73239574567645,-1.0122572
9429685,0.31141504817892,0.456539143563352,-1.37498785602339
"Zfp641",1196.45146449049,0.297572713660143,0.233335883369772,1.275297
69259095,0.202203894140353,0.334257865871013,0.326092835509198
"Lalba",1.61401089164752,-3.38007065024165,1.99770792870126,-1.6919743
8808739,0.0906508570942184,0.183023914066839,-5.73982795759736
"Kansl2",2393.04545775858,-0.0465208800966887,0.118054655802587,-0.394
062222962911,0.69353510078302,0.795537557069497,-0.010641056804572
"4930415O20Rik",25.4332186749877,0.73062357803207,0.504357828084669,1.
44862146941718,0.147443322154805,0.26449994437155,0.971666699042847
"Adcy6",11029.1611713064,0.200934162398565,0.163744020579447,1.2271236
6343218,0.2197761151115,0.355529303280846,0.207795582762763
"Enah",3067.1628223279,0.0246319560153529,0.211955601295465,0.11621280
9969651,0.907483884655087,0.943183410607936,0.00144083440258548
"Wnt10b",62.1462922557584,-1.04651826983162,0.381261910704604,-2.74488
020032624,0.00605329908286175,0.0230072564361868,-3.9474099995223
"Wnt1",0.391792154252236,-2.45213200592348,4.63638300165379,-0.5288890
07885849,0.596882447668391,NA,NA
"Lmbr1l",2237.40912377643,0.472098383345258,0.24450491951408,1.9308338
8376597,0.0535035986223487,0.122392393474921,0.991653538750728
"Dhh",92.1113219642044,-0.834096400651221,0.336500929307251,-2.4787343
1543967,0.0131849477280566,0.041869724886262,-2.64674825067895
"Tuba1b",14272.6933714313,-1.27991813056287,0.241062366015576,-5.30948
962178596,1.0993264318128e-07,4.88261201992845e-06,-15.6531814467583
"Prpf40b",3393.70306991745,0.322464181539112,0.207373234735516,1.55499
422068804,0.119947445746254,0.226402255291429,0.479001829456581
"Fmnl3",2808.16151733173,-0.568233978595955,0.210620666969452,-2.69790
228457671,0.00697779193157429,0.0256849135514132,-2.0807884359575
"Nckap5l",706.98399931408,-0.44790876579581,0.257176903789747,-1.74163
682350727,0.0815720136400319,0.168824023134842,-0.79678418225007
"Tmbim6",71904.4168601121,0.899599779259371,0.269588355484238,3.336938
56191785,0.000847066706883472,0.00519047977712568,4.73273069616551
"Faim2",11.5984416269144,1.31422578107958,0.730044320566374,1.80019999
341956,0.0718290648851501,0.152995751270555,2.46725536647731
"Aqp2",5646.71826119541,2.0646475547684,1.14519785089553,1.80287410874
364,0.0714079880480868,0.152365173009586,3.88458314090225
"Racgap1",426.357910699472,-0.705461688272947,0.313969017678845,-2.246
91497743435,0.0246454613325608,0.0676527108351782,-1.90006783320758
"Asic1",27.4149490962386,-0.304648774154565,0.618941684754967,-0.49220
9171975826,0.622571481165453,0.740499506463554,-0.0915257261981776
"Smarcd1",1537.73823372555,-0.0906638309094112,0.123954122036799,-0.73

143054397574,0.464516214421173,0.603406872004266,-0.0458000639044042
"Gpd1",24084.166694952,-1.6412553957821,0.794454148143625,-2.065890649
09179,0.0388388060068195,0.0958459738667215,-3.84876493562143
"Cox14",3623.13344854754,0.157869030493171,0.193232757028343,0.8169889
66679263,0.41393473333393,0.557382557358078,0.0922749941218173
"Cers5",2399.32582595654,-0.140156453127605,0.105286940270099,-1.33118
554654598,0.183127965984006,0.311274438214293,-0.163573837876713
"Lima1",4615.46758680736,-0.638565063652612,0.151924997748448,-4.20315
993494319,2.63214471694068e-05,0.000353210995808048,-5.0755992603911
"Larp4",2692.45909268369,0.212206815545782,0.190595712577349,1.1133871
4117015,0.265542195213202,0.407487936586417,0.1905073848492
"Dip2b",1016.84727227866,-0.401535294455585,0.143082382415596,-2.80632
239746531,0.00501105277784465,0.0199195733052834,-1.57243327259943
"Atf1",1401.19234976489,-0.519358642866505,0.150814242604812,-3.443697
58383771,0.000573817145509507,0.00383029329104566,-2.89013419564873
"Slc11a2",2867.50480945116,0.457383711956222,0.21405909798255,2.136716
99202203,0.0326210171047257,0.0838666391756252,1.13363804895864
"Cela1",2742.95357883303,1.61837675565324,0.527892740455745,3.06573027
364621,0.0021713910087245,0.0106483354849372,7.35123639358373
"Slc4a8",683.726401788272,0.406907028624417,0.231170125313993,1.760205
94387672,0.0783728945577159,0.163644752418756,0.736525055661391
"Scn8a",35.0613523065729,0.107903468538092,0.438575628480544,0.2460316
11268338,0.805657762194991,0.876455674121405,0.0142291383925142
"Nr4a1",2123.57166479139,-1.35785125989325,0.55997060304617,-2.4248616
8471472,0.0153142201523584,0.0468533915786419,-4.15601864031022
"Pcdhgc4",2197.40067046251,-0.705757397689138,0.169973202878258,-4.152
16861092293,3.29339461252158e-05,0.000419429184552225,-5.4884041689910
7
"Krt7",11408.7746955124,-0.512615865923256,0.312884157490511,-1.638356
73251945,0.101347299378033,0.199381459315194,-0.826611234580043
"Krt18",5671.32857339311,-1.56182945487652,0.367974471369137,-4.244396
21875224,2.19182776933856e-05,0.000308120099831026,-12.6274238106498
"Csad",9619.64722741192,0.492608720085771,0.348030230592944,1.41541934
229824,0.156945581781934,0.27763790179497,0.63124729946694
"Soat2",147.800620782979,-0.410290611258307,0.290825633478319,-1.41077
870733459,0.158309874743219,0.279190117379096,-0.523474324969955
"Igfbp6",747.405323704023,-2.93109879011765,0.664738948443183,-4.40939
830136668,1.03658222619524e-05,0.000176068399660679,-25.3382879944979
"Amhr2",21.8348037304299,-1.05512923489944,0.65275091400805,-1.6164347
107853,0.106000358722177,0.205877461884435,-1.66760446210216
"Prr13",4734.59241995025,-0.140330160004079,0.153823958193488,-0.91227
7655913417,0.361622573528424,0.506423787711954,-0.0954780356514851
"Map3k12",1079.23377288565,-0.305099793458848,0.235028098130868,-1.298
14177915427,0.194238620056992,0.324655331109621,-0.343234576375449
"Tarbp2",1677.85982263564,0.0468785070680063,0.112690021721473,0.41599
5190628965,0.677413522465914,0.782784567006214,0.0114804413155234
"Npff",383.453593625331,0.0514242191919334,0.239867659866432,0.2143857
95986706,0.830246217336957,0.891712060462487,0.00589383264530617
"Calcoco1",3266.7315126536,-0.142434491012077,0.180592821699599,-0.788
705163757864,0.430284349164166,0.572841593544606,-0.0793568142709964

"Fabp2",1.78297789671913,-2.2770776942997,1.8820924636491,-1.209864944
61849,0.22633072061523,0.362469950476403,-2.31080964779281
"Sncg",566.637324685265,-0.614595215713843,0.278875332383177,-2.203834
99129061,0.0275359507663038,0.0737352710903564,-1.60241813735603
"Rttn",357.484506384041,-0.0666024221918896,0.234987499511658,-0.28342
9639152296,0.776847510720667,0.855664487856263,-0.01038178130918
"Cdkn1a",2397.83643455725,-1.88465233116936,0.255599163537648,-7.37346
83051565,1.66245225610165e-13,1.66632367440301e-10,-42.433382822235
"Nus1",10033.2307573795,0.979092483048413,0.262926036797691,3.72383235
594798,0.000196221249875541,0.00166997901792811,6.26124181213121
"Rgn",146.216320111465,1.34757441060316,0.393326986290303,3.4260919224
3055,0.000612333113994908,0.0040253779692086,7.43205677408466
"Cep89",1488.55879946097,0.697270406990068,0.233847321702965,2.9817335
5979568,0.00286621325557955,0.01305734253675,3.02504118083591
"Slc10a2",1815.35684626479,1.16279600822366,0.607470668235763,1.914159
92413377,0.0555997246738644,0.126056212987327,2.40818232003585
"Mospd1",790.004108764026,-0.26793573405309,0.24093154087311,-1.112082
43255375,0.266102709271373,0.40788893218698,-0.240274147113403
"Akirin1",1836.90350927811,-0.710534634081005,0.232003726528939,-3.062
60009143593,0.00219423080431814,0.0107384496840819,-3.22151042110921
"Cxcl13",131.672172406315,-1.84527149117629,0.715581737047536,-2.57870
120999707,0.00991725249685044,0.0335469793595599,-6.2643432574508
"Gtf2ird1",1120.86500587115,-0.260109790504119,0.248803500051706,-1.04
544265032471,0.295818433325696,0.440218027277462,-0.213416222626208
"Lrrc71",54.5761040562543,-0.328942223389528,0.477079816110814,-0.6894
9096625191,0.490514356208272,0.627027399026348,-0.153538730287492
"Noct",34808.6584521778,0.401319153882408,0.177030400084427,2.26695049
94115,0.0233932497918,0.0649803804045465,1.09707408908642
"Abcc1",1747.98213592139,-0.473917441304508,0.114522472882172,-4.13820
475036463,3.50033936853888e-05,0.000440320109727556,-3.66243804951338
"Ndufa5",4606.50046558019,1.16306189351087,0.283133778783427,4.1078175
0771076,3.99415491151196e-05,0.000487089909119285,8.87074497811918
"Fhl1",6602.7631112999,0.254240173617786,0.265248851450393,0.958496793
586812,0.337812307563949,0.482398152400899,0.185337390828273
"Msrb2",1221.1205446314,0.755587841579316,0.209429085202645,3.60784578
153606,0.000308749910416091,0.0023598611341799,4.57066608322037
"Rfc2",2227.23809939106,0.359661866668241,0.142391656280396,2.52586335
50831,0.011541436223288,0.0379165835689419,1.17694551625209
"Denr",2947.42101289449,0.239272345353837,0.187401387950457,1.27679067
893079,0.201676157183535,0.333555154191582,0.262708365109403
"Prmt5",2231.56395027751,0.237182532479173,0.170806980016969,1.3885997
6597917,0.164954488575257,0.287681808429371,0.295505774487145
"Sympk",5201.17715537337,0.322082909226945,0.17741076272129,1.81546431
730827,0.0694525847681716,0.14920521130562,0.612741046868641
"Ldc1",2069.48005972486,1.66925457353069,1.11114444098762,1.5022840523
2986,0.133023765481626,0.244319647641367,2.35244354608856
"Sult1c2",1851.44178393886,1.61286667201323,0.639448820316892,2.522276
40550489,0.011659804951411,0.0382008232774158,5.26584551338755
"Gzma",32.0178659322905,-1.62943867826,0.399754474169694,-4.0760986644
2247,4.57975928356045e-05,0.000540945058729681,-12.256951957691

"Reg2",67.0392948448351,0.897176369588096,0.483885390375388,1.85410923
213054,0.0637235341741772,0.139636038360093,1.76628544449694
"Nagpa",717.260570402237,-0.340917416379102,0.166885099469612,-2.04282
717547938,0.0410695493912474,0.099959315195424,-0.785130090704073
"Wrb",700.825791729579,-0.544425595605579,0.170691376935668,-3.1895319
2234642,0.00142503399511289,0.00773533208148972,-2.64697376125195
"Ivns1abp",34948.2231112527,0.863001534145602,0.292935574119567,2.9460
4551440841,0.00321865035204275,0.0142565298873534,3.66822274765105
"Lrrc69",2.14097976175493,-0.30414859389993,1.52373730185721,-0.199606
975250405,0.84178797210871,0.898810682885693,-0.0324474397395633
"Tmem52",131.874326137194,0.831180796261927,0.37036327773237,2.2442311
2720843,0.0248175342607346,0.0680484155555904,2.23382817422772
"Rpp14",1156.54862395716,0.138586827769831,0.23719624261804,0.58427075
5051544,0.55903816466245,0.687686419683884,0.0518900030015916
"Psg29",0.843793669448328,-0.443044065137224,2.33230729457126,-0.18995
9559003423,0.849340820700029,NA,NA
"Ssxb2",0.712598244232669,1.06770108835828,1.959386493454,0.5449160193
3835,0.585811278136668,NA,NA
"Slc38a1",707.753919795945,-0.95185479534377,0.276208024015514,-3.4461
5185868136,0.000568630719527494,0.00381133096230563,-5.30161877216838
"Gps2",2195.22374337169,0.168716471591965,0.225256282039457,0.74899785
2865261,0.453858499563073,0.594154224820787,0.087836554565531
"Bsg",21052.758821782,0.30384029690926,0.244812146615522,1.24111610109
952,0.214562862360301,0.349298379418981,0.319587964768978
"Cpn2",29.2058111653616,-2.66741854373216,1.16845376148975,-2.28286187
408157,0.0224385058974773,0.0629233407146572,-7.37764785707457
"Vwa5a",2628.00787269879,-0.57552107352725,0.142452554531824,-4.040089
52607921,5.34308018845648e-05,0.000608169162373172,-4.26176663451184
"P3h3",1624.94853369827,-0.663251396345978,0.25168662413261,-2.6352270
3533312,0.00840810229298967,0.0295709196444287,-2.33528418668789
"Grm2",0.283857027812607,-0.104382380122923,4.02357622999715,-0.025942
6873398635,0.97930305191893,NA,NA
"Il15ra",1033.69472385233,0.209676159476304,0.307498735105743,0.681876
494237285,0.495317049809393,0.631262947720998,0.0964579080871878
"Epb42",37.8828254354764,0.00829095382769162,0.486550104571413,0.01704
02878342712,0.986404475387189,0.991182975731778,7.34257170546242e-05
"Serping1",10013.3112968035,-1.46056602930048,0.316639913318344,-4.612
7034775684,3.97465131106988e-06,8.35105078267678e-05,-13.715500933718
"Serinc2",2222.28009822142,-0.347955808249129,0.216773615974537,-1.605
15755888854,0.108459134871898,0.209496057303787,-0.5438724511215
"Ccl25",441.432171895245,0.69720299969494,0.163433502804276,4.26597354
723465,1.99032363502998e-05,0.000288101931965958,5.68373565172039
"Scg5",292.694422122974,1.30918445045688,0.517423926707404,2.530196967
86695,0.0113998510522579,0.0375532188040405,4.29673837050626
"Kcnk5",4290.51767931795,1.50519621277811,0.529699130542059,2.84160597
21257,0.00448869311372947,0.018267697418388,6.02472989102365
"Guca2a",95.4153650011525,0.558843209951435,0.359120175090487,1.556145
40400194,0.119673520384711,0.22609529290143,0.830887368438964
"Parp3",3050.61669405854,-0.965304801684306,0.225371767549802,-4.28316
648610832,1.84252198298264e-05,0.000270931324466029,-7.92867113588477

"Slc26a6",549.984798682262,0.521007239150186,0.255153837737128,2.04193
377521115,0.0411581009049972,0.1000374783652,1.19946827379532
"Acy1",3413.21325639993,1.95145657641168,0.485984367303415,4.015471911
65785,5.93269645492383e-05,0.000661004088446314,14.2880782282493
"Frs3",713.762711403336,0.915879384266246,0.257553487690728,3.55607447
788103,0.000376437403158412,0.00275418185169263,5.39877453598908
"Gabrr2",38.5402494545739,1.40439523100971,0.377619198601404,3.7190779
3939289,0.000199951352727178,0.00169500673184033,8.96013786840639
"Creld2",1995.80658853183,-0.156470056256468,0.197265319818594,-0.7931
95967747188,0.427663656438461,0.570745104259898,-0.0877503745869442
"Cd4",515.358088730752,-1.74548618252857,0.518951438930661,-3.36348654
534089,0.000769645877996533,0.00480825226823979,-9.31639567877031
"Twf2",1518.45312848679,-0.729046683495878,0.190292761405941,-3.831184
52908801,0.000127527813255063,0.0012029691837674,-4.90135348361568
"Bmp15",35.8513954092242,1.02535724010779,0.313183677327648,3.27398046
046658,0.00106043943783238,0.00616287575524763,5.21826013172477
"Zfp605",187.0668299474,-0.19249399967742,0.198296523369196,-0.9707381
4712842,0.331678690497654,0.476465859229593,-0.142707199049506
"Ube2j2",2274.04865664776,0.196660386404127,0.101606572460741,1.935508
51722818,0.0529279245066809,0.121426137544981,0.414648419144525
"March5",3540.60250126843,-0.322314725092236,0.322536678808594,-0.9993
11849687368,0.317643646907799,0.462678606559709,-0.248415249462198
"Ache",330.262080421715,1.15469827976225,0.410264016872774,2.814524872
4563,0.00488493973148668,0.019529742645997,4.54468078628478
"Dtwd1",338.649957568444,0.196553635578813,0.133081729952576,1.4769392
8872773,0.139691912084734,0.253864563503416,0.269466065385853
"Gcm1",81.2781461862214,2.11772313717421,0.400195184658394,5.291725683
76079,1.21167544798032e-07,5.29714078132741e-06,25.7268278079895
"Wfdc1",264.752649735186,-1.30158931443703,0.248070638615886,-5.246849
53325903,1.54722160049616e-07,6.37221589562125e-06,-15.5716461337826
"Mx2",272.269918518646,-0.680410335287583,0.378756230218366,-1.7964333
8116261,0.0724256188967136,0.153980451122901,-1.27299985370874
"Poc1a",157.133754008622,0.149238300036424,0.306137944433117,0.4874871
04262009,0.625913179364593,0.742700533790825,0.0443927777655448
"Trip6",2977.34963759161,0.190157820502013,0.131165327568867,1.4497567
6138324,0.147126360977915,0.264004213726062,0.253250324627418
"Clec4n",229.001615587144,-1.3624752475112,0.540437892097608,-2.521057
95584208,0.0117002579533866,0.0383028256506266,-4.44470981855437
"Agap3",3224.61220588394,-0.0772683475875314,0.126942522477084,-0.6086
87664934972,0.542731484646579,0.673252225959982,-0.0305700813198927
"Tmem176a",33829.6336888987,1.15819573502945,0.456009111820587,2.53985
217621076,0.0110899330642221,0.0367702351085434,3.8255976366599
"Kcnk16",0.271161112340423,-0.542973133656971,4.78471990596049,-0.1134
80651810061,0.90964950160498,NA,NA
"Dlx2",0.39108001921673,0.88206772639745,3.8559629136816,0.22875420385
0801,0.81905996299954,NA,NA
"Slc17a9",143.000955018624,-0.804201725254697,0.388370964459347,-2.070
70507027793,0.0383863661228168,0.0949867275521609,-1.89310542302606
"Stk31",0.619917886113063,1.90256827595876,3.08566568664514,0.61658276
3386562,0.53750996651257,NA,NA

"Nfatc4",1013.77390179875,-1.21745735010945,0.251521373405191,-4.84037
333935899,1.29595433300262e-06,3.37512266435095e-05,-12.535542052678
"Cela3b",0.658791083092537,2.08829768018679,4.25703472826583,0.4905521
83265249,0.623743213170837,NA,NA
"Gnb3",7.43523872293619,-0.222315779175428,0.819491568753406,-0.271285
01091672,0.786171833915397,0.861748280733214,-0.0330788246825634
"Pisd",2129.89393732174,-0.320705097006644,0.164305761579618,-1.951879
79973082,0.0509524770927005,0.118089689321436,-0.685125782950835
"Tpi1",21076.1245754422,0.569134829059145,0.382790376112895,1.48680548
042643,0.13706619364813,0.250188788967538,0.788558780930427
"Rab12",2373.94636295802,-0.575500657509457,0.114903221692429,-5.00856
850689486,5.48363571169435e-07,1.69445212530296e-05,-6.32220045973782
"Tulp2",5.41505750262103,-3.52049925448493,1.24175833967881,-2.8350920
9641831,0.0045812476828413,0.0185591455059297,-14.0355003487498
"Celsr3",11.4462074567793,-2.27477070731319,0.798259218957386,-2.84966
418588224,0.00437654106350148,0.0179423460168528,-9.14592413589856
"Prph",57.0406823416997,-0.0165843730285179,0.635927995156288,-0.02607
9010760396,0.979194318355339,0.986683763270925,-0.000222325010810915
"Pcbp4",3034.53207367086,0.614920729431256,0.198904407149557,3.0915389
8721256,0.00199121831886011,0.00995762369354565,2.83442595372175
"Cdca3",251.954476825111,-0.0845054830217622,0.343183293749985,-0.2462
40083829156,0.805496388195746,0.876377912897755,-0.0111511638519637
"C1qtnf12",1887.47132564232,1.21283078103294,0.310949819648663,3.90040
67678936,9.60312028278716e-05,0.000960357102143514,8.42699733508735
"Ccndbp1",2134.75221837826,0.177013611247217,0.101071745811394,1.75136
592156561,0.0798828991752465,0.166129533107255,0.317737215092154
"Ogfod2",1697.38686832862,0.68131932806124,0.167563426467382,4.0660384
0960405,4.78190579995092e-05,0.000559985581305964,5.10144627528046
"Mrps23",2118.72310874709,-0.585485500606489,0.362230502992786,-1.6163
3406289406,0.106022105978542,0.205877461884435,-0.925344688606371
"Rhebl1",463.899946292964,0.565616404759229,0.226316484485459,2.499227
60175947,0.0124464344660312,0.0400264851094633,1.82027458505062
"Sfi1",2477.99859081928,0.0620289154824506,0.16730413628004,0.37075542
1005397,0.71081970745472,0.807944418794408,0.0132284112837173
"Hes7",60.3820231754476,-0.278944521092345,0.371867482352184,-0.750118
077891426,0.453183592556703,0.59370917742865,-0.145432099866163
"Pigx",974.779442728979,0.19467832216081,0.193262671905498,1.007325006
12433,0.313778616781126,0.458968902475499,0.151610186295346
"Pisd-
ps2",131.983526762756,0.037274196165584,0.18217922610208,0.20460179221
9154,0.837883255036838,0.896388779904753,0.0040770908626087
"Tiam2",257.432221977965,0.419835209130166,0.154841292534962,2.7113904
9704955,0.00670016675970585,0.0249122523800047,1.55019765082235
"Nox3",0.578006930376695,-2.36137682810029,2.91937182394188,-0.8088647
04637674,0.418592972861674,NA,NA
"Synj2",669.961328735068,-1.62071069250445,0.242510877308388,-6.683043
29476932,2.34030396176339e-11,8.00548186780045e-09,-30.2151351996173
"Rsph3b",5112.74361704564,0.694957847177841,0.23801855422036,2.9197633
3296455,0.00350297301242732,0.0152423494301066,2.90747956325787
"Rps6ka2",373.072065141293,-0.106328660975019,0.269027927750623,-0.395

232799300976,0.692671088683072,0.794780255819605,-0.0244225887040892
"Prkn",515.472004888798,0.970696307583292,0.319035454757143,3.04259696
879836,0.00234546235376629,0.0112918086354902,4.35228940296978
"Agpat4",755.186603651229,-0.739160841128647,0.292882769963069,-2.5237
4300209552,0.0116112779731319,0.0380944188104775,-2.4153466377148
"Slc22a3",296.081668373221,-2.83685733224341,1.01827734041795,-2.78593
77987131,0.00533731122906286,0.0209072208777343,-10.9720015675931
"Slc22a1",8313.3198129506,2.19010847453754,0.743444826268774,2.9458924
1481353,0.00322024384867461,0.0142571104817115,9.30905501001161
"Igf2r",3981.69183042379,-0.0632432854097582,0.241431067331338,-0.2619
51728536094,0.793358657994379,0.867334292883342,-0.00900146754969746
"Acat2",5373.20121448722,-1.22218037361336,0.62120274922262,-1.9674419
9078772,0.0491322773974178,0.114852073455803,-2.64493313060855
"Lnpep",4096.04659891291,-0.325254335229905,0.186721703249292,-1.74192
035296324,0.0815223820092496,0.168739214906194,-0.578757844782722
"Chd1",1738.73470368804,-0.136361026165479,0.152859465059693,-0.892067
927309765,0.372356524541215,0.517624947633296,-0.0897943273027817
"Mpc1",14114.2018933902,-0.395261331622555,0.604377254965077,-0.653997
694942035,0.51311329067743,0.647049731058646,-0.172069954776103
"Pde10a",174.380900873985,0.242471713249871,0.368226463306394,0.658485
300248818,0.510226339647537,0.644576465624444,0.106484318965264
"1700010I14Rik",83.5911323461036,-0.0865651309763425,0.214058637822521
,-0.404399149022498,0.685919225870462,0.789160770985108,-0.02049734301
00659
"Zfp54",302.684654084331,-0.057575644181065,0.194806245202796,-0.29555
3379826853,0.767571181237275,0.849837719005984,-0.00936812538074665
"Phf10",2143.25340254143,0.289760689258338,0.110923913536952,2.6122472
6047742,0.00899491725800609,0.0311736396545686,1.00494292969471
"Thbs2",1907.72095603343,-1.7096413249377,0.326091121151674,-5.2428331
0413256,1.58129561710906e-07,6.47584112469157e-06,-20.4258235687769
"Smoc2",4722.27320065085,-1.31055091066194,0.440475542148366,-2.975309
14944764,0.00292693413311228,0.0132765409462539,-5.66388209991892
"Zfp51",375.99062925321,-0.131958067211083,0.122336781437172,-1.078645
89586945,0.280745613170329,0.42460269667973,-0.11303546229395
"Zscan10",0.757876871647182,-1.30412941088521,2.01538643874897,-0.6470
8652683737,0.517575944646772,NA,NA
"Mmp25",80.5353637908778,-1.84511836887788,0.468544110440023,-3.937982
20437576,8.21696868783999e-05,0.000849690852292997,-13.0461640125625
"Hcfc1r1",2744.54457744727,0.0397531939051497,0.240139863817251,0.1655
41835800333,0.86851752383637,0.917552810997049,0.00342056917636296
"Tnfrsf12a",1640.09919944965,-0.449767374337159,0.281248377091444,-1.5
9918211435909,0.10978014326506,0.211562675184049,-0.698593972436278
"Cldn6",179.484401240412,-0.28540415704056,0.637191611007289,-0.447909
470417203,0.654218533287545,0.764516209891552,-0.0766344554506588
"Pkmyt1",529.725113921249,-0.734990528440505,0.25121607011589,-2.92573
053985536,0.00343648449583517,0.0149999047906412,-3.08674811969851
"Paqr4",709.102564759376,-0.711059087970713,0.24232101597843,-2.934368
21853704,0.00334227446128634,0.0146774025779642,-3.00169769026275
"Flywch2",229.264172264624,0.359161084782139,0.254002289454444,1.41400
727353111,0.157359766882847,0.27806785704928,0.459686718503425

"Slc25a27",478.995639928541,0.138061437844962,0.196050575264235,0.7042
13377894374,0.481299901074916,0.618818692460186,0.0662616141945931
"Pla2g7",1750.51682503746,-0.855544351585785,0.168653516403314,-5.0727
9284672523,3.92019181817123e-07,1.31559208383309e-05,-9.61515381379105
"Mep1a",32602.893938188,1.95865470204106,0.738956103071984,2.650569761
72001,0.0080356130479704,0.0285805149962289,6.96307639615369
"Tnfrsf21",6728.58305171556,1.52136372085683,0.291861528252634,5.21262
164960619,1.86190391506307e-07,7.26068050717993e-06,18.0023531989746
"Adgrf4",35.82111903725,0.399400315633623,0.290978188967919,1.37261255
577358,0.169872826489824,0.294412485406923,0.488376115200981
"Cenpq",194.387928856555,-0.0276054794232261,0.172550398212905,-0.1599
85022979573,0.872892872180263,0.920379906001015,-0.00229039220013445
"Mmut",6321.65092230512,0.317239758337883,0.430318774481922,0.73722035
1865477,0.460988358906484,0.600265388635653,0.161913908748821
"Tbc1d5",1007.26838632171,-0.32710968445442,0.113658251355831,-2.87801
088396419,0.00400191331669728,0.0167841053665226,-1.33700292029134
"Rhag",1.77650970587815,0.876601990663127,1.65852593052751,0.528542831
033288,0.597122627552822,0.71937360670923,0.288730286547443
"Satb1",459.039745622854,-1.01843649661956,0.274394404350543,-3.711578
95522713,0.000205970398622674,0.00173253271455776,-6.47539334133652
"Crisp2",1.67105676624272,0.168470667968794,1.44343835924021,0.1167148
33640332,0.90708603507378,0.943127331475567,0.00986462768282925
"Efhb",36.0414406624659,-0.709805729538151,0.45506871030249,-1.5597770
4787994,0.11881257719146,0.224801039537503,-1.05941311470914
"Cdc5l",2209.83519057798,0.0837267093241627,0.0719296244611718,1.16400
870922604,0.244420482334622,0.383589066690314,0.0802255462118352
"Spats1",30.5351185646817,0.99660429595711,0.286855805001756,3.4742343
6646509,0.000512313342061158,0.00352846539862539,5.62771705903939
"Aars2",1100.50122830551,0.150347794400087,0.182793889783462,0.8224990
15575356,0.410792946717931,0.554300406581745,0.0887124889180401
"Mrpl14",1644.61359077007,0.598647318768764,0.215407565789337,2.779137
84771249,0.00545033904579884,0.0212137895290915,2.30665029428915
"Sgo1",59.9487000953482,-1.22331178798938,0.490265939802956,-2.4952004
385233,0.0125885998065219,0.0403306800899711,-3.92761719296166
"Slc29a1",3241.50205181307,-0.206873280666101,0.213522295663273,-0.968
860324508415,0.33261488341497,0.477453897020871,-0.152938866572444
"Sult1c1",0.179410683008769,-0.0649340025024865,4.78471990596461,-0.01
35711188488881,0.98917214616915,NA,NA
"Hsp90ab1",69278.2675079032,0.181485556739923,0.123028916742412,1.4751
4553119168,0.140173421645936,0.254573526010847,0.248302292169439
"Slc5a7",1.65511322561672,-4.17091985762976,1.51945778046296,-2.745005
42973884,0.00605098965346705,0.0230029628121077,-15.7332613214434
"Nfkbie",1138.13046691651,-1.00201283936758,0.401480953421999,-2.49579
172019738,0.0125676369760444,0.0403165162485875,-3.21745727677281
"Tcte1",1.91373421936495,-1.44925292610843,1.55811578866795,-0.9301317
24900506,0.352302886639537,0.497335772060359,-1.01228850721798
"Vegfa",14599.2421859381,0.246223375261572,0.231004397961521,1.0658817
6430557,0.286477093353357,0.430599010654005,0.207462397025968
"Gtpbp2",5140.02128241173,-0.342290342877846,0.141007227856547,-2.4274
6664891586,0.015204684733322,0.0466353536149617,-1.04925557256648

"Polh",367.488535428499,-0.288285494958926,0.125848023914842,-2.290743
12008268,0.0219782751192396,0.0620072939191049,-0.801578757679153
"Clic5",4362.8613270481,-0.106646498389688,0.26875176859922,-0.3968215
68637662,0.691499044360999,0.793748588603183,-0.0246341154306979
"Enpp5",4409.0979771288,-0.225040825990402,0.306448293861587,-0.734351
701406587,0.462734415111108,0.601934460326666,-0.114232233181749
"Enpp4",1527.12825670191,-0.577138721146172,0.162186199289518,-3.55849
464180318,0.000372986398738046,0.00274019924739805,-3.40495953568956
"Cyp39a1",402.119557438468,-0.128736457598762,0.340306796315946,-0.378
295288229393,0.70521124526446,0.804213269037301,-0.0280504878019629
"Calcr",81.0953016457194,-0.299863670205795,0.505083737956188,-0.59369
1001454902,0.552718802744408,0.682514811701318,-0.114539240974968
"Fbxl17",1003.84667861663,-0.0940128260314656,0.260263707585054,-0.361
221420012017,0.717933930786349,0.813096873981891,-0.0194517256825972
"Rsph9",661.376462500433,1.14767601317349,0.313611655643758,3.65954514
929499,0.000252663325517012,0.00202483745208824,7.11819174591698
"Mrps18a",1750.78896045095,0.198713888845097,0.315062586588309,0.63071
2427638243,0.52822857171025,0.660683859850311,0.0823628986898109
"Crip3",56.3799543912734,1.17681806919411,0.400489882686873,2.93844643
789469,0.00329861649928091,0.0145249377829673,4.98016257082292
"Rrp36",1031.66967392484,-0.0277274814091281,0.156762423654853,-0.1768
75814769082,0.859605936097363,0.911029575198301,-0.00258364443591243
"Ptk7",1620.34982618745,-0.559560916077411,0.300052614706207,-1.864875
98725076,0.0621987890070295,0.13717577132645,-1.11156338016907
"Cnpy3",2414.16758498716,-0.015167269984046,0.101564171452082,-0.14933
6815997184,0.881287866009993,0.925956848531672,-0.00116678236519217
"Ubr2",3417.87531794932,0.18951789328153,0.182040461693598,1.041075657
12791,0.297840436323743,0.442157212383154,0.154663615079955
"Guca1b",132.80390474502,-0.305600260065025,0.168633374541708,-1.81221
695228213,0.0699526806854799,0.150015108667563,-0.579729580887553
"Taf8",1377.84224489764,0.0210938374574804,0.115181525881558,0.1831355
96581445,0.854691626030549,0.907618501682864,0.00204464973284769
"Guca1a",79.4254793993585,-1.05116143152195,0.406396493974317,-2.58654
158465348,0.00969444716724911,0.0330010304533149,-3.58573921111457
"Gm20517",31.5723202103923,0.0352918219899147,0.543361020895855,0.0649
509637841303,0.948213042993192,0.967971618444045,0.00114883745616768
"Pgc",0.37482137212789,-0.443418814973781,3.89783568332835,-0.11376026
3643322,0.909427839056949,NA,NA
"Bysl",819.143679883608,-0.252561881815716,0.212068471920902,-1.190944
97889303,0.233675185409202,0.371267826361524,-0.250246286609654
"Tfeb",1644.45609057872,0.70416051143322,0.208002503790867,3.385346323
24045,0.00071088502296967,0.00452229565346252,3.80157636917608
"Foxp4",2219.06023463064,-0.264648965726457,0.195111639508439,-1.35639
763159804,0.174972686433438,0.30122953181074,-0.317547726314597
"Trem2",702.256997221227,-1.00265752196787,0.385752571354078,-2.599224
46776782,0.00934346536115333,0.0321147375040432,-3.44757797240862
"Treml1",12.2931526562587,-0.301796756584673,0.50988997788585,-0.59188
6033602797,0.553926906791149,0.683530588482616,-0.114828798231276
"Nfya",2224.26274239661,-0.233420869910791,0.154078102774994,-1.514951
61029899,0.129784658942178,0.239838999152175,-0.333275381068033

"Tspo2",5.80210003113187,-0.457766258092179,1.31934750953221,-0.346964
127938123,0.728618268389885,0.821808237234105,-0.089835803795139
"Kif6",35.2999404169196,0.802197517786519,0.549949917910589,1.45867376
584815,0.144654919951757,0.260760463334725,1.07827624670462
"Brd4",5949.58089335145,0.00666989936290588,0.166836905197557,0.039978
5608286598,0.96811021860616,0.980477693900995,0.00013149962266573
"Stk38",5515.36416443161,-0.0442411295219978,0.0983745352740664,-0.449
721357247018,0.652911369620836,0.763594501103844,-0.01193264615721
"Ppil1",1364.56420095565,0.262126720802841,0.139188493186073,1.8832499
3541254,0.0596665046768294,0.133018237844594,0.528780116731172
"Cpne5",114.779426191048,-0.984320862993098,0.324299104338943,-3.03522
535160729,0.00240356023310957,0.0114877093334588,-4.39644705501092
"Pi16",1365.17154526437,-1.88868368621703,0.418619593279143,-4.5116944
2744554,6.43117942203264e-06,0.000120340821852968,-17.045715252749
"Mtch1",9459.88349642048,-0.422435710279555,0.229290348798276,-1.84236
149708684,0.0654222898340439,0.142461894927875,-0.823192323658768
"Fgd2",1527.87645134615,-0.796655998430518,0.422776528760624,-1.884343
01394623,0.0595185926723927,0.13276438850547,-1.60859125085939
"Pim1",2815.54765730668,-1.71703380126963,0.40979609153457,-4.18997115
087121,2.78989922834554e-05,0.000368796306130721,-13.5736090712476
"Ccdc167",811.902616197511,0.057367448063616,0.146707856748276,0.39103
1873378452,0.695773676265957,0.797323799710386,0.0129934062757659
"Cmtr1",2906.61345124976,-0.211137876382383,0.165925779816558,-1.27248
38576369,0.203201255931169,0.335366352475532,-0.230674834675621
"Glo1",4490.73339344629,0.486111430404668,0.232394420289898,2.09175172
879914,0.0364607304828636,0.0913124367411748,1.16349229031001
"Glp1r",74.8486710236289,0.0248547927320824,0.538505430039231,0.046155
1385475754,0.963186598593607,0.978046740493088,0.000551722169821557
"Tff2",5.14861123068763,0.753918584586009,0.811503132136712,0.92903964
8437238,0.35286853920135,0.497881957981613,0.525776987233747
"Tff3",0.405010826162729,0,4.78471990595309,0,1,NA,NA
"Abcg1",1102.8971114961,-1.11056771171238,0.198390329514921,-5.5978923
6918857,2.16973379963432e-08,1.4488174529202e-06,-14.9313195505812
"Tff1",1.17304717444168,-2.37840661156628,2.09069699947292,-1.13761420
816402,0.25528161114163,NA,NA
"Rsph1",80.2613098252229,0.252422616968382,0.232506358472732,1.0856589
84238,0.277629893322652,0.421164526570067,0.218277844290304
"Tmprss3",5.12531780704664,-1.36297365930587,0.88723143571265,-1.53620
983707716,0.124486924149411,0.232851692926441,-1.98633448611799
"Slc37a1",537.993763777035,-0.653215937484037,0.189596400684978,-3.445
29714237234,0.000570431949812539,0.00381946502659508,-3.63687439048608
"Wdr4",2047.58864534078,0.461499626076409,0.223417867210943,2.06563437
310356,0.0388630162598643,0.0958935827682656,1.08199335664471
"Ndufv3",6060.64914599656,0.929897259611098,0.318391355735494,2.920610
88613101,0.00349345852084672,0.0152043424641673,3.89271192008841
"Cbs",5204.85570531429,2.05823322390199,0.688206060091398,2.9907223188
7734,0.00278318452212716,0.012786647458161,8.97256686679114
"Cryaa",38.437767345585,2.50311296187239,0.98114353599643,2.5512199490
0601,0.0107346556572093,0.0358520582398538,8.33124679262774
"Sik1",5052.4256859187,-0.0710639167266969,0.234375034489108,-0.303206

000082741,0.761732876716665,0.845664505366508,-0.0119126265538225
"Arhgap28",706.341618563919,-0.7471895083138,0.269032820342163,-2.7773
1730784186,0.0054809642613276З,0.0212884145751726,-2.87637495610361
"Epb41l3",1056.07876851261,0.429560712161565,0.238682296905558,1.79971
752296122,0.0719052527804814,0.15310785396023,0.806119078451759
"Akap8",7565.31814945551,-0.0283356560703592,0.166156678963333,-0.1705
3576327565,0.864588811242826,0.914852681572238,-0.00252165323398204
"Myl12a",6699.95927649633,-0.614332015170048,0.205685458575231,-2.9867
5472454632,0.00281955837187175,0.012902076774174,-2.67256971989623
"Myom1",747.163399259446,-0.667912621756729,0.227168540825521,-2.94016
33665012,0.00328039239349742,0.0144551552586898,-2.82974818494469
"Wiz",2028.65262717506,-0.225080723870077,0.165887844545033,-1.3568246
9374045,0.174836919286206,0.301084231538661,-0.270179071361565
"Lpin2",3635.72890117533,0.433518522261295,0.223345538418143,1.9410216
3549678,0.0522556531546653,0.120250292129076,0.918270237754707
"Emilin2",149.294045721687,-1.70161855302238,0.39962094238235,-4.25808
15281555,2.061887573693e-05,0.000294652042427947,-13.8336745809488
"Smchd1",1601.78814559144,-0.226800754303795,0.113354906460932,-2.0008
0227124498,0.045413702570038,0.10793256411429,-0.504914873820352
"Cyp4f13",1908.69578138931,0.583462087884835,0.168096345049144,3.47099
806194034,0.00051852774198261,0.00355994856097779,3.28956460965234
"Ndc80",168.386625414542,-0.623807656326192,0.424600738584117,-1.46916
290915167,0.141788607942913,0.256671124924648,-0.848353262623375
"Clip4",1085.37196151774,0.627804141975461,0.218915025725328,2.8677983
1532974,0.00413338930141717,0.0171986715608152,2.5507199714139
"Lbh",6903.53536877006,-1.54525317885687,0.362067966952623,-4.26785388
351981,1.97362526760146e-05,0.00028589706885359,-12.6090785173164
"Galnt14",2124.70677400491,1.56063924452819,0.475989133090919,3.278728
72725876,0.00104275821845188,0.00607278964855879,7.96540469564859
"Ehd3",1530.4357444267,-0.0352706098960753,0.33382941226114,-0.1056545
90640098,0.915856410032486,0.948401052768082,-0.00186855983701822
"Xdh",5104.42779649682,-0.539821530865381,0.209014542666217,-2.5826984
2844113,0.00980309710048348,0.0332534424608954,-1.83733493466526
"Dpy30",1392.59198765322,0.0196456991891382,0.249006903554113,0.078896
202911374,0.937115183466507,0.960969382940462,0.000782148919495163
"Spast",1440.17532384522,-0.091625011847065,0.148815005478092,-0.61569
739928245,0.538094254099303,0.669345827082798,-0.03678326608499
"Slc30a6",825.070781949545,0.207706429246987,0.104547161039681,1.98672
472003474,0.0469529108665602,0.110914448276737,0.456745630161538
"Prkd3",2357.77918184355,0.172939138074177,0.106926227676618,1.6173687
3947527,0.105798709378461,0.2056283129447,0.273535248561288
"Yipf4",2758.32571821908,-0.0847152762848148,0.274639491693391,-0.3084
59922360296,0.757732391720366,0.842854396336032,-0.0144830131551069
"Birc6",4854.62679262707,0.475759649043675,0.157490729541165,3.0208739
9321699,0.0025204622614З016,0.0119079169307888,2.10787777503715
"Crim1",4419.10660332149,-0.124932149286992,0.184025160279384,-0.67888
624086686,0.497209944363633,0.632972022408077,-0.0571351019252312
"Vit",71.6470979192561,-1.82473584152694,0.456290186047048,-3.99906878
851608,6.35921968993797e-05,0.000696584637721407,-13.2645910348414
"Strn",1364.72031785704,-0.311572402534599,0.134680233512488,-2.313423

39115939,0.0206993722593466,0.058971721559066,-0.881967141654779
"Ttc27",575.556429129567,0.31409435337118,0.204914854478376,1.53280421
846785,0.125324100943508,0.234013533824605,0.456183203732856
"Eif2ak2",2076.410308987,-0.0389474772042008,0.210838888120368,-0.1847
26250225554,0.853443755800904,0.90668808100071,-0.00381516984117277
"Cebpz",2697.65498759366,0.388711629140353,0.198967526700381,1.9536435
698158,0.0507433841606677,0.11777103968155,0.831459181764505
"Ndufaf7",1645.05295700025,0.43524270358446,0.162599628430187,2.676775
51164475,0.00743344219523994,0.0269700885602509,1.5725435778767
"Pja2",2595.44849745382,-0.442877249466754,0.142329667490486,-3.111629
90313567,0.0018605759442271,0.00943989325020554,-2.06505273646997
"Qpct",173.815557991309,-1.15724768631607,0.338525433513308,-3.4184955
4494573,0.000629683486376924,0.00412130382311354,-6.35512484988663
"Man2a1",3993.68263474513,-0.297323927542709,0.183913878430898,-1.6166
4758570367,0.10595437392876,0.205828273673704,-0.469983821499383
"Cyp1b1",4208.7604031865,-1.11776922705652,0.36436861521079,-3.0676879
961516,0.00215721721680206,0.0105947979937346,-5.0829350126881
"Vapa",4921.20141948683,0.0918045013716612,0.202944775137254,0.4523619
85222693,0.651008228238935,0.762180763432374,0.0249314888135679
"Hnrnpll",2161.65443360456,-0.231516420463705,0.0750143801078296,-3.08
629385633677,0.00202668421565372,0.0100935603670029,-1.06401651114083
"Ralbp1",7480.59405429619,0.176705773519693,0.154685414616857,1.142355
75446708,0.253306174702171,0.393699784323868,0.164719225200372
"Srsf7",7415.76426469503,-0.0936642514614777,0.131070183937221,-0.7146
11429143488,0.474849180515917,0.612463406873013,-0.0459204056089105
"Twsg1",4082.54116245302,-0.397105923725154,0.205784961657304,-1.92971
303892681,0.0536424034699953,0.122640018396612,-0.833327533220098
"Ndufv2",9355.38549735407,0.645744077654544,0.329648543800297,1.958886
48622619,0.050126081990717,0.116643793609619,1.38746541215333
"Washc1",2186.34429953389,0.237643207634322,0.100105299166295,2.373932
34537514,0.0175997749519779,0.0521045265735901,0.702117677201648
"Washc2",3347.59039187542,-0.0311078068236627,0.111937056837682,-0.277
904455436698,0.781085703632663,0.858635003068708,-0.00474118303306208
"Themis3",5.74539138980163,0.253502065674125,0.610077610468159,0.41552
4289572918,0.677758137709541,0.78297292368848,0.0620210964073763
"Lhcgr",26.4121077672497,1.02823370575549,0.477589783853203,2.15296419
755817,0.0313214924018999,0.0812278594437383,2.58137762749129
"Nrxn1",16.845447150458,-1.25344083070126,1.11160050245814,-1.12760009
367525,0.259488847127057,0.400753547824425,-1.1461571185157
"Cacna1h",132.757141853882,-1.53495234520948,0.444524946699512,-3.4530
1733143691,0.000554353384814904,0.00374394953138933,-8.57672152090946
"Prss41",6.84617358627412,-0.221471210517983,0.758844376578955,-0.2918
53267090713,0.770398816011611,0.851399201643393,-0.0356289956842721
"Tedc2",334.267866776071,-0.331908276415002,0.177189193449299,-1.87318
57736571,0.0610427361304063,0.135328171582793,-0.663833994540146
"Lrpprc",3264.71251138097,0.613661224238376,0.226857603144139,2.705050
28587679,0.00682940503134315,0.0253047234043298,2.25628762318461
"Atp6v0c",19220.7281981235,0.697094011250299,0.232627428287765,2.99661
143305929,0.0027299844185836,0.0126105747911734,3.04854515670527
"Pdpk1",2140.17965272697,-0.268144427554521,0.121680092523423,-2.20368

362641493,0.0275466014535756,0.0737374567740001,-0.699118050661796
"Prss30",58.0464766668792,-0.978023052853545,0.484561693825326,-2.0183
6642333948,0.0435531135957945,0.104697153111922,-2.20708834162181
"Prepl",1372.57228451686,-0.117049841963847,0.132751843440353,-0.88171
9145515581,0.37792870495626,0.523058907526772,-0.0758554595640386
"Sbp",0.581795970106808,2.39837846758131,2.90609764665393,0.8252917689
61306,0.409205963732695,NA,NA
"Abca3",10093.6625336373,0.979118500356931,0.242846753268993,4.0318368
9786618,5.53425909914042e-05,0.000624100543175476,7.22511033282545
"Slc3a1",12563.2733391841,2.13542333429005,0.729526282390297,2.9271369
4603754,0.0034209820940238,0.0149523220957803,8.97494653109416
"Eci1",4196.60843170528,-0.125376466941841,0.470675095114606,-0.266375
825369118,0.789949781407654,0.864674161448741,-0.0182300561205901
"Six2",2.15897205684043,-1.27383143152478,1.66366735517354,-0.76567676
0780045,0.443868658046429,0.58512278375967,-0.682689022859857
"Srbd1",531.762877919521,-0.195783285288801,0.121550372194614,-1.61071
728332788,0.107241356904378,0.207686926591336,-0.307717189180107
"Dnase1l2",639.907265185214,-0.285742126690344,0.252566603711013,-1.13
135356176104,0.257906308802447,0.398878179505839,-0.26262536668574
"E4f1",1729.54957426572,0.352457757466975,0.252072877107409,1.39823753
15881,0.162041749723558,0.284080419357564,0.443567352599261
"Epas1",9826.4618183402,-0.923341075687071,0.262299398759023,-3.520179
91674984,0.000431254159074476,0.0030733163719423,-5.34152632139997
"Mlst8",962.179829632179,-0.304800904345487,0.175138807674985,-1.74033
903959837,0.0817995028262916,0.169160500457647,-0.541602954384595
"Rhoq",2956.34743949828,-0.605847296294925,0.274495909890213,-2.207126
86224446,0.0273051975771698,0.0732838152048225,-1.58333071270245
"Pigf",469.51036204006,0.666734333681422,0.228196549524117,2.921754667
50849,0.0034806559026033l,0.0151587734619074,2.79306734118632
"Cript",1883.75730488268,0.120453372752161,0.219748120751296,0.5481429
02612062,0.583593783851881,0.70756286968297,0.0416682896887315
"Mcfd2",6176.10936159053,0.419270611812732,0.243376369102642,1.7227252
3153597,0.0849382252715057,0.174161825583397,0.732788756485071
"Msh2",1015.22929905002,-0.155549645194888,0.11335516339281,-1.3722325
5244105,0.169991054711871,0.294412485406923,-0.190201981489809
"Gtf2a1l",0.926180460063555,-2.06580673984413,1.80151261275836,-1.1467
0678696004,0.251502824517549,NA,NA
"Meiob",23.2625675324127,-0.901761366337275,0.432661007379502,-2.08422
148277002,0.037140025516866,0.0926030968705695,-2.14568047757596
"Hagh",5645.16739000807,1.17075838421876,0.236248765424907,4.955616940
95238,7.21011415176616e-07,2.10768824184613e-05,12.6059462495512
"Spsb3",2048.84220031691,0.288171210178307,0.129913872914034,2.2181711
9076262,0.0265431622552525,0.071790065442131,0.759045612104095
"Mapk8ip3",5708.1358296753,-0.273057295469495,0.194931324438351,-1.400
78715545717,0.161277730743308,0.283005555564767,-0.344677152329667
"C3",14469.08716614,-2.39658901242072,0.498244741665753,-4.81006383410
759,1.50882102614262e-06,3.8058204874164e-05,-24.3886337178679
"Jpt2",1733.55006571495,-0.355397387921471,0.282966249778807,-1.255970
94423552,0.209126504798105,0.342708740170907,-0.380585945563936
"Tmem204",1051.18561114811,-0.749256697631685,0.147221859087035,-5.089

303329533,3.59381362241065e-07,1.23149697732448e-05,-8.47011842909362
"Ift140",935.36062168815,-0.0243112734901951,0.107638355582498,-0.2258
60692116967,0.821309770991881,0.885430604600226,-0.00295822477221844
"Telo2",1124.6050887478,0.0799883969290956,0.241899082848715,0.3306684
58875972,0.740894929430145,0.831231332874675,0.0147856267219901
"Prss28",0.137590427313787,-1.07702982564828,4.77989601809126,-0.22532
4948821452,0.821726492309683,NA,NA
"St6gal2",0.654848874639427,-2.37048139897661,3.12860635538653,-0.7576
79659793358,0.448642778600939,NA,NA
"Tpsab1",0.568238103477033,-0.999642690269772,3.04986915844695,-0.3277
65762508583,0.743088776088877,NA,NA
"Pot1b",1369.23783093224,0.0338746115399553,0.209226931115271,0.161903
68686951,0.871381699425165,0.919336326396839,0.00284896504539372
"Tekt4",2.63059645102005,1.00751523834741,1.4198395642034,0.7095979459
57134,0.477953493744263,0.615408916398709,0.489116737604105
"Sox8",213.637268603723,-1.33246775421038,0.514348529912193,-2.5905931
0315877,0.00958107010150007,0.0327165857861731,-4.55687065466552
"Nme4",256.327443650613,0.654836133044034,0.185648386983876,3.52729233
839726,0.000419832943686551,0.00301062991393909,3.80172054921599
"Tmem8",6688.66292836168,1.45158199627003,0.411401604620688,3.52838195
079084,0.000418108388655945,0.00300047013692073,8.43221991555742
"Mrpl28",2855.23690685852,0.606999884597203,0.246395345781628,2.463520
09073729,0.0137580184737638,0.0433296469393389,1.90532297996658
"Axin1",1672.57993477907,-0.328584369397603,0.107806643319978,-3.04790
464927417,0.0023044303512725,0.011141032231369,-1.47768346708137
"Pdia2",15.6924557963857,-0.312397620233236,0.708426207780857,-0.44097
4115302455,0.659231736347315,0.768252194884268,-0.0823596404401215
"Rgs11",1577.16759228621,0.402325458806901,0.241293990659858,1.6673662
6016535,0.0954415985516466,0.19058995171651,0.66690715597604
"Fam234a",3622.4789503822,0.286347999358293,0.16201419814539,1.7674253
4071814,0.0771570221730261,0.161625227581614,0.521862079811036
"Luc7l",4663.78265684312,0.204011301833082,0.161407005965951,1.2639556
7907454,0.206245935324677,0.339250486306313,0.220539593014429
"Dusp1",3620.87360614404,0.487916415011603,0.271266770497242,1.7986589
8840921,0.0720726400814155,0.153397264087702,0.91470872317301
"Bnip1",1084.61615553069,0.328059919486356,0.113190569513588,2.8982972
7773368,0.00375194774047738,0.0160042610028064,1.35649505278406
"Phf1",3718.00748018911,-0.00926571168163462,0.134060449484652,-0.0691
159228337169,0.944897346748685,0.966007976066987,-0.000320437849091496
"Cuta",2999.54560884515,0.412887969772047,0.182333297470437,2.26446828
69238,0.0235453281275624,0.0652668194243974,1.12688336736946
"Plin3",4166.0302156646,-0.293188748262159,0.162261417249982,-1.806891
33147697,0.070779224399146,0.151388033933145,-0.553513669861384
"Kdm4b",1250.64349783319,-0.181039264338571,0.102925454313934,-1.75893
578070962,0.078588416910155,0.164024507912032,-0.327271815406877
"Rpl36-
ps2",0.211547398484995,-0.574942108692901,4.78471990596542,-0.12016212
4427823,0.904354720653253,NA,NA
"Rfx2",245.034674550567,-0.55380131703767,0.138964903759678,-3.9851883
6090729,6.74266174074971e-05,0.000729974562027417,-3.99983049464712

"Uqcc2",2302.76547939162,0.288740594787938,0.162409636685805,1.7778538
3109089,0.0754278646249391,0.158942235432515,0.531055871907629
"Acsbg3",4.16909971484732,-4.01775530062096,1.30165823633174,-3.086643
78135351,0.0020243002086198,0.0100915434173577,-18.4658343474857
"Ip6k3",42.7963076575906,-0.83583203989297,0.38041146042552,-2.1971789
1505694,0.0280076708422922,0.074592755919002,-2.16957915741588
"Grm8",1.49624869954271,1.14244103328155,1.38245776948548,0.8263840375
44051,0.408586275444101,0.552354932996538,0.67811237761197
"Mllt1",1232.66893262868,-0.439972995259389,0.15718662982066,-2.799048
46717161,0.00512534436742287,0.0202459409392243,-1.71580710619256
"Nudt3",4381.61138942418,0.195693395405131,0.119894745786836,1.6322099
3648095,0.10263526826026,0.201249995263966,0.313737096947303
"Spdef",11.6864388292586,-1.12514671915866,0.685382005906448,-1.641634
45999812,0.100665781662849,0.198348693794854,-1.82018215886955
"Snrpc",1666.47308564066,-0.540496869867335,0.278288325281437,-1.94221
89893188,0.0521105952004838,0.120001462763557,-1.14598921615745
"Taf11",1263.67911296509,-0.112536101673953,0.152360726437788,-0.73861
6205797,0.460140080779859,0.599618143593097,-0.0575579681866496
"Anks1",2741.12459793827,-0.0238486490611118,0.179859376031863,-0.1325
96084715022,0.894512829023656,0.934784603939503,-0.00160833253430359
"Zfp523",2088.54272706126,0.441565639204341,0.187535087518061,2.354576
12251796,0.0185438451206204,0.0542162081514254,1.28706465021105
"Fkbp5",3067.46285345617,0.704303286000426,0.207242977161274,3.3984422
3262796,0.000677707735913569,0.00435300106064291,3.82921933257281
"Armc12",17.9077691688597,2.56603115238685,0.863299464159591,2.9723534
6356301,0.00295526243000982,0.0133910058387729,11.0677337264469
"Clps",0.463808857968945,2.97459882643398,4.6723496353792,0.6366387489
30561,0.524360179152226,NA,NA
"Pdzph1",0.379725329660286,-1.7679046889696,3.18294029729201,-0.555431
306856023,0.5785996837859,NA,NA
"Nudt12",1522.69776114232,0.676406934541332,0.428142617969341,1.579863
59253254,0.114138108461616,0.218033196706632,1.030240778972
"Cul2",1592.07440643433,0.0138172187935452,0.176042766784577,0.0784878
529570788,0.937439992350691,0.961150810898343,0.000547492801930919
"Bambi",525.117131057487,-0.811975294703848,0.272189447255154,-2.98312
555057541,0.00285320926242486,0.0130113472465128,-3.52554268219768
"Lyzl1",0.0756979249635498,-0.0551506907693369,4.78471990596623,-0.011
5264199061198,0.990803451155754,NA,NA
"Mtpap",1038.88284539441,0.00170812236975698,0.165012199513391,0.01035
1491433931,0.991740852303105,0.994546270544975,9.34113250172293e-06
"Map3k8",476.984537590945,-0.782777076022685,0.275457097690293,-2.8417
3863220904,0.00448682589874365,0.0182639105164309,-3.13332220839897
"Svil",2052.25096790466,-0.618989942228491,0.157274022225932,-3.935741
79300432,8.29401105113867e-05,0.000855191049577482,-4.3726598761686
"Zeb1",1178.26118934806,-1.09411617748325,0.267276195463778,-4.0935788
3736985,4.24765558659423e-05,0.000510609054423023,-8.29329812896346
"Epc1",2116.71695517399,0.205020394776371,0.0939572945475697,2.1820593
6817999,0.0291051514517345,0.0768337973129077,0.52610502187586
"Sos1",1600.96614293328,0.0675394550267839,0.131595382119365,0.5132357
52950067,0.607786419229303,0.728071293686213,0.0214340718682597

"Map4k3",3101.0472070013,-0.00296902293237652,0.108407452930546,-0.027
3876274381126,0.978150566422553,0.986106382522817,-4.15397102184256e-0
5
"Tmem178",1447.75381013544,1.91784798188937,0.547612842983934,3.502196
86492202,0.000461438532568346,0.00324337725595444,10.9914555305427
"Thumpd2",267.261375372844,-0.144841549483223,0.138283079923722,-1.047
42785280107,0.294902291223334,0.439199883461088,-0.119175721660065
"Pkdcc",2060.32501863766,-0.769085400746235,0.246975240725313,-3.11401
822501558,0.00184558089430124,0.00938579454279751,-3.59051976311365
"Cox7a2l",6439.77140784457,-0.197277991207661,0.282104116300252,-0.699
309155055688,0.48435884685094,0.621849528932616,-0.0937183163958487
"Thada",601.970116914628,0.000129184650974266,0.182146355815887,0.0007
09235440893723,0.999434112039178,0.999843721980895,2.01902990440805e-0
8
"Dync2li1",1111.67201645958,0.482636147550134,0.292244018047565,1.6514
8341024924,0.098639905508189,0.195237120860691,0.788405671942596
"Abcg8",3.62518958485635,2.22030411199644,0.939545261192257,2.36316886
871308,0.0181194110748443,0.0532628188055653,6.51107905318756
"Polr2d",801.639572766858,-0.0866614636392435,0.143757994264019,-0.602
828831070676,0.546622564821197,0.67654069058522,-0.0338640661739733
"Slc25a46",2489.67905345077,-0.0321657900321581,0.221594129141847,-0.1
45156327727293,0.884587446081074,0.927993437194059,-0.0024037693743195
6
"Sap130",1551.22439513824,-0.106170536053721,0.171066084851964,-0.6206
40474385079,0.5348362028869,0.66668177240051,-0.0430460422303887
"Syt4",11.8891428152821,-0.267993887097285,1.09265379633996,-0.2452688
01513324,0.806248306861545,0.876804589613832,-0.0352334587134482
"Adad2",0.616185992272172,3.19615839427203,3.40316310779669,0.93917284
9796588,0.34764200618187,NA,NA
"Celf4",257.306957699513,-0.610122592629642,0.336922775381829,-1.81086
776320835,0.0701613237503364,0.150352545066183,-1.1560434748055
"Tpgs2",637.456544177708,0.202442495222315,0.12336304278525,1.64103033
332866,0.100791118717865,0.198547709917249,0.327294016279251
"Slc39a6",865.923463165237,-0.777232305485928,0.181530814528098,-4.281
54474768594,1.85600390760864e-05,0.000272297698950739,-6.3800004571963
6
"Elp2",2932.27427940387,0.100635790767694,0.107686708385754,0.93452378
9205232,0.350033770557706,0.495006318604112,0.0707655535368869
"2700062C07Rik",424.761788868953,0.243439651842174,0.182101840465425,1
.33683246264825,0.181277330204763,0.309125886333083,0.285799778878971
"Zscan30",46.6035016255466,0.169140744795128,0.286779727544131,0.58979
3240420384,0.555329275041522,0.684727380155028,0.0640594362742868
"Zfp397",1717.9768231012,0.208384244510983,0.14788276585639,1.40911784
618194,0.158800324677414,0.279766626857931,0.265439746227183
"Mapre2",2455.56850481976,0.219352716101167,0.103597373590801,2.117357
88754248,0.0342294854196266,0.08704580959819,0.535510337781299
"Wac",3189.22120767126,-0.149565979081077,0.133306250607952,-1.1219727
3870483,0.261874031145785,0.403414700109072,-0.135774532096971
"Ccny",4246.00245198057,-0.305432626271967,0.261937853673813,-1.166049
96944167,0.243594242839223,0.382507896180975,-0.293522581042529

"Thoc1",2659.17052689869,0.384450156199471,0.255151429382279,1.5067529
0015119,0.131874006437678,0.242619303408365,0.544482038229845
"Rock1",7737.76475012633,0.412847013724308,0.211001526848439,1.9566067
596319,0.0503937196748511,0.117126802505692,0.885349656732257
"Cyp4f14",35.9866798478929,1.94110629451835,0.56254302621608l,3.450591
69531461,0.000559359114590112,0.00376601658020649,10.8347456910762
"Esco1",1223.36901469118,0.127656064949851,0.117433370240392,1.0870510
204087,0.277014262240433,0.420557820226112,0.110572275746204
"Mib1",3709.42057661485,-0.0824464273073003,0.10757084242859,-0.766438
427420807,0.443415475281241,0.584753579151138,-0.0442378468802755
"Cyp4f41-
ps",42.1741765039549,-0.173953902449968,0.314629876292983,-0.552884247
673867,0.580342674555373,0.704841252552677,-0.0608460613620674
"Zfp871",4385.03977466625,0.333347865149611,0.127048778284899,2.623778
59629708,0.0086960293250909,0.0303427308125666,1.16511689228158
"Adamts10",6029.53586129461,-0.487895284741657,0.284792218585218,-1.71
316227376369,0.0866826900365151,0.176700375361419,-0.845668787447906
"Myo1f",1259.53968208778,-1.32734876720826,0.422912458104779,-3.138589
89436391,0.00169762855043209,0.00885509937836407,-6.27406142815009
"Kifc5b",164.179336664207,0.0754992532836435,0.209302925808564,0.36071
7620128721,0.718310551370736,0.813426624683554,0.0155905620897148
"Dtna",418.911818215754,0.309298545076976,0.203202897673006,1.52211680
35443,0.127979816309568,0.237448896983915,0.444710329381792
"Cdh2",472.108151783135,-0.288556194432628,0.138887611291871,-2.077623
7113491,0.0377440305117972,0.0937754591947663,-0.682969830794517
"Ccdc178",13.9629292036984,-0.385748076377747,0.681176315320363,-0.566
296959688515,0.571191926853748,0.697652795512507,-0.138882317856838
"Tapbp",9715.63221862292,-0.748544052128659,0.205425399609844,-3.64387
292686463,0.000268566052184111,0.00212434113001452,-4.60675985522114
"Pfdn6",3481.07554902761,0.448772311588225,0.15124693703119,2.96714975
124209,0.00300574523195242,0.0135536587725736,1.93021402750432
"Wdr46",942.001951701864,0.162592112085775,0.163795921043854,0.9926505
55945433,0.320880278236534,0.466139447552968,0.124101753898858
"Mep1b",4642.86261610834,2.42652369042312,1.0278723338525,2.3607247811
8798,0.018239261021639,0.0535103515290921,7.10457056433833
"Rnf138",1322.81643642376,-0.106908380280386,0.103295955326142,-1.0349
7160119037,0.300682173930182,0.445290978831133,-0.0864918009708979
"Vps52",2576.1264015441,0.262982201473306,0.126873601295106,2.07278897
098233,0.0381919233020122,0.0946689703207009,0.619946088098236
"Ring1",3021.9516620723,0.130153279707195,0.147422345410549,0.88285991
7502585,0.377311962378793,0.522549565232802,0.0844740908957373
"Slc39a7",6022.98831580278,-0.286300693073976,0.119074079418487,-2.404
39140467985,0.0161994184324754,0.0489927502088422,-0.86350663802012
"Col11a2",236.251945833646,-1.0963655476087,0.259319219282685,-4.22786
07449205,2.35923726686215e-05,0.000326939646263527,-8.79913931765222
"Dsc2",288.71607112222,-0.90790951813415,0.310038830505572,-2.92837357
389604,0.00340740373626624,0.0148996541937024,-3.8190462810134
"H2-
Oa",286.178528922252,-1.1010378842654,0.509496716411795,-2.16103038311
929,0.0306929924982368,0.0800283455109149,-2.78053287333746

"Brd2",11433.5803162663,0.186035113835456,0.207830380817834,0.89512954
3156242,0.37071784288589,0.515830312736545,0.123151044475941
"Psmb8",3506.20542622914,-1.28474447687167,0.323004490184891,-3.977481
78713016,6.96489556255324e-05,0.000748929027993717,-9.24613427418577
"Tap2",4492.26972625603,-0.854358925673871,0.161117798783684,-5.302697
35636674,1.14104002142364e-07,5.02212152092958e-06,-10.4245955055899
"Btnl2",14.6419144983645,-1.88136632496314,0.671949031664885,-2.799864
62708591,0.00511240414255506,0.0202194897788058,-7.33941378414814
"Pfdn1",3448.5145539289,0.968136971632587,0.150541900070501,6.43101336
690443,1.26756012229805e-10,2.90763379583145e-08,16.8004111216742
"Psd2",85.5199541257155,-0.142767364935461,0.358059580296017,-0.398725
164168018,0.690095723913028,0.79243147378423,-0.0332147197171798
"Tmem173",1196.7063070061,-1.25551978603997,0.322141170727412,-3.89742
10691696,9.72224944640351e-05,0.00096765911029084,-8.71409916077534
"Dnajc18",1338.34346621947,0.236967480186241,0.154896436066165,1.52984
462525024,0.126055192724394,0.234979296662492,0.343190017646373
"Spata24",309.172805984818,0.885628998430292,0.211997866519339,4.17753
731662908,2.94682328957286e-05,0.000384843673811732,6.96341152233439
"Mzb1",201.432606346014,-0.159530099244666,0.668877775277463,-0.238504
111126266,0.811490127127588,0.879810136779931,-0.0204276933599805
"Slc23a1",5568.40645845088,2.29087390053864,0.675665608697337,3.390543
88894438,0.000697540846344611,0.00445073106418574,12.4043649267711
"Sil1",2091.34114554133,0.43416358551927,0.196879180317158,2.205228530
61387,0.0274380620894279,0.073546637422214,1.13309556054053
"Hspa9",22915.8173913711,0.00753948089168286,0.449965742702037,0.01675
56775464914,0.986631529121906,0.991307222479141,6.58255497609552e-05
"Etf1",4384.68885138088,-0.470729962450907,0.23157097839875,-2.0327675
1562685,0.0420760164950469,0.101824273255607,-1.07538578260736
"Cyp21a1",8.13008196766357,-1.96086185168874,1.07883076980702,-1.81758
057571856,0.0691282626918538,0.14874714669126,-3.73643680141478
"Gfra3",42.1030346313893,-1.43688743667641,0.372887624010007,-3.853406
07774596,0.000116485959282818,0.00112049493542002,-9.76219138205165
"Nelfe",1437.56935901315,-0.0266694633900171,0.136341045111678,-0.1956
08471155343,0.844916629349658,0.900720312010354,-0.0027885721357286
"Cdc23",1859.55390121725,-0.235691018565189,0.117894044792478,-1.99917
662490978,0.0455892467663261,0.108257110394572,-0.523999168309267
"C2",2407.35825135269,-0.336432629170724,0.169131828486907,-1.98917396
081227,0.0466820049839443,0.1103504044597,-0.741529301197603
"Epb41l4a",675.242059990432,0.0968047120217856,0.171318222917767,0.565
0578810186,0.57203439555038,0.698230039091105,0.0347728974341632
"Stard4",2171.89218571755,0.662210526057424,0.195509840826797,3.387095
62269083,0.000706367632869193,0.0044994302860776,3.57845621622932
"Tslp",40.9532903356078,-0.599370074340026,0.29410239003492,-2.0379639
698571,0.0415535362768916,0.100759961488227,-1.37556283748573
"Bin1",2345.40141847855,-0.401137018857208,0.176473511289888,-2.273072
12241202,0.0230218340650442,0.0641623385649274,-1.10165818593362
"Ercc3",1357.5597347494,0.229706651114226,0.146548835701201,1.56744098
317216,0.117011650782312,0.22212120902214,0.345507023599162
"Map3k2",2107.12662662488,0.0380654800229781,0.119778245870899,0.31779
9611659086,0.750636945295427,0.837870343449178,0.00673347553833901

"Iws1",1691.97050032086,-0.181495628569651,0.169283806538286,-1.072138
1582863,0.283657982008739,0.42765967164233,-0.154167388821625
"Proc",5562.85217424511,2.27455099719686,0.753214063776294,3.019793584
03537,0.00252947010824035,0.0119360135940151,10.0721555727825
"Csnk2b",3434.18919505178,0.537709962670166,0.122419462268135,4.392356
8418592,1.12128461688859e-05,0.000187181570719981,4.61539660228996
"Myo7b",1382.18113738107,1.39865414762261,0.57207091076235,2.444896465
29788,0.014489373007308,0.045132010626144,4.3332592492329
"Apom",1198.14542903076,2.3722427364603,0.725848569546463,3.2682336729
5821,NA,NA,NA
"Bag6",7530.35765070163,0.073610966204736,0.154158537036885,0.47750171
7515155,0.633004898118843,0.748110649989163,0.0213622251144925
"Prrc2a",11874.4012917217,0.0478110953270307,0.163402959612366,0.29259
6262885636,0.769830770606108,0.851060858721885,0.00771057368684232
"Lims2",2223.49469331159,-0.0328458121285842,0.302073726700652,-0.1087
34422180098,0.913413138761413,0.946721740355626,-0.001798310242439
"Aif1",1481.70132267898,-1.02290979037976,0.406826237659267,-2.5143653
3755842,0.0119246832938234,0.0388289036177301,-3.32301489551183
"Ltb",839.020974059203,-1.52997449351242,0.4446295408024,-3.4410095441
5074,0.000579548090002385,0.00385992634386817,-8.50223223942273
"Wdr33",2626.10840080353,0.127538563673373,0.160199863169346,0.7961215
51855218,0.425961388574808,0.568862681810383,0.0719465707188352
"Tnf",147.816412472659,-1.43655965373416,0.501303841299752,-2.86564661
066537,0.00416158520978241,0.0172790861201741,-5.82993028699451
"Lta",40.9764903507682,-1.7550364060663,0.643502906102948,-2.727316985
55147,0.00638516671124083,0.0239834291149632,-6.54697401574474
"Atp6v1g2",434.607365618455,-0.0223504393077219,0.197545695039934,-0.1
13140604269831,0.909919084094055,0.944807876326866,-0.0012689166361895
"Riok3",5766.765635509,-0.0272511659755619,0.131163790353853,-0.207764
398253846,0.835412932074226,0.89489514062099,-0.00302620734231663
"Pou5f1",0.400860425948983,-0.0557145947770885,3.07812356895465,-0.018
1001813374274,0.98555893328955,NA,NA
"Psors1c2",32.5385791930921,0.333955555519288,0.493826279881854,0.6762
6120586208,0.498874818701559,0.634416469355902,0.151966358145423
"Rmc1",1277.21404410854,0.194438584263196,0.219196193056562,0.88705274
2804806,0.375050505056042,0.520146151759208,0.127093895095623
"Aqp4",689.88386140965,1.60981904444432,1.23277661777493,1.30584813277
033,0.191604207895536,0.321478258802785,1.8268634796144
"Npc1",1351.67554481879,-0.380206323192206,0.160340181915639,-2.371247
92207263,0.0177281350095718,0.0523652744154873,-1.12142296419749
"Mrpl27",2149.80527731816,0.607498345297671,0.255551901215089,2.377201
43113457,0.017444559007857,0.0517786590858877,1.7986672506246
"Zfp521",283.726438222202,-1.05740599181284,0.238364433293705,-4.43608
963468948,9.16077158253784e-06,0.000159479218139574,-9.24553179226309
"Lama3",855.48883137172,0.347643668782404,0.29143678647858,1.192861316
45552,0.232923691368829,0.370224678756781,0.345434468968859
"Dhx16",3486.11798419996,0.422994360235331,0.204912085506215,2.0642723
8876793,0.0389918974086656,0.0961507544801014,0.990584249695132
"Impact",2626.8050163369,-0.297781372411069,0.189359165649567,-1.57257
437943168,0.115817404004529,0.220334446607504,-0.450426687201029

"Ttc39c",1099.47099243985,1.54462010398609,0.285206983543763,5.4157864
0464487,6.10199107225026e-08,3.14752968060147e-06,19.5686262688005
"Ndfip1",12258.6654693292,0.593499937154845,0.232120952266998,2.556856
37749827,0.0105622816827581,0.0353976225937466,1.98294894268068
"Atat1",1311.88790869768,-0.0962569186542427,0.189456337904565,-0.5080
69139934133,0.611404856414437,0.731046292412381,-0.0301552224865094
"Spry4",500.22436650197,-1.27715567522562,0.292164280407595,-4.3713614
5953187,1.23474186406733e-05,0.000200124662224313,-10.8769858040691
"Gnl1",2919.80045622166,-0.0757278811280189,0.115783118852494,-0.65404
9414789865,0.513079970043139,0.647049560565366,-0.0329667992425689
"Cabyr",10.8786645314428,1.32750001008264,0.580489282047029,2.28686394
587917,0.0222037656293951,0.0624446880487446,3.68178690274411
"Nr3c1",5290.45336219626,-0.167780783801781,0.126996504229531,-1.32114
489937879,0.186453054536759,0.315165833289948,-0.193729143028267
"Mrps18b",1402.51268588091,-0.0502792876093204,0.247958765888716,-0.20
2772777276549,0.839312636486436,0.897124821651609,-0.00545833314043571
"Gm8615",11.6950523591241,-0.802511853486652,0.58634649000613,-1.36866
488870474,0.171104050436712,0.295918642488471,-0.977195185557577
"Pcdh12",595.170735174073,-0.686371667099411,0.208346422262238,-3.2943
7702671707,0.000986400947193398,0.00581228336910755,-3.53329156123764
"Dele1",2246.48208153406,0.329688196325904,0.0780463097877479,4.224263
73806158,2.39723187423566e-05,0.000330794246877897,2.64212581654239
"Rpp21",1154.08279144091,0.554406227633377,0.177442360283383,3.1244299
6558412,0.00178149925094746,0.00915059862881284,2.60234733827165
"Arap3",2679.35511809144,-0.348897546809217,0.238075967775483,-1.46548
830639741,0.142787740428623,0.258167225786098,-0.472458825223767
"Hdac3",3059.55127925822,0.0321715765178755,0.13391588771852,0.2402371
89671605,0.810146384524389,0.878977384869427,0.00415000821940326
"Diaph1",4115.48107313367,-0.206234858480711,0.117710314885961,-1.7520
5425863072,0.0797644785145318,0.1659816224201,-0.370372687470558
"Trim26",3496.995209778,-0.247769766136999,0.176439559428301,-1.404275
58842145,0.160236800642591,0.281696613373838,-0.313905618365158
"H2-
M5",93.1508119730266,-0.858135944201826,0.34017525675984,-2.5226289306
7399,0.0116481241456062,0.038173290336522,-2.80234522741921
"Gabbr1",3444.5113702183,0.129905270620681,0.193729911162917,0.6705483
41455839,0.502508300838217,0.637386602247173,0.0585065924446451
"Myot",0.115788110020585,-0.542709664906681,4.78471990596623,-0.113425
587196851,0.909693154959224,NA,NA
"Dcp2",1302.78624491234,-0.280247512002003,0.216056370255505,-1.297103
67563144,0.194595517011925,0.325051409007336,-0.314934286767805
"Ik",4748.90583205243,-0.136401306913869,0.0973173031469557,-1.4016141
2722148,0.161030506051067,0.282684145762466,-0.17233283446582
"Pggt1b",3637.27911590256,0.318585554339102,0.153804951651106,2.071360
84319176,0.0383250872878612,0.0948904827858688,0.750279132248792
"Mal2",2951.02440908262,0.0550980687042909,0.275291063330097,0.2001447
77813669,0.841367354148903,0.898419223263988,0.00590202129147937
"Ap3s1",2515.73192687814,-0.930745756192812,0.383605761358621,-2.42630
807445743,0.0152533158409546,0.0467330534674629,-2.85115680854017
"Lvrn",20.8337332779251,-3.35823719167296,1.00901938543849,-3.32821870

435481,0.000874032217745148,0.00531230678977397,-17.5895367118081
"Ankhd1",4574.83944011555,0.881740173736338,0.388161103307352,2.27158302628319,0.0231117070431792,0.0643668138734334,2.41875181019423
"Slc4a9",5098.93293463495,1.20979351435118,0.520236945487522,2.32546635690678,0.0200470368470465,0.0575581099166121,3.45391237512994
"Hbegf",1433.501726928,0.944504631148437,0.412773974178592,2.28818842812939,0.0221265503130091,0.0623077861026284,2.62163211493188
"Yipf5",2545.80460055616,-0.008957438429916,0.13789888126163,-0.0649565706985065,0.94820857875002,0.967971618444045,-0.00029158712130374
"Rbm27",1703.92809829496,0.108324843066007,0.132186106355272,0.819487358034936,0.412508411789903,0.555961084750088,0.063592855081738
"Lars",2325.15858879204,0.122489108246625,0.134343133744621,0.911763071415841,0.361893454051073,0.50669241469825,0.083274359464763
"Pou4f3",0.0831863295579107,-0.0587437094118094,4.78471990596623,-0.0122773559510892,0.9902043333289,NA,NA
"Tcerg1",2468.94936729481,-0.136187479091321,0.171442086223711,-0.794364336616932,0.426983359696661,0.569993259438963,-0.076555168913649
"Ppp2r2b",141.820974770888,0.51899172425321,0.313577240599452,1.6550682162426,0.0979106612366191,0.194109005876116,0.85080150159793
"Dpysl3",3814.80692473434,-1.16653514156329,0.345729926330668,-3.37412255266435,0.000740513918897298,0.00466736301593826,-6.26098195363392
"Jakmip2",136.789959699147,0.276269470526152,0.280362341400728,0.985401495599847,0.324426895312098,0.469263768901727,0.20902279097402
"Spink1",11956.945094113,2.15544185105436,0.77386994682456,2.78527659576242,0.00534820799312772,0.0209396304116475,8.33317900983268
"Dtwd2",178.607318231755,0.143785976386777,0.150477945247838,0.955528573638887,0.339310462648056,0.483935001149279,0.10436053406658
"Hsd17b4",8193.0349091281,0.2754412078674,0.355909725565017,0.773907505421856,0.438985490860857,0.581042638028986,0.149545608304497
"Rab27b",898.127150228527,-1.68599853828413,0.426915522438062,-3.94925564817977,7.83945912260206e-05,0.000818275021266033,-11.9846037530301
"Mbd2",5298.7176873788,0.0530620713273838,0.185568117326014,0.28594390077344,0.774921088911536,0.854443956493554,0.00834689543004494
"Smad4",2092.71669526508,-0.172030425030266,0.0726331292031901,-2.36848428420333,0.0178611391957073,0.0526749891613051,-0.506391257870879
"Sec11c",1966.9273151765,0.0948546147945302,0.168026132817468,0.564522989394596,0.572398259006171,0.698586708444061,0.0340239683263427
"Grp",0.302304364586444,0.809465924717853,3.89255900728404,0.207952126917825,0.835266347324022,NA,NA
"Rax",5.52066862650852,1.04926793123989,0.776158564652006,1.35187315972005,0.176415895109296,0.302848839834571,1.25337307835102
"Cplx4",0.505995766060354,-0.392318702570167,3.69058326239829,-0.106302628792399,0.915342245935766,NA,NA
"Pmaip1",1414.43813033232,-0.572352587003631,0.374113812024977,-1.52988894985096,0.126044219015654,0.234979296662492,-0.828914137413898
"Gnal",103.4351300189,-0.503705812811668,0.313718091954355,-1.60560014143193,0.108361794383705,0.20935773552254,-0.787650238157713
"Impa2",860.158236531233,-0.687483410207199,0.448160416053951,-1.5340118974819,0.125026725868848,0.233637253305616,-0.999590955288963
"Cidea",4939.27529553803,-3.82032914662277,1.59398711217806,-2.3967126

9449764,0.0165428892472095,0.0497306898769799,-11.465316002304
"Afg3l2",4572.49861330806,0.27486748120804,0.249147926127058,1.1032300
5886818,0.269927270866317,0.412302540333054,0.243532005058926
"Srfbp1",660.087947552754,0.0942989878554268,0.143167853290737,0.65866
0346495031,0.510113901893859,0.644476178262584,0.0414271879461907
"Lox",1538.27830518953,-1.79714086984071,0.272126020988481,-6.60407580
029541,4.00004832781947e-11,1.19989141993575e-08,-32.777063312827
"Prelid3a",27.5488873382594,-0.662366997322614,0.370495381577383,-1.78
778746040663,0.0738103047269822,0.156446713943548,-1.22871715082697
"Spire1",1496.7365744847,-0.286395062633112,0.170501912939778,-1.67971
759199129,0.0930122753472795,0.186728660033372,-0.480599191379747
"Sncaip",344.46372928602,-0.565732060976659,0.213375670890072,-2.65134
28574906,0.00801724106103507,0.0285525565066663,-2.01174818840492
"Snx24",630.843679102125,0.200474852058771,0.221431535276124,0.9053581
81294183,0.365275662634951,0.510255399774773,0.134888280257061
"Psmg2",960.835636951765,0.225784985286065,0.121003227031451,1.8659418
4985974,0.062049501614096,0.136921817844233,0.448938489178643
"Ppic",3394.14415132259,0.326293697436007,0.218323245123743,1.49454400
630161,0.135033488857099,0.247195234203011,0.456020512192091
"Ptpn2",1259.74307834102,0.051606049694748,0.0872061435058272,0.591770
804442231,0.554004076040588,0.683582551236513,0.0196313465600697
"Cep192",837.34780304993,-0.351886307501464,0.197301907182531,-1.78349
166780189,0.0745063031275731,0.157459776542534,-0.650491834333676
"Ldlrad4",383.119179009781,0.0990776939078324,0.187862262641473,0.5273
95404030228,0.597919034455319,0.720088037912898,0.032535311422499
"Setbp1",902.990112838237,-0.0859489763115348,0.190331770811797,-0.451
574510891944,0.651575535890804,0.762707558311755,-0.0232819102658102
"Slc14a2",1610.66697176985,0.376573601074675,0.682617892113349,0.55166
0900520529,0.581180703009986,0.705595351636905,0.131316246519185
"Galr1",0.996819834238811,-2.88839358257266,3.28994840387559,-0.877944
948671567,0.379973594757762,NA,NA
"Me2",483.10127903984,-0.866617021326797,0.149565080957544,-5.79424699
78858,6.862839441616e-09,5.96319630519015e-07,-12.4207789434199
"Mapk4",1088.9771093922,0.62825754806059,0.423707564395946,1.482762171
01825,0.138137610014907,0.251673249866441,0.866758978087593
"Cxxc1",2824.21583532886,0.0919615734732702,0.202919209773229,0.453193
039614344,0.650409744034598,0.761890444336146,0.0250091804968522
"Mbd1",3284.03576855957,-0.48265774193367,0.0729064957448003,-6.620229
61058437,3.58641356736852e-11,1.10996653550081e-08,-8.84052855489278
"Smad2",1686.68920594232,-0.996124674013876,0.200513128641396,-4.96787
756873205,6.76896746055657e-07,2.01191048088311e-05,-10.7719335484264
"Sall3",227.330424018944,1.75518592353024,0.564621600923963,3.10860569
389836,0.00187972398726514,0.00952217674816722,8.16886663266814
"Atp9b",3158.86080385739,0.0091457243187055,0.121497301362926,0.075275
1231188765,0.939995814189235,0.96275875322066,0.000347102319649478
"Rbfa",1834.42021740574,0.358084920972031,0.134702291350749,2.65834320
545907,0.00785258803741,0.0280832284580741,1.27928800556055
"Gm16286",3517.71811608628,-0.306577051965792,0.0977850649579503,-3.13
52134612543,0.00171729102385382,0.00892035531976702,-1.44686571703283
"Pde6a",256.405391384562,2.66624947695604,0.822952829313389,3.23985699

056478,0.00119589662192178,0.00673771971628047,13.3313373235822
"Csnk1a1",9697.08516761217,-0.454122872879615,0.15041855341548,-3.0190
6156234102,0.00253559001918154,0.0119562114133015,-2.01017687319223
"Il17b",0.338084053487133,-0.990259771449806,4.7518312828918,-0.208395
398004781,0.834920249314603,NA,NA
"Pcyox1l",531.514480599446,0.270470826276614,0.193465109288914,1.39803
413272159,0.162102817580655,0.284157218123494,0.340313861063983
"Grpel2",2532.05692547619,0.675700142703905,0.178458917883223,3.786306
39879851,0.000152903121807705,0.00138023382824381,4.44982487997611
"Napg",1959.4314184575,0.0032467898024206,0.111496296529441,0.02912015
82786499,0.976768758633074,0.985263880786686,4.82011089726056e-05
"Txnl1",3888.27437914982,0.331812410606543,0.199699782839946,1.6615561
9143803,0.096601793385652,0.192178529479996,0.547268328410715
"Nars",5965.70223031385,0.182958573827519,0.0670296991687286,2.7295150
6714915,0.00634275505668209,0.0238626232667534,0.683431127073318
"Fech",5155.33614907587,0.251695466189228,0.238211129190221,1.05660666
252179,0.290691138555658,0.434354802633015,0.209887228658471
"Nedd4l",4763.01573134218,0.815105758875601,0.182652958580885,4.462592
69605339,8.0973880704635e-06,0.000144053715873994,7.20987477690226
"Lmnb1",922.467395048332,-0.831057506384554,0.259681894780793,-3.20029
052116275,0.00137289126341284,0.00751084002638148,-4.0650413061714
"C330018D20Rik",847.444717473444,0.112038541179129,0.262025073468645,0
.427587099570211,0.668951759763036,0.776058869034312,0.028404783971741
5
"Megf10",35.4544000256443,-2.03912299385131,0.483781686533182,-4.21496
524282229,2.49816659856558e-05,0.000341819314658468,-16.2747061053807
"Prrc1",2073.53484919772,-0.215975296954832,0.0861521710553419,-2.5069
0486738976,0.0121793486498021,0.0394015165047024,-0.698453521828643
"Slc12a2",2898.19960992144,-0.0372481692104729,0.177563183051272,-0.20
9774169230326,0.833843934460277,0.89375155694601,-0.00418399160153224
"Fbn2",623.823002031808,-1.9249757757815,0.5363757745279,-3.5888566695
1645,0.000332131370929827,0.00250699377208762,-11.5280464963616
"Slc27a6",11.5374091175083,-1.26880735763774,0.629236583592263,-2.0164
2337830109,0.0437557229306965,0.105080562347915,-2.85865845586238
"Isoc1",3806.58032560737,-0.26219519562908,0.25648537406105,-1.0222617
8232943,0.306657016337847,0.451124076094412,-0.208710748606247
"Dctn4",2709.04783795636,-0.196911352929392,0.104822086081639,-1.87852
923262785,0.0603088018946571,0.134066927299284,-0.395676851958466
"Rbm22",1683.9791602879,-0.168675331393205,0.108095888713467,-1.560423
18908462,0.118659908440879,0.224580750803753,-0.251919971614165
"Rps14",34991.212704646,-0.348193292945626,0.247471143020997,-1.407005
63586957,0.159425717592495,0.280624956361692,-0.442461813582959
"Cd74",55010.4924388605,-1.41988048114351,0.445087349098296,-3.1901164
6594776,0.00142215468432056,0.00772455574713343,-6.90537711001926
"Tcof1",1790.85364550833,-0.0685324324412044,0.22735692864624,-0.30143
1026752823,0.763085837932842,0.846396995439143,-0.0114289322405901
"Tmx3",2735.85105806077,-0.257297277821275,0.100721265347257,-2.554547
71079564,0.0106325856941658,0.0355843041305947,-0.858305287232086
"Camk2a",135.245644260647,-1.04132791690909,0.337629030823038,-3.08423
690454179,0.00204075019922711,0.010140302595446,-4.78098371891414

"Cdx1",0.71568303416572,-3.1772638867989,2.39815437071787,-1.324878800
79539,0.185211353120856,NA,NA
"Pdgfrb",5347.72225973286,-1.00679746917061,0.259492776604096,-3.87986
703270228,0.000104513583135141,0.00102556912127276,-6.92929121330751
"Csf1r",5143.31850446994,-0.334769618193315,0.348503084699003,-0.96059
2984370428,0.33675685755907,0.481243418989061,-0.244844495758754
"Hmgxb3",1405.68578610984,0.222292762596841,0.137929624837179,1.611638
99966559,0.107040518883179,0.207421589861282,0.349666935901036
"Gnaq",6092.47808250054,-0.00539403504745805,0.121606482541063,-0.0443
564761906228,0.964620254446543,0.978674457342007,-0.000116274992406211
"Psat1",1245.75524033824,-0.260117687103369,0.28264953923818,-0.920283
428745222,0.357424666442478,0.502564804665424,-0.178968949880604
"Tle4",807.372647435036,-0.159229685751688,0.197215523592793,-0.807389
209788893,0.419442281566041,0.562390703202769,-0.0916459941825351
"Cndp2",47687.701694794,1.82373553236559,0.49708625967056,3.6688512242
8099,0.000243642805347417,0.00196791525213916,11.3632958728603
"Timm21",1154.57530853374,0.183109411499096,0.185397462300508,0.987658
67249195,0.323319841007735,0.46818345785138,0.138960827079456
"Cyb5a",26197.097555522,1.24502453945853,0.34529340508049,3.6057003149
7796,0.000311312241328925,0.0023775817005215,7.52202915245468
"Cbln2",0.301006728816405,0.39711544115484,4.78471990595943,0.08299659
10147065,0.933854250239434,NA,NA
"Slc22a6",19141.0479622221,2.34729965609575,1.03001887451781,2.2788899
4480282,0.0226736082995397,0.0634093537900781,6.47419020985893
"Scgb1a1",14.5918192472001,-1.27230521204912,1.3135707878812,-0.968585
190678124,0.33275219523409,0.477498416487162,-0.940480932780565
"Asrgl1",596.129533574425,-0.351770671992149,0.215039657547682,-1.6358
4092350105,0.101872884685105,0.200112583678237,-0.565955093518972
"Anxa1",3847.34520218185,-0.779714470514514,0.231404946792483,-3.36948
05634978,0.000753100022750205,0.00472304414396381,-4.17560626183298
"Incenp",818.739419298073,-0.0163588887405501,0.412298776535796,-0.039
6772672429452,0.968350425514357,0.980571103436974,-0.00032096333473811
9
"Fth1",120497.179864328,1.00895550898347,0.276712878075935,3.646218116
04517,0.00026612810982564,0.0021191122726102,6.21532933222051
"Rab3il1",1650.22491993224,0.0133609986477565,0.233347435739809,0.0572
579621687143,0.9543397067138,0.971783768618428,0.000382417962693522
"Fads3",6876.36888846598,0.941699634783369,0.212269169790095,4.4363467
1824732,9.14984335326446e-06,0.000159431318768499,8.23412492394678
"Fads2",5158.18923996974,0.37944666971855,0.294337269372746,1.28915604
376971,0.197343843027396,0.328423544840233,0.42249535290556
"Tmem138",487.274335299878,0.0417998352327393,0.11573765244443,0.36116
0213205541,0.717979682884161,0.813101461164858,0.0086483599410985
"Tmem216",481.497609569244,-0.258580736528449,0.24100699158109,-1.0729
1798811341,0.283307914456317,0.427288976954136,-0.219869838489928
"Sdhaf2",1922.22970834103,-0.152084808833406,0.151705058041127,-1.0025
032177383,0.316100613254325,0.461371633463077,-0.117645418982777
"Cd5",158.202864972322,-2.04400860255478,0.593396074289232,-3.44459407
656697,0.000571917572851344,0.00382284841805125,-11.3785042553629
"Cd6",224.666240819044,-1.75268292758182,0.604155998689112,-2.90104365

658002,0.00371922058728231,0.0159029304848312,-7.25830146804442
"Ms4a7",1142.86147821648,-1.25054019795885,0.474033202069403,-2.638085
67100277,0.00833755156034461,0.0293810916905113,-4.41116043556347
"Ms4a1",103.530295624935,-1.79711882625983,0.773607464984198,-2.323037
0796597,0.0201771605771376,0.0578720619201279,-5.1209269971478
"Ms4a4c",282.33393852639,-1.57960076028382,0.59237707634993,-2.6665460
6220906,0.0076635103906725,0.0275586731090608,-5.67303816297861
"Ms4a6b",787.739842484068,-1.31139842937644,0.430384988196696,-3.04703
57130047,0.0023111024965592 4,0.0111679870123161,-5.89434762959556
"Ms4a4d",199.159774707028,-1.91420969402497,0.352239765163624,-5.43439
40785214,5.49829460422239e-08,2.9371437861131e-06,-24.3833427243725
"Ms4a6d",323.790773138258,-1.33654491846944,0.474024006706861,-2.81957
221482238,0.004808770935256,0.0192726445417516,-5.27810752960369
"Ms4a2",40.066856686677,-0.0599565107327263,0.550632164300933,-0.10888
6684469015,0.913292368098798,0.946646887097463,-0.0032873633587483
"Ms4a3",0.138584434245715,-0.110890727031842,4.78471990596623,-0.02317
60122245752,0.981509872939268,NA,NA
"Mrpl16",2000.43329275058,0.735594002383008,0.30614884059983,2.4027332
6184014,0.016273052422012,0.0491519109477181,2.21622670549968
"Osbp",3752.26892507659,0.0876845117074723,0.0854712169145907,1.025895
20627855,0.304940982477009,0.449651310318136,0.0700847275180818
"Fam111a",825.896969839362,-1.24883475975122,0.262896819565013,-4.7502
8477642878,2.03130385148944e-06,4.81871058438007e-05,-12.4139408814784
"Keg1",37412.1814698515,2.32160705917083,0.785886788838268,2.954124044
5112,0.00313557943146173,0.013967906729413,9.91556739946356
"Zfp91",8601.53246779548,0.0959537038379377,0.11509512612332,0.8336904
18264337,0.404455458918836,0.54829121448929,0.0576632554893972
"Lpxn",360.729231529286,-0.759354420315021,0.337870868120439,-2.247469
34987108,0.0246100472654088,0.0675840424761889,-2.04599193102646
"Gna14",645.659299222374,0.425899738903882,0.504966163200253,0.8434223
31913721,0.398992286604774,0.543264776830997,0.259866328557895
"Rfk",3832.5855272697,0.386105621625061,0.176313047531699,2.1898868349
8108,0.0285324449885603,0.0757730335585603,0.996157448814639
"Pcsk5",1519.47891069547,0.861059755155527,0.133769544436745,6.4368893
4414136,1.2194676793843e-10,2.83061676340895e-08,14.965378803703
"Ostf1",4561.27668713919,-0.0311050468974918,0.149973441924351,-0.2074
03700937808,0.835694592510933,0.895098503888056,-0.00344710815859244
"Carnmt1",1402.61909757601,-0.295863291867869,0.266469200048863,-1.110
30952850692,0.266865668512047,0.408875279321695,-0.264603888303699
"Trpm6",997.164146240536,0.460200279895305,0.494036636379581,0.9315104
30618594,0.351589591589315,0.496616477601309,0.322111307142351
"Ms4a20",1.16929112124443,1.87160326487979,1.40360079774885,1.33342989
536736,0.182390767197896,NA,NA
"Ms4a8a",17.3394593238205,-2.0425140903602,0.576537934092469,-3.542722
81072941,0.000396018721434552,0.00287367809100517,-11.9531243753434
"Ms4a10",0.548246749768225,-0.550585764698889,3.66699172664504,-0.1501
46443118001,0.880649078962485,NA,NA
"Ccdc86",552.06864169485,-0.127155376873895,0.135824797298308,-0.93617
2034879814,0.349184619114573,0.494150217992158,-0.0896338244242201
"Zp1",1.44313813880636,-3.12247700832463,2.11947390460515,-1.473232107

99632,0.140688460145438,0.255223631923683,-4.26410184632262
"Prpf19",4142.21894866564,-0.0788566251889528,0.117366119269216,-0.671
88576805603,0.501656421378164,0.636844645096129,-0.035582438584126
"Tmem132a",2438.12369684201,-0.538406155854007,0.173424897502286,-3.10
454936752613,0.00190569122378469,0.00961624417219306,-2.50052052221078
"Slc15a3",795.829354306067,-1.63928042891814,0.407963691448066,-4.0182
0177452439,5.86439704850715e-05,0.000655266553878467,-12.0166934691908
"Pga5",20.8879318618647,0.69169269916161,0.707510126820386,0.977643531
789627,0.328250659157079,0.472900203357819,0.517988533666985
"Ddb1",10907.5377378447,-0.310944090924327,0.151043820512927,-2.058634
97009276,0.0395292201232198,0.0970336600864708,-0.725338456733309
"Fen1",463.068430258555,-0.144123291272768,0.272347045801387,-0.529189
84617102,0.596673759702795,0.719227062271019,-0.047499890337809
"Syt7",470.84471389461,0.0826510416660982,0.256274111457502,0.32251030
4283326,0.74706613276249,0.83551080971682,0.014853383422089
"Aldh1a7",1634.90463334404,-2.39874524901683,0.755474037137499,-3.1751
5246202994,0.00149757793652279,0.00803295037312831,-11.5720349852847
"Tmc1",7.34596109294006,0.587898407665574,0.844172269031816,0.69641994
7956638,0.486165874128693,0.62312068144054,0.278084805735612
"Zfand5",5281.84681699584,-0.379906061155319,0.120172619905617,-3.1613
362632328,0.00157047084571876,0.00833679296210848,-1.81863944347114
"Cemip2",1361.75246931968,0.192309668179882,0.204504215622825,0.940370
190385545,0.347027707301263,0.491776018385059,0.136488308749383
"Slc22a19",2653.75233470052,2.33892266353106,1.04775847206361,2.232310
90551284,0.0255944216075707,0.0697470345918118,6.22827125701407
"Rtn3",13188.4736297595,0.303100147962019,0.205673514399517,1.47369557
4498,0.140563574944608,0.255068272151695,0.414102707378978
"Atl3",4755.11674465634,-0.715535259330907,0.262422558128306,-2.726653
0150242,0.00639802801290248,0.0240131664467608,-2.66834045687378
"Gm16437",0.745595946683453,1.03664705833185,2.7157935788648,0.3817105
4913723,0.702676074460471,NA,NA
"Naa40",2148.22776679198,0.211455810953761,0.13222934293544,1.59915950
771231,0.109785165017551,0.211562675184049,0.328440352496946
"Lipo3",1710.880262891,0.607368229334161,0.241966784173062,2.510130600
81731,0.0120686517367483,0.0391212920304435,1.96853412687375
"Otub1",3987.41623842608,0.478141987688732,0.100421354412568,4.7613576
8617845,1.92294879317545e-06,4.61176575268572e-05,4.77392005470067
"Cdc42bpg",3792.82064004301,0.235765486757422,0.19169598249681,1.22989
268573401,0.218737293566569,0.354230876242605,0.244678927674601
"Lipn",0.589958286556142,0.0263515544155277,4.78471990594527,0.0055074
3929290081,0.995605721433107,NA,NA
"Lipk",5.71400726952306,-3.12930659918581,1.17436055741947,-2.66468980
026213,0.00770593675990767,0.0276805829021989,-11.2248981558028
"Ehd1",5424.4881650036,0.385486344551312,0.210258656439767,1.833391076
86985,0.0667444336451075,0.144678484403814,0.745238140167406
"Atg2a",2206.35144462649,0.136428097560535,0.163710205279868,0.8333512
09396551,0.404646683053345,0.548512307158934,0.0819312884773125
"Ankrd22",41.2258971451908,-2.64641824673956,1.04731090252913,-2.52686
975791887,0.0115084172419134,0.0378272284866535,-8.66629491823351
"Stambpl1",273.09024194437,-0.834617535976683,0.164090993702938,-5.086

30923088707,3.65098597611133e-07,1.24452665318564e-05,-9.4263125078922
1
"Ppp2r5b",2125.92803071129,-0.492147854145774,0.23050176117697,-2.1351
1537453253,0.0327515870922154,0.0841025214176236,-1.21841966106208
"Fas",687.771738739634,-0.477279284383026,0.164974191853994,-2.8930542
3484316,0.00381515412874927,0.0162029786979641,-1.96761257415966
"Cdc37l1",3769.37019472556,0.508606890223103,0.130600958765549,3.89435
801260954,9.8459143219954e-05,0.000977970644168448,3.52466146752214
"Lipa",6380.10577281781,0.800999976330587,0.182487535119151,4.38934076
131606,1.13694795135369e-05,0.000188826330114942,6.86832076279351
"Ak3",9536.8427481102,0.606428699927373,0.254396969132062,2.3837890128
8153,0.0171354234322078,0.0510982930548488,1.80352149230383
"Gpha2",2.50889225468344,-1.69267562384531,1.40656044636849,-1.2034147
7553668,0.228815852937525,0.365393243290407,-1.70415387133671
"Rcl1",530.778142908229,-0.0231687649445051,0.26554583051142,-0.087249
5903998338,0.930473122175427,0.956679177759194,-0.00102607929093058
"Majin",15.1295372189431,-0.495449922604259,0.570931997511545,-0.86779
1479131875,0.385508495530743,0.529967576600244,-0.314580701625097
"Snx15",1207.9414286994,-0.0433057962188099,0.095025207175980 7,-0.4557
29563826262,0.648584459981155,0.760438953740984,-0.0118597011757107
"Jak2",2147.56681356902,-0.343441779339622,0.132578401110603,-2.590480
62476713,0.00958420165484397,0.0327176979288041,-1.17451563463359
"Sac3d1",478.59120349957,0.352485869505404,0.148468245315641,2.3741498
9822252,0.0175894080852386,0.0520817431808629,1.04157488090045
"Cdca5",88.1756847296495,-0.787951164163158,0.422238959845666,-1.86612
614916247,0.0620237182807592,0.136880414152602,-1.56695721023691
"Zfpl1",1209.84243688484,-0.0159814857691319,0.0688336408234778,-0.232
175511536809,0.816401697073932,0.882692059184005,-0.00199415194374262
"Tnfrsf25",471.005113541442,-0.130618467170302,0.23361070906358,-0.559
12876466101,0.576073844900809,0.701661107332962,-0.0462787426516091
"Kif20b",719.817065247845,1.89270017980597,0.489412032951422,3.8672939
2081342,0.000110049758368645,0.00106876228693663,12.9484427539353
"Vps51",2181.59444336065,0.318594583086838,0.155988206153456,2.0424273
7924311,0.0411091561816885,0.100008095118064,0.733565348160595
"Htr7",24.3771315670812,-2.14045370055208,0.492967502662178,-4.3419772
8854937,1.41206132735151e-05,0.000222561435715121,-18.0018735519412
"Tm7sf2",894.797647205935,-0.0853230001154738,0.228544022378429,-0.373
332888900476,0.708900701225223,0.806991234965518,-0.018296875065631
"Rpp30",793.251846339346,0.202834201380816,0.194599339851662,1.0423170
0649874,0.297264730217544,0.441546881315835,0.165811093284346
"Ankrd1",1514.47146419905,-0.607500933753747,0.6273252608093,-0.968398
646931612,0.332845314967973,0.477543631438229,-0.449003787348493
"Pcgf5",1280.27761315095,0.309209032707311,0.13030044451945,2.37304664
498789,0.0176420358109122,0.0522058604099507,0.912958383019055
"Mlana",1.70349814178322,-2.30240361137926,1.60774192090398,-1.4320728
8523315,0.152122963464298,0.271024629169123,-3.00589285749645
"Syvn1",3324.53865976115,0.0818991753521809,0.144232921951108,0.567825
807341983,0.57015325424634,0.697024775929221,0.02956022335484
"Il33",1387.26244447646,-1.61582372045751,0.278811900149545,-5.7953900
8051966,6.81625605418342e-09,5.96319630519015e-07,-23.1587757330338

"Tnks2",6015.18202168987,-0.165549773148572,0.128351843028361,-1.28981
220092017,0.19711587068251,0.328212232840955,-0.184438146124351
"Tjp2",2404.44616359902,-0.358637764237678,0.142133118434786,-2.523252
62533538,0.0116274836578163,0.0381157828446709,-1.17171506349022
"Trpd52l3",0.12787631826076,-0.0652521028551441,4.7847199059608,-0.013
6376013931041,0.989119105682124,NA,NA
"Frmd8",1984.85455750907,-0.134710851075185,0.120023548556607,-1.12237
017398008,0.261705080547963,0.403277737435626,-0.122334935500557
"Uhrf2",2559.55365368075,0.0510424352228493,0.132123870870313,0.386322
584152491,0.699257776603206,0.799608710821026,0.0114147618114994
"Slc25a45",2156.36250574013,0.418597874934477,0.214640886283352,1.9502
2431272426,0.0511493891881387,0.118417334408731,0.893095377803999
"Rad9a",765.146947617301,-0.435530589884616,0.186001124472342,-2.34154
815526063,0.0192039484052693,0.0557034492719339,-1.25768743693212
"Dpf2",2744.15198612765,-0.104768080332259,0.0841454027158417,-1.24508
383049826,0.213100937106871,0.34749870968552,-0.110739266574385
"Gldc",1848.09783138014,1.13734636942958,0.501775132626711,2.266645545
93561,0.0234118873956562,0.0650097580447032,3.10861545154578
"Mrpl21",1251.755885598,0.396614337729649,0.180592075510132,2.19618904
43382,0.028078415208068,0.0747505379201133,1.0286584983524
"Rps6kb2",1891.27652693864,0.47979425654568,0.217965469081018,2.201239
75861213,0.0277190550304142,0.0740364568469885,1.24899927968415
"Ighmbp2",472.999926843531,-0.184047786770047,0.158311808885474,-1.162
56511795144,0.245005990240031,0.384198713020759,-0.176059262232116
"Pola2",789.126505555791,-0.621645558798332,0.21236740414602,-2.927217
3914736,0.00342009729548309,0.0149518065173384,-2.61272850964944
"Coro1b",7011.85634892797,-0.310332872964372,0.134115873250892,-2.3139
1605961383,0.0206723270108401,0.0589196070833738,-0.878732777257214
"Dmrt1",0.109801196260762,1.56396771900948,4.78466789306688,0.32687069
4886831,0.743765686733259,NA,NA
"Eif1ad",1522.91657288289,0.134455366602227,0.142308213883191,0.944818
032166367,0.344751784239417,0.489278244552788,0.0961119154553227
"Cabp4",98.9688834194337,-1.71639843403554,0.365726399177362,-4.693121
51897233,2.69067551174758e-06,6.05579488705372e-05,-16.6695068044579
"Chka",5885.32977485319,0.797592734261117,0.300909603508476,2.65060577
981404,0.00803475627104691,0.0285805149962289,2.8354661676149
"Banf1",2889.23177679964,0.0695709167138155,0.164664862612522,0.422500
074454409,0.672660046379735,0.778687386312288,0.0174028597420796
"Tmem134",3092.5467483286,-0.207830246679338,0.247321730983102,-0.8403
23435604362,0.400727064816676,0.544827857875709,-0.126212272683011
"Cst6",27.9201756459806,-2.22381562861957,0.940276956201664,-2.3650644
7802665,0.0180269321189194,0.0530389501214262,-6.53074324098861
"Aip",2742.21553123732,-0.373343974099313,0.182808939768091,-2.0422632
2067691,0.0411254283196311,0.100010195258576,-0.859618207876893
"Pitpnm1",2417.13288486579,-0.605611158890587,0.206940386143355,-2.926
50057428162,0.0034279887297582,0.0149695239088075,-2.54461995494042
"Sf3b2",8994.24291039025,0.173483359477732,0.0918752054522839,1.888250
03028517,0.0589923921997959,0.131831938465515,0.351516729658609
"Pold4",1211.85524908731,-0.387594773277378,0.161433856793866,-2.40095
095895711,0.0163525278648269,0.0493257407655315,-1.16639252063499

"Pacs1",991.533560675968,-0.728308538318793,0.185332767557351,-3.92973
432554725,8.50397688382894e-05,0.000872227992008926,-5.13054015427437
"Cdk2ap2",2410.72490160206,0.0376128105757602,0.158142081994249,0.2378
41883080356,0.812003732605836,0.880216199385589,0.0047989327653879
"Cabp2",18.4020469783728,2.15516132344238,0.97101053708436,2.219503538
97668,0.0264524867234062,0.0716357819245265,5.68135132935308
"Grk2",4888.33212715686,-0.307419286748615,0.181312523182667,-1.695521
5301863,0.0899765294793915,0.181950048930634,-0.523849561701442
"Klc2",1951.28698387712,0.0842468315342332,0.146574389231662,0.5747718
40946106,0.565445613345867,0.692939272146326,0.0309028257713907
"Mbl2",8.89456876473492,2.01502608833472,0.808657354044203,2.491816933
65838,0.0127091523551254,0.0406101364503827,6.45561479837794
"Acy3",46717.3290430332,2.09827559970676,0.785214384050232,2.672232758
75767,0.00753483642902249,0.0272494631535133,7.55950252965632
"Pip5k1b",178.380309225379,0.145441429796256,0.362130530467905,0.40162
7086256254,0.687958492640306,0.790675784821732,0.034159432341078
"Dkk1",5.80966381078975,0.0915489986715877,0.751742263620465,0.1217824
28768444,0.903071330171479,0.940747323571544,0.0055918764202819
"Gm49405",466.888035046612,0.283734839451816,0.195480131453612,1.45147
661474305,0.146647189413251,0.263386781427743,0.378539636709371
"Rab1b",7398.93867059951,0.235232691684721,0.229957957532616,1.0229378
1962886,0.306337246422404,0.450760277440248,0.187438023153852
"Doc2g",891.333667482986,0.612262934512458,0.325893804389612,1.8787191
6024671,0.0602828501909311,0.134035265255713,1.23043563910264
"Cnih2",48.862316903706,-0.757225986319681,0.332749486559585,-2.275663
87599545,0.0228661350267495,0.0638104910192588,-2.08376299091253
"Yif1a",1725.52412276183,0.190070295630878,0.290092701787087,0.6552053
68697554,0.512335538214925,0.646570765314861,0.0828844532539797
"Cbwd1",443.251527145471,-0.0127150006539005,0.189461136425499,-0.0671
113923086827,0.946493024593085,0.967127187585996,-0.000425002255747269
"Rin1",292.689831455344,-1.00590193407961,0.261683999322383,-3.8439565
9147805,0.000121066429608336,0.00115598222147837,-6.8027185168376
"Aldh3b1",712.763399600796,0.243074587571519,0.175528961201839,1.38481
186185572,0.166110002910053,0.289050677085249,0.301692815087515
"Asah2",2011.99461548033,0.729599497254215,0.198668988564824,3.6724377
6960264,0.000240247666921633,0.00194823172382901,4.55330871124904
"Rce1",1264.67636957633,0.175222263705852,0.163137805363579,1.07407515
575768,0.282788998558609,0.426700192996112,0.149232182524195
"Slc29a2",248.571691809392,0.683841948674246,0.286208966156302,2.38931
0013093,0.0168800518568826,0.0504641599364454,2.04228843983603
"Pcx",22620.0554485848,-1.16968124687477,0.815857733913185,-1.43368285
701541,0.151662786465444,0.270427161454341,-1.52965356391685
"Minpp1",2046.93320332214,0.425453333151488,0.246549761009356,1.725628
65771889,0.0844142402845298,0.173271803039032,0.745774510535599
"Apba1",345.287430603607,-1.11851483531981,0.228681770554632,-4.891141
22479911,1.00252998039983e-06,2.78451988003361e-05,-11.7319343320255
"Papss2",6764.87344400821,1.74828746352423,0.39843572952065,4.38787822
976509,1.14461826847936e-05,0.000189494617453548,14.9848339842147
"Cpt1a",8835.57995076206,1.22356674839865,0.255069568534502,4.79699226
931944,1.61065815124663e-06,3.98247320908244e-05,12.3959821718355

"Peli3",169.322481796937,-0.362413529355047,0.215242949327817,-1.68374
169972503,0.0922315986811843,0.185528908602675,-0.61050174208269
"Mrpl11",2021.1060501327,0.280061729572326,0.226658317966748,1.2356119
646728,0.216602835149289,0.351884859168541,0.292510826757881
"Lao1",0.573124667653522,-1.23511863290739,3.65736929734738,-0.3377068
41308915,0.735584118735739,NA,NA
"Tesmin",27.1326667796784,0.561840069469683,0.402276218931758,1.396652
45676626,0.162518107585533,0.284663558116959,0.705922434881425
"Mus81",980.489461986248,0.120397734918306,0.171169206892828,0.7033843
12539863,0.48181628018905,0.619319258430094,0.0576866934026283
"Gal",1.81869402776987,-0.824966797555859,1.72330785797939,-0.47871121
4444962,0.632144086664816,0.747477804124188,-0.240107137207405
"Ppp6r3",5327.09034195865,0.263997533926762,0.110124318606316,2.397268
26252183,0.0165178256097559,0.0496936527910847,0.792488406170553
"Efemp2",1808.99494794154,-1.43383889926705,0.252651336819317,-5.67516
846464363,1.38552235521476e-08,1.02718307536036e-06,-19.7707606376907
"Ctsw",385.015454487755,-1.04897017857327,0.431333031036887,-2.4319263
8424105,0.0150187594614798,0.0462323605904537,-3.22461329584062
"Fibp",2474.39165315052,0.161731042200442,0.166793680140932,0.96964730
3565626,0.332222325608139,0.476989829353233,0.119723039159539
"Fosl1",25.2244274355175,-1.15072553699666,0.557902388680598,-2.062592
95594352,0.0391513175705429,0.0963732344388896,-2.69215319523581
"Lrp5",1588.68053211265,-0.365340965452651,0.190195544267441,-1.920870
2646521,0.0547480653624663,0.124472688717044,-0.761249627658327
"Drap1",5523.94725089125,0.198815851853401,0.153653272253223,1.2939252
7043453,0.195691234751598,0.326369660010834,0.222619002896252
"Smarca2",10473.5285318185,0.994018358383221,0.253221345926498,3.92549
196335032,8.65526599659548e-05,0.000883099824184294,6.99000897618166
"Ovol1",420.077002627003,0.734718460950164,0.301109616787511,2.4400365
1822468,0.0146857772659179,0.0455204433586897,2.26998156448054
"Vldlr",2919.37930257078,0.220026547661924,0.208207260981381,1.0567669
2841947,0.290617971193313,0.434291736856911,0.183510670292748
"Rnaseh2c",1864.8130373525,0.651027021361103,0.173743635677213,3.74705
536017793,0.000178922609229509,0.00155196078648198,4.21099649725651
"Kat5",1674.03002530642,0.15677853052741,0.142237477581257,1.102230812
81687,0.270361339426852,0.412803868139761,0.13871493065864
"Rela",3287.63947146499,-0.413484369672649,0.118823198928688,-3.479828
63111439,0.000501734602676162,0.0034715476518736,-2.34162598261134
"Slc1a1",5771.3105922078,1.94683372527663,0.682313608990408,2.85328285
94131,0.00432700877634955,0.0178066730943992,7.84220056719592
"Kcnk7",3.48139404436967,-0.607387206151424,0.878371927930694,-0.69149
2051188764,0.489256374270663,0.625870672190617,-0.284628643828716
"Ehbp1l1",3524.72136704708,-1.20070819030572,0.228689338517802,-5.2503
8988738102,1.51777611843316e-07,6.30993576912789e-06,-14.3765415080451
"Fam89b",1243.71408397174,-0.481094481227434,0.170561019807375,-2.8206
590331763,0.00479251118590977,0.0192193301322231,-1.90120780307323
"Ltbp3",7251.39326008716,-0.880830163013411,0.239234467118972,-3.68186
98142495,0.000231529637466944,0.00189957556137266,-5.51939173478583
"Scyl1",3465.14831415411,0.187267664304836,0.111253596432254,1.6832504
3243766,0.0923266215433174,0.185628401040984,0.315360328630449

"Capn1",3100.32209265592,-0.915598286063642,0.219971974949197,-4.16234
061759505,3.150020003361e-05,0.00040487205026719,-7.15259838593808
"Smc5",1544.88047305593,-0.196959085074748,0.169756198239054,-1.160246
7958041,0.245948339356432,0.385057067670765,-0.187970606688063
"Arl2",1960.19223038042,0.0390576281314353,0.197899562853472,0.1973608
61076556,0.843545155536199,0.899849187145465,0.0041216772691563 7
"Men1",2048.08285209635,0.236957340065022,0.0851079626786958,2.7841970
6696065,0.00536604203856747,0.0209799654437514,0.91564754959941
"Map4k2",4548.31902532034,0.167815046594203,0.179278001560463,0.936060
448764016,0.349242065292304,0.494195644754289,0.11828003853319
"Sf1",8580.71173205134,-0.0760480495394508,0.185737298648933,-0.409438
761587629,0.682217698810618,0.786209627720872,-0.0182919757766057
"Rps6ka4",1923.25068378091,-0.239886718422357,0.116696421507531,-2.055
64759675921,0.0398164964362154,0.0975671795291352,-0.558267757697075
"Prdx5",51074.825189331,1.7834705203159,0.444766985297566,4.0098986194
3709,6.07448234160496e-05,0.000672573859719555,13.0271796144326
"Esrra",7199.0279952694,0.784315482214001,0.197686194143351,3.96747727
180816,7.26374367821154e-05,0.000773006501976605,5.61979540932447
"Kcnk4",35.3116786297442,-0.219962363955881,0.450658295865062,-0.48809
123447656,0.62548521956688,0.742509029596044,-0.0654872493952025
"Gpr137",2770.44293732382,0.383377171020989,0.201338258589693,1.904144
66533295,0.0568913459972474,0.128391884428234,0.786946087348855
"Bad",1379.67667433111,0.171007350061309,0.203214364675941,0.841512116
203047,0.400061100222256,0.544226005172228,0.104039275888433
"Plcb3",3541.74858853943,-0.400799233262142,0.131258663574069,-3.05350
688745944,0.0022618352973913,0.0109978216031261,-1.80762781316722
"Vegfb",2172.30753308579,-0.314502519786012,0.369131559985803,-0.85200
6584855838,0.394210437309606,0.538596812182937,-0.194610390664269
"Dnajc4",1075.79989924717,0.0995825652417322,0.257522257720024,0.38669
4983662335,0.698982030272454,0.799387156211643,0.0222975221871306
"Fermt3",1598.26073486659,-1.09595565608987,0.364583797479536,-3.00604
597260355,0.00264668979458629,0.0123299913150192,-4.81751492669563
"Stip1",4911.58844519244,-0.228345597081745,0.162447323161865,-1.40565
934013095,0.159825306141621,0.281128749063708,-0.289757440328385
"Rcor2",139.359713849897,-1.0441476175912,0.374349374618428,-2.7892329
689491,0.00528330449816707,0.0207610521644609,-4.04573427783601
"Mark2",6171.3714653827,0.152812919819262,0.186460583142168,0.81954543
5523757,0.41247529027004,0.555954874165992,0.0897115980728534
"Spindoc",1114.500604572,0.0711371868677675,0.166220008215216,0.427970
059871863,0.668672918486537,0.775920047887311,0.0180479165611578
"Lgals12",1030.55629736508,-3.41609829939795,1.15765212899584,-2.95088
499717192,0.00316864872230244,0.0140702147090533,-14.5652017439322
"Plaat5",4.84940577835516,-1.20396193357367,1.01992342780853,-1.180443
45364296,0.237823887904501,0.375929482477662,-1.17790061211002
"Smc3",3229.97839354228,0.144521864358725,0.114222921928312,1.26526148
971595,0.205777606548658,0.338758170591501,0.156440407243758
"Pdcd4",2756.05280851005,-0.369033096792537,0.154198879711346,-2.39322
813163982,0.0167008532668964,0.0500830048066946,-1.10491223560432
"Shoc2",2044.60927099337,0.103625987206573,0.0811783733127321,1.276522
09545224,0.201771021218589,0.333598861334694,0.113762269026971

"Gpam",5466.55194896406,-0.509371735893618,0.460997159452828,-1.104934
65187119,0.269187906092744,0.411463295154933,-0.45234016406156
"Tectb",3.07218353969614,-5.16804226536561,2.05820653530347,-2.5109444
4445713,0.0120408644744137,0.0390701906344016,-16.7568369365716
"Acsl5",5170.71830862189,-0.169611865441354,0.212303879385843,-0.79891
0815629046,0.424342127079249,0.567477557873196,-0.0960942937048516
"Zdhhc6",2745.83735320122,0.499651642167717,0.103747917967312,4.816016
08935556,1.46452720142482e-06,3.71330967144098e-05,5.09694734674546
"Vti1a",1411.55525113851,0.120906997990795,0.200250922747398,0.6037774
82430432,0.54599159210116,0.67592025795482,0.0473568737088854
"Tcf7l2",1557.04765991997,0.103917104953304,0.170082865955942,0.610979
268071732,0.541213305459068,0.671719947300579,0.041350047042038
"Hhex",319.595015238796,-0.721158064636801,0.192134948011248,-3.753393
4981708,0.000174456671579842,0.00152239950905652,-4.67848955318168
"Cyp26a1",69.8332025224928,1.33731040071123,0.501827738568185,2.664879
39572022,0.00770159377093707,0.0276752073987709,4.79722435067905
"Cep55",82.6378001996261,-1.02312409238835,0.441451946825494,-2.317634
1156624,0.0204692144575391,0.0584860409573708,-2.90461570844209
"Rbp4",1808.5278647129,-3.7094109594605,1.03703612177542,-3.5769351535
3153,0.000347646370943653,0.00259311742182836,-22.0891508810045
"Eif3a",14787.759368647,-0.109701207530516,0.148165834447448,-0.740394
760638463,0.459060497024109,0.598713148560272,-0.0562737223648265
"Fam45a",1289.44189102522,-0.230490967829796,0.133790049740248,-1.7227
8109080079,0.0849281195189691,0.174161825583397,-0.402845286405327
"Prdx3",6906.24784124897,0.22377605522129,0.40362547246616,0.554415096
386295,0.579294796839688,0.703965219429095,0.0785512870178566
"Plce1",1196.73491086728,-0.733388610198179,0.210948168220356,-3.47662
943170042,0.000507759079594359,0.00350328610542492,-4.14661870508567
"Noc3l",752.627010704739,-0.131234128077241,0.0877793651396164,-1.4950
4530898017,0.134902617714671,0.24702584900457,-0.183499733237452
"Hells",315.625438972421,-0.205877605772783,0.504549481635629,-0.40804
2448295413,0.683242511177514,0.786972027583835,-0.0493205692279836
"Cyp2c55",2.97143668768741,-0.156866982051088,1.32639375659962,-0.1182
65772339909,0.90585707674535,0.942698616694109,-0.00925650852169993
"Cyp2c39",0.0721532113593249,-0.544048378937457,4.78471990596623,-0.11
3705376621746,0.90947135024012,NA,NA
"Cyp2c40",1.31498271775533,-1.50481443360017,2.10601656392702,-0.71453
1148223351,0.474898802530089,NA,NA
"Sorbs1",2055.40489611756,-1.64951843564977,0.572787924888771,-2.87980
658106591,0.00397919207000174,0.0167206020153349,-6.74836741485425
"Aldh18a1",546.864925663726,-1.6458021701898,0.244385624668637,-6.7344
4754543706,1.64553952824806e-11,5.72941602174655e-09,-31.2334612208621
"Tctn3",480.9672282488,0.395509603338558,0.159492104180074,2.479806792
76768,0.0131453590105989,0.0417780293380043,1.25589513980551
"Ccnj",350.006958865264,-0.586587827996302,0.18995287156698,-3.0880703
3638059,0.00201460782569902,0.0100539604262809,-2.69818003334957
"Tll2",0.381232259994954,0.91508513379408,3.79200396243164,0.241319667
083702,0.80930736911824,NA,NA
"Dntt",3.76172072310076,-0.467437869139342,0.881767313692548,-0.530114
761435041,0.596032365039322,0.718626344930788,-0.154447896199217

"Tm9sf3",9677.40615388217,-0.348774547675311,0.295970281439556,-1.1784
1070386838,0.238632912502331,0.376719660591891,-0.340492538199174
"Pik3ap1",878.034070904703,-1.40059625261388,0.416312427049409,-3.3642
9124285463,0.000767405149292556,0.00479579025670072,-7.47920748499391
"Lcor",253.102976128131,0.593711963646241,0.312544224511932,1.89960945
39049,0.0574843906513334,0.129442676000501,1.21385429314737
"Slit1",16.3182511640987,0.900059295315954,0.662824639377273,1.3579146
6074883,0.174490764797026,0.300576104957364,1.08192014257366
"Smndc1",2057.18009807065,0.192319196297293,0.11849667272491,1.6229923
7501598,0.104591034868617,0.203982924247763,0.305733479131763
"Mxi1",2086.48059214152,0.264731907294265,0.152126015562092,1.74021456
038341,0.081821349872606,0.169160500457647,0.470404060704243
"Add3",4402.18553333433,-0.235863916800205,0.227515421466186,-1.036694
19540978,0.299878398061202,0.444303115911651,-0.191344191153816
"Xpnpep1",3743.64201143225,0.846618028011816,0.184562650439199,4.58715
794337121,4.49320756533041e-06,9.15449959339731e-05,7.87243008044776
"Trim8",4530.63072372876,0.195568196986514,0.207465379706849,0.9426546
11882015,0.345857596273536,0.490617054357323,0.139262428355206
"Arl3",2597.93180787963,0.64301338325834,0.134758911216368,4.771583396
2618,1.82783269168808e-06,4.41624310068578e-05,6.44790421959053
"Sfxn2",2091.22174364886,1.12652231451793,0.234093581331865,4.81227339
984563,1.49223023190183e-06,3.7737360650612e-05,11.4734720101507
"Maoa",698.696825966686,-0.570099121638125,0.168634923032941,-3.380670
57158001,0.000723091787845253,0.00458349924558087,-3.07015054975835
"Efhc2",25.9687458544529,1.19228115845972,0.40278359051146,2.960103605
37713,0.0030753559996884,0.0137751645490293,5.1087912029136
"Fundc1",1275.73732291123,-0.224847798191144,0.186741616183209,-1.2040
5832822262,0.228567034936496,0.3650557050452,-0.226580322535918
"Nt5c2",4229.47865990219,0.302817087279829,0.12558321584701,2.41128629
520629,0.0158963647075276,0.0482258159432664,0.918099256743905
"Msr1",238.449450546621,-1.39431707765009,0.48514672005484,-2.87401113
933632,0.0040529466809478,0.0169611030611471,-5.68439733328467
"Pdcd11",2367.41857766685,0.0730963632990287,0.0999060277364834,0.7316
51182167215,0.464381500039414,0.603312265976046,0.0369370807830148
"Taf5",319.542476002729,0.15210706940137,0.191511940591533,0.794243267
190284,0.427053824513778,0.570048296732364,0.0854893736406494
"Pcgf6",1116.79114570736,0.403271612733797,0.140465899804978,2.8709573
8747764,0.00409230686602432,0.0170860383883816,1.64111125413153
"Nr0b1",1.05790615474879,2.14763942861236,2.95444472235698,0.726918128
594747,0.467276111020568,NA,NA
"5430427O19Rik",100.931739670044,-0.944262162172691,0.371993153485328,
-2.53838586362567,0.0111365121910913,0.0368901946116141,-3.11588524560
81
"Gk",14767.8825377331,1.71971621889181,0.38250494462047,4.495931995331
84,6.92658734211702e-06,0.000127051775199543,15.427429530009
"Slk",4769.48813014599,0.0577403019108193,0.133208784033285,0.43345716
5230122,0.664682678496872,0.772651756259624,0.0148927736858996
"Col17a1",138.109668214733,-1.72785039157768,0.451433892123407,-3.8274
7157828669,0.000129466327891227,0.00121654672830031,-11.5968808323529
"Sfr1",3331.68829738693,-0.107276859726638,0.0979656892230278,-1.09504

52201935,0.273496825921672,0.416384876381726,-0.0939901124607211
"Gsto1",3042.60871613105,-1.12914241967298,0.43654757089111,-2.5865277
8062215,0.00969483549269606,0.0330010304533149,-3.85174924396913
"Gsto2",85.138003734839,-0.550061014619219,0.406622861438982,-1.352754
76807337,0.176133987311515,0.302444780678109,-0.657794073495663
"Habp2",3043.30912877872,1.42240405698851,0.624353975809973,2.27820132
825009,0.0227145851879375,0.0634875260886475,3.9214425744956
"Casp7",845.846953222257,-0.515797483374603,0.130795702331334,-3.94353
540812812,8.02891306016838e-05,0.000834031682723298,-3.65661172161149
"Dclre1a",633.689735163513,0.302333648712061,0.258894969242141,1.16778
495000145,0.242893520300129,0.38174575679606,0.291147439785008
"Nhlrc2",1663.33649476574,-0.0520451316452215,0.0855736081657492,-0.60
8191389387418,0.543060544817269,0.673574713921573,-0.0205659645410705
"Tdrd1",4.36739836701111,-1.71369527774396,1.12843865576636,-1.5186428
3360635,0.128852423240783,0.238681792546911,-2.45508102831801
"Vwa2",1499.33873241918,0.828704934441583,0.534913820004287,1.54923074
231834,0.121326261796091,0.228518107853572,1.22328435703896
"Afap1l2",1604.34513820999,-0.670283006735451,0.257649598815797,-2.601
52940201029,0.00928091091925834,0.0319602969098727,-2.30795924763194
"Ablim1",4743.85405640115,-0.283635783089587,0.287616442989961,-0.9861
59831965822,0.324054686905597,0.468999024272306,-0.214756135332734
"Trub1",1005.68804986627,0.143387321725469,0.115398134134739,1.2425445
419945,0.214035717037733,0.348725764749894,0.151054161523782
"Gfra1",165.800988300231,0.0433967942271074,0.377271628947741,0.115027
98222105,0.908422942645398,0.943794136715524,0.00251038356149447
"Pnliprp2",0.386920668513701,2.19145377030188,4.611694478444,0.4751949
16000004,0.634648050459983,NA,NA
"Hspa12a",1865.1277527413,-0.500076465262253,0.148977799872945,-3.3567
1801898499,0.000788735088614036,0.00490550454794146,-2.65910525980642
"Slc18a2",21.483535847934,-0.101449482726257,0.716235911909139,-0.1416
42552459903,0.887362353760581,0.930103815375971,-0.00735093513262802
"3110040N11Rik",671.534279376268,0.218201870721594,0.263372309659054,0
.828492072701436,0.407391882535463,0.551225184893259,0.129963624105461
"Btbd1",3947.26070210915,-0.148894677798398,0.204568393590516,-0.72784
7910349436,0.466706692621094,0.605337893480938,-0.0747404343849038
"Hdgfl3",883.127304029766,-0.221609429419938,0.168474094163426,-1.3153
9172547779,0.188378284993655,0.317458928332436,-0.254276172598574
"Bnc1",3.70635698455588,-1.94087229198589,1.54899403048292,-1.25298887
780787,0.210209754415738,0.34398815734479,-2.07119807882825
"Gcgr",1942.40505110583,1.67435803640527,0.461225439390864,3.630237826
03272,0.000283160193301124,0.00220408347540002,10.2427907097668
"Bhlhe22",49.3600081942388,0.193846906082187,0.328829051893429,0.58950
6629557204,0.555521466208977,0.684848099907855,0.0733823391897485
"Ppp1r27",0.120670194526411,-0.0666950295869129,4.7847199059604,-0.013
9391711318002,0.98887851071652,NA,NA
"P4hb",26002.5314572946,0.638214191897145,0.147078026335687,4.33928988
440804,1.42943863574058e-05,0.000224044972023069,5.36333747447895
"Arhgdia",10845.597576259,-0.42394018699055,0.163056170154835,-2.59996
408960167,0.00932335163265179,0.0320668036926168,-1.45832623641451
"Ints4",1646.85295876252,0.253964129584637,0.186117788861122,1.3645344

2273668,0.17239942796512,0.297691310713319,0.307727878542713
"Alyref",1313.65543611633,-0.309941100917284,0.224626034224231,-1.3798
0934395115,0.167645354896964,0.2912544889763,-0.382330281615805
"Anapc11",2439.81645304658,-0.0370793951563302,0.225482411021679,-0.16
4444734240336,0.8693810500394,0.918103311111439,-0.00316826209554205
"Pcyt2",10607.829790839,1.0561793536299,0.285736973498275,3.6963342219
9132,0.00021873497628634,0.00181716854181128,6.66499418350289
"Sirt7",4713.98976164672,0.23802303840201,0.124985460875016,1.90440581
436933,0.0568573525483956,0.128340432969277,0.488677700733858
"Tollip",2986.0224522899,-0.110099475194554,0.140167837637678,-0.78548
3153982528,0.432170343524628,0.574244061472209,-0.0610722646391445
"Pycr1",54.830155571257,-1.76977978239895,0.510063595724286,-3.4697237
7020139,0.000520993878480183,0.0035743626469411,-9.97088336776624
"Myadml2",7.86053365955957,-0.683898540394445,0.999269205981726,-0.684
398694866768,0.493723439932685,0.62984949174349,-0.316148780673449
"Aspscr1",3713.84434206343,0.611132991990128,0.21917578849273,2.788323
45576527,0.00529816157662668,0.0208016907922234,2.36674757645093
"Cenpx",2452.88591914992,0.94259591491624,0.206333063313797,4.56832220
574709,4.91643854853671e-06,9.83095427268742e-05,8.69769960839084
"Lrrc45",3053.19952742637,0.266467191153367,0.182072566893003,1.463521
91162308,0.143324622415144,0.258945838384959,0.360033510009753
"Mob2",1873.08133401851,-0.187568535211204,0.144652381679335,-1.296684
73504297,0.194739683742162,0.325255641048885,-0.210666440775235
"Cbr2",1517.18311281888,-1.76368902436879,0.54842585304065,-3.21591153
041078,0.00130030911105434,0.00718838305850228,-8.7043150768241
"Maged1",8524.95361334051,-0.0769453153044983,0.101846108433256,-0.755
505698628865,0.449945614366601,0.590737296405628,-0.0405027726426311
"Fasn",90828.5679666302,-3.78770966366649,1.13307848111618,-3.34284846
706758,0.000829231683272206,0.00509995567340939,-19.9935142086477
"Arhgap19",666.582412249782,-0.970381547074685,0.291813091491012,-3.32
53530268863,0.000883066441989687,0.00535719647974951,-5.07443028063577
"Dus1l",2507.76228283057,1.02298536554697,0.263317331870971,3.88499062
43477,0.000102333837472083,0.00100773594731116,7.05864925155352
"Gps1",3938.78348048324,0.199347116269934,0.082568603591498,2.41432103
243734,0.0157645654672707,0.047901231264627,0.605738952311224
"Zdhhc16",1566.6371829448,0.210461671144132,0.101996279049816,2.063424
99064442,0.0390722676248152,0.0962758845126559,0.492593415098109
"Rfng",2443.40507334106,0.65582679313052,0.162708546827323,4.030684348
9084,5.56146897047675e-05,0.000626806485470264,4.836639112969
"Mms19",2455.04431404011,0.2281387941515,0.157554104955627,1.448002857
2772,0.147616251497715,0.26467501307899,0.303254078747698
"Slc16a3",199.252309695039,-1.86901219467187,0.251272297894107,-7.4381
9438249227,1.02070793559008e-13,1.10565351822974e-10,-42.8478789891353
"Csnk1d",6261.32588704689,-0.286589167562547,0.0786391276920046,-3.644
35842529934,0.000268059640177521,0.00212205719211584,-1.76406216004921
"Cd7",79.5076422632101,-1.3493243638931,0.449770959333275,-3.000025537
20518,0.00269956971788624,0.0125056332523986,-5.91216735159629
"Sectm1a",170.150897000968,0.166798390718922,0.545957979796392,0.30551
5070557494,0.759973893666375,0.844657846202704,0.028159513028864
"Ogfod3",833.853512977975,0.804307857310727,0.294981864643865,2.726634

93493669,0.00639837855522154,0.0240131664467608,2.99938705669172
"Rab40b",122.258234531826,-0.51877621569085,0.424483802484296,-1.22213
430207397,0.221656843251233,0.357503939921627,-0.533617830570125
"Ubtd1",736.12907359757,-0.173926212241939,0.142576366189137,-1.219881
08471788,0.222509957321925,0.358405547117958,-0.178463968086392
"Ankrd2",6.946723983651,-0.123966669617576,0.627278281799385,-0.197626
274038965,0.843337476508526,0.899676868028847,-0.0131057086653824
"Wdr45b",1819.99317853559,-0.206842704694743,0.18531304521154,-1.11617
994544651,0.264345117006485,0.406258460738744,-0.186316817639339
"Fn3k",905.640719244383,0.947763682433269,0.490420395308598,1.93255356
32279,0.0532912176170779,0.122057108081497,1.99339936125653
"Hoga1",4461.11664025816,1.94365586317022,0.505990952382265,3.84128580
564389,0.000122391508407698,0.00116409250289429,13.1309764478249
"Pi4k2a",3551.6077936655,0.186833938307862,0.143066997610998,1.3059191
9469693,0.191580038328567,0.321465368961309,0.212031383001045
"R3hcc1l",1227.21136486702,0.0801552293513357,0.0995674864984814,0.805
034175012123,0.420799958923768,0.563823364423834,0.0459300894464743
"Loxl4",122.891340021798,-0.921512363824932,0.448938874426236,-2.05264
550770451,0.0401069705619281,0.0981278881721511,-2.13927590405653
"Hps1",1297.0618413421,0.0120308153130168,0.162137175693752,0.07420146
0963776,0.940850083376832,0.963431275966676,0.000448197451303374
"Cnnm1",431.488728509716,1.2978590594631,0.507908845023113,2.555299188
38889,0.0106096559886623,0.0355197583219331,4.33182026955419
"Got1",3036.34441992449,0.589418839738189,0.261542259104597,2.25362754
667676,0.0242196088698955,0.0667091303496571,1.59580049358144
"Entpd7",529.30384351256,0.165969913911126,0.173213575670785,0.9581807
50373594,0.337971622185825,0.482554971395666,0.12093570955768
"Cutc",669.316926337355,0.891979438623573,0.199773835559561,4.46494625
34729,8.0088854120238e-06,0.000143001142640696,7.89638885718756
"Abcc2",6304.89257808172,1.83757132706548,0.769416674721265,2.38826553
600645,0.0169281059342643,0.0505418405148178,5.48506544326452
"Dnmbp",1226.56412611286,0.0174889813540834,0.192380881236038,0.090908
1050139572,0.927565609992919,0.955515781951002,0.000795818581000009
"Cpn1",2084.31895627903,0.967246410539844,0.620335561452192,1.55923095
60257,0.118941726542142,0.224961275013937,1.44296431344965
"Cyp2c23",512.342999466898,2.05856169321444,0.764450410282141,2.692864
9203742,0.00708409638057288,0.025997687037156,7.51323081546637
"Erlin1",2848.76051248485,0.995169569071787,0.192029090752873,5.182389
63258175,2.19060994181123e-07,8.2456588118601e-06,11.6492795278907
"Chuk",6021.44247033578,0.673560700773564,0.148312051318645,4.54151024
670568,5.585267077453e-06,0.000108144525795609,6.15098462492507
"Cwf19l1",542.904037689439,0.117848357759304,0.202410738346553,0.58222
3842084565,0.560415915876473,0.688661342897804,0.0439581031160715
"Scd3",11.4059198037068,-1.76646591868174,1.18436510138944,-1.49148764
735587,0.135833511951793,0.248287411271778,-2.46098429872259
"Scd2",12288.3927781229,-2.35534029847017,0.506113007098342,-4.6537833
7532533,3.25899190039688e-06,7.06827853992639e-05,-22.5107138613151
"Ndufb8",11690.6034041556,0.547150896684176,0.401794901154607,1.361766
65037777,0.17327153948168,0.298898387756685,0.66076765780763
"Sema4g",1726.65100568519,1.24841704814364,0.34780002502691,3.58946796

523964,0.000331353545681923,0.0025022197651844,7.47873849869883
"Mrpl43",2234.08898117139,0.454275570396348,0.200226151990338,2.268812
36981605,0.0232797384226803,0.0647607846719106,1.24337723660951
"Twnk",1401.46897666217,0.683295879644365,0.169874770140588,4.02235057
671525,5.76202083593526e-05,0.00064642049631223,5.01816617213327
"Sfxn3",2377.33469575346,-0.0642349392432781,0.212406501094994,-0.3024
15128125248,0.762335623744031,0.845988616491811,-0.0107432534512288
"Kazald1",194.771222394656,-0.445728872560601,0.226297744818523,-1.969
65671451144,0.0488777269843545,0.114405914262483,-0.966341311492932
"Lbx1",0.222265746865732,3.19052280584978,4.69750997731396,0.679194471
381224,0.497014649864957,NA,NA
"Btrc",1168.86033104718,0.310156020143002,0.104912954959428,2.95631764
697643,0.00311336270372199,0.0139038813552843,1.32609912325877
"Poll",651.752257943941,0.0933582660834896,0.121416725995613,0.7689077
86945787,0.441948054397999,0.583509700922065,0.050291556647177
"Fgf8",0.303203908982835,1.52294585941981,4.78471990596136,0.318293628
331796,0.750262217052019,NA,NA
"Oga",7678.23948521311,0.282236700129891,0.130189261508754,2.167895392
13197,0.030166646536543,0.0790088724422334,0.716371815921532
"Kcnip2",74.663196542855,0.781145183599139,0.328203207295381,2.3800656
6126002,0.0173095533966717,0.0514719287858587,2.31743801737863
"Ldb1",6502.62418271214,-0.0737792552201613,0.106873831721119,-0.69033
9758872722,0.489980550504858,0.626431878077023,-0.0345076824070809
"Gbf1",2646.72614344037,-0.174096413443991,0.198007303487384,-0.879242
383375439,0.379269868455356,0.524133807863245,-0.112467722643125
"Nfkb2",3343.31121478472,-0.740203327481456,0.300390459765418,-2.46413
727007009,0.0137343500346156,0.0432760757878048,-2.32435341585241
"Fbxl15",678.218519885811,1.06805428165572,0.247783487084595,4.3104336
5408398,1.62934708961628e-05,0.000245131246553536,8.87950081471308
"Mfsd13a",689.60150948268,0.290828620050469,0.144026203724185,2.019275
74656773,0.0434585672558209,0.104557832930612,0.656695367741087
"Actr1a",4678.9718924115,-0.391682462297452,0.159062277067819,-2.46244
72220428,0.0137992480557188,0.0434384466565071,-1.22847693350578
"Pitx3",1.14023440115707,2.03779320176295,2.99529275761889,0.680331896
299476,0.496294332594747,NA,NA
"Sufu",1863.74505958795,0.339307329793793,0.183070530521982,1.85342408
101587,0.0638215984074754,0.139803788984266,0.667592378602821
"Hexa",5276.73589123373,-0.813901554449529,0.184863010153965,-4.402728
01882683,1.06898144078139e-05,0.00018045887560481,-7.01583761929995
"Arih1",3690.1638735114,0.00363800747712502,0.256176238367237,0.014201
1901662394,0.988669470467774,0.992458878882467,2.75386220547948e-05
"Bbs4",634.339098606327,0.440498959411331,0.144775335403833,3.04263815
506014,0.0023451413965 4038,0.0112918086354902,1.97505536807726
"Adpgk",1343.73331006356,-0.261171551270569,0.147628796707878,-1.76910
980171006,0.0768755525016046,0.161122167330569,-0.476792798690391
"Parp6",1201.42029900098,-0.192280453470931,0.181615365370722,-1.05872
349004413,0.28972572674428,0.433476996628298,-0.160730413002529
"Limd1",5506.0787620114,0.451632547344501,0.155811266755065,2.89858722
51104,0.00374848027340253,0.0159929690089283,1.86777431335983
"Sacm1l",3320.05918170283,-0.0669972793905153,0.206724938195966,-0.324

089004331954,0.745870651331369,0.834806879332208,-0.0120966846096342
"Fyco1",1963.12149468455,-0.398266048239187,0.256335102810079,-1.55369
297405305,0.120257669794236,0.226877991838221,-0.590765109743102
"Slc6a20b",7428.24316447722,2.20545530211177,0.821589132057133,2.68437
740478583,0.00726650276395875,0.0265372299853283,8.00405299762141
"Lztfl1",1653.20597098165,-0.0760598526059355,0.211547550938042,-0.359
540218114896,0.719190996655479,0.81414043269295,-0.0156396099022654
"Tbl1x",3650.32918712148,-0.434376160026233,0.145047386177436,-2.99471
897752684,0.00274697799329538,0.0126715306681082,-1.89752772349059
"Zfhx4",39.3914129239498,-0.762400694369171,0.569364907735745,-1.33903
702882057,0.180558618624811,0.308277753585514,-0.897158146775073
"Ribc1",106.78816409985,0.473015523776568,0.310933235233564,1.52127682
144127,0.128190384317109,0.237703862426533,0.679595430560899
"Hsd17b10",4591.2353709486,0.519781160140556,0.288504976351258,1.80163
672292334,0.0716025795934521,0.152649982837012,0.976984656483319
"Huwe1",11060.5978694626,-0.222360830837137,0.263967907501655,-0.84237
8275986989,0.399576248953669,0.543794074272258,-0.135458803513301
"Fam120c",186.047356937547,-0.875296444435886,0.342405423189632,-2.556
31594932749,0.0105787017474439,0.0354404685002854,-2.92340341212203
"Tsr2",1055.60342295311,0.0943759626482345,0.0867794985085172,1.087537
54366271,0.276799315791938,0.420369907637233,0.0817881177821215
"Fgd1",509.04303602607,0.16892009447964,0.241406837178188,0.6997320227
3039,0.484094674054567,0.621714111490683,0.0802834843450491
"Gnl3l",1545.84575171039,0.109150510257212,0.156459356720459,0.6976285
25037518,0.485409537764572,0.622740163783139,0.0516965108142507
"Maged2",1533.21538838788,-0.697062766376822,0.157327511259809,-4.4306
4763940554,9.39504977417765e-06,0.00016283082710837,-6.08033823880767
"Apex2",640.718099860362,0.254748317716462,0.185435235108277,1.3737859
3430592,0.169508149093915,0.293940758718709,0.311907989383037
"Alas2",175.44751951089,0.639968364449596,0.44799213915714,1.428525879
16753,0.153140551259741,0.272414420989182,0.832234550153229
"Pfkfb1",359.613672864664,-2.17647592237579,0.64521952416816,-3.373233
2033532,0.000742910031946166,0.00467870148672039,-11.6762158340841
"Tro",79.5966075007032,-0.566917770954002,0.460017697918432,-1.2323825
2249705,0.217806226501015,0.353188694939162,-0.590021267863655
"Abhd6",1049.03036712835,0.0363851641937429,0.365352975243598,0.099589
0731955395,0.92067056902354,0.951226658634901,0.00181936400606546
"Flnb",5126.96774950917,-0.0803176320640816,0.191345061336045,-0.41975
2835548892,0.67466602296284,0.780412797563447,-0.0199133329707642
"Dnase1l3",271.853972880417,-1.48899528536171,0.59248230736129,-2.5131
4725665511,0.0119659375993899,0.0388853085521508,-4.83497432223718
"Polr3a",1061.59482154233,0.602596588349933,0.183816698862119,3.278247
25435822,0.00104453858084801,0.00608134166896819,3.07476713265833
"Sat1",15667.844011595,0.612416495099111,0.153392482404868,3.992480501
63686,6.53857109344019e-05,0.000711569972138382,4.43881729385393
"Acot9",2036.42820264163,0.0981608356020532,0.147709021267821,0.664555
453414528,0.506334869495181,0.640989305636739,0.0436562960240425
"Prdx4",1058.49417987093,-0.184340734563861,0.230224106621883,-0.80070
1270030855,0.423304607142014,0.566517484388426,-0.104751131168067
"Rps24",24937.7347798251,-0.533047528275131,0.253740776207297,-2.10075

627671151,0.0356623663942438,0.0897798347262706,-1.28485503641836
"Ptprj",3803.61916974205,0.155463131820199,0.375203219553365,0.4143438
10816069,0.67862233304642,0.783570906194209,0.0379164818518885
"Banp",1343.61610679692,0.0518270575760672,0.305263774707995,0.1697779
49007029,0.86518476928985,0.915172388195816,0.00459409700500005
"Car5a",117.349095683465,2.37296968911296,0.593013293337232,4.00154552
313469,6.29300844690226e-05,0.000690107304261531,17.2720477655594
"Jph3",1.2798821612521,0.0391285643488475,1.8085118178677,0.0216357802
930929,0.982738491659173,NA,NA
"Itgb8",3901.38388737871,1.12812268451005,0.470645408949897,2.39696948
712857,0.0165313002755282,0.0497111802548193,3.38608891032102
"Sp4",556.919198466891,-0.049398940527701,0.202635547027549,-0.2437822
05305691,0.807399502723623,0.877322531436982,-0.00646536235679569
"Atp10a",314.852933685117,-1.18859671675212,0.227776721857625,-5.21825
367868393,1.80617976125821e-07,7.14338600101677e-06,-14.0840672029893
"Ube3a",6322.78145736022,1.03547698744349,0.300151159642621,3.44985169
69796,0.000560894592932292,0.00377454001990441,5.7774197794354
"Padi3",3.24184857925848,-3.212896414278,1.42366264111351,-2.256782134
68118,0.024021691605589,0.06627634681276,-8.71955092246461
"Padi1",88.9843591658604,-2.02737999967939,0.534389679401337,-3.793823
26760243,0.000148345279473456,0.00135160572858572,-13.3938088559961
"Padi4",159.720724092012,-2.66398977124203,0.479400922623199,-5.556914
15165649,2.74585723794086e-08,1.73264480341006e-06,-35.3401194193382
"Kdm5c",2576.98601843876,-0.0890644184195833,0.137103690616206,-0.6496
13573633848,0.515941863026941,0.649579326590757,-0.0384250901359653
"Gpr143",5.05299157828243,0.423707119189845,0.866874306245755,0.488775
726927273,0.62500048443801,0.742162500975133,0.126343978385525
"Sbds",3560.73295221698,0.0164241386896188,0.116476842629442,0.1410077
60159419,0.887863811878621,0.93047936597739,0.00118344752726533
"Rabgef1",1562.12953737681,-0.244234322567751,0.132732870696897,-1.840
04400180174,0.0657617775158096,0.142977602364324,-0.475052191387554
"Mettl7b",6722.27440251969,2.2954958343359,0.77765503465477,2.95181762
097761,0.00315909459191591,0.0140501884929691,9.79056397041374
"Itga7",1442.95731182831,-0.884031930429574,0.282931652060105,-3.12454
23549917,0.00178081881972868,0.00914951392544661,-4.14969408240028
"Rdh5",703.157726441891,0.941222744693144,0.216198142179438,4.35351911
540459,1.33969407929613e-05,0.000213409764363693,7.95549367679437
"Cd63",48354.5545048113,1.19914994563341,0.250862910838484,4.780100580
13507,1.75207518559039e-06,4.25429165238375e-05,12.0694408343625
"Gdf11",599.211287883612,0.0180429701802847,0.238062735790812,0.075790
8209378021,0.939585518951289,0.962511041050371,0.000689417229454858
"Ormdl2",1454.24962137249,0.289057703389556,0.175581150951557,1.646291
1982466,0.099703820739765,0.196883238483283,0.469760514881677
"Dnajc14",3337.95962341384,-0.357193197513843,0.1505326153015,-2.37286
249759512,0.0176508334943386,0.0522160448297094,-1.05456485856751
"Mmp19",556.95457244349,-2.67895691333145,0.535542418349462,-5.0023244
12673,5.66431658366294e-07,1.74475268164708e-05,-29.3514895158619
"Dgka",1936.04970294128,-0.248581495899038,0.189299178712062,-1.313167
3237587,0.18912657209603,0.31841722672726,-0.284474854838263
"Cdk2",1420.42043317866,-0.376808778832779,0.127186471711095,-2.962648

25781709,0.0030500488641425,0.0136994265323637,-1.61666087680727
"Pmel",22.3060969065736,-0.276629809383995,0.359748438148574,-0.768953
468728469,0.441920934137128,0.583509700922065,-0.149018874413236
"Rps26",11454.6695248325,-0.128874393731984,0.167335741054615,-0.77015
4618013864,0.441208180361339,0.582916187876771,-0.0695550391392735
"Pa2g4",4228.30610894031,-0.0416144673642084,0.0759814861227837,-0.547
692200925904,0.58390326819109,0.707842944739485,-0.0143791733897115
"Esyt1",2813.48183792615,-1.40393001364965,0.16034443900753,-8.7557137
7678849,2.02815815010289e-18,5.64928965867231e-15,-46.0590781413729
"Smarcc2",7415.31773362246,-0.208641430949687,0.191202209503631,-1.091
2082631855,0.275181252736756,0.41855714688051,-0.181714553167184
"Cdh9",4.28441048597629,-1.4771907496792,1.75297449903191,-0.842676690
673473,0.39940928691875,0.543718653657879,-0.900086811288514
"Chmp6",2109.80805877723,0.343751531916557,0.225436969813897,1.5248232
4527486,0.127303190345837,0.236428590490624,0.495727198428692
"Baiap2",3668.60916442816,0.812245022734791,0.209271971433256,3.881289
10513869,0.000103904233275245,0.00102010309184327,5.59462316835575
"Rnf41",1380.34822114445,-0.16834886600946,0.106409531984249,-1.582084
45117849,0.113630292701855,0.217333272222541,-0.256954794514837
"Nabp2",1668.2962715155,-0.0364995857445815,0.214103698737061,-0.17047
6203633485,0.864635647175563,0.914852681572238,-0.00324817954464472
"Aatk",449.067837901811,-1.97835387149702,0.395741727068611,-4.9991035
4703138,5.75974674427213e-07,1.76577896257576e-05,-21.6517646167098
"Tepsin",1623.56931396886,0.179485076658597,0.184377635105394,0.973464
469028467,0.330322510984405,0.474902545286384,0.133652784053405
"Fscn2",4.0205705558911,-0.595320066625357,0.756917833027382,-0.786505
536861649,0.43157137657966,0.573682758423112,-0.330806693281605
"Cnpy2",3916.39313278591,0.316348141705353,0.206231675680475,1.5339454
5557341,0.125043072002034,0.233645406602364,0.459954592376434
"Il23a",41.3769853188594,-0.292030740481283,0.470454464940256,-0.62074
1776822903,0.534769537199635,0.666641291242151,-0.11841939157772
"Faap100",1079.2906633315,0.314990142584098,0.220755509637936,1.426873
30024387,0.153616418085386,0.273086517125169,0.408846691162122
"Pde6g",44.1002884687995,0.438445664341512,0.363775076835013,1.2052658
1467912,0.228100702160482,0.364579677901884,0.442396128330378
"Mip",0.203352729283892,-1.35109207303677,4.77055421753436,-0.28321490
7834143,0.777012101201405,NA,NA
"Atp5b",65822.9411972998,0.806980308564514,0.401957251531076,2.0076271
9291836,0.0446829226847091,0.106662711764243,1.80608943427173
"Prim1",523.587907651504,0.044928074815199,0.204394254301212,0.2198108
50206138,0.826018469049188,0.88881525868841,0.00529548676312626
"Hsd17b6",1.82532944521096,-0.0332791320123673,1.53532859202308,-0.021
6755762807203,0.982706746499676,0.988843275184532,-0.00037337282151711
6
"Tac2",3.07668707898284,-1.78573885984484,1.32360682283489,-1.34914600
698427,0.177290073292046,0.303894668048203,-2.12694734871788
"Myo1a",36.7516874641433,-0.800164588150919,0.462519914496295,-1.73001
110454311,0.0836282912503102,0.172020721890342,-1.40840196489779
"Nab2",2318.01146207957,-0.455311819898815,0.234940896259584,-1.937984
5192884,0.0526251112569553,0.120900721018984,-0.961976237294012

"Shmt2",6291.47156808092,1.48559669978801,0.36802255664726,4.036700123
28052,5.4208286528555e-05,0.000614187169781651,10.9863008173865
"R3hdm2",4180.83893540152,0.402275623168321,0.140022042044413,2.872944
98276726,0.0040666492916464,0.0170044022921985,1.63898473684614
"Inhbc",0.979955805755812,0.0914925638366043,1.50371895453764,0.060844
1913700133,0.951483295869316,NA,NA
"Gli1",542.01205997626,-1.52057485904006,0.331377321726811,-4.58865094
061455,4.46119677143584e-06,9.1083156701001e-05,-14.1470295791005
"Ddit3",1052.96040157167,-0.0424349803736757,0.182922855556652,-0.2319
82932064679,0.816551270463734,0.882692059184005,-0.00529498945325167
"Mbd6",4608.99939957168,0.0613960667259199,0.193284943581124,0.3176453
66412885,0.750753957553593,0.837952983249182,0.0108544123828422
"Dctn2",7338.36472069134,0.137554580446995,0.119112649302786,1.1548276
4636802,0.248161013329449,0.387525503595835,0.13039811310944
"Ttc4",2126.24978307246,0.268352108206952,0.127873304141078,2.09857804
183185,0.0358541136964734,0.0901299469421487,0.645790098469761
"Pip4k2c",6384.50645269406,0.5797860641352,0.18809788101323,3.08236361
309365,0.00205363813862306,0.010196546072542,2.65872857006854
"Bsnd",1785.38528904445,1.45370438782663,0.580106656250618,2.505926060
60118,0.012213156461584,0.0394910993148916,4.6979073470134
"Katnal2",73.8746211415956,-1.29164530780747,0.472880503786935,-2.7314
4123613403,0.00630579852124186,0.0237566830080486,-4.83061317628703
"Hdhd2",2452.19144206153,0.226144484605096,0.316421524507988,0.7146937
45808646,0.474798303154089,0.61243829815417,0.110880241756731
"Agap2",558.57215090766,-1.02247598869838,0.33180665735118,-3.08154151
233983,0.00205931759799021,0.0102188348150627,-4.68654191562469
"Pias2",1425.97241954388,0.317935194864818,0.153455671626766,2.0718373
683711,0.038280610482968,0.0948209855101526,0.748980454290161
"St8sia5",1.04207279396354,1.62976522570097,1.5642488646403,1.04188359
18898,0.297465652248586,NA,NA
"Rnf165",134.967467357678,-0.053730731329969,0.492503473469298,-0.1090
97162201676,0.913125425690858,0.946557337756813,-0.00295109225814372
"Atp5a1",51837.7934574819,0.719114137899492,0.3881261676982,1.85278447
512115,0.0639132563061256,0.139897617487064,1.41438563491415
"Pstpip2",201.524747406903,-0.897222803614244,0.363812827914113,-2.466
16593691428,0.0136568051105993,0.0431013897776733,-2.82104797229482
"Crisp1",203.233251601759,-2.75300111016842,0.679703078735495,-4.05029
960330625,5.11520991138028e-05,0.000588068177193943,-20.4786603514218
"Avil",104.649415178499,-0.45766776091003,0.381693843587367,-1.1990441
2554475,0.230510788359734,0.367257668854232,-0.458441943598371
"Crisp3",3.83025770371221,-3.27122025329109,1.98876098966493,-1.644853
38876354,NA,NA,NA
"Atp23",218.960947862672,0.578674514805085,0.162472886698486,3.5616682
0547097,0.000368505901209315,0.0027164945413154,3.41904802687042
"Usp33",3393.98876641897,0.130306306024089,0.123359403429897,1.0563143
335736,0.290824629542187,0.434471074419518,0.1086267127556
"Clns1a",1661.26540836778,-0.167442958554579,0.183867405497144,-0.9106
72329888177,0.362468047551201,0.507351183858816,-0.113618728433239
"Paip1",3892.1946906694,0.208617306529483,0.0956533574796804,2.1809721
2712894,0.0291854781441601,0.0770142716003293,0.534845650583261

"Nnt",1545.25094084295,1.49267947911811,0.327299340644168,4.5605942137
8705,5.10090696100736e-06,0.00010097206489921,13.7336463243591
"Cd163l1",47.6233681570305,-1.77703966450074,0.758659687931684,-2.3423
4096363475,0.0191631991260883,0.0556100694389745,-5.13456239389169
"Paox",897.230141835243,-1.26573527458733,0.430630014548219,-2.9392639
4312118,0.00328992773055492,0.0144899505060673,-5.35950279519777
"Echs1",8450.43860435844,-0.41197502944031,0.522201356867847,-0.788919
875488888,0.430158838290998,0.572820450006685,-0.229545467233607
"Fuom",1415.14391795454,0.422363350756464,0.320167810350415,1.31919367
626058,0.187104371874874,0.315913884789154,0.486683215079719
"Prap1",5.0419530430018,-0.376085398184303,1.04666141162241,-0.3593190
63460399,0.719356414731467,0.81414043269295,-0.0773315844827398
"Caly",3.83375936431535,-0.991513121097754,0.900732379578499,-1.100785
47588323,0.270990034472674,0.413461163970684,-0.875696148279887
"Msx3",0.170239085201213,-1.03638225477751,4.78078633615322,-0.2167807
09679533,0.828379245940515,NA,NA
"Zfp511",1113.59497277958,-0.261748529364117,0.115135561495349,-2.2733
9430115768,0.0230024292632602,0.0641174218406073,-0.719033460340082
"Adam8",907.137724072775,-1.24757156970318,0.427564690564103,-2.917854
53110548,0.00352448731657982,0.015305796489843,-5.21425490158393
"Tubgcp2",808.896397888131,-0.526914144656845,0.127521108977053,-4.131
97586567147,3.59658191253755e-05,0.000450734837584457,-4.0596792295880
2
"Adgra1",496.734109277834,-1.65213785227403,0.646060800744853,-2.55724
825027189,0.0105503894242266,0.0353749819381806,-5.52103238420427
"Inpp5a",2063.0320576075,-0.429321188819543,0.172513839647719,-2.48861
882441568,0.0128240375556963,0.0409302806123698,-1.37206120445509
"Dpysl4",34.9662611283854,-1.22566920093833,0.507439920378369,-2.41539
766919484,0.0157180384394889,0.0478087413512404,-3.72670454562528
"Cyp2e1",31394.4225561674,0.913240943936664,0.685558312670528,1.332112
71318295,0.18282315334749,0.311013755279343,1.0665906633239
"Syce1",4.08462307923617,-0.678996034709237,1.324508538794,-0.51263998
2923389,0.608203179322263,0.728311736237813,-0.215259473845555
"Urah",111.910010110743,-0.826945245216037,0.660388082186207,-1.252211
0369988,0.210492975333916,0.344311412169521,-0.881696267121009
"Odf3",0.738493350834187,-2.11133973920993,3.66695513996185,-0.5757746
30074121,0.564767523702761,NA,NA
"Bet1l",1467.41136561341,-0.0620314919569345,0.134400574366886,-0.4615
41866536978,0.644409891461821,0.757366128012212,-0.0172390780087207
"Ric8a",2276.58598086652,-0.293701183620314,0.0991043718381436,-2.9635
5426277242,0.00304108439268097,0.0136734405314807,-1.26065358313862
"Sirt3",2945.81046300372,1.34308146732437,0.259136164189594,5.18291791
315442,2.18441194102928e-07,8.2456588118601e-06,15.7218748722235
"Psmd13",4368.57441088562,-0.125738150503456,0.308670583685156,-0.4073
53849538539,0.683748117570028,0.787368343750319,-0.0300588496983356
"Cox8b",1743.49936549365,-3.95517994996737,1.57420041171168,-2.5125008
992132,0.0119878799586833,0.0389501222186982,-12.8364311965679
"Ifitm5",49.8333255287204,-1.02175290398278,0.406967507221308,-2.51064
983285547,0.012050916914655,0.039089798369979,-3.31241436091248
"Ifitm1",815.367679192351,-1.31350717355541,0.440612860811643,-2.98109

131707102,0.00287223131605316,0.013075593322532,-5.69669097746762
"Ifitm3",6683.17309752018,-1.19310189456331,0.233536905500062,-5.10883
661838617,3.24148492281602e-07,1.1429018630211e-05,-13.5767299413632
"Sigirr",3013.9632258673,1.30421664802415,0.284146503889901,4.58994437
788156,4.43364140449253e-06,9.06154508436011e-05,12.1408038614472
"Ptdss2",2306.91861963401,0.562217082512238,0.21002297141502,2.6769313
7909786,0.00742998505247967,0.0269625625448071,2.03146232806884
"Drd4",2.31199713253182,-4.50443943585093,1.57699685475569,-2.85634015
20219,0.00428555735795842,0.0176733919977735,-18.1785439226614
"Cdhr5",6130.83073599355,1.63995759430206,0.578845725053939,2.83315143
106438,0.00460915455086433,0.0186373487002805,6.53127497138753
"Irf7",2800.67324681032,-0.704320238710149,0.35147254548878,-2.0039125
3243031,0.0450794308276678,0.107268579726369,-1.5723382341805
"Hras",2657.27481531287,0.660899191341809,0.183530768296679,3.60102666
967241,0.000316963057281128,0.00241223485201695,3.98337268774307
"Lmntd2",369.83453744522,0.670425337820276,0.250219274164446,2.6793513
0120978,0.0073764958255409,0.0267983818905155,2.42654669980397
"Taldo1",14373.9869696308,-0.396067408114841,0.434176467886018,-0.9122
26795807871,0.361649340946936,0.506423787711954,-0.269476911529788
"Eps8l2",7845.95758342271,0.735618628914092,0.264863670930538,2.777348
15926119,0.0054804439860l078,0.0212884145751726,2.83183178819896
"Tmem80",1469.07850087184,0.12880093589074,0.133854507215725,0.9622457
88878513,0.335926153299799,0.480478883292215,0.0944074794400543
"Pidd1",371.811663317594,0.00907310902893917,0.221082255173218,0.04103
95172684954,0.967264392160644,0.980031236663284,0.000183012173176718
"Rplp2",12874.8923347256,-0.479869127659927,0.315128297730364,-1.52277
383883348,0.127815297234277,0.237233951972768,-0.690391785952762
"Pnpla2",14336.4453836762,-2.41395769887611,0.715441946110363,-3.37407
907378097,0.000740630894005179,0.00466736301593826,-12.9560996930147
"Cd151",6628.19497845952,-0.699515737911385,0.239156116096731,-2.92493
350924152,0.00344529829173561,0.0150350103160835,-2.93612901449112
"Tspan4",2773.68948926541,0.452284245917671,0.106091310887186,4.263160
12249689,2.01555977631816e-05,0.000290783946044037,3.68291896365822
"Chid1",1512.80173083518,-0.454839559142262,0.278635990999881,-1.63237
906743518,0.102599656024828,0.201236102320902,-0.729233540901323
"Muc2",37.5368343217253,-1.01544908823451,0.44556070000992,-2.27903647
743596,0.0226648970072553,0.0633940843275661,-2.800999258092
"Tmem192",872.21969768893,0.0820661309872567,0.338531513935808,0.24241
7995397669,0.808456291849064,0.877981551658296,0.010679240787768
"Apool",1582.24726150051,0.519909605119974,0.308347548852669,1.6861155
7657749,0.0917735403568512,0.18489424554011,0.87759247284421
"2010106E10Rik",0.377176584997985,-1.46274577043762,4.76608547779842,-
0.30690716254491,0.758914037090838,NA,NA
"Zfp711",131.656607868892,0.875981436388901,0.2207348664637,3.96847788
671826,7.23331679865117e-05,0.000770263303878212,6.27971657965939
"Chm",1011.2886983265,-0.0591181717751901,0.127518169535819,-0.4636058
68798047,0.642930148853425,0.756128357702158,-0.0165261380080668
"Crcp",959.904235063649,-0.0137860572453753,0.104069804304039,-0.13246
9329961451,0.894613080353635,0.934839264737402,-0.000928913826836012
"Asl",5088.53439180873,1.7541239554022,0.365341564510391,4.80132600776

749,1.57618424213446e-06,3.92997958480021e-05,17.7943442638025
"Gusb",2341.39017744603,-0.407799235239187,0.18884279540245,-2.1594640
8953601,0.0308141804054837,0.0802964097932769,-1.02848205570813
"Phkg1",558.555578118988,0.0828190564583155,0.305969640180624,0.270677
366582595,0.786639186029308,0.862066706901947,0.0122922217691276
"Sumf2",868.540868983262,-0.0974829704751051,0.207544316419732,-0.4696
971333966,0.638571416205119,0.752681989672797,-0.0276961270233673
"Tm9sf2",7296.98432220531,0.0608125394066343,0.219828357439393,0.27663
6463625492,0.782059263002178,0.859027182131512,0.00924075200721919
"Clybl",3198.0161618623,0.879436298780947,0.45503791903992,1.932665964
71006,0.0532773605477562,0.122039705822386,1.84981407596015
"Fgf14",8.22479444619517,-3.43301089421796,1.29804513498012,-2.6447546
404236,0.00817501708723181,0.0289548561610982,-12.1597841362834
"Farp1",2635.94873396345,-0.139295342828182,0.212905476496639,-0.65425
9087743011,0.512944899294538,0.646962911342578,-0.0606584289652254
"Slc15a1",15.666236570391,0.124448716091615,0.515090712432474,0.241605
435873453,0.809085909642563,0.878225077448683,0.0161599601993509
"Dock9",3364.47904002526,-0.119916374169962,0.320133491691473,-0.37458
2407908545,0.707971058456503,0.806372100212635,-0.025807200512471
"Tnrc6c",3327.18758593692,0.256699171761704,0.18669242438928,1.3749844
0336524,0.169136280093895,0.293407746828895,0.314762473382349
"Tmc6",2407.4639486342,0.435661169417933,0.16813968323937,2.5910669095
154,0.00956788869355381,0.0326830227307134,1.49035308466823
"6030468B19Rik",4.62407253902719,-3.70054680919536,1.29348466200158,-2
.86091278691239,0.00422423238619746,0.0174776768571376,-14.97548688014
31
"Tk1",506.963299685833,-0.211716316653925,0.360787719403207,-0.5868168
6007532,0.55732671212962,0.686422603316488,-0.0796607626287787
"Cant1",3521.05351227184,-0.109326223480379,0.0756105140837255,-1.4459
1297659106,0.148201611762581,0.265469455778301,-0.144994503989901
"Rbfox3",44.5053540691751,-1.61992023573781,0.778515434480531,-2.08078
114317504,0.0374539429183321,0.0931713420543046,-3.84458114535814
"Cbx2",403.006569753395,0.179606298785051,0.245865481193469,0.73050636
4346934,0.465080724166176,0.603906352529865,0.0905819471882353
"Cbx8",511.572929181477,-0.182275897356122,0.233125332138169,-0.781879
410891693,0.434285446110115,0.576386742104351,-0.100429720708281
"Gaa",3876.09038392075,-0.121255635052539,0.128160912385578,-0.9461202
54572901,0.344087254044844,0.488655425792926,-0.0868308799933328
"Eif4a3",2988.25957449672,0.345088334920844,0.194659095429799,1.772782
99870296,0.0762646716410841,0.160151703571121,0.632075446368314
"Nptx1",7.13089081335542,-4.11299013071332,1.18423350452543,-3.4731242
7405231,0.000514437092819706,0.00354050170985102,-23.2116056589989
"Rptor",1561.73204482424,-0.217235966000343,0.145453320471641,-1.49350
984423005,0.1353037793447,0.247574426582485,-0.303270973156705
"Pde8a",1954.99526363292,0.285205725515744,0.223206549214351,1.2777659
3706421,0.201331968184422,0.333127182252195,0.313506955821958
"Cpeb1",1098.75871638509,0.061015729228376,0.192914757232306,0.3162833
68383797,0.751787433817722,0.838578454495305,0.0107416444308017
"Nat1",24.8685947093957,0.14769753596349,0.704280029381134,0.209714218
49527,0.833890727547183,0.89375155694601,0.0165904865430137

"Tma16",579.818819164162,0.0594590267028949,0.195382749652008,0.304320
759170377,0.760883531176135,0.845194957606807,0.0100002923557038
"Dach2",5.37802603088385,-2.43829671064474,1.23496699039079,-1.9743820
9249072,0.0483383126939261,0.113485902577504,-5.30592055277826
"Klhl4",114.234403878466,-1.20513516627795,0.37666605950115,-3.1994790
501539,0.0013767619280805,0.00752305564578233,-5.89284931340389
"Zfp202",293.553369577941,-0.350915350747432,0.16976520661595,-2.06706
284369145,0.0387282326946055,0.0956865787173977,-0.823485064535766
"Copg2",1768.79148792552,0.0148967153387832,0.227596748973957,0.065452
232538206,0.947813937647926,0.967716193970742,0.000488856907999892
"Podxl",15723.0658961357,-0.463472354875627,0.370229433589224,-1.25185
172443598,0.210623898419714,0.344467771463478,-0.493947861169329
"Mkln1",4894.66525030265,0.29932983973946,0.196016708021461,1.52706288
540816,0.126745374834514,0.235786214019464,0.432480659705245
"Map3k7cl",1035.61378051579,0.603487444124887,0.420846615597657,1.4339
8431104847,0.151576740053734,0.270323175484104,0.789444308716609
"Bach1",1742.10059240816,-0.293096979398121,0.16559777515669,-1.769933
07501137,0.0767382901771478,0.160920970302649,-0.53544184337217
"Cct8",7292.68139683699,0.123718060196324,0.107841213544735,1.14722429
514392,0.251288932988275,0.391281873135711,0.116088005618252
"Usp16",2318.63048655264,0.458832610737648,0.203432727267264,2.2554513
0766913,0.0241050153421204,0.0664498217362736,1.24403660913832
"Phf6",797.622905478208,-0.574920985192322,0.144508887201584,-3.978447
25210797,6.93667958557589e-05,0.000746420411476593,-4.13955847416568
"Hprt",4589.61069258772,-0.0207404847082422,0.184003222131876,-0.11271
8051716384,0.910254089258972,0.945059346995571,-0.00117199402659308
"Ccdc51",618.926025146036,0.402862595022794,0.158118828160242,2.547847
08254049,0.0108389971313235,0.0360831084286402,1.33828151501025
"Atrip",817.197636839099,-0.022836950005307,0.130049984881777,-0.17560
1327643884,0.860607159252532,0.911681431725247,-0.00211161075484472
"Shisa5",6222.76302313117,-0.494392935741053,0.216410564050603,-2.2845
1387255499,0.0223413482388911,0.0627140236052259,-1.36905818196939
"Pfkfb4",907.997553640277,-0.374266594609491,0.189785084764164,-1.9720
5483810581,0.0486033467206872,0.113951028577994,-0.812901989679482
"Col7a1",1162.34131067734,-0.382015350118139,0.299044653011164,-1.2774
5253516998,0.201442527519279,0.333253555198618,-0.419778198458682
"Uqcrc1",16743.9625313755,0.372654177392904,0.399194958811354,0.933514
23700971,0.350554523132362,0.495477498516476,0.261690186878711
"Tmem89",1.14469579297437,2.26121111668969,1.41903091342183,1.59348968
038832,0.111050381549881,NA,NA
"Arhgef9",311.91660099468,-1.2821650910996,0.401590730920934,-3.192715
84819529,0.00140941563802681,0.00766335362806659,-6.24581796350873
"Cnksr2",14.7583680135485,-0.236540825470069,0.408399499023312,-0.5791
89803209251,0.562461110346359,0.69047829311423,-0.0876078013032181
"Rps6ka6",385.744484949688,0.555978951308392,0.280755571648156,1.98029
534389846,0.0476703516887664,0.112242062218037,1.21598015781709
"Tmem47",798.91612934225,-1.13468598443429,0.23324253006145,-4.8648331
1656475,1.14553421267468e-06,3.06387189008653e-05,-11.7930705976802
"Alox5",42.3279095629962,-0.963778336028355,0.479320523814618,-2.01071
785609812,0.0443552668714732,0.106180355243706,-2.16138487696988

"March8",6020.39553033454,0.690547035041619,0.170597662378486,4.047810
65234985,5.16989415904055e-05,0.000592607856043343,5.13143959472041
"Myo3a",3.23280153065283,1.87243781416152,1.12002774936709,1.671778056
58797,0.0945680953992103,0.18921382494549,3.11737968898981
"Wdr73",1313.65515190063,0.171095675138551,0.127471079810126,1.3422313
154749,0.179521005723153,0.306890555141827,0.202109177485628
"Nmb",132.454384403706,0.133617819674,0.1982768656978,0.67389515768143
8,0.500377970907525,0.635594115749506,0.0605549414363688
"Sec11a",4157.95525099403,0.700969038589897,0.216711571966623,3.234571
33474099,0.00121825483875577,0.00683751665113992,3.49456245098723
"Slc28a1",445.98986402352,1.99547980958922,0.815979653821229,2.4455019
1937311,0.0144650678662925,0.0450831031420991,6.18448632169757
"A930017K11Rik",254.558122383095,1.55646048611587,0.543390031301839,2.
86435230029331,0.00417862977694758,0.0173314025507177,6.31181342320308
"Pigq",5821.25698345075,0.542777498595096,0.142162417335449,3.81800977
198038,0.00013453259805156,0.00125268223343329,3.62709339565806
"Rab40c",1442.3824574738,0.00399062658683227,0.166586228862668,0.02395
53210014863,0.980888247144798,0.987676050548919,4.9485841050553e-05
"Mettl26",6873.13187294529,1.74583769599242,0.407986277028968,4.279157
88909847,1.87601751921624e-05,0.000275026989396077,14.3134970756156
"Mcrip2",3736.80647435108,1.41024901773977,0.463712933043468,3.0412113
1253324,0.002356283996126,0.0113215439518149,6.31939329224725
"Rhot2",4517.13623089039,0.68714939205354,0.134064870448045,5.12549924
344154,2.9675035812162e-07,1.05756066307006e-05,7.87264342179139
"Rhbdl1",485.805596483383,0.235331796337301,0.261623045610806,0.899507
135496709,0.368382596813137,0.513381736306378,0.156904082942835
"Jmjd8",2926.71920166165,0.431819783426354,0.154457747507293,2.7957146
2354754,0.00517851048000513,0.0204228554488552,1.68025423571223
"Wdr24",1411.97846378362,0.250399897412826,0.133895701072716,1.8701115
5254968,0.0614683285487097,0.136024227674809,0.499528330363402
"Fbxl16",142.274495958041,0.13313362867427,0.255485170838618,0.5211011
98309338,0.602296276224799,0.723839545970854,0.0430268627094132
"Gng13",1.63627449657215,-1.94950784325254,1.70730974339415,-1.1418594
960847,0.253512427060595,0.39392979750482,-1.81612751609445
"Prps2",1863.67208244236,0.687744286651146,0.211843372412417,3.2464753
5025191,0.00116843565940586,0.00662464625969625,3.45038440946039
"Sdc3",2924.51812472861,-1.40828997167216,0.245758012614169,-5.7303929
0435312,1.00198272842766e-08,7.97414662811532e-07,-19.7750542981774
"Hadha",9790.88871415513,-1.16663970064228,0.493142580408777,-2.365724
93836412,0.0179948082369211,0.0529748974923613,-3.42751407033982
"Il6",12.4283209810181,-0.627412254824915,0.748009429901814,-0.8387758
62634875,0.401595098021055,0.545589549973142,-0.38014176073213
"Tyms",872.617188286772,0.406803614948795,0.206645204181746,1.96860902
995362,0.048998004832771,0.114579407319425,0.881334817642485
"Hspa4l",1768.60567999839,-0.0626415526901568,0.206606622718254,-0.303
192375278212,0.761743259362759,0.845664505366508,-0.0105007640772085
"Plk4",249.505089017112,0.129782241783984,0.412701352682239,0.31447011
4867567,0.753164010887708,0.839656463060073,0.0226810616768815
"Mfsd8",555.735060733523,-0.68837385820372,0.348463634017162,-1.975453
93838663,0.0482166568897947,0.113268479040629,-1.49927437908233

"Larp1b",3600.10825718293,1.27408204187744,0.258893849654526,4.9212526
4303344,8.59920374100377e-07,2.46207451603659e-05,13.5204581940463
"Jade1",4069.58466997324,0.243314492080369,0.146191618878899,1.6643532
2316202,0.0960418620961655,0.191318372205868,0.402397489679715
"D3Ertd751e",883.301272240261,0.235273396362302,0.228037626854853,1.03
173059467092,0.30219834082024,0.446722005254968,0.189587723579965
"Crispld1",78.893748638192,-0.493426049049911,0.293436878523302,-1.681
5406827289,0.0926579412312568,0.186156057509227,-0.82953304037848
"Gdap1",4.76710364738094,-0.397104211774077,1.05238814159484,-0.377336
266039908,0.705923724270529,0.804612885314632,-0.0863280750138764
"Ly96",204.595887281417,-0.0902659011799744,0.202975570104199,-0.44471
3130420748,0.656527079132892,0.766111974919691,-0.0240492677084351
"Itih5",1607.00483760748,-0.963293675434724,0.374986032180334,-2.56887
881885549,0.0102028129086435,0.0343760922918145,-3.24667917357984
"Atp5c1",14604.8080537987,0.833034331971757,0.336732001500492,2.473879
31132093,0.0133654839078571,0.0423475119518404,2.63392594491714
"Taf3",1637.57939279604,0.114821596732384,0.176972303832665,0.64881110
9115429,0.516460476026772,0.650051408015622,0.0494541014753393
"4930412O13Rik",50.956836274714,-0.952142230721287,0.529488051943513,-
1.79823175844365,0.0721402887865,0.153490981095502,-1.78442257876919
"Clec3b",626.371430868941,-3.42170082738043,0.753218644915379,-4.54277
234170808,5.55192001185278e-06,0.000107712772528463,-31.2608037954515
"Exosc7",1744.74320135277,0.414513896168479,0.123778535517185,3.348835
03376828,0.00081152098788248,0.00501046112151127,2.19535982468139
"Zdhhc3",2834.7751962404,-0.204928030673464,0.151673299806402,-1.35111
47376304,0.176658681206601,0.303025509471832,-0.244671421970338
"Tgm4",16.1150143779441,0.28399432654629,0.617063734672211,0.460234997
762669,0.645347552787942,0.758044543758034,0.0786701573596227
"St8sia2",85.3141697354231,-1.38523948875249,0.551768844984292,-2.5105
4314020198,0.0120545592082607,0.039091896889023,-4.49072488694407
"Slco3a1",6941.89344592189,1.18498948299516,0.366900578738049,3.229729
11918242,0.00123907554505432,0.00692844708272702,5.89190940703173
"Pgm1",3369.25332408205,-0.651304966033601,0.560832034446262,-1.161319
12235838,0.245512145817393,0.384652134925474,-0.622267119506707
"Slc25a10",12763.7984903356,0.501118069539004,0.501389923119486,0.9994
57800071467,0.317572972016732,0.462610221022206,0.38629710410088
"Hgs",2486.88227655681,0.0958312500870423,0.101157516162144,0.94734680
8451049,0.34346208615206,0.488071114043647,0.0687391958649086
"Rpl14",19707.6190184622,0.058115364376802,0.188444915896387,0.3083944
40361319,0.757782211907408,0.842854396336032,0.00993546410629986
"Rassf3",3139.91904787357,-0.267954139258512,0.174714851840638,-1.5336
654922898,0.12511196727031,0.233729341557584,-0.389495857362341
"Ccr1",122.505302299725,-1.53884301311573,0.447118893859281,-3.4416863
9314992,0.000578100038099785,0.00385416619912237,-8.55381367465255
"Itgb1",16238.1977077744,-0.726844614906854,0.201140185914076,-3.61362
206962139,0.000301949097191033,0.00231878830131184,-4.40955586705182
"Nrp1",4149.45426625297,-0.491645846643525,0.203071658700358,-2.421046
0966834,0.0154759146749709,0.0472295170343689,-1.50086508392813
"Pard3",2005.87990382604,0.777231986340397,0.183535306978529,4.2347818
4734953,2.28773687267144e-05,0.000318844128258383,6.25734564951087

"Homer2",305.856617296588,-2.26243980126343,0.563644704857562,-4.01394
669685608,5.971183643921e-05,0.000663396801647702,-16.5568449914249
"Dhtkd1",1126.38387379089,0.823489888272803,0.218593123556024,3.767226
8682402,0.000165070981939863,0.00146231440520829,5.37552367469109
"Sec61a2",1225.83216047833,-0.183317294370319,0.216987638593709,-0.844
828284036793,0.398206720291538,0.542610733733863,-0.112073428804296
"Nudt5",2238.64872585517,-0.41714776987508,0.275601133712822,-1.513592
35811327,0.130129260515552,0.240248113013184,-0.59488737874208
"Zfp282",982.551038526264,-0.150458111013467,0.125041937797296,-1.2032
6119111631,0.228875262065396,0.365458181946694,-0.1514516490552
"Pdia4",5911.71085015224,-0.386490205833134,0.158056904912728,-2.44525
986413903,0.0144747805317081,0.0451061644250032,-1.19763125295459
"Iscu",7518.97267229274,0.88366138010215,0.238259380881803,3.708820936
37489,0.000208226613933387,0.0017469890354876,5.61112739779493
"Pramel7",28.8923411112365,0.814586122038688,0.54130318718869,1.504861
12278281,0.132359785312928,0.243375244627637,1.15113303829049
"Fam20c",2065.30946361525,0.317168611098468,0.329723160843299,0.961923
967631751,0.33608779808562,0.480533833473155,0.232439439810178
"Prkar1b",355.681319335041,-0.603941578347075,0.200744593953539,-3.008
50730997445,0.00262534486103617,0.0122637695497085,-2.6580108608601
"Pdgfa",4041.493265417,0.00749321272368076,0.343169732060212,0.0218352
961337686,0.982579338649583,0.988843275184532,8.40695597417902e-05
"Dnaaf5",719.993974250584,0.0985426499732361,0.167732161609375,0.58750
0029974742,0.556867930337719,0.686291062873703,0.037096720686457
"Get4",2562.49454018033,0.0788246227954494,0.128402885247864,0.6138851
36952254,0.539291232499587,0.67020568239919,0.0315432985736214
"Xiap",3404.21313250362,-0.431399784358838,0.164093854125564,-2.628981
9728942,0.00856409006832118,0.0299789278549598,-1.5130314445101
"Stag2",3252.37432311415,-0.21873227035753,0.100739706227359,-2.171261
74523354,0.0299113922918156,0.0784731333296315,-0.55667340131172
"Cplx2",1978.06609660767,0.563734773619396,0.289619713039256,1.9464654
7951999,0.0515988507092452,0.119160771186647,1.19922265590465
"Higd2a",4210.56604944157,0.87796797668061,0.274717329826677,3.1958958
5860685,0.00139397415898896,0.00759655818149191,4.28453640877795
"Nop16",1468.09846747901,0.127931050688426,0.187162152276949,0.6835305
59635385,0.49427164658796,0.630247037464415,0.0590585269320993
"Arl10",257.094173853015,-0.518222098794009,0.267135108835079,-1.93992
508530185,0.0523887949511017,0.120485577253666,-1.09667467713107
"4833439L19Rik",17049.0284278303,1.38161673158969,0.35219639609791,3.9
2285879951368,8.7504439338099e-05,0.000890016462292256,9.7048497860342
1
"Thoc3",1472.5502475383,0.031289143433107,0.186602279001017,0.16767824
9165095,0.866836408549259,0.916273245901195,0.00273594320888419
"Faf2",2359.89691082418,-0.135289948932511,0.155552006340176,-0.869740
944624308,0.384441991037891,0.529161847486556,-0.0861067686162889
"Tspan17",916.108973557553,-0.577783857717477,0.235833332872018,-2.449
96689264036,0.0142869350486682,0.0446564058318264,-1.7961899093043
"Unc5a",470.404864682348,-2.36546354898893,0.497978255314277,-4.750134
21518993,2.03281684284857e-06,4.81871058438007e-05,-23.5136990103401
"Hk3",389.856887862687,-1.4364035629138,0.568834002972172,-2.525171764

36105,0.0115641752712411,0.0379593079863063,-4.69882156031556
"Uimc1",2501.03393606295,0.281593543508402,0.206987332230242,1.3604385
3734571,0.173691190314642,0.299516302180497,0.339485355276357
"Smad7",1481.29584959775,-0.166038605884974,0.151189016050982,-1.09821
870809044,0.272108998989114,0.414753162172851,-0.146125882406388
"Myo5b",2912.88070890077,0.817864146765613,0.245513558363569,3.3312382
1029255,0.000864605809106793,0.00526979808251461,4.29032166960931
"Casp12",1010.84273322953,-0.914229640526732,0.17845803257301,-5.12293
914342413,3.00809458058913e-07,1.06639687513321e-05,-10.4666859452223
"Casp1",443.866894740709,-1.06819575373168,0.261739275542854,-4.081144
30482859,4.48145194543725e-05,0.000533451465397653,-8.05007538096208
"Snca",117.029222211001,0.618845425034891,0.300578210163286,2.05884992
361459,0.0395086174620686,0.0970078105119523,1.4437439638684
"Gria4",315.039476542213,-0.875526229291095,0.313554867378905,-2.79225
845418975,0.00523415300672678,0.0206131115583031,-3.39864216351873
"Kbtbd3",321.641114272396,0.42368500858746,0.150606914664343,2.8131843
0519425,0.00490535266049639,0.0195872550019166,1.66630067778209
"Aasdhppt",1287.90903559466,0.101276821230014,0.212298902791822,0.4770
48255540561,0.633327759913602,0.748446855130336,0.0293454733842838
"Cwf19l2",2357.13007819149,0.604241888256016,0.274510132252042,2.20116
424592018,0.0277243984417088,0.074040586332891,1.57292736023913
"Alkbh8",974.044591432884,0.216182740557608,0.123357017666497,1.752496
49065018,0.0796884727759434,0.165876571173838,0.388374739376647
"Rp1",22.8361373578782,0.59885761095481,0.44015662039219,1.36055572768
896,0.173654130548806,0.299505372617481,0.721996463359107
"Sox17",1523.80677373855,-0.320792275298694,0.237382122660212,-1.35137
503912995,0.176575325435319,0.302989148582051,-0.38304467484617
"Lypla1",8996.07643322677,1.00223309764396,0.320101669631064,3.1309836
6153193,0.00174221852969043,0.00900238677426743,4.72078402254266
"Oprk1",0.488178588788027,-1.78247207366888,3.77462455369514,-0.472224
998357502,0.636766198250702,NA,NA
"Rb1cc1",3075.00092635621,0.408718236786593,0.122996037191907,3.323019
55508439,0.000890486717299435,0.00539382106676122,2.13453149330256
"Sntg1",73.229247736107,-0.683268490992105,0.512930174810634,-1.332088
70241326,0.182831042312638,0.311013755279343,-0.798001664154618
"Adhfe1",7260.45993178328,1.32831473312694,0.520223266837025,2.5533551
0309475,0.0106690660023798,0.0356757758385187,4.42764940800583
"Mybl1",159.958405973123,-0.221331041323089,0.282026197995168,-0.78478
8941227656,0.432577325057149,0.574667349183368,-0.122609413206384
"Sgk3",1163.67825868449,-0.175570790482397,0.0813716963918239,-2.15763
955118967,0.0309558671289213,0.0805629334329561,-0.442213067660975
"Ppp1r42",15.1093073203782,2.69297129638504,0.484606117479552,5.557031
1625269,2.74401785820722e-08,1.73264480341006e-06,35.7245843187777
"Cops5",2862.5413721264,0.292918852100693,0.143428043959501,2.04227042
365162,0.0411247142138286,0.100010195258576,0.674440719991837
"Stau2",877.374799131277,-0.0549807482438277,0.18960918340837,-0.28996
8804545786,0.771840103141744,0.852438480943061,-0.00877790933056
"Rdh10",3234.64072144374,0.265772089705665,0.231284838726773,1.1491115
9403593,0.250509968227571,0.390255941235313,0.250078911049405
"Gm5045",0.485947653249792,-1.09283392030318,2.60139201932969,-0.42009

5822614529,0.674415454256636,NA,NA
"Terf1",849.581920516619,0.2152615262169,0.15797595328337,1.3626221063
5801,0.173001638124568,0.298512030102021,0.260239355523225
"Tfap2b",3028.30618084425,1.44884486260952,0.595963893896891,2.4310950
3351924,0.0150532656394235,0.0462785048340853,4.45241281505801
"Il17a",0.751411000708648,-2.05552594007546,2.39517984190323,-0.858192
735307142,0.390786046809755,NA,NA
"Msc",52.3630092243147,-1.19374876912694,0.423931348837393,-2.81590114
154268,0.00486406313778385,0.0194662362603673,-4.70226448589313
"Paqr8",224.763655516464,-1.56877802945927,0.346965283639061,-4.521426
50413183,6.14242867609919e-06,0.000116256100862452,-14.2126818990291
"Eya1",44.3634860707754,-0.79759347344439,0.397140019284663,-2.0083432
4095827,0.0446068298841063,0.106521000499731,-1.78614129322912
"Tmem14a",281.496476326495,0.172161016763368,0.241934494384049,0.71160
177965395,0.476711404698777,0.614215222944344,0.0839129816036673
"Gsta3",2898.61820315018,-0.640017958160098,0.615383395451219,-1.04003
124375954,0.29832538523428,0.442674913340791,-0.521563183738376
"Tram1",5192.06908042441,0.182439456980648,0.11296882674041,1.61495398
549083,0.106320659982596,0.206272659536383,0.287990971587035
"Gm4956",209.337219013324,1.48170860338796,0.507614927284529,2.9189618
4242319,0.0035119920989841,0.015261789234993,6.19710252275201
"Lactb2",20717.2460020765,1.54280295279787,0.474088273295491,3.2542525
0887036,0.00113691175117726,0.00648931654697136,7.77202116574827
"Slco5a1",63.0713532705302,-1.84227972544472,0.531652468578285,-3.4651
9546946003,0.000529846219922815,0.00362362034235533,-10.3541309504349
"Ube2w",2065.70700302043,-0.182792256184341,0.203399650737145,-0.89868
5201876598,0.368820361411552,0.513786888720227,-0.121729902147277
"Tmem70",2467.09345409075,0.169410952782954,0.341820923780714,0.495613
17343942,0.620167356675727,0.738263820774365,0.051408437625249
"Pth2r",3.93122165023066,1.74905072240032,0.8942633712265,1.9558563826
6885,0.0504820747314946,0.117276241286153,3.7486058945609
"Pikfyve",2099.1279046745,0.243328975350511,0.122695493857534,1.983194
06605958,0.0473457564720159,0.111639564601689,0.533493855994121
"Idh1",14453.6517593205,0.421095288825778,0.382788439895064,1.10007316
036298,0.271300246886708,0.413783808964098,0.371579385302104
"Akr1cl",0.263212321644214,2.34899760513778,4.74735974378913,0.4948008
43397411,0.620740708968433,NA,NA
"Mettl21a",567.482327342433,-0.273437179599514,0.162380434644926,-1.68
392934898489,0.0921953235579916,0.185475074157421,-0.460696469536556
"Creb1",2406.87462305729,-0.187481998540162,0.127581889764727,-1.46950
322562157,0.14169634756263,0.256598438262855,-0.255021059846233
"Klf7",3541.43897681105,-0.283376296300357,0.236331462978148,-1.199062
92936781,0.23050347718349,0.367257668854232,-0.283855650630331
"4933402D24Rik",0.102419796623515,1.37442010063246,4.78471990596623,0.
287251945284957,0.773919414831981,NA,NA
"Fastkd2",988.312800391704,0.723093418086642,0.20854475030061,3.467329
75557682,0.000525656600136335,0.00360128334134162,4.06845981824041
"Mdh1b",9.76476788042871,0.64250177290804,0.524681232797011,1.22455642
158753,0.220742395713098,0.356586178203633,0.662534550441262
"Adam23",231.979121685474,-1.35877340365827,0.314243437050005,-4.32395

157211207,1.53258953232338e-05,0.000236038157197798,-11.3478192925325
"Eef1b2",12081.3869209629,-0.21853679924166,0.292160844531259,-0.74800
1668711901,0.454459151013333,0.594700974930064,-0.113572823434465
"Ndufs1",7366.62939401492,0.668740757956589,0.328818044475299,2.033771
47085621,0.0419746422495322,0.101618482247067,1.52909570397702
"Nrp2",2521.55185465661,-0.974698112154193,0.167285224628572,-5.826564
26661913,5.65800846306572e-09,5.20376646287054e-07,-14.1026272149971
"Maip1",1272.53240642825,0.468581016724715,0.19363601664565,2.41990630
070752,0.0155245067743261,0.0473506934602932,1.4292535469721
"Boll",4.42077315688008,-0.103007850603931,1.23552800767887,-0.0833715
221053117,0.933556131727469,0.95858009670979,-0.00435745409803466
"Rftn2",334.899416047764,-1.12474851306436,0.239283119409335,-4.700492
52049529,2.59534762588001e-06,5.87054385259958e-05,-10.9584002274275
"Mob4",3285.72977042148,0.19104348602847,0.137075000466922,1.393715012
78653,0.163403680900073,0.285806849497834,0.239270321714787
"Hspd1",19781.1486943581,0.309672363000397,0.38876728088904,0.79654944
8019989,0.425712746579804,0.568655542503614,0.174803979838368
"Coq10b",1876.14833108774,0.740762350292599,0.185735359099089,3.988267
79071941,6.65575078686718e-05,0.000722571430079824,5.35770761209847
"Sf3b1",13762.7097003805,-0.000544529493334727,0.116642026633982,-0.00
466838162066095,0.996275183910585,0.997451786181699,-1.38934851068297e
-06
"Ccdc150",28.9429832101995,1.92717864586069,0.427667253449051,4.506257
21356587,6.5981100487529e-06,0.000122523761648175,17.3584942413823
"Slc39a10",1298.95818558353,-0.3489158967924,0.130266392741811,-2.6784
7976326445,0.00739572010494927,0.026853212403408,-1.26215783568184
"Cps1",15.1101445740761,0.50266051983l403,0.584908830672352,0.85938268
2346573,0.390129420845426,0.534369051467798,0.315001549715772
"Slc40a1",2676.47524278059,-0.0750931668122025,0.265123329054372,-0.28
3238623624865,0.776993922674659,0.855777410851861,-0.0116953830654379
"Wdr75",1166.67654453939,0.371732005585877,0.243646181333793,1.5257042
1399959,0.127083544580005,0.236145521035066,0.536523424160777
"Ikzf2",1133.33642104239,-0.570937723421233,0.284126368096814,-2.00944
997553585,0.0444894347866017,0.106322536162412,-1.27963016504786
"Lancl1",1897.57559500598,-0.0142933961368302,0.147253806916864,-0.097
0663946562677,0.922673667985972,0.952320744184557,-0.00069828078120015
9
"Acadl",14883.4372190978,-0.224044836929343,0.51677279136248,-0.433546
116734678,0.664618070503515,0.772651756259624,-0.0577871770922932
"Kansl1l",3104.04390942643,0.587643704094387,0.25465531908340S,2.30760
427942179,0.0210211562821002,0.0598001904272528,1.65524330775205
"Rpe",1724.62382577904,-0.589821392090195,0.326713340482148,-1.8053177
4802879,0.0710249739238724,0.151797099810113,-1.11193749301718
"Icos",141.459955638074,-1.38925453424088,0.450950522945519,-3.0807249
654941,0.00206497294883926,0.0102345812294021,-6.36554062557046
"Ctla4",94.4627069063059,-1.49979913759256,0.619160629713507,-2.422310
24651315,0.0154221776903153,0.0470876324155602,-4.58300355650048
"Cd28",118.440150080531,-1.59360522395246,0.478721994281665,-3.3288740
5005009,0.000871978275052809,0.00530478390233375,-8.34913082103752
"Raph1",2449.49582707742,-0.160537045898808,0.203522967588369,-0.78879

0807254241,0.430234283258828,0.572841593544606,-0.0894425812489897
"Carf",574.584333909471,0.257252917985118,0.192511743831136,1.33629727
135385,0.181452128039753,0.309369848943608,0.301813701369986
"Ica1l",34.8466205991135,0.889507583826349,0.296263820740187,3.0024171
7535404,0.00267844846944021,0.0124343781564632,3.90252791468618
"Wdr12",956.51314384382,0.135069299592168,0.145506361159538,0.92827075
4046785,0.353267140644784,0.498155978035148,0.0941219665516392
"Nop58",2505.27421284335,0.30402080606141,0.290520586710778,1.04646906
266946,0.295344521318766,0.439724150631742,0.249785828852625
"Sumo1",4447.87862948326,0.118634915117367,0.167653874545628,0.7076180
93759474,0.479182453515851,0.616584140345282,0.057367157092153
"Cdk15",112.954641349601,0.0730767947585749,0.367061461637807,0.199085
990756181,0.842195479636696,0.898991228193709,0.00778136339474699
"Als2",1709.79658788975,0.499053772408196,0.139588705838385,3.57517300
136014,0.000349996381818569,0.00260466773003758,2.96959460125305
"Stradb",1472.41875162313,0.467921735595122,0.231452047223919,2.021678
96636676,0.0432095282048824,0.104089372490585,1.05867545262564
"Trak2",4144.03734980061,0.468630108923368,0.143425685098069,3.2674071
4958368,0.00108537462753938,0.00626484147062252,2.37726775142844
"Casp8",2207.66055347939,-0.0301634783173013,0.132695122734856,-0.2273
14144601775,0.820179475173022,0.88465228782008,-0.00369685419057287
"Cflar",6355.51189851556,0.557047649551261,0.143646621906153,3.8779028
8528464,0.000105360756595555,0.00103232363422117,3.83022792115386
"Ndufb3",7004.12825733066,0.422344880228105,0.383320993114247,1.101804
72192982,0.270546577104136,0.413008380421072,0.373474253780901
"Clk1",14398.9000671201,0.316484443730432,0.19836648499278,1.595453202
39934,0.110610926959604,0.212691504325282,0.48989022638779
"Ppil3",816.551343488192,-0.188947518321441,0.216662311971457,-0.87208
2996817338,0.383163101296007,0.527942488097629,-0.120693614262356
"Nif3l1",805.105585119795,0.491608491251997,0.166043260554893,2.960725
35319476,0.00306915496352968,0.0137628953396613,2.10692537333994
"Orc2",952.15716555708,-0.212610687880755,0.160304762441376,-1.3262905
2713583,0.184743483369111,0.31301081323696,-0.246951043580783
"Sgo2a",90.6409421519123,-0.765917856559751,0.377449576728526,-2.02919
251678119,0.042438685479818,0.102561909951102,-1.74421605169747
"Col5a2",2323.43218349,-0.3325547306107,0.179654915700051,-1.851075041
92052,0.0641587580770779,0.140305906795297,-0.653114275799701
"Col3a1",18347.3561666712,-2.21392599638112,0.368975850466634,-6.00019
213610111,1.97084184248356e-09,2.5281233055753e-07,-33.6309049766962
"Poglut2",1200.0149057152,0.0907914979560417,0.167796267794784,0.54108
1748415765,0.5884512377703,0.711984004594807,0.0308418567188404
"Ercc5",1704.93839980739,0.302910158115233,0.176679681945608,1.7144594
9403784,0.0864443701305653,0.176398987839431,0.525551206321597
"Tex30",252.788573414004,0.00525901652803786,0.163349440233971,0.03219
48855196883,0.974316634830588,0.984077077375299,8.44127600224026e-05
"Ecrg4",93.7656674434028,-0.910777960984426,0.389644590625913,-2.33745
8245016,0.0194153696274395,0.0561496406104739,-2.62279916996725
"Khdrbs2",10.2967121343992,1.48350449275788,0.748160533356951,1.982869
21939264,0.0473820396671162,0.111684599785956,3.25195526741563
"Slc9a2",1307.06888554607,1.57380583236953,0.277545244714632,5.6704478

3630755,1.4242473053874e-08,1.04398398347532e-06,21.6751879623607
"Ptp4a1",143.750846699772,-2.61713617292776,0.720849826398626,-3.63062
607090162,0.000282734558296406,0.00220246041456785,-16.012110643954
"Slc9a4",291.817572101955,-0.962230247931804,0.323833079673574,-2.9713
7725676987,0.00296467354598389,0.0134149929913191,-4.14854247635046
"Il18rap",249.445201858767,-1.29829911555689,0.440591720574646,-2.9467
1700562955,0.00321166979674375,0.014228842921361,-5.5209964534624
"Il1rl1",316.333088053327,-1.33029282707484,0.425487785091258,-3.12651
237870323,0.00176893057275414,0.0091046636252037,-6.25100410410145
"Il18r1",676.588020978772,-0.980854191255291,0.337416029863243,-2.9069
5789305813,0.00364962320774115,0.015687908576838,-4.07531676670183
"Il1r1",1607.48913349035,-1.20600036712238,0.160601978499018,-7.509249
75142664,5.94671785854245e-14,7.2468190503663e-11,-28.1575438943644
"Il1r2",32.5788735410139,-0.992576507540981,0.530302881717748,-1.87171
622436945,0.0612458737191925,0.135701368838274,-1.98247169367286
"Map4k4",5889.55986518816,-0.862641571672707,0.219435779592144,-3.9311
8010780222,8.45299150343609e-05,0.000867911681590294,-6.08112326386873
"Npas2",546.475925953607,-2.16311405361021,0.278306704822956,-7.772410
85508975,7.70061977357516e-15,1.25122236954307e-11,-54.3034966991639
"Pdcl3",2420.93213255932,-0.19757080118036,0.176755221380538,-1.117765
00652848,0.26366737063113,0.405605324654762,-0.178282892235308
"Chst10",105.591541827941,-1.89753782221499,0.353337662448461,-5.37032
426451787,7.85951904494767e-08,3.81206224722363e-06,-23.6762250317822
"Rev1",1067.35698769807,0.580091660421375,0.212037768660568,2.73579402
427117,0.00622299646019658,0.023528405076772,2.1750808644733
"Eif5b",14316.379456436,0.563295059757835,0.250758673833281,2.24636321
107818,0.0246807527491278,0.0677209846752735,1.51659262599429
"Mrpl30",3053.80595076044,0.27022184743978,0.304479041473795,0.8874891
55679823,0.374815602086505,0.51989433090152,0.176760135421317
"Mitd1",658.518884928019,-0.0653326204958998,0.163256301835425,-0.4001
84371208898,0.689020724965465,0.791523366250907,-0.0152744974112206
"2010300C02Rik",164.053516069699,-1.22473405121837,0.349328252682561,-
3.5059690758288,0.000454948074176272,0.00320932617593667,-7.0320484938
4653
"Stk17b",1307.22173042414,-1.200148285736,0.191016527952641,-6.2829551
9031505,3.32196836723301e-10,6.54259992164739e-08,-19.8532683331038
"Asnsd1",1715.65813371014,0.0690519636304661,0.174829079449189,0.39496
8410564301,0.692866201298795,0.794957349236431,0.0158451341310591
"Osgepl1",744.58526991477,0.482562947341765,0.222750432550916,2.166383
88449128,0.030281864395569,0.0791787303184663,1.22380259133632
"Ormdl1",674.412374222997,-0.260535979312928,0.310155949539424,-0.8400
16061919235,0.400899380862356,0.54500721026255,-0.158133942183685
"Pms1",194.661794821545,0.0298779694836666,0.151946146630861,0.1966352
56280979,0.844112978687335,0.90015942126699,0.00314266631389976
"Mstn",0.225873622438741,0.455031861125789,4.78471990595967,0.09510104
45896777,0.924234568375759,NA,NA
"Inpp1",930.566899057402,-0.157493567914556,0.180038334450157,-0.87477
7965456891,0.381694728654934,0.526589104883175,-0.101006093545434
"Gls",12964.5958541039,0.0123750093035973,0.256938612556704,0.04816329
15366749,0.961586105328162,0.976714205130679,0.000291569977980912

"Stat1",4920.56059405785,-0.99658534875414,0.333865140180654,-2.984993
7259544,0.00283584140306588,0.0129431731453601,-4.33234267325925
"Nabp1",5003.87497605796,-1.11913577183885,0.476409317394762,-2.349105
55057745,0.0188185713539815,0.0548549116848455,-3.24892226525878
"Tmeff2",73.9432091473511,-2.00175905280825,0.571148293513917,-3.50479
739769978,0.000456954882925087,0.00321721533076632,-11.4885738200409
"Mgat4a",1421.9689146497,-1.03178154834957,0.180888620153576,-5.703960
52263312,1.17055198169331e-08,9.05691370597466e-07,-14.356793730978
"Unc50",1551.21659815226,-0.129164047108483,0.275765232534439,-0.46838
4088600989,0.639509944528187,0.753465949815754,-0.0365626542706027
"Coa5",4390.78995458781,-0.201717183550888,0.320868950821958,-0.628659
092860672,0.529572264524629,0.661855010172503,-0.0832504496822632
"Inpp4a",1229.52762584672,-0.324916639696943,0.161341449216206,-2.0138
4480724193,0.0440258297373125,0.105541314181887,-0.730624711329645
"Cnga3",1.82829289201341,-0.34294604385684,1.12043290023606,-0.3060835
18062157,0.759541056758967,0.844280914696217,-0.0580504776826442
"Tmem131",1909.13464183853,-0.375693746687858,0.186465217210234,-2.014
81945163142,0.0439235701575358,0.105379816775149,-0.845380115143887
"Zap70",242.602382652723,-1.33946890113477,0.413030935225402,-3.243023
19002761,0.00118268584501403,0.00668599843609267,-6.70771162647006
"Sema4c",1408.10991171146,-1.59904167730705,0.197114303663627,-8.11225
59225118,4.96885102285887e-16,9.68826572437022e-13,-44.2337954554443
"Plekhb2",6883.09633671339,0.593369336515196,0.26227810721502,2.262367
00735659,0.0236747375814837,0.065560294470071,1.61680388747726
"Cfc1",0.612388385130176,-1.21229829385891,3.62846742169171,-0.3341075
31629345,0.738298416910788,NA,NA
"Prss39",1.34531693879114,1.12692849614752,1.55334487088146,0.72548505
9546396,0.468154507304665,0.606356818157632,0.563787290412085
"Ptpn18",1884.81876309553,-0.112863985389332,0.296243358687756,-0.3809
84018981139,0.703215106894693,0.802909653582756,-0.0247751213326767
"Imp4",2350.1616212889,-0.405568110833488,0.165589798563616,-2.4492336
7472832,0.0143160537588772,0.0447330795177223,-1.26011716115519
"Dst",9610.6544833926,0.103656328720566,0.199684311308003,0.5191010152
05353,0.603690300426451,0.724800090992299,0.0333627636347076
"Prim2",256.531880933415,-0.17182025025936,0.208585632520131,-0.823739
622827458,0.410087520504628,0.553616733005555,-0.101594332782007
"Zfp142",1157.92058389936,0.0371746446029876,0.203055940359841,0.18307
5878189573,0.854738482300788,0.907618501682864,0.00360338071768216
"Col19a1",69.0647411325136,1.97578184217753,0.538010321273038,3.672386
50273929,0.000240295883508302,0.00194823172382901,12.3305247706058
"Rhbdd1",879.264809661641,-0.36127942365664,0.205055901500984,-1.76185
821043003,0.0780932567586726,0.163323213587965,-0.654647035538419
"Col9a1",0.743435922580991,1.08327665871538,2.33899443675262,0.4631377
6625282,0.643265621125088,NA,NA
"Tm4sf20",9.04975882041967,2.57690507040089,0.900389804311911,2.861988
28336377,0.0042099246975496,0.0174352403892995,10.4345632926789
"Mff",3388.12489907584,0.0696108146417594,0.164047047436844,0.42433445
6056324,0.671321914040511,0.777744187757687,0.0174972084427748
"Fam135a",1568.10985707015,0.589711200186867,0.155425552363552,3.79417
149380618,0.000148137264885633,0.00135097305460246,3.89618071714282

"Sdhaf4",992.917677366208,0.642495178079657,0.271590106114315,2.365679
61650718,0.0179970110262284,0.0529748974923613,1.88761042657913
"Smap1",4210.0537900284,0.56212070756573,0.205810559771082,2.731252993
97156,0.0063094016744657,0.0237629348751656,2.10212248940906
"B3gat2",2485.64317424366,1.79202581474356,0.702463071186833,2.5510605
3008008,0.0107395671890839,0.0358623190705187,5.96398402507979
"Ogfrl1",1746.64722919628,0.217568094270434,0.227170773198499,0.957729
250145774,0.338199303210045,0.482844696052534,0.15840267399178
"Agfg1",2276.97725372113,-0.31062594031527,0.146861089882396,-2.115100
33436367,0.0344213967419646,0.0873208943110625,-0.75735745281697
"Nhej1",202.882736491839,0.207375814610381,0.171200452769971,1.2113041
2481453,0.22577886847316,0.361863846560386,0.21079487200321
"Sphkap",9.06825674433389,-2.00859426357128,0.81401677859577,-2.467509
65875203,0.0136056550742402,0.0429886667699782,-6.32068211288035
"Ccl20",64.4667157486098,-0.389262236160433,0.779923517824954,-0.49910
3088013047,0.617706762221645,0.736255617912247,-0.119183499312573
"Wnt10a",171.863378637542,0.226863758632683,0.527315394988172,0.430224
038192118,0.667032677567118,0.774569897397634,0.0579517515759137
"Cyp27a1",3008.48570731069,0.149535927475345,0.213719797402973,0.69968
2150612334,0.484125825969999,0.621714111490683,0.0710706759279979
"Rnf25",2261.41574091253,0.261337731952778,0.164959026394964,1.5842584
5292669,0.113134916294479,0.216541140464293,0.399840124407456
"Bcs1l",759.515896421927,0.211498747086156,0.213286393757879,0.9916185
6206471,0.321383630963888,0.466696807666186,0.161178006302576
"Plcd4",119.613111897064,1.48836420286566,0.461931500680695,3.22204526
141307,0.00127279027117971,0.00707181383926476,7.36984115795887
"Cnot9",1371.55791037931,0.0386378200486071,0.178080242011534,0.216968
595797981,0.828232818888673,0.890530688358406,0.00447958056535695
"Vil1",5009.08112462995,1.3849900300123,0.592168709696019,2.3388436560
9805,0.0193435264066703,0.0560166460533576,3.99168860376315
"Ctdsp1",7120.10901652042,-0.0323357572512856,0.138438811713996,-0.233
574362932909,0.815315432656066,0.882334478877059,-0.0040479216058703
"Slc11a1",966.069924302281,-1.56522283132213,0.399683320891131,-3.9161
5749146679,8.9971485347965e-05,0.000908821270936982,-10.9618222623722
"Pnkd",3586.62349231436,0.678583769811861,0.235807193541372,2.87770597
504188,0.00400578305631327,0.0167967221574185,2.77308110852056
"Cxcr2",265.761708219578,0.71543312733004,0.367567553383152,1.94639902
446523,0.0516068266949697,0.119165076610436,1.52190193552997
"Ppm1f",1339.6156680905,-0.719213528946441,0.241722911221875,-2.975363
50737676,0.00292641547688595,0.0132765409462539,-3.10826584414166
"Igfbp5",12871.665921878,0.110904681946089,0.324285829636417,0.3419966
94923219,0.732353379531653,0.82482822285745,0.021358037960916
"Xrcc5",1844.78598859018,0.819607708441469,0.149791938590329,5.4716409
7183521,4.45887448267845e-08,2.47689842345483e-06,10.5799089043512
"Tmem169",328.321188284715,1.18863680817974,0.595400717404673,1.996364
42052162,0.0458942706366249,0.108769477193742,2.63701990957249
"Pecr",12415.4697790762,2.06665514841504,0.654458127261169,3.157811114
7672,0.00158958517317889,0.00841078200994357,9.87497599845083
"Atic",1614.40661579406,-0.689093298567366,0.219850616480887,-3.134370
55395872,0.00172223221006195,0.00893458103779566,-3.25102229520599

"Fn1",6149.5369629977,-1.33901828336265,0.342133605641962,-3.913729201
93028,9.08815531790747e-05,0.000916240188151809,-9.36674356247064
"Bard1",118.414154177913,-0.587566419473664,0.310727318487769,-1.89093
904692127,0.0586324812984207,0.131253285919243,-1.19312784920391
"Zfand2b",1239.70242287867,0.382314616009369,0.138586850081213,2.75866
444605192,0.00580380958447277,0.0222410926254029,1.45501819642422
"Abcb6",1267.18720465701,1.34086754525382,0.305818087442473,4.38452661
995033,1.1623826882089e-05,0.000191420081543051,11.4792209111396
"Ankzf1",2052.56525264981,0.289841073008058,0.143811524280875,2.015423
13425436,0.0438603324739768,0.105269479488122,0.652499424639604
"Glb1l",3954.82602265569,1.15667082933124,0.329674062949941,3.50852845
074099,0.000450593037113473,0.0031866750227198,6.64944291284494
"Stk16",3781.64820628239,0.194326043363437,0.140168319039545,1.3863763
5590546,0.165632012349888,0.288450605287435,0.241592242808004
"Tuba4a",4926.72497890198,1.06452099128106,0.305274646836448,3.4870926
9607765,0.000488302079169588,0.00339789933606304,6.0513737709306
"Dnajb2",3362.00291427785,-0.0365114467331301,0.148843312403711,-0.245
301224109413,0.806223203999422,0.876804589613832,-0.0048002010977698
"Ptprn",42.8510193609226,-0.801384242366085,0.604272143885872,-1.32619
75592862,0.184774267543733,0.313035769273413,-0.930757964344689
"Slc23a3",308.691870717638,0.957278867387579,0.304170037530398,3.14718
331614734,0.00164851572862133,0.00865681650327463,4.54650813434835
"Speg",1975.94249883977,-0.720149205113956,0.282058345943819,-2.553192
32871555,0.0106740536799496,0.035686333788007,-2.40024827756739
"Des",2874.65822287309,-1.21085111928116,0.224622613169385,-5.39060205
113041,7.02220238743794e-08,3.50871259537519e-06,-15.2086065379795
"Dnpep",2137.47115844289,-0.593338520617024,0.283709528685705,-2.09135
915654891,0.0364958805729597,0.0913709141514598,-1.41975707134591
"Obsl1",991.319747975288,-0.964825141409281,0.299336816460439,-3.22320
9068694,0.00126762995267697,0.00705372397753869,-4.77993630827343
"Stk11ip",1847.24981029838,0.09829225168533,0.145837681366602,0.673983
9166686,0.500321538923844,0.63558156308292,0.0445475082852834
"Trip12",7139.33395722923,0.041165852271836,0.0930095884834748,0.44259
7940094639,0.658056574992835,0.76718241266631,0.0109102138092269
"Slc16a14",490.048340655895,1.99323592437324,0.64757758676623,3.077987
81969393,0.00208403433032829,0.0103028654596199,9.11971854501334
"Sp100",2928.80351858627,-0.926777306641144,0.226557009451036,-4.09070
241916942,4.3006866540081e-05,0.000516665362784042,-7.01395738467407
"Itm2c",5848.260374861,-0.960581034859791,0.358213800637722,-2.6815857
824285,0.00732741254317849,0.026679717977011,-3.48100314754759
"4933407L21Rik",8.43704136983281,-2.24203270135504,1.33518415545319,-1
.67919360950929,0.093114317027885,0.186841921725811,-3.76099272289161
"Spata3",0.132379703865316,-0.0614346238760028,4.78471990596043,-0.012
8397534408383,0.989755640445417,NA,NA
"2810459M11Rik",41.6598966547369,-0.157068565680342,0.607699788357169,
-0.258464078299179,0.796048768635389,0.869397798176934,-0.021982451477
3437
"Htr2b",32.2890123953117,-1.88583450427129,0.820086607033829,-2.299555
30074094,0.0214734269566338,0.0608117471024614,-5.28028437387313
"Psmd1",8430.60357173033,0.362117092255782,0.177385038774037,2.0414184

5760209,0.0412092513620266,0.100120356242196,0.833369850086293
"Ncl",17367.8020835318,0.735129171041886,0.232202235015331,3.165900496
14875,0.00154603669963692,0.00824073908406798,3.52763880323404
"Epha4",445.47567738068,0.0354873503824214,0.291987614233827,0.1215371
77100953,0.903265569876381,0.940839535899979,0.0021641136975540б
"Nmur1",1.14635837263623,-0.23191010782201,1.79272589647772,-0.1293617
21319282,0.897071435089192,NA,NA
"Ptma",71350.1606271057,-0.113953812114063,0.157777888833184,-0.722241
962779362,0.470145728094689,0.608129201342044,-0.0566769702091456
"Pde6d",1229.68462999597,0.0414809462705494,0.113194745737393,0.366456
464037504,0.714024493203799,0.810363769993461,0.00872228291854281
"Cops7b",930.929606350338,-0.0628513210712863,0.0940410147236816,-0.66
8339460776351,0.503916929814574,0.638799317178634,-0.0281677580060196
"Nppc",2.34731496817051,1.63222855038778,1.12714691216901,1.4481063051
9036,0.147587322460338,0.26467501307899,2.1696439976133
"Farsb",1853.94442337003,0.242214114888123,0.218407121860642,1.1090028
2383041,0.267428963800692,0.409563734807471,0.21621551721737
"Ecel1",3.85835271716793,-2.68903957861271,1.64072285186733,-1.6389358
9679224,0.101226610744301,0.199232998461033,-4.33817455826731
"Mrpl44",1493.85754566672,0.16211509598841,0.30204866729482,0.53671846
1433152,0.591462101802162,0.714562740004867,0.0544843670550733
"Serpine2",4934.4425167839,-1.18852847009326,0.29976422965389,-3.96487
756883314,7.34336276069987e-05,0.000776698269304374,-8.51040891787089
"Chrng",0.113058343341289,2.23400583696369,4.75316341366045,0.47000400
4184502,0.638352156775581,NA,NA
"Eif4e2",2718.12282977223,0.110310118402243,0.128099425804356,0.861128
906000857,0.389167050431655,0.533350611465271,0.0693383220459812
"Efhd1",3150.71348528704,0.992872265051573,0.389521576830403,2.5489531
8798699,0.010804680235562,0.0360119068774339,3.30021373015833
"Snorc",0.500716019844024,0,3.56693976570851,0,1,NA,NA
"Ngef",1556.4151647116,1.56432020190152,0.390783115568435,4.0030393831
7819,6.25338874680488e-05,0.000688084502173824,11.3907522686817
"Ndufa10",9287.88726186263,0.794884741049489,0.339073987698432,2.34428
11005498,0.0190637973601477,0.0554123316827907,2.29956428883528
"Rnpepl1",5077.08141836295,0.45580571763689,0.168923885889389,2.698290
50662114,0.00696965897872094,0.0256695146896677,1.66936619297591
"Capn10",995.776435956069,0.458544196271271,0.13862216065224,3.3078707
9146469,0.000940081718092635,0.00560370325263534,2.37724333469809
"Gpr35",213.112368893313,-1.20302474999813,0.418686290742143,-2.873332
07845355,0.00406166935037297,0.0169872220063432,-4.90267938219577
"Agxt",1.69568554714864,-0.0422076275508129,1.15255910930432,-0.036620
7921225744,0.970787364932118,0.982120689121903,-0.000761471051595087
"Mterf4",675.204301070489,0.23150297322985,0.19603272367278,1.18094045
163744,0.237626380139758,0.375762612811882,0.226594574009048
"Pask",173.990536854622,0.178677022475936,0.289921957750229,0.61629351
5201315,0.537700818033321,0.66903400732284,0.0718139380353556
"Ppp1r7",1749.40090363844,-0.621962977142445,0.333450363228189,-1.8652
3406698711,0.062148602348635,0.137093952776749,-1.23589566683149
"Sept2",748.307535600169,-0.843411438906071,0.237185550510169,-3.55591
408115694,0.000376667170342401,0.00275478487897079,-4.97141781536791

"Stk25",6793.65887036766,0.00733095926665399,0.114631437448321,0.06395
2432507522,0.949008102585916,0.968428323871889,0.00023518308374079
"Bok",2661.64405125006,-0.810848060945381,0.321849066088974,-2.5193425
9371511,0.0117574196526621,0.0384512191190215,-2.64203884230846
"Thap4",2585.53409522101,1.34160686475084,0.286276967389846,4.68639470
713642,2.78059768141628e-06,6.21031999911279e-05,12.9957607122809
"Atg4b",1921.52006942784,-0.210951418590065,0.165531101037397,-1.27439
144226079,0.202524723664198,0.334567211512715,-0.2309743974
"Dtymk",2167.15863080471,0.0133178978226204,0.090330363942163,0.147435
449625196,0.882788326491544,0.927053739965106,0.00100874743239662
"Ing5",1084.43526446457,-0.236892596739948,0.106479460378739,-2.224772
7016773,0.0260965008535967,0.0707986049316027,-0.6272716931224
"Pdcd1",127.363726770923,-1.78159516419701,0.564951838217474,-3.153534
59122864,0.0016130611021567,0.00851650835901745,-8.49063508561712
"Inpp5d",1481.43999642009,-1.10865086303225,0.332254822086159,-3.33674
875227172,0.000847645371378115,0.00519239379551696,-5.83212488942517
"Atg16l1",2130.18252731166,-0.0430676398394753,0.106285577848325,-0.40
5206808970215,0.685325500417641,0.788665566859083,-0.0102248140296416
"Spp2",3822.0626924853,2.00594939441832,1.14096312593039,1.75811938951
364,0.0787271972950071,0.16426141175581,3.62333866034031
"Iqca",3.84431119995601,1.25453681854345,0.897464574246415,1.397867787
25708,0.162152773546912,0.284219235762109,1.57821908743779
"Mlph",839.803594309002,-0.330408341496061,0.186360354230943,-1.772954
0323078,0.0762363241842508,0.160109419317592,-0.605274322725516
"Rab17",1592.64693829548,1.53554617066036,0.572947468218956,2.68008195
486701,0.00736041371132976,0.0267549117344347,5.56026981588352
"Lrrfip1",4208.1240731242,-0.265860122314014,0.195004325370616,-1.3633
5500153001,0.172770655509824,0.298208440644914,-0.321680797317418
"Scly",3827.12996729702,0.770686481271577,0.20215922306633,3.812274649
56524,0.000137693750168106,0.00127662992904315,5.13549367623568
"Klhl30",52.2816420387626,-0.86073450425499,0.281801519054174,-3.05439
980289646,0.00225511330035967,0.0109678971028254,-3.88430779588655
"Ilkap",3630.7891329852,0.265912162372488,0.140244140730223,1.89606611
005592,0.0579513032267552,0.130210474882058,0.542089358593607
"Asb1",636.675324674351,-0.287229887382274,0.151913590358029,-1.890745
17102343,0.0586583694387991,0.131296164311526,-0.583162757418739
"Cdh7",0.220934797106265,0,4.78471990595636,0,1,NA,NA
"Hdac4",1080.69754604335,-0.15076028507889,0.206809405750325,-0.728981
762371574,0.466012818529989,0.604786870054429,-0.075814206112596
"Serpinb8",476.681067775957,0.143315248389807,0.158000898679529,0.9070
53374933593,0.364378572425786,0.50936717845985,0.096678481687093
"Cln8",1168.60533565905,-0.324458334650866,0.173723123836732,-1.867674
99619566,0.0618073858825958,0.136579786133905,-0.645946681960406
"Relch",1995.173571836,-0.0106512255059899,0.108041159019804,-0.098584
8874875317,0.921467869599741,0.951630324229648,-0.000528073216225052
"Tnfrsf11a",467.935888507396,-0.753258793437099,0.248695054464877,-3.0
288450852306,0.00245490530130077,0.0116631928764041,-3.35299390157851
"Htr4",50.4987685950815,-1.91982155771448,0.625062782087738,-3.0714059
6549708,0.00213053260431714,0.0104875346425084,-8.74971711279948
"Serpinb11",0.370843636912122,0.40318925314568,3.12692061325656,0.1289

41314159484,0.897404085543737,NA,NA
"Gin1",741.609322904377,0.401106377529156,0.16388535822181,2.447481470
46962,0.0143858522041398,0.0448936213630469,1.24481742092094
"Pam",6362.20922182882,0.198865138928653,0.238125789316966,0.835126424
14361,0.403646530236072,0.547499133672552,0.119795245971461
"Ccdc93",1013.68085365015,-0.0116034610341815,0.113956313655336,-0.101
823766160746,0.918896561024733,0.950333906903954,-0.000591102063786674
"Actr3",10353.8638843529,-0.730566011936503,0.201733498854119,-3.62144
123849655,0.00029296635368031,0.0022694707842903,-4.44783828356506
"Slc35f5",2158.02394663375,-0.56689400908337,0.202471631793787,-2.7998
6882142947,0.00511233771752556,0.0202194897788058,-2.21151492360161
"Gpr39",311.031495208827,-1.23833736602055,0.364534837190482,-3.397034
35634461,0.000681204137862837,0.00437163075242599,-6.7274153130674
"Lypd1",57.4328971701152,-1.31320923234364,0.590316027971397,-2.224586
78084084,0.0261089908701587,0.0708226355017186,-3.47682205928021
"Tmem163",72.6525775687063,0.8719350188452,0.551175229838189,1.5819561
0332703,0.113659591858643,0.217333272222541,1.33085472393422
"Acmsd",3308.30356241521,1.52737787066118,1.00931585292912,1.513280373
26333,0.130208455935233,0.240348809412589,2.17752770296151
"Ccnt2",4772.3188583718,-0.0372239543815091,0.175686763109964,-0.21187
6829663088,0.832203127464531,0.892673419561928,-0.00422620217661538
"Ubxn4",7228.05546272367,0.528719270997724,0.278017475804416,1.9017483
32431,0.0572040638878121,0.128943911871047,1.0830167964536
"Lct",17.3183709526539,-0.703166200466634,0.548540724555024,-1.2818851
3448485,0.199882943438766,0.331361807179863,-0.776678308348289
"Mcm6",1408.69817875128,-0.257348229636913,0.297805438628023,-0.864148
857799592,0.387506122401573,0.531859381570172,-0.162483533134283
"Dars",3085.64225144297,0.017683609539364,0.156183259703553,0.11322346
3083872,0.90985339442398,0.944789981598698,0.00100429847934781
"Rgs18",60.3052469151402,-1.4792006539153,0.406250684286537,-3.6411031
6887981,0.000271472315594081,0.00213692660858029,-9.09469754793145
"Rgs1",272.500227388108,-0.986984359834957,0.556685567173017,-1.772965
59860012,0.0762344074737666,0.160109419317592,-1.80805450381434
"Rgs2",3116.66278796197,-0.777723900893207,0.348984946322164,-2.228531
37102153,0.0258451017808823,0.0702828165304942,-2.06503423173941
"Cdc73",1695.61317194677,-0.538495921656307,0.234692093078552,-2.29447
832942575,0.0217630384308393,0.0614890194644987,-1.50180948024065
"Cfh",27212.4952584397,-1.18976276511711,0.403992518507796,-2.94501187
673393,0.00322942266172108,0.0142809391158345,-5.05509920560145
"F13b",2220.77214054876,1.78873279904243,1.00642246048159,1.7773180441
4072,0.0755159264734329,0.15905904011872,3.28854916189441
"Tsn",4978.51953465573,-0.358766695924775,0.190841148081999,-1.8799231
6924557,0.0601185497218398,0.133781269399273,-0.721676835286304
"Nifk",1136.79269140798,-0.287174706650113,0.19987083210844,-1.4368014
7633701,0.150774410803639,0.269163107658795,-0.376898921533998
"Tfcp2l1",12112.9489816524,0.991501057922562,0.457559133688725,2.16693
534216951,0.0302397846361779,0.0791111392507979,2.51534060954532
"Epb41l5",3163.59938252636,0.710994480791648,0.193102775022593,3.68194
85411769,0.000231458135741431,0.00189957556137266,4.45518015338448
"Ptpn4",1203.76970740676,-0.435334652243933,0.281799419498386,-1.54483

871194216,0.122385280961593,0.229957425863847,-0.639881467390508
"Dbi",22038.7906169097,-0.241531615333135,0.551361043815041,-0.4380643
4647958,0.661339638940148,0.770031274500517,-0.0631180437670541
"Sctr",139.843823788953,-1.13081260701846,0.69277925840927,-1.6322872
9559451,0.10261897829536,0.201249995263966,-1.81292712399879
"3110009E18Rik",91.1313199868124,0.500205946284076,0.217076400961344,2
.30428523814134,0.0212066370617667,0.0601873376172237,1.40572541314767
"Steap3",958.446946211458,-0.99105519366482,0.23824591660153\2,-4.15979
928555235,3.18527387304189e-05,0.000407926180399811,-7.7346152661303
"Marco",6.28670127324505,-1.75337378957397,0.811142927223395,-2.161608
92331996,0.0306483330714046,0.0799332795915258,-4.43000933733094
"Nek7",4312.67930986385,-0.0210744289372936,0.164123434556634,-0.12840
597075137,0.897827706298126,0.937244063465086,-0.0013658665603022
"Atp6v1g3",2746.66618129187,1.11106066620617,0.493298800242215,2.25230
76594969,0.0243028364658566,0.0668911215995584,3.00507359796468
"Ptprc",3902.05420514404,-1.21873978761484,0.388068028282368,-3.140531
29552858,0.00168641688363118,0.00882258019775709,-5.7651765528012
"Nr5a2",58.0829681557657,-0.557304238787982,0.313724084760636,-1.77641
521916684,0.0756645044499074,0.159268758260207,-1.02385828150907
"Cd55",955.819563928164,-0.54871183158632,0.277143361768443,-1.9798844
4711433,0.047716514416811,0.112337188855226,-1.19962141505232
"Cd55b",2.4290851871443,-0.271241668586209,1.17493117202528,-0.2308574
9620436,0.81742551192647,0.883000699808439,-0.0337504071131686
"Ddx59",321.059343967181,0.0233109047639696,0.24305266800166,0.0959088
61876021,0.923592956269935,0.953017329665072,0.00112177181527517
"C4bp",69.7588666894706,-1.0595252440308,0.454505954596303,-2.33115811
424711,0.0197450238634636,0.0568752364145094,-3.03754788404928
"Cacna1s",75.7098151684006,-1.16661358584395,0.655357728664528,-1.7801
172318838,0.0750567770500458,0.158314262107507,-2.15027092035291
"Pfkfb2",892.47049451512,0.143190368437702,0.118293483526657,1.2104670
8718688,0.226099712693488,0.362212817196421,0.145413162226884
"Tmem9",2519.34121696011,0.307243354723884,0.235956318712831,1.3021196
3129418,0.192875493244746,0.322999773880104,0.347216838400269
"Pkp1",155.053300874434,-1.88573648042536,0.590503275858229,-3.1934394
9055093,0.00140588798200791,0.00764630512501821,-9.19019847262163
"Tnnt2",157.538311873455,-1.61988505203351,0.56273654881237,-2.8785851
1314433,0.00399463472315822,0.0167716166735872,-6.62219911919802
"Fcamr",121.376689969383,1.1171088214632,0.662019139801797,1.687426774
09245,0.091521319173881,0.184499863653053,1.88803294125553
"Pigr",4736.46364022796,1.02351646319438,0.479282775948478,2.135516890
14671,0.0327188120407146,0.0840404949505802,2.53469401201195
"Tnni1",11.1875735546415,-0.980025596901491,0.694443705852418,-1.41123
836049248,0.158174342669979,0.279052056947091,-1.25086246269776
"Il24",2.01855924948381,-0.806025759507687,1.26294816340915,-0.6382096
92891858,0.523337188391356,0.656418687632979,-0.339301742561233
"Csrp1",5569.71520399625,-1.36508910289472,0.236753373080195,-5.765869
71131485,8.12378024192897e-09,6.81317024540035e-07,-19.3832252149554
"Gpr37l1",27.9319823696317,-1.18336088024663,0.398512201115745,-2.9694
4705063857,0.00298336222163699,0.0134839120531938,-5.09585726211152
"Srgap2",2300.98423011992,-0.539790093977503,0.141965826964349,-3.8022

5372203872,0.000143385773389655,0.001317500381504,-3.57989815278349
"Arl8a",3440.84798430198,0.248324776131207,0.134349210897879,1.8483530
6788637,0.0645512833780999,0.141037754740721,0.486400607752902
"Eif2d",1446.19208078658,0.382331590736396,0.106899327332992,3.5765574
9830334,0.000348148766887273,0.00259487945595109,2.27648457507064
"Ube2t",76.1041626656727,-0.509151248799051,0.32644457647974,-1.559686
65275299,0.11883394781583,0.224801039537503,-0.759928368990799
"Rassf5",717.547935176884,-1.11894409873945,0.275877129437186,-4.05595
092649468,4.99307428165052e-05,0.000578117353584453,-8.34254919855257
"Rab29",850.564444299394,-0.0113496128995073,0.169575857406609,-0.0669
294147945435,0.946637895914442,0.96717384691573,-0.000378816257230382
"Nucks1",7695.907256117,0.432833298099721,0.2517557003171,1.7192591768
7085,0.085567189753616,0.175048690149617,0.754294742584907
"Slc45a3",1254.7436430328,0.735350959252108,0.289836000219889,2.537127
75050105,0.0111766160498326,0.0369684202356855,2.42496041512627
"Elk4",3414.42109359141,-0.168570047324593,0.0940529244680206,-1.79228
926987708,0.0730866412063737,0.155301147585209,-0.313943028733742
"Cdk18",1981.40125738493,0.310351582003952,0.198979424031462,1.5597169
582463,0.11882678285367,0.224801039537503,0.463212006417094
"Rbbp5",1308.53824179328,-0.0414691175409543,0.091531414640661,-0.4530
5885092846,0.650506364830712,0.761913443952017,-0.0112763786650819
"Nfasc",111.906818631399,-1.60600363743984,0.683392520094531,-2.350045
67685009,0.0187711074156167,0.0547492972909042,-4.66542570358006
"Lrrn2",69.8719553718577,-1.53989005462183,0.443038103533463,-3.475750
82671307,0.000509425362773307,0.00351353934324511,-8.70212102429682
"Pik3c2b",478.230343870364,-0.841006732610299,0.234467474982488,-3.586
88015330533,0.000334658039832105,0.0025213147065867,-5.03172209165478
"Chit1",1.12132588271889,-1.28363812595777,1.65774914883528,-0.7743259
14665463,0.438738082486388,NA,NA
"Syt2",179.100966303977,0.168152127209047,0.709260703234429,0.23708084
5508888,0.812594071874129,0.88066028644332,0.0213693318283144
"Klhl12",1175.23773964954,0.0559523357827739,0.138898084205057,0.40283
0147751864,0.687073181193982,0.789937666544033,0.0131936301440893
"Cyb5r1",1867.66261244908,-0.222720759679503,0.0931801923511734,-2.390
21571065364,0.0168384796860761,0.0503707697022263,-0.665566298954956
"Adipor1",5854.98083260741,0.0879605776768431,0.195309259835428,0.4503
65629110267,0.652446823964113,0.763269225034636,0.023762062696196
"Ppfia4",662.608030621695,-1.05455357292611,0.29037552320956,-3.631688
92911492,0.000281572405886095,0.00219516144340947,-6.45544936409417
"Atp2b4",1878.73368344393,0.140099760960827,0.302050740822664,0.463828
562642328,0.642770577728004,0.7559862905381,0.0391903915414277
"Tor1aip1",3710.28772307369,-0.0896090214219726,0.145266694660537,-0.6
16858679350924,0.537327943565175,0.668761664983645,-0.0360521769675144
"Lhx4",1.33808274606045,1.59472444967812,1.76029392501733,0.9059421423
96714,0.364966476982853,0.509933096969665,1.07400823567064
"Xpr1",2896.75357425012,0.648116818394663,0.156785327420439,4.13378489
593391,3.56837471663781e-05,0.000447723103121004,4.99784628818119
"Stx6",2067.0011251021,-0.43527696832055,0.196895032777411,-2.21070568
505722,0.0270562245993718,0.072780934665807,-1.14055678180838
"Mr1",770.963725164124,-1.61584522522974,0.395048830786051,-4.09024176

078335,4.30923770974447e-05,0.000517373872318951,-12.2266884354431
"Glul",34888.7143166233,-0.445523877877787,0.479697202990071,-0.928760
632959139,0.353013150315564,0.497977890670877,-0.310619069084295
"Rgs16",532.647570397313,-1.18547345587824,0.413495492503416,-2.866956
1757519,0.0041444039766589,0.0172297630569073,-4.81434638137676
"Lamc1",3559.96938328298,-0.767165557506368,0.182944280259032,-4.19343
833226234,2.74757794649054e-05,0.000364189495585809,-6.07429124833247
"Lamc2",1747.37324378855,-0.797284473554208,0.292716441213396,-2.72374
339565358,0.00645466350379593,0.0241978521432442,-2.96708731501003
"Ncf2",1317.60702386375,-0.905524237092278,0.347235183289457,-2.607812
45873184,0.00911228635775146,0.0314629643002369,-3.13215777287059
"Rgl1",5149.42461583885,0.892842596178323,0.207042026580044,4.31237372
878565,1.61511125352714e-05,0.000243553280906977,7.42860649285514
"Fam129a",1410.8782407769,-1.07944142706782,0.21411421283383,-5.041428
18349732,4.62070265317154e-07,1.48916463357915e-05,-11.9976785866675
"Rnf2",1495.87901179167,-0.12843528952759,0.104042644826928,-1.2344485
258063,0.217035816329029,0.352354682565698,-0.133973032591997
"Coq8a",4759.22821904711,-0.157556659663105,0.559793806370629,-0.28145
4810449956,0.778361583696059,0.856799760566011,-0.0243505454995806
"Cdc42bpa",2961.1120971979,-0.0255483748297036,0.154759066156662,-0.16
5084834537843,0.868877209517806,0.917816026877906,-0.00219098557374685
"Ahctf1",2407.70626739618,0.143131498989493,0.0941915516912177,1.51957
894757602,0.128616832305105,0.23833596239165,0.205261160423522
"Tfb2m",1751.46563062284,0.0298581649328354,0.272332635398104,0.109638
585508446,0.912696008295832,0.946353538447837,0.00164635116135028
"Kif26b",502.227834408802,-0.446150145778065,0.329717605105624,-1.3531
28067381,0.176014720399032,0.302385494236688,-0.533619043910036
"Efcab2",739.23565366353,0.979986527567546,0.174024055350511,5.6313279
5402167,1.78827309459191e-08,1.26332423182439e-06,13.3099457718876
"Parp1",2210.65651238355,-0.263776210657083,0.157902408046721,-1.67050
150735532,0.0948201814468312,0.189601972098892,-0.438614544742436
"Mixl1",0.517852816592561,-0.798681168239809,2.63868532637244,-0.30268
1475603536,0.762132616295052,NA,NA
"Acbd3",2602.3913481574,0.0645332559029303,0.166280497366425,0.3880987
66391835,0.697942944823012,0.798479817999125,0.0145229244258819
"Cox20",2297.80898707459,0.876445976205393,0.20063434693773,4.36837455
591495,1.25174682137419e-05,0.00020170710349714,7.45741052746794
"Desi2",992.83690971471,-0.102868181089393,0.141000577733534,-0.729558
578715868,0.465660048958512,0.604449745995145,-0.0517876223092163
"Sdccag8",742.454613180162,0.96508849221518,0.200876320322348,4.804391
53139849,1.5522282122318e-06,3.89014725990946e-05,9.79997029850941
"Capn2",7049.99777014202,-0.599001158597082,0.196900159388745,-3.04215
679894123,0.00234889503224364,0.0113027530450855,-2.68514785508023
"Trp53bp2",3615.87005258357,0.960809109646745,0.239761672727655,4.0073
5071087917,6.14036499525979e-05,0.000677899121608382,7.01055526481373
"Srp9",4040.97581115213,0.428253442176341,0.226473975144951,1.89096094
552252,0.058629557786903,0.131253285919243,0.869622720841918
"Cnih3",1.00644585780926,-1.65026697090985,1.96876465815593,-0.8382246
01438955,0.401904572633354,NA,NA
"Nvl",1817.72234624971,0.108072402291709,0.132559302530126,0.815275882

031353,0.414914410849331,0.558277633202695,0.0629952833133798
"Tmem63a",1823.07163199266,-0.621816730683505,0.132534941649099,-4.691
71920209415,2.70918793972891e-06,6.07868198490612e-05,-6.0366823656055
1
"Pycr2",996.649106839888,-0.493081940520473,0.138771229738215,-3.55320
00505483,0.000380574930536408,0.0027802360418l299,-2.90189542568886
"Wdr64",0.761566859870984,-0.525980123356306,2.11453365531419,-0.24874
521246537,0.803557873262815,NA,NA
"Opn3",107.222400486405,-1.05348667527308,0.431933870625953,-2.4389999
1854396,0.0147279715767633,0.0456253558633192,-3.25242063425159
"Fh1",5856.41808548207,0.877211786602318,0.318765156874157,2.751906121
74914,0.0059249503169 6305,0.0226171891688538,3.32379500336078
"Rgs7",129.310571369846,-2.74347518843287,1.15574073657809,-2.37378081
5718,0.0176069988339796,0.0521179995847782,-8.10489760678831
"Spta1",4.62497493557629,0.174101263194063,0.624982994489257,0.2785696
0065984,0.780575144682676,0.858170519933624,0.026629216156745
"Ifi202b",2.43579839124074,-0.598895918381607,1.07549974773953,-0.5568
53611207587,0.577627451269881,0.702769252768011,-0.211246564910688
"Ifi211",968.078278021842,-1.13666532475961,0.349639350699165,-3.25096
509442271,0.0011501397751221,0.0065456582998 6303,-5.71623684060938
"Apcs",27.4187416530533,1.34348175768953,0.724423388007783,1.854553262
53919,0.0636600475317683,0.139543647538025,2.64582320349159
"Dusp23",1192.47117814898,0.402669709990351,0.183345850200266,2.196230
29128023,0.0280754642875178,0.0747505379201133,1.0443637050081
"Cfap45",77.2930469353887,-1.08314141192204,0.311099552444421,-3.48165
532033527,0.000498324684552361,0.00345186513418501,-6.14015598481715
"Tagln2",7226.83870954288,-0.634602777506493,0.259723373264761,-2.4433
7954466494,0.0145504259554549,0.0452695396967385,-1.96417231068179
"Slamf9",1078.76532643134,-0.546714768124043,0.408238266701858,-1.3392
0509838759,0.180503913137554,0.308211340603908,-0.643466640371043
"Copa",10068.8722378194,-0.0818759800713071,0.0968741523505538,-0.8451
78802442848,0.398011015693268,0.54245902306636,-0.0500788479672089
"Dcaf8",9243.81157935066,0.0924582267090613,0.158679446310855,0.582672
985434639,0.560113462340241,0.688466842649681,0.0345135577004572
"Vangl2",609.617014957821,-0.583980954552831,0.298882200765448,-1.9538
8334620542,0.0507150145192779,0.11771920870201,-1.24939985042226
"Uck2",1021.29400706778,-0.617882231958182,0.145003784981998,-4.261145
54206217,2.03381725131536e-05,0.000291607969668831,-5.02962329685523
"Tada1",1004.67068132461,-0.194993773058643,0.129473315595881,-1.50605
37544838,0.132053374928084,0.242880549414941,-0.275952364647976
"Dusp27",6.95760800756864,0.408303073017779,0.555803413771549,0.734617
785535235,0.462572303142832,0.601895504436738,0.207283804711454
"Pou2f1",1300.82178278856,0.139327984274873,0.401520238070431,0.347001
149791193,0.728590454972799,0.821808237234105,0.0273428660090741
"Mpzl1",2079.9545658423,-0.584056342544098,0.189233059175718,-3.086439
25690465,0.00202569330225405,0.0100915434173577,-2.68435652847021
"Adcy10",37.8569698752997,-0.671862017937239,0.775798520848929,-0.8660
2642294554,0.386475671886458,0.530818727137374,-0.425513527004675
"Mpc2",11209.2045719687,0.82399022639057,0.417715009661566,1.972613402
27676,0.048539624887455,0.113876261106437,1.79023647299944

"Dcaf6",2171.45735890054,-0.031436877944945,0.191623076195564,-0.16405
5804598719,0.869687213156731,0.918321253960533,-0.00267867348749954
"Tbx19",0.349997099048342,-0.292270863735201,3.88789699865886,-0.07517
45387894846,0.94007584212201,NA,NA
"Xcl1",92.6053698602858,-1.08151513803374,0.38097170071148,-2.83883326
770458,0.00452788091924425,0.0183964622136746,-4.32129853699407
"Dpt",1502.28908539464,-2.15409848821432,0.43393978592565,-4.964049294
57977,6.90384084336217e-07,2.03956195096781e-05,-23.2646738348008
"Nme7",827.151392714505,1.03913031087802,0.211867227562407,4.904629766
63975,9.36037232725381e-07,2.64122343902742e-05,10.9541825733034
"Atp1b1",58725.9546113048,1.74551685786069,0.568525208273946,3.0702541
1091289,0.00213876715792853,0.0105174481829232,7.95033996552332
"Blzf1",1054.2042121108,-0.038459303067727,0.154479151642001,-0.248961
122966643,0.803390854080187,0.874672783117734,-0.00514990931160253
"Ccdc181",1361.7768282018,0.921471583514839,0.17008020327189,5.4178650
1772799,6.0314923218195e-08,3.13605432775564e-06,11.6773902296934
"F5",134.337499143744,1.25343625858456,0.326154287747003,3.84307766500
02,0.000121500998142494,0.00115788194613018,8.47468666810865
"Selp",105.749241657992,-1.45073076333964,0.466124032623971,-3.1123277
535659,0.00185618296557267,0.0094274174167064l,-6.76640135608705
"Sell",123.659211921977,-1.47756324649002,0.508278162434399,-2.9069973
0126754,0.00364916345965212,0.015687908576838,-6.13907584426677
"Sele",44.5731232790272,-2.1892070946041,0.708320498424538,-3.09070131
313915,0.00199684391035394,0.00997552205075101,-10.0870365422985
"Scyl3",1336.65301141307,0.613240802097118,0.243134135506842,2.5222324
3280399,0.011661262701158,0.0382008232774158,2.00216879819239
"Kifap3",1694.60758046302,-0.11040628518508,0.0950255587777228,-1.1618
5883677185,0.245292809925318,0.384432055937936,-0.105547107302862
"Prrx1",992.809143909952,-1.66969957817135,0.299330174875616,-5.578119
80988948,2.43132180257498e-08,1.58548202363234e-06,-22.2982068553044
"Astn1",38.6378496844398,0.473400088500272,0.43251856132353,1.09451970
581711,0.273727109586989,0.416670528196218,0.414442590446653
"Sec16b",367.237213588168,-0.929723143178496,0.252854970161314,-3.6769
0278180161,0.000236082961935858,0.00192519681799471,-5.81330506807663
"Tex35",2.01894396557128,-0.573394079413245,1.11024941458098,-0.516455
196357525,0.605536523464556,0.726479887676096,-0.183224913064162
"Ralgps2",953.904398025057,1.05134459899609,0.287356604367787,3.658675
60729689,0.000253521997139819,0.00202672074630266,6.5197413078099
"Abl2",1373.26929231877,0.0104124734133397,0.128253307742538,0.0811867
825993396,0.935293410773421,0.959685105942958,0.000428473936648796
"Soat1",1707.12242863956,-0.293219263674646,0.35002467452254,-0.837710
267353633,0.402193445579657,0.546021988714117,-0.177425812861063
"Axdnd1",38.009867594409,0.761818248526446,0.324815156853637,2.3453900
8556711,0.0190071818841252,0.055264245806244,2.20594311156875
"Nphs2",4990.69838722391,-0.545311924748516,0.538181986351402,-1.01324
819220623,0.310941626684609,0.456141189673276,-0.428044171782126
"Smyd2",1116.4097166461,-0.0321504029017083,0.133239874994395,-0.24129
7155998239,0.809324814965617,0.878239940015561,-0.00417426178669526
"Ptpn14",3023.67293060907,-0.766594128108793,0.194379321665897,-3.9438
0493531318,8.01989002362358e-05,0.000833538463116272,-5.43502259182277

"Cenpf",229.371154406321,-0.883848864115677,0.428703368831852,-2.06167
930642585,0.0392382779917723,0.0965174553830399,-2.06646639909819
"Kctd3",1675.18404690318,-0.232283755971478,0.179568879140054,-1.29356
354555351,0.195816221195186,0.326522250993221,-0.259985262256576
"Ush2a",1.04072355285707,-1.11839839441349,1.8980429232938,-0.58923767
2493024,0.555701848896045,NA,NA
"Esrrg",1541.1489916343,1.20318499364328,0.366320007128098,3.284518918
51635,0.00102156639462115,0.00597256418660365,6.16100372950481
"Spata17",53.8427581572468,0.669897996656189,0.269289733724416,2.48764
773684891,0.0128591032010563,0.0410153434016352,2.1395262862910l
"Slc30a10",3.17230059117734,-2.41218629865144,1.37638972656848,-1.7525
4599194469,0.0796799687293671,0.165876571173838,-4.33351997782186
"Eprs",6411.97842218572,0.17041806905425,0.109227766500483,1.560208310
70913,0.118710662327725,0.224633200122864,0.254482989725875
"Cr2",118.975725088359,1.11192664051136,0.344683519632125,3.2259350307
7286,0.00125561855739048,0.00700287489473674,5.51675121237278
"Bpnt1",5638.22464371478,0.763541977135288,0.404517610892649,1.8875370
4801722,0.0590881281846018,0.132000495341816,1.54613402409492
"Iars2",3417.7399551707,0.291088468155276,0.226076171398714,1.28756810
748491,0.197896347106198,0.328941263316066,0.32365430348213
"Mark1",622.708422620372,-1.22163388616161,0.269638600337792,-4.530634
2809642,5.88068624967655e-06,0.000112303252199994,-11.1099145023472
"Marc1",264.420165319016,-3.37698630113035,1.06301672927897,-3.1767950
6645291,0.00148912221498492,0.00799234906571188,-16.308380531309
"Nek2",186.286504535572,0.250068044845325,0.386484201881898,0.64703303
1693598,0.517610565505281,0.651078692098701,0.107310391048022
"Lpgat1",4250.7039992134,0.35922894113322,0.155912248717601,2.30404566
727712,0.0212200801985304,0.0602167259075747,1.00936332029751
"Ppp2r5a",11525.1293433864,-1.38693775301178,0.510267424965631,-2.7180
6054071587,0.00656658218185608,0.0245184257720854,-5.14322339324263
"Pacc1",790.234908362742,0.768445651720529,0.261802937768593,2.9352063
7419186,0.00333325914931749,0.0146466718190058,3.24556259919465
"Atf3",737.007098708708,-1.07838560338747,0.245863996267788,-4.3861062
2033869,1.15397784719027e-05,0.000190680170038271,-9.23627891216008
"Batf3",501.634645561332,-1.02652128213302,0.379690250049682,-2.703575
56455217,0.0068597848746435,0.0253847192039854,-3.77103668349103
"Tatdn3",812.980218152877,0.231689474335819,0.151997059898453,1.524302
34170718,0.127433202474106,0.236637388746678,0.333916967272325
"Angel2",2922.55977048649,0.130880488487634,0.130645162964332,1.001801
25706887,0.316439589987048,0.461683562224443,0.101154329893547
"Traf5",1396.36169524002,0.0532828165985281,0.203464606024984,0.261877
569959197,0.793415832879854,0.867348167161437,0.00758293370333256
"Irf6",1841.64692083533,0.1499241615051,0.270755844101961,0.5537245631
84836,0.579767362168946,0.704405784370021,0.0525335297327447
"Lamb3",622.796866026878,-1.40421137828457,0.471097781308424,-2.980721
69727593,0.00287570002265235,0.0130852739887224,-6.08903658976902
"Plxna2",888.096703440522,-0.317758273704866,0.259953729281885,-1.2223
647438437,0.221569725566307,0.357392993803098,-0.326947614094662
"Usf1",2776.91262306498,-0.103631983985499,0.12679292968390 8,-0.817332
514075129,0.413738430627325,0.5571600733577,-0.0606146267729966

"Nmt2",1953.67016477173,-0.110590122427595,0.201868467877534,-0.547832
574301235,0.583806869495592,0.707770089002366,-0.0382239243563568
"Acbd7",3.35406070713459,0.491758376860515,0.965078047974157,0.5095529
60916258,0.610364685810105,0.730026416631421,0.154743850042731
"Olah",0.407128752309781,0,4.78471990595371,0,1,NA,NA
"Suv39h2",72.8904328152223,-0.2156072840682,0.243220559044657,-0.88646
8170762707,0.375365298477251,0.520471667594186,-0.140795831011191
"Dclre1c",575.693719973369,-0.258615368455686,0.238635438140203,-1.083
72574698543,0.278486416279527,0.422233914822567,-0.222977089149532
"Cfap126",231.573503984935,0.348855356045066,0.201244511898111,1.73349
003535405,0.0830086070930415,0.170908323241829,0.616297635626196
"Meig1",3.8667903337931,0.440976342378778,0.996512707019082,0.44251953
7656366,0.65811329542808,0.76718241266631,0.116872259765041
"Fam107b",4613.69099075963,0.613559761542391,0.141710918261181,4.32965
765144197,1.49341326120743e-05,0.000230733532226803,5.13810102519696
"Fcgr2b",3882.87524505502,-0.170253995797338,0.359930228155547,-0.4730
19442323031,0.63619930829634,0.750839181233704,-0.048788630239001
"Frmd4a",1098.094987123,-0.613100044562391,0.232780719721353,-2.633809
38634562,0.00844328733956309,0.0296679071088126,-2.1566955193964
"Dusp12",874.659845570721,0.110686238844727,0.120014572128526,0.922273
327993799,0.356386022764953,0.501508056195825,0.0763885146881163
"Sephs1",1891.52448817505,0.220197703055135,0.123648237640479,1.780839
80214409,0.0749386251032138,0.158099254735172,0.406161764366133
"Atf6",1933.22878398448,-0.122006484740383,0.138036032525424,-0.883874
177692781,0.376764138023183,0.522038226000312,-0.079305979770236
"Phyh",14511.4594292308,1.14622413705378,0.420975698451158,2.722779821
4266,0.00647351856779712,0.0242405732734604,4.26364140727318
"Uhmk1",5761.31768315705,0.155679600532949,0.24044183761165,0.64747301
0850945,0.517325856460862,0.650871298644513,0.0668555698416488
"Ucma",0.325902077166119,-1.56889382950155,4.76196181546753,-0.3294637
56808289,0.741805184033529,NA,NA
"Mcm10",260.805524137507,0.486907945643141,0.248738295266887,1.9575109
8607758,0.0502874214219254,0.116946160865753,1.04492471335859
"Uap1",2978.23994137746,0.247588617201401,0.139714091953161,1.77210912
471453,0.0763764445595802,0.160317355584314,0.453235711713018
"Optn",1539.19500847767,-1.04966480135772,0.333548475255204,-3.1469632
7289341,0.00164975682807206,0.0086610012476438,-4.98477935966513
"Ddr2",2824.83110148814,-1.32087833332135,0.24474040746333,-5.39705865
088608,6.77422487002035e-08,3.4070609385032e-06,-16.6294098221495
"Hsd17b7",785.023271438774,-0.206128028999946,0.223916254884534,-0.920
558577161981,0.357280937111488,0.502435175751878,-0.141875582982623
"Ccdc3",309.703766264702,-1.88547507817737,0.50349001376088,-3.7448112
7062199,0.000180529431293541,0.00156234480752839,-12.1831244956359
"Rgs5",8805.14963728918,-1.1596226734351,0.271212604696608,-4.27569608
99082,1.90541004376146e-05,0.00027849838855518,-9.49278517798868
"Enkur",75.8116003159668,-0.0775659101912062,0.425952574242185,-0.1820
9987421535,0.855504347444062,0.908085566250984,-0.00747864667784946
"Nuf2",124.603088211616,-1.21416802055576,0.437423360804708,-2.7757274
2873657,0.00550783624866457,0.0213627170063864,-4.66982142499524
"Lmx1a",20.2157371161072,1.19550390096866,0.452499983502989,2.64199766

752204,0.00824186234615549,0.0291122884103876,4.22801301319898
"Aldh9a1",11032.4057878013,1.45299138064307,0.343657160190351,4.22802591931523,2.35750638972412e-05,0.000326932144998868,11.6613570754369
"Mgst3",4452.48564276171,0.476986982843745,0.386279907212971,1.23482214305484,0.216896704474324,0.352266232255077,0.497672977035791
"Fmo3",17.6496727906529,1.30579314247435,0.576758972497735,2.26401877515564,0.0235729600394406,0.0653155570341073,3.56288927540411
"Fmo4",771.917023108626,1.59026185172119,0.373591000979134,4.25669207115085,2.07473800448777e-05,0.000295717232962268,12.9226377333569
"Eef1aknmt",669.82895167075,0.0816652730335808,0.12775288269098,0.639244072723745,0.522664165101269,0.655934181451545,0.0344378232838344
"Vamp4",1301.07487442669,-0.187709941861483,0.176013961719673,-1.06644916134799,0.286220648521249,0.430279892433872,-0.158299429802495
"Myoc",164.951671921487,-4.00554653066844,1.13489350684519,-3.52944704195477,0.000416429039851665,0.00299193069965884,-23.2795810527948
"Pigc",1078.50446829599,-0.147292395630415,0.102915911978708,-1.43119166704648,0.152375291115842,0.2713254270481,-0.192133553571375
"Tnfsf4",2.40077980423598,-4.21092338844899,1.45701057733634,-2.89011174932392,0.00385104902504112,0.0163127859852817,-17.3313440104303
"Prdx6",7240.60587571668,0.400238285311682,0.310405474959464,1.2894047225293,0.197257420477386,0.328307740885025,0.445786953527447
"Klhl20",1469.26048175492,0.232543356601575,0.124562709387831,1.86687779789328,0.0619186549835836,0.136727009604437,0.462707555542692
"Nsun6",223.062127565188,-0.396884557850492,0.205512128625891,-1.93119773759422,0.0534586036909389,0.122339889057034,-0.833835457977616
"Cenpl",258.463317592782,0.192138232009321,0.198974001257715,0.965644912374553,0.334221893473724,0.47898996537675,0.141428270346221
"Dars2",849.084896987138,0.480484293553216,0.180678107191136,2.65933876009073,0.00782941959265949,0.0280106464619587,1.71781332501842
"Mrc1",1996.12176436037,-1.10120341863338,0.307824290815096,-3.57737661221431,0.000347059956753606,0.00259072551178477,-6.55856639587099
"Serpinc1",162.287398708561,0.545870281227912,0.471054483742231,1.1588262081518,0.246527032367323,0.385669501917448,0.520091278216717
"Stam",1248.83190654375,-0.0886532010646175,0.111549194331919,-0.794745328243487,0.426761659201166,0.569775323959486,-0.0498685926606583
"Rabgap1l",2159.44075023871,-0.259308439575624,0.12920417742456,-2.0069663748065,0.044753243450545,0.106792160176077,-0.580039466759519
"Trdmt1",382.44784528247,0.0282691011230817,0.167966674245344,0.168301844696823,0.866345822705944,0.915953305016836,0.00248174135328153
"Tnn",131.370941668486,-0.445267260681946,1.43867929208838,-0.309497233421354,0.756943316342921,0.842236690922524,-0.0764498055006946
"Cubn",7113.74141329976,2.0593440990947,0.82522794153305,2.49548518106282,0.0125785009096458,0.0403248291246752,6.61211709972796
"Rsu1",2250.31936277884,-0.597006126155289,0.164949423083043,-3.61932836742768,0.000295368639806035,0.00228083078769825,-3.6317169332476
"Vim",13730.628078986,-1.11993890586613,0.269776670355801,-4.15135565424939,3.30511690880673e-05,0.000419551884686937,-8.70900707265464
"Pter",13108.3714984955,1.68403394717504,0.56677674691046,2.97124742035523,0.00296592729146418,0.0134169441350724,7.26026926549485
"4921504E06Rik",0.130737126706725,0,4.78471990596536,0,1,NA,NA

"Ptf1a",0.087583724926668,0,4.78471990595887,0,1,NA,NA
"Pip4k2a",1866.01815630635,-0.904319242089008,0.272258989243188,-3.321
54043693025,0.000895220093459512,0.00541408231460098,-4.71941774827988
"Bmi1",2745.17599865952,-0.245327960761197,0.104591284637749,-2.345587
03061051,0.0189971429084076,0.0552480488011951,-0.710450701347848
"Dnajc1",3280.71702351651,0.25613671686226,0.145438129579354,1.7611386
8902933,0.078214932125224,0.163437439350297,0.463946837597581
"Mllt10",2569.97649375443,0.181004275270845,0.155742988742119,1.162198
54731666,0.245154824616083,0.384344188693584,0.17307932945247
"Plxdc2",3056.39153634997,0.588017693349289,0.240193815382728,2.448096
7272714,0.0143613095464351,0.0448385610146342,1.82561075474272
"Nek6",1000.55613557612,-0.522674705055412,0.138457604561693,-3.774980
12268817,0.000160020422897934,0.00143122853470822,-3.42311272337489
"Psmb7",6326.21442818582,0.278873023831393,0.313799967742181,0.8886967
89352495,0.374166057064271,0.519296663885932,0.182739901783136
"Nr5a1",0.580960465210693,-1.53504002134171,2.61899672165099,-0.586117
58031298,0.557796502994913,NA,NA
"Ppp6c",2856.08437553978,-0.100648634811893,0.206383170744376,-0.48767
8498439947,0.625777583997036,0.742584829454945,-0.02995486214408
"Golga1",1361.4129746999,-0.0857936309830242,0.136667394087125,-0.6277
54934204213,0.530164492379451,0.662467782133718,-0.0353283890361664
"Arpc5l",3592.88466379208,0.313461436136496,0.188103404235478,1.666431
48969323,0.0956275031810885,0.190746297393848,0.519346361668661
"Orc4",1876.40615476256,0.0498843857101828,0.166860401613346,0.2989588
01656109,0.764971480253226,0.847825885870919,0.0082349137713462
"Kif5c",228.56390034463,-0.834944430234317,0.376059814849098,-2.220243
68801371,0.0264022301740252,0.0715284333817482,-2.20229972511718
"Lypd6b",281.240500167285,-0.153336225227936,0.272506300607976,-0.5626
88733749768,0.573646857258781,0.699642597594282,-0.0547694991526564
"Mmadhc",3029.35906195757,0.189819666362832,0.274262195626704,0.692110
212014759,0.488868119054869,0.625456075153007,0.0890774673534569
"Mindy3",1704.38991979086,0.490764416071926,0.104983537014322,4.674679
76436131,2.9441265493023e-06,6.52324766571549e-05,4.72976812184319
"Itga8",823.411007784307,-0.464464593918372,0.403467283779656,-1.15118
279124715,0.24965704332479,0.38924551713642,-0.438243238303501
"Il2ra",107.739454657086,-0.996170907182687,0.278371001300445,-3.57857
284892805,0.000345475576060849,0.00258293907517811,-5.93601044897897
"Spopl",1081.21695074968,0.460477320164453,0.264582220015438,1.7403940
4513872,0.0817898504444244,0.169160500457647,0.818225515320263
"Pfkfb3",3795.58005854121,-2.38370063036917,0.637907560291348,-3.73674
930154532,0.000186414617426792,0.00160260679479171,-15.3417921894132
"Potegl",2.03364252942864,1.05210875169089,1.37313418786159,0.76620971
2780775,0.443551530043636,0.584823352230918,0.564398941543134
"Yme1l1",4177.09523483896,-0.145612994163946,0.145792421250399,-0.9987
69297574498,0.3179064622737,0.46295766684686,-0.112139429756943
"Prkcq",486.767608248519,0.121777307962569,0.190417407274738,0.6395282
3276743,0.522479353130732,0.655806774001739,0.0513765170609713
"Mastl",105.2509235139,-0.145796854272276,0.228583687837179,-0.6378270
28042909,0.523586283420712,0.656564801396286,-0.061341676587581
"Acbd5",3838.87224619213,0.140060879800149,0.223903123184305,0.6255423

22984295,0.531615179959447,0.663914743299168,0.057369151555025
"Abi2",1153.67375475589,0.254310569504555,0.160255318946829,1.58690876
019493,0.112533310583049,0.21560500876514,0.390190539980413
"Pdss1",758.210855164222,1.46544132249631,0.221125129415417,6.62720391
106364,3.42104913327275e-11,1.09350190164839e-08,26.8634378381914
"Pkn3",748.972033721106,-0.791361353318331,0.240119251968703,-3.295701
39349541,0.000981763067075403,0.00578845366853226,-4.07700671567271
"Apbb1ip",572.379736216067,-1.22480668800259,0.334029151299484,-3.6667
6585932003,0.000245637518614555,0.00198031064414007,-7.62381113478382
"Gad2",7.77377335096777,0.210669501666411,0.680334044122063,0.30965597
486492,0.756822585147728,0.84217137114544,0.0361870703996617
"Zbtb43",1413.17091726168,0.225958452514343,0.17966607540223,1.2576578
6339171,0.208515517205038,0.34187988180826,0.242520264022445
"Odf2",2409.65700447742,0.188829953943154,0.196600084283387,0.96047748
2150857,0.336814958649218,0.481255903835735,0.138101943540429
"Slc2a8",875.719263698203,0.0125849431030407,0.168330996212158,0.07476
30762380751,0.940403220457113,0.963074845972624,0.000473497768545372
"Lrsam1",760.975075551706,-0.321285235571153,0.252251164563917,-1.2736
7196154111,0.202779697261858,0.334869021530592,-0.351491125343836
"Fam129b",5218.85252624242,-0.748075327695226,0.142122833597024,-5.263
58297792121,1.41274700993695e-07,6.00125080604588e-06,-8.9945156025613
6
"Stxbp1",982.100456115626,-0.428621475982356,0.236641226786088,-1.8112
7135708187,0.070098857195196,0.150262479946343,-0.81239699321912
"Coq4",926.51556255064,0.444853246836103,0.165925590900709,2.681040606
34207,0.00733936094269393,0.0266933146167965,1.61185551262454
"Med27",444.942555885073,-0.165620460866472,0.145152121617066,-1.14101
3021521,0.253864504250925,0.394347522616677,-0.15411360195707
"Ttf1",1459.96152723259,0.311357977536155,0.19331606341923,1.610616169
33993,0.107263407364534,0.20770899958225,0.489335558868981
"Barhl1",6.81488899786252,0.975064408199664,0.901182427898565,1.081983
37874095,0.279259913590739,0.422979087640195,0.838977144787588
"Ddx31",384.532567763541,-0.300256510597727,0.184987437037805,-1.62311
838796039,0.104564099536191,0.203981071811571,-0.4773262045027
"Ak8",11.5060384806998,-1.11835433314642,0.731030651028598,-1.52983234
228666,0.126058233823452,0.234979296662492,-1.61966546222194
"Spaca9",13.8176846769197,-0.696756653471336,0.623797057288629,-1.1169
6046868164,0.264011227990922,0.405905292806102,-0.628220474784507
"Dpm2",1601.72081973771,0.0126759249088885,0.104132253627401,0.1217290
94179069,0.903113570756599,0.940747323571544,0.000774254296953616
"St6galnac6",899.369508010792,-1.34533576082166,0.24740290722535,-5.43
783327330201,5.39324056445216e-08,2.89689819629995e-06,-17.15554670261
47
"Tsc1",2387.72341818995,0.365834624255947,0.24795863065209,1.475385725
81185,0.1401088707611,0.254503704313391,0.500622692184278
"Eng",6774.65782199668,-0.530567378620026,0.176286682674865,-3.0096849
6638274,0.0026151878828398,0.0122397823666852,-2.33612200900731
"Gfi1b",3.83356182372442,-3.55044484068352,1.5605116543274,-2.27517995
834117,0.0228951366265504,0.0638822801866743,-9.76625570723591
"Gtf3c5",970.945278018789,-0.318892962295484,0.104726237106132,-3.0450

1499440212,0.00232668709736801,0.0112235885760716,-1.43174572082887
"Ak1",1601.32917672725,-0.146015694297688,0.148096439113183,-0.9859500
68563733,0.324157615624964,0.469013445343985,-0.110551963581023
"Cel",1.98980616468468,3.81736690350092,1.58863964963548,2.40291554121
593,0.0162649434861734,0.0491375686540764,11.5022280481024
"Slc25a25",4015.44712227844,0.606901914567266,0.424467062620983,1.4297
9742837946,0.152775167393517,0.271938124323424,0.790295973700786
"Ptges2",1526.13117580339,-0.109069181282548,0.376791833341192,-0.2894
68007614124,0.772223258657553,0.852690514061897,-0.0173811139831726
"Ralgds",1247.79358863863,-0.603501602088897,0.201365481067715,-2.9970
4596283785,0.002726096079618,0.012604558065068,-2.63953301332542
"Lcn2",4005.00303027482,-0.857914405602587,0.59049806580816,-1.4528657
336556,0.146261036620921,0.2629407069231,-1.14602498749212
"Kcnj3",2.77863948279055,-0.0386840523793604,1.06414012114692,-0.03635
24047356348,0.971001364590548,0.982286220130046,-0.000691382527598577
"Dnm1",604.733752527075,-0.52450365041356,0.304469270018766,-1.7226817
3527408,0.0849460950506775,0.174161825583397,-0.916711944337523
"Nr4a2",273.473556083217,-1.99024715439057,0.50698877330773,-3.9256237
2023678,8.65052932300917e-05,0.000883078642617973,-13.995609647169
"Gpd2",3392.2334270149,-1.11228311809719,0.576803060761754,-1.92835855
730075,0.0538105428698682,0.122928876962705,-2.33151150935308
"Galnt5",7.13633199980767,-3.09559126866199,1.30088733513964,-2.379599
82009487,0.0173314482473739,0.0515213565981545,-9.18077725069131
"Gbgt1",50.0150932352195,-1.43713450091324,0.366307653375498,-3.923299
13849781,8.73445895431337e-05,0.000890016462292256,-10.0948216207853
"Ermn",0.103872140811095,0,4.78471990596623,0,1,NA,NA
"1700007K13Rik",30.0576133151579,-0.0981126160636567,0.393904780604229
,-0.249076987370291,0.803301230005445,0.874624044150445,-0.01314327875
81436
"Cytip",1354.76950977337,-0.328362716749271,0.377572841230776,-0.86966
7202966468,0.384482300407326,0.529161847486556,-0.208990044688759
"Olfm1",1374.42458831897,-0.179055563985707,0.173792704775802,-1.03028
239428516,0.302877461204572,0.447488424533359,-0.143979404190353
"Acvr1c",436.010629524444,-3.86427126292406,1.79875496689779,-2.148303
31759336,0.0316896675209942,0.081958500772562,-9.66663782179464
"Fcnb",0.725023989220248,-3.34468781683484,4.27030427423073,-0.7832434
41695348,0.433484168997292,NA,NA
"Acvr1",818.640717436571,-0.762604482742486,0.146818750604615,-5.19418
997643012,2.05613097663213e-07,7.91735059471335e-06,-8.95789842256218
"Col5a1",2686.27043724733,-1.74159907021867,0.314665600891658,-5.53476
155411828,3.11652040922666e-08,1.89893484184692e-06,-22.9442048418311
"Upp2",558.253530683196,0.447872466325573,0.188104765937373,2.38097351
810154,0.017266953205032,0.0514121255189118,1.32923228593022
"Lamc3",717.017140283671,-0.267604219942902,0.172851797424444,-1.54817
146208662,0.121581020309581,0.228887393453337,-0.394589146667956
"Fibcd1",2.04489837267094,-1.00944132940473,1.45245674725638,-0.694988
908490062,0.487062252520845,0.623856226874441,-0.476290095706369
"Abl1",3048.82081429582,-0.26757950026047,0.174994333433776,-1.5290752
2780863,0.126245795682699,0.235194011486839,-0.387279724609849
"Fubp3",1679.924039349,-0.183065363614417,0.0966009621705337,-1.895067

70430758,0.058083432602308,0.130398476554957,-0.372933493333768
"Tor1b",2491.40047225975,-0.266311309441,0.0882314425563054,-3.0183265
933916,0.00254174819200955,0.0119823516073023,-1.17824656207337
"Tor1a",1171.62409098106,0.237497859114116,0.0914722692574026,2.596391
9015259,0.00942085440870392,0.0323313009777889,0.815026021856648
"BC005624",1968.15680063955,-0.328694380617206,0.147396336032053,-2.23
000374002328,0.0257471945861679,0.0700654291753107,-0.873776742774546
"Crat",6446.3977601187,-0.66087584439657,0.567820070890564,-1.16388250
130021,0.244471631936733,0.383620732278136,-0.633185735242483
"Usp20",3238.97079957828,0.84080170800508,0.206859861021629,4.06459573
090967,4.81157874257906e-05,0.000562784417053428,6.29139456541908
"Dolpp1",822.092956787962,-0.0406790883006843,0.191659332332334,-0.212
246843426061,0.831914463363256,0.892654640167043,-0.00461933478280954
"Ntmt1",896.011075959716,-0.0151050069884115,0.130790338341622,-0.1154
90235593378,0.908056559557892,0.943527140861166,-0.000878056298404117
"Miga2",2042.75440432687,-0.905960712984807,0.45326701414571,-1.998735
14884445,0.0456370178331302,0.108330968311465,-2.01355586226195
"Sh3glb2",3056.4867927573,-0.599707733632559,0.336250251310362,-1.7835
1609045795,0.0745023310994257,0.157459776542534,-1.10861086492577
"Hspa5",23547.9152379939,-0.394635480294786,0.176419356183864,-2.23691
713217397,0.0252917566145174,0.0690863926127572,-1.05462286728704
"Kynu",251.727498431227,1.20698107432579,0.479220677724439,2.518633127
55429,0.0117811337960958,0.0385027729649695,3.93116755197372
"Gapvd1",3695.33073954762,0.034734143747353,0.139278693256825,0.249385
91061667,0.803062282630663,0.874461542875722,0.00465947984813978
"Psmd5",1888.52245824419,0.0938503446185082,0.259060417035219,0.362272
035583689,0.717148753299636,0.812537997085031,0.0194826370717071
"Cutal",1565.60096403784,1.64241834503595,0.498024964012617,3.29786348
821329,0.000974234865746379,0.00575276541863201,8.47171634386292
"Zeb2",4395.17348201512,-0.486813323959302,0.252875211785886,-1.925112
86701955,0.0542152395763309,0.123581354386139,-1.01785636446696
"Phf19",85.0932257379298,-0.953464500159456,0.326812926127479,-2.91746
263361548,0.00352891928220929,0.015314237294573,-3.98450177442592
"Hc",18.2194905600864,0.990484939212815,0.777438270591407,1.2740367649
5028,0.202650386982213,0.334683825629272,1.08415410637092
"Traf1",1523.09502571345,-0.911168911468239,0.453208638263317,-2.01048
443154087,0.0443799423284279,0.106200308728484,-2.04323091956237
"Rab14",10231.4470552097,0.338824618777007,0.157455847481526,2.1518706
6213442,0.0314075425003402,0.0814232500560607,0.849804135555007
"Gsn",18304.6556969936,-2.28460261099626,0.478202004295528,-4.77748439
043426,1.77501824734464e-06,4.3046400232246e-05,-22.9676399593744
"Stom",1625.6812236951,-2.00428958264487,0.449512890840498,-4.45880334
799132,8.24184928774308e-06,0.000146356627880159,-17.696803232603
"Dab2ip",2871.58364256436,-0.471982824009646,0.142575175839333,-3.3104
1376053795,0.000931581535618011,0.00556494386687499,-2.4501895107132
"Ttll11",55.6226653338065,-0.415597623930048,0.348234291346433,-1.1934
4255938483,0.23269609547247,0.369953397710553,-0.413261231093014
"Mrrf",1072.68719329918,0.0621230954178548,0.290301928109803,0.2139947
73725229,0.830551132747391,0.891990415109261,0.00710066308783845
"Grb14",899.272312935502,0.944899146099203,0.218803877934745,4.3184753

1688174,1.57110788783694e-05,0.00024000609213858,7.87558910546989
"Rbm18",1628.24733730944,-0.0727329962345959,0.108586785116405,-0.6698
14436044186,0.502976089792317,0.637813982750429,-0.0327086540004527
"Lhx6",65.8562905664407,-1.56665153439716,0.487276528432465,-3.2151179
9354869,0.00130390913786951,0.00720011905131115,-7.72932243439667
"Gca",876.49456844167,0.912914760565278,0.205959137299043,4.4325042944
7939,9.31448466812183e-06,0.000161722014300124,7.96940959469434
"Morn5",21.4323611904535,0.188855318028096,0.424041092292188,0.4453703
22501586,0.656052155556151,0.7656892142228,0.05042038830103
"Ndufa8",7394.13442399101,0.784684420694118,0.303128220590139,2.588622
13213429,0.00963607693210822,0.032852636478798,2.68026484256343
"Ifih1",974.403588028546,-0.810970715384744,0.171334154803937,-4.73326
941912289,2.20931917771308e-06,5.17134517731689e-05,-8.00411278275989
"Slc4a10",2.12828399449812,0.521591662955009,1.9028425110344,0.2741118
40538747,0.783998674778688,0.860390831646905,0.0784309755645302
"Psmd14",2312.70762826627,-0.23532549445554,0.229855877209296,-1.02379
58555276,0.305931708355619,0.450410901302331,-0.187694437479072
"Strbp",2782.32066585089,0.6214428625063,0.123710272190987,5.023373172
65701,5.07717568955184e-07,1.59411870523159e-05,6.86483347008812
"Wdr5",2515.89961845774,0.364980691480969,0.0654460321547332,5.5768192
4273194,2.44956165327247e-08,1.59205177051689e-06,4.87266995931282
"Brd3",5848.43628378819,0.220172831253669,0.209187008434684,1.05251675
475064,0.292562527576045,0.436482069223179,0.182525034765017
"Lcn4",1.01697395886539,0.96819323989293,1.83268843497727,0.5282912367
50747,0.597297212978061,NA,NA
"Egfl7",5057.37073929418,-0.253977585796737,0.162032971465951,-1.56744
385725288,0.117010979453581,0.222212120902214,-0.382013491136987
"Agpat2",9786.65557176608,-3.14507880440223,0.814427056670271,-3.86170
717026601,0.000112597475295289,0.00108954122744791,-21.4557230860116
"Notch1",2801.16356155227,-1.18856354322511,0.228898317876594,-5.19253
943956853,2.07444907140687e-07,7.96212755793131e-06,-13.95470385982
"Sec16a",3556.04983213719,-0.0396571625942362,0.135437543606062,-0.292
807751369031,0.769669103073574,0.850978631796345,-0.0063994073341453
"Inpp5e",1585.49249215802,0.43030961749548,0.231859935920041,1.8559032
8828468,0.0634673442129801,0.139246796158961,0.848358598744121
"Pmpca",4441.73694774988,0.130220366338972,0.164671063540296,0.7907908
2590067,0.429066064311895,0.571678782997034,0.0728163657702906
"Entr1",5060.271804312,0.19326735935712,0.140846701842567,1.3721823573
3447,0.170006676233283,0.294412485406923,0.236322202220203
"Card9",376.895148658626,-0.622171213241934,0.437259563905099,-1.42288
760407072,0.154768740242242,0.27453428832271,-0.804267732634486
"Gpsm1",1183.22830220236,-0.286053109003317,0.27459222062996,-1.041737
84802448,0.297533236473276,0.441802074842429,-0.233674865805718
"1700019N19Rik",0.705558652691576,-2.71174956349691,2.47805618981787,-
1.09430511488774,0.273821182762988,NA,NA
"Nacc2",3270.96230562919,0.263737261421525,0.146672048187459,1.7981426
2281554,0.0721544093082558,0.153504274161743,0.494250713901899
"Camsap1",1504.45439998116,0.0561401907428296,0.152858142308251,0.3672
69874506379,0.713417722968161,0.809958004101153,0.0118328296556511
"Lhx3",2.50346479342393,0.70151621616171,1.48078588781479,0.4737458817

87775,0.635681130607049,0.750318462653687,0.201515826526472
"Fcna",18.488032918377,-1.67296323799461,1.08264678568075,-1.545253041
08549,0.122285069159491,0.229863271552888,-2.45970869438035
"Tmem141",585.854266805766,-0.0297025888661055,0.236088929546637,-0.12
5811019276268,0.899881518965676,0.938676966139543,-0.00187969500957331
"Ccdc183",0.385352917787171,1.61481522965431,3.81543357659935,0.423232
431448478,0.672125686904567,NA,NA
"Mamdc4",243.401789722315,-0.487140609239357,0.28523739831021,-1.70784
270269345,0.0876655331327114,0.178386825821499,-0.839733424459862
"Traf2",1880.36722577831,-0.14319758413708,0.181798805661944,-0.787670
653917039,0.430889375235627,0.57330895304314,-0.0796651868049902
"Lcn12",1.03371941091515,3.38060208437278,1.62095792351461,2.085558196
99679,0.0370186613236379,NA,NA
"Abca2",3548.36731383004,0.0881420940569223,0.183735979477271,0.479721
469402381,0.631425459645092,0.746787189867767,0.0257352897478896
"Nmi",955.284120918999,-0.185254334930937,0.136413314645657,-1.3580370
4654599,0.174451929180959,0.300535758541291,-0.222710637564304
"Neb",42.3435254429911,-0.498980096190314,0.531675181944624,-0.9385055
25808585,0.347984678552151,0.492817046240772,-0.35308693683116
"Sapcd2",39.3585145251684,-0.674628481040943,0.58833653700764,-1.14667
106087308,0.251517595166794,0.391512858898463,-0.632624033202639
"Uap1l1",3378.74519022394,0.846095089028285,0.323630653165443,2.614384
88830585,0.00893882760782825,0.0310233643106862,2.9385006572015
"Dpp7",2953.17049684332,1.1144337534713,0.247286327003672,4.5066533478
608,6.5858094525077e-06,0.000122473077570503,10.0383950165052
"Grin1",11.0305928219478,-2.14908489257832,0.818441764555268,-2.625825
05640595,0.0086439231135727,0.0301825237944915,-7.52285491307314
"Arl6ip6",849.686443805235,-0.00673402539034785,0.192545752402035,-0.0
349736377268256,0.972100762055521,0.982990387851807,-0.000115528517127
241
"Lrrc26",88.0906481004942,-1.10009298736814,0.600445095884187,-1.83212
91903437,0.0669321753434988,0.144956520587309,-2.12463317199888
"Tmem210",0.322228676776401,-0.948735596185645,4.77597479756428,-0.198
647529854951,0.842538471007386,NA,NA
"Anapc2",4357.48365618898,-0.19430494021931,0.0771666078562066,-2.5179
9250501435,0.01180258325546,0.0385375074765853,-0.632680841552486
"Ssna1",1587.42919721731,0.0811557712738249,0.149341668938269,0.543423
492256346,0.586838254516998,0.710252780047947,0.0277661765725284
"Fam166a",9.4558215172073,1.63310563715639,0.791744753036511,2.0626668
2651585,0.0391442937861975,0.0963681111418282,3.82078117124038
"Rbms1",8304.01799815732,-0.163133436021475,0.191091163670037,-0.85369
4293803993,0.393274396650009,0.537881580771407,-0.101161792987301
"Itgb6",4215.3616181926,0.472708820860638,0.360391968804577,1.31165192
839512,0.189637603402343,0.31904364758945,0.540035489913754
"Arrdc1",2594.57689551536,0.0753894497130003,0.1633753165513,0.4614494
48450361,0.644476181672093,0.75738382711807,0.0209496060465618
"Zmynd19",672.364444051699,-0.136489543279281,0.103584779010384,-1.317
66022559742,0.18761741490321,0.316504962431457,-0.157019803343464
"Dph7",590.109079564542,-0.171469336918397,0.154880685854544,-1.107106
00209656,0.26824810083041,0.410445065525491,-0.152695704897871

"Pax8",3641.32570928672,0.520155523421388,0.559027875883727,0.93046437
5500257,0.352130700843251,0.497200695563886,0.363464664382377
"March7",2917.91391603592,-0.276634647740391,0.164670963866847,-1.6799
2365651104,0.0929721704157247,0.186671957446792,-0.4643042679933
"Psd4",808.516165972504,-1.4587145426562,0.393842742298267,-3.70379947
62678,0.000212394172482439,0.00177204175227326,-9.2418652097358
"Ly75",231.558109107977,-0.846667257811883,0.276892259201586,-3.057749
82750775,0.00223005665990464,0.0108704111887052,-3.82838444192783
"Il1rn",258.384279543384,-1.49750459293617,0.471390404209632,-3.176782
08882294,0.00148918884786805,0.00799234906571188,-7.2318548466754
"Il1f5",18.2506990082279,1.06699087766225,0.699713737723534,1.52489628
277648,0.127284969195465,0.236428590490624,1.53871721118867
"Il1f6",112.30037850731,-1.0093308438668,0.659567788733283,-1.53029129
243143,0.125944642666018,0.234869431232412,-1.4622433627602
"Il1f8",0.24961578387899,-0.923986736915312,4.78284172616633,-0.193187
813817944,0.846811882131672,NA,NA
"Hnmt",626.076225545473,-0.0388595776125521,0.43645508211235,-0.089034
5403345516,0.929054459896007,0.956274289133313,-0.00173743090248372
"Baz2b",3792.06527096825,0.832733339079355,0.224326721612844,3.7121450
9396047,0.000205510114750652,0.00172964263644837,5.29605118254893
"Wdsub1",947.479260262215,-0.361881903796233,0.120029621944759,-3.0149
3829550493,0.00257031541026508,0.0120819695924177,-1.59808534387688
"Dapl1",22.082016987871,-0.875648610717779,0.451669034232619,-1.938695
24884631,0.0525384575374273,0.12074809416459,-1.85116386834384
"Pkp4",13626.2496061449,1.16957915451038,0.213156160394042,5.486959196
24509,4.08911539506228e-08,2.29768218942145e-06,15.185359240067
"Galnt3",1934.5136829252,0.652751736997213,0.454370517532789,1.4366067
1590583,0.150829774250074,0.269197728115129,0.856612057975829
"Nup35",627.221678791397,-0.106951517003949,0.106690106296006,-1.00245
01869669,0.316126213481247,0.461371633463077,-0.0827324972512931
"Dusp19",347.57527283724,-0.27342481704518,0.261701090899709,-1.044798
15542673,0.296116267606398,0.440469559489591,-0.224184819158733
"Nckap1",6642.14176836062,0.0190974692991004,0.15853404292146,0.120462
892052539,0.904116473391451,0.941409020842224,0.00115305874535147
"Frzb",1245.58740253203,-0.389405367348703,0.484905793870168,-0.803053
649330009,0.421943726367355,0.565161693804402,-0.222211604770106
"Dnajc10",2979.83165720641,-0.221101528766634,0.124177322314491,-1.780
53065282462,0.0749891574221761,0.158188747313382,-0.40770378023434
"Itprid2",4508.83684551161,-0.0522235598064087,0.0760998614454525,-0.6
86250392766377,0.492555225206797,0.628953179092865,-0.0242159843679606
"Itga4",794.328698802936,-1.17574935855909,0.378533869383755,-3.106061
18409744,0.00189597481312518,0.00958209354751548,-5.46471744688983
"Slc25a12",1832.44408790019,0.710047176366974,0.210541989922274,3.3724
7299994222,0.000744963902150935,0.0046855826335932,3.80817117612551
"Ube2e3",2059.34358032904,-0.0319820446360519,0.180912320391481,-0.176
782015546786,0.859679616066957,0.911029575198301,-0.00298008428727451
"Dync1i2",7530.27247869937,0.159922313726562,0.196069987245368,0.81563
8925535453,0.414706679442918,0.558036634629263,0.0932875896621847
"Cwc22",964.129303508573,-0.209269220531477,0.112159287516878,-1.86582
159324065,0.0620663302053693,0.136943454378668,-0.416066381453736

"Cybrd1",68.9066955696977,-0.749415717041673,0.350375278118846,-2.1388
943907951,0.0324442224377677,0.0835001912739696,-1.86072904744512
"Zfp385b",221.514702163624,0.279193563534932,0.369764777762056,0.75505
7215629645,0.450214652836171,0.590891579227225,0.14689008026138
"Hat1",1111.76489715549,0.0594336421340358,0.195375435626363,0.3042022
24519652,0.76097383018173,0.84523772814549,0.00999301545233552
"Xirp2",52.8210010682174,-1.43996086973532,0.67728128000746,-2.1260898
7172871,0.0334957770976731,0.0855291594880081,-3.54071677961028
"Stk39",1682.88718623222,0.586996717934888,0.172919704602965,3.3946201
7520023,0.00068723871167901,0.0044020303549057,3.18486187044858
"Cers6",2463.99249802339,0.409221548800883,0.139155331636278,2.9407536
4550529,0.00327414817522606,0.0144334933575758,1.73436434181936
"Abcb11",10.5786929073792,2.21754108362463,1.07751980012172,2.05800495
116111,0.0395896581228397,0.0971211819425174,5.17085294482214
"Ssrp1",4155.87550767708,0.0438189748415098,0.177245908553228,0.247221
361548951,0.804736914184593,0.875649330474424,0.00581870308416814
"Dhrs9",30.6268333006899,-2.5770387272441,0.718778453296487,-3.5853032
5363148,0.000336686747533217,0.00253170775295127,-15.4077568643994
"Lrp2",34964.4815468399,2.03131373122158,0.793835321739575,2.558860352
5102,0.0105015917482864,0.0352548271191614,6.79505485538354
"P2rx3",31.0721514198191,0.453183769469407,0.453297091797252,0.9997500
04291014,0.317431506271225,0.462473250338216,0.349480168916112
"Prg3",1.44023577335418,-1.54645096509672,1.613515476192,-0.9584357807
00684,0.337843059868168,0.482406736090044,-1.1273127567405
"Prg2",20.8929804969638,-1.88915089247315,0.781548642440452,-2.4171891
4202718,0.0156408873457689,0.0476434965580068,-5.75059258686024
"Slc43a3",2988.19819639722,-0.838559421498577,0.15962800627204,-5.2532
0989143655,1.49471033252737e-07,6.24065568814102e-06,-10.0496527489722
"Slc43a1",352.809032449076,-1.87202208920372,0.465080680276203,-4.0251
5556675448,5.69376624563e-05,0.000639499160468282,-13.7683953297469
"Timm10",912.629335124102,0.317927426517844,0.313289129644452,1.014805
16377525,0.31019871177908,0.455337987071332,0.250118372976954
"Ube2l6",1381.45839698124,-0.903554971654283,0.213962865990311,-4.2229
5227478959,2.41122892347034e-05,0.000332255417313249,-7.23711982428278
"Clp1",624.223616661455,-0.00173574503369075,0.16285366811288,-0.01065
83109475166,0.991496059258412,0.994470947578839,-9.62365471134927e-06
"Med19",1172.58012702661,0.0493154864341465,0.158178680789755,0.311770
753099747,0.755214752588964,0.841054217842108,0.00853646892093245
"Tfpi",743.888336364851,-0.620937992634364,0.269932755972854,-2.300343
24806734,0.0214287813159778,0.0607117666519813,-1.73963090206947
"Fastkd1",1151.11543261063,0.771405363316327,0.177810482906549,4.33835
705694444,1.43551803072607e-05,0.000224581036196255,6.48078778595772
"Itgav",7229.47568931672,0.842225860932365,0.384003395823825,2.1932771
1705645,0.0282874192905528,0.0752145235684165,2.17918440157108
"Phospho2",1020.34163750344,0.295975113811132,0.164205748554253,1.8024
649953917,0.0714722772810899,0.152435622667544,0.556733012634992
"Zc3h15",4662.64224148919,0.232751022298272,0.102414686754351,2.272633
24894546,0.0230482902550033,0.0642268920097262,0.638979126849238
"Mtx2",2891.70227625376,0.34924022634312,0.189422264044234,1.843712660
20537,0.06522502699204,0.142124751804153,0.681385169182928

"Hoxd8",4376.53912741206,0.989832076237262,0.237787487584693,4.1626751
9494571,3.14540639179589e-05,0.000404545737646677,7.73330635257918
"Atf2",2406.02421458134,-0.000762989022588981,0.104256033926413,-0.007
31841595976618,0.994160801020032,0.996250456664318,-2.866237309713e-06
"Chrna1",4.71419905194103,0.214972387230912,0.859237102516912,0.250189
833052142,0.802440547340972,0.874030362654743,0.0289439174830866
"Ola1",1692.08498145203,-0.0379730990254316,0.248815880540918,-0.15261
5254873922,0.878701694281953,0.924405181564126,-0.00298486767469134
"Sp3",2516.57208776131,-0.500129940475887,0.109205451434188,-4.5797158
8329806,4.65607980851406e-06,9.38823620541957e-05,-4.63793901231966
"Itga6",5097.28631764884,-0.124980718397733,0.139700396838321,-0.89463
3953992101,0.370982795039891,0.516098107609161,-0.0826695460906645
"Kif18a",128.024531900536,-0.0183650833891582,0.274255375456932,-0.066
9634400367192,0.946610808467683,0.96717384691573,-0.000612971756376534
"Arl14ep",1172.74319770575,-0.224085201044909,0.175034201476224,-1.280
23665749318,0.200461917290584,0.331997491151942,-0.247082587224265
"Slc12a6",3426.22122069827,0.533024863528591,0.142601807890402,3.73785
488006052,0.000185597015850076,0.00159682282172788,3.43254119370757
"Emc4",1994.05517952859,0.0912976249433339,0.260410594565162,0.3505910
54468365,0.725895168584878,0.819590248252241,0.0181637319478703
"Katnbl1",789.764424900812,-0.0694752461342741,0.0882517852382843,-0.7
87238988386325,0.431141977746666,0.573385600034411,-0.0386419122988508
"Nop10",2638.32290503208,0.194208269346432,0.3421866549498,0.567550681
878355,0.570340103264026,0.697122074563815,0.0700693221827996
"Lpcat4",1445.98204237374,-0.399580438229112,0.304936098722789,-1.3103
7433712419,0.190069230814215,0.319590364126903,-0.455807485638254
"Ccdc34",1633.86547930019,0.674202765812023,0.234459687476941,2.875559
43227269,0.004033122104062,0.016896823116674,2.75117180032012
"Lin7c",3478.29785632458,0.0936713227530399,0.0841865208308837,1.11266
413944353,0.265852702455229,0.407616733903639,0.0840632606938194
"Commd9",1552.98221631089,0.583015499539803,0.158067233527988,3.688401
99532292,0.000225666916235366,0.00186206243451425,3.66487657339498
"Traf6",1219.03194065472,0.0051847174366989,0.114842686910306,0.045146
2568160586,0.963990731379284,0.978290286807541,0.000113798513824009
"B230118H07Rik",1447.49462676933,0.783173160945444,0.247271419508314,3
.16726115174467,0.00153882060278257,0.00821800167435073,3.760349704189
43
"Dnajc24",385.349381324066,-0.295852865732492,0.280885814665812,-1.053
28517954703,0.29221030928541,0.436190216693227,-0.245462274874457
"Elp4",355.183298156619,-0.314256802992522,0.21814709376028,-1.4405729
5275203,0.149705366176734,0.267597655822695,-0.414275539045671
"Pax6",14.3970747846369,-0.104225471609919,0.776426100493981,-0.134237
46515426,0.893214801090498,0.93382853574598,-0.00713552990666968
"Eif3m",4008.99229339667,0.26058025004082,0.205734822936023,1.26658310
111095,0.205304398052304,0.338093340643905,0.282581891573483
"Prrg4",73.1094590325114,-0.328506587177729,0.39179248536021,-0.838470
872854294,0.401766299711799,0.545746085535784,-0.198944067569528
"Depdc7",783.377856433137,1.21040721611876,0.330663562251884,3.6605400
6034306,0.000251684198048057,0.00202114435483568,7.50947701900153
"Tcp11l1",276.8076806618,0.938836463371035,0.192411997403642,4.8793031

4138127,1.06461325171533e-06,2.92776152072573e-05,9.80022028577096
"Cstf3",1335.65525676516,-0.0386172275419315,0.12888908308211,-0.29961
5969161097,0.764470101855066,0.847537274462391,-0.00638807950638115
"Hipk3",4190.60320037556,-0.333961778929993,0.231324160386777,-1.44369
605998615,0.148824490224611,0.266486554289164,-0.441641572779108
"Fbxo3",3781.82730151153,0.237760518969751,0.121389126642982,1.9586640
5455762,0.0501521426877909,0.116662667397584,0.510821031486356
"Caprin1",10574.5311784149,-0.200572986204697,0.184482309836044,-1.087
22070090598,0.276939284318294,0.420476729920425,-0.173769643880919
"Nat10",1562.14066119918,0.119988675839997,0.164595331228717,0.7289919
76529784,0.466006570460483,0.604786870054429,0.0603398049861126
"Elf5",167.199670957148,0.407359950067317,0.843484136849602,0.48294915
3719475,0.629131839489754,0.745204580910712,0.119803132723992
"Cat",25998.1118461842,1.13929955354359,0.427963376861427,2.6621426391
644,0.00776449676976436,0.0278343372940074,4.08038410885758
"Pamr1",1095.31973002516,0.0399274212641117,0.275911670934312,0.144710
882033031,0.88493914698352,0.928212571299406,0.00297437362381425
"Trim44",5156.64917564267,-0.1365951364198,0.170152978359555,-0.802778
404096794,0.422102826541624,0.565297129741644,-0.0779143838811098
"Api5",3649.10388329872,-0.111167773990278,0.0935909651586198,-1.18780
454717898,0.234910422506511,0.372896150617271,-0.109662041473754
"Ttc17",1090.14491558977,-0.0206776974678705,0.126451155074956,-0.1635
23199575468,0.870106508370901,0.918485014358499,-0.00175821820151089
"Hsd17b12",4953.40884259416,-1.49468040525049,0.573696991664563,-2.605
3481663094,0.00917809424158275,0.0316611327503014,-5.16063138888081
"Ext2",2137.14679953348,-0.259879792032741,0.179863772450694,-1.444870
12860793,0.148494368208659,0.265944997826071,-0.344201887997026
"Gatm",18026.8750825288,1.48795387049937,0.641492939692314,2.319517142
64081,0.0203670127203353,0.0582708751314889,4.22973635435961
"Sema6d",907.520186650449,-0.94676512345997,0.215757376598216,-4.38810
083060585,1.14344764905838e-05,0.000189494617453548,-8.11486582904022
"Myef2",2269.5263040012,0.116443200667305,0.177814931227561,0.65485614
6575707,0.512560377120324,0.646687086391359,0.0507567456998023
"Slc12a1",28975.233607059,2.00181787477264,0.640004081749781,3.1278204
8092512,0.00176107709064945,0.00907454764381448,9.41311165233122
"Dut",1083.1065875517,0.277700757204174,0.235656646487921,1.1784125817
9157,0.238632164202754,0.376719660591891,0.271106467804259
"Fbn1",1628.39264257745,-1.63409963559435,0.31130967046224,-5.24911299
147245,1.52833315991225e-07,6.34030637275935e-06,-19.5578578818478
"Cops2",3758.06634900666,-0.0775648563410019,0.181987276313522,-0.4262
10325865724,0.669954593965851,0.776621562018202,-0.019608558369984
"Galk2",2205.81125353011,0.482872808361617,0.229218546314121,2.1066044
4421407,0.0351518811957654,0.0886846337500367,1.16984081143197
"Fgf7",73.4257486097854,-1.51453789398415,0.450624079547385,-3.3609786
13272,0.000776668391513757,0.00484281429412704,-8.0728796363177
"Fam227b",0.346678464072157,3.05774640292831,4.63393282306745,0.659859
890006826,0.509343745950438,NA,NA
"Meis2",2503.63504901005,-0.333624558178573,0.241159874225267,-1.38341
653747478,0.166537182649934,0.289587263650086,-0.413460447391402
"Cd82",5952.13234934955,0.0139290111411907,0.190639997595649,0.0730644

739659222,0.941754811737815,0.963817152236969,0.000513335297934348
"Tspan18",1087.19770757328,-1.77884839416374,0.564112991206612,-3.1533
5477447321,0.00161405516016306,0.00851944978691371,-8.47693039771911
"Slc28a2",522.671708299594,-2.62625034795367,0.508971133010593,-5.1599
2003793859,2.47055338975798e-07,9.10602079272232e-06,-30.4817709432912
"Syt13",225.066357601422,-0.974829201416998,0.373396531906605,-2.61070
770111176,0.00903550854485906,0.0312643026810403,-3.37805449904276
"Chst1",1081.43824200558,-1.5023561999545,0.390445481601497,-3.8478001
9426081,0.000119183192315325,0.00114081192133737,-10.1799882339403
"Pex16",2202.73212125783,-0.832984205169298,0.522599251845717,-1.59392
536867851,0.110952751642011,0.213243642337697,-1.28722707383378
"Mapk8ip1",1449.4820663919,-0.388316372225892,0.344354265274449,-1.127
66534753506,0.259461277462175,0.400742711340105,-0.355090344029911
"Duoxa1",40.3562935623838,-4.36294005211698,1.21014304688889,-3.605309
35853699,0.000311781299873149,0.00238023170905507,-26.3545899889993
"Duoxa2",1.29500572304148,-3.52149588618111,2.26526281155713,-1.554563
94207983,0.120049957000213,NA,NA
"Sord",33641.5880390637,2.05102525825727,0.595726435532101,3.442897840
22309,0.000575516673507739,0.00384032310063446,11.4082101792432
"Terb2",0.100532412451372,0,4.78471990596383,0,1,NA,NA
"Creb3l1",535.13298012838,-1.29176255448112,0.246217798427524,-5.24642
232499433,1.55081186747944e-07,6.37221589562125e-06,-15.4540830691678
"Patl2",2.634769017546,-0.0357985970667125,0.822545796880712,-0.043521
706879385,0.965285661330556,0.97904389433121,-0.000758171383829531
"Eif3j1",2922.15193163648,0.081060324365436,0.127792667115435,0.634311
233931867,0.52587772651526,0.658546172870555,0.0338605707348626
"Frmd5",18.8839454643706,-0.796349585311385,0.462378019970706,-1.72229
11793295,0.085016784765434,0.174251789063012,-1.39142496821144
"Mdk",3480.66616976706,-0.0362157421810254,0.369243491184003,-0.098080
9223336539,0.921868036248935,0.951892335475387,-0.00178555717954954
"Wdr76",288.111295318127,-0.659060752772643,0.215921044419723,-3.05232
29199073,0.0022707766854283,0.0110220572099778,-2.9709515045727
"Harbi1",546.576129714143,0.256291926904241,0.172831301549832,1.482902
25558676,0.138100382085767,0.251628936539416,0.353631536842728
"Atg13",1881.49867465435,0.00995632334969941,0.125823965002332,0.07912
89906458987,0.936930024603118,0.960836919719902,0.000397760921707279
"Hypk",2072.08239950207,0.185095459365594,0.193589577989733,0.95612305
8315725,0.3390100665016,0.483577311921004,0.134480009554139
"Ell3",372.782596625573,1.55093520826878,0.379341049113978,4.088498231
05428,4.34174857211985e-05,0.000519979357567822,11.7277400228714
"Arhgap1",3146.66625930985,-0.411367344850665,0.131026282124344,-3.139
57885533436,0.00169190872366672,0.00883708446130557,-1.94527318817152
"Pdia3",22949.2983476041,0.109560857192568,0.178876409753171,0.6124947
23836135,0.540210487757115,0.670935988934851,0.0437237159497237
"F2",29.1571049302679,1.08420543325515,0.543375541619095,1.99531511857
258,0.0460085220045735,0.109000505716303,2.4030359115996
"Lrp4",886.817505221699,0.10562655509745,0.220954014719191,0.478047684
40931,0.632616266210023,0.747878006339843,0.0306861416903384
"Map1a",307.964328400875,-1.34839926731635,0.31194054535871,-4.3226162
4011007,1.54189799090625e-05,0.000237097216298817,-11.2551430323076

"Arfgap2",3769.30662068227,-0.408312910939595,0.157797765800905,-2.587
57092578083,0.00966552949053311,0.0329357731573601,-1.39365194615174
"Pacsin3",1718.22917869869,0.0442576143120988,0.137784464403297,0.3212
09031103508,0.748051988659198,0.836178753102684,0.00791825676285913
"Adal",608.089630277274,0.282255301651442,0.16165306162764б,1.74605602
151596,0.0808012177163387,0.16754888259419,0.504243489091004
"Hao1",2.83134070608883,0.133758650365933,0.966685068571694,0.13836838
3576634,0.889949282881567,0.931963645610656,0.00942483138586029
"Tubgcp4",1802.91748873021,0.0620995922343712,0.154726115593406,0.4013
51717492595,0.68816119180508,0.790862131192705,0.0145705281293999
"Lamp5",0.105552103896451,0.877046898575489,4.78471990596623,0.1833016
17610232,0.854561365232847,NA,NA
"Ubr1",1981.15867670316,-0.0992299033407562,0.124020464542774,-0.80010
9108658693,0.423647584212278,0.566821696652259,-0.0563338573156822
"Snap25",8.2041601378393,-0.710889029286131,1.60878732285581,-0.441878
810944512,0.658576904462202,0.767631066666907,-0.187991790948273
"Mkks",1053.66911426989,0.345894090370373,0.186154769216284,1.85809953
635137,0.0631548806774738,0.13871734408577,0.68325044646842
"Jag1",2840.31473389087,-0.555313485723453,0.123244329688508,-4.505793
38722498,6.61254052407843e-06,0.000122674895469535,-5.00113816349254
"Slx4ip",339.425913506761,-0.688863551944543,0.146267802041724,-4.7096
0486401539,2.48197492129753e-06,5.66006397841629e-05,-6.73673440305641
"Mtch2",6681.33850164246,0.0311594740070445,0.361127397894378,0.086283
8826096434,0.931240750504381,0.957159009853384,0.00136434084037076
"Cdan1",1217.56941942521,0.119760991059901,0.162133929414054,0.7386547
12759461,0.46011669199695,0.599618143593097,0.0612532263948401
"Haus2",614.031101940875,0.0599811780421906,0.138102885049245,0.434322
411300838,0.664054331605259,0.772267942171891,0.0155005587890747
"Lrrc57",719.12211326819,0.112290089064329,0.116962624769131,0.9600510
35841364,0.337029529801561,0.4814566467925,0.0820772154731005
"Snap23",2659.3105982427,-0.197688523159547,0.117546088211953,-1.68179
584847678,0.092608434041817,0.186075767410073,-0.332433284765105
"Zfp106",6404.40569862466,0.0294810883028533,0.138922122895862,0.21221
3056411129,0.831940821168005,0.892654640167043,0.00334774013679552
"Vps39",1985.58012751773,-0.0732844160373234,0.151642604733431,-0.4832
70622831549,0.628903596107277,0.74497948462331,-0.0215748293659589
"Ehd4",3956.04912150905,-0.720288297589878,0.176667416721205,-4.077086
26161975,4.56035776512552e-05,0.000540205684716995,-5.41913265156593
"Itpka",42.7566538480152,-3.14737126298942,0.807283731073131,-3.898717
56588676,9.67034893216945e-05,0.000964956312586693,-21.853544902727
"Ltk",207.748765679937,0.441524257130485,0.407683339720299,1.083007849
75271,0.278804938828548,0.422562290119642,0.380337123914116
"Tyro3",598.050188670296,-1.00840466969592,0.290355401816979,-3.473001
23705481,0.000514672983053104,0.00354050170985102,-5.69091847872127
"Ubox5",416.684746186618,0.171333120186799,0.126492540553782,1.3544918
8890275,0.175579499392038,0.301864833713601,0.205218687762458
"Oxt",3.28454661051291,2.76367856019497,1.13366680204168,2.43782260821
057,0.0147760230082006,0.0457161054607894,8.52679024677095
"Ptpra",3731.81084194366,-0.110869636576505,0.130028621602823,-0.85265
5632351158,0.393850301798794,0.538293367760611,-0.0686672839450112

"Rtf1",4317.5442308123,0.312559016063458,0.202540147245968,1.543195363
05995,0.12278338203931,0.230438962653044,0.458764514376195
"Ndufaf1",1193.04019953374,0.341699227031951,0.335524528361448,1.01840
312152634,0.308486419362665,0.453438990179663,0.270248056229011
"Nusap1",230.848835210827,-0.0815927973347796,0.353232951743631,-0.230
988634927803,0.817323631316621,0.882982372804105,-0.0101542270084333
"4930402H24Rik",6170.63009976634,-0.0836073633964207,0.237960362809628
,-0.351349957653695,0.725325820103506,0.819137146850748,-0.01667998274
89419
"Atrn",3334.54536673879,-0.692207095094962,0.33207717562339,-2.0844765
792636,0.0371168384399345,0.0925689582888007,-1.64731542589809
"Chac1",50.6695619168875,-0.609092645981673,0.418917667154712,-1.45396
743498222,0.145955334934848,0.262506883180487,-0.814647999133483
"Dll4",902.510262459912,-0.357412320133895,0.194822846166338,-1.834550
3474923,0.0665723413717933,0.144401770171012,-0.691648522081367
"Spint1",3977.78563132886,0.413508034393327,0.139422051433749,2.965872
54412778,0.00301825545324935,0.013600458267013,1.77711354448095
"Gfra4",122.845821439607,0.0925100292532128,0.168573321864225,0.548782
145538567,0.583154964594413,0.707324412438034,0.0320330648903652
"Ppp1r14d",38.0751766728078,1.29305851945262,0.344035171188394,3.75850
676832266,0.000170930415687109,0.00150058588251565,8.40733674642449
"Adam33",392.139827849686,-0.96088199785567,0.277240650999398,-3.46587
700754518,0.00052850498565672,0.00361698498081247,-5.40218859218677
"Siglec1",304.242532342693,-1.62892659770618,0.397439696623296,-4.0985
5032485626,4.1574585184551e-05,0.000502555029093847,-12.3730094688755
"Rad51",130.132367992787,-0.647052999114494,0.376733227809343,-1.71753
631310152,0.0858812254465854,0.175488590836043,-1.1259894953081
"Rpusd2",80.9204933400393,-0.0607969783109568,0.218967886900863,-0.277
652486725063,0.78127913700517,0.858750809703298,-0.00925795061544412
"Knl1",54.0467627346497,-0.730150946911126,0.425321001682358,-1.716705
6035865,0.086032975788577,0.175761835910067,-1.26945898608327
"1700037H04Rik",2484.33606193212,-1.75643871977081,0.622254893625819,-
2.82269972926402,0.00476211494828915,0.0191328492193987,-6.94908381157
905
"Spef1",996.519769135272,-0.180944472274194,0.145093598035853,-1.24708
791237972,0.212365264596673,0.346646959322388,-0.191701336206641
"Cdc25b",906.340013141638,-0.236800004198947,0.139125289045258,-1.7020
6298095751,0.0887435625318136,0.180129292342838,-0.405894244767846
"Knstrn",265.526025445275,-0.427619784921818,0.354952487435051,-1.2047
2401253439,0.228309862926181,0.364764479460397,-0.431256005043683
"Ivd",13238.4028318864,0.747563878932086,0.302442667335366,2.471754020
41784,0.0134451992803007,0.04255064041021,2.36010429869236
"Smox",779.769791368192,-0.456519659023058,0.22086746365629,-2.0669393
8285762,0.0387398661591469,0.0956865787173977,-1.07130429054107
"Adra1d",46.9916730540754,-1.83064086766957,0.537957532028181,-3.40294
681025801,0.000666632569130873,0.0042997028888236,-9.97554534045469
"Prnd",113.991461790617,-1.2183294149484,0.494344377739767,-2.46453579
692526,0.0137190858945272,0.0432629365633983,-3.82611355059919
"Rassf2",1762.45004851847,-1.59812047944851,0.315131248377357,-5.07128
533802154,3.95137798998631e-07,1.32150888591343e-05,-17.9535272390903

"Slc23a2",1487.71590495399,-0.845818561894649,0.256848634174762,-3.293
06233070777,0.000991025017989873,0.00583247986742123,-4.35115542855834
"Tmem230",1202.61467768378,0.00481268517875591,0.157272683383155,0.030
6008969595249,0.97558782681582,0.984729070106894,7.40611194028089e-05
"Pcna",2850.65562111621,-0.468295722045185,0.21610609286689,-2.1669713
9739429,0.0302370351419463,0.0791111392507979,-1.18802016147581
"Fsip1",24.1981917512708,1.10848278695968,0.510388731256026,2.17184024
46539,0.0298677149540049,0.0784007412726424,2.82211059939389
"Gpcpd1",1904.60710136841,0.15645520712799,0.130810452721989,1.1960451
4679346,0.231678941567347,0.368697029275232,0.156107881429194
"Rasgrp1",561.076413646184,0.0038317899803357,0.39909145443919,0.00960
128295836402,0.992339402262036,0.995044158668305,1.90369542810793e-05
"Fam98b",1780.82752730069,-0.180066370425136,0.134448197122671,-1.3392
9925635851,0.180473270746963,0.308196037130405,-0.211941577837681
"Chgb",9.33763017481655,0.0218249940742551,0.773024513868265,0.0282332
496353077,0.977476118413618,0.985665564585681,0.000315112885332461
"Spred1",2114.75705824752,-0.365750164342616,0.151317173215952,-2.4171
0941705629,0.0156443136636367,0.0476464898178052,-1.11332386588985
"Mcm8",164.507190389514,-0.190525922755906,0.178817559976172,-1.065476
58284396,0.286660317018038,0.430757492647987,-0.160462839176472
"Tmco5",0.772349650284562,3.01273139185741,2.12397438723875,1.41844054
709816,0.15606218472495,NA,NA
"Fermt1",2429.8096773791,0.10224520464868,0.332279267352766,0.30770864
9604464,0.758304037445848,0.843242393185761,0.017432891837966
"Crls1",1904.98166075422,-0.235236632479627,0.424170027449739,-0.55458
0987001729,0.579181296794458,0.703965219429095,-0.0825742513726428
"Bmp2",508.411508352105,-1.46481106129827,0.310446146480108,-4.7184063
2556259,2.37699373862633e-06,5.44613677035679e-05,-14.3815427684306
"Slc27a2",93294.28226768,2.36887361684213,0.701592339904053,3.37642457
323021,0.000734345036707945,0.00463674207439492,12.7297187234944
"Hdc",52.2427942938454,-1.25169065605858,0.369172770014138,-3.39052811
508999,0.000697580989442589,0.00445073106418574,-6.77751301349654
"Gabpb1",940.070323418287,-0.163743718377229,0.0992356351285391,-1.650
04958314755,0.098932795248439,0.195677788776026,-0.267112820199541
"Usp8",4221.72434282453,0.348188809619108,0.151497531009017,2.29831342
662861,0.0215439568202116,0.0609583616427929,0.974080563917687
"Usp50",64.2055618777441,0.590079002952424,0.433977449568045,1.3596996
884049,0.17392497678071,0.299760381620286,0.710910572866001
"Trpm7",4729.00712412451,0.967161018458043,0.174827886277519,5.5320752
2581832,3.16464118799459e-08,1.92224840758625e-06,12.72978784343
"Sppl2a",4972.34783261034,-0.244289328479324,0.152991794547904,-1.5967
479118812,0.110321911011071,0.212240416467081,-0.378657157530749
"Stard7",5008.45533352008,0.59302278041697,0.17945761536283,3.30452836
575359,0.000951363481073771,0.00565194550700073,3.06934089102166
"Dusp2",264.359965897948,-1.39862986192402,0.440758628418136,-3.173233
08438373,0.00150751445225824,0.00807736103053892,-6.73956382016425
"Fahd2a",2199.99607114849,1.57231708234937,0.293209260684271,5.3624400
4940363,8.21052224295289e-08,3.93338483275419e-06,19.569074479418
"Mrps5",2704.15444523809,0.523310612784341,0.220161695451431,2.3769376
0357049,0.0174570408459981,0.0518078207633594,1.54911148663141

"Mal",11073.4318678655,0.328730943549607,0.247225190993101,1.329682231
12337,0.183622991734956,0.311735402076463,0.383168959154214
"Prom2",1576.95678942666,-1.41723718504479,0.591122450441692,-2.397535
71190845,0.0165057719195176,0.0496650526059805,-4.25518911518987
"Mall",139.111516350466,-1.55483071100151,0.454881556064658,-3.4181001
4117281,0.000630599005614178,0.00412459557580182,-8.53724473130649
"Nphp1",1170.14911160014,0.687118765100242,0.127558335139026,5.3867022
0453529,7.17621869637913e-08,3.53839172465398e-06,8.62460359747363
"Bub1",88.6619040647329,-1.16600468936332,0.491649593730274,-2.3716173
1491842,0.0177104233520104,0.0523288126257765,-3.43995653630869
"Acoxl",10.4442162549795,-0.636395644027219,0.66459205637852,-0.957573
353336559,0.338277941202592,0.482921606206482,-0.463232990358851
"Bcl2l11",880.003455636892,-0.614110573070751,0.167367803134369,-3.669
22766248965,0.000243284354087667,0.0019671799592104,-3.82661771017451
"Ndufaf5",569.978262419382,0.242218758026037,0.285212411724019,0.84925
742383335,0.395738066430413,0.54012655561814,0.149195081120538
"Fbln7",1413.27014333081,-1.03140674271164,0.43127136474225,-2.3915493
2840965,0.0167774292690483,0.0502266721768623,-3.08515320893885
"Zc3h8",386.283960413856,0.115451422410832,0.126872646431194,0.9099788
30412781,0.362833674225143,0.507753605113173,0.0782482381048978
"Ttl",753.008727497267,-0.621888947073586,0.216794073706918,-2.8685698
6650988,0.00412332120307539,0.0171897619879333,-2.5270092350623
"Polr1b",1266.79179425889,0.0627238548253947,0.151935634009283,0.41283
1757568882,0.679729882233099,0.784419442002853,0.0152300669496029
"Slc20a1",2973.7918831563,-0.00495157058103183,0.24758682174414,-0.019
9993301184215,0.984043906947482,0.989729087881048,-5.11201231531977e-0
5
"Il1b",392.175002640596,-2.01246238229307,0.523900604800849,-3.8413057
054173,0.000122381585006442,0.00116409250289429,-13.5958204560565
"Il1a",13.1716859654522,-0.75660784989088,0.542079789963449,-1.3957499
6873043,0.162789801250469,0.284989271019361,-0.94977266744036
"Pdyn",3.79118165003165,-0.115215658720637,1.2803914020269,-0.08998471
75935787,0.928299359149946,0.955900760744951,-0.00519636194054693
"Tgm3",7.4310627117744,-1.52071437736006,0.784200466454645,-1.93919086
051451,0.0524781020853749,0.120648276672638,-3.21612199084073
"Tgm6",0.247954256230582,-0.0561589643304147,4.78471990595559,-0.01173
71477190364,0.990635326061519,NA,NA
"Snrpb",4011.03633922759,-0.527404708183559,0.324895719265814,-1.62330
457715899,0.10452431158054,0.203946922211882,-0.838518374449639
"Nop56",3190.1191634092,-0.00118810898136493,0.25125816872393,-0.00472
863822656591,0.996227106625734,0.997451786181699,-3.03141971921385e-06
"Idh3b",11349.1558504617,0.168067644355735,0.370895307547099,0.4531403
90120445,0.65044765290899,0.761890444336146,0.0457064173069534
"Cpxm1",1883.55701353139,-1.09965073374009,0.420712582625344,-2.613781
42502422,0.00895463027220411,0.0310575110419134,-3.81789343555324
"Vps16",2839.26434437087,0.394486676286014,0.211613031522612,1.8641889
5588602,0.0622951737550087,0.137293014341038,0.783307674294826
"Lpin3",822.989621536733,0.24461831933443,0.203112206819236,1.20435065
506493,0.228454075710762,0.364935078503067,0.246584025263774
"Otor",0.0979576720737022,0.875197899124178,4.78471990596623,0.1829151

79221434,0.854864572537654,NA,NA
"Pcsk2",12.9536635787751,-0.516320416802662,0.66812882344822,-0.772785724372626,0.439649203349482,0.581489980400399,-0.279929071161804
"Bfsp1",72.2152712048549,-0.721364296128814,0.338622370218129,-2.13029132028145,0.0331475696991722,0.0849068988432027,-1.77902857698292
"Rrbp1",14382.8570373212,-0.184733879620908,0.170857127053468,-1.08121845899409,0.279599948844549,0.423329694251515,-0.15879797899258
"Snx5",5044.78268365309,-0.155808927254388,0.132100153806775,-1.17947574445895,0.238208789593091,0.376324337991094,-0.152272673254367
"Mgme1",726.107043536533,-0.106782500026541,0.185560665488581,-0.575458703736498,0.564981112028024,0.692644567194389,-0.039214623930777
"Kat14",1711.07610380109,0.490933329853312,0.145972736830651,3.36318507491481,0.000770486903514235,0.00481196465237686,2.61993926964392
"Polr3f",1009.31797332454,0.199043124648029,0.11247688371671,1.76963584045722,0.0767878242965763,0.160990214853188,0.363534690299085
"Rbbp9",2055.17451237823,0.229202977841928,0.172534469385248,1.32844746130204,0.184030329147534,0.312231392216994,0.266794724256797
"Sec23b",3148.41550442813,0.426475070411343,0.164929168696745,2.58580743346559,0.00971511905019018,0.033046997774007,1.45420516749147
"Dtd1",825.779801180154,0.260735712743936,0.199908551481104,1.30427493377432,0.192139858622264,0.32207212546569,0.29540828691435
"Xrn2",2104.07762757878,-0.2329210141158,0.127282059650961,-1.82995949904116,0.0672559952743258,0.145480074978789,-0.44900559906548
"Nkx2-2",2.95792332305356,-0.157151142910593,1.5133111512165,-0.103845889712942,0.917291648584775,0.949331351328659,-0.00817144815209777
"Cd93",2407.43173085569,-1.42584984198222,0.303185801828294,-4.70289120857229,2.56503109843636e-06,5.81782255149808e-05,-13.9048868110246
"Napb",404.761450697291,0.640721114698052,0.245219819959679,2.61284391613779,0.00897922995480346,0.031135875094924,2.22291436556161
"Gzf1",1458.35009060064,-0.210467815454614,0.230023786106075,-0.914982832938663,0.360200642482826,0.505411062689273,-0.143619700415628
"Cst8",2.25248769806398,1.16433646322638,1.04262880078132,1.11673153700901,0.264109129281994,0.405991785141937,1.04955891462796
"Cst12",10.105980448891,-0.634975020086856,0.758758276493617,-0.836860749672755,0.402670845182102,0.546479859355511,-0.383688625153072
"Cstdc2",0.922382400264741,-1.65404646978928,2.44516977192571,-0.676454653079825,0.498752027750951,NA,NA
"Cst3",22046.9380675171,-0.734625532859875,0.25663526306093,-2.86252763590583,0.00420276607311255,0.0174166913695108,-2.97547308587841
"Acss1",5374.3018517381,1.0005046246366,0.277918106585217,3.5999979883636,0.000318219642172377,0.00241896552946472,6.02745536574717
"Gins1",186.634234881373,-0.545991251048909,0.213774897430107,-2.55404754071941,0.0106478717146594,0.0356232331318512,-1.82074827627753
"Nsfl1c",5925.16030922651,0.17179553588579,0.105353739069159,1.63065437832268,0.102963268086035,0.201746337166266,0.275000695166668
"Sdcbp2",140.234765504985,-1.96736676113163,0.506165119868261,-3.88680824479505,0.000101570925893825,0.00100325730145786,-13.5836902375662
"Snph",31.1140644440064,-1.07350306190989,0.466157130890941,-2.30287813008064,0.0212857009763966,0.0603590165267278,-3.01380073781174
"Fam110a",556.565632074926,-0.274025625109893,0.209724538289788,-1.306

59782276529,0.191349336468295,0.321133530939818,-0.311265230240758
"Angpt4",88.5182058707802,-1.5160336053876,0.410622606041193,-3.692036
39323139,0.000222465621029351,0.00184032018618171,-9.5477002542764
"Slc52a3",1170.72788027559,0.638384331848396,0.290074592965464,2.20075
920928518,0.0277530747111,0.0740867265494289,1.66140728374531
"Tbc1d20",3725.13238269372,-0.413162709985949,0.240413799649734,-1.718
54823054208,0.0856966651219581,0.175221641835984,-0.719606971785722
"Rbck1",4142.23488968524,-0.152981675418177,0.126731795812824,-1.20712
939035538,0.227382317824395,0.363759471032166,-0.154704619854909
"Tpx2",272.498174823946,-1.0565824442537,0.388858006252195,-2.71714206
025231,0.00658483375164377,0.0245677551644757,-3.91603313242058
"Mylk2",14.4194537294716,-0.905601714192258,0.641194920628953,-1.41236
570199893,0.157842309888662,0.278693231749447,-1.15703626133226
"Pdrg1",3306.0759154009,-0.0649039060444214,0.127246758035894,-0.51006
3337143043,0.610007089538085,0.729822558250818,-0.0204417347820907
"Ccm2l",871.621944153954,-0.179753194487445,0.239729054371515,-0.74981
8143481582,0.453364239832792,0.593866036161221,-0.0936696616864218
"Kif3b",1354.10153084676,-0.384475762670008,0.130878942671548,-2.93764
416812934,0.00330716365589469,0.0145559993143645,-1.62623714391987
"Dnmt3b",249.315063103692,-0.228678082768271,0.227720671651199,-1.0042
0432238378,0.315280139137274,0.460555300974729,-0.177299226585765
"Mapre1",5161.67328083255,-0.400667297145571,0.163069747997693,-2.4570
3020986602,0.0140090905517362,0.0439886736259378,-1.25161375655261
"Bpifb2",0.148413830377466,-1.23093098304697,4.774987514986,-0.2577872
67334998,0.796571091654078,NA,NA
"Bpifa1",0.68029147470362,0.41331334129593,2.67584671468474,0.15446076
9007325,0.877246441745702,NA,NA
"Bpifb1",0.339050003714232,-1.6517244729272,4.75955277413097,-0.347033
545232364,0.72856611753449,NA,NA
"Cdk5rap1",522.891775526537,0.138574253407505,0.276243099102377,0.5016
38787929139,0.615921629781394,0.734998624295786,0.0426651628626266
"Snta1",2604.44228710681,0.548735352912222,0.126596182152026,4.3345331
8721145,1.46069751987393e-05,0.000226396504312416,4.60565799405484
"Necab3",45.8550448539657,-1.53951726722723,0.470648698719562,-3.27105
391221862,0.00107147482009428,0.00621034959637286,-7.82311563194836
"E2f1",409.718692740185,-0.535913543235636,0.253155805886841,-2.116931
67122221,0.0342656472689393,0.0870844095998148,-1.30809925893529
"Fam210b",789.596684743707,-0.79070510290143,0.377743742971367,-2.0932
3150313933,0.0363284938148821,0.0910102752669371,-1.89514844096793
"Aurka",130.393916578286,-0.626869591665028,0.341589184610601,-1.83515
64390999,0.066482513407032,0.144230652695516,-1.21383360920107
"Cstf1",1361.87501796143,-0.061937376593903,0.111254436313185,-0.55671
8263526563,0.577719936618581,0.702794068142569,-0.0218447777731321
"Pkia",1178.00910946929,0.0672885856216768,0.296223471481448,0.2271548
07433586,0.820303367567757,0.884736976481698,0.00824048855430885
"Stmn2",117.098739049309,-0.420641066177693,0.383618582292648,-1.09650
857803547,0.272856271474699,0.41549491395867,-0.369442699071979
"Rtf2",3332.85848374981,-0.467751094226283,0.265704474260911,-1.760418
58356879,0.0783368607296687,0.163615752176731,-0.846739203145432
"Fam209",9.13099480447347,-1.17801301568243,0.647178792044628,-1.82022

808868743,0.0687242772619639,0.148064746746273,-2.25012928506702
"Tpd52",5771.90045698915,0.34439428628652,0.198640351158759,1.73375794
131209,0.0829610410748394,0.170900621117509,0.608432105697781
"Pag1",825.931265312614,-0.678768813519731,0.22899639390088,-2.9641026
3042628,0.00303567024657429,0.0136570139519394,-2.91429529007642
"Rae1",1794.5566630666,0.138006951736502,0.0950618927479916,1.45175893
03882,0.146568647113794,0.263294221616431,0.184167952879669
"Rbm38",649.527198715159,-0.337335312469539,0.203657517343044,-1.65638
527303329,0.0976438215458893,0.19369816181725,-0.553720464941282
"Pck1",58988.0498830491,0.983659823899716,0.740147094639599,1.32900585
7111,0.18384603751988,0.312053109884488,1.14555240864068
"Zbp1",561.660966945378,-1.31294338709397,0.475166131397525,-2.7631249
3744542,0.00572508475468923,0.0219912731573937,-5.01164877954001
"Ankrd60",4.44623216616325,-0.603238113414486,0.784940856328304,-0.768
514097019025,0.442181818851659,0.583643559123388,-0.324822506051626
"Rab22a",3304.39924457911,0.0168078550261694,0.0888142154844431,0.1892
47351164335,0.84989895726333,0.904351117044336,0.00168982124057864
"Zdbf2",11.6669647478343,-1.4184776373833,0.54118719119743,-2.62104805
962753,0.00876599033551247,0.0305522681314343,-4.94809873865834
"Stx16",6452.79624704147,0.0574771236422381,0.183628366541613,0.313007
868690118,0.754274683979708,0.840343282568787,0.00999784682841239
"Gnas",32246.7858203592,0.280014913635585,0.198078942323186,1.41365311
401306,0.157463778039798,0.278176021040137,0.358279417020414
"Edn3",466.727381528325,-0.163464061704084,0.495913575990093,-0.329622
074527255,0.741685540918126,0.831876263309317,-0.0300890866734437
"Phactr3",8.41409195598093,0.214455142504781,0.616850092509467,0.34766
1684919808,0.728094274666188,0.821454818160013,0.0421786819070863
"Fabp9",0.135890711193464,-1.02856587253889,4.78024249005756,-0.215170
229267282,0.829634600901222,NA,NA
"Fabp12",2.1740778612296,1.77464176218921,1.23556118780742,1.436304231
39013,0.150915790529667,0.269280472986238,2.32833317225027
"Impa1",3879.12432089042,0.688049807348877,0.194241107513199,3.5422461
0926974,0.000396735134125735,0.00287487747858919,4.02629217250306
"Fabp5",3518.57507479937,-2.8302058430273,0.658246606808747,-4.2996132
6614118,1.71096414684595e-05,0.000253884162368359,-23.4302339284099
"Snx16",510.951923196071,0.336038373483843,0.119431578814664,2.8136475
8650067,0.00489828952898608,0.0195692834632513,1.32190577511215
"Chmp4c",906.553430238155,0.0462674891100771,0.188899068617248,0.24493
2330523161,0.806508827851619,0.876948921651031,0.00607522346708451
"Ptpn1",3163.67284570139,-0.339531957728286,0.161050674389903,-2.10823
058651888,0.035011048794611,0.0884141211497636,-0.82361067622291
"Nfatc2",623.453204005937,-0.443590130359675,0.34856305808946,-1.27262
519668916,0.203151073109743,0.335311912595765,-0.48470831736175
"Atp9a",2512.63158983044,-0.512347394681571,0.321907320407002,-1.59159
907899512,0.111474817168577,0.21407820202432,-0.789739396146215
"Sall4",1.16238332585273,-0.941160310680961,1.91516311394494,-0.491425
666998315,0.623125414229042,NA,NA
"Lrrcc1",1319.0309853562,0.334012880623575,0.205054860323969,1.6288952
1416788,0.103335203410745,0.202251535444962,0.533833792207487
"Zfp64",640.235271669366,0.088378003051099,0.12563542421971,0.70344812

0623561,0.48177652694908,0.619308987503505,0.0423464048974473
"E2f5",1318.85046599616,0.53225596960469,0.150014893100396,3.548020857
15905,0.000388137484274208,0.00282595394636987,3.12376200533832
"Car13",359.05539939021,-1.97654788874268,0.389988257328895,-5.0682241
1085001,4.015444600585e-07,1.33285410705371e-05,-22.1879416660718
"Car1",7.8854946735959,-1.54611744318134,1.1514956955781,-1.3427036237
4661,0.179367960861512,0.306707295890264,-1.82729655655118
"Car3",40530.0399155754,-3.67576044076961,1.16384185737776,-3.15829888
525528,0.00158692764076399,0.00839899976645392,-17.5688213946351
"Dok5",16.5067963017196,-1.43463926700143,0.65488639712992,-2.19066890
576568,0.0284757607936075,0.0756327998847241,-3.70404523959753
"Car2",9892.04584556951,1.27039102215806,0.465408013781046,2.729628593
709,0.00634057147985386,0.0238618920506062,4.74551081319681
"Psma7",6367.6681449478,0.229715878062627,0.123360463190736,1.86215155
262061,0.062581731120164,0.137737734888922,0.455389646552119
"Ntsr1",0.255630164157412,1.45079204318902,4.75720974231816,0.30496701
2550104,0.760391276440168,NA,NA
"Mrgbp",1425.23306524022,-0.0690756065187204,0.143473295963898,-0.4814
52705568999,0.630194785287306,0.745919864234317,-0.0202486233310889
"Col9a3",100.086037407012,-1.33783413782539,0.350773214219775,-3.81395
751896603,0.00013675898627174,0.00126977462586971,-8.92190329203994
"Gid8",1570.86217382129,-0.121291637237247,0.149775770790616,-0.809821
48579165,0.418042782023037,0.56120890690479,-0.0700655769911342
"Nkain4",51.2846934686531,-2.14705097886648,0.485399391866272,-4.42326
672600776,9.72195943606321e-06,0.000167602798483077,-18.666275861071
"Arfgap1",2834.98022283833,0.19647219792935,0.265537463620806,0.739903
873639154,0.459358323819592,0.599061507446619,0.100670586477688
"Chrna4",1629.78111553044,1.61291896946104,0.802224146247254,2.0105589
902849,0.0443720594178519,0.106200308728484,3.61685508325901
"Srms",53.5981623790531,-0.483762440583704,0.311118892713866,-1.554911
80996397,0.119967074289261,0.226417385973479,-0.718568704085503
"Helz2",2451.52974312527,-0.81283793443937,0.208139738631303,-3.905251
05770035,9.41276125310511e-05,0.000944570349526729,-5.66123770639205
"Stmn3",12.0145305004698,0.0876959688201435,1.08240186474182,0.0810197
87268254,NA,NA,NA
"Zgpat",2495.94310074311,-0.224893097278964,0.183722149882661,-1.22409
354246398,0.220916941791912,0.356694147984325,-0.231837029853501
"Zbtb46",1470.2996321605,-0.955956955024745,0.173564337976465,-5.50779
593417613,3.63354257402915e-08,2.10227932072464e-06,-12.4967362159991
"Oprl1",4.29995514971442,-0.627333085886937,0.821940565101484,-0.76323
4122420373,0.445323782210352,0.586502540139127,-0.334731308882489
"Pcmtd2",4072.44784547672,0.292339679404193,0.103019811660676,2.837703
49306302,0.00454393717889016,0.018438644560666,1.16740196212723
"Raly",7485.9182017553,0.0119614196804184,0.111706476272144,0.10707901
7077555,0.914726293393257,0.947780490412462,0.000641519091494829
"a",3.40916983754705,-4.70617805721224,1.7386376777042,-2.706819320415
6,0.00679312174838098,0.0252050024452773,-17.3220897880561
"Ahcy",11064.912111909,1.73049827770378,0.394388628823052,4.3877996251
261,1.14503190995938e-05,0.000189494617453548,14.8323602052766
"Itch",3498.23757303538,0.232945702969998,0.134142549046866,1.73655342

488394,0.0824660265706147,0.170168545462361,0.412538893993607
"Armc1",2220.82446821482,0.354668981598346,0.166628898310128,2.1284962
2841675,0.0332959623306659,0.0851751080455687,0.873566033092089
"Mtfr1",2906.68723286228,0.845897368196844,0.244856421228349,3.4546668
7764735,0.000550973124408048,0.00372930520572292,4.72986333192419
"Map1lc3a",3878.09697010135,0.224625859555182,0.327084950021531,0.6867
50825864644,0.492239762327419,0.628660308643597,0.10426328644578
"Ggt7",223.739586399834,-0.652256052635646,0.328650705679622,-1.984648
26444488,0.0471836185406883,0.1113380363435,-1.43182226782375
"Acss2",11124.8727759849,-1.13476179132284,0.684195163537564,-1.658535
24227746,0.0972094802821947,0.193032940884228,-1.8665633459345
"Dnajc5b",0.569785479848724,2.0465204039241,2.86534577042541,0.7142315
68506393,0.475083999290907,NA,NA
"Gss",12540.3618080138,1.91604402204004,0.475064119132592,4.0332324519
4458,5.50148105439202e-05,0.000621482488983404,14.1469248409764
"Procr",371.854770392852,-1.21504986102418,0.248289859312047,-4.893674
93457367,9.89702606491272e-07,2.75589540336503e-05,-12.7570309238425
"Mmp24",35.8475812920995,-2.04857144167541,0.587563395256413,-3.486553
89054898,0.000489286792365853,0.00340232306617311,-11.642639572135
"Eif6",3425.77519999915,-0.38997075130071,0.293829481557454,-1.3272008
9636226,0.184442235972644,0.312690610989881,-0.453357003014884
"Hps3",1751.78059014136,0.497024528967724,0.156893641998495,3.16790739
660751,0.0015354041862162,0.00820425070508182,2.38725994669208
"Nfs1",3989.29997976891,1.31758767990824,0.246899732182845,5.336529401
06262,9.47425154378851e-08,4.36711481325741e-06,16.260887123904
"Rbm39",20153.8880140854,0.312735103761148,0.2001883510415,1.562204304
76655,0.118239866836776,0.223955117619019,0.467948542698208
"Epb41l1",2995.3944990308,-0.152356147504914,0.311941290605399,-0.4884
12890801438,0.625257412911324,0.742283794261141,-0.0454057331817339
"Aar2",1168.19050193687,-0.241399866087617,0.287476905876574,-0.839719
160575839,0.401065868282282,0.545136444738093,-0.14646192357497
"Tbl1xr1",2232.29262612452,-0.0668484123210573,0.180051953642634,-0.37
1272907450578,0.710434274902987,0.807887990905077,-0.0142608958168148
"Ndrg3",2648.80839208191,-0.135189638439384,0.283022810254851,-0.47766
3402174727,0.632889796660235,0.748110649989163,-0.0392326257674249
"Dsn1",244.112693872288,0.181537239725941,0.235440441455764,0.77105376
8857503,0.440675061697583,0.582409161050598,0.0981357736376231
"Sla2",56.8741846791223,-1.36487710466562,0.499121307233174,-2.7345598
8531459,0.00624637319705248,0.0235959348425855,-5.1137588891171
"Rab5if",3304.53512050419,-0.461413883043704,0.406771806509965,-1.1343
3103194284,0.256655716945347,0.397511571133559,-0.425668719620296
"Samhd1",5515.42425241129,-0.906048900854241,0.25583307023104,-3.54156
286376893,0.000397764064113989,0.00288098206615697,-5.30004543888941
"Rbl1",683.438948558217,-0.398041323615051,0.163731668918312,-2.431058
85528864,0.0150547688431769,0.0462785048340853,-1.22321190896471
"Rpn2",7752.22358465393,-0.14915151954114,0.163312240732353,-0.9132905
09470013,0.361089772430899,0.506106294483983,-0.101573462082324
"Ghrh",0.908491171975813,0.307647455035478,2.6676134173738,0.115326850
971663,0.908186056354468,NA,NA
"Src",2061.28660222425,-0.240900825124745,0.133733457962643,-1.8013504

5331766,0.0716476602442227,0.152726148402958,-0.45267887030998
"Ctnnbl1",998.681235926425,-0.181939860729685,0.122578236621705,-1.484
27539622045,0.137735874753175,0.251199521647873,-0.251351327387804
"Tti1",857.489148088325,-0.237203495919674,0.14329225553166,-1.6553825
2601003,0.0978469282571288,0.194022109951947,-0.388962297530145
"Rprd1b",1225.52490870906,-0.00547446606514879,0.152001205431142,-0.03
60159384895719,0.971269650122392,0.982455677427184,-9.68983137134569e-
05
"Ralgapb",2824.01612106098,-0.090002170986684,0.146758158445995,-0.613
268604210537,0.539698749145066,0.670556694757567,-0.0359690997789495
"Fam83d",49.2189687217325,-1.09329703981299,0.431811111579031,-2.53188
723146809,0.0113450471166779,0.0374100674244859,-3.59237228114522
"Dhx35",739.415028276539,-0.185177919257026,0.161904018913485,-1.14375
122062892,0.25272682467105,0.393018633548902,-0.17293733558949
"Ccn5",111.651412208758,-4.58525142807782,0.979052154747391,-4.6833576
8002152,2.82213423694247e-06,6.29587795788377e-05,-44.353276171681
"Skil",2576.99595952003,-0.849090750945915,0.13889573403262,-6.1131521
1989524,9.7682174657982e-10,1.53597342054946e-07,-13.3213264766425
"Slc2a10",150.583602082988,-1.45439096326652,0.326697454829482,-4.4517
9765488417,8.51543900251404e-06,0.000150121184151012,-12.8045564966112
"Zmat3",1030.63951837932,-0.127319823566921,0.123397326259989,-1.03178
753888612,0.302171658068101,0.446722005254968,-0.102596706171931
"Pik3ca",5472.78582914565,0.423869971543482,0.175590186326193,2.413973
01530315,0.0157796309687544,0.0479313358200301,1.2877109528877
"Zfp639",1461.45804761757,0.128913116906644,0.0987260499766506,1.30576
597500996,0.191632154170685,0.321488867487121,0.146289620516694
"Mfn1",3782.44449228946,0.772826867067218,0.227004520867684,3.40445584
128997,0.000662960184121502,0.00428191464783333,4.21449927649829
"Gnb4",1003.47515830279,-0.968449536488519,0.165625981093676,-5.847207
84802944,4.99892734538219e-09,4.7778963421697e-07,-14.094906844492
"Ocstamp",46.4967645267642,-0.197808603715793,0.386201597513034,-0.512
190019382604,0.60851802752715,0.728552688340024,-0.0626450613356507
"Actl6a",1757.53065667276,0.344708954542021,0.139437393750228,2.472141
40533559,0.0134306380177895,0.0425183601349016,1.08852858717923
"Ndufb5",5867.00412432112,0.841613856562638,0.327771207021739,2.567686
96741205,0.0102379561978349,0.0344706734493842,2.83425784174292
"Pex5l",70.9313722244419,1.08904093468524,0.381370660292167,2.85559705
576441,0.00429559915481075,0.0176998293154058,4.39340924652055
"Ccdc39",319.206573926967,0.1659044226422,0.415234424782486,0.39954399
9101487,0.689492410430089,0.79192524850176,0.0387035565443575
"Ttc14",4185.90035585828,-0.0135954957829963,0.189730908335075,-0.0716
56726372626,0.942875094940101,0.964603710314293,-0.000489953455665854
"Ncoa3",4050.66775709148,-0.099148986227323,0.170351555888377,-0.58202
5715645888,0.560549359544074,0.688781914065437,-0.0369657743764048
"Dnajc19",3185.81662102958,1.15941561076518,0.232229395171176,4.992544
59113833,5.95889501094222e-07,1.80413874104583e-05,12.6641136896875
"Fxr1",3459.94659033642,-0.213508356463634,0.161173493392021,-1.324711
35278007,0.185266906291805,0.313679588301286,-0.247538008691569
"Mecom",2927.15438970641,0.380882576029277,0.255036488243725,1.4934434
6235386,0.135321143298919,0.247582964440492,0.531715711007119

"Slc2a2",3694.72987931521,2.19581067815968,0.745782317706909,2.9443050
9550192,0.00323680745699498,0.0143012172663694,9.32650959791405
"Tnik",200.608543608579,-1.12262034921421,0.302811514968686,-3.7073238
4245791,0.000209461020738192,0.00175521653955485,-7.12320915375126
"Pld1",832.879391093843,0.0910977046092835,0.146725423209813,0.6208719
83984781,0.534683855883202,0.666636878171889,0.0369410112339989
"Nceh1",5709.00199512363,1.36514707797271,0.438375759396793,3.11410256
774042,0.00184505338504722,0.00938555984911316,6.37330238217826
"Ect2",172.390216896141,-0.111508633156351,0.305648194747476,-0.364826
735680471,0.715240746434157,0.811177528732736,-0.0233352274255369
"Lrrc34",31.453642454987,1.72568037070714,0.411459301986736,4.19404874
886696,2.74019052813247e-05,0.000363457380391339,13.6671271226152
"Lrriq4",5.17078236158579,2.04514995447904,0.884181628416416,2.3130428
0563027,0.0207202857810823,0.0590217870211165,5.78746480029571
"Sec62",19136.398447201,1.06621446128482,0.151619598538578,7.032167817
10138,2.0334900922771e-12,1.20148453997633e-09,21.8997336235107
"Dcun1d1",2226.03322331965,-0.299296183687095,0.138017906495054,-2.168
53154266486,0.0301182673780213,0.0789309109323466,-0.759967588331496
"Mccc1",4376.48081217628,1.01210847008118,0.398379288238931,2.54056498
407658,0.0110673524703615,0.0367116771805219,3.34467477674816
"Acad9",4673.47591174963,0.99370231429793,0.145351949504399,6.83652553
464965,8.11369018812543e-12,3.46090133983301e-09,19.3590469237955
"Anxa5",9257.36028018898,-0.963867559532544,0.278429839887865,-3.46179
691056366,0.000536581893233809,0.00365618261225184,-5.40858442566314
"1810062G17Rik",24.5848656524951,-0.708793323161637,0.523162895761362,
-1.35482338083271,0.175473835475055,0.30179638259343,-0.84913632823299
5
"Exosc9",1084.59096138917,-0.0138458934244015,0.139969219435165,-0.098
9209876305346,0.921201004914906,0.951510982800244,-0.00068819697484326
7
"Ccna2",389.121759316759,-0.505487284318473,0.38517501178423,-1.312357
41897411,0.189399566920266,0.318716006395577,-0.578001879183256
"Trpc3",179.595222688625,0.370380308342749,0.499756702426421,0.7411212
42685646,0.458619930611584,0.59837870764619,0.190201931274918
"Il21",4.39670429876253,-2.20381700136173,1.72932302345755,-1.27438134
545592,0.202528300209487,0.334567211512715,-2.41299777584607
"Adad1",0.398340086055756,2.29058749941777,3.59814418095252,0.63660247
7339135,0.524383811125015,NA,NA
"Il2",7.04870600356169,-0.347162085454865,0.642100976734112,-0.5406658
73490209,0.588737904682733,0.712177251758292,-0.117836688375204
"Spata5",482.633938123192,0.212563785260593,0.358976812870976,0.592137
925457022,0.553758232531179,0.683408951066076,0.0809149219427488
"Slc7a11",82.3908816089374,-0.561856939237684,0.237486439871923,-2.365
84850714295,0.0179888035930032,0.0529744015976767,-1.65070561174454
"Rab33b",1628.94961259396,0.0285318453134615,0.179767346559387,0.15871
5394422512,0.873893108928609,0.921235285353051,0.0023407471138524
"Cog6",1570.67725171012,-0.0732364866882955,0.173202919724054,-0.42283
6328654132,0.67241468016221,0.778547591080925,-0.0183329350876352
"Stoml3",2.35494381944182,0.941457913886462,1.04145905974821,0.9039797
62885808,0.366006132295921,0.51105611339916,0.631977993251668

"Ufm1",4035.99506114655,0.198726721666749,0.208146984127241,0.95474225
8217235,0.339708054040378,0.484360339135596,0.14406219662864
"Trpc4",27.2668599081055,-0.0501124982252275,0.608645756645814,-0.0823
34424709362,0.934380780380032,0.959022817068478,-0.00209672756297996
"Postn",1509.16262793697,-2.89659485579754,0.678458148183252,-4.269378
8313309,1.96018099529196e-05,0.000284160662053552,-23.6535089200546
"Supt20",3226.08336353045,0.0291100088355493,0.177967556342834,0.16356
9188866493,0.870070301694456,0.918485014358499,0.00247521502141779
"Exosc8",985.360515512217,-0.145374296310141,0.236198412812522,-0.6154
75331011343,0.538240855921883,0.669470541513452,-0.0583340699291529
"Aadac",3519.4088104662,2.02351370378304,0.673006669296179,3.006677045
11637,0.00264120200914879,0.0123112973402 78,8.89787126114555
"Sucnr1",850.25804407877,0.982120901411365,0.585403681856862,1.6776814
5614688,0.0934093014434761,0.187221891400586,1.64550505205675
"Mbnl1",8391.27543131833,-0.551578500617578,0.174570672034121,-3.15962
867181819,0.00157970329295547,0.00837189478915665,-2.638131044014
"P2ry1",240.049651232518,-0.157320368387258,0.286254176553718,-0.54958
2787861037,0.582605567081665,0.706841101795676,-0.0545822055761024
"Dhx36",2202.22494423783,0.10239566405156,0.0921731586775483,1.1109054
4710281,0.266609050606823,0.408546311594768,0.0916594788017952
"Gfm1",3190.79221548889,0.151875528853654,0.434560068976206,0.34949260
1129832,0.726719524503,0.820283506354029,0.0300873409826288
"Mfsd1",7336.85587968284,0.795416569776468,0.231498661510655,3.4359445
7344134,0.000590491764392681,0.00391213334085236,4.40952891802297
"Il12a",6.97149668224735,-1.04348321084474,0.688018800845152,-1.516649
26825101,0.129355259226965,0.239317662435465,-1.49214288145056
"Schip1",1012.09572448129,0.0118675720412083,0.257958923506593,0.04600
56658629411,0.963305733987405,0.978046740493088,0.000263434206338703
"Ift80",966.712111989887,-0.0368367864688566,0.165664810663537,-0.2223
5733902278,0.824035719034086,0.887241065201093,-0.00440710000424604
"Kpna4",3657.55921566755,-0.308920160385169,0.161792919796574,-1.90935
524727276,0.0562162797998532,0.127187865344342,-0.637021183062894
"Ppm1l",971.082232812162,0.176030805412076,0.211047521598887,0.8340813
67449732,0.404235133992383,0.548100444892579,0.105846745073704
"Nmd3",940.269440202117,-0.0304021502809146,0.13129783984114,-0.231551
031743544,0.816886744812433,0.882761056939136,-0.00379116998977883
"Bche",463.60987169973,-3.56221701518793,0.81919376614952,-4.348442532
62756,1.37107734008937e-05,0.000217520471741762,-30.0409511880122
"Ccna1",1.5798140900808,0.670516905451927,1.64095899516947,0.408612833
974367,0.682823810761519,0.786582693030137,0.16096255937919
"Sohlh2",1.01638935049753,-1.7834648032824,2.2194830194648,-0.80354964
9914628,0.421657111617515,NA,NA
"Smad9",75.1245302249472,-0.275850413858562,0.2386565698672,-1.1558467
2155499,0.247743858488927,0.387060076347524,-0.261830416421046
"Dclk1",461.053257590901,-1.5445524515575,0.385122650508701,-4.0105468
9854604,6.05782656438559e-05,0.000671492338558216,-11.2845124926038
"Nbea",851.832384712828,0.0754413876592786,0.148885497702607,0.5067074
28348528,0.612360117662097,0.731784602486857,0.0235580117542364
"Tm4sf1",4408.02252455926,-0.698419395034132,0.222611554344321,-3.1373
9058644666,0.00170458881674449,0.00888190078805025,-3.29915141810808

"Tm4sf4",82.7858614211477,1.26799501369587,0.532749594809137,2.3800956
8857607,0.0173081429250381,0.0514719287858587,3.7617845085419
"Wwtr1",3248.6800175105,-0.314067125301375,0.190450761454888,-1.649072
56291421,0.0991327698188406,0.19599378887931,-0.511826497473462
"Ppid",2659.26431617267,-0.150900939316993,0.106786802236869,-1.413104
76721901,0.15762492174852,0.278385029370711,-0.192964595131901
"Pfn2",2109.37300689166,0.246297427177046,0.286059723086893,0.86100001
9573642,0.389238032284456,0.533410398754732,0.154789108684835
"Tsc22d2",1796.46525562373,-0.0668492187944725,0.104014438206436,-0.64
2691725756361,0.520424151023009,0.653858502264749,-0.028401847105309
"Serp1",10069.0795236306,0.0800259531413046,0.190029717303871,0.421123
36047597,0.673665004133486,0.779532359085739,0.0199313502156033
"Etfdh",5266.44610691087,-0.271043643522248,0.459247324413172,-0.59019
10128025,0.555062596698125,0.684542094270717,-0.102726933995969
"Eif2a",2392.69850735355,0.153237667622543,0.0945832483405923,1.620135
38666739,0.105203197396529,0.204858877742686,0.242948198852627
"4930579G24Rik",92.2624703706831,-0.465341490056899,0.298635641678596,
-1.55822489050962,0.119179946588757,0.225346256651241,-0.6934133308682
76
"Mme",5220.36000792241,0.205554825318547,0.221984072943567,0.925989070
264527,0.354451660315546,0.499573404137091,0.142655200154179
"Slc33a1",4708.72479339775,1.18471962061787,0.244003320620312,4.855342
20438493,1.20179021804396e-06,3.17858608063866e-05,12.2695357140193
"Gmps",2208.73307206054,0.178841203488361,0.13407967269966,1.333842780
84395,0.18225538715312,0.310312656117172,0.209275491279095
"Vmn2r1",859.88794661421,0.548893898330134,0.273892181227346,2.0040509
9506846,0.0450645980754469,0.10724637291286,1.22547508546221
"Kcnab1",204.981462221287,-0.713546110406439,0.377635018783865,-1.8895
1255819532,0.0588231816470014,0.131564182111945,-1.44725736893011
"Ssr3",8386.22527456462,-0.148503386079706,0.193468881806951,-0.767582
800359007,0.442735083964992,0.584141877598417,-0.0798371110030703
"Ccnl1",6070.83915168236,-0.325283507864651,0.171707440465219,-1.89440
543160703,0.05817216033251,0.130509886357121,-0.662376855558176
"Veph1",1197.46208425364,1.04093356229403,0.567017139938047,1.83580616
700186,0.066386329068337,0.144062397793482,2.01681789057927
"Ptx3",60.7842066223652,-1.23244678448237,0.465853283219536,-2.6455685
2742319,0.00815537665234556,0.0289062959402715,-4.36741656166054
"Shox2",2.79210651529833,-4.75748900379914,1.89867919330519,-2.5056834
3539773,0.0122214985789029,0.0395051026676806,-15.3729952197517
"Serpini1",476.194534343348,-0.12310225422963,0.279569400082895,-0.440
328069499483,0.659699513655017,0.76866386502005,-0.0323883891742733
"Pdcd10",1679.68979314025,-0.115511831919692,0.180311174984765,-0.6406
24919278861,0.521766405294592,0.655122761957238,-0.0488537238024254
"Wnt2b",13.3420216220798,-2.77234740006696,0.910597772124677,-3.044535
67198864,0.00233039792071992,0.0112387085476619,-12.4433800591949
"Ptpn22",597.101694599949,-0.690764402968041,0.460168995045946,-1.5011
1026689026,0.133327043750558,0.244761387726992,-0.972231190123643
"Dclre1b",421.836024996243,-0.173453090180782,0.210032735257374,-0.825
838362616341,0.408895789100095,0.552662769614041,-0.102858945795324
"Olfml3",3440.47001782958,-1.3525252147703,0.34955419713443,-3.8692861

532146,0.000109154472181857,0.00106149321626027,-9.26219907059518
"Syt6",3.14167925495401,0.41571394228431,1.2961075514969,0.32074031341
3185,0.748407194308879,0.836345004550785,0.07429392898464
"Nras",3797.47048103693,-0.23841114325894,0.100404529944774,-2.3745058
4540433,0.0175724579704744,0.0520405581266944,-0.704679421550736
"Sike1",2118.99806276497,-0.332463883094589,0.168456949431003,-1.97358
366168658,0.0484291030283964,0.113657998416908,-0.722963111624169
"Sycp1",1.38597590354255,0.207724189814709,2.12786328392396,0.09762102
26399736,0.922233230585452,0.952088732371109,0.0101986433261794
"Tshb",1.07127345139979,2.56443088348682,2.80459793877601,0.9143666719
67959,0.360524208103341,NA,NA
"Tspan2",624.804883217057,-1.03275971754392,0.252566420693144,-4.08906
185830092,4.33121348794429e-05,0.000519053476262679,-7.81128188597816
"Ngf",393.510144365715,-0.614889434828767,0.335844567914546,-1.8308750
3438562,0.0671191974618486,0.145297003676155,-1.18610655061941
"Vangl1",1152.60706430316,0.229425313438914,0.219928805101773,1.043179
92057814,0.296864971325006,0.441181246713627,0.187738618744051
"Casq2",26.2994057235729,-2.36509746143652,0.568022333432901,-4.163740
26553289,3.1307624509213e-05,0.000403727554682959,-18.4826933440533
"Cd2",280.814114785694,-1.64091013627423,0.518603300431508,-3.16409505
089709,0.00155565980423607,0.0082716811734374,-7.86802873626092
"Ptgfrn",1650.17902300527,-1.47069744089655,0.261846014521422,-5.61665
00894984,1.94695135094228e-08,1.32733067974379e-06,-19.9019787488543
"Gdap2",656.6870563233,-0.0148587777874665,0.16795641766209,-0.0884680
561439499,0.92950467226861,0.956333813513448,-0.000663418418865256
"Spag17",11.2800827023483,-0.261543315892285,0.892641714996187,-0.2929
99208415218,0.769522756748359,0.850913327912409,-0.0422248614023895
"Tbx15",13.0251497244535,0.624402066383119,0.725455353864895,0.8607036
43658552,0.389401285806163,0.533596617516941,0.392196380969366
"Hsd3b6",0.141341260214298,-0.569882494411888,4.78471990596623,-0.1191
04671874582,0.905192429472853,NA,NA
"Hao2",7826.0872543974,2.06604604082026,0.799272417784753,2.5849084678
1185,0.00974048520508608,0.0330986372479555,7.04162862473993
"Hsd3b1",3.11575179662712,0.613465024062151,1.33191633761084,0.4605882
56738837,0.645094038384054,0.757941763206525,0.17002104976264
"Hmgcs2",2001.65059678708,1.90933761031498,0.590167799421251,3.2352453
1868289,0.0012153825787713,0.00682532532283489,9.52208675713796
"Reg4",0.194022338728155,0.863911481469598,4.78471990596623,0.18055633
3170591,0.856715833519269,NA,NA
"Notch2",2822.64449260811,-0.620794612721622,0.165671796269682,-3.7471
3515939121,0.00017886571942556,0.00155196078648198,-4.01544613957962
"Sec22b",2636.08015921802,-0.473459083788886,0.214837326662224,-2.2038
0271503414,0.0275382215652875,0.0737352710903564,-1.23443756762422
"Prpf38b",8466.11007559785,0.167626719176565,0.161128249237616,1.04033
104045812,0.298186127723181,0.442569317069848,0.136642290997619
"Stxbp3",2279.09669890621,-0.239670363257417,0.123860980837621,-1.9349
9487600231,0.0529909244691397,0.121512059896423,-0.505163233924062
"Gpsm2",271.65918621534,-0.660533635958906,0.234416250501504,-2.817780
91128827,0.00483567936625331,0.0193645669096749,-2.60534964419162
"Clcc1",1744.33574023537,-0.00266276769520512,0.132320275543499,-0.020

1236559118996,0.983944729276125,0.989723009277618,-2.75068259471672e-0
5
"1700013F07Rik",1.97526008502235,-0.560266202555178,1.10368042499495,-
0.507634447315436,0.611709728128043,0.731321128152589,-0.1753087603787
1
"Sypl2",1344.31887375376,1.75491367490346,0.619405769224651,2.83322139
072129,0.00460814585852808,0.0186371350237669,6.98911776611515
"Ampd2",2256.65842584258,-0.0482625882522045,0.170838933352628,-0.2825
03451087381,0.77755750072358,0.856253029995954,-0.0074898397354358
"Gstm4",729.469326436633,-0.964968229305289,0.432676210007144,-2.23023
176913137,0.0257320602006103,0.0700340186755305,-2.56563260671509
"Ahcyl1",8460.24393317577,0.240758821819325,0.24563533245455,0.9801473
56707623,0.327013385839552,0.4718038973258,0.180856065126518
"Slc6a17",739.85813963991,0.835109283563197,0.308474105504168,2.707226
53429305,0.00678479427019724,0.0251809053172351,3.07459620909327
"Kcnc4",1.8042731952566,-0.820569974488994,1.45585723078341,-0.5636335
4670941,0.573003551346694,0.6991503907483,-0.293673302888823
"Slc16a4",2386.49047146457,2.28098881369155,0.727769573602314,3.134218
43455355,0.00172312533700058,0.0089355047395844,10.7610725720582
"Dram2",4889.23185452221,0.0446873071978388,0.307261254976187,0.145437
494881358,0.884365462727599,0.927951660474155,0.00334152188651181
"Dennd2d",1052.99298754397,-0.861346996246427,0.267096718266773,-3.224
85054041781,0.00126038449951047,0.00702488188651019,-4.27081412168445
"Chil6",20.1159604657225,-4.70867305315606,1.37729451255759,-3.4187844
4314152,0.000629015352464325,0.00412130382311354,-25.858081622408
"Ddx20",797.816224738503,0.0511478068764637,0.108303891117297,0.472261
950598513,0.636739825587389,0.751204279016331,0.0146322446426138
"S100a11",14961.9147284176,-0.420697553696296,0.249718857412493,-1.684
68476131691,0.0920494081147136,0.185315370100226,-0.709168268463577
"Tchhl1",60.949792010707,1.71983580824753,0.697922586231512,2.46422145
116971,0.0137311245309477,0.0432760757878048,5.40055156088931
"Crct1",1.3858135589868,1.22525904640655,1.68325186204157,0.7279118912
8433,0.466667523480033,0.605337893480938,0.615041415291232
"Npr1",3065.94934676519,-0.708013775426844,0.176359886071932,-4.014596
4663306,5.9547585779766e-05,0.000662361615482035,-5.18244765616036
"Slc27a3",583.311670460196,-0.411291348559418,0.233636901195591,-1.760
3869356883,0.0783422229111682,0.163615752176731,-0.744533819457697
"Ints3",2274.06926247145,-0.180767862438334,0.101415835452714,-1.78244
217612957,0.0746771528628761,0.157701194251095,-0.333887860963273
"Rab13",1139.35919268488,-0.46332108994754,0.177483317430852,-2.610505
01339684,0.00904086467821259,0.0312717366499537,-1.60542637443875
"Crtc2",2935.97571405688,-0.0879125558102602,0.113739170788841,-0.7729
3121798357,0.439563088168546,0.581489980400399,-0.0476628064484076
"Jtb",3086.12825719864,0.419359140403809,0.140960999198623,2.975001190
31439,0.00292987410972325,0.0132821867917656,1.81218985503535
"Creb3l4",52.0880435780992,-0.673018743255566,0.294261239958806,-2.287
14710557796,0.0221872381804806,0.0624072086040118,-1.8670041254622
"Nup210l",64.9184027170769,-1.01320569034934,0.369146326489318,-2.7447
2646114402,0.00605613536343423,0.0230090661177398,-3.82167706882394
"Tpm3",8298.98938355633,-0.860672553948133,0.132257551158111,-6.507549

44735983,7.63866120682652e-11,1.93426774299615e-08,-15.2863638465107
"4933434E20Rik",2897.20342855284,0.3070856690415,0.13158343543259,2.33
377148143256,0.0196076908580953,0.0565715827687395,0.882026349535711
"Hax1",3623.55480695803,0.919295624173621,0.176407477124963,5.21120555
180558,1.87617434921269e-07,7.3017260401096e-06,10.8728768758914
"Il6ra",1354.07429896603,-0.712179889884156,0.32484955300771,-2.192337
60148426,0.028355138583994,0.0753843048964704,-1.84109624610395
"Chrnb2",157.710478611467,0.565291185222317,0.313311809770539,1.804244
74148076,0.0711929482135543,0.152072754630574,1.06466728357611
"Adar",2656.95018246412,-0.41596435991783,0.195276056647517,-2.1301349
8459091,0.0331604707681102,0.0849287874736127,-1.02574405553388
"Pmvk",1587.1241927272,-2.26840347529481,0.565092159174943,-4.01421863
40132,5.9643043222204e-05,0.000663010294610339,-16.6018099747687
"Slc50a1",1685.87965421478,-0.406979760136541,0.118582363844888,-3.432
04290200263,0.000599052784134813,0.00395809257372436,-2.25140920174138
"Efna1",1817.72850031851,0.195136341016522,0.118684000854243,1.6441671
9702743,0.100141670731969,0.197587765222823,0.316427707263481
"Gask1b",576.657225960845,-1.49030512450316,0.242457811482967,-6.14665
749636138,7.91327459029499e-10,1.30756803357264e-07,-23.6212269870346
"Tmem144",482.134964320606,1.4433648586393,0.435431363462484,3.3147930
5294383,0.000917109931525512,0.00550548320347427,7.50839949245167
"Slc35a3",4790.27133894314,0.861436557676665,0.213401915996967,4.03668
614525891,5.4211514985856e-05,0.000614187169781651,6.37050496886557
"Sass6",175.402789405892,-0.596627618880126,0.255273308489401,-2.33721
11342573,0.0194282085140889,0.0561701081861958,-1.71791199296637
"Lrrc39",124.724631900545,-3.28958617802832,0.822813310043187,-3.99797
394849588,6.3886980532158e-05,0.00069858672145787,-23.9036175398305
"Vcam1",5503.18363712444,-1.52681831318079,0.4584206750459,-3.33060526
344698,0.00086657392257517,0.00527805586330754,-8.0069367149417
"Extl2",717.41562352091,-0.255148265544796,0.208221333247737,-1.225370
43426394,0.220435681350295,0.356330203529104,-0.263286842042384
"Olfm3",342.782203791781,1.97357257647956,0.789258168724398,2.50054121
032317,0.0124003704878165,0.0399112617648476,6.35706820244665
"Col11a1",20.7467563509991,-1.13235830745113,0.701489903489099,-1.6142
1896711408,0.106479939310486,0.206540574679254,-1.78602165034755
"Neurog2",27.4815091613363,1.64955133309057,0.905888916912845,1.820920
09549254,0.0686190039993405,0.147914776267314,3.15248890586796
"Larp7",2549.31399938325,0.222843215838924,0.205800271067808,1.0828130
3364028,0.278891419179866,0.422650776555964,0.191914589366733
"Ndst4",3.37870974495773,0.845367247273033,1.3138215808732,0.643441438
000418,0.519937702649621,0.653366896609661,0.359802201982592
"Ndst3",125.746572825658,-0.181083480124394,0.457677649607303,-0.39565
7249769062,0.692357896948568,0.79446764799336,-0.041664232855047
"Prss12",61.3549687968341,1.45379496390273,0.311979432539208,4.6599064
3059467,3.16353159659894e-06,6.92284389118812e-05,13.9245918178546
"Rnpc3",1305.9898529729,0.0979472380074051,0.108876814770019,0.8996152
04709098,0.368325062853537,0.513374942849257,0.0653062051706425
"Cyp2u1",449.255399596448,0.413782395550384,0.289434076889536,1.429625
70267186,0.152824474565607,0.272001059340959,0.538723707711163
"Hadh",14771.4613114258,0.16208842822313,0.543979681461359,0.297967798

701033,0.765727740800111,0.848400925680223,0.026647656136521
"Lef1",145.013044112501,-0.696408315300226,0.30602406433189,-2.2756652
0567793,0.0228660553817264,0.0638104910192588,-1.91640263303605
"Trim2",3553.42784365617,0.575227198624557,0.267448991030047,2.1507921
8062906,0.0314926065670072,0.0815353661988457,1.44193260974328
"Mcub",277.882304672291,-1.30436925664024,0.410416355208228,-3.1781610
0671344,0.0014821242154739,0.00796978983819916,-6.3028389749116
"Tlr2",541.855057175118,-1.22756164721688,0.371890789233178,-3.3008659
6053659,0.000963869190355975,0.00570711250335888,-6.34163511844183
"Sfrp2",744.640179148792,-0.817942475524672,0.276023994184429,-2.96330
207792788,0.00304357722049972,0.0136799604991479,-3.51046453712103
"Casp6",924.069429297613,-0.145743874941479,0.171477825042776,-0.84992
8408557331,0.395364890134503,0.53972027080043,-0.0898808730163369
"Plrg1",1207.61723315609,-0.207007912671872,0.0910310437305992,-2.2740
364625995,0.0229637943004347,0.064028036789629,-0.568947630317354
"Pla2g12a",1385.70797563986,-0.198335560205459,0.384557807944171,-0.51
5749663921145,0.606029262906803,0.726745051278228,-0.0633046546477226
"Fga",730.340327724513,1.45476427663492,0.688323954046179,2.1134877960
9278,0.0345590384662683,0.0875789098018326,3.54266388352235
"Lrat",97.4831544042272,2.31717016341005,0.821764951791846,2.819748102
371,0.00480613612330429,0.0192660448462556,9.15145748728195
"Npy2r",4.01353681066133,-0.96295763646337,0.969601594914633,-0.993147
743891811,0.320637959875611,0.465887607245795,-0.735517500560791
"Gucy1b1",799.070887669299,-1.15260287458781,0.217138203408022,-5.3081
5331663199,1.10741476019959e-07,4.89622970393912e-06,-14.0929273267481
"Snx7",2458.21440435102,0.626837355162507,0.372036214195418,1.68488263
035934,0.0920112184084604,0.185257614263544,1.05685274097639
"Asic5",2.4597028870328,1.25510074105632,1.03488177358312,1.2127962566
3009,0.225207726976738,0.361229044142188,1.27799760729952
"Gar1",810.058538971635,-0.468759144461203,0.20437138174249,-2.2936633
3223623,0.0218098445030074,0.0616034112877935,-1.30644956342711
"Tdo2",5.95249230611705,0.587843431128821,0.615424008036542,0.95518443
1306614,0.33948443753344,0.484112306225921,0.426444175511454
"Rrh",2.47484170688894,0.740824428849473,0.979895443949485,0.756023954
82478,0.449634833482046,0.590445824246468,0.390323643827524
"Ppa2",5100.82744831482,1.40233437162304,0.372939238587257,3.760222112
68321,0.000169762560501319,0.00149572092392893,9.12239118149001
"Ctso",1924.86629367554,-0.593058893266864,0.143934343802195,-4.120343
18982194,3.78308471440201e-05,0.000466556519680015,-4.54883963226496
"Ints12",686.851941405185,-0.251662455156926,0.146913162859663,-1.7130
014102094,0.0867122801549073,0.176742216021366,-0.436146893210573
"Egf",23544.3758255464,2.77334199243458,0.738299912845326,3.7563894349
4714,0.000172382377641374,0.00151030072091436,18.0140945243624
"Gstcd",462.668712036362,-0.331643683837901,0.194475599406817,-1.70532
285206715,0.0881342278663533,0.179153583709149,-0.57026523819251
"Pdgfc",693.726558500458,-0.332484377662596,0.296503617462107,-1.12135
015588836,0.262138843931773,0.403758841850202,-0.301542552050373
"Glrb",52.8934624709915,-1.14795978376455,0.353878133997895,-3.2439409
8837251,0.00117888167191305,0.00666642541733195,-5.75204935116555
"Pitx2",195.676624713521,1.13900768615476,0.615915896516013,1.84929093

825581,0.0644158135320706,0.140773316772956,2.23314344098558
"Enpep",5226.36823348285,-0.196698399729668,0.184005929360773,-1.06897859440176,0.285079314913115,0.42925913060282,-0.166346756456556
"Alpk1",833.443733620132,-0.581753584894815,0.312286899285639,-1.86288181228731,0.062478896230948,0.137620144454476,-1.15376743122782
"Aimp1",2161.07276070869,0.110370440780234,0.117165857885471,0.942001729617521,0.346191754384904,0.490912496508863,0.0785273966928934
"Tbck",1322.38853842445,0.447316646363109,0.144148965053072,3.10315544893727,0.0019146904054652,0.00965415917397479,2.07571316009991
"Dkk2",4190.34684156482,-0.0493590359533857,0.380463045013315,-0.129734113734117,0.896776791784804,0.93639768040594,-0.00324363005996269
"Papss1",3647.0845424749,0.464753335448957,0.167534879021127,2.77406912616895,0.00553599136240588,0.0214253194887237,1.78613160799383
"Kcnq5",23.0874380023248,-0.284268607067607,0.833229740849507,-0.341164739004377,0.732979566742991,0.825266497622903,-0.0545934782346947
"Fubp1",5493.38861502691,-0.320923428816416,0.0832087032567567,-3.85684929887856,0.000114857924819542,0.00110811470466671,-2.18391447600355
"Dnajb4",2337.04800677784,-0.487434953875938,0.151660351068462,-3.21399067351428,0.0013090392514731,0.00722435531424356,-2.40319922669987
"Ptgfr",674.767705954933,1.97787474367486,0.591642346060351,3.34302430656832,0.000828706407320063,0.00509880641524979,10.4407039353968
"Ifi44",889.036402541925,-0.49074003444319,0.305614449015698,-1.60574879893189,0.108329114542897,0.209336082790626,-0.76742626721018
"Efna3",57.7575066503424,1.69808626047842,0.372609510868043,4.557281043424,5.18200653010448e-06,0.000102265954781353,15.6019040430786
"Efna4",483.82286448232,0.522415152594515,0.343211035866902,1.52213972745652,0.127974073480337,0.237448896983915,0.751129994893856
"Adam15",5001.65292723385,0.030018261124901,0.168213464653706,0.178453378786879,0.858366931777922,0.910331163220339,0.00282012047616932
"Zbtb7b",3443.78210025776,-0.349290334835312,0.251069617056651,-1.39120909542988,0.16416202994697,0.286755092898595,-0.436307905079288
"Cks1b",388.486268954801,-0.954392496003994,0.276302340567121,-3.45416001198205,0.000552009747754912,0.00373329381259982,-5.33549724897924
"Thbs3",932.00676234104,-0.84412807759703,0.307226130838494,-2.7475790398857,0.00600370362588377,0.0228590508866755,-3.18946039959139
"Gba",1776.884447848,0.0089972316656087,0.0860682877420362,0.104535966749742,0.916744027283571,0.948913045812766,0.000471797839461505
"Scamp3",2167.85060538038,-0.0614991074156553,0.123675863041458,-0.497260386167993,0.619005442928177,0.73719570768468,-0.0187511932024765
"Hcn3",95.169286550004,0.416039558754969,0.410168459018098,1.01431387423335,0.310433005791783,0.455647602147561,0.327021891933742
"Ash1l",2767.41357273536,0.0487612860175662,0.173013243144022,0.281835570107056,0.77806959502902,0.856623431048889,0.00754614354423086
"Rit1",1684.08447869604,-0.316910697632865,0.154241248078645,-2.05464298026996,0.0399135010798029,0.0977311872477706,-0.736986778657823
"Arhgef2",4347.81909537285,-0.162121830572886,0.20239458506808,-0.801018616769578,0.423120868318747,0.566349330025327,-0.0921734265151142
"Khdc4",4586.75645617088,-0.0290356265355816,0.104726993787841,-0.277250644608427,0.781587654398563,0.858896245587735,-0.00441652638355096
"Lamtor2",5212.80485934513,0.38955638808735,0.251727545157018,1.547531

82789098,0.121735055757518,0.22913613606844,0.573987563770907
"Lmna",5685.38922594743,-0.26953933158772,0.104077486146968,-2.5897947
9199856,0.00960331593877736,0.0327695929601472,-0.921353962362113
"Sema4a",5042.14749104328,0.148244219494221,0.125565340100094,1.180614
16769985,0.237756032808773,0.375852693992659,0.145065564516149
"Pmf1",820.102418731633,-0.0926571963845156,0.177670801700773,-0.52151
0543643325,0.602011162574465,0.723601466855331,-0.0299759464022024
"Iqgap3",190.015402183096,-0.595322673965687,0.388460829835633,-1.5325
1660976367,0.125395002016674,0.234123503717429,-0.864352865401735
"Gpatch4",1402.9085861853,0.406398744073222,0.220057258702077,1.846786
3612871,0.064778109322059,0.141438250342834,0.794872072859087
"Naxe",2936.24240988615,0.568169418213495,0.233982546518405,2.42825555
438941,0.0151716486460355,0.0465486711723683,1.74272152401595
"Sh2d2a",214.383197183296,-1.49780595852087,0.490250194101431,-3.05518
687507339,0.00224920328963458,0.0109473204546418,-6.76208548747319
"Ntrk1",88.3336734525185,0.351019625122853,0.347556630597465,1.0099638
2810892,0.312512620391504,0.45755839134377,0.274444964558879
"Pear1",3092.2884932374,-0.305284588058124,0.210816703323655,-1.448104
36386265,0.147587865309512,0.26467501307899,-0.405800323665944
"Cd1d1",1223.71425445397,-3.13743842352336,0.699633608593958,-4.484402
09987713,7.3118666513318e-06,0.000132581880464041,-28.0120232829456
"Dclk2",246.123814886994,0.351489697264785,0.215984206517703,1.6273861
0814109,0.103655120068387,0.202755570936336,0.560891128853481
"Lrba",2430.41250962374,0.469756832048566,0.195444947027883,2.40352508
055145,0.0162378531172572,0.0490709330564602,1.41607652772826
"Rps3a1",32510.0626369671,-0.274417829345523,0.257532992593544,-1.0655
6378109824,0.286620879155643,0.430750310500328,-0.2311220180938
"Sh3d19",3080.82783171101,-0.0184622442420635,0.176529815109403,-0.104
584283570578,0.916705686200172,0.948913045812766,-0.000968125226597338
"Gatb",2126.54224745824,0.944685748664707,0.3293156631013,2.8686329091
3106,0.00412249953378513,0.0171897619879333,3.83867509197746
"Fbxw7",1546.25670078959,0.509451419340012,0.172037370048313,2.9612834
6531305,0.00306359831544841,0.0137436174575559,2.18411031680232
"Fmo5",7933.75431714194,-1.51221340531409,0.537109410472336,-2.8154662
2686102,0.00487065160859656,0.0194846050604054,-5.95529389926257
"Chd1l",923.224782162353,-0.28989805292013,0.17908264081828,-1.6187948
2899908,0.105491415105319,0.205297096688643,-0.458994745572752
"Acp6",3090.65075166977,0.917215163279151,0.17550518637447,5.226142783
73583,1.73082719799502e-07,6.91550588248094e-06,10.8981161649792
"Gpr89",1048.57841005051,0.030589772541211,0.172952137240491,0.1768684
27469478,0.859611738830458,0.911029575198301,0.0028503524880521
"Rnf115",1819.19374285671,-0.0683681290830859,0.182554236582103,-0.374
508586396665,0.708025969619464,0.806372100212635,-0.0147135037072499
"Polr3c",1794.74454557771,0.312485045276189,0.0907293817289059,3.44414
388505232,0.000572870747606375,0.00382630759142687,1.73924645672377
"Nudt17",127.241664920117,-0.324735441884986,0.28657486021486,-1.13316
095362139,0.25714666810051,0.398019031088651,-0.299164298254026
"Pias3",1339.51873600649,-0.393511378796865,0.103835586019659,-3.78975
449440198,0.000150796247903649,0.00136782233847703,-2.59502469328261
"Pex11b",1396.37836873267,0.231305957912428,0.135902719486626,1.701996

53683303,0.0887560174721538,0.180130349017643,0.39647565085462
"Polr3gl",1822.23001880247,-0.117895330936585,0.174488815283855,-0.675
66124937461,0.4992557442634,0.634706168197677,-0.0535944056670395
"Rprd2",1563.10906620164,-0.035892848947527,0.107761467391557,-0.33307
6839211073,0.739076281797044,0.82981166316243,-0.00669604484151271
"Tars2",2110.0578421344,0.303802964512101,0.178125072828647,1.70555980
518449,0.0880900684628795,0.179082489301348,0.522513375484192
"Ecm1",6185.84316917148,-0.0721211284572768,0.408712618058197,-0.17645
9265681412,0.859933147758309,0.911149685631535,-0.0067107329684442
"Hormad1",0.552162964512389,0.335098517720611,3.11376845705249,0.10761
8315986738,0.914298467317918,NA,NA
"Ctsk",1166.32822563283,-1.37821352041307,0.360332900343803,-3.8248339
7740833,0.000130860241150561,0.00122551055809493,-9.24009161107677
"Mettl14",838.526062417475,-0.105746808682463,0.123337311747376,-0.857
378900061138,0.391235516459044,0.535695933983037,-0.0660059488943507
"Bnipl",106.987949484958,-1.62333313280148,0.501158347899571,-3.239162
11234456,0.00119881414320127,0.00674977711930073,-8.11381800329511
"Myoz2",1.657335033859,-2.02858552816542,1.51968257438508,-1.334874507
58344,0.181917422501183,0.31000051598742,-2.3758414705823
"Bcar3",1229.44289776044,-0.10650375744032,0.221889737375784,-0.479985
053386896,0.631238021084868,0.74661200761491,-0.0311214235092238
"Gclm",9867.6209254319,1.50484374655956,0.292720104954581,5.1408964436
9681,2.73430734145277e-07,9.90957705272231e-06,17.3388230249728
"Abca4",274.307308726249,1.09600281623579,0.445986870805376,2.45747775
995423,0.0139916473746087,0.043944771345058,3.42481330691871
"Pip5k1a",1468.5041993775,0.0267387979278648,0.137409257343374,0.19459
2405525101,0.845712048921057,0.90127792211635,0.00277927368788038
"Abcd3",10272.2409688072,0.862244115532264,0.336932741369024,2.5590986
2611985,0.010494396360174,0.0352428074803088,2.88463235437387
"F3",2166.90064641514,-0.559844441372141,0.181967119195008,-3.07662419
369388,0.00209359067962549,0.0103317719745224,-2.55990632861814
"Tmem56",680.481804750374,0.368079165452678,0.540225888658582,0.681343
069964018,0.49565443652985,0.631527818300923,0.169174075114447
"Rwdd3",332.078886079023,-0.126356474934549,0.39859097586307,-0.317007
866675729,0.751237633140053,0.838204942544478,-0.0223009887130681
"Ptbp2",1863.22986985666,-0.123944222811468,0.149738351349178,-0.82773
8663439938,0.407818517898053,0.551508751601596,-0.0737589060200995
"Snx27",2976.52373708887,0.580787647012837,0.141666839398736,4.0996725
0965591,4.13735177538213e-05,0.000500745406060837,4.41364505415282
"Celf3",49.2289121010191,-0.955074058296876,0.956618180706082,-0.99838
5852955391,0.318092290969998,0.46319368852375,-0.735034525594463
"Adh5",7343.22747858023,0.717465781082601,0.24233294915773,2.960661286
78035,0.00306979340610253,0.0137628953396613,3.07489981472111
"Riiad1",26.7468188186271,-0.656909447655448,0.476998520360361,-1.3771
7292531467,0.168458790450264,0.292381119476521,-0.807799658553054
"Mrpl9",2765.92082919109,-0.00706300989092869,0.236318998927493,-0.029
8876092188244,0.976156687847161,0.984997314063238,-0.00010676703305985
9
"Oaz3",5.87890837102351,-0.0783115353228375,0.840415292844168,-0.09318
19494357514,0.925759014253985,0.954545175035653,-0.0036430767787817

"Them4",829.425821932634,1.18845092726279,0.175246882928376,6.78158097
538613,1.18867820963877e-11,4.65112049330873e-09,22.8018068854916
"Them5",2.36840316382187,-1.28069060895163,1.58197968870255,-0.8095493
37515188,0.418199235777111,0.561303001251608,-0.739591667715367
"Rap1gds1",2138.1385490586,-0.429636240453547,0.119865623525572,-3.584
31573471012,0.0003379630602511,0.00253934633864198,-2.56744111659843
"Rorc",1737.26099331727,0.482064142154617,0.352363729399634,1.36808672
95166,0.171284930056288,0.296178925703929,0.586571564392551
"Tspan5",1088.97647787744,0.308831666205199,0.123231741122821,2.506104
86706828,0.0122069409731103,0.0394776803937144,0.998150066250185
"Eif4e",3532.32729139146,-0.185545106350348,0.17416389202882,-1.065347
72615006,0.286718602789412,0.430795971117204,-0.156251372213874
"Mttp",622.670531352301,0.748637550203939,0.260822224834513,2.87029815
300033,0.00410084921614568,0.0171143745754299,3.04533518953369
"Dapp1",917.079798196427,-1.01401434992424,0.237868939210695,-4.262911
9770281,2.01780017949319e-05,0.000290783946044037,-8.25704789071371
"Ppp3ca",6368.21141289194,0.382950784278761,0.151124097908447,2.534015
35280467,0.0112763791087347,0.0372466271153837,1.2599823288792
"Nfkb1",3757.45124805511,-0.712613738580979,0.210077974114814,-3.39213
923584161,0.000693491896918257,0.0044318928240289,-3.86160290613452
"Manba",3592.42321616139,0.519714337980046,0.293464792700392,1.7709597
5703852,0.0765673940319805,0.160683569996293,0.950203209792737
"Cisd2",3343.31017838913,0.301650510973251,0.0859180954547233,3.510907
7939491,0.000446579269183357,0.00315943490222681,1.73671121805135
"Bdh2",6500.32778609116,2.17353259178181,0.674311717899258,3.223335045
33067,0.00126707253266191,0.00705263495342333,10.7684498461612
"Tacr3",5.00532402300934,0.429108515401297,0.860946997630149,0.4984145
55811758,0.618191879434914,0.736586730947321,0.131190612231432
"Wls",8221.96744849108,0.336503135312737,0.127956302284498,2.629828537
59368,0.00854279427713711,0.0299097509096102,1.18098155571798
"Depdc1a",49.7318642638453,-1.37899619303822,0.458732989379236,-3.0060
9771907685,0.00264623941703359,0.0123299913150192,-6.06168206432737
"Lrrc7",16.5554891396364,0.976824275263279,0.649482150923391,1.5040048
0424981,0.132580129726846,0.243711451816163,1.37905152749253
"Cth",4028.62687346753,2.02783437386333,0.51587520287901,3.93086227550
062,8.46417499786679e-05,0.000868602547938983,14.2934438697777
"Zranb2",4658.52133973195,0.121809437072282,0.0994561084830264,1.22475
571315029,0.220667275753393,0.35650929594402,0.125633639950439
"Lrriq3",90.8956110564804,0.913985940409015,0.27228119192393,3.3567722
1754033,0.000788580502813266,0.00490550454794146,4.8600263966755
"Adgrl2",1709.18232299415,-0.188351300843989,0.106913114775143,-1.7617
2307055242,0.0781160979579452,0.163353461817248,-0.341262222874047
"Dnase2b",1.84350750534439,-2.08637870862759,1.70427675364747,-1.22420
182295062,0.220876101675977,0.35665774165451,-2.15101193342271
"Uox",23.8819068988928,-0.330632115178106,0.659345870960586,-0.5014547
44376295,0.616051120148983,0.735024154978881,-0.101785730278473
"Rpf1",1207.04402267241,0.319643172368049,0.127677653956085,2.50351696
216153,0.0122965785996724,0.0396885763178965,1.0313900296967
"Spata1",272.211760292332,0.532479134013573,0.266914023678856,1.994946
26274953,0.0460487410750101,0.109069284922321,1.17985233294051

"Ctbs",1230.56325373828,1.01520701861409,0.294136294985507,3.45148502895269,0.000557510685297134,0.00375773821588713,5.66885309063295
"Bcl10",1457.73672852346,-0.319140048914657,0.140801903847994,-2.2665890175689,0.0234153436113993,0.0650097580447032,-0.872279293211918
"Ddah1",6197.68602423612,1.78977395109782,0.568767401899969,3.14675901804336,0.00165090964633465,0.00866472039952435,8.4987337749768
"Ccn1",5511.74589276397,-0.486844016869443,0.317902515163256,-1.53142549570402,0.125664266898875,0.23451396209746,-0.706040741515279
"Col24a1",48.2873891156306,0.385591732054499,0.452131262869339,0.852831387078692,0.393752815452533,0.538212447023555,0.238874815961969
"Cryz",10503.3367932055,1.74041295308554,0.527884144015673,3.2969600864425,0.000977373896637406,0.00576780757767438,8.9726350018364
"Asph",2939.60357210391,-0.381880717363751,0.286938095158465,-1.33088190033761,0.183227873657982,0.311389966057992,-0.445543762342338
"Trp53inp1",2531.31900882599,-0.320644580485992,0.187077247248693,-1.7139688829168,0.0865344408672348,0.176508895075776,-0.556120832277641
"Ccne2",96.9734030274351,-0.49047006725247,0.29941398866946,-1.63810004145774,0.101400826356398,0.199426398254695,-0.790789814622779
"Gem",954.42678227716,-0.935175068350021,0.244184242244739,-3.82979286358995,0.000128251167966339,0.00120629101447548,-6.28456808065946
"Cdh17",15.9372457498667,-1.92973110659077,0.801153153503644,-2.4086918938677,0.0160098073491295,0.0485322176140124,-5.83845437790426
"Fam92a",909.752521605428,0.494135881214512,0.14889991076499,3.31857741670787,0.000904772384713028,0.00545493257796371,2.57505820855193
"Pip4p2",1780.21651262824,0.176853452313834,0.166601161016906,1.06153793427579,0.288445502906535,0.432452171941507,0.148253338744481
"Calb1",8275.86758706061,2.3041179702575,1.04597693571033,2.20283821907865,0.0276061533404743,0.0738563087037004,6.0036877724889
"Decr1",9223.273201155,0.192625594451308,0.510712816453666,0.377170081199212,0.706047212931137,0.80468252032565,0.0418589794398533
"Nbn",878.723443983522,0.0492078555155121,0.185947381156132,0.264633226935283,0.791292020998258,0.865657399170961,0.0070990235180416
"Mmp16",30.2966122969455,-0.0533574495104321,0.548837156800045,-0.0972190910351785,0.922552407405413,0.952296407411232,-0.00260805559068138
"Cpne3",2166.0929549428,-0.366214825848833,0.156637442046808,-2.33797756822022,0.019388411828916,0.0560966395370536,-1.05494749364532
"Rmdn1",646.739428377879,0.0127647699972271,0.356835059319955,0.0357721856746751,0.971464011483228,0.982550331771967,0.000224707287346224
"Tmem68",902.374623301595,-0.232669590480038,0.113511090578796,-2.04975204884077,0.0403886330367701,0.0986221123294857,-0.538969748269097
"Tgs1",1491.87138023282,0.162818590337537,0.271734193708113,0.59918329789747,0.549050659285946,0.678802216394482,0.0630800705896153
"Rps20",12829.780805658,0.193118383458641,0.219253442625627,0.880799777399113,0.378426200263776,0.523301705868305,0.12506290620862
"Atp6v0d2",1649.57641025033,0.533478555477341,0.39731443665569,1.34271122883876,0.179365497332022,0.306707295890264,0.630497723000793
"Cyp7a1",0.893155919996283,2.64026475859727,1.82656873117534,1.445477913606,0.14832369240256,NA,NA
"Ubxn2b",1138.21100892498,0.490271014478402,0.112853503360348,4.34431364450367,1.39711785855756e-05,0.000220753679142263,4.12732842088386

"Nsmaf",2338.35219971382,-0.187196311459399,0.144746598785712,-1.29326
915471451,0.195917984949346,0.326636072727007,-0.209455573130069
"Faxc",23.4231729094525,-1.1527071005576,0.472334991042998,-2.44044401
20183,0.014669219617948,0.0454844580465273,-3.56230826354262
"Coq3",1067.4595979678,0.407211238821067,0.342151938481938,1.190147396
58581,0.233988464225754,0.371660726802096,0.403047041956416
"Pnisr",12125.7151673026,0.247407976908405,0.161226762134403,1.5345341
7803031,0.124898291673061,0.233464374560574,0.359910763420486
"Sdcbp",8525.95023819218,-0.539158940087446,0.16366867906979,-3.294209
63834835,0.000986988576507604,0.00581398890173573,-2.77531438216449
"Tstd3",514.152637332925,0.42880993246792,0.300586819695944,1.42657596
531238,0.153702156097952,0.273189119379933,0.556419899927083
"Ccnc",1057.94176709934,-0.161870784384261,0.152755654558686,-1.059671
30874407,0.289294159936378,0.43316368687141,-0.135427507115458
"Clca1",2.47484113342608,-0.263648550760893,1.34824038481102,-0.195550
106443257,0.844962315467299,0.900720312010354,-0.0275672214144456
"Odf2l",1373.12130021159,0.676468745926491,0.233453361593246,2.8976611
9155365,0.00375956488075196,0.0160262224873938,2.79620317402389
"Fhl5",2.52272669649324,-1.54533484570068,1.07322619481698,-1.43989669
015134,0.149896629658008,0.26781677678657,-2.03590479372892
"Ndufaf4",2356.86721443746,0.976919726434791,0.20097818557672,4.860824
68916442,1.16897742248548e-06,3.10951183951186e-05,10.1389220264584
"Clca3a2",364.607672253815,-1.163814150776,0.527722217819153,-2.205353
70973301,0.0274292836394478,0.0735445781630849,-3.03739613224055
"Spaca1",18.3642742586582,-0.956253058466608,0.707589333026731,-1.3514
2379037317,0.176559717192548,0.302989030612594,-1.1418222616085 9
"Lmo4",4647.09698944198,0.436414458017206,0.1907176856413,2.2882747163
6799,0.0221215279522783,0.0623032719938642,1.21137365013635
"Gbp3",1767.6089829688,-0.931697956966772,0.332119946474538,-2.8053056
3387345,0.00502688888682092,0.0199662414983162,-3.64639780685846
"Gbp2",2235.46040909515,-1.48844217475665,0.542691198045191,-2.7427055
7569039,0.00609352942839969,0.0231196024119356,-5.60707246009788
"Gtf2b",1307.51659852336,0.0716802556779026,0.18865194623276,0.3799603
296404,0.703974862989348,0.80354184981655,0.0156783363746475
"Pdlim5",3497.90376651976,-0.511488898300694,0.147177620147517,-3.4753
1708820964,0.000510249830249318,0.00351798132609661,-2.88984472857551
"Rngtt",703.963872452131,-0.386466208219462,0.177263539039225,-2.18017
879093537,0.0292442111097098,0.0771122398340087,-0.990317053123853
"Ube2j1",2229.05839759637,-0.341039678347243,0.125448510719032,-2.7185
6298964817,0.00655661703893653,0.0244859067276737,-1.26514042190982
"Rragd",2056.46434899387,0.713123278752255,0.447510905660389,1.5935327
3793385,0.111040730109662,0.213349640882754,1.10164920903568
"Gabrr1",0.639795019950342,-1.739405439738,3.50539127350459,-0.4962086
40925551,0.619747215154295,NA,NA
"Casp8ap2",633.896109635572,0.0541590554381038,0.217720023647731,0.248
755509625208,0.803549907614069,0.874797102102687,0.00724449413327379
"Map3k7",2663.13198208254,-0.187974718841004,0.126973623343034,-1.4804
2336582905,0.138760298163287,0.25254814651244,-0.258682042654936
"Epha7",308.761722060214,-0.606663876195908,0.315142220534999,-1.92504
792016128,0.0542233634797662,0.123582365999822,-1.26844159673643

"Akirin2",2356.98769010842,0.107363308784555,0.0999418013485692,1.0742
5829168419,0.282706932852754,0.426622495788285,0.0914580510244295
"Rars2",1153.21903123606,0.483547002208516,0.192383521798882,2.5134533
2327378,0.0119555597644014,0.0388710195574952,1.57032193820826
"Slc35a1",1617.17136229089,0.134900113812373,0.137247438415837,0.98289
7133596383,0.325658068732924,0.470521009570549,0.101703174415987
"Cfap206",11.4756184633487,-1.28698824165913,0.724333638319258,-1.7767
8927716992,0.0756029167755038,0.159173487883465,-2.36517622248414
"Smim8",883.477068653418,0.887119734218039,0.230797156853327,3.8437203
7469165,0.00012118307830043,0.00115598222147837,5.99941767498328
"C9orf72",1103.97069177686,-1.02166455432636,0.342251637225672,-2.9851
2685756037,0.0028346074111948,0.0129405701951478,-4.44157214824815
"Aldob",107420.739591887,2.14659674576319,0.798149547017882,2.68946684
713847,0.00715662553407752,0.0262095951659361,7.81711047950695
"Rnf20",3170.82202460482,0.140679261942594,0.122210466703581,1.1511228
5990863,0.24968169467767,0.38924551713642,0.132737212099969
"Ppp3r2",0.269403261492963,0,4.78471990594841,0,1,NA,NA
"Smc2",721.896976487902,0.48456885397675,0.333956108589783,1.450995629
40462,0.146781077235936,0.263554419739045,0.646170340572009
"Polr1e",516.187907535924,0.511369666862349,0.1346799302484,3.79692553
98276,0.000146501770717346,0.00134044651593,3.38258378213916
"Exosc3",1049.15360010247,0.0662809921390376,0.202018650234084,0.32809
3431285854,0.742841020432019,0.832478429265397,0.0121524852426543
"Stra6l",881.165712315162,0.667672842321574,0.501444942588809,1.331497
80886129,0.183025265491976,0.311177045582018,0.779436769589309
"Tmod1",389.515680280514,0.285744835888083,0.320791990784347,0.8907480
36412718,0.373064358040684,0.518350235379267,0.187764113251191
"Xpa",718.479451225491,0.618272519780769,0.10020413133794,6.1701300288
272,6.8233855829219e-10,1.1803365705416e-07,9.86286625897201
"Ncbp1",2153.76949722306,-0.126221078837773,0.130969012157907,-0.96374
7658763662,0.335172453989076,0.479788011737684,-0.0926981379820982
"Trmo",258.531097063808,0.0813645614741666,0.141160562687336,0.5763972
59441259,0.564346696395022,0.692182920444747,0.0299344308110875
"Hemgn",3.42872024767577,0.398647909211161,1.02699583563108,0.38816896
3670817,0.69789099936686,0.798467240092421,0.0897202322280171
"Anp32b",8398.19869561847,-0.110616249638051,0.154062453102759,-0.7179
96159416407,0.472759654879003,0.610478294069122,-0.0545905063699195
"Nans",1242.86382350081,-0.45994464975326,0.175662027447826,-2.6183498
8719955,0.008835615705651,0.0307362774360006,-1.60167061061707
"Coro2a",834.586233403427,-0.811557753590432,0.279164764887371,-2.9070
9235428709,0.00364805475908278,0.015687908576838,-3.37191292022847
"Col15a1",5217.82412186292,-1.24790746508774,0.26837325241094,-4.64989
507664092,3.32103937558255e-06,7.16301169746777e-05,-11.9100225377503
"Nr4a3",319.629334720364,-3.06955037215545,0.627844724961916,-4.889027
89195473,1.01335138655186e-06,2.81057259388167e-05,-32.1674674114793
"Erp44",2922.49819397897,0.758325229552269,0.123757014655927,6.1275333
0920746,8.92519187262565e-10,1.45019492610379e-07,11.9408906290627
"Invs",1066.35799203673,0.406149289947172,0.225978260978842,1.79729363
429874,0.0722890162703474,0.15375708947739,0.760466320452055
"Tex10",711.658334795399,-0.40072421788936,0.126537768093842,-3.166834

88199489,0.00154107793686279,0.00822245592804336,-1.92383142370696
"Tmeff1",238.966592520013,-2.13716895886064,0.443015227099543,-4.82414
334345312,1.40606272817557e-06,3.5884045908334e-05,-21.8743896943535
"Cavin4",7.4535219989192,-0.616753749455149,0.86855521043194,-0.710091
588937026,0.477647341842884,0.6150959560962,-0.299728135155867
"Brinp1",7.60614665745194,-0.87835430161289,0.800838543091894,-1.09679
324152122,0.272731785286702,0.415397890269667,-0.771650456393712
"Fmn2",9.27741579285354,-0.28062154916889,0.784875525634325,-0.3575363
736079,0.720690301008147,0.815318798320676,-0.0572962074917901
"Ambp",126.104062065354,1.65911084369771,0.325324151764626,5.099869882
69499,3.39887052735588e-07,1.18278652441857e-05,18.822699374526
"Kif12",9729.84800749861,1.17237058394336,0.612747662148766,1.91330078
654584,0.0557095571075412,0.126275859623673,2.42597050874703
"Zfp618",2177.32504168509,0.191787035734751,0.499312714238006,0.384102
047205897,0.700902814205989,0.800973102296822,0.042562896510374
"Orm3",129.078731104403,-3.45238121830554,1.46848680200355,-2.35097871
740844,0.0187241047794933,0.0546367247815865,-10.0362413934549
"Slc44a5",5.92297209131928,-2.41235406407933,1.00388622892321,-2.40301
539614393,0.0162605028149556,0.049131765672711,-7.26902466452373
"Tnfsf8",143.16237514094,-1.82319690599848,0.608659226839422,-2.995431
31131976,0.00274057018131893,0.0126474881409128,-7.96791132959317
"Tnc",3504.51290166254,-1.71904163932787,0.383336128449143,-4.48442375
176734,7.31112429280251e-06,0.000132581880464041,-15.3481368954258
"Txn1",12809.9633809052,0.807587389080301,0.224611032927014,3.59549296
646848,0.000323777876647918,0.0024564284197981,4.85283045312645
"Svep1",1541.10370213146,-1.19132147532657,0.23084689586549,-5.1606562
4733689,2.46085677018239e-07,9.08745933807127e-06,-13.8295925741783
"Pappa",211.52531977213,1.1758230831017,0.324548162781013,3.6229540571
9207,0.000291257570069639,0.00225982495074327,7.16366441823831
"Astn2",230.055608922811,-0.618122028356414,0.350886884221798,-1.76159
912539135,0.0781370518195676,0.163379757252325,-1.11983807319988
"Ptgr1",1168.45301199107,-0.583253295078014,0.380743183750639,-1.53188
112084498,0.125551773095078,0.234348886828244,-0.846268046670073
"Ugcg",1455.84778241064,-0.624964057739384,0.142762268493044,-4.377655
69527802,1.199626608875e-05,0.000195898824286807,-5.33588827477743
"Ptbp3",5995.35518919282,-0.343581998673136,0.228794057921407,-1.50170
857492794,0.133172388372227,0.244500492324076,-0.483948286292255
"Hsdl2",2778.2299505126,-0.218373709696185,0.422503167935929,-0.516856
975920476,0.6052560033382,0.726232710959275,-0.0698544254304507
"Snx30",1093.82253245452,-0.068116614742432,0.11816397617165,-0.576458
383928136,0.564305391265922,0.692175800132294,-0.0250611453742682
"Slc46a2",25.5249532484572,-2.0423057717671,0.692707885671356,-2.94829
294427297,0.00319534111803124,0.0141661575987661,-8.69389026871653
"Zfp37",170.604218728369,-0.872275614230123,0.243057043103676,-3.58876
913456918,0.000332242891941451,0.00250699377208762,-5.22377162611559
"Wdr31",1171.28042994719,0.933440433618482,0.314577472016503,2.9672831
5487738,0.00300444127780787,0.0135529537335495,4.01486803765706
"Bspry",1815.97718123669,0.042771388889287,0.390184277360979,0.1096184
32548262,0.912711991694931,0.946353538447837,0.00235837419777275
"Alad",3546.75087796134,0.697083967569412,0.198210972278879,3.51687880

622789,0.000436653104232254,0.00310045965998561,4.0264998299306
"Pole3",1548.38355460987,0.25649078667628,0.120324733634291,2.13165472
242678,0.0330352415663581,0.0847081983246778,0.63315850767432
"Kdm4c",1015.6730317432,-0.162267712623067,0.127923219834577,-1.268477
39474431,0.20462751834748,0.337133488906033,-0.176429841037581
"Dmac1",1344.33812746517,-0.0235453214543973,0.247836680624777,-0.0950
033763970749,0.924312145076176,0.953406242643775,-0.00112344590529699
"Ptprd",7213.75347623082,0.58983523288029,0.110927811789207,5.31728899
512717,1.05324713586172e-07,4.74277428522677e-06,7.23072423601068
"Mpdz",1098.33490166482,-1.14966266309343,0.224315947844849,-5.1251936
1257636,2.97232148611341e-07,1.05756066307006e-05,-13.1716396849791
"Zdhhc21",1329.36043119126,0.251478064654048,0.0964837447352553,2.6064
2935599242,0.00914916947845513,0.0315679537233973,0.869010818040532
"Aco1",8804.59725006126,0.420896973555273,0.349941699492728,1.20276312
93024,0.229067996589037,0.365616224418226,0.423494355243079
"Smim27",409.658608071164,0.628759324863237,0.220682610030725,2.849156
64526397,0.00438352921176755,0.0179634410615897,2.5272456920323
"Smu1",2689.05529435436,0.0574432410878824,0.109700820314209,0.5236354
74405311,0.600532103012109,0.722231598322123,0.0186925716634655
"Dnaja1",17469.1437354966,-0.0284641602910538,0.238364542746558,-0.119
414406031515,0.904947048597699,0.94200296586183,-0.0017006440842901
"Aptx",937.556381054642,-0.168870846400565,0.170877817081136,-0.988254
937271245,0.323027808783889,0.468073440522315,-0.128194937648764
"Slc44a1",3070.20817863902,-0.544415564423881,0.224284847454945,-2.427
33992332337,0.0152099973758016,0.0466443109206322,-1.66874493533264
"B4galt1",4087.35569109578,-0.373042730598212,0.164479022653349,-2.268
02618705017,0.0233276106262496,0.0648715485999973,-1.02040099307827
"Fktn",1876.34597122733,0.279286540427933,0.144033419252768,1.93903985
531168,0.0524964853139122,0.120676311088265,0.590591448605625
"Spink4",4.92363443206515,4.24901786842124,0.973839894848514,4.3631585
5501298,1.28197960161871e-05,0.000205728710060589,36.0697095758451
"Bag1",9933.11330696489,0.215242853358781,0.314206385585526,0.68503653
4052847,0.493320867223408,0.629542633496221,0.0996061491257727
"Tal2",35.9973882173093,0.249396462859998,0.507608106231614,0.49131694
2732594,0.623202298458144,0.740791222053093,0.074828028112276
"Chmp5",4407.01320860031,-0.0447349653301091,0.28978167431339,-0.15437
4721714561,0.87731428386889,0.923443851591212,-0.00356292887384504
"Tmem38b",1694.74431698667,0.43002234722187,0.265021980368179,1.622591
25308952,0.104676811573051,0.204078439361199,0.683413381860127
"Nfx1",3173.08259024806,0.138413904413077,0.142046007138426,0.97443009
6286977,0.329843032084181,0.474471957435559,0.103194868715829
"Rad23b",5894.38791331841,-0.275420340896331,0.199960739342939,-1.3773
7208714745,0.168397238188736,0.292326331036678,-0.338735216154702
"Aqp7",1582.65723088201,-0.75968726192493,0.770560692814008,-0.9858889
36471221,0.324187616513185,0.469022048436157,-0.575163404321224
"Nol6",1792.3594908919,-0.261560465344814,0.191299350097816,-1.3672836
0661482,0.171536428063723,0.296508623615821,-0.317973558740381
"Elp1",1181.08165334861,-0.0224194205817891,0.0808817155317631,-0.2771
87747989653,0.781635946876513,0.858900906909279,-0.00341003248377828
"Ubap2",2769.52991658408,0.0142751662880543,0.18407000308252,0.0775529

203509312,0.938183693645314,0.961736711162456,0.000556939257373321
"Epb41l4b",1509.32989006185,0.308221496058066,0.175068342301938,1.7605
7813768797,0.0783098316156907,0.163600674685818,0.557981613328959
"Aqp3",3827.05767387571,-0.55368240288304,0.670328384133108,-0.8259868
08837107,0.40881157472853,0.552621192738275,-0.328379352717522
"Dcaf12",2547.25412982062,-0.0725706342631158,0.071292958902477,-1.017
92148033001,0.308715272583974,0.453604399762045,-0.0573692509466787
"Ubap1",2378.63592870099,-0.00593346493416164,0.17359873996388,-0.0341
791935551848,0.972734257983416,0.983326857669524,-9.9763521666746e-05
"Kif24",158.99646055363,0.36239137991468,0.219281302152782,1.652632378
39671,0.0984057042894752,0.194872890292855,0.592657681252559
"Fam219a",1183.02849512918,-0.327687071463374,0.1384453844958,-2.36690
499041746,0.0179375369447833,0.0528397182881681,-0.963561254988083
"1110017D15Rik",15.26512978029,-1.27875428066523,0.714298529031981,-1.
79022387516071,0.0734179283884228,0.155818304965437,-2.37728689494509
"Nudt2",714.198586167532,0.129428646645714,0.260789991980539,0.4962945
30563781,0.619686624599182,0.737826685786202,0.0393523043713808
"Cntfr",428.959988763453,-0.365316465229413,0.38299232561892,-0.953848
003714061,0.34016058648424,0.484828297899832,-0.264474681939424
"Enho",389.779334250404,-2.25797243625349,0.520533914718003,-4.3378008
0876524,1.43915492177533e-05,0.000224664873216776,-18.9690022619997
"Dctn3",2666.37000501964,0.25648157701453,0.274409430213226,0.93466750
3282357,0.349959679126568,0.494959656434776,0.180378112614787
"1700022I11Rik",0.35099700026186,-1.07747086923113,4.78001494840159,-0
.225411610813357,0.821659079933927,NA,NA
"Vcp",18537.8355879862,-0.0377019876123637,0.202257863464652,-0.186405
54669437,0.852126743029962,0.905988369724028,-0.00372227237050056
"Fancg",504.70893029057,-0.396871974313109,0.153767371705847,-2.580989
51624351,0.00985175735137584,0.0333778566180931,-1.34931017228392
"Pigo",1459.2695491036,0.426033400876985,0.181634371969117,2.345554953
38417,0.0189987776803782,0.0552480488011951,1.23375960698295
"Stoml2",2380.1381121832,0.177356560044304,0.209648973052808,0.8459691
33364801,0.397569963721358,0.542123166140222,0.108588692728724
"Unc13b",1307.08141442955,-0.124725313878866,0.223413802299251,-0.5582
70404940348,0.576659750849561,0.702029958920126,-0.044125221681813
"Atp8b5",72.7019129004496,0.669761804762288,0.341573551558268,1.960812
83725516,0.0499008599930454,0.116202910324185,1.44160695509777
"Tesk1",2054.37884962101,0.0597206481487371,0.143725756529506,0.415518
064338572,0.677762693918052,0.78297292368848,0.0146110843968633
"Cd72",1972.03571210043,-0.89329490599802,0.460583171425322,-1.9394866
3654737,0.0524421101898493,0.120593969156938,-1.88960994294252
"Sit1",47.5700241844378,-1.49425147884445,0.564698855023456,-2.6461032
5583605,0.00814249577397785,0.0288763882504584,-5.29671958870092
"Ccdc107",6807.19250279409,1.83206291648598,0.400212640303587,4.577723
76978459,4.70062842168897e-06,9.4573896198323e-05,16.9761314278018
"Car9",270.541204924345,1.90870459790756,0.366153843572418,5.212848728
51556,1.85962535754472e-07,7.26068050717993e-06,22.5857721286312
"Tpm2",3071.70307876411,-1.47801045667794,0.252714242105661,-5.8485443
6522012,4.95893385922016e-09,4.76301932941255e-07,-21.5157143212941
"Tln1",12948.851932713,0.0625901197380529,0.203583543595761,0.30744194

0701911,0.75850700886456,0.84337190116567,0.010662055047871
"Creb3",2570.66713008592,0.069103284138989,0.0954230957049207,0.724177
764601966,0.468956607617962,0.607029383948134,0.0344948447405564
"Gba2",1116.17627202853,-0.285117086131675,0.186610852469708,-1.527869
80155915,0.126544867319174,0.235571111608674,-0.412205874921621
"Rgp1",3198.22790918381,-0.231263712620869,0.217971227504083,-1.060982
7511135,0.28869774012124,0.432680042314775,-0.193742737763628
"Npr2",1747.69078483651,0.0806796937178672,0.295744237722698,0.2728022
50820237,0.785005229265436,0.861018238024753,0.0120728764832124
"Hint2",4866.17114901844,1.34017605741243,0.328271396765183,4.08252461
414137,4.4549091940749e-05,0.000530940216785284,10.106081537562
"Spaar",197.911464268689,-1.50270208415415,0.564354463543006,-2.662691
94491742,0.00775183444514495,0.0278053628465892,-5.38346984084168
"Reck",561.704317794136,-0.990598179483924,0.229643597892575,-4.313632
90148118,1.60593525342046e-05,0.000242732756365831,-8.24529296951272
"Clta",6761.74941017544,-0.0720289019714472,0.334996905924908,-0.21501
3633551568,0.829756689109473,0.891333586262823,-0.00828595467045781
"Gne",1440.99775206363,0.207397695324487,0.175954680465889,1.178699508
16508,0.238517851566546,0.376663950077401,0.202503497165614
"Glipr2",730.424094237747,-1.55507086310744,0.314080153832116,-4.95119
11024428,7.37606266198735e-07,2.15297110454236e-05,-16.7109107186571
"Snapc3",811.690136367886,-0.0684274830190127,0.174031650774419,-0.393
189875028589,0.694179247317937,0.796135931074945,-0.0156004629838153
"Psip1",3040.0612563731,0.074657764075504,0.217208047264743,0.34371546
0894079,0.731060282723199,0.823832860708391,0.0144677495631217
"Bnc2",331.441558806658,0.946431787131392,0.415463408951549,2.27801478
238428,0.0227256968771425,0.0635032150631473,2.608995139665
"Sh3gl2",1546.26087019561,-0.717187778372095,0.400255155150547,-1.7918
2646155386,0.0731607686593869,0.155407851325932,-1.33519015014802
"Saxo1",3.52038498022816,-0.12029844996672,1.07408534335946,-0.1120008
30018273,0.910822748453423,0.945169934243248,-0.0067836949227636
"Plin2",9541.49596391089,-2.15964926341322,0.653105967613083,-3.306736
38047762,0.000943896751862055,0.00562128859737518,-11.1895625692877
"Rps6",25347.4881747271,-0.370953396847608,0.193248381916507,-1.919567
93205068,0.054912497901464,0.124788331478175,-0.772004589131819
"Mllt3",788.525615828088,-0.0518055531172354,0.3179551562143,-0.162933
520984698,0.870570777550058,0.918676680233319,-0.00439420065835833
"Hacd4",1058.44952022664,-0.459906901604227,0.331110257328952,-1.38898
415686143,0.164837567743464,0.287580788820871,-0.573159644656342
"Usp24",4877.63119597245,0.441099287334317,0.15477174832829,2.84999873
748723,0.00437194026376912,0.0179285511635273,1.7738193829004
"Plpp3",3657.46655610073,-0.844043876198191,0.282385838100521,-2.98897
381637721,0.00279916118219265,0.0128418928777393,-3.67584701280459
"Prkaa2",3266.54414953123,1.29721580001459,0.324165303248262,4.0017108
1548822,6.28861294996576e-05,0.000690088293617841,9.44203297743778
"Dab1",260.015843670121,0.667177107626377,0.275711904593602,2.41983424
186849,0.015527583314291,0.0473506934602932,2.03500614301196
"Slc35d1",2073.35723371853,0.904750358445026,0.138984775746382,6.50970
837335455,7.52968403471723e-11,1.93176025406469e-08,16.0704012472177
"Mier1",3002.65743021427,-0.139462010797946,0.0953575764290447,-1.4625

1630987831,0.143599778255268,0.259284797271773,-0.188249757519337
"Tctex1d1",0.490784232825913,-2.1731878428611,3.69007208043407,-0.5889
28290692214,0.5559093787867,NA,NA
"Sgip1",255.934375861848,-0.247946317415807,0.271245407120128,-0.91410
3283990342,0.360662577098491,0.505949991241555,-0.168930151755916
"Pde4b",858.077066330997,-0.590100507823852,0.145688691660088,-4.05042
080548461,5.11256104969015e-05,0.000588068177193943,-4.38956156911499
"Ak4",7641.65827473124,2.39930390601358,0.792463583132949,3.0276519414
66,0.00246461778001703,0.0116979351204411,10.6729265868781
"Dnajc6",565.629041495069,1.62242904298642,0.463607951742241,3.4995712
1505212,0.000466007106306391,0.00326606993485335,9.2870562019183
"Jak1",5979.2849143896,-0.415266887500943,0.0986008175840379,-4.211596
79682178,2.53571853951337e-05,0.000345502725949906,-3.30988888984284
"Cachd1",1454.18096361286,0.154951949263563,0.233427384103021,0.663812
216629976,0.506810505674223,0.641412370501258,0.068811480803512
"2610528J11Rik",1502.69570429696,1.6032403894834,0.47967883427298,3.34
232047556013,0.000830810780787165,0.00510691948417028,8.46055428574348
"St3gal3",1765.36004145344,-0.0536377109939317,0.15117743784388,-0.354
799709261663,0.722739646202851,0.816973599725387,-0.0108427827308447
"Artn",140.605928329747,-0.808596590174951,0.370730202010867,-2.181091
76373833,0.0291766299147761,0.0770013440820662,-2.07318699742907
"Dph2",442.306980850583,-0.0248168503319717,0.142840409453949,-0.17373
8302955317,0.862071133871386,0.912858294041004,-0.00226266688841176
"B4galt2",456.633998845564,-0.496263761230885,0.23037333624091,-2.1541
7187305011,0.0312266957646114,0.0810837813315213,-1.246749711475
"Slc6a9",1574.32168081121,0.171940533414757,0.162633544399024,1.057226
7489473,0.290408114738945,0.434164807635328,0.14345533022958
"Slc5a9",932.221778048357,1.79641675400398,0.456228323262942,3.9375388
6465448,8.23216019116667e-05,0.000850612927437031,12.6998641301972
"Bend5",1470.61538324232,1.79505209509636,0.528467104341302,3.39671491
441983,0.000681999788996193,0.0043742210150815,9.75077113951645
"Elavl4",5.69506227315206,-0.0750899617534836,0.724396850459105,-0.103
658597778129,0.917440283777101,0.949331351328659,-0.00390448149372151
"Itgb3bp",346.470525708927,0.65035738216158,0.232298132693124,2.799666
85320165,0.0051155371360575,0.0202194897788058,2.53711458099619
"Atg4c",684.680762661657,-0.0555210553260783,0.177204281486668,-0.3133
16669666671,0.754040086704643,0.840195581302428,-0.00966735832020917
"Cdkn2c",927.624656147514,-1.16626700678478,0.483382235702508,-2.41272
210818799,0.0158338867739131,0.0480811593704653,-3.53946277455964
"Eps15",3609.18447826261,-0.0935007442156731,0.135673204520698,-0.6891
61463724467,0.490721664569815,0.627210161637643,-0.0436156295504632
"Angptl3",87.6128725269414,1.31542581327559,0.468456473535628,2.808000
07596767,0.00498502150485036,0.0198525223246675,5.15571177989101
"Ttc39a",255.456886291532,-1.1315133324404,0.299989742455366,-3.771840
07419438,0.000162048109875124,0.00144472521552134,-7.39991178186212
"Dock7",3299.3186665526,-0.110892850474501,0.179011005779533,-0.619475
042842195,0.535603458125316,0.667339524987374,-0.044851315283491
"Rnf11",6748.21870387219,-0.25200903692753,0.360829583879069,-0.698415
673732534,0.484917277102034,0.622361576417552,-0.119511264346629
"Calr4",5.56600641919233,-0.887197551859725,0.787865178774918,-1.12607

788205498,0.260132555116536,0.401556848995505,-0.809484515089374
"Osbpl9",9307.62097527938,1.12891009821768,0.192881747425539,5.8528612
1307815,4.83187325177387e-09,4.67559815385824e-07,16.4546984370064
"Usp1",1275.30151929775,-0.372592960063918,0.140675010299002,-2.648608
01696036,0.00808240159779893,0.0287207337987759,-1.32275567529245
"Tm2d1",1457.02722410608,0.505542364599001,0.3046132028812,1.659620659
30597,0.0969907895696382,0.19273607980318,0.832341878712148
"Nfia",5875.44138102367,0.300043891737759,0.185586805122369,1.61673073
438557,0.105936416622229,0.205813895107634,0.474304265409748
"Txndc12",2159.73629373716,-0.00372020576541574,0.17664531037753,-0.02
10603143523303,0.983197542426626,0.989235031850681,-4.02650133148995e-
05
"Btf3l4",3052.47229411937,-0.0133677797907819,0.319825117253093,-0.041
797154349836,0.966660403532195,0.979724739997439,-0.000273820579288901
"Cyp2j13",6416.87960682339,1.51551855873474,0.61802730603965,2.4521870
5375052,0.0141990828751154,0.0444316671319212,4.71902599237489
"Hook1",1779.50372810477,0.654535578915748,0.21236274285081,3.08215824
550531,0.00205505556658019,0.0102009861092619,3.00122293384454
"Fggy",2742.54603354874,1.91018492121493,0.321731406431028,5.937203776
29479,2.89924228228349e-09,3.21189920567974e-07,28.5596202808109
"Eqtn",13.8879746019359,0.558820919807412,0.553991560966954,1.00871738
701584,0.313110190767834,0.458266213751031,0.436050763976448
"Ift74",822.250228970275,0.520885257092768,0.204358893622395,2.5488749
1246276,0.0108071055524603,0.0360138342269477,1.73134557141006
"Plaa",2698.94597413221,0.11730691727018,0.122776257667512,0.955452784
59176,0.339348771508538,0.483954238361137,0.0851372460821603
"Caap1",509.521750782948,-0.0188971835840527,0.13722336596832,-0.13771
1121212515,0.890468729691578,0.932257264364604,-0.00132557067716559
"Pum1",3075.05022812461,-0.112465848423457,0.0922932018730376,-1.21857
131555767,0.223006941706216,0.358968822701873,-0.11522348168521
"Laptm5",4321.54182991463,-1.36641228451674,0.401351995976012,-3.40452
34562591,0.000662796077156735,0.00428191464783333,-7.45153129361031
"Cc2d1b",3122.11842170802,0.23066066766068,0.181182975140067,1.2730813
5591859,0.202989174081716,0.335073054202954,0.252205919143052
"Pdpn",1608.42749308234,-0.890004999757286,0.279241382483502,-3.187224
58627661,0.00143645188776411,0.00777566321699741,-4.32253758837041
"Lrrc38",0.516905301515876,-2.10458901329418,2.33421138351777,-0.90162
7431069442,0.367254814221981,NA,NA
"Orc1",22.1012938616955,-0.542338088289164,0.463063492299389,-1.171195
95327226,0.241520021531933,0.380077270365587,-0.524647401582388
"Pramef12",36.0338494543021,-0.618470811514147,0.304063424715486,-2.03
401909352581,0.0419496703858114,0.101601766478765,-1.41425371489789
"Gpx7",379.470867539398,-1.34902446636993,0.244420984889445,-5.5192661
4230815,3.40418290706318e-08,1.9992397084915e-06,-17.7029021761375
"Tnfrsf1b",1558.11010363716,-1.54457267735106,0.321540746472837,-4.803
66079352104,1.55790670333981e-06,3.89436729509225e-05,-15.682655324128
8
"Podn",1710.6482711559,-0.339075574024651,0.311002957253966,-1.0902647
9046552,0.275596520290234,0.41902533941196,-0.294936033094881
"Echdc2",8912.83156888748,1.2617629825587,0.260366630090849,4.84610098

505496,1.2591144376761e-06,3.29975985293126e-05,13.020227822047
"Tnfrsf8",25.9722132896332,-0.938379763297162,0.509956484197165,-1.840
11732839219,0.065751013770988,0.142970142356053,-1.82526074337267
"Scp2",31661.4515590137,-0.0641051319231897,0.528007517910737,-0.12140
9506017729,0.90336668786657,0.940860193366934,-0.00390789517977125
"Cpt2",4940.06590464102,1.12580446603023,0.451110013060062,2.495631738
23928,0.0125733057971727,0.0403246250689771,3.61472504569937
"Czib",2580.03646652872,0.244551649650493,0.140834401735084,1.73644824
444602,0.0824846081067272,0.170188877128568,0.433063412493737
"Magoh",1714.20835230489,0.137118992632957,0.11480305230105,1.19438455
585125,0.232327575321105,0.369491213261853,0.136519552131654
"Dmrtb1",0.247153390650072,0,4.78471990595868,0,1,NA,NA
"Lrp8",156.287758576436,-1.0788338115294,0.339626039343304,-3.17653444
245741,0.00149046090017435,0.00799697485734713,-5.20935614289768
"Ndc1",734.627800845394,-0.734493693151253,0.307066912128781,-2.391966
2592732,0.0167583829011374,0.0502004839155597,-2.19740726654661
"Lrrc42",904.166506879816,0.00193747906100275,0.226324181165092,0.0085
6063656578283,0.99316968367967,0.995774283559737,8.20458447925537e-06
"Tmem59",14290.9317780903,0.445177528274681,0.196521698034827,2.265284
35651817,0.0234952353907313,0.0651650212871237,1.21570523458491
"Tceanc2",927.83419867412,-0.025850133708287,0.196588763908979,-0.1314
93444458787,0.895384972112729,0.935495455270281,-0.00172366084207048
"Cyb5rl",989.954468722896,0.267750506067036,0.162515609223392,1.647537
16487005,0.0994476837264841,0.196482316730517,0.435679029654241
"Mrpl37",3396.48811841219,0.35108942579854,0.193510423203287,1.8143179
0591307,0.0696287963981803,0.149468487523034,0.667305031558462
"Col9a2",9.67589010580492,-3.61675121039014,0.937043698204069,-3.85974
658099935,0.000113504673090468,0.00109668687607431,-24.6498289007903
"Exo5",505.740687118982,-0.19743349629179,0.159208148443597,-1.2400966
7986143,0.214939636871225,0.349707363127098,-0.207435196775615
"Dyrk2",2290.11179898224,-0.109535078643531,0.100416572244673,-1.09080
678811302,0.275357908921345,0.418760510736167,-0.0953454769193147
"Kcnq4",113.154209648383,-2.66325726285843,0.610445048454112,-4.362812
45888202,1.2840100856995e-05,0.000205885104037573,-22.6062397706449
"Ctps",1454.25951669525,-0.0115988681063343,0.193230893100285,-0.06002
59509244962,0.952134966102878,0.970226019466797,-0.000350589995592648
"Hivep3",607.670624123707,-0.906427847394733,0.240832842613463,-3.7637
2191416413,0.000167403035870706,0.00147827191730391,-5.90708286691881
"Edn2",2.31164687389821,-0.0951274547272369,1.43639926358845,-0.066226
3321477814,0.947197632767533,0.96734021808618,-0.0031587087496506
"Ppcs",1372.18777906609,0.555537047531867,0.276923771278869,2.00610097
48868,0.044845475878568,0.106945158049906,1.24186924727554
"Ccdc30",339.034979446675,0.867295958211668,0.206424042773759,4.201525
88117957,2.65121930357342e-05,0.00035460820897817,6.89023015080934
"Ybx1",31459.077979974,0.225307398411529,0.246995420596871,0.912192614
207456,0.361667331249642,0.506423787711954,0.15329497107004
"Tfap2c",19.5920361998569,-2.4093868131469,0.797528288451843,-3.021067
52579772,0.00251885179737693,0.0119031925218748,-10.675869212577
"P3h1",1160.6726310668,-0.19405939936862,0.184684966989376,-1.05075904
407413,0.293369270463494,0.4373510234343,-0.160490839328719

"Tmem269",11.1655678523485,0.546858083414352,0.475604879711422,1.14981
596434873,0.250219678526218,0.389897170295228,0.51507044827965
"Svbp",1219.27164361746,-0.00435284842048703,0.165814626859875,-0.0262
512934046856,0.979056903739654,0.986669833155619,-5.84144033668179e-05
"Ermap",26.818532263854,-0.570563042376716,0.436983974538022,-1.305684
13402325,0.191659995674956,0.321488867487121,-0.647470582924354
"Slc2a1",2195.27368107993,0.654807483025452,0.178905730309004,3.660069
92562215,0.000252146430086885,0.00202318975054901,4.06182315504029
"Rragc",3065.30077364113,-0.375189642221569,0.171589104158317,-2.18655
866327852,0.0287747577731248,0.0762283466871084,-0.965746348864747
"Mycbp",890.749658127898,0.551269361387737,0.211734149645368,2.6035921
0978038,0.00922524749490266,0.0317966900575591,1.90099345149607
"Ndufs5",5252.70695074703,0.193527478836313,0.364440606795925,0.531026
112972867,0.595400683842065,0.718119666803945,0.06408063628879
"Macf1",10163.6708934981,-0.216687110583793,0.144142366814755,-1.50328
536551831,0.132765472283846,0.243937163455562,-0.305711842820187
"Ppie",1250.07459928133,-0.295277951746148,0.136380862233811,-2.165098
14434172,0.0303801700604684,0.0794144732322045,-0.747961092742261
"Trit1",1074.86860500883,-0.227850381290298,0.284205866001838,-0.80170
8931964216,0.422721347854745,0.565969981492263,-0.129695681259448
"Mycl",727.807269176311,-2.14819662256252,0.44165409055899,-4.86397990
754167,1.15048597638674e-06,3.07290076268339e-05,-22.3204148337806
"Mfsd2a",648.999814291444,1.98442879326208,0.431660207109867,4.5972011
3315192,4.28204211653628e-06,8.81646957343854e-05,18.52723038143
"Cap1",7318.93936919882,-0.510424544662089,0.170633030922277,-2.991358
36656729,0.00277739342847506,0.0127684694759166,-2.22584734367496
"Ppt1",3947.60790373443,-0.312933571539826,0.139122191786479,-2.249343
311239,0.0244906622642167,0.0672940998911638,-0.844508420443266
"Epha8",2.96076503252363,-0.437212373652703,1.12496289827783,-0.388646
038302256,0.697538005252151,0.798110206349771,-0.0985951449828027
"Ephb2",876.968877309861,-0.268096263232334,0.255555312756476,-1.04907
33310945,0.294144369294884,0.438305457585911,-0.22113631262847
"Eloa",2002.34110903792,-0.493866046505991,0.182202542043026,-2.710533
23937361,0.00671751151655408,0.0249672206537879,-1.82246028117824
"Pithd1",1014.96681605388,-0.0172295554106359,0.199496748590626,-0.086
3650938291308,0.931176194308587,0.957144469219718,-0.00075467069520762
"Lypla2",5172.97663920973,0.140718247701016,0.118551029512408,1.186984
6114351,0.235233690503556,0.373202466609375,0.138696717594009
"Gale",879.263944050371,-1.48940408419703,0.509532293895386,-2.9230808
3715461,0.00346586523312706,0.015107856095576,-6.24438679376709
"Hmgcl",3455.80542482511,0.755251201144509,0.21011817904528,3.59441150
963788,0.000325125621559479,0.0024632011275109,4.53626038302924
"Fuca1",6626.84186924346,0.944833277694195,0.156344191372538,6.0432899
3229331,1.51002943870847e-09,2.13351840550274e-07,14.5129445159179
"Pnrc2",3827.84692396234,-0.126056892075641,0.0829955754999361,-1.5188
3870093458,0.128803101773721,0.23863577331661,-0.180616439084955
"Srsf10",5036.61984064816,-0.1200888266263,0.146046064214521,-0.822266
777760654,0.410925080345722,0.554440330536357,-0.0708279200936104
"Rnf220",2987.5763997956,-0.081913537337126,0.104171864201808,-0.78633
072341336,0.431673757595652,0.573740622058625,-0.0455093480029735

"Kif2c",119.296798055546,-0.966283298962753,0.422744645795171,-2.28573
752163129,0.0222696187528743,0.062620857577667,-2.6772394589264
"Plk3",952.691477142162,0.127421789638613,0.228380947529407,0.55793528
7584381,0.576888574137535,0.702204143630637,0.0450475672819635
"Ptch2",652.84231356631,-1.14775399277017,0.409031377976646,-2.8060292
0599333,0.00501561460502379,0.0199255202870322,-4.49432222206545
"Eif2b3",703.276651073777,0.0656971091620312,0.151744408362974,0.43294
5832210719,0.665054122435963,0.772962880260873,0.0169185988895245
"Urod",2638.27058877611,0.674401301076488,0.1622008733779,4.1578154730
7238,3.21305391544247e-05,0.000410538173285041,5.25900942869219
"Mutyh",165.028419399514,0.125679715304223,0.321713264663552,0.3906575
48533659,0.696050381509739,0.79739073670252,0.0284552022777459
"Toe1",839.102387555058,-0.0599223424212274,0.0834181267977687,-0.7183
37185471662,0.472549407287574,0.610425887325634,-0.0295775717131404
"Ccdc163",848.995457339884,0.311197317586616,0.218420879301453,1.42475
993404054,0.15422660763074,0.273855949114857,0.403048153156211
"Mmachc",620.412838031624,0.144423110359251,0.287584121736038,0.502194
312702047,0.615530843142772,0.734673137830422,0.0445299182760833
"Prdx1",35044.7166436291,0.49249132499239,0.355882246245429,1.38386033
635617,0.16640122377052,0.289476805101269,0.610531697836296
"Akr1a1",55370.2886453468,1.25221164588046,0.284087644704713,4.4078356
4234916,1.04408725313377e-05,0.000177022724013933,10.8181474845713
"Nasp",1972.81453810765,0.33278129959951,0.168957708572492,1.969613002
04145,0.0488827403693069,0.114405914262483,0.721470690574549
"Ipp",502.184018375278,0.328344472354157,0.32383656037309,1.0139203306
0095,0.310620769414055,0.455888879340252,0.257916613786066
"Pik3r3",1251.3492733238,-0.296376966687273,0.1831176443162,-1.6185057
8514161,0.105553641090286,0.205377197283544,-0.469137176333323
"Tspan1",988.299055214363,0.423834044441457,0.539866964446733,0.785071
271911982,0.432411782213824,0.574486571927306,0.234922121908277
"Pomgnt1",2495.07672844484,0.0834498236503907,0.121737214999601,0.6854
91479747291,0.493033835334099,0.629342315887709,0.0386439399899565
"Lurap1",597.576686375082,-0.293673561828681,0.242014246297062,-1.2134
5568007682,0.224955649311345,0.360973191529306,-0.299239141326846
"Rad54l",155.205358622938,-0.703582127999297,0.360439163506491,-1.9520
1354135488,0.0509365969330853,0.118078916538021,-1.5031343314408
"Lrrc41",1946.12613136372,-0.0336030925588166,0.0858533618493376,-0.39
1401010222361,0.695500845713264,0.797229599630642,-0.0076148828905423
"Nsun4",999.776935770257,0.251230748260317,0.116078750488966,2.1643129
9615167,0.0304403359827032,0.0795333582650046,0.636010422263662
"Dmbx1",0.220838897876658,-0.0640482484514447,4.78471990595296,-0.0133
859974482014,0.989319838260088,NA,NA
"Mknk1",1498.00894406277,-0.387288506387523,0.159818492412394,-2.42330
221328936,0.0153801258374768,0.0470034002475114,-1.1841482928083
"Mob3c",1501.05420274759,0.299689309660479,0.183507568679719,1.6331168
8894716,0.102444415195819,0.200972050255365,0.48087829977828
"Atpaf1",1178.96737591578,0.700964393600958,0.258728606176546,2.709265
1406418,0.0067432427678927,0.0250437614263565,2.58454721421332
"Cyp4a31",7168.09875928125,2.64220907751274,0.939081106867573,2.813611
15476614,0.00489884462940453,0.0195692834632513,10.3939065125423

"Cyp4b1",57804.9701420961,2.21954081107583,0.668433871016191,3.3205091
8919109,0.000898534010655577,0.00542739037786941,11.5777837500341
"Cyp4a14",1687.31550255692,3.42375124323122,1.22748637739176,2.7892376
699986,0.00528322780344639,0.0207610521644609,13.2659286198241
"Pdzk1ip1",17849.50087418,1.57393779800456,0.417599139125751,3.7690159
0673682,0.000163892444026297,0.00145517981494751,10.281946669733
"Tal1",220.543116693585,-0.823138243858616,0.191416082144424,-4.300256
46036135,1.70600563357282e-05,0.000253534282343009,-6.81559413183208
"Stil",71.2988646814584,-0.795820204331405,0.396771662736239,-2.005738
51177583,0.0448841540100309,0.106960551807331,-1.77889307261477
"Cmpk1",5456.90054752305,0.220984841466404,0.25080539746663,0.88110082
039126,0.378263253260029,0.523177036858622,0.143161796447064
"Ebna1bp2",2125.88659219192,0.00611252661193472,0.155231964074382,0.03
9376726619305,0.968590034982883,0.980658846153693,0.000119381564150273
"Cfap57",5.57094690355381,0.697724153765958,0.657100217283294,1.061823
04527401,0.288316025747371,0.432394664944291,0.584983464968574
"Aldh4a1",13184.3842907393,1.1594075342168,0.382630872379437,3.0300940
6169157,0.00244477586273029,0.0116292363433802,5.16427127298924
"Tas1r2",3.27356444034218,-0.251851228744096,1.08324015601162,-0.23249
8054421641,0.816151198167115,0.882553161880229,-0.0314653485552273
"Mrto4",1009.34726495496,0.182187781732533,0.131593162694041,1.3844775
6709158,0.166212272390041,0.289202827687044,0.226027083114801
"Akr7a5",9213.34539542981,1.68516359424898,0.432550209199493,3.8958797
3467326,9.78429333009895e-05,0.000972345317789344,11.6879569009944
"Pqlc2",877.5810882736,0.838916624494791,0.191761217279007,4.374798180
77184,1.21544876706275e-05,0.000197985129993229,7.15370936202269
"Capzb",9282.77687489945,-0.375904653311846,0.0993955407672562,-3.7819
0661683769,0.000155631749002541,0.00140032664607824,-2.47008817852507
"Htr6",1.07433677093861,-3.00945142581752,1.6811242398991,-1.790142188
41919,0.0734310560649092,NA,NA
"Pla2g2f",0.0764107020153886,-0.544048378937456,4.78471990596623,-0.11
3705376621746,0.90947135024012,NA,NA
"Pla2g2c",13.8446593054809,1.43423693583105,0.758325718405107,1.891320
44584681,0.0585815810054089,0.131169461006369,2.91331557098188
"Pla2g2e",9.43573580438282,-4.56328502048739,1.33185773459211,-3.42625
559920251,0.00061196421451342,0.00402430969800427,-25.1683508102749
"Vwa5b1",2.46990890468335,3.31557835782826,1.26699559368623,2.61688231
147027,0.00887369304378031,0.0308302328880307,11.5357574436693
"Cda",5177.40820011798,2.26389473113492,0.696572379011239,3.2500495272
9886,0.00115384911441743,0.00655528847112793,11.3816926253268
"Pink1",10910.7910055684,1.02350410148596,0.191355152139233,5.34871462
85,8.8581085046774e-08,4.17472072147031e-06,12.6775957805959
"Ddost",5443.38982322206,-0.0584569792713283,0.130949086644069,-0.4464
09980928081,0.655301124428106,0.7652858891684,-0.0156375812181708
"Kif17",39.5234745688771,-0.265736709072763,0.356148635776215,-0.74613
9904463198,0.455582906020345,0.59569175842172,-0.137660100155532
"Hp1bp3",7204.59239013627,-0.388152532344008,0.107558309764044,-3.6087
6377841487,0.000307659590267467,0.00235522053044173,-2.34875790566721
"Eif4g3",10504.2950557884,0.704966315310109,0.213203705639808,3.306538
75454256,0.000944562834649367,0.00562182116910664,3.65250070563596

"Hspg2",7317.87415731535,-1.07978508223101,0.187293091648604,-5.765215
75209449,8.15534443285866e-09,6.81317024540035e-07,-15.3321254914793
"Alpl",7066.94642010597,1.54137992450937,0.426563969670286,3.613478948
30589,0.000302115895095535,0.0023191557962884,9.35086152611754
"Ptpn12",1851.12650459158,-0.263583598209407,0.173914209036269,-1.5155
9553224567,0.129621657476968,0.239587097326642,-0.376618331169424
"Zcchc17",1768.81181982047,0.443330002158627,0.245843611271452,1.80330
088655067,0.0713409734872361,0.152306476371964,0.834285217844069
"Fabp3",1947.60758127089,1.14884061491352,0.538981718420311,2.13150200
767593,0.0330478072213179,0.0847292761605861,2.83567650209869
"Tinagl1",4114.68999185567,-0.215608667555037,0.164118966409489,-1.313
73400815282,0.188935732196205,0.318123394331744,-0.246940043532449
"Gnat3",4.31150926641589,-3.33182210559238,1.3628764504506,-2.44469856
713043,0.0144973251689825,0.0451475556851654,-10.3213822838927
"Hcrtr1",14.2539663955969,-0.0352300061788018,0.557425864540779,-0.063
201240595151,0.949606256904458,0.968734515624084,-0.00111906995419825
"Pef1",2717.57023177566,0.165090543864231,0.187331934517008,0.88127282
8841907,0.378170168810142,0.523177036858622,0.106951493501509
"Sema3c",3536.10977574199,0.110254288317325,0.250933721008931,0.439376
134359401,0.660389016334548,0.769369460877227,0.0289069082971988
"Adgrb2",100.540369421638,-1.47329561197825,0.548411333485679,-2.68647
914807676,0.00722094551770491,0.0264005242273036,-5.35450345349886
"Spocd1",0.366353220158493,-1.38557779097994,4.76874047520826,-0.29055
4245546238,0.771392257703944,NA,NA
"Hpca",42.6980088006184,-1.22554812810393,0.539061136614281,-2.2734863
3552275,0.0229968886496815,0.0641111431071619,-3.36674871919948
"Tmem54",232.94545995009,-0.819374071504271,0.217396271023888,-3.76903
461887916,0.000163880159249381,0.00145517981494751,-5.35266420086602
"Ptp4a2",13432.597917301,-0.283969532838508,0.111202432560605,-2.55362
698728506,0.0106607396405127,0.0356571529177321,-0.946699193077138
"Azin2",498.406590690928,0.047496008787179,0.171593383172867,0.2767939
41053837,0.781938333816651,0.859027182131512,0.00721724241114561
"Khdrbs1",4090.33381906306,-0.25487070971593,0.122274686671157,-2.0844
110637664,0.0371227923153543,0.0925719663083231,-0.606533394317988
"Ak2",8408.35126338612,0.23926954894386,0.349280464845007,0.6850355889
50087,0.49332146359569,0.629542633496221,0.110724783663932
"Rnf19b",1711.174102286,-0.674951326965892,0.147139022191612,-4.587167
40748037,4.49300395761873e-06,9.15449959339731e-05,-6.27615636974159
"A3galt2",47.8645627159428,-1.44178383250962,0.383568006474835,-3.7588
7406710552,0.000170679714231559,0.00150009955003354,-9.37480115940811
"Ccdc28b",868.450892998588,0.216986260733586,0.193193590835878,1.12315
455080453,0.261371861344808,0.402927621165486,0.197240149345223
"Phc2",4411.68139772867,-0.341312669321472,0.163314958333701,-2.089904
51826262,0.0366263776401619,0.0916566477013238,-0.815636856535253
"Tmem234",5732.49810358598,0.167190768123522,0.15081516045885,1.108580
64676555,0.267611130810738,0.409755715173708,0.149166619151777
"Eif3i",3819.00153113349,-0.0598605658275649,0.246735058184334,-0.2426
10702621933,0.808306989400371,0.877917205844944,-0.00779402455659608
"Zfp362",1565.09253105854,-0.44381999422356,0.168124823817857,-2.63982
429331426,0.00829490204103945,0.0292666309865536,-1.5672647793399

"Hdac1",4166.412698309,-0.110479032490742,0.105557892838362,-1.0466202
9072441,0.295274739760797,0.439687404601803,-0.0907796541560377
"Stpg1",23.636641519842,0.829652253069601,0.473077430336368,1.75373458
945125,0.0794759957246827,0.165504962580355,1.49234039114832
"Nipal3",1760.88080270026,0.365083735068191,0.146499484317666,2.492047
91927153,0.012700890075958,0.0405903875923665,1.16981006562853
"Csmd2",1.32781154242331,0.886957951565432,2.25332903172993,0.39362114
4127583,0.69386076818754,0.79586430131894,0.202516078700724
"Zbtb8a",606.377906172759,-0.35461957848296,0.232079012399075,-1.52801
226968843,0.126509491621964,0.235550235642194,-0.512720005822699
"Srrm1",6444.07539373971,-0.235119176856613,0.17377796364401,-1.352986
14350357,0.176060057138165,0.302385494236688,-0.281214903875773
"Yars",2048.14539470453,-0.114160266215803,0.139619152441415,-0.817654
771709814,0.413554342968434,0.557052354056913,-0.0667947208927243
"Sfpq",8117.10116778779,0.12140130390732,0.122233314694614,0.993193256
76987,0.320615783886186,0.465887607245795,0.0927276343564564
"Syf2",3606.57259704333,0.0701129152909179,0.104894069526029,0.6684163
90056446,0.503867836116335,0.638778613042673,0.0314244223502005
"Tmem50a",4209.23068339393,-0.333889091606183,0.140420262045016,-2.377
78427944497,0.0174170118452609,0.0517126384892487,-0.9889972085434
"Rhd",1.4163128890566,2.07418906827422,1.49645520368563,1.386068265301
68,0.165726059514875,0.288562842330865,2.57789010387035
"Maco1",2757.58402878916,0.108994185757971,0.151180945820145,0.7209518
71062096,0.47093912701905,0.608910550306196,0.0540702609321956
"AU040320",2468.97316123083,0.198537195679536,0.100779369883651,1.9700
1822802371,0.0488362815283065,0.114365444848718,0.430499379270844
"Stmn1",3143.17787663976,-0.251035902438866,0.271399856292598,-0.92496
696891476,0.35498308941545,0.500105511374454,-0.173951859297218
"Ncdn",1508.93566500517,-0.109691427721002,0.0889719642816116,-1.23287
631791302,0.217621912228661,0.353037154993813,-0.114208737097103
"Trim63",426.260690743186,1.92349409221952,0.808373763513661,2.3794613
0742653,0.017337963119539,0.051532866601337,5.70418999226835
"Slc30a2",1956.94382522377,1.15407913275537,0.585142571274397,1.972304
16895129,0.0485748940118776,0.113945293965783,2.50670237329928
"Psmb2",2877.21574993515,-0.29153517933496,0.247439211378888,-1.178209
29718593,0.238713177014214,0.376785357842074,-0.284561540142351
"Extl1",191.023652599285,-0.594196915367362,0.189363758840436,-3.13785
974151501,0.0017018629233009,0.00887243937928369,-2.80746485731344
"Zfp593",444.347781702273,0.164398924241987,0.178089266380844,0.923126
51729622,0.35594127712748,0.500986371651578,0.113628653425455
"Cnksr1",682.425887442272,0.399872186851745,0.225119219345829,1.776268
53901559,0.0756886661270286,0.15928522527197,0.734588725405184
"Ago3",1178.13047259995,0.0969708304246851,0.207679675094421,0.4669249
91001636,0.640553544613201,0.754152105118544,0.0273614059157039
"Sh3bgrl3",6514.61281735376,-0.269931900422485,0.252428887364812,-1.06
933839165712,0.284917217187193,0.429114467844577,-0.228370909732371
"Tekt2",159.989936718015,-0.235049293113768,0.27861812723762,-0.843625
271062519,0.398878838146743,0.543242089586847,-0.143427130657401
"Trappc3",3332.0076971053,0.0699082231985135,0.12550079074348,0.557034
125318015,0.577504113787288,0.702663039664557,0.0246690613862876

"Gpn2",766.243323192082,0.336323330064638,0.159272853911453,2.11161740
249606,0.0347192786882968,0.0878821882207466,0.817856992757103
"Map7d1",4181.70491793766,-0.889844674207708,0.153144531774562,-5.8104
8937168461,6.22904874921266e-09,5.60381183856537e-07,-12.8090013931967
"Gpatch3",538.134230447856,0.275483129506735,0.196393366641572,1.40271
096838778,0.160703045218999,0.282312638587265,0.348414581330223
"Nudc",5428.92524515645,0.0594281209588038,0.153869444219573,0.3862243
16726584,0.69933054615737,0.799645026329838,0.0132873770834169
"Slc9a1",2187.15382779919,-0.214628526601529,0.14202608369637,-1.51119
090955413,0.130739817805521,0.241078585925104,-0.305337477871954
"Tmem222",2032.32229297629,-0.178003853034301,0.144280563394753,-1.233
73411391028,0.217301997626698,0.352668083046892,-0.185520588888949
"Csf3r",499.042114811743,-1.06180769021048,0.475732368081329,-2.231943
33926204,0.0256187080070445,0.0698034612522854,-2.82660818415942
"Sytl1",503.718492931208,-1.12199800819096,0.44377436185157,-2.5283074
1169819,0.0114613948069122,0.0376980897343412,-3.67807312097863
"Mrps15",2072.28609141724,-0.0852185822459899,0.277314303215068,-0.307
299628104287,0.758615318733614,0.843396138936481,-0.0145143024343137
"Map3k6",591.046811752362,-0.094359302772956,0.3040696619671,-0.310321
332823942,0.756316610113802,0.841774546120265,-0.0162527351006087
"Meaf6",1339.58434184051,-0.252809058292941,0.106556357930741,-2.37253
846886603,0.0176663233766694,0.0522450871710519,-0.746244159222869
"Hgf",99.6154093637875,-1.474296542012,0.331895032407864,-4.4420566686
8867,8.91030845378931e-06,0.000156234886899266,-12.9209560355349
"Cd164l2",204.783889905313,1.70921506861639,0.319059201683036,5.357046
77878051,8.45932246624193e-08,4.02362491182574e-06,21.2341382957762
"Wasf2",3825.26114475827,-0.633168189015773,0.202832995774254,-3.12162
32181497,0.00179856964411273,0.00920433357504201,-2.9683436712937
"Gnl2",2170.14522036462,0.356725894357729,0.23145817065001,1.541211067
88292,0.123265424667376,0.231121189553275,0.52253675457797
"Rspo1",115.663290263921,-1.59188608213973,0.414634532022946,-3.839251
09752228,0.000123410168152041,0.00117092528400413,-10.7451689735815
"Cdca8",238.000560249157,-0.558953258015168,0.31698841713941,-1.763323
91908611,0.0778458733650938,0.162875720449898,-1.01437038565124
"Fgr",386.245504383578,-1.18814160927548,0.482313900969194,-2.46341979
131838,0.0137618682940786,0.0433347719634923,-3.72933878646518
"Epha10",1.70206954108835,-2.27632364365411,1.99300806795302,-1.142154
75604776,0.253389698546984,0.393798209968843,-2.12134392325146
"Fam76a",2014.75558369735,0.237936493701092,0.0904560193577444,2.63041
083822269,0.00852817368721795,0.029874801696546,0.835333143655062
"Stx12",2677.36885191721,-0.505493945787174,0.228130091785131,-2.21581
441462481,0.0267042148409125,0.0720662672620224,-1.32953460932905
"Ppp1r8",1202.60438095991,-0.575246789618999,0.16409777392344,-3.50551
245068919,0.000455729185489072,0.00321019062813076,-3.30273627486632
"Sema3a",90.6026539660211,-1.33032816185451,0.231762625660985,-5.74004
61271977,9.46507665478427e-09,7.62668681937531e-07,-18.7395906668276
"Rpa2",601.868270905694,-0.286038001098288,0.194439579330358,-1.471089
38459645,0.141266945456492,0.256011051446294,-0.389736692210324
"Smpdl3b",191.322708598524,-1.08041066256013,0.389499906257262,-2.7738
4062281783,0.00553988111951163,0.0214361186878821,-4.15167027373913

"Eya3",1283.13936520889,-0.289566336251624,0.108314856233362,-2.67337599218856,0.00750920336102453,0.0271893123738637,-1.04386675197041
"Yrdc",1071.55379980475,0.0305837526532193,0.152350122885052,0.200746491529217,0.840896804638289,0.898239455056056,0.00328220528775902
"Mtf1",1533.84295355796,0.290721304985093,0.11838004221354,2.45583038786788,0.0140559481197431,0.0440962048107642,0.90745212017302
"Sesn2",1845.47545758245,0.409752461479285,0.216315382396659,1.89423635498986,0.0581936445853681,0.130540690534458,0.834284815513925
"Inpp5b",2298.23149828287,0.580799640322763,0.239699791795411,2.4230293901068,0.0153916813929376,0.047031343645118,1.77547024111444
"Rcc1",996.01512341665,0.2138513908368,0.129350881194846,1.65326582131797,0.0982767757609941,0.194695293446214,0.349929209537867
"Trnau1ap",1820.64514482165,0.302490774261949,0.140212108258763,2.15737982987673,0.0309760815003453,0.0805850942630228,0.761805343853071
"Taf12",1354.19200955436,0.00738523009615747,0.187953912849838,0.0392927712127907,0.968656969903876,0.980658846153693,0.000144238279268594
"Gmeb1",884.475413267529,-0.125426439941986,0.181544688932505,-0.690884655891077,0.489638029545836,0.626190000710156,-0.0587122969530096
"Sf3a3",1721.48998989705,-0.179313030841581,0.102485341165015,-1.74964564495974,0.0801794782946931,0.16657852613638,-0.321380674523679
"Epb41",6100.25931489615,0.964421345058256,0.1758556567861,5.4841644714979,4.1542814147999e-08,2.32092203512231e-06,12.5119647050095
"Utp11",2736.99154722597,0.0389636612252866,0.130469015184651,0.298643023940526,0.76521243428727,0.847955652132721,0.00642615747829785
"Ptpru",1683.49885650806,-0.708254787478955,0.273177776773109,-2.5926515540362,0.00952392089605286,0.0325784929177612,-2.42513729637427
"Mecr",1867.62193846925,-0.213999473725975,0.371216611663502,-0.576481404662892,0.564289835284861,0.692175800132294,-0.0787336825434267
"Srsf4",3885.93834418837,0.0636636137163179,0.141580386398232,0.449664076613319,0.652952677756218,0.763594501103844,0.0171712472935031
"Casp9",1173.47154062494,-0.111353115338826,0.190027128065252,-0.585985361524745,0.557885352014555,0.686782595104151,-0.04183954031663
"Plekhm2",2675.17818184859,0.118854435383053,0.145611746022496,0.816242086436429,0.414361690218528,0.557726372765489,0.0693975369233803
"Arhgef19",1170.4625977686,0.458238224787819,0.301170070584515,1.52152643819641,0.128127781895991,0.237645709614936,0.65847656579841
"Fbxo42",1312.67979874043,-0.0882197472044014,0.131380212531471,-0.671484278374637,0.501912071574977,0.637027425567715,-0.0397820395353096
"Necap2",3431.37629185566,-0.212171523250959,0.0854203761323243,-2.48385142816844,0.0129970020913458,0.0413942415513004,-0.67568429742796
"Cdk14",472.301410458255,-1.18306653395006,0.234950623326187,-5.03538367849991,4.76892653600349e-07,1.51440601952762e-05,-13.1295566534387
"Padi2",402.242532108071,-1.2692910066062,0.309280932042879,-4.10400666546823,4.06055755735387e-05,0.000493903626034222,-9.6633284016451
"Kcnab2",376.577488802543,-0.925153790249437,0.343434471275521,-2.6938291512014,0.00706363620226801,0.0259344087403786,-3.37883248938988
"Psmc2",4332.05340537028,0.160062487538273,0.149000831021135,1.07423889142986,0.282715625606751,0.426622495788285,0.136350149022964
"Xrcc2",176.859862514412,-0.050909461092819,0.244006739526379,-0.208639569512035,0.834729618289984,0.894359715211458,-0.00568389973794315

"Rpl22",10404.1571708748,-0.0816125931320202,0.174511245269958,-0.4676
63805881223,0.64002502813157,0.753711904240464,-0.0230755586726823
"Acot7",1021.25545186934,-0.560776042579697,0.247410692620016,-2.26657
965604163,0.0234159160297441,0.0650097580447032,-1.53272311555734
"Galntl5",0.45512698397963,3.27980682463686,2.76255742727449,1.1872357
0857047,0.235134659473074,NA,NA
"Hes2",24.0807557818413,0.451144722170297,0.447703298213102,1.00768684
07513,0.313604824320824,0.458749108305756,0.351555347351761
"Espn",1479.50154541382,1.21079894637993,0.354351023739069,3.416947786
98175,0.000633274238581778,0.00413710747863871,6.64457210322571
"Prkag2",2129.93327911785,-0.0976146343065505,0.21329474951755,-0.4576
51369887653,0.64720293101108,0.759641388686132,-0.0268351233514539
"Rheb",4226.86808499115,-0.0171321078982385,0.189730087335369,-0.09029
72645975522,0.928050993808096,0.955739739483284,-0.000775564391750838
"Hes3",1.09005442187109,-3.02165967929863,1.84485486649391,-1.63788476
490902,0.101445734653524,NA,NA
"Nol9",1302.86541403198,0.215633216342066,0.0902237068199603,2.3899840
0689031,0.0168491064919394,0.0503948271789898,0.644283316120121
"Smarcd3",1286.2349482607,-0.0868006739123609,0.167620030930881,-0.517
841891749521,0.604568587376745,0.725586502318834,-0.0278434846509427
"Tas1r1",8.17104777456371,0.359684257539435,0.609694896196481,0.589941
394922753,0.555229940577087,0.68466186322869,0.136259256673429
"Zbtb48",941.027748314489,0.286291819732172,0.211565653849611,1.353205
56301487,0.175989968554066,0.302385494236688,0.342420076672442
"Abcf2",1955.99556789522,-0.285430752019317,0.161060315656985,-1.77219
789278948,0.0763617133007411,0.16030369142311,-0.52253385089628
"Nub1",2937.11255679026,-0.349458162161268,0.133681552742263,-2.614109
08979356,0.00894604674598668,0.0310428936560328,-1.21345340781395
"Vamp3",3567.44729370296,-0.168997294908922,0.15485326649701,-1.091338
26319502,0.275124067000355,0.418502813104457,-0.147208755428731
"Per3",2109.94573407519,1.94990234737164,0.18920930300556,10.305531051
5802,6.65232383778574e-25,2.59414020378293e-21,92.4273832120578
"Tmub1",559.769279028026,0.304738659786986,0.159473960963238,1.9108991
7091377,0.0560175396469802,0.12684124817522,0.629230171353144
"Fastk",5558.54486521957,0.910953057263056,0.26329949189663,3.45976002
726466,0.000540656983360648,0.00367820302217932,5.10619337065939
"Ube4b",3537.67511878544,-0.224831628212987,0.192288879606236,-1.16923
884872277,0.242307413133819,0.381039595232131,-0.216929242718445
"Pgd",10429.4378068868,-1.7053960298235,0.514226486424718,-3.316429773
34884,0.000911754988807649,0.00548515852260769,-8.87779594630232
"Slc4a2",3924.64315569788,0.0033615938679062,0.122828944553854,0.02736
80921065989,0.978166147521479,0.986106382522817,4.7032184770997e-05
"Uts2",0.167411724685723,-1.38296167455407,4.76914167930786,-0.2899812
51879012,0.771830580500136,NA,NA
"Park7",13869.3351581289,0.885457087810665,0.262815793967124,3.3691167
2789888,0.000754094860258684,0.00472776256762824,4.74100573510126
"Tnfrsf9",105.156979177285,-1.66250726038815,0.460745228741725,-3.6083
0054589689,0.000308209327814907,0.00235757766721658,-10.0583694224982
"Errfi1",12343.0831619764,1.22807959752454,0.310577440521607,3.9541815
8982189,7.67970684978281e-05,0.00080504797933906,8.74958656106282

"Cdk5",2347.80731576854,0.226700131161095,0.160042370155021,1.41650071
128981,0.156628956056401,0.277186237164506,0.290871153949312
"Abcb1b",295.877913114053,-0.354521985273499,0.367970670768677,-0.9634
51746121275,0.335320868741776,0.479895775117426,-0.260285196629901
"Cort",3.065513704554,-3.63800952280378,1.59179486943014,-2.2854763466
515,0.0222849118221007,0.0626548249037232,-10.0777032466858
"Car6",1.52481356730311,-2.42770820754154,1.64702774852333,-1.47399350
722424,0.140483339329373,0.254993869879363,-3.31750155979274
"Abcb8",2338.17477727636,0.904055630804867,0.249445242394124,3.6242648
7724491,0.000289784505539387,0.00224929072014609,5.51215406337499
"Dffa",1074.4644915639,0.049178532424708,0.11194219647367,0.4393207742
37937,0.660429123484461,0.769369460877227,0.0128938234393235
"Pex14",2034.08020829065,-0.250403971838146,0.310038482558657,-0.80765
4487828849,0.419289510368451,0.562263196228615,-0.14417890551912
"Slc2a5",1739.45249975666,0.504677825550142,0.686476397398332,0.735171
416617985,0.462235104516136,0.601525733688555,0.256520654038593
"Casz1",1958.1212723706,0.360588822164954,0.164759571766255,2.18857586
420849,0.0286276816681558,0.0759742122180077,0.929367678776658
"Nos3",1224.83994465619,-1.18700534954175,0.320088409897178,-3.7083671
6619341,0.000208600039978091,0.00174936928150229,-7.53570342189651
"Masp2",53.2140342095048,1.63366499154573,0.359569264744974,4.54339442
139031,5.53555363353109e-06,0.000107502215883057,14.9284292187598
"H6pd",9111.0388296497,-0.209584885618501,0.135186949621164,-1.5503337
1346733,0.12106143947795,0.228129501009091,-0.30973330972685
"Slc25a33",475.828861666767,0.326556493562162,0.324047111876538,1.0077
438791881,0.313577434021968,0.458743458025235,0.254473765166278
"Klhl7",1526.79388175358,0.00432319307676962,0.163472178923827,0.02644
6047916105,0.978901566053235,0.986669833155619,5.80164342572718e-05
"Ctnnbip1",1296.05994908111,0.345681231596696,0.22476541132314,1.53796
453627697,0.124057286130561,0.232337812407423,0.504543493405368
"Angptl7",192.089253076173,1.69388629262061,0.412034088293482,4.111034
35552181,3.93890580557195e-05,0.000482114158173521,12.9367679712505
"Lzic",546.408508289074,-0.729074073177886,0.269306709450109,-2.707225
80460979,0.00678480918392967,0.0251809053172351,-2.68420963059655
"Mtor",5133.14034880881,1.00565390338201,0.284183119480354,3.538753129
39385,0.000402021633156003,0.00290534388557293,5.87422911887929
"Nmnat1",776.467262988417,0.409162283125627,0.244683724285463,1.672208
82517006,0.0944831508533913,0.189082672209732,0.681488808707632
"Fam126a",1513.46472612632,-0.408443274331467,0.174245254745178,-2.344
07114804239,0.0190745323571782,0.0554352708153614,-1.18143819443446
"Rbp7",347.67723649693,-3.89205777920436,1.48629948881013,-2.618622833
76024,0.00882855002549179,0.0307281450191073,-13.5543880575059
"Tomm7",3755.71034071376,0.580278978554709,0.247005387193034,2.3492563
6703309,0.01881095005645,0.0548408947668454,1.68473504668043
"Rint1",1172.52668095626,0.371712420484817,0.175578587604224,2.1170714
8096386,0.034253781670527,0.0870656022698391,0.90738473463405
"Fbxo44",777.305043483487,0.840041043530862,0.167131749142006,5.026220
61842425,5.00240373018533e-07,1.57571676787001e-05,9.28935464082032
"Mad2l2",678.839276810404,-0.0625207798362806,0.154070379871327,-0.405
793637222776,0.684894234183577,0.788408771880469,-0.014863601267799

"Kmt2e",8662.02678651926,0.802263826590179,0.380307063882391,2.1095159
7480261,0.0349000686014113,0.0882252738999505,1.94778570200232
"Draxin",7.74882675643157,-0.437281939373125,0.598227661186338,-0.7309
62420737878,0.464802106983836,0.60365759554858,-0.220717244058693
"Agtrap",4042.11122284423,0.824455460101259,0.213676981662273,3.858419
62801755,0.000114122584034516,0.00110211101709014,5.61497685715219
"Mthfr",2324.90989636926,0.0506048155039746,0.171010233514909,0.295916
884410095,0.767293556339588,0.849739749379717,0.00823973685631798
"Orc5",832.505832644668,0.0791799501220491,0.128481431013833,0.6162754
37604086,0.53771274710977,0.66903400732284,0.0318240362018188
"Dnajc2",3475.33688212502,0.807152707397425,0.230610343006298,3.500071
57907649,0.000465133242076447,0.0032626253289658З,4.62112931921249
"Slc26a5",0.320386768280302,-1.9165164075799,4.7523482065286,-0.403277
774332079,0.686743890229271,NA,NA
"Clcn6",1445.37985430181,0.231522086794569,0.161222940284266,1.4360368
7159131,0.150991849657561,0.269378633417799,0.303673011733594
"Pmpcb",6031.50486446568,1.22315288013064,0.286171435844447,4.27419625
764293,1.91828014149995e-05,0.000279711746163075,10.0075322549791
"Nppb",1.53653831768835,-2.31431317024156,2.02012735683629,-1.14562735
978488,0.251949371656269,0.392041426196663,-2.16709453861766
"Mfn2",5103.67667832434,-0.296227065260721,0.365119621460251,-0.811315
108391044,0.417184735898467,0.56049812145886,-0.171494553606598
"Miip",1627.02919474708,0.222101839702694,0.20721826998293,1.071825566
92994,0.283798387255066,0.427825959076795,0.188573080296491
"Trp73",10.7838501884132,-1.10204791932978,0.79658970663782,-1.3834573
9362013,0.16652466278002,0.289587263650086,-1.36576644195649
"Dffb",401.673426779766,-0.21048590913579,0.212428084549492,-0.9908572
5685556,0.321755286219025,0.466993045310299,-0.160272582971921
"Lrrc47",2382.00248948049,-0.199265031363327,0.137551705170628,-1.4486
554791607,0.14743381942609,0.26449994437155,-0.265005402345467
"Wrap73",437.812410119946,-0.0906115229603716,0.177292772444112,-0.511
084133386967,0.609292145637621,0.729135527146067,-0.0286237864829779
"Tprgl",5057.61653313716,-0.0234756727258971,0.105074879501865,-0.2234
18507232078,0.823209801645971,0.886645567723203,-0.00282435729260346
"Arhgef16",3276.77618220695,0.835219551642892,0.345952799508587,2.4142
5868739692,0.0157672634247808,0.047901231264627,2.53790988115898
"Acap3",1064.79162789709,-0.198171489142411,0.149870526646649,-1.32228
459842302,0.186073399417662,0.314689837960411,-0.229119488011484
"Ints11",1321.04913753381,0.0223077723425831,0.112064258535344,0.19906
2329364785,0.842213988284126,0.898991228193709,0.0023753762558744
"Atad3a",3400.174487709,0.665043198968988,0.189366443042649,3.51193795
628937,0.000444851837852793,0.00315064334705912,3.83074765802246
"Ssu72",2406.86568810771,-0.344172872930252,0.290806236423192,-1.18351
269616309,0.236606017804355,0.374733501352393,-0.337819499762296
"Pex10",713.868917814295,0.531579837698735,0.201932381995266,2.6324645
5296703,0.0084767868004668,0.0297427369147925,1.86859366506054
"Rer1",7616.64076173647,0.535674899845399,0.212703952382376,2.51840595
271319,0.0117887361526179,0.0385148755870886,1.74453819595452
"Morn1",194.249044880721,0.454723441012416,0.186366150691261,2.4399465
2100597,0.0146894363325918,0.0455204433586897,1.40491070103294

"Ski",5160.24731358152,-0.396346454565289,0.127202293102838,-3.1158750
7502604,0.00183399949422324,0.00933907603509136,-1.85234416308259
"Prkcz",2535.30859674186,1.7355131627917,0.373274158872717,4.649432920
92043,3.32848910975859e-06,7.17114703448321e-05,16.5617588738229
"Gabrd",19.3695322830572,0.186759896207555,0.514018355836936,0.3633331
26311159,0.71635605048028,0.812017340402564,0.0388896825110312
"Plch2",770.3258442214,-3.07429733869338,0.649699258403475,-4.73187755
55446,2.2245259151034e-06,5.20069619816381e-05,-30.3252777199958
"Pank4",1593.48165718923,0.213623676011172,0.166561734929572,1.2825495
3697198,0.199649939319576,0.331130870776887,0.23610577384771
"Prxl2b",658.543827068327,-0.328218457608092,0.33228377850371,-0.98776
5515024766,0.323267500156955,0.468142433011015,-0.249112126329313
"Mib2",3327.91640303088,0.0682916172799131,0.130954249640938,0.5214921
81179007,0.602023950934823,0.723601466855331,0.0220933283023929
"Mmp23",1278.56231841957,-1.05622267906835,0.221758097426605,-4.762949
76970537,1.90783344016016e-06,4.58114980495602e-05,-10.5526948554967
"Cdk11b",6681.51247885749,0.180814507784996,0.169826784491191,1.064699
58980101,0.287011895769982,0.431037352208512,0.152166352745864
"Nadk",7067.35806738154,-0.170694866064309,0.255483234700606,-0.668125
508369822,0.504053480369317,0.638889342796654,-0.0764753987326684
"Gnb1",11216.3203141144,-0.599518008299872,0.135955282026727,-4.409670
58699502,1.03527979391307e-05,0.000176068399660679,-5.18261615862501
"Mrpl20",3203.46541749179,0.945868443505424,0.275951379974792,3.427663
39342759,0.000608799815289321,0.00400890874654211,5.22047136114479
"Ccnl2",14839.1559721182,0.370175730958043,0.206602551534657,1.7917287
4782211,0.0731764272119166,0.155413875443659,0.689142667812509
"Mxra8",3719.06164209231,-1.3199692755007,0.274977594865355,-4.8002793
6875014,1.58444446096696e-06,3.9455297701065e-05,-13.3849401875703
"Dvl1",6486.35578797868,0.554199842658735,0.221076922452723,2.50681905
877015,0.0121823055741639,0.0394045444732992,1.79221253256534
"Tas1r3",18.4843662582692,-0.997386297216597,0.41177418792995,-2.42216
808739423,0.0154282124398198,0.0470986826603423,-3.04752405170756
"Cptp",733.3042258895,0.37850752696342,0.244670892621934,1.54700676859
135,0.121861613508136,0.229327066902966,0.557392475933617
"Ttll10",618.513056996786,0.739723064128689,0.26598418529796,2.7810791
2055012,0.00541785311000163,0.0211147911130945,2.85368993968816
"Tnfrsf4",161.732057029867,-0.945308001662758,0.386976604159108,-2.442
80401322166,0.0145736492037564,0.0452984237485801,-2.92523958149676
"Sdf4",7000.86078964466,-0.408493454471891,0.249586634953816,-1.636680
00310785,0.101697349390842,0.199854774949045,-0.657741577764794
"Bst1",526.460618653741,0.110803915369975,0.440955509976972,0.25128139
4297038,0.801596558973381,0.873450861007208,0.0149921471783808
"Cd38",929.096145205512,-0.851154093671236,0.132920061454327,-6.403503
61230992,1.51851260917558e-10,3.36454077883017e-08,-14.6461398100908
"Prom1",4874.87374920907,0.211196005168816,0.498346605434655,0.4237934
05765476,0.671716487669719,0.778062619650934,0.0529992721405965
"Kcnip4",25.4620535419096,-1.33444490871271,0.64497034024092,-2.069001
97645406,0.0385459012310974,0.095328257509378,-3.13651801388396
"5730480H06Rik",527.52814248489,0.229763186506089,0.143460144389706,1.
60158201069381,0.109248071450225,0.210756792038834,0.35775287401739

"Adgra3",2084.54424655443,0.686894246963043,0.295850166743164,2.321763
93383396,0.0202456502540587,0.0580172969802523,1.95559759882424
"Sorcs2",528.736131900772,-2.67071658176389,0.637571728250347,-4.18888
803161487,2.80324660486312e-05,0.000369808540606367,-21.1054049400848
"Afap1",2714.21779309353,-0.645714680153178,0.225541078718934,-2.86295
819733068,0.00419705932019557,0.0173967395036508,-2.61609525345254
"Ablim2",394.486505156322,-0.479268311170507,0.269915278935061,-1.7756
2497781318,0.0757947502477731,0.159435920964036,-0.87999086621398
"Htra3",1294.64576203214,-2.8409815830795,0.6149051323143,-4.620194943
5468,3.83379651451674e-06,8.11716389097534e-05,-26.7590482722336
"Trmt44",343.640380832275,-0.0203830596145824,0.151457179519807,-0.134
579685685464,0.892944203944884,0.93382853574598,-0.00139547395874237
"Acox3",7068.92778833903,1.18885109011822,0.270738938691395,4.39113448
499311,1.12760766328829e-05,0.000187795454709123,10.2005292338409
"Rgs12",878.702553288259,-0.49941835433592,0.266452639003615,-1.874323
16753727,0.0608858962007415,0.135095543015473,-0.999722189811209
"Hgfac",413.21380336128,1.10524027548287,0.237049437548722,4.662488495
69915,3.12408556004299e-06,6.85190329018203e-05,10.5974847507724
"Lrpap1",13604.5643006562,1.15283377973494,0.386464229250572,2.9830284
2146737,0.0028541148924677,0.0130113472465128,5.00552753568346
"Htt",2759.90408285511,0.0888716887629262,0.142338536542167,0.62436843
1219598,0.532385652336318,0.664724895777591,0.0362935992872294
"Add1",8681.48373028514,-0.145485008102602,0.0776561433833372,-1.87345
136861148,0.0610060822301894,0.135262291485585,-0.291048503277811
"Pcdh7",515.008367888294,-1.28629032240728,0.312927420578522,-4.110506
90294019,3.94791477246876e-05,0.000482610923094645,-9.82249849467277
"Rnf4",2835.21979789038,-0.898597324687541,0.267284289451819,-3.361953
39625273,0.000773931844075334,0.00482883939064988,-4.79235354215985
"Nelfa",917.876318564544,0.0395343399716317,0.0785705096584247,0.50317
021161632,0.614844606245809,0.734168665109975,0.0122167628850068
"Nkx1-1",0.0789318165836824,0,4.78471990596069,0,1,NA,NA
"Man2b2",2037.43013372546,-0.16950503685406,0.135281753941504,-1.25297
781789076,0.210213779526844,0.34398815734479,-0.180886969293905
"Ppp2r2c",45.8496813222877,-0.613469008086012,0.570702836023218,-1.074
93597256463,0.282403395203988,0.426349314726083,-0.522980047962838
"Crmp1",46.339550203947,-1.4702870860932,0.656795488624328,-2.23857671
308423,0.0251834708340064,0.0688677860198398,-3.93385125105983
"Evc",6391.60477797046,1.24868783460089,0.365121896248664,3.4199204359
699,0.000626394535846145,0.00410673862136118,6.861697096012
"Stk32b",284.106793535843,1.50311585894508,0.479530924747445,3.1345545
8526837,0.00172115229027838,0.00893458103779566,7.09143911262466
"Stx18",1217.59291933139,-0.326467610901436,0.212188479354429,-1.53857
368644468,0.123908406585421,0.232103574945003,-0.476829308792958
"Nsg1",204.438467434866,-1.41495988558499,0.216833885983184,-6.5255477
9050875,6.77534499555482e-11,1.76221515544072e-08,-25.2628481909267
"Zbtb49",337.220213909401,-0.12063479639785,0.163330029911473,-0.73859
53242232,0.46015276433829,0.599618143593097,-0.0617001448422969
"Rab28",1367.18797681939,-0.37556066442295,0.24560011715542,-1.5291550
7033447,0.126226005849205,0.235194011486839,-0.543565671325455
"Rnf32",135.37290140963,0.533288801158988,0.289697177124079,1.84084914

617783,0.0656436684449923,0.142816363238168,1.03788434348351
"Dnajb6",4842.38316384414,0.00569023899216897,0.174474207148861,0.0326136400626488,0.973982692420283,0.984025832261292,9.1630658087724e-05
"Plb1",22.8222329117719,-0.679632464821342,0.993516936576035,-0.684067316621259,0.493932658964116,0.629951529597222,-0.31406658923509
"Fosl2",3821.37077784034,-1.49046629447969,0.153005228176375,-9.74127689781664,2.01012445564806e-22,6.53223443937097e-19,-62.4093527291768
"Rbks",405.766537497156,1.10201969854363,0.260696903356604,4.22720670769222,2.36610286184401e-05,0.000327425646559507,8.84288108343235
"4930548H24Rik",53.1197957590416,0.354209424646882,0.407632035339883,0.868944032702293,0.384877743624501,0.529481848951564,0.225226327522361
"Slc4a1ap",966.73074350121,0.275729794028613,0.107326192402853,2.56908204656744,0.0101968312044516,0.034367815872843,0.929384421951708
"Eif2b4",1651.63499013004,0.45724388959439,0.206593664953288,2.213252229674,0.02688026104216,0.0723910676519386,1.20057264368139
"Snx17",3396.15723429183,-0.0928950468339337,0.169405348320662,-0.548359586960004,0.58344501993349,0.70750736977817,-0.0321423581024692
"Ppm1g",3569.66288706854,0.0547456114666375,0.0568263767128013,0.963383812825513,0.335354946605556,0.479895775117426,0.040193479775944
"Nrbp1",3898.27197105867,-0.151215926869783,0.0764667956440644,-1.9775371204733,0.047980948893049,0.112809904921822,-0.329960884732683
"Krtcap3",758.739259734881,-0.119842947945219,0.238245462250676,-0.503023003305403,0.614948099270195,0.734247277377236,-0.0370206151744458
"Slc30a3",10.5729483444059,-2.142981246383,1.00560420517474,-2.13103846956429,0.0330859730611933,0.084813450231217,-5.28738006693737
"Ociad1",5376.88889102734,0.0627494570684264,0.204861479238355,0.306301884091239,0.759374804819118,0.844144238561183,0.010631759748785
"Ociad2",976.634133062306,-1.12501093429971,0.356581632096824,-3.15498845996147,0.00160504453797667,0.0084833717542611,-5.36590536038679
"Cwh43",414.205760943192,2.8203904446123,0.520721105161886,5.41631675123957,6.08392821323417e-08,3.14653666582599e-06,35.7321136949333
"Spata18",0.833385576587899,-1.24233704359448,1.74733706498998,-0.710988777429504,0.477091190236011,NA,NA
"Sgcb",998.91760860253,-0.684080559732169,0.239821169160029,-2.85246111562275,0.00433821196249968,0.0178301974799365,-2.75469785458231
"Yipf7",2.35208504694664,-1.59632712893506,1.62951208765154,-0.979635033720857,0.327266305155701,0.472048788280162,-1.19831956908329
"Cgref1",5793.06064268979,2.15660718235406,0.668373084908226,3.22665174742963,0.00125247798549357,0.00698935768779439,10.7040320951304
"Khk",17471.1807244426,1.95310955156699,0.532258081875945,3.66947843174733,0.000243045841689954,0.00196635179305839,12.1709493076707
"Emilin1",4171.6918042613,-1.27676045056926,0.362953064372342,-3.51770125643428,0.000435302121432971,0.00309311981184405,-7.37785608394759
"Agbl5",1078.64861801008,0.418916261805767,0.204892878526242,2.04456233334685,0.0408980261801031,0.0996164540236914,0.966200165301041
"Mapre3",2096.27808732485,0.414444338631016,0.155362593392163,2.66759410732082,0.00763964905443981,0.0274880747856555,1.48951420156107
"Ppargc1a",2704.47437262207,1.19501002253325,0.275588723964705,4.33620797448264,1.44961794501553e-05,0.000225395938532,10.0352782764163
"Dpysl5",31.6114583459444,-0.394616164286413,0.52169287976936,-0.75641

4702191973,0.449400595869196,0.590220451182647,-0.208064989592218
"Dhx15",4461.90176583965,-0.234546377792666,0.130237284855858,-1.80091
575198495,0.0717161598261572,0.152822041998952,-0.440590951532706
"Pgm2",939.948644250674,0.323155455508736,0.218752634193527,1.47726429
306833,0.139604805512242,0.253753565570775,0.443172708594225
"Sepsecs",1973.71143126154,0.656809897648796,0.15463055832127,4.247607
36027458,2.16065632847802e-05,0.000305304920043773,5.31635037332994
"Tbc1d1",3406.61493806827,0.192065383153344,0.164841735109806,1.165150
21529835,0.243958193540969,0.38298686454604,0.184335632411209
"Slc35f6",1515.87439793544,-0.287158048975767,0.0848299244372208,-3.38
510320362575,0.000711514975310101,0.0045248268064567З,-1.5501296816919
9
"Anapc4",2185.62209101764,0.200993208879635,0.192561325504341,1.043788
04182621,0.296583465392365,0.440961897202114,0.164572597894552
"Cenpa",506.95511751129,-0.493659373599064,0.307313615438629,-1.606370
00379714,0.108192637530151,0.209134534208672,-0.772467095162418
"Klf3",3042.27716600395,-0.526961222691734,0.111465524917747,-4.727571
35518439,2.27221225269878e-06,5.27423744084772e-05,-5.19061618512833
"Zcchc4",979.810869483031,0.296325258900492,0.173921309078012,1.703789
26234724,0.088420464725377,0.179623069516087,0.508759261131204
"1700001C02Rik",0.163890645083712,0,4.7847199059659,0,1,NA,NA
"Olfr109",4.69287517654586,-0.304214169435442,0.894831901882607,-0.339
967952411414,0.733880662275525,0.825975822734252,-0.0581626389672939
"Fam114a1",1121.95787885814,-1.12400892510634,0.194100843735643,-5.790
85027902915,7.00309836862256e-09,6.01526044014989e-07,-16.100074549924
9
"Pi4k2b",1209.82249210853,0.0226826475791644,0.112008150084139,0.20250
890280864,0.839518899161722,0.897124821651609,0.00246243439159679
"Slc34a2",664.791561976782,-0.229448748768421,0.647484314881675,-0.354
369586250675,0.72306192427283,0.817195768575913,-0.0463203319003466
"Sel1l3",1990.7163639473,0.796693816236713,0.262165442987314,3.0388971
4509499,0.00237445906646172,0.0113892258002142,3.56527451902418
"D5Ertd579e",3499.78250953079,-0.241806978466098,0.242907682410883,-0.
995468632634998,0.319508392789745,0.464665729102311,-0.185329813714814
"Rfc1",1810.54723067724,-0.157896642722788,0.12606010295029,-1.2525504
8209069,0.210369345478034,0.34419581254768,-0.168403805501218
"Tbc1d14",2473.93749675487,0.334243781278514,0.171266062474117,1.95160
545206689,0.050985065452585,0.118135092833571,0.713920134798822
"Cckar",2090.56710939542,1.868808183267,0.777065539489418,2.4049556804
371,0.0161744272177791,0.0489323478498459,5.63878593673249
"Klb",170.320870535638,-4.59032486718581,1.3093908052539,-3.5056950520
5568,0.000455416674209601,0.00321019062813076,-26.3550057572973
"Tada2b",1057.44598758388,-0.0522442968353305,0.230982735571004,-0.226
182691559952,0.82105933194719,0.885258507758588,-0.00636730384603869
"Grpel1",3149.64085637887,0.182094501211008,0.392526527575517,0.463903
681454944,0.642716755110106,0.755968588474202,0.0509419297302898
"Lias",2137.01644447048,0.181542688749939,0.275959283113348,0.65786041
5862042,0.510627828405964,0.644832992115251,0.0796543822474072
"Ugdh",5404.12954309039,-0.618970219522428,0.401567526908547,-1.541385
14208942,0.123223077971131,0.231064009836614,-0.906828824241326

"Pds5a",3964.68048045405,-0.0973171168617847,0.0784661388025006,-1.240
24347759397,0.214885351574814,0.349677398181082,-0.102255402820644
"Ube2k",3532.3355062608,0.0369130132080788,0.218879765556505,0.1686451
6057127,0.866075756287717,0.915816752323765,0.00324609296535792
"Rhoh",226.040435413677,-1.67041755035643,0.452725697075763,-3.6896901
6149505,0.000224527342873011,0.00185344374739118,-10.508112196492
"Chrna9",0.409070079979442,-1.15224946028544,3.18367318097848,-0.36192
4542748229,0.717408419017919,NA,NA
"Nsun7",186.305692168715,0.736609946406977,0.37269683571336,1.97643198
391279,0.0481058720777533,0.113076346446297,1.60558284865583
"Apbb2",2502.56642372131,0.282999439084366,0.130794560671146,2.1636942
5175031,0.0304878224669989,0.0796424922912037,0.71604730491147
"Guf1",2328.018991489,0.391843318227463,0.109115731156119,3.5910799852
2071,0.000329310545010781,0.00249065050683483,2.34918351077896
"Gnpda2",962.179181369735,0.231940181309231,0.120310966852833,1.927839
06053173,0.0538751475438329,0.123059855728471,0.485934257922322
"Gabra4",111.507370115515,0.00764005236338871,0.574869341468196,0.0132
900675201713,0.989396372460413,0.992978528091061,5.38336306663312e-05
"Gabrb1",90.4920745923692,0.19634901827344,0.532006302948542,0.3690727
29374096,0.712073515061503,0.80895003185161,0.0416295515161045
"Commd8",2205.12001546582,-0.24728141651135,0.163247466818695,-1.51476
418795511,0.129832132648926,0.239881258636289,-0.353021728110268
"Tec",744.030611830533,0.232933600135087,0.327452081858311,0.711351715
381298,0.476866312024628,0.614293033288597,0.113504632803189
"Slc10a4",20.4260337153018,0.261904480648253,0.676017841827539,0.38742
2438348999,0.698443494934998,0.798911842323278,0.0587987801303318
"Slc30a9",6549.34017133796,0.724875748938484,0.153250456211732,4.73000
711943715,2.24511963640346e-06,5.23628500844433e-05,7.14532727817073
"Uchl1",1356.33968437811,-1.69924242786455,0.49294336263341,-3.4471352
2216188,0.000566564930897416,0.003803927744183,-9.46770493892125
"Fip1l1",2709.46795436639,-0.166331430486387,0.134106999577332,-1.2402
8895591294,0.214868535854036,0.349677398181082,-0.174771797342451
"Lnx1",289.293652443836,0.513945430081939,0.330492431221438,1.55508986
448644,0.119924668509982,0.226381177907602,0.763483961768477
"Chic2",1475.88342536944,-0.496289699774083,0.227441128429258,-2.18205
785031728,0.0291052634605157,0.0768337973129077,-1.27353429224057
"Pdgfra",3594.12989338485,-1.26392709539821,0.285688445658142,-4.42414
495443275,9.68249955179797e-06,0.000167070244478723,-10.992495549037
"Srd5a3",852.117931646429,0.0191793749721779,0.185186516522722,0.10356
7880277205,0.917512278375059,0.949355466130168,0.000996790118330657
"Tmem165",1883.19540791237,-0.304685462307557,0.136008146079216,-2.240
20009897126,0.0250779352513397,0.0686292075218737,-0.816263647979796
"Pdcl2",0.100283711116373,0.85015961336909,4.78471990596623,0.17768221
1305409,0.858972556261423,NA,NA
"Clock",2602.03103781905,-0.248154171650977,0.159575391842042,-1.55509
047345229,0.119924523497745,0.226381177907602,-0.368641725389515
"Epha5",795.36114010709,0.97173861445113,0.383628385880707,2.533020626
77214,0.0113084298247447,0.0373208809618945,3.19527314476064
"Ppat",1621.83138875805,0.634532761315283,0.277075679639113,2.29010630
648548,0.0220151548840744,0.0620843925267114,1.76353193447107

"Paics",7705.30923544124,0.394318249587914,0.228722594833897,1.7240021
6897799,0.0847074516062496,0.173837058353716,0.689913745922836
"Thegl",0.251688987505115,0.0331084650725692,4.78471990595309,0.006919
62449701099,0.994478982506144,NA,NA
"Rest",2165.49549436541,0.313539354500479,0.233695068371039,1.34166012
439197,0.179706221832214,0.307123200566775,0.370135185517646
"Polr2b",1714.809634436,-0.245557082498587,0.207117682733075,-1.185592
07141696,0.235783434866479,0.373643157755738,-0.24173966699473
"Cenpc1",697.65764380259,0.0156265184713292,0.21364064566651 8,0.073143
9395466037,0.941691576575317,0.963817152236969,0.000575894687271433
"Stap1",520.791474781058,-0.606385451606327,0.396096095447478,-1.53090
48954933,0.125792899158364,0.234706341352565,-0.878907296784387
"Ugt2b34",556.831890609068,-0.576689919623278,0.494350455992418,-1.166
56091368534,0.243387735710842,0.382214406482764,-0.554645113734022
"Pigg",1043.54091032587,0.431094442378269,0.197004158473109,2.18825047
003824,0.0286513625925944,0.0760267103743067,1.11078835314602
"Dr1",1848.24047191912,-0.160371810993262,0.19615172809075,-0.81759060
9852112,0.413590991161782,0.557052354056913,-0.0938329132318791
"Mtf2",1694.90013894303,0.177783482975967,0.145383028885411,1.22286269
820457,0.221381559496106,0.357240407713861,0.183000772692049
"Ugt2a2",15.591163217479,2.00001120420232,0.984858324853787,2.03076031
722557,0.0422793143287627,0.102252799650486,4.56063975700057
"Sult1b1",82.9045237404373,1.85442139099869,0.598417295317133,3.098876
66267389,0.00194255869860554,0.009767202047965,8.58360709056749
"Dipk1a",961.655354431661,-0.0560741336835605,0.137577432791444,-0.407
582352322013,0.683580322752177,0.787268111814645,-0.0134121708884502
"Sult1e1",15.7489873057987,-4.12710991567783,1.11664672859997,-3.69598
531923548,0.000219035629868585,0.00181811694813864,-26.0418735450015
"Sult1d1",15414.9422254559,1.96741903747312,0.702234233128774,2.801656
40559471,0.00508409899122552,0.0201442312804136,7.68245128237304
"Gfi1",65.2227612178844,-1.50442637800074,0.509012492251887,-2.9555784
99351,0.00312083268118882,0.0139295754550897,-6.42952864601924
"Glmn",284.18888967473,0.182368115660955,0.209778789543814,0.869335341
564004,0.384663737651955,0.529262123825969,0.11603562751357
"Brdt",124.760072276054,-0.108883339945158,0.257733171011212,-0.422465
371911408,0.672685370967982,0.778687386312288,-0.0272366900253662
"Cdc7",271.681196583178,-0.882085888684591,0.265628433011479,-3.320751
01556042,0.000897755882061235,0.00542437068126122,-4.60171275842487
"Enam",0.0789318165836824,0,4.78471990596069,0,1,NA,NA
"Tgfbr3",2115.75707077431,-0.63372191083701,0.179313135716019,-3.53416
334116563,0.000409068192678193,0.00294643946096764,-3.6927975757521
"Zfp326",1768.39743306088,-0.0580835983269365,0.196808221886441,-0.295
12790558339,0.767896173055276,0.849936453053083,-0.00944402677831893
"Rufy3",4021.7351239037,1.08113305589824,0.200812665931914,5.383789169
27879,7.29339261541422e-08,3.56407441642472e-06,13.5623884020382
"Gbp9",1104.33650423543,-0.960901368122446,0.319086478488905,-3.011413
62264229,0.00260034373383659,0.0121878610871024,-4.23499483590011
"Abcg3",141.224503105621,-0.952253193796939,0.344194888702385,-2.76661
050193666,0.00566423848314385,0.0218220355550956,-3.64221135823461
"Spp1",147211.097291917,-0.166074280313068,0.554905197046017,-0.299284

06004692,0.764723315775433,0.847721599351265,-0.0274359692882371
"Ibsp",8.74147643562378,-4.88772509620626,2.01601568402378,-2.42444795
193796,0.015331680867122,0.0468994530196337,-14.9552132769555
"Dmp1",2.12197930043872,-0.485636584975079,1.07004432817898,-0.4538471
65193187,0.649938833682262,0.761477429343634,-0.132333418268718
"Sparcl1",7371.25298780017,-1.64398226394225,0.256296105968107,-6.4143
8642905808,1.41391148590649e-10,3.16878691404653e-08,-28.387187399495
"Nudt9",2442.19350219728,0.14696648158305,0.17245770460129,0.852188552
102247,0.39410944945886,0.538572052533561,0.0909478540540483
"Hsd17b11",8875.81866381589,0.820265928692792,0.311621568731628,2.6322
5017456739,0.00848213788196441,0.0297561576866754,2.88300439830061
"Klhl8",467.795588779031,0.332742884735395,0.154759675919909,2.1500619
1217146,0.0315503178501972,0.0816522560981078,0.833616033700804
"Aff1",3553.60529485753,0.0420414682523218,0.122296170147136,0.3437676
60113486,0.731021023081676,0.823832860708391,0.00814711559437252
"Gpat3",640.279533996632,-1.68243599664639,0.673001546808386,-2.499899
15272127,0.0124228664600593,0.0399572831141927,-5.4173502272089
"Coq2",3719.43440662198,0.285406290206634,0.12965291185399,2.201310299
36333,0.0277140642447474,0.0740364568469885,0.7429689831051
"1700016H13Rik",1.8790664133808,-3.98541175467465,1.51690835710126,-2.
62732533314708,0.00860590109462775,0.0300605266110806,-13.967044380645
3
"Slc10a6",150.831051746067,0.108329113660105,0.281992487639224,0.38415
602687508,0.700862807840936,0.800973102296822,0.024041254071795
"Plac8",1747.56793101079,-1.05973104914138,0.375603753302657,-2.821406
97430003,0.0047813502538793,0.0191864102181804,-4.18970292330815
"Enoph1",627.756607896934,-0.315872138512831,0.111045378494979,-2.8445
3205341737,0.00444767105941295,0.0181523546702427,-1.26631718677374
"Hnrnpdl",8074.36220990601,-0.255425991721384,0.0937865526496501,-2.72
348203985657,0.00645977278246412,0.0242116654104184,-0.950419865076664
"Cds1",2520.35657161164,-0.232172168642277,0.225872077221128,-1.027892
29859068,0.304000492656895,0.448739617368775,-0.186042455366428
"Prkg2",394.622155481857,-0.93597717220206,0.415021004135959,-2.255252
53631606,0.0241174819997726,0.0664653942094086,-2.53750324005712
"Bmp3",671.549721467944,-1.60802608433876,0.450015344154076,-3.5732694
5675924,0.000352551643747329,0.00262168266582206,-9.55800884073309
"Fgf5",0.350618861579457,-2.43677084655973,4.73945718277076,-0.5141455
55617228,0.607150229201517,NA,NA
"Antxr2",815.983089018644,-1.2329146210739,0.187243536729342,-6.584551
02167857,4.56262029934164e-11,1.30826427347887e-08,-22.3798408867677
"Crybb1",12.5856076811532,-1.53502693726919,0.850834855819445,-1.80414
20456274,0.0712090418426855,0.152073811374445,-2.8910532702652
"Tpst2",1279.7177099165,-0.508017589442871,0.120790219718467,-4.205784
13241519,2.60178485269487e-05,0.000350778232908731,-4.0414609557063
"Tfip11",1368.83233542877,-0.0299248959770512,0.0858246019978473,-0.34
8675033503817,0.727333289730534,0.820786230070954,-0.00590994494074015
"Srrd",468.255358850225,0.0448336127455301,0.13196405241349,0.33974110
3168389,0.734051505063428,0.826072737257689,0.00856646821637674
"Asphd2",29.9104154613239,-3.07848033534427,0.631804171389651,-4.87252
296636972,1.10182042929417e-06,2.99210233013619e-05,-32.0683732793917

"Crybb3",118.558578755226,-0.534915828509879,0.241423688876884,-2.2156
7250089805,0.0267139395349759,0.0720725602674638,-1.40687243106876
"Tesc",817.83668663486,1.38743599082445,0.41853405047196,3.31498951939
491,0.000916465606364471,0.00550500505018314,7.21757742612669
"Nos1",41.7850304056128,1.11215835485443,0.292130112303858,3.807065098
77909,0.000140625737792225,0.00129720639021244,7.39311966079865
"Rfc5",623.502872123265,0.00239255547595892,0.365587932791811,0.006544
4049470894,0.994778357606261,0.996772106305919,7.73540599502296e-06
"Wsb2",4530.87436175469,0.233101101797075,0.135261064695585,1.72334220
732098,0.0848266593259268,0.174026747005147,0.407588062816925
"Dck",400.837815997286,-0.974413515575322,0.255198596497149,-3.8182557
7785342,0.000134398543016245,0.00125203191195927,-6.51199336180808
"Alb",331.660394070093,1.2986786990213,0.575485246997638,2.25666723134
369,0.0240288758958461,0.0662774115457924,3.52449209582183
"Afm",1038.97371348935,2.20892955568358,0.635333598347581,3.4768026772
5289,0.000507431117956078,0.00350226263288765,12.4900521141301
"Rassf6",1193.20903957344,-0.0204869300474274,0.153515883144457,-0.133
451533664106,0.893836291868807,0.934428181805694,-0.00138943398351185
"Cxcl5",238.953551559582,-1.2383074681946,0.706548544935544,-1.7526148
4447267,0.0796681414753796,0.165869666042387,-2.22468492673971
"Ppbp",55.6924471604013,-0.12741305520013,0.544746562831785,-0.2338941
88405324,0.815067125072046,0.882212657080868,-0.0159676772813048
"Pf4",362.034313373989,-1.57552966166593,0.250842534459336,-6.28095097
612456,3.36508249802262e-10,6.56123785464451e-08,-26.0584751416706
"Cxcl15",4.21058465552941,-3.85299478070669,1.51129389194047,-2.549467
57957152,0.0107887541760502,0.0359809069545836,-12.8103092954211
"Mthfd2l",736.960542833417,0.460948081511495,0.132918559485526,3.46789
856356884,0.000524545247632853,0.00359697894590595,2.59405952637682
"Ereg",11.0873774881045,-3.42973686310574,0.843114588924482,-4.0679368
0024073,4.74312408580991e-05,0.000556112047054249,-25.7043027184655
"Areg",27.4062460285429,-2.23712326332672,0.706415656298825,-3.1668653
4815471,0.00154091650056003,0.00822245592804336,-10.7401745154372
"Cxcl3",9.44444525260655,-2.21210552314088,1.17472484576955,-1.8830839
673709,0.0596889896002965,0.133053151849386,-4.46183142376631
"Cxcl1",619.620183116624,-0.555557172669938,0.502888482945497,-1.10473
234426836,0.269275583955197,0.411532790089232,-0.49326064563746
"Shroom3",2562.29591620813,0.0511345537314299,0.25690962837621,0.19903
7124667629,0.842233704247332,0.898991228193709,0.00544490964507966
"2010109A12Rik",17.5825556505292,0.318553723077617,0.471664324097178,0
.675382272524782,0.499432925407756,0.634828199671814,0.144751088396321
"Ccng2",2057.33978832015,-0.30700207129887,0.12588402593479,-2.4387690
8939901,0.0147373819032723,0.0456400051381836,-0.947706380950922
"Tctn2",506.766075276748,-0.0231185797114826,0.1434506136586,-0.161160
549417396,0.871966951900603,0.919801537987338,-0.0019326520409993
"Gtf2h3",1053.8938904871,0.0391569373660589,0.120530193874387,0.324872
433266532,0.745277621258268,0.834419928756458,0.00708812973942138
"Eif2b1",1440.60432660615,0.165654049367336,0.190837545529107,0.868036
994020497,0.385374080981271,0.529865582890687,0.10521217472691
"Ddx55",1415.85979049961,0.198332275525583,0.219515375167781,0.9035006
10715733,0.366260264269416,0.511191312292418,0.133083212073584

"Tmed2",10158.8482970886,-0.170388575669695,0.174183393267998,-0.97821
3665912087,0.327968658863993,0.472637279873301,-0.127693805033447
"Rilpl1",766.055812090313,-0.191390816251459,0.17755118065266,-1.07794
730256327,0.281057274034648,0.425008122314842,-0.163762977544745
"Cdk2ap1",3557.18466831209,-0.24029887415225,0.121851361904261,-1.9720
6555919378,0.048602122982117,0.113951028577994,-0.521925909844881
"Rchy1",2267.33233960941,0.228702840563745,0.156975982544611,1.4569288
6807541,0.145136016368292,0.261394979415201,0.306855789193567
"Rilpl2",1526.32837308149,0.165010543600993,0.197338865546373,0.836178
636905241,0.403054414760887,0.546886219833526,0.0995862566754023
"Snrnp35",361.897246351091,-0.19985424423955,0.199214420554447,-1.0032
1173378575,0.315758712741048,0.460966111187852,-0.154773272299225
"Cdkl2",743.898981285733,0.102591038046395,0.140783610165372,0.7287143
57629317,0.466176408366444,0.604918648364763,0.0515685440916427
"Arl6ip4",2467.04969211107,0.333855070713064,0.12706460106415,2.627443
58316218,0.0086029106105882,0.0300566581904305,1.17005220861766
"G3bp2",4785.17342657375,-0.602110473094578,0.232274383584948,-2.59223
795496318,0.00953537927063857,0.0326103757462444,-2.06109927281378
"Pitpnm2",1629.9951011921,-0.697721847270891,0.341615635667067,-2.0424
1777724389,0.0411101078249708,0.100008095118064,-1.60650744546092
"Uso1",3654.74177137786,-0.052105706028895,0.160929798751455,-0.323779
103889694,0.746105277411833,0.834819275736022,-0.00940716488558162
"Abcb9",572.399763444846,0.21460321451969,0.180842613896784,1.18668498
478005,0.235351899882079,0.37323231751938,0.211503103276336
"Ppef2",95.6393757540471,-0.642585668108127,0.483438295415503,-1.32919
893645546,0.18378234588244,0.311978772419973,-0.748496721849166
"Naaa",2628.34165911449,-1.05249593064618,0.277269588389726,-3.7959299
3504505,0.000147091039885878,0.00134394615543339,-6.95925581122917
"Kntc1",85.1100369485516,0.0440949129108751,0.543364505857376,0.081151
6255396507,0.935321369793519,0.959685105942958,0.00181450844300871
"Sdad1",1236.33415669281,-0.197579757095722,0.168858786259665,-1.17008
869643236,0.241965276847315,0.380624362078979,-0.190850648263015
"Slc15a4",764.583813862949,-0.305831209536796,0.180336706030048,-1.695
88996200163,0.0899067210106721,0.181903211192911,-0.521222178937306
"Cxcl9",1051.05533375171,-1.67644515383845,0.733825127812922,-2.284529
50205575,0.0223404307830407,0.0627140236052259,-4.6423619525332
"Ajm1",110.240570754725,-0.483694406082896,0.403389741336561,-1.199074
63308378,0.230498926706448,0.367257668854232,-0.484512614983807
"Rimbp2",2.65269689146716,-0.816360318072543,1.80297355312602,-0.45278
5520152044,0.65070319122418,0.762051070880455,-0.221839143411849
"Rsrc2",4577.33804781361,0.0223247610944341,0.144249126471775,0.154765
312210069,0.877006338503799,0.923169550728666,0.00178469422417589
"Piwil1",0.121527413527063,0,4.78471990595379,0,1,NA,NA
"Scarb2",4951.58476686892,-1.19339001355845,0.263555214531067,-4.52804
553945858,5.95317537070721e-06,0.000113465311219989,-10.8407708695482
"Zcchc8",2729.5577831987,0.291317354412063,0.167131533427951,1.7430424
3153281,0.0813262032730643,0.168386780441564,0.518979443473146
"Stx2",1242.67688095487,-0.181591328245275,0.143830368286339,-1.262538
15803184,0.206755204744758,0.339746182543081,-0.196038091866747
"Ran",6794.55715338106,-0.540299371687428,0.283521315032238,-1.9056746

1083479,0.0566924353066263,0.128054256000972,-1.11047796312422
"B3gnt4",2.99088790164142,-0.517516073411167,1.20407459783469,-0.42980
3995817058,0.667338225972701,0.774740144686855,-0.132084386947114
"Nipsnap2",3060.81592708558,-0.132213086996422,0.285491504667791,-0.46
3106904530382,0.643287741168927,0.756356251194976,-0.0369195502373607
"Diablo",2517.06112011484,0.172437125918022,0.136220715678303,1.265865
65823987,0.205561183240739,0.338459039928047,0.18681013948275
"Vps33a",1526.70883284692,-0.0796890369944424,0.176025287344945,-0.452
713574262095,0.65075500374729,0.762051070880455,-0.0216548346542627
"Mmp17",210.440836243054,-1.6781506554472,0.344769469236382,-4.8674572
5821975,1.13043269761115e-06,3.03597475730333e-05,-17.4567824968
"Il31",0.0721532113593249,-0.544048378937457,4.78471990596623,-0.11370
5376621746,0.90947135024012,NA,NA
"Bcl7a",5111.54105001347,0.849463544228457,0.261742500089839,3.2454169
4198265,0.00117278771083339,0.00663966747556022,4.25979922515557
"Sfswap",4256.9493070113,0.353959248773741,0.208345530433333,1.6989049
2989002,0.0893370979906852,0.181070138942035,0.604870629861902
"Psmd9",1468.80407996749,0.132660564200462,0.174852821811752,0.7586984
46075327,0.448032966677682,0.588756190336407,0.0702760210922897
"Wdr66",611.249450204857,-0.272242394244251,0.416789661838879,-0.65318
8932381661,0.513634484450681,0.647463094731714,-0.118341994733867
"Hpd",636.54373132374,2.33187928808496,0.81794610693653,2.850896004406
2,0.00435962256484004,0.0178917955734058,9.38211348656518
"Psph",823.668688706351,-0.647282381117307,0.390070830503542,-1.659397
03894734,0.0970358125767082,0.192786251642618,-1.06553891397138
"Cct6a",6949.43930851285,0.145188722633003,0.173148316620007,0.8385222
88100758,0.401737435423215,0.545744897643826,0.0879268178755804
"Rhof",263.047720446643,-0.186314288643717,0.36407540440449,-0.5117464
30518885,0.608828486282786,0.728814945084833,-0.0589378100204948
"Tmem116",1449.15514284986,1.98859131366051,0.562589328953184,3.534712
10938306,0.000408219654383814,0.00294250159747712,11.5904948785707
"Mapkapk5",1966.40420184515,0.487022995854731,0.204768763611734,2.3784
0472963046,0.0173877294261391,0.0516335589934299,1.44333329018835
"Aldh2",26952.3028345915,0.0311411226807299,0.508690223756029,0.061218
2448697213,0.951185399678966,0.969888762835502,0.000952105307581141
"Acad10",2104.84369261833,1.24516555094435,0.308368617123986,4.0379126
8565993,5.39289172654861e-05,0.000612765751073688,9.21114681649124
"Brap",4349.5909889892,0.996577331167983,0.158330576779673,6.294282200
17654,3.08825551995204e-10,6.23655985775684e-08,16.5334690799433
"Fam168a",2046.04939829152,-0.276159270793154,0.135790689736652,-2.033
71285121777,0.0419805556723829,0.101618482247067,-0.631446415608856
"Vps29",4084.81180827448,0.478451431698333,0.145443055729899,3.2896134
4560073,0.0010032510478521,0.00588666534186588,2.45687948700096
"Fam216a",583.090672638003,0.0443228450013631,0.131683861699981,0.3365
85246127922,0.736429579780128,0.827704291313188,0.00838142004880477
"Gpn3",727.646480425923,0.332768543423945,0.149350454575415,2.22810532
696379,0.0258734921538578,0.0703155716575755,0.883421284456578
"Arpc3",6861.78134931427,0.0126176423680766,0.154615222031208,0.081606
7279942838,0.93495945097726,0.959465230271295,0.000522107966407172
"Anapc7",2649.54250884626,0.340262571332105,0.144515899842579,2.354499

20529681,0.0185476833633398,0.0542192998828184,0.991769559636491
"Atp2a2",11524.3690470365,0.14012898205182,0.190447091317347,0.7357895
62773207,0.461858773999338,0.601156366851741,0.0713116670616652
"P2rx7",934.606583980529,-1.15915353358517,0.337077005087631,-3.438838
94804339,0.000584214679372359,0.0038863929779605l,-6.43361949521796
"Ift81",992.275569356167,0.27341428525425,0.163043644751233,1.67693923
716816,0.0935543644257572,0.187454834813834,0.457754938355481
"P2rx4",3290.9531844457,-0.0687216025988742,0.21295136964606,-0.322710
310401358,0.746914643022943,0.835437225198562,-0.0123561488328315
"Camkk2",1889.43296542743,-0.0459833900780516,0.219385981490576,-0.209
600402749648,0.833979565275415,0.89375155694601,-0.00516519662508551
"Anapc5",12504.1107346268,0.0564352150438855,0.158487932926809,0.35608
5248899975,0.72177672385739,0.816263706384281,0.0114573535753249
"Rnf34",1053.09516702742,-0.0524135376463546,0.157775502536486,-0.3322
03268591928,0.739735775806624,0.830394570563789,-0.00974128160820097
"Kdm2b",1122.89764820388,-0.314694145172799,0.191930380412613,-1.63962
653799919,0.101082841768357,0.199041935851286,-0.507991796196546
"Morn3",2.43892572707901,-0.0560338731215999,1.30347934949712,-0.04298
79254651926,0.965711159039829,0.979271696430132,-0.00117369394011747
"Ncor2",4250.3243989688,-0.256210570773381,0.194223478257722,-1.319153
44669817,0.187117818139081,0.315913884789154,-0.295227756144253
"Dhx37",908.028283177413,-0.0150587443552449,0.226075689799327,-0.0666
093040282731,0.946892738654668,0.967309892988783,-0.000500497945221077
"Aacs",6270.70972551785,-0.711839096017175,0.627096642076993,-1.135134
60008255,0.256318926350001,0.397171877473188,-0.657302363179324
"Anxa3",3445.77758233646,-0.706199846611935,0.267056372080864,-2.64438
49330736,0.00818395270913951,0.0289664321535938,-2.50108982803519
"Mrpl1",1110.4357472273,0.232712487201769,0.26135531407255,0.890406564
0585,0.37324761904765,0.518462851644006,0.152865784238517
"Mfsd7a",539.976804648968,-0.0368336926674267,0.190729038088055,-0.193
120528665495,0.846864575730043,0.902205523854463,-0.00379066328584409
"Pde6b",0.216813168483108,-1.39575995508774,4.76764712241052,-0.292756
556693745,0.769708236753709,NA,NA
"Pxmp2",1264.94258450799,1.09947059731587,0.43733528298153,2.514022170
40492,0.0119362929968192,0.038847244341843,3.57121038647645
"Pgam5",1159.42684484452,0.0647194136265362,0.128915197953123,0.502030
906007452,0.615645781074949,0.734751291440922,0.01994802580718
"Ankle2",1634.97522010872,-0.212923611821379,0.110495142473887,-1.9269
9522399094,0.0539802251424393,0.123215456547329,-0.445823882295232
"Golga3",1792.70030250659,-0.299699499958479,0.136891228553826,-2.1893
2581089836,0.0285731676449324,0.0758398615220381,-0.772964345041059
"P2rx2",23.1811315186316,-1.05300791228865,0.640902557066387,-1.643007
81870586,0.100381315574707,0.197920405609832,-1.70575732502914
"Ddx51",809.421767270801,0.235575360152459,0.359973744057376,0.6544237
29623211,0.512838850427334,0.646943486948844,0.102592205865763
"Ep400",3588.07231098105,-0.188543427271349,0.189220385302234,-0.99642
2383191939,0.319044961984563,0.464095692985303,-0.144738098301489
"Pus1",1513.63209589999,0.48286929696709,0.129792031526732,3.720330834
54462,0.000198961974516003,0.00169109005192373,3.08185628847124
"Gpc2",472.575427527466,-1.813849635698,0.37066243387343,-4.8935351142

6349,9.9040633472922e-07,2.75589540336503e-05,-19.0439393773481
"Ulk1",3285.94594458902,0.0436713987550525,0.171938330272846,0.2539945
49590839,0.799499768854713,0.872022897947082,0.0059803437155608
"Prkab1",2765.22921087215,0.117218070987776,0.111012558541073,1.055899
19310262,0.291014272824811,0.43463778564061,0.0976710517247835
"Cit",633.638588386009,0.620001977671019,0.339562908652416,1.825882515
05899,0.0678679604869945,0.146576151260015,1.19053411023126
"Ankrd7",1.65554876459847,1.83288850772328,1.04742374201719,1.74990162
452628,0.080135290333108,0.166504464078745,3.28588000388077
"Rab35",2162.82050898608,-0.307883166515095,0.0840186942557909,-3.6644
6026378081,0.000247860715356454,0.00199619505494429,-1.91395951714278
"Chek2",473.83388258369,0.0395361302689877,0.346886413270248,0.1139742
83098215,0.909258179692371,0.944413356295671,0.00226112392236418
"Pla2g1b",2.79157189861427,1.46428452233493,0.963416134945508,1.519887
89602091,0.128539153050061,0.238259949250889,2.10035943605997
"Sirt4",1004.05220445622,0.501956426528109,0.151847075618602,3.3056706
8534717,0.000947493754099166,0.00563410589125512,2.59959057757539
"1700123K08Rik",2.16716047944131,-0.88091641117776,1.54991804409012,-0
.568363220582351,0.569788359011512,0.696797128951736,-0.31824072052584
9
"Pxn",6561.31802704316,0.142011250848758,0.152888202141307,0.928856830
414579,0.352963287840164,0.497943577621555,0.0990199727606391
"Ccr9",62.8054892639893,-1.08156168628628,0.508057668246531,-2.1288167
7392863,0.0332694225787447,0.0851295539947983,-2.66451484035501
"St7",447.275234073525,0.395510748774624,0.196029457166945,2.017608753
75885,0.0436320244784626,0.104873916824589,0.891875331457264
"Triap1",863.110872583618,-0.49960269345938,0.277076085018742,-1.80312
455846049,0.0713686550613489,0.152310882701077,-0.940168118984107
"Gatc",760.631040028373,-0.0283751516995175,0.21039380320236,-0.134866
860466541,0.892717141631633,0.933808949974977,-0.00194322720509746
"Srsf9",2523.03622667659,-0.0885733609786631,0.142100182332065,-0.6233
16307727752,0.533076683375721,0.665254043296199,-0.0361012877077689
"Cyp2w1",0.271943130842482,-0.383314022047795,4.77572921836925,-0.0802
629304386491,0.936028140285264,NA,NA
"Cabp1",290.45636022906,-0.616307621926421,0.391623135421474,-1.573726
28474346,0.115550742417959,0.219943244534619,-0.933327477365286
"Acads",7260.2925301835,0.367666870761453,0.481385444775897,0.76376815
0349073,0.44500541999126,0.58620535095352,0.196365405725425
"Uncx",54.077896322939,2.4854239204898,0.760660356332257,3.26745557304
18,0.00108518897173506,0.00626484147062252,12.6080634220953
"Ints1",2260.74913435861,0.0745695081168955,0.152449114268705,0.489143
597026482,0.624740037863445,0.74207622651608,0.0222443310916808
"Sppl3",2623.3444889396,-0.153871379231415,0.0814532477346478,-1.88907
604682242,0.0588816395341215,0.131629695899599,-0.31201463306504
"Psmg3",825.212111669885,0.178559690391364,0.222587506661883,0.8021999
66517447,0.42243729575449,0.565628512060229,0.101746481980149
"Tes",1670.98954947081,-0.456257638629873,0.243010110215531,-1.8775253
3516078,0.0604461283098431,0.13430085620139,-0.916016050953234
"Tfec",864.867214940178,1.28441777925634,0.584481774951513,2.197532642
25029,0.0279824278537891,0.0745507491397438,3.33470201259246

"Mad1l1",587.044639895942,-0.121403371552588,0.159539702574492,-0.760960247471334,0.446680811411578,0.587796616110073,-0.0645106293773993
"Hnf1a",1129.49299462617,1.95647782366576,0.677635376278645,2.8872132302332,0.0038867072899792,0.0164418056423236,8.03707015672239
"Mrm2",503.470793013698,0.235362027137087,0.232239066290757,1.0134471813731,0.310846612873073,0.456117343302165,0.184760408305265
"2210016L21Rik",2151.96294678333,0.288948654435133,0.146985331063605,1.96583327291412,0.0493178729686174,0.115161663130791,0.624538930095691
"Snx8",3253.34089476832,0.874056369708088,0.215840201125798,4.04955316548591,5.13155193048448e-05,0.000589271394818223,6.50002832131675
"Oasl2",1982.08635044951,-0.880974368886049,0.330767482471329,-2.66342496034934,0.00773496615768575,0.0277644274931069,-3.1574118422867
"Foxp2",220.521842286281,-0.853093119020919,0.361235656471934,-2.36159721150672,0.0181964005088567,0.0534338828499583,-2.49897439551546
"4930519G04Rik",5.82908390087451,0.114656859825191,0.579566055281242,0.197832255323429,0.843176308794124,0.899603374493452,0.0121308457177104
"Tmem168",1853.72887177966,-0.213387487465201,0.109649038597837,-1.94609538025999,0.0516432834297925,0.119202342055509,-0.453860862507939
"Lfng",1062.4357219392,-1.15323119884164,0.236241128809039,-4.8815852034547,1.05236435401677e-06,2.91463070662202e-05,-12.0434036902087
"Tmem106b",9006.08278568588,0.695957816996153,0.198528213199799,3.50558646440715,0.000455602491343019,0.00321019062813076,3.99578958014241
"Mmab",973.51297506044,-0.771563290166715,0.355979171629164,-2.16743942246846,0.0302013640394029,0.0790636675671695,-1.95784326270424
"Radil",94.2724372069822,-2.31171242437703,0.565490126755356,-4.08798016977072,4.35145339215055e-05,0.000520261834844208,-17.4792699220735
"Ube3b",3899.83658800676,0.234978163355352,0.129900355875749,1.80891085148458,0.0704648542857913,0.150864579868712,0.444431275421525
"Wipi2",5218.1447861357,0.346263644168126,0.165686103026877,2.08987741181864,0.0366288131546166,0.0916566477013238,0.827468229712045
"Actb",84657.3317931993,-0.802846350083558,0.16187725523867,-4.95959947492221,7.0638669137308e-07,2.07426622114342e-05,-8.65734749985132
"Fscn1",1716.88005790248,-1.55563235645861,0.287203171583839,-5.41648738723801,6.07812761484703e-08,3.14653666582599e-06,-19.7086301773147
"Spdye4b",0.0874363708740191,-0.193076388122813,4.78471990596623,-0.0403527044251972,0.967811935912226,NA,NA
"Zfp12",1237.23256941014,-0.0618756849498317,0.12068401794831,-0.512708194521114,0.608155456683543,0.728311736237813,-0.0196162079088397
"Ung",203.370767009376,-0.412184473532799,0.211601824035269,-1.94792495486286,0.0514239433279446,0.118827215810413,-0.877987885568523
"Usp30",1067.48187313348,0.0766714261845962,0.1337168614973,0.573386372713693,0.566383115090435,0.693801532472899,0.0280287223327343
"Rbm19",584.134473916894,0.164403045125159,0.13154863002559,1.24975110035869,0.211390484062039,0.345325899063672,0.174804278799998
"Lhx5",9.7527434573823,0.471980540538192,0.76488887009406,0.617057665488257,0.537196690781037,0.668683674466845,0.18994581408842
"Sdsl",453.233580967578,0.454161534752911,0.300115595921311,1.51328868251149,0.130206346199391,0.240348809412589,0.647481767636112
"Sds",1.88040416398935,3.09514310619918,1.69239302129627,1.82885598513

548,0.0674211854268801,0.145788873622414,5.95999448807921
"Plbd2",2758.77553453224,0.177063364848487,0.111475113025796,1.5883667
6673755,0.112203427813352,0.215096100236431,0.272088026884306
"Ddx54",2790.61110185223,-0.199190286167104,0.0930300199515989,-2.1411
3988442373,0.0322627591467468,0.0831319251808206,-0.495451271953914
"Rita1",339.966800324721,0.346363938921454,0.213383798329991,1.6231969
8886329,0.104547301341971,0.203968709382205,0.550645470048638
"Iqcd",135.148318794958,1.32981808262805,0.322768448195062,4.120037414
01758,3.78810881775849e-05,0.000466880820029425,10.1989553894448
"Rasal1",231.9614642791,-1.46943264841107,0.802917961346458,-1.8301155
5246179,0.0672326618147097,0.145461877503685,-2.83283285457928
"Dtx1",59.6748737317514,-0.76173404860045,0.451629755410002,-1.6866338
8422874,0.0916737723270204,0.184749892799198,-1.28638012610673
"Oas1b",476.015947364021,-0.451077777971304,0.295338017660379,-1.52732
71675102,0.126679677166672,0.235709547227385,-0.651877289992801
"Ankrd61",11.4766137515406,0.305233085816811,0.462115537277152,0.66051
2493510358,0.508925003737846,0.643288397429534,0.13465728034451
"Rph3a",12.2032993197869,2.20275352830613,1.11281503124935,1.979442644
50948,0.0477661912146964,0.112426991345262,4.81401041625345
"Aimp2",940.870817593205,0.139882717177575,0.124446562115874,1.1240384
2098368,0.260996726960462,0.402476604102743,0.127309822193035
"Eif2ak1",2527.2679675342,0.292877418128002,0.136365347641653,2.147740
78014041,0.0317343538246463,0.0820523048498811,0.73231021920275
"Rpl6",29209.388644183,0.380318221756555,0.163007532026815,2.333132813
11437,0.0196411757556137,0.0566514267578337,1.09183204355356
"Erp29",6542.51015228025,0.326467276475958,0.186936781071513,1.7464047
1824038,0.0807406506693975,0.167485977892604,0.583349895975228
"Ccz1",2316.60720775328,0.278745513430152,0.134431610878244,2.07351166
596236,0.0381246865603007,0.0945143214943094,0.657561754894995
"Ocm",0.102112886877019,0,4.78471990595847,0,1,NA,NA
"Arpc1a",4447.91708464104,-0.342775080932335,0.211334814052313,-1.6219
5274105423,0.104813467458839,0.204304007648951,-0.544376928821421
"Arpc1b",8975.92732249058,-1.13517705125141,0.173756505100226,-6.53314
850339914,6.44012288984377e-11,1.72013035761882e-08,-20.2950139286532
"Pdap1",5967.92197745464,0.438183009529084,0.19417150840429,2.25668025
721225,0.024028061364427,0.0662774115457924,1.18918756022759
"Ptcd1",868.903130549355,0.285841324875807,0.117090332505635,2.4412034
6026048,0.0146384049538569,0.0454345144524518,0.883673663018645
"Cpsf4",1133.83047510113,-0.0456198927754782,0.0954611709437133,-0.477
889515962224,0.632728843751422,0.747965744844503,-0.0132479300801319
"Zkscan14",442.493304921066,-0.00579003378932632,0.162477786692828,-0.
0356358484884624,0.971572723505215,0.982603261106138,-0.00010161416890
6889
"Phf14",2627.18086079841,0.501678139595202,0.21630879229592,2.31926836
755154,0.0203804894775632,0.0583008779098485,1.42583856815193
"Cyp3a25",2.51471282539855,0.2421943768084,1.69883891558115,0.14256465
0825384,0.886634016798741,0.929540276349169,0.017695966426719
"Ndufa4",17313.6115421483,0.634358521690886,0.506804603186033,1.251682
63607509,0.210685529699786,0.344495679070232,0.676019212740827
"Gm5578",1.07420713757917,-1.73976542959392,2.26860747493495,-0.766886

933423248,0.443148743156701,NA,NA
"Rnf6",2703.37485138332,0.316599977214468,0.110436942680216,2.86679411
37344,0.0041465267028325,0.0172349131638943,1.28565492343597
"Cdk8",2654.33252204256,0.321318571798302,0.119722033270037,2.68387165
688672,0.00727750374865891,0.0265624799871493,1.16582590292365
"Wasf3",719.977835591412,0.415694552111467,0.317984790978994,1.3072780
9601097,0.191118280539894,0.320966772951494,0.472402635283462
"Glcci1",993.514589917135,0.0657601100223127,0.32624778729916,0.201564
922682564,0.840256869969325,0.89775756004222,0.00709257145010143
"Usp12",2631.14881015545,-0.152081694193595,0.0905656476921378,-1.6792
4260543656,0.0931047716304051,0.186841921725811,-0.255115879800256
"Rasl11a",1127.56240164417,-0.764872698363881,0.354749115881723,-2.156
09472757332,0.0310762691237004,0.0807900127165214,-1.92434469502609
"Polr1d",4606.29164588495,-0.0659600410236408,0.276911167723915,-0.238
199280895034,0.811726534833266,0.880013565536782,-0.00843087763754369
"Pan3",4211.44055473411,0.300914454984385,0.18821226110027,1.598803676
36661,0.109864231927888,0.211589568760367,0.467352309481171
"Flt1",4459.94550671542,-0.0444400775739699,0.225385940828386,-0.19717
3246080187,0.843691966158434,0.899907328017349,-0.00468680566922663
"Pomp",5355.38156683065,-0.270579756438054,0.249429198779277,-1.084795
83690398,0.278012089788975,0.421645902824007,-0.233669812224525
"Slc46a3",967.505287727224,0.731760397702608,0.188355840196188,3.88498
916168683,0.000102334453568371,0.00100773594731116,5.04918267408278
"Mtus2",79.3903090996999,-1.83775200091776,0.544879882959811,-3.372765
37158063,0.000744173367409369,0.00468363212322397,-9.85711617983914
"N4bp2l2",2355.70976386028,0.203615205083697,0.0891030476338267,2.2851
654403614,0.0223031288074526,0.0626599269455372,0.564020869828776
"C8b",1.10340272805288,2.24102943654224,1.68361791144718,1.33107958837
045,0.183162824453591,NA,NA
"Hsph1",5763.26801540009,0.495392360530872,0.22989034483984,2.15490720
532871,0.0311690964370578,0.0809665657247206,1.24527717839745
"Wdr95",11.913501485657,-0.910422053577806,0.619190173553272,-1.470343
1877048,0.141468829799158,0.256281635549938,-1.23951987010165
"Medag",271.444514638645,-1.7421810529605,0.315266051526639,-5.5260661
4167365,3.27490710949564e-08,1.95871591475294e-06,-22.8978713762694
"Tex26",4.62759011658255,-0.654453442502916,0.796557584793554,-0.82160
2172895677,0.411303352218245,0.554835530756285,-0.385527757306403
"Col1a2",23632.679289204,-1.3887775374315,0.36455287409227,-3.80953665
744519,0.000139227463868081,0.00128717737814123,-9.24273605685086
"Gngt1",0.495202687383534,1.69934662421739,3.06405797020011,0.55460655
1424484,0.57916380690145,NA,NA
"Tfpi2",491.424847124761,0.628690976249618,0.352523961277989,1.7833992
7297553,0.0745213315127877,0.157474468606951,1.16213020105279
"Tspan12",2122.02457080916,-1.13674132406132,0.466530730072535,-2.4365
8402498928,0.0148267243513222,0.0458220750359931,-3.50456135747245
"Ing3",1075.04294909498,0.0137354419541789,0.125947580836744,0.1090568
14453492,0.913157427592863,0.946557337756813,0.000754401725229286
"Wnt16",106.993532530799,-0.354160721076405,0.451806911014414,-0.78387
6280867925,0.433112708720868,0.57522182376129,-0.195850713052133
"Fam3c",2120.67103658179,-0.996414691036515,0.209769639762056,-4.75004

243782256,2.03373964548435e-06,4.81871058438007e-05,-9.90477961265902
"Auts2",1302.06061316833,0.392328368986079,0.191382316335162,2.0499718
9133716,0.0403671738472377,0.0985820585763329,0.908972257225242
"Limk1",521.195226838786,-0.815937170425557,0.225383468742927,-3.62021
746748524,0.000294355513564586,0.00227571126228481,-4.96535516123876
"Eln",3921.35541978277,-1.51949866411997,0.353530626298373,-4.29806797
795658,1.72293327791855e-05,0.000255271679732948,-12.5710892541747
"Pot1a",592.356414588263,-0.142235898789289,0.144844271277732,-0.98199
1883659368,0.326103846754642,0.470990578075704,-0.107091853127072
"Hyal5",0.508774917275483,-2.44477546303939,4.54667309538006,-0.537706
452993851,0.590779723525297,NA,NA
"Hyal6",1.19635814697524,-0.655602605503425,2.35886035026529,-0.277931
928199858,0.781064613938983,NA,NA
"Hyal4",0.353421772999755,1.62409248149331,4.72772323675802,0.34352528
6942772,0.731203320726435,NA,NA
"Bcl7b",1729.25462157853,0.190790477361157,0.230045095757149,0.8293612
02998949,0.406900048169303,0.550800967731538,0.113783959758916
"Lmod2",0.63666969227087,-0.0721943441908471,3.11301838608549,-0.02319
11075480762,0.981497831850048,NA,NA
"Wasl",5007.87221955907,0.365726557824347,0.0967422319601533,3.7804229
9018887,0.000156562141295828,0.00140610254766746,2.40170200361749
"Asb15",251.122395179223,0.0370113300905363,0.488500087260021,0.075765
2476545737,0.939605864978601,0.962511041050371,0.00141419336143104
"Cul1",3223.19090917486,0.0779768773488573,0.128861403732375,0.6051220
54318167,0.545097901366811,0.675028191860914,0.030644976993493
"Ezh2",983.620931160761,-0.335389095627635,0.239433841886652,-1.400758
94445368,0.16128616952691,0.283005555564767,-0.423357882471483
"Aass",10680.7306096457,2.23932424726376,0.823986513405589,2.717670994
40558,0.00657431748192938,0.0245426081299366,8.30194657643279
"Fezf1",1.2047805377649,-2.66181918488046,1.84498237360348,-1.44273420
871854,0.14909535875036,NA,NA
"Ssc4d",144.901041352007,-0.127297895001395,0.325989570688065,-0.39049
6833173859,0.696169196576586,0.797419964553361,-0.0288169091509785
"Slc13a1",8009.47278092766,1.76073131449792,0.665604057880173,2.645313
37159441,0.00816152943354246,0.0289123366452055,6.239128349829
"Rbm28",3287.63666414289,-0.0723798114724828,0.152033053157024,-0.4760
79444367448,0.634017780747162,0.748944546771366,-0.0209243039139018
"Lrwd1",971.743373510739,-0.125428061676697,0.177310674896231,-0.70739
1485313008,0.479323226810262,0.616724579407845,-0.0606234889029658
"Cux1",6985.83027809665,0.631235304303598,0.122436681468607,5.15560612
009441,2.52811815493607e-07,9.28309751128879e-06,7.31432248892536
"Fscn3",0.783867247402907,0.180646067215266,2.37259971254653,0.0761384
511091328,0.939308949348298,NA,NA
"Gcc1",1046.38505268503,-0.198433136585648,0.132711873276886,-1.495217
6597767,0.1348576461009,0.247013093816378,-0.277471819528986
"Ephb4",2647.02658948201,-0.28337737227821,0.0954273198905784,-2.96956
23077662,0.00298224326670108,0.013481979878075,-1.22033668693544
"Epo",0.946300746917918,-0.0749428933948144,1.94877603825847,-0.038456
3910493211,0.969323800645105,NA,NA
"Actl6b",0.284694131055269,-2.13860194741628,4.74578785920475,-0.45063

1593923511,0.652255092040634,NA,NA
"Gnb2",14705.6235841494,0.0191746966815296,0.140897981133709,0.1360892
22338347,0.891750747588622,0.932998286997368,0.00132980191815117
"Gigyf1",5837.66173980481,0.0353217069101849,0.155171054945584,0.22763
0771232248,0.819933295895312,0.884533551143454,0.00433379095797526
"Pop7",477.790613865538,-0.0705961292158516,0.242788887804023,-0.29077
1665270101,0.771225957291646,0.852168407303214,-0.0112933411898643
"Tfr2",116.298487318087,-2.4472351389775,0.844164304105265,-2.89900334
221232,0.00374350903806475,0.0159822507607153,-10.1224411952211
"Pcolce",6326.49084710153,0.0944313677102036,0.249592960832702,0.37834
1470028474,0.705176942292021,0.804213269037301,0.0205757248372757
"Gm20605",5081.63924165977,-0.139767517998137,0.143326000723794,-0.975
172106193666,0.329474896006128,0.474278443877998,-0.104261074922641
"Agfg2",1565.3099798709,0.0785010472366743,0.204907702024664,0.3831044
24387256,0.70164233925788,0.801536344671323,0.017366391203266
"Tsc22d4",3143.42433432684,-0.090671172640707,0.128992701830519,-0.702
917074795737,0.482107429551916,0.61965264742276,-0.0433948790409548
"Ppp1r35",1387.03081236632,0.321666495202098,0.207118496398338,1.55305
538035318,0.120409904622355,0.227077311183545,0.476859237685734
"Mepce",3251.74984082493,0.290285631700835,0.184193702905364,1.5759802
1605537,0.115030362684982,0.219222169057939,0.440557676461701
"Cyp3a13",61.9987575279216,0.102196848130209,0.448085568437314,0.22807
4402142917,0.819588399808713,0.884451454032746,0.0125485110336655
"Zkscan1",2368.73845231241,0.367135575490377,0.201197085044119,1.82475
593724368,0.0680378678672468,0.146861767704592,0.704262770477817
"Mcm7",1618.97442702059,-0.2377505321033,0.151684534912969,-1.56740126
631705,0.117020928168219,0.22212120902214,-0.357606008827669
"Tpk1",2558.51995857858,1.55594647679005,0.367952565907623,4.228660487
66916,2.35086792312173e-05,0.000326475945619853,12.4898224290306
"Nobox",4.50377752722681,0.851833083716501,0.745586266219257,1.1425010
3886168,0.25324581446939,0.393637347777755,0.794185476258827
"Asns",650.702425333868,-2.00458358308838,0.492240490612541,-4.0723662
9516985,4.65379156527628e-05,0.000547944613162784,-15.0530923610752
"Dlx6",22.8702879307437,0.777036675257768,0.595887550417466,1.30399884
12468,0.192233977457933,0.322174496516656,0.88011988791934
"Dlx5",3.19219405491388,-3.96664502335026,1.69672932357074,-2.33781839
462909,0.0193966709695371,0.0561122092824976,-11.4255316868089
"Dync1i1",19.0886599590407,-0.492258236416652,0.591884261937526,-0.831
679887559859,0.405589656056653,0.549446752851568,-0.294785601350552
"Pon3",762.501427469386,-2.58134268297766,0.595073491099982,-4.3378552
7600314,1.43879841350679e-05,0.000224664873216776,-21.6856036000355
"Cald1",12978.4344451868,-0.34810341416285,0.306429279573677,-1.135999
1925287,0.255956902242915,0.396775932575319,-0.321781072954562
"Akr1b8",1081.33877570829,-1.63653060908222,0.397494071611166,-4.11711
953954144,3.83637112523381e-05,0.000471636596467899,-12.5346818262481
"Exoc4",2019.10186856783,0.0413474515463412,0.117050897381504,0.353243
353714561,0.723906008592643,0.817960092462874,0.00830842845679148
"Plxna4",542.377611440068,-0.305816862934498,0.220597642965745,-1.3863
1065510517,0.165652064684697,0.288459762188285,-0.380191424684751
"1700012A03Rik",0.121527413527064,0,4.78471990595379,0,1,NA,NA

"Calu",6353.79323970805,-0.838435757502815,0.219403782073311,-3.821428
00629873,0.000132681149222843,0.0012389928388635,-5.61203323410161
"Ccdc136",313.067475602891,0.283873188534077,0.398445742630192,0.71245
1303056204,0.476185356298767,0.613780794414845,0.138563450362449
"Irf5",1582.49433339151,-0.809102233790468,0.313347401694617,-2.582125
23676519,0.00981939446587209,0.0332913499035948,-2.75293610796669
"Ahcyl2",7015.40105848257,0.438644948924161,0.222738025498003,1.969331
22641915,0.0489150675874106,0.114440363309255,0.950851285736308
"Klhdc10",3964.32276723431,0.395739260384946,0.113732068174836,3.47957
499354177,0.00050220978946832,0.00347360286450986,2.24089846745915
"Hibadh",14471.9978404184,1.02598202968508,0.435276871558004,2.3570791
2991734,0.0184193214055209,0.0539168186105461,2.99618847042761
"Gars",3159.81045563577,-0.242672984638391,0.236179229829668,-1.027495
02914971,0.304187424907963,0.448947574813069,-0.194344455402115
"Adcyap1r1",278.55197478619,-1.12435900757051,0.416186886092751,-2.701
57240687286,0.00690124518867657,0.0254956481392192,-4.1255514805417
"Nt5c3",1711.18068890016,0.558139545335645,0.161470888415632,3.4565954
9416099,0.00054704534162586,0.00370872394680842,3.12394466008582
"Fkbp9",4930.26328914173,-0.836956038787247,0.229785045578441,-3.64234
337652551,0.000270167359577378,0.00213209003980534,-5.14782578668564
"Tmem209",885.565243081351,-0.321876091181202,0.119459545055281,-2.694
43593672026,0.00705078793391711,0.0259047038129858,-1.17591980948846
"Ssmem1",2.0378436794154,0.562854435963412,1.11212101018367,0.50610898
5271716,0.612780143156761,0.732107060739616,0.175514064545534
"Avl9",1399.96141889361,-0.0778420576213038,0.145241626751365,-0.53594
8676439429,0.591994021762807,0.714972279742851,-0.0261169060317113
"Cep41",192.565720490451,-0.539206720813021,0.176230698953427,-3.05966
397463768,0.00221585450923012,0.0108255402708516,-2.44036717196209
"Sspo",168.451884251199,-0.838032260201062,0.507865971489263,-1.650105
16011463,0.0989214295508535,0.195675158099071,-1.3670814824545
"Herc6",954.076531871662,-0.279528923221517,0.251190764651871,-1.11281
528844789,0.265787767898303,0.407616733903639,-0.250857061196677
"Abcg2",9452.5451263965,1.62051937665169,0.388730559057253,4.168747063
7291,3.06278645175562e-05,0.00039707977804615,12.6908922517031
"Herc3",1904.56139696309,0.481033848615056,0.266177486057185,1.8071920
947954,0.0707323330869478,0.151337543128422,0.908308581891205
"Tmem176b",42290.4177287123,0.522473118828766,0.403695042081225,1.2942
2723681517,0.19558694139513,0.326271903883953,0.585182528712552
"Aoc1",2636.13122307128,-1.10907315911051,0.580802696773165,-1.9095523
5792175,0.0561908742669646,0.127145139428719,-2.28738133578728
"Doxl1",1.67672214375798,-3.53489658088358,1.55878618773858,-2.2677238
2812286,0.0233460445924272,0.0648896903012324,-9.66817730076099
"Igf2bp3",35.167403325455,-0.545543907852373,0.580109312276893,-0.9404
15704949034,0.347004369566909,0.491776018385059,-0.387189921526206
"Malsu1",866.185937560595,0.607422363115232,0.230297281423738,2.637557
6791877,0.00835054231473367,0.0294156892672905,2.14190919700929
"Gpnmb",1010.52292024703,-1.28959921250829,0.350345352520994,-3.680937
11884194,0.000232378313325613,0.00190374468622807,-8.07796234824089
"Tra2a",6038.07047701294,-0.08999630570692,0.129688129022822,-0.693944
051664765,0.48771729620289,0.624475429561593,-0.0423741570599328

"Npy",110.464872407165,-0.00379275874996845,0.653620681217194,-0.00580
269085565874,0.995370148537298,0.996955214269288,-1.15657542926226e-05
"Gsdme",237.247533083253,0.476703198863051,0.20487535248066,2.32679623
532582,0.0199761128831752,0.057393110326336,1.3623371852393
"Osbpl3",6034.16929710835,0.37498909411703,0.153839409905636,2.4375359
6264472,0.014787743221755,0.0457378517350538,1.15677725410305
"Luc7l2",11719.6771867622,0.0624437843126762,0.187105478961315,0.33373
5733765373,0.738578982392475,0.829396590375423,0.0116805371042695
"Zc3hav1",2177.93742101017,-0.737429178312016,0.163068682895179,-4.522
19988056221,6.12002258062737e-06,0.000115965209209983,-6.6827456934386
"4921507P07Rik",52.1533745498433,-0.381173491568015,0.32363544664419,-
1.17778659760679,0.238881693027585,0.3769595103672,-0.371879527712353
"Tmem213",2553.86704697717,1.54487870546415,0.576760648801338,2.678543
8789467,0.00739430431895848,0.026853212403408,5.58839760872158
"Svopl",608.988789341264,1.42412770710883,0.320833914705316,4.43883156
310607,9.04485645646091e-06,0.000158125452649099,12.4641392024741
"Npvf",0.997636755383997,-2.31108202400547,2.41833073485488,-0.9556517
60404953,0.339248201725003,NA,NA
"Nfe2l3",337.545521431581,-2.09895404703211,0.508099482228895,-4.13099
032855646,3.61203779974476e-05,0.000451747998841715,-16.1669543888582
"Trim24",2821.23363666658,0.291652657835993,0.142120909038797,2.052144
6127,0.0401556103775339,0.0981546976999415,0.676987210832335
"Cbx3",5304.64555474768,-0.0756757119032507,0.0756398885015983,-1.0004
7360463324,0.317081365224788,0.462288796503808,-0.0583887671709505
"Ptn",1150.42312882122,-1.07890159903354,0.4988331622869,-2.1628505893
3795,0.0305526731474216,0.0797584711512152,-2.72827492336251
"Mtpn",5316.19367530862,-0.385381937782751,0.233684481360371,-1.649155
0296335,0.0991158782383717,0.195980265073702,-0.628072865373529
"Slc13a4",156.016995107464,1.23671071556502,0.49008865347357,2.5234428
6446885,0.0116211943651381,0.0381079797732025,4.04074397123602
"Hoxa1",70.2267233731325,-0.0216021720950445,0.214702692901275,-0.1006
14350957291,0.919856604103296,0.950822460202834,-0.0010893526353743
"Slc23a4",23.6154465064892,-0.220268823039505,0.493434632899973,-0.446
399195259075,0.655308914019674,0.7652858891684,-0.0589231885233691
"Stra8",2.72929365161118,-0.948572467330841,1.26074335295185,-0.752391
408695426,0.451815711621875,0.5924346163553,-0.496591691566107
"Tcaf2",360.895173364027,-1.14032636824328,0.19991652209584,-5.7040126
3631731,1.17019396288858e-08,9.05691370597466e-07,-15.8671479258877
"Epha1",814.249940264688,0.303727735939718,0.245779438280268,1.2357735
783958,0.216542738781886,0.351868486146964,0.317242949587851
"Zyx",3501.3725442276,-0.773993196050023,0.160021697943054,-4.83680154
628443,1.31945043721908e-06,3.42109635969382e-05,-7.95894447483264
"Fam131b",11.6327580295314,-2.10778063793342,0.681391631933751,-3.0933
4682017103,0.00197912694367738,0.00991750633457249,-9.72414852376583
"Clcn1",8.38408723292289,-0.47111709729679,0.540534937333999,-0.871575
664693223,0.383439911287651,0.528164999763265,-0.300735971134088
"Casp2",1147.71367221296,-0.34573998779505,0.196041288383406,-1.763608
01668918,0.077797997074899,0.162793018562608,-0.627613401770017
"Gstk1",1630.78327118361,0.624160633765615,0.394733520501662,1.5812202
4441293,0.113827688071814,0.217589437453355,0.951935660960868

"Kel",14.3125908166587,-1.0818102552216,0.831790168483602,-1.300580718
80531,0.193402010364199,0.323647020064466,-1.22039209759223
"Llcfc1",20.1118298841326,2.04276139682464,0.458535625869101,4.4549676
8752226,8.39058309076259e-06,0.00014837254732704,18.0085437188137
"Trpv6",427.847292228935,-0.243649039851006,0.464060517825957,-0.52503
7210647566,0.599557323593371,0.721348185574697,-0.0795838991656948
"Ephb6",496.805711951049,-0.322759019661044,0.385268332963098,-0.83775
1229587715,0.402170434800478,0.546021988714117,-0.195300201985168
"Ccdc184",59.3461190917117,-2.99256674909105,0.537698760325088,-5.5655
0799425644,2.61389798915676e-08,1.68760870836352e-06,-39.7777897466714
"2210010C04Rik",0.877516630454103,2.41604698609079,3.43160425221797,0.
70405758022045,0.481396915867123,NA,NA
"Mad2l1",467.427939489217,-0.353144734319126,0.181259395383336,-1.9482
8374867012,0.0513810206096336,0.118770599889683,-0.752396531910409
"Ssbp1",2411.50601660748,0.408224538653278,0.14013379753729,2.91310551
649504,0.00357853659726644,0.0154607371091294,1.70207243890198
"Prdm5",377.92427358382,0.441248448968957,0.312276327384949,1.41300639
937725,0.157653842504761,0.278410888611343,0.564205523866767
"Clec5a",179.243808653602,-0.863236622839158,0.491244187512717,-1.7572
454693254,0.0788759780837559,0.164501424823732,-1.55800057422526
"Agk",855.539106245667,-0.1050783470003,0.106510635874652,-0.986552621
129424,0.323862006606726,0.468759661852717,-0.0796141952325651
"Qrfprl",0.221314475300897,1.56465187791422,4.7308669841675,0.33073258
7314448,0.740846485107455,NA,NA
"Mrps33",2470.67013219115,0.542933329282497,0.267114955008688,2.032583
04749294,0.0420946656527989,0.101844123451827,1.24022903026137
"Hpgds",857.447516882047,-0.990260496000811,0.434981729003794,-2.27655
65309346,0.0228127211777513,0.063707023420767,-2.72664312549158
"Smarcad1",1216.55300987562,0.148779901322121,0.120738359634248,1.2322
5047758491,0.217855532648947,0.353188694939162,0.154843101607111
"Mkrn1",4442.61681274356,0.460047062228473,0.173208350552255,2.6560328
1112986,0.00790659210847648,0.0282452449308553,1.64090973500666
"Rab19",230.767376480502,-0.22493993732908,0.193378599395236,-1.163210
08649637,0.244744275164555,0.383880932922411,-0.215362634749652
"Slc37a3",1723.66651253173,0.234447619062486,0.113470866351057,2.06614
81365371,0.0388144942135226,0.0958082443354487,0.549874979019417
"Tbxas1",689.411922646351,-0.311576423170247,0.364626759965965,-0.8545
07834804363,0.392823670183439,0.537342214202097,-0.193526384429168
"Gfpt1",2227.64674708839,-0.588674225011138,0.171673321615757,-3.42903
731034413,0.000605726252433193,0.00399215263776492,-3.25149774605629
"Nfu1",1873.56651991622,0.00354654470266274,0.241822377151158,0.014665
9078636295,0.988298718016177,0.992279228099683,2.74883152682519e-05
"Anxa4",5021.63960332026,-0.158261079598282,0.116883773208161,-1.35400
385574849,0.175735147594522,0.302101846822939,-0.189437100166921
"Pcyox1",4679.73922114298,-0.515145131647161,0.300120804208339,-1.7164
5925381953,0.086078019499767,0.175798598953227,-0.895536674821505
"Tgfa",500.911141289832,-1.33725150455646,0.276631071554678,-4.8340611
0904698,1.3377549133986e-06,3.44293125023463e-05,-13.742401818931
"Add2",62.8056379590614,-1.50401793057699,0.286890078278853,-5.2424884
8060582,1.58425285489685e-07,6.47584112469157e-06,-17.969152035654

"Dusp11",8155.73747932398,0.539164595734936,0.189366795822027,2.847197
12024731,0.00441060422798581,0.0180376590288519,2.16490411736301
"Nat8",4495.69550245511,2.31384603403284,0.802706830661765,2.882554309
55442,0.00394465129845898,0.0166369913513635,9.47780644599083
"Cct7",7126.92892531395,0.0321054504832112,0.134847803089163,0.2380865
66838488,0.811813953318285,0.880059405192923,0.00410198074489882
"Pradc1",368.582422662147,0.370702650739018,0.168536317731998,2.199541
65207584,0.0278394308390782,0.0742562547880091,0.963913363978376
"Zfp638",3133.02458951561,0.297112720253826,0.162533190807403,1.828012
5971679,0.0675476610785744,0.1460137800122,0.571661000477088
"Reg3g",49.6492572990801,-3.06170328260984,1.00341892410467,-3.0512712
1789309,0.00227874634240316,0.0110469905977565,-13.7948018359604
"Fbxl14",1674.78187352123,0.0868760595280814,0.120560741598707,0.72059
9909855007,0.471155709330174,0.609093179960848,0.0430717618707842
"Prickle2",321.185940157078,-0.937831466650905,0.134625719981369,-6.96
621319299677,3.25585643031981e-12,1.67059707048357e-09,-18.95365341958
66
"Adamts9",1331.44232239915,-0.261303916367595,0.242274522363267,-1.078
54475913813,0.280790718394901,0.424637976209088,-0.223811584596742
"Lrig1",1062.2571922926,-0.211309772235443,0.272794068712029,-0.774612
781110388,0.438568502617395,0.580648378083383,-0.114870085542625
"1700003E16Rik",71.9004131480886,0.608443455333832,0.219026881586399,2
.77793963429015,0.00547047802485031,0.0212623484391234,2.3430046747539
6
"Kbtbd8",143.951112740514,0.405957675503725,0.229867676117614,1.766049
41747448,0.0773875585265771,0.162038511184622,0.738810999697573
"Wdr54",156.287816808545,0.0453697643653657,0.236431513953343,0.191893
896066321,0.847825318825956,0.902791705035749,0.00463966736491159
"Ino80b",1176.48793758561,0.342925188214094,0.114988589623082,2.982254
0596259,0.00286134442024372,0.0130382083444525,1.48825112367594
"Wbp1",3141.96316002408,0.179945253368473,0.146023061518012,1.23230708
559193,0.217834393988943,0.353188694939162,0.187278529582604
"Mogs",1662.32585829021,-0.305299501435292,0.125524284082522,-2.432194
7236487,0.0150076366062761,0.0462054158454398,-0.938691634372702
"Mrpl53",1704.89056431752,0.0261814142868006,0.286536860942357,0.09137
18891199395,0.927197097525462,0.955393000028413,0.00119472351304783
"M1ap",12.1658432086878,-2.13936497960064,0.883878914660284,-2.4204276
6731561,0.0155022630916468,0.0473025235932598,-6.52761252351801
"Pole4",1802.02790359453,0.071775883072946,0.193584581228463,0.3707727
26926213,0.710806816519932,0.807944418794408,0.0153070691975836
"Tacr1",10.7890624287993,-2.27968216480962,0.895704536736601,-2.545127
40676227,0.0109237873039367,0.0363095814613293,-7.55868201684186
"Mrpl19",1365.58497076283,0.470367754468441,0.161368724928247,2.914863
17858427,0.00355844509265847,0.0154012347206781,1.96298932268794
"Bmp10",1.51654787900059,-4.43626249632237,1.80656687249243,-2.4556314
874754,0.0140637292810528,0.0441072210908745,-13.8461598012569
"Arhgap25",670.799839499429,-1.22009762352315,0.254338066065288,-4.797
14909529098,1.60939809405213e-06,3.98247320908244e-05,-12.360836389911
5
"Gkn3",18.4228405517841,-0.205437532877511,0.54366336803585,-0.3778763

56870828,0.70552244697253,0.804366546080598,-0.0447237941746507
"Gkn1",0.0910336030428901,0,4.78471990596623,0,1,NA,NA
"Aplf",373.32362984466,-0.450256952601134,0.173575721089741,-2.5940088
2666272,0.00948640514427342,0.0324739565506512,-1.54317323948514
"Gp9",6.29570554397786,0.265618672998817,0.60869326038704,0.4363752488
89601,0.662564493844256,0.771126514712308,0.0690350440602944
"Rab43",2951.59647478464,-0.16573417253517,0.251062157414345,-0.660132
033604919,0.509169103425173,0.643449295390759,-0.0730741937827836
"Isy1",1708.45325649918,-0.921964668257914,0.157966361364876,-5.836462
01819086,5.33209103484368e-09,4.9983226441049e-07,-13.3767791632313
"Cnbp",14080.181400974,-0.173243232011321,0.145971431173119,-1.1868297
1468477,0.235294795467692,0.37320246609375,-0.170754454506856
"Copg1",5676.2060857048,-0.101178078967099,0.0852134845757118,-1.18734
821690342,0.235090296515394,0.373036775227565,-0.099769505180581
"Tmf1",2480.58121330425,0.421288707923573,0.106235733998004,3.96560264
67656,7.32107375047995e-05,0.000776698269304374,3.01662035628794
"Hmces",1229.9836476763,0.264308030469824,0.13570796143064,1.947623615
32423,0.0514600159429148,0.118882392281393,0.562875819751618
"Uba3",2277.28882795894,-0.100047833124355,0.160289174203918,-0.624170
868814105,0.532515376043582,0.664724895777591,-0.0408577356356672
"Rpn1",10040.3734939207,0.168955123315305,0.115364533373982,1.46453262
86511,0.143048474662302,0.258590687832891,0.228513295012123
"Frmd4b",2515.16295956815,0.326899447592975,0.195230644299754,1.674426
92598534,0.0940467246571952,0.188325258022593,0.545786321101762
"Foxp1",3455.66490485151,0.257061575161477,0.28255736861995,0.90976772
7583964,0.362945017646395,0.507872969288748,0.17416536689177
"Gm20696",19.7809907888114,-0.23854796317112,0.628379899335754,-0.3796
23796724375,0.70422469371741,0.80365361648451,-0.052143466427108
"Prok2",4.10349549887496,-0.956511807454034,2.12590181672831,-0.449932
259301642,0.652759286016712,0.763578694975694,-0.258008622876467
"Gxylt2",1374.11640670903,-1.02567001322782,0.452641941641749,-2.26596
326780429,0.0234536323373603,0.0650774047692971,-2.80231291716387
"Cntn3",21.6172632546281,0.307209102873207,0.599700582146604,0.5122708
09832407,0.608461491538007,0.728552688340024,0.0972916886871777
"Chl1",77.1543648696666,-1.43289249206756,0.389692503378564,-3.6769824
4037193,0.000236009279822228,0.00192519681799471,-8.95948567835756
"Ruvbl1",1034.1190386212,-0.354012392274048,0.139504596614608,-2.53763
962525217,0.0111602839794104,0.0369382476711158,-1.16771261685742
"Sec61a1",6484.41308205509,-0.178919335661792,0.100101866791526,-1.787
37261747988,0.0738772842820179,0.156571661840303,-0.331759652089652
"Abtb1",2355.23010162549,0.310048492726545,0.140036764682572,2.2140506
7040321,0.0268252934863364,0.0723029544369073,0.814463374064458
"Plxna1",2303.75821476129,-0.822659914176267,0.14206132916033,-5.79087
862290672,7.00191652606518e-09,6.01526044014989e-07,-11.7836127913483
"Chchd6",974.081520939509,-0.0292339228823427,0.406157239969773,-0.071
9768602044823,0.942620322959827,0.964484207444937,-0.001057151874597
"Klf15",2664.77954226608,1.64759139873091,0.402087055957361,4.09759870
237064,4.17458182475691e-05,0.000503999977827308,12.5100532996398
"Aldh1l1",13399.5455002929,1.25649788900713,0.372712922215114,3.371221
69400376,0.000748356089991437,0.00470168049780502,6.73462171656583

"Slc41a3",342.695708759958,-0.550591935372894,0.273531781120646,-2.012
8993169172,0.0441252224587481,0.105720519476612,-1.23715600445652
"Nup210",1533.76065015392,-0.158092592595337,0.344156147605462,-0.4593
62977227923,0.645973530045444,0.758518030040715,-0.0436950076999227
"Cntn6",17.1634858526048,-1.15244833405237,0.501221577618676,-2.299279
17215317,0.0214890917463776,0.0608296037849695,-3.22648505848122
"Wnt7a",58.0304124179455,-0.911564009042265,0.847082124751951,-1.07612
235272843,0.281872540032285,0.425778647780406,-0.778325869967329
"Xpc",1692.28056854163,0.169050925190358,0.0995178529688339,1.69869948
101975,0.0893758212865996,0.181076217700908,0.288880517111563
"Tmem43",2460.99606170488,-1.00046436938078,0.214826040195672,-4.65709
077200099,3.20709065263683e-06,6.99461449050479e-05,-9.57222795690453
"Slc6a6",12784.6265324621,0.616470979460321,0.194026237045035,3.177255
76112282,0.00148675858399866,0.00798590051537354,2.97760274772476
"Grip2",422.294369451132,-0.0366751900000739,0.361660960672825,-0.1014
07655202387,0.919226859830043,0.950561510035433,-0.00185952036622751
"Sumf1",2522.02295201765,0.0773790001809531,0.240597121121699,0.321612
327779322,0.747746403440426,0.835944176232897,0.013865809455847
"Itpr1",8441.55117351603,0.570185352055292,0.17373361459705,3.28195181
673825,0.00103091219347429,0.00601818142166518,2.9153406877195
"Bhlhe40",3910.68210987825,0.131301393516903,0.246866373882865,0.53187
233016678,0.5948144225709,0.717545728595398,0.0435813747332488
"Edem1",3737.39456540132,0.285423984129226,0.153893141467064,1.8546894
384524,0.0636405878251242,0.139516998135178,0.56216218382039 7
"Arl8b",3987.08093167853,-0.0415717584993565,0.174876745278321,-0.2377
20335160595,0.812098010553112,0.880220524196152,-0.00530384165068137
"Usp18",511.082613434479,-0.994348808326776,0.210704501744145,-4.71916
261919356,2.36817454282981e-06,5.43231379248184e-05,-9.76506244034419
"Slc6a13",3139.17700700762,1.91960956969578,0.563717797544758,3.405266
92266332,0.000660994113763219,0.00427606610157772,10.4709362195169
"Slc6a12",67.3718505501826,0.57140571655077,0.679852569996737,0.840484
748852994,0.400636649178034,0.544780904224375,0.347055590450739
"Ret",67.3885648365398,-1.45699172215106,0.30110130512526,-4.838875479
28409,1.30575819669716e-06,3.39010297193093e-05,-14.9954537548367
"A2m",23.5512537025639,1.50455734543776,0.693891202119446,2.1682899867
3306,0.0301366298144975,0.0789578081326353,3.81983284356201
"Klrg1",46.6429809421954,-1.71608231260179,0.450771190088064,-3.806991
99579842,0.000140667296201519,0.00129720639021244,-11.4077296901714
"Mfap5",691.132599852387,-1.89433022767858,0.417870174178475,-4.533298
48535565,5.80696649477466e-06,0.000111004149720702,-17.2496630242854
"Gdf3",21.5038236545251,-2.39030655493426,0.653105518096669,-3.6599086
8045378,0.000252305148093168,0.00202363051317178,-14.8267419298182
"Mlf2",7373.78585069024,-0.297518579159026,0.304094883973241,-0.978374
168192869,0.327889299431954,0.472624052659439,-0.222976793049935
"Ptms",36077.6099360388,0.464453540850832,0.166051970795242,2.79703720
845053,0.00515735934913397,0.0203517896355827,1.8088544567177
"Plxnd1",4919.4666752035,-0.708844819527664,0.157673055365095,-4.49566
235579264,6.93537153541043e-06,0.000127091987027662,-6.35876293145098
"Lag3",343.251011170904,-1.59857168589473,0.365847549683673,-4.3695022
3467924,1.24530065851101e-05,0.000201163430006141,-13.6060716905464

"Lrrc23",66.2639391779123,-0.582152542039764,0.374778258856736,-1.5533
25275099,0.12034544489754,0.226977702129255,-0.863276039115491
"Tmcc1",2377.56561533265,-0.053518827974516,0.269254966106646,-0.19876
6354241795,0.842445516232372,0.89912212547838,-0.0056909991759223
"Cops7a",2845.23945350239,-0.0890710612140592,0.208753730857015,-0.426
680092606672,0.669612352284794,0.776320632881409,-0.0225518714760875
"Mug2",2.48558288568834,1.0244533884309,1.31517895416598,0.77894600212
8324,0.436011523895009,0.578165988364042,0.56129214594866
"Rasgef1a",155.892498115369,-0.358031488664253,0.2990110703791,-1.1973
8539516388,0.231156373531959,0.368075702011118,-0.35784053256343
"Tuba8",191.558213924835,-3.00819201144246,0.776372009882398,-3.874678
5988564,0.000106765516928146,0.00104242065551577,-20.6548772195825
"Bms1",2167.85240881204,0.0735559332112662,0.112762750583361,0.6523070
14778688,0.514203135684992,0.647901830040152,0.0319244585991426
"Clec4e",27.0288013168851,-1.65904790222453,1.07302326778568,-1.546143
45469711,0.122069926090625,0.229608278884335,-2.4410908270093
"Clec4d",137.159343425927,-1.06895301260579,0.42518908024451,-2.514065
06486732,0.0119348412845603,0.038847244341843,-3.47208566613113
"Zfp248",170.523657159584,-0.543146141970482,0.215029814802696,-2.5259
1084854375,0.0115398761265152,0.0379165835689419,-1.77737334898348
"Clec4b1",164.89248293536,-0.924109324571218,0.355323121135953,-2.6007
5764734049,0.00930181410733218,0.0320040182574136,-3.18068616725926
"Clec4a2",426.855900510388,-1.18798674709918,0.41533159835496,-2.86033
316945916,0.00423196150731687,0.0174967738485293,-4.8062840368988
"Klrk1",328.073603630314,-1.17641551716852,0.304580129078648,-3.862417
16006612,0.000112270641183528,0.0010869180545166,-8.02834071429418
"Klrb1f",21.7180461783906,-1.04836141067486,0.511903822522772,-2.04796
558366825,0.040563371791876,0.0989744241268925,-2.424748611497
"Clec2e",21.0210238211081,-0.379387457998929,0.513885838663328,-0.7382
71867903106,0.460349258091281,0.59977775838176,-0.193941387051031
"Cd69",124.102394075871,-1.02242933662896,0.504918435405158,-2.0249396
0397492,0.0428735662827006,0.103382240338869,-2.32022147758051
"Clec2d",5277.14314181273,-0.17714140934545,0.302046758168229,-0.58647
015587828,0.557559611226611,0.686666075018725,-0.0665887254322714
"Clec12b",40.3324103190192,0.288885849402551,0.353131358919606,0.81806
908988878,0.413317737561734,0.556859400703336,0.169126051969039
"Clec1b",24.8498025859579,-0.563711421128334,0.398391752388307,-1.4149
67598473,0.157077996345929,0.277746147887271,-0.722141230521474
"Tmem52b",4992.16504007704,1.73317980089802,0.635944427591863,2.725363
6099322,0.00642307077017791,0.0240886774142968,6.45785110333884
"Gabarapl1",20604.5388603581,1.08891674756313,0.31242154847311,3.48540
858620337,0.000491386088983015,0.00341205340562352,6.18552733537598
"Olr1",49.8888609081409,-2.00814128624407,0.582186650679983,-3.4493083
6854229,0.000562024484391396,0.0037787425505736,-11.2021430245058
"Klrd1",193.536343956013,-1.15528523390131,0.347503380606553,-3.324529
4819429,0.000885678686671503,0.00536803327097326,-6.03901520328492
"Rad52",796.968539121582,-0.0664596877715088,0.201241475037486,-0.3302
48462744218,0.741212231031303,0.831491633430087,-0.0122640748424554
"Klrc1",47.0486901405719,-0.894027197997019,0.365964568364912,-2.44293
375719795,0.0145684110581115,0.0452984237485801,-2.76655200412503

"Adipor2",7503.57168781239,-1.84529380244352,0.599244365862552,-3.0793
677964538,0.00207440414245816,0.0102656680126013,-8.44950834069276
"Wnt5b",237.684547597,0.613057554164368,0.370812872924283,1.6532801284
1008,0.098273865309422,0.194695293446214,1.00315805518268
"Erc1",1182.14638964028,0.341598165200791,0.24189185031338,1.412193774
85532,0.157892912967558,0.278732097513939,0.436393084756307
"Klra5",6.22928389308405,-0.128707093000626,0.762901296418752,-0.16870
7398460074,0.866026799072267,0.915814650629735,-0.0113186666653266
"Ccdc77",570.331270002793,0.0165383515882789,0.185913056914386,0.08895
74506641287,0.929115725452378,0.956286867339024,0.000739220332325367
"Klra13-
ps",27.5984818733762,-1.01271058621237,0.51414169600349 7,-1.9697110623
0894,0.0488714944351831,0.114405914262483,-2.19555908600061
"Kdm5a",5371.18955729097,0.752953877215732,0.299845268844233,2.5111414
3010502,0.0120341472756818,0.0390548940714453,2.44166910653911
"Klra2",328.462418289488,-1.27443184423843,0.510974845994655,-2.494118
55442246,0.0126270362184815,0.0404258080326176,-4.08874297205392
"Magohb",345.537494933776,-0.23508885728956,0.142590355490877,-1.64870
096915217,0.0992089115200353,0.196104557666023,-0.382984974619278
"Ybx3",5544.1614776562,-0.735346939636095,0.18659019552498,-3.94097309
114855,8.11517303722029e-05,0.000840752624228062,-5.20714837174132
"Etv6",2691.81036245704,0.170288658510257,0.151759213117558,1.12209766
387195,0.261820917066163,0.403364747231041,0.154607465003326
"Bcl2l14",279.867454853694,-1.21467248935109,0.327046622146652,-3.7140
6523442523,0.000203956181570125,0.00172153144080273,-7.73083287431165
"Lrp6",5306.6200420905,0.548791284088662,0.113585111075738,4.831542434
48979,1.35479334415942e-06,3.47643328492193e-05,5.63439545030337
"Dusp16",1489.34051734622,-0.579125597157941,0.195693774517911,-2.9593
4604248197,0.00308292703543084,0.0137932334412186,-2.48072916767855
"Ddx47",3042.387951992,0.0371336977416399,0.0938024696039718,0.3958712
16380721,0.692200036096131,0.794426764600763,0.00854574404299641
"Gprc5d",3.6996650270617,1.11992722535885,0.790334071130459,1.41703017
276853,0.156474106055481,0.277004913734317,1.43767301563497
"Gsg1",5.04070170269502,-0.951601902896544,0.738954127721476,-1.287768
57344414,0.197826534944993,0.328861947170047,-1.05829287206846
"Fam234b",1430.79671932382,0.45258757165844,0.246832903433911,1.833578
77074772,0.066716545895942,0.144634112950753,0.875099832274915
"Emp1",3773.84310978949,-1.32139714046824,0.225429107236251,-5.8616970
8370185,4.58160259363445e-09,4.53462372440023e-07,-19.3007938034633
"Grin2b",10.9761660903484,-2.1742325695723,1.62604627310855,-1.3371283
4962302,0.181180744776237,0.309015234573747,-2.5533419803224
"Atf7ip",2610.07767872549,-0.452067545273205,0.174064592048112,-2.5971
2523928043,0.0094007640985057,0.0322704398578634,-1.55222077008834
"Plbd1",2289.39311144113,-1.71623226020896,0.294279714056449,-5.831976
10379542,5.47747648803591e-09,5.06767814185337e-07,-24.8771519948959
"Wbp11",2102.31367351266,-0.0989804792122371,0.102702052256161,-0.9637
63401391028,0.335164559472308,0.479788011737684,-0.0726923442901549
"Art4",1221.01394569502,-1.27418393066015,0.406644190172579,-3.1334123
5766675,0.00172786510670386,0.00895561588021094,-6.00838179616308
"Mgp",29215.5137789105,-1.5063901427201,0.425913687937663,-3.536843697

16846,0.000404939237364446,0.00292064084352835,-8.79122089732162
"Erp27",17.0269267557212,-0.580619553691017,0.671308112411933,-0.86490
7697308942,0.387089452471049,0.531413598981819,-0.367076190896233
"Arhgdib",1607.62770091916,-1.56165616583056,0.42904030416086,-3.63988
219914428,0.000272762798642563,0.00214275948748295,-9.59740908217536
"Rerg",508.892974913567,-1.02109116468682,0.468259047282276,-2.1806117
1612834,0.0292121478154636,0.0770637881350167,-2.61718100637096
"Ptpro",1618.56518737906,-0.516690159905696,0.45023348287385,-1.147604
91958006,0.251131697962456,0.3910995085361,-0.485065236445492
"Strap",4921.77181212282,0.0681913173461419,0.166013261429039,0.410758
253642825,0.68124980698669,0.785512048292518,0.016462712580869
"Dera",1847.29644671412,1.10820193590804,0.228204094786525,4.856187777
63264,1.19667227518971e-06,3.17020598120229e-05,11.4800067022926
"Lmo3",3.94396818468213,-4.3767451913116,1.97355753781114,-2.217693230
35082,0.0265757561485259,0.0718190011620177,-11.5266232620272
"Pik3c2g",111.986373056779,2.23069008070828,0.961026027517604,2.321154
70011806,0.0202784960578757,0.0580897180761463,6.34802332710986
"Plcz1",1.17475240967519,-1.70147846644247,1.7790389597161,-0.95640315
078541,0.338868593502183,NA,NA
"Plekha5",2034.36631948686,-0.116392837948057,0.188657645655926,-0.616
95266864686,0.537265945495854,0.668727188335663,-0.046834044478366
"Aebp2",3440.23185932002,-0.141643262506846,0.121305577496694,-1.16765
663566217,0.24294529521441,0.381765583985378,-0.1363951690396
"Slco1c1",9.6037448082628,-0.436555989181716,0.53596893804621,-0.81451
7331495203,0.415348647569008,0.558684432397304,-0.254150012772299
"Slco1b2",11.4013691499961,0.952392552207329,0.650836896164272,1.46333
521934648,0.143375675131227,0.258990079091037,1.28664950209317
"Slco1a4",130.876518020304,-0.0732140620233716,0.371560905384487,-0.19
7044578593678,0.843792652900935,0.899917269871446,-0.00772060164726149
"Recql",791.390781490926,-0.185466882449726,0.100356853404948,-1.84807
39098241,0.0645916514937922,0.141105959511171,-0.363189444054882
"Gys2",624.416285536203,0.0838486711834584,0.750856884231111,0.1116706
43160342,0.911084557067771,0.945280083387515,0.00471850103315697
"Golt1b",1133.34843362395,-0.562481655497198,0.157422511025038,-3.5730
7002559332,0.000352820361636464,0.00262225187696904,-3.34323449775527
"Ldhb",43878.9137991235,0.834842427384018,0.244900035902488,3.40891100
447379,0.000652227535004217,0.00423752590333112,4.56139176257133
"Kcnj8",446.408099028814,-0.571705502243779,0.358549063925484,-1.59449
72662447,0.110824702552622,0.213060545293929,-0.883958986560139
"Abcc9",811.123484546399,-0.608127614455424,0.432718876713745,-1.40536
419181388,0.15991300904358,0.281203449705242,-0.771517427108838
"Rad18",134.822993552507,-0.881264389620498,0.249729327074993,-3.52887
824566899,0.000417325086026498,0.00299705507452842,-5.12025636244038
"Sspn",730.559610062723,-1.68640040396564,0.388831332410471,-4.3371000
8273043,1.44374895346937e-05,0.000225021719382461,-14.1646058983792
"Bhlhe41",533.260530556119,-0.056396243233358,0.186040383648179,-0.303
139792164742,0.76178333020091,0.845664505366508,-0.00945384684130622
"Srgap3",928.347918536354,0.193743155640416,0.22484351607111,0.8616799
76482586,0.388863647249434,0.533047201495323,0.121892595891629
"Rassf8",1755.95636027763,-0.0400073321833684,0.158911538652515,-0.251

758509939612,0.801227728766539,0.873097762001341,-0.00542930485196975
"Lrmp",681.953423019776,-1.10967367811261,0.403021986422624,-2.7533824
8407361,0.00589829415431207,0.0225323157172368,-4.20877559296635
"Thumpd3",1286.07843663246,-0.0237640780519637,0.129894486726426,-0.18
2949089302103,0.854837965150243,0.907625280140461,-0.00230330172145195
"Kras",2805.01447369012,0.0195263481751562,0.157911663609165,0.1236536
15754973,0.901589538967684,0.939775885413669,0.00121285669903715
"Bcat1",1382.52853681,1.62705235410015,0.468302053477471,3.47436519233
291,0.000512063594253015,0.0035279915055637,9.18800783950145
"Mtmr14",1281.73674088459,0.311260014503806,0.123232116717308,2.525802
71113763,0.0115434285680066,0.0379167402660027,1.01855562060762
"Cpne9",6.83655541309183,-0.0917132843686834,1.11530693594936,-0.08223
14301225212,0.934462680449475,0.959053301443094,-0.00383440635134532
"Ogg1",531.443803281239,-0.121605790138836,0.156334320503851,-0.777857
285251963,0.436653152200487,0.578583972925933,-0.0665392331265513
"Camk1",3609.91771718411,-0.236215269099492,0.155338534061744,-1.52064
824434098,0.128348133110062,0.237967754985774,-0.33911538771286
"Etnk1",8039.62807844335,0.103398129172345,0.13905350260973,0.74358521
8867474,0.457127448242311,0.597110670987377,0.0533175352600304
"Ttll3",463.626499405508,-0.230794012898923,0.335295493119401,-0.68833
0197199328,0.491244869746501,0.627673163192482,-0.107489008560676
"Cidec",6712.01139129208,-3.6981749904684,1.60321982613458,-2.30671735
103528,0.0210705823273824,0.059905834677647,-10.4102937072042
"C2cd5",1849.97245954802,0.0903620372020309,0.153325807095841,0.589346
561505769,0.555628816349912,0.684937134803729,0.034195544798205
"Il17rc",1634.38075929461,-0.432778936086876,0.216830720901207,-1.9959
2997840956,0.0459415450665999,0.108868284602402,-0.959737720596907
"Cmas",2242.30925261271,-0.00378590815282132,0.148942064278502,-0.0254
186630966935,0.979721024918548,0.98690848025738,-4.9890580341842e-05
"St8sia1",2532.09150963493,1.89661359892379,0.651141256276749,2.912752
92517741,0.00358257940732467,0.0154713473497268,7.90653738322274
"Creld1",2421.79477379296,-0.409336666609007,0.282894348966498,-1.4469
5950309521,0.147908266094772,0.265090116032343,-0.543470336532708
"Emc3",3420.39810661107,-0.0912631023647639,0.204725066749766,-0.44578
3722597654,0.655753480838678,0.765529958651212,-0.0243843080147778
"Itpr2",4497.83572783605,0.65335092686598,0.183276499687606,3.56483743
403881,0.000364081779845184,0.00268998353293725,3.86667465762199
"Med21",2195.83856117141,0.885285418934596,0.166514948584299,5.3165522
1624993,1.0575188495925e-07,4.75103744916003e-06,10.8510740067693
"Smco2",0.504347048388131,2.53068533297104,4.66855537053199,0.54207032
6282253,0.587770059718515,NA,NA
"Sec13",2958.24428064941,-0.0655444467564621,0.146452511787231,-0.4475
47440167405,0.65447984291844,0.764684083007176,-0.0175850830775086
"Ccdc91",2002.26819530788,0.235685977845199,0.254073556514025,0.927628
916125278,0.353600092869331,0.498553374124391,0.164047958268441
"Atp2b2",165.463984826387,1.23101958922261,0.452067197376587,2.7230898
3347254,0.00646744690326407,0.0242271430777796,4.5797396003626
"Far2",5.63000469099731,-0.167517168129647,0.94380855450538,-0.1774906
22785717,0.859123030233129,0.910835780734357,-0.0156448740298328
"Ergic2",3414.44450737488,0.584846464412602,0.196164079132804,2.981414

67590841,0.00286919987947483,0.0130648433559085,2.53696475492834
"Tmtc1",830.550455759104,-0.533195470112506,0.179576496403142,-2.96918
294315925,0.00298592771045703,0.0134923797215507,-2.29574252820564
"Slc6a11",0.10500178835157,-0.544048378937457,4.78471990596623,-0.1137
05376621746,0.90947135024012,NA,NA
"Caprin2",917.828330145481,0.609726788011425,0.2772992809836,2.1988040
7135814,0.0278918568901906,0.0743655716730393,1.58453492326381
"Slc6a1",5.13015553761901,-3.28287956593514,1.32653652390151,-2.474775
10553556,0.0133320098091497,0.0422678906111872,-10.3861365864481
"Dennd5b",1863.99401829286,0.694833680796209,0.206606543305238,3.36307
684006731,0.000770789059197769,0.0048123103029901l,3.70803413300186
"Atg7",944.679508376997,-0.0651188799406058,0.16620532092703,-0.391797
805132816,0.695207616927646,0.796940332462534,-0.0147803882606171
"Vgll4",1621.91185698981,-0.937149991820619,0.223182938187692,-4.19902
166102186,2.68070678917901e-05,0.000357268769483338,-7.43818029331755
"Tamm41",501.133053883564,0.272990225250541,0.118190581256251,2.309746
02501248,0.0209022191005254,0.0594878803126616,0.770373687303298
"Timp4",284.839708180507,-3.63744468804624,1.17905477588685,-3.0850514
8567865,0.0020351691989794,0.0101228900616583,-16.7066236835405
"Cand2",343.258388133205,-0.382098611530815,0.252364452163069,-1.51407
461810001,0.130006915370875,0.240067699204596,-0.545191410798129
"Efcab12",138.085234484464,1.0412338812049,0.317335576144314,3.2811760
1517006,0.00103375211040349,0.00603119574607334,5.3215485203091
"Mbd4",514.725327252165,0.192062113317007,0.220005047338759,0.87298957
746762,0.382668758671775,0.5274477206689,0.122863195277923
"Ift122",1628.17375039422,0.924529625896094,0.21320032209956,4.3364363
4677231,1.44811336909459e-05,0.000225341695695181,7.76410055945464
"Rho",74.6814070649869,0.645596231974993,0.50111885597801,1.2883095981
5913,0.197638213319727,0.328660970857828,0.718373354729966
"Klrb1c",44.0329145577076,-0.605901708255266,0.304323040307081,-1.9909
8204212167,0.0464828615504152,0.109950604696105,-1.33766378067916
"Necap1",1464.80349283871,-0.106014738680503,0.230368920520959,-0.4601
95491825721,0.64537590657418,0.758044543758034,-0.0293674746107122
"Pianp",168.224874561783,-1.74093718644737,0.463505041073807,-3.756026
43374518,0.000172632467708311,0.00151076654191052,-11.3076274725789
"Ing4",3229.51706950209,-0.18570450602899,0.127960469077582,-1.4512646
551522,0.146706179295197,0.263444196343503,-0.247713786974393
"Mrpl51",4064.8137100276,0.807364690428326,0.234851672267824,3.4377642
8173614,0.000586538042247182,0.00389520393315244,4.47926676525311
"Cd27",169.66188215557,-1.40070623406219,0.494453196922788,-2.83283886
681173,0.00461366360757019,0.0186404832747525,-5.57819988095655
"Vamp1",1141.70241438675,0.234511894015861,0.183142900614024,1.2804858
5683427,0.200374315685786,0.331908793411048,0.258642010933739
"Ltbr",3513.88626297391,0.0269153593324206,0.0817424536139222,0.329270
264623381,0.741951417503761,0.831876263309317,0.00495435248186943
"Scnn1a",3050.57373883053,1.44953219867124,0.465609168203266,3.1131951
3802707,0.00185073605093137,0.0094071041504327,6.76393776925851
"Tnfrsf1a",4047.4092603625,-0.944335863918164,0.1392512704916,-6.78152
422297024,1.18914534313449e-11,4.65112049330873e-09,-18.1181767880808
"Cd9",7420.60628504957,-0.302998567449486,0.193269044779885,-1.5677552

9053073,0.116938252682283,0.222141665120729,-0.45584315555847
"Akap3",5.78316683032388,0.4201728649666,0.906808544206858,0.463353447
263893,0.643111041255922,0.756249869272539,0.117389341459397
"Dyrk4",7.48934578754865,-1.19780640267536,1.05902084234384,-1.1310508
2995757,0.258033697971083,0.398980257180029,-1.10059644058064
"Rad51ap1",109.20055290251,-0.922410280163671,0.433990418444528,-2.125
41623262029,0.0335518969831495,0.0856500245322661,-2.26681013556306
"D6Wsu163e",613.575195101533,0.249018737914906,0.13444914650802,1.8521
4071180475,0.0640056197246347,0.140033839025014,0.489538759713049
"Prmt8",1.64637905634584,-4.56346839511205,3.25508018236971,-1.4019526
8301803,0.160929376820654,0.282532053777157,-5.76805643584068
"Tspan11",100.447762846237,-2.08004680542584,0.520605005566653,-3.9954
4142523526,6.45738217775364e-05,0.000704961017367528,-15.0956652306876
"Tspan9",2901.14201133792,-0.360364495119884,0.198661987973118,-1.8139
5796345624,0.0696841977842766,0.149554484138451,-0.684726598829091
"Tead4",103.500093375509,-0.583857324180911,0.235554944614844,-2.47864
601244341,0.0131882119786999,0.0418732709918077,-1.85264210066839
"Fkbp4",13628.1697417388,0.384643956460344,0.143447787783645,2.6814213
1993338,0.0073310151602939,0.0266828698143383,1.39384719039163
"Pzp",1189.33011687131,2.43072944288988,1.11110228156532,2.18767388315
095,0.0286933657797569,0.0761070937251667,6.26062030354736
"Klrb1a",14.7449066057894,-1.87539506241766,0.662449472563192,-2.83100
091416974,0.00464025884189086,0.0187204152491595,-7.46058528772631
"Clec2j",0.121821870984685,0,4.78471990596557,0,1,NA,NA
"Clec2h",1403.88648856492,1.8338844844156,1.08287741767477,1.693529160
81069,NA,NA,NA
"Clec2i",184.966545946502,-1.21675184363411,0.43434209046202,-2.801367
56338723,0.00508865230156545,0.020154937716932,-4.75057149029959
"Ceacam12",9.20547217062443,-0.448244091352071,0.465974995246661,-0.96
1948808250528,0.336075319325516,0.480533833473155,-0.328499106930071
"Psg28",0.239436804505814,-1.10798154977452,4.77879592743038,-0.231853
70679143,0.816651641409327,NA,NA
"Strn4",3587.53028180748,-0.268548610130664,0.161074708005848,-1.66723
015335787,0.0954686490903504,0.19060492729507,-0.44513340306777
"Slc8a2",74.6875447886671,-1.40268756513385,0.329525635743315,-4.25668
722850587,2.07478292487631e-05,0.000295717232962268,-11.3983890374345
"Sult2a8",12.4183107983566,1.23658364285821,0.529843885328349,2.333864
13828648,0.0196028370732035,0.0565659489793285,3.55189865597217
"Mzf1",118.289422357575,0.64251433141217,0.222244651275457,2.891022698
29575,0.0038399038536355,0.0162726462373799,2.64604731943321
"Slc27a5",13.9945487672631,0.647702483151966,0.526571165934938,1.23003
788481649,0.218682918423004,0.354201656704747,0.672243202434733
"2900092C05Rik",0.0968692525312441,0,4.78471990596623,0,1,NA,NA
"Zfp606",811.887881809459,-0.0307433378244207,0.105159592225401,-0.292
34934420937,0.770019534597347,0.851190157182055,-0.00495335810272824
"Zik1",93.8673120538023,-0.481001269148072,0.221665615012509,-2.169940
83237009,0.0300113285215076,0.0787247253480902,-1.22260806427973
"Mark4",1694.29708773532,0.146599058083243,0.169443029222641,0.8651819
95127212,0.386938906002496,0.531277002412613,0.0927197601702603
"Ckm",8.39199190400363,-2.0468308627549,1.42897559074927,-1.4323763652

8254,0.152036138790989,0.270919451119238,-2.67302546455759
"Ercc2",1944.54351034706,0.115021158582028,0.169529230256548,0.6784739
03337888,0.497471264852943,0.633180672504908,0.0525646090818124
"Rtn2",274.70400246085,-0.831841611419482,0.249661990589003,-3.3318712
6104778,0.000862641518946296,0.00526355995442522,-4.36462941627381
"Ppm1n",4.17500077063101,-2.27479805297532,1.02560751829688,-2.2180005
629764,0.0265547940313157,0.0717922038300879,-5.99177118926175
"Vasp",3344.85067021427,-1.06484545188312,0.174079784077509,-6.1169966
2615047,9.53553845237047e-10,1.52072264455194e-07,-16.7169129183797
"Gipr",54.2057571408185,-4.2881962806102,1.06289456470745,-4.034451226
86321,5.4730056345331e-05,0.000619342216263067,-31.6762737788397
"Qpctl",717.741254382041,-0.0945401855346929,0.135783332735292,-0.6962
57660128274,0.486267483549855,0.623192020129819,-0.0447081094402891
"Dmpk",2467.56082547333,-1.04451813696234,0.237146676409785,-4.4045236
1709439,1.06016591311689e-05,0.000179281135940616,-9.01059356683652
"Dmwd",2597.58264767131,0.106411520934172,0.164369789054948,0.64739099
2870347,0.517378923873797,0.650871298644513,0.0456977204811424
"Nova2",384.825904423949,-0.734126391284747,0.322170570352543,-2.27868
855457968,0.0226855855956485,0.0634337513185078,-2.02454381247083
"Pglyrp1",126.46646276208,-1.57987023295516,0.484715779652405,-3.25937
44608193,0.00111658184532973,0.00640397398956772,-7.97966630967454
"Pdcd5",2402.28706770346,0.294419118327417,0.129053486525552,2.2813728
3427149,0.0225263941364315,0.0631153373864265,0.813418629959207
"Uri1",1675.05188734944,0.0938568100478772,0.129432600538128,0.7251404
17929168,0.468365890895072,0.606548760671633,0.0469256040560183
"Pop4",1198.27931773376,0.00875894804733542,0.13761640490928,0.0636475
57521282,0.949250863055475,0.96857466784529,0.000279670607340033
"Zfp939",108.922640184495,-0.114918754566954,0.223851514584861,-0.5133
70458002369,0.607692206415658,0.728035799735331,-0.03647579379013
"Lilra6",41.4997273592306,-0.829111051712515,0.50675899435244,-1.63610
525112039,0.101817561394001,0.200024061668537,-1.33430301726753
"Ttyh1",111.094107283192,-0.211021398167841,0.37273232551839,-0.566147
295849256,0.571293654245471,0.697652795512507,-0.0759748205879352
"Tmem238",1057.45055570882,0.807196823536377,0.243467606471721,3.31541
774790534,0.000915062655030506,0.00550166254942486,4.19960691000849
"Rpl28",17680.7085264747,0.367664996031702,0.209979941898563,1.7509529
3725395,0.0799540170419039,0.166201852090095,0.659794046933744
"Sbk2",9.52695414416286,-1.69077808347923,0.913657765684085,-1.8505595
2784825,0.0642329467115773,0.140436644425021,-3.31899538489352
"U2af2",8705.4738791232,-0.104292858095578,0.100462054581786,-1.038131
84520006,0.299208677377313,0.443580504220108,-0.0847771585514113
"Zfp583",113.157932070872,-0.993937865397282,0.245359517661788,-4.0509
4481302071,5.10112386954089e-05,0.000588068177193943,-7.39357346450286
"Zfp273",325.870788833083,0.253030285633963,0.220003305323799,1.150120
38233496,0.250094292528569,0.389808065496281,0.238380034710286
"Cyfip1",3027.50616249447,-0.591203953286961,0.1117929229714,-5.288384
43054406,1.23401416914861e-07,5.34684628223548e-06,-7.17662694733198
"Oca2",0.250033303070452,-0.994214896319586,4.78149651183207,-0.207929
650028887,0.8352838977209,NA,NA
"Herc2",3010.8276456365,-0.364103209499768,0.186416425143565,-1.953171

28959726,0.0507993018554224,0.117844710003215,-0.778593765794406
"Nipa2",1996.88712715869,0.00543856674021222,0.183587604821898,0.02962
38231632701,0.976367065512544,0.98508443949892,8.17303247014194e-05
"4933421I07Rik",0.146752147186871,1.29557472313525,4.78471990596623,0.
270773367845369,0.786565344309565,NA,NA
"Psd3",1066.41234887929,-0.791772437178952,0.130920378854834,-6.047740
19220398,1.46891579064844e-09,2.10595000632818e-07,-12.1721781510507
"Siglecg",361.405657279235,-1.46350815015395,0.508881319663661,-2.8759
3215471387,0.00402836287462493,0.0168805113538442,-5.97344827519697
"Zfp719",403.538766115788,-0.495765034390986,0.150315005021832,-3.2981
739535516,0.000973158255753855,0.00575027176367368,-2.55740797636101
"Csrp3",0.115675247240766,0,4.78471990596623,0,1,NA,NA
"Zdhhc13",716.306453648142,-0.403859146246378,0.126688576071191,-3.187
81028858858,0.00143354557050856,0.00776855795824788,-1.9618147508503
"Siglece",439.470710113159,-1.03927187337671,0.471332129577163,-2.2049
6717316736,0.0274563981452952,0.0735624528215277,-2.71210522252404
"Cyp2b10",12.6538942757505,-1.95945072253088,1.08662285610329,-1.80324
821213278,0.0713492418705852,0.152306494196701,-3.68741267966948
"Lypd5",0.252479961219923,-0.219867064805833,4.04080678974426,-0.05441
16747585817,0.956607177522949,NA,NA
"Zfp108",113.169080927892,-0.138451142777588,0.211603339798443,-0.6542
95640652298,0.51292135390582,0.646962911342578,-0.0602908082841481
"Tdrd12",12.7161455717797,1.6159921744279,0.638965954136217,2.52907398
89459,0.0114363915542347,0.0376539619257799,5.29935077080932
"Slc7a9",2567.23481092466,2.00009185613778,0.697555213463202,2.8672882
3401345,0.00414005767951291,0.01721536460549,8.12427912110039
"Faap24",234.188779504918,0.152898218204258,0.149964442284179,1.019563
1436052,0.307935692125232,0.452800160260767,0.121141930754128
"Rhpn2",1026.11154020514,-0.077983774431885,0.320195990856875,-0.24355
0127605261,0.807579257627188,0.87746895320228,-0.010193548104132
"Slc7a10",169.691266470343,-2.92765207766971,1.64812694169621,-1.77635
10829188,0.0756750684266637,0.159273800106119,-5.3784790554996
"Gas2",7582.66233308089,1.76300488044144,0.614731348933482,2.867927401
94581,0.00413170327658845,0.0171986715608152,7.16295331230539
"Kctd15",1483.4946557588,-0.813983851806052,0.217308546889823,-3.74575
166718476,0.000179854440246401,0.00155788621764741,-5.26193785850715
"Prmt3",846.624915082959,-0.0164288994739338,0.103708007769861,-0.1584
14955867162,0.874129829505395,0.921435011931459,-0.00134426231117726
"Asb7",801.526706177684,0.160940187430454,0.132623881473768,1.21350834
888878,0.224935524311319,0.360973191529306,0.163990259088305
"Cers3",277.75101879863,-2.40013270588459,0.760712037901402,-3.1551133
4946915,0.00160435761633003,0.00848204034794007,-11.4481631836152
"Snrpa1",1227.51928828447,0.0168105142724131,0.113080232564519,0.14866
0061013065,0.881821877659677,0.926338180626496,0.00128627171554427
"Pcsk6",1064.29701266342,-0.885098097665477,0.364211438197561,-2.43017
655361326,0.0150914693889833,0.0463610320066797,-2.71839824700725
"Tarsl2",363.722196766026,-0.1199065256662,0.24257175236783,-0.4943136
39142848,0.621084694176443,0.739220447262379,-0.0362308476033329
"Tjp1",4069.80121145954,-1.11008337100168,0.213431381116953,-5.2011253
696259,1.98085420997624e-07,7.64805849230034e-06,-13.0779573936213

"Fam189a1",11.5338741836733,-1.27860590828266,1.07742438016172,-1.1867
2449948714,0.235336308045067,0.37323231751938,-1.26013544612785
"Apba2",28.3424667730564,-1.62345001607607,0.507881803953131,-3.196511
47853663,0.00139100291824829,0.00758651046153988,-7.92468219952609
"Mphosph10",937.765402218239,0.0436816420614463,0.0961199770361684,0.4
54449152073867,0.649505581912884,0.761147830971077,0.011921929276845
"Mtmr10",1749.90580898643,0.757780713714523,0.175473239930467,4.318497
30485858,1.57095140162543e-05,0.00024000609213858,6.31598574080914
"Trpm1",37.6413081238996,0.388649010866866,0.479801780966259,0.8100199
42160651,0.417928714322354,0.561094406930885,0.224587089856768
"Chrna7",15.0588159657059,0.497752984440824,0.541542669958188,0.919138
993201136,0.358022875987907,0.503079420294913,0.341959898127047
"Crtc3",1515.20851726725,-0.301805462322424,0.188240922140408,-1.60329
358192003,0.108869852800594,0.210151904752598,-0.470793781326973
"Blm",137.900727571966,-0.943115487388312,0.39401812411876,-2.39358402
484057,0.0166846592307949,0.0500565449579996,-2.82425553738172
"Furin",7039.22916451439,0.443330465867511,0.160334851489636,2.7650286
9930414,0.00569177879154052,0.0218952539815427,1.6941808932968
"Hddc3",666.758001663974,-0.277511013406224,0.234690440563733,-1.18245
554756996,0.237024993748494,0.375153285827432,-0.272077507145452
"Unc45a",4380.78157362078,0.0101242451694349,0.116112980083319,0.08719
30525094616,0.930518061619992,0.956679177759194,0.000448374280180391
"Vps33b",692.970979184424,-0.172135021628031,0.142277411431731,-1.2098
5488768627,0.226334580318505,0.362469950476403,-0.174684978776454
"Iqgap1",8692.54605478365,-0.241094441808117,0.190776808951864,-1.2637
5130778578,0.206319303055505,0.339334776126212,-0.260567186302804
"Cib1",5583.68285119216,1.23783569274496,0.255355323106887,4.847502991
84806,1.25025147653229e-06,3.28094256923642e-05,12.780399418723
"Sema4b",2028.95162456426,-0.125761702967284,0.202695346954625,-0.6204
46916304583,0.534963592350002,0.666797936689915,-0.0509672218146592
"Idh2",11486.7094780518,1.28968913317633,0.245721502920652,5.248580681
16568,1.5327551037421e-07,6.34515053349542e-06,15.4347670566072
"Mesp2",3.42988771260589,-1.41593470051272,0.898105315934193,-1.576579
80126739,0.114892243435376,0.219044584189201,-2.15006850861896
"Mesp1",1.04301602627917,0.442843738203621,2.08231064238041,0.21266939
1968041,0.831584842285062,NA,NA
"Pex11a",2691.03336936791,0.504344225204569,0.475092684921406,1.061570
17611838,0.288430858986868,0.432452171941507,0.422783464415453
"Plin1",7729.49998473901,-4.02977204057614,1.64255918765555,-2.4533496
6974791,0.0141532683800661,0.0443309922690005,-12.5570567867381
"Rhcg",4134.07355750583,2.14557357180511,0.629184074337612,3.410088810
75687,0.000649417285259418,0.00422500441374312,11.7292820832056
"Nr2f2",10322.4525835048,-0.164107797207823,0.220174762994237,-0.74535
2441742466,0.45605868622343,0.596153946365275,-0.0848857995337793
"Pgpep1l",40.2707890440425,0.485591730868242,0.40695062990976,1.193244
81934313,0.232773506350592,0.370046296520493,0.482739926113414
"Synm",442.954158701158,-1.64329217026401,0.407794586208118,-4.0297057
0439441,5.58467267469311e-05,0.000629058046280567,-12.1131782481639
"Ttc23",666.197304624915,0.165699429197393,0.16374300988655,1.01194810
888232,0.311562865005963,0.456673563614532,0.129872966737484

"Lrrc28",1005.65443019476,0.777698719516579,0.275757624534282,2.820225
62687073,0.00479898934818966,0.0192413519043804,3.07244933130113
"Mef2a",4069.55473690851,-0.191351632778803,0.120224340399284,-1.59162
139832328,0.111469799074939,0.21407820202432,-0.294952066685623
"Rab38",283.209743836549,1.34559625245624,0.405974354527461,3.31448584
732024,0.000918118274789667,0.00550814465287659,6.99915582511467
"Ctsc",3557.69893077936,-1.28725918090788,0.254576087903165,-5.0564811
1537296,4.27063137856423e-07,1.39851241308359e-05,-14.3883605481742
"Nox4",4883.41881254169,2.18854992459858,0.502165422998827,4.358225047
69248,1.31121516957375e-05,0.000209386350338649,18.5399274209094
"Cd22",219.46521915766,-1.75674148276816,0.600839493257118,-2.92381160
440197,0.00345773950842471,0.0150758061125369,-7.36894036808558
"Tyrobp",1847.82334023856,-1.29293250868426,0.368658818065818,-3.50712
486810345,0.000452976534532294,0.0032000494457 6473,-7.42736588974901
"Sipa1l3",2107.29811666517,-0.0173989213688857,0.148068823394632,-0.11
7505636703239,0.906459377546746,0.942925053407193,-0.00102250809769088
"Dpf1",15.9507556546791,-0.454303804217374,0.814920797052508,-0.557482
157604209,0.577198046301113,0.702407057119226,-0.160479270527267
"2200002D01Rik",1279.97926792673,0.0840008752059418,0.272665245723785,
0.308073274916145,0.758026576412487,0.843078100900728,0.01433858642053
66
"Yif1b",1377.36860417231,0.427519764137819,0.188761899736965,2.2648625
8473536,0.0235211134152152,0.0652254922678961,1.16708829929185
"Rasgrp4",406.353526715992,-1.16635663726646,0.419373145933643,-2.7811
9056638646,0.00541599345341052,0.0211117633655734,-4.49971629962715
"Fam98c",1711.07778933679,0.463362545395907,0.136404780886629,3.396967
04458639,0.000681371723080448,0.00437163075242599,2.51727224658952
"Psmd8",5432.77533455222,-0.137397935893116,0.175463866196899,-0.78305
5445381176,0.433594553470034,0.575744116287028,-0.0758562956651424
"Ryr1",76.1727060222683,-1.70283568388253,0.492471112558751,-3.4577371
9606626,0.000544732481286319,0.00369700513956261,-9.53627524322475
"Nfkbib",1006.6520952858,0.112372920617087,0.0896275756081079,1.253776
19392977,0.209923364386911,0.343667989825021,0.120023182921034
"Fbxo17",114.422635164529,-0.331024684187525,0.174634957909542,-1.8955
2360048663,0.0580230681151619,0.130292961201132,-0.674618314809764
"Lrfn1",139.182496889924,-0.144661794059611,0.2238332531104,-0.6462926
84618493,0.518089824574088,0.65146146403213,-0.0619929361264371
"Pak4",2237.0689397767,0.397475585358133,0.101172973015601,3.928673572
70248,8.54156890553802e-05,0.000875623083702315,2.79845660156026
"Psmc4",5199.91931007055,0.252412606888739,0.122590099734901,2.0589966
6803907,0.0394945576777541,0.0969972963297994,0.588896829437298
"Zfp626",709.825151931888,-0.129001839324343,0.14874719726969,-0.86725
5596691697,0.385801980054807,0.530107436237116,-0.081874317593826
"Mfge8",5522.4398178602,-0.572787645938856,0.197285142979238,-2.903349
11838311,0.00369194820262402,0.0158140610840868,-2.37526776753825
"Hapln3",16.0469501511711,-0.655709133455106,0.614207089581881,-1.0675
701153197,0.285714470092732,0.429883535602137,-0.55357650464822
"Acan",0.110907021323848,-0.186186710250895,4.78471990596567,-0.038912
77105244,0.968959934454095,NA,NA
"Aen",1670.20424888772,0.160926722080477,0.175716642484809,0.915830850

196159,0.359755620008003,0.50485929746049,0.109989473031126
"Det1",407.085569327399,-0.164852940880089,0.147740461610124,-1.115827
98025313,0.264495774425995,0.406425928738124,-0.148425936108865
"Mrps11",1104.82118829726,0.577641660915268,0.217986193766616,2.649900
21126619,0.00805155481529524,0.0286215525594579,2.05270466760896
"Mrpl46",1167.6342194251,0.557833200994393,0.299422208614572,1.8630321
4973762,0.0624577431253967,0.137589094504461,1.10645301222255
"Ccdc90b",1098.997365362,-0.0414315220861877,0.208844767175861,-0.1983
84295888533,0.842744403334078,0.899290191342374,-0.0043979354826574
"Tmem126b",1850.84109163051,0.380707891917625,0.186537512921493,2.0409
1866539386,0.0412589121953537,0.100183293255181,0.875915143567552
"Tmem126a",1217.89354259938,0.444786117479513,0.238888084985668,1.8619
0164112286,0.0626169556945941,0.137799706787042,0.881545646130671
"Sytl2",4831.5116422655,-0.569200433022209,0.245731406095298,-2.316351
99613624,0.0205390574950692,0.0586168827632991,-1.61466937996443
"Ccdc83",1.04434075302753,0.917570190803262,1.78877313783318,0.5129606
27256933,0.607978862579912,NA,NA
"Eed",1349.69064971044,-0.0559074954888463,0.108703307895801,-0.514312
734093032,0.607033360067171,0.72746997262383,-0.0177887897880456
"Me3",1507.35515921464,1.25594514158192,0.207078822543009,6.0650583490
7898,1.31905755746176e-09,1.99371970972011e-07,19.3768391457048
"Zfand6",5064.02410043405,-0.155632249679288,0.159518446526902,-0.9756
37947007224,0.329243912769792,0.474028765207942,-0.1161774936595
"Fah",12604.3087197573,1.61142351713124,0.41399860414989,3.89234045955
337,9.92817866811706e-05,0.000985138054305071,11.1554479426318
"Sh3gl3",35.0920656886122,-1.53758037673297,0.52343680616507,-2.937470
88210698,0.00330901244352243,0.0145608496104266,-6.50307148340792
"Ddias",51.240982123573,-0.299348206532653,0.336550007934974,-0.889461
296909228,0.373755214625784,0.518940338586736,-0.19636235250698
"Rab30",249.639794181768,-0.883222696263713,0.297476635159645,-2.96904
896678598,0.00298722990029977,0.0134951363753579,-3.80264989575745
"Ndufc2",6699.78200952519,0.44219898593451,0.353763501150105,1.2499847
6241019,0.211305113650407,0.345292248236308,0.470218537399169
"Anapc15",1198.6569901448,0.278128754537267,0.18018387563964,1.5435829
2910467,0.122689403175854,0.230329095236163,0.40836147135562
"Tmc5",57.051683050955,-0.441129555658423,0.366643873261439,-1.2031553
9909178,0.228916190648852,0.365463676841997,-0.444035886607208
"Art2b",76.5047067112163,-1.86274304980191,0.75717856550084,-2.4601106
458551,0.0138894187765679,0.043679981823471,-5.83199769605095
"Coq7",1947.77057398213,0.206878837923399,0.40139109195527,0.515404656
629631,0.60627027914057,0.726787484894267,0.0660194151660981
"Gm45837",2139.66759226346,-1.10236235347075,0.312231471233822,-3.5305
9334190312,0.000414628687142569,0.00298098456559949,-6.41079007656816
"Arl6ip1",19920.3706695861,1.06411058387395,0.265057256900819,4.014644
21806843,5.95355318450108e-05,0.000662361615482035,7.78896907474478
"Smg1",5498.85729521746,-0.0532954967795145,0.184925404434071,-0.28819
9974160474,0.773193668564249,0.853273219584609,-0.00845668845111145
"Xylt1",691.339678809142,1.06987801921752,0.274436308805298,3.89845652
667105,9.68077760528396e-05,0.000965502822239523,7.42801494464255
"Nucb2",1316.92623511631,0.249784798154754,0.167665391466099,1.4897814

9855845,0.136281697369155,0.248896640624183,0.347380106660767
"Pik3c2a",2786.98451470215,0.330978334495491,0.158930750485782,2.08253
175350796,0.037293925598822,0.0928680665805658,0.786594978275811
"Ipo5",3715.51828091914,-0.264119030134894,0.147896166279661,-1.785840
95030201,0.0741250141824205,0.15693247852973,-0.489132501374084
"1110004F10Rik",3157.05318291024,-0.195140989147857,0.165575648310909,
-1.17856092449919,0.238573059037271,0.376706060797423,-0.1905142109745
97
"Calcb",6.25536971220257,0.207172228199525,0.822707652888123,0.2518175
53261168,0.801182088905563,0.873096823691072,0.0281150986545096
"Calca",105.278086141567,-0.479317820643322,0.328365380042856,-1.45970
875669282,0.144370134245606,0.260352282419611,-0.645027401531224
"Cyp2r1",43.9617502983612,-0.318610750969625,0.377152305687605,-0.8447
80069390667,0.39823364446259,0.542610733733863,-0.194786855423167
"Pde3b",2273.85339029243,-2.23084643718683,0.612423073241771,-3.642655
76307923,0.000269839592314081,0.00213095681285539,-13.7223473396742
"Mylpf",55.548484975679,-0.223336286048999,0.290450607507013,-0.768930
345733935,0.44193466162691,0.583509700922065,-0.120309962388962
"Qprt",2322.7486425463,1.76709909253641,0.543619061880278,3.2506201795
5062,0.00115153587137284,0.00655017325822581,8.88544035922104
"Kif22",229.51766587383,-0.574810944813784,0.554604216267407,-1.036434
5022156,0.299999481118589,0.444448741193697,-0.466126006696596
"Maz",7420.5223252937,0.0205328418349,0.14756434883414,0.1391450034993
12,0.889335566450144,0.93147103967796,0.00145763012565433
"Pagr1a",303.822736049649,-0.129290919598187,0.549154414621768,-0.2354
3636572099,0.813870063436636,0.881651119333714,-0.016285336522801
"Mvp",7036.12320945181,-0.552486812653968,0.146807492024744,-3.7633420
8175731,0.000167657613948113,0.00147984977671359,-3.59990169466144
"Cdipt",3899.47493622232,0.0309525443172973,0.131941819631271,0.234592
371120835,0.814525131300544,0.881968734941856,0.00388759844627302
"Sez6l2",103.50272784819,0.451706783119802,0.445652235381209,1.0135858
1256395,0.310780430252047,0.4560545517464,0.354654349075578
"Kctd13",592.825596196871,0.0125528023752838,0.10494171054318,0.119616
902662537,0.904786629476253,0.941985577839354,0.0007502221839778
"Stard10",14259.7196131396,1.79906931452847,0.415006114763553,4.335042
9078701,1.45731693692435e-05,0.000226232210478909,15.101295365131
"Ino80e",1667.72102976193,-0.285913239533161,0.0857560931912033,-3.334
02827593467,0.000855979556682138,0.0052303006569064,-1.50198416586461
"Fchsd2",1182.67139845862,-0.264198788135575,0.222137996634068,-1.1893
4532650348,0.234303805809639,0.372084672232965,-0.261195861666238
"Klk10",5.15112265039442,-2.29659785101966,1.00637707379765,-2.2820450
8112775,0.0224866788381176,0.0630312340404855,-6.34808329846379
"Aldoa",55671.3292833176,-0.751035545743773,0.497334291361345,-1.51012
218298476,0.131012250122338,0.241466759890854,-1.06723912646593
"Ppp4c",3965.93224252093,-0.108347839264432,0.113841660819545,-0.95174
1554756291,0.341228069180037,0.485781607248274,-0.0782267199796407
"Tbx6",193.247811982927,-0.601037772222532,0.283143283027209,-2.122733
64141495,0.0337761782691936,0.0861323468339965,-1.47366663222147
"Plekhb1",638.370382523984,0.409746403552022,0.209441727455773,1.95637
425516625,0.0504210826419336,0.117176432580741,0.878526819114806

"Gdpd3",347.417556784821,0.260530979638321,0.215960152219956,1.2063844
9714079,0.227669270804298,0.364128901824476,0.263200731288386
"Rab6a",5336.26119020365,0.10459222924538,0.0911050234510833,1.1480401
9891986,0.250951969293032,0.390850826525723,0.0982570057126754
"Mrpl48",2365.92668910061,0.213494550534171,0.191878263144684,1.112656
25941792,0.26585608806301,0.407616733903639,0.191595971219285
"Coro1a",4580.68035179866,-1.16069514412998,0.415059590720222,-2.79645
422026248,0.00516667301131692,0.0203824217255232,-4.51868187186575
"Dnajb13",28.4954066378238,-1.82158267741127,0.530116642673108,-3.4361
9220899377,0.000589952266392423,0.00391121216310453,-10.0986866630029
"Sult1a1",1753.02957612141,-1.09410085540745,0.417039945872278,-2.6234
9174518291,0.0087033554098079,0.0303628598640964,-3.82337387768566
"Klk7",1.46249748982339,1.2768185381821,1.41128727058858,0.90471909213
0404,0.36561422393135,0.510605264192508,0.85822438795598
"Sgf29",605.867403546599,-0.20149461785476,0.203735785555605,-0.988999
636491287,0.322663319520235,0.46778863885832,-0.153083261332987
"Nupr1",4999.06932745628,-0.930614090499367,0.308610650851013,-3.01549
569962391,0.00256559577045359,0.0120656021061997,-4.11089158996742
"Ppme1",1688.88937652255,-0.270990353591768,0.1735007480758,-1.5618973
2088865,0.1183121797351,0.224053115819249,-0.405366917719555
"Cln3",1480.8892870509,-0.120619979715017,0.181717835475601,-0.6637762
2977582,0.506833541507482,0.641412370501258,-0.053565246891874
"Nfatc2ip",437.738588349236,-0.292476667505139,0.119957128920175,-2.43
817662308144,0.0147615595328444,0.0456931080761074,-0.902526782027408
"Cd19",184.41534231174,-1.58878069767284,0.722164448558639,-2.20002618
634007,0.0278050371810091,0.0741848132122762,-4.13272971515872
"Lipt2",155.800584566478,0.186197512059467,0.164198714232792,1.1339766
7533186,0.256804332076041,0.397678569360546,0.171694814012964
"Pold3",1753.1879718208,-0.0148577220296716,0.114455638877227,-0.12981
2057976531,0.896715122755394,0.936383433134355,-0.000976601569548343
"Rabep2",1711.5083357011,0.0440942278937662,0.13287389807032,0.3318501
86787103,0.740002385973033,0.830564501594646,0.00818607960551749
"Pgm2l1",635.274504486074,-0.166305926799419,0.209954572001971,-0.7921
0433577916,0.428299842182602,0.571046931268898,-0.0931785301569506
"Atp2a1",12.4931189077099,-1.94276845291901,1.00716961459153,-1.928938
70582754,0.0537384719908993,0.122793006782908,-4.07448126536657
"Syt3",396.292624724197,0.394069827921744,0.13963632151882,2.822115504
30045,0.00477079912402927,0.0191558981302147,1.5586031576104
"Chrdl2",8.05248411546646,1.19071976269136,0.667977093885839,1.7825757
403814,0.0746553918013319,0.157672316869841,2.19954082046794
"Sh2b1",3738.57910185245,0.0313777327204412,0.123697235607592,0.253665
593788866,0.79975391696075,0.872162120833555,0.00429184483708243
"Gm9755",4.04629294778113,-1.52647037058418,1.64022031288867,-0.930649
595416749,0.352034850854469,0.497106868401129,-1.06692684566657
"Slco2b1",2901.94854606042,-0.824943046775051,0.129616949104084,-6.364
46894080658,1.95966569875087e-10,4.15321323850483e-08,-14.021390686363
1
"Eif3c",7654.45895542174,-0.026949538253997,0.210439714108821,-0.12806
2986438297,0.898099127350362,0.937427022755747,-0.00174138124247852
"Myh14",2178.55539396309,0.69822658015078,0.269435617932974,2.59144127

085853,0.00955748530797232,0.0326659206108804,2.38892843004551
"Spns1",3183.82941996686,0.389422846464675,0.12193671785965,3.19364710
89283,0.00140487737521999,0.00764294058650652,1.89803638330532
"Lat",567.36275915365,-0.790637013717469,0.441427774038201,-1.79109032
149175,0.073278802045235,0.155590774504845,-1.47100068801751
"Rps3",26401.9281711702,-0.568578836756629,0.22665935947959,-2.5085169
130544,0.0121239164019203,0.0392612725468597,-1.84078346638287
"Il21r",614.380925507771,-1.52670189164197,0.468024177775713,-3.262015
00721101,0.00110623287000111,0.00636075744596924,-7.72145941596693
"Dgat2",14101.7123171095,-2.16545912491005,0.928291653520707,-2.332735
74818558,0.0196620187379614,0.0566863878977927,-6.21534662182999
"Il4ra",1790.02706667926,-0.88868905936711,0.196194830575021,-4.529625
25445998,5.90883965129118e-06,0.000112730484854085,-8.07863738350624
"Nsmce1",1197.17242964143,0.176233147937984,0.198404884082257,0.888250
048647589,0.374406262486995,0.519511336889512,0.115409210501879
"Psma1",4813.54082775062,-0.489807133032099,0.257458208889924,-1.90247
238627189,0.0571094255600118,0.128800013052572,-1.00385695858259
"Kdm8",199.956454935553,0.16745920290914,0.143354462473309,1.168147820
58368,0.242747143787079,0.381577217797522,0.161337236566366
"Thap12",2245.58376313627,-0.596178055513756,0.213310547870986,-2.7948
8314789917,0.00519184775738499,0.0204671750047498,-2.31849645162688
"Copb1",4243.83685792242,0.441332075921999,0.15818193219144,2.79002835
410985,0.0052703425799763,0.0207221495511954,1.71084679637248
"Zkscan2",58.6726223914823,-0.966737706923266,0.465039643810012,-2.078
82858975829,0.0376331081162822,0.0935573558652647,-2.29037621960859
"Far1",8436.61049974985,0.3943784102653,0.148499317358208,2.6557590787
7061,0.00791301242788794,0.0282475130572975,1.4066491390624
"Acer3",1512.97552252518,-0.0212078871900798,0.119934456511089,-0.1768
28976484493,0.859642727756942,0.911029575198301,-0.00197614918310142
"Myo7a",2141.30007849233,0.0698593548261821,0.253295541901441,0.275801
754352885,0.78270033617858,0.859489251791505,0.0105778910951543
"Aqp8",31.5758342288368,-0.605071500137829,0.784210327372827,-0.771567
880475219,0.440370403999271,0.582164359426251,-0.327345174048096
"Lcmt1",1584.85904371701,0.393248650573764,0.17193645398326,2.28717436
857254,0.0221856474603889,0.0624072086040118,1.09090104891011
"Arhgap17",1893.58775783288,-0.22868461025753,0.131665292836562,-1.736
86326389294,0.0824113090722396,0.170091638011065,-0.405096021221628
"Disp1",293.12278425091,-0.672127054349306,0.205394580735639,-3.272369
95222572,0.00106649923795898,0.00619071215889378,-3.41756789403299
"Slc5a11",1208.68183133858,2.19078140295458,0.845614633526089,2.590756
25716095,0.00957652929082978,0.0327068082173059,7.49284925716709
"Parva",6260.66527676669,-0.179855333707668,0.102312649566644,-1.75789
92868376,0.0787646472078164,0.164321965681361,-0.324805705817715
"Dkk3",2713.65449032861,-1.11217217456772,0.323872055852285,-3.4339862
1298459,0.000594774447242317,0.00393382366768341,-6.15936896897499
"Pak1",2646.52968369354,0.0283583713613262,0.254794412464862,0.1112990
31587818,0.911379223316629,0.945417753576699,0.00159170949767218
"Trat1",52.2301806449733,-0.905142963508255,0.252670355306943,-3.58230
771634721,0.000340572284099584,0.00255599630306916,-5.40308182985687
"Rbbp6",6189.11389251939,0.305201999947451,0.179885995552368,1.6966412

4775406,0.089764506541328,0.181672031196057,0.520537955854183
"BC017158",1743.00510505864,0.152987656802886,0.118065642100679,1.2957
8473534602,0.195049658495263,0.325633893427573,0.171649329072106
"Slc5a2",12636.4909244196,1.85954441576113,1.00782322770034,1.84510970
242693,0.0650215821093196,0.141795191585674,3.63238120474095
"Tgfb1i1",1748.35751566419,-1.06815823733626,0.215432370183089,-4.9582
0677472221,7.11468186140432e-07,2.08604611930318e-05,-11.5122409670658
"Cox6a2",25.0459162156957,0.677709459521161,0.561099877867815,1.207823
21695821,0.227115269075451,0.363391885477855,0.686027294530094
"Itgam",1053.49581928453,-1.51526598047878,0.384968599440865,-3.936076
81945898,8.28244704487836e-05,0.000854450542227716,-10.7054328493591
"Lyve1",136.317313143934,-2.02090237798791,0.516487130762407,-3.912783
60605961,9.12382877111953e-05,0.000919361309453688,-14.1298086175984
"Rnf141",1617.37098033815,-0.61173930782158,0.200227527602034,-3.05522
080379205,0.00224894884316193,0.0109473204546418,-2.76179532602624
"Itgax",1620.43647636386,-1.45723589070641,0.47268306984325,-3.0829026
5439305,0.002049921991463,0.0101806874654981,-6.68472390358655
"Adm",387.715086298036,-0.216182270627292,0.207517375873095,-1.0417550
3240507,0.297525267212873,0.441802074842429,-0.176597846653092
"Dkkl1",29.0778465433534,-1.56945241428654,0.403815864720415,-3.886554
61907907,0.000101677057225853,0.00100379709457705,-10.835445126214
"Pycard",1430.1580453704,-0.134190721191773,0.203827409124996,-0.65835
4643116133,0.510310273313983,0.644640732690381,-0.0589180626584433
"Fus",18521.0279134392,-0.104518939022162,0.182499465208795,-0.5727081
93432696,0.566842288859108,0.694064992977574,-0.0381692371676368
"Tead2",737.434544397312,-0.981747967775612,0.345883675612606,-2.83837
612757181,0.00453437155427973,0.0184189951177805,-3.92146738238364
"Cd37",809.571677976489,-1.33057906064859,0.44241667946927,-3.00752463
095373,0.00263384779663488,0.0122876278810099,-5.85343335541246
"Prss8",8451.33506733223,1.91574904320612,0.425103500032566,4.50654733
037804,6.58909931128615e-06,0.000122473077570503,17.2563381073959
"Kat8",783.464285566528,-0.389802835411753,0.163505703740667,-2.384032
0337082,0.0171241116923322,0.0510763239677365,-1.15944289136993
"Bckdk",3560.7994696497,0.504427035111579,0.243633907126088,2.07043034
798323,0.0384120623476798,0.0950334210956809,1.18718246922875
"Gm21974",0.77877798716069,-2.12925847848195,3.00194438888195,-0.70929
3112280131,0.478142601780606,NA,NA
"Stx4a",5045.1636606769,-0.169801003301708,0.161433024134704,-1.051835
60929901,0.29287497842052,0.436847944403557,-0.140624113501793
"Stx1b",210.741155296375,-2.61574762919728,0.567523937128272,-4.609052
51403708,4.04508161510556e-06,8.43740065777616e-05,-24.5363697925117
"Fbxl19",2537.7959953229,-0.0179130830289105,0.169551615541923,-0.1056
49733691162,0.915860263748601,0.948401052768082,-0.00094899599421782
"Bcl7c",1825.75635867479,0.0252724571776451,0.187166752758263,0.135026
423257372,0.892590982902907,0.933727076808889,0.00173295999728183
"Phkg2",2626.99680527404,0.479695656246205,0.223962855400042,2.1418536
3635132,0.0322052617313184,0.0829947387308018,1.19395196482401
"Rnf40",3870.09239374595,0.070331253387369,0.112715114036688,0.6239735
81435377,0.53264493512656,0.66484290026872,0.0287095345757917
"Prr14",2550.77240701079,0.234924198865241,0.195476893577199,1.2018003

4870701,0.22944089033882,0.366121489469377,0.236049500394607
"9130019O22Rik",209.288997624769,-0.0635105000119308,0.173558039043503
,-0.365932343796599,0.714415560865204,0.810574911557037,-0.01333793637
2086
"Nucb1",7913.81789075872,-0.229346620476304,0.181871957883821,-1.26103
343882628,0.20729679979805,0.340358215204035,-0.247179843506967
"Hsd17b14",225.201665450479,-0.0164847937147839,0.413475814220814,-0.0
398688221845554,0.968197707623757,0.980477693900995,-0.000325004027088
057
"Bcat2",4347.50229438306,0.1231122575007,0.300662695263578,0.409469679
611474,0.682195013409892,0.786209627720872,0.0296124153826958
"Fgf21",3.67946027100879,-3.99689536922251,1.5775537883704,-2.53360322
715289,0.0112896482642756,0.0372841398809021,-13.1465372761307
"Itgal",834.303432362099,-1.50963886942776,0.47719641866794,-3.1635586
7389326,0.0015585293272736,0.00828467961373519,-7.23622339798097
"Abcc6",462.883332382409,2.02435236123128,0.672782918845471,3.00892353
911894,0.00262175088044056,0.012249915808011,8.91167727618988
"Nomo1",2759.40188262464,-0.143991573630854,0.174729796125268,-0.82408
1392091956,0.409893312411768,0.553513680875572,-0.0851665247212889
"Ush1c",3946.56858412576,1.94770392213759,0.662016413338403,2.94207799
51901,0.00326017803087044,0.0143849176840715,8.2613322719932
"Sergef",703.755942225635,0.798928978647254,0.181293872112706,4.406817
33660903,1.04900579305802e-05,0.000177702128175893,6.89907277754981
"Lamtor1",3338.39534077815,-0.336903310295231,0.318971444994344,-1.056
21777617493,0.290868731196495,0.434486977772869,-0.280838996392079
"Rgs10",772.020094833019,-0.939917567030049,0.308294108605436,-3.04876
914866052,0.00229780978718397,0.0111255761684909,-4.22822731670473
"Tial1",4445.94183982297,-0.00638711298408078,0.148059793901641,-0.043
1387402060269,0.965590937810552,0.979228745789391,-0.00013406557340621
6
"Bag3",2101.41676531892,-0.424285306535304,0.21113683422118,-2.0095276
5111007,0.0444812052735084,0.106322536162412,-0.950941327848792
"Fgfr2",1405.47629035495,-0.126410505499271,0.16233142055634,-0.778718
655119504,0.43614546447543,0.578304268367354,-0.0692293621964293
"Ate1",2138.82038833159,0.00840592266176975,0.140715947163992,0.059736
8161262731,0.952365249051226,0.970244861929682,0.000253916061326205
"Ldhc",0.437352914812939,3.40102936315834,4.68366244651603,0.726147411
773496,0.467748406460144,NA,NA
"Tacc2",5737.70675412737,0.790691292998286,0.20494346649711,3.85809465
660343,0.000114274393637123,0.00110303075600823,5.38436548110424
"Ptpn5",13.990764057177,-1.86054879726484,1.00379646075999,-1.85351201
164446,0.0638090061147187,0.139791910249976,-3.66081640413338
"Fam24b",1.66094648996878,-0.934277647914555,1.50257651544279,-0.62178
3741668048,0.534084078399386,0.666297994793731,-0.379333793262146
"Acadsb",5384.51415182313,-0.298139548789032,0.352600436062846,-0.8455
45037090914,0.397806598187162,0.54225622563292,-0.182466367354422
"Cpxm2",670.30503086732,-2.45339619768675,0.572209646778624,-4.2875827
2688807,1.80627996019486e-05,0.000266204434345271,-20.1945076243844
"Chp2",57.8998274498009,-2.88257429982333,0.568383644389353,-5.0715292
8884899,3.94631513936072e-07,1.32150888591343e-05,-32.3832757768293

"Ern2",28.8636305929552,-1.99799990583791,0.583745136626268,-3.4227264
2712755,0.000619964389761354,0.00407005578167235,-10.9971884326764
"Plk1",194.438965578775,-0.438324944591252,0.349368910844658,-1.254619
2033273,0.209617028908419,0.34328177638639,-0.468658636589975
"Dctn5",3676.47401098206,0.205825664561393,0.0790644597589435,2.603264
03530647,0.00923408081058431,0.0318215107184116,0.709607191633156
"Ndufab1",7557.15654629801,0.81243225354006,0.363338088731084,2.236022
80833641,0.0253502771520045,0.0692171550076717,2.16960564829218
"Ubfd1",2505.69644113664,-0.0350596017053708,0.0872065136236543,-0.402
029621969213,0.687662226121855,0.790381872490211,-0.00824738800907585
"Ears2",608.691218429399,0.146536175000564,0.208635714764906,0.7023542
21403002,0.482458286787411,0.620062729930851,0.0700347123978168
"Gga2",3226.61115082307,-0.00359130353635888,0.271973455742402,-0.0132
04610451986,0.989464551353022,0.992978528091061,-2.53051810369112e-05
"Scnn1b",1880.09752547591,1.21392467795494,0.328428465203057,3.6961615
8941767,0.000218883687332566,0.00181762952964666,7.66013431000867
"Mettl9",3637.28817900357,0.272997334386037,0.185927199335726,1.468302
29983236,0.142022126512367,0.257021294109721,0.370893179616348
"Mfsd13b",29.1801051759441,1.16923317275653,0.472235120075779,2.475955
56334079,0.0132880115623202,0.0421421030322249,3.7026200022893
"Cdr2",2418.97708217033,0.604406243891255,0.232534651895923,2.59920936
06839,0.00934387659557069,0.0321147375040432,2.07821475146973
"Mrpl17",2624.54914127249,0.0107068242800178,0.366033113083568,0.02925
0971830992,0.976664428962715,0.98525471005355,0.00015905075640312
"Polr3e",5956.55204707097,1.59432040414839,0.423190134238982,3.7673855
6775535,0.000164966116453933,0.00146204969937218,10.4075895544116
"Arfip2",2898.42611906641,0.0132352950433108,0.130581411713623,0.10135
6654592899,0.919267343839178,0.950561510035433,0.000671061300187312
"Dnhd1",588.675840572257,0.38820617484168,0.228846167006135,1.69636301
940453,0.08981715258472,0.181740851089339,0.661958882544443
"Uqcrc2",13873.7916407628,0.217573348333182,0.414169094530281,0.525324
924545462,0.599357333537579,0.721152069689338,0.0711258696065772
"Pdzd9",4.57180905367802,0.0710743336454207,0.801747288220884,0.088649
2972157574,0.92936062876535,0.956303244594089,0.00317561649192843
"Rrp8",843.088330016055,0.385909537778,0.333415461753327,1.15744343633
203,0.247091236357057,0.386350034201275,0.36700440687321
"Vwa3a",110.823259224536,-1.73226007358726,0.73380211292729,-2.3606637
853316,0.0182422609122716,0.0535109979340261,-5.07182897111763
"Ilk",6110.74208162419,-0.332073958531196,0.140232273170017,-2.3680280
6532695,0.0178831792780221,0.0527194178353304,-0.977217758286162
"Tpp1",7812.24582836356,0.30030162714678,0.142775317980216,2.103316115
10325,0.0354381456788001,0.0892845282911544,0.725506827644899
"Hpx",85.3961224282055,0.0114326232547756,0.334837312594964,0.03414381
4995329,0.972762469523751,0.983326857669524,0.000192224740592775
"Cnga4",10.3817268739861,0.462722645398836,0.579574791145885,0.7983829
73979909,0.424648280558885,0.567650508458571,0.26201639794925
"Cckbr",1.74154780006492,2.09958346745293,1.10317454541839,1.903219645
67868,0.0570118907229175,0.12861481491559,4.30610559135528
"Crym",605.454248820066,1.21721497669517,0.591931766001034,2.056343393
96653,0.0397494283562048,0.0974395717990045,2.83431291038081

"Anks4b",1569.98990831214,1.44687679530602,0.579677482641017,2.4960031
0281854,0.0125601502875632,0.0403057620649945,4.6462987944741
"Zp2",0.330971878098449,0.433072734184038,3.90222404385468,0.110981001
940688,0.911631411911495,NA,NA
"Tmem159",1613.19231259228,-0.0606687207238001,0.167120205748088,-0.36
3024449690124,0.716586620231021,0.812137056571986,-0.0126243213812411
"Trim30a",1493.84558747028,-0.92802282523459,0.245641786959101,-3.7779
5177572578,0.000158123487473524,0.00141728516627766,-6.08691293239353
"Lyrm1",267.627496946785,0.666781969220186,0.242201930311513,2.7530002
2738296,0.0059051855230071,0.0225539586536696,2.52833366163309
"Rexo5",384.374341199677,-0.0826577574544225,0.336508006822317,-0.2456
33850543317,0.805965682933222,0.876643918656252,-0.0108822567286721
"Eri2",532.723413496962,-0.0402495217270029,0.164821373376712,-0.24420
0863652614,0.807075258213452,0.87716574050423,-0.00527507489609827
"Chst15",1584.66922074238,-0.767759682231731,0.138268346208523,-5.5526
7856515671,2.81325098393258e-08,1.75735172875028e-06,-10.174122963144
"Oat",7626.06077171353,0.202459914066158,0.176048959625662,1.150020508
48045,0.250135424494028,0.389828191734038,0.190727194194071
"Acsm3",19280.3548019459,1.77923931764267,0.630420749849397,2.82230449
754028,0.00476798827872135,0.0191485551922778,7.03783096290926
"Thumpd1",1763.16206634144,-0.457802549912719,0.20955287815597,-2.1846
6362257252,0.0289135192931638,0.076503704597382,-1.17674305096546
"Acsm2",115980.612752769,2.16030530341336,0.899428531398059,2.40186432
606871,0.0163117570144785,0.0492332257381273,6.50508230275898
"Lhpp",422.37694443425,-0.870296583688521,0.218977214352163,-3.9743705
1276438,7.05656399905925e-05,0.000755567736703225,-6.25572763202762
"Gp2",200.402933774158,1.44967487189669,0.608667308166039,2.3817196232
613,0.0172320119031002,0.0513431797198421,4.30440821991602
"Fam53b",1659.1943070899,-0.700325514685197,0.231416818577048,-3.02625
158789845,0.00247606184042112,0.011740820468028,-3.11272531611251
"Eef1akmt2",597.333151651469,-0.221804127373445,0.161102307586832,-1.3
7679050471636,0.168577027366543,0.292560291908577,-0.272616016625011
"Umod",63880.7818143256,2.13305759558903,0.607022764799707,3.513966393
48913,0.000441468661480534,0.00313009307692634,12.3006873192556
"Abraxas2",1566.02120786217,0.0415639166397843,0.0820017115199383,0.50
686645277737,0.612248525593673,0.73173191136267,0.0129821183384514
"Trim21",710.8713603482,-0.562633526072638,0.222346988787077,-2.530430
15847372,0.0113922762937589,0.0375404095277525,-1.84675303067744
"Zranb1",4029.57443980333,0.386547859987257,0.191607808154481,2.017390
95974424,0.0436547299412377,0.104902628098873,0.871558238616285
"Pdilt",168.38427249381,1.75360161625686,0.528214888664144,3.319864043
76393,0.000900612964893219,0.00543594278428342,9.14454546778471
"Ctbp2",2257.52145265725,0.155549575677343,0.191918892456558,0.8104964
22141204,0.417654920543275,0.560907347617129,0.0899386935489942
"Acsm5",2338.74338449175,2.09544241855957,0.565261774655464,3.70703010
978723,0.000209704018802206,0.00175636123651435,13.2945576616893
"Rrm1",1166.8721338726,-0.39383547995577,0.179361102152766,-2.19576862
11157,0.0281085087030595,0.0747963798883041,-1.02120980406365
"Uros",1337.21163295004,0.25425205044729,0.231622610019137,1.097699617
60764,0.272335676805142,0.414941081999427,0.223644868235506

"Knop1",4444.6917621738,0.706656437384142,0.249956520458536,2.82711743
66158,0.00469690994430571,0.018922597200762,2.80358765578209
"Mmp21",0.215137371833175,1.59126336803356,4.75777103092869,0.33445564
2713632,0.738035757496647,NA,NA
"Vps35l",2115.18033393058,0.370455114233651,0.172830401309566,2.143460
36013715,0.032076150796241,0.0827057376653143,0.92334688792445
"Bccip",3757.35398256694,0.308930184026875,0.147243627742643,2.0980886
491525,0.0358973150773462,0.0902198826215645,0.74313324073672
"Dhx32",1384.25968578935,-0.633285294790776,0.303352048546356,-2.08762
491575527,0.0368316826491006,0.0920225715392315,-1.51084227325261
"Stim1",1910.39841042504,-1.15512983816908,0.20919106273774,-5.5218890
4751276,3.35374253384602e-08,1.99474571569132e-06,-15.1610721681054
"Pgap2",1200.20411493348,-0.622182573906064,0.140719176140745,-4.42144
83837211,9.80414863099098e-06,0.000168275783456921,-5.40670830374388
"D7Ertd443e",32.366343716499,-0.224716377034794,0.551866109950941,-0.4
07193652559623,0.683865763188047,0.787457311205371,-0.0536951058861487
"Art1",0.166770394071505,-0.542540875129236,4.78471990596014,-0.113390
31036141,0.909721121378941,NA,NA
"Mki67",1092.66373600583,-0.668508608569072,0.436916607253044,-1.53005
9964468,0.126001886869159,0.234941645804233,-0.968279976278325
"Atp6ap2",4702.67539527056,0.221331742690313,0.280064472806224,0.79028
8537751991,0.429359279305206,0.571990108492273,0.12364334391898З
"Usp9x",7128.77299462169,-0.320170045179108,0.151653711514477,-2.11119
162189805,0.0347558446701953,0.0879176776569108,-0.778446842815135
"Cask",1285.40710137243,-0.272087252560884,0.192054413356748,-1.416719
60464388,0.156564922893274,0.27711536552043,-0.349175387750135
"Swap70",2298.80449618018,-0.607344593092023,0.183766489033442,-3.3049
8012062198,0.000949831367605642,0.00564456324461287,-3.14426088995051
"Wee1",586.838826211314,0.436223963153645,0.184716268008832,2.36158930
589041,0.0181967884974727,0.0534338828499583,1.27783531519085
"Tmem9b",2475.88275868832,-0.0456892145309184,0.20688084400815,-0.2208
47970482557,0.825210810592806,0.888212000272621,-0.0054162199609603
"BC051019",3.57193615263202,-1.41032625581477,1.24757694290412,-1.1304
5232507408,0.258285676472238,0.399338206316366,-1.29460417202918
"Akip1",834.857011218886,0.445424878594468,0.198528974413843,2.2436265
5330078,0.0248564393205205,0.0681167749643722,1.19664808649274
"St5",2605.39595155632,-0.250607050821152,0.255646246141013,-0.9802884
04794018,0.32694377669326,0.471749408566948,-0.188282918851411
"Trim66",12.7038546382824,-1.661947092355,0.831402343564515,-1.9989684
9608295,0.0456117626353245,0.108291569422451,-3.69438868069896
"Stk33",7.89083937677454,-1.63981671262402,0.903609785108909,-1.814739
87958904,0.0695638933853083,0.149362051891503,-3.117918049475
"Tub",34.9698531765272,-0.836872476904669,0.451824902270235,-1.8522052
9612185,0.0639963485708015,0.140029267695375,-1.64521080192361
"Eif3f",5154.38823062012,-0.228675396626386,0.184024505895664,-1.24263
556917815,0.214002156469686,0.348705837108971,-0.240915609866697
"Ndufb11",7439.46162538284,0.646495641247849,0.326866164449111,1.97786
039536222,0.0479444578993539,0.112751301425836,1.41102248181793
"Rbm10",2980.87269928872,0.261444892608225,0.146746440868987,1.7816097
6893224,0.0748128902670732,0.157902331070296,0.482569386516021

"Cdk16",5435.50307594919,-0.0352767576410432,0.15516270767435,-0.22735
3325871839,0.820149010517941,0.88465228782008,-0.00432354080465287
"Usp11",1022.41788211773,-0.614389227099613,0.178390372745439,-3.44407
165949667,0.000573023805875806,0.00382630759142687,-3.41960136152372
"Glrx3",3801.47580735169,-0.383147340428908,0.354807966977446,-1.07987
242702829,0.280198993228106,0.424072030579958,-0.328683694938624
"Mrgprf",132.020667374767,-0.928217290437087,0.261466073927481,-3.5500
4867933472,0.000385159923547382,0.00280913468248173,-5.45316406767969
"LTO1",1116.522196965,-0.13851037519431,0.104634014620149,-1.323760496
97741,0.185582599444914,0.314070691728827,-0.1604140160926
"Fgf15",0.693865130813784,0,4.28809205650332,0,1,NA,NA
"Fgf3",0.738994073664182,-2.51013608827861,2.25122312170634,-1.1150099
0909156,0.264846175446164,NA,NA
"Ano1",2123.09304619582,-1.62678094968146,0.292441639630411,-5.5627541
677628,2.65549851931461e-08,1.7031878332104e-06,-21.6085457790921
"Fadd",655.198968677032,-0.233791396659996,0.205739092619476,-1.136348
92466647,0.255810562570354,0.396612146071626,-0.216209450835727
"Cttn",7548.81780875697,-0.236065543174104,0.0996843308417241,-2.36813
089059024,0.0178782096772117,0.0527127373788407,-0.694716851854568
"Zfp300",61.1101312958488,-0.620248162245654,0.228703215123728,-2.7120
2204966861,0.00668741442258115,0.0248838180174594,-2.29090988724319
"Slc6a14",14.3143663921846,2.09470637406322,0.680601910412147,3.077726
26261767,0.00208586422647197,0.010306752365993,9.58318955505657
"Nadsyn1",1075.35812381402,0.852305328667216,0.237512783651405,3.58846
086330299,0.000332635913826636,0.00250801819326827,5.10382795480451
"Dock11",486.593380350563,-1.12346974722289,0.33272182098772,-3.376603
74630001,0.000733866902140941,0.00463620638581795,-6.0373678285595
"Cul4b",1224.78532835239,-0.0206187724375546,0.230698211893566,-0.0893
755190745353,0.928783478544973,0.956146793277185,-0.000924624690227363
"Tnni2",214.790368842606,-2.06420176590738,0.419355342939615,-4.922321
36936099,8.55236178356561e-07,2.45587555310696e-05,-21.9103501817011
"Syt8",1068.61755117467,-3.05974531070948,0.832285532216722,-3.6763168
3150866,0.000236625614479252,0.00192720393948055,-19.1285640540734
"Smarca1",146.590597547278,-0.833944633651618,0.25507989465531,-3.2693
4678555724,0.00107796098598812,0.0062312728445883,-4.23491650489175
"Sash3",517.903188289722,-1.38451250289929,0.392107983573855,-3.530946
9862873,0.000414074730260233,0.00297809999653782,-8.05297511495761
"Elf4",896.242475723102,-1.30554380301774,0.234655960705074,-5.5636507
1270706,2.64188487096469e-08,1.70004855491979e-06,-17.3439584815553
"Rab33a",17.3612499088108,-0.273451410301047,0.407691848985095,-0.6707
3062898308,0.502392147029794,0.63732219146304,-0.123184379336822
"Slc25a14",282.659514748677,-0.0200203852669815,0.178535505795926,-0.1
12136715762666,0.910715006035462,0.945169934243248,-0.0011289603974528
8
"Rbmx2",455.859267320245,0.131484209899589,0.209115800991028,0.6287626
72531044,0.529504440868896,0.661855010172503,0.0542646858714267
"Enox2",633.639144559809,-0.291275097097288,0.211311569594083,-1.37841
528344524,0.168075107339727,0.291845083525691,-0.358714647550908
"Igsf1",10.2249267113897,-0.986209385931256,0.693699839626184,-1.42166
58698706,0.155123272648791,0.275088091869588,-1.27286485082311

"Stk26",398.162527420231,-0.810469405982644,0.260003313978977,-3.11715
029158504,0.00182608453233283,0.00931092997160711,-3.79021391374667
"Gpc4",4680.33438958269,1.01731958835063,0.250737250563521,4.057313327
25492,4.96404655078904e-05,0.000575782151381824,7.58898151740659
"3830403N18Rik",9.05122246206231,-2.22160917399529,0.800548909698797,-
2.77510736331046,0.00551834872623955,0.0213825046630005,-8.54249211881
543
"Slc9a9",346.403317001713,-1.23384671747987,0.283275627634353,-4.35564
022144713,1.32678548768471e-05,0.000211526275052137,-10.4397761395893
"Vgll1",3.29392779645023,-0.225438919641133,1.07940025081212,-0.208855
722862318,0.834560869870043,0.894228050817502,-0.0252028191143123
"Cd40lg",25.6709753417945,-2.02760105238904,0.677832576333433,-2.99130
068866982,0.00277791812067074,0.0127684694759166,-8.84191495822455
"Arhgef6",1236.59049708148,-0.87153571083101,0.206883801384135,-4.2126
8221581434,2.52355988277863e-05,0.000344568421529536,-6.94894410927034
"Rbmx",3217.79449449136,0.195124875102529,0.145462197247177,1.34141294
986052,0.179786415391327,0.307201430838673,0.230296449950155
"Fgf13",21.9758705110992,-0.485766766587036,0.393597824753793,-1.23417
035368754,0.217139432023238,0.352492269218975,-0.506521923215334
"F9",5.89361832073395,-0.793279192572644,0.636091810884792,-1.24711429
859348,0.212355590810881,0.346646959322388,-0.840438391346144
"Mcf2",6.77303823907837,0.0748532892990661,0.610103140312649,0.1226895
65670335,0.902352926827283,0.940206133024014,0.00461516468295214
"Cacna1f",11.062140212688,-0.762594107364166,0.64838753369661,-1.17613
937303273,0.239539186496501,0.377783309739447,-0.742335415430154
"Ccdc22",1769.43967743369,0.440784888387143,0.156998033230972,2.807582
2309103,0.00499149343122959,0.019870823860434,1.72721680204182
"Syp",692.461420345567,-0.294570432086916,0.152031749251264,-1.9375586
5822525,0.0526770903953465,0.120977374384978,-0.62217745051521
"Prickle3",787.212278688109,-0.429120595946685,0.181886275419783,-2.35
927969252381,0.0183104494102587,0.0536625796785246,-1.2551946335986
"Plp2",3006.10278743507,-0.464200053102238,0.179889340340241,-2.580475
59807743,0.00986643289613589,0.0334074657364515,-1.5778047834944
"Magix",353.996087081889,0.9466305262461,0.286650128254174,3.302390032
09766,0.000958646688427158,0.00568309307721275,4.89432560253439
"Gpkow",1663.58051043897,-0.135511850939054,0.122923015825488,-1.10241
235157651,0.270282444147521,0.412715725271232,-0.119927478496097
"Praf2",788.078574342997,-0.777979035922406,0.176227093415114,-4.41463
920697952,1.01178591717088e-05,0.000172747826733781,-6.7401596057675
"Ccdc120",754.632192230703,-0.227074797765803,0.266640127884644,-0.851
615244739314,0.394427675580439,0.538742614253408,-0.140449701416668
"Gripap1",3002.69037624224,0.295686709421849,0.147985530437305,1.99807
851854218,0.0457081482407203,0.108447003455532,0.656866154095536
"Otud5",5095.48946364476,-0.0421610661092595,0.174238892097773,-0.2419
72762806602,0.808801268301511,0.878121647011892,-0.00547967986784957
"Pim2",472.130916175714,0.0581429778001297,0.198267501405399,0.2932552
10198288,0.769327086414307,0.850807928113535,0.0093940956447292
"Slc35a2",1368.5951713115,-0.0054240155527919,0.154495898178058,-0.035
1078288599008,0.971993758734667,0.982933162585371,-9.33698414506512e-0
5

"Pqbp1",1443.85153442291,-0.111377316558901,0.110226350672109,-1.01044
183972139,0.312283650895196,0.457374760062674,-0.0871251487074149
"Timm17b",1582.26746499427,0.558909272568183,0.180427685941808,3.09769
129748992,0.00195034474741786,0.00980351170022,2.58496355729263
"Eras",0.163104059883235,0.4033283383319,4.78471990596623,0.0842950781
359167,0.932821825554686,NA,NA
"Hdac6",3788.08159791087,0.688128165380845,0.238906212674023,2.8803276
3015571,0.00397262111143098,0.0167151848145622,2.81543351989653
"Gata1",2.18761699429034,-0.669019297905822,1.31521970046494,-0.508674
936719179,0.610980093076054,0.730717650419977,-0.209890183289156
"Glod5",964.149640438143,2.09513828079679,0.689888737605588,3.03692199
421668,0.00239007298572299,0.0114406469888881,9.36648903532461
"Was",382.603973946214,-1.1073538475413,0.39748205001287,-2.7859216472
9311,0.00533757716878815,0.0209072208777343,-4.28286893845845
"Wdr13",3747.05150056654,-0.0834548997156315,0.155772090901845,-0.5357
50012935359,0.592131333506917,0.715061113632966,-0.0279897106773239
"Rbm3",8367.4019944936,0.0067892450166736,0.204434505116936,0.03320987
82091123,0.973507220797044,0.983749354293898,0.000111235911130205
"Ebp",2482.52952405719,0.982692107275704,0.190778557417494,5.150956798
17524,2.59160887806102e-07,9.44508222512782e-06,11.3697637495429
"Porcn",389.98726438044,0.0503350642325888,0.205717181745005,0.2446808
95419718,0.806703521720975,0.877006148119073,0.00660603806933093
"Slc38a5",0.568340198278774,-0.211842184148549,1.99760906363564,-0.106
047868927365,0.91554437237708,NA,NA
"Ftsj1",807.90732888511,0.205908923455943,0.169721654973631,1.21321538
779441,0.225047482440382,0.361051289463199,0.209766679574843
"Otc",3.43534500120076,2.23466352158494,1.00226008486513,2.22962438126
591,0.025772389840216,0.0701144212508066,5.93890162399654
"Rpgr",431.617356058373,0.319685733705059,0.149806416066844,2.13399226
881186,0.0328434136831294,0.0843050131639887,0.790683184023066
"Dynlt3",4593.93094681539,0.013860003467745,0.236944431634361,0.058494
7422994643,0.953354550362935,0.970983442487281,0.000408119761316209
"3830417A13Rik",2.74111205853951,-3.99169455697013,2.16510737607163,-1
.84364738723152,0.0652345452201339,0.142124751804153,-7.78799595770347
"Aff2",10.409510373296,-0.423187322666331,0.57146201566165,-0.74053447
3102918,0.458975751646112,0.598649428034514,-0.217128576673463
"F8",242.281497646669,-0.236320522856379,0.283770548483634,-0.83278734
9212911,0.404964670768567,0.548714464951044,-0.14183415970793
"Vbp1",1610.23129158156,0.00753725427081616,0.221493347604978,0.034029
2579994703,0.972853819697094,0.983343897172314,0.000126598552873519
"Fundc2",2748.20592754524,0.0820669692861726,0.261181428014093,0.31421
4413751289,0.753358196091657,0.839720296103966,0.0143359853458336
"Mtcp1",378.846790999096,-0.0565476528011361,0.2312715834881,-0.244507
569621262,0.80683774014842,0.877024383612742,-0.0074202103448148
"Brcc3",1363.13498519423,0.261620696406587,0.111039564580643,2.3561034
0687696,0.0184677759630658,0.0540424276944105,0.763405488316827
"Rab39b",47.8760511289025,0.172987601251363,0.297811539070322,0.580862
654923911,0.561333023501243,0.689516725650283,0.064310618707425
"Asb12",1.81582278093592,-4.6090211409563,1.86985949937196,-2.46490238
571634,0.013705058259201,0.0432326849923801,-14.4776658080517

"Msn",11659.5020346944,-0.775613768490715,0.227785642286372,-3.4050160
5239726,0.000661601646229549,0.004278576748983,-4.23030209260171
"Heph",685.740291482537,-0.908945694685679,0.273374072081832,-3.324915
51873871,0.00088445330599935,0.00536394107632203,-4.75201926423114
"Gpr165",2.85768724945636,-2.37734040712942,1.54072586950906,-1.543000
25343699,0.122830714427279,0.230505608267862,-3.48869970501362
"Ophn1",905.982327524567,-1.38702381544159,0.407426849356132,-3.404350
54202623,0.000663215829134098,0.00428191464783333,-7.56393328928618
"Stard8",2610.81585932611,0.443988808614862,0.194295934034983,2.285116
3140394,0.0223060084587637,0.0626599269455372,1.22986372224147
"Efnb1",2334.88536687087,-0.986930509023832,0.144380504455763,-6.83562
169798501,8.16501495703758e-12,3.46090133983301e-09,-19.2271203959271
"Awat2",40.0610778910906,0.315127377450006,0.300670285407674,1.0480828
7597402,0.294600425102415,0.438850873912972,0.25953752736099
"Igbp1",2864.33349390632,0.378464703495533,0.212300288944936,1.7826857
6729867,0.0746374694702013,0.157651541515544,0.699163625872053
"Magee2",3.04525397874006,-2.60158049064432,1.2590026674027,-2.066382
03347112,0.0387924210286092,0.0957799955952669,-6.10253107730939
"Pbdc1",876.782043084177,0.144805669952511,0.134447741255226,1.0770405
556879,0.28146214782971,0.425452634466881,0.123751165124797
"Magee1",325.18084368577,-0.731339917380522,0.213353068913555,-3.42783
87515337,0.000608406718668436,0.00400767371641796,-4.03666309178847
"Atrx",6886.50087606761,0.456214088077094,0.273018835109963,1.67099858
840635,0.0947219566302714,0.189443913260543,0.758986165608058
"Fgf16",9.34590168522448,-0.104369200192967,0.561619723166324,-0.18583
6066448219,0.852573320215344,0.90617957840098,-0.0102822221564391
"Cox7b",9353.93207062866,0.971123219189559,0.327282002361982,2.9672368
5439773,0.00300489378347186,0.0135529537335495,4.17694737974504
"Magt1",6810.75923440154,0.772746930659978,0.160489738590762,4.8149304
5876552,1.4725116033216e-06,3.72870535604734e-05,7.87959565284765
"Pgk2",0.117622969735108,-0.976778180958925,4.78226736430637,-0.204250
015013662,0.838158128732496,NA,NA
"Itm2a",166.148818658456,-1.99119932366673,0.589193366814106,-3.379534
52265351,0.000726086877784487,0.00459799998150111,-10.7169012952739
"2610002M06Rik",879.231070988922,-0.178710657338188,0.157596271945563,
-1.13397769586781,0.256803903983352,0.397678569360546,-0.1647911013118
96
"Hmgn5",3029.3215444208,0.875768513950806,0.232544538357622,3.76602486
618711,0.000165867279815609,0.00146803459911245,5.7133655876334
"Sh3bgrl",5912.61974894167,-0.988839520232875,0.23436828292864 3,-4.219
16953896847,2.4520383892645e-05,0.000336452107768327,-7.90780377595705
"Tnmd",4.79248130614867,-0.367304119400922,1.02864864381813,-0.3570744
21483284,0.721036092623336,0.815615346868353,-0.074861142846205
"Srpx2",363.857912064188,-0.536642753162168,0.304501873970642,-1.76236
272757358,0.0780080318422218,0.163162476384857,-0.972938026963486
"Sytl4",307.900651985022,-0.343190132979176,0.15562898028459,-2.205181
4022787,0.0274413676913841,0.073546637422214,-0.895669809882664
"Cstf2",1229.70799493653,-0.31881241743381,0.15631654675325,-2.0395308
3698212,0.0413970768183197,0.100480543234607,-0.73256435762798
"Nox1",4.00476454893547,-1.43884588296765,0.953464378309078,-1.5090714

6160968,0.131280521656399,0.241778370761922,-2.04277812691869
"Xkrx",91.5320888296348,-0.480280549222717,0.39651818935483,-1.2112446
8464908,0.22580164170922,0.361863846560386,-0.488199055850586
"Cenpi",96.2943100382589,-0.398970695864122,0.332626650181136,-1.19945
499149529,0.230351075766842,0.367123203801038,-0.399791690614114
"Btk",316.098682603274,-1.00238742060984,0.442388994223772,-2.26585071
893269,0.023460524803421,0.0650782212948342,-2.73868815587514
"Gla",440.981012543885,-0.436447867024396,0.148447668446837,-2.9400789
6244392,0.0032812861408842 4,0.0144551552586898,-1.84910049504215
"Serpina7",21.415727610702,2.00268997798074,0.615215185621411,3.255267
46541194,0.00113285623145923,0.00646995629788872,10.0947309152862
"Col4a6",473.966354834586,-1.2353470446884,0.263374787639336,-4.690452
93120495,2.72600935241923e-06,6.10939429350232e-05,-11.9866932290253
"Col4a5",2738.16205966216,0.0920003617700278,0.201018669572337,0.45767
0732602879,0.647189017906803,0.759641388686132,0.0252917103466668
"Acsl4",2166.97673550504,-0.425007086676951,0.167165276040336,-2.54243
642426312,0.0110082625654565,0.0365467569387485,-1.40641765208977
"Chrdl1",320.043012091701,-0.917025112392775,0.709490223250746,-1.2925
1268353092,0.196179656381172,0.326960504326873,-1.02515695132558
"Pak3",202.557413117449,-0.760091954939643,0.302437901057045,-2.513216
60507185,0.0119635854953677,0.0388841456890613,-2.4681467879243
"Dcx",8.02151556746095,-0.0439923508141702,1.05860672440215,-0.0415568
405150788,0.966851979776666,0.979820926114828,-0.000896803976506628
"Glt28d2",197.965692022667,0.509500934304979,0.220523963988235,2.31041
073763829,0.02086542518587,0.0593918336166561,1.43862609589621
"Il13ra2",1.45690344407661,-0.878538049872125,2.05821011164,-0.4268456
58226846,0.669491748220631,0.776320632881409,-0.222436747890025
"Lrch2",111.974795652362,-1.17558176868645,0.31516329196612,-3.7300719
9332347,0.000191425065650899,0.00163845738808658,-7.54018157817966
"Cdkl5",440.164884954621,0.397397503838339,0.13171755078254,3.01704291
855855,0.00255253669217788,0.0120215846434986,1.75691484024163
"Rs1",0.717786344179441,-3.09647403642828,2.21750127895659,-1.39637981
985078,0.162600148732861,NA,NA
"Phka2",1205.85080843373,-0.597406212265773,0.345812115912086,-1.72754
563757867,0.0840697163382241,0.172710075773121,-1.04912951487443
"Slc7a3",108.415768386077,-0.70318843210355,0.235521317496722,-2.98566
787744527,0.00282959776117215,0.0129334511204862,-3.05741985545104
"Adgrg2",719.258419120553,-1.6188279923038,0.293497043024619,-5.515653
49899627,3.47486298071504e-08,2.02247398202931e-06,-21.2247598503457
"Pdha1",19597.4018860193,-0.500053224462386,0.536751425428013,-0.93162
9057274765,0.351528260997358,0.496601799226669,-0.350020646767845
"Nlgn3",139.618269934137,-1.28893415760613,0.33818183749771,-3.8113642
2684099,0.000138201957604038,0.00128003966800037,-8.5854154006123
"Map3k15",947.545305638564,1.76196113633741,0.441305685913924,3.992609
18809253,6.53502250272123e-05,0.000711569972138382,12.7707591576339
"Il2rg",1200.30946925549,-1.35487021698515,0.346629297973631,-3.908700
80776675,9.27937987891345e-05,0.000932625509685848,-9.45361618013112
"Rps6ka3",3000.46605110953,-0.677918766135519,0.175921652804974,-3.853
52658599142,0.000116428614565463,0.0011204949354 2002,-4.60576978236162
"Zmym3",2472.58608663269,-0.124323472584377,0.178523692810751,-0.69639

7607661911,0.486179860844955,0.62312068144054,-0.0588068759350506
"Nono",8754.84898530795,-0.235425764587088,0.110694661004086,-2.126803
24824698,0.033436434309929,0.0854111877603819,-0.579212872223292
"Itgb1bp2",12.559394291975,0.0643618068263458,0.549164014481805,0.1171
99607274118,0.906701878125136,0.943048212742681,0.00377403930509554
"Taf1",2069.73667479147,-0.27838885971071,0.159438395184932,-1.7460590
9315512,0.0808006840254683,0.16754888259419,-0.497336167374971
"Rps4x",22519.5003864195,-0.667510440662574,0.261946850062229,-2.54826
672091685,0.0108259664714542,0.0360581985412392,-2.21788422102396
"Dmrtc1a",15.4140822676095,-0.115694187626338,0.687092797774197,-0.168
382186512685,0.866282621122149,0.915936150243461,-0.0101589471555466
"Cdx4",4.49936158041728,-2.77757019575294,1.64665295116856,-1.68679756
944644,0.0916422829939133,0.184724618403321,-4.69100806160348
"Chic1",658.872287396236,0.654712078440173,0.247457943841573,2.6457509
0326998,0.00815098141892198,0.0288978722602396,2.32029129821116
"Flna",19915.3680478651,-1.33480298961018,0.187215522304817,-7.1297666
6238658,1.0053921908213e-12,8.16797372359737e-10,-27.9315935716758
"Tsx",15.5266440812506,0.703549629593321,0.542336758064056,1.297256029
82312,0.194543107983145,0.325010840498275,0.790717136415195
"Zcchc13",6.58718922779573,0.017015046198192,0.59250529699043,0.028717
1208166714,0.977090201561746,0.985429304818234,0.000249744981660882
"Abcb7",1926.18357383701,0.465356923524655,0.170612774066749,2.7275620
2500167,0.0063804261062877,0.0239779863672454,1.73606942919583
"Mtm1",626.896658772769,0.161604946670077,0.167156386851441,0.96678894
3659703,0.333649552691862,0.478380688167213,0.119159156083647
"Gabre",351.71763915943,0.172112185531369,0.243485739251391,0.70686762
2147137,0.479648746534042,0.617021261458122,0.0831046779415346
"Gpm6b",1195.35597817658,-0.907182160097255,0.205402928641126,-4.41659
798182457,1.00266461584987e-05,0.000171490830524918,-7.86615905521538
"Gabra3",696.754688774011,-1.38049421172584,0.301580429850769,-4.57753
247586041,4.70492764962423e-06,9.4573896198323e-05,-12.7918375306282
"Gabrq",6.23553388716812,-1.28444529319525,0.859774356223412,-1.493933
01149062,0.135193129095298,0.24739517889255,-1.79407247672317
"Cetn2",2397.31550693375,0.666048155397275,0.17569302677451,3.79097661
202058,0.000150056085496897,0.00136337071529287,4.39444930828981
"Nsdhl",2204.24187950282,-0.407615705857082,0.397571747430205,-1.02526
326000728,0.305238986397732,0.449916416661541,-0.325559996106071
"Zfp185",869.571943284418,-0.229635416289388,0.138730584122817,-1.6552
6165510911,0.0978714334752192,0.194050967042894,-0.376518136665229
"Hccs",869.774692342928,-0.262515042566078,0.214411924848157,-1.224349
07830797,0.220820570107366,0.356597604435433,-0.270691675085234
"Rbbp7",4810.77059884634,-0.403793235962989,0.188635868631729,-2.14059
626566202,0.0323066102040178,0.0832229205651921,-1.00392385907422
"Amelx",0.118013966992314,0,4.78471990596623,0,1,NA,NA
"Arhgap6",476.067707886589,0.207050300216815,0.221088064920764,0.93650
6004026133,0.349012722730084,0.49398817470247,0.14602091979652
"Syap1",2946.04472436053,-0.480809164265747,0.293580623779259,-1.63774
147651946,0.101475634461232,0.199553395937983,-0.774907354780205
"Msl3",1522.30453988678,0.31526457972093,0.0969583748286803,3.25154562
747142,0.00114779350988844,0.00653612086910186,1.58591054491478

"Ctps2",1591.07773533384,0.252268948060244,0.120733160208454,2.0894752
3302366,0.036664965139616,0.0917353381614567,0.602632072861863
"Xlr4c",88.6563689576948,-1.19625131387817,0.496884613522331,-2.407503
23379536,0.0160620199547901,0.0486602338530914,-3.61613988538948
"Grpr",0.802980540204741,-0.875234030546609,2.60331058171526,-0.336200
389109907,0.736719759501976,NA,NA
"Zfp275",728.086248434569,-0.877461032761459,0.16972795460851,-5.16980
856091377,2.34333921522157e-07,8.76975585765647e-06,-10.2173331531242
"Ap1s2",942.293132183917,-0.837661860973065,0.231992684726395,-3.61072
532076163,0.000305341904731848,0.0023383960952325,-5.07479848745395
"Zrsr2",2103.33165949594,0.502482301871366,0.303894367234189,1.6534768
5264643,0.0982338532311113,0.194650779502054,0.822336506809912
"Haus7",670.462673141344,0.529113912446983,0.232221001688594,2.2784929
3819049,0.0226972247676132,0.0634571965183428,1.45897323693645
"Trex2",4.22792534721652,-1.50662260701908,1.22508424610105,-1.2298114
2873565,0.218767727479919,0.354250739174774,-1.56349808324653
"Car5b",3768.79260914014,-1.05987224654726,0.566974885160064,-1.869346
02270443,0.0615746890246231,0.136228672030194,-2.11277095050697
"Zfp92",5.73870590314144,-2.08777221283794,0.821639006979279,-2.540984
78176389,0.0110540730820391,0.036686351821889,-6.90081888176367
"Bgn",21702.6205205937,-1.51235949030279,0.425183353426291,-3.55695837
599384,0.000375173573809289,0.00275108474694754,-8.91650857591106
"Atp2b3",1.27392464675057,-1.30393807085489,1.45612337453127,-0.895485
982617804,0.370527355673966,NA,NA
"Bmx",122.546242001995,-0.251308798063164,0.260868966259442,-0.9633526
04438312,0.335370602639119,0.479895775117426,-0.184507485109088
"Abcd1",1079.51727623578,-0.717487294647437,0.161210806504567,-4.45061
537873461,8.56245754731601e-06,0.000150813728326619,-6.31350438733665
"Pir",207.571010991051,-1.76098861413075,0.391200000388011,-4.50150463
288373,6.74740941228373e-06,0.000124466403709279,-15.8338846206049
"Vegfd",646.733027460607,-0.886207512115865,0.24364079224045,-3.637352
77646471,0.000275454543595517,0.00216042344771737,-5.43905521324868
"Piga",1030.59251246605,0.846862151566103,0.22471966502871,3.768527117
81103,0.000164213647710972,0.00145736809425058,5.53096099889312
"Asb11",72.6403438901648,1.21660760201779,0.540623615472393,2.25037820
620309,0.0244249472564331,0.0671665624176732,3.28554583954267
"Dusp9",210.516439893285,2.46598832818048,0.41695529544289,5.914275115
65983,3.33340323609332e-09,3.57113715919492e-07,36.608118470607
"Asb9",819.423029287802,1.91939757386296,0.686997990025549,2.793891105
54978,0.00520780120595006,0.0205176213201888,7.45968336427479
"Plxnb3",56.694391088631,-0.6651982053318,0.371675197597789,-1.7897298
7606143,0.0734973470804103,0.155917422165619,-1.23622347387835
"Hcfc1",2943.68391894706,-0.307219365554832,0.137333604760259,-2.23702
979391781,0.0252843928342478,0.0690850264466397,-0.821018336854006
"Renbp",2794.44807080714,0.942973132798514,0.355802278957789,2.6502728
8628015,0.00804267806006851,0.028600426192817,3.35164114583506
"Naa10",1999.23615666025,0.190925677285993,0.1459812294677,1.30787826
61448,0.19091460230278,0.320762853571702,0.217092652854795
"Arhgap4",1752.61351791536,-0.631803953265158,0.326318594945483,-1.936
15675922701,0.0528485046218589,0.121299604840142,-1.33278520854645

"Avpr2",640.917158928732,1.84395816227652,0.682380208625788,2.70224449
503009,0.00688730963147985,0.0254709345821743,6.76772714252595
"L1cam",2606.30954352796,-0.025429860536515,0.218464031081317,-0.11640
2963044518,0.907333187189388,0.943127331475567,-0.00148901947884162
"Irak1",5678.57162986928,0.0161929321319754,0.131224864217867,0.123398
353113103,0.90179166113302,0.939872450757517,0.00100414159215962
"Mecp2",2998.08855482768,0.0294979662072836,0.160043290631211,0.184312
42003925,0.853768371058934,0.906934115985132,0.00288152272228085
"Opn1mw",0.480199823535145,-1.32081674326388,2.9737678606628,-0.444155
968169448,0.65692982432936,NA,NA
"Tktl1",5.20703032750787,-0.271685443022347,0.795597364902016,-0.34148
6102151441,0.732737665657455,0.825165704342674,-0.0522100793187388
"Plxna3",581.667624417154,-0.501188778769355,0.179338279258322,-2.7946
558918826,0.00519549845229447,0.0204732879593447,-1.94893986030476
"Fam3a",1341.80958196445,0.256405442591407,0.0884574552206192,2.898630
10361211,0.00374796773870718,0.0159929690089283,1.06039190995812
"G6pdx",3085.27272598534,-1.90532438408673,0.51749560442117,-3.6818175
223302,0.000231577141726475,0.00189957556137266,-11.9390003875856
"Mpp1",4875.74311089946,0.61523221905817,0.253280548628423,2.429054352
53439,0.0151382627139776,0.0464754914812055,1.88804319555968
"Dkc1",1774.98257681602,0.0283916972915629,0.147961287771582,0.1918859
83956784,0.847831516612452,0.902791705035749,0.0029034321249125
"Tceal6",2.58150053884819,-3.57834307878717,2.07683615551929,-1.722978
03525692,0.0848924971637416,0.174125174594849,-6.25487515563955
"Nxf7",1.43125132871094,2.17842423455348,1.56217627826202,1.3944804212
3457,0.163172577770687,0.285467826054092,2.73092914960213
"Morf4l2",6009.73375576131,-0.605684798515481,0.129062371183906,-4.692
96196063544,2.69277575498798e-06,6.05579488705372e-05,-5.8823561417916
3
"Plp1",714.811320163581,0.162845662800398,0.203672853005558,0.79954525
3072849,0.42397431777576,0.567112015095244,0.0923658054580639
"Zcchc18",142.9110739454,-0.519114602294844,0.346747275779336,-1.49709
785355372,0.134367798199917,0.246359649262375,-0.72726025809119
"Psmd10",1385.60208404496,-0.268404572483572,0.266486875774727,-1.0071
9621445998,0.31384049187714,0.45899054235077,-0.20901353162112
"Vsig1",101.960578436206,-1.39921757332178,0.547643973637817,-2.554976
6649074,0.0106194916992844,0.0355465835455189,-4.66906483383985
"Tsc22d3",5009.2546900496,0.324557127439794,0.2228121502334,1.45664016
571724,0.145215734283357,0.261490246311129,0.435347395149279
"Prps1",1030.45586675138,-0.280457632794823,0.323249887125754,-0.86761
8656540214,0.385603129660558,0.530031006141808,-0.178040050504449
"Rbm41",117.396602891773,-0.434622929971967,0.335504128666193,-1.29543
243387145,0.195171095435723,0.32578084229139,-0.487442822674647
"Morc4",494.901938296848,-0.574674563614841,0.405373697541991,-1.41764
146785896,0.156295466868757,0.276738922358066,-0.738273341234652
"Rnf128",6260.86451608509,0.868572425454899,0.228736767966026,3.797257
5776882,0.00014630574206469,0.00133991045503866,5.74573904460034
"Atp11a",40874.9774645197,1.14226889719112,0.364708537189339,3.1320048
2224552,0.0017361702362863l,0.00898642083013287,5.38241746271984
"Mcf2l",971.466062070826,0.147242164105584,0.315205029065228,0.4671313

92358319,0.640405875568294,0.754023777888321,0.0415710827692476
"F7",36.0646816866669,-1.39535655251318,0.461393082077591,-3.024225127
58551,0.00249270871263037,0.0118025338705359,-6.19460313875971
"F10",98.6998165758815,-1.66506617792781,0.496441429425053,-3.35400327
054933,0.000796514213428315,0.00494757379210745,-8.8395998418924
"Proz",204.962244001725,1.97258429449499,0.758975392195539,2.599009552
58742,0.00934931715048223,0.0321277733169021,6.78181268220808
"Cul4a",3903.26489852911,0.235544557979996,0.125302359456997,1.8798094
3855118,0.0601340536483063,0.13380049960455,0.473775595232609
"Lamp1",35012.5165659,0.351621424054643,0.290021211022274,1.2123989925
2623,0.225359686388819,0.361443050523088,0.357827818698134
"Adprhl1",38.7872572789095,-3.01831550166819,1.72309229757274,-1.75168
533102956,0.0798279306082827,0.166078210734133,-5.41877111291403
"Atp4b",8.84246098541648,-3.06162897378808,1.74997118556151,-1.7495310
7745354,0.0801992616937522,0.166601875613124,-5.4868931334507
"Grk1",56.5794050178139,0.185780091996362,0.26065836681897,0.712734044
426005,0.476010345605132,0.613595776716175,0.0907385103575113
"Gas6",16054.181759349,0.427336878063846,0.258657668239857,1.652133033
48722,0.0985074342055929,0.195014514381222,0.69855959929633
"1700029H14Rik",3.90226174520944,0.39119511129865,1.39520242047275,0.2
803859178843,0.779181439834651,0.857300145384565,0.0602312134809781
"Rasa3",1749.29271079983,-1.57969339117412,0.254651136547938,-6.203362
8146668,5.52692201885164e-10,9.97814125218234e-08,-25.4651059695123
"Coprs",603.946442903097,1.03023409004372,0.291898268075605,3.52942858
083994,0.00041645809410576,0.00299193069965884,5.9875519654799
"Myom2",1725.75722842413,-0.507296491331058,0.548761372206331,-0.92443
9140625804,0.355257724100469,0.500379256477071,-0.351246487574521
"Angpt2",534.700102232489,-0.309242153391329,0.307762047416595,-1.0048
0925438064,0.314988706830992,0.460255531009493,-0.2399636980359
"Agpat5",1189.68079857994,0.0287491443915407,0.208655468522771,0.13778
284650327,0.890412041555592,0.932247972198407,0.00201693748253864
"Nek3",331.343035456749,0.0485788795326245,0.20891736237656,0.23252677
0298125,0.816128897250225,0.882553161880229,0.00606926313021716
"Vps36",2469.60335935127,0.294031255175211,0.180492344649807,1.6290511
1430456,0.103302198791891,0.202207235422578,0.469997843941012
"Thsd1",341.190761930741,-0.500567543703713,0.185012918706283,-2.70558
157345969,0.0068184899543482,0.0252750793022588,-1.8410555778849
"Tpte",2.21797752975227,-1.34351426183314,1.74638967794891,-0.76930955
2614321,0.441709567690216,0.583420481697862,-0.723948783540375
"Slc25a15",4403.04959720868,1.37236909342726,0.404409783712254,3.39351
110853374,0.000690027598411117,0.0044140938693635,7.44229268758727
"Erlin2",4609.46927854778,0.2684619369692,0.179202049025799,1.49809635
787452,0.1341082172412,0.246010162740513,0.376486304052816
"Plpbp",5647.47836987805,0.915259103170007,0.160727802450934,5.6944666
026241,1.23758206992999e-08,9.35285860445541e-07,12.7060054408549
"Adgra2",2068.46725801538,-0.990448093775788,0.279175639459218,-3.5477
5973897419,0.000388522457695417,0.00282770096310013,-5.81223791003289
"Brf2",361.071288850109,-0.0852621594585583,0.173245138192865,-0.49214
7487357714,0.622615084058032,0.740499506463554,-0.0256153371479189
"Rab11fip1",1251.2025404163,-0.850519350752255,0.239002117000797,-3.55

862684994301,0.000372798732671552,0.00273995300565272,-5.0179064065621
5
"Adrb3",2416.41260819572,-3.64694801366593,1.53721856339681,-2.3724329
7765494,0.0176713688528612,0.0522450871710519,-10.7650954936674
"Eif4ebp1",1697.09462273048,-1.50257464780786,0.465352235243247,-3.228
89745446789,0.00124268445632253,0.00694663461278,-7.46704165463017
"Chrna6",0.407003317994645,-1.33195359927032,4.75681738672066,-0.28000
9403553868,0.779470290373038,NA,NA
"Chrnb3",0.592152097833988,-1.49407366301003,2.0070363435855,-0.744417
841652493,0.456623728424507,NA,NA
"Ggn",58.9845276200838,-0.0581104085810482,0.437136893639428,-0.132934
120698997,0.894245481952107,0.934655502926947,-0.00392694240171248
"Cd209a",701.956993322855,-1.68702340434246,0.462261660131704,-3.64949
886577616,0.000262752399957351,0.00208622378014661,-10.4129826721951
"Cd209d",14.1314120229634,-2.04210818033256,0.72748427192281,-2.807082
24101542,0.00499924766870892,0.0198848084546076,-8.00056986266357
"Tnfsf13b",1168.75515970487,-1.16959708840668,0.462573155950682,-2.528
45863051201,0.011456458703743,0.0376980897343412,-3.83410984853666
"Col4a1",17683.0119855687,-1.12793714784165,0.191183180307367,-5.89977
186292359,3.64004531854901e-09,3.81578514091767e-07,-16.6697258129726
"Col4a2",12165.5134381412,-0.936709422316997,0.19208995268195,-4.87641
02923589,1.08033784464507e-06,2.95019990124503e-05,-9.7708651930757
"Rab20",1500.29470649343,0.311212570352449,0.230963483949942,1.3474535
6724832,0.177834198283752,0.304666654787504,0.369887665679557
"Naxd",3095.88488567051,0.300054865585843,0.160136020727151,1.87374997
969441,0.0609648936550973,0.135186341008426,0.600440136143186
"Ptpn7",707.648519343856,-1.1557671042712,0.427589626923406,-2.7029820
9193516,0.00687204487063017,0.025420438415395,-4.24421000196861
"Ankrd10",4651.9886083172,0.332877050436683,0.216935405488313,1.534452
38543423,0.124918398523182,0.23347957576735,0.484223155095277
"Arhgef7",3130.20081863501,0.137498158499569,0.12650540125634,1.086895
55650635,0.277082970316513,0.42059663333837,0.119084545385037
"Tex29",2.34461524097061,-0.755362850176825,1.18602944313011,-0.636883
725401716,0.524200584308164,0.657078945216366,-0.317215465548614
"Leprotl1",1772.11415817159,-0.274402511817617,0.146816794044091,-1.86
901310305966,0.0616209914198993,0.136284719907577,-0.546886690188656
"Dctn6",1893.32290644513,-0.0688034818100033,0.101240343844723,-0.6796
05374666944,0.496754365592818,0.632474638913985,-0.0315199177864286
"Gpm6a",3312.73176239619,1.90738068069323,0.590998076397394,3.22738898
292231,0.00124925507119532,0.00697536522856995,9.47085193608978
"Spata4",0.357315357168995,-0.834499504116438,4.75553396496656,-0.1754
79664379246,0.86070274832574,NA,NA
"Asb5",20.7479270926893,0.541346519478691,0.408701552372624,1.32455214
89606,0.185319735766893,0.313714551830429,0.627567741680766
"Vegfc",313.699359392988,-0.206797821993288,0.237278963982803,-0.87153
8793503314,0.38346003367775,0.528164999763265,-0.132008675088238
"Aga",1265.73098406426,0.402343898637858,0.205279621608842,1.959979736
3448,0.049998158797766,0.116377737823147,0.865407131553018
"Dlc1",3986.50365510217,-1.61627232660329,0.414389426772062,-3.9003705
7459077,9.60455610411249e-05,0.000960357102143514,-11.2301920449787

"Eri1",727.884363014326,-0.459331756393592,0.176919165241535,-2.59628037339256,0.00942391315418067,0.0323313009777889,-1.57629772126078
"Tnks",2040.27605925471,-0.207252863375018,0.186205296623512,-1.11303420006393,0.265693741462631,0.407592177107662,-0.186006993158476
"Dusp4",307.27985726254,-1.71728075807564,0.543202914632895,-3.16139827643635,0.00157013649525242,0.00833679296210848,-8.22075465880373
"Saraf",7499.6515429661,0.0260722861791672,0.232010785932215,0.112375319424954,0.910525823603708,0.945135887384215,0.00147116956779435
"Mrps31",1088.65087694853,0.120439793207063,0.292797941644217,0.411340983241647,0.680822522084051,0.785257263795684,0.029115584157912
"Smim19",1184.79330040357,0.149249596523448,0.289420350477263,0.515684526942665,0.606074763210461,0.726745051278228,0.0476374188997741
"Dkk4",0.0925025812792845,0.894916885650085,4.78471990596623,0.187036420780699,0.851632077140017,NA,NA
"Polb",1724.08457807416,-0.100715857952243,0.158094323648648,-0.637061822510943,0.524084574997971,0.656975764646421,-0.0423115526676123
"Ikbkb",5287.4594144174,-0.0869691373419906,0.156040564764173,-0.557349542238767,0.577288632817947,0.702444693128079,-0.0307165086886935
"Plat",4981.41214309416,-0.907655809090962,0.428791551960568,-2.11677633325768,0.0342788348608015,0.0870952204998575,-2.21536388438124
"Ap3m2",649.908947047802,-0.55344672332597,0.165080406927943,-3.35258879975712,0.000800595544153508,0.00496659622014161,-2.9360462343003
"Kat6a",2447.36735869635,-0.356475845510857,0.135795459894515,-2.62509398906093,0.0086625051533079,0.0302419920285045,-1.24713924956541
"Ank1",56.512418134263,-0.731376482196362,0.563451395524573,-1.29802940946743,0.1942772292247,0.324655331109621,-0.822792091045757
"Gpat4",4790.80545379526,-0.53514104434851,0.297228852544049,-1.80043437831865,0.0717920765609922,0.152939357830681,-1.00484171867874
"Gins4",841.525900490231,-0.40669525403603,0.190856809139497,-2.13089203298362,0.0330980378915443,0.084813450231217,-1.00343966291706
"Sfrp1",5179.00220707673,-0.0951531257046666,0.280439434617096,-0.339300091068098,0.734383674050168,0.826236908055152,-0.0181622323058936
"Ido2",352.973548367368,2.04745150016044,0.713824334749744,2.86828481530858,0.00412703828817064,0.0171986715608152,8.31863806366019
"Ido1",13.156263620963,0.103668050680343,0.590629805451632,0.175521197412434,0.860670116190784,0.911682834002711,0.00958547131978195
"Adam18",0.198109811992685,-0.551446265386195,4.78471990596623,-0.115251524900878,0.908245759765151,NA,NA
"Adam3",2.0995018017944,-2.69990959055501,2.20250874749519,-1.22583376507607,0.220261238061805,0.356107265317502,-2.78771952353703
"Adam5",31.0606596282167,-0.807028347002692,0.423199636404844,-1.90696843186951,0.0565246755188979,0.127737380999939,-1.66068585061182
"Adam9",3483.67258832974,-0.16793725843569,0.161507952157729,-1.03980798587355,0.298429118182137,0.442761447748844,-0.136822539568501
"Tm2d2",2790.57682746508,0.775591085438904,0.250764145039685,3.09291061254456,0.00198203825738374,0.00992527796041369,3.57754607853889
"Plekha2",2234.5983831184,-0.83138687515754,0.191353604500684,-4.3447672560282,1.39423407178092e-05,0.000220476690442696,-7.00004352071635
"Slit2",1312.13161795559,0.781391298679417,0.35253845942503,2.21647107652828,0.0266592566187265,0.0719648602453177,2.05629162844127

"Tenm3",313.533332936049,-1.01649065965607,0.324309084631538,-3.134326
81298754,0.00172248897943758,0.00893458103779566,-4.79562608471292
"Dctd",175.637532502653,-1.47126509972225,0.306566991326177,-4.7991634
4991255,1.59329727767346e-06,3.9625140714384e-05,-14.9128118686528
"Wwc2",4828.88896310526,0.679230110624086,0.145741816462805,4.66050257
303766,3.15438217311058e-06,6.91057793385506e-05,6.50693764966856
"Fgfr1",4025.69429012114,-0.323198070960906,0.143863230931585,-2.24656
480233372,0.0246678537393445,0.0676951178338832,-0.87028876673029
"Rwdd4a",1380.85539807678,0.319025269164285,0.169840195304664,1.878384
96412471,0.0603285209270925,0.134095474354361,0.640986603666297
"Plpp5",1005.45006149105,-0.358738045742577,0.151426073201712,-2.36906
391453939,0.0178331713944405,0.0526117681720081,-1.05641734131182
"Star",119.049019088961,-0.142561332253033,0.24720266856249,-0.5766981
92952538,0.564143353365786,0.69210766399837,-0.0524645307231885
"Ash2l",1799.77670790143,-0.0335416539123903,0.0756938693008523,-0.443
122464503379,0.657677157173834,0.76708675064757,-0.0088937499945917
"Kcnu1",28.4216090130384,-0.38770721620433,0.378325151950517,-1.024798
94399154,0.305458064797721,0.450005390814202,-0.309582551473773
"Tti2",796.29615422324,0.0390930909159515,0.118267143133269,0.33054904
2449597,0.740985142502206,0.831284712802533,0.00722373580877118
"Mak16",1270.04457940112,0.0896995314378465,0.186100550410287,0.481994
981960506,0.629809510995831,0.745690177641287,0.0263218858663069
"Wrn",705.586143168753,-0.208117594628404,0.201353327100475,-1.0335940
1915695,0.301325996566548,0.446041169226735,-0.168022496216534
"Gsr",5038.29437823957,0.583178197271913,0.206909427086172,2.818519221
11329,0.00482457223385006,0.0193240569875942,2.30145705223963
"Gtf2e2",780.951403594072,-0.0869415676447824,0.214438201821478,-0.405
43880197784,0.685154994059219,0.78861582492129,-0.0206465349035483
"Rbpms",6025.35235753147,-0.111974330273197,0.182016552948685,-0.61518
7621450924,0.538430821660515,0.669664104148544,-0.0448993582706963
"Frg1",1605.94021716217,0.101812074335359,0.122233967801374,0.83292783
6399778,0.404885429355843,0.54864522215444,0.0611181213475587
"Asah1",4152.64441059848,-0.455576089117649,0.19134063306225,-2.380968
86075126,0.01726717151124,0.0514121255189118,-1.35209572341247
"Pcm1",1673.91526569634,-0.178288106182674,0.126028368583404,-1.414666
46110463,0.157166312370621,0.277826723354703,-0.228343857128953
"Fgl1",80.379615736217,0.432745095174856,0.421707272051278,1.026174135
12906,0.304809510752202,0.449525439879467,0.346006893279105
"Pdgfrl",207.769709386954,-2.32941247103919,0.436931230261729,-5.33130
229588724,9.75109414661756e-08,4.47357255695881e-06,-28.6921263623005
"Slc7a2",1878.45461152825,-1.64192123953992,0.395502795307701,-4.15147
821714511,3.30334709935019e-05,0.000419551884686937,-12.7681104862021
"Vps37a",2651.91772962993,0.273966851059904,0.195225999687732,1.403331
78725231,0.160517923504408,0.282139950643554,0.346664517243094
"Cnot7",3189.16323260141,0.179008258215668,0.100874215243683,1.7745690
2919378,0.0759690748111714,0.159737411912889,0.32834124117917
"Fgf20",2.80973310204484,-0.965557718705443,1.16963229852044,-0.825522
448317177,0.409075043415088,0.552785722954286,-0.572367971657764
"Msmo1",1908.30612762765,-0.861005653463571,0.269333020379282,-3.19680
688335607,0.00138957924488599,0.00758086628897231,-4.20353963648535

"Klhl2",2104.51881718992,-0.193059945939608,0.266983252206435,-0.72311
6316638213,0.469608425606199,0.607698276793034,-0.096158675345238
"Galnt7",870.129183206858,-0.0256975317647505,0.138135474558261,-0.186
031371354302,0.852420159939101,0.906143729064039,-0.00253268048246651
"Sap30",2198.05541401369,1.71662189380191,0.396547228137424,4.32892168
195161,1.498412038741e-05,0.000231139540596298,14.3723978508746
"Scrg1",1.26722959665138,2.33663757995395,1.77804958595086,1.314157714
39488,0.188793135237629,NA,NA
"Hpgd",1006.77986810085,-0.94968356953427,0.187885062861173,-5.0545985
6718884,4.3129697015883e-07,1.40861445965777e-05,-10.6082683223929
"Ednra",445.136291107325,-1.38293719852212,0.265552329248279,-5.207776
57057995,1.91116852858382e-07,7.40834273763962e-06,-16.3365040817896
"Tmem184c",1128.04328120136,-0.386974570873979,0.182106498106969,-2.12
499045831232,0.0335874090821916,0.0857182332833209,-0.950676449745607
"Nr3c2",672.257585280477,0.331227265883838,0.220259517044888,1.5038045
5894823,0.132631696994668,0.243783260558261,0.467519245200417
"Iqcm",0.58610431808208,2.44676019385114,2.94334291324521,0.8312861484
26872,0.405811997721974,NA,NA
"Sin3b",4708.49382751711,0.217758467439576,0.139299093448938,1.5632439
6697814,0.117995222249601,0.223561446256216,0.326217204200405
"Sorbs2",2162.68472455391,-0.452511842351684,0.240833769676385,-1.8789
3850168827,0.0602528909035124,0.134019035687507,-0.909446278311096
"Irf2",1789.12876244019,0.0888891013312169,0.245485320806581,0.3620953
83296881,0.717280753500683,0.812640333048014,0.0184415251497812
"Casp3",1142.64194456973,-0.712845437848971,0.216294043144142,-3.29572
385576018,0.000981684579537874,0.00578845366853226,-3.67250134867002
"Cenpu",64.4299948758171,-0.138449661026186,0.373173000108864,-0.37100
6640313732,0.710632585873663,0.807944418794408,-0.0295260531947201
"Cfap97",860.651969553247,0.00440029886286448,0.112100502777384,0.0392
531590300079,0.968688551488365,0.980658846153693,8.59406583116961e-05
"Slc25a4",11506.7320639359,0.635770350985301,0.191926509013909,3.31257
184977624,0.000924423777790923,0.00553265814823505,3.30415376323652
"Ufsp2",1546.87096227203,0.461478346819841,0.112027277549262,4.1193391
1914366,3.79960603376838e-05,0.000468002011664029,3.53817200446022
"Anxa10",0.635487422916337,-2.03453069699547,2.56551302738638,-0.79303
0740938452,0.427759912637714,NA,NA
"Pdlim3",429.755915271479,-1.1079290920945,0.261898268421989,-4.230379
60033141,2.33297286567593e-05,0.000324221703028862,-8.90119429238821
"Lrp2bp",19.6895807680598,0.0768949396200186,0.485683444325668,0.15832
3164024627,0.87420215608564,0.921461435796184,0.00628957176815092
"Tlr3",956.0242359854,-0.588010977313419,0.165046936895495,-3.56268942
867893,0.0003670748538373,0.00270901797884923,-3.47583230731968
"Cbr4",1200.76433212292,0.00743985121723567,0.394711058361347,0.018848
8542685437,0.984961680662857,0.990294583074532,7.25595405671896e-05
"Sh3rf1",1536.10505005865,0.167650812600658,0.213197994597023,0.786362
052408344,0.431655408462155,0.573740622058625,0.0931430801507655
"Nek1",895.226224539136,0.0884488446075951,0.139612677431533,0.6335301
79599706,0.526387479169184,0.658888301267302,0.0369009690765546
"F11",2.25999564434558,-3.06222470397352,1.65925115875185,-1.845546219
94481,0.0649581214908859,0.141704346926526,-5.9836252139826

"Mfap3l",3139.1526658371,1.21673947773865,0.322225813415825,3.77604595
001355,0.000159337609360009,0.00142708530422667,7.97222448262895
"Triml1",11.5063698760412,0.410349122544032,0.581035799723535,0.706237
245173675,0.480040611959841,0.617443884952371,0.197856841964791
"N4bp1",1839.44258720618,-0.255076271505421,0.121057426308754,-2.10706
834998172,0.0351116553257744,0.0885993342231072,-0.618210742611919
"Cbln1",3.13507617675997,2.40387479812191,1.99837439932355,1.202915128
88456,0.229009165206185,0.365552247498174,2.41912993662376
"Heatr3",936.177512751111,0.130539851323819,0.153992034201784,0.847705
220600992,0.396602158204367,0.541068351572121,0.0801788383637289
"Adcy7",2583.64748408578,-1.21699267936749,0.454742998477162,-2.676220
81800697,0.00744575701592243,0.0270047191771681,-4.39546557439206
"Brd7",2607.95954188045,0.223207177572154,0.144932745546752,1.54007416
840215,0.123542275531724,0.231510367797332,0.326581256367854
"Nkd1",849.366746901969,-1.75415822135703,0.323383009567974,-5.4243982
1962358,5.81501126975739e-08,3.05609406301158e-06,-22.2749552332896
"Snx20",601.842806221557,-1.38806256409493,0.38081813077268,-3.6449487
3518377,0.000267445107643454,0.00211805227816088,-8.54665981214096
"Sall1",1996.48416001037,0.937828025273211,0.50755531770312,1.84773559
169321,0.0646406024715493,0.14118544494122,1.83596814422288
"Rbl2",2081.50511918494,-0.0610163180827928,0.132580187403746,-0.46022
1993026604,0.645356886353638,0.758044543758034,-0.016902321266251\4
"Aktip",4589.82351623003,0.628283740406012,0.158470314351772,3.9646778
1979248,7.34951434437671e-05,0.000776698269304374,4.49879971902151
"Eif2ak3",1596.47410276793,-0.27514829885955,0.203925172796677,-1.3492
6107986622,0.177253122051121,0.303858042355615,-0.327755182266312
"Gins3",192.685648025833,-0.788255890802397,0.169508563403325,-4.65024
22944075,3.31545291011056e-06,7.15888159926198e-05,-7.52356486567616
"Setd6",1297.81354793903,0.528115330032403,0.163707274638635,3.2259735
0177722,0.00125544979727241,0.00700287489473674,2.62020962631946
"Got2",8278.35117126876,1.16449155136269,0.241485947882778,4.822191773
77541,1.41989333608749e-06,3.61424024373809e-05,11.9104705910837
"Cdh11",2369.38741226088,-1.89930520277088,0.436671199798826,-4.349508
74627384,1.36442842212778e-05,0.000216995313006913,-16.0218450713493
"Smad1",1524.29526226931,-0.503276891281208,0.146277122632997,-3.44057
14456382,0.00058048715828753,0.00386423305301818,-2.7962024071701
"1700011L22Rik",2.52538351917862,0.638682360012125,0.951813311770917,0
.671016418990621,0.502210069915062,0.637174124362563,0.287862001911269
"Lsm6",1864.80167803291,0.403386107736832,0.178626951212959,2.25826004
977219,0.0239294514160753,0.0660592444726938,1.0960820615318
"Slc10a7",590.498231988735,-0.280790701116063,0.193583144334047,-1.450
49147787129,0.146921513972543,0.263705777315835,-0.374271899955982
"Pou4f2",0.14728986473751,0,4.7847199059615,0,1,NA,NA
"Tnpo2",3711.13028976586,-0.376501912393986,0.161537324134649,-2.33074
25352679,0.0197669398565798,0.0569215467912558,-1.07908510231149
"Vps35",4994.55101845266,-0.0804644380071742,0.175473152154672,-0.4585
56975919874,0.646552338650283,0.759060543051736,-0.0221819323935517
"Orc6",602.729771671588,-0.439361041033262,0.164677997243802,-2.668000
87678258,0.00763040589690192,0.0274598844920254,-1.57951572135662
"Mylk3",114.810857724521,1.05487891758675,0.524920547272137,2.00959730

585639,0.0444738266373905,0.106322536162412,2.3642769220578
"Gpt2",2317.67402776224,-0.055949446911996,0.4053293727341,-0.13803452
3712397,0.89021313272295,0.93218988516821,-0.00392869997904771
"Dnaja2",5464.03426947984,0.187363509896552,0.168008790170109,1.115200
63745978,0.264764452973896,0.406550425585528,0.16863602298213
"Itfg1",5001.01609600631,0.510265848987684,0.220699262451947,2.3120414
7815757,0.0207753976511865,0.0591609014046527,1.44277383283572
"Rfx1",1733.04128105021,0.028012753105968,0.183926608553571,0.15230397
2363024,0.878947191980227,0.924555139904302,0.00219739282225306
"Tecr",10277.9530971806,-0.0294363459532799,0.355822100007782,-0.08272
77056502003,0.934068054454004,0.958802786298719,-0.00123838325927748
"Tbc1d9",1391.38376279113,-1.31592461762045,0.199825290559839,-6.58537
572463272,4.53736483492575e-11,1.30826427347887e-08,-23.886636639668
"Ucp1",4119.60964951187,-3.43217462821199,1.59100225111472,-2.15724058
580512,0.0309869236717286,0.0805899743565913,-8.64352345763214
"Zfp330",1740.92769586797,-0.267375586870269,0.0806944217418853,-3.313
43333403535,0.000921580741342027,0.00552212086499288,-1.39008387878959
"Il15",530.451996082919,1.02445765594383,0.250578915247148,4.088363360
23484,4.34427312277148e-05,0.000519979357567822,7.7466634255861
"Gab1",1534.85793054889,0.133955305931849,0.0708984952546865,1.8893956
1341388,0.0588388382406484,0.131564182111945,0.271696251706464
"Smarca5",3251.63188635116,-0.194443958717979,0.0975143698929202,-1.99
400312929773,0.0461517125380675,0.109240636651976,-0.430538232299938
"Hp",4328.86280402555,-3.80476946367397,0.954251718928808,-3.987175907
78354,6.68644504940392e-05,0.000725096249017116,-27.5054628238394
"Txnl4b",652.237728953341,0.126028235173832,0.224532918815799,0.561290
682179313,0.574599392577715,0.700352500873931,0.0448876653136545
"Ces1f",16290.783114813,1.86587670277332,0.579759380805093,3.218363970
55316,0.00128924106611667,0.00714339934843502,9.22035383873265
"Pmfbp1",5.7355624677888,-0.894295126345535,0.714007841950888,-1.25250
042618866,0.21038757311692,0.344196752864046,-0.953803159819494
"Zfp821",1365.1463422462,0.274341009998069,0.249436087042861,1.0998449
0716826,0.271399702279082,0.413903120456594,0.242002608340259
"Ist1",6847.79257150427,0.196829741458147,0.100344074753975,1.96154822
236129,0.049815105499104,0.116043797716845,0.423929455009496
"Dhodh",886.880541035789,-0.0754217976865044,0.123262978475756,-0.6118
77131472521,0.540619052939359,0.671229641760802,-0.0300664442861295
"Ap1g1",4776.69286038935,0.547483532398803,0.112519786486519,4.8656645
1549742,1.14072875973908e-06,3.05542200598079e-05,5.69164304347356
"Phlpp2",933.752543474169,0.00721587605284825,0.169430369258831,0.0425
890357461531,0.966029135768239,0.979458345928641,0.000149769608642403
"Irx3",1973.99096206833,1.09998562326765,0.277565363411282,3.962978700
76011,7.40203878222175e-05,0.000780556799219901,7.87095047982035
"Irx5",207.683144033955,0.380506886466297,0.433255416193009,0.87825073
2119612,0.379807666103377,0.524579575949823,0.245487117002993
"Irx6",1.91129202427765,1.04277126217388,1.36061057352099,0.7663994992
15559,0.443438630696929,0.584753579151138,0.559514304425358
"Mmp2",1932.67719311164,-1.6296355102266,0.346888443701014,-4.69786624
437451,2.62893495039217e-06,5.92589290898804e-05,-15.8622105669009
"Gnao1",436.75926011808,-1.2501702116452,0.347111212873721,-3.60164167

931969,0.000316214012963301,0.00240841438467127,-7.53700951930342
"St3gal2",975.163632071296,-0.613596038200688,0.173899461091582,-3.528
45278731223,0.000417996503374891,0.0030047013692073,-3.56437097368111
"Il34",2144.28535528479,-0.0822326804603064,0.376614873071649,-0.21834
6874592662,0.827158854758068,0.88967030836677,-0.00961335599583311
"Amfr",7484.50577974569,0.307806255066956,0.119860560846692,2.56803616
546277,0.0102276484956018,0.0344478649796586,1.03678635077795
"Cog4",2864.45819223174,-0.121979742505512,0.199702277319816,-0.610807
969456283,0.541326716807717,0.671766059337886,-0.048529045911184
"Nudt21",2367.014200288,-0.0392042150667945,0.184096551260413,-0.21295
4641455169,0.83136234200559,0.892330627692681,-0.00446608757857304
"Bbs2",1214.15719277327,0.707025999580967,0.203880795329804,3.46784010
939951,0.00052465935594197,0.00359697894590595,3.9789026208657
"Cenpn",99.6582998518575,-0.677626356585232,0.23491321365069,-2.884581
69744698,0.00391934049862008,0.0165481379476168,-2.77927209704644
"Cdyl2",101.196767319451,0.152122255034133,0.221963768694861,0.6853472
34499602,0.493124832081153,0.629415290384807,0.0704271278405811
"Mt3",26.7698339327954,1.52995588752953,0.580449647063294,2.6358115562
1358,0.00839363312186429,0.0295360149088811,5.38872635080117
"Mt2",5178.54515654448,0.215833862213212,0.274648079530991,0.785856076
553622,0.431951809638699,0.57399280204016\5,0.119817669843121
"Mt1",6373.16813588582,0.508354687544819,0.195809451995247,2.596170319
4857,0.00942693233618019,0.0323313009777889,1.7445306674649
"Slc12a3",27841.2307244557,2.07390828654646,1.0643861461707,1.94845479
153189,0.0513605692079298,0.118770599889683,4.41858889757005
"Nudt7",1465.83089165909,-1.16416708367616,0.596721480317503,-1.950938
79150576,0.0510643274085457,0.118290632744662,-2.48504350565188
"Herpud1",7414.86495460557,1.08158116870023,0.203588548363172,5.312583
52886751,1.08081857901948e-07,4.82238001229332e-06,13.2409794625591
"Cntnap4",0.265138276062047,-1.44228574196135,3.99054174850896,-0.3614
26050109675,0.717780977099895,NA,NA
"Fam192a",2156.86781558942,0.0816221387524394,0.106170567953925,0.7687
83103692741,0.442022080927329,0.583556539638503,0.0439628212990354
"Pllp",1655.1248923821,0.658742566592313,0.310726162622759,2.120009982
52621,0.0340052034556373,0.0866031161152059,1.61156064801143
"Arl2bp",2908.96029763209,-0.0911632354277243,0.0973261353169902,-0.93
6677852570706,0.348924291962276,0.493961072001776,-0.0642972992660492
"Cx3cl1",6803.09708465085,-0.740235648871235,0.432363005899479,-1.7120
6980886643,0.0868838043935594,0.177054809580437,-1.28156697034815
"Ccl22",349.113692957667,-1.63703667871804,0.515213362253279,-3.177395
61636848,0.00148604172971044,0.0079842495579758,-7.90735286764095
"Ccl17",357.16542544186,-1.52111122308125,0.63677555707333,-2.38877137
507036,0.016904818426846,0.0505072248983518,-4.54148881701463
"Ciapin1",2451.53287556042,0.459959801764666,0.11131787863969,4.131949
03986131,3.59700178111301e-05,0.000450734837584457,3.54382070135073
"Coq9",4534.94431565026,0.453725503946001,0.363351783999452,1.24872237
849446,0.211766632880423,0.345872491866517,0.481713603609198
"Polr2c",2270.58713322084,0.332874472406955,0.130528605170235,2.550203
24451351,0.0107660135451871,0.0359198720232817,1.1072951481664
"Adgrg1",12625.3516815096,0.21531410428955,0.207350506141107,1.0384064

5627855,0.299080865040491,0.443458456772585,0.175082912145818
"Drc7",2.61715308837803,-0.444664670231466,1.63444942177881,-0.2720577
73282166,0.785577597328548,0.861339031249622,-0.0663738002581615
"Katnb1",836.420916925498,0.224918354121709,0.128162395310935,1.754948
11544395,0.0792681829757041,0.165142753676705,0.405065586462641
"Kifc3",3726.11956042347,0.634659370420672,0.235265532197716,2.6976300
5439874,0.00698350002727251,0.0256980564619502,2.32370368470849
"Cngb1",32.5753947814255,-0.127854968832261,0.522918098234089,-0.24450
2856688326,0.806841389785257,0.877024383612742,-0.0167771908358689
"Mmp15",917.728557544383,-1.47092076059663,0.328956781938902,-4.471471
1517023,7.76833403358139e-06,0.000139344045066026,-13.0596649041213
"Tmem38a",573.643319417714,0.548702744377163,0.119287622810594,4.59982
965079618,4.2283657311624e-06,8.73556217361482e-05,5.12791420811823
"Usb1",770.513775769152,-0.313044059800805,0.122869207031016,-2.547782
86085774,0.0108409925800976,0.036083590530342,-1.03990640683205
"Cfap20",1438.3844226349,0.0574301413502886,0.115950795621596,0.495297
518593241,0.620390122254434,0.73848391964084,0.0174102891515721
"Tpm4",10444.0328608008,-1.06210706587682,0.206708306734106,-5.1381924
7352763,2.77393687138751e-07,1.00159668737618e-05,-12.2262649847479
"B3gnt3",534.292419534358,-1.18155790381849,0.204902624543503,-5.76643
616181519,8.09653580117856e-09,6.81317024540035e-07,-16.7772220184448
"Jak3",2380.86285836619,-0.822159896924653,0.199599822215964,-4.119041
2285793,3.80452078546865e-05,0.000468294357674069,-6.30301772502925
"Pgls",3145.46775546197,0.0598261412446436,0.267541653581722,0.2236143
06197631,0.823057430803787,0.886628385956477,0.00719884012300955
"Slc27a1",8912.24487065031,-1.02253334437249,0.547846476314749,-1.8664
5965353444,0.0619770838075212,0.136800259945805,-2.0340574542516
"1700018B08Rik",0.216724841653744,1.27584478035588,4.78429427823541,0.
266673558555944,0.789720514083426,NA,NA
"Map1lc3b",7289.59555346787,-0.212921122434176,0.200844646186708,-1.06
012844492873,0.289086168664326,0.433054503829519,-0.178191916714442
"Mvb12a",2615.02140684526,0.39728742256113,0.124882344495983,3.1812937
542497,0.00146618878484812,0.0079059040174139,1.92292891250092
"Mthfsd",1539.32935652998,-0.57754841861982,0.199734780022283,-2.89157
661252281,0.00383314122448037,0.0162511195105709,-2.37926469951174
"Cox4i1",45573.7246749315,0.556069580473585,0.387038257281446,1.436730
27152255,0.15079464999709,0.269174593577154,0.72978297218967
"Emc8",2031.90042876311,0.0226167533763477,0.214916763596374,0.1052349
43044383,0.916189384110091,0.948641103041716,0.00119246235688483
"Babam1",3232.87383685291,0.262748593023151,0.120570071619307,2.179219
01757482,0.0293154018180137,0.0772421222496799,0.67284931534619
"Gins2",205.28909410651,-0.928506055384624,0.398550311843672,-2.329708
51556835,0.0198215621690042,0.0570367206569132,-2.65929704975222
"Gse1",2248.08887193747,-0.292135626888398,0.175460019577547,-1.664969
76115567,0.095918788849146,0.191145447231997,-0.483402850585949
"Zdhhc7",1638.94844517125,-0.0397311330237628,0.177387132652012,-0.223
979791711866,0.822773027666767,0.886370986985283,-0.0047923571399393
"6430548M08Rik",699.592552339154,-0.540965555447792,0.245333790214393,
-2.20501853811108,0.0274527937002733,0.0735624528215277,-1.41171482238
678

"Crispld2",3747.77006271157,-1.04454183129071,0.242395857779198,-4.309
23960855055,1.63816809992992e-05,0.000246078594856961,-8.6799959453105
7
"Usp10",2843.14606522935,0.88615865592613,0.0810401897846512,10.934804
7960022,7.85749009412352e-28,5.10684472850735e-24,47.5259829416797
"Cotl1",6491.85200343364,-0.0860570349559447,0.296948696890212,-0.2898
0438660676,0.771965891792296,0.852502886167092,-0.0137328684127663
"Klhl36",651.536187461666,0.643124868746716,0.155741821546041,4.129429
47733916,3.63664527196994e-05,0.000454242854022229,4.95005402792921
"Dnaaf1",13.9765345161575,0.657261287544902,0.445161238625761,1.476456
68696113,0.139821334552708,0.254076083980307,0.900527830113925
"Taf1c",854.361571219137,0.0623231233271493,0.182350348603257,0.341776
825789057,0.732518850852484,0.824966935477487,0.0119917150393684
"Mast3",2455.92958714449,0.296705926609542,0.269016724871787,1.1029274
3602076,0.270058678551184,0.412406337279996,0.262806135181945
"Pik3r2",2478.30711474981,0.181585543911436,0.0862388309338571,2.10561
230880678,0.0352380425834584,0.0888638926510553,0.439554933536659
"Mbtps1",3881.70451244904,-0.253710259764449,0.095604110012287,-2.653
75892035029,0.00796006749935754,0.0283791179562028,-0.903741744443151
"Necab2",4.69393816703683,-1.21601639021547,0.79633533365394,-1.527015
49061731,0.126757159470306,0.235786214019464,-1.75693666595529
"Hsbp1",6702.83192808875,0.244549314092686,0.176930287202073,1.3821789
2459184,0.166916769578703,0.290168732780833,0.302579078189132
"Rab3a",497.118438601329,0.256020565651725,0.177005942905102,1.4463953
0995287,0.148066356577786,0.265300295952368,0.339711871406247
"Cdh13",946.750484767892,-1.17253425180916,0.308256101459559,-3.803766
5637674,0.000142512483783646,0.00131071151359128,-7.78232692288402
"Pde4c",5499.28943262276,1.64664966097089,0.546396993542903,3.01365066
138783,0.00258124840656808,0.0121238823926065,7.26597010099053
"Mphosph6",767.812095424696,-0.279649981021564,0.123742926848058,-2.25
992699659466,0.0238257820843318,0.0658476398412902,-0.760763078282896
"Hsd17b2",430.178728628654,0.994979908749918,0.449789167849322,2.21210
286923413,0.0269595579250686,0.0725778239020283,2.60992767913402
"Bco1",105.539104576398,1.23463995402142,0.38302073344507,3.2234285149
9572,0.00126665909538071,0.00705234695651999,6.11689378773558
"1700030J22Rik",83.1698275616551,0.504563579153233,0.245155700307953,2
.05813521170188,0.0395771557428646,0.0971027284100131,1.17663510436051
"Lsm4",3627.23800893125,0.559171731446419,0.273801288560295,2.04225383
447486,0.0411263588872327,0.100010195258576,1.2874834871542
"Comp",42.3861117597043,-0.697695141534837,0.338032938888586,-2.063985
66905574,0.0390190751814036,0.0961934413815915,-1.6335792883659
"Ntpcr",864.477656234127,-0.241077509662849,0.158448863562756,-1.52148
462439029,0.128138266896171,0.237645709614936,-0.346422192796182
"Map3k21",829.783436516967,0.0717421750263809,0.472132313953178,0.1519
53536977127,0.8792235821164,0.924555139904302,0.00562764180512505
"Mau2",3758.11723052097,0.174055872122648,0.202288220462634,0.86043503
5340077,0.389549280009431,0.533724394745548,0.109285450453128
"Pbx4",111.193335929598,0.315740687123896,0.272306404096572,1.15950518
377056,0.246250324777017,0.385313284046406,0.301121435736452
"Lpar2",609.923203498891,0.626163350068633,0.230075295857482,2.7215583

825936,0.00649749068927291,0.0243023352118633,2.32756365615458
"Atp13a1",3898.83440875759,0.149097489280633,0.126562930017982,1.17805
023366201,0.238776580409892,0.376854926730781,0.145503490622553
"Ints10",920.816207233915,-0.0854834294048687,0.251928914278368,-0.339
315674223936,0.734371936016567,0.826236908055152,-0.0163165412765788
"Dctn1",4772.03153269491,-0.214645316670232,0.124082503073805,-1.72985
966073363,0.0836553520208297,0.172058233512884,-0.377759077795997
"Pgr",128.155285635259,-0.41826912122298,0.524183184115452,-0.79794456
1935538,0.42490266207014,0.567909819198452,-0.236653591114802
"Cdh5",2958.12758268635,-1.72636032564556,0.438578824168283,-3.9362600
9855677,8.27612730593833e-05,0.000854450542227716,-12.1968253614172
"Bean1",35.157403882903,-1.77339872335434,0.579798642301671,-3.0586458
7111544,0.00222339804849943,0.0108488025900005,-8.02232480761423
"Cmtm3",2412.05414022883,-1.1731470326694,0.235851209948599,-4.9740980
0409787,6.55521414098927e-07,1.9543358611775e-05,-12.7202866813633
"Ces2g",329.612839147933,-0.506932737730593,0.385420963857359,-1.31527
027657531,0.188419083866116,0.317500241744148,-0.58159211808572
"Nae1",1800.51774298397,0.292202511407044,0.154587255256874,1.89021087
748468,0.0587297624858123,0.131425790077857,0.592970331850357
"Ciao2b",1015.56650006588,0.20956649697923,0.198312669658153,1.0567478
9886333,0.29062665824746,0.434291736856911,0.174786582529363
"Rrad",963.044684649946,-0.705361555660261,0.345798301146153,-2.039806
30709385,0.0413696213913892,0.100438908963928,-1.62106588240438
"Cdh16",24739.7155501467,1.34303607111623,0.57234490746098,2.346550224
53885,0.0189481123085684,0.055166536328575,3.89131101412925
"Car7",223.365801719832,-0.188852873230969,0.190496006104016,-0.991374
449750144,0.321502771576208,0.466800285962685,-0.143878272684848
"Ces2d-
ps",6.48611421409088,1.01635505972375,0.657665528203936,1.545398103043
93,0.122249998879864,0.229857226922597,1.49434370917764
"Cbfb",1834.2491842611,-0.512751549136309,0.154362638916952,-3.3217335
0192706,0.000894600939003352,0.00541201647802897,-2.67611879955842
"Ces2e",394.171788471038,0.858227460770789,0.317069897904601,2.7067453
1528379,0.00679463613097964,0.0252058248253122,3.15886079390226
"Tradd",919.990418971837,-0.0350090395073865,0.156064349592482,-0.2243
24386695634,0.822504901851469,0.886180061684254,-0.00423032145962511
"D230025D16Rik",1154.60791557808,-0.151129184664065,0.207730703701795,
-0.727524540045927,0.466904687891026,0.605469827505788,-0.075829151214
835
"Hsd11b2",6360.99039558508,1.71233476510888,0.609898836354243,2.807571
78574828,0.0049916553117451,0.019870823860434,6.70978850440704
"Tsnaxip1",9.63363088816805,-0.36894973949813,0.889340180617797,-0.414
857832288464,0.678245980338266,0.783299184068916,-0.0901124896099429
"Ctrl",67.1057812015624,-0.5039560463500 7,0.445049822073584,-1.1323587
1885541,0.257483652798359,0.39844573510019,-0.463732271868976
"Psmb10",2661.60812116711,-0.608756512583635,0.207996044172137,-2.9267
6966529148,0.00342502436798125,0.0149619073969528,-2.5581457247297
"Dpep3",20.976097362629,-0.809300741852142,0.702881833650345,-1.151403
69704694,0.249566193583574,0.389189925817206,-0.763727247412317
"Dus2",753.29638359519,-0.527160278818437,0.217572040081589,-2.4229229

0232124,0.0153961938068059,0.0470377603956598,-1.61142595846162
"Nfatc3",1677.84576150089,-0.215952399032428,0.122094293826525,-1.7687
3457607494,0.076938179304224,0.161218766262629,-0.394111625132959
"Pla2g15",1503.45745468369,-0.935916952946725,0.138570810682175,-6.754
06998298752,1.43754354090294e-11,5.19059702972695e-09,-17.853942176374
6
"Slc7a6",1010.29624882618,-1.43520019243076,0.328668565388895,-4.36670
96387285,1.26132224328979e-05,0.000202914695541784,-12.2031123929249
"Smpd3",68.6348875589075,-1.68005622740192,0.391950432912107,-4.286399
72386168,1.81592114865919e-05,0.00026742319151478,-13.8212818859439
"Zfp90",819.368715553566,-0.104381736404505,0.280735419443757,-0.37181
5343469392,0.710030338979968,0.807543923291356,-0.0223124135902757
"Has3",116.012192763364,-0.220423892713567,0.226388506446045,-0.973653
195446561,0.330228764075705,0.474837790704137,-0.164167753509528
"Vps4a",2325.41667996698,0.0157226941079717,0.128379086831416,0.122470
836146532,0.902526141482671,0.94028611844131,0.000968063079261903
"Cog8",2097.293844865,0.274236640535327,0.123060334692647,2.2284730593
3513,0.0258489859151237,0.0702835765406612,0.728157824822169
"Nip7",1077.55935283305,-0.309171167411892,0.145295465588699,-2.127878
98203988,0.0333471186341597,0.0852276994532503,-0.761311982754819
"Mtmr2",2043.66676598135,-0.00420190498290016,0.182970004288557,-0.022
9649936296304,0.981678196616964,0.988267321232836,-4.95910917864717e-0
5
"Tmed6",319.217811540579,1.22721543124297,0.643993314149513,1.90563380
749332,0.0566977326934243,0.128054256000972,2.52229738514245
"Terf2",1865.97486289036,0.0541910881134205,0.101885920462579,0.531880
046500872,0.59480907788969,0.717545728595398,0.0179870301069535
"Cep57",1379.06196109456,0.355274485460072,0.142276950694734,2.4970628
3221054,0.0125226765885518,0.0402251116528057,1.1415906468876
"Cyb5b",16464.2279621314,-0.42988013027868,0.387411245182663,-1.109622
23121839,0.267161847351053,0.409200447733765,-0.38411945520924
"Maml2",1527.02239603371,-0.0355280267085045,0.227577605975823,-0.1561
13895987986,0.875943245245694,0.922498725062145,-0.0028660205161169
"Mre11a",563.479091413038,-0.421743981635083,0.158476783756721,-2.6612
3511367133,0.00778545720608417,0.0278763732154262,-1.50983324260097
"Wwp2",3167.86291602975,0.353555490501671,0.147149520255842,2.40269550
241795,0.0162747326432695,0.0491519109477181,1.06520596604573
"Ankrd49",695.992339359364,0.175167108443333,0.218335487016863,0.80228
4185849294,0.422388588062592,0.565602135158603,0.0998214972914708
"Gpr83",5.03808645243978,-0.880932933335543,1.00448343653124,-0.877000
955214999,0.380486119241305,0.525330573075128,-0.567080800251303
"Izumo1r",59.4673933985351,-1.34189413935987,0.651001323947462,-2.0612
771280142,0.0392766089642186,0.0965962817336446,-3.13629515101255
"Panx1",392.347033710156,-1.22819345892636,0.358410781353108,-3.426775
98673109,0.000610792723497463,0.00401796104006529,-6.77591961980012
"Med17",1172.95462750638,0.0131094765384365,0.0950405230232117,0.13793
5652303121,0.89029127324842,0.932221644691354,0.000920084368333762
"Hephl1",73.9381745340836,-3.89638775197134,1.96839371354014,-1.979475
81582331,0.0477624598848899,0.112426991345262,-8.51536542001426
"Vstm5",490.776371201829,1.08616243465241,0.338746799040435,3.20641386

938317,0.00134400532854688,0.00739013420643179,5.33046095645889
"4931406C07Rik",22448.5833119665,1.35879857465082,0.476619656141499,2.85090754680798,0.00435946431566866,0.0178917955734058,5.46700787553483
"Taf1d",3291.28976076243,0.338734497417398,0.264496495262371,1.28067669509717,0.200307248779366,0.331825890968571,0.373673223937052
"Kars",2890.36325676048,-0.1252286426643,0.15349419400557,-0.815852635180167,0.414584424770115,0.557910629730671,-0.0730779873110534
"Adat1",610.332612673661,0.216532745887312,0.296940465873588,0.729212656315738,0.465871590507504,0.604684081461543,0.108926610893871
"Gabarapl2",4329.28380600423,0.449136274331968,0.233764668083695,1.92131804183156,0.0546916239564474,0.124402389629353,0.936105044021424
"Tmem231",611.01180808393,0.447775097897515,0.200483486942358,2.23347620657784,0.0255175576700141,0.0695683602203607,1.19352007919557
"Chst5",5.33530470008527,-1.70937471702205,0.875389879286773,-1.9527010278149,0.0508550318428879,0.117931899485327,-3.6540413495759
"Tmem170",944.626560448452,0.307670524850718,0.129756727485421,2.37113351124924,0.0177336239301875,0.0523735533763701,0.907429145967196
"Cfdp1",3576.52808268516,0.160376210984696,0.0904004709289881,1.77406388856841,0.0760525831153928,0.159809598618809,0.294093434198445
"Bcar1",4305.34319549666,0.279578702812836,0.17794151721163,1.57118308978071,0.116140126817388,0.22084066634342,0.422251574234707
"Ctrb1",3.08384837992003,1.9626405714453,1.16884630277421,1.67912630325051,0.0931274309287008,0.186849006817021,3.29224013768709
"Ldhd",28175.5302739435,1.59229956025223,0.516238803393679,3.08442439774903,0.00203946437042361,0.0101390811561753,7.31081716622412
"Wdr59",923.285598885727,0.0759740255404404,0.130022757798279,0.58431329120329,0.559009551569548,0.687686419683884,0.0284463716846446
"Aars",5147.58014274159,0.143689225207872,0.114041430550908,1.25997389294174,0.207678780318436,0.340765872140779,0.1546900190491
"Cdh15",1.38206462817117,-1.54889640402143,1.43544237570237,-1.07903767524178,0.280570932825107,0.424388906329339,-1.3275668713296
"Bmper",89.9970758529944,-3.27715052571784,0.744411737108265,-4.40233591486376,1.07091577289908e-05,0.000180629028892615,-28.2459740319051
"Tbx20",0.806691348952345,-1.31408783681861,3.53636372854992,-0.371592951881523,0.71019593751512,NA,NA
"Glb1l3",4.13465753938315,-2.3888648649916,1.1379356170165,-2.09929703339004,0.0357907247852738,0.0900216140174495,-5.75168303056492
"Afg3l1",3435.73141570665,0.421722095926164,0.173856038669235,2.42569714088849,0.0152790148549502,0.0467676972750109,1.29155029476783
"Acad8",2758.27910579948,0.386222568885279,0.156172389878723,2.47305281801223,0.0133964342306256,0.0424307463659418,1.22041775476869
"Dbndd1",19.5764635899536,0.161582666869702,0.360577797879896,0.448121508921975,0.654065505795651,0.764383195397004,0.0434150086553305
"Ccsap",33.8637596461823,-0.768615587778157,0.400423119793849,-1.91950851432821,0.0549200097959263,0.124790857825541,-1.5995782593198
"Acta1",23.2683613276855,-3.7761593245199,0.914570815825663,-4.1288867512253,3.64523880942672e-05,0.000454601090549151,-29.0616629509288
"Abcb10",1595.85557853464,0.295577022313847,0.139595594576093,2.11738073261853,0.0342275480758381,0.08704580959819,0.721598318329812
"Urb2",496.637825118246,-0.15431766199398,0.176379154810311,-0.8749200

67283132,0.381617399416273,0.526519675475409,-0.0989896225307049
"Cog2",1579.58630651821,0.167688059453944,0.122834880851059,1.36515017
796347,0.172205853758823,0.29748114969341,0.203305744191367
"Agt",2335.22981909789,1.65126584908262,0.302724515688494,5.4546816115
2098,4.90606705927638e-08,2.67202501457461e-06,21.1901562305116
"Capn9",5.1775134714164,-3.60559143740653,1.61411396067461,-2.23378988
426542,0.0254969014526118,0.0695395977791335,-9.61199815456466
"Arv1",208.051538120941,0.0563045232881034,0.14835174095907,0.37953395
7094834,0.70429139303835,0.80365361648451,0.012307432772593
"2310022B05Rik",3506.11347808593,-0.7429074207327,0.260772909034807,-2
.84886732859793,0.00438751723564669,0.01797222921442,-2.98569108830761
"2810004N23Rik",1416.75979793053,0.288337897561768,0.14381690594399,2.
00489570867324,0.0449741975871808,0.107135846616353,0.644048152181346
"Gnpat",3809.8749310908,-0.603598366522116,0.362263982719878,-1.666183
76464119,0.0956768186384057,0.19078611268272,-0.999922446662231
"Sprtn",1591.24009959937,-0.311965318203414,0.182503949711958,-1.70936
200940189,0.0873839135329385,0.177906603953768,-0.538607049740926
"Egln1",3580.46235869887,-0.145602157213204,0.301886788939107,-0.48230
7151382411,0.629587767813018,0.745550640027836,-0.04275348458382
"Vps26b",1644.43030529954,-0.376817545030664,0.219205315944016,-1.7190
1645454119,0.085611375813457,0.175102339831195,-0.656561116467926
"Jam3",1352.47569023853,-0.201751900919369,0.126792242773341,-1.591200
66422383,0.111564423455437,0.214123745303092,-0.31094026816808
"Spata19",0.091991025955798,0,4.78471990596463,0,1,NA,NA
"Snx19",2525.67739246696,0.375144863806379,0.0885643585944452,4.235844
64179599,2.27694171705832e-05,0.000317750641796379,3.02150812891993
"Adamts8",83.168435974087,-0.592146615044273,0.322623102183153,-1.8354
1293551914,0.0664445285182669,0.144172648236053,-1.14683625664959
"St14",4969.24237320787,0.239900505395066,0.296280048292917,0.80970860
7708503,0.418107669669464,0.561218734903979,0.138577218922762
"Aplp2",20193.2368607981,-0.430466889142122,0.288034678336024,-1.49449
674472855,0.135045832122806,0.247195234203011,-0.601610551508814
"Trpc6",62.4454723286836,-0.232955419948009,0.328495244477174,-0.70915
9185298943,0.478225698275104,0.615596795733016,-0.113021369543898
"1700128F08Rik",21.0745833169617,0.28396470477601,0.40888995664057,0.6
94477084027832,0.48738306712294,0.624170446158495,0.133841594591365
"Birc3",2184.69186953402,-0.83703314408339,0.280094518180853,-2.988395
30855413,0.00280446561643668,0.0128582046395005,-3.64425245184471
"Dcun1d5",2278.04545056378,-0.0576130645296988,0.0926768346588363,-0.6
21655505842265,0.53416841466414,0.66636057256055,-0.0233865460417907
"Pdgfd",799.604390836972,-0.458145278948082,0.158055808211206,-2.89862
982027129,0.00374797112532346,0.0159929690089283,-1.89470840584386
"Sesn3",2180.23558745689,-0.570412403779274,0.182986947120511,-3.11723
001424585,0.00182559075702285,0.00931084713063865,-2.66757650149015
"Usp2",5755.42058676978,2.58459010977525,0.435902978392276,5.929278389
67261,3.04268850741832e-09,3.33294047851924e-07,38.5471990339309
"Thy1",2064.71352020785,-1.48374167789735,0.348875434035337,-4.2529267
8459861,2.10994576578777e-05,0.000299633813119665,-12.0375212323242
"Nectin1",1738.59389797419,-0.349200525734332,0.261243109356204,-1.336
68798612483,0.181324505093183,0.309179291675284,-0.409903428412342

"Trim29",318.034019527826,-3.08294502561564,0.780780548112024,-3.94854
230560737,7.86285218147827e-05,0.000819400811515036,-21.9102966957326
"Oaf",1776.55298529015,0.0482332654682234,0.212394769643805,0.22709252
939285,0.820351792991238,0.884740265487204,0.00590670175436355
"Pou2f3",22.2292521470425,-2.73626038013184,0.6921756599818,-3.953233
35978535,7.71021747866204e-05,0.000807378197631324,-19.4868757680219
"Grik4",29.2490559943705,-0.7986295629898,0.599626588762557,-1.3318781
6877488,0.182900225934416,0.311073696146945,-0.932580049670874
"Sc5d",2834.48525158309,-1.5962570396414,0.577357582306161,-2.76476327
420073,0.00569641184647032,0.0219070292273133,-6.09921470313633
"Ubash3b",1238.03383000088,0.20174199956488,0.40660188496115,0.4961659
23048183,0.619777351298159,0.737889649851103,0.0613216982167086
"Crtam",31.2202411039462,-0.303440594340735,0.59246505475004,-0.512166
231422301,0.608534674443822,0.728552688340024,-0.0960982196280672
"Jhy",0.255546039209667,-0.466787283766769,4.78471990596179,-0.0975579
120493865,0.922283346338399,NA,NA
"Clmp",865.212285158007,-0.76860669736934,0.293509804617037,-2.6186746
9256162,0.00882720814411359,0.0307281450191073,-2.67673144407218
"Rexo2",3315.62694212191,-0.213631209748143,0.204127673330147,-1.04655
682526017,0.295304023451513,0.439697430260222,-0.17553402436903
"Nxpe2",59.1373512676419,-0.895795175091506,0.29318762234073,-3.055364
91595287,0.00224786837620443,0.0109462881116968,-4.04429562516528
"Cul5",1687.54993569998,0.00295611190622372,0.12643377542659,0.0233807
137076287,0.981346589032964,0.98808619920146,3.54300029770982e-05
"Barx2",39.763941686466,0.169521217551819,0.450210836140251,0.37653739
9688464,0.70651741682396,0.805124289493487,0.0367451846000708
"Kcnj5",61.1364781521368,-0.117695986352498,0.574273274407886,-0.20494
7699288027,0.837612987472796,0.896267041474293,-0.0128896963960748
"Ets1",3182.50704283677,-1.22519995641648,0.17752962749346,-6.90138301
823235,5.14986708963086e-12,2.44907581740543e-09,-24.2927196455167
"Kirrel3",167.487269118862,-0.919451603516361,0.461004323583134,-1.994
45331959138,0.0461025365264882,0.109170179401684,-2.03644493404739
"St3gal4",1200.65910718021,-1.09398488626866,0.153758430317751,-7.1149
5873109444,1.11946327485969e-12,8.73091797328572e-10,-22.8194093159315
"Dcps",816.701504141871,-0.219909021440196,0.209735268795663,-1.048507
59103584,0.294404806726202,0.438592979947089,-0.181245375695009
"Tirap",743.378282444264,-0.214978780040168,0.195819943269468,-1.09783
90477028,0.272274777408985,0.414880713497999,-0.189130642484314
"Srpr",4647.76639300674,0.0327781613403901,0.138928716434346,0.2359351
05294666,0.813483027008682,0.881280812346665,0.00414246997702857
"Rpusd4",755.711485741413,-0.04407280893221,0.0932257042167487,-0.4727
53832245034,0.636388815515639,0.750971925493187,-0.0126218800053957
"Abhd12",2687.28902445796,-0.80343871517728,0.228305465055052,-3.51913
921545218,0.000432949455351382,0.00308089360600046,-4.645912966711
"Acat1",20974.4896064915,1.28295197253041,0.38059518248549,3.370909647
7576,0.000749204253548739,0.00470466490682554,6.87559714662956
"Rdx",11019.1539373858,0.831642002854925,0.309076314031346,2.690733534
40659,0.00712951154308849,0.0261319254363876,3.03100037726803
"Fdx1",3137.53197521824,0.952741622855748,0.375007591641886,2.54059289
48915,0.0110664691327283,0.0367116771805219,3.14848750793296

"Pou2af1",172.450588540785,-2.05112044670644,0.902271126913435,-2.2732
8613930392,NA,NA,NA
"Btg4",0.399383866395143,-1.46337485789666,4.76251468280563,-0.3072693
64056757,0.758638352407435,NA,NA
"4833427G06Rik",0.587769145515544,-1.60379334043389,2.35583556125647,-
0.680774739463783,0.496014036111927,NA,NA
"Ppp2r1b",2418.04605676358,-0.998453965195622,0.428091518948337,-2.332
33764511022,0.0196829356058851,0.0567299155127194,-2.86501732180188
"Alg9",1008.87294312243,-0.326176311531272,0.352193328516013,-0.926128
592229827,0.35437915657067,0.499507322693192,-0.226409748487017
"Cryab",6639.54554373761,0.0760880208296933,0.329950948051924,0.230604
037596883,0.81762243060162,0.883107669311625,0.00945836110737468
"2310030G06Rik",1690.43041101096,1.34896406906908,0.342477342243556,3.
93884179383098,8.18758940853349e-05,0.000847804664299448,9.54103440675
156
"Dixdc1",709.024318072197,-0.349084132345518,0.397238584124022,-0.8787
77002781104,0.379522197805267,0.524371018481228,-0.225353284464367
"Tex12",0.329417671968437,0,4.78471990595562,0,1,NA,NA
"Bco2",637.966489772057,1.39760934849901,0.296868619458763,4.707837935
33675,2.50358016780704e-06,5.70266426540908e-05,13.657427350785
"Pts",3725.428632869,0.33940822236969,0.174398300192477,1.946167032563
38,0.0516346786261265,0.119202342055509,0.721898506687815
"Plet1",2615.4406977511,-1.17200846495616,0.437018060728756,-2.6818307
3029467,0.00732204980620841,0.026670152647385,-4.24760505338953
"Cadm1",2130.69368034475,0.152845374259362,0.217039939401279,0.7042269
48648238,0.481291451106918,0.618818692460186,0.0733570602964104
"Bud13",577.92174801835,0.0130112440358787,0.164448329342415,0.0791205
607737528,0.936936729645835,0.960836919719902,0.000519806783939545
"Zpr1",1597.21346744407,-0.100070514842113,0.102301588484901,-0.978191
212122606,0.327979762004956,0.472637279873301,-0.0749955492122714
"Apoa5",8.28118798830356,1.52330831103965,0.739264748732851,2.06057209
362505,0.0393438813723529,0.09673732648148,3.55807644828881
"Apoa4",510.8918264003,2.21189457970693,0.982720321579147,2.2507874632
6585,0.0243990019531643,0.0671179091538936,5.97500049010294
"Apoc3",407.8811023042,1.53344124077266,0.457580426288916,3.3511950089
5008,0.000804636181020812,0.00498321423858778,8.12981501302003
"Apoa1",106.353890665303,1.38866656380336,0.61720106794281,2.249941932
91324,0.0244526316091251,0.0672231981751915,3.74903433643983
"Tagln",11431.2646263799,-1.56142900337514,0.28432522049849,-5.4917006
6812074,3.98081673967513e-08,2.26182406888254e-06,-20.297544876994
"Bace1",1802.05819167943,0.00423861914744281,0.161281400082898,0.02628
08925596142,0.979033295176193,0.986669833155619,5.68814681052778e-05
"Dscaml1",47.1780060594494,-1.18366298259664,0.493992322747053,-2.3961
1615017493,0.016569838652212,0.0497886753029481,-3.55095074431778
"Il10ra",1738.40558170102,-1.07883324676569,0.381976094727277,-2.82434
754859713,0.00473769806959585,0.019050451012782,-4.27289654455799
"Tmprss4",120.407651403869,-1.0029981908683,0.365053005121798,-2.74754
125235501,0.00600439549702131,0.0228590508866755,-3.78973652877758
"Mpzl2",926.083541259925,-1.48922206697697,0.498872892854294,-2.985173
35439175,0.00283417654933955,0.0129405701951478,-6.47422603361505

"Cd3e",278.896255395836,-1.57728803541531,0.49475126233937,-3.18804246
796117,0.00143239497274477,0.00776663992730184,-7.66233538625356
"Cd3d",349.781093658353,-1.314984013545,0.51263574102213,-2.5651430603
0066,0.0103133272707468,0.03470047543141,-4.41966375606723
"Arcn1",6498.7886113311,0.0649526274037632,0.105163503055315,0.6176346
88049511,0.536816173400471,0.66829534854823,0.026177536856295
"Ddx6",9700.99931970751,-0.285618050199625,0.158220800602693,-1.805186
48061224,0.0710455057800737,0.151824339291964,-0.538398904200609
"Treh",2870.61399430282,2.02248365959884,0.833267551993594,2.427171986
66868,0.0152170402036354,0.0466512342595098,6.19902546171894
"Pate4",0.226700398769636,0.922820795098962,4.7498124416823,0.19428573
3685121,0.845952155671611,NA,NA
"Ddx25",5.53000438998649,-1.04118214480835,0.790607769935355,-1.316938
92268916,0.187859098338393,0.316775637758539,-1.19690293212046
"Pus3",561.6956282008,0.242887917670632,0.112886554628757,2.1516106897
7082,0.0314280294897314,0.0814438754639531,0.609124312482764
"Pdzd3",3641.78401793966,1.85949918495728,0.719007231962192,2.58620372
966577,0.0097039554537965,0.0330205451026395,6.34205502573596
"Pate6",0.767637256814793,-1.6675313986699,2.82668050197336,-0.5899256
73420029,0.555240481100858,NA,NA
"Nlrx1",858.854313315641,0.109677752422691,0.224807770609204,0.4878734
93542844,0.625639450528658,0.742564703306792,0.0326450641891097
"Acrv1",0.126850756555144,-0.0644028076075004,4.78471990596623,-0.0134
600998330528,0.989260718437602,NA,NA
"Trappc4",1897.15146297583,-0.693291498481595,0.37836659376484,-1.8323
2745677459,0.0669026487871643,0.144924769031455,-1.33912061141412
"Chek1",120.926006525712,-0.578238518659821,0.245402094292708,-2.35629
007293685,0.0184584974773171,0.0540233839406679,-1.68749560988075
"Slc37a4",6384.59105648955,1.56224006859405,0.43007974328434,3.6324428
0389462,0.000280750817711567,0.00218963177749605,9.56719237347476
"Hyou1",5098.348673882,-0.422975348001382,0.183959960792267,-2.2992793
9851551,0.0214890789007556,0.0608296037849695,-1.18419507418051
"Stt3a",4383.76490339433,0.426607086737667,0.146835290199646,2.9053443
9069536,0.00366849210089325,0.015744719124635,1.77095278127676
"Fez1",48.5348451165496,-1.66849609044226,0.471686034302532,-3.5373022
92423,0.000404236706479478,0.00291702712914021,-9.7393291743338
"Hinfp",928.933366937475,-0.345981556767895,0.213111531575223,-1.62347
646892009,0.104487589609679,0.203946922211882,-0.550074521651027
"C2cd2l",2352.08998739617,0.844359901389816,0.215087473625967,3.925658
18527461,8.64929070555263e-05,0.000883078642617973,5.93762013702893
"Tmem218",363.663391047847,-0.269307240588666,0.191578305147666,-1.405
7293198261,0.159804517110375,0.281128749063708,-0.341735411990173
"Slc37a2",408.808314303463,-0.839303627540764,0.28451549306374,-2.9499
3997867362,0.00317835668564484,0.014103686539987,-3.57654054435684
"Dpagt1",1164.76271287826,-0.147419406062073,0.0942013908583846,-1.564
93874154886,0.117597273609273,0.222986542164932,-0.22122402631297
"Robo4",1613.57821800399,-0.487364493942978,0.267637063933618,-1.82099
028729391,0.0686083333037126,0.147913012246328,-0.931417360072045
"Hmbs",1544.66741450007,0.173949519767637,0.25850436616053,0.672907472
903632,0.501006157695584,0.636143400804148,0.078682806860964

"Vps11",2538.94098736589,0.0869060302579304,0.107813385477887,0.806078
297910005,0.420197704078856,0.563209928791471,0.04989299922286
"Robo3",734.661802052646,1.14517205910812,0.594770291235638,1.92540225
358099,0.0541790538877629,0.12356804219249,2.39451277721566
"Abcg4",41.9435024393071,-1.02910220476723,0.379798735965824,-2.709598
81462016,0.00673646354647899,0.0250281185650243,-3.79507715470184
"Mcam",2546.7808062878,-1.78434173543961,0.35368181227984,-5.045048044
56215,4.53407423236402e-07,1.46852789672149e-05,-19.8573414134236
"Pin1",1427.23786631783,-0.386616083615138,0.27905003318468,-1.3854722
7249125,0.165908105195104,0.288790057808662,-0.480198555111021
"Olfm2",161.870991488041,-1.66395617355514,0.315122484398286,-5.280347
34408877,1.28939214593587e-07,5.54979427405244e-06,-20.1367811218566
"Icam5",17.5989933512173,-0.397606411083139,0.501442449704523,-0.79292
5312401114,0.427821338743717,0.570849861403586,-0.222909701994402
"Tyk2",2105.1448943559,-0.377287167036267,0.14674409699815,-2.57105515
488656,0.0101389178123097,0.0341962669960931,-1.27358519716081
"Pde4a",1251.76441653622,-0.46313539368916,0.219364072635211,-2.111263
65464287,0.0347496561971054,0.0879134288998521,-1.12606865187662
"Ilf3",1906.70133111881,-0.290759510145244,0.130371657140018,-2.230235
59356134,0.0257318064372399,0.0700340186755305,-0.77306387639125
"Bmp5",32.6477285940595,-1.67410928533216,0.732666550587997,-2.2849538
9067321,0.0223155315760418,0.0626686208655714,-4.637105970057
"Tmed1",953.048894790334,0.355660692545936,0.145488517986836,2.4445962
9850733,0.0145014361472666,0.0451531462790487,1.10172846156919
"Scg3",4.13870443648813,0.363085641272433,1.08072572084856,0.335964652
518259,0.736897521925503,0.827985240736671,0.0685360274752392
"Yipf2",1550.60893502415,0.244097020849824,0.111835001282932,2.1826531
7699851,0.0290613605240434,0.0767594699942832,0.626616217208078
"Lysmd2",161.778349675466,-1.46488722721874,0.29336743944066,-4.993353
14788757,5.93399093875596e-07,1.79939277331048e-05,-16.0045905441971
"Carm1",1885.6964904788,-0.0767320473074241,0.187549933548806,-0.40912
8629669474,0.682445266976022,0.786285990397594,-0.0184490465648536
"Tmod2",117.810182075525,-1.37362211212786,0.431298497174675,-3.184852
53513774,0.001448277884919,0.00782939529637445,-6.66188867436409
"Smarca4",7356.66543693777,-0.0662429999900433,0.224631047215427,-0.29
4896902325859,0.768072638362669,0.849936453053083,-0.010770694030711
"Bcl2l10",14.9413754889472,-2.52565762730315,1.09810507238102,-2.30001
453488122,0.0214473965968577,0.0607468534058006,-7.07446802495506
"Gnb5",844.997588178836,-0.546678579003424,0.274677872425897,-1.990253
43459695,0.0465630247406526,0.110113505930048,-1.20610610148454
"Ldlr",1800.55700156817,-0.989974462914206,0.214673677743291,-4.611531
66667237,3.99712749917205e-06,8.38923487393506e-05,-9.29187669173685
"Kank2",3831.43782864858,-0.894186792239853,0.149944997380744,-5.96343
19774558,2.46994302452767e-09,2.95453675412518e-07,-13.4438782539236
"Dock6",4348.23535360521,-0.310256624162691,0.240307807201689,-1.29108
008506064,0.196675908916863,0.327643974017842,-0.346192936958732
"Polr2m",5825.52104838418,-0.556436407152358,0.289206064571444,-1.9240
13619759,0.0543528763114956,0.123831376708811,-1.16230356658864
"Rab27a",813.314241969694,0.403028155548443,0.185575123667831,2.171778
99484977,0.0298723367842677,0.0784023182958205,1.02607009939213

"Aqp9",135.530139844723,-1.20584997478167,0.422898256856747,-2.8513949
9922351,0.00435278597466717,0.0178750254705267,-4.85276326906485
"Lipc",16.1363514826591,-1.22608297477769,0.715201121070735,-1.7143191
4556021,0.0864701288125516,0.176414625048355,-2.12715370493563
"Sltm",6664.49858257081,0.493210386375592,0.229979149419499,2.14458740
116453,0.0319858505168221,0.0825165537677953,1.2304397452883
"Rsl24d1",1821.61843439856,-0.0338984939854591,0.16516356429217 2,-0.20
524196199528,0.837383086277821,0.896217774522174,-0.00371432048184251
"Nedd4",16162.4117106797,-0.940243706548785,0.196211324207106,-4.79199
511214925,1.65130927141304e-06,4.04291786585898e-05,-9.51146658768973
"Rnf111",1891.64679179155,-0.11743599499631,0.10994328730789,-1.068150
66087151,0.285452556666975,0.429654308441239,-0.0992069136949801
"Ccnb2",192.815907534906,-0.352719909035257,0.383507460577711,-0.91972
1114431319,0.357718515882216,0.502869186926565,-0.242468556995748
"Myo1e",2378.17308142158,-0.379986586040459,0.234980428798388,-1.61709
88706744,0.105856940618451,0.205679982877783,-0.600923630434125
"Mns1",127.581623610569,-0.666823184081388,0.325674123492536,-2.047516
63082827,0.0406073856570465,0.0990368448677633,-1.54187079461442
"Fam81a",1056.5417476198,0.907739527758958,0.357410176121624,2.5397696
7754287,0.0110925491285492,0.0367702351085434,2.99832410625232
"Gcnt3",62.9334715850551,-3.63832191007418,1.13602872525063,-3.2026671
7663455,0.00136161244005158,0.00746799419300304,-17.8173311218718
"Tcf12",2363.28122667393,-0.501853187451268,0.122458460971736,-4.09815
037253406,4.16464704697143e-05,0.000503112070147763,-3.81142320290899
"Anxa2",13266.0134608994,-1.33386939312341,0.258317042501353,-5.163691
02172741,2.42127245692644e-07,9.02676297612844e-06,-15.4933154765237
"Cgnl1",12201.4854630613,0.560883991008953,0.229122880078809,2.4479615
0788621,0.0143667002931143,0.0448482104250948,1.74124841683651
"Ice2",802.221783227064,0.29554850217152,0.155408827347844,1.901748486
33282,0.0572040437580384,0.128943911871047,0.605394979105719
"Rora",3188.24673126595,0.566387228462465,0.107810886973454,5.25352535
687724,1.49215121477764e-07,6.24065568814102e-06,6.78782543201112
"Rp9",2924.41772810946,0.518739537260674,0.17239474858223,3.0090216873
0066,0.00262090406376533,0.012249915808011,2.28361397699332
"Itga11",57.9209904244001,-1.53305762005505,0.327479491771824,-4.68138
512051689,2.84943060275961e-06,6.34950833058364e-05,-14.8163090835189
"Fem1b",1767.52129515362,-0.253669687671251,0.121854310537072,-2.08174
570561521,0.0373657034257694,0.0930184034661418,-0.602454833462188
"Cln6",1382.80938700832,0.351361035062646,0.18802953347959,1.868648124
36917,0.0616717856973418,0.136350660792241,0.700095760021128
"Calml4",6577.30546701327,2.02873661765725,0.602706366769643,3.3660447
7654812,0.00076254329266185,0.0047699933013541,10.8444297710878
"Anp32a",12377.0986934726,-0.230883186315236,0.223658917899529,-1.0323
0038168633,0.30193142233898,0.446722005254968,-0.186050009831915
"Irak1bp1",567.94135028746,0.272871020023869,0.205397237793792,1.32850
384433026,0.184011714420147,0.312231392216994,0.317624793667151
"Glce",636.292545137194,-0.65732050826144,0.283155772731401,-2.3214095
2635625,0.0202647518810805,0.0580635021568418,-1.87087746408525
"Phip",4998.38162048188,0.423514077154452,0.314259256169616,1.34765824
344047,0.177768328200298,0.304580567948102,0.503481850987571

"Kif23",701.59514333937,0.481868369732584,0.267121879395626,1.80392699
700538,0.0712427521122628,0.152129140366323,0.907370433234755
"Ankk1",5.78407745425451,-1.23553057030131,1.02025838491992,-1.2109977
1250425,0.225896281397512,0.361946396145015,-1.25561925343548
"Lca5",167.599021985181,-0.84575149368401,0.242652114199728,-3.4854486
9049799,0.000491312437744648,0.0034120534056235 2,-4.80424145815078
"Drd2",1.46068008793563,2.18200521989334,3.27661018623176,0.6659337229
25624,0.505453464309382,0.640372426712432,0.972531411445824
"Sh3bgrl2",2118.33315562676,-0.346874840831494,0.203837101608296,-1.70
17257314523,0.0888067944674655,0.180163862087883,-0.594504799694115
"Elovl4",73.7679861821854,-2.46834130781768,0.597878383938142,-4.12850
066857921,3.65136378343892e-05,0.000454915597760333,-18.9948703818613
"Bckdhb",2345.85184935357,0.751458607106415,0.336866757116135,2.230729
48348937,0.0256990534167992,0.0699734874348206,1.99860851284724
"Zw10",1133.17023582568,0.175183639308415,0.132137034852619,1.32577244
149461,0.184915083931825,0.313186787539992,0.203380409763446
"Tent5a",2444.03308865072,-0.56244139495574,0.172056377069924,-3.26893
663887369,0.00107952472508203,0.00623846268217232,-2.85552705017521
"Usp28",706.619239100101,-0.0651416138220607,0.178909171553441,-0.3641
04384679924,0.715780062734737,0.811600352593737,-0.0135981318722262
"Tmprss5",29.145954787849,-1.1511785465919,0.522102728331273,-2.204888
96940121,0.0274618867416812,0.0735624528215277,-3.00413917498357
"Htr3a",40.1768629582066,1.21277985692257,0.906433279464628,1.33796925
201034,0.180906460284385,0.308682433064229,1.42555269016319
"Nnmt",602.739055264136,-2.54496950800871,0.529004535439107,-4.8108651
9588386,1.50278351059216e-06,3.79550166962771e-05,-25.9055216943754
"Paqr5",3355.44250472112,0.568140160099981,0.239253558610965,2.3746361
9516647,0.0175662543305705,0.0520368925763389,1.67931001429399
"Idh3a",6753.22272687925,-0.598269215172171,0.506620088698737,-1.18090
3064284,0.237641233900058,0.375762612811882,-0.585584522148097
"Tle3",5078.01109194529,-0.0557685555784819,0.12079375485051,-0.461684
096561937,0.644307877364233,0.757337532725332,-0.0155006604923637
"Acsbg1",473.844418814727,-1.80398887472909,0.292484913462774,-6.16780
145468491,6.92460138705164e-10,1.18434980565555e-07,-28.7716412565025
"Dnaja4",1379.38038496976,0.443917226141934,0.167750075938746,2.646301
19335405,0.00813773234889045,0.028864745377236,1.57374618993602
"Imp3",1940.24205130986,0.300197120253078,0.167695970995476,1.79012720
74162,0.0734334638335912,0.155825765895617,0.558071484217871
"Thsd4",861.118743338942,-0.649943988265488,0.241346086756309,-2.69299
575974375,0.00708131696097599,0.0259923791613535,-2.37226429038135
"Ptpn9",1725.2417948066,-0.426124183009759,0.12656517409543,-3.3668359
8829852,0.000760358951786334,0.00475938325583626,-2.27875752744317
"Crabp1",4.2208320667243,-0.107497629781519,1.28634152184964,-0.083568
4986883945,0.933399513702563,0.958469755538897,-0.00455975608831309
"Nr2e3",5.38616489156916,2.62218513489904,1.15847096908418,2.263487998
2983,0.0236056236195714,0.0653781888259095,7.15218454140147
"Ireb2",4220.51971254664,0.209358992487714,0.116338521930113,1.7995672
3718289,0.0719289982313981,0.15312497079548,0.392862335399099
"Pkm",25960.2292314466,-1.60662458595885,0.493007398974936,-3.25882449
086841,0.00111874855061901,0.00641192217518209,-8.11280537464897

"Man2c1",4109.00973388706,0.0801054009171186,0.146311917165653,0.54749
7445655258,0.584037024873843,0.707961076219471,0.0276656950216842
"Celf6",3.66000660355344,-0.525315713228482,0.944912700438257,-0.55594
1001729405,0.578251187578318,0.703264823814998,-0.184922558155945
"Neil1",784.681628240594,-0.051399085433063,0.174474812283553,-0.29459
3155082639,0.768304691699799,0.849989526276341,-0.00835395768660948
"Commd4",2567.48492922727,0.0744429980066035,0.135187158149867,0.55066
619511357,0.581862522271544,0.706159309053315,0.0258997922308704
"1700017B05Rik",1304.96103025956,-0.34995075095966,0.162215118727439,-
2.15732512299092,0.0309803408367677,0.0805850942630228,-0.881330522614
882
"Psma4",7719.13432875858,0.696155992412919,0.136497587262282,5.1001340
4907475,3.39413014310951e-07,1.18278652441857e-05,7.89792617698702
"Chrna3",2.42254554390399,-0.0785595123745368,1.75920578591162,-0.0446
56238061329,0.964381315842794,0.978482952401665,-0.00170881979420161
"Fam219b",1287.03050532417,-0.441707730365902,0.169926239782742,-2.599
40860770323,0.00933845413106838,0.0321074199695947,-1.51888629247038
"Mpi",1406.85906932534,0.319761546245616,0.0906490546435617,3.52746697
142006,0.000419556101648035,0.00300975160777534,1.85650283419696
"Ube2q2",1938.41632442437,-0.0435590623284504,0.209737595114927,-0.207
683616781159,0.835476010679805,0.894913544813209,-0.00483628268745556
"Ulk3",1312.08148876616,-0.0903730570041553,0.130781225022645,-0.69102
4701661165,0.489550017933754,0.62612722474937,-0.042312818391014
"Fbxo22",3048.44264141443,0.568265068964509,0.139926898141126,4.061156
76480854,4.88301681291196e-05,0.000569491712762559,4.24537554200805
"Cyp1a2",3.53268455876917,2.82031630455403,1.27188059353015,2.21743795
675516,0.0265931783610034,0.0718561241245626,7.42614506553145
"Nrg4",351.572312840516,-2.4938848181148,1.02783491398832,-2.426347640
24289,0.0152516528130943,0.0467330534674629,-7.63952629552467
"Csk",3644.85966895112,-0.478942030135602,0.133302196838295,-3.5929042
5435819,0.000327012769888367,0.00247615339311976,-2.87415457859468
"Tmem266",12.1161880961632,-0.708027755784771,0.582109099845678,-1.216
31452930812,0.223865128841208,0.359919295794712,-0.723516182271383
"Etfa",12899.2869999935,0.356219831754113,0.535179420342223,0.66560823
943179,0.505661538540286,0.640469577657431,0.158715048582936
"Cyp1a1",123.467479384863,-1.11246965464322,0.553169720991658,-2.01108
197435122,0.0443167988180478,0.106114324371153,-2.49553446375448
"Clk3",4945.11685241491,0.196154793067475,0.163510823886656,1.19964408
719173,0.230277596570983,0.367078381195377,0.196582387172281
"Isl2",1.81082726137536,0.452930009900215,2.01411280173145,0.224878174
40555,0.822074048756174,0.885911667642064,0.0548670497015733
"Rcn2",2056.83571878441,-0.486141735817284,0.165349090845944,-2.940093
18908334,0.00328113548066725,0.0144551552586898,-2.05963871581974
"Pstpip1",938.859542496779,-1.09235065851875,0.350123669654365,-3.1198
9949036321,0.00180912758805049,0.00925108043844964,-5.11549444277207
"Cyp11a1",7.02437660907392,-0.61382170797214,0.789005555859096,-0.7779
68803152203,0.436587404847384,0.578575492402249,-0.335874794905567
"Tspan3",8392.2118529222,0.697717285446335,0.174518841684816,3.9979481
7975256,6.3893934273585e-05,0.00069858672145787,5.06992862920959
"Stra6",1035.92052171033,-2.06156500691958,0.641074883250242,-3.215794

38031868,0.00130084000632293,0.0071892796041056,-10.1741620374992
"Tmem30a",8926.00700453762,0.379708279417363,0.104422693090916,3.63626
208229248,0.000276622896218366,0.00216776444928953,2.32915293183878
"Hmg20a",2347.28729716701,-0.103282893558916,0.12804475035567,-0.80661
5603310776,0.419887981314091,0.562872180107401,-0.0593569588430749
"Cox7a2",8837.97334878912,0.661139643911721,0.437916610034996,1.509738
67800741,0.131110117796461,0.241559289207569,0.939241643261859
"Col12a1",1670.20017613708,-1.79380142326062,0.357529302965482,-5.0172
1511602591,5.24258567278244e-07,1.63828482480969e-05,-19.7663922706096
"Stoml1",1759.67201704884,-0.158303688868056,0.187193117613066,-0.8456
70454590505,0.397736609712145,0.542209017230218,-0.0968982754585769
"Loxl1",2364.39532912822,-1.38407581409465,0.327327678585023,-4.228410
56423261,2.35348034352015e-05,0.000326606119131359,-11.109638349387
"Nptn",4969.88490048009,0.280320093943887,0.221758674445032,1.26407724
363166,0.206202303565945,0.339250486306313,0.303030659952029
"Hcn4",29.8713998752989,-1.65158763436518,0.744167854219411,-2.2193751
3828462,0.0264612136123473,0.0716385369360662,-4.35378670340619
"Neo1",1997.11910489877,-0.264136649668422,0.128336086295632,-2.058163
5087419,0.0395744402418854,0.0971027284100131,-0.615962917635903
"Mto1",786.191055057233,0.193287029040493,0.20694722528063,0.933991885
024727,0.350308078994232,0.495306507749544,0.135799311023285
"Impg1",0.616810550281104,1.11885979253143,2.63717983823622,0.42426374
4288192,0.671373477114436,NA,NA
"Cgas",271.441324619714,-0.792671534720797,0.315397180155095,-2.513248
64201704,0.0119624990303624,0.0388841456890613,-2.57393817890825
"Ooep",6.72124953599769,-2.29060903920176,0.896142687250797,-2.5560762
4967507,0.0105859919118819,0.0354587992265716,-7.64922281308964
"Gsta4",6913.29187553164,-2.27402691580699,0.685926181003367,-3.315264
78910684,0.000915563546107882,0.00550236700945189,-11.8307999339225
"Elovl5",4491.26107275705,-1.59447009133989,0.458900532648441,-3.47454
399788503,0.00051172243632954,0.00352688726177214,-9.00451407600725
"Gclc",8433.28332699322,1.29312666639571,0.303821847683729,4.256200389
321,2.07930357540536e-05,0.000296145077525594,10.5062159198777
"Lrrc1",1767.12482307865,0.272040440526228,0.202381507680749,1.3441961
355252,0.178884970996701,0.306063457747778,0.322085767192638
"Tmed3",2422.60862895975,-0.305583997307903,0.26034944181152,-1.173745
54437928,0.240496961756739,0.378817175930462,-0.296630867693266
"Mlip",11.5997173663077,-1.19368700266256,0.585784409332537,-2.0377582
3262808,0.0415741173008905,0.100784799096452,-2.73923442218172
"Rasgrf1",7.50491898481644,-1.15962085658633,0.902880003906308,-1.2843
5766831609,0.199016837447308,0.33036185054892,-1.2843574551702
"Tinag",3074.92740205599,1.91609797746433,0.568527570003974,3.37028154
58025,0.000750914186194565,0.00471388435364509,10.2650024910742
"Fam83b",21.3916235145274,-1.91539737433339,0.699010204485924,-2.74015
652710254,0.00614099306023748,0.0232795456314746,-7.20223923202752
"Ctsh",8063.20622503968,0.15652924082746,0.19170858658257,0.8164957220
63247,0.414216669263481,0.557646662797719,0.0914177315039157
"Hcrtr2",1.58999202386541,-2.76128227319328,1.86041802581974,-1.484226
78928657,0.137748765041535,0.251199534304139,-3.81473271752573
"Adamts7",462.423246861437,-0.91419450336711,0.316005864982379,-2.8929

6688660537,0.00381621528218104,0.01620395591724,-3.7687667250628
"Tpm1",13844.872316118,-0.891162051092021,0.241882838780239,-3.6842715
0758588,0.000229357663872107,0.00188533546803471,-5.59083836230277
"Zic1",5.82183910208689,0.870156140313366,0.895713113536716,0.97146745
6669872,0.331315551173728,0.476154685397313,0.645666740216714
"Plscr1",2147.8566542371,0.0516553067949143,0.414992375874073,0.124472
905522941,0.900940851784832,0.939378270034268,0.00323036985328054
"Lactb",2458.20077301182,0.937994698786301,0.141374082726983,6.6348419
7876441,3.2485036062477e-11,1.05565538857696e-08,17.2276974088535
"Plscr2",8802.062267091,1.40386708771361,0.481353478141261,2.916499311
76278,0.00353983504110021,0.0153459361383661,5.86381887776957
"Car12",6604.23663981929,1.56304307154595,0.551894547942074,2.83214080
909893,0.00462374822495172,0.0186692571733503,6.22228238452354
"Plod2",2681.89009905313,0.192622516150182,0.282758625741774,0.6812259
5957901,0.495728524361505,0.631580944066951,0.0885156645737918
"Aph1b",620.264202769946,0.0464223824764286,0.239867591551966,0.193533
366371303,0.846541277394047,0.901940400842908,0.00479110719436554
"Usp3",2152.71246984423,0.322081201577295,0.114096953172901,2.82287293
937831,0.00475954302828723,0.0191264571239786,1.27437312236097
"Plscr4",1117.9565255522,0.0844621271071133,0.0957903445176329,0.88173
9464790898,0.377917714191838,0.523058907526772,0.0547366263804545
"Dapk2",692.815994919949,-0.704271261091815,0.163824150236882,-4.29894
652329021,1.71611869669989e-05,0.000254455379074179,-5.82882014711342
"Ciao2a",2776.10468494324,0.378990868060122,0.222343780304564,1.704526
51088771,0.088282767575533,0.179380721361791,0.651199087110596
"Snx1",3684.17675769461,0.10763225177534,0.0828149517778137,1.29967173
155048,0.193713502983354,0.323957962189676,0.1213167796449
"Ppib",10250.4318941057,0.313434034288736,0.194207549797933,1.61391271
665214,0.106546359937292,0.206648853681222,0.49420218800875
"Csnk1g1",1056.72474078635,0.277138167171691,0.256844438812506,1.07901
174910779,0.28058249007547,0.424388906329339,0.237536512166278
"Trip4",2326.11694145728,0.0879994772765115,0.157737494182432,0.557885
604387344,0.576922502231529,0.702204143630637,0.0311105532627834
"Rbpms2",3518.04083803947,1.11997364898398,0.301425533020976,3.7155898
4323348,0.000202730212998398,0.001711924509763,7.13438568905462
"Spg21",2857.93349838455,-0.211729991241229,0.223693051699752,-0.94652
0196458405,0.343883326060752,0.488491701262753,-0.151690309589566
"Parp16",647.375294544176,0.189959567242789,0.127053215039859,1.495118
14544162,0.134883611032016,0.24702146318548,0.265616673304101
"Dpp8",2770.52262976207,-0.431128695231107,0.205441586458675,-2.098546
36864299,0.0358569083203532,0.0901299469421487,-1.03751241011947
"Igdcc3",62.4340959261678,-1.04555205884889,0.467714905127186,-2.23544
737913489,0.0253879925783486,0.0693104283523719,-2.79074563180316
"Dis3l",1125.44258251187,0.0903347340299668,0.189571953171971,0.476519
50891712,0.633704312062595,0.748710336096254,0.0261431524679171
"Tipin",528.186354929979,-0.326189686330261,0.210679442422849,-1.54827
487000641,0.121556132136476,0.22886263657754,-0.481010172168974
"Snapc5",1728.81218199117,0.193551911608471,0.181341474833674,1.067333
94434999,0.28582106521148,0.429958470493484,0.163370714640169
"Rpl4",30420.7161015776,-0.205847862588938,0.229966142669085,-0.895122

47411632,0.370721621313838,0.515830312736545,-0.136266636756486
"Zwilch",224.833457865567,-0.889426469750365,0.366686496569814,-2.4255
7737487076,0.0152840572974314,0.0467757886023101,-2.72377032421667
"Lctl",424.133050052191,-2.11771155244776,0.60095219558527,-3.52392680
81637,0.000425201684592602,0.00304018424869327,-12.2739113040732
"Smad3",2387.65883910008,-0.584907045607289,0.145330016627619,-4.02468
161209948,5.70524509093223e-05,0.000640419509401247,-4.30104793141688
"2300009A05Rik",670.414505330411,0.894237447161912,0.293116057547378,3
.05079651604339,0.00228235195547854,0.0110589707822864,4.0281045142300
6
"Pias1",2560.32733165916,0.0572554846137127,0.102199284606918,0.560233
712338892,0.575320034913104,0.700967946056096,0.0203424797093249
"U2surp",5521.14975340284,0.462703030352547,0.231022581721062,2.002847
63032913,0.04519364564807,0.107514116989516,1.03188935234506
"Atr",1097.74147854193,0.55349463473816,0.272539293721905,2.0308801244
0121,0.042267156456956,0.102236076987685,1.26222827482745
"Xrn1",1776.23398573632,-0.0521871464814788,0.154709611239689,-0.33732
3234563791,0.73587324640945,0.827406525488233,-0.0098873317999228
"Tfdp2",1579.35844734442,0.504462321987507,0.166750789611437,3.0252469
7581945,0.00248430172541599,0.0117655853879429,2.24111245745768
"Atp1b3",5514.52892695207,-0.527390092954372,0.177437493150741,-2.9722
5847586975,0.00295617695965031,0.0133910058387729,-2.27472418382591
"Rasa2",1580.62000700459,0.472359084168964,0.182588191282673,2.5870188
0362944,0.00968103089563817,0.032971308367363,1.61174471585277
"Ube2cbp",74.9262417483219,-0.231366569969301,0.303168228262438,-0.763
162325073912,0.445366594294425,0.586502540139127,-0.123452176427108
"Rwdd2a",40.6946269294454,-0.82034034365661,0.417933086810084,-1.96285
091931328,0.0496634991665296,0.115733107057367,-1.76903814320093
"Me1",33343.2241654925,-1.47699219029246,0.789912257700333,-1.86981804
104727,0.0615090901834768,0.136098983249822,-2.94567310915789
"Tbx18",127.944744494328,-3.18826651379956,0.958583799527721,-3.326017
52227647,0.000880963903329332,0.00534805466457168,-16.6778935454245
"Nt5e",3468.5209360899,0.500774180569241,0.33462830340821,1.4965087396
0399,0.134521132152433,0.246535673908087,0.701208332355233
"Snx14",1513.98831313502,0.12416236304305,0.169026538175778,0.73457318
8228753,0.462599471877381,0.601895504436738,0.0630336843249953
"Syncrip",4447.58209521253,-0.489547379560558,0.15116054732364,-3.2385
9226648883,0.00120121160824508,0.00675937198775254,-2.44618272902622
"Zfp949",882.620903977103,0.158440382892913,0.102820463377185,1.540942
12074976,0.123330873257183,0.231177212725298,0.232047143736288
"Crtap",2536.17910431679,0.14958648052566,0.169407468351317,0.88299814
6312225,0.377237273054875,0.522547055273086,0.0970876454094923
"Cmtm6",7835.58068325433,0.567895888745712,0.136718649986108,4.1537558
2485211,3.27062177566868e-05,0.000416801198575085,4.41987758937666
"Dync1li1",2623.86092184789,0.206662652060514,0.127538887023183,1.6203
8933288597,0.105148669845293,0.204773148685929,0.327736484324628
"Cmtm7",1075.06619341319,-0.761992192024934,0.31887022533819,-2.389662
41271594,0.0168638657655302,0.0504235017169617,-2.27629768166865
"Stt3b",6089.62686553876,-0.470338147958348,0.286509350485232,-1.64161
535098866,0.100669744270893,0.198348693794854,-0.760879528840193

"Tgfbr2",5401.0184251806,-1.21990750708125,0.229601946754107,-5.313140
95689141,1.0775162428247e-07,4.81867240885228e-06,-14.9353423721366
"Zcwpw2",41.1276675389103,0.281026118872324,0.287120795769102,0.978773
126201282,0.327692090916143,0.472420504860408,0.210737493128768
"Eomes",70.5604879176593,-1.24304211774954,0.52703020742278,-2.3585785
0317179,0.0183450791715088,0.0537479118987345,-3.63397205233222
"Slc25a36",5653.01354239223,0.338778534783181,0.158588784853722,2.1362
0739383091,0.0326625130571137,0.0839622517584184,0.839285864862141
"Clstn2",189.775909314934,-0.752174700632455,0.337339593550458,-2.2297
2551996019,0.0257656705922266,0.0701059231380456,-1.99909080096216
"Rbp2",1.80512612738153,2.14085930821118,1.28335493215225,1.6681739825
6369,0.0952811940853151,0.190328114155873,3.55169796567093
"Nmnat3",821.49787567334,0.307171670415958,0.183010935265079,1.6784334
2241293,0.093262517494715,0.18700458310489,0.515010827653148
"Copb2",5199.87104539264,0.189003555818567,0.143293217191778,1.3189986
206089,0.187169573620057,0.315940814340219,0.217769968708701
"Mrps22",816.049134746048,-0.0404462933626951,0.295271393058111,-0.136
980060763065,0.891046555740723,0.932561768321665,-0.00282395576040192
"Pik3cb",1210.02630933457,-0.558836872206622,0.323133766602443,-1.7294
2889281568,0.0837323626514486,0.172180300250785,-0.983113142959389
"Faim",665.467287839343,-0.237776267334772,0.117456489453666,-2.024377
43917564,0.0429313312602769,0.103508729678852,-0.539300191469247
"Armc8",1681.04808053243,0.0376702067077997,0.0642835510619004,0.58600
0712243261,0.557875036211368,0.686782595104151,0.0141541090026174
"Dbr1",662.389262539116,-0.0355989927404107,0.123409083441611,-0.28846
3310379044,0.772992111469462,0.853169927663183,-0.00565299685804089
"Mras",774.462280779019,0.215122644656637,0.152012116103288,1.41516775
222356,0.157019317093694,0.277717946724677,0.275604217108623
"Cldn18",23.9587530785208,1.3285658659655,0.98721512093264,1.345771390
44465,0.178376254713156,0.305353837962872,1.57605649660863
"Nck1",1409.13735829647,-0.806335629602426,0.295045665599337,-2.732918
06529165,0.00627759465638255,0.0236705744749849,-3.01853564097308
"Cdc25a",772.69896797904,-0.442216243420416,0.134037399002989,-3.29920
04224922,0.00096960657665457,0.00573585832269745,-2.28228593719956
"Nme6",525.146989725332,0.262980480032176,0.125863363715841,2.08941245
703476,0.0366706108357864,0.0917370979391868,0.62821524561907
"Map4",6801.46709617757,-0.158520655633238,0.167701340243759,-0.945255
746929771,0.344528325059243,0.489051636721398,-0.113387795674646
"Dhx30",3466.85782635405,0.672967628866753,0.231391641113307,2.9083489
1713835,0.00363342694388484,0.0156322426263145,2.79848182022092
"Smarcc1",2384.94375476213,-0.0397459772696844,0.148941741963216,-0.26
6855864217704,0.789580139888337,0.864369894061783,-0.00579315439480048
"Cspg5",254.736542696691,-0.77023569132255,0.46497779136145,-1.6564999
5684161,0.0976206137923122,0.193671828217593,-1.26441133131496
"Ngp",1.14066404264284,1.47076785152593,2.3707317629165,0.620385601834
842,0.535003949409786,NA,NA
"Scap",2957.41965573859,0.0413555156207363,0.140389430039085,0.2945771
3169163,0.768316933643171,0.849989526276341,0.00672156371446473
"Ptgs2",64.3576651875284,-0.386236740813968,0.41462204943723,-0.931539
317164175,0.35157465647228,0.496616477601309,-0.27034147280026

"Kif9",234.50344815129,0.331034509021631,0.262992473232069,1.25872236932622,0.208130628755951,0.341354022868744,0.35580752616423
"Nradd",553.074819210889,-0.939235521551657,0.159480051335302,-5.88936054188335,3.87692807259649e-09,3.99053761831314e-07,-13.8388555512589
"Pth1r",16941.7223097963,1.42275916408865,0.271745334895208,5.23563418167712,1.64419322828522e-07,6.6479685193027e-06,16.9609954596411
"Prss44",0.819588041308358,1.20509834952674,2.66227406908707,0.45265750942764,0.650795380545834,NA,NA
"Tdgf1",2.6767164462349,0.217458625597938,1.43574532888952,0.151460444427239,0.879612511586482,0.924764625844561,0.0170087509046018
"Lrrc2",12.0290833265373,-0.633265036015712,0.549909302773877,-1.15158087492132,0.249493344090556,0.389107440655707,-0.597738725922945
"Ltf",43.4145108118872,1.06017186538982,0.713856435718395,1.48513316171607,0.137508553089902,0.250855330103566,1.46608928193136
"Lrrfip2",2761.24863399742,0.399879293234396,0.1429093763039,2.79813196010347,0.00513991087765109,0.02029114846982,1.55855774646641
"Mlh1",718.874685646305,-0.0355780067908356,0.114658568495874,-0.310295229197074,0.756336458789667,0.841774546120265,-0.00612806477778294
"Dclk3",1017.28611294219,1.22396357901679,0.500759829608552,2.44421278754242,0.0145168616182781,0.0451795319765662,3.79075121971398
"Trib1",1864.73356319421,-0.41481307503895,0.136836384417727,-3.03145305105862,0.00243379766049015,0.0115829834967607,-1.84932719982984
"Stac",111.653896361981,-1.48671017031652,0.347224700678333,-4.28169472797329,1.85475315065903e-05,0.000272297698950739,-12.2038308230224
"Arpp21",6.64214736352347,-0.23324263085301,0.80445758099913,-0.28993776224139,0.771863851730933,0.852438480943061,-0.0372381739253401
"Pdcd6ip",7575.18354980897,-0.233545015448663,0.092133946288925,-2.52991471484212,0.0114090254917831,0.0375765505133085,-0.766348793681833
"Fbxl2",320.181181351768,0.99971793629299,0.358111444241013,2.79163917369859,0.00524417997180847,0.0206384215803105,3.87950623846387
"Myd88",1691.13581541557,-0.601613191009013,0.0993356425504269,-6.05636784101546,1.39229426166063e-09,2.0411243243503e-07,-9.26760746724023
"Scn5a",55.1713295406138,-1.38915221859418,0.396668962974257,-3.50204414325286,0.000461703116105963,0.0032440675163366,-7.96113041543927
"Wdr48",2340.85976336782,-0.0288378397735525,0.140432511896189,-0.205350167024501,0.837298551480184,0.896217774522174,-0.00315981526993339
"Gorasp1",1284.4445723389,0.270393826533357,0.178118440626497,1.51805633140678,0.129000198463985,0.238774052558455,0.387268194622312
"Ttc21a",16.3019797119918,-2.13545432133844,0.652422368153578,-3.27311635157757,0.00106368681665939,0.00617622559595735,-10.8631585043726
"Csrnp1",642.421061609715,-0.539703065396875,0.133053103868328,-4.05629819753003,4.98565997669341e-05,0.000577945292660929,-4.02404297159161
"Mobp",0.126704868686727,-0.545572912234532,4.78471990596623,-0.114024002022404,0.909218766666244,NA,NA
"Rpsa",37638.1814974369,-0.115774225816367,0.201276046954897,-0.575201210317441,0.565155224334148,0.692695283132211,-0.0425082532741461
"Slc25a38",1602.07980143711,0.473984663756834,0.165685912441111,2.86074209191019,0.00422650725001032,0.0174816373272595,1.91802835220236
"Hhatl",264.862274337565,1.56693541094793,0.676126420601683,2.31751838591594,0.0204755102812576,0.0584868882894755,4.44845548691346

"Nktr",6301.00131792055,-0.328488959152263,0.20229680972292,-1.6237970
3170892,0.104419133782946,0.203901533173698,-0.522336233746717
"Ss18l2",1125.80139600386,1.01147200308429,0.303254162497787,3.3353936
3401706,0.000851787326474445,0.0052112172235954 6,5.31724947109047
"Pccb",11800.4189864993,0.770389147431027,0.39386093032596,1.955992808
91723,0.0504660012259035,0.117252870817763,1.6512693455898
"Vipr1",124.559974873682,-0.646866643675128,0.299134453319643,-2.16246
118257703,0.0305826460247975,0.0798153436208676,-1.6353044374776
"Lyzl4",3.39642261126466,-0.829128992058428,1.46350187581561,-0.566537
703681695,0.57102830983443,0.697525998317987,-0.298665108143316
"Amotl2",5344.48660238436,-0.289613609930275,0.190776681479841,-1.5180
7656828792,0.128995097382807,0.238774052558455,-0.414795490317571
"Cck",415.96419479433,-1.30434052922785,0.592171914709148,-2.202638282
6151,0.0276202534192845,0.0738838936986157,-3.39814675764261
"Cep63",2149.76123423869,0.251792832976379,0.144903659144094,1.7376568
2981127,0.0822712997406622,0.169892586564651,0.446325177148898
"Trak1",5088.54459397665,0.10017167314748,0.216810488899864,0.46202410
9884025,0.644064030886734,0.757096549962594,0.027874219620404
"Ephb1",290.084198398459,-0.560356231539166,0.263789343094036,-2.12425
65183515,0.0336486994973203,0.0858521778066935,-1.37574645579519
"Abhd5",2344.15119021776,-0.389016613111398,0.452727519430874,-0.85927
3175177053,0.390189820079552,0.534414239386844,-0.243751590005064
"Ryk",3347.95959797907,-0.0936477983692468,0.0882783418331321,-1.06082
416620675,0.288769817533546,0.432692534200459,-0.0784513721457181
"Slco2a1",2874.70616390572,-0.21995790519553,0.315364138665934,-0.6974
7278852315,0.485506963047594,0.622757312967028,-0.104171707983785
"Rab6b",1562.71939060198,-0.119648369903548,0.175710778458027,-0.68093
9273922397,0.495909915960123,0.63177076389353,-0.0549459612987736
"1110059G10Rik",789.317003024728,0.159247524346669,0.130380114175753,1
.22140960953602,0.221930970601789,0.357798087050907,0.163672448410981
"Srprb",1990.13222934588,-0.113948647240423,0.135420822748721,-0.84144
1108741894,0.400100863831794,0.544242126621481,-0.0693219181623642
"Trf",13836.981295885,-2.74076616567912,0.62528456203158,-4.3832301836
6908,1.16932447806291e-05,0.000192076568435304,-23.454425683945
"Topbp1",1477.62840672899,-0.114814866669547,0.196727636962972,-0.5836
23472746524,0.559473664336792,0.688067207470593,-0.042925691893615
"Bfsp2",12.4898988751315,-1.85708447127478,0.984811197693418,-1.885726
39671885,0.0593318338631964,0.132438705971906,-3.75434759096957
"Uba5",3775.23982945939,0.331755582850806,0.168020489899687,1.97449479
553876,0.0483255086438823,0.11346950476137,0.721973518909581
"Nphp3",941.798243168006,0.379662986410382,0.289867791863828,1.3097798
2744884,0.190270329280055,0.319861983585881,0.4327648072338
"Dnajc13",3869.87196446988,0.818564597735455,0.191550738461873,4.27335
65232294,1.92552203489149e-05,0.000280177825644136,6.69592885906962
"Acpp",382.633926633905,-0.247784297559514,0.301053128988974,-0.823058
369768978,0.410474801115533,0.553947790154393,-0.146362427730887
"Gnai2",12403.0974121224,-0.574921378256531,0.141041041867551,-4.07627
007461011,4.57638629627195e-05,0.000540945058729681,-4.32466947468359
"Mrpl3",1536.21087854095,-0.191177155337741,0.2073973297212,-0.9217917
87747397,0.35663719187067,0.501710820136675,-0.131860883324455

"Cpne4",254.259048660106,1.69276048347616,0.691947184689573,2.44637238
351592,0.014430187255954,0.0450031655656737,5.24928825459256
"Nudt16",601.602298250458,-0.000737561654491029,0.138201983675815,-0.0
0533683840762484,0.995741839244444,0.997173825453938,-2.08742908491759
e-06
"Aste1",509.608041041996,0.426825952448753,0.115802290812411,3.6858161
3933849,0.000227970894678465,0.00187551751242224,2.67998494592302
"Atp2c1",3444.00940432224,-0.177083463731897,0.127990158392527,-1.3835
7093979685,0.16648987164823,0.289582472559963,-0.219462218218642
"Pik3r4",1377.37109216524,0.358865505986917,0.122326612637643,2.933666
6670396,0.00334983750663827,0.0146973743709345,1.51444344676135
"Col6a4",218.043102939127,-2.94361875750032,1.06539442346704,-2.762938
01869274,0.00572836432111828,0.0219995366423408,-11.2350094638011
"Manf",4482.06464152463,-0.690019073066709,0.288639283117549,-2.390593
08079593,0.0168211846659972,0.0503267544295861,-2.06261774135468
"Mapkapk3",1947.72964343508,-0.487217607549208,0.12241555641918,-3.98
00301680102,6.89065231961571e-05,0.000742711683730168,-3.5105015882637
9
"Cish",1579.08951140811,0.237562846138196,0.239799203778965,0.99067404
0590933,0.321844770912766,0.467055009263894,0.180858551188639
"Hemk1",893.116601655207,0.237815479923212,0.24556269181786,0.96845118
5164583,0.332819086998199,0.477541140502678,0.175770601882334
"Rbm5",8588.78842338206,0.0366222855670128,0.158242926220295,0.2314307
90884326,0.8169801466965,0.882813062529836,0.00456466788636773
"Rbm6",3930.11594294646,0.0870636177199009,0.141730033965968,0.6142919
41401825,0.539022426782276,0.67001525420125,0.0348650435683427
"Mon1a",1283.95914216435,0.255533586845573,0.153321924602503,1.6666473
9898133,0.0955845379955976,0.190716039260532,0.423411401183807
"Mst1r",280.390165295184,-2.2360441930398,0.636554430579326,-3.5127305
4686743,0.000443527026876326,0.00314354415486536,-12.8849909051481
"Traip",84.0807480125358,-0.863761467419578,0.292470836951041,-2.95332
511242538,0.00314370682564283,0.0139977153885325,-3.68727777045779
"Bsn",55.7242212561557,-0.675945201178697,0.338332540528762,-1.9978722
7123438,0.045730509540704,0.10848685667656,-1.50135962987305
"Apeh",6173.02005705699,1.39705934144308,0.281481485535888,4.963237062
58456,6.93278762055758e-07,2.04192587652012e-05,15.0868885974827
"Mst1",30.5157184246779,-1.82928513607838,0.812130175321067,-2.2524531
0624641,0.0242936529511841,0.0668752852240841,-4.94808057612659
"Amigo3",79.4362878701948,-0.409525515487017,0.248159124466069,-1.6502
5370865625,0.0988910559344379,0.195634923763156,-0.668142922242935
"Ip6k1",7282.97424810104,-0.0649060106333077,0.120374137843979,-0.5392
02288762677,0.589747281267408,0.713153838386996,-0.0219420039137116
"Cdhr4",23.9997145275935,-0.845894212328044,0.51184688511755,-1.652631
35700001,0.0984059122896428,0.194872890292855,-1.38338197387953
"Uba7",3190.21338194051,-0.715612225873984,0.266135502798171,-2.688901
77503557,0.007168750976681,0.0262441431737375,-2.60505208236158
"Nckipsd",1137.7821124041,-0.0216397305060837,0.239793522627822,-0.090
2431820048377,0.928093970016233,0.955739739483284,-0.00097962285360860
3
"Ip6k2",4535.33848995113,0.403283140469998,0.174182062925117,2.3152966

1377001,0.0205967049533434,0.0587470089497204,1.14311212653574
"Prkar2a",3423.421312885,0.453369342646552,0.0882416190824202,5.137817
58948793,2.77947478633614e-07,1.00174120857638e-05,5.21881872587212
"Slc25a20",3712.75236925395,-0.169474305598048,0.459640820447493,-0.36
8710301737459,0.712343669286279,0.809209791642034,-0.0358772145054673
"Qars",4070.2571655782,0.322819151010223,0.110902225802498,2.910844653
24547,0.00360453182002473,0.0155420525048302,1.34428540163096
"Nicn1",1388.1032884071,-0.0498625063871342,0.236004063044541,-0.21127
8169298822,0.832670216805049,0.892938284416723,-0.00564632106874173
"Amt",2414.69198193427,0.797079368050624,0.190542146195825,4.183218169
65075,2.87411331113987e-05,0.000377370110037745,6.28280605142557
"Klhdc8b",3012.74571571593,-0.535071790555384,0.187540853056089,-2.853
09457558752,0.0043295734115949,0.0178070294681291,-2.15535584633683
"1700102P08Rik",63.4673623473256,0.223253134082816,0.349553218845452,0
.63868138539878,0.523030224662506,0.656160296002415,0.0940677451561191
"Usp4",4943.98605813155,0.231534403242601,0.107300534431081,2.15781220
91397,0.0309424352536362,0.0805387268155652,0.58323912836562
"Nt5m",816.482058507288,0.342880869932963,0.139328330325028,2.46095585
250383,0.0138567415625888,0.043598313214032,1.07415552512756
"Srek1",7629.83789977752,0.68516324007271,0.235584518182266,2.90835427
285003,0.00363336471165553,0.0156322426263145,2.84919628965807
"Oas1d",0.115678569700437,-0.0595056820677523,4.78471990596051,-0.0124
366072073778,0.99007727891101,NA,NA
"Eml4",1684.46265643372,-0.430789453896304,0.211265441428244,-2.039090
9700327,0.0414409492650428,0.100574511920563,-0.989461520286093
"Thsd7a",1260.26743482674,-0.39688565114539,0.262737665764817,-1.51057
767065896,0.130896086601123,0.241298278958938,-0.564260838964596
"Flcn",2672.56934475968,0.225992702387393,0.154806616317934,1.45983878
313871,0.144334386872812,0.260311893002136,0.304157878252026
"Atxn2l",6772.09002141166,-0.273444465992757,0.175512673707891,-1.5579
7561632418,0.119239028445702,0.225414250206932,-0.407381792208289
"Chsy1",1027.28374062298,-0.639760099537087,0.193920400492265,-3.29908
610911004,0.000970001516882135,0.00573645422389077,-3.30174684829953
"Gpr19",222.195258304881,0.111442723736418,0.128799673759334,0.8652407
29915609,0.386906674461891,0.531277002412613,0.0704843724964041
"Fhl3",515.138612017527,-0.696862714627878,0.259430831533171,-2.686121
4240019,0.00722868137271485,0.0264188996073466,-2.53217306411619
"Pygm",506.887765313821,-0.747339901912843,0.255349920732312,-2.926728
54477324,0.00342547720894437,0.0149619073969528,-3.14050746970136
"Colgalt2",508.132436989772,0.703830969411034,0.208383232985555,3.3775
7966093089,0.000731267685813254,0.00462630023945063,3.7837937534536
"Crebl2",1539.22001722467,-0.023479426737481,0.18188913235868,-0.12908
6473903126,0.897289224719621,0.9368324125078,-0.00153205297761492
"March3",196.072863269274,-0.139692034694548,0.328684384707101,-0.4250
03563278587,0.670834077170158,0.777363772534395,-0.0351810006477218
"Fam189b",674.485676488645,-0.565746545261178,0.271511926215031,-2.083
68948336037,0.0371884214526469,0.0926763601078359,-1.34570841128593
"Oas3",217.514864641674,-1.44158448918869,0.529421249537158,-2.7229441
4032112,0.00647029970222818,0.0242331739519872,-5.3627415262041
"1700025G04Rik",963.858590391844,-1.15890623624325,0.298092557810668,-

3.88773958248003,0.000101182097880732,0.00100043029638869,-8.004942105
34419
"Pon2",1750.4661325983,-0.855401510045626,0.28653029540711,-2.98537894
162378,0.00283227219646589,0.0129405701951478,-3.71876219694551
"A930018P22Rik",2.13998045292294,-1.34246725468711,1.38773984745423,-0
.967376743666925,0.333355731673922,0.478060352875084,-0.99076539200257
3
"Prorsd1",889.734273679294,0.1225601257473,0.22354974755719,0.54824542
2267567,0.583523397120661,0.707558407777279,0.0423978490757422
"Cd59a",3250.56093096004,0.120733320394506,0.214905955059371,0.5617960
67313033,0.574254971855819,0.700020221396983,0.0430590626244784
"6820408C15Rik",48.1029887272419,1.21291904761308,0.443643145277943,2.
73399704362205,0.00625706062160969,0.0236276808999876,4.54278978018688
"Malt1",1557.72792536056,-0.191367301812214,0.176611102227719,-1.08355
193642056,0.278563511252452,0.422285130026459,-0.16497287667799
"Oas2",1220.33233611024,-0.70716728702052,0.447523085559891,-1.5801805
7579262,0.114065518784331,0.217915881369478,-1.07747271847239
"Nlrp3",131.050071736829,-1.44697659328503,0.50814623949884,-2.8475593
8509377,0.00440558737524169,0.0180273122019858,-5.8108660232347
"Lmo2",1827.95196154954,-0.679833953202273,0.28338669762673,-2.3989621
2100165,0.0164416154100191,0.0495025659766141,-2.04339783356129
"Kank1",3107.72477520624,-0.108065982702315,0.190934941725901,-0.56598
3270141569,0.571405153383927,0.697724053148785,-0.0388963613804595
"Exd2",1377.78640851556,-0.073548751619601,0.155115963258001,-0.474153
337121526,0.63539056603166,0.750111725386613,-0.0211477017578759
"Resf1",4258.91713405048,0.267166444928166,0.229913000675454,1.1620327
8693796,0.245222147181713,0.384373508700805,0.255448663642061
"Syde1",1891.15509564985,-0.329195584870427,0.121409472537268,-2.71144
893384967,0.00669898588428907,0.0249122523800047,-1.21552030708555
"Trib3",122.062721766894,-1.09637542703248,0.216006617321481,-5.075656
66565092,3.86160027685884e-07,1.30357945468295e-05,-12.3318241995995
"Mdfi",368.849861767351,-0.826220943235319,0.231295387115807,-3.572146
22192892,0.00035406761550239,0.0026285112682089,-4.90885759790603
"Mansc1",345.299267324135,-0.0133713741323314,0.16666780592385,-0.0802
276963941121,0.936056162617768,0.960085379206798,-0.000544657008898402
"Sbspon",85.4734490117124,-0.780301823968083,0.73024228675974,-1.06855
19561329,0.285271607009304,0.429494949749053,-0.659468416040849
"Abtb2",519.600436755869,0.57897816272999,0.277856970035406,2.08372733
156996,0.0371849766315921,0.0926763601078359,1.37718169039271
"Folr2",228.018241219412,0.914397432550729,0.329729001280379,2.7731786
6793642,0.00555116334240517,0.0214584819290674,3.51278144034012
"Bmp8a",34.8214186524649,0.745989384414081,0.417740389599113,1.7857727
0234744,0.0741360683012282,0.15693247852973,1.38152731141945
"Mier3",1445.89849752963,-0.0955450329360586,0.124206655914023,-0.7692
42455108006,0.441749391411297,0.583433559150407,-0.051482023689621
"Snx33",1216.96120703694,-0.526739485397088,0.074684525586529,-7.05285
976258644,1.75277483525711e-12,1.13918679126144e-09,-10.8471205021392
"Ablim3",725.190971502769,-2.03009433867811,0.528322914333343,-3.84252
562893236,0.000121774693385787,0.00115935691974418,-13.7232140275219
"Inppl1",5970.65713338053,-0.173644105604071,0.157117406905915,-1.1051

8693646753,0.269078596214399,0.411425224983403,-0.154218190253057
"Pram1",69.8810925018861,-0.82210002095737,0.290893425346828,-2.826121
00970379,0.00471154612528486,0.0189687644746653,-3.25959503718056
"Ccdc88a",1967.73159049353,0.0106657050411268,0.287028690502212,0.0371
590206625864,0.970358212803502,0.981941181421263,0.000194370412968584
"Tpcn1",5592.98225371731,0.69985880649774,0.156439639084389,4.47366671
64018,7.68895566722276e-06,0.000138429600738236,6.21834953638072
"D430042O09Rik",1734.27627784362,0.226758665029312,0.208787348977872,1
.08607473651742,0.277445928449953,0.420954256731232,0.196198650322536
"Heyl",851.670827749857,-1.46869697794577,0.250635449576376,-5.8598932
4506234,4.63164826410308e-09,4.56100393199403e-07,-21.4437879075529
"Gpbp1",2986.94301835396,-0.356708656101866,0.148476644767248,-2.40245
633689421,0.0162853785876901,0.0491688311710717,-1.07458317459657
"Gab3",138.964994277602,-0.371052275001425,0.37176149438014,-0.9980922
73165898,0.318234616584343,0.463296610448278,-0.285483094547174
"Slc8b1",1976.19410811409,-0.506788145379652,0.149324054791506,-3.3938
8148873439,0.000689095063349744,0.00441102283164586,-2.74864085664147
"Bet1",1618.29147953878,-0.0773009188366591,0.178956719597363,-0.43195
3150519185,0.665775463734094,0.773579226723292,-0.019845247518096
"Kap",667869.924159994,2.52117809327019,0.943604535389316,2.6718588123
6782,0.00754323789983755,0.0272720290322701,9.08101543930127
"Ilvbl",2061.46269147712,0.718247024170663,0.132302099799866,5.4288407
0061745,5.67212822812044e-08,3.00530315738838e-06,9.13260580047824
"Gng11",1530.34330190155,-0.693139248158442,0.182832536711886,-3.79111
541426958,0.000149972238032229,0.0013632441478566,-4.57325513950608
"Trpa1",0.715634209851955,1.0042717228535,2.19019219458431,0.458531322
199378,0.646570764708842,NA,NA
"Chrm1",14.5465324548412,0.0414595167069589,0.557004173199348,0.074433
045032359,0.940665815588138,0.963293176068147,0.00155048116306992
"Mctp2",144.056033304579,-1.24863477366238,0.317222703092303,-3.936145
68405926,8.28007195517871e-05,0.000854450542227716,-8.82166952536855
"Gtf3c1",3330.90531267233,0.22902349967068,0.151227794730467,1.5144272
9214473,0.129917501580878,0.239993505052009,0.326849380188669
"Cntrob",744.919984272811,-0.0736183811314435,0.193990224865461,-0.379
495313140137,0.704320084033609,0.80365361648451,-0.016092015768719
"Troap",66.5424809504645,-0.976893621534203,0.659857306590613,-1.48046
192984006,0.138750013303246,0.25254814651244,-1.34435544861111
"Alas1",5708.99101940153,0.284668781135138,0.393600289050357,0.7232433
23377534,0.469530406459148,0.607653250042511,0.141808001592374
"Pdxk",3978.77459078619,0.0891414716961788,0.24251337996143,0.36757341
6816655,0.713191339500764,0.809795291031091,0.0188065141636734
"Lama1",776.4537345881,0.673592449361799,0.472569699877838,1.425382223
05816,0.154046743899074,0.273626984835942,0.872968720980228
"Srxn1",2319.33504461051,0.0656697414690127,0.206958588395713,0.317308
60737922,0.751009446837079,0.83805364594581,0.0116020809983018
"Cdv3",4883.759305933,-0.0211503552847928,0.114104557945961,-0.1853594
25298414,0.852947130824998,0.906427978408292,-0.0020778892044893
"Slc10a3",351.597563131526,-0.346238447424348,0.176696237315937,-1.959
51228324838,0.0500528228042968,0.116487221179062,-0.744403556390743
"Alox12b",3.49887759399103,-0.354755445115909,1.02949847767963,-0.3445

90548512015,0.730402212274798,0.823201291036648,-0.0690192775232475
"Cyp2c38",2.80733728310569,1.16654475559894,1.03126954207456,1.1311734
7890664,0.257982082188135,0.398936288927702,1.07200044639204
"Arap1",5204.49429643893,-0.821097227134783,0.209878031188139,-3.91225
905106158,9.14367507335689e-05,0.000920559224143257,-5.73990415895931
"Fanca",409.730753010211,0.865876188584088,0.362982276068921,2.3854503
2545799,0.0170582259393476,0.0508875901721847,2.57869721602096
"Igdcc4",237.398051181809,-3.13581710153454,0.520127615587359,-6.02893
791361834,1.65040670288606e-09,2.28224325481365e-07,-47.9558527752074
"Loxhd1",0.336219051263774,-1.65347479902343,3.83581333134509,-0.43106
2373528906,0.666423018422428,NA,NA
"Ank2",297.133215253891,0.201296771973165,0.282858808048084,0.71165106
4933239,0.476680877196029,0.614215222944344,0.0981140367373569
"Ppp1r9a",1761.23039885626,0.251240177443231,0.211851013767584,1.18592
860602905,0.235650495137262,0.373547375204309,0.247398825979754
"Pwp2",914.968375938089,-0.133465969452974,0.100441154532523,-1.328797
6434974,0.183914739859822,0.312121994759058,-0.155402590846432
"Trpc1",1569.23042274127,1.24066567181143,0.289322542188647,4.28817492
901219,1.80147199158595e-05,0.000265696678456451,10.214593035253
"2410131K14Rik",797.450303507716,-0.22103276038098,0.231660850179062,-
0.954122201529233,0.340021789175535,0.484701333919037,-0.1600768726837
29
"Prr5l",287.980347514547,-0.889749563518225,0.212615803291796,-4.18477
624778025,2.85447126353964e-05,0.000375549802270553,-7.01756110259096
"Abcc10",1661.34936992058,0.7822793145192,0.240072467434378,3.25851324
343568,0.00111997648757888,0.00641707362762651,3.94956639480981
"Alpk2",48.3056833049633,0.546244274045785,0.405935477515324,1.3456430
9921683,0.178417645054402,0.305397879314435,0.647922090454724
"Zswim6",1180.82138600874,0.353999962623978,0.258615647199748,1.368826
54416714,0.171053501418392,0.295857462135706,0.431128585047734
"Abcc4",1644.60977661178,-0.573023245056204,0.232002545648534,-2.46990
067912569,0.0135150570193395,0.0427231812196955,-1.8067500964391
"Rnft2",249.36086255129,-0.962132084193973,0.350688399347781,-2.743552
64098661,0.00607783025638946,0.0230824959756684,-3.6259686152663
"Rspo4",0.969682059987668,-3.3093454009672,2.96832529893939,-1.1148863
6442564,0.264899120609913,NA,NA
"Ugt8a",2245.98954678823,1.76619257137542,0.612825131401365,2.88204983
913865,0.00395097234823614,0.0166528445408362,7.23286631032146
"Pkd1",4731.67297766397,-0.582623672922545,0.164638923394775,-3.538796
66429496,0.000401955341569891,0.00290534388557293,-3.40322344826613
"P2ry2",299.050055059534,-0.688854345988723,0.226337993972007,-3.04347
641286385,0.00233861773524119,0.0112699872965232,-3.089933374436
"Rag2",0.345239651971042,-0.465485296666782,4.78471990594864,-0.097285
7985037043,0.922499433653354,NA,NA
"Fbxw8",3702.24855589715,0.0778359934283532,0.156259766524733,0.498119
22262173,0.618400012554892,0.736789700262467,0.0237752200437913
"Psmf1",1271.09463064752,0.297967997626844,0.13127428041627,2.26981246
198408,0.0232189641323836,0.0646009364516576,0.816291187553369
"Smap2",2015.63136312375,-0.359333817659051,0.182593936348496,-1.96793
948827102,0.0490750005918918,0.114731937834617,-0.778014075635196

"Cyb5r4",2464.6411947255,0.424413439712789,0.170644328354834,2.4871230
3423453,0.012878085318182,0.0410624542165024,1.35500827831586
"Arhgef17",3535.76951851628,-0.543933194468603,0.255717343856009,-2.12
708761269976,0.0334128043138081,0.0853620097629234,-1.33854024680884
"Ccdc85a",144.272734344711,-1.79394475361438,0.478237422967121,-3.7511
5929339916,0.000176018821348441,0.00153146585392767,-11.6275066093278
"Acsl3",1200.21307043728,-0.51357788061317,0.260000348561231,-1.975296
89269713,0.0482344656665715,0.113283017533945,-1.11850381047467
"Rims3",55.5221000655275,-0.705027074061864,0.294675583575546,-2.39255
341588597,0.016731592417503,0.0501529639200561,-2.1099187779223
"Rangrf",457.714587372916,0.776537631784374,0.18430680686278,4.2132878
5953369,2.51679970277937e-05,0.000343886199052503,6.19304274397995
"1700031F05Rik",1.75029690476247,-0.0409722814930489,1.28749106072497,
-0.0318233522102887,0.974612923695024,0.984305541603935,-0.00064813725
8232409
"Nfyc",3263.28472568178,0.776929495960492,0.137573953792268,5.64735892
619344,1.62931436768914e-08,1.18538699780608e-06,10.6015457973892
"Fbxo21",2347.99480469679,-0.672795438579632,0.411149866528234,-1.6363
7518421383,0.101761089573942,0.199933265267303,-1.08304701774378
"Styk1",324.069395228214,-1.31197576425889,0.368675716445668,-3.558617
25016065,0.000372812356344069,0.00273995300565272,-7.7404136506785
"Tex43",0.291913614356984,1.1431185991579,4.75616617243151,0.240344545
946237,0.810063164186048,NA,NA
"Slc16a1",2329.49278649085,-1.29116582294777,0.491285188650732,-2.6281
3911914145,0.0085853396457381,0.0300084645805516,-4.52718345868866
"Atg12",2122.98799737482,-0.185953317732985,0.302409053825562,-0.61490
6582262079,0.538616415624511,0.669812533151446,-0.0745221694405636
"Sgpp2",1227.55176650403,0.672686648980631,0.456214179001149,1.4744974
6181374,0.140347700600041,0.25481883474249,0.91969882670124
"Cspg4",1895.37105636124,-0.921337831055713,0.274928500050695,-3.35119
069461996,0.000804648717706852,0.00498321423858778,-4.88463850574794
"Lrig2",1269.29783531183,-0.313878963810862,0.11749932643232,-2.671325
64365513,0.00755523115128089,0.0272998859302633,-1.13023820921022
"Adgre4",62.501703794826,-1.18224040286985,0.359733140008562,-3.286437
28192992,0.00101463365759534,0.00593915552560609,-6.06038694831296
"Odf4",11.5830797469335,2.98471010687893,0.811410745593779,3.678420729
68205,0.00023468260595261,0.00191778769944006,18.6740865061388
"Itgbl1",146.177769956381,-2.19159373758362,0.469647703374734,-4.66646
3227299,3.06428454936789e-06,6.75112092017798e-05,-21.0463500441723
"Hspa13",1300.99610154455,-0.459021785312287,0.196200606217081,-2.3395
5334880268,0.0193068139546275,0.0559472887398753,-1.32351830912506
"Camkv",1.68568254715628,-2.25625994168664,1.50627644778698,-1.4979056
1022284,0.134157775877202,0.246054775096763,-3.16373018304043
"Nup93",934.178611174908,-0.0295441819567012,0.125561998603084,-0.2352
95569403079,0.813979333708263,0.881720502702429,-0.0037190264568308
"Rbm11",129.671912299402,0.277609506889045,0.443557321651196,0.6258706
44758177,0.531399790357985,0.6637136065851,0.113793396647113
"Ucp3",528.106508013464,-3.51719102574486,1.2998353405271,-2.705874287
36826,0.00681248297171983,0.0252624178361722,-12.9377671831928
"Rasgrp2",1902.81762739469,-0.619435700625897,0.200718902321067,-3.086

08553286654,0.00202810472750351,0.0100968921544931,-2.84663386482279
"Lipi",0.353797169457995,-1.82943708587333,3.19040436359351,-0.5734185
63097983,0.566361324434837,NA,NA
"Ap4b1",1138.27309437703,0.18545597441355,0.162158126345504,1.14367363
876915,0.252759009758431,0.393037337289249,0.173188185182789
"Pebp1",17446.2066590993,0.799380326971142,0.274567894026983,2.9114122
385066,0.00359798967359786,0.0155241430971036,3.32970591764214
"Ift57",992.34377109163,0.623512601985244,0.233693708593797,2.66807611
440249,0.00762869734646639,0.0274588039249403,2.24157047521021
"Fkbp1a",7925.05942964014,-0.520118504090733,0.207642215195364,-2.5048
7842080364,0.0122493491349521,0.0395688882427593,-1.679833036468
"Inha",328.79585880022,-0.00816421009981045,0.240868289321525,-0.03389
49146141541,0.972960948409808,0.983401201186793,-0.000136653385824761
"Fam207a",1807.64338816902,0.035774759901948,0.160822929292805,0.22244
813012213,0.823965047814298,0.887213966328871,0.00428113335738968
"Guca2b",10790.1827431226,2.13100620706772,0.771081553131272,2.7636586
537623,0.00571572980039384,0.0219596649552865,8.13734931844521
"5730522E02Rik",5.41555993088899,0.674872375464959,0.771705834741923,0
.87452024473892,0.381835000126595,0.526743278741858,0.432621528028473
"Slc16a8",13.300101759475,1.15683956121503,0.804571754293898,1.4378326
7936156,0.150481534634692,0.268738684952118,1.52010546489155
"Chpf",1478.904310425,-0.889507763552143,0.24428784527632,-3.641228087
07899,0.000271340608630006,0.00213675249881577,-5.46911013244964
"Foxj3",1969.71792370711,-0.152027739603381,0.138068127297136,-1.10110
66969584,0.270850222691284,0.413291410395574,-0.134332066558416
"Nlrp4f",7.75252535013933,3.4910065122059,1.32717579472738,2.630402487
80531,0.00852838319394384,0.029874801696546,12.2560158763399
"Mycbp2",7110.78128134074,-0.263211019019313,0.106756323673971,-2.4655
3093962984,0.0136810359261957,0.0431708753016611,-0.827164104092721
"Sox10",48.773098875789,-1.3256247094838,0.385578331536189,-3.43801661
312852,0.000585991745749985,0.00389290189391251,-7.35536180828492
"Asic4",6.91200097026395,0.0249089054006573,0.725782063544618,0.034320
0895307411,0.972621905176315,0.983313383134283,0.000419152285619326
"Ogfod1",886.308277185645,-0.478271046183663,0.19644034043834,-2.43468
854267123,0.0149046126660949,0.0459898936166354,-1.47275610749864
"Trim33",1533.98760331967,-0.0852334225177013,0.11413870386313,-0.7467
53026211945,0.455212655075461,0.595447225859475,-0.044188629989795
"Nfatc1",808.836784986167,-0.711328322711439,0.191947233984421,-3.7058
5346788157,0.00021068008373316,0.00176075451034255,-4.5112533722665
"Polr2f",2869.75630787661,0.688905557933712,0.139547804206967,4.936699
37587824,7.94558047527842e-07,2.30540071587766e-05,7.35590686824613
"Gmppa",1974.97729684999,-0.0484031173443937,0.123788964071005,-0.3910
13186899517,0.695787488566634,0.797323799710386,-0.0109630354826287
"Cdo1",12577.4779876314,-1.5229181790485,0.639188499124573,-2.38258069
588905,0.01719176359557,0.0512467522682194,-4.52474682131153
"Klra9",19.5832179074585,-2.78878972665188,0.610488687015896,-4.568126
79082333,4.92102337170116e-06,9.83095427268742e-05,-25.7332489241054
"Klrc3",1.45228626529532,-3.5678574545682,1.71296251848797,-2.08285786
528332,0.0372641812243889,0.0928177063762308,-8.48121766263942
"1700088E04Rik",295.316179637771,1.04649850667985,0.288341921513343,3.

62936648679933,0.000284117634052742,0.00221058484786926,6.398813195020
37
"Cip2a",227.167574150193,-0.156375346444834,0.297282465572631,-0.52601
6043844433,0.59887705973674,0.720885597650757,-0.0511777143079849
"Afap1l1",1876.01327546777,0.794580211201806,0.160031499264662,4.96514
88291547,6.86484049636179e-07,2.03111775414358e-05,8.58491412791829
"Calhm2",321.91232330083,-1.1696607197275,0.27749310830308,-4.21509826
633242,2.49669454504296e-05,0.000341819314658468,-9.33532921455606
"Gm7879",0.124297893645588,-0.542933062309523,4.78471990596623,-0.1134
72276952413,0.909656140889958,NA,NA
"Micall1",3839.53565451756,-0.193914911356495,0.178484248306907,-1.086
45392070147,0.277278216887546,0.420794728586034,-0.167854714490717
"Dhrs7c",0.101658679199014,-0.544048378937456,4.78471990596623,-0.1137
05376621746,0.90947135024012,NA,NA
"Eif3l",4430.77847946,-0.195040954424543,0.21728366041429,-0.897632864
121776,0.369381312219686,0.514332416315035,-0.129679902154837
"1700028P14Rik",0.645412776555757,-0.733518792818315,2.11865479196721,
-0.346219117715364,0.729178048300999,NA,NA
"Npat",655.286123773094,-0.253115495328702,0.138106581610759,-1.832754
76357879,0.066839049168703,0.144834836295139,-0.489059950789897
"Ankrd54",1475.16956968441,0.338020665917247,0.127545522612439,2.65019
625145416,0.00804450271088395,0.0286016983692223,1.20142319527657
"Pygb",3437.45611264127,-1.27125812354183,0.346512769519897,-3.6687194
1055214,0.000243768437832974,0.0019681147001521,-7.92080154748748
"Lmo7",3632.88692852059,0.433102927706619,0.177296337529748,2.44281937
089619,0.0145730290826674,0.0452984237485801,1.34022966563391
"Resp18",0.22558165094264,0.00101168431383369,4.77171825507405,0.00021
2016774619479,0.999830835090167,NA,NA
"Cntnap3",17.1156586523661,-1.04012313242601,0.528899413067993,-1.9665
8023572489,0.0492316238693376,0.115017576314095,-2.24944343261856
"Pfkm",2086.68260682723,0.231747447581272,0.263742546728088,0.87868813
9082078,0.379570391437736,0.524400445848011,0.149592902533094
"Gas7",1430.59731987326,-1.32951768847334,0.253749198745307,-5.2394951
1977691,1.6101650428219e-07,6.55427933297315e-06,-15.8683152992054
"Entpd6",1363.60521840728,-0.559048198682206,0.317968978953312,-1.7581
8471513315,0.0787160850947951,0.164255803422337,-1.00982569450362
"Senp1",1182.06684230299,-0.431489898952124,0.150586462064612,-2.86539
635128013,0.00416487590846625,0.0172855967488205,-1.75093495697713
"Vsx1",0.113734556814598,0,4.78471990596623,0,1,NA,NA
"Clec1a",308.819748911541,-0.0568970717710036,0.213980216193807,-0.265
898748879993,0.790317188671428,0.86497920542888,-0.00825290958113572
"Tbc1d4",1787.06792002569,-0.941877427039245,0.166008774386549,-5.6736
6050692055,1.39778010905372e-08,1.02844968174828e-06,-12.9860955163431
"Triobp",2373.96560188277,-0.0391454121448772,0.123869073416778,-0.316
022482974148,0.75198544303391,0.8386553891366,-0.00688784653060014
"Apmap",4718.69835089908,-0.313364500369875,0.467083325406777,-0.67089
6354728505,0.502286558730565,0.637229704088006,-0.141209904755449
"Nol12",1103.5602027772,0.0913276581652282,0.182993144985776,0.4990769
36309974,0.617725184810055,0.736255617912247,0.0279625118314027
"Cdc14b",1368.08710401128,0.40264043338729,0.196747280310025,2.0464853

8344636,0.0407086390915062,0.0992536385845264,0.930130283358216
"Lss",1431.47134295014,-1.29464430587131,0.526279442143488,-2.45999406
816717,0.0138939312153196,0.043687126404822,-4.05314527242186
"Slc7a6os",1774.62240099618,0.124172693157788,0.129012631100161,0.9624
84774544162,0.335806147658413,0.480377715850604,0.0910412551013512
"Rnf125",79.2942124633007,-2.51268247159717,0.596978085760308,-4.20900
286213527,2.56500140008801e-05,0.000347549668512272,-20.012518281484
"3830406C13Rik",1459.3195415371,0.0728798909748646,0.145669829384946,0
.500308754960322,0.616857690403701,0.735671371184253,0.022372068875619
5
"Slc35d2",660.201734095644,0.559202419751772,0.133319819664924,4.19444
326550417,2.73542603828546e-05,0.000363072409084342,4.4293910709531
"Hsd17b3",0.195819844963184,-0.00681195359704331,4.78471990596399,-0.0
0142368910425717,0.998864060828067,NA,NA
"Atg9a",2423.42884859491,0.379558974771941,0.128929846670479,2.9439186
0824921,0.00324085216872831,0.014312601491702,1.61184080018877
"Ybey",296.219780652344,0.290668885318857,0.124397539226364,2.33661282
310361,0.0194593250960266,0.0562350556873169,0.83660753118557
"Gga1",3726.43637052721,0.0602867339103998,0.116783672790246,0.5162257
05785777,0.605696778555168,0.726519153348486,0.0192610346882454
"Slc22a15",625.204687172449,-0.753875598039435,0.176919303836952,-4.26
112686230217,2.03398727433383e-05,0.000291607969668831,-6.136622927015
04
"Phldb2",7766.85802684325,0.890824147414816,0.255936918618997,3.480639
49594138,0.000500218268419192,0.00346351413268373,5.04693887218167
"Podxl2",465.912150640642,-0.398742588460267,0.359225985989226,-1.1100
0485491667,0.26699693442829,0.409012117181238,-0.356480059691684
"Abhd10",680.740422091073,0.219538153536549,0.152664652895359,1.438041
80845338,0.15042219196426,0.268657314181473,0.288543086613351
"Cnppd1",4401.62443246838,0.467789151437069,0.137488063493944,3.402398
28498039,0.000667972143475782,0.00430691827165701,2.54829667520363
"Atp1a1",85091.7373473408,1.41901798273428,0.387097132686955,3.6657930
6047672,0.00024657326005555,0.00198664686965418,8.82814658227777
"Dis3",752.580800963488,0.0600032894125388,0.232496267898503,0.2580828
0689793,0.796343000325003,0.869670427934183,0.00837891679090503
"Card10",3744.05044304881,0.0437741963777137,0.239404722470071,0.18284
600205907,0.854918852588759,0.907661736334093,0.00424099765535805
"Mgll",10225.074030125,-1.5696248109018,0.675043836952378,-2.325219082
04983,0.0200602485070886,0.0575875626312151,-4.48041343996636
"Tmprss7",11.3140837578225,0.630064353496879,0.58051918284933,1.085346
3108736,0.27776830186045,0.421341710726238,0.544571620797973
"Kbtbd12",9.05362380927426,-2.29043979962633,1.06445422239969,-2.15175
040074789,0.0314170182129997,0.0814369876518304,-5.74425287310909
"Tmed7",5682.19763080373,-0.435967786008094,0.225486375821979,-1.93345
511195004,0.0531801576896598,0.121859997018802,-0.917660945294413
"Mzt1",1951.81658752305,0.106822848122628,0.0941163984517813,1.1350078
188272,0.256372042367196,0.397198417328216,0.0986316007058157
"BC016579",22.1408626889094,-0.849502980174909,0.474007390914869,-1.79
217243540297,0.0731053486428234,0.155323971650623,-1.58198030183742
"Tie1",3450.9207859395,-0.678055353988996,0.192625217595461,-3.5200757

3283059,0.00043142359448098,0.00307340052801979,-3.92253033311668
"Lpcat2",666.300531061858,-1.1777672172362,0.351501300238812,-3.350676
70144041,0.000806143589478451,0.00498759665537849,-6.24310978119739
"Myh2",0.639290963518614,-2.1265557365812,2.87123215351831,-0.74064221
3126302,0.458910405776468,NA,NA
"Tpsg1",0.132294999522605,-0.470076037063128,4.78471990596623,-0.09824
52570477477,0.92173754633477,NA,NA
"Mamdc2",107.599326217883,-1.37944791744613,0.324945849332433,-4.24516
244869741,2.1843511123318e-05,0.000307512476449426,-11.1555882700594
"S100b",84.3709660338109,-1.43205231429783,0.514614315231465,-2.782768
12733768,0.00538973100676595,0.021043046690012,5,-5.52941905400152
"Ttc28",2274.96764737035,-0.661943008025694,0.209029068797033,-3.16675
097791514,0.00154152261442358,0.00822245592804336,-3.17791314498129
"AA467197",150.647434418898,-1.42534633289657,0.39744464917206,-3.5862
7631763514,0.000335433529359876,0.00252423116767999,-8.5261634203169
"Slitrk5",4.27152993270479,-1.55843266712988,1.34193191612166,-1.16133
512319606,0.245505641218948,0.384652134925474,-1.48895134736318
"Eefsec",1163.85859403813,0.655849195922747,0.128663873600532,5.097384
196274,3.44378952146271e-07,1.19478662080925e-05,7.43402299536281
"Gm9758",4.65928813721771,3.75442887587005,1.09888713178853,3.41657370
194099,0.000634144958315167,0.00414084340161726,20.600009822077
"Rac2",1665.46517495567,-1.38579280153528,0.502021888694529,-2.7604230
6668925,0.00577265524077365,0.0221391093400088,-5.28043816832855
"Ttf2",526.451338028716,0.0354669387277995,0.243676590925592,0.1455492
24047662,0.884277254320871,0.927951660474155,0.00265206295563602
"Wnt6",48.5694594610836,-1.63488430790286,0.410657173229773,-3.9811414
8364847,6.8585108305128e-05,0.000740460931192351,-11.7846346799615
"Scaf11",4062.70758682001,0.266632950300088,0.0810192195168287,3.29098
394047977,0.00099837612708794,0.00586688900722141,1.37007495541557
"Trim45",184.906552023145,-0.399786896916391,0.22582672384325,-1.77032
589461773,0.0766728667796648,0.160835670410963,-0.73055942773686
"Arid2",3057.84185557273,0.390060038552902,0.205199629118332,1.9008808
1654361,0.0573176253263783,0.129110334867574,0.79848719734081
"Hsf4",178.599549107822,-0.248027332121879,0.263176172920924,-0.942438
403025198,0.345968233493259,0.490702598141526,-0.176574884404718
"Szt2",2137.49823347138,0.159948412108156,0.181837414482345,0.87962322
0355926,0.379063455452426,0.523922822315971,0.103392395611297
"Gm5134",7.67298865253724,-0.298624033915902,0.687994062157171,-0.4340
50307032566,0.664251909928615,0.77244490605285,-0.0771031105323133
"Shf",424.648259775714,0.657498601007528,0.14351394357934,4.5814266168
7807,4.61814658165897e-06,9.34072842833886e-05,6.10062787521241
"Ttll4",2288.71477649154,0.680624012633054,0.209164831774921,3.2540078
8869452,0.00113789119901286,0.00649001998870535,3.42863643166796
"Duox1",58.1824143619674,-2.25426655111355,0.898182310792975,-2.509809
56096021,0.0120796287024207,0.0391470797400074,-7.30479172240284
"Slc35a4",2916.71305384151,-0.338239470181441,0.206663930863763,-1.636
66426341428,0.101700639914064,0.199854774949045,-0.544621120225998
"Stk36",387.431338112182,0.103520875611708,0.245883331092839,0.4210162
40310415,0.673743222931732,0.779576604398725,0.0257771453978784
"Ptprm",856.647608197875,-0.989263328831712,0.262342226303996,-3.77088

866999771,0.000162667235875399,0.0014489199475096,-6.4667519223296
"Rpgrip1l",500.639678306894,0.261965817201899,0.294354829608754,0.8899
66091434936,0.373484093715975,0.518669750235761,0.17197739385523
"Wdr3",1010.74331906071,-0.134988317730018,0.190045678284059,-0.710294
066925596,0.477521798268783,0.614974902420392,-0.0656277862320806
"Kctd17",2211.37480147909,-0.529676599619272,0.285442149808318,-1.8556
3554637941,0.0635055234588438,0.139314884833544,-1.04400240302886
"Noc4l",726.587277191961,-0.166908868502729,0.13960854969721,-1.195549
04670759,0.231872585484877,0.368975081350211,-0.166412509474707
"Ptprf",6691.32964708481,-0.166002133225656,0.208513208517484,-0.79612
2866296678,0.425960624649155,0.568862681810383,-0.093644493662199
"Lpp",5348.88706367471,-0.190892475936435,0.103502617263999,-1.8443251
0966881,0.0651357742368005,0.142012448403124,-0.372591677210297
"Mif",5284.33259897489,1.07993258683954,0.24172246058595,4.46765511248
61,7.90816669807769e-06,0.000141461866311118,9.57196128787029
"Dpyd",1773.41100158028,0.496627010370453,0.35565827801714,1.396360048
58158,0.162606099463356,0.284732261098924,0.623865817773491
"Fbxl8",332.013533893463,-0.081016307734513,0.169220432904533,-0.47876
1969485204,0.632107974665067,0.747477804124188,-0.0235798504556554
"Galnt9",29.7222365143887,-0.822365507381484,0.579903848194804,-1.4181
0665671808,0.156159628094377,0.276573751347458,-1.05696809868342
"Gstt2",7041.04578116427,1.76686405966297,0.300367324586946,5.88234443
307938,4.04495470595479e-09,4.12924224380663e-07,25.9729047459031
"Fem1c",1727.25516584978,-0.256600827307991,0.18761053545459,-1.367731
43728967,0.17139615577595,0.296323570463898,-0.312104427455142
"Ctdp1",857.67178151338,-0.123766060319866,0.169236858272012,-0.731318
588536658,0.464584579024524,0.603455474107,-0.0625121355951074
"Kdm4a",3147.25082386419,-0.033284552294657,0.164252625746717,-0.20264
243657196,0.839414518486999,0.897124821651609,-0.00361338005156647
"Tnxb",2246.00702167402,-0.59868622002363,0.240214941821755,-2.4922938
4102122,0.0126920987617942,0.0405755929918778,-1.9185430063159
"Dnm2",4773.48962990711,-0.0576975094027204,0.136273372733183,-0.42339
5328415988,0.672006852706769,0.778306481382631,-0.0144610093514997
"Plppr5",8.71615871796973,-1.30626976935129,0.585495776188444,-2.23104
900577602,0.0256778830527911,0.0699451472147696,-3.47473500399858
"Chst2",659.439870527364,-0.455154435174935,0.198599944776094,-2.29181
551731087,0.0219162908758081,0.061859270338232,-1.26664623137602
"Map2k4",1961.76950408874,0.0542551639698108,0.126971628360941,0.42730
1474118142,0.669159759263854,0.776161629156848,0.0137479599019273
"Rtp4",907.159809734066,-1.24467624914423,0.221136341576429,-5.6285468
0633328,1.81734189685302e-08,1.27557867205757e-06,-16.8928837418123
"Pus7l",232.040610471817,-0.240904594865295,0.189888777104011,-1.26866
157410314,0.204561793014366,0.337126687532254,-0.261934720874172
"Prrg3",197.484935346536,-1.69658860191852,0.37236700428665,-4.5562270
0826756,5.20806498550383e-06,0.000102558077974414,-15.5833042505495
"Usp37",992.138103873232,-0.0701423870241037,0.103122983080093,-0.6801
81904451176,0.49638928871279,0.632092368816744,-0.0321756977016567
"Ipo13",2109.2354905115,0.348088251916427,0.224459076502306,1.55078715
167418,0.120952700660808,0.227946622606267,0.514698531654674
"Trim69",17.1884946917227,-1.07007864990427,0.493762024124865,-2.16719

512157878,0.0302199792160684,0.0790911617120673,-2.71495448323241
"Fntb",552.243389012328,-0.48069537072294,0.147816156530506,-3.2519812583798,0.00114603578319427,0.00652992568694386,-2.41855132392894
"Palmd",861.157274062913,-2.432525624383,0.578034122326548,-4.20827340536964,2.57329398584988e-05,0.000348431153722924,-19.3679390489729
"Atp6v0b",10830.9988013151,0.595481496644414,0.180316691136449,3.30242027452578,0.000958543323069717,0.00568309307721275,3.07879394764475
"Trappc8",2269.01639314073,-0.0549187340999556,0.141670358633772,-0.387651549904836,0.698273914863598,0.798811593053886,-0.0123364042727423
"Rtp1",13.467212016635,-0.617812445660679,0.671967759728353,-0.91940786848231,0.357882275217344,0.502990600604612,-0.424550701456948
"Frrs1",3922.86152136825,1.00153515954793,0.265516459668391,3.77202664120623,0.00016192696177011,0.0014443055354957,6.55016680911898
"Arhgap44",486.419755896418,0.170124617993678,0.257182572769984,0.661493569184535,0.508295837370023,0.642762321618828,0.0751917233801834
"Clasp2",1881.11912699563,-0.247097159796129,0.095504487602215,-2.5872832366298,0.00967360390033797,0.0329575273193762,-0.843227853821976
"Spg11",1101.19096180811,-0.255504731631638,0.142862857566731,-1.78846157765179,0.0737015692527005,0.156284197638842,-0.474237004960777
"Agl",2128.72510417758,-0.19694798301201,0.345291978821658,-0.570380996639753,0.56841931570854,0.695299344328008,-0.0715734208920212
"Nudt15",143.084645798311,-1.11145591808446,0.382089589514516,-2.90888825182775,0.0036271648536917,0.015615860669339,-4.6230655154674
"Syce1l",4.98102794362967,0.0206475664805202,0.817436312562514,0.0252589298557019,0.979848432697305,0.98698583151997,0.000270474753426642
"Ctdspl2",676.819018986405,-0.293768327717341,0.152913093739642,-1.9211456686471,0.054713345499455,0.124422767733657,-0.612233786670388
"Gucd1",4150.215126636,0.404093554447295,0.243231007591173,1.66135707140803,0.0966417541223689,0.192218802598995,0.666399150008444
"Cacul1",2846.84216625639,0.216373614726597,0.105021547241009,2.06027829917656,0.0393719432793016,0.0967819150352776,0.505296248796889
"Snap91",56.5163757839664,-0.655378102565372,0.414672592087414,-1.58047123217446,0.113998989952023,0.217819506296288,-0.998854237073698
"Antxr1",920.128890427078,-1.56969670848187,0.288214746945963,-5.44627478335162,5.14356569760477e-08,2.78581233255272e-06,-20.0779451488293
"Eri3",2551.65829822655,0.0223397212321434,0.152146608290902,0.146830228311302,0.883266024521086,0.927455487431317,0.0016824150763371
"Upb1",3711.57794621947,2.21193004155676,0.634483688346783,3.4861889788218,0.000489954751221922,0.00340575320475046,12.5688260941362
"Mcee",2102.25123267466,1.05370876026388,0.247204342289426,4.26250101638668,2.02151579023147e-05,0.000290844253564796,8.58005818363251
"Terf2ip",1420.98769274491,0.0959051483811403,0.147637769578406,0.649597651434365,0.515952150523698,0.649579326590757,0.0413763884213289
"Gtpbp6",1590.37950838289,-0.144119598672053,0.172626596778441,-0.834863232906254,0.403794718181673,0.547627378078758,-0.0867830849660853
"Armcx2",934.088668711787,-0.707157585804864,0.238274214294146,-2.96783094175641,0.00299909234197242,0.0135416393905845,-3.04218199237101
"Trmt13",538.3662703168,0.0797891135257116,0.224717612542483,0.355063907198762,0.722541715113899,0.816797214824374,0.0161464778405163
"Specc1l",2322.16951841716,-0.494288707016228,0.102911269210081,-4.803

05714631889,1.56261264309902e-06,3.90112948977525e-05,-5.0178505697328
1
"Lpar6",1801.30647162905,-0.535465177884463,0.191154760929167,-2.80121
287736527,0.0050910922882997,0.0201596501700381,-2.09049470894581
"Tagap",386.305841999035,-1.05448447080016,0.359615558275723,-2.932254
86643621,0.00336510461969363,0.0147577170208696,-4.44569508262883
"Adamts15",950.324278401837,-0.0689770423677536,0.248598894408318,-0.2
7746319038113,0.781424466452173,0.858793869499128,-0.0105001234936624
"Zbtb1",1405.68757797033,-0.262484602570949,0.123011606740758,-2.13381
980388343,0.0328575341467827,0.0843234613130906,-0.64914939633508
"Fan1",171.832078869353,-0.234430277424471,0.255730498179327,-0.916708
328077789,0.359295500888629,0.50439510953463,-0.160442996585533
"Armcx1",767.194490668025,-0.296869520867391,0.143249795601829,-2.0723
9053724416,0.0382290351299453,0.0947368742963489,-0.699618142511976
"Crlf2",976.389026130961,-0.79241736985799,0.220584869160734,-3.592346
89520149,0.000327713200046472,0.00248049377892318,-4.75394765230339
"Cysltr2",72.7346393639567,0.0663847886242282,0.358145338339904,0.1853
57120469413,0.8529489384906,0.906427978408292,0.00652188739939357
"Tomm6",3989.85886221167,0.203625991397316,0.304097854717516,0.6696068
00042932,0.503108478062145,0.637940372325921,0.0915320131239312
"Fam160b1",1450.73315415322,-0.19235032478066,0.0984210226422203,-1.95
436218418387,0.050658399569868,0.1176017948343,-0.411716441456615
"Catsper2",209.392239804311,0.296074128367187,0.275196736926019,1.0758
6351376972,0.281988301798396,0.425920513476267,0.252700020058633
"Fndc3a",2987.27741638346,-0.14950144530183,0.0908337749479232,-1.6458
7946925626,0.0997885766548989,0.197010699434712,-0.242864685249048
"Cryzl2",8422.92198920668,1.86687003308422,0.424206798233233,4.4008489
2759733,1.0782818109812e-05,0.00018155732945174,16.081104752788
"Prss35",63.3045756579364,0.0745390654640255,0.992493456800853,0.07510
28280874421,0.940132897693661,0.962848578591817,0.00282198333525729
"Strc",3.74968212271689,-0.721428120700018,1.07280704489952,-0.6724677
32319551,0.501285975672406,0.636457247747644,-0.325968661535037
"Larp4b",8175.44920271645,0.937003286524966,0.161155603108847,5.814276
81352226,6.08965728199145e-09,5.54841764879763e-07,13.49714149225
"Crygs",5.79687603465926,0.0236169632910384,0.767717161854095,0.030762
5834936419,0.975458880334107,0.984649888628827,0.000365334206258881
"Cdc14a",1649.21933229275,-0.298258534663069,0.314604932771584,-0.9480
41507281823,0.343108323946572,0.48771058542759,-0.214159503737766
"Asprv1",47.3030414654786,-0.175543979543125,0.386367440736299,-0.4543
44649768085,0.649580784039593,0.761149406682932,-0.0479104478703209
"Otud7a",3.2109291998443,-1.05566207559316,1.53434402846036,-0.6880217
57840364,0.491439079895277,0.627839023705813,-0.491380585593242
"Idi2",2.41089270901206,-0.232443879183931,1.00941317460762,-0.2302762
48647426,0.817877115545692,0.883194948987035,-0.028871696744988
"Ppip5k1",3069.78752150118,-0.130526960645227,0.293065589834913,-0.445
384805219726,0.65604169110251,0.7656892142228,-0.0348479466090888
"Ttc7b",1486.9939871816,-0.11066037318645,0.358732384175557,-0.3084761
17763304,0.757720070046376,0.842854396336032,-0.0189186143384587
"Acsm1",4311.2878036069,2.41387103735613,0.944989428123836,2.554389462
4817,0.0106374199112044,0.0355943733359641,8.05163027859662

"Casp4",991.433227859133,-0.781784187164095,0.337145576841022,-2.31883
269681079,0.0204041095711481,0.0583513242033214,-2.22126247978311
"Idua",876.303419758097,-0.483382457965898,0.15807538831966,-3.0579235
8383078,0.00222876402864911,0.0108695450301652,-2.18575417452681
"Arhgef5",3339.32063876893,0.42093545126759,0.14557305512966,2.8915753
0487127,0.00383315717658815,0.0162511195105709,1.73408294038337
"Gtf2a2",2440.48114019552,0.276850012874059,0.148037412905208,1.870135
44374309,0.0614650116254882,0.136024227674809,0.552294254594138
"Angptl1",41.8147824017612,-2.15790878103862,0.918909693162781,-2.3483
3607382174,0.0188574978354974,0.0549427234639161,-6.26109470375893
"Znrf1",4251.99956921712,-0.0358950155373484,0.199908254919124,-0.1795
57445248423,0.857500015371466,0.909560183859909,-0.0034026360780399
"Dph5",702.826689327081,0.283838084006891,0.187251675883389,1.51581064
718294,0.129567239029783,0.239550732657189,0.40560179989622
"Fam20b",3730.32691424889,0.0864371014945206,0.236344725839624,0.36572
4689592498,0.714570520956763,0.810653169117063,0.0181444419682323
"Rbfox2",2045.55153674264,-1.15661698325527,0.236004614399594,-4.90082
359702058,9.54357207577045e-07,2.67741824940104e-05,-12.1769474249874
"Adgrb3",25.0442408602344,-0.731186846284964,0.589352722334447,-1.2406
6084464445,0.21473106466484,0.349513840790971,-0.76863247216815
"Apol6",1143.24828568485,-4.14959793963422,1.39469425262268,-2.9752742
8096231,0.00292726687495875,0.0132765409462539,-17.9335524480187
"Myo6",13463.6141966011,1.36994293985568,0.356779174353674,3.839750294
6673,0.00012315951192943,0.00116911595111978,9.24917991397816
"Tmem35a",425.410392653885,0.0430985323986975,0.486084553146761,0.0886
646821415966,0.929348401518562,0.956303244594089,0.00192565168385555
"Fa2h",399.800746028466,-0.36513864411589,0.444810160399449,-0.8208864
73879077,0.411710936293793,0.555242335307695,-0.214829547776146
"Igf2bp2",416.498806264288,-0.0761881397488318,0.186708973210596,-0.40
8058265431606,0.683230899050062,0.786972027583835,-0.0182518268887422
"Ndn",352.746445019282,-1.82099921724913,0.317550660927512,-5.73451559
486696,9.7791427298114e-09,7.84665534756636e-07,-25.5996220745654
"Reep4",1237.75556239653,-0.940715359828315,0.284548962644585,-3.30598
766231776,0.000946422548707269,0.00563117084366626,-4.8723767815277
"Myo5c",638.365908352251,0.12014225646463,0.266165952630574,0.45138100
9769428,0.651714967730238,0.762779184873292,0.0325329243679357
"Spata2l",483.517104722258,0.725073265412517,0.250213120781949,2.89782
271667676,0.00375762928505916,0.0160214860704316,2.99732568555547
"Lgi3",25.7239776799635,-1.57062829150341,0.834234191563111,-1.8827186
7466918,0.0597385032767742,0.133133082282609,-3.16702436161309
"Rfwd3",1865.51648044055,-0.0820333238593634,0.135606737315501,-0.6049
35458837162,0.545221881559797,0.675095970448525,-0.0322309273797795
"Caskin1",81.2436771844822,-0.350261501800337,0.36123824227657,-0.9696
13570238146,0.332239146191726,0.476989829353233,-0.259284618002704
"Pank1",7490.00445774969,1.68800651469078,0.424583349140952,3.97567760
984052,7.01791442602861e-05,0.000752454088464517,12.1404307857216
"Cplx1",4.84749338343022,0.404517504888799,0.923444974335557,0.4380526
3565364,0.661348127970087,0.770031274500517,0.10571019260934
"Map3k13",923.418260900064,0.325916898993743,0.282105806847297,1.15530
021390223,0.247967508402313,0.387316388594753,0.309136538932085

"Pcgf3",1949.45091834588,0.0119565037676939,0.113796303234815,0.105069
351356889,0.916320778735618,0.948726770591923,0.000629323817956323
"Pdpr",406.152481247047,-0.905216047646674,0.459487356263649,-1.970056
48862135,0.0488318968965893,0.114365444848718,-1.96283092084627
"Pik3c3",1231.84844667112,-0.151536017083349,0.150978448342423,-1.0036
9303531098,0.315526595115772,0.460771351898851,-0.117418189253961
"Hacd3",3137.72355542467,0.106867002787929,0.1449597567634,0.737218419
608385,0.460989533769118,0.600265388635653,0.0545431449964588
"AW554918",449.12411674634,0.0710152572690323,0.152398506338999,0.4659
83945479518,0.641226992251892,0.75476268608073 5,0.0199802777189182
"Clec18a",147.385897722169,-0.114318003446222,0.553889764627714,-0.206
391254626376,0.836485301577206,0.895748594582182,-0.0125858968090341
"Nat8f2",1376.54712213751,1.41338712582764,0.56922406050416,2.48300664
693583,0.0130278656638848,0.0414654464111045,4.49866255461506
"Piwil2",29.9292506492086,-0.809750735920008,0.50361331020159,-1.60788
191955426,0.107861042409442,0.208596965373865,-1.26916385574986
"Vps8",2192.63061520589,1.43479656659972,0.268554326169201,5.342667858
18129,9.15884915491337e-08,4.26203438717186e-06,17.7423556674547
"Ddx19b",889.081622212153,-0.114852954758004,0.149022211592486,-0.7707
10309091904,0.440878660023135,0.582596758971309,-0.0620504570521495
"Zfp7",1348.79166900069,0.525604533965509,0.210019376250971,2.50264781
920606,0.0123268137662538,0.0397429311221036,1.69524454320547
"Cep350",1995.56921975878,0.301072706705528,0.155073343563482,1.941485
87879179,0.0521993706151058,0.120191701494253,0.637872898141887
"Gabrb3",313.364280360667,1.41141917646878,0.496325700736537,2.8437358
2583829,0.00445879995519825,0.0181773578262783,5.65637319533843
"Qsox1",1814.91731172491,-0.666375645798793,0.214524563770544,-3.10629
064609846,0.00189450404955498,0.0095796265451823,-3.09739174806377
"Ucp2",9877.98204017836,-0.488942046439388,0.243287051228027,-2.009733
12788898,0.0444594417674933,0.106322536162412,-1.0958550572467
"1300017J02Rik",9.15732337257568,0.108352622723624,1.05241678876444,0.
102955999828578,0.917997892358643,0.949731665874555,0.0055883722520874
6
"Arhgap39",971.790303835368,0.286661852757651,0.240746200919715,1.1907
2222806643,0.233762648780315,0.371346270646781,0.283973052758273
"Acbd6",1929.5648298934,0.0584570511818966,0.242184650307311,0.2413738
9016075,0.809265347209998,0.87822551595972,0.00759075720240049
"Fcsk",1076.91224461219,0.413491745847416,0.189119473186256,2.18640491
579724,0.0287859942895261,0.0762283466871084,1.06433680171274
"Stard9",1689.99824732737,-0.505138200578037,0.245608023704077,-2.0566
8443953873,0.0397165899080317,0.0973713152303285,-1.17657982639823
"Smyd5",864.983237901318,-0.276504841391119,0.213020428098629,-1.29802
030659284,0.194280357133401,0.324655331109621,-0.311065507533496
"Lrrc24",50.8601663872247,-1.12545011423162,0.52745704334583,-2.133728
4778539,0.0328650135159484,0.084327152721932,-2.78329594629391
"Ccar2",2587.12163111214,0.0705240264328775,0.0715909129873377,0.98509
7458463075,0.324576201662124,0.469444906164831,0.0533305743678213
"Foxn3",4701.12239786542,0.352505780706442,0.220792620027922,1.5965469
3468406,0.110366735678824,0.212284760014374,0.546322870135184
"Akr1c14",4636.98026454782,1.96869228586328,0.676014867732126,2.912202

6449918,0.00358889721900029,0.0154902300360012,8.20461521635483
"Adra2a",142.133405755845,-0.138603854731103,0.410159840727339,-0.337926439812625,0.735418622326166,0.82703842992938,-0.0263214425010582
"Sfxn5",1282.71942041567,0.259846492957708,0.386794649810307,0.671794434295155,0.501714572465942,0.636877000907614,0.117237211532456
"Vav3",825.815305114326,-0.0445025060013817,0.201982384606711,-0.220328649392048,0.825615209343878,0.888500129803893,-0.00526110785628901
"BC034090",4184.34621622075,1.36646102189924,0.410134110849102,3.33174194916451,0.000863042423545873,0.00526355995442522,7.1697494937716
"Emx1",1185.08212104458,2.08571531945395,0.811499898742476,2.57019788010576,0.0101640439079023,0.034275082690467,7.03581990976201
"Lrrc14",1030.89517819915,-0.0535142693870175,0.118734178622471,-0.450706527874946,0.652201076898132,0.763134120245071,-0.0144660566627609
"Egr3",168.649584835988,-1.65173479442597,0.31567982882531,-5.23231021941537,1.67404491889098e-07,6.70236711058241e-06,-19.6771988522744
"Sf3b3",3200.19563462963,-0.426189282082075,0.121553546154368,-3.50618550890182,0.000454578276392287,0.00320814928903259,-2.44720487809635
"Spr",5078.68691477676,1.0094980326221,0.348479293068942,2.89686662220811,0.00376909960227597,0.0160558635928748,4.17092400570703
"Fndc3c1",1.33700849301298,0.67234609126234,1.46168938364309,0.459978774414162,0.645531455888429,0.758135906933658,0.186167665824901
"Fkbpl",507.125130165151,0.239360572666647,0.264956965009557,0.903394151793719,0.366316742725878,0.511233544461325,0.160593891976557
"St18",7.49049938511059,-0.609865417327772,0.754710435340528,-0.808078686566192,0.419045286206771,0.562017703451704,-0.351417827069342
"Gadd45gip1",1650.48032906484,0.689420417652754,0.185659957158941,3.71335008476033,0.000204533644211932,0.00172491219500184,4.38649196200993
"Mnd1",166.317565233744,-1.07080243029665,0.428818545768916,-2.4970991596844,0.0125213937487594,0.0402251116528057,-3.44077069736139
"Rbm4b",1320.18703024286,0.377663229108615,0.126553407112881,2.98422016225729,0.00284302124634185,0.0129729062160481,1.64090603106426
"Recql4",179.657494762976,-1.69894139851148,0.485498526044357,-3.4993749875077,0.000466350227211429,0.00326694747661703,-9.72456935160745
"Mtss2",1502.54941155074,-1.13298943223734,0.212955752357111,-5.32030442801753,1.03593757834283e-07,4.69737462849499e-06,-13.9000885480665
"Calm4",0.247490694217328,-0.064973835720255,4.78471990595238,-0.0135794439376535,0.989165504321392,NA,NA
"Tmem131l",1159.19192323076,-0.46463666548624,0.325180172951161,-1.42885914989665,0.15304472060107,0.272318485333059,-0.604391473642054
"Nrxn2",394.926012063304,0.0802589608256899,0.510979170465272,0.157068948138551,0.875190509583112,0.922100125725339,0.00650911930958848
"Exoc6b",1128.69525468915,-0.0556237234234503,0.184124712906465,-0.302098086375331,0.762577291188252,0.846115505390862,-0.00929469067079727
"Clcnka",1364.23900896595,1.9050078224954,0.604728730682765,3.15018573756957,0.00163166693172275,0.00859147767613561,9.06209275056402
"Rpap2",682.682493203148,0.203831389065595,0.197647725269904,1.03128628871011,0.302406586230507,0.446928190580036,0.164157106273698
"Npbwr1",0.797662443327109,1.03664520406992,1.4085611793244,0.735960368130502,0.461754817003593,NA,NA
"Tlr13",275.495798237581,-1.06464916172272,0.408009305660555,-2.609374

70531239,0.00907078570402403,0.0313529834527673,-3.68629007221716
"Asb13",2745.75919988198,0.325819263867312,0.192034369022589,1.6966716
1938593,0.0897587611608812,0.181672031196057,0.555701776595851
"Dysf",2533.35133317022,-0.0525343102335242,0.188436511746069,-0.27879
0504805765,0.780405601740964,0.85803250382008,-0.00804370144398058
"Tubgcp5",640.544925469783,-0.0587766168762312,0.173650338039327,-0.33
847683534552,0.735003880743948,0.826648853340777,-0.0111896147249001
"Atp7a",184.761723266534,-0.141467142551925,0.378132839816922,-0.37412
0223518322,0.708314873246223,0.806418509783654,-0.0304369992486276
"Atp6v1h",5389.80507887467,0.559496165800321,0.200830741127824,2.78590
898314823,0.00533778569747468,0.0209072208777343,2.16394132283306
"Lpcat2b",0.0965030460389225,0.886496493659922,4.78471990596623,0.1852
76570224,0.853012114121315,NA,NA
"Tasor2",1194.1864986408,-0.3873872162228,0.195428525239151,-1.9822449
959582,0.0474518268749857,0.111808546272927,-0.848752714919197
"Ephx4",29.8746941704641,-0.551220069968621,0.409834786722674,-1.34498
11675982,0.178631316289573,0.30570994514299,-0.653261112552392
"Tmem87a",2756.03106962057,0.150484790139621,0.0985656457939239,1.5267
468591871,0.126823970346356,0.235865487773106,0.21737431678262
"Alg3",536.717785329107,0.287490054708652,0.124156865290424,2.31553892
759909,0.0205834567409184,0.0587178111974291,0.815037804030881
"Tcea1",4239.50891353662,-0.0307779474640467,0.105377280449312,-0.2920
7384488207,0.770230163962008,0.851274060036913,-0.00495590072789858
"Ppp1r16a",3093.71946519741,0.572137030411279,0.155138159196705,3.6879
1942210586,0.000226095219709667,0.00186480735782533,3.595650965062
"Tpsb2",6.39671631892438,-4.02157748251486,1.55608902083935,-2.5844135
0633374,0.009754476739106,0.033140405551331,-13.7015226448848
"Dnah8",43.509404163001,-0.983090734568151,0.466092766126444,-2.109216
88988722,0.0349258646885278,0.0882786123464013,-2.38621427656683
"Fgb",122.742888324446,0.321783538445387,0.490208791867581,0.656421393
870696,0.511553028935828,0.645750418114125,0.140729520473142
"Foxh1",2.28145060646723,1.07679333444006,1.16536762874989,0.923994547
192935,0.355489154803422,0.500492998798261,0.745315071253888
"Mrpl15",1736.21421585236,0.151916028953481,0.273560722477787,0.555328
36577377,0.57867008018223,0.703554855857899,0.0534151083230573
"Pla2g4c",49.5291604299593,-0.784543078778304,0.324853614107776,-2.415
06649366695,0.0157323373621554,0.047835303886146,-2.38500416304668
"B3galt2",1502.07088149752,-4.00501817809212,0.817783123737372,-4.8974
0869166959,9.71087327124965e-07,2.72043975636244e-05,-42.1012794405038
"Gm28042",372.476039483269,-0.459372762541239,0.277282461159997,-1.656
69606587981,0.0975809388034209,0.193612816199155,-0.754241799210281
"Kcnk10",11.6452596152044,-0.642072634304262,0.728684219616469,-0.8811
39754394855,0.378242182418554,0.523177036858622,-0.415957362353613
"Ston1",239.184468402469,-1.44304581340159,0.343997623939819,-4.194929
59536849,2.72956358011363e-05,0.00036254108096087,-11.4323468482896
"Engase",697.787624729102,-0.371405422030563,0.182234939800024,-2.0380
5824743666,0.0415441080560012,0.100758537102215,-0.852385971507127
"Fgg",435.244145474519,1.16983012387288,0.670954408029925,1.7435314678
2025,0.0812408225229091,0.168227862951538,2.08514719971044
"Cdk10",2499.82578572945,0.174932526092803,0.141360581609152,1.2374915

5600162,0.215904647258435,0.351072371966055,0.183112876363688
"Klf9",13106.572861154,1.23488779704403,0.186447590648002,6.6232435224
9419,3.51401386547972e-11,1.10510068305038e-08,22.6240635654389
"Ppargc1b",757.065442091034,0.510572873488746,0.430005082698532,1.1873
6474063191,0.235083781572916,0.373036775227565,0.503464816357742
"Best4-
ps",0.363706797611643,-0.591314394033538,3.15189705730233,-0.187605871
41118,0.851185623253044,NA,NA
"Lgals3bp",4804.93915048653,-1.23671609464243,0.245351548601698,-5.040
58809365865,4.64103426784156e-07,1.49078889875411e-05,-13.744392752032
9
"Rbm46",10.8054072198623,0.368896289872999,0.658803558193472,0.5599488
4254202,0.575514332269072,0.701117054081997,0.130987851136475
"Zfp267",733.474417637415,-0.0304086665681711,0.166742327062768,-0.182
369210648741,0.855292986943279,0.907910641301179,-0.0029377605350221
"Pxk",2330.5485021749,-0.0957220428409474,0.11617587390223,-0.82394080
3075036,0.409973194556942,0.553513680875572,-0.056616602780421
"Cfhr2",35.0410730881196,-1.35308559871217,0.516246494062396,-2.621006
85287876,0.00876704996386167,0.0305522681314343,-4.71999062081464
"Map9",640.116473419186,0.803485189781606,0.199210794397569,4.03334162
795453,5.49892455724977e-05,0.000621482488983404,5.932454818327
"Mapkbp1",1500.22644730537,0.00760187925281763,0.198041892150919,0.038
3852081509328,0.969380554476441,0.981104700294921,0.000145014185875523
"Ccp110",1071.3187850534,-0.00388379646893092,0.150497998468824,-0.025
8062998076048,0.979411837003793,0.9868009506976,-5.16037353617723e-05
"Zdhhc15",228.282231010555,-0.712226546277723,0.226186205874874,-3.148
85049476326,0.00163914029816771,0.00861686641511838,-3.38594906381035
"Usp36",2252.73706077,-0.122690277503803,0.194193234040496,-0.63179481
0514449,0.527520965096687,0.660052863855175,-0.0509698784277938
"Gucy1a1",3260.4773477617,-1.39921874033168,0.358977985933401,-3.89778
4251849,9.70768429743127e-05,0.000967196566763314,-9.71212952330464
"Chmp2a",5975.9442646736,0.223955565784269,0.145419445311272,1.5400661
5349749,0.123544228994319,0.231510367797332,0.327676246077512
"Gde1",5137.7103172781,0.168592977173372,0.316891173389167,0.532021688
613987,0.594710974067297,0.717509719223078,0.0559676058734769
"Parl",2417.29397596483,-0.146465690680779,0.277791515242386,-0.527250
41134889,0.598019705396499,0.720120319652973,-0.0480901011316587
"Rbm34",1436.62853836909,0.760438503084231,0.139702701696164,5.4432626
8462644,5.23134133849445e-08,2.82550397279681e-06,9.71598877363638
"Vhl",1209.82234510666,-0.120338067310732,0.128169793621861,-0.9388956
9383068,0.347784300327998,0.492632933555417,-0.0851982612658101
"Ndufb7",6306.13175095214,0.715739951196373,0.25049202545353,2.8573362
76077,0.004272129675165,0.0176329348870441,2.89014849322828
"Brk1",5604.62405118357,0.128147059504844,0.151232468638114,0.84735150
2351581,0.396799227845538,0.541299331458217,0.0786544687151256
"Mga",2493.26099751386,0.0714480443439622,0.252497978248522,0.28296481
7538615,0.7772038067789,0.855911879388591,0.0111164478047398
"Zswim5",343.864600737537,0.975003937557458,0.34730637343473,2.8073309
6808744,0.00499538885269705,0.0198775697652831,3.82022467516576
"Trim36",230.571567375755,-0.31467194125084,0.236112796761666,-1.33271

870718838,0.182624131831221,0.310743112177777,-0.367785013544236
"Aspm",111.110790651033,-0.857549104762774,0.431669450892139,-1.986587
42931764,0.046968135400023,0.110923534887312,-1.88567689503511
"Ppp3r1",3162.17747527371,-0.397456419236196,0.202106211104069,-1.9665
7201708431,0.049232572157013,0.115017576314095,-0.859567203276822
"Tnks1bp1",3719.58107648588,-0.642365859762947,0.214691895034975,-2.99
203591108225,0.00277123662227216,0.0127497809488113,-2.80215481122982
"Jcad",1471.16784131433,-1.23306022473414,0.258333592391833,-4.7731315
6727939,1.81383212707062e-06,4.39330420045005e-05,-12.371100699485
"Zfp446",544.068607670388,0.13875637469717,0.157162955612175,0.8828821
91650648,0.377299926347916,0.522549565232802,0.0900578044203046
"Fancd2os",109.149837890472,2.77284629516815,0.912618834950044,3.03833
998267188,0.00237885405167333,0.01139841097336,12.4064943861355
"Zbtb41",1524.26440685112,0.155416132185713,0.170692336713065,0.910504
450161513,0.36255653589629,0.507438614378426,0.105431123059647
"Slc16a2",5462.91187975727,1.35786977938101,0.291088981805269,4.664792
77559666,3.08928198803529e-06,6.79084782443203e-05,13.0319510756612
"Cdkl4",18.3640124884433,0.202367713066069,0.416456627782988,0.4859274
6414765,0.627018595302441,0.743559699015144,0.0599628124591939
"Rnf225",29.195619525211,-0.891194536161307,0.411608874864469,-2.16514
898143232,0.0303762779371672,0.0794144732322045,-2.25746228315145
"Rfc3",505.592844379612,0.0545711612981507,0.197241194811539,0.2766722
30414608,0.782031796707141,0.859027182131512,0.00829234518443994
"Zfp944",833.979753913698,0.463142010656257,0.126027392604746,3.674931
30726577,0.000237913417085605,0.00193526733681065,2.89348425198069
"Gria2",33.5196647175895,0.588451782177636,0.504396076156268,1.1666462
3298007,0.24335326440283,0.382191055120933,0.565993314606118
"Coil",530.030546014612,0.147797229069327,0.136222087051987,1.08497257
873404,0.277933800417246,0.421559956478838,0.12766251226418
"Tesk2",600.193798869973,0.725175193183612,0.221743488750915,3.2703336
5114141,0.0010742070106908,0.00621879106129726,3.68402033470116
"Dnah17",28.9610627553682,0.457976357243148,0.485900433107595,0.942531
280151662,0.345920704153107,0.490670878042869,0.326070778717276
"Ttc37",912.200364341912,0.105071793772617,0.114571719900296,0.9170831
49873756,0.359099070040664,0.504191954176775,0.0719529775534504
"Kcnk1",2192.09645412683,0.496084555800458,0.232802271432943,2.1309266
1315958,0.0330951885100688,0.084813450231217,1.22398998884474
"Pqlc1",1787.97765737823,0.0916672337709694,0.180465510333626,0.507948
7687232,0.611489272037386,0.731102393069962,0.0287103386252912
"Scaper",838.03493662737,0.326680934576672,0.319942810392977,1.0210604
0193689,0.307225822355604,0.451892658742423,0.259486131769282
"Rxfp1",101.246163978134,-2.16613293360387,1.2861758004932,-1.68416551
825438,0.0921496851535932,0.185456876626582,-3.64978863471992
"Pds5b",1706.28703617398,-0.100609930993419,0.0887764061983031,-1.1332
9583052374,0.257090042121029,0.397962975649082,-0.0927016185662729
"Cpsf1",3561.37570974735,0.39840557989552,0.235565907941282,1.69127011
364831,0.090785225305169,0.183224337335699,0.676111794882978
"Fancd2",82.7170206946308,-1.05876524118914,0.330823818365147,-3.20039
000342027,0.00137241743069439,0.00751084002638148,-5.17885273390554
"Cct2",7863.39214845608,0.110602495045351,0.0950307192964966,1.1638604

4285606,0.244480572563801,0.383620732278136,0.105968349030653
"Cd226",182.046768783473,-1.591860578071,0.501821963255806,-3.17216203081878,0.00151308560655932,0.00810053354110201,-7.66612252586023
"Ccdc182",1.3452241905428,1.47508811467189,1.82834790407877,0.806787434372416,0.419788959836591,0.562778131112064,0.847983626863906
"Rpap1",948.893877403114,-0.100787105846812,0.152290576107798,-0.661807896605966,0.508094344703779,0.642674248007414,-0.0445597226942764
"Ccdc17",314.891993897163,0.463406426578418,0.16813381097673,2.75617630913365,0.00584814643914613,0.0223802079039198,1.76074899621582
"Fgd5",1577.38675117422,-0.470416322241132,0.159252830350651,-2.95389614869227,0.00313789582027894,0.0139750325956598,-2.0089048729562
"Galnt17",275.109618947544,-2.10909077137391,0.339978304865138,-6.20360399823317,5.51845431371453e-10,9.97814125218234e-08,-33.9991420439114
"Lyl1",563.639196547823,-0.810801769838895,0.285984831965059,-2.83512158413338,0.00458082483082827,0.0185591455059297,-3.23249848976484
"Gpbp1l1",4412.61459980539,-0.0898109645639363,0.122817966536711,-0.73125265868240 5,0.464624841128483,0.603467569432664,-0.0453601931627205
"Phka1",1021.51408780242,0.0594962302838342,0.126499521797552,0.470327709056885,0.638120903650408,0.752370526252533,0.0169282455566878
"Myrfl",4.57948813024606,-0.309280839422922,0.696710782309988,-0.443915678177798,0.657103548919816,0.766568421796476,-0.0822165336173464
"Ypel4",138.028223552509,-0.784383572152031,0.321083619791054,-2.44292615320106,0.0145687180080166,0.0452984237485801,-2.42726166318173
"4930590J08Rik",16.5525201402829,0.0364110729789408,0.478051822920787,0.0761655352687027,0.939287401884432,0.962488215363814,0.00139212118115808
"Poglut1",1438.81750046645,-0.360859781434217,0.0885856319639856,-4.07357009747274,4.62979081184953e-05,0.000546108053535645,-2.71102900712377
"Farp2",472.884505711648,0.484196857797569,0.204807221863846,2.36415910235558,0.0180710499621372,0.0531527126507394,1.42091738688487
"Zfp551",33.1482833814726,-0.335600021108016,0.464759431851432,-0.722094051477575,0.470236655197139,0.608203933866257,-0.166875444481029
"Zdhhc5",3584.64112652597,0.0807268048973882,0.176467443224091,0.457460047148049,0.64734041284221,0.759735899250053,0.0221824672192717
"Ccdc174",1325.95623079442,0.144801806056278,0.1939530539145,0.746581727556145,0.455316081565501,0.595502613252223,0.0750579440382128
"Hdlbp",11682.2754054912,-0.0231042141993617,0.183374347829598,-0.125994799560686,0.899736041119896,0.938633136958573,-0.00146320309115256
"Fstl5",0.385409917755174,-2.58557631080884,4.73743584408327,-0.545775477685475,0.585220281695948,NA,NA
"Ctnnd1",6124.14100293748,-0.485670903714104,0.158717110614218,-3.05997823318866,0.0022135307827334,0.0108168980455478,-2.19846012431328
"Meak7",625.465394164096,0.159351168881618,0.148069945497791,1.07618847529052,0.281842972897557,0.425766970291823,0.136064083125743
"Ano7",14.0157688665274,-2.08657241766931,0.790902951566761,-2.63821548969549,0.00833436026079929,0.0293771536893514,-7.36046347664042
"Ccs",5285.28975872221,1.39603797554447,0.259081784992729,5.3884065048558,7.10851258291796e-08,3.52676280767772e-06,17.5274384904506
"Golim4",2474.03368175819,-0.729688346290371,0.139300079272004,-5.2382

4788976285,1.62108228940697e-07,6.57128118063559e-06,-8.70722636813701
"Kctd7",210.429817660754,-0.441770085555647,0.201164191370233,-2.19606
721527585,0.0280871327577827,0.074763537817235,-1.1456975731703
"Tmed8",1082.65468699788,-0.0135507742859174,0.14507270851431,-0.09340
67780541974,0.925580405953564,0.954461960825185,-0.000631567564452016
"Atp2c2",40.074873164875,-0.844462577003303,0.463764888124845,-1.82088
510498875,0.0686243238339379,0.147914776267314,-1.61386848170151
"Scn11a",3.15446280932001,-1.64349048425609,1.23523338003637,-1.330510
09697268,0.183350261845349,0.311516504484194,-1.91680781366059
"Vav1",965.597993979901,-1.05412517055232,0.505200459240988,-2.0865483
2209779,0.036928982829849,0.0921829864570985,-2.51301300164905
"Ptgdr2",1.15738598702307,0.372664983140435,1.61024796545439,0.2314332
89242054,0.816978205971024,NA,NA
"Tpst1",775.482581880307,-1.70856142681439,0.283668461313225,-6.023092
65860826,1.71115413821253e-09,2.34958333710337e-07,-26.0792383913067
"Srsf2",15665.9484384756,0.4032109294188,0.138767384746145,2.905660650
42167,0.00366478666825054,0.015739209351883,1.67397060305019
"Mks1",606.551553012843,0.0737958372917037,0.170525864360232,0.4327545
12452209,0.66519312260745,0.773078351588488,0.0189931944082324
"Pomt2",1110.79278255238,0.135820223448901,0.0873079215270635,1.555646
0521947,0.119792281468488,0.226262705034639,0.201836803385996
"Tspan8",3432.78921208785,-0.140744074752649,0.357698419300857,-0.3934
71335511468,0.693971391175144,0.795944363831351,-0.0321214558343171
"Sik3",2521.70361585679,0.380347903103522,0.116481528762375,3.26530658
675884,0.00109345653106394,0.00630387255968936,1.92706731690089
"Tmem63c",6.09428987740665,-0.372053490931365,0.721511517007686,-0.515
658422854256,0.606092998196495,0.726745051278228,-0.118751865421869
"Zbbx",1.07288486811133,-1.41282640425551,2.2239836328085,-0.635268346
139469,0.525253414530061,NA,NA
"Exoc3",2010.95142363598,0.251860089658394,0.163894506156844,1.5367207
5754247,0.124361705702152,0.232751443442174,0.367157649532152
"Ino80",1154.19481255683,-0.0241000908455793,0.121286689008827,-0.1987
03510191668,0.842494678043389,0.89912212547838,-0.00256271708867513
"Tspoap1",500.785479240396,0.311929742853262,0.377738284378151,0.82578
2706581554,0.408927365741192,0.552662769614041,0.18497660940288
"Cipc",2174.67319196726,0.358546177520965,0.124140710992232,2.88822397
306393,0.00387423897762486,0.0164003281775357,1.47378754574207
"Lrrc58",6247.51610240654,-0.296950297307462,0.283086921296811,-1.0489
7215295975,0.294190935122749,0.438341345944014,-0.244911938354464
"Mab21l4",143.16453176633,0.392503419132101,0.491047282590039,0.799318
992382636,0.424105468944557,0.567238882801549,0.222539529477107
"Ogt",16614.7534492396,0.151556054754911,0.14410357184274,1.0517161567
6747,0.29292979602874,0.436896287230809,0.125497421987309
"Scx",566.051876782414,-0.280708254105425,0.557189602325602,-0.5037930
58832762,0.614406805885958,0.733795019999878,-0.0868863777729846
"Zfc3h1",3256.38608315314,-0.0255335182698611,0.142613536603107,-0.179
039934623604,0.85790634498346,0.909892184208415,-0.00241110881949597
"Emid1",624.199353014499,-0.719991726453578,0.16744912556355,-4.299764
02701685,1.70980067023888e-05,0.000253884162368359,-5.96054686936929
"Ccnd3",6056.11496520335,0.150442895888751,0.0717992607763727,2.095326

52929844,0.0361419757146738,0.0906542310099544,0.361168661653054
"Irf2bpl",3442.50499318188,-0.135937609235633,0.159309856298785,-0.853
290639975735,0.393498149389338,0.538038353211312,-0.0842575874870268
"Faah",3576.05403327878,0.737037285910181,0.320997485575035,2.29608429
670361,0.0216710619894536,0.0612913209559567,2.05789435711865
"Zbed5",146.40229699527,-0.280120017525194,0.205288265159905,-1.364520
36022128,0.172403850680829,0.297691310713319,-0.339420920865315
"Rhbdd3",890.415652591349,0.312895848350741,0.182408748972347,1.715355
48658456,0.0862800712918949,0.176137454721952,0.543340698195311
"Rnf43",411.648505980548,-0.189336722428363,0.325949453181241,-0.58087
7558101267,0.561322978470663,0.689516725650283,-0.0703886386961968
"Nsf",2022.68274637753,-0.184617860383796,0.13125788546091,-1.40652776
582155,0.159567465646026,0.28079841562872,-0.234486513600657
"Hsdl1",1263.29539582101,0.020409458449361,0.0879680318723117,0.232009
947420285,0.816530287666896,0.882692059184005,0.00254666942895498
"Chmp7",2553.1635662742,-0.04955244540432,0.128489305860742,-0.3856542
38478224,0.699752758218382,0.799940154769115,-0.0110610156293988
"Lsm3",1249.54953958789,0.116892835935935,0.117305299224467,0.99648384
77779,0.319015111221206,0.46408689387384,0.0897366982611854
"R3hcc1",1215.28844475247,0.0815011930539029,0.106146465756786,0.76781
8245033677,0.442595173517258,0.584085018896853,0.0438239027209141
"Gas2l1",1731.14875396705,-0.579183319801152,0.14147692281377,-4.09383
600011959,4.24294473916296e-05,0.000510357412240589,-4.39044081425918
"Chchd4",1600.99938414486,0.362038049891663,0.214049658815532,1.691374
10400484,0.0907653749563555,0.183203238188304,0.614436189455503
"Loxl2",2002.26044503912,-1.25741885058331,0.351211930919637,-3.580228
17530942,0.000343294301295751,0.00257345339741044,-7.4973681297757
"Polq",78.9448584339098,0.17716912748517,0.302186851296595,0.586289994
832632,0.557680653396821,0.686771766559162,0.0665718771323028
"Rasl10a",84.4384861521936,-1.00213074821514,0.319589149126807,-3.1356
8452168416,0.00171453531439318,0.00891229260464894,-4.73038158511243
"Efcab14",1783.25110326067,-0.187692064462379,0.154243205108786,-1.216
85791169862,0.223658281523061,0.359720298039813,-0.191901715818193
"Mrps17",2824.79524453528,0.328217510441772,0.199810355956911,1.642645
14153887,0.100456375164316,0.198008330262216,0.531530592555733
"Ankmy1",19.3029306246152,-0.113787908964137,0.434091764938032,-0.2621
28697558639,0.793222222652995,0.867233761202653,-0.0162087141548128
"Vps18",1499.55938736245,0.049999114570474,0.0665452993275579,0.751354
567125198,0.452439297538191,0.592918982052535,0.026134412846976
"Atm",286.315741024275,0.0935221590282924,0.51559075476879,0.181388355
325012,0.856062758378243,0.908458449473496,0.0089787003236432
"Sept14",1.18322765973356,-1.12939644789199,2.17646002185373,-0.518914
400701953,0.603820435007756,NA,NA
"Gpc1",989.890914956861,-0.40338984882132,0.310899437255327,-1.2974930
1697846,0.194461606251699,0.324930362387148,-0.453468435208452
"Slc38a8",2.51300348992079,1.58222769546295,1.26280140734266,1.2529505
3225548,0.210223710012837,0.34398815734479,1.6884711975349
"Rhov",643.438227359675,0.177162195007573,0.387814738785175,0.45682171
7406953,0.647799194646999,0.759961344919123,0.0486288450190825
"Foxj1",384.936548941283,-1.21461818805422,0.562589413060838,-2.158978

0395012,0.0308518706935978,0.0803673713805972,-3.06223101768055
"Usp54",931.532740060334,0.0971462696662839,0.169509393553337,0.573102573431817,0.566575244906037,0.693868734701206,0.0355042880439614
"Golgb1",5980.93701274404,0.389626321503355,0.278654789660419,1.39824017372237,0.162040956571457,0.284080419357564,0.490343912911111
"Hdac11",1865.84070026027,0.489857288022944,0.237784902122363,2.06008574829897,0.0393903440782523,0.096802738698987,1.14385595256756
"Plekhm1",2319.2091119107,-0.283713355121638,0.156077462982451,-1.8177727245191,0.0690988770987616,0.148707495107247,-0.54069354204645
"Slc25a37",2777.51275370829,0.0130454353393385,0.157224476332901,0.0829733107949211,0.933872761319218,0.958753743692193,0.000549487062986958
"Senp6",4135.04116225987,0.357290195611526,0.153579275092027,2.32642194330865,0.0199960521308268,0.0574273120550488,1.02086214818652
"Agpat1",4854.2753653216,0.193666895128576,0.136863951489149,1.41503217626981,0.157059062199982,0.277737855502924,0.248102316558942
"Arhgap27",2639.20701846476,0.190286347288384,0.182378971525314,1.04335683931616,0.296783055293951,0.441124867519549,0.155735540926378
"Flvcr2",410.922696444688,1.45627019989111,0.273410861987737,5.3263070431943,1.00229647612291e-07,4.57676269120482e-06,17.9041411258055
"Exosc4",1345.52431124244,0.593429791182111,0.157868563542695,3.75901178717966,0.000170585801952151,0.00150009955003354,3.85861331564366
"Ints14",1158.1103652653,-0.0952488242179724,0.101545814353531,-0.937988678552171,0.348250228306649,0.493150043686763,-0.0673353755258041
"Zdhhc14",523.431417679477,-0.430548810670452,0.160584426424663,-2.68113677183036,0.00733725205444152,0.0266906232383397,-1.56006905904095
"Batf",178.065583725796,-1.35603418341715,0.4582188213526 2,-2.95935941569196,0.00308279323717796,0.0137932334412186,-5.8086770256412
"Setd5",3462.97148073929,-0.417320224579466,0.188770210885808,-2.21073135756528,0.0270544457065662,0.072780934665807,-1.0935047038358
"Jdp2",518.730522404047,-0.638130309352319,0.158006070591221,-4.03864425565795,5.37610269853918e-05,0.000611322731377981,-4.72209124645997
"Thoc5",1153.28513403473,-0.199391376762923,0.130537352701799,-1.52746606726747,0.12664515882114,0.235667809381044,-0.288186747908645
"Igsf9b",378.482631331458,0.0199258304105115,0.308377774645757,0.0646150016271467,0.948480540100063,0.968143313311226,0.000645101782740502
"Dnajc17",494.487035184574,0.213735815869538,0.122299299119536,1.74764546819382,0.0805254369199426,0.167101422846428,0.382406360113614
"Evpl",1886.36123781051,-0.19971226884095,0.247740481002863,-0.806134984611745,0.420165021429551,0.563204838982084,-0.114657186569219
"Nipsnap1",4586.4708089867,1.3658597972267,0.26905850822061,5.07644157495584,3.84568975734362e-07,1.30179268903969e-05,15.3648070283394
"Nek9",4592.88804130397,-0.338781914078966,0.0991000128486487,-3.41858597532549,0.000629474277380355,0.00412130382311354,-1.86044991604967
"Traf3ip1",980.359374057227,0.467172581565209,0.107608847196972,4.34139565411452,1.41580512499468e-05,0.000222803618459615,3.9285569272137
"Fhod3",1073.69378560094,0.270165132979172,0.401843589780484,0.672314153690385,0.50138372111109,0.636539900652692,0.122035799263065
"Med13",2902.0277108455,0.0186349954446415,0.123971527972137,0.150316736023693,0.880514729570424,0.925363887089103,0.00144548356387823
"Fam53c",2108.20307616081,-0.0747038221710677,0.120839901396671,-0.618

204924926608,0.536440264043023,0.668157622653313,-0.0301229097089715
"Ccdc15",101.09637207014,0.586085706656439,0.309379591485039,1.8943903
3080106,0.058173218911157,0.130509886357121,1.19345001537618
"Sdr42e1",634.493740042883,-1.47830513870111,0.452561814387019,-3.2665
2645385786,0.00108875634975453,0.00628089588939682,-7.49536576562609
"Tmem132d",0.817848690604366,-2.64895026734815,3.06726098680135,-0.863
620760915612,0.387796255541769,NA,NA
"Kif4",105.536123669997,-0.749352201138462,0.505028508809953,-1.483781
9807524,0.137866768989256,0.251344203997431,-1.03480439530468
"Iqsec1",3187.18012590127,-0.163725560310295,0.241950604275774,-0.6766
90024397218,0.498602647074624,0.634123958819452,-0.0745787650527434
"Trim59",298.893673737442,-1.15138206917166,0.299359037370718,-3.84615
77084303,0.000119984550544844,0.00114791892371118,-7.79463163689852
"Slc26a2",1233.99698344653,-0.284701650001837,0.282221077958012,-1.008
78946413844,0.313075614982073,0.458250006825348,-0.22216419299971
"Exosc1",747.566346605171,-0.158454137225808,0.0982471335307369,-1.612
81180968232,0.106785399594826,0.207030100556818,-0.249548007776677
"Tmem132c",26.1111066476303,-0.45741377924422,0.663976986626395,-0.688
900049937419,0.490886168012282,0.627338172897914,-0.213278109250678
"Kctd9",1103.28831537059,0.155511246587836,0.185294026253451,0.8392674
59033582,0.401319239529437,0.54536677811158,0.0942859592444684
"Brip1",117.784717254822,0.38940301491191,0.255226892339252,1.52571310
704324,0.127081328844185,0.236145521035066,0.56202811649158
"Plcg2",1319.71471819111,-0.216461477785419,0.337832806451502,-0.64073
5516657093,0.521694534537898,0.655074705977585,-0.0915645027731331
"Zbed4",782.450061201857,0.295410848691386,0.170445482378197,1.7331691
3167523,0.0830656118058653,0.171007633723024,0.52170946726962
"Fam151b",201.006930588748,0.540481455171836,0.19086650448832,2.831725
01440613,0.00462976456013928,0.0186858102656998,2.15111124752114
"Ina",3.18166167395387,-0.230106166896931,1.13305324363938,-0.20308504
3168692,0.83906856195986,0.89708607891064,-0.0249903255279537
"Wbp2",7160.29511441266,0.397466747901727,0.207388264272348,1.91653442
539913,0.0552971056778988,0.12547224095283,0.82501009576266
"Cbl",3166.23448510013,-0.107874175003423,0.292024493098426,-0.3694011
20634989,0.711828762815978,0.80874727151427,-0.0228983224211364
"Ube2f",1937.50587663991,-0.277703446516899,0.202374032109397,-1.37222
865810561,0.169992266652953,0.294412485406923,-0.339568410637554
"Gtf2h5",2977.64967453969,0.414667363533128,0.231061981005108,1.794615
28776541,0.0727150157042552,0.154578867880677,0.774205049890733
"Smc4",3573.88319542263,0.452551511360543,0.27161775467091,1.666133761
79643,0.0956867753418201,0.190786455221884,0.749697065376894
"Ramp1",319.325767825006,-1.14152586528158,0.335223264702644,-3.405270
41371712,0.000660985663139816,0.00427606610157772,-6.22670605366198
"Mtmr3",3125.0718370801,-0.254290894940203,0.130742896475399,-1.944969
10956881,0.0517786962604423,0.119448771851172,-0.54033450574688
"Skint2",2.34850389571903,0.239189775284747,1.09518840144004,0.2184005
73791908,0.827117018507786,0.88967030836677,0.0279623192081829
"Cpne2",732.606359664649,-0.647515510478379,0.215407849917838,-3.00599
774207559,0.00264710963404813,0.0123299913150192,-2.84629731108458
"Csta1",0.656731174526656,1.24732419564233,3.09858403130954,0.40254651

2548565,0.687281864665994,NA,NA
"Tkfc",10793.3571316676,1.61071968926816,0.496180145119312,3.246239707
71914,0.00116940330056043,0.00662804209194821,8.08008806661481
"Tulp4",3476.10265000064,-0.0647374270249396,0.137055159782445,-0.4723
45784921201,0.636679995080614,0.751179144625548,-0.0185220973777779
"Wdr5b",431.129445410975,-0.260171399912605,0.115082216398565,-2.26074
373656093,0.0237751304477213,0.0657449288710353,-0.708179457856683
"AI661453",1045.46604550037,0.490897662106799,0.225448657777948,2.1774
2552537306,0.0294488329045711,0.0775517752530155,1.25513180497154
"Barhl2",0.132823448656454,-0.537132324054662,4.78471990596623,-0.1122
59930489326,0.910617311757158,NA,NA
"Ssu2",105.098424583127,-0.471847422751982,0.357792171525581,-1.318775
14463238,0.187244296121056,0.316039584986873,-0.543515291755998
"Cmip",2798.55436894272,0.578713227089276,0.170236119259941,3.39947379
912727,0.000675156481621723,0.00434175497317294,3.14789708053613
"Fbxo15",2.92802026092371,1.40981197063801,0.820532622101709,1.7181668
7437353,0.0857661817574956,0.175327009006883,2.45462704209282
"Lif",359.747924612122,-1.33650114294551,0.492980350820698,-2.71106371
830144,0.00670677366785918,0.0249320634844458,-4.93382843955517
"Spata6",1144.06493292251,-0.085645465191998,0.107483071963966,-0.7968
27478290823,0.425551234104173,0.56854857904366,-0.0483612979186451
"Kcnh5",3.31172693353819,1.7248044824499,0.971186763134867,1.775976102
55977,0.0757368560920041,0.159369438817386,3.16765562007195
"Pja1",2988.15637060338,-0.247022430780317,0.168054438400331,-1.469895
30970834,0.141590110077913,0.256453689391468,-0.336149897143699
"Tbc1d10a",2218.78019447037,0.479267972248813,0.150300426248973,3.1887
3328712259,0.00142897655333046,0.00775241648214728,2.32912285407177
"Neurl1b",1470.78713044577,0.0182508085090934,0.236728586547698,0.0770
959214315929,0.938547237571455,0.961881421191497,0.000709301223409429
"Pkd1l2",16.2708365173358,-2.76806168265112,0.866132361494651,-3.19588
760991955,0.00139401401032814,0.00759655818149191,-13.5083071092196
"Parp14",3060.36830567037,-0.642511436818838,0.273608530286285,-2.3482
8729991188,0.018859967593804,0.0549427234639161,-1.86422382146976
"Gcsh",3223.23759513916,0.528638415673601,0.340503318164756,1.55252059
957258,0.120537707658709,0.227274366495457,0.783229264335872
"Myo15b",4019.26449063907,1.80180803462599,0.596659984354179,3.0198238
2240072,0.00252921759766795,0.0119360135940151,7.97875750396802
"Zfp707",1343.67770252141,0.472496864763785,0.193901250906809,2.436791
21487912,0.0148182323806299,0.0458030905132406,1.45689858657149
"Zxdc",915.30538821444,-0.21357080468389,0.119201828108825,-1.79167390
359913,0.0731852171589388,0.155413875443659,-0.397596983804977
"Cops8",2162.99036620567,-0.337205008417336,0.153462709492631,-2.19730
910220589,0.0279983780316143,0.0745781932869421,-0.875352885518845
"Tmem30b",1427.5372395838,-0.25667717602964,0.360344324104462,-0.71231
0861750192,0.476272299486811,0.613852280234918,-0.125258709779989
"Gm9761",1.74738065009195,1.14921316180068,1.33668726731064,0.85974721
9791251,0.389928399325143,0.534131230156079,0.720687341955143
"Gbp8",635.073222886199,-1.10207370207918,0.544882269844312,-2.0225904
9903399,0.0431153845604467,0.103901096052353,-2.49544293145509
"Trmt5",378.22305424334,-0.383924740392381,0.19812718552887,-1.9377691

1213649,0.0526513977528526,0.120946860672139,-0.811004156477142
"Cyb561a3",1622.81516108459,-0.401107364887131,0.130358076755273,-3.07
69659607678,0.00209119179437728,0.0103225462295616,-1.83443437873759
"Dhrs11",807.478211884298,0.720566611993751,0.208531128731375,3.455439
08181674,0.000549397325548808,0.00372247008705943,4.03039410628625
"Gulo",8.68156720836084,1.20362232970276,0.78833915447485,1.5267823789
6803,0.126815134708522,0.235865487773106,1.73862475630058
"Slc24a1",3.6238312231445,-2.59424109794246,1.37808811614976,-1.882492
9026966,0.0597691226185664,0.133170877935871,-5.23030701496233
"Polr3b",1019.52489577913,-0.256535198366287,0.108833003249157,-2.3571
4526575168,0.018416041120174,0.0539168186105461,-0.749163027582318
"Uroc1",1774.47269663904,2.45246919224102,1.02523604114767,2.392102007
54909,0.0167521857030941,0.0501896307373893,7.337657431865
"Eda2r",69.0174915716146,-1.43271773382906,0.28383558916734,-5.0477029
2559886,4.47153722109757e-07,1.4506827410476e-05,-15.9617523405496
"Ifit1",1407.60046438679,-0.272058066477385,0.310390758191588,-0.87650
1826479826,0.380757283889166,0.525630524020883,-0.174975978820733
"Six4",320.323360348006,-0.336963233438988,0.290744620682792,-1.158966
35558606,0.246469899285121,0.385625910468728,-0.321088060956273
"Pkd2",8420.88569045214,0.474292081579892,0.202475293851357,2.34246891
34077,0.019156629768879,0.0555992806242338,1.37050729224617
"Scara3",327.915093337702,-1.17306942534243,0.270513069239282,-4.33646
118703713,1.44794980481233e-05,0.000225341695695181,-9.8513111169938
"Dynlrb2",22.6970282013154,0.946202539531497,0.524898445841955,1.80263
924770011,0.0714448889822653,0.152393878719638,1.78007835003491
"Caskin2",3170.77664012612,0.348245245464497,0.17286584639978,2.014540
48163525,0.0439528191523787,0.105411078454253,0.783512638538794
"Rasd2",165.627496940132,-0.0211928245350368,0.213598439610079,-0.0992
180681362842,0.920965129344194,0.951413483731752,-0.00105554089349147
"Sec22a",1078.98651836533,0.10271287149234,0.154729510910358,0.6638221
1698353,0.506804168376183,0.641412370501258,0.0456130098302077
"Diaph2",910.469236349393,-0.0449254164789995,0.16490061105315,-0.2724
39357210868,0.785284214064042,0.861162632498352,-0.00671508756033068
"Snx2",3954.7131374237,-0.190738117966027,0.105212274477331,-1.8128884
5729804,0.0698490268831932,0.149825778456381,-0.362074762157105
"Uaca",2981.94094886747,-0.194872427338532,0.159374492247162,-1.222732
85135439,0.221430614741946,0.357259460231522,-0.200580821809529
"Gbx2",8.30819218050911,-2.82060352656092,1.12615666358746,-2.50462801
292288,0.0122580237888961,0.0395903508093251,-9.10820788607945
"Poglut3",994.949380945631,-0.657163574289192,0.157857265172289,-4.163
02394173589,3.14060422716466e-05,0.000404545737646677,-5.1342519258954
5
"Edil3",70.034395301243,-0.595058731665088,0.367809168414164,-1.617846
38004193,0.105695708238982,0.205489572165088,-0.941597128720041
"4930556J24Rik",3.45859733140394,2.82363061589696,0.93525617235541,3.0
1909861635636,0.0025352799118849,0.0119562114133015,12.498813210006
"Pcnx4",472.3028115623,-0.346790951430919,0.131727976740889,-2.6326294
5359786,0.00847267278378165,0.0297365367523305,-1.21910155715252
"Mad2l1bp",553.122039154944,0.141882986305261,0.214602361891268,0.6611
43638191403,0.508520202208313,0.642920950762397,0.0626744681967893

"Hmgxb4",1054.3759945081,-0.249209945414097,0.137332049999284,-1.81465
24821802,0.0695773317434927,0.149374456544222,-0.473822618635387
"Gjc1",531.169287614669,-0.721987230210212,0.142694734618322,-5.059662
72786009,4.19998710693439e-07,1.37864223250853e-05,-8.0803556999393
"Zfp395",1597.49863252734,-0.830421490515704,0.227290544971187,-3.6535
6812629832,0.000258621128193489,0.0020593263236369,-5.13646947041096
"Ice1",2218.99749595668,-0.0315953616123886,0.130898802206319,-0.24137
2427247951,0.809266480933854,0.87822551595972,-0.00410271667613573
"Hsd17b13",6.60647303333655,1.74116461822744,0.699529777262957,2.48905
003735521,0.0128084937485859,0.0408940578205219,5.56610370817457
"Fbxo16",85.5982667383877,0.105490782039023,0.35910298336991,0.2937619
20463794,0.768939834907463,0.850514442726844,0.0170804224398324
"Scn10a",2.91274089055543,-0.176775286229507,4.77347756374775,-0.03703
28097008415,0.97045884531684,0.981941181421263,-0.0032215296836522
"Zfp418",113.269425308559,-0.0450364927151777,0.191559643936193,-0.235
104282873793,0.814127794604797,0.881747956198947,-0.00566779882194409
"Morc2a",2649.36172006093,0.206052744021703,0.222762573764919,0.924988
163582411,0.35497206440331,0.500105511374454,0.142781401336001
"Rsrc1",1620.99332184586,0.393588860768637,0.229478356299939,1.7151458
9486687,0.0863184816675219,0.17619741996999,0.683329382572347
"Hdx",71.7838410206572,-0.787355647843612,0.277752536922465,-2.8347379
1659157,0.00458632936279453,0.0185758724378412,-3.13831435829445
"Zswim2",0.0860324900549444,0.00784021510142923,4.78471990596623,0.001
63859437031058,0.998692591435574,NA,NA
"Tex16",0.133378345269972,0,4.78471990595892,0,1,NA,NA
"Zfyve9",1295.55169697833,-0.237843259657592,0.20632884255024,-1.15273
87868697,0.249017616146252,0.388570747549445,-0.224828481573991
"Washc4",2232.57371880585,0.30490444352452,0.188641361841787,1.6163180
7864196,0.106025560063781,0.205877461884435,0.481893585845649
"Ccpg1",5175.1209865579,0.436920136136987,0.135487879473812,3.22479130
852023,0.0012606452826392,0.00702488188651019,2.16637974659899
"Atp5h",15769.6678755225,0.684369072910546,0.280888886059237,2.4364405
5310262,0.0148326072622106,0.0458329915053221,2.10973951823195
"Inpp5j",1523.59158322051,1.72371700609785,0.556570467174521,3.0970328
2469954,0.00195468228206729,0.00981772157025967,7.96971969268599
"Ptpn13",3543.85670471283,-0.134044779546146,0.249181613748396,-0.5379
4008927759,0.590618410412971,0.713853072541044,-0.0451835620939688
"Daam1",1460.7454209829,-0.222578669932127,0.148292360008497,-1.500944
95710617,0.1333697988253,0.244816827103718,-0.313222718707652
"Tent4a",1109.18954119296,0.0359778965715185,0.150608500457233,0.23888
3572057971,0.811195864425916,0.879780674411865,0.00460814383768967
"Pla2g3",47.9829388053842,-0.127582672773385,0.326273404020837,-0.3910
29949732701,0.69577509814359,0.797323799710386,-0.028896762136855
"Exph5",168.177358173121,1.03242220233864,0.376947267708556,2.73890353
049828,0.00616444586111936,0.0233564790427002,3.878687209644
"Hid1",516.824573732609,-1.12691685314295,0.265811094437982,-4.2395403
2289604,2.23977960151387e-05,0.000313730047918947,-9.09081308104459
"Slc41a2",920.452627391753,0.654536389870351,0.319972143517926,2.04560
429128007,0.0407953189243446,0.0994159640528522,1.51095966864932
"Myo5a",3202.25305560506,0.230910364929348,0.188774733325764,1.2232058

8598513,0.221251943011415,0.357116753711637,0.237766673124367
"Ppp1r18",1806.88512928259,-1.10905558502994,0.261156828746166,-4.2467
0337112992,2.1693886600978e-05,0.000305847723026659,-8.97494716528656
"2700049A03Rik",297.493841652581,-0.00887267656154214,0.17151226205408,-0.0517293209583083,0.958744373763993,0.974843439698078,-0.000226061
562990422
"Mon2",3116.96227104359,0.0679527959249044,0.139335080645733,0.4876933
7635565,0.62576704409966,0.742584829454945,0.0202239865253969
"Pof1b",197.142269569201,-1.89607052969594,0.535277399151489,-3.542220
41263381,0.000396773786792671,0.00287487747858919,-11.0953216623133
"Tut4",962.392614924971,-0.397755835001608,0.184329505697403,-2.157852
23042136,0.0309393225239392,0.0805387268155652,-1.00195376263631
"Chst11",1346.91840638982,0.193640069474559,0.251292597637,0.770576098
521924,0.440958232889314,0.582625440460517,0.104606450703811
"Ppm1h",3388.78666779016,0.0514862264787013,0.17138584164607,0.3004112
00739824,0.76386352107259,0.846961099452566,0.00855188862187484
"Pik3ip1",1166.11308121497,0.096236916753596,0.208803350226224,0.46089
7378558964,0.644872232609107,0.757726816404264,0.0266992348206797
"Ssh3",2905.54346522693,-0.234703448890999,0.151422007152009,-1.549995
62682712,0.12114256564789,0.228217331031619,-0.346764229492369
"Mtrr",549.526694130737,-0.222613916882501,0.144088623107643,-1.544979
13909688,0.122351309332528,0.229943820486864,-0.327224706282512
"Rxylt1",1054.99056036835,-0.201425478165733,0.22054306360491,-0.91331
5861643124,0.361076442502755,0.506106294483983,-0.137172475559244
"Gpatch8",2185.00245156396,-0.122059414068461,0.174514945023069,-0.699
420981121851,0.484288979561231,0.621828113343429,-0.057989398606958
"Prss55",0.244662202050615,-0.548972222239719,4.08143125059311,-0.1345
0483140196,0.893003390900573,NA,NA
"Ly6d",2799.3148991997,-3.35865293341196,0.929228258147837,-3.61445414
941052,0.000300981073189065,0.00231471540289721,-20.3818796467812
"Zyg11b",1487.58111653667,-0.086515836688645,0.159145712949727,-0.5436
26561376334,0.586698477931174,0.710171773199778,-0.0296099070381338
"Setmar",222.736354615479,0.559323888119525,0.139322998392226,4.014584
05700472,5.95507186347321e-05,0.000662361615482035,4.09408245096927
"Tiparp",1208.67705584694,-0.151207383681285,0.232763191810893,-0.6496
1896468636,0.515938379836609,0.649579326590757,-0.0652354492430992
"Cd300ld",304.972476184369,-1.23631805992546,0.450690532507066,-2.7431
6403552603,0.00608502798705617,0.0230963355444075,-4.65854697294063
"Zyg11a",3.85258019680537,-1.66535498600608,1.21599514280853,-1.369540
820829,0.170830283823707,0.295497593505556,-2.03022524716543
"Ankrd12",6766.1787389136,0.551114697538965,0.269620979576412,2.044034
92044571,0.0409500974826221,0.0997183715144455,1.27054277274383
"Lrrn1",304.854190995015,-0.499387243841412,0.334062415759595,-1.49489
203299299,0.134942621861486,0.247052698690635,-0.698220077808914
"Cd300a",497.895860150125,-0.851765369445201,0.285215063366756,-2.9863
9685923573,0.00282286043643299,0.0129111266219963,-3.70489470607932
"Ythdc2",577.685644080139,0.0350469005891299,0.293979655663633,0.11921
5394378276,0.905104710677152,0.942116786717014,0.00208970771008509
"Cacna1a",362.977702476113,-0.516348806171789,0.376455059674408,-1.371
60809212758,0.170185473348447,0.294617451775549,-0.631018277704299

"Tmem109",3990.17184869017,-0.878899895235408,0.308388555555774,-2.849
97571862375,0.00437225668184288,0.0179285511635273,-3.53437358563695
"Bmp2k",1464.35398774195,-0.0618376321722633,0.162929316425988,-0.3795
36559341998,0.704289461029132,0.80365361648451,-0.0135168980453346
"Itga2b",417.304304187959,0.96687382990619,0.284219940706856,3.4018507
9027732,0.000669311696348966,0.00431412874559079,5.26545940684084
"Xpot",3208.55752799315,0.784526583561059,0.176493270213955,4.44507930
874652,8.78594495154247e-06,0.000154471014125496,6.88461624280543
"Pbx2",3314.42554326107,-0.199867125847331,0.104613221922031,-1.910534
0814022,0.056064482558272,0.126921213516434,-0.412563472877254
"Tdg",1538.88808279538,-0.156996547724336,0.120035342974273,-1.3079193
497034,0.190900665723186,0.320762853571702,-0.178513427418496
"Dbn1",1993.69457712371,-0.352700945109358,0.273106167995525,-1.291442
61990867,0.196550239936124,0.327494152988767,-0.393714801858285
"Gpr142",1.69825305527279,-0.295773614968764,1.70254791809545,-0.17372
4105985593,0.862082291750818,0.912858294041004,-0.0269670468292057
"Rnps1",2856.46545585665,-0.286200247951864,0.0964590740624638,-2.9670
6401894891,0.00300658349492934,0.0135543040425739,-1.23096190288634
"Ppp1r1c",2.23344122092813,-1.59597358793335,1.25767946687039,-1.26898
278136382,0.204447205324836,0.336966323704451,-1.73605583380636
"Sema3f",1854.5369185412,-0.814543091669183,0.25949577848591,-3.138945
44420657,0.0016955701217894,0.00884909695788267,-3.85070348236091
"Fam171a2",907.744671895598,0.146667886259376,0.362254651918794,0.4048
75094032621,0.685569326583334,0.788813332333403,0.0347933734746171
"Prr7",86.3253292695463,-0.998805907681232,0.303700162223622,-3.288789
5099173,0.00100619242596444,0.00589859889400323,-5.12691110771332
"Fras1",1537.95761698818,0.836457963731368,0.376984146004798,2.2188146
9710592,0.0264993337185397,0.071711867986688,2.2041495623793
"Neurod1",0.414174839119458,1.45961045624537,3.12076350495903,0.467709
409548653,0.63999241120397,NA,NA
"Dnaic2",34.9755848837743,-1.43126867888672,0.477427082809401,-2.99787
911164251,0.00271865489312887,0.0125761169884287,-6.26316839203529
"Gns",9728.07188421827,0.119984779297687,0.0912763845567695,1.31452160
249689,0.188670733239954,0.317841883248024,0.137526703093926
"Grn",12131.2510764245,-0.138341147321824,0.198040382171771,-0.6985501
93676322,0.484833179175523,0.622294603881532,-0.0656209692862518
"Ppp1r21",2453.08087122288,0.744734764527662,0.207649625722851,3.58649
702321956,0.000335149888491024,0.00252404500803321,4.4549233042317
"Ttyh2",1826.93769114422,0.481417444179205,0.152378275849704,3.1593574
7070694,0.00158117418800802,0.00837189478915665,2.3025594782919
"Tmx4",1455.65842768476,0.030518127568391,0.123979407882166,0.24615480
9816452,0.805562395922428,0.876400825560512,0.00402630934420243
"Cnot6l",2753.57055583488,-0.128922232955813,0.133561632835398,-0.9652
63977527869,0.334412609559209,0.479106759883857,-0.0948650820422128
"Mrps10",1429.64851293683,0.307817466116242,0.207639439089144,1.482461
46043618,0.13821755094872,0.251724809303021,0.424609204266796
"Adgrb1",38.9446082891433,-3.23625185854965,0.597984907460179,-5.41192
899381856,6.23494139391797e-08,3.19918124470033e-06,-40.9470508867031
"Dgkh",2448.6969976305,0.926777578387367,0.250620141019119,3.697937343
01931,0.000217358521556035,0.0018072735408527,5.85346777900603

"Pabpc5",0.267502283911741,-2.05091740934146,4.74801900672581,-0.43195
2232380754,0.665776131051136,NA,NA
"Nostrin",487.175297117415,-1.05012690681159,0.246535627189683,-4.2595
3408350032,2.04853461523538e-05,0.000293477795208371,-8.54142622858362
"Mfrp",58.1608323699372,-0.241765245961803,0.482047009104819,-0.501538
732520653,0.615992025829185,0.734998624295786,-0.0744362920228053
"Nagk",2794.55516300379,0.154266677637221,0.18242229475866,0.845656929
386356,0.397744157013222,0.542209017230218,0.0944272059018794
"Sirt6",541.33536902405,-0.533099852200555,0.163461365788112,-3.261320
18798613,0.00110894741075123,0.00637260731353596,-2.69521775549758
"Mast4",1256.87257262551,-0.126142157483357,0.222761159480431,-0.56626
6389426109,0.571212704940285,0.697652795512507,-0.0454154311675566
"Pcdh11x",6.24349355313225,-0.850973325175422,1.07551018795878,-0.7912
27581758657,0.428811198997772,0.571455181331321,-0.476178481247222
"Tmub2",2433.08233338844,0.171068089083236,0.0812815146324194,2.104637
07347063,0.0353229118993257,0.089040224461933,0.413756750561429
"Tle6",158.083600697444,-0.661904989871099,0.382998529909926,-1.728218
09531949,0.0839491307130258,0.172553252228819,-1.16299975311677
"Map4k5",1651.51351001369,-0.466203387892215,0.156129813827787,-2.9859
9848717198,0.00282654041463992,0.0129249261267939,-2.02733092164044
"Glis1",507.142613621856,1.69079972412415,0.612494830579048,2.76051264
387926,0.00577107240509559,0.0221373932234023,6.44277053614096
"Dusp5",317.86945001827,-1.1397940374895,0.301589174721935,-3.77929359
878514,0.000157273896796968,0.00141054574045413,-7.48135571112168
"Asb16",16.6311626917754,-1.86067403605594,0.617426638355952,-3.013595
33338315,0.00258171913499345,0.0121238823926065,-8.21036935428055
"Tle2",1903.65229545565,0.763833719207978,0.252371137894566,3.02662866
118664,0.00247297552379679,0.0117323558436366,3.39555007970476
"BC030867",42.5040111271224,-1.02991590371342,0.670046845212313,-1.537
08044605013,0.124273610742011,0.232653563345917,-1.50182829839798
"Dsg1c",0.278375941021959,-0.931779969723793,4.78266383184478,-0.19482
447491284,0.845530361204747,NA,NA
"Vax2",0.844790771797237,-2.3693293040558,2.31499597775028,-1.02347016
013321,0.30608560157046,NA,NA
"B3galt1",128.602214318292,-1.69686786088923,0.506356016765198,-3.3511
3596897592,0.000804807756555181,0.00498321423858778,-8.99625068360451
"Gna11",4599.61350497924,0.022009988599311,0.143080459758479,0.1538294
51180574,0.877744211533575,0.923746782343696,0.00174577264819352
"Cd207",8.16789495702596,-0.202084765850843,0.616920568857388,-0.32757
0154169327,0.743236691400526,0.832756522751837,-0.0369843351038934
"Dio1",688.786754374156,2.41326721416594,0.801999851132905,3.009061922
83821,0.00262055698620965,0.012249915808011,10.6237723262643
"Gpsm3",1349.1519926669,-0.704483810706506,0.371192250965066,-1.897894
71325145,0.0577099529420954,0.129829083011997,-1.43822937068322
"Rab24",4059.86642977066,0.482633388615446,0.153271950743474,3.1488696
1557117,0.00163903305688606,0.00861686641511838,2.2944554354042
"Gna15",207.663220551987,-1.41394066359054,0.275175008687027,-5.138332
40284805,2.77187252008223e-07,1.00159668737618e-05,-16.2763376510417
"G6pc3",2697.46057634463,0.532102132552859,0.105325319336234,5.0519868
9077041,4.37237716648288e-07,1.42087683320139e-05,5.93913845728232

"Ccdc122",228.302496166248,-0.769459097684288,0.352692671417039,-2.181
6702189835,0.0291338803777691,0.0768989305003035,-1.9738651920165
"Cpne7",891.589884392787,-0.125161792967942,0.368920924266271,-0.33926
4554367227,0.734410442351192,0.826236908055152,-0.0238900986475365
"Unc13a",84.2794921100854,0.337196924458418,0.342581324224812,0.984282
856695187,0.324976456187731,0.469884385817454,0.254674249788491
"Zfp661",344.46219395436,0.186088918639725,0.167060421000416,1.1139018
896598,0.265321278667617,0.407180975321621,0.167200434792857
"Sos2",3412.43329829777,0.701447603641693,0.220338009314235,3.18350703
913878,0.00145502575475232,0.00785657495601239,3.3994988394577
"Colgalt1",3812.29067682973,-0.704729811023381,0.148214729705528,-4.75
478930079038,1.98653486609948e-06,4.74093718717351e-05,-7.016776706093
97
"Scn7a",1525.13316954954,-1.47052906394797,0.290831514617044,-5.056292
01114711,4.27486613087402e-07,1.39851241308359e-05,-16.4368626632982
"Grip1",236.765080631497,0.935500147983202,0.368606288827341,2.5379386
5253721,0.0111507529127841,0.0369255061639715,3.08607722903149
"Celf5",23.2029306418701,-1.13942119256663,0.432176328210684,-2.636472
93521169,0.00837728827236609,0.0294891436603347,-4.01501616525843
"Cpsf7",4475.26780006267,-0.07202723760682,0.158086155690359,-0.455620
147711724,0.648663152417099,0.760485487693379,-0.0197209316127421
"Nrip3",67.4464784173874,0.186139535863147,0.57973958069978,0.32107439
6263346,0.748154012740077,0.836199881937863,0.0332980550680782
"Nup54",941.431029050656,-0.189792638404296,0.136490221071614,-1.39052
187705605,0.164370460805585,0.287012776889152,-0.236904588969839
"Nxnl1",31.3362484450759,-1.37690959479205,0.801483234259162,-1.717951
83721438,0.0858054005299994,0.175370408756177,-2.39699984315275
"Tet3",2754.9672774544,-0.342357844231205,0.116585741867935,-2.9365327
0756744,0.00331903817728268,0.0145885045943773,-1.44732511874665
"Tespa1",119.399525730186,-0.930379299472722,0.570792839949067,-1.6299
7717272652,0.103106321606463,0.201945460440263,-1.48838136996755
"Gnat1",9.11170833582668,0.606819949759685,0.697825978111433,0.8695863
56475231,0.384526496459418,0.529185321002664,0.386190280267154
"Larp6",167.878154901528,-1.07122253769983,0.322958260905,-3.316907066
2507,0.00091019887060078,0.00548256335464134,-5.5769800528863
"Art3",681.644077709838,-2.08728764360622,0.601014315581513,-3.4729416
4130958,0.000514787278050449,0.00354050170985102,-11.7795803394952
"Plvap",6043.8060496418,-0.077938254271533,0.291605675215597,-0.267272
762143294,0.789259156819714,0.864112248844448,-0.0113830847516221
"Ttc21b",599.167857587346,-0.0415081148041724,0.132225790956458,-0.313
918445894122,0.753582980248908,0.839812582812826,-0.00724634255545459
"Tmem127",4710.22322203844,0.098641135383071,0.109200906607784,0.90329
9601141222,0.366366908176435,0.511266960751798,0.0661747271779498
"Acot11",1863.74849787918,-0.905608633055427,0.514538574709259,-1.7600
4031100514,0.078400971976745,0.163685849834305,-1.63897615325465
"Mfsd12",1638.35953721453,-1.10566134864082,0.220526305595206,-5.01373
904422246,5.33823743820989e-07,1.65998066661989e-05,-12.1690409399371
"Cxcl10",1197.7433242624,-0.903972487268238,0.5524225852955,-1.6363785
8286458,0.101760378712319,0.199933265267303,-1.45518927495286
"Fam214a",2323.61827896334,0.187310275192227,0.268277428036784,0.69819

6179093175,0.485054515682303,0.622441990314558,0.088804708656367
"Ano8",2220.93665686277,-0.15982158532267,0.196870615577596,-0.8118102
58497807,0.416900514754278,0.560254065523393,-0.0925950183491857
"Ankrd27",1877.25448204876,-0.337763900969915,0.150525519901997,-2.243
89791970041,0.0248389700370466,0.0680784703095776,-0.90760344087668
"Myl12b",9046.48060092248,-0.170924539092071,0.162636916346619,-1.0509
5782022692,0.293277962659845,0.437340877750839,-0.141361832028773
"Fam151a",3993.02612958012,2.16720826669812,1.02805014486057,2.1080764
1780163,0.0350243799075992,0.0884330660935182,5.25658386756257
"Gipc3",73.8132645438754,-0.876331545756194,0.348445607113512,-2.51497
372291657,0.0119041255890206,0.0387878744543323,-2.8477687003164
"Nudt19",23789.790317039,2.23876929538382,0.550063049651816,4.07002305
790389,4.70084849072854e-05,0.000551819047996538,16.7958973411475
"Mrpl34",2378.09073058824,0.383897739921432,0.343879770049324,1.116371
98043481,0.264262942080309,0.406196203758917,0.34586075859408
"Tbxa2r",305.772157758631,0.0518840945048665,0.284133224309475,0.18260
481374876,0.855108107029431,0.907813234828479,0.0050180537361552
"Lrr1",15.7545957979128,-0.982480002329637,0.75672298235056,-1.2983350
9123487,0.194172213021172,0.32458588901636,-1.10549150608773
"Cactin",1513.43561065833,-0.180023656136261,0.106473709184269,-1.6907
8035803846,0.0908787599595858,0.183394127684952,-0.305341316583261
"Sncb",0.529844454077075,-1.26306284411672,3.72862186598923,-0.3387479
04591184,0.734799649319712,NA,NA
"Rps29",10207.6780652788,-0.0229183315959395,0.174877334815435,-0.1310
53756166443,0.895732782793072,0.935808701650288,-0.00152049842024957
"Cog3",2207.644778275,0.0778384242251705,0.13240428929153,0.587884460
822134,0.556609847276013,0.686059725784671,0.029328785883438
"Filip1",204.808840563418,-1.01651849151608,0.328487978882519,-3.09453
787311843,0.00197119766114448,0.00988539403214896,-4.69295783112181
"Pip5k1c",3709.49038299962,-0.156855276763982,0.1823103372456,-0.86037
5111657402,0.389582300622313,0.533732131642346,-0.0984833410430888
"Cobll1",3693.17109347781,1.07551407170329,0.232419605323885,4.6274670
7707608,3.70165053400138e-06,7.87922476044029e-05,10.1622054498087
"Ncaph",190.069201625048,-0.89007833388959,0.239667742944947,-3.713801
12714645,0.000204169261959085,0.00172258427939344,-5.6643958800522
"Sidt2",5964.10385144058,-0.0414208196924186,0.111375387453232,-0.3719
02811200648,0.709965211993624,0.807542976516841,-0.00885407314611394
"Pygo1",319.204445000913,-1.15000111024605,0.279029900196499,-4.121426
08887504,3.76534277062725e-05,0.000465546311615029,-8.82315234318572
"Ushbp1",2982.81161835968,-0.34644361713463,0.341091421124229,-1.01569
138265853,0.309776372872913,0.454957797399522,-0.272841884534401
"Mdga2",501.088036394291,0.649502738538694,0.353644798442648,1.8365963
2885574,0.0662695096861043,0.143856924945409,1.25934427439052
"Tjp3",1362.71557696424,-0.189994009839616,0.212375415330979,-0.894613
953048743,0.370993490422095,0.516098107609161,-0.125673133862153
"Cdhr2",867.396098467745,2.08969390787465,0.653408250886427,3.19814435
315092,0.00138315024449864,0.0075542474698136,10.2095032092614
"Ttc22",345.380432369702,0.190913282748449,0.218342785312975,0.8743741
29077781,0.381914541572143,0.526743278741858,0.122383429973205
"Ly6g6f",1.34458572026801,1.14648378212331,1.68489676825845,0.68044749

3117542,0.496221157728173,0.632001674114596,0.526079258198671
"Dhcr24",7519.6579066999,0.509547102560708,0.369346098981805,1.3795924
8511194,0.167712152551865,0.291344578622262,0.628398247478425
"Rnf44",4843.53980126959,-0.188758332149547,0.149612582636805,-1.26164
744183163,0.207075676904524,0.340148403393801,-0.203551941018418
"Rtkn",2414.1567077561,0.747451279204736,0.357561597478624,2.090412629
53139,0.0365807493597757,0.0915712497827386,1.78688537408721
"Dhx8",1155.71224696199,-0.0317094460259342,0.11339840701244,-0.279628
672583167,0.779762406759557,0.857497366018682,-0.00487492054645977
"Mrpl54",1898.00277456732,1.02453001272139,0.19185817077351,5.34003847
003654,9.29268602682445e-08,4.30377178505993e-06,12.6591120578069
"Arl4d",1329.61487627844,0.695388409261962,0.314097626717967,2.2139244
3021024,0.0268339778540871,0.0723063709506621,1.82667619979075
"Synrg",2389.82951503363,0.215183464995848,0.131131382125072,1.6409761
0738677,0.100802374953901,0.198549823906573,0.347890380433997
"Tmem106a",3455.76628852272,1.16141927932062,0.223882171967821,5.18763
628703564,2.12980050931397e-07,8.11071295519607e-06,13.6145340015183
"Zfr2",471.685960650751,-0.821111626837147,0.224184919096221,-3.662653
26921801,0.00024961630806976,0.00200784602918489,-5.09967203495196
"Cog7",1425.78513003123,0.247580216033251,0.171135371916442,1.44669224
871953,0.147983136374751,0.265175553077373,0.328628869762931
"Cebpa",6627.7797402165,-2.13502031514261,0.609882336052355,-3.5007085
6119915,0.000464022992197335,0.00325801955414607,-12.226483699826
"Atcay",11.2585745369611,-1.37378186586203,0.955935716670551,-1.437106
95385126,0.150687605639365,0.269057411607724,-1.80354272849473
"Rubcnl",169.32150934795,-0.840672144106536,0.39029926820944,-2.153916
78278889,0.0312466985717772,0.0811249172772986,-2.11157097199315
"Lbx2",28.086646590784,-0.5228042366215,0.373296388319385,-1.400507084
93381,0.16136152327756,0.283112299187065,-0.659732753272707
"Dop1a",1137.66174990643,0.173121464680661,0.14969146129712,1.15652197
647423,0.247467715776243,0.386814616178065,0.164432451398159
"Dapk3",1646.65737115983,0.14875601303602,0.132321430716225,1.12420196
963438,0.260927354078262,0.40240145138163,0.135413350388825
"Parm1",1920.19343391228,-2.35005806472887,0.540425243311249,-4.348534
96170865,1.37049973027095e-05,0.000217520471741762,-19.8185510064411
"Hrh2",82.9140911551608,-1.70615230797802,0.428963124994997,-3.9773868
8610569,6.96767492450817e-05,0.000748929027993717,-12.27895010702
"Otoa",28.3548869747214,1.08970693281354,0.593048300815632,1.837467422
66159,0.0661409214493233,0.143641756117053,2.11450316622026
"Vat1",4916.48687460233,-0.879863083748296,0.192358041986892,-4.574090
4547586,4.78293184351395e-06,9.58454317418653e-05,-8.14117404498842
"Eef2",52614.175982765,-0.192849843089319,0.247045469469484,-0.7806248
92670377,0.435023146709162,0.577061585576321,-0.106030055612159
"Htr2a",31.3015779227501,-0.37595520212071,0.480402594391226,-0.782583
621550017,0.433871662519645,0.575955179453161,-0.207424047341551
"Foxn2",892.11495224165,-0.569210370599293,0.126797067899582,-4.489144
58376973,7.15097508297843e-06,0.00013055216495123,-5.09086823917405
"Dpp4",3333.47636294212,-0.0180913489701454,0.400523757103354,-0.04516
92281651024,0.96397242157294,0.978290286807541,-0.00039708405540127
"Igsf6",354.020044292342,-0.997729921130417,0.426466240058919,-2.33952

849583726,0.0193080985706545,0.0559472887398753,-2.87679988277598
"Rundc1",1617.03982977334,0.304232918752872,0.109021317631846,2.790581
92802471,0.00526133827649215,0.0206950925388428,1.17977226540778
"Zbtb7a",5151.68524701654,0.228487387005191,0.189450425521462,1.206053
70178652,0.227796784823183,0.364237436612871,0.230760682416858
"Epgn",3.68903256368484,-0.210874635572842,0.84951964892137,-0.2482280
84942577,0.803957937582293,0.87519244413933,-0.0281120098992596
"Baz1a",2261.82242768582,0.00556731883737368,0.276486295583273,0.02013
59666873503,0.983934908688359,0.989723009277618,5.75113144596816e-05
"Ncapd3",974.179475717459,0.270690511628721,0.177179724717273,1.527773
63245521,0.126568751042381,0.23557948911278,0.391339137472842
"Map2k2",5447.58189295781,0.657531432280989,0.170548505821289,3.855392
51202815,0.000115544090720117,0.00111363256592232,4.47129791996431
"C8a",600.80236769364,2.52258626890448,0.812491472452723,3.10475414749
816,0.00190437243531349,0.00961456596161118,11.7160914917182
"Nek11",32.3815912475547,0.269374549711914,0.43580048097807,0.61811439
2869313,0.536499935391336,0.668157622653313,0.108620215178354
"Tbr1",2.93680645582274,1.65556542627188,1.21314377292276,1.3646902067
3717,0.172350438969238,0.297660520728266,2.00621688881043
"Creb3l3",19.3360886700843,0.882300299044002,0.471996716822747,1.86929
329717211,0.0615820201605586,0.13622943375205,1.75879047572147
"Ccl5",697.097497144646,-1.18839643334067,0.562158269445221,-2.1139890
63104,0.0345162014529968,0.0875044722312485,-2.89501140387897
"Zc3h12b",136.649821362598,-0.185460305590208,0.243791624650803,-0.760
73288348545,0.446816629593506,0.587935662290065,-0.0985049703424338
"Kri1",2050.6920076501,0.171040991824436,0.208988663838499,0.818422342
546828,0.413116068274128,0.556664623304004,0.10019450163221
"Anapc13",5226.76070085978,0.816628869129687,0.243591689483156,3.35244
962938753,0.000800998154920181,0.0049675133661377,4.33208213599776
"Rrp12",638.831549382876,-0.184683367438229,0.0814861159151021,-2.2664
3969177087,0.0234244757095272,0.0650150074568486,-0.504764972862107
"Dhx57",1651.84514587404,0.10819887306518,0.303792528044002,0.35616041
5668645,0.721720434463115,0.816247391598714,0.0219684604991163
"Zc4h2",248.71436124578,-0.585732994385376,0.255618540626359,-2.291433
91926938,0.0219383296762331,0.0619125129580536,-1.6295285030346
"Eef2k",1107.21315954396,-1.41265248106784,0.318359978173179,-4.437280
36788405,9.11026000055982e-06,0.000159025827655251,-12.3556857194393
"Xkr6",400.590194139547,0.295676551772919,0.186192043042563,1.58801926
731814,0.112281982350131,0.215225530069096,0.454179533042286
"Oma1",733.780188344795,0.0216928219375452,0.142646572385068,0.1520739
09487193,0.87912864205633,0.924555139904302,0.00170164107182382
"Mtmr9",852.522755285429,-0.414711234246416,0.191299529548763,-2.16786
332525039,0.0301690869853547,0.0790088724422334,-1.05261803239423
"1700020L24Rik",10.5623705415211,-3.23863183209893,0.977593177367743,-
3.3128625557916,0.000923463494026545,0.00553000344180884,-16.833005420
9172
"Becn1",6203.32649701323,0.180083187123645,0.0922404906454789,1.952322
51978998,0.0508999254240182,0.118007936494353,0.384838382869199
"Secisbp2l",4449.42077555134,0.215330184953944,0.129739866979419,1.659
70715067947,0.0969733801539782,0.192721125903809,0.354543535066086

"Fam167a",138.871465201344,-1.35756780587885,0.431054373710065,-3.1494
1197370144,0.00163599386569452,0.00860856703294952,-6.45523139399129
"Eva1a",1370.97488388462,1.32676671562147,0.3431902933805,3.8659797238
218,0.000110644130660954,0.00107267062523678,9.07190517450637
"Egln3",674.174050618311,-0.571805427456563,0.29978377737121,-1.907392
82982788,0.0564697371888705,0.127677043950106,-1.17691926301918
"Dcbld2",1189.91495863348,-0.124741342173824,0.129766259768563,-0.9612
77164004718,0.336412826808753,0.480857510235105,-0.0913336509634409
"Shc4",74.4526614930278,-0.847790800480693,0.397876382440477,-2.130789
45596256,0.0331064913991052,0.0848239644283512,-2.09165011357663
"Wnk4",6327.46453230518,1.82077076181143,0.482644692063208,3.772486866
12092,0.000161628477017866,0.0014429633905194,11.9097641896883
"Neil2",251.386460615029,0.591592217103132,0.246524760568973,2.3997273
7723282,0.0164072863621878,0.0494297387963438,1.7790379113415
"Gcfc2",557.006278659266,0.146394224678095,0.24011681271054,0.60967919
332901,0.542074340115025,0.672522619213716,0.0580774485291812
"Wdr78",307.906976253007,0.260611362213349,0.213160112213292,1.2226084
8667867,0.221477606162704,0.357273960863772,0.268234868493954
"Gm6781",1.59234364225293,-2.56603662506559,1.57875450217702,-1.625355
06408828,0.104086924827667,0.203414017318022,-4.08644381688008
"Brinp3",2.40603790460316,-2.13634149514546,1.24202044060927,-1.720053
41079368,0.0854227333372312,0.17489997423179,-3.72479908140318
"Arhgap5",4812.34802676895,0.0328619918785876,0.215901515986809,0.1522
0824980495,0.879022687376097,0.924555139904302,0.00257777965649376
"Secisbp2",2064.44362774259,0.440452217209565,0.153367244546225,2.8718
7931499163,0.00408038762684619,0.0170508782572326,1.79332479558425
"Nubpl",520.643636085292,0.10045638643362,0.32429369327892,0.309769781
268052,0.756736033060249,0.842162615387458,0.0172566143916977
"Gpr33",24.8339885248074,-1.2154743923433,0.631769095766033,-1.9239218
8932495,0.0543643750450007,0.123831376708811,-2.53892484955779
"Eif2s3x",4927.069335679,-0.215924663113067,0.275292811069282,-0.78434
5447577729,0.432837438872478,0.574939919023151,-0.11951208398331
"Elmod2",1676.86259477054,-0.783613672880056,0.369643978606103,-2.1199
1461577434,0.0340132465877904,0.0866122870535115,-1.91696477778028
"Ap2b1",6181.06976081623,-0.240834060737459,0.121705829380656,-1.97882
10800011,0.0478361542426515,0.112537323289481,-0.526094864899427
"Mitf",1211.01007172231,0.198357294766659,0.213286917027338,0.93000216
5773885,0.352369963138191,0.497382473828456,0.138531938022797
"Ints6",1254.31190396143,0.0116164856234838,0.372272261736543,0.031204
2739077476,0.975106631490147,0.984521038460692,0.000181217308623404
"Zc3h12c",596.616898369772,0.365738066718962,0.248223302571366,1.47342
357840803,0.140636856388897,0.255177501011606,0.499523549650003
"Kcne3",59.0962208947064,0.144814761103972,0.362566796753581,0.3994153
97109282,0.68958715068,0.791987410258504,0.0337722190748587
"Tanc1",4342.53249647195,0.354053651020665,0.145879856926481,2.4270221
9813047,0.0152233243691444,0.0466558281278807,1.08515936704645
"1110059E24Rik",719.751867646937,-0.355023489381637,0.196520381156199,
-1.80654793814723,0.0708327932234734,0.151452769193035,-0.670100253056
863
"Plekhh3",1205.34022099625,-0.453767270494185,0.200862264685884,-2.259

09665612804,0.0238773731099892,0.0659621735475445,-1.23364520289628
"Ccdc186",1674.13791226006,0.154867817316604,0.107408859327845,1.44185
329111354,0.149343765407285,0.267122716990299,0.204432814986851
"Ppp1r32",22.685661590476,-0.113369607914169,0.337951050085761,-0.3354
61623467066,0.737276889241324,0.828268309888646,-0.021360889672704
"Heatr5a",1690.72090848818,-0.274089594255055,0.22629047796472,-1.2112
290217434,0.22580764289159,0.361863846560386,-0.278608578570059
"Slc24a5",13.2745092749981,-0.083814505379072,0.721780803896641,-0.116
121826635714,0.907555990677563,0.943208075594644,-0.00490049733455501
"Fam124a",567.557796477028,0.239612316326732,0.277887389510218,0.86226
4087438632,0.38854221020387,0.532793868384208,0.15086486669304
"Ubd",921.138247650638,-1.77613823889848,0.729037826153128,-2.43627720
700107,0.0148393075979049,0.0458391665944153,-5.4751528416779
"Nkx6-1",51.5779169189518,-0.525778962492405,0.473564122206988,-1.1102
5928240103,0.266887313544192,0.408876313309079,-0.470226514415336
"Ano4",55.8073476821643,-0.0319029190125121,0.397224065455692,-0.08031
46681858596,0.935986992375232,0.960064936468634,-0.00130018287590362
"Tubg1",875.977578159432,-0.108480319723369,0.26512909250682,-0.409160
37805462,0.682421969363295,0.786285990397594,-0.0260824328318553
"Arl6ip5",3846.73905013337,-0.371008600531465,0.10901801899887,-3.4031
860415232,0.000666049116000859,0.0042973612388434,-2.02190564560563
"Chrnb4",13.8602015965562,0.0571281476084877,0.637885454342943,0.08955
86303458398,0.928637960445306,0.956091596063947,0.0025651428754368
"B020004J07Rik",0.21312868678269,0.886940425949497,4.78471990596623,0.
185369351473122,0.852939345785746,NA,NA
"Lars2",15161.9315562501,0.795719824815144,0.348227749973721,2.2850557
5697282,0.02230955859409,0.0626608720062758,2.20415829902943
"Epn1",10692.4409679511,0.39814661084021,0.131594588152016,3.025554594
84608,0.0024817759558087,0.0117564790053949,1.76910505607883
"Sppl2b",2055.02941104991,0.294029024368309,0.138148299303229,2.128357
9012647,0.033307420802007,0.0851932429224102,0.724144430250646
"Slfn8",728.05617312451,-1.06797122470363,0.37048508195238,-2.88262949
502754,0.00394371000596344,0.0166369913513635,-4.37454541432339
"Xrra1",8.1693732577751,-1.06449580107718,0.639806803703978,-1.6637769
3221545,0.0961570154819603,0.191449963021266,-1.75974866007603
"Leprot",4044.42623570124,-0.451186044444467,0.163033942066042,-2.7674
3626956957,0.00564990908780218,0.0217797404891196,-1.72658750981167
"Lsm7",1712.02294349288,0.150060087289298,0.169757208266396,0.88396886
8372362,0.376713018545492,0.522006284954872,0.097550408937991
"Rims4",126.576589389012,-4.96515502519443,1.33188844432098,-3.7279060
767929,0.000193077303182014,0.00164753665533607,-31.8190670972837
"Spcs2",5023.1043111212,-0.366861446276382,0.178126786997951,-2.059552
36974325,0.0394413536254815,0.0968914593662138,-0.856314745155947
"Ccdc106",361.020128438808,0.315699949839769,0.29314033976222,1.076958
39506718,0.28149885311775,0.425452634466881,0.269797699463866
"Pdk3",1184.78940372693,0.984111777143996,0.293047583826681,3.35819788
818336,0.000784524271940665,0.00488243033970606,5.23755325855159
"Abraxas1",460.283266799016,0.0976059225742569,0.240341191055697,0.406
114000457115,0.684658839207714,0.788230871922059,0.0232267199035759
"Trim13",316.81072185864,0.412560110130711,0.173587836685748,2.3766648

5168303,0.0174699531308041,0.0518315890697245,1.22107705008312
"Scai",1306.01783717689,0.17582717050158,0.182970711804353,0.960958006
708684,0.336573282720691,0.481051595549629,0.128666938238328
"Lcat",198.255883329023,-0.348398936064901,0.352625591385914,-0.988013
7590003,0.323145909793957,0.468111873190333,-0.264451503899532
"Kcnk15",0.0837058623428614,-0.544048378937457,4.78471990596623,-0.113
705376621746,0.90947135024012,NA,NA
"Neu3",264.847065078996,-0.699105721195879,0.183899105782686,-3.801572
16219426,0.000143780849028441,0.00131988653218293,-4.63521739875372
"Oaz1",14650.6993088412,0.149806881294995,0.345636320263549,0.43342343
5305545,0.664707178146422,0.772651756259624,0.0386392156930987
"Eogt",1977.70388587862,0.0535385772134835,0.175300314661409,0.3054106
16728742,0.760053436846161,0.844657846202704,0.00903857798682529
"Pcyt1b",51.8195243779054,-0.879057695471528,0.371759107451565,-2.3645
8953621212,0.0180500636716485,0.0530989953937542,-2.58055937104403
"Hectd1",4835.97223328791,0.195766043994543,0.155976069881156,1.255103
06897529,0.209441346529607,0.343024920198014,0.20946030570184З
"Tut7",4002.94614656224,-0.245592688505807,0.14952103814466,-1.6425293
1596287,0.100480355823799,0.198035578474925,-0.397690346494663
"Abi3bp",1147.66406240557,-0.97073720611171,0.342456944300537,-2.83462
555590579,0.00458794255101785,0.0185785470113699,-3.86911363549958
"Amh",32.0437729887252,0.357029307591776,0.500731021931579,0.713016154
290839,0.475835760945625,0.61341128376316,0.174487231830446
"Helq",531.907630751547,0.481292481383584,0.178863695970717,2.69083381
494241,0.00712736893916832,0.0261318991304821,1.75411786630562
"Pkig",2291.11584438084,0.201133802661912,0.215230306820448,0.93450502
2239754,0.350043446554185,0.495006318604112,0.141434223070848
"Impg2",89.0281765893249,0.943441485989588,0.353517133136537,2.6687291
7196126,0.00761388164255144,0.0274257277418193,3.39287383889828
"Hpse",148.44664528441,-1.64138317983388,0.344323252665091,-4.76698325
520986,1.87004845228168e-06,4.50707104111103e-05,-16.4257777178112
"Tpbg",254.140314944878,-1.06050052342734,0.282613512305822,-3.7524763
5817126,0.000175096349134945,0.00152616388709573,-6.87734372608703
"Raver2",242.822018688246,-1.0462258295889,0.200527921389373,-5.217357
3751727,1.81493876118296e-07,7.14902544758491e-06,-12.396242848796
"Arx",1.64481835122102,-0.5426230680295,1.26150718846659,-0.4301387047
10101,0.667094746621098,0.774595841449391,-0.138593463486007
"Plekhj1",2146.36981601935,-0.0682226185322992,0.109904944430489,-0.62
0742031997002,0.534769369278499,0.666641291242151,-0.0276644882149028
"Ssc5d",546.885039256507,-1.24260486382194,0.270934236493365,-4.586370
77360419,4.51017345337968e-06,9.16990218915506e-05,-11.5524959839353
"Adrb1",186.371385623672,-0.48143459974174,0.431922117091359,-1.114632
89489273,0.265007768008363,0.40685995752969,-0.432947509777059
"Vps13c",745.805556621092,-0.913566102738947,0.309896891418724,-2.9479
6794687612,0.00319870228858277,0.0141746130051788,-3.88841389800297
"Nat14",353.077733809625,-0.211549266359918,0.204795112181184,-1.03298
005556284,0.301613232294688,0.446364690951182,-0.17063965099172
"G2e3",887.127650384401,-0.243725921521238,0.114873071664063,-2.121697
60928822,0.0338631395226174,0.0863202372090462,-0.597053247603747
"Wdr38",68.2736418796754,0.018024686913613,0.203372650583817,0.0886288

636248284,0.929376868489762,0.956303244594089,0.000805346882466393
"Sgcg",3.91954146518909,-1.80912625962099,1.11819933634644,-1.61789244
620021,0.10568577847693,0.205489572165088,-2.86268901018311
"Cops4",1802.51701294811,-0.264632158875959,0.19476740820056,-1.358708
63262429,0.174238935505327,0.300274948248441,-0.318367499680727
"Klhl35",14.3540753993859,0.00412262209753831,0.646021168876348,0.0063
8155883453317,0.994908287291494,0.99685106811971,1.30023386536469e-05
"Mid1",1910.01164885073,-0.00528893410038294,0.293682168373287,-0.0180
090406226516,0.985631641207476,0.990729196996256,-4.92613668288321e-05
"Ror1",513.618923234692,-0.52745040795066,0.288160775139406,-1.8304032
1048377,0.0671896679981415,0.145417265692947,-1.01700243587833
"Lin54",467.398989354821,0.10635374811322,0.104620243031032,1.01656949
966819,0.309358269809704,0.454434996306428,0.0838812434679631
"Gnptab",1608.83755146927,-0.702969235195655,0.226797277170293,-3.0995
4883042014,0.00193815626747968,0.0097523021685984,-3.25492463830376
"Gdpd5",927.789115124268,-0.643678676660482,0.168737943270166,-3.81466
470543551,0.000136367960309661,0.00126725250828176,-4.29391872405561
"Sec31a",5351.68277476412,0.220334930114323,0.1113830214652 7,1.9781733
9856438,0.0479091485897277,0.112681855151087,0.481032326917064
"Fbxo33",1525.14704478415,0.414823922394691,0.118885511688963,3.489272
29652662,0.00048433750222151,0.00337512959911186,2.36089633132753
"Lzts2",4782.02966641113,-0.403936687157206,0.14528608200445,-2.780284
8117608,0.00543112416058403,0.0211611338396684,-1.55741388378032
"Nup37",305.384711144587,-0.182231792728722,0.17226728613653,-1.057843
29001558,0.290126893010857,0.433910727922504,-0.152148323661949
"Ccl12",201.857721952064,-1.57507463180427,0.598610743834925,-2.631216
77655458,0.00850797484288937,0.0298187150278683,-5.53263717499871
"Uvrag",1729.18053597241,0.098581895756413,0.162873750688207,0.6052657
06351484,0.545002463753487,0.67502713939123,0.0387429198949954
"Kcnh4",12.5608844974864,-2.06527567066239,1.01027854253235,-2.0442636
200959,0.0409275111469713,0.0996758196781971,-4.76217906530545
"Nfkbiz",3379.75020761295,-0.479567918890124,0.297720196679907,-1.6108
0075936444,0.107223155551256,0.207672304255328,-0.753781930474844
"Pdzrn3",1265.03825447157,-0.077610748010162,0.128819390884996,-0.6024
77216178187,0.546856524744297,0.676733213979709,-0.0303053016653508
"Parpbp",44.3417827406599,-0.536519193758244,0.422225679790171,-1.2706
9294796298,0.203837905364666,0.336217873174881,-0.584803719907633
"Rmi1",1615.63688894919,0.0583290683271,0.131761720016058,0.4426859965
0939,0.657992872711426,0.767179634762147,0.015459203 8318107
"Gm49322",287.137829735201,-0.327847841802272,0.203585609926188,-1.610
36844362986,0.107317445393731,0.207772371193225,-0.51515130301201
"1810055G02Rik",349.465721789264,-0.560994116425417,0.208609254941271,
-2.68921010519572,0.00716213247570988,0.0262248373730312,-2.0426066884
7185
"Ccl7",182.407079624374,-1.52872662799732,0.549325923412681,-2.7829136
8173583,0.00538731369328781,0.0210378214283448,-5.9030761231198
"Hacd2",3985.45769415081,-0.838794269685404,0.548056048298212,-1.53048
994220568,0.125895501147199,0.234833108329484,-1.21531240456927
"Shq1",242.491326394445,0.146292521158067,0.180672467794515,0.80971120
250846,0.418106177989454,0.561218734903979,0.0845050772106512

"Pcsk7",1813.7957344956,0.190760517965305,0.12816970767052,1.488343239
85262,0.136660397170895,0.24954655649113,0.264796541013736
"Ccl2",520.355983366816,-1.61366935018611,0.567450375022266,-2.8437188
8929105,0.00445903695164064,0.0181773578262783,-6.46690665020432
"Brsk1",364.397959830021,-0.20156951813678,0.322899319410527,-0.624248
816952471,0.532464191715347,0.664724895777591,-0.0823173658744294
"Dennd1a",2262.54469273321,0.462112287319191,0.13042973054854,3.542998
09848348,0.000395605554880001,0.00287281829014914,2.70449448500572
"Cfap53",44.9138480320293,-0.140215036343966,0.3697843556493,-0.379180
55267039,0.704553791910523,0.803759675923176,-0.0306306708610812
"Dcaf8l",0.253795162058599,1.33353138756081,3.97321945166281,0.3356299
35316742,0.737149947066294,NA,NA
"Klf16",650.500330204851,-0.172234605444708,0.118726147209259,-1.45068
807076793,0.146866738726438,0.263659669614961,-0.229605287300997
"Oser1",1604.7370862571,-0.136314426063188,0.111659782392415,-1.220801
46622646,0.222161198538719,0.358012281548298,-0.140020540929949
"Emsy",3300.88861255948,0.165516871654351,0.186760269051383,0.88625312
2760353,0.375481143387838,0.520533323508865,0.108066192404457
"Crb2",427.235224466076,-0.366496218053705,0.501537994873435,-0.730744
672985727,0.464935123391374,0.603790131602838,-0.184907825874562
"Kank4",386.333160546609,-0.0973295936976902,0.330436209003044,-0.2945
48814705696,0.768338568090087,0.849989526276341,-0.0158191006815536
"Tmem98",1149.77104089869,-1.03418388452211,0.232511413280102,-4.44788
438525489,8.67201819380281e-06,0.000152605605363508,-9.08804984694718
"Ptprh",35.2560680460304,-1.44766673651762,0.916820984434294,-1.579006
98292903,0.114334455373895,0.218279957982983,-2.20331522931897
"Sstr1",0.580052293987603,-2.66329180858297,2.94229653016267,-0.905174
506131688,0.365372945595085,NA,NA
"Abca17",23.5294151290656,1.08516708377781,0.671050314310479,1.6171173
1689277,0.105852959555976,0.205679982877783,1.71611990414299
"Rabgap1",2167.20246203136,-0.0410284843487918,0.176755284428847,-0.23
2120270018336,0.816444601592249,0.882692059184005,-0.0051194884502532
"Haus8",538.95979475753,-0.84597043022866,0.206556548501104,-4.0955875
5879454,4.21099033833898e-05,0.000507452964257932,-6.4176218071514
"Myo1d",2449.39437704168,-0.368373878753425,0.205888480719377,-1.78919
130136043,0.0735840120952131,0.156068864117531,-0.684238983211278
"Thyn1",973.780350925815,0.250299652863313,0.127057696373356,1.9699684
4746668,0.0488419868322532,0.114365444848718,0.542738829469981
"Ccr3",6.34774851003471,-1.82906381143768,0.985158852058817,-1.8566181
5616359,0.0633654989628282,0.139074054122989,-3.60828330057597
"Foxa1",533.187319950247,-2.71463605611897,0.739172031678859,-3.672536
21589726,0.000240155103435785,0.00194823172382901,-16.9415906243291
"Fignl1",135.061908819387,-0.999148606134304,0.362650889079847,-2.7551
2520779817,0.00586696788927922,0.0224346224563966,-3.79391699792191
"Prdm8",3.69916104409063,-2.52385523238509,1.06518191181216,-2.3694124
0214202,0.0178163749604524,0.0525860852223586,-7.43352023934815
"Tnni3",361.164250197406,-0.882919489533436,0.475895803659088,-1.85527
899751333,0.0635563957866906,0.139379440450893,-1.73984165974119
"Stab2",154.364674493719,0.257552539697345,0.442066561254374,0.5826103
17700877,0.560155658098563,0.68847169828579,0.0961394870835743

"Rcbtb1",1689.4198526942,0.48658096764421,0.238080635265229,2.04376541
209302,0.0409767276342923,0.099770758668011,1.12151080781105
"Slc25a21",660.40747996798,1.4690830783155,0.546629322599674,2.6875306
8592954,0.00719824880236666,0.0263430556363713,5.34239492336659
"Galm",3388.25956554605,0.984554064275998,0.371400532719937,2.65092259
579073,0.00802722356479642,0.0285663086450813,3.50060882921523
"Tab3",748.088465133289,-0.363689324187255,0.243588147225844,-1.493050
16820075,0.135424055215415,0.247701522381816,-0.507539652237799
"Mbd3",5403.81536335619,0.126626018181474,0.134381281532705,0.94228911
0039896,0.346044642046396,0.490775271357334,0.0901284682281183
"Plk5",72.9657756947528,2.04800483542506,0.378231776525696,5.414682114
33031,6.13976853371755e-08,3.15865981188456e-06,25.9387241144533
"Tgfbi",3176.34919295587,-1.27498753016668,0.22319941001378,-5.7123248
2239968,1.11443111540203e-08,8.69167115524347e-07,-17.7933822628216
"Tstd2",2121.80667956936,0.0662747769670907,0.168639322557364,0.392997
172676301,0.694321569612977,0.796145898336474,0.0151088478375549
"Cdcp1",1929.68321166978,0.461074078079467,0.154917543468542,2.9762547
7241765,0.0029179234242972,0.0132464891564486,1.99369479428362
"Reep6",1128.81774165362,0.430973570715112,0.528846349879673,0.8149315
40726659,0.415111497788281,0.558427210147365,0.251098562456582
"Cox18",535.352968491272,-0.0810580096130819,0.208030303515222,-0.3896
4520189315,0.696798922439473,0.797732686848972,-0.0183176281513792
"Slc12a8",70.9674530986601,0.17126536484848,0.22972924226297,0.7455096
40659647,0.455963685270716,0.596109691257103,0.0886008110649351
"Fbxl21",94.2080784383527,1.23597529031525,0.289953049742614,4.2626738
7569265,2.01995212747426e-05,0.000290844253564796,10.0642039853415
"Ntng2",756.967772735624,-0.860721704758192,0.272215746201425,-3.16191
005395148,0.00156737969630433,0.00832491673073869,-4.12156781596739
"Tdrd7",1526.15525644142,0.52486104924571,0.206992074689283,2.53565770
589808,0.0112236379343903,0.0370912699058885,1.72908841032011
"Gnptg",2421.19739973385,0.860053660885819,0.143574282987331,5.9903044
1239752,2.09448626020303e-09,2.65183721437913e-07,13.0236575263138
"Npffr2",0.207362170746708,0.880352364710176,4.78471990596588,0.183992
455569343,0.854019373469052,NA,NA
"Prdm4",1017.47456822081,-0.184211319608003,0.092881119903164,-1.98330
209411835,0.0473336956400797,0.111624624769022,-0.403904244890136
"Eif1",18293.3931299339,0.37518408582455,0.164017603102009,2.287462313
3665,0.0221688527355467,0.0623830786777362,1.04093369476213
"Ccdc180",26.7805595256031,2.26341232407816,0.721986214347065,3.134979
97482556,0.00171865843175681,0.00892420828292791,10.681014833712
"Gc",1087.02247741805,-0.826982722672995,0.626938610625813,-1.31908086
159743,0.187142080671656,0.315921756617831,-0.952899692316279
"Leng8",17485.3414080976,-0.0509536761152617,0.203708970507352,-0.2501
29761042717,0.802487001346046,0.874030362654743,-0.00686041131113227
"Capn5",1195.44358340879,-1.19167871614769,0.263829804382998,-4.516846
45309347,6.27673847544281e-06,0.000118359619723582,-10.774900289578
"Igfbpl1",1.99831484129547,0.351506943123727,1.8314893674726,0.1919240
97058121,0.84780166159349,0.902791705035749,0.0359463029037895
"Krt17",37.7572664677984,-1.75117764302971,0.729833708453265,-2.399420
06342921,0.0164210647468252,0.0494483274800922,-5.26548829578813

"Mpv17l2",910.218704658313,0.0223468143078527,0.290976742762965,0.0767
993142532937,0.938783196275565,0.962072677440396,0.000864045897020032
"Wdr20rt",2.15177835041599,-1.51646556259096,1.394479860628,-1.0874775
6450784,0.276825808496362,0.420369907637233,-1.31419972380055
"Aldh1b1",129.869087457249,-0.191905135325252,0.337056149051882,-0.569
356577131345,0.569114178774378,0.696059983549293,-0.0695309609916089
"Pcdh17",1302.54575734539,1.72717098352628,0.223419209790116,7.7306288
2618203,1.07016629690912e-14,1.60508480439492e-11,42.9292828757198
"Ankrd11",11435.1769169219,0.0734205557556578,0.212806340045768,0.3450
1112955501,0.730086003706389,0.822987622146452,0.0143033608373822
"Dcaf10",1262.58094990386,-0.164151545941509,0.148801999736019,-1.1031
5416615852,0.269960221577905,0.41230755236153,-0.145435925915663
"Utp6",2034.0839942405,-0.0972860433838181,0.183972376853695,-0.528807
884355299,0.596938727788655,0.719305337063229,-0.0320527687320252
"L3mbtl1",36.4185827830908,-1.62222732753988,0.596405549117743,-2.7200
0709909494,0.00652805148061985,0.0243979198330699,-6.02374763343408
"Iqcg",404.451800647726,1.67747529974619,0.534707751346992,3.137181564
17301,0.00170580457446793,0.00888586096526203,7.92320888605965
"Kcnh8",44.599968153305,-2.20006246135615,0.591622421965756,-3.7186935
1071263,0.000200255851413253,0.00169543623183836,-14.0359919480703
"Tsen34",3133.38993915496,-0.197278512431847,0.213654081414502,-0.9233
54757024811,0.355822360988795,0.500889783738324,-0.136392286259281
"Krt33a",0.45339459023758,-1.49943290713355,2.69681544964572,-0.556001
30417917,0.578209963215383,NA,NA
"Chrna5",2.30194063203284,-1.22100363837437,0.969051322744128,-1.25999
89388764,0.207669745035053,0.340765872140779,-1.31448322451393
"1600002K03Rik",452.242780121268,0.0477454375542648,0.181711125444462,
0.262754619110306,0.792739715441601,0.866852053820893,0.00682220034296
491
"Mboat7",2216.39541538141,0.0839219760577083,0.111992956186305,0.74935
0485204626,0.453645986457463,0.594035557014614,0.043707916368417
"Prpf39",3779.89183067371,0.0912089841552544,0.172659263936402,0.52826
0008040174,0.597318884714927,0.719543038068185,0.0300204281635555
"Trmt10b",416.897235106825,-0.128605775064748,0.141593630254963,-0.908
273732604864,0.36373361691368,0.508647928177791,-0.0869374004070032
"Ky",37.1494728113855,-0.612836271960478,0.60476801792173,-1.013341072
60909,0.310897275548908,0.45612303074888,-0.481072000755241
"Togaram1",1287.79984934981,0.223783736955111,0.1379838071572,1.621811
5847475,0.10484369718071,0.204342503761444,0.355359120087597
"Frmpd1",36.985549660724,-0.0192425271060133,0.318372959020598,-0.0604
40205616735,0.951805037719222,0.970064012620845,-0.000584842307147056
"Ric8b",1139.9556573868,-0.251752825040507,0.190183720809979,-1.323734
88103141,0.185591109681334,0.314070691728827,-0.291564308238654
"Midn",4392.84773998306,-0.292202126020752,0.137252534260561,-2.128937
92886938,0.0332593962000499,0.0851150685271786,-0.719913335880385
"Rsf1",2017.05948417085,0.21207144886876,0.174721308952416,1.213769803
69645,0.224835640243277,0.360958856604645,0.216098960902456
"Rubcn",2361.89099797601,0.108025027344538,0.149140015843889,0.7243195
37806757,0.468869584898831,0.60695917981393,0.0539362198359688
"Cnot3",2789.24432140803,0.166943499753077,0.204505601691822,0.8163272

71096718,0.41431298093285,0.55770432534374,0.097482706786738
"Grhpr",4960.30351448897,1.37811696827623,0.469162189895731,2.93739989
700045,0.00330977005499262,0.014560897232005,5.8286294249721
"Muc20",613.860002361877,-0.607252507552612,0.623621388168063,-0.97375
1893494967,0.330179744216886,0.47480231969473,-0.452316117444694
"Cbarp",690.14216736019,-0.676698254593641,0.248098405509894,-2.727539
71635926,0.00638085756473417,0.0239779863672454,-2.5245034363993
"Aamdc",1400.39463145033,0.287582227907565,0.38208016964621,0.75267509
4794514,0.451645179472149,0.592250837941217,0.150642766601804
"Zcchc7",3010.77500843375,0.201470449341836,0.195943764865394,1.028205
46231843,0.303853189531239,0.448624071594737,0.161492667869934
"Lrfn5",0.561724898437956,-1.24886476490044,2.12712612553448,-0.587113
641221741,0.557127386557504,NA,NA
"Gtf3c4",1056.85446785242,-0.426252472117529,0.160769187404731,-2.6513
3188142857,0.00801750163473167,0.0285525565066663,-1.51575754272293
"Zswim4",581.206568062451,-0.380742860163223,0.186217313526817,-2.0446
1579297992,0.0408927512613542,0.0996160499867421,-0.878157509952227
"Sbno2",5707.96881630917,-0.61368141956991,0.285287385897925,-2.151099
03173035,0.0314683839258916,0.081504213775998,-1.53856102900769
"Ndufa3",4532.01899487772,0.781410375723078,0.309052672757699,2.528405
17038891,0.0114582035400139,0.0376980897343412,2.56157718500308
"Tnfsf9",84.10759945619,-0.712949878392281,0.346607720198985,-2.056935
94471289,0.0396923878387944,0.0973242175654945,-1.66096462528411
"Kcnc2",252.769164867858,0.732803412956305,0.239900674932758,3.0546117
1862773,0.00225352065776899,0.0109655966521537,3.307136295179
"Melk",82.5689283240169,-1.35130433379106,0.533539135788209,-2.5327183
0152581,0.0113181869539583,0.0373404414937865,-4.44265395831002
"Thrsp",21999.9880354577,-3.80249897884527,1.21363785343585,-3.1331413
799267,0.00172946115918844,0.00895882935224659,-17.9292226845124
"Isg15",1028.88359479423,-1.12138049543037,0.340002327336314,-3.298155
35151074,0.000973222731568527,0.00575027176367368,-5.78465043843244
"Caps2",115.337016939055,0.61549405770775,0.360701415902429,1.70638104
141583,0.0879371580883124,0.178827566583846,1.05947038831109
"Rnf38",2617.89298432318,-0.271140479150355,0.138001664137942,-1.96476
238778781,0.049441745344581,0.115326612122101,-0.585659929305085
"Arhgap45",5092.11506296423,-0.903455496006969,0.40179499506122,-2.248
5484068046,0.0245412420453911,0.0674140796563871,-2.43653037928086
"Slc51a",2221.20388948845,2.28093341719548,0.71564456600549,3.18724339
643485,0.0014363584654034,0.00777566321699741,11.077938253253
"Alg8",518.592770469817,-0.0904462159980714,0.16500738500658,-0.548134
351650168,0.58359965484878,0.70756286968297,-0.0312879502113368
"Dok3",1028.3036156773,-0.255535732953045,0.383643973161652,-0.6660751
91660506,0.505363040608033,0.640299471426046,-0.113922758515397
"Usp35",697.701558097154,-0.962611988406038,0.272439429780355,-3.53330
642771536,0.000410396499153507,0.00295491541377219,-5.60652680873728
"Abca7",1658.77427900661,-0.368795048206887,0.161740014678041,-2.28017
197192055,0.0225974909646697,0.0632599913019853,-1.01805954140824
"Prkx",1901.32511301528,-0.153562545643931,0.135637236975196,-1.132156
2505141,0.257568749534117,0.398518163651883,-0.141277880096554
"Supt16",2983.352473689,-0.260923237635781,0.0962734223743031,-2.71023

124763689,0.006723631273455,0.024985203462898,-0.962668846548926
"Dagla",591.942989995657,-0.806694705890078,0.3100003157061,-2.6022383
3660503,0.00926174617785113,0.0319112081596998,-2.77890029165915
"Grin3b",33.6167147537512,1.46774313238526,0.520198354854212,2.8215066
7853727,0.00477986422480059,0.0191843953592346,5.80295448313685
"Wdr18",2561.68256435101,0.903806156111493,0.251887501215482,3.5881341
9383726,0.00033305286672168,0.00251019126221079,5.41144566092004
"Selenoo",1895.10772773539,0.609047729741927,0.132957538615992,4.58076
868812215,4.63270010455495e-06,9.35310419996925e-05,5.65026805983624
"Bbs10",301.889485447256,0.223470305584474,0.146764887276832,1.5226414
8278844,0.127848425436166,0.237255315053602,0.321488502180137
"Tmem161b",1246.30046540713,0.421783995371824,0.133352307955904,3.1629
298497878,0.00156189963226351,0.00830033225126028,2.02096103056855
"Fbxo45",924.426669131233,-0.512264872499089,0.14338213225893,-3.57272
46096048,0.000353286235919236,0.00262414286779172,-3.04439063047864
"Dym",1152.51073894105,0.346343014513156,0.203520744156952,1.701757803
35228,0.0888007796477117,0.180163862087883,0.593593308684569
"Xylb",11988.2143980279,2.11821854946461,0.682287577260004,3.104583199
31773,0.00190547328832488,0.00961624417219306,9.83764560615469
"Dync1li2",4005.63552878506,-0.502945604002574,0.143732217216113,-3.49
918489914028,0.000466682837978318,0.00326728257626616,-2.8787583663352
7
"Mrps2",1769.56539431398,0.275736711307228,0.250247149646657,1.1018575
5041191,0.270523605859514,0.413008380421072,0.243830498051374
"Kiss1r",144.668619805375,0.179680789160405,0.247633092182824,0.725592
801739717,0.46808843520587,0.606350538775183,0.0898937621688232
"Krt20",761.455420329683,-3.41327663126427,0.68490958774924,-4.9835433
6443301,6.24303141592712e-07,1.88140071943968e-05,-37.1395520821757
"Cd99l2",2140.20000745829,-0.574229056286385,0.261309308800133,-2.1975
0708049055,0.0279842513567435,0.0745507491397438,-1.49085665163866
"Ggta1",965.338128289354,-1.57981355338936,0.280822472357636,-5.625666
42237028,1.84793146904393e-08,1.28682027797923e-06,-21.4275424894579
"Ugt2a3",37.2256713430772,2.16103277825444,0.688624851532472,3.1381858
6919316,0.00169997041313396,0.00886494333118106,10.212286130214
"R3hdm4",4679.01059124565,-0.118846703847926,0.119085468804735,-0.9979
95011824656,0.318281777462031,0.463296610448278,-0.09143920433073
"Acta2",8486.12188005702,-1.62261078965238,0.275454967277511,-5.890657
21228274,3.8466272667953e-09,3.98944353446675e-07,-23.9082676432765
"Cep19",735.934255796508,0.486739648596699,0.165470138814889,2.9415558
123222,0.00326567987446394,0.0144026750039127,2.06394224669984
"Zdhhc17",1747.53095508111,0.0221804689628717,0.11260013276496,0.19698
4394407161,0.843839749977416,0.899917269871446,0.0023389846223409
"Twist1",178.005287431022,-2.44567151112416,0.480810746489662,-5.08655
750517162,3.64621199295061e-07,1.24452665318564e-05,-27.6218303136029
"Mlc1",0.120701025406302,-0.999956691173236,4.78169649435351,-0.209121
740025541,0.834353203805696,NA,NA
"Ugt2b35",4.03641368403791,0.0756845941503143,1.02296461793925,0.07398
55443903625,0.941021887639446,0.963455406699954,0.00281766741791208
"Plekhs1",157.64885056555,-1.15008370794771,0.502809978190749,-2.28731
28176295,0.0221775708727468,0.0623980197513443,-3.19058722410232

"Tk2",1608.40196798917,-0.132044775863801,0.117657690260155,-1.1222791
7760271,0.261743756654418,0.403277737435626,-0.119913793206368
"Pim3",7881.05674049434,1.46120126757644,0.401336118723211,3.640841677
10852,0.000271748212339361,0.00213802139914187,8.98328195016504
"Ppp1r26",425.354485141055,-0.174987427644274,0.2012644286074,-0.86944
0411577232,0.384606287483995,0.52925777354527,-0.111340944679669
"Krt25",0.825195398248248,1.18105198704686,2.17976296235196,0.54182588
0816191,0.587938460545641,NA,NA
"Polr3g",382.371312320293,-0.232394377059984,0.278137312046729,-0.8355
38300668343,0.403414691465752,0.547298890495354,-0.140078083070362
"Plppr3",812.135442024287,-0.853330387666327,0.433056236391863,-1.9704
8400636394,0.0487829262026724,0.114268319930287,-1.85104932229565
"Ugt2b1",3.22445395467382,0.881744823734616,0.747398974716248,1.179751
18184952,0.238099190945747,0.376242647302065,0.861924086395491
"Lysmd3",985.225549658929,-0.154377860169884,0.11863364452066,-1.30129
914488971,0.193156079825156,0.323414104287753,-0.17426508168917
"Ddx11",432.667961888422,-0.666426014137809,0.220013581024636,-3.02902
216778693,0.00245346679505634,0.0116620418259407,-2.96653942193159
"Alg12",709.314508108626,0.171105720697955,0.149452919253972,1.1448804
1820841,0.25225869662118,0.392415834268372,0.160057971806167
"Ids",2380.83541474992,-0.306433676821041,0.184629678310697,-1.6597205
802708,0.0969706772028953,0.192721125903809,-0.504546443716972
"Krt222",38.4939946842846,-1.77183411275118,0.703546927548159,-2.51843
060266921,NA,NA,NA
"Ythdc1",6608.97966896442,0.0697497031359318,0.123030972337364,0.56692
8000411725,0.570763099592739,0.697376796331572,0.0251398448352801
"Misp",1063.35580123825,0.705426967792582,0.43519433320031,1.620947043
60015,0.105028996444193,0.204634284838564,1.11918166052566
"Cdhr3",5.96175398426524,-4.137209358324,1.07626505463939,-3.844043194
0905,0.000121023689989798,0.00115598222147837,-27.9791396719563
"Tmprss11b",2.98921318425137,-2.4263452895071,1.29461309633798,-1.8741
8565158224,0.0609048410824479,0.135099270924411,-4.85692559631173
"Palm",5266.32857676976,0.47301412913339,0.173297544459438,2.729491237
79479,0.00634321347787378,0.0238626232667534,1.76691680871974
"Syt1",32.9071965364159,-0.685102790740276,0.612935418787615,-1.117740
58039493,0.263677805746537,0.405605324654762,-0.618219424540147
"Zfp983",488.738035548456,-0.502454947320265,0.172996663671011,-2.9044
1986948249,0.00367934372562035,0.0157843441060826,-2.0845532848459
"Pawr",1576.89418956697,-0.122240252365973,0.244637668274082,-0.499678
783027887,0.617301274328665,0.736020317180965,-0.0374663385667353
"AI182371",53.1993454608588,-0.183843431638577,0.291672516689385,-0.63
0307694825975,0.528493289496559,0.660930162183561,-0.0761308702384717
"Zhx3",2541.30343989364,0.511701964831798,0.172878951658639,2.95988586
188438,0.00307753037624412,0.0137785734273267,2.19245898476994
"Hykk",5832.25576031179,1.78137420533997,0.633351540959902,2.812615254
14485,0.00491404095189722,0.0196139141207967,7.00350129802979
"Cox8a",18750.9925119336,0.493004944716577,0.395382951568878,1.2469049
1271901,0.212432365192287,0.34672746162056,0.522198834393716
"Rnf126",2354.69154723428,0.301116708599976,0.192213269724046,1.566576
0695517,0.117213815029741,0.222491721714358,0.452537877852595

"Cerk",1753.92990755186,-0.935359833228542,0.191047056792502,-4.89596567951552,9.78241395544844e-07,2.73654960263032e-05,-9.82710330255871
"Rnase1",81.7139718348318,-2.55787380439714,0.736309680894433,-3.47391032709221,0.000512932425258133,0.00353148178943611,-14.4418519973016
"Uba6",1078.99705831629,-0.0645129685179489,0.126042654425674,-0.511834416784609,0.608766900893269,0.728785966330016,-0.0204103026130239
"Gramd4",1813.99672659231,-0.32254306771409,0.153975279544897,-2.09477176250257,0.0361912864132306,0.0907266945585906,-0.774072284873203
"Dennd5a",3450.02366208896,-0.366572970456611,0.0987505510601736,-3.71211063149653,0.000205538105890392,0.00172964263644837,-2.3313456091761
"Dcdc2a",3748.59541355058,-0.570283368444814,0.575653202155417,-0.990671755684678,0.321845886986854,0.467055009263894,-0.43416142490525
"Cd276",397.323084254591,-1.60629771797994,0.286808825998532,-5.60058677548564,2.13627433951451e-08,1.43251186746293e-06,-21.6144723702279
"Ptprq",2.36546449250475,-0.391207358479912,1.39525467243437,-0.280384195236095,0.779182761329448,0.857300145384565,-0.0602330991451079
"Bbs9",545.815094979465,0.0909176326013462,0.13362442434849,0.68039681401534,0.496253237803593,0.632001674114596,0.0417187590979384
"Myf6",0.121527413527064,0,4.78471990595379,0,1,NA,NA
"H2-
Q4",9177.48614750066,-0.917272154087488,0.251883440000242,-3.64165327457258,0.0002708927627588,0.00213449719005771,-5.64078728038489
"Chst4",2.22407550786999,-4.11773753169202,1.91745842184157,-2.14749769006061,0.0317536808914832,0.0820913908806868,-10.2940231785472
"Cog5",1446.59563535175,-0.0692434981345262,0.204499773043299,-0.338599388664677,0.734911543200456,0.826648853340777,-0.0131822501448363
"Pknox2",566.157945970262,-1.16257296628552,0.34893821696831,-3.33174444572549,0.000863034681842488,0.00526355995442522,-6.09995953262751
"Aldh5a1",2284.85094258924,1.01142922020166,0.379216783205489,2.66715310343633,0.00764968146998844,0.0275190939671281,3.63393895085911
"Ibtk",2000.40953776146,-0.25585195184313,0.192325938622284,-1.3303039292355,0.18341815310715,0.311576733065859,-0.298351426692114
"Ttc38",2643.10435700049,0.697064396311923,0.258366463384004,2.69796779033156,0.00697641903895568,0.0256847089164573,2.55255184546403
"Acss3",1298.05162352817,-1.35596667991841,0.577735128471199,-2.34703865680899,0.0189232913740224,0.0551107296804614,-3.93014853831476
"Fbxw2",2216.80162211883,-0.478629401830276,0.185002265249449,-2.58715427719176,0.00967722528379896,0.0329641052731503,-1.63324435216808
"Ascl3",13.5790231465263,1.35569697106022,0.441649113031896,3.06962457538619,0.0021432800007371,0.0105369827166848,6.17230399764654
"Pip4p1",2294.0612395944,-0.282922566720225,0.127808185113718,-2.21364982585812,0.0268528769347298,0.0723472978407298,-0.743033079280234
"Dock4",1196.02786480325,-0.01483107783303,0.254964916471494,-0.0581690925884237,0.953613939562375,0.971185435097736,-0.000433628912070937
"Tdp2",1614.23470493619,0.55086500387573,0.0931234580476682,5.91542684759142,3.31016004734177e-09,3.56582876260054e-07,8.17852698598087
"Apex1",1277.68612548298,-0.658320822820687,0.211107485186879,-3.1184153524349,0.00181826363052254,0.00928076027956245,-3.08081756065656
"Tmem59l",30.3538758724022,-0.486269827799474,0.530624401861335,-0.916410602478377,0.359451576452697,0.504577886081692,-0.332624642372888

"Ints6l",3254.56411139026,0.339857089391138,0.288162850159935,1.17939244841072,0.238241940741584,0.376346217336094,0.332123902492884
"Rusc2",1684.85500837792,-0.267828418197764,0.223591998734708,-1.19784437597672,0.230977607770944,0.367851130957924,-0.267849029763999
"Nme5",159.768136474044,0.914474914310022,0.373364613164997,2.44928116394871,0.0143141662074636,0.0447330795177223,2.84131198234443
"Fnip1",2454.70970137869,0.651742526260514,0.140422884177481,4.64128428979405,3.46250358360131e-06,7.4352307128919e-05,6.19591797285527
"Fam214b",1740.33231770868,-0.027091082157906,0.135979176278012,-0.199229638680247,0.842083115809571,0.898978131409057,-0.00288510733469428
"Ripor2",254.248076444117,-0.6640821595967,0.25061774181694,-2.64978111598248,0.00805439339764743,0.0286264240735198,-2.35976619382105
"Mettl25",246.946982555239,-0.00243389704307889,0.190978005555358,-0.0127443840247529,0.98983172800443,0.993244765201501,-1.64973306206181e-05
"Tmtc2",356.93237479009,-0.954691146154728,0.318065377652325,-3.00155632531088,0.00268603342681811,0.0124547633189297,-4.18694326181868
"Fam122b",277.189062742516,-0.31831444897523,0.153299851744326,-2.0764172003644,0.0378553817875704,0.094014040771118,-0.752594397086118
"Parp2",1155.16732459264,-0.290090948612863,0.160958998179708,-1.80226611679691,0.0715035467570964,0.15248563432898,-0.545569663344903
"Tmem63b",2507.26612928728,-0.0499085558677719,0.140010903082293,-0.356461923814872,0.721494662189136,0.816039382989951,-0.010146043164691
"1810046K07Rik",0.370165826670535,-0.0725501076226697,3.64882828723982,-0.019883124639321,0.984136607072032,NA,NA
"Prtg",65.9399295184162,-0.156932074986039,0.367107324857119,-0.42748282139867,0.669027694756913,0.776067587400414,-0.039784739416796
"Zfp57",505.834314859805,-0.475873645591662,0.308258029333509,-1.54375101476046,0.122648662439129,0.230296958805676,-0.698766045535201
"Adamtsl2",2802.69104151147,0.0985881591586241,0.485926143785389,0.202887126818527,0.839223256341316,0.897124821651609,0.0107027573773801
"5031439G07Rik",3306.3521012568,-0.447981669998638,0.140337961177162,-3.19216316270341,0.00141211539801374,0.00767375307426753,-2.1816480939611
"Dnajb5",866.16099138617,-0.602798851090404,0.195241289611854,-3.08745579528177,0.00201877794320702,0.0100721935354786,-2.77165511262437
"Fmnl2",1351.68220612643,-0.747861676403676,0.233273125632474,-3.20594871087699,0.00134617980455442,0.00739923133738739,-3.66929294179496
"Sugp2",1684.1206329831,-0.145689826160788,0.171138693477574,-0.85129682364835,0.394604488929193,0.538908617016278,-0.0900668002265653
"Ptpn23",3691.27699979895,0.147056758690441,0.231392361814142,0.635529874614269,0.525082888867536,0.657853297321304,0.0615834477878179
"Smug1",675.31913282226,-0.297941351055571,0.164288767487288,-1.81352234612524,0.0697512952428248,0.149665539192759,-0.565895564522141
"Phf24",170.512179744048,-0.421359658044161,0.425836969849314,-0.989485854629444,0.322425487313939,0.46758290454795,-0.320308599180672
"Slc2a6",207.417248766388,-1.1722042474971,0.505744906891082,-2.31777766127766,0.0204614077662814,0.0584723037706221,-3.32812473377607
"Galt",2108.94908399637,0.962585484670808,0.197962069641794,4.86247434375978,1.15927408699533e-06,3.09213763997742e-05,9.99554814279413

"Sigmar1",1833.99150656248,-0.568856812793393,0.268227853603727,-2.120
79694614344,0.0339388938127786,0.0864681238157007,-1.39254978416233
"Slc17a3",13755.8710398271,2.16124960073171,0.835977323341944,2.585296
92180141,0.00972951694732098,0.0330844299684103,7.36703530674413
"Zranb3",286.988844935156,-0.0918270805776213,0.24604102465991,-0.373
218574847623,0.708985772613936,0.807040959450437,-0.0196859682112312
"Slain2",2429.8092863063,-0.111667229200536,0.0873745483149466,-1.2780
2925856652,0.201239110017537,0.333055410893401,-0.122772133562954
"Hyal3",103.783965904896,0.525132503063883,0.285125877856162,1.8417567
2517805,0.0655107426428573,0.142606727704637,1.02278198298775
"Arl5a",3849.45245213037,-0.177232293173356,0.199686279610049,-0.88755
3684306499,0.374780876719356,0.51989433090152,-0.115932906384761
"Dgkb",185.192496349369,-0.169542775617129,0.321650178024146,-0.527103
005689622,0.598122059599747,0.72019909331661,-0.0556486160833889
"Slf2",1262.32185500767,-0.293162490082139,0.0946636054635768,-3.09688
701002348,0.00195564399888825,0.0098200223256047,-1.35538748733605
"Myrf",448.484293277344,-0.020927661679801,0.427334319206343,-0.048972
5742567749,0.960941152278652,0.976364282810274,-0.00050057965604135
"Vezt",1500.6417354691,0.0939200723666974,0.120257643480261,0.78099046
055325,0.434808105362595,0.576923337077909,0.0516603494166526
"Colec12",3614.99209463047,-1.33023323883513,0.302413254327353,-4.3987
2664243478,1.08887874479092e-05,0.000182867853281079,-11.4489806439162
"Rab3gap1",2785.38694429235,0.101752378594976,0.116879128193431,0.8705
77836845078,0.383984702186424,0.528626260201292,0.0648644550171552
"Prr5",1341.95193136175,0.669439748244681,0.238323822348116,2.80895019
914056,0.00497033345483348,0.0198061642555372,2.6253834085964
"Mbnl3",172.211485808842,0.188425058850596,0.3407436287498,0.552981898
860249,0.580275805055169,0.704803939884488,0.0659177962127607
"Slc17a2",11.2363335522618,1.04933307727108,0.643694121009896,1.630173
46752333,0.103064839860674,0.20188449343012,1.6789954217352
"Lmo1",310.623263923755,-2.12985815655182,0.64734290598636,-3.29015447
123275,0.00100132396536607,0.0058806670712974 7,-10.939132691877
"Metap2",8479.12398826817,0.628960144494735,0.257727828282939,2.440404
4712015,0.0146708255541394,0.0454844580465273,1.94372874001446
"Rpp25l",1113.88844447157,0.343609259278502,0.180255214501983,1.906237
55450205,0.0566193918943894,0.127936597885827,0.706536391508876
"Il5",2.29618086237511,-2.43916002536168,1.51000979544494,-1.615327286
43191,0.106239837601039,0.206197924899966,-3.85123554946916
"Rfxank",1100.80482020182,-0.0211557009457107,0.214040527162534,-0.098
8396974449885,0.921265548793209,0.951522177686725,-0.0010512751077896
"Slc9a3",2833.17991970501,2.35662053023785,0.833143316453945,2.8285896
120108,0.00467536097584599,0.0188542271576101,9.35818286957078
"Frmd7",3.84744425952953,-0.305831710611291,1.22099121417136,-0.250478
223808391,0.802217542956123,0.874026024376205,-0.0411787496980856
"Fam110c",394.806731131262,-0.747148797138443,0.362485783704615,-2.061
18096412654,0.0392857788970143,0.0966066486232796,-1.74616721715174
"Acaa1a",8170.6830212424,0.133729457087853,0.23603662047566,0.56656232
7567486,0.571011575942392,0.697525998317987,0.048171422234233
"Hoxc9",883.944167322784,0.322398986726791,0.302183024932804,1.0668997
2674171,0.28601711826192,0.430139743298953,0.271990337865596

"Meox2",114.807518380628,-3.94233066926089,1.25055955604533,-3.1524533
5594236,0.00161904686047578,0.00853514339801809,-18.7795409896503
"Tm6sf2",157.491908015586,0.46327042027514,0.278802789252794,1.6616419
8542177,0.09658457975126,0.192163891427558,0.764121321159677
"Mgat5",1868.31890640625,-0.369092416709955,0.169645054123203,-2.17567
449058612,0.0295796089593981,0.0778330925088184,-0.94236254502518
"Prickle1",539.205292930988,-0.731961959814948,0.15881133572998,-4.609
00323299007,4.0460404221052e-06,8.43740065777616e-05,-6.86598703927053
"Surf6",901.174919292281,-0.132660707527278,0.143825512693567,-0.92237
2568279479,0.35633427336306,0.501501780138079,-0.0915555381577254
"Pphln1",983.475734112439,-0.376584512467822,0.171978887385996,-2.1897
1362236227,0.0285450125120001,0.0757960852456731,-0.97147831805674
"Ccdc38",1.17055208076051,-1.77660983396541,1.69134737565043,-1.050410
96793152,0.2935292051427,NA,NA
"Sostdc1",3207.85396021707,1.86666433185225,0.482856143413363,3.865880
87842601,0.000110688957738045,0.00107267062523678,12.7635111823443
"Cd200r3",167.337246221544,-2.93199257684342,0.636048202860968,-4.6097
0184909762,4.03246853805948e-06,8.43740065777616e-05,-27.5028268367031
"Gatad2a",3518.79436949012,0.275956556878162,0.0726050505985078,3.8007
9009109366,0.000144235450408967,0.00132264609837491,1.82907343013129
"H1f2",4126.24445264929,0.0624806356378605,0.407032655496544,0.1535027
59039418,0.878001814695877,0.923773558241121,0.00495398364586698
"Sapcd1",25.6411711351213,0.314617728195539,0.485466965227227,0.648072
373056897,0.516938141664579,0.650568636556894,0.135256827125234
"Dipk1b",473.244984869151,-1.78774527714658,0.499816205113623,-3.57680
535135945,0.00034781897064898,0.00259341273029209,-10.6456309193634
"Ankmy2",1475.69971372339,0.00579825830288223,0.12631063038413,0.04590
47531094479,0.963386165739358,0.978046740493088,0.00012870868353381
"Rorb",37.2422647298412,0.959560642611245,0.351198591686709,2.73224513
231315,0.0062904319360359,0.0237098089868216,3.59054776109315
"Slc26a8",54.3437476196228,0.446315064608102,0.329067902249427,1.35630
08168138,0.175003475718782,0.30122953181074,0.535525742929766
"Gxylt1",1104.58686875248,-0.310505807257399,0.103186029325675,-3.0091
8456971904,0.00261949927574226,0.012249915808011,-1.36691990962368
"Arhgap36",148.521298846557,1.1365321555217,0.495050608830417,2.295789
83491571,0.0216879009351528,0.0613155240548429,3.17288207331169
"Ndufa13",10258.6273285256,1.22344614336771,0.258163804574029,4.739030
49804523,2.14743185243011e-06,5.05683892013071e-05,12.1025542373281
"Rif1",1316.33536724277,0.00802254623425788,0.120729525060676,0.066450
5739604783,0.947019106947527,0.967309892988783,0.000266640286265877
"Sh3bp4",1665.40437271331,-0.126614604869745,0.137338575974016,-0.9219
15812595144,0.356572490436779,0.501682811558605,-0.0873371348454269
"Nepro",295.063578602369,-0.315259361379467,0.160325155037584,-1.96637
490420991,0.0492553201346741,0.115056934465781,-0.681694204530909
"H1f6",1.85466682411972,0.0616752104778594,1.56003175177468,0.03953458
66567768,0.968464178897641,0.98063536353065,0.00120603328503055
"Znrd1as",438.51702648289,0.239081456426644,0.277186089898054,0.862530
498967594,0.38839565732437,0.532667829113777,0.150587190825048
"Leap2",1.54393976182988,0.868760160823238,1.29553487638655,0.67058029
5951852,0.502487938372592,0.637386602247173,0.391271242641464

"Pdzrn4",39.1467546744502,1.48061889901343,0.522365315503419,2.8344510
1554361,0.00459044948635634,0.0185809807110185,5.9011795047581
"Ska1",43.0754936981417,-0.378632726349065,0.518914519136065,-0.729663
003030722,0.465596200959487,0.604407105613055,-0.190644338774135
"Kctd1",1021.54662225696,0.491180844798495,0.167657984065811,2.9296597
3279084,0.0033933335888767,0.014854786330471,2.0675929326301
"Agr3",0.0778527997966676,0,4.78471990596551,0,1,NA,NA
"Ube2r2",8533.03405242548,0.320497983199327,0.12766232580686,2.5105134
2809003,0.0120555736995433,0.039091896889023,1.03900320562242
"Armh4",421.481959959249,0.739534491462997,0.2789708256469,2.650938461
92735,0.00802684649335949,0.0285663086450813,2.62943505517727
"Gmip",1906.12443234959,-0.672191364656341,0.248757415883193,-2.702196
28335413,0.00688830844454841,0.0254709345821743,-2.46708837359949
"Rbm43",931.901845550971,-0.305475033153845,0.157424557939592,-1.94045
349183107,0.0523246011037192,0.12035213782238,-0.646792475757642
"Trpm8",0.231468339891574,-0.0623891562215573,4.78471990596593,-0.0130
392494122312,0.989596479014976,NA,NA
"Igfbp7",57546.2183722138,-0.135822259746413,0.316438869540436,-0.4292
21163454628,0.667762282900451,0.775093998808965,-0.0346035686184228
"Pnpla8",4291.28645408104,-0.06908497371823,0.36500830069122,-0.189269
596300695,0.849881523257508,0.904351117044336,-0.00694563677590739
"Fstl4",1.27173421292661,0.104023257794799,1.68488666838686,0.06173902
36070848,0.950770662884819,NA,NA
"Edc4",2405.25040045048,-0.0107615123768052,0.101425699744853,-0.10610
2421811009,0.915501089666898,0.948362433280434,-0.000570559633552743
"Lrrk2",2696.60354516324,1.20166243648002,0.318398137123788,3.77408752
241798,0.000160594377574319,0.00143504545093679,7.86675374667101
"9530068E07Rik",8884.10005252319,0.402815660507055,0.231733153891658,1
.73827375902967,0.0821625878372153,0.169704040005299,0.714473855181726
"Macrod1",1371.17392590716,0.513000343840037,0.446509693271198,1.14891
19980391,0.250592269774349,0.390321782717707,0.48262241090998
"Snapc4",1351.12421443234,0.177691768830063,0.200076660320497,0.888118
426934073,0.374477051586615,0.519572586055349,0.116343464466097
"Naa30",1590.70814915363,-0.154430766623363,0.215337729637826,-0.71715
610117697,0.473277782769068,0.611042922025645,-0.0760707538144782
"Noa1",909.92434962514,0.428184520406373,0.171332495958482,2.499143656
37989,0.0124493833022456,0.0400293660335066,1.37795856462898
"Ap5m1",1554.18298429644,0.677079415861857,0.115691685555342,5.8524466
3531135,4.84393695291499e-09,4.67559815385824e-07,9.86893254254788
"Gramd1c",369.790584456366,-0.111476167346232,0.123927708123546,-0.899
525772195344,0.368372674595943,0.513381736306378,-0.074325127669415
"Lrrn3",57.4400032421851,-0.87439863655495,0.374424324861753,-2.335314
71780793,0.0195269858703271,0.0564032827548108,-2.51409537067135
"Slc2a13",199.073450344206,0.07662821620262,0.231499081396691,0.331008
726861044,0.740637893712518,0.831038594211122,0.0141822768534652
"BC031181",3648.97598996044,0.0254449813281989,0.284938567153324,0.089
2998851731682,0.928843585325028,0.956158187353751,0.00114074715549799
"Zdhhc23",686.271418499608,0.698848576938173,0.280533253678633,2.49114
344832269,0.0127332698666774,0.0406805334852493,2.23771705250375
"Lzts1",63.8280741530207,-0.658876308822584,0.4183779193596,-1.5748352

8249079,0.115294470083283,0.219627594506204,-0.998739150953709
"Skp1a",13013.9165061234,0.436140323982556,0.232731794501446,1.8740040
4365397,0.0609298677766911,0.135124405092686,0.872960767662202
"Znrd1",1021.57711108203,-0.0751258794827274,0.169765426873273,-0.4425
27556207351,0.65810749429746,0.76718241266631,-0.0199106629045437
"Srp72",4778.26103138459,0.314892327656022,0.198485676582109,1.5864738
1049563,0.112631868506672,0.215768930255756,0.482902250918369
"Qsox2",919.099614548239,-0.0933506712834928,0.107541638655173,-0.8680
42113276858,0.385371278594421,0.529865582890687,-0.0592899912526369
"Slc18a1",3898.931530425,2.11509632221535,0.733525566329377,2.88346639
74965,0.00393324608051944,0.0165996606229368,8.66845371907592
"Kidins220",7927.56035521652,0.875012375300717,0.183545493047161,4.767
27791445081,1.86731649973592e-06,4.50605657324889e-05,8.75668903031267
"Igsf10",507.674526135276,-1.66275285007074,0.350955709438652,-4.73778
543945129,2.16066398482669e-06,5.08186084151396e-05,-16.4400497299497
"Tmem260",1349.85827157175,-0.0258467859229727,0.0871751757695678,-0.2
96492501389319,0.766853992703965,0.849455695360863,-0.0042171484872276
4
"Ubac1",1539.52019875379,0.292382666351164,0.195946361461071,1.4921566
5027418,0.135658083648592,0.248059768824931,0.407606452723909
"P2ry12",79.0285930667502,-1.06594112175206,0.427071208687986,-2.49593
299681045,0.0125626328441064,0.0403070948155975,-3.42297973722815
"Csgalnact1",539.895387673507,0.519253433203062,0.203175521960268,2.55
568893434222,0.0105977810657457,0.0354860956929263,1.73358695291323
"P2ry13",71.567772137677,-1.63776419210442,0.507799642488016,-3.225217
30042981,0.00125877085624228,0.00701844842865653,-8.12202164754844
"Rmdn2",216.978229963068,-0.390399992589524,0.234013656838451,-1.66827
867169749,0.0952604198311962,0.190325613881408,-0.647680972651839
"Serbp1",13905.7763075734,0.00805355440104437,0.0887843490363948,0.090
7091676455613,0.927723685938379,0.955560297328395,0.000366093738609546
"Tmem258",1408.91466187388,0.0483124820740763,0.281024554852747,0.1719
15518554567,0.863503946183872,0.913989465430385,0.00434504266306497
"Abt1",816.859732216187,0.00223438767738298,0.212934611505342,0.010493
3043134086,0.991627708141529,0.994535108962683,1.22441722819756e-05
"C530008M17Rik",33.0228081777164,-0.871321455500352,0.808612895228136,
-1.07755077941779,0.281234277898957,0.425209828665777,-0.7451301625534
69
"P2ry14",289.177657597335,-0.218342719435729,0.385248340739764,-0.5667
58364270853,0.570878361713458,0.69743021909079,-0.0786804134973194
"Gadd45a",3714.68798615072,0.0835685215042693,0.293051456194878,0.2851
66716416848,0.775516418567943,0.854826113926042,0.0131083248139437
"Sec24a",1668.90518277722,0.427939110350665,0.154090124088265,2.777200
11508028,0.00548294099493653,0.0212918509299487,1.64732201969011
"Glb1l2",4113.71327732864,0.743596751668629,0.291465262719971,2.551236
27676705,0.0107341527285777,0.0358520582398538,2.47495344665292
"Ppp1r11",2463.40496669411,0.0575530377923962,0.164243025650315,0.3504
13891637206,0.726028102471842,0.819664876286572,0.0114449803885961
"Gng12",7862.03413329784,0.223188885293324,0.269704954559044,0.8275297
93281806,0.407936842563071,0.551584861117042,0.13278836519593
"Cep135",433.335597571582,-0.192659748327181,0.13882511254321,-1.38778

744564111,0.16520177881256,0.287971420785887,-0.239840971891806
"9530077C05Rik",137.78310281299,0.300119228016295,0.307840450900987,0.97491810169166,0.329600886210539,0.474359571349341,0.22382581567898
"Arsi",113.421260075756,-1.81017398598934,0.327471437302983,-5.52773090959543,3.2439910778041e-08,1.94619501646229e-06,-23.8031260531785
"Pcdh8",131.660469214653,0.430884547408208,0.542907604888242,0.793660916753794,0.427392858742186,0.570422750342607,0.241888901123037
"Gpi1",19252.6539269298,0.21913622711154,0.304660495294304,0.719280085525539,0.471968363574408,0.609836921999589,0.108376822964611
"Tbcc",1010.81476789982,-0.0814498922251584,0.134489725239407,-0.605621671694014,0.544766008027,0.674788617274026,-0.0320387872512943
"Siah2",834.868946547648,0.764975366328668,0.175495868421858,4.35893661319602,1.30695958021311e-05,0.000208877851598322,6.48221836316728
"Exoc1",2138.76049681603,-0.0224509504677444,0.14368959604454,-0.156246179861103,0.875838977432934,0.922438739371649,-0.00181256205003552
"Npy1r",307.668159296475,-1.1127976577609,0.349669093518278,-3.18243069916254,0.0014604446140467,0.00787890555054906,-5.38990920523141
"Calm2",10646.1682161844,-0.365541087764568,0.229688548086664,-1.59146414050493,0.111505159422575,0.214102370413237,-0.56340884847134
"Thap11",1094.89246255552,-0.119624646678468,0.177364725026087,-0.674455682553981,0.500021652045851,0.635389870411235,-0.0542517509963844
"Lum",934.853377667539,-2.14274068927179,0.517920833333372,-4.13719733087579,3.51573743493855e-05,0.000441971943948626,-16.5510950955964
"Lcn8",1.28882851876916,-3.6619413469769,1.92896167854509,-1.89840025735446,0.0576433757526507,NA,NA
"Hif1an",1955.61432040298,-0.212508242511055,0.173165586939142,-1.22719673271884,0.219748657162836,0.355514380796629,-0.219773809696269
"Arhgap26",1324.60436665865,-0.433045127669235,0.150458872158603,-2.87816279263843,0.00399998664068851,0.0167796341480517,-1.77011066537111
"Wtip",2021.0610963721,0.103176881091771,0.273318292546702,0.377497166876018,0.705804169426978,0.804593644497616,0.0224325027198182
"Elf1",2058.14685509848,-0.528815451762175,0.167381989935211,-3.1593330439366,0.00158130673170444,0.00837189478915665,-2.52925822577578
"Megf11",55.6098359902529,-0.322599570859422,0.549645041248996,-0.586923462688495,0.557255111176961,0.686379430794964,-0.121402186094071
"March1",346.847729050946,-0.780969327511738,0.413229724008124,-1.88991566225373,0.058769240493972,0.131493541437804,-1.58442865290156
"Tbc1d24",833.636840515504,-0.0962515573397149,0.233389537628836,-0.41240733546842,0.6800408874467,0.784674945167225,-0.0233396291013748
"Btg1",9027.91858933475,-1.0899094182048,0.198446855657981,-5.49219797205171,3.96962045367754e-08,2.26182406888254e-06,-14.1681019630423
"Prss56",0.69248999686113,-2.20827163447719,2.93123731748519,-0.753358188129149,0.451234701152934,NA,NA
"Rnf39",55.2411460444069,-0.769510438849277,0.300573077717644,-2.56014425740468,0.0104628722069818,0.0351551063745875,-2.57630971246192
"Eea1",2935.07581046084,0.553387045594153,0.128439395114518,4.30854602749218,1.64331280808036e-05,0.000246471639476545,4.59768546086627
"Akp3",0.0886915477092957,-0.544048378937457,4.78471990596623,-0.113705376621746,0.90947135024012,NA,NA
"Fam13b",1117.3669825136,-0.455433871610224,0.191780401400935,-2.37476

753768023,0.0175600053852962,0.0520262855192988,-1.34626534696856
"Tmem255a",18.4150079911715,-2.14585720902858,0.6055980444357,-3.54336
878849749,0.000395049839805505,0.00286985163059901,-12.5607643304664
"Rnf13",4484.35534850773,0.175440568120474,0.201809774015978,0.8693363
29104572,0.384663197660223,0.529262123825969,0.111627826714201
"Phpt1",1435.74202910716,0.0958570571787665,0.229730713913535,0.417258
343674694,0.67648945419774,0.782058068181402,0.023564184277152
"Cdh8",0.53454226360782,3.23326752731758,2.45408559216297,1.3175039768
9588,0.187669748907101,NA,NA
"Commd2",995.919307695856,-0.0929419717333905,0.192854081070215,-0.481
928985985791,0.629856394163898,0.745700405210254,-0.0272720892631658
"Greb1",158.732321075729,-1.71965413562396,0.721530503961604,-2.383342
25120366,0.0171562356751901,0.051148667155177,-5.11256415122605
"Card11",466.095143977669,-1.42048769063509,0.543673579163866,-2.61275
836287594,0.00898147782430507,0.0311381320445057,-4.92812933804183
"Ppfibp2",1553.56616113985,-0.674445955396259,0.222838244598384,-3.026
61671299644,0.00247307326481416,0.0117323558436366,-2.99818528563645
"Sbf1",4089.30224903415,0.0491371341607814,0.171401701298622,0.2866782
17243439,0.774358717948225,0.854177771133429,0.00774479578309857
"Cdc42ep3",1226.57959824919,-0.535694865905596,0.184486186412122,-2.90
371261027052,0.00368766491895873,0.0158069750173322,-2.22168944920519
"Slc38a7",1086.51875037698,0.0677708313363575,0.165644738130296,0.4091
33619946617,0.682441604988801,0.786285990397594,0.0162944593157275
"Rnf113a1",130.256456704989,0.144541840002933,0.177599572636579,0.8138
63670149188,0.415723055135232,0.559018491657018,0.0840615919819296
"Adamts2",1909.40125947195,-1.45328103115577,0.380375164220082,-3.8206
5173507204,0.000133099488762735,0.00124230437141973,-9.72359420836828
"Cnot1",3544.75848956941,-0.295552323488269,0.187215523764602,-1.57867
42335528,0.11441079784403,0.218355983660476,-0.449720866868318
"Akap14",0.459239347958079,-0.0423581076203728,3.13525640273183,-0.013
5102531274524,0.98922070553667,NA,NA
"Ermard",1317.95574573213,0.418383094811925,0.18164857637092,2.3032555
6726413,0.0212644680231843,0.0603251269483554,1.17482247610784
"Sh3tc1",2878.17404257732,0.624442559165783,0.191071622275016,3.268107
27689851,0.00108269319162628,0.00625306630637711,3.16884824336979
"Iqce",1583.69200601736,0.140513151263429,0.147824477246767,0.95054049
1537593,0.341837689585748,0.486329899419402,0.101291445562659
"Stpg4",0.853618365523545,0.210268708557129,2.34062948280169,0.0898342
561700288,0.928418925747508,NA,NA
"Lgi4",259.489288960921,-1.40937216105084,0.244423532838304,-5.7661066
6200949,8.11237285095487e-09,6.81317024540035e-07,-20.0120109020045
"Ppp6r2",1624.37064754272,-0.0146791034574819,0.134145058461493,-0.109
427090537932,0.912863747621649,0.946353538447837,-0.000809391972988589
"Ndrg4",320.971959773677,-1.13427174786637,0.409661605782251,-2.768801
69353549,0.00562628686119884,0.0217015511809407,-4.3446822193824
"Ttyh3",2929.49076340234,-0.464749668396728,0.193749225540383,-2.39871
755409861,0.0164525998670858,0.0495203445829637,-1.39674549734469
"Bicral",1362.19680432698,-0.199955949097042,0.163242716818417,-1.2248
9966470885,0.220613026780571,0.356498657066764,-0.206239526031663
"Fxyd1",1638.33289294332,-1.90495656886522,0.394493305446231,-4.828869

19135531,1.37310566584402e-06,3.51349268669641e-05,-19.5378343550184
"Upf3b",3621.72922171849,0.552032344207954,0.225024770490186,2.4532070
0919026,0.0141588830872165,0.0443414555789509,1.72004183593227
"Tex44",0.137986538933697,0,4.78471990596107,0,1,NA,NA
"Fxyd7",47.7438350096643,-0.866496723093974,0.357190442090665,-2.42586
760726938,0.0152718403290141,0.046765981375982,-2.65373220045395
"Spg20",970.190742469448,-0.398884489716947,0.151570484234032,-2.63167
655452661,0.00849647082189206,0.0297903593032281,-1.40150875648234
"Fgf1",7012.45428071019,1.21134516517896,0.365644752112127,3.312901821
18487,0.000923333860261572,0.00553000344180884,6.2960474636119
"Grifin",0.649303034752547,-0.971279696681938,2.93816472895565,-0.3305
73601646622,0.740966588979732,NA,NA
"Fut7",34.0629543855653,-1.46383567649925,0.431112714757097,-3.3954824
9539339,0.000685077543618444,0.00439107230291664,-7.94596620865526
"Arhgap21",1620.83691719677,-0.204791192058678,0.197200748063934,-1.03
84909492954,0.299041546755275,0.443433879278603,-0.166537544249421
"H2-
Aa",36602.611824198,-1.1854686273764,0.415589656727052,-2.852497910348
58,0.00433770976234448,0.0178301974799365,-4.7737182968435
"Cpz",62.2237519859889,-1.82567260510965,0.459584705979022,-3.97243986
007008,7.11402004003356e-05,0.000760466900989991,-13.1112101611194
"Ccdc113",17.1553812174037,-1.18846033285575,0.586657031445981,-2.0258
179296453,0.0427834458767248,0.103203220054977,-2.69905904785094
"Chst12",401.698791672496,-2.02598387175803,0.33918066201984,-5.973170
34436214,2.32686711229761e-09,2.83557843472367e-07,-30.5434283476354
"Alx1",367.143998376392,-0.858781100369413,0.622126915801291,-1.380395
34789025,0.167464951772113,0.290992926624423,-1.06012775619373
"Plxnb2",13393.2070952792,0.628740570525471,0.259891416333507,2.419243
31090118,0.0155528332592725,0.0474123112709966,1.9169503711421
"Eepd1",479.174948631538,-1.82477826345029,0.442374628077006,-4.124961
39614192,3.70796945316277e-05,0.000460121546918555,-14.0216324364346
"Eipr1",786.143353802013,0.376319282700331,0.249035107216044,1.5111093
6488913,0.130760589104689,0.241094086653733,0.535339777114147
"Rfxap",649.165914918104,0.132383936739429,0.190311259228581,0.6956179
95887591,0.486668093124254,0.623582472217698,0.0625215243834786
"Etl4",2469.39182462974,-0.715587018484318,0.184148022976397,-3.885933
75545518,0.000101937303570503,0.00100534827770241,-4.93928304441817
"Mgat4b",5533.94251604204,0.376712884497432,0.145673457568006,2.586009
08351178,0.00970943717312864,0.0330334332580112,1.2846795018454
"Atp13a2",2419.45036275428,-0.495260377835723,0.145075409739098,-3.413
81340039909,0.000640604340936538,0.00417463350253363,-2.71339725016022
"Alg5",1582.90524905665,0.352655138739462,0.164050421218856,2.14967530
177197,0.0315809074055568,0.0817205749958257,0.883206976327046
"Mag",98.3505172089168,-0.998353795184169,0.478378194932478,-2.0869550
6141346,0.0368921969218461,0.0921265440038622,-2.38066664162202
"Clcn7",3379.53238945634,0.480491325202913,0.161917949824984,2.9674988
2099096,0.0030023343403189,0.0135476776134084,2.06685272237362
"Nudt1",716.272779389124,0.215012983877951,0.272417122311921,0.7892785
22778602,0.429949236541778,0.572697787545538,0.11984761857595
"Ccdc148",496.815788441764,1.17053047416753,0.349346505389419,3.350628

83443684,0.000806282934544722,0.00498759665537849,6.20474924545245
"Tbc1d9b",3371.84656568284,-0.336217264887336,0.113566331592049,-2.960
53645630722,0.00307103773010819,0.0137638945726424,-1.4409285009716
"Man1b1",3378.72567557405,-0.29398412052321,0.0809118937752296,-3.6333
8573362141,0.000279726355486821,0.00218513801253287,-1.80096915002058
"Colec11",74.9830331200053,-0.339340010205752,0.352193660594714,-0.963
504026826443,0.335294644317377,0.479895775117426,-0.249138798014621
"Olfr11",59.9339755991852,1.20666111972678,0.28038815284828,4.30353817
545107,1.68091868132893e-05,0.000250953694093043,10.0035128411802
"Dennd3",1985.90823959706,0.656299527066303,0.230842885203607,2.843057
20095049,0.00446830502618246,0.0182029068242648,2.62925008975399
"Tcaf1",2050.56241655147,-0.603392307690181,0.152579247310172,-3.95461
583621243,7.66577250764128e-05,0.000804019539289885,-4.29970539311235
"Cenpt",760.300070143485,-0.53090474960244,0.24710474276718,-2.1485008
4889975,0.0316739890267065,0.0819288190558135,-1.32827296564121
"Tmtc3",950.151314635691,-0.122955465405871,0.26147996653667,-0.470229
008495102,0.638191411103223,0.752370526252533,-0.0349840704360215
"Aaas",802.676939965489,0.277674778968065,0.244027575508084,1.13788279
209809,0.255169427600266,0.395932953951137,0.257268567828757
"Cc2d1a",1736.50417949826,0.417602802697234,0.210521329490416,1.983660
29564831,0.0472937225852175,0.111570841254273,0.915842845029678
"Tmem184a",1290.76962392763,0.180275331902816,0.343143615492651,0.5253
64085949829,0.599330114765043,0.72115206968933 8,0.0589329522592387
"Nop14",1154.50161254299,0.0375098128724043,0.108361944153915,0.346153
007545954,0.729227728618876,0.822272521346108,0.0073400478807934
"Ago2",3110.49724509718,-0.0400098575302051,0.16500645765622,-0.242474
495231957,0.808412517138708,0.877981551658296,-0.00520647065128126
"Zcchc12",26.796567067845,0.427843299695657,0.452477287080454,0.945557
516171156,0.344374321699084,0.488878353121086,0.306182458015043
"Cab39",4832.46457344413,-0.133959830264643,0.140701750611878,-0.95208
3607219419,0.341054581950408,0.485641001889218,-0.0967572573538067
"Cyld",2317.99287413278,-0.217395303750476,0.128427889667546,-1.692742
16303978,0.0905045559562723,0.182828204728077,-0.369399913001122
"Micall2",592.457923594065,-1.06730010452843,0.241408525801819,-4.4211
3674727719,9.81830107443917e-06,0.00016837047875938,-9.27413752019789
"Zscan12",390.725348920056,-0.0431626177066367,0.1579112202051 8,-0.273
334710798598,0.784595935489994,0.860795158124235,-0.00647002090635381
"Cysrt1",18.8040532569372,0.150472257983093,0.551592879721696,0.272795
867232756,0.785010136591602,0.861018238024753,0.0225166073526833
"Rbm42",3235.22160692664,0.157685655648383,0.132017731135329,1.1944278
5671526,0.232310645474367,0.369491213261853,0.156996304256171
"Oxsr1",2634.60937099531,-0.0705260451578035,0.137897477493168,-0.5114
38254273345,0.609044214017923,0.728938928544685,-0.0222978899720512
"Psma8",13.898129975506,-0.372816581459477,0.49262965051615,-0.7567887
58185506,0.449176428925788,0.58996578047794,-0.196731858856042
"Olfr701",0.515827883234203,1.00675794881321,2.42265072492316,0.415560
500924103,0.677731635090907,NA,NA
"Ttll7",219.12595425823,-1.09442576422169,0.254368810630961,-4.3025155
5411596,1.6886981188932e-05,0.000251922233528536,-9.06883883445335
"Cuedc2",2960.25541677987,0.0102898086398108,0.122527610974371,0.08397

95092549634,0.933072723454427,0.958336070475897,0.000437900898971302
"Pramel5",0.123716792206209,0,4.78471990596188,0,1,NA,NA
"Cox6b1",20313.3196223679,0.770332314368483,0.328919801155089,2.342006
50633758,0.0191803805531014,0.0556433655742257,2.22533015475967
"Tubb4b",8686.41349188599,-0.0678530796051953,0.241542748378635,-0.280
915407565168,0.778775282781283,0.856964866170182,-0.0104736899209094
"Kcnk9",0.305082769878068,1.37468936562649,3.88110971207208,0.35420007
8743087,0.72318894458264,NA,NA
"Dnajc12",5827.31384440638,2.39141928271244,0.657610723618649,3.636527
19887705,0.000276338476609966,0.0021664847675678,14.6705187427767
"Dner",5.1267237722637,-0.652187333422093,1.02687284990205,-0.63511985
2944115,0.525350249869977,0.65797014208407,-0.27300282996854
"Kif15",140.71241192193,-0.87948493573833,0.476079279985835,-1.8473497
4343873,0.0646964679079208,0.141284954481049,-1.72113132866862
"Wdr44",393.276238274475,-0.509521649480864,0.161773830612109,-3.14959
25364008,0.00163498320475284,0.00860661515288093,-2.42288966151916
"Stpg3",18.2372910597766,1.76146834702077,0.642110245079051,2.74324909
237963,0.00608345191230394,0.023094849179537,6.63746931684531
"Decr2",2163.96433633799,0.613444527762321,0.215324536845111,2.8489299
7681722,0.00438665339577442,0.01797222921442,2.46538910313298
"Anln",197.82950610002,-0.921308963894584,0.323918633275323,-2.8442604
6929969,0.00445146416667185,0.0181616757316944,-3.69301320873702
"Tent4b",2374.14727615626,0.20077784308226,0.092080024005949,2.1804712
2869221,0.0292225492752065,0.0770712490629429,0.514598625736571
"Rps27l",6112.88497102702,0.970116267206104,0.230577475665715,4.207333
19421257,2.584020081617e-05,0.000349156088367071,7.72211723237817
"Klhl13",437.373882331034,-1.33027661480973,0.193100726253549,-6.88902
957859942,5.61742596977952e-12,2.60782313235145e-09,-26.2925847392311
"Slitrk2",11.4419751626107,-1.28609205672488,0.570912364734783,-2.2526
9609867766,0.0242783171079549,0.0668519456250396,-3.47923252351808
"Mbd5",743.907200867357,0.457948669168483,0.197622809885933,2.31728649
862235,0.0204881302839135,0.0585143641827662,1.29987951839761
"Fam135b",25.2727396959824,1.28236363232514,0.511160408692586,2.508730
35258167,0.0121165937983792,0.039250597421631,4.15202236766832
"Noxa1",2.36284582984023,-1.63458610871876,1.50702505120857,-1.0846442
8471703,0.278079233138319,0.421705322778381,-1.41138091734736
"Cnep1r1",1701.85699133302,0.0180946531591309,0.0880587395341122,0.205
483899211632,0.837194076275816,0.896212259757652,0.00198277584980319
"Entpd8",423.490668545347,1.63606501981512,0.599836340805483,2.7275190
0563102,0.00638125814235492,0.0239779863672454,6.10353541871656
"Slc6a20a",709.391251977318,1.41013428024887,0.583929956500043,2.41490
313102092,0.0157393949645003,0.0478463865010645,4.28646933011344
"Dpp10",248.519802697418,0.229260624126931,0.39126627216918,0.58594527
6744428,0.5579122897387,0.686782595104151,0.086141811991406
"Atoh7",21.0690253871444,1.77477086430769,0.408639496210976,4.34312121
261866,1.40472577577341e-05,0.000221596627637783,14.9340788859996
"Sun1",2426.50976214834,-0.447125868985015,0.15829299653247,-2.8246724
6675249,0.00473289692855124,0.0190389982077351,-1.77118444267991
"Jmjd4",461.225978869927,0.193195352155794,0.133865735489102,1.4432024
1060881,0.14896346067364,0.26658922039602,0.25541320159179

"Amdhd2",2735.1553556102,1.27600704012098,0.320836155159407,3.97712982
031901,6.97520887020468e-05,0.000749325744084026,9.18257637352667
"Topors",1774.74831427302,0.341364727538097,0.127198092954109,2.683725
20066992,0.00728069224095984,0.0265686007678965,1.23847982799412
"Ssx2ip",1359.44382971559,0.546640413474418,0.179078387045977,3.052520
30963442,0.002269283741609,0.0110175533849334,2.46440012560544
"Igflr1",238.278663231967,0.548462656321,0.696360924236085,0.787612626
200514,0.430923326963191,0.57330895304314,0.305126516167667
"Lpar3",506.798680272484,1.55340802238902,0.548201537130523,2.83364404
72606,0.0046020561629179,0.0186163674407828,6.18833346557252
"Pnpla7",1117.98724605594,0.22177126876747,0.2088930222288,1.061649960
35418,0.28839462394647,0.432447771876357,0.185909461566958
"Plch1",169.590391410823,0.334055314271521,0.392204251252532,0.8517381
26766071,0.394359454281196,0.538718941764175,0.206633685433487
"Psenen",3959.45149384752,0.426679749522424,0.133567802255104,3.194480
57330089,0.00140082712784264,0.00762516117774309,2.08061923857621
"Siah1a",1003.02506525712,-0.0981926999311445,0.144046446153622,-0.681
673880565053,0.495445186939161,0.631384983982991,-0.045152880590621
"Lin37",700.474865855439,0.164122825643251,0.217468391128998,0.7546973
8287573,0.450430577188925,0.591055615723108,0.0863031937669057
"Mrpl41",1224.61939751903,0.308750825826423,0.255183766901574,1.209915
62110419,0.22631127243685,0.362469950476403,0.313324568972654
"Mcoln3",434.405655176743,1.68265815681387,0.551297050927762,3.0521805
8754745,0.00227185376486773,0.011024540743502,7.5848029356754
"Hspb6",326.370338208753,-0.506772216882406,0.431522599308552,-1.17438
163770433,0.240242196824435,0.378616420439931,-0.492193226346972
"Gjd4",0.456791483589828,0.408162662505849,2.67472826204411,0.15259967
4627851,0.878713981603504,NA,NA
"Wnt4",2303.22989030309,-1.12979450272629,0.524730084430278,-2.1530964
8950841,0.0313110961011635,0.0812199226058974,-2.83645610150133
"Ptcra",9.66529598302485,0.778083122905326,0.556975878541745,1.3969781
329534,0.162420146945634,0.284534413759746,0.977973510104013
"Mrpl55",1240.11986746201,0.216115851626083,0.253415543592459,0.852812
138365273,0.393763491475587,0.538212447023555,0.133884183689779
"Dchs1",637.088174277296,-0.847550855929841,0.245348801471317,-3.45447
318612202,0.000551369041057831,0.00373025453245857,-4.73889313979296
"Syde2",634.927675564232,0.36313241602498,0.177834945572498,2.04196320
838944,0.0411551809903732,0.1000374783652,0.836007217325917
"Proser3",526.933315275199,-0.0578573101190709,0.257380139756685,-0.22
4793218986385,0.822140141551233,0.885933927266825,-0.00700726692418672
"Smad6",697.832574548263,-0.760177149212788,0.209059972298959,-3.63616
784625669,0.000276724059610301,0.00216776444928953,-4.66297136981745
"Abcc12",2.2333942924898,-1.82365364715614,1.31055860078596,-1.3915086
6360533,0.164071234339589,0.286672012693437,-2.27850301110607
"2410004B18Rik",941.144696149948,0.0595211449573112,0.140719733009055,
0.422976534168673,0.67231238186487,0.778475373929642,0.014905161371323
"Dna2",607.652528673834,0.184912476460342,0.304411545473872,0.60744238
9126509,0.543557364564679,0.673933708144608,0.0729708139754188
"Phkb",2834.20336344878,0.132845866041164,0.23095278016043,0.575207910
244178,0.565150693640297,0.692695283132211,0.0487763634805733

"Acaa2",8268.60527955829,0.040528161424967,0.51702281408107,0.07838756
8829046,0.937519761740715,0.961159922940607,0.00160550166764779
"Arhgap33",368.750078673194,-0.378007254245947,0.275419171444289,-1.37
247981781257,0.169914117470782,0.294412485406923,-0.462217247008315
"Arhgef26",218.103713278279,-0.0175429346818881,0.231238459210733,-0.0
758651252986459,0.939526402848817,0.962511041050371,-0.000670311002237
881
"C1qa",7472.26699434531,-1.29097005521701,0.411008938139848,-3.1409780
5526981,0.00168384648829595,0.00881283135234201,-6.10828486093526
"Gtdc1",474.874767513097,-0.0545741478789663,0.177564451934929,-0.3073
48386933696,0.758578209310799,0.843396138936481,-0.009294987859852
"Prodh2",2443.36925703796,2.47845497996629,1.00605252333372,2.46354432
048291,0.0137570885993865,0.0433296469393389,7.7796674232912
"Ehmt1",2122.91149736494,-0.203483159967256,0.175444274238715,-1.15981
647648637,0.246123534329235,0.385238554415303,-0.194101063164904
"Rap2b",867.655413649055,-1.09405934710992,0.172812925903743,-6.330888
39499954,2.43753614599355e-10,5.05607231644491e-08,-18.3802963546571
"C1qc",4831.59835444531,-1.43858282295657,0.402452854311135,-3.5745375
080491,0.000350847516725542,0.0026100056776477,-8.55727021051071
"Zfp157",954.683481441884,0.414549027101874,0.228765385019676,1.812114
30683107,0.0699685361997181,0.15003260957023,0.786359132053898
"Trpv5",448.326204626088,1.43819778722484,0.673330561159002,2.13594610
164325,0.0326838072839509,0.083994843076641,3.56241573769717
"Neto2",51.9781243402913,-1.31161587748136,0.370221152765725,-3.542790
21520779,0.000395917519939101,0.00287367809100517,-7.67578925903274
"Fzd8",811.115706510688,-0.0940468641184631,0.145947157685708,-0.64438
9829920425,0.519322677729112,0.652684902047327,-0.0401261107659732
"C1qb",6374.81889905472,-1.40370504402652,0.40022257704832,-3.50731099
274552,0.000452659791220196,0.00319897086234555,-8.06418178121493
"C1ql2",1.18035068235469,-2.18448575237375,3.35155470673692,-0.6517828
12311772,0.514541291017338,NA,NA
"Unc93b1",6024.11740504607,-1.05727410794203,0.286951644091679,-3.6845
02701801,0.000229149595257937,0.00188441957331896,-6.63348093368894
"Piwil4",14.7535155907715,-1.69382982529282,0.495614646321424,-3.41763
472460882,0.000631678219281837,0.00412947725666933,-9.29845575029004
"Trim67",66.6851519907196,-3.12110753694377,0.885214125250273,-3.52582
211231813,0.000422170434866302,0.00302072628954978,-18.1094711229099
"Kirrel2",18.0949553629331,-0.725543215423498,0.697472654555285,-1.040
24610955696,0.298225574269047,0.442594173169271,-0.591391737917485
"Zfp280c",617.99181213712,-0.00127384678918021,0.156663829980745,-0.00
813108417773758,0.993512404959456,0.99595846332689,-5.15873014906294e-
06
"Ttc7",2164.47224136672,0.416044754321598,0.192421011381073,2.16215865
06354,0.0306059495218572,0.0798547843941084,1.05157204194312
"Stox1",46.0932140235481,0.529538942811961,0.371623544941222,1.4249337
8048938,0.154176343747258,0.273832241791222,0.685879817122667
"Mucl2",3.20100735668892,0.276465168337857,1.56336563146664,0.17683973
7789615,0.859634274681831,0.911029575198301,0.0257610016344488
"Stag3",58.6517244176982,-0.0519357838351611,0.560440624147538,-0.0926
695560553956,0.92616608365517,0.954712466249458,-0.00240696734503401

"Nfkbid",340.533863534364,-0.852081779354867,0.389902903986488,-2.1853
6915381476,0.0288617906594685,0.0763771979205098,-2.19161490579203
"Aifm1",4612.95346171914,1.00664761398956,0.287785638180163,3.49790774
951515,0.000468923300299444,0.00327943562024339,5.75810884840055
"4921524J17Rik",616.393222479499,0.115839854720038,0.17702067452734,0.
654386020329775,0.512863138669006,0.646943486948844,0.0504478321298415
"Try5",0.552698706968563,3.12945375516367,4.27488740797788,0.732055246
489866,0.464134848107489,NA,NA
"Kdm1a",2092.43527560608,-0.184327398861236,0.107879906977392,-1.70863
5129801,0.0875185571598176,0.178143525159424,-0.317995312071089
"Elac1",890.510574367175,-0.514812678775415,0.183684482362484,-2.80270
098025744,0.00506766303634972,0.0201036203220238,-2.0112986758442
"Rab8b",1692.5347023949,-1.00114016432287,0.220515133501374,-4.5400066
1281884,5.62524639521069e-06,0.000108380488353575,-9.14027202537288
"Tmem71",179.990509409955,-0.58588250938285,0.232573975392106,-2.51912
325269878,0.0117647466740063,0.0384622787809818,-1.90885049837059
"Map11",3423.34285846838,-0.0656574677623897,0.248699696261738,-0.2640
03007439503,0.791777601343213,0.865994259886126,-0.00944659940709108
"Slc39a12",4.75716433419747,-2.1761652090293,0.90784788821366,-2.39705
928413984,0.0165272494473078,0.0497066650815376,-6.53201285106609
"Aadacl2fm1",0.268837707829504,-0.550629761653143,4.78471990596552,-0.
115080876723134,0.908381017352687,NA,NA
"Kif1bp",1769.41460513652,-0.00171456199471287,0.136939393262121,-0.01
25205899768444,0.990010275572828,0.993321687496475,-1.14887865036793e-
05
"Lrfn3",320.039509148101,-0.595584379448902,0.20182789286877,-2.950951
77868281,0.00316796371429983,0.0140702147090533,-2.53939022882824
"Cst11",0.125558793514292,-1.03498639904526,4.78089210794988,-0.216483
948115925,0.828610536062342,NA,NA
"Bcorl1",683.341087441146,-0.110282013918996,0.194055631726549,-0.5683
01022432568,0.56983058488716,0.696803235571382,-0.0398396212495572
"Clca2",30.3035319155419,-1.33773172273407,0.643817400413456,-2.077812
31429127,0.0377266491833556,0.0937659918135204,-3.16634818031677
"Wnt8b",10.0718141042005,0.706232903552985,0.723011381612463,0.9767936
18349329,0.32867133605835,0.473387990750906,0.528149179496167
"Cfap221",5.51063143457245,-3.34586194510308,1.45964581797334,-2.29224
233982232,0.0218916630080441,0.0618076520896096,-9.31396992187323
"Trim17",27.3876715557573,0.371701252282173,0.400125281347059,0.928962
176623297,0.352908688359747,0.497902576384829,0.259206184539058
"Spryd3",1215.24848027178,-0.404381633364735,0.135150655442977,-2.9920
8044562797,0.00277083237649512,0.0127497809488113,-1.76401021673868
"Cnpy4",1108.92584356059,-0.595281261740212,0.135394428350273,-4.39664
518690669,1.09936834324796e-05,0.000184470602036564,-5.11824045940506
"Zic4",0.222382911859068,0.183244500456612,4.78471990596545,0.03829785
31780194,0.969450202447996,NA,NA
"Tmem177",582.802582435601,0.0979137221355963,0.28264265649945,0.34642
2310589155,0.729025360507309,0.822165261160119,0.0191728635349823
"Anapc10",417.35888923831,0.240206854253756,0.220153421571031,1.091088
44432043,0.27523396707161,0.418604671603919,0.209178928691796
"Taf6",1218.98232908953,-0.152644803660336,0.165182231643482,-0.924099

415182825,0.355434557769604,0.500488409575485,-0.105656281694365
"Tfb1m",366.752388157548,0.183163776683988,0.114426971718538,1.6007045
7107372,0.109442368257147,0.211063092693518,0.284929236314089
"Zdhhc9",2162.01602315195,0.462038790279113,0.211228190451155,2.187391
69848617,0.0287139416800187,0.0761278324574352,1.18990759990676
"Pml",2267.93180696361,-0.369038694720532,0.20090527929671,-1.83687903
081686,0.0662277555211875,0.143782293414109,-0.715733932331569
"Trim3",2117.25755986857,-0.204758632309055,0.177150297436748,-1.15584
695748063,0.247743761970457,0.387060076347524,-0.194351848936404
"Otud4",3043.22742638093,-0.253115349479249,0.0841536665977043,-3.0077
7565271474,0.00263167336217282,0.0122845025653928,-1.11355991198956
"Nxt1",516.921818447219,-0.0659734105070594,0.173987440174855,-0.37918
490231684,0.704550562132398,0.803759675923176,-0.0144122190851731
"Asap3",823.700718818969,-0.113983138164814,0.195105114554771,-0.58421
3993697313,0.559076347830142,0.687686419683884,-0.0426778322058582
"Zfp39",238.554480988487,-0.200767263031412,0.224794477508862,-0.89311
4747552006,0.371795726132622,0.516992801892302,-0.132451449103772
"Tns2",5936.55728065416,0.338218793612441,0.165572244808188,2.04272638
813504,0.0410795310866321,0.0999711304452261,0.778875208699469
"Xpnpep2",281.55447967436,1.49223056474081,0.262569810735727,5.6831764
4956798,1.3221573235605e-08,9.87717375279031e-07,20.6343691214824
"Zfp113",1005.83497752303,0.488880620737275,0.16211269681267,3.0156837
2095001,0.00256400554881481,0.0120610326153899,2.15976481316267
"Apln",390.959399755703,-0.456631072716411,0.389905277369194,-1.171133
34755927,0.241545181442164,0.380086187374652,-0.441725375615961
"Hk1",4099.26563130662,0.137913585567172,0.184118225037695,0.749049071
806645,0.453827629095458,0.594153703894665,0.0718001892182543
"Ss18",3046.66513985916,-0.381306145604451,0.0738800207203966,-5.16115
374476582,2.45432505557187e-07,9.08745933807127e-06,-4.42643631375347
"Sstr4",3.51111046649254,-4.07660561536512,1.4053546075665,-2.90076653
494888,0.00372251108881658,0.0159100221853892,-16.8804331637822
"Frem2",367.880354838496,0.780977918318037,0.368276206123182,2.1206309
4311559,0.0339528720391827,0.0864811363840606,1.9117003697274
"Zscan21",1406.67558048884,0.0381542666433758,0.0945296020744447,0.403
622418862276,0.686490397233744,0.789593544435084,0.00901344821609273
"Wdr62",188.694813507205,-0.52286327953499,0.308877020693823,-1.692787
88807434,0.0904958489512062,0.182828204728077,-0.888453644764088
"Mmaa",1843.84404662333,0.57378390102005,0.302207704343893,1.898640877
6896,0.0576117099030843,0.129655709976868,1.17216748229581
"Foxa2",3.60866268131917,0.691898425352165,0.91216000760395,0.75852747
2794641,0.448135272759983,0.588836279282576,0.366434317048239
"Zfp146",986.340867193572,-0.934183552676518,0.168782879750786,-5.5348
2411282396,3.115408283037e-08,1.89893484184692e-06,-12.3071372504742
"Tspan15",2050.46124731106,-0.840555098184102,0.208200387308731,-4.037
24079983425,5.40835479605045e-05,0.000613807344664678,-6.2166021494622
9
"Apbb1",722.475191648278,0.193842816389259,0.229210002688946,0.8456996
38389329,0.397720324934756,0.542209017230218,0.118651907308415
"Clca3b",43.8382449965782,-0.405583271846792,0.695238822245659,-0.5833
72589201415,0.559642506196811,0.688155007378132,-0.151583164526009

"Pax1",4.43250565718496,-1.02892942723945,0.996453854282777,-1.0325911
4590916,0.301795273426978,0.446600200461385,-0.829411255844022
"Inhbb",585.309048516618,-1.58540166903505,0.31150772599285,-5.0894457
4001172,3.59111593188645e-07,1.23149697732448e-05,-17.9224822905897
"Smpd1",2964.98649439092,0.484051983765456,0.185390095792116,2.6109916
0501131,0.00902801099328488,0.0312494511533941,1.67760484048126
"Azgp1",1510.69867844078,2.03220505276442,0.718376625649962,2.82888526
742605,0.0046710441276144,0.0188407154324008,8.07138000928667
"Paip2",5925.6530089471,0.279399714466724,0.139888844682657,1.99729803
402517,0.0457928168058722,0.108595030659316,0.620303584905285
"Cavin3",1651.77824464317,-1.14934722241857,0.164672690032148,-6.97958
612441557,2.96050900415518e-12,1.56010823143291e-09,-23.3070498776831
"Sh3glb1",8696.98127073707,-1.13692101304202,0.42635803547778,-2.66658
751199089,0.00766256541675163,0.0275586731090608,-4.08318130596542
"Rbmxl1",1306.17007240697,-0.631630680557432,0.129502471808935,-4.8773
6389687857,1.07512982353297e-06,2.94422490157946e-05,-6.58985334101216
"Scd1",232890.366069334,-3.94761829629614,1.22334694327979,-3.22690003
680606,0.00125139170198079,0.00698529499147478,-19.5957721638932
"Selenof",11451.537528026,-0.001470818017135,0.287440085668025,-0.0051
1695511680893,0.995917278330458,0.997247393347871,-4.05416578323263e-0
6
"Rnf139",1534.88269454691,0.0752330350588589,0.150670716987156,0.49932
0880415484,0.617553347528131,0.736185813774976,0.0230418268643018
"Trmt12",433.255534767191,-0.113999098424969,0.1930212087713,-0.590604
00227853,0.554785782602724,0.684373857344547,-0.0432342647366173
"Prr32",3.2472465166765,-2.99276846414269,2.05579864866917,-1.45576925
351131,0.145456418020946,0.261827428166638,-4.01051822825772
"Slc35b2",2249.39898392312,0.181762498259806,0.138046927844173,1.31667
180935008,0.187948656970921,0.3168444504253,0.208907694636485
"Lrg1",785.381294621374,-2.08051890369154,0.428519973355118,-4.8551270
2570669,1.20309597266968e-06,3.17858608063866e-05,-21.5468711316037
"Gm9762",1.80484953226189,-2.86569345512935,2.33981907117777,-1.224750
02038807,0.220669421300194,0.35650929594402,-2.9556617976685
"Rab11fip3",12523.8435947626,1.3714981369542,0.442970788174948,3.09613
675114969,0.00196059918262165,0.00983983600070963,6.33812660610063
"Ttc29",4.48367269189266,2.46383778928379,1.10549029640096,2.228728553
57037,0.0258319712142505,0.0702569081790286,6.5429593511176
"Dcaf15",1350.43987943428,0.334385930441802,0.166493589345245,2.008401
23488725,0.0446006717892257,0.10651934083125,0.748833451604636
"Socs5",1387.80380638596,-0.305701947432798,0.0933712831428129,-3.2740
4676409151,0.00106019064366148,0.00616287575524763,-1.5557819480667
"Fer1l6",31.3573679166854,-2.09371243665582,0.855089151008099,-2.44853
116682332,0.0143440019603228,0.0447916960638011,-6.50251078626471
"Zcwpw1",329.121702621531,0.0110914258531478,0.186131401249107,0.05958
92244871873,0.952482800732,0.970298309753006,0.000334425554927112
"Ralgapa2",2139.34717403022,-0.465996398738949,0.348385034766144,-1.33
759017247039,0.181030069786974,0.308813632286264,-0.547553837619256
"Setd7",2774.32344694336,-0.83419280765835,0.142873711220829,-5.838672
49286332,5.26183875482198e-09,4.9562962338898e-07,-12.1103414611273
"Sik2",1361.56061526224,-1.42850730440538,0.547555103417482,-2.6088831

8908831,0.00908382446744876,0.0313805086412365,-4.94487563370218
"Fam91a1",3730.9339265139,-0.102518820823028,0.13987232958573,-0.73294
5687875978,0.463591555827447,0.602567039232289,-0.0519315598230594
"Trim58",0.349667120864793,-1.23983330955007,3.89913701178303,-0.31797
6338303411,0.750502885434933,NA,NA
"Psd",205.202334553902,-0.616468375432668,0.280136185422492,-2.2006024
4806622,0.0277641801429639,0.0741030266320207,-1.60423497667741
"Tmprss13",356.776763799099,-3.0059179302069,0.677014763143204,-4.4399
5920598719,8.99759316030137e-06,0.000157623604168514,-26.3177151153417
"Prmt9",894.816117784007,0.305705278529246,0.0953765806758859,3.205244
68756236,0.00134947707573982,0.00740977302809772,1.49947077832231
"Aff3",175.066773495242,-1.57303506680959,0.31812184032685,-4.94475659
135315,7.62390630475226e-07,2.22198692272137e-05,-16.8543214399788
"Myom3",112.660470068361,0.777408843046581,0.515225420522912,1.5088712
8639261,0.131331678916208,0.24184974268117,1.10348415375432
"Tas2r108",2.14734938374968,2.81524492882498,1.10121654924432,2.556486
21586452,0.0105735260169854,0.0354292164081769,9.40353312845356
"Cfap61",13.294798913348,0.135375204345916,0.588658159185555,0.2299725
26896112,0.818113117955809,0.883303038590307,0.0167982955622439
"2210407C18Rik",0.0880002295447563,1.38055633636101,4.78471990596623,0
.288534410267056,0.772937694360212,NA,NA
"Arhgap10",1840.91394676338,0.653582890244733,0.160831677362411,4.0637
6965634686,4.82864791117684e-05,0.000563912898686245,4.8891991969677
"Ddx1",4192.66085272783,0.0451155690480488,0.152690767873714,0.2954701
82489111,0.767634727004482,0.849837719005984,0.00734074821875552
"Lrrc20",363.859801333443,-0.67926778515549,0.164431183640684,-4.13101
560248954,3.61164064939892e-05,0.000451747998841715,-5.23198271822934
"Ndufc1",2870.7546777024,1.00663689762767,0.339620176784485,2.96400793
132634,0.00303660460168584,0.0136580660954257,4.32192003139315
"Il22ra1",19.3744683177794,0.43168711120389,0.599965664025232,0.719519
694356601,0.471820772082374,0.609686620323555,0.213603181945928
"Wee2",0.354476896458541,-0.0630224514913308,4.78471990595938,-0.01317
16072685543,0.989490881793662,NA,NA
"Mgarp",15.8957155774964,-1.30006041155476,0.669949368284904,-1.940535
31968089,0.0523146660724161,0.12035213782238,-2.75266126839413
"Ppp1r14a",433.910447138216,-0.840587093262323,0.220600746309319,-3.81
044537394122,0.000138716661999894,0.0012830633186309,-5.59705323107624
"Spaca5",0.087583724926668,0,4.78471990595887,0,1,NA,NA
"Mycn",267.168911533131,-0.438548007599796,0.435410010343056,-1.007206
99382696,0.313835312861776,0.45899054235077,-0.341508592814489
"Nodal",2.39324739529101,-0.189668073737718,0.927702182954448,-0.20444
9312745696,0.83800239789744,0.89643774368817,-0.0207357337825725
"E330009J07Rik",909.124926487904,-0.0747232761008584,0.321782566937697
,-0.232216669821415,0.816369730961062,0.882692059184005,-0.00932388692
965917
"Elf2",1922.22342601603,-0.567952276491293,0.125697920436199,-4.518390
3959618,6.23115148097517e-06,0.00011761373821496,-5.13889166670789
"Krt80",518.6125497078,-0.394578216223742,0.387178157420974,-1.0191128
002986,0.30814941792685,0.453011939285058,-0.312441551219701
"Grhl3",311.460222437361,-2.4315357168778,0.664880225280502,-3.6571033

7655475,0.00025508152465366,0.00203751723379642,-15.0658518461168
"Cyb561d2",840.688008815962,0.825263934242101,0.236098265721172,3.4954
2565135452,0.000473306294228259,0.00330653032062436,4.71378865608365
"Pacrg",109.175787767788,1.24050340555438,0.266315264873524,4.65802591
580135,3.19256029441022e-06,6.97853594399221e-05,11.8717246718323
"Rbm17",2912.87532776215,0.162834182316026,0.113738289707394,1.4316566
8074434,0.152242099616554,0.271113020214045,0.212534349420143
"Prf1",34.7143504089971,-1.35799589319013,0.474518393298987,-2.8618403
6776522,0.0042118898575782,0.017439674759622,-5.4985355697765
"Atg101",1570.51767790057,-0.321875301670351,0.357685721237675,-0.8998
83004992729,0.368182515262902,0.513249637706161,-0.21468855022265
"Islr",1138.84384285234,-1.47411925459666,0.252142109980347,-5.8463826
3997854,5.02377711226914e-09,4.77822468951335e-07,-21.4543708095241
"Fam193a",1621.86248235548,0.0752014262880559,0.198493439139165,0.3788
61017342401,0.704791073625596,0.803955792052411,0.0164097781553281
"Spry1",1823.31862476479,-0.950237965554518,0.255047482508001,-3.72572
964143924,0.000194751057457327,0.00165819044467378,-6.0834504087593
"Thap1",461.143411981871,-0.22700953946238,0.12502236874867,-1.8157513
8700766,0.0694085174792185,0.149159845013755,-0.431939396226028
"Lipt1",272.894593002002,-0.490564269894378,0.407040578595413,-1.20519
745620248,0.228127084176684,0.364591957973523,-0.494967807358587
"Syn1",20.3666855032088,-1.96426245357417,0.435273384200732,-4.5127097
7016212,6.40045793374389e-06,0.000119881007485243,-17.7353471262465
"Mospd3",1576.7738486355,0.0571467773291666,0.155221052937525,0.368163
829890832,0.71275107865153,0.809483056168643,0.0120785107006205
"Zfyve28",54.6187319903768,-1.12451860595373,0.385439221924615,-2.9174
9915937113,0.00352850599855055,0.015314237294573,-4.69933215137874
"Fgf2",475.65453780477,-2.29083798766487,0.469399630189767,-4.88035746
159139,1.05893726405709e-06,2.92399786079693e-05,-23.9162882383718
"Hook3",4127.33315608729,0.351306856789028,0.228483301195525,1.5375603
1600925,0.124156156734423,0.232478319793315,0.512542056843954
"Mxd4",10559.0534487334,0.0262625121307327,0.174633667790255,0.1503863
05590715,0.880459844955364,0.925356083276179,0.00203735799145248
"Matr3",6745.8682136514,-0.216033967831714,0.0723107224945072,-2.98757
861046298,0.00281196968482833,0.0128794420753542,-0.94020637347447
"Spred3",518.955929375754,-0.310979673731373,0.252653320122461,-1.2308
5528256918,0.21837699491839,0.353794320475178,-0.323120180349452
"Clic4",10622.9389147506,-0.637382200880704,0.147136911514052,-4.33189
873514393,1.47828934277642e-05,0.0002287594095671,-5.34307283032241
"Zfp692",1538.41584646838,0.217470746971785,0.199494751947223,1.090107
60859171,0.275665745094357,0.419081920237683,0.18913188699076
"H2-
M10.5",0.664070677813134,1.17862912433647,2.91259072137706,0.404666922
711069,0.685722359075084,NA,NA
"Pomk",658.987852960096,-0.407698065805488,0.0906171848730445,-4.49912
526389643,6.82336446477094e-06,0.00012551128333406,-3.66239856816648
"Mex3c",1512.45595052636,-0.477225906604725,0.101812828413996,-4.68728
660266865,2.76851134045946e-06,6.19752400875759e-05,-4.62373472251473
"Itih2",259.61009922253,1.06198611309962,0.453987576338252,2.339240473
63878,0.0193229914966426,0.0559789708059203,3.06147145639425

"Aagab",2125.0191997953,0.117657276115149,0.111271518683284,1.05738896
626405,0.290334105081381,0.434120734729813,0.0981770659879482
"Dzank1",266.227612027481,0.212987931782633,0.205368985280017,1.037098
81748808,0.299689806248793,0.444091187280663,0.17288770578904
"Hgsnat",1954.59395270693,0.140332905051949,0.125661278922295,1.116755
34624096,0.264098946236888,0.405991785141937,0.126499217506921
"Kin",1272.65880637517,0.260898532151184,0.195893912957137,1.331835829
97942,0.18291414126191,0.311073696146945,0.304657850583121
"Aldh3b3",161.796071695894,1.60893475428001,0.540544927998805,2.976505
13572771,0.00291554198999164,0.013238760531173,6.95800945240132
"Rsrp1",28507.0120235277,0.0235783186190471,0.117600790215483,0.200494
559397466,0.841093812587645,0.898266262684664,0.00252968829163
"4932438A13Rik",2887.50143763858,0.0136007748422583,0.116261166224216,
0.116984675829145,0.906872197010695,0.943050351856775,0.00079748962570
9417
"Gemin5",804.824187519563,-0.0450795385749999,0.148653403510202,-0.303
252650195165,0.761697327789675,0.845664505366508,-0.00755679862577664
"Tmem97",896.233342326001,0.325281765045726,0.267979073118582,1.213832
71186175,0.224811612049777,0.360950000967273,0.331467268350499
"Ovol2",164.941154000648,0.553270669583687,0.259458030344685,2.1324091
1776243,0.0329732284928582,0.0845714297755524,1.3666664015886
"Galnt6",373.903541540375,-1.98734127270728,0.404051328889394,-4.91853
665763169,8.7193589818261e-07,2.48393874403066e-05,-21.0719384251379
"Stag1",1994.27262922371,0.0923179891512167,0.11863689581829,0.7781558
04856994,0.436477167482265,0.578543018940467,0.0505203153806138
"Tbcel",4308.60384694745,0.982727028022599,0.206471426927376,4.7596272
4066543,1.93950823692302e-06,4.64576555325861e-05,9.80463738292364
"Ldlrap1",1380.25229214348,-0.667201598910085,0.146528107506179,-4.553
40350916597,5.27848881379411e-06,0.000103541222224706,-6.1219354540524
"Lsm1",743.522492909049,0.0359026370086075,0.154320754952176,0.2326494
38630168,0.816033633986174,0.882553161880229,0.00448554090111471
"Ttc13",1560.15973442484,-0.189707561094775,0.184202372790807,-1.02988
663077767,0.303063227317641,0.4476611216848,-0.152471525312594
"Man1c1",885.25351749375,-1.36790072856872,0.210460666905637,-6.499555
23129668,8.05578223053706e-11,2.01373899911553e-08,-24.2401414122209
"Tacc3",448.87708019396,-0.0903583289036722,0.277904075720397,-0.32514
215082827,0.74507348879384,0.834239283593792,-0.0163760923705065
"Jade3",439.230695760658,0.136279918907929,0.174441608740619,0.7812351
64544752,0.434664195188267,0.57681089483297,0.0749867722556754
"Bag4",1552.92627991596,-0.0692741803506205,0.252654438351974,-0.27418
5487508097,0.783942080217139,0.860390831646905,-0.0104166571903221
"Traf3ip3",344.068977622581,-0.960431272998921,0.481550290683378,-1.99
445684403171,0.0461021517125117,0.109170179401684,-2.12720864580507
"Tap1",2894.40519968644,-1.23060554545153,0.332663418855826,-3.6992511
8212311,0.000216236515119595,0.00180024746874546,-7.77721924830084
"Bbs7",456.215757172744,0.528773831107749,0.15789456339625,3.348904609
0887,0.000811317234714422,0.00501046112151127,2.80050641459071
"Capn15",2391.28466218463,-0.0897273250996568,0.205683846101394,-0.436
239047452587,0.662663300169893,0.77118525311471,-0.0233135510000066
"Larp1",10446.9762375105,0.286474052119297,0.142114856971702,2.0157924

2468886,0.0438216860315754,0.105213056796411,0.645073066822924
"Hand1",1.49047149848955,0.173424208914893,3.39011351322465,0.05115587
08103354,0.959201315764919,0.975155495843599,0.0043630648277738
"Mfsd2b",294.846540289033,0.753523658105308,0.24299318958336,3.1010073
1381531,0.00192863526874146,0.00971442275120668,3.49193684987869
"Map4k1",599.998438080666,-1.16827718937382,0.485324845561973,-2.40720
663707426,0.0160750714557997,0.048692208054246,-3.53080968413509
"Fam53a",1558.96216806042,0.150823438423374,0.13477140897736,1.1191055
9938355,0.263095091349423,0.404975770990056,0.13633353483593
"Slc9a7",39.6929417624434,-0.0453847759355739,0.402658376056124,-0.112
712856938677,0.91025820784577,0.945059346995571,-0.00256458260464955
"Taf2",1494.33393298905,-0.0863337925707402,0.132605652722412,-0.65105
6654058827,0.515009915968411,0.648812001134075,-0.0373490587518096
"Slc12a9",2222.78516434385,0.209907534782445,0.164978723935129,1.27233
093926088,0.203255560189333,0.335427584644233,0.229292325375169
"Hrh4",0.68698123661467,-1.15230856617333,2.09144065242196,-0.55096402
7996165,0.581658333986564,NA,NA
"Chst7",737.2554509431,1.17911410661971,0.383393021110118,3.0754709702
4763,0.00210170386895763,0.010369185738091,5.38727422814273
"Paqr7",3032.8545146529,1.34652715491312,0.350894695217947,3.837410976
18125,0.000124338287269835,0.00117801162545541,9.08088127386477
"Nudt22",892.962480104441,0.556802867389905,0.155987133846558,3.569543
54926237,0.000357603768845935,0.0026501551824241 9,3.30358773948142
"Actr1b",5697.73342856952,0.123870956891391,0.0942720037812398,1.31397
394690831,0.188854971838936,0.318069814366034,0.141892246039978
"Letmd1",2758.15367885701,-0.327165790396795,0.340753851721342,-0.9601
23528300836,0.336993048224252,0.48143980468028,-0.239149709959837
"Uvssa",1665.41716500841,0.408468722458545,0.211051866778988,1.9353949
7514273,0.0529418454648021,0.121442365043848,0.861180934954594
"Dipk2b",43.0166233002925,-1.49419938816082,0.500459755411014,-2.98565
343567672,0.00282973138167988,0.0129334511204862,-6.49668661883357
"Sf3b6",2028.71563798318,0.336266991496257,0.254376871006732,1.3219243
9574177,0.186193327875138,0.314810744615802,0.388651884571366
"Ccn3",115.662280385208,-0.981412436805608,0.320771525820011,-3.059537
2650261,0.00221679207217882,0.0108274077713784,-4.44155334023077
"Letm2",596.002300912673,0.423316486260454,0.162238883690477,2.6092172
0262861,0.00907496205738312,0.031356301647148,1.46566561261635
"Srrt",7972.87965443721,0.0678918588753253,0.159531715199682,0.4255696
66760911,0.670421447224306,0.776977970635967,0.0171320544106727
"Pafah2",3556.84804361815,1.01444135944855,0.220129648491046,4.6083813
1711193,4.05815908738213e-06,8.45363097070265e-05,9.51376524133333
"Kdm6a",1292.1700022868,-0.245933196407992,0.118996445412131,-2.066727
25018155,0.038759861925799,0.0957117259155557,-0.577061208288994
"Enpp1",2021.63767536513,-0.0904633957184401,0.231550336262889,-0.3906
85659016851,0.696029600510514,0.79739073670252,-0.0204818591263429
"Ctbp1",7330.03943915785,-0.0596978978810575,0.161113830437734,-0.3705
32422442336,0.710985823317755,0.808043925335135,-0.0127239418331048
"Hhat",234.317753921432,-0.0476091517615224,0.224812272387664,-0.21177
2921717662,0.832284194720033,0.892673419561928,-0.00540528012524394
"Trmt6",1233.77063685393,-0.0197289360190516,0.113922783134319,-0.1731

78142916246,0.862511403479106,0.913137174623208,-0.0017927519156641
"Spon2",59.3730615944842,-3.01915899712734,0.623575647423996,-4.841688
42962285,1.28740514665208e-06,3.360351479173e-05,-31.0999941969213
"Rims2",223.896991805036,0.372466752281392,0.426029643555547,0.8742742
62168399,0.381968912284935,0.526781005215141,0.238740150786637
"Muc3",1.10637024828738,-0.171485790319005,1.53767375603072,-0.1115228
69949781,0.911201731121239,NA,NA
"Nmur2",2.23252716785896,-4.55818606959635,2.78986969722726,-1.6338347
5368995,0.102293552786547,0.200736684000814,-7.31935860464728
"Rcor3",1247.94320659633,0.253125993630864,0.136263229870433,1.8576250
8250796,0.0632222740598725,0.138849729625974,0.499762599319352
"Atp11b",2127.64072644523,0.121695730117072,0.12762837670811,0.9535162
41888699,0.340328570892857,0.484961373621934,0.0880695001898031
"Icam1",3575.02639948343,-0.70118622449157,0.353064110590491,-1.986002
55154468,0.0470330405962165,0.111036472399205,-1.54113478474649
"Htra4",120.221021094692,-0.520997875294611,0.4087128009598,-1.2747285
4794645,0.20240533866619,0.334420751911989,-0.570677806158015
"Cnnm4",577.297991372707,0.144724659694285,0.133981252013646,1.0801859
0302131,0.280059404571694,0.423926579484427,0.124201979375212
"Tbc1d2b",1768.66277759283,-1.18284589030907,0.182275155041383,-6.4893
4239030286,8.62118412173929e-11,2.10119810007091e-08,-20.9105543262497
"Serpine1",1304.82566587364,-1.25934291870101,0.307934699131769,-4.089
64277897802,4.32038054156989e-05,0.000518073676503873,-9.5274242190932
2
"Ranbp10",2355.88280311075,0.0968103081609908,0.110193218122195,0.8785
50511644342,0.379645038636287,0.524429257054929,0.0624857039056889
"Dmxl1",2827.37729522802,0.63344993477384,0.150441678692239,4.21060134
585243,2.54691835943713e-05,0.000346061422803521,5.04789555942042
"Best1",27.3764994581534,0.0556591759270266,0.36139031854724,0.1540140
20493886,0.87759868073032,0.923643478186321,0.00442095980445815
"Endod1",1922.59208635439,-0.724326813682492,0.218244670968242,-3.3188
7514352133,0.00090380831053977,0.00545080557961783,-3.77518546301801
"Depdc5",726.02787107952,0.0443478279193998,0.213422737955527,0.207793
360464905,0.835390317048754,0.89489514062099,0.00492476991941681
"Vgf",14.3331133081695,-0.8626926708817,1.04759225874252,-0.8235004255
54152,0.410223475280115,0.553723594393333,-0.509928703564692
"Fer1l5",1.33080173873238,1.18090413916733,1.86294840881097,0.63388987
7777689,0.526152691932271,0.65870417653793,0.493004737170139
"Slc30a1",2458.34018120709,0.421462231559534,0.221839950461503,1.89984
820444987,0.0574530428822385,0.129400419327468,0.861824370603335
"Adam32",13.023933232457,0.776037353114402,0.788297866701042,0.9844468
51749137,0.324895851417421,0.469837522134309,0.586194048466354
"Vnn1",1891.9216670964,2.63509360730928,0.619315837283144,4.2548461522
7843,NA,NA,NA
"Cep85",2251.05236411987,0.814202211614308,0.408849698481147,1.9914462
8121048,NA,NA,NA
"Tulp1",38.8854699224741,-1.20263396746705,0.522285198009294,-2.302638
42829726,0.0212991951282146,0.0603884988526871,-3.37574136261117
"Arid5a",837.805403395974,-0.854056850480785,0.330534241981801,-2.5838
6799915214,0.00976991784200169,0.033166969280104,-2.90908912692959

"Slc22a20",0.619583298269589,-2.6884766422384,2.61335054765691,-1.0287
4704070942,0.303598558701306,NA,NA
"Slc18b1",1152.01267900758,-0.47744674090963,0.317530011490705,-1.5036
2713328471,0.132677400714905,0.243844279304291,-0.673784995132182
"Azin1",4201.37596164439,-0.0309660480060211,0.0809287561489913,-0.382
633435623452,0.701991575487307,0.801841343810868,-0.00683868189540638
"Ints7",1587.09397236543,-0.0408031810803618,0.132288128279432,-0.3084
41744630125,0.757746221648052,0.842854396336032,-0.00697575495557956
"Fbxo27",41.2598414421737,-1.88664159204277,0.399547532291372,-4.72194
529953179,2.33599516381192e-06,5.37113605000057e-05,-18.5492451008932
"Klf10",2240.34110237351,-0.934953831935238,0.222313305706586,-4.20556
848346815,2.60426712427938e-05,0.000350778232908731,-7.43789090314396
"Tedc1",135.935912204087,-1.03665780266994,0.324568315642525,-3.193958
72211909,0.001403361818568,0.00763682632945543,-5.05347163671776
"Acp7",0.0852369980448597,0,4.78471990596509,0,1,NA,NA
"Uggt1",2885.77065109324,-0.0741618986311977,0.124488812967152,-0.5957
31430508267,0.551354657652612,0.681305096324903,-0.0284592623093295
"Dtl",128.114605498999,-0.976750775477422,0.319902520755968,-3.0532762
7043776,0.00226357439613988,0.0110007910209211,-4.40493902174196
"Thoc2",6931.86977923732,0.399086389540723,0.246675833475051,1.6178576
7141671,0.105693274265257,0.205489572165088,0.631498335384967
"Tbx10",81.7041230403846,1.79997799230542,0.841855431545249,2.13810818
919528,0.0325079634445235,0.0836311216837734,4.46635659803592
"Erv3",2.48350683837848,-1.17146084415736,0.993143407234394,-1.1795485
2806155,0.238179824978498,0.376324337991094,-1.14487325916453
"Asxl2",1841.01801026042,0.124879032723256,0.160277602168242,0.7791421
32362151,0.435895993608753,0.578091421805433,0.0684366138198742
"Ubr5",6203.94223560633,0.000438187999394253,0.10624323571796,0.004124
3849213845,0.996709226278067,0.997783872976832,9.72157877672058e-07
"Slc2a12",290.051785398449,0.510158438013532,0.357187027217041,1.42826
698379373,0.153215027077991,0.272498467850344,0.663268355398291
"Zmat4",12.5037120045705,-0.472951331910071,0.907227928556036,-0.52131
4784326394,0.602147503559796,0.723705358097078,-0.15293871364451
"Cib2",925.837140243729,-2.22548166025781,0.694986782807307,-3.2021927
831034,0.00136385690476533,0.00747820076746751,-10.895430891956
"Nenf",2120.55899041509,0.0973888230420071,0.235456305353551,0.4136173
92389524,0.679154333772307,0.783976745005769,0.0237020945082225
"Fam168b",5626.17528253526,-0.528875661347342,0.133728778418708,-3.954
83805057592,7.65865121839942e-05,0.000803704959399096,-3.7689151605222
2
"Arhgef4",212.503383664996,-1.4353823926225,0.29096325469133,-4.933208
47041401,8.08897391695966e-07,2.34004174232759e-05,-15.3051332199185
"Pank2",2530.10656933115,-0.153468507697955,0.160328143675002,-0.95721
5022766362,0.33845873601806,0.48307359892249,-0.111661577270027
"Ppfia1",2796.60011404091,0.462256815170403,0.123568641854625,3.740890
95932796,0.000183369067889408,0.00158270477454966,2.98091854941553
"Mavs",2347.74281423893,0.0618096302293133,0.233241849039989,0.2650023
16195479,0.791007677522709,0.865540585685941,0.00892537319989888
"Bcdin3d",405.672606503587,0.502018865231962,0.172765250455113,2.90578
611097719,0.00366331766239955,0.0157366358917201,2.08426374592155

"Atg14",922.891263312279,0.292223986883793,0.0922801246812538,3.166705
59227318,0.00154176319996546,0.00822455928043336,1.40293414680316
"Mrpl47",864.575377632032,0.597124413346927,0.188162012867553,3.173458
89452853,0.00150634230086118,0.00807329911550064,2.87765783348228
"Rapgef6",1353.21720111231,-0.469777538997995,0.158709874194907,-2.959
97675873067,0.00307662251408993,0.0137776724344799,-2.01285861023876
"Fbxo34",706.102818306431,-0.12339311228721,0.146708450524908,-0.84107
7060290132,0.400304765363605,0.544428287215633,-0.0750253628110325
"H2-
M11",0.146686701365375,-0.548352236011353,4.78471990596623,-0.11460487
6939106,0.908758313945078,NA,NA
"Shank2",1043.34440538157,1.0107760451106,0.37573232613965,2.690149275
93672,0.00714200637021877,0.026165885044443,3.68255916659447
"Aldh8a1",8303.11877850935,1.99281921921063,0.91986063125653,2.1664360
3552033,0.0302778827845969,0.0791787303184663,5.05388434393731
"Dlgap5",104.731390828008,-0.351169800522852,0.381430829352435,-0.9206
64438999442,0.357225647739785,0.502393658369181,-0.241735207394176
"H2-
DMb2",934.608242235527,-1.60884046228616,0.621295931655649,-2.58949138
45627,0.00961178279761082,0.0327927455796703,-5.49828943889513
"Plekhg2",2933.90808735012,-0.733269785657417,0.296307150124201,-2.474
69487438982,0.0133350048677692,0.0422705129103827,-2.31982034531707
"Zdhhc18",2155.05523124075,-0.394491983031323,0.12181907361374,-3.2383
4331791232,0.00120226037841021,0.0067633216555806,-1.97097707519518
"Rps16",15078.5155305106,-0.146885816168633,0.194606757530191,-0.75478
271172493,0.450379368717099,0.591028195668731,-0.0772460101705338
"Vash2",83.2914069158857,-0.824127010900707,0.441551162642116,-1.86643
605685322,0.0619803824167217,0.136800259945805,-1.63938095417455
"Mcrs1",2306.8563636749,0.15937645309729,0.130101767802355,1.225013739
5474,0.220570043814555,0.356488288651874,0.164389462613048
"Wdhd1",314.348369602957,-0.364997782259026,0.401795369919342,-0.90841
709383634,0.363657897809799,0.508632576739703,-0.24674922307565
"Tob1",5557.86661688602,-0.471587057437186,0.475751410282423,-0.991246
788227564,0.321565089100951,0.466856002032043,-0.359224091643107
"Ephx3",50.7203018820901,-1.30316500238473,0.378199874056238,-3.445704
48532446,0.000569572853070614,0.00381502284066329,-7.25707650793987
"Pkd2l1",1.34553949087394,-0.0936824469310277,1.78853370776542,-0.0523
794695757082,0.958226332562496,0.974621650094082,-0.00240819484284674
"Kcnh3",93.2907253491752,-0.528084392791063,0.49477921924298,-1.067313
20203593,0.285830428481718,0.429958470493484,-0.445738427089863
"Gch1",230.665593132549,-1.03487982329293,0.247074164051265,-4.1885392
0751587,2.80755810457546e-05,0.000370126896031186,-8.17727331555051
"Nr0b2",54.752203903387,2.22685192389942,0.639599113994443,3.481636974
12309,0.000498358824122003,0.00345186513418501,12.6236685416445
"Rskr",42.350925592521,0.245688910887041,0.421161690702294,0.583360064
105904,0.559650936095756,0.688155007378132,0.091824059783399
"Clba1",752.664184121823,0.963325494617235,0.325948968508305,2.9554488
2079506,0.00312214492289021,0.0139295754550897,4.11700363251614
"Kdf1",1028.90440777572,1.09144430146228,0.260949706082423,4.182584904
37825,2.88213329076252e-05,0.000378168471758329,8.60076748054248

"Nme1",5054.83732594802,0.676040507116095,0.291708176464332,2.3175233389413,0.0204752407967168,0.0584868882894755,1.91924701059441
"Adgrl3",237.851857406727,-0.447568170515964,0.575218689412318,-0.778083498944078,0.43651978986194,0.578543018940467,-0.244928267358094
"Osbpl5",2299.60296397851,-0.265737001035023,0.146023237208688,-1.81982680369732,0.068785384516042,0.148163657456229,-0.507408280623103
"Bclaf1",5851.48902850128,0.262228101007401,0.159901757624594,1.63993257424375,0.101019187034288,0.198936684051565,0.423437723337304
"Kcnmb2",21.6202937089271,-0.736051592734068,0.713153133440864,-1.03210875507582,0.302021172897788,0.446722005254968,-0.59312420384738
"Tnfrsf23",176.751852352983,-0.980174368623841,0.244548817535661,-4.00809285647397,6.12110543306762e-05,0.000676200077812762,-7.15431384190808
"Spag1",397.149981201165,0.197758318312616,0.199969300114892,0.988943393805922,0.322690837849823,0.467793751404896,0.150242488608658
"Atoh8",608.753685764714,-0.646012688824279,0.264206531466627,-2.44510491560607,0.0144810009876639,0.0451183376889535,-2.00164868424551
"Wdtc1",3324.90249406954,0.477686104467214,0.186531152365509,2.56089183179004,0.0104403856815328,0.0350855980728244,1.60023151313934
"Kcnk2",495.413843027985,-0.725974853976912,0.35074069785411,-2.06983352208211,0.03846793701114,0.0951595833345862,-1.70763803129465
"Cldn11",62.9802956855536,-1.26218706819935,0.686319704116234,-1.8390657599211,0.0659055144271139,0.143242193768796,-2.45270515868499
"Rgs22",1.16864891056882,-0.552271812181936,1.92219950913342,-0.287312430139427,0.773873105841834,NA,NA
"Cdkn3",44.6321442883208,-0.347886836729277,0.331103305742677,-1.05068971132425,0.293401122940957,0.437365068432934,-0.287697903888266
"Slc25a43",53.1306972215924,-0.274315918450362,0.392538428418321,-0.698825639965187,0.484661002885481,0.622172975632144,-0.13017308838944
"Zbtb42",543.243087386686,-0.469800382945316,0.224917673173051,-2.0887659752013,0.0367287946335757,0.0918243412957377,-1.12182593341933
"Zfp60",999.328627322598,0.00509639441973246,0.101338191136208,0.0502909550939433,0.959890531440116,0.975703554479167,0.000125353332179732
"Prkci",2197.43543633791,-0.578545923933235,0.153640295802857,-3.76558715218561,0.000166158151526707,0.0014692751194865,-3.77384702917424
"Vps13b",1498.89274876243,0.0595699641126494,0.120673261578773,0.493646755986316,0.621555674006062,0.739607569346831,0.0179684185992305
"H2-
DMa",2672.00038107596,-1.13011672020916,0.446180387080946,-2.53286955888569,0.0113133043979149,0.0373306497123954,-3.71575662636846
"Phc3",2780.17955365694,-0.0196737928725514,0.134977085060886,-0.145756539813234,0.884113585609342,0.927895720325706,-0.00147230644555029
"Kctd8",7.04639194944388,1.73673550353481,0.625085146333379,2.778398292972,0.00546276119505124,0.0212388669553558,6.68977043910264
"Slc20a2",1865.2757633508,-0.019463438175153,0.10947057913104,-0.177796064747722,0.858883137986945,0.910680484228041,-0.00182106105373067
"Gdf7",3.35283808378736,-0.260107562938472,1.29396920212352,-0.20101526567372,0.840686637418998,0.898077364474886,-0.0279613191427591
"Gpr160",683.732378298367,0.721658196506084,0.127095953711764,5.67805799815395,1.36232527452616e-08,1.01384038941645e-06,9.96015689051226

"Cdkn1c",3499.41859818322,-0.601306853067751,0.249945022855329,-2.4057
5645875451,0.0161390197052514,0.0488479674344911,-1.81537095076148
"Ldah",1605.69025091636,-0.24382666983832,0.268232619952866,-0.9090119
98172204,0.363343792536726,0.508324253891018,-0.164981839169557
"Rfx7",1822.84961000447,0.313471758836181,0.162029365596093,1.93466016
288432,0.0530320119591513,0.121563386924469,0.660585134157636
"Inf2",1962.98348996777,-1.05579496759758,0.200964102484743,-5.2536495
5503799,1.49114485783911e-07,6.24065568814102e-06,-12.6530959243251
"Esyt3",80.2760614688886,-1.71397631626519,0.597660304714606,-2.867810
19710461,0.00413323408521419,0.0171986715608152,-6.96375402473682
"Armc3",28.4715113361809,0.0955085701848893,0.594884558665168,0.160549
755063732,0.872448031032209,0.920135116312274,0.00794963236007788
"Atp8a1",1753.09541931715,-0.810166750590235,0.195349614826953,-4.1472
6566677854,3.36469559186849e-05,0.000425879091789407,-6.28799182053682
"Aspg",3055.09378133813,0.314247113086082,0.561665345731129,0.55949172
4875818,0.575826177208859,0.701409308628621,0.111452034371979
"Tmem247",0.548761693872035,-0.47052619180003,3.62476210081469,-0.1298
08847784597,0.896717662628188,NA,NA
"Ahdc1",2344.51134370267,0.149574187304934,0.271080270564844,0.5517708
35233672,0.581105371378438,0.705547825589188,0.0521685927939026
"Ddhd1",2246.93505600342,-0.32098905930739,0.1652474769635 9,-1.9424747
9722862,0.0520796480867643,0.119958532592526,-0.680692338672472
"Lzts3",4630.53536319493,1.0837659112123,0.385296272486968,2.812811824
56535,0.00491103813542478,0.0196059421712761,4.26128366497936
"Tecta",10.3859832878627,-0.426561601658391,0.762135812631893,-0.55969
2373181285,0.575689285431552,0.701286373069124,-0.151360672799036
"Cd81",14921.6842413242,-0.00726493177096843,0.138476250171413,-0.0524
633773804212,0.958159475745198,0.974604489440235,-0.000186879778550595
"Spag6",9.61312791472902,-1.03035757609366,0.73973822467722,-1.3928678
3043183,0.163659761989276,0.286166087280683,-1.28916579241693
"Fam13a",2796.28394828381,-0.52363668001606,0.568395734338398,-0.92125
3711774532,0.35691798166319,0.501997172795851,-0.360869868694424
"Cisd1",7712.31850897678,1.07152243237835,0.458842263414651,2.33527405
344967,0.0195291087228294,0.0564032827548108,3.08087121158683
"Fermt2",4543.90677965857,-0.445314718763918,0.232465374144458,-1.9156
1741357313,0.0554138114086646,0.125693170642874,-0.923543314345372
"Ccdc33",57.7803244703773,-1.63516646995802,0.67403896329191,-2.425922
77154439,0.0152695192307464,0.046765981375982,-5.00785957843676
"Tmem33",4216.42051565046,0.434466412782393,0.268916782906365,1.615616
57880487,0.106177237197883,0.206117460263274,0.686156749462885
"Gnpnat1",1708.2237148395,0.587970005429856,0.124444639836195,4.724751
55381376,2.30396856993828e-06,5.32260416785031e-05,5.78619093692808
"Ckap2",150.76536224645,-0.0407340797622842,0.409585407430817,-0.09945
1980034627,0.92077941318854,0.951272028736828,-0.00203487960648294
"Avp",0.184661161985439,-1.5136018459531,4.76413058682178,-0.317707883
604182,0.750706530585793,NA,NA
"Mynn",966.739229661581,-0.321930517697255,0.204185002454617,-1.576660
93898747,0.114873562739011,0.219044584189201,-0.488845049008
"Themis2",818.456029514741,-1.24203517685377,0.345487307911319,-3.5950
2403825665,0.00032436162610356,0.00245990003335947,-7.46169346153561

"Limch1",2308.58611248379,0.532323152167559,0.291350372079087,1.827089
31644357,0.0676863413131399,0.146248701565115,1.02336287183229
"Actrt3",5.2981971538578,0.813060927178888,1.00151364492621,0.81183210
1637307,0.416887979202825,0.560254065523393,0.471058970658678
"Nek5",87.4798875126535,-0.400256181035874,0.33189669111833,-1.2059661
6883165,0.22783053528211,0.364237436612871,-0.404238460109419
"Mrps26",2231.24066294307,0.598155842301123,0.169775239702087,3.523221
89826229,0.000426334256156678,0.00304604812258809,3.46566125146013
"Eef1a1",167201.388656036,-0.200419150373812,0.213553648310641,-0.9384
95558185344,0.347989798576415,0.492817046240772,-0.141820053401093
"Phyhipl",1465.9123821724,2.31658730831394,0.771693749525153,3.0019516
287898,0.00268254797040342,0.0124415604964143,10.1622030997167
"Fam222b",739.447441663784,-0.174061754418124,0.0947090765184179,-1.83
785716022978,0.066083456170191,0.143564927956143,-0.337848275591125
"Xkr8",240.12002326574,-0.599130323912864,0.218532289179176,-2.7416100
667011,0.00611388731897201,0.0231923297558981,-2.25508679826133
"Ppp1r16b",2120.10460253476,1.33049384046125,0.241982004508067,5.49831
729498255,3.83432537302209e-08,2.19887282715249e-06,17.3330958936986
"Ptger2",99.5502518618292,-1.54049918754081,0.283730459808201,-5.42944
592054786,5.65292788947192e-08,3.00329122585622e-06,-19.588682334679
"Actr5",728.48108678904,0.169238211518313,0.167415370526216,1.01088813
402478,0.312069973923371,0.457189897930565,0.132455376616233
"Slc16a9",3565.87807929027,2.02642316494651,0.573377898274804,3.534184
29807578,0.000409035757614015,0.00294643946096764,11.8082875516781
"Slc32a1",0.355826751147007,-0.527903618314056,4.78471990595454,-0.110
331143450442,0.912146759127146,NA,NA
"Mrpl23",297.270744256464,-0.148455022808588,0.237798580231759,-0.6242
88936729155,0.532437848197864,0.664724895777591,-0.0606263612742221
"Pced1a",1050.12425552215,-0.155775882769723,0.161096716995761,-0.9669
71181503479,0.333558440394069,0.478285223621382,-0.114892194260377
"Mbl1",129.884232614111,2.386240553682,0.805204874005786,2.96351975840
729,0.00304142535567914,0.0136734405314807,10.2424602691381
"Dzip1l",2091.3152908441,0.220186355418303,0.135629222762299,1.6234433
1799494,0.104494670985819,0.203946922211882,0.350073296571874
"Apopt1",1462.12087798692,0.583792005842019,0.264165389974427,2.209948
87293348,0.0271087108077378,0.0728754506175751,1.52895327790752
"Vopp1",1149.95225897158,-1.18194420370922,0.2363855932105,-5.00006869
139739,5.73098929148025e-07,1.75972959378397e-05,-12.9396477489097
"Phf12",2748.7878077642,-0.0781261341660694,0.170874178955089,-0.45721
4393911461,0.647516953448321,0.759802178559905,-0.0214610280328344
"N4bp2",1013.74259241834,0.627876426657634,0.308400050713432,2.0359154
4555569,0.0417588465724288,0.101169730426096,1.43843706180244
"Adh4",10.9121686730896,-1.1204573980529,0.631875657190073,-1.77322450
280097,0.0761915133974022,0.160049787569764,-2.05298085750815
"Mat1a",13.5807855803741,1.00833904350537,0.779172004420357,1.29411611
015914,0.195625317656832,0.3262876341893,1.1293155015262
"Iqch",1.95145992063425,-3.26062889360136,1.46928903447358,-2.21918820
402112,0.0264739232440535,0.071662807618712,-8.59431147268667
"Rpl10a",12982.0205055298,-0.19477206173652,0.181754691711604,-1.07162
054471513,0.283890501705614,0.427907361097486,-0.165331982743008

"Fam76b",1566.28468396969,-0.185140731871396,0.113722382559612,-1.6280
0609435304,0.103523593533987,0.202579589193666,-0.295599846433984
"D630003M21Rik",415.476583166221,-0.900544672390389,0.370012042756105,
-2.43382530385366,0.0149402038085118,0.0460577691670332,-2.77174936178
207
"Ctnna1",15370.3299586193,-0.161428779412162,0.0838889904360997,-1.924
31424639836,0.054315205923147,0.123762753896169,-0.337287487100586
"Fbxw17",908.536029178778,-0.327736689393185,0.233763204691507,-1.4020
0289359351,0.160914382620658,0.282531169053362,-0.414248163948088
"Abhd18",869.295917052143,0.554015389406803,0.231369057840387,2.394509
42393948,0.0166426157617786,0.0499534670756096,1.66019762128838
"Tgm2",12373.0646409899,-0.519078315584858,0.15651220975754,-3.3165356
0057062,0.000911409747710387,0.00548477384594356,-2.70220721132848
"Smim14",4636.03721749495,-0.288026982825641,0.204005765702532,-1.4118
5707096937,0.157992050154933,0.278857736618199,-0.367825875671355
"Tspan14",3906.4899189764,-0.199512994335557,0.168606000528044,-1.1833
0897898484,0.236686715554335,0.374800420683702,-0.195794392578249
"Ppm1k",1039.9367533521,-0.0661371057080233,0.132796967473778,-0.49803
1747005687,0.618461666001467,0.736818132940034,-0.0201992101684665
"Sh2d4b",202.113262175245,0.276782110211388,0.355285406472743,0.779041
596330307,0.435955212036308,0.578130634855739,0.151664258552469
"Vstm2l",4.99600882831468,1.63612580680205,1.29991387018579,1.25864170
259849,0.208159776998121,0.341354022868744,1.75856552699662
"Fdxacb1",437.530014118771,-0.0301390257317561,0.114050875643086,-0.26
4259485618277,0.791579976683213,0.865923832213268,-0.00433876374743802
"Rtkn2",14.817895729846,0.17625937782589,0.712206952959751,0.247483371
361935,0.804534159052464,0.875540100057596,0.0234273963768323
"Nmrk1",2590.32395042903,1.7263724398173,0.297903416068697,5.795074331
80693,6.82909274914499e-09,5.96319630519015e-07,24.7432140395234
"Ifi206",221.575713025776,-1.06076073576794,0.479782051880474,-2.21092
208766535,0.0270412328466085,0.0727642779524112,-2.77975547057537
"Iars",1626.35933413484,-0.392128778993432,0.137417847754044,-2.853550
57878129,0.00432336451214583,0.017799189454776,-1.57973092407275
"Cpe",5794.49357659294,-0.356093348205503,0.440001265162547,-0.8093007
37064822,0.418342182385361,0.561340298131564,-0.205618257520836
"Zfp365",24.1208331723111,-0.889311310630606,0.499084946812528,-1.7818
8365790295,0.0747682060306519,0.157842191553232,-1.6418105536708
"Nufip2",1805.53081262189,0.0350028787723566,0.167293522153989,0.20923
0329552972,0.834268436806175,0.893963837153594,0.0039234711147933
"Aim2",424.29208549963,-0.888340465267321,0.482467028039238,-1.8412459
5804519,0.065585523269956,0.142705784255954,-1.72957243193266
"Egr2",471.395904867863,-0.837263724118652,0.424557546683368,-1.972085
36430299,0.0485998624329481,0.113951028577994,-1.81852550301118
"Ackr1",39.6188411280771,-2.24139706145019,0.596887061899914,-3.755144
32213648,0.000173241622356985,0.00151444421523809,-14.5527338571076
"Jmjd1c",2604.34189859102,-0.181569903350474,0.205825307485766,-0.8821
55384915582,0.377692784865094,0.522880851980942,-0.117730226257574
"Stk35",1249.25280450551,0.257858650694494,0.182134900221202,1.4157564
002359,0.156846838933635,0.277488400828238,0.330568639097652
"Dusp8",893.798826693255,-0.500587672016222,0.238389878137666,-2.09986

965858987,0.0357403084627767,0.0899180044396414,-1.20584416075168
"Wdr19",572.784277791626,0.244833288407453,0.133238550591521,1.8375559
2747369,0.0661278681490718,0.143629405499677,0.47510361607475
"Pcdh18",665.401469709592,-1.45445581054307,0.368010875561305,-3.95220
877188663,7.74331383915868e-05,0.000809539588396332,-10.3543361931104
"H2az1",7135.52463357878,-0.238667944562395,0.249415506727983,-0.95690
9005752762,0.338613185257936,0.483223295481171,-0.173577568928974
"Rcor1",1583.21778738842,0.0839247340598431,0.119974966444421,0.699518
712503427,0.484227922898834,0.62180427032939,0.0398751516504329
"Sirpa",5753.35264573949,-0.415763836027635,0.310534293912726,-1.33886
609040508,0.180614270532493,0.308345770671793,-0.489160078529691
"Ankrd9",445.763220176309,0.0673592919045729,0.429770238121637,0.15673
3263333857,0.875455070703271,0.922183844871549,0.00545682186598709
"Bri3bp",1304.34826678274,0.223620909978519,0.103741214071267,2.155564
80594971,0.0311176631597651,0.0808652800598561,0.562400040554236
"Ankrd13b",1320.34427387824,0.224094382473763,0.251030448658569,0.8926
98012019085,0.372018915267696,0.51725042834118,0.147729321652818
"Tmem156",28.696242631916,-1.02683225463189,0.410266675935981,-2.50284
099309132,0.0123200880756147,0.0397381434736702,-3.31198968803019
"Ndufv1",12760.6044766217,0.847657428463783,0.312285562075565,2.714366
37297588,0.00664026834757369,0.0247367123119969,3.13588052320779
"Ddx60",446.385899281764,-0.754348578950158,0.277357221215193,-2.71977
262984216,0.00653268183166851,0.0244105462540959,-2.80070015548543
"Bank1",102.119563232579,-1.38615532033568,0.574266033373186,-2.413786
01515665,0.0157877313481345,0.0479484713124495,-4.21062471106842
"Ssh2",3136.59186638991,-0.19333101490656,0.235616810311057,-0.8205315
00495776,0.411913179271578,0.555389196420526,-0.113695293428365
"Bicd2",2941.07752961338,-0.84491832475656,0.23330720768765,-3.6214840
215641,0.000292917899891129,0.0022694707842903,-5.1440390189744
"Smarce1",3416.13034654105,-0.00982681769402411,0.189427747074433,-0.0
518763372620528,0.958627229011562,0.974775160952669,-0.000251059849753
131
"Scarb1",1690.54983786572,-1.27318767220287,0.385896921415221,-3.29929
471200144,0.000969280928795833,0.0057356720939791,-6.57098567314149
"Chchd5",550.724650998111,0.109133628596698,0.141129838558311,0.773285
293255732,0.439353557528566,0.581293049107144,0.0592050220895924
"Inpp4b",484.487926848031,-0.611492423469946,0.256997782326757,-2.3793
6848300298,0.0173423302703894,0.0515379904910916,-1.81334168531384
"Crp",7.53380485443732,-0.401878047452411,0.846080178431992,-0.4749881
3670023,0.634795429048273,0.749472235630487,-0.115896004996195
"Ccr7",198.403832516258,-1.48276429942271,0.416596003262941,-3.5592379
3749609,0.000371932455895816,0.00273864766807274,-8.74874089877563
"Fgd3",998.347613055482,-0.688033725367644,0.328078446303189,-2.097162
22787707,0.0359792169291419,0.0903558438542515,-1.6540327894625
"Ano10",1385.70581704916,-0.165682427061045,0.248058621666453,-0.66791
6422126325,0.504186944059407,0.638933903241278,-0.0742181502951178
"A4gnt",232.876951262201,1.79961738929085,0.682334531826878,2.63744146
800333,0.00835340401441109,0.0294156892672905,6.34585960492316
"Wdr20",1293.51911506045,0.111504052723248,0.130994063617188,0.8512145
48539419,0.394650182570326,0.538933272149897,0.0689277371779333

"Nsrp1",3205.76990120686,0.424085126628375,0.254213117002472,1.6682267
6826802,0.0952707189665461,0.190326688361651,0.703562853063214
"Card19",1140.40467180892,0.13814563999837,0.15514563566543,0.89042556
3090152,0.37323742120415,0.518462851644006,0.0907460611650502
"Rflna",145.549086922202,-0.274674133033252,0.416981267413265,-0.65872
0557729578,0.51007522930516,0.644469078602386,-0.12067215702710З
"Zc3h7a",6251.45834317878,-0.139359425884657,0.182471304969269,-0.7637
33376643126,0.445026146480548,0.58620535095352,-0.0744297960510507
"Ninj1",4178.50867022042,0.896980786344686,0.243932325380537,3.6771706
4536398,0.000235835280788571,0.00192519681799471,5.60857604705643
"1110032A03Rik",1909.7149937883,0.58899192818408,0.27845448490177,2.11
521796243238,0.0344113746963869,0.0873068293858363,1.43615467994179
"Snn",2693.00472834611,0.383636927264344,0.20834231364206,1.8413778773
8811,0.0655662024251035,0.142679666839807,0.746999891089709
"Itprid1",15.6732597271714,-2.15257239175958,0.933915682892866,-2.3048
8943615535,0.0211727663968647,0.0601199483775878,-6.05177123852832
"Muc5ac",3.77482962479787,-2.34149960348022,1.37791978547594,-1.699300
37158981,0.0892626026100745,0.18093795879938,-4.00303024048453
"6430571L13Rik",126.595984086963,0.538362568597041,0.365068773015221,1
.47468808178396,0.140296421744776,0.254749453453124,0.736197220143084
"Ccdc92",353.463362798369,0.267504009653077,0.285418623214939,0.937233
900997514,0.348638255046881,0.493627819105664,0.18885072814617
"Neurod6",0.220934797106264,0,4.78471990595636,0,1,NA,NA
"Wnk2",160.253679882934,0.472998174961026,0.220511331777227,2.14500620
511818,0.0319523508688773,0.0824846997539215,1.18019783002235
"Sh3rf3",30.2539023885391,-1.24457748250473,0.309843625965402,-4.01679
227264046,5.89956835404356e-05,0.000658441807482206,-9.1173196342511
"Rmi2",78.3263545247428,-0.605945889569956,0.396433603170082,-1.528492
75319879,0.126390241434595,0.235373154488228,-0.876551103220389
"Rara",1578.66991575316,-0.0170919623958407,0.216927071096781,-0.07879
12836762326,0.937198637112843,0.961004418954837,-0.000679854540730171
"Dhx38",1655.43814772482,-0.444295132516442,0.0822797268733239,-5.3998
129235464,6.67104258173933e-08,3.36974062846511e-06,-5.59841847180274
"Slc9b2",51.83793493052,-1.04886207475817,0.461034411956502,-2.2750190
6052324,0.0229047864464812,0.0638909193324021,-2.88497698475372
"Igsf9",1162.39474593972,-0.823695252258049,0.332958104417663,-2.47387
056007745,0.013365811290985,0.0423475119518404,-2.60439721672972
"Slc24a2",0.558696808128979,-0.539814244391325,2.57312247817178,-0.209
789564612901,0.833831918064125,NA,NA
"Parp11",834.455276098799,-0.631137210418874,0.14121424310657,-4.46935
943949065,7.84541954691003e-06,0.000140597417578724,-5.5979408787628
"Arap2",1181.98542539798,0.714833958405648,0.211447331844658,3.3806714
5217423,0.000723089470696756,0.00458349924558087,3.84958998722702
"Acd",1109.88934992422,0.247675667218508,0.184552744874326,1.342031880
30158,0.179585659031263,0.306966001559706,0.292509490900638
"Cramp1l",2124.17719816335,0.1025849276064,0.158679826636115,0.6464900
40864793,0.517962044808334,0.65135272168158,0.0439785655550261
"Hpf1",1387.04714482863,0.266136246620652,0.169724938828496,1.56804443
977191,0.116870761522768,0.222078365478114,0.400461853516471
"Acer2",3039.19344298186,-1.53225282589355,0.404372410986732,-3.789212

08337289,0.000151125853363188,0.00136862605149811,-10.103595341084
"Dnajc22",2252.39049660783,1.77406495622838,0.630970672097754,2.811644
08217301,0.00492890099357754,0.0196531107510787,6.97122309745486
"Ccdc138",866.145502694916,0.581957808390532,0.274085771404915,2.12326
895120283,0.0337313209369316,0.0860292080612547,1.42758231674579
"Dnah10",46.4815870852649,-0.236965134219436,0.435880630249298,-0.5436
46855984186,0.586684509589786,0.710171773199778,-0.0811009390196107
"Wipf2",2434.61043919344,-0.109710696599941,0.198701799387206,-0.55213
7408610729,0.580854213766355,0.705374654958669,-0.0382919066398269
"Fam120a",9667.48772472052,0.10229585831828,0.0736818332576163,1.38834
572642376,0.165031794480867,0.287778071785476,0.127416210325031
"Prm2",0.305058504246225,-2.03044571567107,4.74850910443877,-0.4275964
66809566,0.668944938738351,NA,NA
"Rapgefl1",557.066310654643,-2.01994362834953,0.59905127844033,-3.3719
0437788328,0.000746503615872261,0.00469223968480895,-10.8306251808019
"Mindy4",496.634419013425,0.567163992145928,0.236319465887626,2.399988
46483355,0.0163955885056127,0.049409557070517,1.70580889442204
"Atp6v0a2",3369.77720539156,-0.10504313613115,0.116109833766678,-0.904
687679962003,0.365630869763863,0.510605264192508,-0.0706056330788293
"Dennd4c",1397.07676313173,-0.388279966292701,0.158753547430286,-2.445
80340142136,0.0144529786883016,0.045059797558573,-1.20357658630771
"Phf2",2144.87352025954,-0.620181748572059,0.202170775365609,-3.067613
24652641,0.00215775683829726,0.0105947979937346,-2.82020961728234
"Kcnj9",34.3095598856848,-0.74430932559389,0.532562964274104,-1.397598
73578216,0.162233598216282,0.284309787706369,-0.936111016110281
"Tigar",490.397160454314,-0.229219942957101,0.21633858411686,-1.059542
58641761,0.289352745176052,0.43321813909565,-0.191745666697977
"Igsf8",1537.54819778771,-0.181840884790977,0.151103624561731,-1.20341
84177805,0.228814444187477,0.365393243290407,-0.183073970829602
"Socs1",242.582754889455,-0.918827065866402,0.362700621609127,-2.53329
333098469,0.0112996350808213,0.0373044843897483,-3.02169303571824
"Gcc2",1596.68977347388,0.173644092167539,0.124888313310278,1.39039504
630133,0.16440894989614,0.287012776889152,0.216747512589827
"Ptpdc1",1642.58960618863,0.922706115523204,0.220512056666686,4.184379
43698433,2.85946154675484e-05,0.000375952671872056,7.2765040665657
"Cct8l1",4.52341327234767,-1.46054345746048,0.870397735408036,-1.67801
844840024,0.0933434975921245,0.187109233684717,-2.44796240394018
"Sult6b1",2.5024041309472,-1.72132736394179,1.33652855101025,-1.287909
16036974,0.197777586374785,0.328808610090003,-1.91459701875734
"Mrm3",531.466174980746,0.9637682586906,0.2362122434804,4.080094429019
6,4.50174095832175e-05,0.000535539629074786,7.25932965277413
"Haus6",546.006449251206,-0.166891819890386,0.170574874459427,-0.97840
7988980128,0.327872578527219,0.472624052659439,-0.125077912413446
"Dexi",706.990281646305,0.278938627238589,0.147388372337219,1.89254160
83732,0.0584188553191972,0.130910336859408,0.56714991128521
"Kmt2c",3802.14207405618,0.0224262775981166,0.177414449603057,0.126406
150391317,0.899410433898256,0.938343658844678,0.0014271863967602
"Dbil5",12.5044703666675,0.577470229345819,0.446584399253,1.2930819578
8243,0.195982714496713,0.326688122361024,0.64604442492573
"Nod1",1454.77014620682,-0.630264944431568,0.160815676181761,-3.919176

03678895,8.88522027190912e-05,0.000900904965479376,-4.4194876127651
"Smim3",561.822628303176,-0.377308089939977,0.198778858083515,-1.89812
98794938,0.0576789749818115,0.129789342665362,-0.770403725288235
"Dlec1",1760.77582676152,1.29864004120426,0.420453023376881,3.08866857
651348,0.00201055591698188,0.0100363080566597,5.97575182962478
"Cldn22",54.8667780539272,-3.56079820012192,1.02980819052976,-3.457729
53921658,0.000544747962148086,0.00369700513956261,-19.9412967694683
"Mturn",3321.41191968884,1.22618266456076,0.251561580817659,4.87428430
277492,1.09203628158416e-06,2.97797530326264e-05,12.7788855879169
"Csf3",12.322520262083,-0.995970191667351,0.679072600130295,-1.4666623
1486332,0.142467939240158,0.257660688183341,-1.35064685140904
"Rnf144b",871.149903613015,-2.24462999647643,0.494162468742487,-4.5422
9153053331,5.56460146501473e-06,0.000107851490422323,-20.5041459046032
"Cdkn2aip",731.080257330455,-0.341720587012108,0.129790366091186,-2.63
28655762634,0.00846678499586197,0.0297290426524971,-1.2013634356908
"Cntln",593.823674817939,-0.42584063892021,0.171462027031235,-2.483585
70287189,0.0130067032251102,0.0414116099743955,-1.35595920881162
"Npy6r",114.998491748359,0.131023333411332,0.554661363842691,0.2362222
10437741,0.813260245707378,0.88113737890656,0.0165799192812658
"Galnt11",12052.3165046824,1.94041252726377,0.478208254213845,4.057672
59382365,4.95641860181573e-05,0.0005752395827274,14.4768821924021
"Fkbp14",783.739934002782,0.0339028739485302,0.154260297004919,0.21977
705609791,0.82604478934725,0.88881525868841,0.0039959918372831
"Kcna6",90.1903718299424,-0.768470751194922,0.437589037772513,-1.75614
717202862,0.0790632822996869,0.16480392113313,-1.38555189488461
"Tmem237",951.378213400214,0.763104458922925,0.216467843803453,3.52525
550915453,0.000423074508940109,0.00302608465712188,4.42637649199671
"Kdm1b",1447.4968027887,0.448282931801283,0.14891037546471,3.010421069
73616,0.00260885744262285,0.0122189532587702,1.97458185880966
"Opa1",3721.08250926974,0.115007892198536,0.260981065651209,0.44067523
4088589,0.659448128365956,0.768416877241344,0.030295710668003
"Cnbd2",303.432708902386,0.108305401884881,0.328241637130162,0.3299563
17643922,0.741432968803938,0.831691406382418,0.0199600154552035
"Hspb2",176.903354028492,-0.277111755265104,0.351427458952422,-0.78853
1881063456,0.430385657975751,0.572848628521483,-0.154388317137583
"Hsd3b5",2.26684180167208,0.31280997605495,1.49099091283233,0.20980005
5360986,0.833823729853016,0.89375155694601,0.0351371447357317
"Atp13a4",4.31974455916284,-0.0348209947047919,1.04760930252137,-0.033
2385314076396,0.973484371466894,0.983749354293898,-0.00057051189976721
5
"Sbno1",6692.08289876059,0.819893137282395,0.313090042188733,2.6187135
5457596,0.00882620268278462,0.0307281450191073,2.85534038260948
"Trappc11",2206.71762856231,0.264513297984858,0.108324819774365,2.4418
5310934119,0.0146120906966051,0.0453745093808577,0.81808799740653
"AW551984",7.21252187860876,-4.16779193750617,1.42933516212846,-2.9158
9547919594,0.00354669294669982,0.01535977282463,-17.4047130574322
"Ano2",3.4805393514348,0.0879435445889339,0.702679878440634,0.12515449
3941246,0.900401242472554,0.939025643224746,0.00553274746345921
"Phf20",2558.65246744864,0.180417120666229,0.316793089713404,0.5695109
09563867,0.569009469239607,0.695975574382652,0.065390509949341

"Cdon",756.291780321688,-0.888773982083031,0.160957546184027,-5.521791
3241972,3.35560875344524e-08,1.99474571569132e-06,-11.6651531613565
"Fam210a",2802.3704482392,0.386223349569706,0.312241462597195,1.236938
06183566,0.21611007813787,0.351365780706656,0.40396161776865
"Tbc1d32",696.040074963568,0.401879979846583,0.162258578502074,2.47678
725868689,0.0132570895805279,0.0420782569821151,1.27324585069469
"Mphosph9",601.610625679797,-0.0514461373846656,0.138228631816034,-0.3
72181484463613,0.709757730746611,0.807448289520825,-0.0110031076446485
"Ccdc50",5740.21245859755,-0.0855279684392448,0.0803379453114568,-1.06
460238816996,0.287055898658279,0.431044297820934,-0.071975584165736
"Camk4",24.5232268361024,-1.39619907767822,0.531238860404858,-2.628194
55002628,0.0085839407053011,0.0300084645805516,-4.89545901631015
"Rbm24",147.127623922257,-1.56345068536197,0.551895687755887,-2.832873
53035727,0.00461316335386531,0.0186404832747525,-6.22631656437705
"Crygn",11.5214289241168,-0.851000777944596,1.25447676635899,-0.678371
095237221,0.497536431269551,0.63322228047609,-0.388850989047956
"Tmem181a",1717.26297079445,-0.448406392426412,0.101647255906696,-4.41
139692780307,1.02705842754771e-05,0.000175048821855991,-3.878915122723
38
"Stox2",1287.71302189737,-0.0397550521402373,0.282625259867468,-0.1406
63478412651,0.888135798168491,0.930645719183114,-0.00285745846020274
"Snrk",5794.47180487707,-0.350913118881832,0.342695009519764,-1.023980
82590577,0.305844331479312,0.450404288155863,-0.279891700960818
"Notch3",1752.87308511721,-1.35973258529395,0.257493075175339,-5.28065
690453246,1.28721535628992e-07,5.54979427405244e-06,-16.4551434043002
"Cd84",986.794214281976,-1.23742822345851,0.435807745437225,-2.8393901
5865139,0.00451998535974533,0.0183758704220839,-4.94565025004218
"Cldn16",553.261664289843,1.24243372286973,0.54986505952393,2.25952477
130557,0.0238507612097299,0.0659073330594265,3.37880542734442
"Ormdl3",3504.51979151471,-1.94644135604205,0.522408249793023,-3.72590
087697357,0.000194618878826486,0.00165778894685837,-12.4616441208415
"Prdm1",203.571598024299,-1.03234288673266,0.355942415818238,-2.900308
70403437,0.00372795310386023,0.0159255425315413,-4.27372509564442
"Gstp2",465.498769093682,-0.207063252866043,0.317045795557227,-0.65310
2030582416,0.51369050328011,0.647484480765116,-0.0900022074200296
"Spon1",3849.44515289976,-1.32218597668661,0.451357248476359,-2.929355
80662522,0.00339665365794288,0.0148659827211157,-5.56465729952334
"Atg5",1651.97784439017,0.312526648330445,0.139528522394958,2.23987642
788753,0.0250989463494706,0.0686559001012877,0.837148945813681
"Plekhg6",395.531575054685,-0.380188838532237,0.42230525142583,-0.9002
7021271605,0.367976468943071,0.512999084187902,-0.253768909401953
"P3h2",3942.51216826048,0.918137186287695,0.295055702836474,3.11174187
606381,0.00185987043029277,0.00943876982036659,4.28120908387807
"Pde4dip",3887.54413228389,0.714141702893051,0.178213195803732,4.00723
245925931,6.14343905981132e-05,0.000677899121608382,5.21074355433686
"Ttc39b",1268.91123777118,-0.575187417918005,0.171898428286364,-3.3460
8887150385,0.000819601221359478,0.00505075367069124,-3.04171634470882
"Enpp6",3420.91108173775,2.48334578966016,0.650090548470129,3.81999983
772147,0.000133451761860854,0.0012444009817135,16.6113508149843
"Fam126b",2243.30809864538,0.147930966735605,0.385350358063848,0.38388

6932086604,0.701062252357958,0.801108351197061,0.0328050337780511
"Mylip",1896.64287294119,-0.758991473233228,0.17901148185982З,-4.23990
386173981,2.23615536601759e-05,0.000313447572441488,-6.12345094677478
"Slc43a2",5114.36783572384,0.389207635852626,0.211860750459242,1.83709
17454458,0.066196352550671,0.143746127857555,0.754948595245325
"Slamf7",593.228871148664,-1.2956176608793,0.463109651341065,-2.797647
72150068,0.00514762222325657,0.0203174773500115,-5.04808116003821
"Spag4",60.9263607694816,-0.941760768301304,0.518600613400226,-1.81596
539604265,0.0693756804756711,0.14912845196601,-1.79212106485085
"Chpf2",3020.29310966559,-0.360674047594215,0.125046914754232,-2.88430
984725281,0.00392272581078082,0.0165588456069722,-1.479064701044
"Btbd10",655.916610939846,-0.397963299169903,0.166080577006333,-2.3962
0614489272,0.0165657705951553,0.0497841237768709,-1.19391343522421
"Scarf1",1272.97117003913,0.169692183458499,0.211899808038163,0.800813
29487537,0.423239741167724,0.566469554728739,0.0964414882883912
"Cer1",0.115127663772937,0.87947850337457,4.78471990596623,0.183809819
730078,0.854162652365074,NA,NA
"Hand2",2.59680805481168,-0.44634427969311,2.17428579661066,-0.2052831
69484382,0.837350892850831,0.896217774522174,-0.0489067656140863
"Rilp",721.141452850529,0.699765457795814,0.375543232907271,1.86334194
435771,0.0624141724111466,0.137509749323647,1.3883769762384
"Iqca1l",0.938199495798466,-1.8268124820516,2.6043234085545,-0.7014537
73387364,0.483019873646879,NA,NA
"Kcna7",1.63352904382893,0.0882916494848912,1.45005841813118,0.0608883
396564675,0.951448135822282,0.969930765084564,0.00269559581830449
"Hoxa13",19.0788958926362,-7.79121089671383,3.79397546965028,-2.053574
39947602,0.0400169020383046,0.0979597684799577,-18.1005287696747
"Asb10",26.8580447101141,-0.959623830328153,0.400578517547913,-2.39559
484168637,0.0165934207745624,0.0498518518123909,-2.87762363029254
"Prkab2",1558.6451348368,-0.754273075392032,0.214384575310602,-3.51831
783746212,0.000434291866217666,0.00308862728392595,-4.35971989627994
"Fbxo8",2058.75593030736,0.737209383353089,0.243424204808661,3.0284966
2765688,0.00245773820320313,0.0116709643171102,3.2810618295869
"Pgap3",700.673472510605,0.378921329454423,0.154082756855477,2.4592065
7955151,0.0139244469003759,0.0437689610935886,1.18558056102887
"Hoxa11",438.760635393171,-1.15528724127658,0.271000495204273,-4.26304
476088045,2.01660103402317e-05,0.000290783946044037,-9.40742315871959
"Mfsd14b",2239.65117888557,-0.201842387916571,0.13072896222824,-1.5439
7605913963,0.12259413255067,0.230216738560431,-0.296452792350694
"Tapbpl",1080.76328195319,-0.639647188151187,0.214445759494917,-2.9827
924303924,0.00285631636253035,0.0130183395130006,-2.77696186168098
"Bend3",506.03451447228,-0.0301076495746544,0.167239128533279,-0.18002
7544024562,0.857130944458181,0.909267636291926,-0.00286371313857385
"Cep44",679.183845473977,0.322710016147904,0.155056876009528,2.0812364
111352,0.0374122723085117,0.0931033165885592,0.766128252410182
"Pnmt",0.0904006337861851,-0.0578135967474291,4.78471990596623,-0.0120
829636600753,0.990359424431597,NA,NA
"Tlcd2",517.914273787712,-1.4338690627805,0.526716908781553,-2.7222764
997187,0.00648338717240637,0.0242635476175776,-5.33224381259895
"Serpinf2",9436.5533839703,2.04425939210184,0.823652897657431,2.481942

81585843,0.0130668242968599,0.0415690879654389,6.50155943410879
"Primpol",604.78105824111,-0.222255868757561,0.15904403321626,-1.39744
864527768,0.162278699508158,0.284337714145405,-0.279507338737925
"Hoxa9",2884.43419309721,0.421225955733158,0.163525289989535,2.5759071
0134265,0.0099977503532062,0.0337676440200367,1.42721977647092
"Gask1a",27.5348094600182,-1.56451967180845,0.479694326203726,-3.26149
296821994,0.00110827181292553,0.00637060253786024,-7.91030724672275
"F11r",5964.39196340034,-0.0608798404788998,0.112607382961222,-0.54063
8090309447,0.588757058215812,0.712177251758292,-0.0206643498567673
"Hoxa7",3429.30029086706,0.99701546384908,0.17954090927897,5.553138100
13028,2.80586226808749e-08,1.75735172875028e-06,13.2121523962689
"Hrc",68.2187054484032,-0.129144443440901,0.344831163626084,-0.3745150
0056688,0.708021198462886,0.806372100212635,-0.0277931731176846
"Pdss2",641.237804782919,0.309694049917206,0.25468860036154,1.21597138
418282,0.223995823044397,0.360084966420947,0.316326227755979
"Cep250",2923.32890113569,-0.16183746132991,0.18178130526245,-0.890286
60618458,0.373312011134908,0.518472654256602,-0.106305848384653
"Aox4",6.11743934162624,-0.827907184793878,0.874880171227172,-0.946309
234134994,0.343990884873791,0.488608892931389,-0.59294126373778
"Mical2",843.049360389549,-1.36011941254526,0.247733696534235,-5.49024
792175297,4.01369949652969e-08,2.26182406888254e-06,-17.6806532698788
"Fam50b",2.39084529705107,-4.15160486791209,1.68686790183021,-2.461132
17484766,0.0138499331863483,0.0435839246719529,-13.0072557802422
"Sobp",166.917279410045,-0.4302995104153,0.32679627487392,-1.316720977
25506,0.187932169429601,0.3168444504253,-0.49456229741945
"Usp38",1229.88063745037,-0.0236887169023033,0.137944608013082,-0.1717
2629828385,0.863652709896156,0.914097304177355,-0.00212767917938914
"Ncapd2",778.253554994094,-0.0302890050857201,0.199791328992856,-0.151
603201392204,0.8794999082509,0.924763448232804,-0.00236912437361688
"Hoxa5",2128.73961596755,0.304006734757454,0.282610554191145,1.0757090
6411315,0.282057392404391,0.425991869643751,0.259419592548788
"Bcl9",1553.03778028372,-0.417866897848993,0.188480945343517,-2.217024
62860322,0.0266214085858445,0.0719147974548591,-1.09993984865288
"Glra3",0.097334754584379,1.42101320893202,4.78471990596623,0.29698984
2009374,0.766474264357238,NA,NA
"Gdf5",31.3207459848072,-1.39072557752242,0.52377217517631,-2.65521087
876476,0.00792588435415417,0.0282779310406767,-4.95887347722338
"Trpm4",1195.65263111248,0.0908067744846352,0.249018684911454,0.364658
477402707,0.715366357484215,0.811272799291992,0.0189923192865141
"Sema7a",1253.11727300596,-0.648458423420478,0.177977612324766,-3.6434
8310414007,0.000268973316071421,0.00212669980403916,-3.9900839234038
"Slc22a23",2176.28911973542,0.489210276557752,0.386548624691754,1.2655
8535022046,0.205661573701576,0.338595741284583,0.529789479796943
"Ovca2",1353.85097186491,0.250671778529076,0.100909873644247,2.4841154
7330649,0.0129873686373043,0.0413703175445449,0.798437649047719
"Iffo1",1883.54964511599,0.165668692826649,0.181283530226105,0.9138651
07437061,0.360787730138828,0.505973439875808,0.112865292835532
"Fau",15883.2684488095,-0.110866908010535,0.235374964440704,-0.4710225
16239038,0.637624655813963,0.751975173232967,-0.0316028305245867
"Asic3",31.7874447773765,-0.0188396075751826,0.680842875232299,-0.0276

710064253145,0.977924548366188,0.985964778119031,-0.000266291202813549
"Nop2",1429.19083992708,0.131968132204798,0.122826671711207,1.07442569
570789,0.282631930950093,0.426562225378505,0.112436456029372
"Ostm1",1498.85024617888,-0.182539329280957,0.277585790842193,-0.65759
6084897336,0.510797710968921,0.645005748881032,-0.080042774719202
"Bphl",6631.27784651938,1.87136414644779,0.414921729073366,4.510161833
71996,6.47781885315957e-06,0.000121097326940465,16.8776760063085
"Smg6",2543.63305759946,0.115775697111144,0.159820670361563,0.72441003
3127905,0.468814042058251,0.606949482192152,0.057807930533376
"Snx25",730.155580678966,0.702623371472261,0.233210870637449,3.0128242
716634,0.0025882874715481,0.0121437558691238,3.09922964765982
"Ccdc155",7.40512941303651,-2.56051991285824,1.14433626216608,-2.23755
900910761,0.0252498271740384,0.0690008591786127,-6.84589904375439
"Atg9b",114.181827202149,-2.20170995456737,0.544550543980521,-4.043169
1399543,5.2733550748221e-05,0.000601301361596591,-16.3287932256321
"Galnt18",1287.63576715267,0.526519936325062,0.182668956378553,2.88237
228023536,0.00394693107881243,0.0166404899589003,2.15658164179281
"Pdzk1",29545.7107949054,2.01842239008036,0.738892528959094,2.73168601
788921,0.0063011591072269,0.0237454886021011,7.54963186135385
"Wdr36",1228.36182409711,0.0308297907881455,0.135368229908373,0.227747
609679268,0.819842457718291,0.884533435181012,0.00378266009784193
"Snx10",1464.07040486309,-1.77119735598499,0.395360158615485,-4.479959
1395035,7.46573298433032e-06,0.000135034194553314,-15.7813374788374
"Afg1l",1178.17140428484,0.302364569740762,0.340094266591224,0.8890610
61720834,0.373970264133216,0.519164984697006,0.198210121835177
"Cd160",35.2246397093497,-1.10832369313999,0.658120312011403,-1.684074
58774923,0.0921672548216014,0.185456876626582,-1.86745104885281
"Spats2l",704.301564432486,0.00619142996491041,0.213786040926604,0.028
9608710562914,0.976895797867732,0.985334462293851,9.14730519733231e-05
"Edem2",2036.06153672515,0.0829723699311068,0.0848249889829685,0.97815
9513203902,0.327995437171281,0.472637279873301,0.062181737170482
"Kcnh2",395.451563314738,-1.42675461714077,0.276803716728689,-5.154391
11151536,2.54456365100649e-07,9.30842440287515e-06,-16.5283682946894
"1700066M21Rik",427.361085765755,0.0426498583669091,0.0960276894391301
,0.444141253590653,0.656940462126323,0.766456745484625,0.0113438819530
64
"Trpc4ap",4916.95332995678,-0.104758638758495,0.0944679447300955,-1.10
893318424362,0.267459007014688,0.409563734807471,-0.0935141342717277
"C87499",0.425166218019242,0,4.78471990595279,0,1,NA,NA
"Satb2",413.090229150263,1.48765013319057,0.480312954074208,3.09725174
091542,0.00195323924122189,0.00981553059931555,6.87858090585448
"Sesn1",1385.99282541262,-0.46541031878214,0.131972529846294,-3.526569
65297396,0.000420980416304626,0.00301663952852172,-2.70106082074107
"Tsr1",1006.58302130706,-0.338485258961427,0.116030066200854,-2.917220
25199477,0.00353166291134997,0.0153227332989546,-1.41433280292468
"Mlxip",2482.22849117791,-0.490236735024404,0.156969694115121,-3.12312
983590874,0.00178938792541217,0.00918183079951073,-2.29946945617763
"Txlng",1735.23401486376,-0.213251947036921,0.359335847382109,-0.59346
1377679233,0.552872423127014,0.682574970311563,-0.0814372743845281
"Zfp384",3137.65415778356,-0.0736708616821724,0.141813164077556,-0.519

492405104809,0.60341740781462,0.724784859087627,-0.0237132074765098
"Tcte2",120.553832573039,0.540576881543869,0.316665251121715,1.7070925
2003179,0.0878048571940326,0.178595775669648,0.931214095779719
"Plcl1",759.090502715952,0.339707813948733,0.217516875295986,1.5617538
3397943,0.118345991299551,0.224073619961025,0.508128364765419
"Sgsm2",1637.60552309485,0.209792725275935,0.175901176114872,1.1926738
0644988,0.232997147885963,0.370281065244153,0.208427588472399
"Arl5c",199.253879182923,-1.26455649240156,0.397160176000012,-3.183996
20308739,0.00145256917692955,0.00784707079166669,-6.13008297587356
"Ankrd35",149.967199259381,-2.25750817099814,0.528188173412428,-4.2740
604289059,1.91944977108521e-05,0.000279711746163075,-18.470369652181
"Camp",0.853483320595862,1.84040262611233,2.25509661219129,0.816108106
483296,0.414438307904568,NA,NA
"Fbxo25",947.519050078803,0.183111227322039,0.126855288802427,1.443465
45619575,0.148889396673934,0.266530063932094,0.24212215871654
"Lasp1",9092.28976331761,-0.38313860140113,0.092578296932054,-4.138535
85665252,3.4952925201967e-05,0.000439969099798549,-2.96120394480737
"Focad",431.847367524039,-0.318309823284914,0.211220337100915,-1.50700
36704507,0.131809716383166,0.242546795964418,-0.450905158769573
"Ncoa6",1694.55761886913,0.292616689011601,0.151263019422546,1.9344892
7655074,0.0530529992613408,0.12159719990568,0.616555459586938
"Pcp4l1",1116.82158418967,-0.474311822454531,0.183926782853365,-2.5788
078011057,0.0099141930891062,0.0335427619037641,-1.61025741775062
"Sbf2",2697.14901359,0.228042905410445,0.219820107950975,1.03740693941
113,0.299546245620984,0.443912191009953,0.185200143642556
"Gmds",653.502732280562,-0.0924869459147217,0.231151561896235,-0.40011
3869687973,0.689072649077613,0.79153637985833,-0.0216215461953316
"Rbm8a",3620.33317561466,0.32084570832699,0.18408520841666,1.742919548
43425,0.081347668854054,0.168413341188824,0.571533355232785
"Trp53inp2",9164.177772661,-0.0240715231014101,0.234253599843872,-0.10
275839140766,0.918154729232439,0.949818596698541,-0.00123930463734781
"Ttk",53.5499472727778,-0.622759628147005,0.422143226616294,-1.4752330
2254251,0.140149906213918,0.254554529236979,-0.852084761704331
"Pigu",1248.89128009902,0.0912092273887789,0.143608115166987,0.6351258
58192076,0.525346333552185,0.65797014208407,0.0381797927072975
"Setd1b",2038.47469779213,-0.14125020650464,0.176562096650636,-0.80000
299715591,0.423709060667377,0.566864228413099,-0.0801786259429605
"Rras",2703.99024707614,-0.845083864249892,0.168450229101095,-5.016816
354359,5.25347398181993e-07,1.6389157711604e-05,-9.31188661158285
"Mpp6",3604.50390630726,1.21278592350621,0.239201301468896,5.070147679
20866,3.97507135740985e-07,1.32488788592782e-05,13.6215234962741
"Gpr162",469.102458748163,0.314575327269015,0.395108521461064,0.796174
494302864,0.425930620173245,0.568862681810383,0.177457040269978
"Txnip",34459.4537177032,-0.0201813700153066,0.211245220550281,-0.0955
352739471946,0.92388967382102,0.953104226982415,-0.000969331667061563
"Upf3a",2639.79885061791,0.311842273511228,0.150364521047237,2.0739086
011737,0.0380878000879181,0.094442484103661,0.735874252911225
"Pmepa1",7992.76456715159,-0.968378380590659,0.292482914518077,-3.3108
8871357239,0.000930001861046273,0.00555790211755689,-5.02833820731615
"Foxf2",8.12940102826827,-3.77460111466863,1.38463747851708,-2.7260573

0614136,0.00640958690024278,0.0240473591265987,-14.0706941758681
"Hjv",12.5125747801014,-0.508349851275747,0.496470999314842,-1.0239265
7774029,0.305869955679669,0.450408005123646,-0.405460567783797
"Scaf1",4939.49956601872,-0.182448219518331,0.177684660820778,-1.02680
905980037,0.304510382471923,0.449220204088488,-0.146002742532617
"Higd1a",2582.12784699678,0.29552496834957,0.331622649478486,0.8911483
24200163,0.372849602991391,0.518162620037502,0.194297662488371
"Foxq1",2619.3781401074,-1.52423725513181,0.501615213991725,-3.0386583
4331922,0.0023763418615007,0.0113949260624006,-6.82033236662975
"Cdc16",2023.98812044821,0.262756416167383,0.124789450232675,2.1055979
9468194,0.0352392870026648,0.0888638926510553,0.636041154802031
"Fig4",839.470092522276,0.168110074974152,0.16107936894814,1.043647464
42653,0.29664852432388,0.440992141450672,0.137636463041723
"Egr1",3759.0033694541,-0.11832809272892,0.28539494840265,-0.414611728
032327,0.678426161252987,0.783460867810397,-0.0288760971623731
"Fcrla",97.4215486630421,-1.99081365011785,0.520435056015461,-3.825287
37660344,0.000130619627529565,0.00122487520347775,-13.348238922575
"Hdhd3",510.243925981665,0.858872885512832,0.293078811768374,2.9305185
1933813,0.00338396822548371,0.0148237727388185,3.61716301511513
"Poli",647.27226479942,0.489661947526749,0.204684419039377,2.392277584
31651,0.0167441732336238,0.0501810466814012,1.46512626821531
"Usp5",3948.58470484686,-0.455321752072791,0.203185174353784,-2.240920
15335719,0.0250312475563252,0.0685572783892723,-1.22030131765513
"Mllt6",8631.75869819701,-0.0802164887862398,0.154470454254876,-0.5192
99882771646,0.603551635114743,0.724786357979485,-0.025819950748705
"Cdc40",1443.00788551144,-0.0535898679065749,0.0964749613483788,-0.555
479547828556,0.578566695517543,0.703473025018774,-0.0188489363280633
"Spsb2",1824.69164645359,0.750001491016508,0.188887422741513,3.9706269
4874536,7.16837475512492e-05,0.000764600497677384,5.38212851412105
"Srcin1",2770.0515575073,0.999885663632757,0.478728194951677,2.0886291
5152446,0.036741118934041,0.0918433765353759,2.38739758409491
"Dennd2a",628.548232653104,-0.975080600777851,0.231509462969905,-4.211
83906812745,2.53299984148703e-05,0.000345373642722476,-7.7722540714064
6
"Tmem255b",42.3872672646524,-1.50697490507445,0.401159203092572,-3.756
55074957037,0.000172271349154914,0.00151030072091436,-9.78847486530953
"Abhd17c",4212.24917315802,1.15075414119424,0.289407215026631,3.976245
51650636,7.00118446909258e-05,0.000751288358714184,8.27820424695693
"Uqcrfs1",14254.7441042928,0.567452525428592,0.45592483853211,1.244618
58067561,0.213271987105706,0.347690401721326,0.599481160597379
"Olfml2b",332.11532268726,-1.85618213064007,0.318287623541032,-5.83177
602066195,5.48405009709232e-09,5.06767814185337e-07,-26.9057551619036
"Chmp4b",6833.55828651651,-0.246747902394044,0.13399982850693,-1.84140
461329981,0.0655622872679994,0.142679666839807,-0.480455980943564
"Nos1ap",218.189330617126,0.373397810146669,0.466421776145568,0.800558
269882608,0.423387417263967,0.566589420851945,0.21213489945941
"Cdk19",1570.64807060164,-0.328027965073109,0.0807024780270209,-4.0646
5790261457,4.81029640679385e-05,0.000562784417053428,-2.45450661805027
"Tfdp1",2155.09991365035,-0.51260994622162,0.116654457838145,-4.394259
3855509,1.11151037489524e-05,0.000185868175726479,-4.40356183497302

"Socs7",2688.74174542828,0.473993613832818,0.118293195946461,4.0069389
4556156,6.15107552849916e-05,0.00067797439601287,3.45844749442508
"Sv2a",916.630964596484,1.01247920922432,0.354872928592687,2.853075362
04436,0.00432983519891271,0.0178070294681291,4.07843026191129
"Polr2l",3424.57113047933,0.420893493710845,0.274419444863069,1.533759
73018554,0.12508877326504,0.233708403710401,0.611844907951079
"Otud7b",3191.7986982977,0.105874374121859,0.103021170526412,1.0276953
1331151,0.304093173110569,0.44884244430809,0.0848141989747301
"Slc19a3",410.155689566005,1.81226308789077,0.431538442768811,4.199540
31502509,2.67457408609885e-05,0.000356695249868367,14.3868839111181
"Tmco3",2479.28195851921,1.33739947875069,0.234775133238763,5.69651248
963651,1.22282937561425e-08,9.32213774251015e-07,18.5707328597712
"Prr3",1104.27054085243,0.179469007751535,0.174986885697907,1.02561404
550834,0.305073544285118,0.449767416407745,0.143400166894055
"Ptov1",5571.58526364793,0.162090611837034,0.159005376749393,1.0194033
3811795,0.308011522048377,0.452843361250133,0.128409640572661
"Mesd",2575.06652886177,-0.519403545133496,0.189532404676184,-2.740447
18643705,0.00613556417700409,0.0232655057026888,-1.95336426019693
"Dcun1d2",1423.17810816634,0.788222288104484,0.175746547055268,4.48499
444974362,7.29158326084066e-06,0.000132581880464041,7.03749304559223
"Parp12",1850.17123178281,-0.459530504282427,0.133137619342103,-3.4515
451496969,0.000557386491817936,0.00375773821588713,-2.5659898638189
"Gdf15",726.254699137377,0.572252436005296,0.483599029610628,1.1833200
6676285,0.236682322892261,0.374800420683702,0.561586569748114
"Rpf2",738.793508044641,0.708229063336147,0.128121808173165,5.52777917
697609,3.24309895172327e-08,1.94619501646229e-06,9.31295322968702
"Grtp1",1562.55795203988,1.66800420581577,0.356583164397922,4.67774245
212092,2.90050527039261e-06,6.44123596379443e-05,16.0965825660563
"Tbkbp1",1196.73884462276,-0.850790606422439,0.20515313083445,-4.14710
028046802,3.3671260980887e-05,0.000425879091789407,-6.60328799012841
"Jarid2",2505.98057366386,0.410594857089843,0.178848453325189,2.295769
68352801,0.0216890537186507,0.0613155240548429,1.14626678631571
"Tbc1d17",2910.72405777739,0.429509243309038,0.170693223359057,2.51626
417766776,0.0118606243412932,0.0386722086841845,1.3970363933457
"C1s1",5253.66611137314,-1.48660286663024,0.23276658948236,-6.38666773
412902,1.69539455791257e-10,3.71424753822238e-08,-25.4335520314867
"Mfsd4b1",3852.87524073167,1.86760428120216,0.482826760593325,3.868062
90294916,0.000109703368870141,0.00106629924537887,12.7810643351888
"1700003F12Rik",12.4890423932106,-1.22561378564171,0.694046172813556,-
1.76589661271852,0.0774131955954256,0.162057385411167,-2.2303778200803
9
"Fchsd1",841.827221333008,-0.265445465123178,0.212816277240817,-1.2472
9869615569,0.212287995358015,0.346607882556571,-0.281255704794462
"Armc10",1555.20168903153,-0.2127614347408,0.119305657347801,-1.783330
64391537,0.0745324959047305,0.157480993189254,-0.393278981738408
"Car14",2595.70622724939,2.70554768958031,0.72570085845337,3.728185874
4751,0.000192863112351596,0.00164642949414686,17.3402509065694
"C1rl",1091.14070543031,0.898706232985698,0.396546186640742,2.26633432
185769,0.0234309215224363,0.0650236418793713,2.45616804771319
"Mfsd4b5",13490.2740312235,2.17614078898875,0.781733328408816,2.783738

02152992,0.00537364182575852,0.0210012564278692,8.40680836883612
"Rgs4",416.169854255506,-0.961673941920007,0.289899234532371,-3.317269
68327894,0.000909018275873273,0.00547714411093235,-5.00760110564073
"Cbfa2t2",988.508115521959,-0.0860320280481735,0.12840727164931,-0.669
993427499444,0.502861979999885,0.637710756815464,-0.0387032678313742
"Osbpl7",1050.85585650029,-0.122247363367321,0.166742835562646,-0.7331
49121249004,0.463467483095218,0.60251166438802,-0.0619364131847161
"Zfp280d",2252.12401628064,0.868268255861698,0.364714257579972,2.38068
086951964,0.0172806753659421,0.0514253065148257,2.57669448124584
"Mc3r",0.21077022159O303,-0.0573622969198357,4.7847199059595,-0.011988
6426054719,0.990434676294085,NA,NA
"Ubn2",5973.09621269254,0.49065127314112,0.260209574413831,1.885600382
86985,0.0593488257715725,0.132461470340444,0.991833623067153
"Atf5",2988.40264400401,-2.06735799193053,0.390613918794999,-5.2925865
988291,1.20598330159324e-07,5.28410391336293e-06,-25.1200690055967
"Tmc3",30.6942338043915,-0.173067417581201,0.381160007990479,-0.454054
501923307,0.649789599442056,0.761348333028136,-0.0471893054355797
"Srd5a2",130.995398439771,1.81646222928494,0.507273719391022,3.5808325
167438,0.000342501157407038,0.0025694834810O132,10.8334722496024
"Pcid2",1197.21671202556,-0.000887169710988346,0.121085987792413,-0.00
732677436227624,0.994154132158512,0.996250456664318,-3.33273330336224e
-06
"BC028528",371.42238057291,-0.890211709428875,0.231776884626532,-3.840
81316332858,0.000122627423598922,0.00116519956400184,-6.01325805545352
"Inip",762.853031791502,-0.607207103495133,0.133936341172929,-4.533550
02964544,5.80005203177191e-06,0.000110980779701167,-5.52932110740949
"Cul7",2596.89027656406,-0.0118466068037704,0.137001537586162,-0.08647
0612027401,0.931092316753511,0.957120165603645,-0.000519193305295311
"Ranbp9",2505.54730960111,-0.204593940478435,0.167028019359227,-1.2249
0790026322,0.220609923453797,0.356498657066764,-0.211023265393042
"Ciart",172.185617510338,2.12768967641957,0.383667729779739,5.54565711
752995,2.92852224438699e-08,1.80697236459043e-06,28.1362658086114
"Fndc4",448.7812308951,-1.03337308158286,0.359019825499544,-2.87831759
748926,0.00399802409855226,0.0167796341480517,-4.22400483492885
"Reep2",114.754987161768,-0.644100101646648,0.310870128395542,-2.07192
664335736,0.0382722828232962,0.0948209855101526,-1.51734817199072
"Sp6",152.105347202796,-0.576361275533072,0.172026206012371,-3.3504271
7556431,0.000806870226760109,0.00498964658463958,-3.054939654448
"Efl1",838.473773524308,0.0751400583643799,0.0799653397637603,0.939657
83908834,0.347393097805255,0.49218650058181,0.0532666046409568
"Ift172",2304.16460462512,0.22827666421716,0.15893108320887,1.43632484
979137,0.150909926172572,0.269280472986238,0.299499392537558
"Cyp24a1",2531.47530687928,0.903938568314998,0.684760494907176,1.32007
990390499,0.186808341550776,0.315576940179953,1.04256004198875
"Rad9b",356.204916397598,-0.128348535190168,0.286886405713687,-0.44738
4513988648,0.654597455139813,0.764729849030322,-0.0344272672102058
"Saxo2",140.437345155111,0.275530140727892,0.272646276466389,1.0105773
1027498,0.312218780059689,0.457339176140321,0.215555490484947
"Bpifb5",0.681740913676252,-2.97059131358217,2.67321323191816,-1.11124
368161631,0.266463473660649,NA,NA

"Susd4",635.542765482343,-0.227382288596338,0.295807921214717,-0.76868
2216698617,0.44208198441427,0.583596109147558,-0.122455851404193
"Susd1",509.992310583965,-0.263844851027697,0.271712466300299,-0.97104
4334550677,0.331526201697483,0.476352091429442,-0.195666816552147
"Sct",42.9074329997433,-1.32380025104522,0.530903835956191,-2.49348405
754307,0.0126496264736705,0.0404729926130008,-4.24558675937626
"Pptc7",2485.29942664165,0.585233139497985,0.0878031439207336,6.665286
83786446,2.6414894151488e-11,8.72945095196124e-09,10.8599158341528
"Pln",274.634863270609,-0.308113521280567,0.457301510705894,-0.6737644
94687456,0.500461051137995,0.635617699745884,-0.139624106276643
"Akap12",2280.46349046441,-1.54058243865782,0.217068491583243,-7.09721
815184326,1.27293048188323e-12,8.99945481602099e-10,-32.0883093053325
"Colec10",0.0983898136351888,-0.544048378937457,4.78471990596623,-0.11
3705376621746,0.90947135024012,NA,NA
"Tctn1",2095.346119703,0.287620538549594,0.194724241359872,1.477065908
90263,0.13965797079817,0.253826539394363,0.394357717202181
"Cep85l",757.961567294726,-0.553599552676183,0.206303527451951,-2.6834
226225488,0.00728728362730377,0.0265733039396239,-2.00837512876251
"Shoc1",6.20120816906198,2.27351109481886,0.701753382750221,3.23975794
161306,0.00119631208839101,0.00673771971628047,11.3676321649163
"Capn8",1.60744160601487,-1.91159461705982,1.37935222358693,-1.3858640
196257,0.165788429312029,0.288645664320559,-2.37526224869579
"Atp6v0a4",5696.39514849112,1.53618400471732,0.53296200947067,2.882351
79509893,0.00394718771463298,0.0166404899589003,6.29208125738246
"Slc35f1",187.353932892123,1.31227990026854,0.271608998884619,4.831503
76334182,1.35505656813041e-06,3.47643328492193e-05,13.4730709360229
"Ripor1",4119.48249710972,-0.165425640211401,0.199881466300402,-0.8276
18704591562,0.407886471924777,0.551561857936702,-0.0984284682499013
"Samd10",662.0867414459,0.272809030884168,0.187977310880338,1.45128701
760093,0.14669995480821,0.263444196343503,0.363903707056962
"Gng10",940.81933762844,-0.974935295924941,0.168272295036586,-5.793795
67927668,6.8813168974653e-09,5.96319630519015e-07,-13.9732494248517
"Dock10",2177.50685345592,-0.957211825380637,0.359502991362461,-2.6625
9766505127,0.00775400642174646,0.0278071762389576,-3.42917418010217
"Phrf1",2720.32735944903,0.0533598463712662,0.162024510862255,0.329331
939268312,0.741904805320765,0.831876263309317,0.00982203076082354
"Mcl1",11584.2275315273,-0.387486091928934,0.154835624089958,-2.502564
21418761,0.012329725631224,0.0397429311221036,-1.24976791572662
"Nfe2l1",11961.7779371876,-0.027400922313512,0.294837195190942,-0.0929
357718783297,0.925954586865094,0.954645861606155,-0.0012718092282268
"Rassf7",1279.97940866416,0.0506089492981729,0.190908734401444,0.26509
4991367718,0.790936285662502,0.865511039277554,0.00730971116547785
"Ensa",4370.09245753496,-0.0231945580471337,0.0825547532945625,-0.2809
59691858972,0.778741316246583,0.856964866170182,-0.00358027388368696
"Med30",785.668918455237,-0.10867290344553,0.242703100367424,-0.447760
672529569,0.654325928912718,0.764595886488084,-0.0291686590289939
"Tm6sf1",1310.85682912095,-0.822453021668276,0.252269871932953,-3.2602
1104052751,0.00111329342816838,0.00639381303753375,-4.15538177133746
"Nepn",246.726783022569,1.27774942431084,0.721918141896074,1.769936714
64595,0.0767376837937555,0.160920970302649,2.3342461888405

"Polr3k",1308.78587444849,-0.659035004238189,0.253854291851767,-2.5961
1527317812,0.00942844278180879,0.0323313009777889,-2.2616232406139
"Zkscan16",3.86937843334453,-0.185383519191015,0.846208206562161,-0.21
9075539274384,0.826591204783812,0.889354117143514,-0.0217380326755862
"Degs1",4800.84710908261,0.202952556445685,0.173497690647178,1.1697709
3867149,0.242093161677426,0.38076995163382,0.195962847610732
"Lrrc56",459.953658218688,0.580448328289591,0.142050213925029,4.086219
31816264,4.38459340974412e-05,0.000523199524499332,4.3856050124212
"Akr1d1",1145.49098034789,2.07110092930869,1.05378798636074,1.96538673
444289,0.0493694936754004,0.115249872075022,4.47492982955526
"Ctss",8206.19601630151,-1.35498370400677,0.431977921059621,-3.1366966
6422548,0.00170862801957014,0.00889344077030929,-6.39883095863539
"Pold1",1086.42570088339,-0.148225280149276,0.357018987342399,-0.41517
4781746608,0.678013958453181,0.783170388739344,-0.036226999079851
"Ramac",2909.30318679287,0.137542597915371,0.191549118999639,0.7180539
31198872,0.472724034100684,0.610478294069122,0.0678789969123261
"Creb3l2",3916.36127127914,-0.116289391076016,0.132836358122846,-0.875
433448487594,0.38133810677548,0.526283296001437,-0.0746479772319849
"Rnh1",6105.11497882535,0.123959860951872,0.106738712258677,1.16133929
601343,0.245503944921291,0.384652134925474,0.118433221964707
"Sycp2l",0.783257822430065,-1.02493380893303,2.24582104347129,-0.45637
3766695506,0.648121226704803,NA,NA
"Ric1",1587.25658973193,-0.726025358059405,0.226511282067665,-3.205250
31438621,0.00134945069317709,0.00740977302809772,-3.56112205182915
"Fsd2",103.106756260122,-3.71090407209028,1.04236655889717,-3.56007590
651838,0.000370747611539369,0.00273095463913661,-21.9058528431842
"Herc1",3401.70517447519,-0.0031720665181911,0.204179594031241,-0.0155
356686511275,0.987604828453759,0.99203126810527,-2.53786001000946e-05
"Dgki",4.69363868921758,-1.95723834789713,1.07068787355908,-1.82801953
420006,0.0675466199957793,0.1460137800122,-3.7658328164985
"Lpar1",753.921187405787,-1.12464400437611,0.25559481489776,-4.4001049
2711277,1.08198547405157e-05,0.000181866834250497,-9.68569937770466
"Mybpc2",346.332885794451,-1.24638216474675,0.605836632249659,-2.05729
08576336,0.0396582562484921,0.0972585748298112,-2.90454672052803
"Arfrp1",2604.82025805578,-0.136567934924065,0.105561855464236,-1.2937
2427496155,0.195760677299552,0.326457550973885,-0.152881705532473
"Ucn",0.184866368983273,-0.551580421051654,4.78471990596623,-0.1152795
63253822,0.908223536518323,NA,NA
"Scube3",663.352790814889,0.119291921799612,0.41858798216478,0.2849864
94792991,0.775654488684589,0.85488164718626,0.0187040481065105
"Trps1",2135.45040250141,0.933926420645224,0.265131244745891,3.5225060
7634089,0.000427487242016456,0.00305316712265086,5.4089056813134
"Pak1ip1",2320.4698032633,0.0497410707124043,0.163295033116025,0.30460
8595639661,0.760664272592996,0.845188726921585,0.00836621585741892
"Rtel1",1073.85903585464,-0.00873149064947465,0.172292949843578,-0.050
6781656324406,0.959581975588976,0.975491624610733,-0.00021666063052953
6
"Atp5j2",15062.4764044039,0.894462955735555,0.312351950391743,2.863638
13196538,0.0041880616357151,0.01736682810999,3.62543127965558
"Hoxb4",5218.82559718815,0.533242725915356,0.347455694598518,1.5347071

1289252,0.124855787877007,0.233407301248886,0.775852316894618
"Josd2",2921.5749263376,0.607825891067351,0.22441051232025,2.708544643
39861,0.00675790195897877,0.0250934245660194,2.23992924004552
"Mapkap1",2181.55241733177,0.244957919203443,0.127455809146924,1.92190
470440675,0.0546177497763942,0.124248849042135,0.510852116744665
"Taf5l",756.599378171548,-0.393545491406293,0.13143841737447,-2.994143
56371224,0.00275216412923489,0.0126919811238935,-1.71852850638714
"Hoxb5",1701.00617257727,0.0551112493576908,0.290065825632103,0.189995
664734354,0.849312527697742,0.903924435865205,0.00556676033324956
"Dsel",442.013083532651,-0.831264255906335,0.216900184294422,-3.832473
71877735,0.000126861155496543,0.00119841996602306,-5.5917079014042
"Aspdh",2601.40387459808,1.84893346431906,1.00810364785968,1.834070800
40287,0.0666434851677415,0.144540008209191,3.5762051400346
"Gmeb2",1232.09874494708,0.0307300038519088,0.125239982290714,0.245368
957179958,0.80617076300923,0.876804589613832,0.00404010827898955
"Golga4",9552.34021358953,0.362840917142288,0.125987371585102,2.879978
46591471,0.00397702333737971,0.0167206020153349,1.48442343480345
"Mindy1",4370.40469576164,0.804132501188551,0.189119403845336,4.251983
05852412,2.11885892712252e-05,0.000300461900807526,6.52166704657344
"Atp5l",15353.9038717412,0.653755028770685,0.384514174970595,1.7002104
7681969,0.0890913422980458,0.180647149763654,1.11871188074747
"Pbx3",599.356578737075,0.160599912025215,0.184237365986045,0.87170108
6072729,0.383371467620976,0.528117625807107,0.102532804609986
"Hoxb7",1427.13989757294,0.349741771970533,0.239055932655187,1.4630123
0881811,0.143464010908419,0.259125640082663,0.47230606128288
"Bud31",3385.71633382734,0.377793246629635,0.15425481314166,2.44915046
043127,0.0143193618023855,0.0447362468230913,1.17379274252238
"Pkhd1l1",31.9368521078427,-3.84289505718635,0.833722313247,-4.6093225
479595,4.0398317271761e-06,8.43740065777616e-05,-36.0473209052952
"Pakap",8667.24792303253,-0.238278982954432,0.144215672858481,-1.65224
055216423,0.0984855226835083,0.194990934330122,-0.389539008434149
"Mboat1",637.865033930692,-0.363801136130937,0.220880989775789,-1.6470
4593410335,0.0995486047182256,0.19665640271489,-0.591648776940213
"Wdr26",7701.74481332985,0.0187658829024479,0.148032375213511,0.126768
775245154,0.899123410055398,0.938238319905358,0.00119634924026263
"Nudcd1",749.342448860316,0.0273265516080377,0.154932848514939,0.17637
6745602807,0.859997972583285,0.911168856677111,0.00254210836785044
"Shank1",667.99796912928,0.00694095631285299,0.335234074528056,0.02070
4805508284,0.983481135601443,0.989469307634517,7.34806594180716e-05
"Mvb12b",1640.18961238869,0.692174200087262,0.143490126530696,4.823845
49252723,1.40816516286463e-06,3.58907246346857e-05,7.08442485391018
"Angptl6",102.875119258638,-0.306739610766653,0.269635159280497,-1.137
60984133214,0.25528343538601,0.396015309344929,-0.284133640239488
"Nlrp6",6319.77940099186,2.18829601825922,0.838378571590385,2.61015261
17349,0.00905018383389588,0.0312984186579109,7.58066816096844
"Ptk6",18.9678471171426,-0.827684305326425,0.409024369876601,-2.023557
43638485,0.0430157082206654,0.103673705672007,-1.87595207101882
"Elovl3",156.478350900617,-3.1575149372799,1.53004101047865,-2.0636799
3776332,0.0390480727599086,0.0962405919191881,-7.39143952635627
"Ttll6",16.1901834053048,-0.131990316599307,0.726478072603918,-0.18168

5203692679,0.855829778450111,0.908332100605316,-0.0126902377218298
"Nup205",1107.72903002377,-0.496596933475546,0.141045977557773,-3.5208
159925875,0.000430221049030644,0.00306931943432107,-2.87345855042263
"Trhr",4.27788571912298,3.68829946709194,1.13790535017251,3.2413060247
3465,0.00118983378549088,0.0067172824668O935,18.4528263200004
"Abcf1",5805.48787035544,0.340593755405623,0.222333666564224,1.5319036
5035085,0.125546212598989,0.234348886828244,0.494182569609981
"Alpk3",12.2602743598349,0.185572942255678,0.579799107209143,0.3200642
08358188,0.748919657197858,0.836582999401546,0.0331116949532574
"Ptpn3",2228.46001494526,0.168379841800638,0.140519298535181,1.1982684
4821946,0.230812525676564,0.367618250746744,0.168499432126271
"Lmx1b",310.922767876399,-0.413256126728682,0.522586887101991,-0.79078
9315477082,0.429066945860016,0.571678782997034,-0.231083740022343
"Gabpb2",2462.90929956807,-0.288456177193199,0.150650005921211,-1.9147
4388221437,0.0555251743160464,0.125901831470435,-0.597754118273472
"9130409I23Rik",1.03936123015701,-2.6299257009966,2.31651778144754,-1.
13529268890533,0.256252704485828,NA,NA
"Kdm3b",2798.31875118997,-0.226181216768528,0.136300337903398,-1.65943
254615284,0.0970286625537457,0.192786251642618,-0.372333459255064
"Ascc3",792.094720687994,-0.599318594309377,0.22921945409761,-2.614606
14967771,0.00893303976339679,0.0310122302508766,-2.0816574002918
"Vill",3707.98894093822,1.00218314780772,0.252030583223017,3.976434665
15694,6.9956207220301e-05,0.000751104696245281,7.20967099682682
"Ephx1",3251.37225748552,-0.357176444158399,0.212831305732301,-1.67821
384607608,0.0933053596900176,0.187052285086372,-0.598758840530694
"Sema6c",581.768117944382,-0.61714053934478,0.305565253468169,-2.01966
857271967,0.0434177770533171,0.10448775820607,-1.39392630176985
"Smurf1",1892.27387466705,-0.805942525189802,0.189083152508324,-4.2623
7089078743,2.02269364977876e-05,0.000290844253564796,-6.56256645058213
"Stap2",2284.63762187267,-0.284676909076453,0.249907199537658,-1.13913
048364801,0.254648734740561,0.395313776271613,-0.26420165309149
"1700028J19Rik",202.844097067776,2.22109695317224,0.411549820289655,5.
39690905856549,6.77987303384574e-08,3.4070609385032e-06,27.96285665172
08
"Cnot4",1662.63808880971,0.326031936655555,0.192318531561665,1.6952705
1817685,0.0900241148606318,0.182008522558337,0.5554611861736
"Scgb3a2",1.56806805918591,-2.26857709206451,1.75496893319397,-1.29265
940220138,0.196128884826235,0.326903829401772,-2.53647166176897
"Lefty1",61.4053439668967,-1.53130290206564,0.383656219366809,-3.99134
127055968,6.57006557052554e-05,0.000714462568288383,-11.0927275209301
"Zscan2",487.530647916381,0.0748445909068649,0.21657053046106,0.345589
913583937,0.729650927235443,0.822592297151585,0.0146167434895317
"Scgb1c1",12.7334903808324,1.95068512982564,0.598850870442787,3.257380
47000469,0.00112445604642245,0.00643706517708309,9.84253794074601
"Ost4",4075.00970238856,0.0888569740444664,0.197083857906299,0.4508587
10542963,0.652091383660162,0.763128131481054,0.0240206463151333
"Six3",0.657037197293791,-1.46313384378166,2.39093360519449,-0.6119508
46565913,0.540570278965137,NA,NA
"Sde2",938.667043287066,-0.480867009566084,0.146992317027005,-3.271375
12552945,0.00107025842469208,0.00620698951952592,-2.44380426577287

"Rap1gap2",1461.79736131763,0.416463745606308,0.187323818749314,2.2232
2899664799,0.0262003625134347,0.0710507188159874,1.10128061483537
"Gngt2",915.936510696823,-0.818546188820159,0.226206720018629,-3.61857
59147772,0.000296228607637796,0.00228656587162381,-4.97733725344951
"Trmt112",2357.37463003947,0.3771589610187,0.178590446057941,2.1118652
7243645,0.0346980067853236,0.0878625631558753,0.917243603565731
"Ctnnal1",2236.04687666352,-0.168294618188404,0.15294724552658,-1.1003
4422397726,0.271182170401146,0.413674868050863,-0.148549444800818
"Hace1",589.638192524442,-0.425538188126266,0.129312682091617,-3.29076
917471078,0.000999138608152099,0.0058696006573515,-2.18640188212901
"Abitram",464.215731286303,-0.558577545420862,0.21370603647038,-2.6137
6587506119,0.0089550378027304,0.0310575110419134,-1.93933353425452
"Tmem214",3471.79287218585,0.198101327000012,0.113370714032178,1.74737
654861894,0.0805720426570788,0.167180343484912,0.354340307126157
"Ralgps1",721.707238845946,0.303343290106797,0.123593939052378,2.45435
409238184,0.0141137927905815,0.0442357710706894,0.945891574237517
"Agbl3",663.677806934995,0.421760386178454,0.201647030685956,2.0915774
695205,0.0364763297284182,0.0913336942397199,1.00937193280402
"Vars2",1300.06505267387,0.181791469700272,0.154531057726764,1.1764073
3438652,0.23943214228775,0.377645034001501,0.177028640096051
"Gcnt1",14567.9418178233,1.85487241469271,0.60320284705528,3.075039223
94903,0.00210474871366577,0.010381581183672,8.47254168976148
"Kif16b",1605.88685087359,0.604244616240445,0.188272461623433,3.209415
81700358,0.00133004988181051,0.00732165798857746,2.97102151730478
"Phb",11428.8379427731,0.250556742514506,0.327399490850812,0.765293622
978415,0.444096720514724,0.58538384644376,0.134170003445266
"Ythdf1",2023.23914629365,-0.130498027404889,0.101267069603727,-1.2886
5215430393,0.197519043118097,0.328599277923746,-0.145233378007991
"Itpkb",3236.01682818888,-0.630253466286885,0.210572829337444,-2.99304
268394903,0.00276211115986738,0.0127198024079108,-2.75080128850394
"Baiap2l1",702.631232937855,0.612053419536957,0.240306185419055,2.5469
7322280587,0.0108661770782244,0.036148903032114,2.03208386821541
"Garnl3",289.030971229366,-1.0651915599889,0.365884989446332,-2.911274
28212012,0.00359957880321995,0.015524143902701,-4.43690503363967
"Pi4kb",1976.14792365819,-0.214722222501458,0.175078160366509,-1.22643
636448977,0.220034508554277,0.355799705406477,-0.221891224573014
"Zcchc2",1506.57417157748,-0.0929747124180129,0.120311724045074,-0.772
781814540208,0.43965151764605,0.581489980400399,-0.0504073130593584
"Bpgm",1492.05099589261,0.480496434980456,0.164598473383793,2.91920347
195497,0.00350927084559586,0.0152569461621923,2.00978223763793
"Zfhx3",2972.31130075393,-0.328370969530819,0.240137541326274,-1.36742
871488245,0.171490966948502,0.296456323571406,-0.399251609305521
"Rnf146",1621.11777201374,-0.400724966026475,0.143721722402441,-2.7882
0041485718,0.00530017436946179,0.0208016907922234,-1.55189599415681
"Nipal2",795.443986718756,0.459004364150875,0.234488349010042,1.957471
94301419,0.0502920073268713,0.116946160865753,0.98504246630679
"Mrps34",3263.45380009411,0.534502469699866,0.267392977819761,1.998939
81531614,0.0456148661230174,0.108291569422451,1.18816049135879
"Shfl",2213.50031350978,-0.390494656484823,0.296734559514668,-1.315972
95954845,0.188183118658523,0.31718485888692,-0.448393503824356

"Man2a2",4503.04848367828,-0.797306024998134,0.213241726821847,-3.7389
775297789,0.000184770239901783,0.00159197973380688,-5.13686485735987
"Ctu1",386.328654291349,-0.317304557377891,0.12937768685359,-2.4525446
7825636,0.014184976270879,0.0443946496516209,-0.988288298957328
"Fam117a",678.118334024477,-0.500572756860781,0.200748042983352,-2.493
5374184559,0.0126477252660838,0.0404729926130008,-1.60539708838596
"Irs2",1515.37054514661,-1.19094466965519,0.399530958204791,-2.9808570
404818,0.00287442944728803,0.013082545602993,-5.16450337497134
"Zfp653",1110.07886329764,0.444886735193849,0.217555930897246,2.044930
39265371,0.0408617210554634,0.0995528973059385,1.02638312506656
"Rpl12",1334.50515720331,-0.444160826438379,0.15710828934207,-2.827099
88313418,0.00469716742495066,0.018922597200762,-1.76216297525595
"Pogz",2446.77464720825,0.165435150641214,0.181047167903873,0.91376823
2646709,0.360838642081067,0.505973439875808,0.112706187293629
"Ccdc68",534.76391204965,0.367561410789981,0.288926939449165,1.2721603
9975619,0.203316134512628,0.335499152905147,0.401426992861738
"Kat7",1478.95042934327,-0.75127110071267l,0.209484891655512,-3.586278
20257367,0.000335431105971196,0.00252423116767999,-4.49396755707822
"Plcl2",1398.29680660559,0.13884985990818,0.316089321579295,0.43927412
4207791,0.660462921128476,0.769369460877227,0.0364042091124175
"Dido1",3259.23977214681,-0.0273679881680461,0.167478565900521,-0.1634
11885102373,0.87019414570669,0.918523389276158,-0.00232594811548415
"Soga3",2.53300372305738,-1.23271184675093,2.4359240012848,-0.50605513
3945376,0.612817945691438,0.732107365056777,-0.384394195733663
"E330034G19Rik",10.2756883510821,-0.0999921892724225,0.60963640658995,
-0.164019386295738,0.869715882430772,0.918321253960533,-0.008520134436
70045
"Rccd1",540.712022886326,-0.524244784129567,0.279691178123972,-1.87437
010936826,0.0608794303785248,0.135095543015473,-1.0494190672748
"Tcfl5",2.40834073075253,-3.29059518255977,1.42327387604673,-2.3119901
502722,0.0207782261164767,0.0591609014046527,-9.30413946626304
"Sccpdh",1312.70184001024,-0.48664197024342,0.344682706145524,-1.41185
490762034,0.157992687271579,0.278857736618199,-0.62146784682176
"Prc1",219.796149363231,-0.791379459138505,0.419951784587513,-1.884453
14005706,0.0595037076311749,0.132761562122972,-1.59795381990583
"Cnst",1605.83769846146,-0.646866524856439,0.362255359189437,-1.785664
4724424,0.0741536010576464,0.15695255247742,-1.19787502030358
"Supt3",570.627574007611,0.0270005809143579,0.278162620330297,0.097067
6106023762,0.922672702361137,0.952320744184557,0.00131906976853138
"Edc3",932.015909897666,-0.303413012958368,0.173511662244978,-1.748660
63198671,0.0803496988859234,0.166825516864842,-0.543354093474719
"Slco4a1",1344.00540592376,0.329187827969401,0.585561774391465,0.56217
438085248,0.573997214601196,0.699881038727666,0.117468998558866
"Ube2l3",5235.54837473174,-0.105213292864972,0.141786195160111,-0.7420
55971994739,0.458053423984845,0.597839591730136,-0.0541251690844322
"Pdk2",7225.01570296628,0.781601060024968,0.349480121938626,2.23646785
885645,0.0253211404129523,0.0691569681708565,2.08795067053856
"Klk1b16",13.9093555761531,2.90855481681353,0.879571110149383,3.306787
57322936,0.000943724281657216,0.00562128859737518,15.0697877939225
"Lmtk2",2778.77618547158,0.494463440898071,0.209438212327132,2.3609036

546098,0.0182304661816662,0.0535005462989354,1.44782031082317
"Cldnd2",1.37449259637713,0.514716225059449,1.69704975952796,0.3033006
08700253,0.761660782348499,0.845664505366508,0.0862832004307886
"Rabggtb",2756.53707170819,0.16640151513661,0.143415033958928,1.160279
4389343,0.245935052998053,0.385057067670765,0.15880757032442
"Ppp1r9b",5382.07996895297,-0.903855794378656,0.144266505292529,-6.265
1811835735,3.72392162005803e-10,7.18901225226649e-08,-14.8667348591506
"Rbbp8nl",32.4925776288708,-1.78487098259113,0.584640502871953,-3.0529
3761520666,0.0022661304388789,0.0110077257840709,-8.04826510716963
"Bloc1s5",840.528883801696,-0.218226164126615,0.256225350103826,-0.851
696227708098,0.394382714880595,0.538718941764175,-0.134986257140768
"Tspyl5",149.507117488595,0.257724315069049,0.27621425462101 2,0.933059
430342098,0.350789284352155,0.495702379400227,0.180865660009213
"Cfap157",20.9052455286945,-2.40407850835515,0.843905511076344,-2.8487
5318006741,0.00438909159624141,0.0179722454385033,-9.66181514060697
"Cables2",1264.27549747547,-0.212979846025953,0.204054445395916,-1.043
7402900619,0.296605563740165,0.440961897202114,-0.174387218130652
"Txndc5",5830.32285900614,-0.131673546916633,0.112739445193752,-1.1679
4567057112,0.242828680395807,0.381674615909508,-0.12682622548160 7
"Hils1",3.3593633056538,-0.379184332893027,1.19085098423625,-0.3184145
9419561,0.750170469356902,0.837493490496471,-0.0672452275217218
"Asb17",0.0824412674207658,-0.0705096924244733,4.78471990596623,-0.014
7364305142611,0.988242455162279,NA,NA
"Ube3c",1575.68131201899,-0.215713055677409,0.133530685374098,-1.61545
681483675,0.106211805141463,0.206164039487132,-0.34062884474389
"Rps21",11462.8665424166,-0.320145791503555,0.223499823481502,-1.43242
078009986,0.152023435019091,0.270919451119238,-0.418089187842396
"Bmp6",1996.24433441765,0.230463248143283,0.23037026893839,1.000403607
65876,0.317115224804515,0.462288796503808,0.177817487260679
"Tlr4",326.509114542084,-1.33026841410234,0.21809966469642,-6.09936019
825608,1.06493852413563e-09,1.63497412154303e-07,-20.787397701696
"Cpq",3401.80896745327,0.254421050986312,0.163524288105262,1.555860930
10562,0.11974116564032,0.226188068945453,0.378168514749864
"Siglecf",21.9106313458096,-2.05360942060328,0.644970027934696,-3.1840
385314947,0.00145235678377544,0.00784707079166669,-9.95510775831454
"Timm8b",2907.99741972567,0.755205086457998,0.284413829217295,2.655303
67681599,0.0079237041060409,0.0282753262554146,2.69288440764832
"Mtg1",865.111778681532,0.426873260725309,0.323273701639638,1.32047011
111704,0.186678108764171,0.315384261735015,0.492596164171466
"Ttc16",74.5850919904617,0.24720211590379,0.335740876604395,0.73628840
9096725,0.461555198595378,0.600841451609873,0.125930736891224
"Arhgap18",2594.95030725194,0.0716182735218178,0.203769676069853,0.351
466787910419,0.725238184547264,0.819133066228497,0.0142884711030227
"Tsga13",1.09384289378068,0.104615038766228,2.05562113860912,0.0508921
789143561,0.959411437757651,NA,NA
"Tasp1",428.947205782717,-0.0199369652664871,0.179256337340754,-0.1112
20420779815,0.911441558654133,0.94543211739311,-0.00111872669849926
"St6galnac5",205.323286186315,-0.40600620057593,0.451698405917606,-0.8
9884355414349,0.368735997305099,0.513773208151926,-0.270389305528769
"Adrm1",4165.30101098365,0.246704514577087,0.202160618203174,1.2203391

3810625,0.222336338875385,0.358214669921687,0.253272488320047
"Arpin",1087.78478621231,-0.327400656659867,0.196541859235011,-1.66580
624572389,0.095752011752489,0.190877489535838,-0.542215912495868
"Usp6nl",1339.91317576234,0.162285254994935,0.149848795850042,1.082993
38726311,0.278811358210544,0.422562290119642,0.139795506456628
"Pigk",2453.00481214545,0.226600242277793,0.189334247500785,1.19682648
685549,0.231374192167127,0.368332299059002,0.226321469977996
"Foxred1",1995.90729131818,0.0733997217249888,0.258320771680868,0.2841
41771671647,0.776301736441906,0.855256597194275,0.0114763211089044
"Osbpl2",1949.49165343614,-0.289229150051335,0.239372085310015,-1.2082
8270212356,0.226938539749213,0.363138912435794,-0.292980405571352
"Eme1",38.7710318139297,-0.587105347623116,0.437697046490337,-1.341350
9465755,0.179806535972895,0.307208888748642,-0.692917784720323
"Myo16",4.73223997900175,-1.97291026078308,0.887518412752768,-2.222951
35789218,0.0262190801183639,0.0710915900636712,-5.21595298518018
"Ak5",298.705331470068,-1.19381771906306,0.439109324184444,-2.71872550
481668,0.0065533967640483,0.0244785689857115,-4.42901273724925
"Hrh3",23.8506989363718,-1.50612458579073,0.921771533953411,-1.6339456
4738952,0.102270263763506,0.200720909374821,-2.41859487346052
"Anpep",4604.94004542042,1.23297558434272,0.295096394428421,4.17821297
590198,2.93808470968492e-05,0.000384217140640084,9.69649321054168
"Echdc3",1079.56626489552,0.212139022392486,0.45296512009605,0.4683341
2327091,0.639545669784118,0.753465949815754,0.0600505009457134
"Atpsckmt",1042.04517126262,0.16540916820645,0.188585236085855,0.87710
5608262709,0.380429278804438,0.525289290994189,0.106491437721003
"Psmd7",4122.82565356574,-0.104126153694893,0.187345260354604,-0.55579
8174439024,0.578348833636338,0.703339709239776,-0.036643584111296
"Zzz3",2330.85748016343,-0.145268569248152,0.0942982800382655,-1.54052
19394161,0.123433179165705,0.231346739149565,-0.212649602277583
"Mtg2",1229.81579646516,0.29874053093289,0.178744062220057,1.671331216
39085,0.0946562738173463,0.189351392930196,0.497149284069323
"Trhr2",1.04379095204363,-0.135397534964967,2.27043651741585,-0.059635
0234531434,0.952446323314952,NA,NA
"Zfp503",4284.88447779288,0.93925610444163,0.20985202726479,4.47580190
996432,7.61250416848309e-06,0.000137306758813213,8.35307975743004
"Chad",3.90844691626114,-1.27123358058683,0.923054738133646,-1.3772028
1156584,0.168449552832983,0.292381119476521,-1.56323227806252
"Ss18l1",727.256127045147,0.279067302272631,0.160859598300512,1.734850
17506563,0.0827673462878098,0.170609759796989,0.493496041649092
"Rreb1",1908.79115757797,-0.682105287523389,0.25647805029043,-2.659507
45785453,0.00782549974907033,0.02800176070974,-2.43885919408065
"L3mbtl3",1116.12633396396,-0.073039442300337,0.151991744429365,-0.480
548746739865,0.630837249803973,0.746453738116146,-0.0213583131834314
"Sptlc3",194.926127779458,-2.11538556606139,0.715770805496104,-2.95539
51485842,0.0031226881892603,0.0139295754550897,-9.04060995822331
"En2",6.83574649145093,1.82439887497813,0.595729535187324,3.0624616830
5968,0.00219524578491894,0.0107384496840819,8.27168684831755
"Rsad1",556.14183831497,-0.52426744603,0.304597117810012,-1.7211832134
1768,0.0852175829531529,0.174589937209265,-0.915011979451565
"Rln1",14.0388032903992,-1.29914283997944,1.07475344225887,-1.20878220

892129,0.226746528394159,0.362921015484265,-1.3167722818373
"Wdr93",94.9434759899272,-0.117353868042768,0.594845002009718,-0.19728
4784517448,0.843604685554159,0.899863458555446,-0.0123822694761894
"March6",5653.46787170365,0.466477617534556,0.196619791195277,2.372485
5707494,0.0176688532512827,0.0522450871710519,1.37695302472111
"Nexn",1637.62027454389,-0.200995917074868,0.331880816143192,-0.605626
801243453,0.544762601013449,0.674788617274026,-0.0790629091041496
"Atp6v1g1",8965.32831364051,0.394273686314625,0.189094577674769,2.0850
60773095,0.0370637843844186,0.0924839605742762,0.938655269682563
"Lsm14b",2616.70688006208,0.282145175208249,0.0731988535169557,3.85450
265478397,0.000115965125312845,0.00111713834651672,1.91773649863952
"F13a1",192.099507321695,-2.06385448567643,0.561987424928274,-3.672421
11500954,0.000240263329603982,0.00194823172382901,-12.8801714416568
"Mycbpap",428.414364623572,0.420284566815965,0.421124765082029,0.99800
4870917766,0.31827699669551,0.463296610448278,0.323361819367849
"Nrn1",51.3452310925622,-1.89472895591396,0.399676622716916,-4.7406549
4007129,2.13028471144925e-06,5.02252615524032e-05,-18.7559076098029
"Itga9",1265.62006665871,-1.19335854159214,0.276502783089153,-4.315900
65119657,1.58953462347787e-05,0.000241331082213307,-9.93988996819324
"Adgrg6",668.411253504866,-1.13927547967979,0.192295414088431,-5.92461
076141873,3.13037961854414e-09,3.40984032415495e-07,-16.965441268866
"Taf4",2221.36694778393,0.0723706747201488,0.162512721094849,0.4453231
36752541,0.656086249975399,0.7656892142228,0.0193214443686175
"Prune2",458.983987800032,-2.05996542377135,0.508690526137273,-4.04954
548576645,5.1317203220912e-05,0.000589271394818223,-15.3191877085901
"Cdc123",2771.36868135604,0.189382133961115,0.182901122494895,1.035434
50897301,0.300466036636197,0.445086144806859,0.153302457232925
"Zc3hc1",554.244054146642,-0.0598490634238015,0.179784046704807,-0.332
894183442592,0.739214160220256,0.829918678872261,-0.0111575148775811
"Gipc2",4243.26401943855,1.8587254670256,0.494196761847129,3.761104099
66742,0.000169165004412435,0.00149112986258302,12.0969963953335
"Whrn",1050.87271788734,0.860987005188959,0.309681094258943,2.78023754
485005,0.00543191480498206,0.0211611338396684,3.31961210325484
"Camk1d",692.074907226258,-1.09944216877855,0.358645753933839,-3.06553
794857243,0.00217278802599349,0.0106525071488109,-4.99362235455253
"Ifi44l",4.79869574416432,1.81494014420854,1.02085601076442,1.77786105
49097,0.0754266778923138,0.158942235432515,3.33806412448066
"Sart1",5626.56806665862,0.393439941314583,0.264817610748861,1.4857015
747631,0.137358073925126,0.250627711528364,0.544436943055362
"Runx2",404.620681765236,-0.943506234131682,0.416089531645791,-2.26755
580800065,0.0233562937675584,0.0648996744876519,-2.58040715478408
"Shd",314.557180158402,1.70901809960717,0.451364055639662,3.7863407115
6861,0.000152882020004736,0.00138023382824381,11.2547427169835
"Cdh26",9.6495645110623,1.06266066442419,0.753070043396108,1.411104682
41697,0.158213749557241,0.279083517289278,1.35621450617378
"Stim2",1350.55897225575,-0.316263659204955,0.156660960150485,-2.01877
77408051,0.0435103256089265,0.104632995649093,-0.713900797589266
"Fam102a",3499.37612619052,0.376758143931509,0.137996741156809,2.73019
595081154,0.00632966903412633,0.0238254607774894,1.40794536831226
"Akna",1052.1551327752,-1.26888493801494,0.335307549816278,-3.78424207

480621,0.000154177704184472,0.00138788406102901,-8.34923110644916
"Ube2h",5435.33520381847,-0.547865366585752,0.253659074164109,-2.15984
927166966,0.0307843399070777,0.0802343349162147,-1.38215679559957
"Cmc1",981.044280184373,0.694761298988788,0.226817726151249,3.06308202
087124,0.00219070005978579,0.010724145057922,3.150921414337
"Naif1",338.394414137237,-0.182066665442284,0.115331472560469,-1.57863
817568812,0.114419072984875,0.218355983660476,-0.277037844413284
"Akap7",1018.36615361136,-0.605874471224086,0.324293187257086,-1.86829
232013367,0.0617213365195471,0.136413808598745,-1.20693969425479
"Adgrl4",2815.36501199348,-0.515591388582954,0.19471098934134,-2.64798
299431929,0.00809735983419211,0.0287634035429182,-1.82965410817802
"Dap",4096.74925157518,-0.538563490534199,0.19579035873533,-2.75071507
102262,0.00594653416365897,0.0226766131670297,-2.03922818715524
"Polg",3388.41123394834,-0.134039905926845,0.119859865881297,-1.118305
15528519,0.26343668589347,0.405373569690702,-0.121030829431425
"Tbc1d19",1689.30398999107,0.64026202545389,0.212354722478082,3.015059
0388682,0.00256929238160822,0.0120800730302862,2.82752390249791
"Tekt5",13.402235473128,-1.22467238925122,0.558752827181955,-2.1917963
1793148,0.0283942171129481,0.0754676179482363,-3.16461666661256
"AW209491",635.010235249703,0.283662301240388,0.219924609695513,1.2898
1609485687,0.197114518366034,0.328212232840955,0.316026702852632
"Nubp2",1279.41788315254,0.124495706496103,0.14732858605511,0.84502071
0709419,0.398099275719729,0.542541390786557,0.0761279916235378
"Fanci",53.0704826352886,-0.804388811798248,0.431913683181146,-1.86238
325647323,0.0625490875217756,0.13769697510439,-1.59486156907534
"Rbpj",2431.52608411164,-0.918658595393825,0.195197365528893,-4.706306
32183324,2.52245404899482e-06,5.73895088066522e-05,-8.97129743455644
"Nlrc4",247.916776035819,0.211237140209218,0.246404491138549,0.8572779
62886003,0.391291284497763,0.535734672083238,0.131836532915337
"Rlbp1",1.89249862636685,2.18659912858521,1.69643492282751,1.288937818
45797,0.197419705013911,0.328521755428969,2.43401766697114
"1110008P14Rik",1808.009879358,-0.237130029548193,0.356161654198663,-0
.665793261999857,0.505543252269622,0.640402958209011,-0.10567881848430
1
"Orm1",1339.21506323218,-3.52630721575421,1.57123494572404,-2.24429021
60181,0.0248137346553024,0.0680475665694918,-9.47712099814617
"Adk",3390.04580085745,0.299105344000746,0.408220743513888,0.732704912
117161,0.463738427352453,0.602637420455754,0.151478777577216
"Ptchd3",0.220934797106265,0,4.78471990595636,0,1,NA,NA
"Zdhhc1",2932.6025616815,0.23996608341011,0.172579946870077,1.39046330
562822,0.164388234523105,0.287012776889152,0.299532515248955
"Atf7ip2",23.0502070574881,0.467659840632341,0.493848230781777,0.94697
0772563102,0.343653672449222,0.488307776229043,0.335222961787508
"Tbc1d25",511.335293736405,0.205702801203745,0.193105684213198,1.06523
431478402,0.286769908793507,0.430806663198691,0.173221478679665
"Abhd2",5517.82125229844,-0.175050654052064,0.231168305361132,-0.75724
33157677,0.448904103817763,0.589647818393879,-0.0924669786816599
"Ciz1",3815.31849543182,-0.0417672193465213,0.149450891944463,-0.27947
1194872777,0.779883240854743,0.857554896807229,-0.00641837203822048
"Daglb",934.459853083471,-0.499093797541565,0.133420715923021,-3.74075

190714405,0.000183470555905717,0.00158288004382729,-3.21841098438892
"Metrnl",1356.89653110778,-0.03222325955,0.280244100535556,-0.11498282
9213605,0.908458732068774,0.943794136715524,-0.00186402573076609
"Rpl39l",1.43025349238833,0.156953918868488,1.56513813716097,0.1002811
92529875,0.920121088405398,0.950948988554825,0.00789397501032553
"Gpatch2",1061.37282127606,0.20559102241088,0.145338510814833,1.414566
73292057,0.157195568435988,0.277853249330513,0.263292647930909
"Il18",172.66291590087,-0.847425810609789,0.193745116635695,-4.3739208
7772324,1.2203462493467e-05,0.000198407203388849,-7.22446524669531
"Srrm2",25811.654957819,0.240572913716009,0.196750761939911,1.22272926
083753,0.221431971325159,0.357259460231522,0.247620011703568
"Arid4b",3874.02086790603,0.543886710827298,0.220459619420919,2.467058
19530998,0.0136228215449137,0.0430258234255412,1.71104300791078
"Ppp1r10",2861.74620462419,0.138147836385296,0.181081019593124,0.76290
6221180468,0.445519325750646,0.586624514687068,0.0736839573806473
"Rpl22l1",3604.22952783309,0.0822937678353574,0.184587495747529,0.4458
25257567368,0.65572347555341,0.765529958651212,0.0219878190703359
"Tbcd",1725.93677456134,0.48654468560068,0.162576397909614,2.992714144
59054,0.00276508604291156,0.0127304953163376,2.1231617839789
"Suv39h1",723.687103371347,-0.279580354674163,0.160133869177432,-1.745
91643922861,0.0808254728669581,0.167576663667769,-0.499418396425804
"Stx11",472.920375705775,-1.46602216683626,0.316585536844478,-4.630730
07519116,3.64378639138572e-06,7.79019156351303e-05,-13.868657680768
"Tbce",2100.6375370361,-0.0288063687768562,0.162829863013894,-0.176910
845735946,0.859578419332232,0.911029575198301,-0.00268417507205197
"Sec24d",1332.46471762608,-0.438774328819772,0.241454669192379,-1.8172
1202695061,0.0691846541134178,0.148809970866345,-0.835902458175177
"Isg20",389.111946354669,-1.25481076119377,0.210935935524372,-5.948776
61823909,2.70154027352368e-09,3.09850777959792e-07,-18.8060684909485
"Zfp750",288.050481444891,-0.498229450957695,0.369009183752422,-1.3501
8171063182,0.17695770331219,0.303431650618334,-0.594187894032882
"Tgfb2",626.402820741042,-1.23075230337232,0.367132218747761,-3.352340
76586973,0.000801313220913099,0.00496788718008382,-6.52884638135781
"B3galnt2",1600.51102134618,0.116360165851832,0.103784270862851,1.1211
7341948279,0.262214051663252,0.403810882183879,0.105516401887596
"E130309D02Rik",747.696903868413,-0.1079074054709,0.123596317155652,-0
.873063275299746,0.382628589715688,0.527429638902544,-0.06903266291118
42
"Lyplal1",1057.32184222832,0.642822121363418,0.405080363634406,1.58690
022788559,0.112535243317408,0.21560500876514,0.986286614570515
"Lgi2",477.260291451438,-2.73784055659848,0.486988045314853,-5.6219871
9853252,1.88773256646445e-08,1.29602146411704e-06,-37.1147453602324
"Fn3krp",859.052468674556,0.119278196041471,0.213636131941522,0.558324
076351093,0.576623107160666,0.702029181605911,0.0421982767639938
"Pomt1",1252.52808181473,-0.0255767228947026,0.241692931833209,-0.1058
23214194584,0.915722617942123,0.948401052768082,-0.00135499888730032
"Vstm2b",143.309738177999,-1.50736622658749,0.510837542098738,-2.95077
417449507,0.00316978577828095,0.0140720589947454,-6.42675249788663
"Prrc2b",5530.32741637128,-0.348910084868477,0.104147037824019,-3.3501
6810999505,0.000807625287351965,0.00499208105956173,-1.84918939972143

"Npepl1",4869.27005032559,0.159126938206725,0.106604953076853,1.492678
65717279,0.135521322735924,0.247832934787567,0.221982144019968
"Gimap3",1949.67883235632,-1.26274878394755,0.387457427683053,-3.25906
459323448,0.00111780214661534,0.00640838172734666,-6.37706887783093
"2300002M23Rik",2.75580949625136,1.84320026853651,1.27377062241262,1.4
470425334864,0.147885011345664,0.265072803016892,2.44731056173374
"Megf9",1112.72141397243,-1.06054577223038,0.310431340276491,-3.416361
79931379,0.000634638676493305,0.00414267991773066,-5.81859145161154
"Foxk2",1909.13610237603,-0.114260708025229,0.109704719328267,-1.04152
955975695,0.297629841548925,0.44188975683729,-0.0933161314513112
"Pcsk1n",9.49638608521057,0.0482745239676476,0.99277332633011,0.048625
9276788786,0.961217407101879,0.976507253256082,0.0011476351283801
"Azi2",3083.78742049752,0.118434613809383,0.0985735511292238,1.2014847
0307337,0.229563236748037,0.36625677032266,0.118958169806954
"Fndc3b",2382.27181398999,-0.633406387172674,0.135507857005383,-4.6743
1484174022,2.94936584383444e-06,6.52743873133756e-05,-6.10408087212028
"Cybc1",1921.13422220201,-0.46193631850843,0.134779449184565,-3.427349
80223775,0.000609503375475757,0.00401083253966463,-2.54931403417949
"Cdk5rap2",881.197026021419,-0.148607647822579,0.183793555109022,-0.80
8557447699559,0.418769749352896,0.56172073286205,-0.0857095306095686
"Tnfsf10",2468.08678946064,0.0806253248304128,0.277437961816825,0.2906
06679426389,0.771352151016291,0.852226186911947,0.0128922562932431
"Hexdc",3679.19537108488,0.358519319003704,0.107741477532332,3.3275886
61443,0.000876011086377725,0.00532101687295729,1.8772397266298
"Ndst2",1121.14360683639,0.131541178116048,0.161801527027016,0.8129785
95029483,0.416230330605179,0.559584843559249,0.0763675622804291
"Minar1",5.51488414937437,0.419215058014916,0.861180294133267,0.486791
280375074,0.626406250240351,0.743104713262739,0.124472627028419
"Clnk",36.3943247696123,0.161641797757236,0.494007354113993,0.32720524
5855382,0.743512653467204,0.832969990651777,0.0295412772161589
"Rftn1",933.420664806238,-1.07228367864881,0.274945545619445,-3.899985
63618473,9.61983950960434e-05,0.000961015533740277,-7.44971228698349
"Rab3gap2",1714.98212126908,-0.0879120438103634,0.143665927327685,-0.6
11919927331457,0.540590736569257,0.671229641760802,-0.0350456054931322
"Uts2r",1.38556916417534,-4.36164795655824,2.5868049399974,-1.68611397
369708,0.0917738490274482,0.18489424554011,-7.36233641036224
"Igfbp2",3222.42141363699,-2.18440007970756,0.529384730428775,-4.12629
97478947,3.68646714423771e-05,0.000457826346358897,-16.7958972162525
"Rnf122",598.671128154172,-0.381574948679614,0.238643341880033,-1.5989
3398103448,0.109835272575926,0.211589568760367,-0.592626676956516
"Tex19.1",1.54470862602035,-4.19578448906006,1.86160028152576,-2.25385
896784524,0.0242050417254517,0.0666829731904271,-11.3613688749811
"Tsga10ip",0.965395286604098,1.54711175168868,2.18871696011805,0.70685
784406095,0.479654823617972,NA,NA
"Tex19.2",4.93085409677884,0.341685595218435,0.960857778969666,0.35560
4755143708,0.722136582048516,0.816623308014265,0.0692177650600217
"Mfsd4b2",32.0387923605016,1.15292462260689,0.616324069885144,1.870646
75702467,0.0613940591908244,0.135927245556886,2.30081730054291
"Mettl22",272.315524398211,0.0932792406154254,0.25469026841022,0.36624
5798073384,0.714181670719636,0.810447806756575,0.0196043428051377

"Atp6v0e2",1392.82390996549,0.727239269539819,0.224895400545168,3.2336
7782434377,0.00122207230163825,0.00685499589681891,3.62367151631232
"C130074G19Rik",13709.0864736048,0.615326791839936,0.225840326480596,2
.7246099110329,0.00643774987919215,0.0241390859893247,2.29142954769362
"Smarcal1",1006.17657481157,-0.231722958243783,0.167913588555596,-1.38
001313793053,0.167582599742905,0.291171407038599,-0.285909786486827
"Exosc2",1193.20645918972,0.0796874376841301,0.092238569339887,0.86392
7511608429,0.3876277125082,0.531935902837913,0.0503012829995482
"Fut11",1006.04588888534,-0.533204477195567,0.153872127259847,-3.46524
407435489,0.000529750462884111,0.00362362034235533,-2.99675934332305
"Drd5",0.125230909864205,-0.959896647578536,4.78290477385422,-0.200693
238306942,0.84093844719866,NA,NA
"Picalm",15888.3805403256,-0.0422288205754932,0.1023827084588,-0.41246
0475124924,0.680001945139027,0.784674945167225,-0.0102398863650785
"Sectm1b",723.544506816475,2.03023008833745,0.831312887663435,2.442197
29835273,0.0145981661102192,0.0453503595909327,6.28018620243312
"Sec24c",6522.1870330604,-0.0277035504556442,0.11891377781383,-0.23297
1746125304,0.815783345474038,0.882553069530513,-0.00346118065707844
"March4",7.20318526117592,-1.52012090118351,0.756362543169247,-2.00977
813472099,0.0444546759737575,0.106322536162412,-3.40701354960042
"Wdr17",27.9958428185966,0.93422758871667,0.733668556535914,1.27336462
820175,0.202888683016311,0.334978554733704,1.02175421766375
"Synpo2l",54.8898548956826,1.11473489319854,0.366515422911721,3.041440
61481156,0.00235449004955244,0.0113185027086227,4.99547985275113
"Hlx",407.462317626234,-0.999504316197991,0.210348400119682,-4.7516611
280585,2.01752292960885e-06,4.80900514443928e-05,-9.93750692882539
"Wdr45",1979.24507717217,0.34087601716903,0.119537428560252,2.85162581
523337,0.0043496269170492,0.0178692544059205,1.37191473236714
"Dusp10",430.939694491421,-2.57819343169206,0.537593175978114,-4.79580
758628718,1.62020746805863e-06,3.99807842880742e-05,-26.1096526402617
"Cdh6",1549.16245575907,-0.639980158681382,0.519668705717283,-1.231515
67843216,0.218130056432496,0.353511748011039,-0.665476047134855
"Ccdc81",5.21427465979759,-2.23635730337818,0.920805098248548,-2.42869
778591792,0.0151531574859693,0.0465138955701243,-6.8611535582364
"Mreg",279.762850493236,-1.69558667993378,0.46832712113843,-3.62051780
347907,0.0002940140179316,0.00227487512763108,-10.3190524827913
"Neil3",179.529302908004,-0.0106362360145718,0.289538013514939,-0.0367
351971696213,0.970696144290529,0.982079359764256,-0.000192336752002431
"Prss23",2866.49035954768,-0.970559870440908,0.353513504069364,-2.7454
6759676391,0.00604247342733426,0.0229750676455077,-3.66225788114426
"Prdm16",1144.25895035278,0.143658823328682,0.212465552153568,0.676151
130724696,0.498944696253743,0.634434830282736,0.0653677394228914
"Heatr5b",856.231110732883,-0.229013837233218,0.208748534139229,-1.097
07997796273,0.272606431860572,0.415288710713136,-0.201253080387771
"Cntnap2",290.974489794919,-2.76043708884558,0.657013984501045,-4.2014
8909150226,2.651650265105e-05,0.00035460820897817,-21.9302842113952
"Alg1",1706.43924070919,0.253979108350331,0.129667907942901,1.95868902
629455,0.0501492163655841,0.116662667397584,0.545666163018457
"Tmem135",3902.27910288459,-0.232351184353447,0.419059510263656,-0.554
458683463026,0.579264974158551,0.703965219429095,-0.0815613831115121

"Mtmr7",114.037980762591,-0.00749174345071215,0.321291307243084,-0.023
3176039370528,0.981396929620242,0.98808619920146,-8.97910840937898e-05
"Ttc36",17386.8206653601,2.36295125521601,0.816627689774275,2.89354780
006193,0.00380916306886305,0.0161810591539634,9.74460754721187
"Prpf18",1764.45814047187,-0.0253866099729357,0.0811979882374718,-0.31
2650726009245,0.754546034909261,0.840597565344577,-0.00440818814528009
"Dcxr",4000.41864430777,1.74415848370021,0.442444483775128,3.942095669
98846,8.07727433712888e-05,0.000838162293908137,12.3561398671893
"Snx22",218.762661847824,-1.33462310454018,0.413173903264447,-3.230172
80132035,0.00123715419993758,0.00691968806379317,-6.63759397871548
"Morc3",1952.41237160747,-0.199568753911311,0.184566868758267,-1.08128
157157335,0.279571882309203,0.423320071543437,-0.171554632491312
"Ppl",1367.15718816228,-0.858345567723946,0.313404290648529,-2.7387805
251414,0.00616675253989621,0.023356515350213,-3.22470069889154
"Mtmr12",1855.18723358574,0.434214589321396,0.157905006254792,2.749846
88338986,0.00596231180739773,0.0227234471502426,1.64322344329799
"Tcta",1735.75317599329,-0.0507885226357684,0.160155023106566,-0.31712
1009698048,0.751151783583622,0.838157117792918,-0.00896669867220953
"Col10a1",2.15485347948128,-0.789644350755396,1.29139333966919,-0.6114
66953172978,0.540890489714919,0.671434258727309,-0.314546275220012
"Slc9a8",5817.91732877974,1.22989967172387,0.227309545337358,5.4106820
278864,6.27851671257704e-08,3.21308448456239e-06,15.5561144157475
"Zdhhc2",259.33682154797,-0.395038476210771,0.311029860737466,-1.27009
823196434,0.204049641352308,0.336453269098292,-0.430313837097806
"Ubn1",4516.56006486046,0.215638866553461,0.19814448622698,1.088291027
72227,0.27646665019639,0.420053514028615,0.187039365417722
"Wfs1",1133.22631547822,-0.382446282069322,0.17212574063069,-2.2219005
7494011,0.0262900255543531,0.0712443249838466,-1.01028543953539
"Prrx2",82.0058398829354,-2.62823053112422,0.466000777048776,-5.639970
27594897,1.70079521709337e-08,1.23051593970637e-06,-35.765163476387
"Tnrc18",4233.88659978859,-0.118680377781284,0.154878948949629,-0.7662
78300480218,0.443510726990162,0.584809099537038,-0.0636684397883361
"Micu3",980.789300644917,0.667075787587975,0.177325263682324,3.7618767
5537893,0.000168643147888365,0.00148720221507342,4.34323614990394
"Nt5dc1",1092.14007516546,0.0903429119729734,0.242863253264856,0.37199
0866294002,0.709899649807346,0.807515510877057,0.0193146835845708
"Nrtn",420.774439330748,0.962850735525334,0.311504392037132,3.09097001
563484,0.00199503779030596,0.00996905352008856,4.43707580496326
"Asb6",1246.04467633383,-0.209320675618792,0.123106125303012,-1.700327
05605487,0.089069423976629,0.180621490244026,-0.358221283908955
"Tspyl4",595.810198812155,0.869819262553578,0.356146146900543,2.442309
90598498,0.0145936129715312,0.0453460184412614,2.69072752601054
"Cntn5",6.96407802546274,0.679614035688085,0.913409004200151,0.7440413
13982016,0.456851481343104,0.596870154330463,0.350718687524112
"Ccdc27",0.557326422036592,-1.35444728537467,2.65175112808115,-0.51077
4661706193,0.609508853633834,NA,NA
"Cdnf",72.5879027299683,-1.29250005306424,0.562397569117167,-2.2981963
7217346,0.0215506150930255,0.0609683536105355,-3.61564009137978
"Dse",1031.41471511676,-1.42026540085676,0.2497276288157,-5.6872577839
7921,1.29095513326197e-08,9.71854949356832e-07,-19.6622383923955

"Znfx1",3693.3216097424,-0.368959641596426,0.136477777977471,-2.703441
15404143,0.00686255981306811,0.0253901691148391,-1.35533384685356
"Calhm4",0.919083919813579,-1.66578713159742,2.20592254869093,-0.75514
3072718466,0.450163141356479,NA,NA
"Nup133",788.342487546072,-0.202819723878037,0.191447516252028,-1.0594
011760959,0.289417114299024,0.433247976552964,-0.169647548155511
"Uhrf1bp1",1066.09636794548,-0.113133002559506,0.186774096503229,-0.60
5721053816208,0.544700000948416,0.674788617274026,-0.0445015223603275
"Ptpa",7385.96371880846,-0.175593963649279,0.172982038494437,-1.015099
40094113,0.310058447169297,0.45527401630614,-0.138167130087689
"Cdsn",79.6094114715764,-3.39553138358355,1.02234450185981,-3.32131818
326064,0.000895933347033855,0.00541671578309027,-17.7187845830252
"Cyp7b1",4610.43279751965,2.38917817469879,0.6509191443105,3.670468437
71906,0.000242106365096354,0.00195956409574459,14.8966053130822
"Foxp3",90.7310634015193,-1.71402433777122,0.677019080634062,-2.531722
34993134,0.011350382806413,0.0374213331010214,-5.63145152955995
"Cep104",1448.41434970257,0.112649494227001,0.113431685073474,0.993104
300214121,0.320659128589526,0.465887607245795,0.0860429070769752
"Atp8b1",1115.91185150066,-1.09282880439362,0.245309658324972,-4.45489
513888564,8.39342084838062e-06,0.00014837254732704,-9.63414294576606
"Tusc3",1810.42498710344,-0.215072632069294,0.27314799056662,-0.787385
005553749,0.431056521649842,0.573350164344678,-0.119636010215799
"Zup1",1110.13293722116,-0.289905236557605,0.151187138238284,-1.917525
78913617,0.0551711678887376,0.125288543151014,-0.602172561872119
"Mmd2",39.4487363818743,0.56814653257648,0.398378335059765,1.426148167
64884,0.153825577510511,0.273283928045552,0.737024677541194
"Stau1",3449.99595391716,-0.250226185788938,0.125087733144951,-2.00040
547140604,0.0454564980595403,0.107968181162738,-0.556983149712998
"Sgcz",8.2085490698172,2.0424993264057,0.81936922551735,2.492770368713
89,0.0126750789785445,0.040527827143926,6.54777581644328
"Ncam1",2015.34199487523,-0.550772804491731,0.483680519886079,-1.13871
198414494,0.254823302603175,0.395490309174299,-0.510912822990799
"Cfap70",87.4519571036176,0.356328809720643,0.590696685182012,0.603234
821964248,0.546352485504659,0.676281155559284,0.139376723589814
"Ajap1",25.5583011025276,-0.764604831737188,0.585578713835789,-1.30572
511204976,0.191646054978707,0.321488867487121,-0.867667723534029
"Rsph4a",35.3962410592061,-2.27452735190592,0.560928628512985,-4.05493
18331918,5.01489250821255e-05,0.000580298956232216,-16.9497030978044
"Ppp1r3f",276.942564300411,-0.830620302738516,0.252647142059027,-3.287
66949813529,0.00101020357205477,0.00591855446151559,-4.2608022661522
"Ubald1",1974.31386330148,-0.0974109484277149,0.125245019943915,-0.777
763047754996,0.436708716032099,0.578618275699502,-0.053294728124213
"Nphp4",135.297075399425,0.416250201887022,0.392234621655879,1.0612275
8906329,0.288586484202814,0.432576953989877,0.348815604637462
"Ccser1",320.293396781079,0.689134623970112,0.247969883025824,2.779106
13805606,0.00545087114271968,0.0212137895290915,2.65530727917534
"Grin3a",25.8845310326161,0.486233485974598,0.470265576215329,1.033955
08956402,0.301157159067903,0.445858943622321,0.392756385303046
"Myo9a",2854.42836718478,0.197711679819732,0.244068756944406,0.8100655
00783316,0.417902530966165,0.561094406930885,0.114250878195641

"Fam149b",1941.65608546374,0.0421492388573948,0.154149834789323,0.2734
3032131692,0.78452244746371,0.860788944394184,0.00631842097891156
"Rcan2",1208.46488748611,-0.0617723070251337,0.322279076787622,-0.1916
73339891813,0.847998090442965,0.902821162359775,-0.00631503196395948
"Rbms3",3172.1464387317,-0.330639151756486,0.307015266862437,-1.076946
93861799,0.281503971548735,0.425452634466881,-0.28256476613905
"Tmem246",1202.26387026337,0.0169777623605176,0.281898247354187,0.0602
265623141181,0.951975190441652,0.970151658066082,0.000514474945805546
"Stub1",7110.02514146101,0.453561530589852,0.163222469352184,2.7787934
614027,0.00545612045608381,0.0212215114009021,1.74745461645457
"Mocos",634.05631837802,0.0407818691421764,0.280681982746465,0.1452956
42930576,0.884477454660435,0.927951660474155,0.0030494902659526
"Gm7488",97.3855325901787,-8.9131482479038,1.52814916043697,-5.8326428
326899,5.45562680405344e-09,5.06767814185337e-07,-129.197981448703
"Trmt9b",203.138257907339,-0.668471173266967,0.361125851088084,-1.8510
7538342338,0.0641587089541951,0.140305906795297,-1.3128307193811
"Prex1",1774.35049756671,-1.34187933549407,0.310691026712447,-4.319015
42086058,1.56726831952406e-05,0.000239910780079641,-11.1848909464383
"Ap5z1",1763.69793737045,0.337564484096951,0.158631669878947,2.1279766
1623653,0.0333390224075934,0.0852213670557311,0.831253585010954
"Hs3st6",248.399463007121,-0.0528489310030529,0.442262153501723,-0.119
496842731416,0.904881741250212,0.941985577839354,-0.00315853299149116
"Strip2",275.312212529252,-0.147496121221157,0.186989834433129,-0.7887
92191127932,0.43023347429476,0.572841593544606,-0.0821768815563593
"Hnrnpu",20576.5215100149,-0.0711751839465842,0.118749799210372,-0.599
370983528934,0.548925522116546,0.678725750817737,-0.0275830980170097
"Ccdc151",136.028752542195,1.23828117523678,0.446281834470002,2.774661
83831421,0.0055259132529521,0.0214033088212277,4.76021256843894
"Lonrf1",516.726206731464,0.277463292946398,0.265786714018613,1.043932
13923766,0.296516787313755,0.440897149320796,0.22722680248541
"Zfp189",406.90900195941,-0.0180137256614667,0.232370895139277,-0.0775
21436799001,0.93820873848708,0.961736711162456,-0.000702797486906993
"Coro7",2516.34925193024,0.0840278468768878,0.191914160252546,0.437840
786559536,0.661501702223539,0.770094768101474,0.0219515772504468
"Kcne1",523.021282058948,1.01669311006712,0.580433743413582,1.75160924
326669,0.0798410220928682,0.166087725249839,1.82520728982946
"Mrpl12",5912.50886321312,0.674131629735082,0.377789481401245,1.784410
79734323,0.0743569378556257,0.157229321582148,1.24717714900158
"Vasn",1331.32523766931,0.108693892733384,0.260896914032777,0.41661624
5294986,0.676959124897703,0.782369095919354,0.0266765964570226
"Kyat1",4000.66263345757,1.10526672291228,0.287202937039289,3.84838238
183156,0.000118900360190515,0.00113922320540279,7.49084424577335
"Cpeb3",1660.66133451652,1.98198119345957,0.351556359059471,5.63773387
220764,1.72302512434208e-08,1.2351302895008e-06,26.9635343275696
"Baat",39.8468970903162,1.83137873737277,0.397663488566252,4.605347963
86682,4.11776738223844e-06,8.55039706271406e-05,17.154428930642
"Rxrb",2540.18609815787,0.067018153645882,0.069611470847591,0.96274583
5274953,0.335675088658896,0.480260704334544,0.0491528689883913
"Spout1",1167.38851377961,0.255165693400698,0.147684092062301,1.727780
4930612,0.0840275859548689,0.172643813315108,0.448204840363652

"Dusp26",82.6960791549102,-0.184895449311694,0.302165196856927,-0.611901871012762,0.540602683648848,0.671229641760802,-0.0737074545557223
"Icmt",3018.39867060192,-0.0786571880299052,0.179031246779576,-0.439348937377107,0.660408719808254,0.769369460877227,-0.0206226547374912
"Oxld1",496.768337742436,0.873959488622759,0.310642743925398,2.81339096345557,0.00490220083590515,0.0195786792090288,3.43755717967538
"Zmynd8",4883.04122906936,0.133920897830345,0.293733205474867,0.455926995430567,0.648442476438675,0.760409659325271,0.0366806614531017
"Kcne2",0.886791142810966,-0.492320172635655,1.94115948871458,-0.253621701615907,0.799787829237979,NA,NA
"Capsl",51.2490239499724,-0.468951430976779,0.367090249693841,-1.27748266636854,0.201431896160133,0.333253555198618,-0.515308054504028
"Tbc1d13",4524.67519874907,0.288763254450759,0.248184110980586,1.16350419577564,0.244624996780104,0.383755586709991,0.276562840040656
"Mrps6",4200.32228618959,0.99867319743696,0.192749547978778,5.18119605420251,2.20467611897937e-07,8.2826156007437e-06,11.6858263621543
"Lap3",12549.6079752239,1.09709844822004,0.296077308906997,3.70544589273019,0.000211019175094\69,0.00176283285175504,6.9565182521273
"Sdk1",260.698251239517,0.263401161012236,0.307999290781827,0.855200543948064,0.392440135136209,0.536926355963036,0.16380768950018
"Gm5422",0.23846483581651,1.37343152846892,4.78471990596563,0.287045335037588,0.77407760780412,NA,NA
"Zer1",1922.77731579373,0.0248082953750133,0.105640148766523,0.234837755007733,0.814334662780055,0.881864627739313,0.00311881747480963
"Tspan10",2.51374592465966,0.855425945890022,1.03764587153959,0.824391027182326,0.409717411566395,0.55338529306744,0.506156205894662
"Msantd3",336.640165247208,-0.855476340401518,0.346651855619361,-2.46782564851137,0.0135936512336229,0.0429577004462202,-2.6926454512536
"Ncoa7",2337.74690065505,1.03688352883864,0.185587118806091,5.58704470175015,2.30966288228664e-08,1.5214123945549e-06,13.8899584584642
"Batf2",132.807971755009,-0.732740109969764,0.395246977514656,-1.85387909751338,0.0637564589652089,0.139692486448325,-1.4422610030591
"Usp53",1159.20361864719,0.197450020359331,0.194874333317443,1.01321716922922,0.310956441334352,0.456141189673276,0.154988964292411
"Nploc4",2490.25765348594,-0.235128679420532,0.19078491516657,-1.23242804188815,0.217789231220804,0.353188694939162,-0.24471083576963
"Lmbrd2",3385.35738553188,0.962910917648639,0.208106138865027,4.62701832295857,3.70967705922873e-06,7.87922476044029e-05,9.09825248451869
"Ldb2",906.807743157741,-0.629748181981253,0.151538044900881,-4.15571008846763,3.24278808491009e-05,0.000413795039787807,-4.90582636364866
"Slc7a12",161.121163835072,1.10495066457836,0.838479555471384,1.31780275066716,0.187569686893902,0.316479210371868,1.27124322648013
"Plekhg5",1592.7552725806,0.279082945764187,0.172789053691593,1.61516565894456,0.106274825151321,0.206213886815172,0.440627698571813
"Cplx3",5.14002463040511,0.417158100360119,1.14273856379731,0.365051214316165,0.715073176805811,0.811034657748805,0.0873714660775326
"Wdr34",874.900098175969,0.616096818332615,0.129287534238746,4.76532267368418,1.88551659372402e-06,4.53874105486802e-05,6.16113809793793
"Dock3",35.4587513880163,-2.7991785814559,0.744577896365747,-3.75941670457661,0.000170309967401727,0.00149854117883885,-18.2037950353457

"Ralyl",27.5349767415102,2.64449250474652,0.782826564573564,3.37813332
406147,0.000729796883603838,0.00461999338847651,14.2203935174127
"Got1l1",4.20506252207552,-2.99971760221536,1.45981662158558,-2.054859
19112033,0.039892607086159,0.0977045663818526,-6.97676414390404
"Trp53rka",698.838826674512,-0.0700703570206549,0.0964738366319559,-0.
726314609918239,0.467645924937976,0.605978616630601,-0.035098983149642
4
"Slc6a5",1.45505254243712,2.93006800066849,1.7098191506245,1.713671296
5218,0.0865891113307197,0.176583463312036,5.08062549941962
"Fnbp1l",4929.16074334861,0.0966415004729954,0.17088636842297,0.565530
775595821,0.571712797561206,0.697924876461833,0.0347565173238081
"Prkrip1",1036.36084848138,0.417799862783142,0.221554718438367,1.88576
37775806,0.0593267941446513,0.132438705971906,0.844638966406615
"Slx4",1396.36401077413,0.502271769520597,0.217854986032919,2.30553258
691404,0.0211367638754238,0.0600414659153575,1.41274977967552
"Alg2",1406.44734365127,-0.0501974665044152,0.130802894563198,-0.38376
4187115615,0.701153233878325,0.801118415128016,-0.0111311129440833
"Bahcc1",834.119068552742,-0.398065590192628,0.147696243121909,-2.6951
6395121887,0.00703540042024071,0.0258627898555531,-1.45490984018439
"Fam71f1",0.180089686221259,-0.0810280888821526,4.78471990596212,-0.01
69347611719519,0.98648866133387,NA,NA
"Htatip2",1400.14577597556,0.114787284467205,0.378554225156973,0.30322
5474288675,0.761718036667169,0.845664505366508,0.0192420867856767
"Orai2",512.989953728837,-0.935764773419534,0.258790358046586,-3.61591
823004119,0.000299284845293482,0.00230650431364913,-5.68198446391463
"Exo1",69.9932865182457,-0.997050106940906,0.659880665346284,-1.510955
17614187,0.130799871516059,0.24114371168874,-1.4181663927466
"Fbxl5",2996.9063325908,0.00722657219048336,0.085262999136229,0.084756
2514067457,0.93245517465235,0.957752251771138,0.000311943257960129
"Alkbh4",438.422662907451,-0.142782831665773,0.155218247728087,-0.9198
84316152709,0.35763321565486,0.502785524467407,-0.0981762746099481
"Dnttip2",1385.47645042859,0.553867367981462,0.163305695189631,3.39159
860492502,0.000694861550996539,0.0044391908654425,3.0004563635886
"Thap3",1271.52889157474,0.0750816019896028,0.178939652015748,0.419591
751430226,0.674783715185477,0.780502632656251,0.0186065098233694
"Il22ra2",0.722086683268075,-2.3747471470379,2.24996484385224,-1.05545
966797064,0.29121514605567,NA,NA
"Dnajc28",503.95585767089,0.199772755882897,0.337815693495396,0.591366
119838418,0.554275135994327,0.683830460745215,0.0759226882493546
"Cc2d2a",1096.02571368455,0.0997621254504013,0.150074093609364,0.66475
2476933676,0.506208823194158,0.640871348265677,0.0443868179919514
"Dnajc11",3252.98939813444,0.514254785232591,0.134930273555446,3.81126
319306893,0.000138258464499373,0.00128003966800037,3.42538129424267
"Ypel5",3165.22318924549,-0.212407872524788,0.243599757924682,-0.87195
4366188088,0.383233273011369,0.528001862434686,-0.135655449222239
"Polr2j",1349.36572448443,0.430795989440347,0.187344641028458,2.299483
91945146,0.0214774754678496,0.0608143794179686,1.20619819011583
"Galnt12",248.797985198075,-0.649436598703829,0.182050958379811,-3.567
33413810938,0.000360631502757619,0.002668536258356,-3.84870752063649
"Cep131",746.916762302578,0.151182247664116,0.272437322056146,0.554924

877851204,0.578946044732142,0.703802604912233,0.0531038761185484
"Cpeb2",1990.92460803809,0.184219950335137,0.224744567159288,0.8196858
89023384,0.41239519632965,0.555885346563119,0.108172705312186
"Kmo",3353.5948306538,1.79607653377012,0.52196875716516,3.440965592509
22,0.000579642236888194,0.00385992634386817,9.98098979730931
"Cercam",263.787717886999,-1.14106837999741,0.320155883082809,-3.56410
248973083,0.000365103288847262,0.00269650906009058,-6.75032922238895
"Zfp597",522.690225019738,-0.414804007095504,0.210429350195169,-1.9712
2695437106,0.0486979221344521,0.11412404877134,-0.900319352578192
"Zfand1",1703.66388484312,0.328788148792404,0.175637982943997,1.871964
95474011,0.061211452110787,0.135655932400105,0.656798222961024
"Cplane1",977.636994614794,0.0510598030810252,0.184046147682799,0.2774
29349779306,0.781450447874322,0.858793869499128,0.00777264752893332
"Ncoa5",2090.63666701868,-0.0402198032825189,0.127129255975657,-0.3163
69375198895,0.751722159151655,0.838578454495305,-0.00708058121079852
"Gabbr2",15.3071905386497,0.513136402119231,0.548601021219182,0.935354
442065862,0.349605667036019,0.494602473941974,0.361248500205145
"Zc3h10",792.04043560502,-0.134978431190813,0.0958798474314643,-1.4077
8729635857,0.15919406315797,0.280318431668204,-0.171669492077051
"Tbc1d2",569.091568392267,-0.397009191763515,0.33277276130292,-1.19303
391963058,0.232856089081943,0.37014740134679,-0.394569161622116
"Xkr5",9.25421103626397,-1.4899781512307,0.694721292190627,-2.14471352
466027,0.031975758876426,0.0825014353013835,-3.71740547505897
"Myl6b",405.871714987874,0.331846452330125,0.26188294792533,1.26715563
177769,0.205099646775189,0.337784687289689,0.360168420860963
"Trub2",914.473732953521,-0.429294513224903,0.216442830687724,-1.98340
83293998,0.0473218374832436,0.111610159338125,-0.941332624534904
"Wdr70",1026.58146037303,-0.0969465748565204,0.194325032874925,-0.4988
88760867567,0.61785775224204,0.736323602054599,-0.0296739486112398
"Olig2",0.411743783806052,-2.26145946411319,3.03877037191005,-0.744202
1565755,0.456754183553175,NA,NA
"Arhgap29",4858.82726589442,-0.251565657008242,0.19302632089505,-1.303
27126291248,0.192482168345385,0.32245187029799,-0.284722361100733
"Zfp335",1133.48234844129,0.0668679282526287,0.180355218000592,0.37075
6826411362,0.710818660584678,0.807944418794408,0.0142603888805075
"Nhsl1",1032.64889688158,-0.0752945564139606,0.183152509662841,-0.4111
03055877136,0.680996969317128,0.785313396483638,-0.0181966168844952
"Slc45a1",0.391392629669804,-0.472401035078842,3.86698422561197,-0.122
162648595775,0.902770206945453,NA,NA
"Epg5",979.890017479155,-0.0595784427454921,0.152024121340631,-0.39190
124711853,0.695131181480805,0.796899563529676,-0.0135258928560535
"Zfp800",494.796758361881,-0.364284694505245,0.168383623475089,-2.1634
2116286111,0.0305088013220059,0.0796652749667164,-0.921611675252095
"Mcph1",246.569002322144,-0.492320412512107,0.261696509654892,-1.88126
472592755,0.0599359168521185,0.133466252487735,-0.991487339619121
"Rapgef1",3425.97008339969,-0.569608713440191,0.16853268475893,-3.3798
1154370716,0.000725355476279551,0.00459486064863505,-3.06609938082525
"Pcif1",2435.36775412612,0.428381920203053,0.135147984468113,3.1697248
1601547,0.00152583369052921,0.00815758302658035,2.06000612716682
"Endov",956.226055404231,-0.169600496394496,0.173443388228149,-0.97784

3538039062,0.328151713968066,0.472792589887634,-0.127047475046755
"4932438H23Rik",17.8658206824117,-1.03538368799169,0.545331553533463,-
1.89863154127603,0.057612938313652,0.129655709976868,-2.11515709765612
"Rere",7669.1097764279,0.270917065322207,0.105074887040786,2.578323640
90049,0.00992809635777071,0.0335723244508868,0.919506940206689
"Trim14",154.372080957885,-1.48725072963582,0.268586596171164,-5.53732
297455389,3.07129869014376e-08,1.88315037296928e-06,-19.6057786016254
"Srrm3",18.5143648349358,-1.13707908555185,0.519053977114344,-2.190675
99071947,0.0284752477225297,0.0756327998847241,-2.93578495358445
"Slc44a3",616.966999493323,0.0404249777079034,0.295524682623749,0.1367
90529132793,0.89119636992862,0.932668499858742,0.00281784113658419
"Neurl2",109.78905461163,-0.687647737362043,0.229404116287963,-2.99753
879088579,0.00272169218954418,0.0125871808139783,-3.00851137255726
"Slc39a5",848.085047854257,1.84099986308922,0.782014920232248,2.354174
85710179,0.0185638762953883,0.0542503687061573,5.36494144840258
"Heca",2129.26687418164,-0.148667594964092,0.116758523642955,-1.273291
15104876,0.202914745612872,0.334978554733704,-0.162596078320864
"Lrrc17",205.728809848608,-1.34661405836163,0.304633082825985,-4.42044
588811405,9.84974489077073e-06,0.000168761270545033,-11.6980705855786
"Tmem120a",2958.77812325268,-0.000631217930139416,0.471655908773016,-0
.00133830175430536,0.998932190011295,0.999496066138463,-3.181722646306
06e-07
"Alg14",799.257390822649,-0.793804448469053,0.321849737557276,-2.46638
215240983,0.013648563214715,0.0430823515558546,-2.49623068333364
"Txlnb",49.6157878338401,-0.885163078719471,0.277502056864592,-3.18975
321740188,0.00142394332127191,0.00773156415431907,-4.30405597929999
"Fgl2",1817.80180072404,-1.69855301157225,0.448724468243434,-3.7852917
1413667,0.000153528377338543,0.00138453178418425,-11.1805438925093
"Armh3",720.571237414332,0.0692606976586767,0.1321894505068,0.52395026
5268058,0.600313132373695,0.722123848172146,0.0225484171455125
"Eva1c",154.779360083427,-0.302200519999558,0.269702527832696,-1.12049
568992924,0.26250258825793,0.404191381651515,-0.273753416871115
"Gpr37",68.2258096604494,0.636321568776097,0.312344403586238,2.0372433
8092842,0.0416256585690739,0.100872121647999,1.45965911986596
"Slc26a11",655.313046229351,0.117914700122307,0.141247543199401,0.8348
08857212094,0.403825338086798,0.547627378078758,0.0710035382678941
"Cited2",3058.61676806146,0.411623366373035,0.26105851125284,1.5767475
4367373,0.114853625995368,0.219035211234123,0.625059458699522
"Spsb1",709.055747980078,-0.972487243666374,0.212805355882753,-4.56984
383514376,4.88087777970114e-06,9.77077566207523e-05,-8.97948975958335
"Pak7",2.63500221239135,1.3452128803249,1.35441595455489,0.99320513450
9051,0.320609996664569,0.465887607245795,1.02748820715789
"Coq10a",1388.22766751023,0.105535880969219,0.243437895102,0.433522812
563753,0.664634996741061,0.772651756259624,0.0272205364191577
"Rhbdd2",1914.80157427266,0.128393246126889,0.245598183532142,0.522777
669933727,0.601128980722043,0.722793097318598,0.0416805909721904
"Urb1",682.316128005172,-0.585825074269848,0.221730549705676,-2.642058
45810363,0.00824038316690304,0.0291122884103876,-2.07182597686573
"Gsap",1035.1109214741,0.108980181137549,0.373145082355073,0.292058468
115752,0.770241920564736,0.851274060036913,0.0175481149175723

"Pik3cd",1520.52662990059,-1.07094517074951,0.400518225436888,-2.67389
872104152,0.00749750904113898,0.0271570557837874,-3.86195506938088
"Ptger4",961.535711078158,-1.06275739437333,0.224948900401211,-4.72443
916141771,2.30751280885058e-06,5.32448340200813e-05,-10.4581808001105
"Plcb4",1085.73893576625,-0.641552499456301,0.318146198233922,-2.01653
360315998,0.0437442080710064,0.10506584983598,-1.44552555189458
"Dag1",7060.86862581165,-0.89357255955797,0.163578584836956,-5.4626500
1894117,4.6907882475988e-08,2.5983235582864e-06,-11.4919186715187
"Clstn1",4848.04410090782,-0.0734908420633168,0.155780431181008,-0.471
759138847962,0.637098719741143,0.751551866109873,-0.0209900909266492
"Stk32a",185.010735442572,0.628857822984649,0.525606446317428,1.196442
37126587,0.231523974809679,0.368480488191913,0.627831223927113
"Mrap",1506.56193163824,-4.02682927898769,1.41643764444042,-2.84292732
178728,0.00447012625356105,0.018203534187956,-16.1320399051799
"Etfbkmt",846.179991574294,-0.418732148007849,0.244401695241418,-1.713
2947772487,0.0866583227478219,0.17668764790725,-0.725818499797045
"Hip1",2583.71769085859,-0.943866416284222,0.188927248294871,-4.995925
28236619,5.85543299061019e-07,1.78389425704558e-05,-10.3203549887421
"Rhou",1099.85988002819,-1.29075902242245,0.236450619972843,-5.4588946
4181019,4.79107943401293e-08,2.6388832430617e-06,-16.5799993806094
"Olfr906",7.16320097948153,1.03611155745706,0.546883411880887,1.894574
84894921,0.0581487494873085,0.130500036545067,2.10991868955121
"Ccdc40",29.3368627829473,-1.32138639673151,0.796930312832657,-1.65809
528819991,0.0972982347931081,0.193174820854554,-2.17257025177237
"Zfp292",1684.61263396667,0.227227724865447,0.161969399545073,1.402905
27410527,0.160645087943001,0.282245849983283,0.287437795589983
"Rsbn1l",2882.68209771757,0.119780988692002,0.192112964290886,0.623492
480760623,0.532960941930765,0.665152176007557,0.0488394318629044
"Tbc1d16",1508.30821475733,0.151168979584819,0.196884200906442,0.76780
6552729202,0.442602120945789,0.584085018896853,0.081284879490825
"Deup1",20.2095090907835,0.233227431392377,0.384959088390273,0.6058499
16071939,0.544614419830915,0.674788617274026,0.0917413620989192
"Zc3h12d",186.765162402558,-0.60589375168412,0.465857941164205,-1.3005
9766754208,0.193396205850571,0.323647020064466,-0.683509832677955
"Dtx4",2963.84595798307,-1.36932477900508,0.222348714942203,-6.1584560
0619289,7.34575840108097e-10,1.22416749832715e-07,-21.7939467390385
"Ccdc32",833.161672278271,0.356140433220995,0.0856249420283587,4.15930
714561001,3.19214412398487e-05,0.00040840174625628,2.7790561105404
"Sinhcaf",1078.34125785676,-0.155417301550469,0.200814777251507,-0.773
933590334437,0.438970064323595,0.581042638028986,-0.0843808923194864
"Phtf2",649.881262423837,0.0499956118032203,0.118666364043283,0.421312
409850062,0.6735269695012,0.779418888440524,0.0124592390211631
"Ankrd13c",9547.23468002649,1.25996126254944,0.290974314125214,4.33014
60004722,1.49010512657769e-05,0.000230404994115875,10.5530222092662
"Cbx4",1689.50457957931,-0.643439553138044,0.319329777261509,-2.014968
84711479,0.0439079133395835,0.105355217117302,-1.44800770662806
"Edrf1",1271.35678406927,0.354098806762164,0.183008486585008,1.9348764
276988,0.0530054614712505,0.121531101571783,0.746293358062879
"Timeless",819.105499599765,-0.558778537916675,0.207360199209503,-2.69
472415654907,0.00704469243059757,0.025887186771917,-2.04178077388782

"Ifi203",4442.43557086709,-0.587972287701508,0.291549822732481,-2.0167
1289727045,0.0437254832210282,0.105033814444204,-1.32497963883118
"Magi2",683.571924505518,0.281502943591664,0.355276562321206,0.7923487
59941997,0.42815734784209,0.570974076207172,0.15775747872162 7
"Ginm1",4604.78673334731,-0.0742048247664255,0.307392852674414,-0.2414
0061852714,0.809244633409744,0.87822551595972,-0.00963563499458638
"Bahd1",2471.74761446838,0.181338834921295,0.100838511538994,1.7983093
180741,0.0721280039309325,0.153481591252354,0.339860646715916
"Gnaz",229.94657940084,0.204696782260381,0.280418236646729,0.729969579
397427,0.465408779548212,0.604204033799257,0.103135109189531
"Slc7a5",306.315112554678,-0.845591628636698,0.353169662743292,-2.3942
9293577611,0.0166524430794457,0.0499752709193523,-2.53358444968023
"Fkbp6",2.79031123211778,1.9135386309189,1.12644147671602,1.6987466019
9619,0.089366938663362,0.181076217700908,3.26992608050116
"Ptger3",1564.92041366186,-0.624133655355666,0.512089575457289,-1.2187
9781442206,0.222920941254813,0.358860015900458,-0.63962645916741
"Saa4",5.73797934547642,1.61227313897815,0.864718707940451,1.864505907
15007,0.0622506928050857,0.137241520614378,3.20199538307075
"Cox15",1738.05205038591,0.136052238361544,0.282444519432957,0.4816954
44594521,0.630022312624444,0.745806268687476,0.0399026801676308
"Lats1",2195.45383310698,-0.135641013698567,0.108640539836796,-1.24853
037275342,0.211836892695024,0.345929290935308,-0.143985769075554
"Rab11fip2",985.551849350785,0.0272664197027401,0.118530764771265,0.23
0036647070974,0.818063292966725,0.883298155181371,0.00338355747219936
"Ythdf2",2047.31245846857,-0.00486842999713609,0.15482856747387,-0.031
4440033681622,0.974915448872732,0.984509085463048,-7.60066712619969e-0
5
"Saa3",51.6646095388919,-1.54875803692876,1.00018696179597,-1.54846853
247094,0.121509532256893,0.228817349906597,-2.28415666224995
"Elavl1",3034.89664206622,-0.171162449169358,0.15473753979026,-1.10614
68949381,0.268662943946862,0.41091858182271,-0.152225066921687
"Ipo8",2647.709150589,-0.0173341646379761,0.107919152547693,-0.1606217
638738,0.87239131237388,0.920135116312274,-0.00144280493231355
"Stat2",3391.09968306234,-0.508592381592888,0.347794905755907,-1.46233
418941977,0.14364965391844,0.25930481144889,-0.686473091454343
"Nup43",332.586383513894,0.0693469997634809,0.140853830284066,0.492333
077656643,0.62248390018517,0.74047898760359,0.0208358623552048
"Disp2",17.9475861048281,-0.378222283459855,0.56135770133999,-0.673763
41779407,0.500461735895928,0.635617699745884,-0.171394452546293
"Negr1",603.670888986331,-1.39979777275971,0.461759281157552,-3.031444
80225855,0.00243386415972041,0.0115829834967607,-6.24060390377687
"Ift88",956.941159330809,0.680492105176808,0.182303046551881,3.7327522
3891089,0.000189398873929477,0.00162429109418756,4.3705856254697
"Rbms2",1673.64725747405,-1.3292580762246,0.365322299405211,-3.6385900
2964997,0.000274134796015185,0.00215180364440583,-8.16357052897876
"Orc3",1628.66816854009,0.190224542026764,0.152914688868919,1.24399129
628304,0.213502766276073,0.348037533387749,0.200771532335914
"Tph1",12.7002522945525,0.900671007256913,0.623885526910387,1.44364786
232055,0.148838054301189,0.266486554289164,1.19107570176427
"Ndufb10",9411.60806399215,0.757556826135681,0.301243542950522,2.51476

535800837,0.0119111628227949,0.0387978370729794,2.46159816575357
"Baz2a",5193.36142415643,-0.0855758133240129,0.163392899053356,-0.523742548298063,0.600457617641436,0.722231598322123,-0.0278471756280461
"Gjb6",214.445923216895,-3.38952771480398,0.860020326764075,-3.94121814254957,8.10688569325396e-05,0.00084034054889455,-24.0036297150024
"Plcb2",732.050075176732,-1.08424247819015,0.402242234480681,-2.69549635828264,0.0070283846067718,0.0258418712168276,-3.96372940077196
"Ptges3-
ps",3.14558689973894,1.27705761954137,1.51138766430169,0.8449570217522,0.398134835553882,0.542551930642269,0.780884304317639
"Bub1b",231.586954109796,-1.05947171437634,0.311543710852531,-3.40071610329453,0.00067209588576473,0.00432635377373414,-5.76673651229526
"Tnni3k",0.656008158460642,-1.7599922729469,3.06618025418848,-0.574001567762594,0.565966740371602,NA,NA
"Bmf",1734.57674780429,-0.711142715131303,0.263686735951827,-2.69692259098396,0.00699835366671276,0.0257411620059546,-2.60254334639091
"Flywch1",3060.35426849313,0.0019250211306659,0.240318540132205,0.00801028971633609,0.993608781856327,0.99595846332689,7.79580765025938e-06
"Klhl42",1313.56725493257,0.0822089405113429,0.119986282910785,0.685152823447901,0.493247490031023,0.629530638255213,0.0380447156318556
"Plpp6",1512.18427528758,0.141563078037687,0.245982646139134,0.575500264996806,0.564953011487283,0.692644567194389,0.0519873843218817
"Gramd1b",5189.47956666558,1.41708135412994,0.238649893329126,5.93790901961818,2.8868015642102e-09,3.21189920567974e-07,21.1871138398618
"Mrps35",1986.91104553322,1.02304444486379,0.237354877285028,4.31018926834718,1.63114879616117e-05,0.00024521310121473,8.5049602190382
"Cacna2d1",1022.80330027391,-0.786517955648639,0.272156042684487,-2.88995220495786,0.0038530040213031,0.0163175222431294,-3.23692704538498
"Rep15",207.922431410376,0.308332663378125,0.308278120771515,1.00017692662221,0.317224893311405,0.462379529773923,0.237838329788718
"Zmym5",3708.3501434083,0.283643181929744,0.141986515502974,1.99767689857705,0.0457517003127081,0.108510722867921,0.629944924766988
"Gorab",431.784446131026,-0.26113518705625,0.116565725518508,-2.24023988093127,0.0250753538577879,0.0686292075218737,-0.699590846206037
"Gpr26",28.7285381982223,0.46228813524826,0.412988947825191,1.11937168702136,0.262981604519094,0.404833043179638,0.418038162697348
"Sdr9c7",2.42433959120787,-0.0254052280355529,1.05009230675641,-0.0241933284075057,0.980698399733826,0.987535887931113,-0.000318643565051592
"Pnrc1",4767.18249620823,-0.268078026252856,0.102332368146235,-2.61967968795335,0.00880123916922114,0.0306549770134823,-0.934241264749863
"Gpr176",180.725250358101,-1.97007392144514,0.489967985255697,-4.02082172862177,5.79954828236843e-05,0.000649136580996668,-14.4600813812044
"Rdh7",22.2646746256924,1.18829834827605,0.408436200598801,2.90938547203678,0.00362140044207055,0.0155975404946966,4.94408406122951
"Abcc8",46.7971250164429,0.0524988090252665,0.557292878092654,0.0942032656238937,0.924947690719855,0.953910402711083,0.00247718409872158
"Ndp",34.552284387048,-0.977084682620704,0.456052236534943,-2.14248413744121,0.0321545437848944,0.0828749893876894,-2.43335314894009
"9430038I01Rik",287.817361539697,0.222932854220711,0.196329287296223,1.13550483114803,0.256163858845859,0.397002060231822,0.205948449165984

"Tdrd6",11.4984035197451,1.32056531960983,1.11930625522152,1.179806968
33369,0.238076997412857,0.376238069018957,1.29089640791733
"Rgl3",4133.96510683527,0.713081241081959,0.303138129717532,2.35233107
015081,0.0186561617366089,0.0544792334192603,2.0750205519427
"Maob",616.345872580117,-2.54832530884656,0.643069570016239,-3.9627521
3081877,7.4090694479674e-05,0.000780875870791721,-18.2335080371347
"Hmx3",6.19585376287822,1.79106786231523,0.693258141002457,2.583551142
6167,0.00977889676731525,0.0331886734846149,6.09956510715202
"Hs2st1",1289.47090539638,-0.461215531857159,0.143352116291016,-3.2173
6116487361,0.00129375625182275,0.00716434518547006,-2.27777683436123
"Thbs1",5159.08490784673,-1.0526322624108,0.176943537751963,-5.9489726
2586873,2.69830757187584e-09,3.09850777959792e-07,-15.7759839450585
"Wfdc5",0.374182831059447,-0.183419022418386,4.67310928262479,-0.03924
98893831483,0.968691158280221,NA,NA
"Tax1bp3",2853.76348157776,-0.132675625177739,0.149582917248372,-0.886
970434982494,0.37509481814155,0.520170609112656,-0.0867165798869579
"Kcns1",7.19189462327047,2.30017393523576,0.782164459065744,2.94078043
124486,0.00327386508145774,0.0144334933575758,9.74860601780366
"Cd209c",336.523443049991,-0.655574374520678,0.366975296329227,-1.7864
2644635278,0.0740302367503647,0.15678381712693,-1.21470548748918
"Ikzf5",1411.65653759919,0.458439458501178,0.3688235387491,1.242977766
70117,0.213876026539688,0.348558572841092,0.483171805007932
"Fmo2",7840.69704754609,1.45011625601257,0.329838438171745,4.396444101
69535,1.1003868170302e-05,0.000184482735670291,12.4680338390004
"Alkbh3",1044.60289401037,0.274715042432298,0.172059286146369,1.596630
14176754,0.110348175958663,0.212270001464286,0.425779806619731
"2310057M21Rik",423.09333251365,-0.393822574472246,0.26741869580474,-1
.47268153143564,0.140836928789827,0.255397920158486,-0.537541222814891
"Fmo1",14221.1327092272,0.793263872996976,0.524645760280456,1.51199901
543649,0.130534113010025,0.240767584473509,1.12954682501193
"Ankrd6",546.249300683695,-0.416804302535665,0.394388883037851,-1.0568
3582996852,0.290586518873879,0.434291736856911,-0.347630946138131
"Arntl2",349.532726762776,-0.949489915710587,0.303654139330293,-3.1268
795406665,0.00176672299041618,0.00909626745897401,-4.46249922237631
"Scamp2",5258.98221149742,0.563099527512652,0.158413186869836,3.554625
33542321,0.000378518053353395,0.00276728346617341,3.31659379077405
"Ccdc114",867.62845998399,0.713558530414972,0.321395249697463,2.220190
03419204,0.0264058705242498,0.0715284333817482,1.88212496363009
"Nemp1",420.018444658401,-0.0785988033234962,0.168983673641503,-0.4651
26610338954,0.641840790734237,0.755302778546452,-0.0220576992732696
"Cd209e",3.24672369431393,-4.41042201142764,1.5746687175996,-2.8008570
705277,0.00509670872943485,0.0201736960630371,-17.2155340983845
"Pclaf",402.307991868822,-0.627528148645524,0.370159931420035,-1.69528
923954074,0.0900205650756281,0.182008522558337,-1.06912081491029
"Cuzd1",5.29045163477295,-2.91857704273355,1.40709410057908,-2.0741875
3410481,0.0380618975144034,0.0944066757073958,-6.88826061065715
"Zfp704",3851.34756491663,0.55809464990211,0.174635080018856,3.1957763
0016747,0.00139455187654566,0.00759736588122023,2.72347604411862
"Emp3",1316.63859546415,-1.14736244220089,0.304382001207234,-3.7694818
9331249,0.000163586775203689,0.00145378985547927,-7.49638595602288

"Kyat3",1482.15970997463,0.838807620625814,0.436290745952616,1.9225886
1414611,0.0545317350482354,0.124122437613076,1.75016102853626
"Ttc12",853.841126167607,0.235079124395892,0.127464884980444,1.8442657
7117265,0.0651444172743019,0.142015412345074,0.458832063616718
"Gas8",982.941037856166,0.171410362945779,0.186967525446619,0.91679216
771106,0.359251557640541,0.504369734366018,0.117321082413659
"Prrc2c",6118.63380206612,0.0370164885750309,0.169999162713051,0.21774
5122883414,0.827627704117364,0.89012548816153,0.00430845379753599
"Gpr34",205.498144070505,-1.41756697995692,0.406234014138361,-3.489532
95544106,0.000483865392543938,0.00337304519979324,-8.06870523552282
"Syngr4",1.89536930522096,-0.0255149538491038,1.12762577729193,-0.0226
271466677355,0.98194768945896,0.988436553901435,-0.000296759903101581
"Tm7sf3",3452.5593000408,0.775443344885782,0.194073354214015,3.9956198
4192152,6.45252057542941e-05,0.000704824908569874,5.62782748815882
"Trappc5",1719.70962941038,0.218252100179105,0.112762443398551,1.93550
346729991,0.052928543593948,0.121426137544981,0.460173449106673
"Fgfr1op2",3790.50694231539,-0.0153062335281834,0.137166543493688,-0.1
11588679996793,0.91114954801441,0.945280083387515,-0.00086134315186050
4
"Tbc1d10c",544.32344597283,-1.14918493405101,0.428525392052591,-2.6817
1957919817,0.0073244828429515,0.026674031840095,-4.16472064187668
"Lrp1",7549.1313596515,-1.64456358405116,0.229655593788243,-7.16099946
412605,8.00909625061939e-13,7.09824357702622e-10,-34.6443823162926
"Ints13",1649.69443629184,0.196787897683526,0.0826111980071028,2.38209
713005986,0.0172143562945876,0.0512984134237916,0.584478840448855
"Gbp7",2723.25509008417,-0.502405518086974,0.316968012139824,-1.585035
39425091,0.112958292753421,0.216266770631009,-0.769304723794782
"Sema3d",615.330255184104,-1.45758789033722,0.44226401723908,-3.295741
53338654,0.000981622814546474,0.00578845366853226,-7.50933261103742
"Nxph4",119.692190143273,2.28640843876608,0.581077178987613,3.93477582
917574,8.32743841461739e-05,0.000857730555775012,16.1448342428874
"Daam2",1712.79305412867,-0.685280766793674,0.281846052047229,-2.43140
097871175,0.0150405589166031,0.0462775473813993,-2.10593520967169
"Klhdc4",1893.44109253411,0.22555913338925,0.162190772571802,1.3907026
263741,0.164315620878811,0.286978321022488,0.281576428966202
"Gbp2b",101.489161248689,-1.70283492959229,0.719892050130326,-2.365403
15910422,0.0180104528682965,0.0529984621228562,-5.0020646341635
"Dnm3",640.577163952581,0.280483074752499,0.264005922285375,1.06241205
62315,0.288048662933236,0.43212840115967,0.235334418220412
"Plekha1",4078.40340131942,0.161432390420148,0.169427504440849,0.95281
1002870596,0.340685838456148,0.48536405657007,0.116692378067263
"Mrps28",986.126117288848,0.557148573133834,0.264017525767206,2.110271
17050211,0.0348350053298078,0.0880950627653168,1.35350263987576
"Bach2",342.601758227953,-0.0781489039942948,0.267999748168979,-0.2916
00662046221,0.770591969184102,0.851516135741095,-0.0125614070393528
"Accs",723.300155221458,-0.168585170406285,0.159121229707828,-1.059476
29185517,0.289382920859035,0.433230051513319,-0.141019201489185
"Cdk6",2748.25231975372,0.18658894914194,0.284861440183462,0.655016519
686797,0.512457118069088,0.64659864674245,0.0813582874267209
"Pacsin1",95.606916985429,-0.39076343589418,0.460131012239443,-0.84924

3857727274,0.395745613578867,0.54012655561814,-0.24069144352109
"Ndufa4l2",351.42389621978,-0.647744275372587,0.221966481523805,-2.918
20760921112,0.00352049869653337,0.0152947155938074,-2.70773164444648
"BC052040",304.719057031982,-0.0341097599683368,0.13181765264486,-0.25
8764735101409,0.795816768637985,0.869241799053467,-0.00477993514711246
"Btnl9",1679.85900124422,-1.8598388728388,0.69330551210693,-2.68256755
55165,0.00730593943269189,0.0266294156048811,-6.74329000835843
"Stac3",115.641952198245,-0.116877699334597,0.238871755260823,-0.48929
0578565805,0.624635990213867,0.741997839477883,-0.0348773512532924
"Hey1",133.704205571741,-1.0710989717409,0.323578368755528,-3.31016864
897464,0.000932397738936278,0.0055662270292408,-5.5601152799776
"Ddx58",2835.50419050733,-0.43000194332146,0.262383323173465,-1.638831
07402059,0.101248445656021,0.199232998461033,-0.69371366095141
"Suco",2514.62801189083,0.193550309267481,0.106883652928823,1.81085043
375503,0.0701640069523642,0.150352545066183,0.366733792156228
"Btbd16",17.5948899136537,0.857533716381243,0.552183836816179,1.552985
90651561,0.120426501628592,0.227086646881458,1.27122824469187
"Rbm48",426.181354874738,0.0563247715995446,0.166401312365709,0.338487
544351554,0.734995811912872,0.826648853340777,0.0107228440009409
"Alx4",11.593025738076,-0.679766034152273,0.484628279414549,-1.4026544
9423104,0.160719893225746,0.282316799830233,-0.859717393198228
"Cchcr1",1111.71201697563,-0.208579682292067,0.203377007667283,-1.0255
8142970269,0.305088924474758,0.449767416407745,-0.166660314369157
"Zfp770",502.699264601411,-0.387822791954262,0.129450302566607,-2.9959
203205007,0.00273617919799405,0.0126332043576813,-1.69533889395383
"Slc25a24",1252.03833889515,-1.25069877257517,0.221817962687679,-5.638
40167595518,1.71635775813054e-08,1.23489090656935e-06,-17.015166379197
9
"Gm15429",0.25587404954482,0,4.78471990596088,0,1,NA,NA
"Dcaf1",1205.27202563683,-0.134206563187071,0.230203585650776,-0.58299
0759278032,0.559899520951241,0.688348566265745,-0.050120774006331
"Cul9",2532.62221247243,0.724973466729567,0.214365801419332,3.38194554
322314,0.000719744111485707,0.00456821962426703,3.90661502544162
"Olfr56",38.1896813105298,-1.25152339816731,0.527539995820907,-2.37237
632801624,0.0176740788357999,0.0522451772499131,-3.69425639572394
"Il7",99.2309991242168,-0.344797454099618,0.230742773459954,-1.4942936
1938158,0.135098892127153,0.24726914471935,-0.481777855991188
"Nsmce4a",2403.70795141849,0.306050001606395,0.166413874620521,1.83908
945275321,0.0659020300894041,0.143242193768796,0.594722000144118
"Fam102b",1492.67869610337,-0.100813052500515,0.153728049708058,-0.655
788274761613,0.511960361690921,0.646180938131123,-0.0440226127327783
"Tex45",2.29450344892923,0.124031847927631,1.26819131761554,0.09780215
824284,0.922089393572159,0.952029272021625,0.00609734322405355
"Arhgap9",1071.80191038112,-1.11017037599824,0.353943623067909,-3.1365
7402943305,0.00170934277044398,0.00889478658609999,-5.24254641016468
"Trim7",7710.09162167318,1.35232119712045,0.422377578857534,3.20168793
234306,0.00136624921752504,0.00748500344009646,6.61941455259017
"Ankib1",3644.01875713248,0.861653637396909,0.17122681621853,5.0322353
4973176,4.84793594925233e-07,1.53201061812839e-05,9.55258903179174
"Mars",1769.03745162271,0.339027441755657,0.20803935794564,1.629631263

54314,0.103179452728756,0.202027813748272,0.542223504810302
"Skiv2l",5564.77572608338,0.224442697509131,0.113730918662301,1.973453
658416,0.0484438993751156,0.11367904080106,0.488022867120679
"Ufl1",1695.64717556695,0.041723045758028,0.118833987214616,0.35110364
2451006,0.725510595412345,0.819298366115481,0.00831569324845487
"Bcor",1172.75825353468,-0.581689643788707,0.145697949097452,-3.992435
35953712,6.53981634050662e-05,0.000711569972138382,-4.2161079447855
"Sec1",12.6066422338552,0.586060587872386,0.470475265689786,1.24567778
714814,0.212882711741989,0.347271949019225,0.61984527604573
"Trim41",3649.352511678,-0.261848816991548,0.198499147379486,-1.319143
28322505,0.187121215274897,0.315913884789154,-0.301724626177967
"Lrrd1",24.8551418800724,0.369028642278568,0.418212170627263,0.8823957
50762284,0.37756283279616,0.522775182066434,0.239353368450155
"Etfrf1",2060.38463584214,-0.0946822113426949,0.462712035379151,-0.204
624483703155,0.837865524913453,0.896388779904753,-0.010356440069211
"Gpr63",18.3467163465528,-2.40792922635747,0.720335891850392,-3.342786
68271271,0.000829416321693568,0.00509995567340939,-12.710310840983
"Cacng5",1.96981341168619,0.630341872549821,1.25438984028453,0.5025087
51511287,0.615309697919327,0.734499111670812,0.194502380879461
"Pex2",2313.38722371409,0.408348742734839,0.118203137474544,3.45463539
682084,0.000551037455005012,0.00372930520572292,2.28329560714439
"Cbln3",16.7640996894499,0.0637494151287315,0.43262268478565,0.1473556
92086089,0.88285127626687,0.927069915157875,0.00482750704021381
"Aqr",1507.14175926492,-0.348801327445188,0.134061836924826,-2.6017943
3197515,0.00927374488831706,0.0319469042106724,-1.20116015448298
"Ppp1ca",8348.64092058197,-0.467649088230164,0.222525326095191,-2.1015
5444522353,0.0355923239394229,0.0896498039233777,-1.12789677197987
"Klhl32",302.789916070465,0.999370065831996,0.379372891287597,2.634268
52256133,0.0084318775122964,0.0296331556839862,3.51664476902349
"Wdr47",752.838636643834,-0.149078296548867,0.128972431694899,-1.15589
273296429,0.247725035468186,0.387060076347524,-0.141501446087167
"Map3k10",1282.99186354818,0.262023240454167,0.126156263399133,2.07697
369432367,0.0378039871923596,0.0939103251722037,0.619793687486821
"Abhd13",1284.1870755898,-0.155422276937867,0.151804885852697,-1.02382
921382834,0.305915949235811,0.450410901302331,-0.123964030795146
"Havcr1",2623.99465732397,-1.12608552482434,0.629208889875972,-1.78968
470239877,0.0735046130139689,0.155917422165619,-2.0927497221495
"Akap9",3425.70925560277,0.47664852534844,0.286048452688378,1.66632093
573218,0.0956495090138592,0.190751163624039,0.78970436948289
"Fbxl4",1045.59529144387,0.0812535344017423,0.251231804713044,0.323420
573659253,0.746376750550041,0.835055102821876,0.0146465642511969
"5330417C22Rik",33.4358331233533,0.323003455953149,0.401347494654753,0
.804797489096084,0.420936550859756,0.563967626514362,0.185002951970279
"Timd2",2119.11830409371,1.94448813055854,0.747910864769709,2.59989287
781943,0.00932528652728888,0.0320678018887264,6.68885018173358
"Slc25a28",2897.58209079281,0.313293731001639,0.169743264642162,1.8456
9167832431,0.0649369862043582,0.141674091642898,0.612246751158267
"Dtx3",7540.6182762552,-0.204643544333103,0.2114372761443,-0.967868807
548577,0.333109894289701,0.477817753171529,-0.151134555446962
"Cdh18",0.276057444612799,-0.542286278449553,3.97874925212163,-0.13629

5665820203,0.891587550086951,NA,NA
"Rc3h1",1866.42665489945,-0.642959186931763,0.157343877193815,-4.08633
115186154,4.38248154962964e-05,0.000523199524499332,-4.85790878457555
"Hipk4",103.710671954479,-0.0885241908519829,0.339486052606355,-0.2607
59433774528,0.79427802952941,0.868052072435444,-0.0125264894650304
"Plekha4",585.684126113754,-0.60269943788082,0.283132603446362,-2.1286
8256973802,0.0332805318889938,0.0851468062946597,-1.48467676454753
"Mterf1a",456.006987139775,0.201722066227332,0.13168735104533,1.531825
68125237,0.125565456930404,0.234351993800039,0.292684808726378
"Pitpnc1",2916.53422147973,0.642196232443163,0.182413370233971,3.52055
461515489,0.00043064529649147,0.00307122238148891,3.71554165843199
"Ltb4r2",26.7813830966287,0.0689295121140429,0.497511680552307,0.13854
853023254,0.889806917829487,0.931914663148699,0.00486049779771746
"Zbtb38",2346.72698123049,-0.175000149478399,0.165246659845065,-1.0590
238231894,0.28958893026341,0.433338830566076,-0.146341309212009
"Large2",1282.94291324739,0.91567586106312,0.323271579788402,2.8325281
8469993,0.00461814947075759,0.0186543771246802,3.64592319151561
"Ppp1r15a",1116.52285972369,0.363355310937262,0.270906776837323,1.3412
5589318666,0.179837384837229,0.307234673578927,0.428811377273472
"Slc26a10",198.178879876851,-0.352887450248487,0.357654281954821,-0.98
6671956839772,0.323803481996137,0.468717233432612,-0.267402443766195
"Rprd1a",1858.00252228224,0.0199905980555258,0.0734309352522493,0.2722
36734924515,0.785439997720572,0.861236591809454,0.00298631598221441
"Spns2",5430.51891934616,-0.188928900844512,0.389090658009353,-0.48556
5245413757,0.62727544270629,0.743683362634516,-0.0559493900718809
"Sgms1",2264.48115184849,0.0141679929448885,0.145170669363717,0.097595
4234211827,0.922253558739005,0.952088732371109,0.000695606548383381
"Usp45",1078.33234116207,0.000356323646878018,0.104356429659647,0.0034
1448675505811,0.997275639028835,0.998197146292824,6.42979180740742e-07
"H2ap",0.40340903606095,-0.0393526536456841,2.64281949187166,-0.014890
4054048029,0.988119614453145,NA,NA
"Arglu1",8709.91314707343,-0.141107556718175,0.156661649067107,-0.9007
15379663414,0.367739669303193,0.512778951017211,-0.0942472076670393
"Os9",9118.60065525661,0.26694409361872,0.108653494300985,2.4568385520
9707,0.0140165662438618,0.0439946890893137,0.833849622748495
"Mybbp1a",3693.35545670512,0.260979771874683,0.143508637690845,1.81856
490364644,0.0689778363414071,0.14849617455943,0.497739598299846
"Gtpbp10",1227.31878608091,0.430858075732912,0.116249113288144,3.70633
429835247,0.000210280703922544,0.00175967946999217,2.73277060191056
"Blvrb",1159.72454946097,-0.36390123929734,0.222057834125385,-1.638767
84951376,0.101261617332487,0.199232998461033,-0.587074697820591
"Ggt6",360.008052988198,0.867766029369341,0.378866299319793,2.29042812
973153,0.0219965104218558,0.0620497627611899,2.41223214080832
"Rabggta",3176.98988374195,1.11696005094426,0.255472946548953,4.372126
54424932,1.23042170148858e-05,0.000199590368848789,9.51565459513921
"Cfap69",864.874155028059,1.35561816561808,0.431717775859565,3.1400564
0124267,0.00168915310587736,0.00882742086797021,6.41192805135513
"Prdm13",0.0810182756847091,0,4.78471990596022,0,1,NA,NA
"Dgkz",4392.29279594841,0.200066203581972,0.207502254595963,0.96416399
8948012,0.334963710556238,0.479665278233367,0.146981988640074

"Bptf",5077.0426955397,0.164596243140791,0.235552366959738,0.698767094830019,0.484697595574389,0.622172975632144,0.0781070286696211
"Dxo",1565.77213865313,0.491586420789817,0.220297941981716,2.23146170303587,0.0256505615854009,0.0698804876054418,1.30809613608424
"Xaf1",998.266422941642,-0.877498385504763,0.255889188809355,-3.4292124242831,0.000605335547704884,0.00399148884313487,-4.84694216190731
"Lrrc52",33.2621037563858,2.29841656206561,0.788542491405674,2.91476564308969,0.00355955730761588,0.0154026294682411,9.5917911605589
"Ltbp4",6175.86999329207,-1.10414688327188,0.148364045358906,-7.44214597681564,9.90626651292801e-14,1.10565351822974e-10,-25.3130248029067
"Sox30",6.35179489459285,-0.58279611587612,0.739705787831431,-0.787875565479462,0.430769494917788,0.573281251239304,-0.324255428557387
"Lrfn2",14.3856191412942,-0.0421438323156117,0.463695319630078,-0.090886904679635,0.927582455675893,0.955515781951002,-0.00191771288174435
"Chrm4",4.03072900904226,-1.25041575482444,1.01092034191795,-1.23690829334002,0.21612113078341,0.351365780706656,-1.30784420922517
"Igsf23",12.0844796650025,-0.282664696236622,0.513367313075753,-0.550609064965754,0.58190169339025,0.706162893989114,-0.0983416879295069
"March9",802.848969863753,0.938400217434768,0.265053618459247,3.54041655001609,0.000399495951637159,0.00289137790089879,5.48590841742998
"Abcg5",6.40201840817135,0.93951975873716,0.726525871953006,1.29316765583529,0.19595307962689,0.32666665069811,1.0511488410268
"Ambra1",2174.59759725849,0.0333376516426165,0.115279292783571,0.28919028593631,0.772435765124391,0.852772764348787,0.00530942650393526
"Pvr",1374.20710586349,-0.42534242196656,0.140179000410081,-3.0342806035302,0.00241110055175733,0.0115139942586736,-1.89881027626616
"Manea",1143.80674082519,-0.0219504468174123,0.149691845989586,-0.146637558460862,0.883418106746728,0.927515278948236,-0.00165168357094612
"Tsfm",1878.03694211114,0.491033706710448,0.271183990632166,1.81070315237188,0.0701868146314326,0.150368367397393,0.930345464455971
"Tlr8",53.7753653137898,-1.65050377008214,0.476746897891583,-3.46201260539189,0.00053615204326099,0.00365520718164433,-9.26197108730894
"Zfp609",1056.57663694632,-0.355998336024778,0.245164786736649,-1.45207776680908,0.146479983162902,0.263231954996338,-0.475157984192556
"Cblc",1246.22006298755,0.824708576723278,0.231272930397651,3.56595376426144,0.000362535289045708,0.00268058895176838,4.88369224246401
"Milr1",164.999997620136,-0.978932518243115,0.326667172779495,-2.99672755579854,0.00272894481200572,0.0126087597024852,-4.28122773650554
"Abhd11",3102.65785296857,0.742438052851964,0.168324996514597,4.41074153111644,1.03017225907708e-05,0.000175426189585022,6.42082120341323
"Matn1",1.64547141874483,-2.85866402562777,1.82454839959814,-1.5667789499349,0.117166369324954,0.222423315071362,-4.29706625964322
"Necab1",6.72885182405993,-1.22210027633046,0.952229107787413,-1.28340991294638,0.199348502737339,0.330743456975207,-1.35214672693457
"Adam22",396.859605778345,0.00616246585955549,0.23402312445791,0.0263327219215204,0.978991955635487,0.986669833155619,8.26991274862822e-05
"Gm9770",0.462978786182601,-0.0644063810549981,3.12681166577592,-0.0205981005379854,0.983566255696713,NA,NA
"Pitpnm3",898.147963429026,0.154961076279751,0.196711862421542,0.787756642493061,0.430839066602158,0.57330895304314,0.0862095765352515

"Tex2",2824.82088700096,0.449617545351578,0.288092968499064,1.56066823
738893,0.118602049236947,0.224493035241432,0.671688415015385
"Ckap5",1277.94169476386,-0.470463451768396,0.124738928413806,-3.77158
484324711,0.000162213983356513,0.00144554307471905,-3.07648762928853
"Otud6b",1737.61661467319,-0.231967931102604,0.257123647835976,-0.9021
64904142074,0.366969275126143,0.511887746917266,-0.155337307034866
"C3ar1",639.951677522279,-1.44976021731877,0.465331736347568,-3.115541
24525887,0.00183607669499,0.00934721237569584,-6.77426139951977
"Aipl1",7.8451738417372,0.431519216356999,0.736494051626854,0.58590998
1762662,0.557936009152208,0.686782595104151,0.162137948231019
"Mettl27",1221.09122504706,0.0994639173264108,0.237096282556139,0.4195
08548401049,0.674844508585967,0.780526647787946,0.0246458057719245
"Wdr7",791.914140206929,-0.227906957485279,0.200999991219876,-1.133865
50965552,0.256850966631215,0.397719198489155,-0.21013227830211
"Gstm2",2840.29895651494,-0.311339012881448,0.19437251565048,-1.601764
59022271,0.109207676054078,0.210699709845876,-0.484854887261846
"Plppr2",827.720000093614,0.0102323481228642,0.2096845817478,0.0487987
62586995,0.961079668313953,0.976454138543351,0.000243811222770667
"Apoc1",848.022322719608,-3.26182829193156,0.861753290229763,-3.785106
86168879,0.000153642543511955,0.00138453178418425,-21.4706365590628
"Btaf1",1655.51240086583,-0.0829151930419047,0.149846207362437,-0.5533
35279560031,0.580033847485999,0.704597841771977,-0.0290309370220362
"Slc26a7",145.280968041828,0.829142936844208,0.484969440036485,1.70968
079304508,0.0873249163566614,0.177805056299309,1.43198583415021
"Rundc3b",39.8725719698931,-0.154041757851657,0.308274917360876,-0.499
689560118611,0.617293684650686,0.736020317180965,-0.0472134222678664
"Cyp2b13",0.408423246610825,-2.46357711921313,4.73888281984416,-0.5198
64536193394,0.603157994663821,NA,NA
"Abcb1a",248.506511300306,-1.39593732106043,0.426906414879759,-3.26989
071235579,0.00107589042400188,0.00622646311848861,-7.08989142488607
"Ofd1",693.287930955043,0.217732218880463,0.11091722364408,1.963015406
68868,0.0496443838644302,0.115702390220973,0.469590476353454
"1110051M20Rik",838.391664482553,0.857574342972155,0.20537941569881,4.
17556131442983,2.97252173259641e-05,0.000387605096854007,6.73669542844
643
"Cd79b",268.01114322547,-1.15608167012478,0.601479120257865,-1.9220645
0928694,NA,NA,NA
"Ranbp17",111.396211054219,0.0063393102600975,0.259466508497255,0.0244
320945189138,0.980507948237357,0.987395102506559,8.04142340682035e-05
"Pogk",1260.92008556793,-0.295918830595486,0.135499637731318,-2.183908
64765457,0.0289689614525633,0.0765569002984655,-0.76042883915855
"Mis12",822.877996026188,-0.43571779405652,0.139485806927656,-3.123742
86426507,0.00178566431901193,0.00916957674271652,-2.04432869767595
"Eps8l3",156.045476556507,-0.436296079663081,0.599446655526423,-0.7278
31368547604,0.466716819812234,0.605337893480938,-0.219006877859002
"Bace2",869.405920310085,-2.73463866923114,0.570468562058525,-4.793671
11723606,1.6375665383299e-06,4.02131893757638e-05,-27.6781408105682
"Kazn",2415.87456368246,0.664136568927702,0.263531381259677,2.52014225
309008,0.0117307415339522,0.0383897278329977,2.16506220869686
"Tlx3",0.984520979584968,-0.169177213301652,2.97354746871589,-0.056894

0684759643,0.954629579331795,NA,NA
"Ildr2",3076.03497274271,0.631600084884117,0.396603171977661,1.5925240
3790581,0.111267006375975,0.213721218630554,0.974611246356254
"Apobec1",1171.50397021471,-1.44386952209167,0.330118811798756,-4.3737
8746828844,1.2210926457412e-05,0.000198407203388849,-12.3092606485608
"Tmem51",2086.33394930636,0.260441343614752,0.23085388053234,1.1281653
2697733,0.25925010416114,0.400479997697188,0.238327648604171
"Pck2",2055.74338453114,-0.341270996046314,0.15661281258561,-2.1790745
6236866,0.0293261296099571,0.0772599479982359,-0.873851548779779
"Dhx33",1386.53328775947,-0.147879972333629,0.121899070746618,-1.21313
453357667,0.22507838872834,0.361051289463199,-0.150650541275265
"Gemin8",454.786731895311,0.223790776690179,0.198825158375853,1.125565
69057087,0.260349397305794,0.401796149174321,0.20405475997872
"Plekhg1",1147.83579393775,-0.306498715716038,0.220575913840221,-1.389
53846038719,0.164669074174179,0.287312537650841,-0.382260500955371
"Aicda",4.08540521463381,-5.11201992927835,2.17031118924984,-2.3554317
7153563,0.0185011942683449,0.0541158544620746,-14.9098608590568
"Mael",0.288091404901093,0,4.78471990595691,0,1,NA,NA
"Dok4",1370.46605324335,-0.398335701545309,0.101222391235099,-3.935252
82978284,8.31091583598107e-05,0.00085648116791733,-2.81331688661782
"Nrl",1.26264602299066,-0.244447814951344,1.70551306859381,-0.14332802
2196213,0.886031125789158,NA,NA
"Erc2",647.224876364764,1.48719966624177,0.492019679797793,3.022642644
80838,0.00250577954923812,0.0118500338712212,6.59636191497248
"Ppip5k2",1186.14360208437,-0.116499209629366,0.152663319776839,-0.763
111989177643,0.445396610515953,0.586502540139127,-0.0621614478819971
"Rimklb",137.29185811868,-0.0591263000361157,0.557389721269669,-0.1060
77126613374,0.915521159055282,0.948362433280434,-0.00313478987903658
"Cyp2b23",6.10942057204285,1.07563270810741,0.848502824038461,1.267683
12094463,0.204911134892103,0.337559759050881,1.16815080357873
"Tasor",3792.25142644519,0.19596932444016,0.105408268716235,1.85914565
172986,0.0630064959028252,0.138531873828742,0.387363714407389
"Oaz2",6184.7039435243,0.319732580654931,0.131396295441911,2.433345472
78985,0.014960019494883,0.0460952054537339,0.983831966303197
"Ppp1r14c",41.5399455265174,-1.44286571785249,0.544261978839722,-2.651
04999788603,0.00802419619960319,0.0285663086450813,-5.13014841394276
"Dnph1",713.630956709077,1.51872367527008,0.469340354371097,3.23586851
444966,0.00121273232546632,0.00681240417226804,7.57692788495496
"Efhd2",3936.57543737184,-0.964651306465104,0.277263358278401,-3.47918
784672764,0.00050293591321341,0.00347739164391314,-5.46134712709746
"Cyp2b9",0.224107459120455,-0.125552415141115,4.77999154655247,-0.0262
66242088162,0.979044980527276,NA,NA
"Rad54l2",1868.41291534958,0.432191977258469,0.203892413148662,2.11970
602821474,0.0340308443132124,0.0866457825044417,1.05710999903476
"Clcf1",2377.2471923374,-0.452037796927863,0.34845466696974,-1.2972642
9225044,0.19454026604833,0.325010840498275,-0.508043807150953
"Sh3bgr",162.167332691524,-1.17827966172602,0.569332251511088,-2.06958
179270312,0.0384915245457991,0.0952058538112383,-2.77097659901975
"Nup88",2347.85546337036,0.314412112850694,0.124383404190433,2.5277657
8111116,0.0114790902719146,0.0377499244597388,1.03025674633703

"Phc1",1519.7513242171,0.344389473550118,0.131859842304454,2.61178435778006,0.00900710477561172,0.0311959368747572,1.19415927967737
"Mthfd1l",239.863332616115,-0.310179105988831,0.296728462914023,-1.04532980403267,0.295870567418685,0.440218027277462,-0.254497352865547
"Kremen2",19.1527252594641,-0.829564692385475,0.467814498709458,-1.77327700332923,0.0761828177486018,0.1600487641916,-1.51999208519386
"Hmgn1",5683.34627395076,-0.566232036596058,0.213432995474243,-2.6529732918657,0.00797861837460245,0.0284400550398535,-2.01576155437725
"Madd",1750.98324287853,-0.236460929121451,0.135260316203181,-1.74819145599403,0.0804308805816401,0.16695122597986,-0.423277730155887
"Tbl3",1625.56593144693,0.196003694177536,0.208110093931576,0.941826945895185,0.346281247050621,0.490967986837769,0.139432393184859
"Col16a1",3112.11765359173,-0.567705040171209,0.276288861835392,-2.05475181445945,0.0399029824970569,0.0977177031810619,-1.32029602101501
"Slco4c1",765.752370397561,2.291420588607,0.790035607833265,2.90040166023833,0.00372684759358504,0.0159250656102829,9.4861626321632
"Apobec2",107.561167324474,2.3617068908194,0.510229467937563,4.62871519429455,3.67941385925996e-06,7.84914785862699e-05,22.3240827818265
"Dnajc16",1444.49453319619,0.0251189805636093,0.104820440963431,0.239638188245866,0.810610757271624,0.879302747617215,0.0032309544416994
"Limd2",1745.60254440357,-0.638913274184671,0.295359625318921,-2.16317065507783,0.030528056385275,0.0797048799410943,-1.61608286749526
"Ap1g2",1508.75188171138,0.30834648319655,0.183469873745016,1.68063822633402,0.09283320607196,0.186469748840123,0.517863681128078
"Cyp2s1",1060.50520512899,-0.00321651159537294,0.200305531601312,-0.0160580268036486,0.987188098952057,0.991714851531105,-2.67602863066844e-05
"Agmat",95.0955734522573,1.88221682295138,0.620686972742198,3.03247354239729,0.00242558359670132,0.0115605057366127,8.39498944209886
"St8sia4",499.087476563937,-1.06135901783691,0.22803647800863,-4.65433875801547,3.25022064981768e-06,7.06271832231083e-05,-10.1445708056585
"Sh3pxd2b",1268.39100785733,-1.27844369247174,0.244770699429416,-5.2230258582907,1.76022798259514e-07,7.00427044992656e-06,-15.1738360799129
"Camta2",3099.79288924432,-0.255282700912773,0.153718389843742,-1.66071672473458,0.0967703528086223,0.192454950944769,-0.420678620199806
"Creg1",2550.40318056556,-0.323325492334868,0.238976405781771,-1.35295989274407,0.176068443741364,0.302385494236688,-0.38671429724759
"Klc3",345.74316945122,0.223677354668896,0.318508210087864,0.702265585578444,0.482513550978692,0.620092882743378,0.106892276601198
"Rsc1a1",29.6426653262424,-1.68661827880034,2.906127606917,-0.580366214747745,0.561667683492419,0.68972140652067,-0.626523911892676
"Il17rd",619.951197136468,-0.568547920875669,0.234953660412786,-2.41983002042529,0.0155277635647305,0.0473506934602932,-1.73416998028444
"Virma",1717.03463532263,-0.356727830999078,0.100031916408879,-3.56614012612691,0.00036227771458848,0.00267970063696745,-2.11256012053166
"Zfhx2",1012.3611593658,-0.0713643036841202,0.24833123804662,-0.287375459670211,0.773824849428632,0.853786279311295,-0.0112808676972617
"Scamp5",2788.57536245068,-0.357477474012857,0.284612331288381,-1.25601541013571,0.209110383007275,0.342708740170907,-0.382813456397864
"Rcsd1",1868.61175622882,-0.678407859024366,0.196851454160244,-3.44629

335819952,0.000568333034755728,0.00381064563674938,-3.77870236289202
"Kcna2",171.050362147947,-0.890913904091347,0.394650132183914,-2.25747
777952388,0.0239782364215558,0.0661658157015984,-2.41935807242504
"Hnrnpul1",5073.50494490409,-0.305483217667312,0.125090910582757,-2.44
208964699486,0.0146025200194562,0.0453520126376802,-0.944951458167089
"Hesx1",54.2673616586612,0.946118782446751,0.301381184533127,3.1392762
0900553,0.0016936572458395,0.00884148031576402,4.47353434714431
"Esrp1",1042.062660165,-0.0663011080796786,0.217701056785731,-0.304551
154039341,0.760708027028133,0.845188726921585,-0.0111515368253286
"Cep126",363.70958972107,0.698715899309164,0.302954331697593,2.3063406
7977816,0.0210916037792422,0.059939380627848,1.96648108458748
"Eif4h",9735.25115663145,-0.310952423280142,0.196399344836834,-1.58326
609255483,0.113360827846599,0.21690965862149,-0.475220606923553
"Erg",811.346044562622,-0.959317301362287,0.278485624343493,-3.4447641
7274249,0.000571557818723838,0.00382175389213903,-5.34056337995638
"Ppp1r13l",657.151428398513,-0.848470172683087,0.284224480391846,-2.98
521144805452,0.00283382359982532,0.0129405701951478,-3.68862898458222
"Ints8",1202.86784899862,0.315933681656888,0.215578886012213,1.4655131
0056862,0.142780980778814,0.258167225786098,0.427820881658001
"Slc25a34",797.390763094671,2.08715204203384,0.434044369411405,4.80861
448534436,1.51979980146058e-06,3.8285602750489e-05,21.2272476010227
"Rnf167",3972.2699069303,0.35367276550388,0.0921516821229038,3.8379415
0422759,0.000124070027057922,0.00117604151073182,2.3857354073007
"Cd53",1615.64212590124,-1.33603083610684,0.378921900119403,-3.5258738
9561236,0.000422087899288529,0.00302072628954978,-7.75199558471025
"Siah1b",163.508090350541,0.116644810365456,0.253411019627285,0.460298
887305754,0.645301699746963,0.758044543758034,0.0323121440425609
"Lat2",865.27505263323,-1.09236869654436,0.355314030260507,-3.07437535
113225,0.00210943849961083,0.0104020819082984,-4.98748162183425
"Myh6",93.4578483181364,1.76254418891691,0.678196681878352,2.598868788
38057,0.00935315170185197,0.0321352866753673,6.0592754335749
"Cmtm5",8.41817284485573,-0.978610777493744,0.60787349254884,-1.609892
17244922,0.107421398121719,0.207932336004892,-1.53694987560494
"Appl1",2173.32561536946,-0.129958277006043,0.12676131023467,-1.025220
36704619,0.305259220228512,0.449916416661541,-0.103796825166677
"Spen",2466.27157441699,0.0288031043881157,0.2033181881049,0.141665163
636295,0.887344492751841,0.930103815375971,0.00208704614637283
"Snrnp25",276.594758678845,0.0592633694016944,0.2139540330051,0.276991
130147482,0.78178691690413,0.858947133837245,0.00901087102663588
"Il25",0.795866050360339,-1.50968635898515,2.544987586324,-0.593199891
071277,0.553047385527147,NA,NA
"Oard1",2286.73082056828,0.868755294009584,0.183492362605058,4.7345583
30798,2.19532629812846e-06,5.15095934547639e-05,8.5778660572405
"Cept1",2403.18002915812,-0.166103915332139,0.14344540096628,-1.157959
15528296,0.246880705032074,0.386144712555381,-0.15805503066079
"Cop1",2723.13061256205,-0.469584127816134,0.223104537077767,-2.104771
75393547,0.0353111810348108,0.0890221628932947,-1.13586292120381
"Ttc3",4871.77705340113,0.0457477175139599,0.246996306855791,0.1852162
00583394,0.853059462703465,0.906427978408292,0.004494425132453
"C1qtnf4",161.910650748985,1.12701845208417,0.266913882957325,4.222404

76814899,2.41709535590279e-05,0.000332828568145428,9.02503191543624
"Iqcc",871.01168580182,0.753389709777171,0.207314205985468,3.634047682
33769,0.000279009263822036,0.00218246903950941,4.61624360559163
"Iqsec3",100.820484343777,-1.76118858362057,0.403540248763521,-4.36434
429779182,1.27504626404672e-05,0.000204784613314521,-14.9587464608893
"S100g",12630.8719391019,2.51549264501453,0.806455264338993,3.11919675
678023,0.00181344819732371,0.0092610301077574,11.7773856187346
"Chil3",28.4777894773133,-1.66448615173728,0.56560204920737,-2.9428573
571646,0.00325198219313598,0.0143552521625006,-7.06347901324192
"Eml2",2448.81845192779,-0.34004352272171,0.119780755758727,-2.8388827
6182407,0.00452717868967148,0.0183964622136746,-1.35867684656097
"Agbl2",160.7250168864,-0.108923366787094,0.233166588762746,-0.4671482
62386456,0.640393806601834,0.754023777888321,-0.0307524840029139
"Tex264",3933.55258168567,0.737621654381312,0.184276316601654,4.002802
24819021,6.25966216196333e-05,0.000688386310400231,5.3707415652648
"Dennd6a",1713.85218429488,-0.307494969772695,0.153129981251376,-2.008
06509123721,0.0446363752291838,0.106565451722618,-0.688479980416352
"Hlcs",850.701781879539,-0.0495519065472328,0.153610711080529,-0.32258
1063512268,0.747012536798825,0.835498792089915,-0.00890578462136615
"1700123O20Rik",1352.11887433675,0.147232857341681,0.183119600452597,0
.804025658519251,0.4213821566641,0.564448288721945,0.0842034288695055
"Snrpd2",3347.82915960213,0.329437914341891,0.192617518289095,1.710321
66372036,0.0872064079071803,0.177619402629709,0.569305680679322
"Catsperd",147.142450093708,-1.19496420217322,0.457924695526048,-2.609
5212026084,0.00906690271077802,0.0313466397568219,-4.13774064663319
"Zmynd15",1884.36813433989,-0.807028464438428,0.479092480142547,-1.684
4941172909,0.0920862154005977,0.185363309699367,-1.36019607263482
"Gpr161",259.323023119919,-0.836481264021867,0.235744612421973,-3.5482
5187913351,0.000387797179727554,0.00282453097135893,-4.90965396850574
"Gm11639",8.53045833515966,5.90727284604476,1.76430940602785,3.3482068
5411656,0.000813362779410437,0.00502024294806733,31.2747384686579
"Six5",1254.53815643763,-0.424986383028489,0.156693715480974,-2.712210
77197631,0.00668360797025625,0.0248744012605567,-1.56986402816584
"Szrd1",5408.05243516629,0.0475528076229489,0.175123758884535,0.271538
299119665,0.785977047338037,0.861583205093441,0.00708459065841983
"Tiprl",2104.9759716958,-0.0205543354934437,0.183537138718768,-0.11199
006172227,0.910831286595416,0.945169934243248,-0.00115907014068949
"Sft2d2",3888.85648099852,-0.179437463021046,0.117049641619043,-1.5330
0309628502,0.125275092015278,0.233966833727385,-0.260646536113945
"Psme4",4544.74579653658,0.323566368063389,0.14945770199877,2.16493605
706617,0.0303925824366784,0.0794256228857198,0.819572337892605
"Plekhh2",747.90488173463,-0.632078514081317,0.305187123120925,-2.0711
1790175651,0.0383477792958948,0.0949225594403144,-1.48835141105873
"Reps2",1494.27114151859,0.718019491072734,0.355976116233479,2.0170440
0472136,0.0436909213090406,0.104963723962996,1.61851679636825
"Dlk1",30.395457589667,-0.824511719235408,0.747432655882007,-1.1031250
9461129,0.269972844445566,0.41230755236153,-0.730505610699793
"Erf",2352.21066879919,0.0558983725545956,0.175519606582047,0.31847366
5951763,0.750125667112961,0.837491425639516,0.0099132551457229
"Bsdc1",3519.0136511052,0.275159178069216,0.111272474744024,2.47284136

262993,0.01340436293008,0.0424489635229331,0.869352430903302
"Crocc",3104.36271858065,1.1283544781817,0.375001026853517,3.008937035
85099,0.00262163441660793,0.012249915808011,4.96728294701759
"Ino80d",2567.36798424246,-0.198721008723156,0.130780032476228,-1.5195
0573004543,0.12863524681747,0.238347433474012,-0.284971066482899
"Rsph6a",0.173370006019489,-0.82915207660533,4.78406116662305,-0.17331
5525811015,0.86240342053458,NA,NA
"Begain",101.337881922016,-0.870350641579846,0.381680187908875,-2.2803
1390978994,0.0225890774093094,0.0632546074000739,-2.40267893618469
"Osbpl10",331.026709014063,1.1197264957327,0.369270986851624,3.0322623
1034127,0.00242728174490071,0.0115657721070562,4.99365010150277
"Wdr25",247.964308098379,-0.163492805777133,0.19380332543239,-0.843601
653441022,0.398892040035639,0.543242089586847,-0.0997633462543197
"Tmem205",5655.54459892593,1.21741618033813,0.301898533610733,4.032534
26168629,5.51785672445393e-05,0.000622703205501692,8.98628515776855
"Gfer",1208.31764326477,0.146068279505872,0.18698358982371,0.781182346
769504,0.43469525494153,0.576812854284059,0.0803722314692932
"Foxa3",12.5376553867652,-0.832188165083141,0.74132084374599,-1.122574
89062089,0.261618084461196,0.403259645593983,-0.755772189421128
"Kcnd3",306.341126527451,-0.773067322690784,0.247527262923732,-3.12316
03079172,0.0017892026664089 3,0.00918183079951073,-3.62609443375961
"Ccr6",10.4033866611488,-1.28210614287844,0.801854787936783,-1.5989255
9371924,0.109837136419678,0.211589568760367,-1.99124786778797
"Gm21988",638.310068192232,0.717151224582017,0.216765830741198,3.30841
453253877,0.000938258181131214,0.00559454373568698,3.71912006686357
"Atp1a3",657.41876114664,-3.43122403294122,0.825815065454193,-4.154954
51279285,3.25352261758799e-05,0.000414893289717009,-26.7206201130695
"Fbxw4",1390.98695760589,-0.320602928264334,0.18735681289724,-1.711189
05849544,0.0870462178507959,0.17733490912987,-0.554551755707436
"Slc19a2",547.624102603265,0.659356785943506,0.179242045417821,3.67858
324985367,0.000234533138655148,0.00191736986897194,4.12546412641695
"4930505A04Rik",5.22610357781631,0.0613196719464742,0.70315998010998,0
.0872058616545318,0.930507880183106,0.956679177759194,0.00271567542169
988
"S100pbp",1904.53600245035,-0.127039516885246,0.120061686804063,-1.058
11870769874,0.290001326505168,0.433756203144965,-0.106112633494646
"Rfx3",422.216628307644,0.404065075006592,0.189364182620691,2.13379884
94686,0.0328592501311346,0.0843234613130906,0.999291375385415
"Ulk4",182.001263871866,0.00645977235359067,0.429571413682501,0.015037
7146798812,0.988002091815756,0.992123643519782,5.10809006500055e-05
"Slc16a11",1278.3960206458,0.824953410956399,0.30536311320237,2.701548
9929515,0.0069017311259721,0.0254956481392192,3.02695824290415
"Arhgef1",7498.10947005396,-0.290915234978795,0.194342914944585,-1.496
91711201176,0.134414827039097,0.246410332607025,-0.407501584944324
"Tet2",3609.55542234554,0.835432613484914,0.235624559311771,3.54560923
498427,0.000391706600001282,0.00284981167418843,4.89605480805968
"Rcc2",3489.8400071848,-0.656681207960163,0.127890356413957,-5.1347202
898912,2.82563950159453e-07,1.01650035059207e-05,-7.54957475451181
"Mgl2",1817.76471677144,-1.86194359145895,0.380496737056582,-4.8934548
1872572,9.90810687126315e-07,2.75589540336503e-05,-19.5488866231963

"Rps19",14168.5589792466,-0.331104762070303,0.209805180466719,-1.57815
34151528,0.114530369399688,0.218504221385041,-0.503593769591565
"Cables1",880.737616816393,-0.575963259772471,0.363145971788007,-1.586
03785947735,0.11273072159246,0.215915875207248,-0.882874736483902
"Asgr2",144.895338809575,-1.80869882040131,0.440308947767709,-4.107794
83263081,3.99454695190141e-05,0.000487089909119285,-13.7950577415724
"Arhgef10l",3096.95658431186,0.126394282879108,0.230057664758623,0.549
402616129836,0.582729179126441,0.706947084034803,0.0438334692599911
"Slc22a2",7950.85559955933,2.29899496107567,0.835590386476324,2.751342
04304397,0.00593516355758834,0.0226465399306962,8.70801402829976
"Arhgef38",467.88255233618,0.866858760157646,0.474442657847712,1.82710
965344076,0.0676832840988691,0.146248701565115,1.66648973777619
"Igsf21",25.8924876017221,-3.86402577217798,1.03193993977702,-3.744428
93741754,0.000180804540087904,0.00156403146523246,-24.963494136832
"Gm11992",821.92176420662,1.61717431957744,0.61387168410639,2.63438493
979658,0.00842898666228376,0.0296283363874542,5.69087534820756
"Sun3",75.4850451816136,2.22063350033153,0.684297146935995,3.245130438
25278,0.00117396835426734,0.00664249418790033,11.1348509570902
"Mill2",1358.01912319561,-0.825568434987806,0.187374269380847,-4.40598
614588752,1.05303693819144e-05,0.000178230158167158,-7.1266657040656
"Sh3kbp1",1320.29464865183,-1.57520631986895,0.276555118017364,-5.6958
1330174498,1.22785182827552e-08,9.32213774251015e-07,-21.8728481879144
"Abhd4",2821.61393289897,-0.4000184327075,0.17871216735281,-2.23833910
490153,0.0251989498419153,0.0689004521129805,-1.07008632484313
"Npnt",9175.72709941214,0.184403548613531,0.410353359411704,0.44937745
5756418,0.653159391399823,0.76373624069048,0.0497028018596264
"Trim62",463.79351290569,-1.18079981583148,0.295286816908117,-3.998823
3413038,6.36581709209398e-05,0.000696915786982866,-8.58305199050334
"Iqcf4",0.148177164577155,2.59891947282788,4.7275994170606,0.549733436
265582,0.58250219767124,NA,NA
"Cmtm8",444.475313234474,0.796351339294752,0.25641681978258,3.10569072
641175,0.00189835151842694,0.00959020717421911,3.7006548567819
"Nrg3",0.132636770796333,-0.978450109585277,4.78042127650866,-0.204678
636670256,0.837823212509415,NA,NA
"Map7d2",41.9103285204028,-0.524452539185429,0.535383808089827,-0.9795
82369247589,0.327292311308081,0.472048788280162,-0.393692326196687
"Iffo2",940.043492316885,-1.2481825614851,0.209935165195117,-5.9455620
9925582,2.7550977455573e-09,3.14145589724422e-07,-18.6895483704358
"Ghitm",18768.5270291239,-0.273809970048634,0.468397224093486,-0.58456
7875222902,0.558838313610005,0.687589413691417,-0.102559193778769
"Gm17018",1349.87412663023,0.153177791286914,0.243404079736498,0.62931
480627909,0.529142979892754,0.661588676543914,0.0632794689348191
"Irgq",1075.76314290662,-0.165635813958261,0.149418228333636,-1.108538
20049594,0.267629450853276,0.409755715173708,-0.147779298198764
"Fam117b",2196.51117783253,0.0760872766084365,0.215828334493142,0.3525
360874749,0.724436259456861,0.818464375181937,0.0152422147025183
"Lrit1",1.97939554397695,3.974868504116,1.29412608674049,3.07146926782
65,0.00213008090282799,0.0104875346425084,18.1157331168816
"Ramp3",1150.17026484993,0.164354698248241,0.434238799946823,0.3784892
05175512,0.705067211426985,0.804176688412013,0.0358188502614757

"Slc7a13",17724.5692094853,2.5045196496613,0.981140002431916,2.5526628
6508902,0.010690291669371,0.0357344945944448,8.34415489417828
"Wdr43",1764.51502226373,-0.00645843358429561,0.149751585603466,-0.043
1276474186871,0.965599780344835,0.979228745789391,-0.00013556259360725
3
"Wwp1",5287.73066705915,0.800080299793461,0.192142072898497,4.16400368
604392,3.12715061711934e-05,0.000403528674603527,6.25283797047459
"Mslnl",5.91345528805336,-0.768117266241849,0.660054660066936,-1.16371
766266138,0.24453844912663,0.383650682416401,-0.735873870734485
"Pif1",58.8395832887657,-1.45070039551013,0.575572689297846,-2.5204468
9139069,0.0117205923685487,0.0383629528289343,-4.7302451837437
"Nacad",111.06470996922,0.265704905104743,0.565671875417037,0.46971560
1308363,0.638558219973065,0.752681989672797,0.0754900755141847
"Fzd7",1176.72060508345,-0.203428990606904,0.184637248917668,-1.101776
54725356,0.270558828757737,0.413008380421072,-0.179889692100166
"Grid1",76.0833905175969,0.960239826812061,0.629002618663349,1.5266070
4156145,0.126858754999658,0.235907678109998,1.3868885405454
"Rwdd2b",763.353349575511,1.23392612097747,0.257324892304609,4.7952069
8493796,1.62506947155867e-06,4.00070764601654e-05,12.495296360125
"Ostc",1862.63478148206,-0.493605479749159,0.229025006555764,-2.155247
09363549,0.0311425034653603,0.0809082654986803,-1.24114101243945
"Tspyl2",2876.10066327411,-0.266277743935025,0.240740159595095,-1.1060
7945256364,0.268692131401827,0.410930988946021,-0.236808783002504
"Elmo1",567.365602268869,-1.10250826248898,0.241886494896425,-4.557957
08214744,5.16535888286138e-06,0.000102040696553223,-10.1322040027985
"Iqsec2",2194.83419525766,1.38832431572743,0.27706731957837,5.01078336
427453,5.42089122285319e-07,1.67820983167894e-05,15.2649004842787
"Ccdc8",180.668624920947,-1.47234050634739,0.235444909019464,-6.253439
5518642,4.01510013546435e-10,7.60062353798873e-08,-24.1352688040038
"Pde9a",1115.51368814826,1.52519511412524,0.399704629382744,3.81580547
736103,0.000135739410233853,0.00126211112100127,10.1806308183587
"Nbl1",2534.22665460819,-1.68371207099024,0.300895647925525,-5.5956677
4261546,2.19774238872724e-08,1.46251130018443e-06,-22.6212702860084
"Msantd4",2057.87013950033,-0.0398268634303911,0.183705851231036,-0.21
6796923796963,0.828366609405016,0.890625428738848,-0.0046131987255248
"H2az2",2583.00311583074,0.494677236639571,0.243918575910634,2.0280424
9242915,0.0425559113661442,0.102781513664942,1.12546480131026
"Zfp598",3788.17408964413,-0.0233487896820511,0.12347727721234,-0.1890
93817171713,0.850019287253201,0.904369055346608,-0.00234696791696113
"N4bp2l1",794.551540473752,-0.770845509453116,0.223496627672764,-3.449
02523800834,0.00056261411496028,0.0037814029691470З,-4.29951430482221
"Smc1a",3418.48544879241,-0.0510960290710694,0.11830838816717,-0.43188
8472682687,0.665822473221371,0.773579226723292,-0.0131177398583045
"Cyyr1",1242.12745824712,-1.15273796408222,0.342060697542188,-3.369980
74425096,0.000751734365308602,0.00471646757847466,-6.17486586598787
"Ripk2",468.284650212478,-0.702905221318362,0.168858679623389,-4.16268
33923259,3.14529343569266e-05,0.000404545737646677,-5.49161978458597
"Pnmal1",1.49523501082598,-0.0709785959133749,1.79875283573764,-0.0394
598938237491,0.968523728687993,0.980644735003297,-0.0013872788413974
"Tmco4",998.03733347505,-0.310107856613525,0.197685694477475,-1.568691

43937403,0.116719854280957,0.221856474826487,-0.466937033311171
"Dnah7b",501.42525026382,1.46547429855828,0.422046102809484,3.47230856
72463,0.000516002873080419,0.00354761072613611,8.26744531894439
"Brca2",278.081407959561,0.504693273073502,0.259274701945281,1.9465581
0723877,0.0515877351817612,0.119149213524518,1.07367370714634
"Osgin2",452.698699539809,-0.533563750853764,0.208338347191181,-2.5610
4436867852,0.0104358027617324,0.0350762424148005,-1.78756194235135
"Otud3",256.22983133028,0.358508167647059,0.148254759606801,2.41818993
60121,0.0155979326297525,0.0475275027996427,1.09217603471727
"Zmiz2",4476.4127317385,0.0495415573479746,0.154574144141229,0.3205035
20321679,0.748586662173851,0.836443709975114,0.00884792660868483
"Spem1",14.3708147793902,-0.439723949374799,0.581438385859465,-0.75626
9211095881,0.44948780394672,0.590295224715643,-0.23179277874473
"Lonp1",6676.45071227367,0.135761718459457,0.285314584405194,0.4758316
81519135,0.634194297153937,0.749016924459838,0.0392342812878501
"Hectd2",208.896458306805,-0.423628679157926,0.154689147850226,-2.7385
8046957563,0.00617050577582298,0.0233661918075347,-1.59134634687487
"Prkd2",1371.13124466679,-0.603206222844602,0.166980993484834,-3.61242
444577615,0.000303347506777429,0.00232494877639399,-3.65787708206237
"Chrnb1",1256.84962001422,-0.405116008321999,0.295592793983217,-1.3705
2058293748,0.170524454733822,0.2950790714665,-0.494448917013757
"Pla2g5",299.621368210089,-0.368048549578016,0.583661971044599,-0.6305
8511233704,0.528311835720646,0.660745617247028,-0.152514298217115
"Rpusd1",329.695610565062,-0.228317995337519,0.132894615880856,-1.7180
3796432365,0.085789690810012,0.175356682190336,-0.397486377188606
"Pla2g2d",131.745102391682,-1.67924540603566,0.547694481583991,-3.0660
2579083707,0.00216924602570877,0.0106404928325206,-7.62896045471182
"Trir",5615.04523558209,0.423492494304972,0.166107640205198,2.54950641
512827,0.0107875526374396,0.0359809069545836,1.40801380357464
"Map6d1",6.66818828552818,-2.57172279794317,0.99666603706698,-2.580325
50754044,0.00987072258342856,0.0334130814117518,-8.74079149645478
"Yeats2",1820.94971047009,-0.0114361516492809,0.180376441388303,-0.063
401581499559,0.949446727932371,0.968673135954443,-0.000363990349243998
"Clvs1",5.14565242506682,-2.52397308640189,0.896558671896948,-2.815178
93420366,0.00487500819782092,0.0194980327879205,-9.93799688303507
"Arhgap11a",487.415924137944,-0.677974254515064,0.29921416530268,-2.26
584945879563,0.0234606019838645,0.0650782212948342,-1.85233775150436
"Elovl6",8692.09368628612,-3.13002578973961,0.803037357031656,-3.89773
372599927,9.70970936151867e-05,0.000967196566763314,-21.7258495794795
"Arhgap12",2228.02901220515,-0.221223648569527,0.110347360854604,-2.00
479328962852,0.0449851502279653,0.107141676447874,-0.494125858516497
"Phf8",876.137664032541,-0.365423764754766,0.195318298144255,-1.870914
13465459,0.0613569835222842,0.135870353744179,-0.72940561454673
"Ublcp1",1971.81858094563,0.367971197683602,0.100822201234663,3.649704
06495242,0.000262542601022513,0.0020860047411316,2.27130392861068
"Chd7",1922.74603635687,-0.351004491899766,0.249375721263795,-1.407532
73863603,0.159269476401667,0.280395360342963,-0.446321401050173
"Vps41",2888.81444005106,0.00677732712250693,0.132114751679598,0.05129
87916666806,0.959087430948913,0.975090558381579,0.000170957615170221
"Pklr",1533.51250795775,0.323897572372625,0.570596407696739,0.56764740

8927906,0.570274408620115,0.697122074563815,0.11686054064114
"Rbbp8",1570.11315479478,0.162703675809663,0.193453677558029,0.8410472
10182178,0.400321487014593,0.544428287215633,0.0989269342675773
"Mul1",1808.63583988977,0.296241445389269,0.241352766977686,1.22742096
185149,0.219664411794898,0.355437560372348,0.306433834965257
"Wnk3",26.4109311906703,-0.619615165432212,0.372579956459041,-1.663039
44882319,0.0963045391836436,0.191665398081319,-1.02360682195885
"Lamp3",0.949892507590736,-2.28680209506637,2.69506130334092,-0.848515
798965888,0.396150777577819,NA,NA
"Kcnj1",5094.79357448731,1.85166153596295,0.59353625278726,3.119710931
33319,0.00181028597386632,0.00925457680085094,8.67065893188483
"Zfp236",1742.3255425873,0.49703981871048,0.299020077316742,1.66222891
509717,0.0964668844653793,0.191968903174726,0.820325393374684
"Car8",279.803607168484,0.163584743953663,0.309980457246721,0.52772599
0879036,0.597689531041563,0.719864563965589,0.0537690277847985
"Rusc1",1039.50571158307,-0.361015514664006,0.126352602270956,-2.85720
680203982,0.00427387281624682,0.0176363962267049,-1.4577050316411
"Uspl1",1810.6871634434,0.0727462905330591,0.101252187657946,0.7184663
58265888,0.472469783802156,0.610371067883783,0.0359139851392207
"Dmxl2",2066.57669806239,0.911059518713036,0.223774780332602,4.0713234
8586782,4.67467785052366e-05,0.000549408491437675,6.83902163457816
"Tox",158.064005441148,-0.680394189306751,0.364033028911204,-1.8690452
1092979,0.061616524608467,0.136284719907577,-1.35603177882308
"Ttc1",1589.58097895038,0.220356325476282,0.131841678701507,1.67137075
048304,0.0946484695944382,0.189351392930196,0.366706148337434
"Sox15",63.4218511227637,-2.45420698347173,0.913616705721272,-2.686254
49611739,0.00722580279311907,0.0264133301200292,-8.91830954561939
"Adgrf1",62.4805318262161,-1.2201760712238,0.751650888670749,-1.623328
18282382,0.10451926799457,0.203946922211882,-1.93995245000511
"Cdk13",2154.35572468583,-0.26670512390685,0.139856106533123,-1.906996
63045235,0.0565210238321513,0.127737380999939,-0.548820158799576
"Katnal1",903.955724387108,0.274551561245321,0.223048549987933,1.23090
493643727,0.218358421117748,0.353793625972566,0.285270469681924
"Cftr",379.752146125789,-0.653054652203843,0.550211160803638,-1.186916
40360401,0.235260596314335,0.373202466609375,-0.643673000125884
"Gtf3c3",895.556171664397,-0.0623923063043094,0.137862207891012,-0.452
570049898186,0.650858369236445,0.762051070880455,-0.016954591593473
"Sntb2",1546.61192038644,-0.115613455632309,0.170767006313237,-0.67702
4550165385,0.49839037800577,0.633936694523877,-0.0526973276191229
"Nkx6-2",10.1461022621978,-0.440391359611394,0.889777362202216,-0.4949
45565395614,0.620638545562451,0.738734531553426,-0.133357835835047
"Slc7a1",928.328910650353,-1.00303354073394,0.173256435693708,-5.78930
033229562,7.06802203927513e-09,6.04439884744678e-07,-14.3624007774852
"Thoc6",590.524719646365,-0.220840450797182,0.110432099825986,-1.99978
49460906,0.0455234908277052,0.108114132053422,-0.491274562755604
"Ak7",82.7003865074854,1.28039450696005,0.291601710721698,4.3909019044
8865,1.1288146113401e-05,0.000187795454709123,10.9859861404481
"Inhba",594.890237622257,-1.4530721722808,0.45093444217327,-3.22235792
25347,0.0012714020307582,0.0070703905960120,-7.19538025699225
"Pcf11",3742.79788232978,-0.121705768089337,0.107534311522756,-1.13178

544006935,0.257724650450351,0.398660470803725,-0.111926122359275
"Atp1b2",1239.05712179901,-0.840630537705257,0.211974316975827,-3.9657
1881772413,7.31750858950698e-05,0.000776698269304374,-6.0193001722206
"Atg2b",1711.27898349151,0.191742976994975,0.122843965122353,1.5608660
6944019,0.118555354566222,0.224426437216718,0.286503755827726
"Ankrd42",270.878145638178,0.573446324415012,0.153348440168615,3.73949
890709339,0.000184387449786163,0.00159008690664777,3.69526896484925
"Wrap53",612.170180321046,0.541867538503708,0.179903300718985,3.011993
31161869,0.00259538315921326,0.0121704619620827,2.38895494143088
"Bdkrb1",3.11973212917265,-2.4521531337248,1.18080757393371,-2.0766746
3171477,0.0378315995193594,0.0939669461692319,-5.79888163475825
"Rap1gap",3669.76118770602,1.047731157905,0.307383627320287,3.40854575
449879,0.000653101317413469,0.00423907106755254,5.72418606052711
"Tmem29",496.046028073415,0.218349604500372,0.164534105369094,1.327078
07910436,0.184482855811146,0.312732283307748,0.253811278886261
"Rgl2",4542.33120027864,-0.412971449512617,0.148558022459023,-2.779866
36249502,0.00543812726973758,0.0211768734781992,-1.59194116120753
"Ssr2",4362.36543278603,-0.0455612631119344,0.195240872438401,-0.23335
9247697016,0.815482455256696,0.882417276907435,-0.00569926896302839
"Nutm1",2.21372199736215,0.708632838312437,1.29927597400872,0.54540594
3377877,0.585474353676897,0.709262438520792,0.243436403592908
"Pum3",1744.0318325002,0.547891261656621,0.182306061232213,3.005337606
18493,0.00265286213908398,0.0123394484712482,2.40795686781645
"Myzap",586.862788847987,-0.92267917610554,0.267475864979655,-3.449579
18418442,0.000561461038628446,0.00377625640951274,-5.14764754048474
"Shtn1",1413.65557996154,-0.248058614541147,0.173231756726207,-1.43194
653930113,0.152159121759096,0.271064281046949,-0.323815538505254
"B4galnt3",773.942621154975,1.6686009491086,0.585152773358413,2.851564
62564787,0.00435046418427043,0.0178692544059205,6.71557431213688
"Ccdc9",4271.08846696334,-0.0836341743837191,0.161099051121876,-0.5191
47529431737,0.603657865953025,0.724800090992299,-0.0269184450789287
"Ninj2",0.135959949781025,-0.534897781702922,4.78471990596623,-0.11179
29141549,0.910987605989865,NA,NA
"Cldn5",2706.26674635086,-1.35680936714304,0.395688475094968,-3.428983
7903856,0.000605845710080336,0.00399215263776492,-7.49423435179313
"Htr2c",0.151288805160611,0,4.78471990595452,0,1,NA,NA
"Mdfic",1862.28808226711,-0.908850496606135,0.147307946961109,-6.16973
160888655,6.84060070115914e-10,1.1803365705416e-07,-14.4982521633099
"Mettl18",233.130264196233,0.11294135856018,0.199702591643197,0.565547
785989522,0.571701231028001,0.697924876461833,0.0406186603701192
"BC055324",93.6090396906398,-0.870549118291372,0.305765926251909,-2.84
710964678961,0.00441181638673684,0.0180376590288519,-3.49551024949394
"Wapl",3279.4369090434,-0.169286642406764,0.17364311531885,-0.97491134
0976082,0.329604240057486,0.474359571349341,-0.126252226725681
"Dicer1",2941.44331900548,0.44553569625676,0.156312378520433,2.8502905
5583413,0.00436793071812895,0.0179183490726022,1.79191338432706
"Pik3r1",6174.2524410049,-0.800386307351606,0.182964137377975,-4.37455
295240801,1.21681584503628e-05,0.000198042365162917,-6.82491772196617
"Meis3",812.325624959791,-0.986043588420055,0.260519517794722,-3.78491
25346417,0.000153762647422476,0.0013847871129069,-6.49034484429097

"Paqr6",157.779700452104,-1.37275451524829,0.322311559133634,-4.259091
78975219,2.05259176166596e-05,0.000293843128993854,-11.1638773732862
"Hibch",2417.17338627504,-0.72666938409613,0.55669741763223,-1.3053219
9554083,0.191783227361243,0.321640234568168,-0.82427680896961
"Nthl1",415.457337923318,0.0350911390271091,0.168399685587681,0.208380
074491517,0.834932213102191,0.894527627400765,0.00391124048234064
"Ccnb1",126.901783360756,-0.638894832681206,0.496429491507913,-1.28698
00114827,0.198101255210788,0.329178308684162,-0.709911637172006
"Utp4",1030.30472192995,-0.191457685532998,0.193447189727068,-0.989715
517724108,0.322313188048854,0.467489588676379,-0.145580250438129
"Mfsd6",1171.21753207855,-0.136162576076117,0.187707632601284,-0.72539
7119920765,0.468208439454204,0.606385131350254,-0.0681139622219625
"Gk5",598.627171077023,1.14044627567474,0.280015183625632,4.0728015563
5942,4.64509998018352e-05,0.000547582584121029,8.56474841583996
"Arhgap32",2707.49069359666,0.339263516416278,0.177147963409505,1.9151
4206478354,0.0554743885567893,0.125801305894426,0.703310759939527
"Mmrn2",1763.46048702045,-0.543232883226964,0.233461730695252,-2.32686
051632193,0.0199726902590672,0.057393110326336,-1.5524677804343
"Serpina3h",21.460501070073,-1.64970843861189,0.627198745710961,-2.630
28019410636,0.00853145199327017,0.0298754042681002,-5.79166397545868
"Rpl21",27625.0718029587,0.00879763452777654,0.163614731424152,0.05377
04303958407,0.957118068550781,0.97379921209576,0.000233578466533086
"Tardbp",12702.9306822563,0.102261057976141,0.0764568862125716,1.33749
964250214,0.181059598892195,0.308835627609135,0.120151230545822
"Cacna2d4",19.7604926070521,-1.12222242206559,0.816179256409639,-1.374
97052669806,0.169140582334434,0.293407746828895,-1.37606016735576
"Gpr12",8.82642099449181,1.97859336877066,0.732993899916653,2.69933128
910846,0.00694789737976703,0.0256135570260347,7.25081953483424
"Shld2",298.702886890176,0.058825306615305,0.243294402338485,0.2417865
18924771,0.808945585374275,0.878121647011892,0.00764553361968167
"Smpx",18.8096347066692,1.15152629104215,0.477395201859383,2.412102774
71815,0.0158608099015765,0.0481329294102629,3.49348748749424
"Dcp1b",860.58166773208,0.0864549577683238,0.150983604743774,0.5726115
6212982,0.566907729384072,0.694101532654986,0.0315679063804024
"Syt15",181.13223428368,-1.37282709860206,0.397656074571036,-3.4522975
6664216,0.000555834376080395,0.00375135294732279,-7.66811619522872
"Serpina3g",1588.0598818845,-1.25367955193929,0.569433400691941,-2.201
62630154097,0.0276917164772184,0.074023888806141,-3.26378849583621
"Piezo2",854.890906541632,-1.06468084162481,0.365312858089969,-2.91443
571735054,0.00356332185529262,0.0154120784238011,-4.44249128694729
"Zfp281",1484.68502285938,0.288116064120612,0.14709007104195,1.9587730
2988344,0.050139373424561,0.116660799884498,0.619012955914513
"Stx3",2540.5421719776,0.811860397447366,0.269469845720705,3.012806109
25509,0.0025884423731705,0.0121437558691238,3.58106763266001
"Cep78",336.80042617217,-0.103957008018832,0.132835474655673,-0.782599
740681491,0.433862193828996,0.575955179453161,-0.0573557254458762
"Kif14",45.3789794093872,-0.524793824653196,0.492504518256269,-1.06556
144197671,0.286621937041299,0.430750310500328,-0.441995362058968
"Rrp9",850.574217729458,0.183685074597958,0.0975018787432795,1.8839131
8162799,0.059576719918019,0.132833263003034,0.370797822693755

"Irf8",3371.88409815936,-1.0250746806544,0.388551901792717,-2.63819241
631521,0.00833492738791118,0.0293771536893514,-3.61598988076977
"Upk2",1838.35590877254,-2.19956820144449,0.660367592200208,-3.3308239
6444681,0.000865893414610613,0.00527599681189929,-11.5358282210915
"Rnf123",3007.68471599202,0.447739200254443,0.219607809316523,2.038812
74371765,0.0414687199560885,0.100629384157289,1.02814843944956
"Ago1",2481.28010293218,-0.271776550956603,0.142487063780938,-1.907377
01897231,0.0564717831072783,0.127677043950106,-0.559384438655119
"Serpina3a",0.157502682527355,-1.19648096621745,4.77621924155054,-0.25
0507965758505,0.802194545266423,NA,NA
"H2-
Ob",631.073793801629,-1.28740653050828,0.423925027356311,-3.0368731436
7077,0.00239046034632937,0.0114406469888881,-5.75545741421191
"Sox5",396.480136340123,0.108470401446857,0.329731642568188,0.32896570
2539228,0.742181611928218,0.831970466191134,0.0199540345724891
"Disp3",2.38576903553268,1.33215089628142,1.10454502005667,1.206063014
26543,0.227793194380214,0.364237436612871,1.34540489931352
"Hspb8",1290.64505427774,-1.72266625719336,0.389366840549117,-4.424275
7158363,9.67663738257065e-06,0.000167070244478723,-14.982194173714
"Ptchd1",47.0155419159774,-0.425310352480567,0.41498138263397,-1.02489
020057005,0.305414998939446,0.450005390814202,-0.339608494724925
"Fbxo2",120.994956409901,-0.0648854225164549,0.311829129816432,-0.2080
8005510791,0.835166460039931,0.89472943065157,-0.00721745980223653
"Fmod",764.825685717723,-1.30840258535613,0.376193816183104,-3.4780013
0962093,0.000505167456780006,0.00349034552526455,-7.40262067175707
"Nop53",5224.61662592364,-0.30942123191744,0.179226022714562,-1.726430
27631221,0.0842700328522249,0.17304866777806,-0.542781281508789
"L3mbtl4",2.20798850960882,-0.676739010640045,1.26140425735566,-0.5364
96532886867,0.591615431472184,0.714675264254208,-0.22733491599862
"Tssk1",12.8631644673243,0.679080403134322,0.344826229707269,1.9693409
1617917,0.0489139556142864,0.114440363309255,1.47204356512547
"Serpina12",1.77107832059974,-0.462798349196899,1.31335649437313,-0.35
2378315544703,0.724554562100476,0.818550602690485,-0.0926615134253748
"Camsap2",1852.18018804691,-0.266178377847341,0.147786041656654,-1.801
10634849903,0.0716861193117436,0.152791424829512,-0.500064400563459
"Selenow",6140.5445525747,-1.04424907105597,0.277499858484786,-3.76306
163454573,0.000167845813975013,0.00148084057958588,-6.80343452470032
"Prelp",1786.65433936033,-1.7357743141157,0.34879168140511,-4.97653587
127744,6.47322847593052e-07,1.9358130187683e-05,-18.8373138025696
"Crx",3.41786847328508,1.01638797149714,1.03376930818232,0.98318644542
1043,0.325515684659076,0.470454734228942,0.766412972959459
"Sdk2",319.344444971116,-1.82559995668599,0.360680929675503,-5.0615372
3826886,4.15889486606411e-07,1.37208345344362e-05,-20.4405035503865
"Tmtc4",929.072100115974,0.324350273673806,0.16184066296533,2.00413337
248432,0.0450557753817807,0.107238465380122,0.72417694550713
"Nlrp5-
ps",98.2917195520533,-0.593474111757088,0.368303606334124,-1.611371981
02451,0.107098670410984,0.207513651562493,-0.93327255174004
"Cdc42ep4",1837.08456872749,-0.900637625578674,0.161258003188644,-5.58
507241668548,2.3360285309296e-08,1.53359879784732e-06,-12.057640549477

4
"Inava",909.695762661369,-0.474959770253726,0.192491391317529,-2.46743
382653536,0.0136085371865687,0.0429908065560137,-1.49458867518791
"Mbp",1064.64413021475,-1.08222598182333,0.200972999636857,-5.38493222
362619,7.24719515661928e-08,3.55039726542117e-06,-13.5802597633437
"Entpd3",234.520530436583,-3.46125936115326,0.755387509889509,-4.58209
768607313,4.60334751153887e-06,9.33015278378221e-05,-32.1193587595263
"Bicdl1",1394.73402235001,1.25849824159616,0.277132382364286,4.5411446
7194198,5.59496204847127e-06,0.00010818090741651,11.4922355203281
"Nppa",2.96016599731382,-0.997710155262744,1.49456199732626,-0.6675602
33063351,0.504414349174075,0.639180540728934,-0.446543466177675
"Ccdc74a",27.7152541792741,0.0866009123221336,0.363107440390136,0.2384
99415569911,0.811493768583971,0.879810136779931,0.0110891730776019
"D11Wsu47e",400.768432440938,-0.156106976530428,0.154169752686793,-1.0
1256552475355,0.311267733944315,0.456421067407232,-0.122440868245634
"Gucy1a2",112.7394144532,0.140111001989618,0.40297246334703,0.34769373
7745443,0.728070200144386,0.821454818160013,0.0275567995972467
"Ggact",2384.62976148528,1.31199084240024,0.310365292725673,4.22724728
94058,2.36567631268335e-05,0.000327425646559507,10.5277419425713
"Fam104a",1856.77439617472,0.207882027217492,0.137282145748086,1.51426
848760769,0.12995775753825,0.24002431333384,0.296650782547672
"Mrps27",1174.79666941186,-0.0139238252612786,0.246456546309692,-0.056
496065816739,0.954946629544866,0.972249458663558,-0.00039185630305653
"Kctd12b",859.535574682783,-1.29616485546412,0.361095856629132,-3.5895
3123296389,0.000331273139942783,0.0025022197651844,-7.76477541670402
"Gcn1",4924.00792476678,0.174467890345469,0.144019227260103,1.21142081
973801,0.225734164062655,0.361863846560386,0.177344386485667
"Kif21b",699.498488606619,-1.29298193662906,0.384028356387459,-3.36689
183265552,0.000760204998978339,0.00475938325583626,-6.91439828674082
"Slc5a12",2345.84762826523,2.13757972217668,0.95741304855122,2.2326619
8994396,0.0255712428198694,0.0696936109172231,5.6937570252139
"Ddx24",3666.22381671193,-0.0115665829214053,0.264879344467672,-0.0436
673646435159,0.96516955363232,0.97904389433121,-0.000244966364557766
"Klf8",83.6090144328371,-0.274763258261831,0.2872593635892,-0.95649887
554148,0.33882025219936,0.483448026153174,-0.199701086236996
"Pcca",3544.59132253214,0.940270740013609,0.468740113965632,2.00595321
799693,0.0448612394958552,0.106945158049906,2.10191799327107
"Pnpla3",1496.18914216698,-4.48248459852378,1.28867818396002,-3.478358
40966083,0.000504494882617897,0.00348693414437567,-25.3651808189962
"Slc39a11",863.755821408116,0.454901292214778,0.282640701631301,1.6094
6845089632,0.107513948195017,0.208070169916272,0.714140395498499
"Rragb",88.4042201420886,-0.90935609169865,0.263533732918063,-3.450625
01725873,0.000559290064576618,0.00376601658020649,-5.07578694892162
"Bbox1",29.2563778275864,-0.54716699075195,0.738763081930347,-0.740652
861702608,0.458903947545328,0.598649428034514,-0.280739954959254
"Prima1",86.6770018234278,2.47491612663868,0.573033634894837,4.3189718
2979997,1.56757787373814e-05,0.000239910780079641,20.6290284422952
"Rims1",28.8259383494789,-6.47618858101835,2.21586633321736,-2.9226440
6202478,0.00347073022353166,0.0151223012063509,-27.1454931235059
"Pyroxd1",1019.85173920701,0.425521760102899,0.108870979674329,3.90849

573849507,9.28725852805684e-05,0.000932936459454159,2.96893913248284
"Lrrc18",33.2530455921745,0.0788963255319583,0.407738467737136,0.19349
7380734804,0.846569457166174,0.901940400842908,0.00814264009515463
"Lrrc29",209.401341722006,0.651445109622237,0.238093020204251,2.736094
94752676,0.00621730840825484,0.0235114583677566,2.44309334874256
"Iapp",0.452010276738984,1.25333708683673,3.07652650435611,0.407387059
744849,0.683723729586044,NA,NA
"Bivm",867.329853237911,-0.0208554310463793,0.195373424404278,-0.10674
6509203954,0.914990084364295,0.948003436151497,-0.00111362062372176
"Fcho2",5389.30883674337,0.265010848824319,0.113744470527177,2.3298789
6111399,0.0198125492852097,0.0570192008801503,0.759088385752738
"Amot",1152.16239233207,0.225206980609704,0.304117618362599,0.74052592
4878149,0.458980936490304,0.598649428034514,0.115548998133076
"Kcnj2",220.767481060398,0.355942545157423,0.263972987653347,1.3484051
846428,0.177528097338385,0.304222432932311,0.423570261153114
"Rasl12",1174.40923506717,-2.08547608112159,0.506066967501177,-4.12094
883690811,3.77315218921807e-05,0.000465920971408321,-15.9987183435002
"Cox6a1",19145.3998506225,0.561283289024446,0.430589175132955,1.303523
91894463,0.192395955859402,0.32236283813239,0.635416205829578
"Slco1a1",4372.70723246914,1.97548450278446,0.983750341762253,2.008115
69655534,0.0446309986528369,0.106565451722618,4.42310172681928
"Lhfpl1",14.9757758517394,-0.869037883605419,0.488900761814768,-1.7775
3432082946,0.0754803691802225,0.159018502244838,-1.5979301271579
"Btbd7",2298.74592427523,-0.333656841214277,0.100040470875097,-3.33521
861998088,0.000852323628045976,0.00521286264104155,-1.75390926759604
"Zic5",4.00487613380367,-1.31936488688461,1.11651583773467,-1.18168040
460716,0.237332536401848,0.375487649688675,-1.29235709003128
"Tmem273",261.967967877653,-0.959948444768893,0.245153531415102,-3.915
7031074681,9.01411207851405e-05,0.000909716135128711,-6.72192181946084
"Mpped1",159.749695948782,2.22680525497272,0.826603853875681,2.6939207
2699872,0.00706169580142629,0.0259344087403786,8.13270401347794
"Trpc5",1.55666413691378,1.15434312631883,1.38907287989463,0.831016963
203826,0.40596404657888,0.549801137750574,0.690526481173325
"Ubr7",1189.42303419698,-0.113448427438775,0.109316577079312,-1.037797
10698832,0.299364523992836,0.443744069394277,-0.0921776758109463
"Gm20604",339.184911448261,-0.152017947853775,0.230161179619053,-0.660
484744236124,0.508942805351728,0.643288397429534,-0.0670645626989084
"Alg13",508.0804413146,0.100245008427113,0.182682849981315,0.548737927
163749,0.583185314101643,0.707324412438034,0.0347114241104772
"Pi4ka",4926.75946575559,1.09918714869903,0.20261650476544,5.424963528
86704,5.79663746286204e-08,3.05467127705092e-06,13.9584001625705
"Khdc1c",0.47942708610266,-2.10927791024867,3.10517581702456,-0.679278
093911547,0.496961673775543,NA,NA
"Coro2b",646.383022391818,-1.10326483784045,0.28214603940383,-3.91030
926358633,9.21780244686223e-05,0.000927392735340143,-7.70424555957202
"Prrxl1",2.86600031593428,0.131626373835768,1.36495079765842,0.0964330
538958422,0.923176641231877,0.95268858636282,0.00637956187110936
"Pgm5",404.763092332279,-1.17593115428732,0.411620189073923,-2.8568354
6507514,0.00427887579617565,0.0176508597274554,-4.74719971740257
"Coq5",1606.80448358433,-0.108565951813981,0.292638431751654,-0.370990

06840672,0.710644928979445,0.807944418794408,-0.0231529932586022
"Kirrel",2151.93566876175,-1.10523759256647,0.288369391754074,-3.83271
465062086,0.000126736931177773,0.0011978267979177,-7.43520557790473
"AAdacl4fm3",0.145470086866329,-1.10220209604883,4.77919322734928,-0.2
30625137678343,0.817606036952608,NA,NA
"Tspo",7240.5436823208,-0.686081041405466,0.375456231945237,-1.8273262
8474665,0.067650725459366,0.146220357499913,-1.31908719454986
"Tmem45b",2790.44001901377,-1.38332364204203,0.64840761301444,-2.13341
671855297,0.0328905567907422,0.0843815889876174,-3.4201377294273
"Rnf10",10283.7799593449,-0.399383843752143,0.236295223238721,-1.69019
008627466,0.0909915943958624,0.183564832646718,-0.677030444699749
"Pde3a",1646.37357503361,-0.91250284182871,0.267597329968312,-3.409984
85275157,0.000649664875557588,0.00422520538479715,-4.98836779479521
"Utp15",917.599109706417,0.0277120717523766,0.121533305515054,0.228020
390253797,0.819630388907462,0.884451454032746,0.00340270022522968
"Cd1d2",3.63978171112918,-1.33113680307434,1.04843291752341,-1.2696442
2885417,0.204211387194891,0.336663027608521,-1.44917238660118
"Trem3",1.56148832621044,-1.92514658927195,1.45088331015398,-1.3268789
9557383,0.184548713561436,0.312789535554667,-2.23745296526221
"Plekha6",8080.02045244233,0.481552143282285,0.263984975858943,1.82416
496134082,0.0681271370168297,0.147021905650708,0.923219097187832
"Gpr155",601.058616190396,0.370578243437908,0.201010968137981,1.843572
25314955,0.065245502831759,0.142124751804153,0.723016708998378
"Tpp2",1768.07388291933,0.0199096749287464,0.127691227214005,0.1559204
60341247,0.876095717531644,0.922559640334414,0.00160478459306839
"Ubac2",2164.82283132612,0.356432571245425,0.121138534724276,2.9423549
8272043,0.00325726305300571,0.0143753089650306,1.51207365137854
"Ppp2r2d",1831.54078157467,0.0742503613081813,0.161509965646322,0.4597
2619095701,0.645712767690004,0.758303170777552,0.0205429968441001
"Slc24a4",1.41209834216381,0.659045223095305,1.36048497687036,0.484419
331561722,0.62808830162866,0.744375644174763,0.194556393311344
"Enc1",975.94871225306,-1.53039641097811,0.256895582157745,-5.95727025
791507,2.56485902201103e-09,3.04936714702263e-07,-22.9607846271662
"Ydjc",202.943439010571,-0.194972081829887,0.214597761999585,-0.908546
668954842,0.363589468601087,0.508592256172178,-0.131822311400708
"Mapk1ip1",1523.95426818595,0.222109929218246,0.237119065731207,0.9367
02110112162,0.34891181052973,0.493961072001776,0.156653814686392
"Cir1",4396.26006754909,0.704615469357173,0.180672650022779,3.89995646
41817,9.62099867607457e-05,0.000961015533740277,4.895330055087
"Tram2",927.931780656365,-0.455413547820976,0.218117260745611,-2.08792
988809869,0.0368041596354268,0.0919773781814344,-1.08671355581299
"Cpsf2",1515.55055025913,0.0503904245767842,0.0763260790655046,0.66019
9307939532,0.509125936348233,0.643436447168644,0.0222187515976194
"Lad1",4550.31157227968,0.928447640560565,0.229755233284148,4.04102934
801192,5.32170972963901e-05,0.000606090515820686,6.87838690606855
"Myrip",126.789838452266,-0.990325896432772,0.360994638762438,-2.74332
577300264,0.0060820313614487,0.0230939527722545,-3.73173067355233
"Abca9",642.312663646154,-1.13011430646425,0.315997801866464,-3.576335
97382369,0.000348443777527272,0.00259608589003697,-6.72841849868429
"Gck",256.716382851656,-5.40289578670443,1.16955981547707,-4.619597659

9115,3.84484877981067e-06,8.12208683735086e-05,-50.8863005573197
"Phlda3",3516.87379837799,-2.87308603383217,0.583884494660706,-4.92064
108587386,8.62611914561558e-07,2.46615940031103e-05,-30.4841996561869
"Efhc1",83.5858548066265,-0.965897760354018,0.362623396452959,-2.66363
883246932,0.00773005069521172,0.0277518925529807,-3.46221342578762
"Poldip3",3550.64310000941,-0.155798708141508,0.105399265203496,-1.478
17641651206,0.139360564546916,0.253427745526559,-0.213861229149024
"Fam169a",33.3049583046809,0.224610078869255,0.38998075395778,0.575951
701692367,0.564647827878469,0.692378048423017,0.0825718669143553
"Oasl1",343.996457661056,-0.645266660772839,0.362858483755916,-1.77828
737554581,0.0753566687081079,0.158843710969804,-1.1871838706742
"Abca8a",959.458106796092,-1.12722807340924,0.335573346024477,-3.35911
086730654,0.000781936900677482,0.00487099223303819,-6.00187810346538
"Sytl3",69.1721992320472,-0.175852773532411,0.346970276619196,-0.50682
3740770774,0.612278496967995,0.73173191136267,-0.0549260440474816
"Ptpre",640.322609896518,-1.19592940408303,0.21782219975802,-5.4903926
4781821,4.01041186148995e-08,2.26182406888254e-06,-15.5462916960176
"Pdcd7",837.74792201984,-0.415908717148962,0.125599565626298,-3.311386
58860042,0.000928348613479434,0.0055524359710497,-2.1600296308886
"Haus1",519.305165375726,-0.023282058488021,0.218657752473375,-0.10647
7169113206,0.915203768828628,0.948174446579203,-0.00123899614438829
"Rpl37",11551.4689069566,0.217835851161078,0.177086915177256,1.2301069
8414975,0.21865704504323,0.354189172073847,0.226097085033862
"Fhdc1",209.520869008019,-0.68549367187553,0.306678812293201,-2.235216
92532238,0.0254031108522398,0.069337924679828,-1.82942033249474
"Rhod",1114.77339071503,0.300599460233104,0.210521243144456,1.42788184
101135,0.153325871156863,0.2726694487246,0.390627503978303
"Ppp4r3a",4097.01214063118,0.177893243938504,0.106128248644255,1.67621
011569508,0.0936970434772261,0.187682859432808,0.297615692147015
"Card6",413.473297257341,-0.765370388384006,0.184810405641893,-4.14138
146456463,3.45220267220326e-05,0.000434825889551803,-5.92439913390051
"Tcf20",1631.87922549408,-0.371095574891424,0.197585211370059,-1.87815
460640116,0.0603600179610176,0.134150191519882,-0.74545503721372
"Oosp1",3.38891311504266,-2.95058160162316,1.74683565348211,-1.6891008
5831001,0.0912001034077923,0.183890342941586,-4.99656109414187
"Mcm3",839.994658478753,-0.864391639442749,0.34384667262936,-2.5138868
8112883,0.011940872727735,0.0388491801177002,-2.80760308820327
"Ankrd13a",3593.22403008354,-0.14184894279533,0.0732739501694028,-1.93
587137676334,0.0528834562020076,0.121365540139683,-0.299152085988384
"Il17f",13.8154078158267,0.888393230754993,0.650668896681962,1.3653537
6945984,0.172141886735469,0.297423350249728,1.07726450638561
"Ipo9",2819.7454602357,-0.446258642453947,0.212770679776237,-2.0973690
6853548,0.0359609170256797,0.0903215200523899,-1.07297514484569
"Ndufa7",5413.53636093881,0.431585665899672,0.270793925727314,1.593778
97691204,0.110985547841335,0.213272512473231,0.666879494199822
"Macc1",291.527457912503,-0.383610927707589,0.35434145485191,-1.082602
45154751,0.278984918688987,0.422726759760481,-0.330300235770537
"Shisa4",1275.52445234269,-0.0448076942963048,0.413210329253912,-0.108
437982122105,0.913648273379911,0.946864783372037,-0.00244645441282801
"Git2",2915.90581447958,-0.391805119698453,0.174458104056941,-2.245840

75252001,0.024714209958937,0.0678032455015272,-1.05440447364825
"Lman1",4328.89789335308,0.244648758230227,0.112049127627956,2.1834061
844957,0.0290059109170055,0.076633773856338,0.628433519083075
"Wipi1",2105.38020162059,0.662055903351607,0.132458586116448,4.9982105
5593614,5.78647818067618e-07,1.77095298144432e-05,7.24382352941822
"Gpr45",9.85585868416232,-1.27956688611371,0.755289957809366,-1.694139
94305571,0.0902386937764699,0.182385617420298,-2.17735203160805
"Dlx1",8.39881359373504,0.56583070460284,0.70702832712951,0.8002942497
3689,0.423540333907189,0.566755159599367,0.321294452715006
"Tdrkh",269.893533730304,-0.688552876750842,0.266744178005083,-2.58132
298106886,0.00984224527420665,0.0333616666189413,-2.34131922117954
"Ammecr1l",1355.17514207591,0.0213456934409249,0.211502346194231,0.100
924144932758,0.91961067608022,0.950769869145348,0.00107759963689237
"Slc16a6",1337.04969849753,-0.00940818646631382,0.35964308565647,-0.02
61597868596325,0.979129890333782,0.986669833155619,-0.0001262560847758
63
"Metap1d",3117.3652677194,0.662619229041593,0.238987055633174,2.772615
55980949,0.0055607771580954,0.0214913841483734,2.5445261962 8026
"Nol4",2.76710694001056,-0.229254604671597,1.03489095303673,-0.2215253
73276174,0.824683382298225,0.887742317012687,-0.0272982081528289
"Rnpep",3835.91492154812,-0.230913649306921,0.0781360692781281,-2.9552
7598764888,0.00312389463601006,0.0139317698108244,-0.986828749814747
"Fam222a",276.615043672489,0.549805222469357,0.27610650383948,1.991279
5056396,0.0464501672956375,0.109886600573931,1.21413836338795
"AW549877",3997.09316356087,0.105567822615961,0.129228020435231,0.8169
11241543563,0.413979153058275,0.55740387586011,0.0617007196668171
"Agrn",16964.7530694717,-0.101720069228512,0.276839515569344,-0.367433
344980815,0.713295802682697,0.80986674201987,-0.0214512938514733
"Mvk",646.805949113104,-1.06583849354303,0.345466067688051,-3.08521905
110942,0.00203402288363323,0.0101197698864712,-4.89567795937619
"Mfsd9",472.636865737178,0.662903086624371,0.338779212177645,1.9567407
4085976,0.0503779573550899,0.117104126431753,1.42172279450263
"Tango6",304.949586975674,-0.079502881428569,0.139068900813135,-0.5716
79800183337,0.567538921729613,0.694623298948431,-0.0289697048539441
"Tnfrsf18",185.60357490542,-1.44763348664775,0.52451538273564,-2.75994
476863106,0.00578111343051067,0.0221671877420053,-5.51424167833327
"Pkp2",1339.6834326707,0.437068501925272,0.133632345761517,3.270678962
00277,0.00107289632817805,0.00621489382258337,2.22066054594001
"Pigs",1519.52700397632,-0.157308776455759,0.108340029244505,-1.451991
26816498,0.146504033169393,0.263250911320322,-0.209951791364494
"S100a10",6864.98833416351,-0.269911245995528,0.182256578050085,-1.480
94103863486,0.138622285952896,0.252408523048645,-0.371588534628897
"Znrf3",728.038353287457,0.0536361052053929,0.159707520795939,0.335839
570598084,0.73699184844893,0.827995797237524,0.0101236646984061
"Dcaf17",1167.18031521947,0.199521447630049,0.150019798398151,1.329967
4427006,0.183528998493281,0.311684383992858,0.232594959370941
"Spidr",434.309210811485,-0.553899682787384,0.151240143188592,-3.66238
533705094,0.000249877609326236,0.00200911902129606,-3.4397496829692
"Mettl8",687.514464870323,0.612903686534865,0.145765520433293,4.204723
34412823,2.61401700220778e-05,0.000351261912536508,4.87502269819604

"Arhgef11",1826.05419983213,-0.173502371940634,0.19452249519831,-0.891
93988470975,0.372425154982321,0.5176834442037,-0.114232458305585
"Rptn",8.815871328202,-1.92301478804123,0.9036886836433,-2.12796156779
171,0.0333402701764856,0.0852213670557311,-4.73542985680106
"Elmod1",21.8630354114784,0.601147493941621,0.521056957858371,1.153707
83342465,0.248619971278928,0.388069411754362,0.569028820006472
"Rapgef5",1236.0066394881,-0.780892856879939,0.192042986465477,-4.0662
3991457411,4.77777509094961e-05,0.000559838093289276,-5.84721867936591
"Zbed3",1533.28480854995,0.363169263330233,0.124848467948832,2.9088804
1556966,0.00362725576835759,0.015615860669339,1.51059099174468
"Tlk1",2487.76497913487,0.133610877876432,0.117759343566778,1.13460956
752586,0.256538942642575,0.397393843143316,0.12329978463132
"Acacb",6377.64665496303,-2.68931653896644,0.81026048870891,-3.3190764
9014414,0.000903156866419958,0.0054856206109417,-14.0178030670385
"Wdr41",1455.04298476361,-0.0853619981760805,0.117826354670675,-0.7244
72877181573,0.46877547273387,0.606949482192152,-0.0426220751321916
"Ncapg2",351.09770399464,-0.693679446732546,0.247764860609101,-2.79974
910496676,0.0051142339512571,0.0202194897788058,-2.70611864663244
"Mat2b",3553.26917486185,0.163955719626609,0.195383588406589,0.8391478
57625694,0.401386343184433,0.54541995396265,0.0993898298959397
"Igsf3",1059.69458265491,0.158130356373175,0.211465251529532,0.7477841
1691479,0.454590383929011,0.594792867121719,0.0821553705498956
"2010003K11Rik",300.738554076154,-1.97982545157043,0.902183881361205,-
2.19448107250962,0.0282008431934448,0.0750150123582246,-5.127880608854
65
"Csgalnact2",1194.35631550674,-0.537936180303853,0.189021024527316,-2.
84590659504184,0.0044285182713663,0.0180983544865018,-2.15816458994356
"Tbca",4619.32400350849,0.45800617793621,0.128693177553429,3.558900220
2239,0.000372410970485259,0.00273995300565272,2.7021514942352
"Sln",0.121028973229319,-0.542699536171107,4.78471990596623,-0.1134234
70304792,0.909694833164759,NA,NA
"Dstyk",2466.6453426782,0.0145783244882777,0.142306407950593,0.1024432
04759544,0.918404891600284,0.95000541340214,0.0007476872179617
"Wdr60",1170.02882650998,0.259943402190384,0.213137916001907,1.2196018
7594242,0.222615834932587,0.358516853846169,0.266644641181359
"Wdr11",2273.55518381538,0.138091874435583,0.105496296733717,1.3089736
6742778,0.190543270700063,0.320265547414343,0.157232069919152
"Zfp386",1235.35756134641,0.32817029915638,0.147863708029464,2.2194107
2308959,0.0264587948042979,0.0716385369360662,0.865096986191147
"Myo3b",372.537703869403,0.727354111979061,0.456289113403028,1.5940641
3743781,0.110921670292105,0.21322594176826,1.12405516562074
"Tmcc2",454.989574046393,-0.841768748132525,0.151565616972249,-5.55382
391434231,2.79487028923726e-08,1.75735172875028e-06,-11.1548690928097
"Abhd14b",9463.88265203925,1.45122943318401,0.331229837854806,4.381336
66514716,1.17953449791093e-05,0.000193427785031684,12.4088915618805
"Svop",9.86152120932017,1.53645467894294,0.664149272859451,2.313417693
47701,0.0206996852164451,0.058971721559066,4.34923802764893
"Hnrnpf",12421.3212135365,-0.475941055117607,0.135981663483124,-3.5000
384825905,0.000465190996412559,0.0032626253289658З,-2.7248687192199
"Arsb",4228.24644919004,0.611318036775036,0.322748040435291,1.89410301

593326,0.0582113375031216,0.130554525991537,1.24462170847489
"Lce1c",0.0924932960431516,-0.544048378937457,4.78471990596623,-0.1137
05376621746,0.90947135024012,NA,NA
"Dao",11039.4287354221,1.93071914550703,0.726591216268468,2.6572288547
9206,0.00787859392148431,0.0281659010416394,6.89197849900786
"Zfp239",260.152060588113,0.27384661401616,0.435538199044656,0.6287545
26277688,0.529509774850995,0.661855010172503,0.113018897842479
"Kank3",2759.3956214681,-0.250153356840722,0.188447410230675,-1.327443
85573946,0.184361900557302,0.312608777899494,-0.290879019034971
"Dmgdh",2567.70564942275,2.40126239697127,0.954979477075485,2.51446492
2665,0.0119213161005278,0.0388244231381479,7.80099499966091
"Uggt2",265.870284162751,-0.394723904774159,0.212883531226093,-1.85417
773982217,0.0637137356611713,0.139630259404464,-0.777115590414836
"Inpp5f",2221.27400592903,0.697566054121763,0.202032457233827,3.452742
51312214,0.000554918417900281,0.00374646790589324,3.89726092832999
"Inka1",210.32756108315,-1.2966796354839,0.349532503023576,-3.70975409
802286,0.000207460649383912,0.00174281246949053,-8.23683983909139
"Csdc2",379.298853620732,-1.33937056824183,0.319173652604036,-4.196369
46005515,2.71227671089736e-05,0.00036049060197053,-10.6185896960559
"Ccdc115",1026.91864050469,-0.230142641109997,0.180062174548797,-1.278
12874462219,0.201204035208877,0.333028546562197,-0.253048063232044
"Klhdc8a",1079.16919626108,0.990724409973396,0.345414628818041,2.86821
786721515,0.00412791173621115,0.0171986715608152,4.02523712369068
"Vwa1",2172.71689075349,0.838273029524647,0.204461753322068,4.09990140
407434,4.13326190546543e-05,0.000500561121942639,6.37069131257916
"Bhmt2",2906.32683729503,2.07742692680348,0.753385428540419,2.75745567
687474,0.00582531101158426,0.0223015735526939,7.90065827378651
"Ssh1",1903.39385448769,0.0275025363525607,0.142762809463077,0.1926449
64441343,0.847237027927554,0.902454387901199,0.00282278139273024
"Rassf4",1116.82304484689,-0.958456798628072,0.300662567628292,-3.1878
1551753728,0.00143351964833776,0.00776855795824788,-4.65586752975059
"Ppig",7038.95615649504,0.632867561394224,0.23923606143252,2.645368585
34069,0.00816019766718285,0.0289123366452055,2.24255791412887
"Msantd2",1753.49624724132,0.194378472236421,0.183069054590254,1.06177
678511248,0.288337031162462,0.432394664944291,0.162970124497512
"Cox10",1205.70401273341,0.574052081788844,0.193745788008847,2.9629138
6609464,0.00304741830758601,0.0136940682556607,2.46313837515569
"Klhl23",304.898301925634,-1.13356461339186,0.307097090114315,-3.69122
551102616,0.000223176155545328,0.0018445622063067,-7.1363711183829
"Dzip1",661.245163660288,-0.329788375743182,0.255495312773795,-1.29078
053199028,0.196779790301663,0.327764595190656,-0.367865618218037
"Gm9774",0.152342421322912,1.89757006682526,4.76651075541791,0.3981046
4387778,0.690553051392667,NA,NA
"Tent2",1521.27467789466,-0.14924511779302,0.0949153420833905,-1.57240
246431284,0.115857243134349,0.220372980140146,-0.22572336121185
"Armc5",1491.52815859639,0.291950071353985,0.149738986900688,1.9497265
0340968,0.051208725634767,0.118540630704819,0.62258338177972
"Pnliprp1",17.4411729524195,2.40057099212479,0.467636237021708,5.13341
525330372,2.84531179526072e-07,1.02169225384887e-05,27.5860778721297
"Bend6",48.401926247504,-1.6128787362208,0.299146574738358,-5.39160021

347886,6.98329961554859e-08,3.50026673274978e-06,-20.2620655437954
"1700069L16Rik",0.221516546435352,-0.548210677397382,4.78471990595398,-0.114575291380213,0.908781765330446,NA,NA
"Nfrkb",1493.95857341411,0.0195225536388149,0.142905560759877,0.13661157435027,0.891337827273157,0.932718531272271,0.00135978109973802
"Tekt3",0.421124142138885,-0.0969241418350593,3.22084221481916,-0.0300927941732474,0.975993047546796,NA,NA
"Cmklr1",445.839009944304,-1.92509880829793,0.418932079104552,-4.5952527970948,4.32224935076126e-06,8.86967069663489e-05,-17.9617259149583
"Slc35f2",549.901930316971,1.13327238161912,0.292307022362508,3.87699334918367,0.000105755247920305,0.00103462911387361,7.78978826409944
"Zfp451",1684.99069954306,0.119103084593419,0.17579659304304,0.677505078635167,0.498085548901801,0.633755679488861,0.0543219357593431
"Chchd7",1572.89263252206,0.955472442586009,0.209737719858856,4.55555845285721,5.22465837327168e-06,0.000102691924356906,8.77484591635896
"Cdrt4",1.03989668556596,0.260049382875,1.45727057577345,0.178449621640771,0.858369882192908,NA,NA
"Slc35e2",1348.9216649207,0.181417219257984,0.124318981209937,1.45928817540442,0.14448580855261,0.260536788602497,0.244008192630687
"Tbc1d22b",1856.09968639975,0.553687791012886,0.12125538478861,4.56629445346412,4.96421332680058e-06,9.88684693012846e-05,5.10595387807774
"Kdm5b",1967.89739902946,-0.628763038661606,0.245139347673179,-2.56492090979975,0.0103199325498992,0.0347167089126872,-2.11297968446947
"0610010F05Rik",703.611748950924,-0.477886691953961,0.174107490009962,-2.74477962967945,0.00605515434027311,0.0230090661177398,-1.80252502481198
"Abhd14a",1745.37818895909,1.08441713131907,0.207134064580257,5.23533940936545,1.64681956858304e-07,6.6479685193027e-06,12.9275526771541
"Fbxo38",1347.45055423456,-0.603146431178373,0.139875226180693,-4.31203185615741,1.61761120343044e-05,0.000243741756139773,-5.01781718531411
"Zfand4",70.0423886144313,-0.268054772436322,0.21241752798877,-1.2619239804471,0.206976142084975,0.340042199053997,-0.2891468107638
"Bag2",665.858204273776,-0.390868184149898,0.173609494243342,-2.2514217085503,0.0243588404641888,0.0670168860407441,-1.05644269512175
"Sgsm1",356.027540943292,0.562480407270154,0.257498971089337,2.18439865949991,0.0289329666462545,0.0765250932379314,1.44565146786951
"Ammecr1",495.516121496129,-0.45679212243735,0.23306129534902,-1.95996560369787,0.0499998107367166,0.116377737823147,-0.982520579367188
"Lyn",1806.06325359273,-0.721759050772194,0.208203961448281,-3.46659614808282,0.00052709318256021,0.00360985699808886,-4.05923578129217
"Rabif",1122.72696314026,-0.0985066986376965,0.131111130629162,-0.751322165898447,0.452458792386894,0.592918982052535,-0.0514892064090767
"Pglyrp3",0.259329838918977,-0.0656596331740386,4.78471990595642,-0.0137227746795168,0.989051153590754,NA,NA
"Tmc7",241.629730712101,-1.15343547142813,0.279227003922481,-4.13081634378151,3.61477289280857e-05,0.000451800268358856,-8.8840718436998
"Cyp2c37",1.27710894408093,0.694952572852923,1.85065111709409,0.3755178739168,0.707275355959861,NA,NA
"Grk3",2668.55868485778,-0.915049136840529,0.277061240910075,-3.30269630582331,0.000957600354847059,0.0056803443014323,-4.73148431394312

"Pglyrp4",0.464513853553168,-2.1810734410382,3.07295635923272,-0.709763884047734,0.47785056881125,NA,NA
"Pm20d1",1753.11696373674,1.51859107560798,0.479714561295649,3.16561388402814,0.00154756068551439,0.0082426806908815,7.28685246074175
"Cilp",76.9531778499691,-3.58762438298188,0.883625947594696,-4.06011660561541,4.90482146700865e-05,0.000570269582371703,-26.7974064361317
"Ptchd4",23.592559248098,0.452499982992326,0.617291407666598,0.73304111700308,0.463533351900222,0.602531553023368,0.229243389667115
"Isl1",2.02490338111192,-3.75099405135853,1.71496390610778,-2.18721457518697,0.0287268633693341,0.076133802089884,-9.65979594176632
"Ccr8",11.982230041192,-1.10102876320469,0.820088165119096,-1.34257365248625,0.179410066545514,0.306746534330449,-1.30112256676515
"Trem1",7.21394090659257,-1.39598271980531,0.866141112870084,-1.61172665638688,0.107021434389224,0.20740522092447,-2.19599362802232
"Slc26a9",4.58021114293841,-0.252164755813733,0.874226658124602,-0.288443224042926,0.773007484977833,0.853169927663183,-0.0400428906154279
"Fam92b",0.615563469784283,2.59972769455121,2.51309837140874,1.03447112302807,0.300915969819192,NA,NA
"Nxt2",1480.97707579559,0.771802217247023,0.280282277422389,2.75366043242152,0.00589328782108883,0.0225176025740917,2.9277992483622
"Sestd1",5269.563806293,0.603680130500476,0.300762861565963,2.00716314294036,0.0447322946743494,0.106755236421109,1.35056319392444
"Pelo",1382.66615099067,0.270308536882634,0.124986416022343,2.16270331996969,0.0305640055932086,0.0797773736353924,0.683479285591089
"H1f8",1.98028719270345,1.12470367597589,1.18677999030883,0.947693494295618,0.343285513847329,0.487904716686405,0.80712688546173
"Gucy2f",5.51667413689474,2.0734206353249,0.771624433220405,2.68708525295317,0.00720785533574467,0.0263625517419526,7.53856507762649
"Itga1",7257.28856935715,-0.135989697142317,0.132596164680755,-1.02559299109241,0.305083472569382,0.449767416407745,-0.108659124549673
"Stab1",4384.37997096666,-0.441691211276747,0.328845733485307,-1.34315627755128,0.179221375807528,0.306549859832652,-0.522244584986553
"Hsd3b7",2453.69150748945,-0.991879947911117,0.262549533817059,-3.77787739132779,0.000158170710935491,0.00141728516627766,-6.50575257219111
"Mrtfa",1367.51123467849,-0.568161601979225,0.182944055135427,-3.10565763702262,0.00189856393950589,0.00959020717421911,-2.6402542295759
"Gm5617",1034.39550812571,0.489365275199885,0.349647084262744,1.3995977579271,0.161633805758944,0.28353899637318,0.616798739145298
"Ttc19",1005.312386891,0.319646604313895,0.217689046555332,1.46836328869972,0.142005568015178,0.257015182879415,0.434278307424708
"Ehbp1",725.347967429578,-0.196873458607923,0.192491452513978,-1.02276467882971,0.306419122056599,0.450842140194655,-0.156836882746716
"Sgsm3",1021.7524800837,-0.445741590212562,0.201956265763288,-2.20711939056654,0.0273057194308665,0.0732838152048225,-1.16490797933481
"Tmem183a",3738.67513735474,0.196128845060547,0.11650862884603,1.68338471581995,0.0923006401151597,0.185628401040984,0.330282631877773
"S100a14",515.483165093968,-1.75541438861296,0.442676904623883,-3.96545283993171,7.32567358530664e-05,0.000776698269304374,-12.5695720744817
"Setd1a",2789.66886915593,-0.0322364830758934,0.148817655959531,-0.216617328555825,0.828506580133056,0.89072680301248,-0.00373032577579652

"S100a13",1344.04830707817,-0.468068033253696,0.233497036963929,-2.004
5994559066,0.0450058848645443,0.107172049717744,-1.04534560387861
"Prox2",107.619715504379,-0.290727559183835,0.332489707794649,-0.87439
5665093466,0.381902817304456,0.526743278741858,-0.186368571994735
"Pbrm1",5591.91066102428,0.295961782736664,0.204035355984224,1.4505416
5396485,0.146907532288605,0.263705777315835,0.394493757446136
"Hps4",1120.62408602985,0.0155718236920568,0.188998230974598,0.0823913
727221589,0.93433549629626,0.959022817068478,0.000651531516035324
"Specc1",1256.70900421468,-0.0197750930533272,0.270849825204366,-0.073
0112823163394,0.941797139483726,0.963817152236969,-0.00072878492100490
7
"Tnfrsf14",166.839628691149,-0.964924653158321,0.392385061195616,-2.45
912688474467,0.0139275384194048,0.0437716221956085,-3.01902688891388
"Ctf1",278.148341632158,-0.10081037635903,0.260601609051471,-0.3868371
21712165,0.698876793432979,0.799313685966461,-0.0225817069785244
"Ubash3a",168.537111362605,-1.27999751519985,0.566162687351989,-2.2608
2987062703,0.0237697941337155,0.065739495889246,-3.48422412138896
"Arl15",1149.52842749139,-0.0211994799342428,0.15559737730845,-0.13624
5740776323,0.891627016363665,0.932918892678226,-0.00147202857783255
"Ikbke",1197.27398822868,-0.885274689819264,0.217990911789791,-4.06106
237434769,4.88499170427111e-05,0.000569491712762559,-6.61368034281232
"Arel1",1724.63146457213,-0.297534765717645,0.155012710196916,-1.91942
173864117,0.0549309819645491,0.124801245204472,-0.619179620135569
"Grap2",436.057062757418,-1.37318715375515,0.452700750908989,-3.033322
01459329,0.00241877346307802,0.0115449804120184,-6.12648071091728
"Frem3",9.46565241255908,-1.69417896894043,0.974161643582803,-1.739114
83797445,0.082014565187472,0.169452155560594,-3.00748051710312
"Gnl3",1685.0910232922,0.197514657785006,0.24296745567001,0.8129263947
73064,0.416260260271554,0.559586497157665,0.114668549954425
"Gjb5",177.81245442991,-1.56651570489926,0.67234785515745,-2.329918498
11496,0.0198104591441223,0.0570192008801503,-4.48707621957238
"Osbpl6",3098.77882432195,1.74239327797234,0.404113279624422,4.3116456
8408072,1.62043952999713e-05,0.000243979381898719,14.4939710034951
"Lgalsl",1262.25448152855,-0.733454724273721,0.141458985941091,-5.1849
2847516355,2.16097782046207e-07,8.21340069071529e-06,-8.5885666952579
"Snx18",2897.3469609997,-0.242368871216465,0.186668947789694,-1.298388
80052789,0.194153765632645,0.324582879388264,-0.272716936494309
"Gjb3",167.977334914642,-1.11717923224977,0.325129969229287,-3.4361004
4591709,0.000590152127595319,0.00391121216310453,-6.1935388125989
"Rbm45",913.033788530593,-0.452793663054725,0.31933300923656,-1.417935
66577171,0.156209548370314,0.276611913007391,-0.581903153611994
"Slc5a10",4079.36427923677,2.5325653143657,0.945989886852295,2.6771589
7343534,0.00742493959643315,0.0269542863994142,9.151710802032
"Dmrt3",0.183426502768653,-0.55695670027347,4.78471990596623,-0.116403
198352109,0.90733300070876,NA,NA
"Fam83g",465.185096701514,0.124983497154339,0.183677117819944,0.680452
190440288,0.49621818436001,0.632001674114596,0.0573503319412493
"Esm1",1059.76947528847,0.196487080167935,0.471388480796334,0.41682622
3322221,0.67680552007081,0.78233068712003,0.0482332071822274
"Smim12",1498.54932508308,0.466823075353463,0.18757101611101 4,2.488780

43651036,0.0128182100171045,0.0409183789969718,1.49204868758131
"Gzmk",59.4200893390108,-1.49287794630167,0.62026731952619,-2.40682992
526842,0.016091661796278,0.0487348899819554,-4.51052182928349
"Tex13b",10.3644287060145,0.352449680969118,0.544866837548174,0.646854
711428381,0.517725979040466,0.651097854704012,0.151234513441175
"Dlgap3",17.4244600098374,-0.563037896697003,0.623824545533412,-0.9025
58100235648,0.366760472599001,0.511633089699887,-0.377318457185955
"Tsen2",373.97288853452,-0.0959402301505786,0.131157117055869,-0.73149
0843228207,0.464479395532933,0.603399244060305,-0.0484667213798845
"Gatad2b",3272.54575199928,-0.120629443198299,0.146923506393899,-0.821
035695097651,0.41162593640151,0.555232273120488,-0.0709745938223151
"Rbm7",2497.03957145186,-0.306258240771183,0.146300976724167,-2.093343
78777658,0.0363184764595964,0.0910085662523084,-0.734040254434474
"Crtac1",12.6011752623894,-1.53405835169009,0.814725238418651,-1.88291
497470685,0.0597118915134844,0.133088987280512,-3.09379269392799
"Dennd4b",1212.69224235663,-0.776753146631133,0.191427529611485,-4.057
68777462471,4.9560965277893e-05,0.0005752395827274,-5.79514079524798
"Atf4",6056.33709005992,-0.138218998926138,0.088416326957293,-1.563274
60869191,0.11798801792761,0.223561446256216,-0.207061594101157
"Zmym6",2148.88742315023,0.152675548709659,0.180308637059064,0.8467456
20175956,0.397136924678121,0.54164631766746,0.0936117975428534
"Agps",7312.81094368438,1.4287842546468,0.283739707838612,5.0355456609
5301,4.76489525238475e-07,1.51440601952762e-05,15.8565078789712
"Prdm14",1.55431073511441,-0.408882790356139,1.61319650087908,-0.25346
1243024843,0.799911807087523,0.872172817034161,-0.0559219547328683
"Ccno",27.3259710566612,-0.530008704051948,0.536288468428419,-0.988290
323685546,0.323010483009862,0.468073440522315,-0.402345545234471
"Nfkbil1",1059.57074906375,-0.0215764318251025,0.154180282693766,-0.13
9942873680922,0.888705126272252,0.930910742025163,-0.00154469730788279
"Fbrs",4177.57611710512,-0.0588033630884042,0.136501608718184,-0.43078
8791726312,0.666621949990885,0.774277403998468,-0.0150433742727888
"Frmpd3",46.4925426154823,1.73881503351708,0.486080717723279,3.5772145
8621399,0.000347275077365264,0.002591339249318,10.3556528236652
"Dhx29",955.384086873425,0.252831102462843,0.192881673888846,1.3108093
5459189,0.189922181302316,0.319407694646124,0.288552838440119
"Mgat3",3400.5649566013,0.573033704653372,0.353777678442452,1.61975652
951373,0.105284587642238,0.204981322839246,0.908164680492679
"Adora1",1322.36367224114,-1.64658464392547,0.544609747188903,-3.02342
117897191,0.00249934127353148,0.0118285247920627,-7.30629340137406
"Mfap4",1251.32985768364,-2.40478736502612,0.574058966729247,-4.189094
6826031,2.80069534859787e-05,0.000369722125301025,-19.0044546245402
"Zfp532",920.89324433862,-0.452222276929553,0.321904252296448,-1.40483
474108659,0.160070425756097,0.281454879735989,-0.573319773420704
"Mindy2",3838.5698161139,0.249261079545604,0.200649257522065,1.2422726
235017,0.214135992544793,0.348807316845312,0.262530645157793
"Zmym4",1507.06173958935,-0.114360762298559,0.114335894069178,-1.00021
750150802,0.31720526131484,0.462379529773923,-0.0882143733993496
"Mios",793.205091586937,-0.0988253291398682,0.123603844212645,-0.79953
2812020406,0.423981528569257,0.567112015095244,-0.0560535722517252
"Hoxd1",2.82630893255667,-0.280135448024809,1.07932628353711,-0.259546

582250147,0.795213545571986,0.868777593520624,-0.0394061268979073
"Mybph",3.71757483195118,-1.32475232552159,1.12428443135764,-1.1783070
9789503,0.238674199135022,0.37675433409445,-1.29317279697606
"Reln",178.324960781142,-0.895597717906012,0.424759948601041,-2.108479
67388566,0.0349895191310605,0.0883826459408494,-2.17279139038273
"C1galt1",3127.27278744915,0.396735863029871,0.130717656656735,3.03505
947992702,0.00240488254071174,0.0114899288847825,1.77193518662954
"Dctpp1",895.888949023559,0.589373058173504,0.203489407147303,2.896332
86781786,0.0037755169152068,0.0160766605836869,2.43433864785683
"Zfp410",1646.23095428195,0.353458417506923,0.138214711633182,2.557314
00319375,0.0105483951715605,0.0353749819381806,1.18116981996345
"Tbc1d8b",872.913669436113,0.426421464512794,0.14405185828663,2.960194
12442645,0.0030744524929248,0.0137742818720238,1.82719553194142
"Fcmr",33.2787362344689,-0.67313256506439,0.779180265248991,-0.8638983
75107443,0.387643719531434,0.531935902837913,-0.42490300398075
"Abcb4",162.461530680079,0.550629845603688,0.436410200349471,1.2617254
2521406,0.207047604751835,0.340130945947534,0.59381277120115
"Tfap2e",3.5433371745812,-0.0689391906002163,0.964299181849459,-0.0714
914954796454,0.943006592864396,0.964676943739244,-0.00247919165628083
"Mustn1",636.790408924168,-0.788054052038261,0.210835371749629,-3.7377
6964225002,0.000185659931113812,0.00159682282172788,-5.07486269699012
"Leo1",1085.51351105213,-0.444768237423716,0.11919438300891,-3.7314529
9464713,0.000190378536212799,0.00163164865893502,-2.8545956357191
"Clspn",108.932180854053,0.0173987167180955,0.53339573436811,0.0326187
773861876,0.973978595608856,0.984025832261292,0.000280173796734201
"Tbc1d10b",2533.60290209539,-0.125817508629237,0.120766342285959,-1.04
18259446106,0.297492383170481,0.441802074842429,-0.102779478773663
"Prdm10",643.373907044927,-0.168721961561162,0.132338624606636,-1.2749
2606230927,0.202335413359557,0.334390226282814,-0.184825903494226
"Radx",87.0787413153064,-0.301821986491354,0.29359085863982,-1.0280360
495203,0.303932870504846,0.448673766588696,-0.241898017819707
"Hoxd11",421.759483196087,-1.01095078631606,0.392204371196213,-2.57761
22362754,0.00994855674693555,0.0336240179323538,-3.4296653509433
"Ago4",922.122068448588,-0.225913940233256,0.311871079923066,-0.724382
460499335,0.468830964780798,0.606949482192152,-0.112801025512186
"Cpa6",435.543279006094,0.731650797247348,0.313972281717799,2.33030378
746924,0.0197901007293769,0.0569714135569748,2.09633000065603
"Cd2bp2",3195.97487357949,-0.0108815797398211,0.0690036505622985,-0.15
7695711040635,0.874696580300132,0.921844759913505,-0.00088552601440527
2
"Sdhaf3",369.853569516615,0.155140638392422,0.267359287340311,0.580270
242099164,0.561732391649192,0.689757426309966,0.0576216066380293
"Usp22",2485.72906405135,-0.451099646733566,0.145465815885563,-3.10106
978734057,0.00192822840157784,0.00971442275120668,-2.09046054818899
"Elmsan1",1320.45779155446,-0.902245549231418,0.199775223257485,-4.516
30354615366,6.29284423951218e-06,0.000118548673412569,-8.1564684268872
6
"Dmtf1",2077.22174115286,-0.0882965140628362,0.165599885495454,-0.5331
91878718178,0.593900762741011,0.716620896832987,-0.0294211325345853
"AA986860",977.588513757602,-0.112130935212857,0.232471252088856,-0.48

2343232573108,0.629562140426946,0.745550640027836,-0.0329252553790972
"Klhl14",328.620655352658,0.111168769970218,0.293966327846569,0.378168379979358,0.705305513413573,0.804213269037301,0.0242226505542653
"Pwwp3b",112.219154670715,0.385389168077548,0.20968042221316,1.83798355616512,0.0660648284211763,0.143540452925796,0.748093706003373
"Ubap2l",4590.61922195219,-0.0568187595889726,0.177714684517285,-0.319718990826817,0.74918136223816,0.83662876293927,-0.010135037139913
"Dnal1",776.18424924242,0.054611972361488,0.22564349068218,0.242027687997476,0.808758708989228,0.878121647011892,0.00709792595655166
"Sun2",5373.86745480626,-0.983152848931536,0.264354204775177,-3.71907399682811,0.000199954473349263,0.00169500673184033,-6.27258258760223
"Kcnj12",57.8374844697271,-0.0235125970394798,0.381625087364297,-0.0616117698180435,0.950872003791887,0.969670623950534,-0.000724160434094343
"Golga7b",70.0719377410802,-3.67512023454546,1.11862487383093,-3.28539112666014,0.00101840892075038,0.00595767690872815,-18.8279153251187
"Gtpbp1",1704.17105501397,-0.115723483263207,0.131604473413997,-0.879327884996494,0.379223520702481,0.52410690435618,-0.074764267252389
"Acot5",6.95304371170335,-1.46400811003933,1.02788204608993,-1.42429582811416,0.154360854663447,0.274035140146398,-1.895156935043
"Sem1",5362.57479601819,0.441421575247415,0.160035169240312,2.75827855428808,0.00581066598846784,0.0222586179652546,1.6796205800823
"Asxl1",2290.7338594722,-0.182683934181908,0.118818876928344,-1.5374992501577,0.124171098532807,0.232483971499201,-0.266523802122855
"Sin3a",1797.97193456426,-0.257894437934012,0.109478559752812,-2.35566158813473,0.0184897534196185,0.0540986212748681,-0.752264298270322
"Adprhl2",2087.40374931987,0.17358163446711,0.12651443932993,1.37203022347857,0.170054029299612,0.294468335993235,0.212218094193821
"Fam227a",338.915944256149,0.197761875432865,0.204968485420724,0.964840399864076,0.334624757245641,0.479355926586989,0.145416659528983
"Nek10",2.89722190280321,-0.690439014351484,1.41660464882572,-0.487390052633115,0.625981941522055,0.742736925442526,-0.205345792449097
"Dhrs7b",1924.36098612789,-0.570444781335667,0.448662442572974,-1.2714342169233,0.203574216233728,0.335868172967103,-0.622376099556928
"Mier2",1288.04986396134,-0.151747294261766,0.133629432375928,-1.13558286945999,0.256131181612283,0.396982971309721,-0.14019354247987
"Ube2q1",3965.54773027655,0.0178121254273873,0.0628068345891287,0.283601705832035,0.77671562970066,0.855598568289921,0.00277787176566094
"Thsd7b",29.9554293095346,1.13639287811267,0.353449330803431,3.21515074177541,0.00130376038769161,0.00720011905131115,5.60657349399958
"Cux2",313.923281123194,0.18444714924346,0.266373743975132,0.692437424540909,0.488662670222476,0.625312858650671,0.086598523231621
"Ipo11",1140.33867832279,0.0821595870480986,0.113914697494022,0.721237810883976,0.47076321198163,0.608723463573037,0.0407832957764236
"Sh2b3",2105.84229308968,-0.475263717052645,0.146563549081291,-3.24271430401185,0.00118396869243869,0.00668937165029544,-2.3797572765074
"Fam199x",1248.25777906009,0.467287095509345,0.169078721280667,2.76372503866799,0.00571456717345776,0.0219595251769963,1.78436114076626
"Tfap2d",0.149762844135821,1.36369449992258,4.78471990596446,0.285010309218446,0.775636243738567,NA,NA

"Kdm7a",2569.27915165679,0.0704468268411943,0.168370405894339,0.418403
854685741,0.675651868735642,0.781275064441201,0.0173882490910532
"Kcna4",2.35907139270284,-0.532288767212847,1.05869679257794,-0.502777
349420997,0.615120820330151,0.734363565686829,-0.164344716791203
"Atxn2",2530.78796058791,0.654588226089079,0.272473990771973,2.4023879
2786974,0.0162884247951156,0.0491704144070543,1.97192292231255
"Hirip3",877.916521143855,-0.0453259783473207,0.259792820826259,-0.174
469710914888,0.861496332861331,0.91235975765196,-0.00415733932712277
"Asb4",61.9681174671916,-0.607949384101924,0.292079028174748,-2.081455
10446643,0.0373922693736333,0.093065415911042,-1.44354701775917
"Stk40",3035.85282807827,0.00573086615336843,0.139915535262698,0.04095
94698873749,0.967328207072501,0.980044966562724,0.000115516061122988
"Pbxip1",10850.2275918269,0.172340763824984,0.167930505284206,1.026262
402613,0.304767914150499,0.449498093048898,0.137807753622994
"Oscp1",385.350213018593,0.0461582438857512,0.217618538681331,0.212106
211931433,0.832024173543422,0.892673419561928,0.00524055205901521
"Maff",556.469098343323,-1.61519249792138,0.293143051181512,-5.5099122
8143172,3.59012563402498e-08,2.08334135750652e-06,-21.1292012818139
"Safb2",8769.72041580486,0.139496914232405,0.237545333675266,0.5872433
36141907,0.55704029113895,0.686350198683975,0.0525020752668755
"Shc1",3580.0894714209,-0.623138851825124,0.141529686328051,-4.4028844
2652769,1.06821077955727e-05,0.00018045887560481,-5.37146166484338
"Zfyve1",2091.5403570856,0.0229255857819866,0.132020597798333,0.173651
582891682,0.86213929053636,0.912858294041004,0.00209023156252493
"Xkr7",0.184412451228424,0.909179615714358,4.78471990596623,0.19001731
210654,0.849295564579289,NA,NA
"Pla2g6",1170.92380196827,0.0515898159033288,0.297656346410088,0.17332
0060282714,0.862399856484,0.913068657782637,0.00469179458781451
"Gucy2c",6.42442997153691,-1.34729445361553,0.933458794664205,-1.44333
575441881,0.14892591242801,0.2665709575435,-1.78127795542982
"Rgsl1",11.3513298995262,-1.57094212511008,0.643527503872008,-2.441142
16666415,0.0146408898271477,0.045434994405495,-4.85652464430659
"Flad1",1941.06171190981,-0.0374952718738954,0.2577357849629,-0.145479
495132167,0.884332304026781,0.927951660474155,-0.00280373285981733
"Itpr3",1966.85965259834,-0.857688490872867,0.149636569262906,-5.73181
07137697,9.9364124473122e-09,7.94017089744645e-07,-12.0472305039865
"Acad12",1471.99367296727,1.36521222135421,0.314639586556396,4.3389715
715556,1.4315103696275e-05,0.000224189471381501,11.4719042477145
"Alkbh5",4825.50458676738,0.233978453442414,0.188873244103528,1.238812
06442434,0.21541510497277,0.350364007070326,0.245392541214463
"Shisal2b",0.222154623861919,-1.33062269906676,4.76837581088075,-0.279
05155798133,0.780205258059674,NA,NA
"Arrdc4",2309.24988394921,-0.588091118679196,0.118507621051803,-4.9624
7510041673,6.96004914246564e-07,2.04686332096222e-05,-6.34939452886401
"Wdr55",1608.23292263923,0.984168564286752,0.172693908841516,5.6989188
0315212,1.20569629551224e-08,9.25538046058966e-07,13.6729461927646
"Dusp15",606.215576315186,1.35950348822152,0.463330059362663,2.9342009
23833,0.0033440765612582,0.0146787043654688,5.73895027098747
"Immp1l",1299.38300180867,0.0185590658407775,0.341961411578798,0.05427
2398031966,0.956718140311973,0.973595527129585,0.000496628194709172

"Rgs8",3.77470909455589,-0.0875236388476242,1.26457628620467,-0.069211
8299247138,0.944821006771626,0.966007976066987,-0.0030268464572001З
"Dcst1",233.426192702745,1.02612715426773,0.335439344660536,3.05905425
407438,0.00222036935235443,0.0108397626295872,4.64274738338265
"Ypel3",5252.42463211268,0.174315628289979,0.141471334967749,1.2321621
7850576,0.21788850838794,0.353188694939162,0.1814194814162
"Zc3h12a",597.595844732586,-0.889240986787206,0.182758899823905,-4.865
65079809533,1.14080788817008e-06,3.05542200598079e-05,-9.2445561864538
6
"Myo15",39.096189716882,0.151411194551445,0.47233813005017,0.320556789
551084,0.748546287701524,0.836443709975114,0.0270414417478795
"Garem1",278.093628288484,0.382271517179051,0.29622249671255,1.2904877
9691436,0.196881346101424,0.32790573898399,0.426243863097568
"Selenok",6147.36211288689,0.0352843104046669,0.128003487012211,0.2756
51165669423,0.782816006984378,0.859567860797511,0.00533941603652372
"Npl",900.1278666827,0.530877297008282,0.430200888461842,1.23402185175
954,0.217194761732031,0.352550081506463,0.553472810604307
"Jph1",63.839095450612,-0.962347432838758,0.320542462677671,-3.0022463
3204516,0.00267995221267827,0.012435437468539,-4.22201549018613
"Mapk6",1623.56527650594,-0.713186884255682,0.224289799965906,-3.17975
620988602,0.00147399001800457,0.00793480324987662,-3.44932602683319
"Stn1",511.984473976842,-0.412959791838568,0.217128657646827,-1.901912
88572452,0.0571825443058836,0.128925213792336,-0.845957541126421
"Dhx9",4487.82418574679,-0.188974776995113,0.180117980072287,-1.049172
19768549,0.294098872097158,0.438271156232834,-0.155888597200012
"Sipa1l1",3095.12899085483,0.291882624464464,0.180395366276267,1.61801
619680995,0.105659107155516,0.20548047744658,0.461876305400777
"Commd10",1222.55331168962,-0.291682935473101,0.225378432179008,-1.294
19187387651,0.195599152964383,0.326271903883953,-0.326691941865032
"Dnali1",23.1928559997315,0.0646191593881606,0.50869176352027,0.127030
087809915,0.89891658481888,0.938079403275451,0.00413050073054405
"Shcbp1l",15.8049439701003,-0.879883320569313,0.585278894129908,-1.503
357338517,0.132746921498322,0.243936827497514,-1.24137985529291
"Atpaf2",1458.65970705105,-0.683546518767453,0.357458167568861,-1.9122
4199300405,0.0558451622024061,0.126519944596196,-1.41313353098207
"Tceal9",4920.07175296287,0.0109725300199622,0.179184668220911,0.06123
58754178371,0.95117135887925,0.969888762835502,0.000335472942857773
"Ppp1r3a",5.47473099666842,-2.31887865677112,0.88207689048092,-2.62888
494392687,0.00856653391340147,0.0299820998462577,-8.13266628925437
"Naa25",1255.86996469401,-0.126999285997352,0.2034692413608,-0.6241694
57496286,0.532516302802645,0.664724895777591,-0.0518642242531002
"Map3k9",193.842681513715,0.304056728865259,0.187360786804171,1.622840
79850208,0.104623441726546,0.204015188197238,0.483316668114957
"Trafd1",3752.44707387538,-0.530492897417746,0.153908680814401,-3.4468
0296530817,0.000567262132306958,0.00380608295103959,-2.95545800700808
"Wdr74",1106.07393818923,0.314717799086832,0.160513657927416,1.9606917
1402939,0.0499149962417429,0.116221948497911,0.677352505644239
"Ttc9",166.12480241314,-2.05077322598255,0.420813207455553,-4.87335756
019291,1.09717382660114e-06,2.9878066021046e-05,-21.3657469122441
"Dpm3",2050.10448220635,0.427852279956256,0.206305662460177,2.07387560

212432,0.0380908654729301,0.094442484103661,1.00963052034008
"Bmt2",1279.64531373268,0.0289249732173048,0.128571511094153,0.2249718
69515659,0.822001158205117,0.885882079520417,0.00350488037690545
"Sgtb",95.2734927649116,-0.303892904759377,0.281130950453957,-1.080965
66482156,0.279712387100498,0.423467048970068,-0.261128965853642
"Hectd4",2283.67304481398,-0.180494455029159,0.177819424832495,-1.0150
4352069063,0.310085082395401,0.45527401630614,-0.142023110190341
"Id1",2290.72914176176,-0.72893937323394,0.341319015928701,-2.13565415
114818,0.0327076140974348,0.0840338726672531,-1.80524410058622
"Krtcap2",1938.14067313002,0.357727374997221,0.124742339327689,2.86773
021032976,0.00413427908531853,0.0171986715608152,1.45341882717707
"Bex2",54.4724300702369,0.254170465653677,0.525372181669161,0.48379125
2224569,0.628534023949667,0.744722678595686,0.074915060464933
"Nmnat2",28.9974429527081,-0.692495712862551,0.604305142129979,-1.1459
3715092632,0.251821157944689,0.391891526666577,-0.648709344094289
"Tmem108",205.223356376,0.5105436356904,0.432849583993882,1.1794943429
9934,0.238201387970339,0.376324337991094,0.498956289536943
"Apobr",474.759822522677,-1.8469612161888,0.273900459583285,-6.7431840
7131839,1.54952694368128e-11,5.49321388143594e-09,-35.1287449250467
"Mrap2",5.45307162376104,1.8640047453409,0.876706370956156,2.126144861
1443,0.0334911995571837,0.0855286717702643,4.58340801025955
"Maneal",16.0138257901804,-1.31111594107722,0.438700676830308,-2.98863
441595365,0.00280227208900676,0.0128531407178202,-5.70881787350944
"Trim46",437.952975543959,-0.432222929580682,0.440992584738254,-0.9801
13826261325,0.327029934986059,0.4718038973258,-0.324682342730851
"Hebp1",3419.86276217236,0.595005950809724,0.345147469018618,1.7239180
47267,0.0847226388236152,0.173849927571338,1.04100029941633
"Smg7",3916.4709949289,0.284382497838665,0.11906284016341,2.3885075935
3934,0.01691695872803,0.0505221716225503,0.848979298397962
"Muc1",2451.43835776851,0.157757175881922,0.556335196215418,0.28356497
4776173,0.77674378178787,0.855598568289921,0.0246028586818164
"Exog",338.648296047877,-0.345929342427187,0.180375346191254,-1.917830
51138482,0.0551325056022609,0.125215328390551,-0.718744450535019
"Fam166b",22.9356333344263,1.49126846488676,0.504772782478807,2.954336
12241043,0.00313342522025931,0.0139646914159122,6.36954043004352
"Rnf214",1505.04776219674,1.08276755360034,0.32008062497098,3.38279629
920903,0.000717518308401454,0.00455784247435214,5.83709800279066
"Lgr6",56.3691358977694,-2.20625400493635,0.794213718620751,-2.7779097
1524362,0.0054709817478245,0.0212623484391234,-8.49588141994938
"Aqp11",1397.24036384443,2.0961389157882,0.465288218410836,4.505033295
16367,6.63625366260265e-06,0.00012299778889109,18.8722601063583
"Olfr769",154.404518403559,0.294566098510629,0.255607403591906,1.15241
614433408,0.249150109999156,0.388696499020926,0.278352134166588
"Gpr153",952.059978596641,-1.72954657884944,0.359865017112408,-4.80609
811069576,1.53904394889466e-06,3.86704625200363e-05,-17.5729447147827
"Hecw2",346.851706628118,-0.278921682388206,0.306841726705605,-0.90900
8319640351,0.363345734253724,0.508324253891018,-0.188728378955374
"Gpx2",37.0953489467039,-3.87245825328112,0.949399918407885,-4.0788483
1059931,4.5259354563241e-05,0.000537107057379229,-29.1569507383208
"Krba1",1683.2276171428,0.229120509545769,0.200912535198649,1.14039927

533257,0.254119994657772,0.394712949560842,0.212989621056419
"Foxf1",77.5720793079991,-2.00316585177651,0.521643290377729,-3.840106
61830998,0.000122980888802511,0.00116798897704401,-13.5263209891113
"Mcts2",376.251904639719,0.326398457709703,0.245724366917671,1.3283113
1810001,0.184075282261481,0.312231392216994,0.379931305179467
"Flt3",327.298830023471,-1.3930117798433,0.364340569415848,-3.82337817
080522,0.000131635647247995,0.00123052468075979,-9.33361720940176
"Snai1",191.344360103236,-1.4937969168246,0.280868112986498,-5.3184994
9408962,1.04626511074709e-07,4.72224007623768e-06,-18.3187710989828
"Fgf11",457.16224708772,-0.532622047775193,0.213433349532613,-2.495495
89575179,0.0125781210343563,0.0403248291246752,-1.71013642223981
"Trim72",46.399676330684,-0.533709351346523,0.346246394143483,-1.54141
490098914,0.123215839718184,0.231064009836614,-0.781916493400263
"Alkbh6",945.897063496813,-0.10930708912701,0.141003713404539,-0.77520
7166448224,0.438217254193441,0.580362048667101,-0.0594743313306805
"Nrep",2293.37627254317,-0.848178146817786,0.223135286157929,-3.801183
40501944,0.000144006656423949,0.00132133731150784,-5.62266703252854
"Serpinb6b",1439.3160295336,-0.987921534623658,0.352247281663326,-2.80
462500649729,0.00503751493690134,0.0199962268403303,-3.86495816787337
"Wfdc12",0.358936840130043,-0.551039971452688,2.72043557928645,-0.2025
55787627665,0.839482249913832,NA,NA
"Lrrtm3",2.46819570816268,-2.47880253117666,2.02529948318871,-1.223919
00642464,0.220982782888893,0.356770911713805,-2.55480610461995
"Vmn1r226",1.46402840273698,3.88983453744972,1.45160096101555,2.679685
83785477,0.00736912857026O2,0.0267815971785524,14.0813577850243
"Zc3h6",616.051981307377,0.653397699852092,0.316675017124145,2.0633067
4830577,0.0390834933218627,0.0962913763949558,1.52919663075139
"Trp53rkb",262.829659629759,0.126632650566439,0.208665182559872,0.6068
70053800685,0.543937153001957,0.674233096581829,0.0499159806039767
"Tom1",2044.37565313692,0.439154661977295,0.147400672825738,2.97932603
398953,0.00288883217003119,0.0131388965829877,1.90249627208337
"Lhfpl4",40.0089508497178,0.350289447029798,0.422846641083006,0.828407
779550117,0.407439602250572,0.551225184893259,0.208636552341827
"Olfr1260",197.441605010534,1.26288103716329,0.351910627583919,3.58864
137134403,0.000332405727359502,0.00250725217487643,7.56284881107453
"Abra",12.166085171979,-0.476488883552338,0.530218913405284,-0.8986644
41243958,0.368831422782696,0.513786888720227,-0.317316205729181
"Aida",2129.20106493178,-0.606710667306348,0.112860606519232,-5.375752
31976943,7.62635686055708e-08,3.7081971587816e-06,-7.58689550234004
"Foxo4",2146.70912401207,-0.00412920521114625,0.136211359107114,-0.030
3146906264925,0.97581608053193,0.984806518540972,-6.32184826895954e-05
"Olfr648",161.673412356044,0.0639646007550846,0.165686615062956,0.3860
57743594918,0.699453904150465,0.79973917921338,0.014294145692973
"Mamstr",117.460922969622,-0.725728272378493,0.28055718719905,-2.58673
919432904,0.00968888966843292,0.0329923106453205,-2.47580803487962
"Gm14117",2.01690277510483,1.10671290619878,2.4963992342608,0.44332368
437314,0.657531626993692,0.766962889634064,0.293629596042946
"Greb1l",540.101723846717,1.42258585490909,0.576639922150313,2.4670263
0231396,0.0136240349691913,0.0430258234255412,4.4753907969042
"Egflam",923.070516777189,-1.26411873397348,0.252145965606392,-5.01344

025447076,5.34653738146112e-07,1.65998066661989e-05,-13.9130418600371
"Gm5436",0.119103556816106,-0.0636404980581492,4.78471990596623,-0.013
3007781665116,0.989387827356347,NA,NA
"Sbk1",2500.1774201852,-0.0531627018496393,0.334924382877734,-0.158730
461463735,0.873881237651179,0.921235285353051,-0.00436145785707127
"Upk3b",890.524659117967,-2.68914775939516,0.674246261052238,-3.988376
22799486,6.65270976221614e-05,0.000722571430079824,-19.4497836112729
"Notum",791.689623610679,1.23927055443735,0.34186015937077,3.625080374
14586,0.000288871596668462,0.00224488576797197,7.55843583411335
"Borcs5",596.939369308991,0.356683458042737,0.186099745145204,1.916625
1827182,0.0552855663767934,0.125460657962607,0.740390343438253
"Nhlrc3",1000.59492620804,-0.213067526233892,0.141841058271578,-1.5021
5691302824,0.133056589443954,0.244356916358502,-0.300238852592302
"Rasef",172.249088853547,-1.56571950826115,0.444381091822293,-3.523371
12688692,0.000426094257265654,0.00304544935050063,-9.07194607907632
"Gng2",1066.59793708902,-1.79750615687491,0.42727441630426,-4.20691267
317751,2.58883121058313e-05,0.000349563926204639,-14.3060353309777
"Klhl6",636.794245960155,-0.741370262675688,0.251186406360761,-2.95147
445841839,0.00316260701375373,0.0140561913731867,-3.16171606042129
"Onecut1",0.816294748029367,0.603144692937581,1.60685284096353,0.37535
7766163522,0.707394410167364,NA,NA
"Nemp2",489.856643435224,-0.518697460016043,0.246495201895858,-2.10429
029054768,0.0353531325041901,0.0890948952698751,-1.25423807288547
"Ptgir",383.893637441357,-1.44445969437512,0.372041755876359,-3.882520
36649123,0.000103379354653675,0.00101546128818002,-9.95581176236588
"Edem3",2525.25458691308,0.124423717574883,0.194925752265507,0.6383133
89220147,0.523269697917676,0.656416556121904,0.0523773705088158
"Wdr63",5.47145192919969,-2.67305926214221,1.37046334284092,-1.9504784
8313919,0.0511191155188555,0.118375358003164,-5.70402700073546
"Gm16433",0.674553218644088,-1.03279874525012,2.41887872682169,-0.4269
74173528401,0.669398138841378,NA,NA
"Trpv3",46.0551091378608,-0.757326806049556,0.488976625491741,-1.54879
960834109,0.121429899742971,0.228691218505598,-1.11734677408174
"Ccdc63",41.9776813066773,0.642565122590341,0.599340685882291,1.072119
97737884,0.283666146956111,0.42765967164233,0.545812523851416
"Tnp2",0.834034823200716,-1.95355908918984,3.10374401353817,-0.6294201
71466669,0.529074015576025,NA,NA
"Disc1",51.9218852077219,-0.163239758229084,0.374882654790221,-0.43544
2280786319,0.663241426237289,0.771545929764016,-0.042337712841216
"Zfp513",1528.92040730968,-0.0205049293445084,0.175889231520404,-0.116
578651047945,0.907193956345062,0.943127331475567,-0.00120064516918614
"Fscb",0.811181975053526,2.38063019770262,1.98777675008093,1.197634592
31812,0.231059302992688,NA,NA
"Tmem18",1269.70375589799,-0.0559231060445093,0.0819438789893582,-0.68
2456172861574,0.494950546687113,0.630919571084292,-0.0257568901039159
"Spice1",1077.16341945492,0.217075555811641,0.152092386185392,1.427261
16182462,0.153504630921802,0.272937560980604,0.281875262820967
"Dpy19l1",1193.10305067468,-0.793753440287124,0.136925921002114,-5.796
95527682351,6.7529690374664e-09,5.96319630519015e-07,-11.3764624681513
"Fam89a",95.1188498626019,-0.762590893247593,0.230388373960073,-3.3100

2333207904,0.000932881946200251,0.0055662270292408,-3.95863817423529
"Synpo",7892.23401366231,-0.562567002485236,0.379271294982514,-1.48328
389184099,0.137999000237607,0.251491214752114,-0.776537798763092
"Tmem82",1611.94001560211,1.8248746575628,0.424234119285984,4.30157447
174261,1.6958875676922e-05,0.000252608218448148,15.1166609385155
"Il17re",1680.46342409213,-0.162336964688755,0.242203145135051,-0.6702
51266135446,0.502697627976187,0.637543797988793,-0.0730731115471789
"Zfp866",1069.12323595525,-0.224406452295891,0.108678595380136,-2.0648
6338465235,0.0389359284364812,0.096024883968443,-0.525817510382904
"Tuba1c",4591.03384452664,0.122381836079476,0.176504322103733,0.693364
528532912,0.488080816844799,0.624724785109099,0.057573809065542
"Hic1",707.834146396114,-1.58208672035965,0.272253752165251,-5.8110740
7254157,6.20732887026242e-09,5.60381183856537e-07,-22.7735823932284
"Qrfp",6.29976643612892,-1.78239847484689,0.833265425208194,-2.1390524
8066851,0.0324314182964112,0.0834782566261948,-4.42599678226495
"Lrrn4",20.9849834902414,-3.95819714992401,1.32934506748073,-2.9775543
2111038,0.00290558150940541,0.0131996803985057,-17.1293461945188
"A530016L24Rik",1602.59241038801,-4.76668286659616,1.47249494688212,-3
.23714718117654,0.00120731126766457,0.00678977649175767,-23.7968884587
28
"Mob1a",3865.99644709995,-0.749922967275312,0.180052407877763,-4.16502
60394431,3.11317030550112e-05,0.000401990692825569,-5.86370889117947
"Tmem186",1033.92200289701,0.0938702744812471,0.171025778363336,0.5488
66231626348,0.58309725371075,0.707305602392199,0.0325065677224944
"Aqp6",2052.04177564407,0.947841200016551,0.556618521032649,1.70285602
113652,0.0885950159771995,0.1798839551727,1.62596786397579
"Crppa",255.843055810646,0.213855739713903,0.2104223474405,1.016316671
28119,0.309478612246728,0.454554725543255,0.168611770234651
"Ppp2r3a",4051.14703533097,0.880566137840222,0.174824155771807,5.03686
766827347,4.73211689155206e-07,1.51177569890021e-05,9.77396760657065
"Hpdl",38.8635099797041,-0.11221908558343,0.379311034109647,-0.2958497
78920461,0.767344805591833,0.849739749379717,-0.0182720898447198
"Arl11",133.081047372793,-1.13687561582726,0.392712578583597,-2.894930
48561787,0.00379242506073744,0.0161351356379485,-4.69160832238285
"Pyurf",2023.20856190057,0.217403652249443,0.315552683258446,0.6889615
07170527,0.490847491311384,0.627329862715611,0.101371579321399
"Tmem212",0.232709513347443,0,4.78471990595262,0,1,NA,NA
"Lor",27.6031773459452,0.460228549275535,0.455546646082482,1.010277549
4745,0.312362334117941,0.457412000798469,0.359977314505389
"Simc1",392.394967304286,0.544524153824155,0.314190769593005,1.7331004
1707947,0.0830778222566641,0.171014714776229,0.961632745751093
"Rfesd",517.39847379299,0.315177757376304,0.233816855051486,1.34796850
854444,0.177668511519808,0.304436298234749,0.374838831376046
"Gm1840",0.632874726207147,0.861334323926041,2.53969608722726,0.339148
580910093,0.734497802520164,NA,NA
"Zmpste24",3379.18832389452,0.243284876783246,0.186963600337537,1.3012
4193342463,0.193175655945156,0.323419108674107,0.274621488658924
"Hoxa6",88.4684746844588,-0.610936044142384,0.321315837982903,-1.90135
677088812,0.0572552977308963,0.129029564858647,-1.2510221171455
"Gm4835",0.18827594438763,-1.53871353359654,4.7633584801309,-0.3230312

26815048,0.746671593171412,NA,NA
"Olfr1009",0.52786307638529,2.97197620866614,3.54539868104436,0.838262
908077431,0.401883062899703,NA,NA
"Fam124b",7.66887897303805,-1.88917664196212,0.67459812221749,-2.80044
752533873,0.00510318036777177,0.020195212261176,-7.37215237167038
"Upf2",2729.36966768734,0.0566692005009849,0.177195807548782,0.3198111
8111603,0.749111471118766,0.836598629009319,0.0101104007255036
"Fam129c",203.711194492498,-0.173960177824903,0.312753490122158,-0.556
221379838019,0.578059525352955,0.703119440132995,-0.0612737326667015
"Exoc3l",439.532717184305,-0.161413523002379,0.224164253934436,-0.7200
68075838666,0.471483082120247,0.609414541244381,-0.079941104709397
"Tmem64",5586.92704806727,-0.332786752291571,0.435475619108564,-0.7641
91467188908,0.444753150733782,0.585932225203194,-0.177891542055295
"Pigv",1167.85186648598,0.304003129971029,0.108334534091756,2.80615163
502293,0.00501370924983982,0.0199227497125525,1.19044372819792
"Fam13c",385.19903231077,0.0358626545246468,0.153252735110854,0.234009
882425302,0.814977306585589,0.882212657080868,0.00449438476301368
"Uevld",675.284584978368,-0.337212151579866,0.146495340952607,-2.30186
263526946,0.0213429199422349,0.060494876149687,-0.945946384217358
"Ifi209",636.158840840132,-1.12552896395889,0.419627432882557,-2.68221
016015866,0.00731374975554898,0.0266448977454585,-4.08021987873336
"Olfr1031",8.79278166498351,-0.423630180021792,0.693375212239321,-0.61
096816347622,0.541220657097758,0.671719947300579,-0.168568282191845
"Trim56",3084.4719487726,-0.219600627287737,0.190667182681159,-1.15174
842466185,0.249424467030265,0.389031138161435,-0.207324050076572
"Teddm1b",8.92894158034772,0.0750273637340244,0.637428995232981,0.1177
03092101422,0.90630291667165,0.942923678875182,0.0044093532155023
"Tmem11",1579.30025720942,0.0583738367832922,0.210209918651038,0.27769
3065854787,0.781247983977088,0.858750809703298,0.00888896312263467
"Pnpla1",627.517901841401,2.39435406999488,0.441311512526228,5.4255418
2710695,5.77789969947279e-08,3.05304846450733e-06,30.4067881739188
"Mei4",10.3537749636717,0.559227825382034,0.722849923619287,0.77364305
799743,0.439141901837846,0.581131315463168,0.303536857767879
"Zfp784",357.390093230169,0.293866476219696,0.151062654460375,1.945328
42858775,0.0517354626841889,0.119363158373721,0.624638080397361
"Smco3",73.2508784614279,-0.247779850509855,0.510763313900241,-0.48511
6772811624,0.62759351486946,0.743924758521778,-0.0732970907048511
"B3galnt1",486.508420627106,-0.1439097828476,0.308451179541119,-0.4665
56111283781,0.640817493611154,0.754371761784114,-0.0405638475728603
"Kcnj6",0.117614786697194,0,4.78471990596623,0,1,NA,NA
"Vmn1r75",0.134652852526316,1.38440950995805,4.78471990595898,0.289339
718346707,0.772321420636896,NA,NA
"Olfr571",37.4076417158625,0.308900925802664,0.348514270760732,0.88633
6519673639,0.37543621541113,0.520532982157876,0.201682040486642
"D17H6S53E",921.364963663945,-0.246373100163354,0.157896732848823,-1.5
603432428158,0.118678789618385,0.22459468504118,-0.367947853049214
"Olfr131",0.0929913071132142,0.911659748055755,4.78471990596623,0.1905
35656417207,0.848889405144033,NA,NA
"Pcdhb19",44.8559138587839,-1.29457088801309,0.299677009930007,-4.3198
872289718,1.56108955175091e-05,0.000239293428302195,-10.7938990746333

"Olfr30",5.28560438528935,-0.560658230968581,0.863542564699899,-0.6492
537297955,0.516174387798855,0.649775209071087,-0.241715914871477
"Cox8c",0.223297874701269,1.37099285966478,4.78471990595911,0.28653565
6550614,0.774467887773744,NA,NA
"Fbrsl1",3649.57334915358,-0.0338808344697529,0.160616847156183,-0.210
94197196392,0.832932552039248,0.893072251837297,-0.00383150877032393
"Olfr975",12.5649466914316,-0.250130024733867,0.561849782382686,-0.445
190213784757,0.656182298320947,0.765755473585218,-0.0667577989336912
"Rhbdl2",465.012643358495,-0.951018271690725,0.196737083902824,-4.8339
5531144738,1.33846645177857e-06,3.44293125023463e-05,-9.77323650950316
"Filip1l",1740.98136263575,-1.13893304027168,0.228854951797072,-4.9766
5893321629,6.46911620267635e-07,1.9358130187683e-05,-12.3601547189863
"Hoxd9",2493.61023614217,0.712913606779403,0.228112437770189,3.1252728
4241128,0.0017764021007788,0.00913645163835007,3.34747385817295
"Olfr78",163.839860256509,0.0904417288999174,0.336728252545572,0.26858
9666047334,0.788245465473197,0.863438768864966,0.0132797665561931
"Hexim2",198.827321581933,-0.41650908378494,0.269354374122002,-1.54632
381650608,0.122026382857713,0.229548520304843,-0.612951783916951
"Olfr1342",0.223214397671005,-0.0691395152828403,4.78471990596524,-0.0
144500653416812,0.988470917183191,NA,NA
"Gprasp1",2710.09984437056,-0.298444872829398,0.131805445863718,-2.264
28332208655,0.0235566946851514,0.0652890451984479,-0.814435470597244
"Olfr267",6.4202528549881,0.0219240126231866,0.6812348680406,0.0321827
517229784,0.974326311185415,0.984077077375299,0.000351903517781805
"Tmem130",15.2296846551206,-0.100772121617844,0.610570448808487,-0.165
045854764995,0.868907890047146,0.917816026877906,-0.00864204733852025
"2510009E07Rik",1875.85032011944,-0.0211913827905531,0.127259671932179
,-0.166520803242732,0.867747113101148,0.91708673701806,-0.001834182488
67441
"Gpr135",295.040087418074,0.642436182929914,0.200526879208704,3.203740
99205564,0.00135654455141486,0.00744648245030601,3.14794597721894
"Hfm1",1.55137908775743,-0.5143749751333,1.68955588293823,-0.304443895
776194,0.760789729877407,0.845188726921585,-0.0865154692487654
"Usp48",4279.89200868201,0.162683856818598,0.157830084335422,1.0307531
514262,0.302656592695084,0.447263774774045,0.130896530653371
"Otud1",559.917589789568,-0.36829393042534,0.223343593119349,-1.649001
54636866,0.0991473179119857,0.19600267714163,-0.600181718578704
"Lrit2",0.864376212958859,4.67577634212402,2.34874745748734,1.99075312
555148,0.046508034988654,NA,NA
"Rnf227",134.850788729837,-0.180229531971574,0.217669062111969,-0.8279
97925947157,0.407671674488247,0.551462627249329,-0.107269230596696
"Hilpda",240.556182842179,-1.41876762930317,0.28783878801081,-4.929035
58657943,8.263652278497e-07,2.38350136281264e-05,-15.1018661634418
"Eif3j2",1625.50423186354,0.461138493420152,0.322266324918501,1.430923
61119881,0.152452109271411,0.271437423666695,0.601335501301831
"Psapl1",82.8447675817921,-3.72161893069792,0.942248315347349,-3.94972
203195289,7.82420031690144e-05,0.000817557651548469,-26.4576928701056
"Leng9",379.581815932528,0.108182025169695,0.139611533001399,0.7748788
57383583,0.43841124645034,0.580519014145245,0.0588329253935126
"Epop",169.498259677376,0.623626224155926,0.453465529985195,1.37524504

712913,0.169055486879496,0.293312322759958,0.764888346236334
"Gpr149",0.196401232263413,-0.544957266022007,4.7847199059633,-0.11389
5332795304,0.909320765382157,NA,NA
"Pgp",2269.97435964009,0.131094994117433,0.247963469664808,0.528686722
663806,0.597022789210016,0.719319677687505,0.0431891679107114
"Gjc2",196.804787863466,-1.5767333975573,0.474115934766983,-3.32562835
782399,0.000882194699936533,0.00535357368794352,-8.24630141873674
"Magea10",0.15302085674268,-1.26591390880537,4.77380538540979,-0.26517
9203298565,0.790871414939366,NA,NA
"Zfp536",58.0426989549864,-1.35731951575947,0.398570142687103,-3.40547
213749784,0.000660497530817504,0.00427569085520574,-7.4039195059558
"Pcdhb12",42.8912684650083,-0.712792031226815,0.271171243545071,-2.628
56791859032,0.00857452310073075,0.0299956779076628,-2.49954922540911
"Elfn2",3.15089820241873,-2.04209495787508,1.43516999366146,-1.4228941
2884476,0.154766848485743,0.27453428832271,-2.63977350066798
"Sptssb",1833.88096215989,-2.90693089625099,0.984886657395606,-2.95153
850894676,0.00316195115795631,0.0140561913731867,-12.3971658480618
"Rab9b",1.88522195458327,-0.748534228112122,1.40345039434843,-0.533352
82182142,0.593789369143451,0.716530828045488,-0.249511913450662
"Zbtb37",1479.36359929603,-0.235120807735033,0.130029303516494,-1.8082
1400543155,0.070573199675818,0.151051706568944,-0.444409614891199
"Adam30",1.87723308572148,0.431829638063169,1.95687696807005,0.2206728
60434888,0.825347164761967,0.888309727231665,0.0511436589237631
"Gm6863",7.77707020633462,0.779111260852541,1.06074882963658,0.7344917
46853463,0.462649088361226,0.601903778265643,0.395521817652371
"Krt34",0.309236822951277,2.75316189084849,4.71739224594471,0.58361945
4840804,0.559476368148288,NA,NA
"Acot6",408.001398372347,0.260597695049168,0.184913477038243,1.4092953
0515114,0.158747866486701,0.279760113951345,0.33195528008478
"Tril",1103.90523923992,-1.3826176607224,0.294084852624515,-4.70142426
032296,2.58353093024e-06,5.85060233191865e-05,-13.4755182862286
"Lgals2",4.07347115231433,-1.62868265978194,0.862488905745396,-1.88835
201117673,0.0589787092059849,0.131816468603656,-3.3002724972114
"Gimap5",493.600160343065,-0.225215503155288,0.206319389759074,-1.0915
8670650529,0.275014801777735,0.418434538046217,-0.196215588741588
"Hscb",771.873171695591,0.652252891361352,0.153858999446496,4.23928982
840014,2.24228011407106e-05,0.000313854828888424,5.26145013318751
"Rai2",262.125615328646,-0.367891015123391,0.175226105602032,-2.099521
72285865,0.0357709349567364,0.0899834459794151,-0.885929228057634
"Sorcs1",138.422851684366,-1.48527131622591,0.610362070697323,-2.43342
662909742,0.014956666339424,0.0460921574657957,-4.57034687883691
"Setx",1849.87291351286,-0.308959349270742,0.112917965305294,-2.736139
89089703,0.0062164592904619,0.0235114583677566,-1.1586801713392
"Casc1",104.606685605863,0.217368799877003,0.323862465094761,0.6711762
65558905,0.502108246722455,0.637127853351192,0.097986579639426
"Zc2hc1a",1342.56532770599,0.0585893165393495,0.239681071701024,0.2444
46990008595,0.806884652569814,0.877024383612742,0.00768811844781249
"Fam90a1b",0.884744766658689,-1.31838273419425,2.07595755036036,-0.635
072106346972,0.525381388307709,NA,NA
"Fbxl7",278.375940955111,-0.0930040402621986,0.280951606802306,-0.3310

32241889408,0.740620131717055,0.831038594211122,-0.0172130986841923
"Mdga1",101.392866370506,-0.488881769689718,0.461622289544279,-1.05905
148161791,0.289576334521883,0.433338830566076,-0.408820212094209
"Pars2",239.867498035479,-0.133429089952715,0.222415436054822,-0.59990
9306293954,0.548566679892991,0.678487554610266,-0.0517556952703793
"Pxylp1",559.933853772203,0.288628294330491,0.23426277690531,1.2320706
6074844,0.217922689945097,0.353205038117166,0.300377355313614
"Unc5cl",47.2717324980629,-1.67092944975162,0.548575860479747,-3.04594
053462422,0.00231953704896337,0.0111926656384197,-7.50664481988973
"Zfp3",209.202428606142,-0.285316567828181,0.188197792502214,-1.516046
30444762,0.129507644275215,0.239533106835844,-0.407735532558979
"Mmp3",325.734700584225,-2.18502117625484,0.396137080115123,-5.5158208
7599536,3.47155709465695e-08,2.02247398202931e-06,-28.6482257252849
"Vps37d",758.3056276513,0.30417996440034,0.355700983433752,0.855156377
314297,0.392464582274345,0.536926355963036,0.189167796258696
"Eif5al3-
ps",0.100341358323926,0.857183598344413,4.78471990596623,0.17915021468
1441,0.85781975412629,NA,NA
"Ubxn10",127.843103676549,0.631774160581923,0.495264453853438,1.275629
92996239,0.202086368753221,0.334091912663243,0.692638737516255
"4933412A08Rik",4.78140615349856,-1.23334166258298,1.21527514270833,-1
.01486619715959,0.310169613357925,0.45532955287252,-0.970311610120226
"1700019D03Rik",72.9026717742089,-1.46392278192933,0.462899711709585,-
3.1625052790004,0.0015641789958278,0.00831018039799742,-7.012591184371
83
"Ecm2",616.123472505756,-1.16252613069112,0.245947427220539,-4.7267261
2935565,2.28168672248879e-06,5.28364937233805e-05,-11.4489159011136
"Fam221b",22.4660892244979,0.292377743784845,0.664551504973827,0.43996
2503427572,NA,NA,NA
"Adamts3",13.0551379956812,-1.46954126579422,0.601659113004378,-2.4424
8152156474,0.0145866763335514,0.045331688739494,-4.54639176328276
"Rbm20",407.025745730517,0.115293668195755,0.291347146734706,0.3957260
93383501,0.692307103866141,0.794456118602909,0.0265287878145413
"Pld6",29.3201925063311,1.18609805507323,0.331483410674788,3.578152079
03991,0.0003460321005369,0.00258602295755787,7.06633824380263
"Npsr1",0.565067600416269,3.42557817434843,2.84101616043256,1.20575807
419091,0.227910785533925,NA,NA
"Gm9785",0.383535217732872,-1.56333306405424,4.68855883811918,-0.33343
5735378629,0.738805392053998,NA,NA
"Tmem221",275.082390509653,0.776625340960672,0.287219458537506,2.70394
40326055,0.00685218292935992,0.0253614014344457,2.85373069829456
"Tox3",371.643263859839,1.04587464176071,0.375722255146313,2.783637720
24477,0.00537530367016994,0.0210035412747442,4.04028091068939
"Diras1",1.81073277744611,0.189863643859044,1.60034660566802,0.1186390
7680161,0.905561305158293,0.942441117052384,0.0112554655661407
"Dpy19l3",628.233754725401,-0.0845814691433397,0.176510226575835,-0.47
9187358059395,0.631805346970044,0.747191160018313,-0.0246499347355978
"Kcns3",378.74228564483,-0.844898177487855,0.197007690595495,-4.288655
81304964,1.79757676458258e-05,0.000265322874760266,-6.95736733726236
"Fam25c",601.145649729264,-1.21886820464966,0.532370732405127,-2.28951

016736607,0.0220497278446714,0.0621369552703286,-3.38652093995685
"Fem1a",1930.72940579447,0.0445183925995572,0.192486723986607,0.231280
327689793,0.817097028646226,0.882841508619313,0.0055474184901874
"1190005I06Rik",149.173978673371,-2.05911704905106,0.762706101151311,-
2.69975164213687,0.0069391255841273,0.0255957379189016,-7.547342317436
82
"Pde12",922.58205904006,0.231404999379734,0.126950348371463,1.82279924
669944,0.0683338018781162,0.147402640670374,0.44304509249759
"Capn13",415.300294770169,-2.2749428756321,0.681338086966699,-3.338933
95826453,0.000841005559791471,0.005160186564254,-11.9816294266112
"Rpl7",27788.0488468774,-0.148980222855566,0.157353403070785,-0.946787
422122343,0.343747112301034,0.488403855594947,-0.106761111171559
"Col6a6",380.351505152349,-1.09980885316604,0.38919665366109,-2.825843
54932235,0.00471562899009384,0.0189812828341969,-4.359974325527
"F830045P16Rik",0.124244059077056,-0.541888455620593,4.78471990596623,
-0.113253955564858,0.909829220433712,NA,NA
"Ptpn11",4758.10715572937,-0.653837674540627,0.218538922072568,-2.9918
5915414882,0.00277284159886101,0.0127541555778703,-2.85197333173599
"B430306N03Rik",83.1798947444044,-1.40510905954871,0.381033699480535,-
3.68762411688073,0.000226357691377225,0.00186618277652141,-8.829508938
64161
"1520401A03Rik",9.72321755422698,0.0817186624666644,0.716580845417836,
0.114039696970988,0.909206325063334,0.944413356295671,0.00467359909403
975
"Dmrta1",6.63827680937665,-0.940018443794484,0.722739787404069,-1.3006
3192891433,0.193384472587957,0.323647020064466,-1.06043649971015
"Pkhd1",4040.91936596212,0.758090380800411,0.372711336123157,2.0339879
8836081,0.0419528065401856,0.101601766478765,1.73352099616557
"Gm12481",880.753766628864,0.750488553493257,0.270520309222847,2.77424
107509439,0.00553306594292681,0.0214225019370904,2.88436272694393
"Bicdl2",249.881079621102,0.137122258720489,0.23691670350964,0.5787783
49897604,0.562738741352769,0.690732135907856,0.0507356714175999
"Defb12",0.177714481740462,0.899380045712553,4.78471990596623,0.187969
215207579,0.850900783701441,NA,NA
"Vwce",482.44710700301,1.87855228406927,0.650729714148685,2.8868395636
8409,0.00389132598314926,0.0164547980957372,7.71547378413246
"Prr33",1.24635414822484,-1.77660275762003,2.20848346844976,-0.8044446
71196529,0.421140211109447,NA,NA
"Oaz1-
ps",1939.01979512153,-0.105472527996828,0.370977274527251,-0.284309943
597583,0.776172866550455,0.855191308976103,-0.0164990761912634
"Ly6g5b",51.5740778799422,-0.0562017000963435,0.378463375176479,-0.148
499706398635,0.881948417646344,0.926371289515079,-0.00429831524605276
"Rtn4r",182.058720464275,0.490937585504595,0.383750048787926,1.2793160
2107992,0.200785793092541,0.332420938585239,0.540695693487024
"Adamtsl5",936.990718318572,-1.11784116205805,0.234877624163833,-4.759
24927305264,1.9431433721305e-06,4.64876189813503e-05,-11.1519462160678
"Lysmd4",605.187269093821,0.287445279387415,0.143049970581718,2.009404
67319572,0.0444942350378085,0.106322536162412,0.644244784003122
"Clec4a3",373.149372930171,-1.35804765839711,0.376414094815262,-3.6078

5548974625,0.000308738360908991,0.0023598611341799,-8.21503739215596
"Tmem145",19.1861876811521,-2.02490351909248,0.82415151620315,-2.4569
5540113882,0.0140120080788464,0.0439886736259378,-6.32544037969522
"Clrn1",4.70937075974898,-0.613612617906002,0.876207024495923,-0.70030
5522269705,0.483736523789657,0.621336939449982,-0.292007057835636
"Mgat5b",39.2335612647576,-0.832104833509075,0.618224935027052,-1.3459
5805889432,0.178316043008268,0.305302600300028,-0.98725232916368
"Taf10",3547.08062117261,0.524345712459846,0.163850873274971,3.2001398
7096609,0.00137360909562614,0.0075118898092302,2.56471551221109
"Zmym1",411.940445214675,-0.32925759568471,0.189225870285015,-1.740024
21121793,0.0818547667475635,0.169211645678964,-0.584960692852032
"Chil5",3.75569558706434,-1.85418722479137,0.964733510768248,-1.921968
2991159,0.0546097467564225,0.124245139119805,-3.86690511461632
"Olfr323",0.11403309701371,0,4.78471990596623,0,1,NA,NA
"Kbtbd7",725.310262292211,-0.0915540058504124,0.252709668409091,-0.362
289288046562,0.717135862163977,0.812537997085031,-0.0190059341358323
"Slc36a4",734.271339871339,-0.524442142633581,0.159675544461641,-3.284
4237005847,0.00102191164031517,0.00597279171548718,-2.68542740791773
"Gm8399",2.43888610756081,-0.782950036521275,1.30518150188903,-0.59987
8281593853,0.548587357597232,0.678487554610266,-0.303697818193077
"S1pr2",493.627548358779,-1.17991827985322,0.323125236790009,-3.651581
9425767,0.00026062991072634 6,0.00207165185460346,-7.29119762899026
"Zfp469",167.73165739465,-2.05030899391076,0.459322610331423,-4.463766
74649515,8.05312311167888e-06,0.000143659464255732,-18.1412670238541
"Trp53bp1",2158.49107437742,-0.0658099629340008,0.133855906970532,-0.4
91647805639901,0.622968341936281,0.740676320055275,-0.0197555957103144
"Olfr922",0.419353641084299,-1.02624826846306,2.38405285033699,-0.4304
63724123399,0.666858349291353,NA,NA
"Ccdc60",24.8568943078379,-0.103546375941256,0.36576603334162,-0.28309
4564564296,0.777104348092884,0.855850687930132,-0.0161179613712559
"Ccdc84",1586.61707059389,0.520201463111542,0.219453096031146,2.370444
85823847,0.0177666938992261,0.0524600222192964,1.53339987171374
"Ncmap",98.3325686221779,-0.995030651849718,0.563976875851825,-1.76431
108163226,0.0776796192621254,0.162580207854543,-1.80755657478212
"Olfr544",0.0837058623428615,-0.544048378937457,4.78471990596623,-0.11
3705376621746,0.90947135024012,NA,NA
"Klhl15",451.995179669317,0.0823985616136492,0.262843828519307,0.31348
8667692255,0.753909428991429,0.84012836751871,0.0143538772527467
"Gimap7",222.738860072679,-1.57049665446364,0.487429297234577,-3.22199
888963144,0.00127299628472475,0.00707181383926476,-7.77653134912674
"Klri2",95.5408207597472,-1.95570639846018,0.407124906717658,-4.803701
18897309,1.55759227352723e-06,3.89436729509225e-05,-19.8570580795489
"A530064D06Rik",87.9968606768375,-0.617078604870434,0.316736746060027,
-1.94823812691909,0.0513864767023066,0.118770599889683,-1.314723842947
26
"Wdfy3",5048.84807398713,0.168545280637379,0.221646811355879,0.7604227
62711258,0.447001921258095,0.587981074053183,0.0895077441404932
"Naalad2",316.37773163473,-1.00224815927635,0.18680023570006,-5.365347
40183965,8.0793561935005e-08,3.88966140891044e-06,-12.4851941892945
"Adam6a",0.128230219553068,-0.069788710760181,4.78471990595917,-0.0145

857463199177,0.988362670833664,NA,NA
"Olfr691",4.91953211691702,0.0130518890369712,0.89078396270427,0.01465
21374243738,0.988309704056613,0.992279228099683,0.000101161685745886
"Ccrl2",257.138505328315,-0.931829980965132,0.297816697115219,-3.12887
084569548,0.00175479421023616,0.00904679468830902,-4.38458132009604
"Thrap3",6798.05114291015,-0.236640268414272,0.152660894378747,-1.5501
0403533451,0.121116547664285,0.228211291685179,-0.349632059183392
"Orai3",1319.43885317549,-0.614845367530354,0.253650818587215,-2.42398
337586656,0.0153513082016613,0.0469521266378026,-1.88058250579812
"Emx2",1987.12088577688,0.730217433310509,0.46150590632577,1.582249378
18035,0.113592652015881,0.217289269989761,1.11469573091146
"Opn5",1.78092369813069,-0.191616801339053,1.17342139265672,-0.1632975
18298363,0.870284187787863,0.918523389276158,-0.0162851114679318
"Spata31d1d",0.143938970413971,1.56167753579723,4.76352019991278,0.327
841065064829,0.743031836253549,NA,NA
"Cep164",1911.57315212215,-0.0964181401020293,0.178283579083892,-0.540
813352511054,0.58863623848085,0.712119462548838,-0.0327348839055058
"Pura",5415.2734027376,0.464957287169683,0.218331324844443,2.129594951
62206,0.0332050682095362,0.0850095089219455,1.14611606717983
"Mgat2",2521.9114537778,0.246720750710758,0.202390618763747,1.21903254
319687,0.222831840995953,0.358781753964932,0.25289888577714
"Gpr75",16.4249028382508,0.0590011341990998,0.446204401936418,0.132228
938000274,0.894803212865721,0.934987837323463,0.00396615996416459
"Gls2",47.7289978860009,-0.438146705658141,0.470436899881817,-0.931361
263897903,0.35166672117125,0.496689440738648,-0.306610821574355
"Cilp2",13.7125176888108,-0.128995418996869,0.510427747523949,-0.25272
0232437632,0.80048441206083,0.872629155001792,-0.01757493019252
"Npy5r",4.90355812406413,-5.35423950429408,1.94184614050239,-2.7572933
7799587,0.00582820342451825,0.0223082666610241,-20.3610911013382
"Adgrd1",128.788127875226,-2.23057187801777,0.511069882789572,-4.36451
44296953,1.274054399713e-05,0.000204784613314521,-18.9454777849311
"Mrpl50",2996.40904986962,0.615978926957914,0.267240164546431,2.304963
88147109,0.0211685963384832,0.0601199483775878,1.7317715157799
"Muc19",21.2282050706407,1.19992963371639,0.566390705241467,2.11855459
9523,0.0341281260223709,0.0868481076982756,2.93214154296238
"Pcdhb21",21.6503073016402,-1.20868182336969,0.407548244179263,-2.9657
3924837728,0.00301956381619173,0.013600458267013,-5.19449360259604
"Rell2",54.1294939858446,0.134397784021331,0.289932778602776,0.4635480
84038694,0.642971557176563,0.756131448843705,0.0375695624299399
"Slc35g1",1578.1156278455,-0.579444865789372,0.282329292246171,-2.0523
7246613482,0.040133478285343,0.0981460882231814,-1.34506433215016
"Irf2bp1",1483.83477648705,-0.081017417647185,0.108946935513586,-0.743
641087886148,0.457093638858712,0.597106510147874,-0.0417774231560989
"Ccdc141",2803.94474884053,0.707391736440759,0.360429746626189,1.96263
416952212,0.0496886973814933,0.115777990146314,1.52519374082726
"Npb",30.7041891478834,0.986929842107016,0.370143197666804,2.666346020
48105,0.00766807241928889,0.0275699937361783,3.54409198758091
"Als2cl",5149.97549944935,-0.629578210577295,0.31275899080477,-2.01298
197361907,0.0441165257944697,0.105712672967994,-1.41468143983176
"Krt13",4.90560077297854,-2.96707454688623,1.40675225239348,-2.1091663

7370794,0.0349302233179037,0.0882786123464013,-7.20185369921943
"Fmn1",1172.22895930544,0.657139193148534,0.347601272474202,1.89049708
728355,0.0586915097657245,0.131355263706623,1.33389378733815
"Pcdhb14",108.605209219451,-1.2056032792791,0.275937364444703,-4.36911
93532462,1.24748576792787e-05,0.000201186745269294,-10.2612234624048
"Ccr10",11.9836576279633,-1.61171914908422,0.732175316670959,-2.201274
90286392,0.0277165684640199,0.0740364568469885,-4.19562349652197
"Otos",8.54083488678386,0.292439491788415,0.961477337850585,0.30415640
6267644,0.761008735131505,0.84523772814549,0.0491700029711159
"Efcab9",4.46581619234855,-2.51047392089245,1.02976876469935,-2.437900
63065799,0.0147728342850079,0.0457157596491842,-7.74559579190504
"Gm9788",0.241801009321614,0,4.78471990595701,0,1,NA,NA
"Cep68",978.270608947563,0.0973891054803592,0.115484570465129,0.843308
375206418,0.399055999992136,0.543313587587925,0.0594140368301616
"Gpr22",4.43271962831365,2.36496983441317,0.901834265637155,2.62239961
878394,0.00873129846828756,0.0304548940133579,8.25731245601159
"Zrsr1",1521.73801729322,0.128197844726252,0.217637261838684,0.5890436
39141511,0.55583199990055,0.685144287145083,0.0484749157661087
"Tafa2",1.36584521537147,-0.93369964802651,1.47339232835138,-0.6337074
1794974,0.526271782980469,0.658785774560425,-0.389685966232023
"Eml6",346.680478779058,-0.266770938639777,0.269653902165187,-0.989308
652675664,0.322512151892499,0.46766157345294,-0.202748681994878
"S100a1",9641.83904967357,0.492318459322551,0.28877084737098,1.7048759
0352248,0.0882175713307479,0.179285616615272,0.846184719614297
"Efcab8",16.8683819666106,-2.4386633974331,0.875771333662382,-2.784589
20005393,0.0053595577154512,0.0209588159518387,-9.42591179443502
"Lmod3",21.0631380993781,0.989504970971975,0.497453855035839,1.9891392
1553726,0.0466858388751701,0.1103504044597,2.18096244548284
"C130050O18Rik",94.1394012215821,-0.340999840396818,0.382008607844491,
-0.892649624627393,0.372044835262005,0.51725042834118,-0.2247966885624
15
"Rsbn1",1701.79439541836,0.267749683253212,0.241732061962518,1.1076299
9777302,0.268021642684955,0.410225740857897,0.238577734978639
"Il1f9",100.064204325242,1.49291788243877,0.401594699964448,3.71747406
669194,0.000201224635245941,0.00170142148223129,9.51927917297739
"Bmerb1",8.86448024506338,0.304844482545898,0.651131537730029,0.468176
497192326,0.639658378050369,0.753507676125308,0.0862758885314548
"Proca1",435.38167033531,-1.34576302209284,0.563601960726539,-2.387789
81598647,0.0169500325249945,0.0505957951886624,-4.01560452452188
"Pheta1",1207.00012755875,0.546147234408621,0.220566718020637,2.476108
99463772,0.0132823022783362,0.0421308491659346,1.72963466354856
"Prss53",168.523851463925,-0.116007912025158,0.176683591338605,-0.6565
85657707371,0.511447373404025,0.645658846657927,-0.0507516072223021
"E130201H02Rik",0.896747081904376,1.00357229902447,2.18830277932958,0.
458607606088191,0.646515973627545,NA,NA
"1810024B03Rik",161.976432684496,1.07659446545976,0.180892205807232,5.
95158017259755,2.6556587586949e-09,3.09850777959792e-07,16.13510967594
08
"Arf6",1969.12897241191,-0.754486388462145,0.188542167634188,-4.001685
12927044,6.28929581371886e-05,0.000690088293617841,-5.4916732904482

"1810030O07Rik",1668.58294381288,-0.102197418376172,0.096965796309454,
-1.05395327286358,0.29190431091646,0.435766806082929,-0.08489008395390
85
"Nkrf",268.245079213569,-0.448786733047173,0.166125282039747,-2.701495
68769308,0.00690283754882235,0.0254956481392192,-1.64670959942847
"Bclaf3",602.662237109003,-0.142910802626808,0.162075745924157,-0.8817
53169247682,0.37791030151622,0.523058907526772,-0.0926149444376904
"Lsm8",937.800174595875,-0.0575536516585179,0.131336933308377,-0.43821
3762182057,0.661231333205739,0.769988565148441,-0.0150433512951531
"Hepacam2",898.867969665478,0.258758747646879,0.395828870170681,0.6537
13680700708,0.51329628761126,0.647210449295633,0.112581730923832
"Tnip3",130.928887270895,-1.58296668747917,0.648290429536489,-2.441755
44687734,0.0146160438827402,0.0453795579021764,-4.89563085126262
"Rnf182",44.873177223763,-0.0872352284209994,0.545079320975587,-0.1600
41346394989,0.872848504251174,0.920379906001015,-0.00723779810845446
"Bcl2l15",27.2041962336797,1.41976308289209,0.529064734244496,2.683533
77383867,0.00728486167663828,0.0265733039396239,5.15068491408903
"Foxo1",3136.10794604765,0.287485153862664,0.153107301771682,1.8776710
8776674,0.0604261742657845,0.134281917692531,0.577216666601907
"Ptx4",0.102773349507105,-0.0520891204135723,4.78471990596623,-0.01088
65558355089,0.991313956752974,NA,NA
"Spink10",3.96741617131925,1.49878912579832,1.68065646539963,0.8917879
15409547,0.372506620233803,0.517722865586905,0.986675645737177
"Wfikkn2",29.1454261834523,-1.20498992988663,0.411675754734263,-2.9270
3642619044,0.00342208797867952,0.0149538035428717,-5.06431913810678
"Nkx2-6",0.200004388940312,0.914187463617524,4.78471990596588,0.191063
9455567,0.848475494505895,NA,NA
"Gpr146",1460.40678968606,0.00531511257167751,0.207946187121438,0.0255
600386102466,0.979608260217061,0.986845872266198,7.03795822466594e-05
"S1pr4",138.12998886091,-1.15922747901898,0.354557356509931,-3.2695062
1030624,0.00107735372349453,0.00622960940115548,-5.88706910500543
"Cdc25c",29.899205156336,-1.13401658833971,0.570506036752833,-1.987738
10491862,0.046840662294344,0.110662696403141,-2.49627496632564
"Olfr983",0.269684169908007,-0.0732154415645561,3.95821540425547,-0.01
8497083682167,0.985242304053111,NA,NA
"Vsig4",1.25899251207904,0.289535881473444,1.33914334358136,0.21620977
5347214,0.828824234128403,NA,NA
"Gm7887",1.14178204665377,-0.924090359800087,4.10760405534803,-0.22497
0651345262,0.822002105876644,NA,NA
"Olfr1094",0.28006539364761,0.957602240392341,4.7847199059617,0.200137
575284016,0.841372986982997,NA,NA
"Kcnj4",4.79360905678649,-1.34203736081884,0.952367756593488,-1.409158
75356717,0.1587882309867,0.279766626857931,-1.70948651765453
"Aqp5",20.3638109847872,-1.52870524454778,0.919807612722596,-1.6619836
8376499,0.0965160460222912,0.19204713392618,-2.52238574005843
"Nkx2-3",0.369870519760414,-1.96936337674366,4.73729535352032,-0.41571
4712674652,0.677618773975231,NA,NA
"Grsf1",4385.19169638081,0.531578144620382,0.289972062390702,1.8332046
8957504,0.0667721367591031,0.144722445812471,1.02750734997393
"Dnajc21",2139.04996231806,0.452276048391401,0.145032082419044,3.11845

517796974,0.0018180179202813,0.00928076027956245,2.1165667921279
"Nxpe4",305.697858207121,-0.932129462190248,0.253502735125853,-3.67699
962577322,0.000235993386601394,0.00192519681799471,-5.82835112410854
"Nhlrc1",120.6830450961,0.318073662758028,0.292502459147555,1.08742218
333821,0.27685027181191,0.420374316625545,0.275645732451228
"Bhlha9",5.09288033273263,-0.220091082912506,0.892468810648502,-0.2466
09271143694,0.805210628788352,0.876115895095719,-0.0291085635643271
"Il20rb",208.261447826084,-0.728792404734569,0.345848132164997,-2.1072
6135825096,0.0350949309074723,0.0885800599137727,-1.76648238002039
"Defb29",1196.79900090289,2.0430969981524,0.70361948106674,2.903695894
05756,0.00368786179884317,0.0158069750173322,8.4733443110838
"Pced1b",587.158542513363,-0.598130444614099,0.276609833281597,-2.1623
6146603358,0.0305903253284789,0.0798247006497165,-1.51202728354881
"Osbpl1a",2320.31648139687,0.64109067437266,0.284885445359241,2.250345
48031839,0.0244270229820351,0.0671665624176732,1.73131648566165
"Pcsk9",112.166927466296,-0.269282565138756,0.366593858111586,-0.73455
2855102091,0.462611859138832,0.601895504436738,-0.136707064759199
"Ctla2a",1002.99627825653,-0.713547151684819,0.192667737003169,-3.7035
1135474791,0.000212635659227843,0.00177329772610115,-4.5202600861605
"Gm4895",0.0892211881005411,0.881785905194793,4.78471990596623,0.18429
2063595042,0.8537843396306,NA,NA
"Crb3",1200.69046920294,0.887545852596833,0.251986604465232,3.52219458
046348,0.000427989881991254,0.00305563775872042,5.13957118907623
"Gm1821",520.080168031423,-0.240190837692874,0.346432196664041,-0.6933
27121456336,0.488104286406401,0.624724785109099,-0.112996355273193
"Nrn1l",37.2018759387314,0.224466309329835,0.356786507217657,0.6291334
02718338,0.529261724060318,0.661636538804134,0.0927133194812306
"Cnr1",93.398068451359,-2.02915320292814,0.462215693921968,-4.39005691
414429,1.13320997317169e-05,0.000188365968089528,-17.4042983293106
"Olfr794",0.646137397889329,0.638357416071215,2.05253328112976,0.31100
953243489,0.755793374926419,NA,NA
"Krt84",0.0907556969386152,-0.0576292604836612,4.78471990596623,-0.012
0444376298394,0.9903901615196,NA,NA
"Zfp879",46.1445589649669,-0.0813916157423307,0.267434167822916,-0.304
34262160632,0.760866876855693,0.845194957606807,-0.0136890897456749
"Cdkn2a",45.7286393289024,-1.70928717339989,0.378072533482802,-4.52105
620488732,6.15318467667853e-06,0.000116256100862452,-15.4856547028498
"Ubr3",4081.6622081342,0.33821377894588,0.182200928575143,1.8562681408
4207,0.0634153477919113,0.139148373986798,0.667030110956756
"Apol7c",146.039431705147,-1.71901527519674,0.572016658215678,-3.00518
394089948,0.00265420283559416,0.0123394484712482,-7.55499298396477
"Mab21l3",292.37063878368,-0.710764541178622,0.278899599014045,-2.5484
6024767081,0.0108199617492234,0.0360443557468577,-2.36187412050876
"Gpr4",431.106104734686,-1.13748460713609,0.313594288297285,-3.6272491
2278299,0.000286456881706213,0.00222789640187784,-6.94627435693019
"1700001O22Rik",84.9712275084542,0.37711518577377,0.32543568237699,1.1
5880097418732,0.246537320335371,0.385669501917448,0.359305728391769
"Dsc1",0.160338031895853,-0.343793665344574,4.78471990596465,-0.071852
4118655304,0.942719361935952,NA,NA
"Trp53i13",762.431226218269,0.0154116572929786,0.145304505148877,0.106

064552349481,0.915531135585065,0.948362433280434,0.000817103510141836
"Ackr3",2170.40079020104,-0.0776042324230949,0.270298550090133,-0.2871
05618573305,0.774031450209582,0.85386507588042,-0.0122600787105864
"Aplnr",722.24497183475,-0.907571451813576,0.312374436898921,-2.905396
03952052,0.00366788672877964,0.015744719124635,-3.76755622858417
"Alkbh2",277.997649656417,0.181454237701114,0.177269971676273,1.023603
91884353,0.306022393491833,0.450496385677897,0.144692557219209
"Phlpp1",776.943812267977,-0.0993915521739323,0.180197045877323,-0.551
571484926548,0.581241977495398,0.705625806438283,-0.0346548724948562
"Marveld1",2573.78048166786,-0.809552375366587,0.175221205394272,-4.62
017353176508,3.83419219591152e-06,8.11716389097534e-05,-7.625129011872
83
"Slc25a53",202.332012192386,0.362590800887506,0.267882849468625,1.3535
4242202046,0.1758824070805,0.302252350895081,0.433837359308929
"Snhg11",6486.48328190718,1.26815803275794,0.344058290792928,3.6858813
3666334,0.000227912534025798,0.00187551751242224,7.96260025273579
"Lacc1",857.143605209665,-1.09178606122025,0.204232462985033,-5.345800
78633365,9.00181732919984e-08,4.20905118188821e-06,-13.5144262598055
"Sowaha",96.6646071540085,-0.0658663661413635,0.560842272551962,-0.117
441871565166,0.906509904919368,0.942925053407193,-0.00387086597594815
"P2ry4",0.284242492515149,-0.56569161680078,4.76354477898689,-0.118754
34010743,0.905469983925661,NA,NA
"BC024139",3.54186224753361,-2.02450379869527,1.24349937622952,-1.6280
6981442473,0.103510083205483,0.202573482117887,-3.23242922069966
"Ccdc89",21.2784866327483,-0.791357109786397,0.424882217243212,-1.8625
3290363858,0.0625280119656397,0.137669438419858,-1.56918188724206
"Tmem74b",13.2807590838216,0.0102668689609288,0.542367656289622,0.0189
297220102784,0.984897169050344,0.99028078600163,0.0001002739818778
"Cxxc4",60.846004807796,-0.714798880361747,0.335442346464817,-2.130914
26259958,0.0330962061631724,0.084813450231217,-1.76362409063209
"Slc16a13",1756.46954732325,1.21334198890888,0.295419254841523,4.10718
654598115,4.00507755323043e-05,0.000488068763330543,9.25179849356906
"Pcare",0.120701025406302,-0.999956691173237,4.78169649435351,-0.20912
1740025541,0.834353203805696,NA,NA
"Gm13080",0.714738378865713,-1.21489295084536,2.39605713453199,-0.5070
38389584419,0.612127882653407,NA,NA
"Slc15a5",12.0070103917329,-5.12597944650476,1.95311018281028,-2.62452
138728247,0.00867708429019589,0.0302874667902326,-17.9256592530663
"Tigd3",87.3876091995312,-0.0116403177194201,0.263404087069858,-0.0441
918644805726,0.964751466923839,0.978756652379469,-0.000249943636248211
"Dsg2",1138.56703570667,-0.661789065083393,0.262343596264487,-2.522604
22783942,0.0116489423275365,0.038173290336522,-2.16115108640466
"Sowahd",14.4329124966264,0.106658892752367,0.705996831684905,0.151075
596894421,0.879916082389448,0.925033901678409,0.00831138266424265
"Adig",891.400171970094,-3.480807123825,1.29215362526237,-2.6938028542
2194,0.00706419349780238,0.0259344087403786,-12.712550414033
"Sptssa",2639.58437372051,-0.0990581684670518,0.211002285875695,-0.469
464906770767,0.638737363329327,0.752786575809673,-0.0281298971029434
"Gm9493",2.62754603663521,0.320647954077004,1.09199266364337,0.2936356
30304676,0.769036346320485,0.850568420248274,0.0518970070957171

"Cryga",7.56575607957508,1.23396020259442,0.628571887785071,1.96311706
993862,0.0496325725254473,0.115699150130461,2.66135846245892
"Camsap3",1676.17861792098,1.07249122922835,0.260604131025244,4.115403
79275286,3.86502187025102e-05,0.000474859460782321,8.20723019754519
"Gm9791",0.504352279450406,-0.596897586243271,2.37996101471222,-0.2508
01413364936,0.801967648788831,NA,NA
"Olfr1442",8.18116087772692,0.783412409126098,0.782482016611259,1.0011
8902734515,0.316735430341305,0.461907810081882,0.605099873934654
"N6amt1",1807.39072904987,0.826984416010872,0.146637554155547,5.639649
54798441,1.70396606688235e-08,1.23051593970637e-06,11.2536676219196
"Pfn3",3.58478303946646,1.42401532770964,0.978188209038,1.455768240255
2,0.145456698220383,0.261827428166638,1.90827974082314
"Dock5",1441.35817431037,0.161434478683249,0.0912765862880917,1.768629
66997606,0.0769556960281216,0.161238143257717,0.29459739820296
"Zfp507",874.044346272083,0.306632984415575,0.363125072342049,0.844428
016049356,0.398430272410402,0.542834139039722,0.187337869106252
"Ffar1",20.7541716180358,-1.74128862954219,0.867357491400986,-2.007578
93579682,0.0446880547954715,0.106662711764243,-3.89714961128105
"Rin3",1234.00788666249,-0.454678707111658,0.222730683314997,-2.041383
34397613,0.0412127387013982,0.100120356242196,-1.04638950794243
"Stk-
ps2",0.297298717109934,0.875696148334188,4.78471990596018,0.1830193126
3382,0.854782865263828,NA,NA
"Shisa2",83.5145530224837,-0.667906229806935,0.321465144972736,-2.0776
9408364189,0.0377375443236334,0.0937754591947663,-1.5808352534409
"Fam160a2",2214.47817343561,0.544949511668592,0.146615380286137,3.7168
6456499352,0.000201710500824789,0.00170479035330808,3.47367883054609
"Tent5c",489.22784692975,-0.180887735448015,0.304417369954067,-0.59420
9638810391,0.552371906734896,0.682302656795502,-0.0691501146620066
"Tnfaip8l1",129.569433044004,-0.822463944711515,0.222350317255306,-3.6
9895557093877,0.000216488489168532,0.00180157599735725,-5.197226520185
19
"Ascc1",2631.95035873024,1.35637466276933,0.283109150132196,4.79099549
462099,1.65955853515917e-06,4.05489628051798e-05,13.7170174473208
"Zfand3",4625.65325034511,-0.244120895622238,0.154059004695783,-1.5845
9348808788,0.113058725449449,0.216416554959097,-0.373639293742285
"Klk1b11",11.5916211623036,1.96029159722539,0.773237627559316,2.535173
57065634,0.0112391622378908,0.0371362794974402,6.45555564677144
"2510039O18Rik",2702.65254162216,0.13071238318586,0.147677130200175,0.
885122720144146,0.376090446725164,0.521254729190165,0.08516126565831
"Hs3st5",1.77446108794787,1.50045568071759,1.26751130947807,1.18378090
15964,0.236499804220869,0.37465489342245,1.47307230593672
"Zfp758",539.996843761172,-0.127423828893174,0.128022148561232,-0.9953
26436286357,0.319577524211833,0.464665729102311,-0.0976623363866692
"Bod1",3613.17214980389,0.682585105828508,0.158204984322328,4.31456131
898982,1.59920142351911e-05,0.000242090290029313,5.68333997306378
"Lingo4",63.5338491681555,1.03793085374328,0.41110807845926,2.52471529
538706,0.0115792054412088,0.0380022467080776,3.3941480611314
"Foxe3",0.334388967438133,-0.954088314838287,4.78136783357248,-0.19954
2965119549,0.8418380377479,NA,NA

"Zfp488",762.499664750199,0.343736631003687,0.16882989297714,2.0359938
9268244,0.0417509685430476,0.101163214198129,0.78750752533061
"Znrf4",0.278522765516491,-0.0433012352174915,4.7847199059559,-0.00904
989969498344,0.992779323319753,NA,NA
"Tram1l1",29.445847775672,-0.90289041449147,0.389151114258692,-2.32015
374338942,0.0203325618875534,0.058197928902454,-2.56773502537241
"Rps2",39856.0405438345,0.149832376911221,0.151857947762505,0.98666141
034349,0.32380865393052,0.468717233432612,0.113536323587438
"Ackr2",37.6719135242556,-0.989757981214537,0.463360844535999,-2.13604
14736935,0.0326760334810432,0.08398593472362,-2.45173537237571
"Prop1",0.127971505821787,0,4.78471990596577,0,1,NA,NA
"Dact1",448.615794133464,-1.06089983696798,0.202498301888193,-5.239055
47392563,1.61400521012288e-07,6.55622366395332e-06,-12.6619411690531
"Tceal3",152.654036366958,-1.33547540554163,0.309556807550111,-4.31415
292110945,1.60216021875997e-05,0.000242350038366034,-11.1180029406001
"9930012K11Rik",198.918140615787,-0.385174865083795,0.279883607887684,
-1.37619658396845,0.168760779419601,0.292827060347369,-0.4730614011381
83
"Tex38",28.7717986307484,1.28190628892683,0.431396949790787,2.97152376
610107,0.00296325938175729,0.0134117064590306,5.52710219888653
"Rasip1",1427.09600048689,-0.542654727606724,0.252734852249998,-2.1471
3057093505,0.0317828881152602,0.0821420489653091,-1.35625979660573
"Cage1",58.5661662686413,0.193631426867022,0.277845279027898,0.6969037
85964922,0.485863008292999,0.622960277220813,0.0916404338688482
"Acp1",3262.45215328681,0.404451514785052,0.148024959831762,2.73231970
638418,0.00628900815232539,0.0237090257065043,1.51341690955943
"Garem2",3.84611080329158,-2.27280077440436,1.31102354847775,-1.733607
89517728,0.0829876786609727,0.170908323241829,-4.01519345838522
"4932415D10Rik",1.03526408421962,2.5845405426995,2.87227967302261,0.89
9822035776789,0.368214965587567,NA,NA
"Tlr7",271.810913785242,-1.06300939797285,0.359641147123697,-2.9557502
1510882,0.00311909583089038,0.013926295056263,-4.54327718116383
"Dnd1",136.803108951162,0.092531087218749,0.221200682053093,0.41831284
7681634,0.675718397424353,0.781305658126077,0.0228356394998622
"Smim7",3531.72307476968,-0.195695650879939,0.23886534716809,-0.819271
833273615,0.412631339073526,0.556088322453388,-0.114839715597482
"Gm9294",0.416589745726892,3.07716016992034,2.95889286082329,1.0399701
2215716,0.298353781944426,NA,NA
"Zbtb39",695.944738425095,-0.306203296421521,0.139937885480393,-2.1881
3722510065,0.028659608075751,0.0760382417010469,-0.78893857587061
"Gm4922",0.157863633167365,0,4.78471990595081,0,1,NA,NA
"Liph",114.433256947439,-1.42304259380149,0.431085385415375,-3.3010689
8063897,0.000963171987909208,0.00570471671332131,-7.35209541803307
"Swi5",6647.64350578252,0.0339140354584755,0.262014939383531,0.1294355
0294601,0.897013056664171,0.936594226134626,0.0022215438766988
"Rnf208",484.029264544468,1.10009121455394,0.220280252413476,4.9940528
145438,5.91252175074411e-07,1.795675219564e-05,12.0212955326457
"Cnrip1",349.047575382075,-0.739330379937787,0.204894495469457,-3.6083
4671641043,0.000308154494081748,0.00235757766721658,-4.47303796132233
"Csrnp2",414.408368156229,0.179690405409567,0.156948269005174,1.144902

11678374,0.252249707031142,0.392415834268372,0.168088370894702
"Pard6b",1747.32025821618,0.480189815934691,0.383174106322912,1.253189
62845058,0.210136703559127,0.343959490093676,0.512473644675203
"Gm7334",12.1700885747321,-2.36996422095944,1.27371533444931,-1.860670
24307601,0.0627907585340667,0.138135418018417,-4.69139343506398
"Zbtb7c",706.839894024297,-0.838117304385962,0.267789511798062,-3.1297
6150095818,0.00174948280455446,0.00902896128194888,-3.9452846293432
"Csrnp3",92.3463216734913,1.58877266780179,0.484426265857626,3.2796996
7728532,0.00103917643395407,0.00605735788019025,8.11303834282599
"Prss42",0.901305357286799,-0.76931009978386,1.49343956692267,-0.51512
6367897882,0.606464718152209,NA,NA
"Plppr4",242.739654229849,0.649046167791056,0.445293071665988,1.457570
7754957,0.144958889856484,0.261201090504158,0.871321573220575
"Fzd1",2997.66812524299,-0.672784045589434,0.242386438319702,-2.775667
03093367,0.00550885942702385,0.0213627170063864,-2.58760014866017
"Zfp612",1108.22660657562,0.702635587985407,0.206122400680263,3.408826
91869738,0.000652428595845728,0.00423752590333112,3.83904324696215
"Ly6k",35.8146727984031,-2.11251882045555,0.887222412464681,-2.3810476
2771607,0.0172634797553359,0.0514121255189118,-6.26970495378367
"Cnpy1",38.8758060725828,0.981212944021756,0.743795427232731,1.3191973
3315964,0.187103149634196,0.315913884789154,1.13063756459707
"Tmem201",1290.93740987239,0.116977009138841,0.134309647478207,0.87095
016132644,0.383781366262159,0.528420950454034,0.0746151958191904
"Il27",21.5601298650185,-1.07353510402289,0.625405218061472,-1.7165432
4751311,0.0860626595709127,0.175798598953227,-1.86625088406989
"Palb2",164.057236731805,-0.143155695059074,0.485284627021727,-0.29499
326186705,0.767999027046766,0.849936453053083,-0.0232762131918362
"Phf11a",192.986864870697,-1.33451943542322,0.460997646633566,-2.89485
086348823,0.00379338710454179,0.0161351356379485,-5.50723615006672
"Ccnjl",88.40757372451,-1.59164763939017,0.328836618107539,-4.84023843
983719,1.29683437434672e-06,3.37512266435095e-05,-16.3883900448976
"Kcnj10",1233.28743760465,1.5232618490622,0.597537692078835,2.54923140
28974,0.0107960638406476,0.0359954946588488,5.06387304044954
"Gemin7",1544.12831169952,0.286941926820496,0.186690286197053,1.536994
4128621,0.124294677571621,0.232670662758398,0.418399001868538
"Slc38a6",2348.31747395029,0.273112306174716,0.201364091361425,1.35631
08711598,0.175000278021809,0.30122953181074,0.327702742446981
"Gskip",886.585841502831,-0.316051351285025,0.127062368182849,-2.48737
179862893,0.0128690827035775,0.0410404603458218,-1.00921418289475
"Dok7",38.500512197153,0.388072422226237,0.391752617800753,0.990605817
530519,0.321878095902225,0.467066987713149,0.295432778296203
"E230025N22Rik",11.2606574868198,-0.809345528519717,0.669335358507456,
-1.20917790795405,0.226594503214387,0.362742831626959,-0.8207258562705
37
"Gpr152",6.68683753241232,-1.83546221862421,0.898182850714168,-2.04352
846100856,0.0410001529189352,0.0998153304549131,-4.22970061666949
"Erich5",3.45381236742462,1.36708057767423,0.890211061754541,1.5356814
09058,0.124616536850628,0.233049418330475,1.99115935784051
"9930104L06Rik",892.871941722757,-0.37714308919154,0.164874269624639,-
2.28745874083424,0.0221690610400059,0.0623830786777362,-1.046368873624

72
"Serpinb1a",712.413169670273,0.405500842425654,0.357346681505483,1.134
75474493649,0.256478092548475,0.39733114957176,0.374271282668601
"Klk14",2.54955387730979,1.73951190474006,0.82984410990346,2.096191180
94653,0.03606523496372,0.0905135733456832,4.17875196707358
"Defb1",7423.26768421121,1.25301855744743,0.515417339264397,2.43107567
788802,0.0150540698485311,0.0462785048340853,3.85062336669792
"Abca6",30.5175355021392,-1.27998141921889,0.417347384571728,-3.066944
86784042,0.00216258740265288,0.0106131711998303,-5.81835967657759
"Atp5pb-
ps",0.646247531394873,-0.581504373329052,2.3843727621574,-0.2438814863
84244,0.807322608045648,NA,NA
"Gm6430",1.69253976084293,-3.05930215834364,1.71387363987059,-1.785022
00347433,0.0742577479118949,0.157104770918633,-5.66228604355251
"Trmt10c",901.250554486894,0.184738891534319,0.180556313638369,1.02316
494954769,0.306229862146651,0.450733739875851,0.147214449121553
"D1Ertd622e",1679.13618383922,0.4869279389037,0.215606372644831,2.2584
1162731223,0.0239200084929913,0.0660425269890038,1.32320544203348
"Scml4",203.560900551044,-0.951195574379443,0.425088903911235,-2.23763
915178098,0.0252445962272636,0.0689962345443211,-2.54321479153137
"Hjurp",4464.68806920673,-0.159619834837149,0.206645900365446,-0.77243
1655091471,0.439858811356887,0.581685234931944,-0.0864861464629835
"Zfp36",3464.05648995,0.408594013153908,0.19611513165589,2.08343950670
18,0.0372111803395302,0.0927212261035349,0.971701062425542
"Spata32",0.399909702550995,-0.0536751919318006,3.10742130121542,-0.01
72732264887308,0.986218644583664,NA,NA
"Fads6",787.329796625392,1.40532886817881,0.338509465229932,4.15152015
682706,3.30274169668087e-05,0.000419551884686937,10.9282917025815
"Setd2",2461.42194423127,0.143449566949388,0.147172202577754,0.9747055
79157181,0.329706324686071,0.474471467911212,0.106949368828111
"Isca1",4803.50348734685,-0.267977902459896,0.409670266577847,-0.65413
0710286668,0.513027597505274,0.647025361976574,-0.116669482020956
"Cyb5d1",207.914542457368,-0.961020761524412,0.398939194454692,-2.4089
4044727299,0.0159989083744252,0.0485067198700889,-2.90809969755794
"Olfr923",0.127104318644313,0,4.78471990596583,0,1,NA,NA
"Zfp354c",407.330372807397,-0.421257545270598,0.220180788775628,-1.913
2347904334,0.0557180015427601,0.126280320130273,-0.871687630598905
"Cd300c2",778.661029425667,-1.06329124261621,0.402493346595346,-2.6417
6104179235,0.00824762227483322,0.0291206099561982,-3.76012650739504
"Shb",2272.00106087297,0.637845174728889,0.141262984525141,4.515302978
15115,6.32263053200689e-06,0.00011888008689785,5.76445894979417
"Olfr543",0.818769352751442,1.56174839061987,3.46271412104471,0.451018
575610476,0.651976161055736,NA,NA
"D630023F18Rik",918.761724982158,1.0293079301951,0.582489251397515,1.7
6708484787587,0.0772140196770378,0.161727248433009,1.87523849153058
"Oxgr1",838.807513723917,1.20287577179346,0.514448671336391,2.33818423
25858,0.0193776930894471,0.0560739477379103,3.46558609681552
"AY074887",6.82335984656125,0.342851787890628,0.662010687100991,0.5178
94642142424,0.604531780448425,0.725586502318834,0.109978276243792
"Olfr545",1.75629213218718,0.78122419126371,1.22512306481633,0.6376699

72673995,0.523688535945131,0.656648171952293,0.328588293772231
"Tlr1",494.158523527256,-1.161452946692,0.48778747536971,-2.3810634863
3018,0.0172627365562381,0.0514121255189118,-3.44705439920821
"Ankrd45",189.867028410608,0.671642292014994,0.498700056675216,1.34678
607516664,0.178049140726403,0.304927724715275,0.797696452263065
"Lsm11",466.083733351578,-0.364547823939728,0.159786635504458,-2.28146
629903573,0.0225208687369918,0.0631089247821021,-1.00720666035985
"1700056E22Rik",55.0213119605039,-1.47980102257951,0.509589087700709,-
2.90391034324624,0.00368533678836277,0.0158031002198147,-6.13754746116
511
"Lemd2",3198.50724876723,-0.10567961537301,0.0843811048261597,-1.25240
852902708,0.21042104004338,0.3442226225997,-0.11270378772682
"Gm1123",11.2279121447665,1.66986689480791,0.581255034938275,2.8728643
9589335,0.0040676867291708,0.0170050934488363,6.80344244701124
"Defb36",69.8529010712739,-2.23400733426306,0.775411341449766,-2.88106
094771092,0.00396338992249298,0.01669796385237,-9.14260462636998
"Ankrd50",1319.81498010079,-0.960173850201653,0.202083977859592,-4.751
36059954631,2.02052432975474e-06,4.81027880116703e-05,-9.5462120512556
2
"Zfp444",2373.01456545162,0.987011269666609,0.299139494571304,3.299501
69595991,0.000968566423088834,0.00573318400649243,5.09444310277158
"Coa4",407.538006632769,0.282526898952828,0.182566912749204,1.54752520
431203,0.121736651632628,0.22913613606844,0.416286143389646
"Uqcrq",12373.3763677252,0.702964536630767,0.419794535929246,1.6745442
7455732,0.0940236806351399,0.188298451625304,1.17375891343808
"Psg22",0.169759183421915,-0.0613667411439099,4.78471990596551,-0.0128
255660414728,0.989766959420302,NA,NA
"4930503L19Rik",819.754607396217,-0.497553955546164,0.214829766239851,
-2.31603824858544,0.0205561804066811,0.0586485814412449,-1.41115849406
754
"Syt16",116.97966480278,-2.91193301954061,0.900276403932906,-3.2344877
7155513,0.00121861138939546,0.00683754960300222,-14.5169349753174
"1700029I15Rik",232.710193611694,-0.442571617003876,0.339567567675369,
-1.30333889079472,0.192459089256997,0.322440911009875,-0.5009182148435
38
"Rassf9",372.034268970816,-0.341149742554047,0.233611433477197,-1.4603
2981980459,0.144199450362213,0.260116651231606,-0.459400802770551
"Olfr1030",0.441167619032422,-2.46820375865554,3.0341679188362,-0.8134
69730311517,0.415948794277982,NA,NA
"H1f10",294.00059054177,-1.47077474420572,0.349764934583358,-4.2050377
2328612,2.61038614590215e-05,0.000351015924640001,-11.6995409217262
"Sstr3",2.50438705865419,1.06195003256772,1.2818653298849,0.8284411847
40581,0.407420690633804,0.551225184893259,0.632510044001902
"Zfp367",1157.59785630108,0.204927804467719,0.127071963912897,1.612690
93635941,0.106811670662149,0.207060444876774,0.322708937922624
"Ttc41",343.36408592973,1.16097333884798,0.342442833687535,3.390269045
33597,0.000698240606885694,0.00445348228755553,6.28558970830113
"Klhl31",28.9571007187884,0.379573180075441,0.567983999977843,0.668281
465833982,0.503953941919371,0.638804704170062,0.170108187343192
"Cfap43",20.124856668097,-0.996048383502679,0.464353794341408,-2.14502

044699639,0.031951212206344,0.0824846997539215,-2.48528261425954
"Ubtd2",75.3609483992129,-1.65030829669545,0.338180587339949,-4.879961
64911889,1.06106471809455e-06,2.92399786079693e-05,-17.2291751396078
"Pwwp2a",1282.19240708455,0.179738630245549,0.157422887883337,1.141756
65725781,0.253555183028284,0.39392979750482,0.167441374100582
"Mylk4",44.4827684047033,1.11363067893169,0.410662713978414,2.71178911
799191,0.00669211524695224,0.0248965584974384,4.11266671539601
"Kctd21",327.626091412641,-0.745256158418254,0.29475861916165,-2.52836
08687607,0.0114596496392243,0.0376980897343412,-2.44305838736876
"Pp2d1",2.70842813733147,0.691763508935058,1.2087738377209,0.572285308
75499,0.567128701018361,0.69419771564166,0.252492619730399
"Fbxo48",15.52204536412,-0.871966181173697,0.586302796241917,-1.487228
41979064,0.136954492411929,0.250008303814978,-1.20877283794599
"Napepld",532.369498023353,-0.966904889243925,0.218653395459912,-4.422
08952305612,9.77509362737038e-06,0.000168055436579517,-8.4035798756046
"Wdr72",869.28747522497,1.3758861118023,0.38733548077506,3.55218197168
291,0.000382050545908261,0.00278997061577501,8.0925826611238
"Sft2d3",409.625191881271,0.0856504219248278,0.168923388760114,0.50703
708085361,0.612128800911147,0.731687552732071,0.0267573371300859
"Tst",3324.11951011547,0.50649746961513,0.53282698447097,0.95058524507
3534,0.341814961663556,0.486329899419402,0.365117858434211
"Shld1",376.048805284392,0.300579734322583,0.150131201728275,2.0021136
9030808,0.0452725054508478,0.107622934806222,0.670028749808992
"Spata21",0.241348162149924,1.82494259336562,4.03034767703832,0.452800
289107234,0.650692555428154,NA,NA
"Fzd5",634.35881870967,-0.0178641559385998,0.254895588505026,-0.070084
2099440554,0.944126634435912,0.965564196694897,-0.000626008055491731
"Tubg2",59.1762565889174,-0.341655032505501,0.341089678501848,-1.00165
74937305,0.316509042707556,0.461719405451449,-0.264030298449868
"Prrt3",2.14138101792142,-0.00403574121278949,1.379761041697,-0.002924
95663439361,0.997666225588118,0.998383086969673,-6.53072378415981e-06
"Gm4779",6.81035874745609,-3.27476207194423,1.52310911942946,-2.150050
85989566,NA,NA,NA
"Acer1",3.12255080227198,-1.27308558013821,1.25809097195673,-1.0119185
4048373,0.311577003629302,0.456673563614532,-0.997827222484252
"Prss22",175.1889151056,-2.54047152412051,0.687901558017058,-3.6930742
4080222,0.000221559306586662,0.00183515860655342,-16.0065567888526
"Olfr1443",7.18976109647,3.67831379797301,0.748613733106791,4.91350029
434779,8.94645983497827e-07,2.53543712009311e-05,38.9259742913697
"Cetn4",528.991906545882,1.18435663565403,0.23489936392817,5.041974639
0467,4.60752363898198e-07,1.48916463357915e-05,13.1637807205182
"Ankrd34b",22.4462463257371,1.0304723821553,0.533871169432776,1.930189
23132739,0.0535833953561908,0.122553553390617,2.16317501722197
"Tmem232",2.13290985061939,1.51319547032069,1.42455047838652,1.0622266
4151191,0.288132808016588,0.432188129141275,1.26941105697205
"Prkce",2186.74441384913,-0.0476686545237325,0.284192464126767,-0.1677
33703531524,0.866792780230339,0.916273245901195,-0.00416817839387885
"Megf8",4503.77905637965,-0.323347962435759,0.117191625096388,-2.75913
882216252,0.00579539101358681,0.022217564684018,-1.23094433709779
"Lrfn4",933.171380880366,-0.218113632757553,0.314499342039419,-0.69352

651532802,0.48797919178858,0.624646683396385,-0.102637534917299
"Gm9795",0.850691894689445,0,4.78471990594274,0,1,NA,NA
"Prlhr",0.285564750435065,-2.14258505567375,4.74570250051814,-0.451479
007679014,0.651644351481625,NA,NA
"Kcng3",1.1061899473126,-0.781105102082558,1.66221994684164,-0.4699168
14298087,0.638414450805715,NA,NA
"Rpsa-
ps2",2.66771968191952,-3.14030546581509,1.44544864692315,-2.1725472381
8774,0.0298144106318168,0.0782927719505601,-7.99930008951786
"Pcdhb7",56.0056523714925,-1.23528314879782,0.273973497991001,-4.50876
875995645,6.52049306431034e-06,0.000121549501211257,-11.1363110064784
"Zc2hc1c",327.805466744723,1.48170574658247,0.486806866712866,3.043724
00617024,0.00233669404554775,0.0112635007416786,6.64721024929904
"9930022D16Rik",1.20762726029114,-1.48532108677421,1.3736298892236,-1.
08131098371319,0.279558803211821,NA,NA
"E130308A19Rik",611.354853054444,0.218818252659251,0.212059720109359,1
.03187089253162,0.302132603289985,0.446722005254968,0.176327859591609
"Rnf216",1937.29393899142,-0.125376537332867,0.0924651730725766,-1.355
93254375307,0.175120631803447,0.301365979497278,-0.150380364386584
"Lingo2",8.52329527540409,-0.742884343972393,0.72621014957194,-1.02296
056370223,0.306326492175655,0.450760277440248,-0.591944817996467
"S1pr5",10.5213764282824,-2.30377593011325,0.675522944617529,-3.410359
2608799,0.000648773582114524,0.00422222540189219,-12.5956482637352
"Aqp12",0.209498224295285,-0.543373207678298,4.78471990595409,-0.11356
4266740489,0.909583215051028,NA,NA
"S1pr1",1378.43812992837,-1.33245631574049,0.384908008533035,-3.461752
64271264,0.000536670152644619,0.00365618261225184,-7.47685966387915
"Arhgef37",305.577989759034,0.259659406644925,0.222424297832888,1.1674
0576085807,0.243046545763095,0.381893911619698,0.249951371680837
"Magi1",1402.78037803111,0.490207894559187,0.147386502115623,3.3260026
360801,0.000881010954077559,0.00534805466457168,2.56428847626093
"Kmt5b",3093.48592656502,-0.0522082749843979,0.142203845001512,-0.3671
36873013826,0.713516924141103,0.810023463575151,-0.0109998692330574
"Slc25a26",754.585168201656,0.423058359118421,0.243606325303789,1.7366
4767772695,0.0824493784239119,0.170152199461202,0.749262563106074
"Poln",92.1888367420491,-3.56034014128829,1.34317609980147,-2.65068753
219667,0.00803281189766548,0.0285805149962289,-12.6571163228767
"Dmd",720.068413075198,-0.272817597045205,0.138350767662752,-1.9719268
7582503,0.0486179547337546,0.113964039600715,-0.592524988099514
"Gm5514",3.0475700459716,0.16300759097109,1.05203146041621,0.154945547
832382,0.876864245251037,0.923069811786239,0.0130488247513971
"Ccdc73",22.6149332007636,-0.102526740411051,0.413275817554332,-0.2480
83086539591,0.804070121626854,0.875265700730259,-0.0136594100916587
"Saysd1",520.921371810964,0.33297188146108,0.18412456557432,1.80840552
384999,0.0705434088486715,0.151016182008278,0.629439483608885
"Prrt2",55.2522537323862,-1.84267010980829,0.642879390184494,-2.866276
53326929,0.00415331270067661,0.0172520858623333,-7.48091306193551
"Rpl18a",27326.1797994856,-0.233797822704145,0.22036293662807,-1.06096
708585233,0.288704859499191,0.432680042314775,-0.195865705607413
"Olfr620",8.49033851093553,0.136111947094036,0.706347654282502,0.19269

8236157231,0.847195304907923,0.902454387901199,0.013970139577674
"Tubb2b",2025.34331244568,-0.364944363078564,0.42160948405715,-0.86559
8087516207,0.386710604757177,0.531103991797945,-0.230935857051637
"Pigw",318.213299772236,-0.241686633439187,0.195581101894604,-1.235736
12735564,0.216556663953409,0.351868486146964,-0.252441155006743
"Bola3",2064.52413837715,0.234181479563989,0.375026513532393,0.6244397
95891288,0.532338796697924,0.6647160972l525,0.0956386041176633
"AI467606",333.728798417043,-1.19717013074006,0.325622584105951,-3.676
55742929219,0.00023640265377075,0.00192642442812267,-7.48481495043006
"Amer3",0.36620761869006,-1.41140293084187,4.76788395328713,-0.2960229
20161219,0.76721257766305,NA,NA
"Borcs6",890.525099459088,-0.213025164454199,0.198820831883806,-1.0714
4287867528,0.283970341667164,0.427952830563175,-0.180803467704062
"Sox3",0.315794374828854,-0.486056741965076,3.2162463809982,-0.1511254
68756602,0.879876742070465,NA,NA
"Shroom2",813.755156498808,0.454735398215163,0.231644027976672,1.96307
844491876,0.0496370597415788,0.115699150130461,0.980756022499804
"Cirbp",3380.01084265189,-0.190500681555877,0.154922486086872,-1.22965
159137103,0.218827601679153,0.354259430217546,-0.197687468459574
"Lrrc3b",44.8906401875828,0.119685864434641,0.31520353461168,0.3797097
78896007,0.704160860405754,0.80365361648451,0.0261617654201906
"Olfr835",0.200136460738522,-0.0674720501675185,4.78471990595068,-0.01
41015673840395,0.988748949988819,NA,NA
"Dpy19l4",1456.31370196169,-0.0600125602800865,0.0811177714605859,-0.7
39820130650974,0.459409142458762,0.599087711320288,-0.0307472709453124
"Vcpip1",1805.31874023242,-0.240908989222889,0.0778913339492819,-3.092
88565246237,0.00198220496347579,0.00992527796041369,-1.11123377493617
"Nudt18",1060.22760183173,-1.27012534671242,0.449198702754715,-2.82753
565164673,0.00469077919650363,0.0189046739920273,-5.04029694876837
"Asxl3",176.286875279936,-0.0379449148291503,0.30705673100109,-0.12357
6235262583,0.901650809746308,0.939775885413669,-0.00235690482071212
"Hs6st1",5064.64706823513,0.317473287227339,0.215589177520535,1.472584
52802948,0.140863099234828,0.255421622698845,0.43330013875523
"Olfr1152",245.790078617446,0.0133247948296798,0.317788399299359,0.041
9297710648264,0.966554683383376,0.97966851466492,0.00027370480885592
"Rln3",7.48715933831374,-1.56590816702872,0.860142664709694,-1.8205214
4519215,0.0686796334763059,0.148001270283047,-2.99172145520845
"1110012L19Rik",566.003291783032,-0.161846854697326,0.264062112459152,
-0.612912065233749,0.539934485096895,0.670726059780292,-0.064640740700
2105
"Kcng4",20.1288387327482,-2.73352581917919,0.691365893200163,-3.953804
84641262,7.69181540298555e-05,0.000805884023253155,-19.4724644811896
"Med26",422.992752550084,0.0437531229860579,0.102168310754507,0.428245
535851025,0.668472367013677,0.775825845954325,0.0111057331058262
"Zfp688",716.495027224706,0.423042909205708,0.15696782599561,2.6950931
2830477,0.00703689602750253,0.0258634116388774,1.54619052250697
"Zfp574",1107.5796611345,-0.183077166320684,0.121121533966375,-1.51151
624591799,0.130656972635244,0.240971128906701,-0.260533115106431
"Morn2",793.894202384999,1.30066744124196,0.267118126117846,4.86925938
027935,1.12017292366061e-06,3.01607735157191e-05,13.5386062532798

"Klhdc9",131.558184831146,1.25615534687834,0.255896199715608,4.9088472
133403,9.16133149448723e-07,2.5888063982538e-05,13.2671717848052
"Zfp691",657.093607420336,0.164117827001321,0.171881854852728,0.954829
275853117,0.339664039874835,0.484333000546989,0.11898256747859
"Cenph",81.1628814265072,-0.513060317067604,0.406393013814954,-1.26247
327002826,0.206778538734165,0.339746182543081,-0.553877580732429
"Lca5l",112.906261219798,-0.425364723137933,0.266861349321099,-1.59395
402976141,0.110946331585173,0.213243642337697,-0.657324574078416
"Gpr20",17.4545036803021,-0.672063158792203,0.466253524470685,-1.44141
142858955,0.149468483449557,0.267272238655491,-0.886778950415435
"Tmem86b",518.029320906776,0.919724496717946,0.26299778472562,3.497080
77456802,0.000470379383820953,0.00328843930646861,5.25837967901342
"Dcaf12l1",207.508529853043,0.481729535471175,0.308372868532143,1.5621
6575655378,0.118248945321972,0.223955117619019,0.720816535743265
"Rtn4rl1",840.402465937945,-0.39217106846694,0.16139460239862,-2.42988
961612445,0.0151034219151463,0.0463831344308587,-1.20428651258581
"Ush1g",3.63173874677777,0.0900651293650688,0.836838575276184,0.107625
451342685,0.914292807002888,0.947381684165506,0.00486831037941238
"E330021D16Rik",9.85371161144721,0.303537520207811,0.703253679745143,0
.431618815443401,0.666018481071225,0.773745716947575,0.077861013483564
2
"Insig1",6358.18545188729,-2.79240380746735,0.676320101089739,-4.12881
97747901,3.6463006513321e-05,0.000454601090549151,-21.49059171009
"Preb",6298.47660855297,0.323498756701407,0.105950964390497,3.05328751
429865,0.0022634895770147,0.0110007910209211,1.45891084261747
"Olfr734",9.55382763385655,0.996780588069544,0.433590156399314,2.29890
040942618,0.0215105951600672,0.06087279890145,2.78995782218178
"Lhfpl2",1004.25510325856,-1.28847158908001,0.229086426495488,-5.62439
079779085,1.86163774684532e-08,1.29175134476833e-06,-17.4710454954262
"Sowahb",447.082634414625,0.518548755165115,0.405591326507912,1.278500
6021449,0.201072972588602,0.332839869219166,0.570452259081458
"Fahd1",4994.35743630215,1.50185704827868,0.360947240774209,4.16087693
33083,3.17027899061779e-05,0.000406939432251913,11.7247669883477
"Adra2c",87.3650115780272,0.661775774895242,0.343580820936975,1.926113
8415425,0.0540901606820186,0.123394167892594,1.38468072474687
"Proser2",530.076143175154,-0.551565690943267,0.324003216076058,-1.702
34634588871,0.0886904616320888,0.180040252046066,-0.945700651121302
"Tlr9",75.6562257827319,-2.28964880007395,0.539443724599896,-4.2444627
5980349,2.19117751580403e-05,0.000308120099831026,-18.5118584399891
"Fndc7",73.0661987895632,-1.38390497669098,0.468695148351783,-2.952676
12979916,0.00315032294100866,0.0140214225782318,-5.9053563251766
"6330549D23Rik",30.4099404379899,-0.0516942860027161,0.287328381969628
,-0.17991360842376,0.857220391552557,0.909313051220921,-0.004914361410
17486
"Cenpe",333.340673423081,0.279636375721569,0.435875062328343,0.6415516
73609902,0.521164319446622,0.65461853144528,0.118482661613479
"Zfp423",229.594589164679,-1.96410853738477,0.353753592126924,-5.55219
390303764,2.82106416606236e-08,1.75735172875028e-06,-26.0277821755717
"Defb11",10.4160301235051,3.17288057765087,0.702857279022533,4.5142601
0990938,6.35381963373947e-06,0.000119351421212574,28.6620209229452

"Olfr167",0.567270198094594,1.05925151824975,2.60056515276845,0.407315
893286549,0.683775991226538,NA,NA
"Nyap1",258.050547393138,-0.523387435167885,0.40834404712799,-1.281731
51745209,0.199936844763793,0.331409385293245,-0.578029530505304
"Sh2d5",48.8243353937565,-1.33920408305732,0.364114651282635,-3.677973
62270323,0.000235094264686137,0.0019203468675354,-8.37705560274381
"Fam186a",5.55864276524228,-3.38755415509286,1.42571876839385,-2.37603
251790612,0.0174999205142479,0.0519034758422279,-10.0216369568659
"Tnfrsf26",125.993861075205,-0.270559280058838,0.297619116687974,-0.90
9078970026292,0.363308442506615,0.508324253891018,-0.183070078667346
"Wdr81",2490.20969010961,0.697436998936602,0.194538341591816,3.5850876
142451,0.000336965063852094,0.00253282375288671,4.16957164546209
"Tmem88",383.072489475881,-0.618191388887848,0.252436916283799,-2.4488
9455151184,0.0143295392539396,0.0447608709345266,-1.92036267404089
"Pxt1",1.96223031508075,-1.09626892120782,1.44117742937133,-0.76067588
8246483,0.446850679960254,0.587940789382889,-0.582260253654111
"Olfr433",10.3153421729328,-1.0081501249728,0.653510979412294,-1.54266
746348995,0.122911480097166,0.230612782807405,-1.47897159740928
"Cxcr4",805.976459560198,-0.741344831622467,0.340711152584193,-2.17587
486056616,0.0295646190616318,0.0778146520604343,-1.89296882382699
"Olfr1033",3061.61024752477,-0.232943344541572,0.192623475518744,-1.20
931960091699,0.226540083316524,0.362739471504112,-0.236220953313463
"Epcam",5353.86371840987,0.20423212916998,0.35328699604377,0.578091272
696256,0.563202498895906,0.691170841104756,0.0754368637947914
"Kcnk13",69.7668074389749,-1.6431036052238,0.42443815963852,-3.8712438
2648153,0.000108281413693025,0.00105510794811924,-11.2620164611645
"Trim39",2692.65622458855,0.246476882672982,0.193280707270069,1.275227
54937244,0.202228712914825,0.33427055310387,0.27009049558518 1
"Akr1e1",2848.41597799096,0.393321937545409,0.216354894259122,1.817947
95487426,0.0690720878145001,0.148666251043948,0.749691834154701
"2410002F23Rik",3424.91775251417,0.14235550365469,0.190354605086198,0.
747843760282172,0.454554403370399,0.594785702765992,0.0739613858275532
"Dipk2a",803.62614053011,-0.705879190455678,0.210762228188309,-3.34917
312520059,0.000810531323199691,0.0050075220975119,-3.73891083305593
"Vmn1r230",7.65674717372318,0.167840985744709,0.911517204256868,0.1841
33645487849,0.853908612442436,0.907033674297375,0.0163772020817095
"Hnrnph2",4141.09508462599,-0.486938341355472,0.188550150719133,-2.582
54018624903,0.00980759393689887,0.0332583182652484,-1.65727047794512
"Tmem60",672.587775056946,0.28732165264543,0.168438437710033,1.7057962
3363674,0.08804602462147,0.179030283457026,0.494250794837516
"Cox19",2180.59495433619,0.223548540082003,0.326720776025881,0.6842189
30920679,0.493836929844531,0.629916105843948,0.103317123355653
"Insm2",0.463801677655936,-1.41126809556118,4.76769717342783,-0.296006
236181843,0.767225318919558,NA,NA
"Gprin3",162.517523106802,0.149573739645799,0.260977305387177,0.573129
297292331,0.566557151735871,0.693868734701206,0.0546650854885065
"Gm9797",86.9496633913723,-8.64366240312139,1.77246206633205,-4.876641
68802703,1.07907187264713e-06,2.95019990124503e-05,-90.1624966112218
"Zfp956",606.864318860833,0.210006715521577,0.159125875002442,1.319752
14916087,0.186917782495861,0.315707113921023,0.24212521606869

"Ttll13",70.9797635132142,0.559940188672035,0.261068267171679,2.14480371260065,0.031968544255855,0.0825014353013835,1.39701694374221
"Hcrt",0.471948197980446,-1.46818818276866,3.04237042382883,-0.482580349608101,0.629393734416373,NA,NA
"Olfr459",0.288859012383133,-0.0627093998507145,4.78471990595721,-0.0131061798983548,0.989543080776425,NA,NA
"Trrap",4310.33853969084,-0.0412514928698314,0.130155237763148,-0.316940705412866,0.751288594563016,0.838213837078833,-0.00728014743073385
"Pcdhb3",33.3346848397119,-1.40675892517587,0.418591272391177,-3.36069817495201,0.000777457339513486,0.00484618388933311,-7.49741131729978
"Hcar2",258.724860257832,-3.36430564110779,0.892283794122757,-3.77044351053735,0.000162957686968875,0.00145072780169893,-21.9880584538362
"Sys1",1649.6050184247,0.139412145498072,0.186561912807956,0.747270133543176,0.454900516903357,0.595158711573619,0.0723447496899315
"A930002H24Rik",10.0330680832447,-1.46066549797157,0.645008511332603,-2.26456778834407,0.0235392154824438,0.0652591530608116,-3.98672429292219
"Gpr150",22.4326832541387,0.866726459144633,0.479833894027288,1.80630520255691,0.0708706796504656,0.151517161384296,1.63556169789674
"Olfr460",14.0278339486143,1.67551077951321,0.538444912832443,3.1117589554321,0.00185976283899121,0.00943876982036659,7.81278884737372
"Pou3f3",4565.61272523602,1.27519110971506,0.515786620961759,2.47232296824079,0.0134238181175748,0.0425036709412916,4.02726371638925
"Onecut3",0.546794048034968,1.37998438118517,2.92079957231966,0.472468016725024,0.636592765475612,NA,NA
"Zfp560",531.351250120984,0.365443723479888,0.260802901718212,1.40122568066645,0.161146597167898,0.28281155279745,0.461546101863693
"Tssk2",0.783781142064145,-1.54960549054495,3.69106624612484,-0.41982597634812,0.674612587030255,NA,NA
"C1ql1",7.80005363650888,1.42498658264758,0.777058774202973,1.83382085107936,0.0666805915208955,0.144572242129926,2.75588978108867
"Kcna5",127.166612947092,-1.18296402265157,0.376221357773185,-3.14432978939157,0.00166467720626463,0.00872289066588225,-5.60938424142083
"Ddx28",512.663208071259,-0.344294265665367,0.25925047724726,-1.32803715279952,0.18416583355423,0.312303480834961,-0.400682735376287
"Sprr3",0.338942840908474,-2.01407229344615,4.74887020508739,-0.424116096348245,0.671481147089063,NA,NA
"Olfr600",85.8344622446342,0.245072053278701,0.340430083741308,0.7198895308704,0.471593014384942,0.609513495590455,0.121333749277821
"Krt14",481.44128717553,-1.11583693323206,0.707966423512883,-1.57611561251076,0.114999161617271,0.219222169057939,-1.69347178409951
"Fpr1",25.6821347093643,-1.85997729758473,0.771000322797747,-2.4124209064341,0.0158469753310249,0.0480984316631885,-5.64411172656313
"Mettl24",76.6166683495185,-0.101348283405239,0.247007122566353,-0.41030510518179,0.681582147574738,0.785782614798063,-0.0244325471827325
"Mc2r",33.109063234866,-2.911365871735,0.86195364249307,-3.37763625351627,0.000731117221768891,0.00462630023945063,-15.6514965641638
"Penk",657.223265102121,-3.46121044018272,0.666391992822092,-5.19395562591456,2.05872230563118e-07,7.91735059471335e-06,-40.6569489740834
"Vmn1r233",193.20521710248,0.348266101278276,0.426103160272029,0.81732

8134942578,0.413740932513393,0.5571600733577,0.203701781388487
"St7l",1088.27002350333,-0.008575568192428,0.196840138331156,-0.043566
1560956679,0.965250229593672,0.97904389433121,-0.000181620257114023
"Frrs1l",12.5664313936254,0.456544892558833,0.614272610858909,0.743228
469718791,0.457343369684282,0.597352694406761,0.235233642893534
"Olig3",0.14505395440001,0.892077966308475,4.78471990596623,0.18644309
0471422,0.852097303472138,NA,NA
"Glb1",4295.69053851122,1.43472154734778,0.285565674162014,5.024138673
382,5.05696891370188e-07,1.5903351593445e-05,15.852210656263
"Zfp553",698.377155035435,-0.101287094106207,0.131232003124391,-0.7718
17024008995,0.44022280735197,0.582048165575962,-0.0548167855975695
"Dbx2",1.98394099588099,-1.10396942316796,2.14856562688838,-0.51381694
3430656,0.607379980766495,0.727795898782271,-0.350769314997036
"Chrm2",1.53722208795043,-1.74972146477712,1.646139664919,-1.062924065
35458,0.28781638854321,0.431846398138938,-1.46921541726295
"Odf3l1",46.3009404449833,-3.62941341351567,1.13938452662684,-3.185415
74744795,0.00144546186170623,0.00781792382234344,-17.6075048793288
"Esf1",2667.26970772263,0.556435535704188,0.24176586313037,2.301547160
13044,0.0213607229216713,0.0605365371405156,1.56052800960732
"Pigz",61.159988895332,-0.248304912559216,0.503150160203673,-0.4935006
13134463,0.621658906713599,0.739678120650623,-0.0748739029390997
"Sh3tc2",1481.7472696374,0.356369183517402,0.153554986497261,2.3207919
9540523,0.0202980727380498,0.0581162734576352,1.01398064489688
"Tmprss12",2.92405271547473,0.116581086699585,0.955107150396311,0.1220
6074119664,0.902850913320033,0.940574220341633,0.00714230734494147
"Mtus1",4730.87408519699,-0.599790416248356,0.351739990605927,-1.70520
96215023,0.0881553362083151,0.179177811465624,-1.03126561023277
"Zfp629",1880.18983479756,-0.105675336499469,0.141143771016216,-0.7487
0705053876,0.454033793182733,0.594303900340825,-0.0549896908622353
"Vwc2l",0.302548309014047,-1.06212995308167,4.77767916045835,-0.222310
857931233,0.824071900175153,NA,NA
"Pcdhb10",36.0113028283569,-1.20073534946539,0.297764541418435,-4.0324
9944988599,5.51867442356613e-05,0.000622703205501692,-8.8631565963843
"Pid1",1103.98459623819,-1.27468417125347,0.193848356583848,-6.5756769
5551811,4.8432304975191e-11,1.34904726058039e-08,-23.0989114984572
"Plekha7",2226.95048696428,1.26480698690477,0.274387006598564,4.609573
18126664,4.03496486158434e-06,8.43740065777616e-05,11.8642072348438
"Henmt1",26.0706273146978,0.75430086962522,0.663209930523823,1.1373485
7532886,0.255392595771964,0.396121615811132,0.698508254702493
"Cdc42ep2",759.731553661883,-0.597910914482926,0.160015717920704,-3.73
657614547105,0.000186542976698078,0.00160300350800314,-3.8480806343805
1
"Mfsd5",1365.83200902805,-0.263819974732632,0.1578730909774,-1.6710889
3035102,0.0947041135302297,0.189427657531024,-0.438929976901978
"Smtnl2",1965.2946821297,0.581499815215379,0.393547945126007,1.4775831
5706411,0.139519385288304,0.25366924415809,0.797657280962766
"Spred2",1565.81199215442,-0.267616840104571,0.164263437404165,-1.6291
9298617931,0.103272171290305,0.202168754600239,-0.427826333562483
"Col27a1",6614.48056000591,1.74663076815863,0.332930159903534,5.246237
73545993,1.55236565310293e-07,6.37221589562125e-06,20.8959277296363

"Olfr1489",31.5326698378411,0.0416998051500856,0.357593909886465,0.116
612179338639,0.907167385860876,0.943127331475567,0.00244168944785281
"Pqlc3",543.950467159226,-1.37401037434307,0.279188108533852,-4.921450
20630942,8.59052602767752e-07,2.46207451603659e-05,-14.5808897809411
"Tcf21",3672.61432182421,-1.30615629952394,0.418278262962769,-3.122697
05404272,0.00179202098984296,0.00918515849937256,-6.1260902639621
"Lcn6",0.410318279429751,-1.66854370216792,4.62375236856174,-0.3608635
51757841,0.718201451692732,NA,NA
"Pcdhb4",38.1426879629863,-0.791376140902357,0.358349974785115,-2.2083
8899563731,0.0272171682426996,0.0731266840838027,-2.06989331712976
"Wdr89",356.928345202083,-0.225227673720593,0.169554095984751,-1.32835
2891816,0.184061554176991,0.312231392216994,-0.262167427627083
"Thtpa",1115.72841507214,0.238738085619315,0.154827200804361,1.5419647
4766074,0.123082160321928,0.230888585910809,0.34994705991875
"Gm21411",0.867953429624288,-2.67925143883264,1.98823027922566,-1.3475
5589773842,0.177801263418848,NA,NA
"Smkr-
ps",25.3711854400019,-0.308679726714979,0.47976478566766,-0.6433980482
4443,0.519965849558524,0.653366896609661,-0.131379167737691
"Prr15",1037.95489664976,0.119792657454816,0.473143972444659,0.2531843
67616196,0.800125745897457,0.872335707532353,0.0163613941746273
"Adrb2",528.649875780077,0.775035216501548,0.19852378741865,3.90399169
07647,9.46190306204525e-05,0.000948037954284472,5.39511006192096
"Pnoc",0.20231518320793,-0.0578160724334402,4.7847199059601,-0.0120834
810751245,0.990359011624255,NA,NA
"Sprn",15.2617626439478,-2.60358891875506,0.783606182408475,-3.3225732
226266,0.000891912591222651,0.0053991032920395,-13.5946978498574
"Bricd5",8.83627102686002,1.2011854751562,0.493280842295624,2.43509451
850215,0.0148879002165001,0.0459746589934408,3.69924877714258
"Mms22l",165.04940489092,-0.19847429450305,0.281288173606744,-0.705590
611784936,0.480442764271972,0.617879626526938,-0.095557755276234
"Tssc4",1562.16058762552,0.0395750024397342,0.146302081927306,0.270501
977267816,0.786774095965949,0.86216609470826,0.00586926340578757
"Zfp764",414.047525380658,-0.0922812270276365,0.148595402800201,-0.621
023432008299,0.534584205976966,0.666636878171889,-0.0374209411635134
"Togaram2",56.5291826776118,-1.57110300810866,0.474649128404539,-3.310
03032364071,0.000932858644436109,0.0055662270292408,-8.15565514698004
"Basp1",1471.96753978567,-1.48357234736694,0.290817081858514,-5.101393
41845371,3.37161874596939e-07,1.18024815635388e-05,-16.8343932476894
"B230219D22Rik",3088.74849024026,-0.197507503322786,0.117908664889028,
-1.67508896406108,0.0939167779185722,0.188103681135729,-0.329988025887
877
"Slc16a5",657.041541913719,1.69100746838731,0.54780310768441,3.0868891
4806503,0.00202263008284455,0.010086250986011,7.77285461118996
"Lrtm1",26.3756946547364,-2.58497860608754,0.971739811363514,-2.660155
09075457,0.00781046774439442,0.0279531020705217,-9.24705552033302
"Ifitm10",285.191727771141,-0.173413966699867,0.357746871018757,-0.484
739296827491,0.627861287437046,0.744196922945138,-0.0512350867131831
"Olfr624",3028.43270921954,0.106116057728161,0.139688532746478,0.75966
1910979856,0.447456701794605,0.588380818154249,0.0562819292256242

"Ccdc149",364.35769063138,0.185392325668561,0.121826141208905,1.521777
86991262,0.128064748324934,0.237583868966657,0.266452262309857
"Whamm",1333.28492082102,0.10925697157628,0.204750665439192,0.53360984
8553718,0.593611492984113,0.716360518054357,0.0364450361391383
"Gm9800",9.18057309356153,-0.621919697928691,0.834314004402912,-0.7454
26415769895,0.456013979674566,0.596135472725088,-0.321711182824677
"Hsf3",9.07174661594695,-2.22670378624153,0.726027049418126,-3.0669708
3535127,0.00216239954305416,0.0106131711998303,-10.1218371821809
"Olfr984",4.91634907153143,-0.495224212266365,0.753758420694498,-0.657
00654038475,0.51117671102669,0.645400732474157,-0.216850691836351
"Zfp36l2",5314.32854843052,-0.586937808517976,0.109538703505107,-5.358
26871906159,8.40232066732043e-08,4.01540314635818e-06,-7.2929210845109
9
"Zswim3",211.06512964834,-0.332279244727088,0.175560414303097,-1.89267
749250935,0.0584007713516166,0.130899901116659,-0.675630818558569
"Ptprcap",311.314359316671,-1.67040337556944,0.469647670960383,-3.5567
1597849859,0.000375519767190145,0.00275155370938499,-9.84801267128863
"Serpinb9",2686.48184267525,-0.999124291153428,0.307275518797069,-3.25
155838989331,0.00114774197939403,0.00653612086910186,-5.02600625298071
"Hdgfl1",0.0949500915947116,2.04836625511614,4.7634299069052,0.4300191
86835683,0.667181684222319,NA,NA
"Ccdc9b",1028.219481726,-0.244540388403868,0.190609324337448,-1.282940
32442475,0.199512983938669,0.330960027295914,-0.27040241116709
"Lyrm2",738.973348631981,0.404106050019915,0.271083845320178,1.4907050
2354216,0.136038956606206,0.248546437022844,0.562566369910488
"Cradd",545.290049897991,0.60687168007776,0.147005638169593,4.12822043
857695,3.65581558633046e-05,0.000455179388903393,4.66976770735036
"Gvin1",2948.63523089195,-0.952354790510316,0.322729323404943,-2.95093
975490834,0.00316808703759991,0.0140702147090533,-4.06055049938923
"Slitrk6",157.851578789104,0.859587874584904,0.315797457642197,2.72195
945148751,0.00648961046703021,0.0242775182053252,3.19612353231895
"Adra1a",296.156753418197,-0.349659637153339,0.503605172431202,-0.6943
13037861234,0.487485916369279,0.624255064024445,-0.16475830549571
"Pcdhb8",25.2336727688522,-1.94497083342942,0.459183651081245,-4.23571
44659867,2.27826134593978e-05,0.000317750641796379,-15.6652689419528
"Gm9803",908.960300999316,0.295341111859323,0.334044786577696,0.884136
270723176,0.376622655592146,0.521918161957048,0.19204392807291
"Paip2b",909.928479474312,-0.650746807733537,0.243518172013038,-2.6722
72062303,0.00753395388190296,0.0272494631535133,-2.34445948850329
"Npas4",80.3281403799808,-0.942739410687955,0.353990007057224,-2.66318
085791489,0.00774057983175856,0.0277794635670216,-3.37826732060677
"C2cd4c",3.77153306398411,-2.97886989909439,1.27281328634309,-2.340382
46697829,0.0192640007502908,0.0558444077652647,-8.59459339511143
"Ccdc42",6.51199173179451,-0.431266783483377,1.03005913574952,-0.41868
1577120876,0.675448861459321,0.781132971573774,-0.106527158824438
"Tmem268",853.845245477631,-0.44059711938988,0.217510937228069,-2.0256
3202110566,0.0428025076064236,0.103227991500003,-1.0005146477918
"Gm20715",1.1303824723758,0.137012958919284,1.93333125274941,0.0708688
481213122,0.943502136613836,NA,NA
"Clec14a",632.0525393887,-0.511724664752682,0.231420823370488,-2.21122

998915896,0.0270199146726845,0.072719379150196,-1.34130575748575
"Ifit2",1224.43329339636,-0.75972647957683,0.273840590342698,-2.774338
45225819,0.00553140985437336,0.0214203434638673,-2.91994352404342
"Mtmr11",2994.25455247949,0.81012697130086,0.279138862331775,2.9022364
1571617,0.00370508810096233,0.015863374570172,3.35695740989331
"BC049762",36.0817227231749,1.71247368157144,0.584658681167637,2.92901
437493652,0.00340038696205344,0.0148789822679798,7.2057564291714
"Mrps12",1277.04573575683,-0.064846392333212,0.354196642843893,-0.1830
80200344508,0.854735091032531,0.907618501682864,-0.00628563479867052
"Cavin2",3056.45354443399,-1.76346956230688,0.607686812434381,-2.90193
817970552,0.00370861718546001,0.0158715140215319,-7.30645884399305
"Wnk1",13771.3251425707,0.762968070716572,0.187751649681581,4.06370901
140168,4.82990327626438e-05,0.000563912898686245,5.70746715426203
"Gpr158",0.371686272981569,2.13924345040761,4.61331655456213,0.4637105
26929288,0.642855154087005,NA,NA
"Teddm2",46.2161634253672,0.565344291854482,0.404861491783004,1.396389
39076353,0.162597268151195,0.284732261098924,0.710188877359448
"Ing1",1256.18602363273,-0.255600669180462,0.0940672850378864,-2.71721
108010629,0.00658346064115799,0.0245673331256074,-0.947342381053174
"Slc25a51",9252.56384727494,0.308823025021816,0.31263635479341,0.98780
2666858388,0.323249301222073,0.468142433011015,0.2343913288219
"C2cd2",1374.99810748451,0.295321329270769,0.127983758433821,2.3074906
7604135,0.0210274814351501,0.0598094577713429,0.831799538061115
"Tmem104",693.179762710825,-0.255808370529721,0.138824200181569,-1.842
67851134852,0.0653759632745037,0.142392820813927,-0.498612900818389
"Eif4g1",14958.9797100965,-0.168012965278403,0.212274875598826,-0.7914
87757580539,0.428659417373394,0.571375819590226,-0.0940382189009761
"4930451I11Rik",0.493952006882521,1.97728924946012,3.62720988209658,0.
545126781667573,0.585666324262972,NA,NA
"Onecut2",9.76181394928311,0.645997613120569,0.609540154638907,1.05981
141390637,0.289230403121976,0.433138960641791,0.540504056374154
"B3gat1",8.06540089728015,-0.815916430965818,0.722988000553942,-1.1285
3384888916,0.259094530017696,0.400271384698917,-0.747063283292911
"Polr2k",1237.88366368977,0.625704255709394,0.243218113610353,2.572605
4956244,0.0100936188730966,0.0340611597071022,2.11462857298709
"Gapt",37.520730735612,-1.53918563569337,0.453935120045312,-3.39076129
54462,0.000696987784224603,0.00444985848618576,-8.33451010818995
"Pnlip",1.67993606150086,2.13666277030313,2.69040123169364,0.794179970
308025,0.427090667257088,0.57005844949197,1.20083947639168
"Zfp830",1283.99820128118,0.718842450053224,0.162753121736167,4.416765
97281196,1.00188601025484e-05,0.000171490830524918,6.23306905324635
"Pofut1",1642.79097906925,-0.686939830393807,0.128553450058033,-5.3436
1256025181,9.11122066558125e-08,4.25001388845702e-06,-8.49647595393204
"Stard5",3285.19548203764,1.08870666366783,0.193805862737939,5.6175115
0500518,1.9372727855233e-08,1.32536648323275e-06,14.7343617016418
"Calhm6",136.368657768994,-1.64516418505534,0.528367338397822,-3.11367
502397859,0.00184772882449687,0.00939426769753323,-7.67905981328603
"Snx12",1504.90856085026,-0.274491664641681,0.164418952737573,-1.66946
486442955,0.0950252880012859,0.189933681747727,-0.455952699720557
"Otulin",1128.75199058567,0.0465781257583533,0.165226625020279,0.28190

4479696517,0.778016754492173,0.856613624659122,0.00720881773291175
"Olfr803",3.13527989957313,0.536311028476637,1.02044411553564,0.525566
290511778,0.599189583262363,0.721152069689338,0.1753228902907
"Rp1l1",0.0878637210809483,-0.0623267594334185,4.78471990596623,-0.013
0262085677578,0.989606883219816,NA,NA
"Sbsn",512.122373418181,-0.228515345994752,0.239953004802937,-0.952333
754613417,0.340927744254872,0.485536333274729,-0.165102601926876
"Eid2",541.966417013729,0.590301248410487,0.177238826329908,3.33054139
791985,0.000866772739476394,0.00527805586330754,3.09565630564596
"Ppp1r15b",4278.49365745716,-0.00569483793919011,0.22881564470049,-0.0
248883241643919,0.980144040317949,0.987144496108929,-7.36846593602257e
-05
"Igfals",383.285805452129,0.169950111053537,0.530617204566617,0.320287
600158658,0.748750322109486,0.836530700234401,0.0303347451490467
"Lrrc8d",2741.85551190632,0.597350632585782,0.190217285749302,3.140359
3539499,0.00168740709683634,0.00882539258962312,2.82554148304728
"Clec9a",913.804393131185,-0.969905120529044,0.520262703559015,-1.8642
6033212474,0.0622851545183693,0.137293014341038,-1.92588031464318
"Tmem174",9126.9469610276,2.23543963318771,0.812575850159875,2.7510534
9580335,0.00594039414143013,0.0226576300801261,8.46618800888113
"4931422A03Rik",0.910217766425725,-0.194940395524212,1.69293484059921,
-0.115149378965592,0.908326721416605,NA,NA
"Hpcal4",89.6573248226649,0.619988402774317,0.318586324407988,1.946060
94259196,0.0516474195140496,0.119202342055509,1.3186737168643
"Mosmo",1822.66616444672,-0.0277028766363114,0.102213099436179,-0.2710
30589906031,0.786367505132262,0.861835909821113,-0.00411915142799492
"Mcmdc2",483.017376616212,0.872525914709862,0.420503686708654,2.074954
25673733,0.0379907744528684,0.0942542461231744,2.0606962825233
"Il17c",1.44339759238912,1.31321905484455,1.24297169090014,1.056515658
77058,0.290732690679366,0.434383601752205,1.0949978466277
"Serinc4",26.4586755130242,0.237176084280856,0.387809076143662,0.61157
9508760635,0.540815998922345,0.671388663376282,0.0944926310965254
"Cep295",1015.25454597069,0.217728649856986,0.20921757524777,1.0406804
9540836,0.298023858443287,0.442395827326016,0.177568657157451
"Vmn1r184",0.600258693882703,1.81616558850919,2.25932648805526,0.80385
2651713241,0.421482077726591,NA,NA
"9930021J03Rik",2199.51378751609,0.235742537556752,0.222535149867522,1
.05934966991548,0.28944056199676,0.433249814049811,0.197184671895286
"Patl1",1258.50979931836,-0.188388075040734,0.155193998851062,-1.21388
762732719,0.224790638221193,0.360946048261288,-0.191972493992338
"1700018M17Rik",1.61816700464389,-1.60699939862666,1.58540192441232,-1
.01362271224841,0.310762815885558,0.4560545517464,-1.26172408071549
"Fut10",145.224841197922,-1.60367078638561,0.338179307254888,-4.742072
48043393,2.11542907550542e-06,4.99353948113858e-05,-15.8840071202526
"Tmem229b",515.324963091068,-1.46137556845941,0.243014737006181,-6.013
5265312006,1.81530204258548e-09,2.45452644100394e-07,-22.2423726084231
"Chrm3",169.066741014958,1.01284562784989,0.480080105539914,2.10974297
031286,0.0348805010894562,0.0881872419919888,2.45948592291689
"Olig1",13.9833560662274,-1.94361089212564,0.723312661738319,-2.687096
45902591,0.00720761351635295,0.0263625517419526,-7.06660141519085

"Gldn",7.49482134740563,-0.0217429186246966,0.850140177806275,-0.02557
56864483251,0.979595779128856,0.986845872266198,-0.000287906889833999
"Kcnrg",8.49314475999954,0.221308182974145,0.657349625010002,0.3366673
90615424,0.736367648229849,0.827704291313188,0.0418492279023833
"Adamts6",447.368003327974,-0.193368529393592,0.238971740136118,-0.809
16902259426,0.418417930577627,0.561403303771165,-0.111634693743112
"Nxph1",2.54113797308501,-1.93744756208137,1.06201078679965,-1.8243200
4096666,0.0681037023363186,0.146987601079648,-3.71487559934611
"E2f8",95.6417775377325,-1.03567930413028,0.481015376539339,-2.1531105
9613409,0.0313099876919932,0.0812199226058974,-2.60017098181145
"4930550L24Rik",0.47127039032991,3.03578668866462,4.39413696897156,0.6
90872112112415,0.48964591308547,NA,NA
"Gsg1l",3.59969211670813,-0.646913862418079,1.02136401843939,-0.633382
271882399,0.526484039094122,0.658966863156836,-0.269816146499693
"Zfp84",552.291092203295,0.0286494612167863,0.142507823990853,0.201037
812623012,0.840669007408656,0.898077364474886,0.00307979021947991
"Cd109",176.792382983528,-1.14111553458817,0.306901748806017,-3.718178
66476032,0.000200664333610638,0.00169742003329293,-7.27877182132449
"Pcdhb20",109.798987911291,-1.3694747537754,0.237763841477297,-5.75981
084956591,8.42082465160204e-09,6.968039239569e-07,-19.414717348036
"Iqub",8.07939944561116,-1.02785261167738,0.777813827234735,-1.3214635
3753002,0.186346852597567,0.315015686834347,-1.18730630840419
"Ttc39d",3.86627945345498,1.53188426648195,0.881357812618596,1.7380957
4788992,0.0821939440261768,0.169750823071962,2.71667980842002
"Scaf8",1635.09165772762,-0.10743519419742,0.113134548837188,-0.949623
216794983,0.342303737251901,0.486850847540854,-0.0773315810466484
"Sprr2g",92.2229453792599,-1.87372387149423,0.518775121504134,-3.61182
291483364,0.000304052172362883,0.00232943389262534,-11.3587577033388
"Pnma2",6.25037633685489,-1.24965712627952,0.851371938671863,-1.467815
73307312,0.142154282656896,0.257212713738321,-1.69684924891886
"Pik3r6",407.128219075704,-0.436549927856862,0.363412454470575,-1.2012
5197275595,0.229653474335607,0.366310818275168,-0.438415338750986
"Olfr735",247.706746310496,0.420849716394945,0.204331571564082,2.05964
11664311,0.039432857684013,0.0968827947483476,0.982369853030065
"Cym",28.7783657440268,2.50056035822095,0.423273255818587,5.9076738816
9849,3.46972022033974e-09,3.6767720030535e-07,37.0484534432846
"Rprml",21.6436754764982,-4.04875333325862,1.44873659146577,-2.7946787
2704331,0.00519513151802647,0.0204732879593447,-15.744120957985
"Plaur",499.443841983873,-0.826417435548488,0.269792451417566,-3.06315
996317264,0.0021901295229984,0.0107240445603774,-3.74802364384889
"Scand1",4502.81169078846,0.757810943069473,0.240661332772103,3.148868
72078902,0.00163903807523215,0.00861686641511838,3.60265882624204
"Vps13a",1774.97944645128,0.391789542820024,0.176872544928882,2.215095
29914638,0.0267535240359082,0.0721510729156009,1.03001201383421
"Hepacam",4.19909948938682,-0.491016728067042,0.863811087189139,-0.568
430685075855,0.569742559540709,0.696784822550633,-0.177393853437719
"Nme9",5.6953948023812,-1.50824234602043,0.844088335332738,-1.78682998
317455,0.0739649717302642,0.156689376227368,-2.79551199855104
"Pilra",274.700919825025,-1.37443441949983,0.539532017067398,-2.547456
64023889,0.0108511337094957,0.0361111802470981,-4.56470666501236

"Sprr2h",0.0986945508552764,0,4.78471990596623,0,1,NA,NA
"C87977",0.397839477546787,0.268358575704588,3.70913098889925,0.0723507949726597,0.942322742036817,NA,NA
"Usp27x",201.293955699986,-0.91091743903582,0.2649170608529 9,-3.43850047295109,0.00058494551589253,0.00388860131908372,-5.05532374380379
"Olfr1444",2.281948308185,-0.791511467210142,1.31046585044647,-0.603992440505396,0.545848668085255,0.675786229622598,-0.310176311716761
"Trarg1",1822.24790826287,-3.34441112506574,1.03180336001997,-3.24132606526986,0.00118975013426706,0.00671728246680935,-16.7323283220747
"She",615.210354495909,-0.804169566787361,0.180474722434228,-4.4558570637527,8.35586936438904e-06,0.000147977058008045,-7.09153177777603
"Pnma3",0.949517026628792,-0.0722622627387008,1.37217060041295,-0.0526627393976767,0.958000627460508,NA,NA
"Ankle1",187.081644858622,-0.413677348805844,0.398753110597213,-1.03742726467081,0.299536777258678,0.443912191009953,-0.335959166467474
"Zfp62",1455.93879702702,-0.130852722458391,0.129344506120304,-1.01166045921335,0.311700427457272,0.45678578989567,-0.10252843755432
"Myorg",1221.7823716129,0.557886957888285,0.412735693457843,1.35168091040148,0.176477414420955,0.302901111477093,0.666311669738081
"Stxbp6",794.772787991703,0.269861554673148,0.219612356824363,1.22880860883877,0.219143573040189,0.354712052726017,0.279697721673379
"BC107364",2.48728200813097,-0.0340059705543623,1.31137388914077,-0.0259315598975693,0.979311927347432,0.986751276906632,-0.000453546824062213
"Ccbe1",513.443319854826,-0.726703279206846,0.338497070754961,-2.14685248999661,0.031805026978014,0.0821805481072511,-1.81591278481767
"Hs3st2",0.148177164577155,2.59891947282789,4.7275994170606,0.549733436265582,0.582502219767124,NA,NA
"Dppa3",10.0416162167946,-0.892483121413126,0.755390348088746,-1.18148600080905,0.23740971148478,0.37551833816259,-0.874140783597059
"Ermp1",4663.85954221744,-1.20852525272955,0.311231034822979,-3.88304866003137,0.000103154914660992,0.001013767402248,-8.33167193333111
"Slc25a23",3279.22137242165,0.35980413488709,0.288624090532833,1.24661851414707,0.21253740986489,0.346869876751119,0.380962620082721
"Rpl37a",17983.0740027947,-0.128770943468634,0.179041113729808,-0.71922554985311,0.472001959384749,0.609839918102434,-0.0636848014218548
"Fam178b",25.6928696919584,-1.00457578800031,0.379576172017265,-2.64657231422476,0.00813121179282333,0.0288468645444813,-3.56197859171752
"Gpat2",43.0891946090849,0.576326024294385,0.568868931990875,1.01310862992186,0.311008276640554,0.456148591690802,0.452379426642345
"Gm11223",0.806833505912045,-1.47689673765954,2.48070472879477,-0.595353699501788,0.551607067839356,NA,NA
"Smco1",14.8643650108264,0.162317077288823,0.482545702741729,0.336376588510829,0.736586901621923,0.82777933186307,0.0306793344954064
"Zfp322a",1026.99307336001,-0.290191848501579,0.144309923779361,-2.01089322828042,0.0443367357852446,0.106149026810007,-0.650874564256481
"Gjb2",2168.67449187458,2.07810747308053,0.509514111257742,4.07860631759677,4.53064824322021e-05,0.000537339291035934,15.6458235294144
"Defb14",0.733296985354579,3.27734867467483,2.8244509227334,1.16034895430335,0.245906760632623,NA,NA

"Rpl27a",11265.0344296671,-0.00267225188745464,0.148526380480676,-0.01
79917660337943,0.985645422102434,0.990729196996256,-2.48894726212245e-
05
"Mgat4e",0.551672334632118,1.30957552710193,3.12852760408897,0.4185916
48477168,0.675514594181005,NA,NA
"Gm5590",12.0277860180226,1.44536492027951,0.655379216405279,2.2053871
7752946,0.0274269370501122,0.0735445781630849,3.77220522332189
"Asphd1",3.23319229874914,0.313494473814946,1.39030996451503,0.2254853
10338187,0.821601751800111,0.885647443420974,0.0380696174215315
"Jrk",223.727826394491,-0.499539750484162,0.218694590384446,-2.2841888
7548162,0.0223604330777571,0.0627585611271208,-1.38295595648323
"Pcdhb17",229.810628486666,-1.31837420730202,0.224394146110433,-5.8752
6114274478,4.22177290294963e-09,4.2872983365475e-07,-19.3305812286525
"Gm9806",0.570411274519477,2.02106566356441,3.52066993643622,0.5740571
25505559,0.56592914515984,NA,NA
"Rbp1",2511.89196810842,-0.991651826492442,0.348470729137123,-2.845724
88756216,0.00443104587880767,0.0181048894687745,-3.97808393134181
"Yod1",917.505510248543,0.34901103272079,0.194510960555752,1.794300083
2632,0.0727652848309642,0.154668867724206,0.651418198036838
"1700067K01Rik",57.8616581690002,1.41799010979423,0.324667876829407,4.
36750972606784,1.25671201801575e-05,0.000202339974626516,12.0608017060
573
"Kcnk6",334.116422522523,-1.26879103427942,0.249843500717679,-5.078343
16536072,3.80740537723912e-07,1.29107460948536e-05,-14.2833523428804
"Lrrc75a",315.978344077601,-0.516192790477479,0.253892316509279,-2.033
11702210812,0.0420407016422368,0.101751439997559,-1.17961527716273
"Bex3",1596.00569696217,-0.0697501174783448,0.254224537547155,-0.27436
4222082249,0.783804735056582,0.860314384380389,-0.0104944208584069
"Hnrnpa1",11705.2217374952,-0.583085151621418,0.172962087115934,-3.371
17319375653,0.000748487858507888,0.00470168049780502,-3.12524036774916
"Gm5564",1.40411424117109,1.64314756656937,1.26942378835663,1.29440426
565233,0.195525818002107,0.326205390554042,1.84070001675329
"Cmtr2",269.718397882896,-0.17463891338417,0.209869517583686,-0.832130
913506922,0.405335054393845,0.549178159305898,-0.104666754564703
"Ppm1e",190.894507923041,0.786461683916088,0.277121974464545,2.8379621
8410932,0.00454025613810072,0.0184311502551563,3.14090232745418
"Camk2n1",10160.1102060336,1.05514484925043,0.370121217959592,2.850808
86490984,0.00436081743686618,0.0178929331616197,4.24521643785248
"Nexmif",77.2432360199983,1.13031063436379,0.320566081315812,3.5259832
5351284,0.000421913647072074,0.00302072628954978,6.5583539018245
"Olfr71",48.4752294787874,0.492983624772674,0.399306232728455,1.234600
37526618,0.21697926928254,0.352320269193118,0.514287745187812
"Tmem150b",70.2761150181102,-1.01610119136632,0.453595950883547,-2.240
10198809555,0.0250843025068079,0.0686351010774262,-2.72208550349268
"Sh2d7",0.79254241683963,-3.42669691171658,1.99707849573055,-1.7158548
9455839,0.0861886040014287,NA,NA
"Sox18",950.505111187742,-0.97493918692271,0.257460623446962,-3.786750
6645091,0.00015263011746396,0.00137968568860097,-6.42085156962767
"Krtap4-16",0.202190776426006,-0.0499425078147937,4.78471990596107,-0.
0104379167007399,0.991671898642394,NA,NA

"Scn4b",863.996972000175,1.71102446160594,0.542226936715743,3.15555046
374047,0.00160195551562182,0.00847163782034018,8.16335146977797
"Olfr231",0.21750204661561,-0.791393820394023,4.78471990596445,-0.1654
00239919478,0.868628964847772,NA,NA
"Mospd4",6.48295326837692,1.7530703980097,0.731305842105626,2.39717816
688314,0.0165218878753112,0.0496982057687162,5.26234296272227
"Rnf222",4.5603949160362,0.600786449182275,0.815804971870641,0.7364339
14842013,0.461466671352049,0.600766318890448,0.306130278906948
"C1qtnf2",380.923546982556,-1.45578443951504,0.307824142438909,-4.7292
7311022707,2.25325107758106e-06,5.24270757883956e-05,-14.3483386518672
"Tafa4",0.295185043076733,-0.54538121435359,4.78471990594886,-0.113983
937424533,0.909250526528299,NA,NA
"Cox17",4584.49996712969,0.379432053536519,0.294934118490558,1.2864976
6082816,0.198269434664484,0.329345496429384,0.421415427911842
"Golph3l",1229.66877842685,-0.0753202146352145,0.157718290330783,-0.47
7561698628899,0.632962197213481,0.748110649989163,-0.0218582565026272
"Kctd4",2.88360295215999,-0.991946244727511,1.16210537883717,-0.853576
846636815,0.393339491822765,0.537896858715126,-0.615094409004368
"Ar",2819.65661057928,0.833490098533463,0.15660958402031,5.32208870707
028,1.02582524392614e-07,4.66236377763915e-06,10.2319144393918
"Zfp526",142.938996590732,-0.55838360520521,0.267480929261744,-2.08756
417418754,0.0368371665211264,0.0920244808236929,-1.33213612509683
"Fam43a",1898.40304137,-0.734506184216163,0.167126787542985,-4.3949039
828654,1.10821726997656e-05,0.000185476569356248,-6.31130461344948
"Spin2c",54.8886472943273,-0.55645043852736,0.367346796049035,-1.51478
233786768,0.129827534722743,0.239881258636289,-0.794394897069139
"Zfp319",856.519522831239,-0.408000001699152,0.0941079044435392,-4.335
44880328235,1.45463028397407e-05,0.000225995069935669,-3.4251589426009
2
"Arsj",14.3664137176238,-1.42865748424232,0.4749707700159,-3.007885062
47342,0.0026307261318392,0.0122830215801247,-6.28543193301765
"Unc119b",5137.33650700903,0.581496059300963,0.21490834222493,2.705786
3518967,0.00681428705585339,0.0252643029121562,2.13895349129381
"4930430F08Rik",392.934285820284,0.245143291698454,0.176090074500312,1
.39214712921267,0.16387784914517,0.28641199148082,0.306508534939762
"Zfp518b",302.653857173274,-0.24962476482051,0.165484879230376,-1.5084
4455385558,0.131440787300235,0.242004954747873,-0.35416688714102
"Lyrm4",881.385967792108,1.00151100294592,0.211780791307606,4.72899830
415332,2.25630268168345e-06,5.24355061829128e-05,9.87081893131633
"Prr12",2170.33617191642,-0.142731237959778,0.143328675195748,-0.99583
1697773289,0.319331926249615,0.464443823512979,-0.109462679935218
"Gm7862",3.20700839043627,-1.16560913136776,0.891667706262022,-1.30722
366996349,0.191136758876752,0.320970159726028,-1.32460651663908
"Cfap58",2.02312441784446,-1.14510032059119,1.37910246902129,-0.830322
87035483,0.4063562607814,0.550255876985606,-0.684050729077561
"Lrrc8e",287.780074642825,-1.77962813474423,0.537063137407203,-3.31362
927520178,0.000920935235970416,0.00551994934858628,-9.25297466736853
"Ticrr",79.9207362956223,-0.474182752552891,0.459400617607965,-1.03217
700276916,0.301989206258957,0.446722005254968,-0.382105371909313
"Tmem215",45.9247206161506,-1.56132091265572,0.552556463279216,-2.8256

314357267,0.00471875242844866,0.0189899349535381,-6.18883593454856
"Bdh1",4260.92242379343,2.0085343679043,0.581952431305828,3.4513720707
3334,0.000557744096665164,0.00375773821588713,11.2155314634059
"Tcaim",1439.04357698965,0.862282347513054,0.243000593276147,3.5484783
6331474,0.000387463830211664,0.00282315760891892,5.06151054891048
"B3gntl1",384.765117107849,-0.231025917427809,0.18688166177264,-1.2362
150209734,0.216378648685246,0.351719057527942,-0.241404191698235
"Hrk",4.42255347267798,-1.72993095655726,1.00064742947306,-1.728811672
92684,0.0838428063136816,0.172389226774667,-3.0412193350706
"Oacyl",10.672872252264,-1.1619439805084,0.582668693078275,-1.99417609
75174,0.0461328133335118,0.10922860891036,-2.57290680568518
"Vwa5b2",9.13185988129946,-1.23757121631756,1.10795242088134,-1.116989
49611311,0.263998816367733,0.405905292806102,-1.1158380378877
"Olfml2a",273.989058333291,-1.83409961544507,0.395860463874668,-4.6331
9725716726,3.60061157851687e-06,7.71480489647494e-05,-17.3685276848937
"Gjb4",132.698528460277,-1.84691362143052,0.698396019547111,-2.6445076
5717162,0.00818098556690927,0.0289664321535938,-6.54106184528509
"Pkd1l1",4.50838276199851,0.0524297046296614,0.998021787998271,0.05253
36272816443,0.958103501621735,0.974604489440235,0.001348677716399
"Gm7729",4.70200286761839,0.0664105132661375,0.878870984988806,0.07556
34380932298,0.939766425441317,0.962574372938369,0.00253315898006209
"Ttc34",2.58027989674788,2.26326112660752,1.41434351561163,1.600220244
67569,0.109549732560803,0.21120104282345,3.51924609621212
"Olfr1440",15.2479251676731,0.200693239641726,0.538824919179701,0.3724
64658737867,0.709546920393719,0.807368060385607,0.0429434623783807
"Tas2r143",0.549370029759281,-0.979283874410449,3.14098814270727,-0.31
1775731049525,0.75521096917985,NA,NA
"Zfp316",812.413909545138,-0.0369431364422547,0.100904437113968,-0.366
12003890898,0.714275505198425,0.810507117520741,-0.00776157402146751
"Rbm12b1",734.89559148077,-0.258902128878556,0.155315540974106,-1.6669
4283942726,0.0955257714377488,0.190660404493114,-0.429068509844008
"Cxxc5",4414.40570927013,0.571675174119087,0.206106965161482,2.7736819
7465421,0.00554258319918551,0.0214423188924046,2.19659828131859
"Mtfr1l",4393.58301380217,0.234144983548173,0.170837890004331,1.370568
22431041,0.17050959412173,0.2950790714665,0.285776743355889
"Tmem251",686.424237380865,0.130964653399528,0.122426552322078,1.06974
05988775,0.284736086515882,0.428907927602493,0.110863282175135
"Olfr981",15.0208747928554,0.650716682969951,0.712639822101462,0.91310
7382984985,0.361186067737325,0.506132177830661,0.443110355406703
"C87436",799.346174850244,0.183612541125833,0.183687503788908,0.999591
901128117,0.317508044121262,0.462550197569962,0.141565304577812
"Gm5424",5.93513203342436,2.82467374192948,0.919562513385542,3.0717582
5548816,0.00212801991144626,0.0104831056678573,12.8748355210038
"Tifa",715.808440340563,-0.663572084104329,0.282424493689706,-2.349555
71818562,0.01879583076368,0.0548132079315334,-1.92689660554344
"Chtf8",2301.47409565984,-1.42505276973564,0.281105933011779,-5.069451
05877905,3.98964708108788e-07,1.32747677111009e-05,-16.0028374075502
"Tent5b",168.961383113263,0.674396348073769,0.303977761864684,2.218571
33211599,0.0265159016940027,0.0717467459380606,1.7767732361732
"Slitrk4",57.35068006434,-0.0862468283338324,0.552910785031116,-0.1559

86880105764,0.876043362788163,0.922554333656149,-0.00695227114832275
"Csnk2a2",2538.84458289929,-0.0638180940929301,0.135062335827669,-0.47
250844361494,0.636563916305151,0.751087639341473,-0.0182668405166236
"Mapk10",31.2523997726139,1.53201996380484,0.704718240178532,2.1739468
0094661,0.0297091307022138,0.0780580286257599,3.90711476068167
"Hmga1",1297.40676583741,-0.618883562901366,0.190087758161374,-3.25577
811473777,0.00113082086162906,0.00646211757328355,-3.1202860440779
"Foxc2",695.961172331178,-0.544486490605175,0.345286865892952,-1.57691
051814864,0.114816116003565,0.21900651827798,-0.826886470264801
"Bst2",3332.60867868298,-1.13158021429251,0.252623847430894,-4.4793087
6597884,7.4885145329728e-06,0.00013532071952169,-10.0799614057389
"Nxph3",251.552756152119,-0.627651903946894,0.390503928953217,-1.60728
703967044,0.107991416019271,0.208807678455349,-0.983117318501308
"Rpl14-
ps1",6.52569162190612,0.600903753640227,1.87849203972559,0.31988623903
2457,0.749054569881638,0.836582999401546,0.107218981091483
"Cdc42se1",6117.42445092268,0.178906558658487,0.207526006360462,0.8620
92235070222,0.388636763990306,0.532848577757048,0.112624897104886
"Cystm1",6384.08822015949,1.23426102210249,0.401929032655641,3.0708431
6339986,0.00213455241080887,0.0105029150531929,5.62341964975168
"Kctd11",728.792553368934,-1.10596650321853,0.17945358633763,-6.162966
84724774,7.139445925994e-10,1.2039002610187e-07,-17.6208435483433
"Gprc5a",788.340020528474,-0.359017675841839,0.415711982273959,-0.8636
21187626107,0.387796021056248,0.5320700034167,-0.226533049547366
"Fat4",416.137040392474,-0.928130713541192,0.189301760732059,-4.902916
43327545,9.44241607533612e-07,2.65364691272972e-05,-9.779704583342
"Gm9812",10.6059143441342,-1.90415851618142,0.74614338702484,-2.552000
79407529,0.0107106274654254,0.0357901995408507,-6.34100243636955
"Tmem45a2",2.13237627659063,0.41702777847011,1.07187367980892,0.389064
295845431,0.697228584550327,0.797943472534031,0.0941304761212449
"Selenov",2.55313417306364,-0.38704217847533,0.889760273348626,-0.4349
96020915489,0.663565315682264,0.771830610581208,-0.100240119359847
"Ccdc66",808.601051053106,0.258463654555174,0.2046023860237,1.26324848
687363,0.20649989259629,0.339523977295054,0.279195207579242
"Mrps7",4014.6691362473,0.662557301430591,0.232217761190286,2.85317237
593929,0.00432851351718856,0.0178070294681291,2.66888813497217
"Fam83h",1657.65302222745,0.281572317484758,0.155391939451467,1.812013
66350602,0.0699840853118076,0.150049449682167,0.534083661577354
"Rhoj",1500.88739251295,-1.335850369854,0.22320476661896,-5.9848648847
83,2.16569565148413e-09,2.70684191106651e-07,-20.2011474703116
"8030474K03Rik",1.92531998095606,-4.44606479568056,2.41796176701896,-1
.83876554887053,0.0659496775003846,0.143306231126992,-8.63768780326001
"Ttc6",163.545711283106,-2.22270256708502,0.7713109945326,-2.881720321
42746,0.00395510619327761,0.0166666653461264,-9.10051034509174
"Epm2aip1",1949.66064507277,0.0545131561553668,0.13996054084496,0.3894
89464861053,0.696914102446843,0.797817706053813,0.0123131671140968
"Olfr1341",0.0809400108165231,-0.0719579730135645,4.78471990596623,-0.
0150391192019072,0.988000971296028,NA,NA
"Riox1",396.18604057999,-0.176287559167682,0.171379912361208,-1.028636
06789651,0.303650722603824,0.448359090445237,-0.141410974156091

"Zfp787",2007.1059779867,0.857051739324382,0.15730984050682,5.44817626
515378,5.08889137194516e-08,2.76387754791606e-06,10.9692988990847
"Gpr61",0.296453422874358,-0.434729465217333,4.76938232601723,-0.09115
00558145362,0.92737335936005,NA,NA
"Ppp1r3b",1057.42287904768,-3.89538891935672,0.812285305022617,-4.7955
9201092314,1.62195098840223e-06,3.99807842880742e-05,-39.4490383587621
"Cldn12",2259.48237444713,0.638075328637137,0.15640465027402‌5,4.079644
2274524,4.51046783474068e-05,0.00053592383815828,4.80567614201009
"Phgr1",0.178275448021284,0,4.78471990596227,0,1,NA,NA
"Mpeg1",7777.1384443373,-1.27682762992241,0.405998996197003,-3.1449034
1572877,0.0016614167074819,0.00871283027500865,-6.05594058222949
"Cyren",509.264052870648,-0.426152420053152,0.135916800644016,-3.13539
178404664,0.00171624735448852,0.00891881421050566,-2.01126567062234
"Lrrc75b",245.320291411894,0.877738098528119,0.476410428117363,1.84239
900456564,0.0654168073049884,0.142461894927875,1.71043130897488
"Atp10d",628.613420346027,-0.492852643467275,0.20133792813732,-2.44788
772799495,0.0143696424042566,0.044850214118488,-1.53002501219201
"Gltpd2",8.6078367406348,1.80734402136681,0.743970588133196,2.42932187
131466,0.0151270961830885,0.0464485230516316,5.54748091645545
"Gchfr",847.236539612416,2.16089122577171,0.64344863895192,3.358296365
80081,0.000784244805746013,0.0048822494324507‌5,11.5005855432078
"Ddit4l",858.800457335454,0.158389680951077,0.486628738399126,0.325483
615028852,0.744815081434813,0.83409364527115,0.0287334084166388
"Slc39a3",1324.95731174736,-0.0859029807541649,0.147430249322642,-0.58
2668625664274,0.560116397833208,0.688466842649681,-0.0320665622565912
"Fam187b",254.242904162825,1.21897048589733,0.285946944594316,4.262925
37458908,2.0176791579021e-05,0.000290783946044037,9.92599136311314
"Mettl21e",1.23859900251847,-0.195233022563767,1.33936262406215,-0.145
765619449381,0.88410641765991,NA,NA
"Krt1",7.91727302838028,-0.888022157463168,1.02163071933238,-0.8692202
97176924,0.384726647625439,0.529311330468587,-0.564940598624306
"Brox",2523.65017316375,-0.15573586355863,0.09771596599656,-1.59376067
125113,0.110989649403317,0.213272512473231,-0.24064064709437
"Ckap4",1960.30465260282,-0.878048058282301,0.216895026921985,-4.04826
27506168,5.1599201964436e-05,0.000592160823956782,-6.52541811383141
"Vat1l",90.3191185626943,-4.11101268364945,1.56158099951238,-2.6325965
0631838,0.00847349462488196,0.0297365367523305,-14.4517668163818
"Il1f10",1.5059928401618,1.02715931757595,1.41538004153011,0.725712732
578546,0.468014894585521,0.606335841503554,0.513909636308517
"Spesp1",0.110547637579088,0,4.78471990596623,0,1,NA,NA
"Pip5kl1",3.17107485028635,0.503071546195125,0.950547912935737,0.52924
375441676,0.596636367698793,0.719227062271019,0.165801398686595
"Gpr1",7.90152712113004,-2.34989778930099,1.1078538122538,-2.121126238
2357,0.0339111804474211,0.0864201014722019,-5.75380692314326
"Hectd3",2599.19147068612,0.313565899949129,0.113284359462536,2.767954
03563922,0.00564094107574582,0.0217494698625454,1.20038228429628
"Pramef8",667.948519823189,-0.341539030178754,0.115936316604258,-2.945
91927863791,0.00321996419167218,0.0142571104817115,-1.45171148231431
"Fbl",2346.54004822326,-0.296135641196027,0.123742287509543,-2.3931644
3195046,0.0167037532186858,0.0500830048066946,-0.886651891659377

"Mbtps2",988.61072198704,0.166885602780152,0.224970850422473,0.741809894334123,0.458202524933939,0.597994165405752,0.0858082849732177
"Atxn1",1821.08826654222,0.126753297591065,0.148953320404695,0.850959866129103,0.394791647416399,0.539050948272055,0.0783265904165595
"Irgm1",3138.32680236953,-0.318817307960545,0.345927018325732,-0.921631705738406,0.356720714584374,0.50179211405859,-0.219846647160632
"Olfr374",313.265790835169,-0.192378073683451,0.256353224305,-0.750441404452811,0.452988901955326,0.593533876105432,-0.100356135993961
"Zfp474",0.21705269860567,-0.0600504268427031,4.78471990596324,-0.012550458129819,0.989986446107058,NA,NA
"Zfp740",5791.37774001111,-0.160606303294815,0.0968021547376126,-1.65911909430268,0.0970917963541989,0.192858174950506,-0.264325886890766
"Ltb4r1",101.723592248333,-1.16046930409334,0.464408904754943,-2.49880932990657,0.0124611336741994,0.0400605415300147,-3.73365150162829
"Tefm",521.92454350173,0.572543011475307,0.235144099922052,2.43486020557224,0.0148975439546763,0.0459765023287349,1.76321767549652
"Myct1",512.439394029896,-0.554676316125649,0.164375828434075,-3.37443966920057,0.000739661270906328,0.00466577659661326,-2.97722567345085
"Gpr6",0.147840099652982,1.35969895153643,4.78471990596623,0.284175245000439,0.7762760853899,NA,NA
"Vmn1r179",2.50367322532395,1.91838753704913,1.48122136812404,1.29513898349898,0.195272288948574,0.325810293506699,2.15135649797842
"Vmn1r193",0.449553789386647,1.93223213651096,2.62373315890372,0.736443845272094,0.461460629918515,NA,NA
"Csl",0.174916656736116,-0.543825578326908,4.78471990596623,-0.113658811594968,0.909508264451127,NA,NA
"Adck2",964.424840955589,0.165606807556961,0.183451012956412,0.902730406816067,0.366668994402218,0.511542075905442,0.111010455668802
"Nqo2",1457.04201005291,0.746466971707228,0.20332787079679,3.67124766900875,0.000241369302571416,0.00195522171231303,4.65590237643747
"4930432E11Rik",7.10784035065527,-1.18795594730695,0.758956335135242,-1.56524939882775,0.117524442787886,0.222951117481826,-1.78288753557149
"Slc26a1",3739.42105547253,1.93513767445949,0.773936318097631,2.50038359643871,0.0124058895105874,0.0399158471414907,6.23304335082044
"Gpr156",17.8458952088722,-2.07742298880598,0.690828558076554,-3.00714694625547,0.00263712252242831,0.0122952211722399,-9.13763769982569
"Zbtb21",1001.41952903839,0.233792161834933,0.208477592993441,1.12142584955059,0.262106638099717,0.403741130484142,0.212045135827265
"Pla2g4f",15.8706931944248,-2.03024516075592,0.521937472732884,-3.88982448438792,0.0001003167507627,0.000993385478096052,-14.0379104382938
"BC053393",0.775333285420099,-2.69023797762836,1.86938366376863,-1.43910425118668,0.15012098774082,NA,NA
"Tshz1",3819.6579498197,0.523669850743552,0.147201450832433,3.55750468342646,0.000374394428256593,0.00274743792327702,3.08812638938588
"Tapt1",2155.9966527397,-0.0389243636147125,0.208275324705691,-0.186888982983057,0.851747677318068,0.905725142470969,-0.00385426688617051
"Wdr27",102.192437020089,0.813526709396753,0.286665067790926,2.83789969829908,0.00454114503158123,0.0184311502551563,3.24899227419073
"Gm5637",0.162627472229157,-0.543527872648682,4.78471990596067,-0.113596591510314,0.90955758944225,NA,NA

"Mars2",538.506740806028,0.171389216374499,0.129474987061147,1.3237245
2984728,0.185594548676764,0.314070691728827,0.198492224680122
"Spsb4",1244.51422717584,1.53493682155269,0.585669129850272,2.62082589
52373,0.00877170463059194,0.0305630266060189,5.35380480783162
"1110032F04Rik",79.8773522713953,0.861196909210788,0.474241400817558,1
.81594628331931,0.0693786125645541,0.14912845196601,1.63881229068921
"Msgn1",0.170959733933281,1.33484854833491,4.78471990596623,0.27898154
4284429,0.780258988216428,NA,NA
"Zfp41",265.770655635407,-0.541316300975263,0.155661137028637,-3.47753
017425072,0.000506056087902426,0.00349524675944793,-3.06187516393815
"Fbxo41",17.330622525278,-2.2715701995078,0.775114044732659,-2.9306270
6700313,0.00338278615258831,0.0148219245849813,-9.56705364128029
"Catsperb",0.520113269193673,-0.568692177230875,2.3458072905797,-0.242
42919676933,0.808447613248521,NA,NA
"Cfap65",261.786657314869,0.798036029236969,0.561328724739792,1.421691
05920404,0.155115956789861,0.275088091869588,1.02999629267064
"Mipol1",321.962008937899,-0.20818552371277,0.24486199991808,-0.850215
728787721,0.39520515855721,0.539539993106601,-0.128458455602271
"Ccer1",0.118313186368239,0.895636117808525,4.78471990596623,0.1871867
39330703,0.851514221559173,NA,NA
"Acsm4",0.0804673502708677,-0.544048378937456,4.78471990596623,-0.1137
05376621746,0.90947135024012,NA,NA
"Spata2",1013.13535030364,-0.204762727654984,0.180061133006334,-1.1371
8448971318,0.25546116949417,0.396196459019831,-0.189578564399395
"Vmn1r68",5.21574441695606,0.572505193573924,0.808701745888896,0.70793
1195257464,0.478987986301653,0.61645595755179,0.276959920584632
"Pcdhb15",36.8616542075741,-0.627771935467251,0.378682647248845,-1.657
77845916114,0.0973621906835988,0.193257456372677,-1.03188882416651
"Ankrd33",1.21422238760543,2.61137053573311,2.11486443129793,1.2347697
077351,0.2169162242 5927,NA,NA
"Zfp445",4950.20491437945,-0.0369853069946807,0.158970401391155,-0.232
65530357237,0.816029079386172,0.882553161880229,-0.0046208056312173
"Nipa1",301.667675429428,-0.545898004078462,0.247524114183846,-2.20543
362362263,0.0274236807764812,0.0735445781630849,-1.42471930340439
"Prr15l",3472.86397608215,1.33593342348938,0.496498843089415,2.6907080
2899895,0.00713005658639769,0.0261319254363876,4.86893963592607
"D030056L22Rik",869.853889508879,-0.070432135088293,0.144369295655081,
-0.48786090400112,0.625648368494652,0.742564703306792,-0.0209637918369
459
"Tmem164",1946.82832539411,-0.338701504717835,0.173378356653392,-1.953
53971081262,0.0507556765797879,0.117785548911296,-0.724445156451018
"Olfr432",38.5123744283814,0.898600529454963,0.454396526199366,1.97756
910021073,0.0479773380076961,0.112809904921822,1.96079230447428
"Olfr914",332.193963010282,0.924150581747803,0.278269768381351,3.32105
9945259,0.000896762742598885,0.00542004958313486,4.82189457432665
"Dusp28",571.756844179692,0.348040105042701,0.20677468554316,1.6831852
7061697,0.0923392313044247,0.185628401040984,0.58610247695607
"Ngrn",1450.77980313098,0.051528628623057,0.201650553138986,0.25553427
8587083,0.798310471676483,0.871233492485619,0.00710297741706975
"Lrrc4b",7.05226751972332,-1.41428634779253,0.991622554144863,-1.42623

455051621,0.153800649624098,0.273264540401919,-1.8347748091724
"Tmem198b",664.771839462936,-0.443184999701377,0.153910305636145,-2.87
95017842996,0.00398304044039493,0.016730142720125,-1.81286740159719
"Ofcc1",1.97862295330844,-4.8854512097242,2.24117886889216,-2.17985778
713822,0.0292680048312582,0.0771589451324868,-12.5159776670593
"Rnf31",1749.10465625374,-0.07608685975241,0.219666475971462,-0.346374
472553994,0.729061307080647,0.822165261160119,-0.0148988614365826
"Pbp2",10.2989901167228,-3.25466849391451,0.894454564032917,-3.6387186
3903278,0.000273997952092935,0.00215159567857755,-19.9886947600792
"Dnajb7",4.2816040706051,-0.511955349841028,0.817952958906446,-0.62589
8279682834,0.531381662981181,0.6637136065851,-0.209852821118824
"Cldn14",18.7260006812265,-0.732672785416782,0.71482828875827,-1.02496
333306774,0.305380489115297,0.449993105862309,-0.585056082821853
"Fam221a",130.857015957849,0.191114101498507,0.289716750458217,0.65965
8446383375,0.509473038430373,0.643791659320506,0.0841628501088751
"Ankdd1b",9.23239243163455,0.509797388082875,0.666471541767769,0.76491
9964520426,0.44431920483051,0.585558354564737,0.272838175941034
"Ticam1",839.056337672289,0.225931749786697,0.153695077293882,1.469999
9099821,0.141561778363473,0.256426194215068,0.307473771166122
"Cltc",11451.904576197,-0.545152196370923,0.179610468926135,-3.0351916
546419,0.00240382880708338,0.0114877093334588,-2.43491005664531
"1700113H08Rik",0.264523067411562,-0.271826437798885,4.02718231441617,
-0.0674979220150585,0.946185316246478,NA,NA
"Cd24a",8022.23131280784,-0.178328510525509,0.42287048942116,-0.421709
518603702,0.67323705586359,0.77924218875599,-0.0444810839223154
"Zfp654",885.571265168205,0.0345614320466088,0.122689956427496,0.28169
7321060123,0.778175608824139,0.856649976273785,0.00534754774207401
"Dmrta2",43.5137047907369,0.540391597076095,0.267558787152947,2.019711
64104278,0.0434133069016052,0.10448775820607,1.22057458941124
"Tet1",234.113421021939,0.512551692834336,0.361454047701052,1.41802725
988077,0.156182806249223,0.276589678133274,0.658741777587028
"1700001C19Rik",3.55358899381879,0.875594071447811,0.816091383252449,1
.07291179568423,0.283310693099963,0.427288976954136,0.744513027755212
"Khnyn",2627.71174359783,-0.296905376434488,0.117024744080405,-2.53711
622074123,0.0111769841670615,0.0369684202356855,-0.979102258361179
"Cyp4x1",62.2634193784058,-0.0711973875910652,0.31135957642229,-0.2286
66124257896,0.819128426129254,0.884056573268471,-0.00877395463134947
"Chst9",50.6397971283958,1.58030178091852,0.458529518805544,3.44645593
381896,0.000567991189528745,0.00380966364411127,8.80261969869009
"Helt",0.125633388153494,-0.0309088942759046,4.78471990596537,-0.00645
991717035907,0.994845767673871,NA,NA
"Neurl3",1956.20353435635,-0.978480323386359,0.290716823837956,-3.3657
5059698561,0.000763356934838086,0.00477355148026716,-5.22964924990751
"Samd14",1085.1393891543,-0.493229574191529,0.29875980258547,-1.650923
48409363,0.0987541998615876,0.195443502730535,-0.805189353625295
"Irs3",328.923288805119,-2.12169928894907,0.569319225604314,-3.7267304
414267,0.000193979721323463,0.001653789508686,-13.5888176312735
"Rab2a",7704.918247513,0.221573423976249,0.176538513541765,1.255099635
3769,0.209442592827847,0.343024920198014,0.237072968194437
"Gm9818",1.82695297901727,0.82641781909736,1.18270406514041,0.69875283

5519552,0.484706508352255,0.622172975632144,0.392165939256037
"Dync2h1",954.539559612253,0.91652043306279,0.314721378533756,2.912164
52257782,0.00358933528067768,0.0154902300360012,3.81964085764154
"Gjd3",10.8633736528319,-1.47246915342753,0.825889679123889,-1.7828884
3007402,0.0746044668445327,0.157598905150022,-2.72068427128852
"Dusp18",533.18330797733,0.325661923399887,0.184059997725971,1.7693248
2572739,0.0768396827835874,0.161064301753858,0.594642911379754
"Ythdf3",5205.98566802371,0.10671365572724,0.085731929948099,1.2447364
2191239,0.213228653178171,0.347669247015251,0.112743371131722
"Rpl9",17032.6789908408,-0.569411614713522,0.288743791585273,-1.972030
6767024,0.0486061046738264,0.113951028577994,-1.23675433825504
"Cdh19",71.838218509826,-1.71292621542875,0.52105614466093,-3.28741198
617553,0.0010111279010396,0.00592219039185042,-8.78568232084691
"Ccdc36",28.3306180438989,0.17098717822755,0.494607156928206,0.3457030
0051758,0.729565929062841,0.822544031737439,0.0334029033258512
"Fam185a",457.481808683239,0.082173876205951,0.131286859545379,0.62591
0898398389,0.531373385725752,0.6637136065851,0.0336834447563494
"Rnase2a",1.04937036671819,-1.82891877170965,1.97459943185094,-0.92622
2676968598,0.354330269945425,NA,NA
"Olfr684",0.143520032595355,1.5495926402256,4.76370967376985,0.3252911
58854209,0.744960721552854,NA,NA
"Gm527",105.245316787912,0.24441483823519,0.318370840775363,0.76770484
8974046,0.442662554740607,0.58408570699955,0.1314237034505
"A2ml1",2.09001002163098,-3.29781593509428,1.91587968805991,-1.7213063
8246589,0.0851952419296169,0.174580263601742,-5.75591124034765
"Cldn2",9651.65440364325,1.26050140552365,0.659204948906724,1.91215403
891334,0.055856439289811,0.12652054522204,2.60589830068044
"Fbxw21",0.338652241692995,1.46480691389144,3.74278118346916,0.3913685
68475521,0.695524821961819,NA,NA
"Mageh1",612.491307301693,-0.739427030707225,0.217967419902396,-3.3923
7410360839,0.000692897656516004,0.00442954705139313,-4.00729369697044
"Taf9b",667.697960009484,0.236075233131684,0.194459759100332,1.2140055
8256313,0.224745592380025,0.360933165326228,0.240575411453401
"H2bc6",199.983302337519,-0.799294103494278,0.396759904477262,-2.01455
362418076,0.0439514408350207,0.105411078454253,-1.79832184402686
"C2cd3",1027.01580936647,0.238528983171132,0.124237775714402,1.9199392
6001592,0.0548655721121306,0.124696226255079,0.496587452738858
"Ptgs1",1942.37648649159,-0.893954350427429,0.318708882901929,-2.80492
448873009,0.00503283687886886,0.0199817254050468,-3.49798719076735
"Prss45",0.395736399883915,-0.05919416732367,4.75501786620883,-0.01244
87791611318,0.9900675678488,NA,NA
"Mc4r",17.549435044436,-0.0783551769927182,0.534147206726641,-0.146692
102862232,0.883375052174399,0.927515278948236,-0.0058959145394155
"Emc6",3483.54814427443,0.308319704048246,0.346080497998481,0.89089014
2124099,0.372988109326723,0.518281225459838,0.202639202965303
"Gap43",15.7521809988202,-0.974807101194286,0.726572695104445,-1.34165
116272936,0.179709128898674,0.307123200566775,-1.15076593118365
"Zfp358",929.567361277733,-0.31289594680925,0.191450285047325,-1.63434
568265023,0.10218628656411,0.200644451889901,-0.502579988431807
"Sfn",2234.60166644712,-1.54102446575208,0.325833538054353,-4.72948387

987922,2.25091325650303e-06,5.24270757883956e-05,-15.1884717992929
"Neurl4",4771.18136297767,0.410411331706111,0.177157445698758,2.316647
37593915,0.0205229483800623,0.0585966389682904,1.16436893531817
"Olfr370",0.733941242170314,2.60528774928346,2.43198246074093,1.071260
91217357,0.284052129935892,NA,NA
"Gpr15",2.78791908213297,-4.03376555156479,1.39366865650712,-2.8943504
8476615,0.00379943806763819,0.016153825434542,-16.6416966585199
"Smgc",0.265666779130041,0,4.78471990595838,0,1,NA,NA
"Kcnv2",2.57298426463078,-1.96175476271466,1.50584092475551,-1.3027636
1232058,0.192655477120907,0.322686753105699,-2.21888829779634
"Pcdhb13",19.2789333911992,-0.712755851615673,0.384680219382506,-1.852
85287806011,0.0639034487226081,0.139897617487064,-1.40187987496793
"Kcne4",221.045045194561,-0.564360469830162,0.278146195213869,-2.02900
661429584,0.0424576166237374,0.102569521611898,-1.28516981729516
"Zfp286",136.080162472726,-0.198051892261946,0.28714480561417,-0.68972
827782253,0.490365079475179,0.626877676344547,-0.0924909963706599
"Mettl21c",1.65934129795106,-2.80026443778894,1.69461829085792,-1.6524
4554062454,0.0984437583142768,0.194928038957222,-4.57878188343584
"Lancl3",69.5578625012697,0.355674098779568,0.283327331466636,1.255346
940722,0.209352841781523,0.342993169402247,0.380587200791806
"Tdg-
ps",31.4288260977003,2.33078239392536,0.751446062085545,3.101729467390
6,0.00192393695478379,0.00969576705721747,10.8056607175275
"Olfr976",36.8359838220218,0.736485678263671,0.467555690110905,1.57518
279392338,0.115214257573615,0.219508265992803,1.11678139193545
"Gm973",72.6786253538128,-1.07003607315873,0.45427467457081,-2.3554825
5946621,0.0184986653841949,0.0541158544620746,-3.12089725503485
"Cstad",59.6298072192935,-0.828297688967195,0.401404276965724,-2.06349
990894074,0.0390651564451629,0.0962705283579104,-1.93870781157231
"Abhd17b",1478.41585259756,0.224270463098959,0.111635608178194,2.00895
096787554,0.0445423339147748,0.106396690956104,0.502496094208577
"Dnah14",1.93633197576868,-0.242937677420452,2.17673569625338,-0.11160
6419575238,0.911135481742701,0.945280083387515,-0.0136710774985698
"Gm7367",1.6185390423641,2.77297339770518,2.34256252557318,1.183735062
53656,0.236517954822901,0.37465489342245,2.72236652488478
"Zfp768",1268.57649138014,0.428145545613831,0.150096119665051,2.852475
777317,0.00433801184312404,0.0178301974799365,1.72408293025189
"B4gat1",1533.39871073034,0.206188220650042,0.20867784521674,0.9880695
30983935,0.323118596674331,0.468111873190333,0.156506749570311
"Atmin",1041.15235458208,-0.188416527332577,0.166485845495006,-1.13172
700521396,0.257749224355765,0.39866685518711,-0.173273332810418
"Defb9",0.440613835344313,0.600114752507314,3.03830979466671,0.1975159
85223338,0.843423773435843,NA,NA
"Odf3b",62.5588978490389,-0.175197276818044,0.388728537077441,-0.45069
3118995639,0.652210742376481,0.763134120245071,-0.0473595876883275
"Tgif1",1749.49182729503,-0.481427451486406,0.188991440581398,-2.54735
055728122,0.0108544332971584,0.0361159966600673,-1.59882990175321
"Ctdspl",4479.41196561374,0.0773026836084057,0.161022750485591,0.48007
3054119908,0.631175447711317,0.74661200761491,0.0225885885418156
"Zbtb44",2479.88312395948,0.248701454688329,0.188290537085696,1.320838

84053684,0.186555105902012,0.315269004996457,0.287083306581767
"Flrt2",558.150846556143,-1.08530350621557,0.316784730019492,-3.425996
91010607,0.000612547350458239,0.00402542913354727,-5.98558315042919
"Gpr68",243.165007792585,-1.42352838200708,0.463566555044262,-3.070817
69924312,0.00213473445254916,0.0105029150531929,-6.48574112931282
"Rexo1",1701.08527144873,-0.0804897855088286,0.132600122343246,-0.6070
1139702175,0.543843348650173,0.674159680334482,-0.031736179221613
"Cmya5",64.6256089726385,-0.356039002227504,0.222665019674981,-1.59898
938210953,0.109822961880679,0.211589568760367,-0.55296662290627
"Fam180a",204.758425083086,-0.482832534106809,0.293747536859512,-1.643
69900516895,0.100238391563967,0.197698579679772,-0.782677202885903
"AI837181",1653.57723740035,-0.0340198733581727,0.0969265084253731,-0.
350986266923725,0.725598651220455,0.819350350466,-0.00677823891821785
"Dlk2",74.1737158474257,-1.01441320950666,0.460490966879706,-2.2028949
1535598,0.0276021560903956,0.0738557485179819,-2.64319708844094
"Xxylt1",602.062557855554,-0.434211923967598,0.196537332595643,-2.2093
101510691,0.027153075614552,0.0729745924648566,-1.13661221442096
"Erfe",14.3823671457471,-1.00303404958621,0.79540341011367,-1.26103815
60256,0.207295100324947,0.340358215204035,-1.0810266089554
"Arl4a",2469.73210283915,-1.25880982627139,0.506182399000157,-2.486870
0072501,0.0128872479087791,0.0410849509034295,-4.01826369258878
"Gphn",1938.94855532623,0.430817026055682,0.191492758239662,2.24978234
172435,0.0244627654895666,0.0672269205800662,1.16306875848934
"Dynlrb1",4884.29338392183,0.162391929779066,0.156214105633279,1.03954
71594626,0.298550337204794,0.442873894919284,0.132263389318763
"Gpr141b",98.9950764063239,-1.43509988068512,0.483005069988837,-2.9711
9009686231,0.00296648098093636,0.0134169441350724,-6.18705553657734
"8030462N17Rik",922.951325143682,-0.209752375262343,0.142497839203839,
-1.47196881324142,0.141029299456844,0.255651662403268,-0.2860895403633
92
"Zfp30",542.683747724131,0.896579574078714,0.237685484262355,3.7721259
1194284,0.0001618625346255,0.0014443055354957,5.86374398529876
"Klhl34",0.0910088248646828,-0.544048378937457,4.78471990596623,-0.113
705376621746,0.90947135024012,NA,NA
"Inhbe",1.44477936503259,0.725715112434474,1.54318053620608,0.47027233
3928376,0.638160460866846,0.752370526252533,0.206485075926366
"Dlgap2",0.48525225490384,1.00592051155983,4.61290903372628,0.21806640
9765565,0.827377368717137,NA,NA
"Rnf152",1644.18976498658,1.03240410016756,0.226756985719638,4.5529097
8970774,5.29089647160185e-06,0.000103680300907832,9.47148006774875
"Adamts12",240.222565281691,-1.88672752739103,0.382354484991426,-4.934
49822468627,8.03570765204929e-07,2.32808659434855e-05,-20.127380475566
6
"Cldn4",3723.69519740036,-1.30111377318965,0.290150920600933,-4.484265
53496679,7.31655058874285e-06,0.000132581880464041,-11.6167472913842
"Mroh7",661.89130320117,1.79024815596265,0.502630422524294,3.561758452
60565,0.000368379227462398,0.0027164945413154,10.5775254804754
"Baiap3",368.208137585728,-0.336783524039514,0.291539432807261,-1.1551
9029723216,0.248012507182443,0.38735564442833,-0.319409545739558
"Olfr1396",52.3068679523164,-0.45796576636292,0.489786308838863,-0.935

031784470702,0.349771919458592,0.494801776507554,-0.322223819316647
"Tspyl1",2666.48501409452,-0.620162360659657,0.274564847622204,-2.2587
0997700692,0.0239014314515968,0.0660134989019576,-1.68553681740399
"BC049715",34.4151006619451,1.14583909974125,0.701041465366422,1.63448
120596167,0.102157849349502,0.200611717858454,1.84065758621248
"Dmbt1",0.258298194424544,2.30192066631965,4.7163227478243,0.488075305
571815,0.625496501824565,NA,NA
"Slfnl1",4.05999580768796,0.448958143482124,0.764719195904851,0.587088
889472555,0.557144009118356,0.686379430794964,0.168954037782079
"Flacc1",137.937695371333,0.349942850837611,0.672452044072495,0.520398
2260479,0.602786046245098,0.724338859218965,0.112855155099907
"Rtp2",0.081018275684709,0,4.78471990596022,0,1,NA,NA
"Mis18bp1",100.185475554709,-0.413584443668,0.468006203418046,-0.88371
5729935669,0.376849686809875,0.522084353660575,-0.268799327431911
"Olfr67",0.10500178835157,-0.544048378937456,4.78471990596623,-0.11370
5376621746,0.90947135024012,NA,NA
"Fbxo28",1578.45758102882,-0.233687386485043,0.151027717203276,-1.5473
1456458758,0.121787411382114,0.229209550881125,-0.344249241341549
"Cltb",4809.74464770199,0.409496439358386,0.189271975497864,2.16353444
973161,0.0305000970510569,0.0796547865270732,1.03604775628373
"Tmem41b",2267.04099088877,0.150110960396843,0.200185556142655,0.74985
9097176179,0.453339571466709,0.593866036161221,0.0782230486411646
"Lxn",536.894800266394,-0.459621488796194,0.222457995365647,-2.0661046
05684,0.0388186034593314,0.0958082443354487,-1.07799924571348
"Mmp10",4.6716504157677,-2.64818753874971,1.26247443255914,-2.09761676
787514,0.0359390126579706,0.0903014006192152,-6.36783786755193
"Tyw3",1079.53361395479,1.48098826864737,0.303491449415066,4.879835235
89132,1.06174504330917e-06,2.92399786079693e-05,15.4614785075754
"Nccrp1",981.848503187449,2.95540011129432,0.625248807673219,4.7267584
9201929,2.28132325924579e-06,5.28364937233805e-05,29.1056918507586
"Mafa",23.1798008488895,-0.988351323435598,0.425813083522507,-2.321091
95720229,0.0202818813749428,0.0580897180761463,-2.81261718551103
"Nxpe5",74.8799401594777,-1.29254527366958,0.455134384754535,-2.839920
06968816,0.00451248390263087,0.0183491994021891,-5.16781487695121
"Olfr1122",967.231787576367,0.432134058668544,0.425104157426333,1.0165
3689129923,0.309373789156402,0.454434996306428,0.340824304070478
"Zfp235",536.8289113251,0.039472521932307,0.116909305965795,0.33763370
337564,0.735639240530986,0.82723882068592,0.00748643137988702
"Frat2",104.772197994434,-0.214393359137932,0.23046099309832,-0.930280
462023638,0.352225891104581,0.497275805313653,-0.149777444650214
"Ankrd34c",0.67077704773887,-2.92898224965028,2.92361300838351,-1.0018
365088852,0.316422561201745,NA,NA
"A430005L14Rik",1143.0490392582,0.56128449008808,0.162300796720647,3.4
5829781140364,0.000543600124332849,0.00369178517040818,3.1441165427374
1
"Paxx",1163.60271927463,0.031966855300995,0.213884868813399,0.14945823
6472464,0.88119206163419,0.925925997938318,0.00246019997964213
"Ddi1",0.33410169352658,1.55180651832756,3.81250482339258,0.4070307029
66213,0.683985438147191,NA,NA
"Apof",8.19413912038541,0.343563820932519,0.72133466122541,0.476289078

288391,0.633868445210737,0.748813507707904,0.0993810968302383
"Fgfbp3",434.963562868706,-0.157160387270908,0.187820261167246,-0.836759497054277,0.402727768304243,0.54651907199305,-0.0949541173016364
"2810006K23Rik",519.883309623319,0.0418202100430421,0.184973643443534,0.226087399612736,0.821133444537335,0.885289460967042,0.00509540002376928
"Cdcp2",1.25038999211103,2.04204235604623,1.44324998834914,1.41489164907738,0.157100266847403,NA,NA
"Nr1h4",3082.8219600225,2.3639624613793,0.60546922823846,3.90434782004854,9.4479820133289e-05,0.000947617043703122,16.4568667482381
"Krt87",1.36714852299574,-0.354990210732252,1.37698163460729,-0.257803155692409,0.796558828905887,0.869808694332829,-0.0495147385685777
"D930020B18Rik",3.35696268141722,-0.0811296447981998,0.941651521674303,-0.0861567606814327,0.93134180282062,0.95715968719524,-0.00355226467583756
"Gm5454",0.0942740624828791,0,4.78471990596623,0,1,NA,NA
"Fbxo30",1004.09642472622,-0.0586593348032378,0.106496217411442,-0.550811439401746,0.581762941499399,0.706082404815144,-0.0204148168929601
"Cd3eap",931.895222510186,0.295869451220794,0.197632088812255,1.49707192287919,0.13437454456382,0.246359649262375,0.414502101279594
"Tssk6",21.8609968927324,-0.0507228221317489,0.369412720343142,-0.137306647385161,0.890788416350353,0.93254188144962,-0.00354254354569347
"Vmn1r74",25.3664636665146,0.0896467413984602,0.499483143420543,0.179479012614008,0.857561595260831,0.909576020475204,0.00849642228031028
"Trpt1",398.557225073206,0.42771422770018,0.221652483574115,1.92966133653613,0.0536488135153609,0.122640018396612,0.897559117096479
"Crry-ps",0.203451760649242,-0.0567762545117845,4.784719905966,-0.0118661605334496,0.990532395897081,NA,NA
"Gal3st3",23.4241066023931,0.0475690374569348,0.365692611820636,0.130079295887625,0.896503690420148,0.936256373895564,0.00313317918978954
"Msl3l2",126.345152368477,-1.38333011000512,0.255299160192075,-5.41846713856939,6.01121849218287e-08,3.13387000429363e-06,-17.5312771034271
"Tctex1d4",6.9849252421287,-1.60826484244942,0.771399913739204,-2.08486520908938,0.037081537500171,0.092504582673789,-3.82846978266175
"Rps8",23251.5340938002,-0.0906150602377059,0.168760008147787,-0.536946289777092,0.591304714979449,0.714505412287388,-0.0304615851484117
"Rpsa-ps10",4.25945209286914,0.0769160752572293,1.12761029126571,0.0682115761562388,0.945617211995904,0.966485527048076,0.0026219886318301
"Gpr82",26.0789088796206,-1.77290934302903,0.583477133189311,-3.03852412062531,0.00237740071914277,0.0113949260624006,-7.93303728448462
"Rtl3",5.07964351805258,-6.04589601256524,1.77767083863643,-3.40102108959765,0.000671346482509642,0.004322956973571,-32.9127431879213
"Yipf6",1824.67992007296,-0.315813585661584,0.25358831489614,-1.24537909323988,0.212992434569127,0.347379881993211,-0.333921179857683
"Ccdc144b",96.4190977482719,-0.11750967215602,0.27671271270681,-0.424663077480385,0.671082303359527,0.777512790475017,-0.0295719182025549
"Olfr1388",92.4335897933214,0.210992115262795,0.331546986166846,0.636386768892588,0.524524362580786,0.657315767182992,0.0885303411947604

"Champ1",957.4460120806,-0.249303200389202,0.184818205596105,-1.348910
40406495,0.177365746028443,0.303970933995128,-0.296876033363176
"Ust",252.57070808737,-1.75033982935855,0.284896915827074,-6.143765453
8211,8.05878146964876e-10,1.32042118567405e-07,-27.7256364953743
"Ppp1r2",4690.73146364859,-0.221062742737273,0.179291596973741,-1.2329
7882593823,0.217583664103678,0.353007678706399,-0.230185000622103
"Olfr736",0.191906843942146,2.0416586567276,4.73101917744957,0.4315473
2207791,0.666070451835221,NA,NA
"Ubiad1",1616.00120535054,1.37835242959205,0.305407710191888,4.5131553
1204511,6.38702142840064e-06,0.000119744369048996,12.4467310464253
"Clec2m",0.419496029866574,-0.0581340165710525,4.78471990594806,-0.012
1499309706267,0.99030599617044,NA,NA
"Bola2",2032.32760326053,0.721297166412017,0.192085669669616,3.7550805
7239582,0.000173285723879177,0.00151444421523809,4.68317098974354
"Ly6g2",1528.81889779399,1.86687813098843,0.587031544318999,3.18020070
480905,0.00147173080649938,0.00792701858152624,9.03098241202252
"Fcgbp",86.8697597669051,0.611784721614803,0.792367291497263,0.7720973
94957799,0.440056745018132,0.581868060108743,0.331288497636998
"Wbp1l",5066.70815155808,0.14845776001126,0.100369116828012,1.47911792
693813,0.139108799730419,0.253087932923739,0.203983677499882
"Tmem262",3.54235205713638,0.202423912093796,0.735455496657238,0.27523
6112876774,0.783134843909275,0.859821318856139,0.0305722211106999
"Samd9l",6574.17873340748,0.541338530458362,0.275728488927179,1.963302
85841929,0.0496109935216472,0.115679879417015,1.16762853857441
"Fbxo40",1035.21914674191,1.83465635001605,0.658209694553142,2.7873432
5124395,0.00531421565240831,0.0208400194671475,7.10173480693704
"Rnf150",1078.21359000608,-1.46540190745116,0.206290011710125,-7.10360
087385284,1.2154755600715e-12,8.99945481602099e-10,-30.5223975575649
"Zc3hav1l",205.95626725633,-1.13644117663034,0.273297345562213,-4.1582
5910892956,3.20682161202316e-05,0.000410010542893295,-8.86347713038358
"Utf1",7.26085805602239,-0.818564168129837,0.843939656418336,-0.969932
105814068,0.332080335458591,0.476869949770855,-0.606156153185996
"Fancb",103.676248755597,-0.668534640725134,0.24315293853867,-2.749440
92694489,0.00596970222147302,0.0227427225311217,-2.52940754766885
"Hs3st3a1",194.8370888287,0.00961516770083535,0.476567489728942,0.0201
758783552445,0.98390307025277,0.989723009277618,9.93262554882123e-05
"Lrrc49",485.245945001722,-0.508937343864489,0.234386512444548,-2.1713
5934383125,0.0299040196430935,0.0784643486746114,-1.29530198859776
"Atg16l2",1302.39098251986,0.268339108022933,0.285414049938082,0.94017
483750764,0.347127886442968,0.491882233275072,0.190390878332766
"Ankfn1",39.0225390898251,-4.26690208469765,1.28156187687445,-3.329454
59887119,0.000870162495032336,0.0052953896155245,-22.3624870157509
"Phf13",739.937788563034,-0.581832634802231,0.136247349111782,-4.27041
435004269,1.95110140647931e-05,0.000283054875175101,-4.75349685654275
"Lix1",217.219901550228,-0.311097435588166,0.309486238481439,-1.005206
03796354,0.314797648369218,0.46004531164016,-0.24154547237606
"Flrt1",2194.51334726604,1.60705681716073,0.472761143611497,3.39929970
742555,0.000675586415305193,0.00434213493390558,8.74140738507663
"Slc38a9",1709.71394032613,0.23248227817306,0.196387455455525,1.183793
93242716,0.236494644665177,0.37465489342245,0.228239467515649

"Sned1",3857.25410406024,-0.62781032663845,0.365467325756408,-1.717828
87933708,0.0858278322682629,0.175397869569918,-1.09282839670503
"Gjb1",1136.43741927114,1.90892195815836,0.651842774711794,2.928500602
00847,0.00340601173122558,0.0148996541937024,8.02972230099729
"Cd300lf",162.672626566701,-1.27502906000866,0.553237333207634,-2.3046
6923230962,0.021185105316381,0.0601349808500214,-3.58431523484096
"Akap10",842.695333646716,0.0396142976412965,0.122331115231652,0.32382
8468058032,0.746067902150455,0.834819275736022,0.00715196584288729
"Ccdc88b",1394.49732792347,-1.31397176489194,0.449313340034705,-2.9243
9962897706,0.00345121359671713,0.0150574541751601,-5.51327062973082
"Tigd4",33.6464537560356,-0.586073382871371,0.366800576574984,-1.59779
84231755,0.110087845695118,0.211873735599982,-0.909448027315878
"Trim16",882.882342860485,-0.62180692900922,0.198600815786299,-3.13093
844326553,0.0017424868030611,0.00900238677426743,-2.9288757500362
"Angptl8",207.413547399744,-2.82797815713008,0.72535101485685,-3.89877
190381843,9.66817943348055e-05,0.000964956312586693,-19.6358619548663
"Pygo2",2592.62867917927,-0.254540156255194,0.117165045637381,-2.17249
227250746,0.0298185518773648,0.0782927719505601,-0.648390138119754
"Cdca4",563.861504482425,-0.715172020843048,0.166715689649279,-4.28977
034103724,1.78857976098413e-05,0.000264194910452034,-5.89217600184395
"Fndc11",6.63361376366184,-0.16242734331372,0.640529965880358,-0.25358
2739240741,0.799817932936701,0.872162120833555,-0.0222168045413895
"Diras2",22.4321381893686,-2.56046552847117,0.494389414988034,-5.17904
601281389,2.23023460526477e-07,8.36252198720239e-06,-29.936324131061
"Bri3",4775.93800268436,0.761458218327755,0.211321677439098,3.60331333
517454,0.000314186402696957,0.00239544130360738,4.59478189483278
"Bex4",495.732552827445,1.04238452141471,0.401526452625887,2.596054418
32727,0.0094301128624603,0.0323313417605505,3.57716991902552
"Stx19",8.93409331635386,-1.34251761661467,0.708793330369578,-1.894088
95243788,0.0582132038655463,0.130554525991537,-2.73331796075155
"Foxi1",1742.01491127047,0.756482374858903,0.517320142199925,1.4623099
1826827,0.143656301839722,0.25930481144889,1.02106286545952
"Lonp2",4315.68341277109,-0.0231077851032355,0.365357078697074,-0.0632
47125758838,0.949569718940364,0.968734515624084,-0.000734011452789938
"Gimap6",2252.09521289225,-0.683125475933668,0.156069123516794,-4.3770
6998373808,1.20285361171875e-05,0.00019626142026186,-5.83120210670071
"Gpr157",297.112591268455,-0.822367713401102,0.171565914792368,-4.7933
0474468862,1.64056126055784e-06,4.02360546645998e-05,-8.32297563581239
"A4galt",1735.33676350683,-0.26910674547818,0.237371583512329,-1.13369
402308513,0.256922917630738,0.397799019134768,-0.248064852636105
"Usp14",2185.90082839753,-0.2510512400775,0.155844997188031,-1.6109034
2717001,0.107200772925941,0.207654012278798,-0.394622922035755
"Cxcr5",46.2593786284113,-2.7729413130418,0.810590907364585,-3.4208887
4652845,0.000624168598419578,0.00409352147056338,-15.2466009926562
"Rell1",1387.37058459974,-0.20279488344053,0.146216060893561,-1.386953
5412266,0.165455929923737,0.288210075577978,-0.252290145576056
"Klk9",0.648550227941978,-1.47331591592877,3.13282086874131,-0.4702841
23369208,0.63815203899488,NA,NA
"Tnrc6b",5181.17027634287,0.495932133638989,0.237483791128745,2.088277
8200645,0.0367727810153508,0.0919107401919382,1.18375895179351

"Ccr4",27.7874043660484,-2.01500326895584,0.496201936029798,-4.0608532
99119,4.88936879251474e-05,0.000569491712762559,-15.053618570431
"Sstr2",3.79640523507252,0.793213595945715,0.878661526542995,0.9027521
65633715,0.366657443577751,0.511542075905442,0.53171125044685
"Gm8566",0.785437030416088,1.85041629420926,2.14750498160402,0.8616586
73698229,0.388875373259062,NA,NA
"Tshz2",3567.34673763892,-0.419640006038971,0.280347021225266,-1.49685
915764298,0.134429909424889,0.246414813760129,-0.587806092351903
"Ankrd16",3010.03297771976,0.446759771150321,0.184177664256922,2.42570
005952028,0.0152788919916918,0.0467676972750109,1.36822974108672
"Pcdhb16",80.2099406043071,-1.633029816635,0.273388123218511,-5.973301
9759961,2.32498959280397e-09,2.83557843472367e-07,-24.619312073132
"Npm2",40.0554149074982,0.180666713792778,0.356059744164745,0.50740561
5921538,0.611870246084681,0.731423337512053,0.0565058391559337
"Trappc9",1681.84516492312,0.158547092648609,0.259623226359498,0.61068
1466646094,0.541410477900617,0.671827244835883,0.0630627478434581
"Stpg2",3.03704244905529,0.00939113323236675,1.08415063684753,0.008662
20330753491,0.993088648149284,0.995744238486822,4.0051709215454e-05
"Marcksl1",3455.46206604885,-1.18878383818308,0.196530113011138,-6.048
86355566133,1.45871132076568e-09,2.10595000632818e-07,-18.275565025994
7
"Gp5",8.09235118191253,0.014441505042087,0.71821355486249,0.0201075361
837914,0.983957588356412,0.989723009277618,0.000149183109860894
"Kcna3",7.06889179021816,-2.26440764609902,0.722688786771097,-3.133309
5068711,0.00172847073590316,0.00895607823774645,-10.6776395436589
"Stbd1",982.174932083411,-0.80990284193629,0.286554172601497,-2.826351
59203422,0.00470815551195176,0.0189591884459671,-3.21164265805357
"Rpl9-
ps7",2.62473265613955,-1.34993656244477,1.11107313820851,-1.2149844290
3714,0.224372030219512,0.360570827101298,-1.37702556729987
"Kcna1",23.03548419457,-1.35030959358009,0.65472404521691,-2.062410268
02786,0.0391686925554057,0.0963805598624973,-3.15898280342308
"Synb",7.06380358095966,-0.129320794685739,0.668320651660539,-0.193501
120105181,0.846566528916346,0.901940400842908,-0.0133467899911107
"Palm3",1343.80439791321,0.62933375208215,0.570142971593471,1.10381743
428889,0.269672340484722,0.411977692922597,0.5580844075531
"4933428G20Rik",0.774540349481555,-1.08445508899106,2.11010680255619,-
0.513933743864218,0.607298314461362,NA,NA
"Ino80c",1128.45259040519,-0.211401326407899,0.12585808494143,-1.67968
014535004,0.0930195648222455,0.186728660033372,-0.354752297732159
"C2cd4a",4.51677615222687,-1.74925388456147,0.933148583609299,-1.87457
165481148,0.0608516757397761,0.135073539796693,-3.50189416655551
"Dipk1c",0.734690332037698,-3.28965978178337,2.93874097167994,-1.11941
12762864,0.262964722518523,NA,NA
"Prrg1",471.92303270006,-0.426113515867013,0.197422322720776,-2.158385
68807483,0.0308978575254179,0.0804549180062231,-1.07383089226027
"Gigyf2",3990.40214605794,0.434725397921808,0.164974017004013,2.635114
33992199,0.00841089450767968,0.0295754050695651,1.53058647285495
"Hes5",3.3296354053715,0.152279769497252,1.30376004001157,0.1168004577
71279,0.907018180986789,0.943127331475567,0.00891658617983103

"Catsper4",54.389336042891,0.00637670081103932,0.370246085486222,0.017
2228716548486,0.986258815952337,0.991182975731778,5.64728545381041e-05
"Tmem196",5.78911755931481,-1.12478809440193,0.729435005889175,-1.5419
9906135684,0.123073821690311,0.230888585910809,-1.64873688103212
"Timm8a1",1081.49650757927,0.974617089024067,0.208990235552232,4.66345
753641913,3.1094037003477e-06,6.82738213393912e-05,9.34851167431425
"Zfp473",60.6612160744118,0.0576471680395214,0.317833099299487,0.18137
5596709648,0.856072772193797,0.908458449473496,0.00553448136475315
"Krt35",0.41033388454827,-2.66917809970653,4.73531307650841,-0.5636751
05865366,0.572975262336283,NA,NA
"Neurod4",0.347557051178682,-2.43206479204848,4.73954195221595,-0.5131
43425370753,0.607850996969186,NA,NA
"Tmem229a",7142.33384524177,1.70936149040585,0.481468550415681,3.55030
767623359,0.000384781165539356,0.0028078080709904,10.0430989914843
"Rgmb",1517.10223169251,-0.455496678159167,0.105622937956756,-4.312478
77563922,1.61434383639298e-05,0.000243553280906977,-3.78981199522802
"Eno4",26.5365159609706,0.713766736773753,0.531593380603612,1.34269304
851631,0.179371386554348,0.306707295890264,0.843573368918054
"Fcrl5",8.23291259353714,-3.58013647519838,1.80366192451487,-1.9849265
6885316,0.0471526418929871,0.11127840857292,-7.86097737794644
"Ccdc187",14.2725043707149,-0.838897579722791,0.487598188109684,-1.720
46902588999,0.0853472192734256,0.174782069256722,-1.46321807727751
"Isg20l2",1184.75295382422,-0.181132580917477,0.0955300286101337,-1.89
60800447019,0.0579494608796752,0.130210474882058,-0.369257441005709
"Arxes2",1235.80515738706,-2.91217736056024,0.954538647124908,-3.05087
423053198,0.00228176131506241,0.0110588571019356,-13.1179717190855
"Zbtb33",1083.09981480178,-0.144161289903234,0.112600057075785,-1.2802
9499848483,0.200441406044721,0.331991720613317,-0.158958761298913
"Ldlrad3",1183.74980798528,0.53095404610813,0.158488236389826,3.350116
43893979,0.000807775963838201,0.00499208105956173,2.81400462864943
"Cyb5r2",3.59929842808308,-2.60294470837906,1.4872744899781,-1.7501441
2330664,0.0800934477186973,0.166470743376949,-4.66691249396367
"Pirt",9.34246710931682,-1.46120699490785,0.990698108603259,-1.4749266
0197761,0.140232277505998,0.254656696173229,-1.99869581301125
"Arf1",13256.9897700835,-0.125782541842457,0.162990021063028,-0.771719
280862093,0.440280708585835,0.582085249254585,-0.0680657591129955
"H1f7",0.176189212199078,1.34515345467051,4.78471990596623,0.281135255
794846,0.778606660958885,NA,NA
"Tenm4",613.244793173416,-1.57769628647682,0.56651329780505,-2.7849236
5949677,0.00535403267749465,0.0209540203022462,-6.09846627555419
"Olfr731",580.134021984285,0.699925745175456,0.333620315750302,2.09797
099316732,0.0359077078338215,0.0902343713550525,1.68356279868113
"Gm4737",5.36648294369293,0.597937912450425,1.45223191925991,0.4117372
0569036,0.680532051526127,0.785055847867496,0.144701225917076
"Lmod1",554.813991534979,-0.781053667491084,0.267983925860182,-2.91455
41658293,0.00356196990750649,0.0154096492692615,-3.25915058711005
"Taf13",1757.61077689475,-0.0722844457500065,0.174200898533762,-0.4149
48753757416,0.678179418432316,0.783299184068916,-0.0176547932395029
"4632415L05Rik",1105.10092847132,0.171208106875253,0.126709228754858,1
.35118892726027,0.176634920638797,0.303011409696926,0.204419880398846

"Tmem72",3819.3516068678,1.25987009271683,0.329519527862543,3.82335487
334875,0.000131648091294744,0.0012305246807579,8.44152601517499
"Rbm15",831.41754773764,0.167481893330658,0.120276993588976,1.39246823
796574,0.163780653280222,0.286278404774158,0.209484760829457
"Arid4a",3713.85246055941,0.500585937848897,0.344844574920425,1.451627
70202898,0.146605151809863,0.26333553661803,0.667944959915904
"Entpd1",1061.36355345443,-1.46273771650957,0.31528236671836,-4.639452
98220953,3.49332598594178e-06,7.49316502463067e-05,-13.894449306342
"Col6a3",4047.08964772322,-1.45127614283818,0.268324310924657,-5.40866
438019359,6.34964921880852e-08,3.23251855008691e-06,-18.3473943476656
"Dmrt2",685.562010981583,-0.0380836514539623,0.377951386011633,-0.1007
63359689836,0.919738313488305,0.950800998695454,-0.00192133899576932
"Nat8l",1796.55682196321,-4.00836009407192,1.21935598579908,-3.2872763
4977339,0.00101161507725019,0.00592326449736462,-20.5583480019936
"Nwd1",14.0438861884261,0.354778998356216,0.421086841806432,0.84253166
5996116,0.399490422505281,0.543753176824292,0.216152600797834
"Olfr49",2.71971322717938,2.36703841839478,1.4745265044935,1.605287128
56732,0.108430630483378,0.209469972571576,3.70009499304127
"Kmt2d",8975.24273217456,-0.381291581091484,0.227200924671164,-1.67821
315711343,0.0933054941404273,0.187052285086372,-0.639184662741101
"Selplg",1075.77320344552,-1.20146367512991,0.377538027154048,-3.18236
465922856,0.00146077769849394,0.00787890555054906,-5.81936893663491
"Mcmbp",1989.85716135212,-0.0879199778956477,0.189349478318692,-0.4643
26486010543,0.642413850703856,0.755782611106775,-0.0246177253038575
"Tmem81",350.199969245759,-0.0159000124375036,0.136400414627903,-0.116
568651795367,0.907201880568193,0.943127331475567,-0.000931008968738583
"Asb8",1509.40241905169,-0.0818254165848573,0.166860509797351,-0.49038
2156234767,0.623863500952394,0.741421506965575,-0.0244810174580609
"Gm7075",1.13637561216309,-0.542696917440409,2.07511208126046,-0.26152
6556729777,0.793686472621807,NA,NA
"Bend7",554.767046022699,0.692821861540287,0.166049527574746,4.1723808
0504877,3.01433297234413e-05,0.000392346223596568,5.43405535285854
"Muc6",3.65131289898863,0.992040824345699,0.968320754955693,1.02449608
69305,0.305601018144473,0.450139374674274,0.791845024403814
"Cracr2b",966.861312158721,-0.483939708283522,0.150279649664377,-3.220
26108900517,0.00128073895177088,0.00710234587077036,-2.39420950723706
"Gpr85",77.799885638544,-0.142753264241285,0.354044409194226,-0.403207
226365131,0.686795784025551,0.789805024885296,-0.0336853689815707
"Nags",3.03272511354678,-0.796324579222674,1.10897238830622,-0.7180743
07006809,0.47271147118946,0.610478294069122,-0.392996166086117
"Amigo2",459.472467277984,-0.617020237449378,0.188048629247039,-3.2811
7381083805,0.00103376018993626,0.00603119574607334,-3.15347319259329
"Mfap1b",2440.3537226703,-0.164392613937732,0.124257042327676,-1.32300
440166776,0.185833914047456,0.314393896407574,-0.190220112637125
"Fbxo43",9.4361772853359,0.897437760956963,0.557955559253364,1.6084395
0037505,0.107738956411665,0.208402199614548,1.40743855082832
"Fbxo10",791.756485420594,0.523489760154319,0.135610131207265,3.860255
53912505,0.000113268509267365,0.00109494764189147,3.56865520592684
"Rnf149",3759.16465893665,0.485757105076918,0.168237053156202,2.887337
2183113,0.00388517584004151,0.0164394875280229,1.9955237350624

"Ovch2",0.0810182756847091,0,4.78471990596022,0,1,NA,NA
"Gng7",114.453166103902,-1.76831871870843,0.409569736990203,-4.3175033
6756628,1.57804001840078e-05,0.000240567820787947,-14.7345307703583
"Crebrf",2714.40973110637,0.293144169230179,0.142626893461968,2.055321
84088652,0.0398479291185726,0.0976209155780501,0.682047841590014
"Bcl11b",738.001494020283,0.724893517315027,0.332876069355663,2.177667
85914704,0.0294307734406337,0.0775146860118164,1.85376139426716
"Dip2c",848.091608405576,0.0115410021786914,0.161089063025299,0.071643
6110679895,0.942885532612582,0.964603710314293,0.000415913769497768
"Rbm33",2703.76191016366,-0.224041597442709,0.176359327562738,-1.27036
999141998,0.203952867327383,0.336322142011951,-0.24413495234861
"Syngr2",4404.09519011012,0.0104023061597951,0.208168911815591,0.04997
05074550712,0.960145890590656,0.975827376348808,0.000254540028508836
"Sacs",282.567558376542,-0.929514346988744,0.197643027585708,-4.702995
89286375,2.5637157863201e-06,5.81782255149808e-05,-9.06462335903043
"Zfp738",1202.04099504666,-0.00906363104324858,0.149771457167574,-0.06
05164109013616,0.95174434579491,0.97005286222212,-0.000275577090034389
"Dleu7",701.473856793505,1.64090175959185,0.809193697731913,2.02782320
746087,0.0425782948854017,0.102822837958331,3.73263765371156
"Tas2r126",0.477522058722585,-0.0912668335024808,3.1568899422976,-0.02
89103627844739,0.976936080770642,NA,NA
"Frmd6",1011.86211430501,-1.39242035520867,0.196620359461869,-7.081770
97742876,1.42323734916487e-12,9.56906270138506e-10,-28.9168330628031
"Slitrk3",1.3711463966441,-3.4999225983523,2.30542073630384,-1.5181274
9110756,0.128982262035063,0.238774052558455,-5.01272060593631
"Ankrd46",2975.76411885408,-0.492300421441085,0.238640102895556,-2.062
94086981913,0.039118246464875,0.0963170272018828,-1.15203721880661
"Pskh1",1364.76761627191,-0.0344726225062643,0.113773576775322,-0.3029
93221126733,0.76189502768755,0.845692203680511,-0.00577760501717907
"Gm9823",0.287192621222655,1.47558876133794,4.7324125635603,0.31180475
9521605,0.755188906682535,NA,NA
"Ckap2l",126.129166573205,-0.85466752104642,0.44107133062744,-1.937708
17031029,0.0526588365775627,0.120949699091685,-1.8053830309133
"Mfsd6l",119.504547392977,0.764297621761914,0.265545955001264,2.878212
25429051,0.00399935949436715,0.0167796341480517,3.12413484266677
"Ric3",157.738534199223,-0.746530426828901,0.466377839278481,-1.600698
75529214,0.109443656991901,0.211063092693518,-1.16130136783855
"Lhfp",2725.83261663469,-1.47396503675322,0.425616608382278,-3.4631285
7093525,0.000533933197915498,0.00364517839389229,-8.27535556376219
"Gm8258",0.151078246467733,-0.0675065776026396,4.78471990596623,-0.014
1087835713149,0.988743192877139,NA,NA
"Npy4r",2.3651999748813,-1.79681528409502,1.56380560270147,-1.14900169
240411,0.250555282804731,0.390295350653244,-1.69053638599861
"Pcdhb18",87.9547420589449,-0.708235092905476,0.370889715843351,-1.909
55710727932,0.0561902622416485,0.127145139428719,-1.46068248028004
"Pou4f1",10.5776512108468,0.286200755236098,0.653426803985643,0.437999
717015566,0.661386488566015,0.770031274500517,0.0747911687264642
"Coa7",867.776975847591,0.905233527131134,0.291688955494436,3.10342064
750683,0.0019129752749463,0.0096480061849206,4.20119241848514
"Arxes1",320.461884923824,-3.07426411941486,1.04790477231965,-2.933724

70535622,0.00334921123627899,0.0146973743709345,-12.9736602476113
"Omd",103.652287915497,-0.284685939481445,0.339736914852512,-0.8379599
83256557,0.402053178448902,0.545945600208698,-0.172302162619092
"Pdp2",2485.34136479507,0.830479724439155,0.312680769774849,2.65599872
047506,0.00790739144125003,0.0282452449308553,2.96218012556524
"Fgfbp1",238.193775246414,-0.930025575860456,0.533139616871291,-1.7444
3156432132,0.0810838650440985,0.167974203211839,-1.65911434914704
"F2r",2921.20327525988,-1.08850246642033,0.391314733243488,-2.78165469
875889,0.00540825483962347,0.0210942494224802,-4.20026413329147
"Olfr1394",0.286703655845716,1.91201515486892,4.7586561100617,0.401797
295422579,0.68783321274769,NA,NA
"Socs4",2210.15136722976,0.25201821996957,0.0799306725836813,3.1529600
8182249,0.00161623906652993,0.00852459433497079,1.20081637476407
"Scrt1",3.42486812487544,-1.76471821638322,1.34208755771606,-1.3149054
2940908,0.188541687806618,0.317679383758507,-2.02362467061074
"Osr1",119.632765393247,-1.13740465192586,0.431988260148738,-2.6329526
9073804,0.00846461369502769,0.0297267719426603,-3.9987817547886
"Fam171b",87.6217411850371,-0.466942795368268,0.285913523310557,-1.633
16092908652,0.102435154874241,0.200972050255365,-0.74925147575266
"Olfr843",1.76487250264877,1.31475170491163,1.30096577644751,1.0105966
8802493,0.312209501641266,0.457339176140321,1.02856967977971
"Tprg",3.64912880297602,-3.43613156317147,1.31688474211115,-2.60928800
622512,0.00907308441137446,0.0313553704099573,-11.8971578838669
"Gli2",53.8806737739666,-1.77963897531808,0.552500379293598,-3.2210638
0740126,0.00127715710129706,0.00708449762762167,-8.80893942370405
"B330016D10Rik",303.07310795409,0.275939887407702,0.17158708339768,1.6
0816235082315,0.107799626450244,0.208498870799211,0.432624530566724
"Zfp407",365.892705748455,-0.337593792884296,0.211489860865394,-1.5962
6466962955,0.110429714559892,0.212384945205048,-0.523052587618883
"Mlf1",38.5132532667555,-0.320830035789362,0.420413011155168,-0.763130
605562891,0.445385509088379,0.586502540139127,-0.171187938631925
"Ranbp3l",1121.35508153215,1.86154025323079,0.688123119019088,2.705243
00343868,0.00682544391781485,0.02529604742626,6.84508292614338
"Timm29",1829.90802041841,-0.167881378843098,0.272359835415123,-0.6163
95507021871,0.537633517982732,0.66903400732284,-0.0674749487676963
"Nupl2",338.896610958683,0.0418556418614228,0.176806288531643,0.236731
63556019,0.812864990817095,0.880807024060893,0.00531218073534125
"Cyp4f16",1015.79519082124,-0.504059213643789,0.129673247714223,-3.887
1488339264,0.000101428568640124,0.00100235896165491,-3.48073003787817
"Smim5",811.85306526798,1.24625966637695,0.342083756165339,3.643141902
86954,0.000269330258150846,0.00212865884613912,7.66731741738413
"Ccdc57",361.108871544619,0.242989725865668,0.259467758818739,0.936492
946067404,0.349019442748037,0.49398817470247,0.171366973314475
"Msx1",86.2362950042658,-0.754225106373832,0.304589841612268,-2.476199
14827604,0.0132789486110358,0.0421270647604906,-2.38867984331446
"Inka2",168.636133092822,-1.59095890451907,0.479220010370233,-3.319892
47128878,0.000900521264564421,0.00543594278428342,-8.29640889063857
"Sult6b2",2.28415407874962,2.07361459376664,1.19488988659949,1.7354022
4670233,0.0826695824254225,0.170470667828793,3.66862241932962
"Spata33",173.022295215767,0.726613607361092,0.243912344049342,2.97899

481140693,0.0028919567531604,0.0131500402922392,3.14720364753561
"Cxcr1",0.555157322704039,-1.76238266959213,2.55316216097384,-0.690274
474739949,0.490021596599984,NA,NA
"Mypop",430.426805349974,0.108933793564596,0.138602141471318,0.7859459
63087005,0.431899145403537,0.573966728806319,0.0604782826987342
"Bdnf",159.485933432,1.57913167636826,0.42770346880127,3.6921180012734
4,0.000222394229821227,0.00184032018618171,9.94508027686
"Zdhhc22",0.964642461825953,-0.0206150348519833,2.35910927032035,-0.00
873848240577004,0.993027788537962,NA,NA
"Zbtb8b",87.6421718604864,-0.227445081404863,0.39128192431754,-0.58128
1851446535,0.56105051009689,0.689310828347143,-0.0846238947784591
"Fitm2",1663.74899517058,-0.928375921298137,0.609718408539213,-1.52263
06247213,0.127851143464365,0.237255315053602,-1.33557871869209
"Depp1",1421.25922433274,-1.88955694771037,0.603491774165625,-3.131040
10460264,0.00174188371448512,0.00900238677426743,-8.90031497603218
"Nrip1",2078.050125079,-0.6282474523344,0.261194878382023,-2.405282432
12919,0.0161599712060852,0.048903789938887,-1.89598822616551
"Tyw5",517.493144900103,0.133813133221623,0.168989754072353,0.79184169
4522684,0.428452987472063,0.571182804579699,0.0749415063199506
"Mmgt2",703.376855850649,-0.874147656564224,0.237978220018445,-3.67322
545944108,0.000239507983252069,0.00194580277393701,-5.45650017212647
"Cd300e",6.5337945114373,-1.26429240390683,0.714081263538906,-1.770516
14215606,0.0766411977437989,0.160803838761282,-2.31058293135317
"Defb15",0.366299305630371,-0.551305484500155,3.86278939216063,-0.1427
22118275205,0.88650964768746,NA,NA
"Duxbl1",9.97004144199268,-1.98958051177692,0.889069965150086,-2.23782
220721072,0.0252326516384401,0.0689829278808615,-5.3199318237834
"Tmem136",885.961148239356,-0.0863432300335052,0.260388230464107,-0.33
1594211764526,0.740195690867765,0.83068582828017,-0.0160169817558071
"Fbxl13",21.1339330925515,0.177699138732639,0.493534184671798,0.360054
367562825,0.718806476745256,0.813754205630784,0.0366232446930587
"Cxcr6",272.314501627866,-1.40722656802939,0.486537983801278,-2.892326
22093522,0.00382400663477001,0.0162299698225393,-5.79903399307714
"Nkx1-2",0.0820881951088163,-0.0627941126595849,4.78471990596623,-0.01
31238847610044,0.989528955554716,NA,NA
"Jaml",499.385305355938,-0.951574973629889,0.413100513610343,-2.303495
01459943,0.0212510073371603,0.060295713192659,-2.67249286006658
"Phldb1",4290.14882121523,-0.463987854375154,0.209084988840347,-2.2191
3518014172,0.0264775292894246,0.071662807618712,-1.22297147886686
"Gm9826",1.35779381165931,0.51626925049476,1.74355266687836,0.29610189
6032246,0.767152266057535,0.849689552578381,0.0840921185506823
"Tob2",3274.92154507302,0.0422653330588416,0.268544136936799,0.1573869
14273941,0.874939925827922,0.92194004938353,0.0034351183213653
"Thnsl1",348.546963274831,0.245889255379079,0.161142269881775,1.525914
06065882,0.127031268497246,0.23613228479095,0.35490747844624
"Sp8",0.321361678232658,0,4.78471990595347,0,1,NA,NA
"Tmem252",3101.40322510564,2.08780986377246,0.552982680621441,3.775542
95448491,0.000159659506957242,0.00142895347383657,13.6768518700048
"Ccnb1-
ps",0.185885547873138,0.406508005289677,4.78471990596623,0.08495962423

68938,0.932293489796378,NA,NA
"Mlec",21409.908996875,0.684543655866152,0.26528139712419,2.5804434961
7808,0.00986735025008845,0.0334074657364515,2.32674737436636
"E130311K13Rik",419.517324301954,0.350731512785853,0.139515466174062,2
.51392567723118,0.0119395592662015,0.0388491801177002,1.13919991065923
"Gja3",58.042020242747,-0.0603840669321068,0.692624980482868,-0.087181
4742950935,0.930527264652945,0.956679177759194,-0.00267424011291097
"Igf2",205.025489465391,-1.41999066109596,0.381882781240017,-3.7183940
4878399,0.000200493351347449,0.00169670979365128,-9.05821175916379
"Gm5946",0.948585271688899,0.453151905612155,1.47137698999903,0.307978
110771227,0.758098988408404,NA,NA
"Gm5763",0.481277464418446,-2.13832142043315,3.78024677278104,-0.56565
6569256202,0.571627264456176,NA,NA
"Morc2b",0.674714426678182,-2.80416558230773,2.5533220597387,-1.098242
0222362,0.272098821137283,NA,NA
"Myof",5010.19676621011,-1.10540367009396,0.18310848904124,-6.03687833
306843,1.57124098423269e-09,2.18828976504064e-07,-16.9513380232051
"Nog",48.0267622595168,-0.289958972390424,0.530509851099846,-0.5465666
12833456,0.584676511875026,0.708648140022333,-0.0998607543436652
"Rtbdn",0.270802945932136,-1.62943893969085,4.76019598996016,-0.342305
010786854,0.732121365040968,NA,NA
"Olfr1336",596.851321942023,0.590344132065355,0.226286767802281,2.6088
3187204817,0.0090851867514504,0.0313805086412365,2.04351654706039
"Gm6377",69.8254722736041,-0.576371245780348,0.453616842030264,-1.2706
1253546202,0.203866525173687,0.336236635750003,-0.628210129710979
"A730049H05Rik",483.210711640097,0.548067741217085,0.504572503977938,1
.08620215508423,0.277389564049889,0.420930945586796,0.474235608510798
"Ctxn1",521.276922341824,-0.455076292170891,0.283970781054836,-1.60254
618619729,0.109034882821248,0.210407971620022,-0.709330561597361
"Exd1",135.676113776863,-1.79010930770352,0.565489319898888,-3.1655934
8640501,0.00154766919731855,0.0082426806908815,-8.58971359924117
"Samd13",31.0542127919328,-1.00932597028509,0.582177604145872,-1.73370
8000956,0.082969906182245,0.170900827249251,-1.78314777748766
"Ccdc169",10.2957526772657,0.599884319169578,0.685051584174613,0.87567
7588414529,0.381205332375689,0.526137295297032,0.385241560148099
"Lemd3",1027.04478528537,-0.133880172914563,0.133220260812633,-1.00495
354158522,0.314919221120649,0.460188486353176,-0.103906962052579
"Rhno1",1223.95246238272,0.0119651177400466,0.196322891562939,0.060946
1160886107,0.951402122354246,0.969930765084564,0.000365302058957567
"Tpcn2",1010.68860707598,-0.54516369384285,0.166053426848539,-3.283061
98907961,0.00102686082827627,0.00599812235761854,-2.78922580377278
"Mex3d",537.402577436645,-0.547818183632783,0.218815867594684,-2.50355
785279482,0.012295157743122,0.0396885763178965,-1.76764048641975
"Vmn1r26",162.3006330659,0.426357677131688,0.403921166336491,1.0555467
5680577,0.291175337023604,0.434811727141475,0.35508864566481
"Krt90",0.67111442609704,-1.84776178078064,2.54872950829705,-0.7249736
68161133,0.468468184696873,NA,NA
"Ccdc6",4598.87731000847,0.90065709234072,0.214524238394536,4.19839314
699862,2.68815642773184e-05,0.000358016899097784,7.1466513136138
"Speer4b",2.27307817508361,0.0338272616279803,1.25394257027989,0.02697

67232006724,0.978478299468738,0.986370069436534,0.000464234203452303
"Lurap1l",846.243339726426,0.0408084304875535,0.232369570286862,0.1756
18651087469,0.860593548638692,0.911681431725247,0.003773337537886
"Tprn",1732.73331232,0.75991069229424,0.19589751504178,3.8791236945102
1,0.000104833439880207,0.00102767340914242,5.2285334940311
"Gm8666",0.22397603279477,-0.927481920711741,4.78269286764269,-0.19392
4624971555,0.846234901762738,NA,NA
"Tbc1d12",658.863784187001,0.220109035999667,0.209532471523715,1.05047
697093935,0.29349887346318,0.437477336196398,0.181970826424475
"Fndc9",1.57879387113114,-0.813011117843657,1.36351318570127,-0.596262
013722674,0.551000203361193,0.68095341098666,-0.312408780456468
"Zfp454",115.840394580724,0.0602022221936704,0.184101733494981,0.32700
5189200522,0.743663960383911,0.833043772237475,0.0109970851737045
"Ggnbp1",1657.02245473644,1.05496879349025,0.272094438503459,3.8772155
7005231,0.000105658736026667,0.0010342038328554,7.25198566105848
"Klhl11",47.8911942150091,-1.22473857559254,0.421419901407782,-2.90621
912135906,0.00365825169656624,0.0157215321974099,-5.08600131174161
"E130114P18Rik",2.73202140740691,-0.35951734319544,1.05540973289808,-0
.340642436760774,0.733372778166394,0.825547164060294,-0.06892266839915
19
"Prss50",1.27553483921765,-1.05902587466892,2.03614426546004,-0.520113
379308929,0.602984553743038,NA,NA
"Mcat",1262.1286477715,-0.0675103220005346,0.26381984296057,-0.2558955
43121161,0.798031496150966,0.871026540077896,-0.00932201643628913
"Foxo3",3317.4906951713,0.425609065105734,0.2030900632952,2.0956666131
2767,0.0361117755351918,0.0906187129195842,1.02192760363172
"Rpl29",14188.2616911198,0.106018405673035,0.171014873878955,0.6199367
53267875,0.535299427373929,0.667088599957616,0.0429196864999968
"Hoxb3",2044.3060902755,-0.117876955048675,0.228849215208453,-0.515085
686185569,0.606493144606488,0.726956619753937,-0.0375896022496544
"Tmprss11f",6.95267128637255,-1.2498052315,1.05811157879949,-1.1811658
2082771,0.237536856269545,0.375680409049263,-1.22357990962936
"Tmem53",660.287243732066,0.623284783883772,0.329529471698791,1.891438
66456197,0.058565811362374,0.131162253908343,1.26609093616318
"Pthlh",22.1139213054858,-1.01424633116737,0.51920421192633,-1.9534632
1903737,0.0507647314964104,0.117792542510652,-2.16930080112416
"P2ry6",751.320618041296,-1.12188537559169,0.388905728331112,-2.884723
19604541,0.0039175794867041,0.0165442852137224,-4.60165355850866
"Insc",96.0577592450822,0.60621915391361,0.470461927875153,1.288561556
19564,0.197550555740186,0.328599277923746,0.674671160069362
"Dcun1d3",1035.80966772173,-0.0439740879745905,0.337230066119787,-0.13
0397886761883,0.896251638436218,0.936150128367139,-0.00290138489925505
"Cfap100",24.8277706940048,0.691021565630708,0.642508804124891,1.07550
520894713,0.282148601357541,0.426063617508468,0.589556524504735
"Cyb561d1",766.655050147175,-0.0485728719269265,0.127685831758596,-0.3
80409253383405,0.703641646819444,0.803255552089317,-0.0106414598498423
"Cep120",1357.94878174728,-0.503116283379872,0.11684002285875,-4.30602
691671927,1.66212840874769e-05,0.000248719721517747,-4.17545456482912
"Ifnb1",0.649044425420644,-1.20603153177195,2.44850371266669,-0.492558
588142164,0.622324514994803,NA,NA

"Slc35e4",538.687141989802,-0.208168626901918,0.242543635669574,-0.858
272889029807,0.390741796021209,0.535095065235394,-0.130170102247642
"Lonrf2",61.9617212993553,-1.81904470393107,0.442657817325696,-4.10936
988511979,3.9674017623011e-05,0.00048468922030919,-13.8829540104814
"Dact2",682.91118793779,0.458498021904354,0.232500921257915,1.97202668
88566,0.0486065598873307,0.113951028577994,0.995851512366593
"Pkd1l3",3.60417388542296,-2.58004800113493,1.17502751006641,-2.195734
12454753,0.0281109791681963,0.0747963798883041,-6.69002781062213
"Vps37c",1606.30781765614,-0.321087149669991,0.160367228510841,-2.0021
9928130943,0.0452633029663226,0.107622882828092,-0.715742425450138
"Slc39a9",2361.4376405986,-0.0243897361734533,0.158312641278279,-0.154
060572652448,0.877561975536121,0.923643478186321,-0.00193725547438115
"Vstm2a",129.531037914648,2.06037952027733,0.608869176594657,3.3839445
3107451,0.000714524368933105,0.00454100265497317,11.1149386938434
"Gm12185",152.041454059998,-0.943342461537652,0.516704022361789,-1.825
69211910864,0.0678966509520701,0.146609363836097,-1.81120207250384
"Slc25a47",172.22475192068,-0.283745182779692,0.237713997046422,-1.193
64104051593,0.232618412843102,0.369860051668824,-0.28222163674954
"Arhgap30",1694.13882411742,-1.21841389430628,0.383163252926851,-3.179
88190412112,0.00147335083451335,0.00793355276756181,-5.89304683467841
"Phf3",3690.17792832069,0.0599891544183716,0.201685359509375,0.2974393
1123361,0.766131134910293,0.848799640234155,0.00983414899294556
"Hexim1",5456.45956327886,-0.0268954226993274,0.200332540242399,-0.134
253889392029,0.893201813999642,0.93382853574598,-0.00184132621382458
"Cdk5r1",147.683282974927,-0.133584822139326,0.307330289061066,-0.4346
62078207278,0.663807727968422,0.77206651634027,-0.0345563324882403
"Zfp710",1768.26199849172,-0.209838886210552,0.213465380267627,-0.9830
11324587958,0.325601865091416,0.470521009570549,-0.158200614072209
"Rimkla",67.4255620348112,0.834160069306763,0.308423910190909,2.704589
50082837,0.00683888437460177,0.025330010349291,3.06617672614988
"Neurog1",0.153961243433233,-0.533217009430887,4.78471990596623,-0.111
441635019429,0.911266145690107,NA,NA
"4930539E08Rik",78.8660146393106,-0.506591069474524,0.436323607588982,
-1.16104437317482,0.245623854646335,0.384734408571195,-0.4838968182539
49
"Rnf24",2607.04984101948,1.86411568959948,0.357291027531981,5.21735936
800881,1.81491924100077e-07,7.14902544758491e-06,22.0870390818069
"Efna5",840.269598157911,-0.269354692507778,0.321704413010497,-0.83727
3850200462,0.402438654535978,0.546278814128551,-0.162858792397384
"Gm5540",0.711852122387559,-2.6010799280979,2.21309260308699,-1.175314
54602023,0.239868897129158,NA,NA
"Fkrp",1077.63552707683,0.00449535344543322,0.11642190041264,0.0386126
100802349,0.969199248400994,0.981062046421496,8.594923962293e-05
"Zfp689",187.877965461945,-0.223375704362251,0.173672357985091,-1.2861
9031234336,0.198376651196949,0.329495523046095,-0.247990034400514
"Cdca2",196.941849733822,-1.21457322278006,0.383107727262937,-3.170317
73662572,0.001522723288941 9,0.00814542076482557,-5.84246081926908
"Ccdc125",421.614339904989,0.0606592820317665,0.233370731083988,0.2599
26691535005,0.794920321846876,0.868554837945466,0.00854838228504837
"Tada3",1423.08047747955,0.261045433553577,0.169896988322646,1.5364924

130252,0.12441765714824,0.232766789395162,0.380530687489912
"Nr1h5",7.31305875682481,-0.643780420072481,0.606857696244072,-1.06084
247436743,0.288761495789282,0.432692534200459,-0.539312810281918
"Atp13a5",0.0837058623428614,-0.544048378937456,4.78471990596623,-0.11
3705376621746,0.90947135024012,NA,NA
"Gm6206",5.02059535350929,0.484282410176456,1.67360679225296,0.2893645
10480104,0.772302450372425,0.852708646855278,0.0771642772091976
"Prex2",1062.95918871356,-0.42281245738394,0.228166776473672,-1.853085
11571459,0.0638701598621179,0.139878734919867,-0.831663398332104
"Mrgpre",120.157067905521,-0.784972166801626,0.326899439310334,-2.4012
649530929,0.0163385017172936,0.0492910577879917,-2.36277611114927
"Yjefn3",81.8467404293473,-0.328496969501514,0.396675777769698,-0.8281
24599259581,0.407599939590436,0.551403845287887,-0.195550240493789
"C1galt1c1",1337.07158431375,0.487411345784251,0.183093941239779,2.662
08342277116,0.00776586290283107,0.0278343372940074,1.74565635844327
"Nrsn1",3.04865179197056,0.477402745668594,1.44787079701518,0.32972744
9889016,0.741605910610303,0.831837545019829,0.0878984939223864
"Gphb5",0.128665072532121,-0.550785773111473,4.78471990596623,-0.11511
3482907261,0.908355173133819,NA,NA
"Elfn1",145.131009290568,0.209557286374718,0.356507787908408,0.5878056
34216768,0.556662761824445,0.686081575856702,0.0789525398184448
"Prss32",6.65689443350293,-1.13492768195058,1.3708410410464,-0.8279061
16003252,0.407723671018263,0.551468922034843,-0.675474674196032
"H2al3",0.425182998559969,1.53899067283787,3.71858951670983,0.41386409
1726789,0.678973642791909,NA,NA
"Atxn7l2",2032.13956850345,1.79342501175485,0.416858902949165,4.302235
11856613,1.69083747746876e-05,0.000252048540792705,14.860120338356
"Ndnf",25.4812054917775,-1.65766270643131,0.531979040865986,-3.1160301
0474412,0.00183303558175308,0.00933660600131178,-7.74760463821622
"BB014433",24.8674264008339,0.00991820326732753,0.599398846472875,0.01
65469175085848,0.986798072442786,0.991374114617139,8.59244067138486e-0
5
"Olfr982",6.22284035216288,-0.206617271414947,0.645881956323898,-0.319
899432693445,0.749044567938178,0.836582999401546,-0.0368666249508515
"Olfr729",13.7851616940089,0.899986193784983,0.603244191056481,1.49191
025314112,0.135722674406636,0.248131336669535,1.25439813762451
"Olfr873",0.896056939259796,-0.261524706172965,1.68951269219293,-0.154
79298106575,0.876984524812974,NA,NA
"Tmem121",17.1695773304977,-2.37006134107944,0.64058296331613,-3.69985
072473712,0.000215726320355921,0.00179676710563851,-14.9829738707412
"Clec4a1",455.02496044798,-1.35007589152783,0.433160551916547,-3.11680
250095336,0.00182824006620518,0.00931704778119934,-6.31283263579952
"Mterf2",253.519215834186,-0.0981887615903984,0.212494892636753,-0.462
075866257388,0.644026916260395,0.757096549962594,-0.0273224457456825
"Olfr398",36.5322452699923,0.311675674470014,0.311654277668695,1.00006
865556757,0.317277283728609,0.462407628627794,0.2403980856988
"Rapgef4",1005.11930288462,0.143167740693246,0.305577303447902,0.46851
5622979358,0.639415901327072,0.753404111921396,0.0405384505751991
"Armcx3",1511.42735431137,-0.741152840790288,0.148925976748451,-4.9766
5254223688,6.46932970349371e-07,1.9358130187683e-05,-8.04328565303449

"Olfr985",0.466085072788222,0.889826436642075,2.27249800881874,0.39156
3131491856,0.695381033674297,NA,NA
"Acap2",2074.06524618896,-0.606808683377724,0.125715609624856,-4.82683
642221107,1.38718968354572e-06,3.54487869590752e-05,-6.21822436295455
"Bmyc",766.763692166717,-0.949864092791959,0.252062222403245,-3.768371
49072019,0.000164316041039819,0.00145761336132593,-6.20351951775554
"Zadh2",3220.59113715165,-0.426564393047771,0.350415659955176,-1.21730
97318263,0.223486392975089,0.359578943905235,-0.436299246126603
"Sephs2",10006.5446427782,1.09516458221928,0.405681263902527,2.6995690
4512705,0.00694293473868272,0.0256001024082518,4.01395224525486
"Gpr137c",130.881827561332,0.251137292233647,0.258854099642698,0.97018
8583376881,0.331952500340009,0.476753819359862,0.186031210757823
"Il23r",6.32777145700154,-0.586531129495607,0.86854247319747,-0.675305
062901921,0.499481967953908,0.634828199671814,-0.266520254582383
"Ankrd34a",14.5371605258851,-2.14829596085564,0.498557688712945,-4.309
02182333521,1.63978188828552e-05,0.000246131387665828,-17.851576936604
8
"Olfr147",28.6794399216046,-0.369083959325877,0.393124679668046,-0.938
847084435224,0.34780926060744,0.492632933555417,-0.261308098911617
"Pcdh10",7.57936779273988,-2.60076348358325,1.30131528211156,-1.998565
23575376,0.0456554149586469,0.108361446240255,-5.77963225612004
"Ccr2",2750.08908570068,-1.21894513706852,0.394460508223277,-3.0901575
9919509,0.00200050314828927,0.00999124241427873,-5.61451777294578
"Dcaf5",2046.0602712666,-0.126290018417223,0.104516410378587,-1.208327
17043922,0.226921441344529,0.363138912435794,-0.127927979627656
"Ntf3",104.665207901094,-0.116391264468566,0.230712210901417,-0.504486
7977027,0.613919337362854,0.733333286767195,-0.0360993316772029
"Themis",102.465798545643,-1.22613326133097,0.582165976200123,-2.10615
754176173,0.0351906698805767,0.0887642537298169,-2.96941429091124
"Oxtr",157.971924202715,-2.29833891073462,0.716461759006363,-3.2079017
2237819,0.00133707180344304,0.00735823483588271,-11.2892918134549
"Agtr1a",1569.90100036718,0.337740299861942,0.249324286501587,1.354622
54640722,0.175537846306442,0.301846452710381,0.404557768676612
"Fam110b",201.326177989389,-0.371826993566346,0.192980948858274,-1.926
75492459837,0.0540101793707284,0.123254971602348,-0.778419859118961
"Frmd3",29.0217062719544,-0.902183614058729,0.590009674812008,-1.52909
969543497,0.126239730860143,0.235194011486839,-1.30577051403441
"Catsperg2",1.06841368729526,-0.548558964258879,2.14954639775444,-0.25
5197545320232,0.798570526870295,NA,NA
"Gm8186",84.5307977519275,-0.0276364458272246,0.320982834221417,-0.086
0994510633572,0.931387359993604,0.95715968719524,-0.001210062863235
"Ivl",542.176562724444,-3.84804867149945,1.10830804954249,-3.472002818
24889,0.000516590913618307,0.00355040170381733,-21.7056670766914
"C5ar1",406.970080873534,-1.46705325824725,0.339863875315793,-4.316590
74352666,1.58457556389003e-05,0.000240811023731315,-12.2227513837507
"Nrap",62.9465933268429,1.57353851064154,0.406900270423801,3.867135573
54642,0.000110121214199184,0.00106876228693663,10.7649766949582
"Plcxd3",11.6569598552559,0.243526053298894,0.571287309652244,0.426275
97214987,0.669906764206117,0.776612288988101,0.0615668048245138
"Ugt3a2",26120.2846204019,2.12908969719399,1.01889171470364,2.08961331

853922,NA,NA,NA
"Fam183b",2.69548328784907,-3.97496717289042,1.77893062446212,-2.23447003398027,0.0254521621069768,0.0694370024852153,-10.6025705608253
"Tex50",0.236555867302509,-0.100453541641953,4.06695318698895,-0.0246999503125153,0.980294294714312,NA,NA
"Zfp518a",867.669060855727,-0.584843321403136,0.189860325127252,-3.08038723209364,0.00206731623099442,0.0102409887885999,-2.67937610444523
"Myoz3",23.4680575842918,0.396893943993945,0.570914473682086,0.695189844170872,0.486936336033693,0.623803198422138,0.187302327970944
"Frmpd4",80.0765267065189,0.0774896302659148,0.494339782863482,0.156753781411347,0.875438899522265,0.922183844871549,0.00627748743887245
"Purg",442.216469932094,0.135030552500233,0.135665414485854,0.99532038443234,0.319580466651264,0.464665729102311,0.1034924107626
"Rtl5",896.238169891886,0.0579636020543962,0.342483085269804,0.169245152672966,0.865603815328622,0.915516310891103,0.0051162789807323
"4930515G13Rik",1.24478060642391,2.49317534328348,1.75841854503359,1.41785091514481,0.156234295539331,NA,NA
"Skint7",12.1240453508113,-1.85705922072653,0.810139939567452,-2.29226968086285,0.0218900862376063,0.0618076520896096,-5.16954793974662
"Olfr788",1.14942372930881,-1.24059016023997,2.14356392186996,-0.578751185156042,0.562757073279788,NA,NA
"Pdp1",533.021627421897,-0.591858860199228,0.157572897154442,-3.75609556521087,0.00017258481322068,0.00151076654191052,-3.84420504058223
"Gm9833",0.668753656911699,-0.546328621656648,4.78471990594358,-0.114181944271805,0.909093564675609,NA,NA
"Tigd2",805.903783121207,0.269285793942681,0.113407461287292,2.37449803466199,0.0175728297623426,0.0520405581266944,0.7959366114916
"Apoo-
ps",4.60178349712889,-1.30211370552987,1.80706056291626,-0.720570041896382,0.471174091360625,0.609093179960848,-0.64556716496952
"Gm7324",6.8184778599883,0.0231239409217114,1.01749294984511,0.0227263893329498,0.981868525625701,0.988407894710616,0.000269620931849817
"Hcar1",749.170241495649,-2.88904325035793,0.732964623789435,-3.94158620565007,8.09445327099402e-05,0.000839498137648092,-20.4622448298643
"Gm5316",12.8074000050202,-1.32947783571695,0.791830772347968,-1.67899238340376,0.0931535281794096,0.186854430262684,-2.23009990774293
"Lrp1b",0.985132912930552,-0.0619507366177694,2.02744703451123,-0.0305560320754343,0.97562360708221,NA,NA
"Kcnk3",1763.92539584526,-3.19348002703453,0.842061933571965,-3.7924526685205,0.000149166687818647,0.00135655414136567,-21.0859339186893
"Olfr509",194.484994152297,0.690173707267824,0.171642031139805,4.02100640900518,5.79500281499765e-05,0.000649136580996668,5.06578350468165
"Scn3b",14.2156910696806,-1.52599890440237,0.498943934567886,-3.05845767164997,0.00222479506875218,0.0108529032400625,-6.90258541587919
"Mblac1",176.673472795885,-0.480392479228169,0.260760253198982,-1.84227647171975,0.0654347195328782,0.14247304985506,-0.936093948046391
"Iba57",740.436015392059,0.692367338786331,0.278928384036944,2.48224052628014,0.0130559114532387,0.0415411490723317,2.20246952365604
"Lix1l",1580.02246886463,-1.45092832068174,0.207684846030463,-6.98620216358452,2.82427031235103e-12,1.52965618195057e-09,-29.4512663845567

"Prss38",1.49828526237668,-3.64550516289949,1.47612936853108,-2.469637
98743954,0.0135249845267523,0.0427476330527828,-11.4922415901657
"Zfp219",4160.59750911242,-0.290964935793671,0.149943443474961,-1.9404
9789074144,0.0523192102669301,0.12035213782238,-0.616069762682938
"Trappc1",1655.53468939376,0.309712530516792,0.168877629781482,1.83394
64552975,0.0666619427570978,0.14456395950149,0.5989943298092
"Prmt6",695.799924466553,0.125616461845131,0.0908859943234272,1.382132
22818592,0.166931104503159,0.290168732780833,0.155424329736923
"Syt12",462.306124344182,-0.766197504284064,0.358154384592029,-2.13929
393927938,0.0324118702428428,0.0834499730615276,-1.90285759444997
"Ccdc71",1572.27077615006,-0.140972027008624,0.135540428817453,-1.0400
7363882909,0.298305689813158,0.442674913340791,-0.114880869024425
"Fut4",242.519918933066,-1.46807569687362,0.221922386152816,-6.6152663
6552431,3.70883312607498e-11,1.12991919206578e-08,-26.8636339717027
"Sorl1",5607.14788620676,-0.3384131632583,0.252900214381879,-1.3381292
0675226,0.180854321364622,0.308647488662355,-0.397823444070775
"Fbxw13",0.145089169843486,-0.636494489189658,4.78471990596623,-0.1330
26488843368,0.894172431602241,NA,NA
"Zfp2",379.353486699493,0.422102663428972,0.163023904014166,2.58920718
39495,0.00961971970881052,0.0328125536093079,1.4423012074342
"Smcr8",1334.83371924186,-0.0108220060772696,0.168987605630095,-0.0640
402355954931,0.948938189170612,0.968428323871889,-0.000347178680024979
"Kmt5a",3831.97734000365,-0.175623037503274,0.302685827018067,-0.58021
5595931391,0.561769237639952,0.689759231454899,-0.0652286234160747
"Tenm2",134.945801955239,1.26395763080044,0.564065231886641,2.24080045
950156,0.0250390031819972,0.0685681502575802,3.38731435103626
"Retreg2",3875.29340471063,0.217215830230339,0.207306570410529,1.04780
002775689,0.29473074972347,0.439011471207655,0.178818530561378
"Gm5105",1.29649558627893,0.155100654766343,1.58944467066064,0.0975816
633503048,0.922264485532251,NA,NA
"Zg16",6.58926056802612,1.10494161306286,0.904344545891734,1.221814869
21374,0.221777644281854,0.357669190091613,1.1360421502907
"Rd3",18.2285000017236,1.6994871516369,0.484691754307882,3.50632569366
42,0.000454338906254253,0.00320814928903259,9.75855898447246
"Dcaf7",2948.08393856867,-0.426196659709692,0.17116756020831,-2.489938
27563479,0.0127765282139269,0.040805363982825,-1.36337828250881
"Brd8dc",201.955545963786,0.396127694810067,0.298764197491209,1.325887
43275279,0.184876986295316,0.313182578521813,0.459891950164062
"Olfr173",1.52099056337352,0.527190433483978,1.25500600034508,0.420070
050134439,0.674434281050676,0.780191018209794,0.13085736114336
"Olfr1183",2.39521561723781,0.974710278840317,1.06645026519056,0.91397
6310621618,0.360729293554622,0.505970776615209,0.664047075781623
"Krt8",10600.981960257,-0.616864625369364,0.215649284899768,-2.8604992
8547677,0.00422974506222173,0.0174913190293106,-2.49586547315792
"Cox7b2",0.125031169926583,0,4.78471990596324,0,1,NA,NA
"Gemin4",417.737686567594,-0.187847387084283,0.174498327060883,-1.0764
9964471431,0.281703859708,0.425589792080163,-0.160474139232302
"Ogfr",3080.210943439,-0.465737378399429,0.18200687091877,-2.558899980
25013,0.0105003947653502,0.0352548271191614,-1.557962704522
"Rarres1",129.53223417248,-0.697349811784293,0.34509028890916,-2.02077

495135732,0.043303066928455,0.104273387294634,-1.57652598929939
"Prokr1",7.5267594061196,-1.97459481970418,0.722270546038882,-2.733871
44267944,0.00625944781935749,0.0236276808999876,-7.3955217247306
"Zfp683",44.8308611690724,-1.46289729414156,0.578176418193211,-2.53019
190701876,0.011400015493954,0.0375532188040405,-4.80122333690581
"Tmem241",229.906153865907,-0.675464734583891,0.329326230883338,-2.051
05051235099,0.0402620278984493,0.098349914803804,-1.56655375368665
"Gm5417",1.1222743216496,-2.12748636639501,4.74580143820052,-0.4482881
11944629,0.653945279141829,NA,NA
"Tmem200a",128.963229240913,0.333225520283,0.333008626068443,1.0006513
1710586,0.316995411166276,0.462217807876163,0.257156483073711
"Zfp260",2116.54177474631,-0.166638532322587,0.136810909867406,-1.2180
2078857665,0.223216072943614,0.359268712458654,-0.170585310605315
"Chchd10",14122.0741678924,0.842013094963583,0.457194586767282,1.84169
524166343,0.0655197406257811,0.142610393248658,1.63993744940976
"Upk1b",3325.75270193139,-2.20758620043898,0.678223866887671,-3.254952
10094782,0.00113411491685015,0.00647524821339507,-11.1257230364139
"Cyp20a1",893.409780179177,-0.857569862491958,0.213793843761823,-4.011
19998313577,6.04109105314625e-05,0.000670018164699918,-6.2672969867589
6
"Aff4",3957.20420607002,-0.502650046422416,0.149215273046426,-3.368623
30618142,0.000755445975526289,0.00473318947005514,-2.69076411047652
"Ctu2",839.697980299938,-0.222582323669441,0.23515463427819,-0.9465359
86214602,0.343875276541887,0.488491701262753,-0.159465276452568
"Tmem67",456.045522424771,0.089347434918394,0.143785040197465,0.621395
903187772,0.534339166927718,0.66647703212575,0.0362526860134407
"Ccnq",657.780818117445,0.0320684180234445,0.171122160084528,0.1874007
31779004,0.85134644889058,0.905400903502742,0.00318687748287608
"Slc36a3",1.83400312095829,-2.18592747849448,1.49266667743709,-1.46444
448150187,0.143072541932925,0.258610218096613,-2.95632111393573
"Pls1",1463.49283094058,0.936777102454574,0.268885895224932,3.48392057
408191,0.000494126101308719,0.00342985785806956,5.31643156623658
"Dtx3l",1269.61893262774,-0.820445226348881,0.281052655418204,-2.91918
68873399,0.00350945756166952,0.0152569461621923,-3.43169298006942
"Proser1",971.105674622685,0.128306722143518,0.147775824406432,0.86825
2453734466,0.385256144643789,0.529852882010623,0.0814947705630255
"Htr1b",74.8607472933183,0.658397703562492,0.39517980695951,1.66607122
116933,0.0956992297704223,0.190791777307126,1.09068360666667
"Espnl",22.0206215144612,1.25866304641102,0.399535497873916,3.15031593
715416,0.00163093988386627,0.00858996916683537,5.98766166453413
"Spty2d1",1176.54356631786,-0.404971857024691,0.0870125993914186,-4.65
417491095698,3.25280595621865e-06,7.06271832231083e-05,-3.870759666468
74
"Rps23",9655.70963571696,-0.139631970000815,0.185347759819017,-0.75335
1268648506,0.451238858086543,0.591871378971728,-0.073232211416697
"Cdc42ep1",1771.86844449103,-0.562962454373409,0.148277548974192,-3.79
668033541204,0.000146646693330603,0.00134114316442781,-3.7235647989624
5
"Tmem202",23.7070707744523,-1.33428496609023,0.555832452278861,-2.4005
1648769299,0.0163719531832133,0.0493537945526118,-4.0145173833737

"Sall2",1049.94786521117,0.433795845033878,0.253170262105472,1.7134549
7463346,0.0866288699582956,0.176646068442465,0.752031583633273
"Tceal1",347.514254088982,-0.399026685329836,0.192886722143077,-2.0687
0997078716,0.0385733109964186,0.095348239825753,-0.937800259870835
"Tecrl",0.214384299096288,-1.10632709487695,4.77898884691053,-0.231498
153755299,0.816927819578957,NA,NA
"Adamts16",689.977818987721,0.826996963139414,0.388609811600916,2.1280
9079557852,0.0333295563235474,0.0852163243111103,2.03653151354233
"H1f1",0.97147109838154,0.83392087960913,1.64631268315712,0.5065385744
40141,0.612478617205491,NA,NA
"Clip1",2781.48100087271,0.0468588117141638,0.123022723949378,0.380895
579368291,0.703280732835715,0.802937564634663,0.0102844933046736
"Fzd9",9.83118790890467,-0.695591045914742,0.646149919501352,-1.076516
49396094,0.281696328316784,0.425589792080163,-0.594229049887085
"Polr1a",1249.96635692753,-0.169640888452333,0.124756999195458,-1.3597
7051024252,0.173902557165125,0.299748487729438,-0.204385298399618
"Tmie",744.035013349701,0.622356851497223,0.232989442646211,2.67118048
109269,0.00755849943969071,0.0273018936782307,2.24098206150824
"Lingo1",21.0634836924563,-1.36236470608123,0.499154614761514,-2.72934
41065994,0.00634604459934547,0.02386866851814,-5.08868914745609
"Defb20",0.302432356641651,-0.563484016601573,3.24811117862133,-0.1734
80520097451,0.862273738436692,NA,NA
"Olfr95",11.614386005667,0.575845507330697,0.519911499688784,1.1075837
0929551,0.268041642144302,0.410225740857897,0.513105805270583
"Ap5b1",274.523473037456,-0.0323300221271514,0.169069016582597,-0.1912
23813686507,0.848350247236102,0.903082002224851,-0.00329578449074616
"Aknad1",14.9232941805217,-4.07687139498141,1.29764210048004,-3.141753
33358347,0.00167939454037397,0.00879292017943383,-19.2991280812373
"Cfap46",20.8625375825832,-1.65204658491672,0.635858885918224,-2.59813
399089493,0.00937319107453102,0.0321871221506174,-5.67674403765131
"Zfa-ps",0.331402347483238,0,4.78471990595584,0,1,NA,NA
"Zfpm1",431.744859201381,-0.397138851728254,0.218228275855884,-1.81983
223837831,0.0687845566304201,0.148163657456229,-0.758311944287761
"Tsku",2183.73096509782,-1.79398476745055,0.644037216155474,-2.7855296
5954296,0.00534403507773046,0.0209274946667179,-6.93678556222595
"Grm5",0.350800973219525,-0.559908898211391,3.86945834187174,-0.144699
554496444,0.884948090916961,NA,NA
"Ccdc69",314.903841826251,-2.50707413388122,0.4992765051839,-5.0214142
0205179,5.12924148054259e-07,1.60787701587813e-05,-27.6731113155418
"Vsig8",18.9836068601401,0.45365535496189,0.398353140669841,1.13882710
752338,0.254775273121804,0.395447243697575,0.420873262743725
"Zbtb45",536.564571243146,-0.387789595353276,0.133145341258146,-2.9125
2845716485,0.00358515532018234,0.0154790430542328,-1.61641105059758
"Hoxb13",0.143365925116282,-0.0582793077263913,4.78471990596623,-0.012
1802966258737,0.990281769675646,NA,NA
"Zfp644",1573.50943988542,0.0734090769972442,0.314203039773932,0.23363
5795026241,0.815267736282361,0.88233183403827,0.00918986686893615
"Gpr55",62.43449066767,-1.79861090765053,0.684262798119023,-2.62853820
57811,0.00857527221876914,0.0299956779076628,-6.30719242650974
"Omg",1.90523728153842,0.181930744671667,1.32587132102626,0.1372159890

53031,0.890860073014569,0.932561768321665,0.0127023846116793
"Prss33",0.354174316968434,0.323809657020622,3.93656634613881,0.082256
8778342149,0.934442444667504,NA,NA
"Slc17a5",2580.78058569609,1.36772054054635,0.227558535994108,6.010411
93454402,1.85052511451215e-09,2.45452644100394e-07,20.8169279298246
"Tifab",725.311745266339,-1.22176941233492,0.402878138083146,-3.032602
90604989,0.0024245441463519 8,0.0115597306871762,-5.44936979175081
"C1ql3",85.5361721375916,-0.855084070720988,0.546465840421884,-1.56475
301376724,0.117640832742568,0.22302002496982,-1.28304827261602
"2310022A10Rik",630.067879824289,-0.085466520588777,0.131758430693153,
-0.648660735705151,0.516557688701444,0.650131797979651,-0.036800189009
2245
"Olfr273",0.21381255255021,-0.0699091834513599,4.78471990595817,-0.014
6109249497145,0.98834258333424,NA,NA
"Gpr3",10.6892423167711,0.728163429292408,0.700862522520588,1.03895329
810707,0.298826456779457,0.443216054639119,0.592505067717997
"Spatc1",5.54746351684579,0.864619647674988,0.858699156096891,1.006894
72155185,0.313985368840552,0.459133547446609,0.673032175205944
"Zbtb5",728.402165320176,-0.580007966852454,0.185293516838009,-3.13021
1875462,0.00174680263554742,0.00901751596184897,-2.73101761016583
"Bdp1",1325.66798719563,0.0385511415369568,0.250764305707073,0.1537345
65325218,0.877819029559227,0.923773558241121,0.00305665463793491
"Aftph",3619.28723241914,-0.00613206317641505,0.105505685820612,-0.058
1206892189791,0.953652494634386,0.971185435097736,-0.00017928837768063
"Morn4",486.339995105223,0.547963653847236,0.276037227469622,1.9851078
018364,0.0471324789695976,0.111244289426124,1.20334280913785
"Zbtb14",944.551611065107,-0.243852022933432,0.119157388459408,-2.0464
7001823561,0.040710149347349,0.0992536385845264,-0.563316876252168
"Olfr714",7.77061532617625,0.318783722545003,0.869654520744322,0.36656
3635260772,0.713944537593323,0.810320192909634,0.0670484445116977
"Catsperg1",142.846123288169,-0.374183865758113,0.293481044216715,-1.2
7498478396378,0.202314627736632,0.334384217310236,-0.409905177304057
"Urgcp",2107.73889832727,0.169170389454222,0.156684913593942,1.0796852
4584719,0.280282366227648,0.424099454967149,0.14511220952133 4
"Cyp2g1",0.222265746865732,3.19052280584978,4.69750997731396,0.6791944
71381224,0.497014649864957,NA,NA
"Orai1",787.684240227662,-0.032084262689224,0.241024975785187,-0.13311
5925371232,0.894101700608427,0.934584130811368,-0.00217061678925874
"Pheta2",88.0223987115932,-1.47763254003102,0.407657420776448,-3.62469
187293741,0.000289306168207832,0.00224736719829335,-9.01059714421696
"Nckap5",274.057888282847,0.343992286941161,0.311713317036312,1.103553
38748998,0.269786920598378,0.412120446398243,0.304928394434281
"Nkx3-2",0.531089081118391,-3.03962661685067,4.72997966898757,-0.64262
9953946776,0.520464241918946,NA,NA
"Ucn2",20.8914374436631,-1.36581546443175,0.64527787902702,-2.11663146
812221,0.0342911372582532,0.0871151262881331,-3.33330643728015
"Nlrp10",167.500902434406,-1.03502423614262,0.44576148966446,-2.321923
85421567,0.0202370361127161,0.0580041157120311,-2.94696376241155
"Lig4",687.387129167143,0.584335274132234,0.118562562141267,4.92849735
683005,8.28644554917363e-07,2.38654527795845e-05,6.21912627188121

"Prss46",3.65445177627552,-0.744769560311911,1.01739801549944,-0.73203
3627907467,0.464148042835536,0.603129526105117,-0.376572844681952
"Gal3st1",827.140613630691,1.64490920158491,0.359372560953481,4.577169
71273673,4.71309090134588e-06,9.46404185318661e-05,15.2407843457444
"Mmp12",1724.45008512579,-1.0380014657448,0.4821009091499,-2.153079253
83326,0.0313124504168905,0.0812199226058974,-2.60600098847576
"Zfp668",451.464154873364,0.166792094134654,0.175876971046646,0.948345
273073969,0.342953710164584,0.487526351763565,0.119825264315486
"Trex1",591.721825677859,-0.712656716627725,0.255503469331688,-2.78922
520501109,0.00528343116422479,0.0207610521644609,-2.7613142607578
"Olfr1361",0.0810182756847091,0,4.78471990596022,0,1,NA,NA
"Zfp646",1484.51684787006,-0.317343398280999,0.118911329901921,-2.6687
3979580201,0.00761364083630417,0.0274257277418193,-1.14125372899551
"Rxfp4",0.130758055495986,0,4.78471990596536,0,1,NA,NA
"Arhgap15",602.984795670084,-0.587018872711781,0.363518057098711,-1.61
482727267212,0.10634810531277,0.206305378309462,-0.926549290738419
"Rpl36al",5969.70922792964,0.466630732864508,0.175363294293242,2.66093
73115688,0.007792346357631,0.0278933668590213,1.67024235028165
"Zfp672",1282.57935858786,0.349785515513073,0.221775998856809,1.577201
84923578,0.114749087635999,0.218900079319706,0.531373137289504
"Olfr1318",0.818614868174449,-0.362958091046538,2.14083337299251,-0.16
9540560991529,0.865371471056361,NA,NA
"Micos13",3918.69721364919,0.419668758504421,0.384317711696707,1.09198
391261138,0.274840172315164,0.418201473372956,0.36586385324721
"Zfp280b",570.092446946185,-0.236264605103909,0.178877212536329,-1.320
8200293032,0.186561379610684,0.315269004996457,-0.272727090183155
"Tmsb4x",65510.8296409212,-1.13983552243774,0.24974259579413,-4.564041
30346005,5.01782001080441e-06,9.9630809135096e-05,-10.5024890856042
"Fzd4",4861.90522936406,0.0680904872937514,0.278253622928464,0.2447065
61507221,0.806683647137461,0.877006148119073,0.00893628245199682
"Bag5",1182.86708842915,-0.107639415684737,0.117106421327581,-0.919158
953578114,0.35801243707565,0.503079420294913,-0.0739490566055734
"Lrrc19",3217.94855922334,2.27827479425016,0.992573310004722,2.2953214
3498732,0.0217147100873374,0.0613791558832856,6.35794725362484
"Sertad2",1803.17881518783,-0.0232818038141607,0.154791261924776,-0.15
0407739588524,0.880442935425518,0.925356083276179,-0.00180612459387318
"Armcx4",695.657167863297,-0.764415785961277,0.202237767773782,-3.7797
8749654877,0.000156962262858389,0.00140839861997831,-5.01862045059096
"Olfr466",25.2159733065246,0.657757601502991,0.539305623048323,1.21963
794440922,0.222602155529565,0.358516853846169,0.67471433457896
"Arhgap23",1266.69693013957,-1.32880447393027,0.189867659217573,-6.998
58248322094,2.58565122815592e-12,1.48279493078189e-09,-27.013714041799
2
"Fam161a",448.524963997239,0.078871253694076,0.212759821690826,0.37070
5582789445,0.710856831696485,0.807944418794408,0.0168202422082139
"Zbtb12",482.947738844445,-0.425496103006725,0.199924875440855,-2.1282
7994549683,0.0333138798343578,0.0851985872259061,-1.04789921934824
"Ceacam19",18.4029881925071,-1.61420304839788,0.691947614291696,-2.332
83996513267,0.019656546247467,0.0566789912353018,-4.63333040254888
"Suox",2067.43710455652,0.502521943642518,0.4067350496255,1.2355019418

789,0.216643754198963,0.351922012777754,0.524806622224442
"Olfr250",3.86618489279301,0.597827684941877,0.73247140271069,0.816178
874328021,0.414397837608451,0.557736525001007,0.349052816753786
"Arl4c",2276.28878961876,-0.973143598875588,0.376621790260529,-2.58387
492184776,0.00976972175208779,0.033166969280104,-3.31472250452231
"Nlrc3",374.889664531741,-0.328098600189729,0.354413911966994,-0.92574
9777622398,0.354576032530001,0.499676458678083,-0.227632995296529
"Calhm5",221.616730782548,-1.47440496924905,0.29257792412405,-5.039358
22794314,4.67095483457098e-07,1.49793219349449e-05,-16.3789286709292
"Rlf",1205.34384408276,0.192144924050208,0.103372162015012,1.858768553
39742,0.0630599516357542,0.138590735894925,0.379722569989505
"2810025M15Rik",924.808212875331,0.572302569669136,0.232695287880737,2
.45945061836602,0.0139149839201343,0.0437462683771007,1.79093445351954
"Vcpkmt",408.219454066616,-0.0927445920423186,0.197815355692532,-0.468
844249818873,0.639180968158864,0.75319636903273,-0.026286535073271
"Rasd1",1010.22495314363,-1.05744743261588,0.436295432334651,-2.423695
85892155,0.015363466248191,0.0469749353537217,-3.23382346520731
"Olfr808",2.11922552205353,1.57540522957108,1.0952742209766,1.43836602
688081,0.150330226709043,0.268517658517124,2.07140360633492
"Stkld1",16.118045848535,0.509172286644093,0.413225029803019,1.2321912
9994815,0.217877632441046,0.353188694939162,0.529922492324129
"Tmem17",146.190166774001,-0.187223831224054,0.246593304785459,-0.7592
41340258374,0.447708200516586,0.588564335853014,-0.0992415558270835
"Rasl11b",2669.81231749626,-0.802586520901176,0.197972681971035,-4.054
0266106947,5.03434858099236e-05,0.000581514980048513,-5.97916831459297
"Gja8",0.111137623211519,0,4.78471990596623,0,1,NA,NA
"2610318N02Rik",24.7468768706032,-0.975729115711998,0.714979074528683,
-1.36469604562232,0.172348603032451,0.297660520728266,-1.1823901367966
9
"Slc35c1",814.855081733569,-0.193078563362237,0.194553415298885,-0.992
419295572979,0.320993030165562,0.466268501986749,-0.147317713214009
"Olfr921",1.81343398540895,1.21140193056338,1.27015926584143,0.9537401
82937513,0.340215174667381,0.484870658260696,0.876901280041327
"Glp2r",0.423996015782847,-2.10160252081108,4.68116106039955,-0.448948
988017026,0.653468455991377,NA,NA
"Lpar4",137.890573162328,-0.849962051041898,0.410474011320006,-2.07068
4203145,0.0383883174228653,0.0949867275521609,-2.0008260585168
"H2ax",848.786770234766,0.0780837855633035,0.215727915381746,0.3619549
44148645,0.717385700118945,0.812712008652559,0.0161928955984801
"Lrrc4",53.0126525820222,-1.87172102476262,0.595152201852359,-3.144945
13997773,0.00166117977575203,0.00871283027500865,-8.87749532265446
"Pgrmc2",3864.12697113779,-0.638709740412919,0.3560934180879,-1.793657
80991564,0.0728678032461881,0.154853016642417,-1.19137179670959
"Rpp38",271.332664209286,0.0188085737413,0.21014064756684,0.0895046910
680025,0.928680825615709,0.956091596063947,0.000844534278623366
"Ccdc137",1266.59288913785,0.130396194820351,0.161102439948248,0.80939
9254674472,0.418285530784283,0.561310141756832,0.0753014148059704
"Mrps16",1546.47378169462,0.158505748592444,0.229043534615372,0.692033
280304632,0.488916429372007,0.625476841407741,0.0743773918365061
"Plekhf2",1945.63047882913,0.185746788365964,0.160988958211343,1.15378

588960194,0.248587960537172,0.388069411754362,0.175822534185164
"Glt1d1",702.792463057768,1.49755296271048,0.521976603640958,2.8690039
9800404,0.00411766593822652,0.0171735295109178,6.08663338954877
"Ankrd55",17.1545967523977,-0.408437332743252,0.467590701077516,-0.873
493274785081,0.382394270305713,0.527258983779881,-0.261425796390635
"Lrrc25",390.257887032148,-1.37187162405207,0.39292744689571,-3.491412
05301495,0.000480474619038645,0.00335300433858823,-7.81678529296437
"Ppp1r3d",258.905746409451,-2.2768501111953,0.506347540138376,-4.49661
532980504,6.90437361742051e-06,0.0001268816934896,-20.4284808258164
"Idnk",2001.53341814307,0.186387375824589,0.143371374232133,1.30003201
003576,0.193589998369969,0.323806964760887,0.210171845477792
"Shisa3",1232.70432980284,-0.953911005204985,0.269465366445253,-3.5400
1338943418,0.000400106732314209,0.00289472395794525,-5.57548154711286
"Apol10b",143.342767392186,-1.65036982171437,0.481152506091869,-3.4300
3476199136,0.00060350392718813,0.00398075763610086,-9.12041088250145
"Pitpnb",3858.22369829659,-0.0382958253518422,0.138823242905259,-0.275
860328215914,0.782655345476893,0.859488252667331,-0.00579868195093006
"Amz1",522.348390258567,-0.648681761109926,0.31825113786348,-2.0382700
4504911,0.0415229338600084,0.100723334710431,-1.48896970143878
"Rap2c",1467.43223309336,-0.298827784442386,0.151752006144378,-1.96918
506736628,0.0489318430164271,0.114442583828658,-0.647763619307319
"Olfr728",0.0868602420236823,1.37431944116025,4.78471990596623,0.28723
0907591176,0.773935522099994,NA,NA
"Fhl4",7.11065132778443,-1.42576188884566,0.609544451158828,-2.3390613
861468,0.0193322567349938,0.0559923264733972,-4.10981656602727
"Tmx2",4534.59508292256,0.434362821373346,0.239646656767016,1.81251358
659943,0.0699068765287133,0.149933371307541,0.824231372161599
"Ccdc158",88.7999880116552,0.303006102758817,0.515827571055166,0.58741
7423498699,0.556923394819573,0.686316037934018,0.114056666558751
"Tdrp",1625.6295874821,-0.238814411530673,0.32826132629101,-0.72751308
9126311,0.466911699988564,0.605469827505788,-0.119825261841349
"Prm3",0.145842946244176,2.09870209063446,4.75617210313392,0.441258651
942303,0.659025756395086,NA,NA
"Klk6",0.329796437349633,1.08119187501631,4.76049911119475,0.227117335
758721,0.820332504268121,NA,NA
"Zfp697",1880.17471994081,1.13587881096869,0.276980605414339,4.1009326
6013882,4.11488302078714e-05,0.000498645053693646,8.63678633585571
"Grem2",135.293051852182,-1.11662818444975,0.70167773149354,-1.5913689
9224801,0.111526558366646,0.214102370413237,-1.72106015063529
"Bex1",177.931436629569,-0.330607746283724,0.269236406971286,-1.227945
91564648,0.219467271508406,0.355206512814054,-0.342197509090699
"Spink8",593.479224116545,-0.26171200205692,0.689213114676429,-0.37972
5801038752,0.704148965769847,0.80365361648451,-0.0572068225249821
"Gpr171",105.604787715808,-1.10014594030316,0.474532649481036,-2.31837
775863538,0.020428799739342,0.0584133652027703,-3.12464611622172
"Rspry1",1678.01595716041,0.182596530876214,0.0921481163843648,1.98155
467567643,0.0475291042245093,0.111950045200469,0.39983208602722
"Cby3",13.3840895040441,1.18119661413522,0.669410723879641,1.764531956
23977,0.0776424600334431,0.162519880379181,2.14618102777142
"1600012H06Rik",1717.58140107987,-0.0604835625320786,0.110791272969342

,-0.545923527287347,0.585118505100303,0.708963498163417,-0.0208033961707213
"Akap4",0.215713028821551,0,4.78471990595633,0,1,NA,NA
"Sprr2b",0.287025600503569,-1.0809510848998,4.77945885888876,-0.22616599845597,0.821072314766984,NA,NA
"Ces2b",525.586197331891,1.30717508646741,0.26846728033515,4.86902942077543,1.121477115545e-06,3.01607735157191e-05,13.6063441265821
"Hmx2",407.733874397452,2.00154228421723,0.73213406424657,2.73384668459184,0.00625991847346443,0.0236276808999876,7.49644904270184
"Agmo",289.8945767841,-1.88898727175406,0.694225587663774,-2.72099920446743,0.00650849181568462,0.0243341463896872,-7.0192400377731
"Grrp1",651.745744919512,-0.575977032325736,0.214575595395894,-2.68426160609297,0.0072690202890805,0.0265414527334254,-2.09024801732285
"Tmc8",204.991814275456,-1.55559623586528,0.308607598138339,-5.04069324685895,4.63848466350619e-07,1.49078889875411e-05,-17.2883054744252
"Haspin",142.219244265458,0.741993626507253,0.328019481372672,2.26204133791753,0.0236948494010936,0.0655787329485482,2.02156447610559
"Bpifc",79.9355771709191,-1.58164064457437,0.670341978385506,-2.35945337689234,0.0183018804782202,0.053645530000652,-4.62686373854598
"Prdx6b",0.730260256044642,-2.19712701175233,2.12727263745241,-1.03283752776681,0.301679938327918,NA,NA
"Vwa3b",11.0650149039094,0.180140496961827,0.768511979952797,0.234401677086272,0.814673156784686,0.882031051751225,0.0226126794601118
"Olfr1023",0.114518533427629,-0.0634343222640453,4.78471990596623,-0.0132576877039232,0.989422205539938,NA,NA
"Sarm1",23.9257538501229,0.241181103368724,0.369078854010267,0.653467682442775,0.513454817430674,0.647354803120807,0.104880196181458
"Kcnk12",8.56240951371405,-0.912896141137978,0.912862483615222,-1.00003687031,0.317292665120607,0.462407628627794,-0.704124520287211
"Fam205c",0.150840222250025,0.0236836517937881,4.7847199059555,0.00494985124715645,0.996050606238997,NA,NA
"Slc25a44",1743.66891542339,-0.728855620392602,0.402662838532263,-1.81008911338662,0.0702819689542389,0.150522608816976,-1.38019160113895
"F2rl3",60.6294202518272,-0.873058632720006,0.332946344413345,-2.62222020865958,0.00873589650033436,0.0304654820181577,-3.04798822602144
"Ubqln2",1507.75767655611,-0.423276405687623,0.257498552549746,-1.643801106827,0.100217292672768,0.197685734766119,-0.686163522040308
"Slc9b1",4.86211726889163,0.57103827393084,0.901748071654157,0.633256994809353,0.526565831928186,0.659026933110968,0.238117749874454
"Gm867",83.7941160605321,-0.196412436777808,0.412132253631201,-0.476576232622573,0.633663911168344,0.748707971152611,-0.056842989362968
"Olfr165",0.181588266188855,0.00835216785474959,4.78471990596461,0.00174559180451458,0.998607219957035,NA,NA
"Mchr1",29.2585782105488,1.95392244337545,0.540653132313206,3.61400374213225,0.00030150470606919,0.00231720092981358,11.8552198836472
"Nudt6",490.231302929601,0.947477466425908,0.182814837548924,5.18271645304692,2.18677355074732e-07,8.2456588118601e-06,11.0910041824007
"Lsm10",567.627961993684,0.00194139944779943,0.181572694054478,0.0106921332963036,0.991469074466148,0.994470947578839,1.07638838537819e-05
"Eif5a2",308.262226534655,-0.0889612090874983,0.285334569086553,-0.311

778588105506,0.755208797723946,0.841054217842108,-0.0153991099237779
"Scd4",46.2642722029018,-0.983587541790132,0.246339986350852,-3.992805
05110222,6.52962498443959e-05,0.000711569972138382,-7.12907869963684
"Lgr4",5221.55295253682,0.974130840292435,0.248319951940979,3.92288590
859573,8.74945903474618e-05,0.00089016462292256,6.84255862051107
"Otop2",0.194214036749975,-0.0531768805041202,4.78471990596091,-0.0111
13896225748,0.991132576340244,NA,NA
"Pla2g4e",1.00427364490631,-0.688230587986069,2.48225008205024,-0.2772
60777615774,0.781579874281131,NA,NA
"Eva1b",2229.41247983371,0.115071945697139,0.332577975309369,0.3459998
98490264,0.72934279102471,0.822340006904516,0.022508226285137
"Snip1",794.762047304576,-0.111799939513739,0.132711624944656,-0.84242
7628780543,0.399548633330995,0.543794074272258,-0.0681068063218499
"Lgsn",0.645956181796341,2.36859390175738,2.81403853223442,0.841706278
938779,0.399952382776304,NA,NA
"Il17d",60.2800557747032,-1.55135566233303,0.281225034777494,-5.516420
91025239,3.45973078775346e-08,2.02247398202931e-06,-20.3401173762958
"Pigm",1464.11691059458,-0.180508392007511,0.0994420899357468,-1.81521
116585688,0.0694914644201041,0.149272289662134,-0.343323927864193
"Cxcr3",309.934014925488,-1.63253272317242,0.46099318508622,-3.5413380
8478553,0.000398103113522032,0.00288236706552268,-9.54891804328027
"Gja4",448.948772467066,-0.3147495522786,0.207874296857947,-1.51413405
618727,0.12999184262964,0.240062601590522,-0.449102145431939
"Hic2",376.516059233148,-0.408264776676537,0.235662927382212,-1.732409
84999898,0.0832006154550663,0.171213255951755,-0.720524208464696
"Klre1",39.0146654813091,-2.1126344263592,0.371618220109002,-5.6849592
1900581,1.30843878655722e-08,9.81228440780486e-07,-29.2271576053703
"Heatr1",1212.53967011204,-0.0560473567436386,0.112394666135322,-0.498
665627745699,0.618014963085036,0.736429065959554,-0.0171472605973043
"Evc2",1887.93316553289,0.676887429892787,0.319862254655314,2.11618413
877,0.0343291496730012,0.0871776192138809,1.65147512382097
"Olfr690",0.131497130174101,-0.152464228627251,4.78471990596527,-0.031
8648179253228,0.974579855611723,NA,NA
"Tmem220",323.184491654039,0.363560667245726,0.301842750402164,1.20447
042959068,0.228407804623834,0.364891058955798,0.366526027492591
"Prag1",564.846295683816,-1.04383984025915,0.334230097087277,-3.123117
42525861,0.00178946338281571,0.00918183079951073,-4.89616068795369
"Dscam",13.608797012864,-1.80046208017473,0.987904829110623,-1.8225055
9681505,0.0683783052927111,0.147466010020715,-3.44636812079607
"Mrgprg",1.18909127530685,-3.78718363676327,2.43703360284696,-1.554013
71254752,0.120181145774172,NA,NA
"Fzd2",567.445108030388,-1.09787325682687,0.301436717806506,-3.6421351
2148046,0.000270386075576744,0.00213209003980534,-6.75263693681591
"Foxc1",1254.44471384121,0.160729725476877,0.234472118196562,0.6854961
12344304,0.493030913021187,0.629342315887709,0.0744307129030288
"Abca12",0.481186282181953,-2.17224681974577,3.74717154363577,-0.57970
3062549972,0.562114877743886,NA,NA
"Gm9843",332.221295305652,0.0321012110399829,0.704883410331731,0.04554
11640697794,0.963675964735326,0.978160984057015,0.000708827390483259
"Slc25a2",15.213266114269,0.191236278293605,0.405715996885426,0.471355

035940598,0.637387219764079,0.751786099507593,0.0545603675982237
"Rictor",1929.65580681699,0.140990328052579,0.15698543097898,0.8981109
08594168,0.369126423909485,0.514124366982438,0.0937994671488133
"Nsun3",586.988566416363,0.0282032628920386,0.158548119373256,0.177884
562765718,0.858813634618746,0.910656311061361,0.0026395354342702
"Synpo2",830.055668221559,-1.24183212144199,0.327378060209628,-3.79326
617259207,0.000148678634853531,0.00135401028602249,-8.20190937371911
"Neto1",1.56139207089876,-1.05598207299877,1.89054307329634,-0.5585601
76657368,0.576461924770372,0.701920602583696,-0.373748997145343
"Ndufaf6",645.248897308813,0.922189831133026,0.24884453355488,3.705887
43887214,0.000210651843915375,0.00176075451034255,5.84853977093276
"Hoxc12",0.100532412451372,0,4.78471990596383,0,1,NA,NA
"Amer1",854.7378434172,-0.0219969619217901,0.187945409789342,-0.117039
10165428,0.906829067756256,0.943050351856775,-0.00128980511281223
"Lgals3",6748.93411808778,-0.396290314493585,0.162555119128809,-2.4378
827109072,0.0147735665991132,0.0457157596491842,-1.22267933825939
"4930486L24Rik",28.7121354467187,-0.365172476252979,1.00334642814933,-
0.363954528573483,0.715891964956183,0.811632837115691,-0.0762141294618
623
"Gpr18",72.2175658934375,-1.51116487979919,0.519336310004847,-2.909800
16356854,0.00361659918167896,0.0155827258775188,-6.28885211842914
"Carmil2",308.012258374949,-1.05769813411599,0.324041948658385,-3.2640
7780997221,0.00109821000711704,0.00632021803977807,-5.35618501328691
"Sprr1a",5800.12249249982,-2.72615026359797,0.727717040945436,-3.74616
796118477,0.000179556394109263,0.00155599580992996,-17.6263049528663
"Olfr524",0.0843067902683353,0,4.78471990595952,0,1,NA,NA
"Hoxd10",1073.90040886214,-0.00406796747225752,0.34164049421707,-0.011
9071583758828,0.990499686662449,0.993710465168224,-2.56664229188108e-0
5
"Ch25h",305.662149481955,-0.448743452522805,0.43397618368744,-1.034027
83237985,0.301123151957699,0.445842449454872,-0.362490312734772
"Snx21",916.017590164682,0.210270837748132,0.132349559462904,1.5887535
9012486,0.112116034815625,0.214970837529262,0.32323945499875
"Il31ra",15.7314461025768,-0.958880744097922,0.472583007961094,-2.0290
2078141765,0.0424561736814723,0.102569521611898,-2.18357708694769
"Sept6",863.85732013252,-0.59542584249057,0.219124830147187,-2.7172906
0595561,0.00658187883917218,0.0245661320073084,-2.20687832359164
"Kif7",303.169575855598,-0.997218690830779,0.218335708167156,-4.567364
17144976,4.93895514294289e-06,9.85667833951898e-05,-9.19911928078718
"C77080",2590.62614493662,0.0933147410354391,0.190563349494368,0.48967
8321057204,0.624361544750043,0.741734001995496,0.0278791401563817
"Armcx6",165.591249342782,-0.736220933702612,0.252183336229749,-2.9193
8771494356,0.00350719717400063,0.0152569461621923,-3.07940631358255
"Tnfsf15",220.946480842076,-0.302842785477074,0.510589890571858,-0.593
123348247048,0.553098605967178,0.682767750500033,-0.115564964546793
"Foxl2",0.526306838582215,2.03508863147303,3.53163705449308,0.57624512
3740535,0.564449509053179,NA,NA
"Tcf19",386.389838391168,-0.465311332894264,0.244595030494002,-1.90237
443481369,0.0571222208004802,0.128804100979272,-0.953638186182689
"Ppp1r3g",11.7335240109672,-3.77151732265344,1.73633217107932,-2.17211

740096311,0.0298468087847287,0.078356412775635,-9.6041215888942
"Mrgprb2",2.26208311668259,-3.84446932622433,1.60019816692736,-2.40249
576938732,0.0162836229129868,0.0491688311710717,-11.581446041594
"Fbxo46",617.423059130698,-0.278720958700765,0.120251153637846,-2.3178
2357398561,0.020458911363679,0.0584723037706221,-0.791345124754879
"Enthd1",0.348200537122973,-1.99248749130102,3.10046017829563,-0.64264
2503602907,0.520456096861855,NA,NA
"Hamp",5.79094959359163,0.746410495883702,1.15310563205064,0.647304527
128456,0.517434872135974,0.650899686252078,0.32050847318341
"Cyp8b1",24.6146034997134,-1.02384515405048,0.654227682477869,-1.56496
764272141,0.117590496493784,0.222986542164932,-1.53642694235723
"Lypd6",168.444845910807,-0.133033626387893,0.392798824392138,-0.33868
1325214668,0.734849810414384,0.82663714322158,-0.0253281980247804
"Krt78",20.8485401419771,-3.13436082597352,1.04511369761053,-2.9990620
4764104,0.00270812149517992,0.012539290646644,-13.7250159884227
"Astl",0.415240773074194,1.07645399727758,3.76818735165796,0.285668916
330276,0.775131714167977,NA,NA
"Fam118b",688.848226185339,0.140080502985176,0.16815458792119,0.833045
977019837,0.404818799639846,0.54859306056281,0.0841041028476368
"Slc35d3",6.22068660378322,-0.064689391451865,0.721932529814768,-0.089
6058686654041,0.928600420650094,0.956091596063947,-0.0029046545100027
"Gm8394",0.148177164577155,2.59891947282789,4.7275994170606,0.54973343
6265582,0.582502219767124,NA,NA
"Fam167b",503.999955233777,0.212868535777234,0.334326648013795,0.63670
8252368954,0.524314897211269,0.657179986232021,0.0893616464446341
"Fbxl22",173.664935120458,-0.125288179609666,0.310984593624936,-0.4028
758407266,0.68703956499914,0.789937666544033,-0.0295431080770907
"Pcdh20",6.51348774555798,-0.0781214603959958,0.713339977012,-0.109515
045999843,0.912793988564676,0.946353538447837,-0.00430754392772102
"Oprd1",8.53989560652076,-1.79194173218315,0.857809484080044,-2.088974
02679677,0.0367100612749762,0.0917892760630271,-4.27962241485876
"Cldn8",2706.98840267382,0.123383333232706,0.441251641653505,0.2796212
44626651,0.779768106179344,0.857497366018682,0.0189686046793399
"Fam171a1",2144.16083683977,-0.15770763200161,0.156082384788739,-1.010
41275231072,0.312297580723258,0.457374760062674,-0.123367138973653
"Htr5b",0.255434488157135,-0.54058796329193,4.78471990595381,-0.112982
154424391,0.910044703320519,NA,NA
"B230217C12Rik",68.1564552385753,-0.802821889182532,0.281354782547235,
-2.85341475952253,0.00432521297056257,0.0178030404264363,-3.2340764795
8653
"Adra1b",133.139463800616,-1.16079779854257,0.337912815818718,-3.43519
909338187,0.000592118638376655,0.00392062018550818,-6.43256730039511
"Fam228b",59.6440356034726,0.182264150111847,0.362874717696442,0.50227
8448244822,0.615471667083314,0.734647478713833,0.0562037963644315
"Fam241a",683.00667169969,-0.529228493480361,0.279530658992376,-1.8932
7530435506,0.0583212675429344,0.130751762050378,-1.0766913957462
"Gm11868",0.855890151280042,2.87358506246409,2.4369930494626,1.1791519
3196705,0.238337682606151,NA,NA
"Lamtor4",4046.01335657805,0.849666943138788,0.211488593066882,4.01755
447335208,5.88052442876916e-05,0.000656692241192103,6.2266100787715

"Hyls1",237.780649769548,-0.163709548034648,0.193030782476784,-0.84810
0732608994,0.396381873666425,0.540843511039045,-0.10062003018611
"Kcnb1",1026.13829808629,-1.95956710203277,0.5182285945502,-3.78127938
643291,0.000156024452659634,0.00140256559610767,-12.8732823131211
"Prokr2",0.809787295315729,0.127764136019188,2.54068611114459,0.050287
2572329015,0.959893478177851,NA,NA
"Tor1aip2",5148.10168054494,-0.0203464311954697,0.129034866374431,-0.1
57681654324568,0.874707657357665,0.921844759913505,-0.0016557608871771
7
"Maml1",1107.59483967799,-0.607368184265361,0.123880437454852,-4.90285
792288001,9.44523006172132e-07,2.65364691272972e-05,-6.39983283472328
"Mmp13",94.9621808262203,-0.409585019420956,0.488137650034152,-0.83907
6886186304,0.401426165657184,0.545436054214898,-0.248278030292823
"Lrrc4c",12.2869486005962,-1.0484006580549,0.869320081369952,-1.206000
73611867,0.227817206598485,0.364237436612871,-1.05883153757426
"Fam78a",319.763239615185,-0.776726562841407,0.300978278195985,-2.5806
7315520901,0.00986078911409049,0.0333968501209895,-2.64032170485546
"Zfp831",85.8243642211958,-1.22435183086846,0.493035618441925,-2.48329
285972809,0.0130174017990311,0.0414389061677563,-3.89776700009156
"Zfp61",455.617168996239,-0.0346638995427585,0.209344761312152,-0.1655
82837256058,0.868485254715097,0.917552810997049,-0.00298266012515643
"Micos10",7973.29080571807,0.726101740961271,0.261957254131469,2.77183
292124776,0.00557416391838602,0.0215303185579815,2.78699135429883
"Txndc2",1.33238399932436,0.90804137495003,1.27331817434968,0.71312998
8436545,0.47576532423596,0.613361034908275,0.443852033769704
"Olfr125",0.347113022069089,-0.0671103590616331,3.13540539561152,-0.02
14040452808955,0.982923346638448,NA,NA
"Zscan29",1624.78926588009,0.359939437705691,0.099366517375249,3.62234
127967282,0.000291948596438634,0.00226338795330315,2.19235241935725
"Rps27rt",4889.45165988466,-1.51189213901895,0.6184868835435,-2.444501
53955871,0.0145052461702612,0.0451577981522836,-4.68322544444839
"Catsperz",30.2170886639964,-0.757097032521632,0.509692974428117,-1.48
539821128809,0.137438369224722,0.250750755417202,-1.04728917519535
"Ccdc121",2.2434787651262,0.771364257250795,1.11405932375782,0.6923906
48146917,0.488692037138113,0.625312858650671,0.362157972218937
"Gpd1l",2019.3368312413,0.141026345086177,0.156533416855458,0.90093443
252695,0.367623182384832,0.512653183388603,0.0942275588879842
"Ubald2",3325.54612979151,0.272806414383142,0.128074845730954,2.130054
60069985,0.0331671058468269,0.0849346243500697,0.672706092502151
"Sprr2f",240.072968186096,-2.62363251381048,0.582657370580322,-4.50287
363772188,6.70407481690631e-06,0.000123901469933686,-23.6022608135596
"Tmem150c",1205.06218747489,-0.234066944510437,0.438261442276371,-0.53
4080623872981,0.593285754708404,0.716056059752674,-0.0781776149796998
"Defb19",25.7259833987527,1.50764916112763,0.71365202216657,2.11258304
369484,0.0346364712873346,0.0877523281133641,3.66846754967355
"Mrgpra1",0.108229817038987,-0.909962624367568,4.78339755770097,-0.190
233534509919,0.849126134161093,NA,NA
"Trhde",27.1664423257255,-2.01588508642529,0.705035906565221,-2.859265
8439855,0.00424622771468794,0.0175483145360291,-8.14981492867702
"Vstm4",1033.83293955836,-1.40680240831628,0.333445615162836,-4.218986

07852221,2.45403423756111e-05,0.000336489167116502,-11.2501209255699
"Gpatch11",1197.09885416905,-0.529387527872224,0.275936389552005,-1.91851291789281,0.0550460057359006,0.125048004175532,-1.10062715363768
"Ism2",4.7136485790353,0.663688431311451,0.764440099166478,0.868202010903297,0.385283753594544,0.529853479163945,0.421544869665442
"Gp1ba",1.8786675089855,-0.123929552981283,1.37130514751297,-0.0903734323509575,0.927990468197022,0.955734108858311,-0.00561097661835239
"Ccdc96",58.656753566102,-0.159228241821787,0.340275072102564,-0.467939778362,0.639827656078878,0.753570500647899,-0.0450508765439373
"Prkaa1",1797.63077477837,-0.46593723336302,0.159605706603852,-2.91930184250552,0.00350816353732596,0.0152569461621923,-1.94888516811835
"Emilin3",1.95029052036657,-2.03989001808288,1.5890815802972,-1.28369118576113,0.199250029706465,0.330678215135204,-2.25736174675093
"2310061I04Rik",2729.46952185075,0.990038183347179,0.228489087082676,4.33297798152149,1.47105827357412e-05,0.000227821240811343,8.30340001716764
"Ftl1",117483.924895955,0.524542400510223,0.203403203088289,2.57883058155452,0.00991353934736935,0.0335427619037641,1.78078692404357
"Scg2",3.52303535995374,-0.569570737450601,4.58075857010106,-0.124339829033608,0.901046212963581,0.939396805708689,-0.0356080279381465
"Zbtb26",511.332418609048,-0.21885733677777,0.124418232540965,-1.7590455378452,0.0785697742102173,0.164003153575722,-0.395665530761063
"Plekho2",961.25504669594,-1.66358656513627,0.256866308846323,-6.47646852795924,9.38941895856583e-11,2.26018383770514e-08,-29.287831739762
"Arhgap42",2113.46577021092,0.887698334209815,0.206149205881631,4.30609630734897,1.66160738133696e-05,0.000248719721517747,7.3671717338733
"Vamp8",9148.38676998876,0.854452546887942,0.301312073447779,2.83577268282291,0.00457149711862751,0.0185389040804907,3.40745749666898
"Ptges",1901.25431839149,-1.21916155227708,0.485277313725469,-2.51229867499386,0.011994752331191,0.0389594670920477,-3.95646388531648
"Trim15",5.81530532548289,1.22066711205826,0.689854128097278,1.76945684941401,0.0768176657142515,0.161035463508921,2.22909764876304
"Pgbd5",58.1967856995505,0.609979933911631,0.345410607626173,1.76595599684591,0.0774032315040723,0.162053925466166,1.11005742000215
"Defb6",0.348597219379843,-2.03662884031691,4.74788653358629,-0.428954825670308,0.667956100161241,NA,NA
"Gp1bb",69.4264089363919,-0.754041145214165,0.463452698904161,-1.62700777662338,0.10373544616481,0.202851642695965,-1.20290705317871
"Prss27",65.7006244231874,-2.32404231517136,0.686486899024471,-3.38541393648433,0.000710709920998654,0.00452229565346252,-12.5468898111572
"Olfr1395",0.103069607447589,-0.492459626968356,4.7813719837464,-0.102995464197808,0.917966570855388,NA,NA
"Olfr1124",25.6563819722926,1.28900257035659,0.554844112355111,2.32317968534486,0.0201695016206345,0.0578634010065126,3.67323221406144
"Tmem37",1592.75342388391,1.39468645727225,0.298909011236465,4.66592308978241,3.0723461219743e-06,6.76124206391139e-05,13.3913872529458
"Ccdc126",239.885892230298,-0.313345136941242,0.141362474619009,-2.2166076095212,0.0266499171290469,0.0719546375235833,-0.824636473564132
"B3galt6",305.352922384614,-0.292972683639335,0.125683333566384,-2.33103845455046,0.019751332066761,0.0568850033438265,-0.839871687069027

"Muc15",1.6272668168544,-4.1649953364431,1.83366943625887,-2.2713992250101,0.0231228212904444,0.0643885703400579,-11.4238289302004
"Ecpas",4394.28500894464,-0.039794251694398,0.170459499735208,-0.233452824607689,0.815409798010199,0.882387625796584,-0.00497920936268175
"Olfr1441",0.384917032975341,1.04823512401209,3.72985545538505,0.281039074181461,0.778680430200186,NA,NA
"Fam131a",277.916343219309,-0.642944540217386,0.193925336880959,-3.31542309302294,0.000915045156075051,0.00550166254942486,-3.34505074241968
"Slc29a4",16.8308396067259,-0.428290571926704,0.364598634857522,-1.17469055278847,0.240118540324488,0.37845212992053,-0.416155252906325
"Sstr5",0.155668627108197,-1.28611468659492,4.77305157651793,-0.269453339436399,0.787580844435911,NA,NA
"Vwc2",11.3010024300715,-2.26964420501419,0.945464485790293,-2.40055997779446,0.016370007817272,0.0493537945526118,-6.82877072489377
"Cdh20",4.36029030217178,-1.816854679563,0.901984435747938,-2.01428606476612,0.0439795081758975,0.105462114181976,-4.08683927127658
"1700020N01Rik",5.42657158205183,2.68894053221586,0.89382542669912,3.00835090599969,0.00262669651371262,0.0122671445806871,11.8335722136557
"Zfp623",762.449877997333,0.311795534744307,0.123770751653931,2.5191374422295,0.0117642725544627,0.0384622787809818,1.01585395084303
"Tmem125",450.927164630319,0.75108326806911,0.325725522259316,2.3058778534129,0.0211174584345458,0.0599953671217797,2.11316371242582
"Zfp940",265.318915750093,-0.0893442669542571,0.237115282351919,-0.376796746578549,0.706324658709004,0.804951674294708,-0.0193852967875044
"Atp5k",4104.7638101795,0.479775489857468,0.411031839930366,1.16724653238238,0.243110824160321,0.381964129692018,0.461749641558095
"Phospho1",940.075117876298,-2.92041150086343,0.726590044967835,-4.01933871939123,5.83617164226332e-05,0.000652486666747994,-21.4204002145429
"Clrn3",1442.57520574316,1.64732194868063,0.59255397883275,2.7800369376063,0.005435271564582,0.0211699810160247,6.35070795342368
"Minar2",72.7804390985189,-0.55523461460111,0.468812663658983,-1.18434218535741,0.236277636105048,0.374395883687625,-0.545485543646494
"Spata31d1a",136.239574929236,0.298698958306082,0.364236734577345,0.820068186292872,0.412177237113094,0.555668379259567,0.175510610090043
"Pdik1l",748.967869098044,0.0783431122421698,0.10525041347765,0.744349686177773,0.456664949371181,0.596666433213113,0.0404561528392534
"Tatdn1",654.989652121255,0.114841771264391,0.187827975471513,0.611419949430319,0.540921598937767,0.671434258727309,0.0457459758387768
"Rtn4rl2",84.3781378402676,-2.16577897032563,0.307453486237725,-7.04424918652909,1.86464190408136e-12,1.15857520300001e-09,-44.5632302237418
"Gm7327",0.149584872585184,0.86399405532736,4.78471990596623,0.180573590995372,0.856702286420036,NA,NA
"Tvp23a",111.098101389531,-1.39110913474157,0.329706802853925,-4.21923091273883,2.45137105478202e-05,0.000336452107768327,-11.1247759048779
"Cdr2l",498.228889990381,-0.767344068042921,0.309854554956094,-2.47646534727124,0.0132690505182252,0.0421093663744067,-2.43055088969642
"Tmem123",5743.71014592602,-1.25462497069126,0.270882793386302,-4.631615596573,3.62823328665128e-06,7.76545473360337e-05,-11.8728177698748
"Ankrd37",78.9819240279001,0.619536590165696,0.360322011303259,1.71939

701359036,0.08554210566082,0.175015737269115,1.07977754930129
"Fgf4",0.419745230551339,-0.353375681421089,3.18277239654227,-0.111027
631697759,0.911594435263831,NA,NA
"Zfp366",538.506500069839,-1.04234299742288,0.280210849040176,-3.71985
239327201,0.000199339242856194,0.0016921583684 6123,-6.65197286554512
"P2ry10",226.569070399736,-1.46697362191396,0.41171812833291,-3.563053
26620884,0.00036656626225954,0.00270628889872643,-8.6730076963092
"Dcaf12l2",3.40068643028362,-1.12884662320361,1.0703191002458,-1.05468
231198002,0.291570644068379,0.435335356309461,-0.938792437601515
"Map10",223.173982944761,0.180735113181994,0.208392792696894,0.8672810
16982543,0.385788055146561,0.530107436237116,0.114708163344973
"Sgms2",2962.7942332294,1.16616153856112,0.343399920261568,3.395928390
64394,0.000683962509429774,0.00438536698745865,6.33165304201931
"Efcab5",70.0282291266286,0.180201679757477,0.685764976426198,0.262774
69096859,0.792724243862042,0.866852053820893,0.0257484698940516
"Zfp438",215.737666738508,-0.527658631452492,0.225434869810283,-2.3406
2561792924,0.019251460900963,0.055816354594346,-1.52265837054557
"Amigo1",1063.96531128478,0.441567400011551,0.188424986071049,2.343465
21243764,0.0191055438475316,0.0554948666150903,1.27677657450075
"Gja1",1297.60395708294,-2.78240063166143,0.623640519320979,-4.4615456
2678337,8.13706161411169e-06,0.000144627554559662,-24.6001742334189
"Zfp169",241.739647658543,0.0735789703762182,0.262457488223748,0.28034
6241496799,0.779211876936366,0.857300145384565,0.0113287475851334
"Insl6",100.011902468676,0.0489154011465225,0.338947938927149,0.144315
381593266,0.885251432697115,0.928340366521182,0.00363719461611818
"Kcns2",0.220934797106264,0,4.78471990595636,0,1,NA,NA
"Prkca",969.326946253242,-0.290400596966495,0.209382363100663,-1.38693
91512545,0.16546031817212,0.288210075577978,-0.361277403261193
"Lin28a",2.20946842269266,0,3.74607156181331,0,1,1,0
"Creg2",57.5259532660938,-0.85386366538906,0.591870351772349,-1.442653
21422534,0.149118184910261,0.26682754141047,-1.12808432247579
"Gdpgp1",151.653456996611,-0.0754262053551585,0.185806531835727,-0.405
939471610413,0.684787074888842,0.788331958799235,-0.0179390677553071
"Gm9847",0.136471251534351,1.35377179245804,4.78471990596487,0.2829364
76756009,0.777225532132816,NA,NA
"Apol10a",1.02316286485731,-2.8637795448797,1.71820805848602,-1.666724
54522364,0.0955691899145298,NA,NA
"Selenon",1297.07139565888,-1.18716096226742,0.195868884221503,-6.0609
9823862217,1.35279314126896e-09,2.0289815898817e-07,-18.2948172136993
"Adgb",75.9594780848506,-0.869634686567724,0.433538210658524,-2.005900
89913134,0.0448668223156615,0.106945391382734,-1.94401349892609
"Fam160a1",458.651768937182,0.0489075525151726,0.125704591326799,0.389
067352265803,0.697226323641298,0.797943472534031,0.01103929148573
"Olfr161",15.1288050568625,0.366228975047587,0.533087349387249,0.68699
6184525001,0.492085132801614,0.628582017778162,0.170035997362962
"Gatd1",1651.24586209792,0.117622135436559,0.11054629455483,1.06400794
264723,0.287325101454064,0.431341609805307,0.0989031494786801
"Hs3st1",275.824116636471,-0.965115352918794,0.219590664690338,-4.3950
6549278759,1.107393612065e-05,0.000185476569356248,-8.29283280424321
"Serpinb1b",4.89249570344728,-1.91734473634463,1.20686910168461,-1.588

69320100109,0.112129674679226,0.21497585023555,-2.94739904692826
"Zfp11",196.820422992873,-0.0576677353214194,0.167796404005911,-0.3436
76824679675,0.731089341924161,0.823832860708391,-0.0111752925209346
"Zfp455",125.998401272214,-0.67994910177597,0.237322433523848,-2.86508
56629095,0.00416896448162447,0.0172986740716565,-2.75863878761857
"Olfml1",473.603375684844,-1.18462130723192,0.240170416686021,-4.93241
975251513,8.12171500516831e-07,2.34603258030773e-05,-12.628299854694
"Gprc5c",6458.80115860871,0.811673259708474,0.386972572396585,2.097495
57877357,0.0359497281425022,0.0903166859067784,1.95161401356168
"P4ha3",11.1074549491392,-2.29137351845996,0.956914442502063,-2.394543
77182213,0.0166410570336132,0.0499534670756096,-6.86647507915546
"Olfr523",2.05958051539412,-0.636149953000343,1.38592868549918,-0.4590
06267534764,0.64622966530203,0.758773094909008,-0.175610784245917
"1700014D04Rik",3.1241187262423,-0.85084997042129,0.957241906961922,-0
.8888557471556,0.374080610961745,0.519244233824454,-0.557630837335388
"Gja10",0.106914658212752,0,4.78471990596623,0,1,NA,NA
"Fbll1",0.184548909977211,-1.07262530427466,4.7810839159584,-0.2243477
25982058,0.822486742541638,NA,NA
"Mb21d2",772.54485682134,0.752797792119368,0.243936192255417,3.0860438
7548666,0.00202838888958608,0.0100968921544931,3.45950303840338
"Lingo3",0.59290642006993,1.57695929340502,2.54579044728082,0.61943798
0486328,0.535627867011411,NA,NA
"Vtcn1",163.566277157026,-1.35256402873279,0.453932629191252,-2.979658
08525945,0.00288570286294657,0.0131277261833253,-5.86069886717012
"Rgs13",1.91089304138964,-2.65382302419107,1.85346878239276,-1.4318142
5519618,0.152196986480009,0.271087328878471,-3.46407304556146
"Olfr986",2502.3865974307,0.551163932857208,0.35025595653532,1.5736033
1087368,0.115579187352368,0.219945636833542,0.834668808324611
"Mblac2",629.175785582481,0.0198512454936167,0.243945557523913,0.08137
57204480787,0.935143157337184,0.959603246237589,0.000818573355922478
"Sv2c",27.8265540605411,0.494978743053591,0.464067577145167,1.06660919
105485,0.286148348395278,0.430237546037255,0.417473877654555
"Fam71e1",199.018930612106,0.392332633835905,0.206953986806966,1.89574
813169388,0.0579933574615279,0.130271253892266,0.79962743526223
"Gm8121",1.31160477654669,-0.235505281274959,1.19084798891097,-0.19776
267287509,0.843230752055585,NA,NA
"Olfr77",39.8687502488462,0.657062966963372,0.663580237788614,0.990178
624295743,0.322086818632735,0.467196011731964,0.500029112573528
"Gimap9",798.040624031879,0.159686923842248,0.196084380514664,0.814378
602839842,0.415428092636432,0.558698920556294,0.0929608731527303
"Gm6293",0.370320733343718,1.1445336344595,4.75821995606906,0.24053819
3910027,0.809913057765823,NA,NA
"Ghsr",0.229540625669495,1.39212599735259,4.78471990595879,0.290952453
793349,0.771087683426917,NA,NA
"Camk2n2",133.372464872752,0.123517690231554,0.239431171709692,0.51587
973842152,0.605938406200451,0.7267384538412,0.0394254419622328
"Nat2",635.610101960999,1.28155302874289,0.217623167093357,5.888863055
61911,3.88861497322544e-09,3.99053761831314e-07,18.8826197892856
"Adnp",343.431578902022,-0.998490474375374,0.804044665598718,-1.241834
58593294,0.214297599107057,0.349003000606815,-1.05108571956616

"Commd3",3316.73867484562,0.210720519571229,0.147735870522613,1.426332
81156302,0.153772297732285,0.273239065085582,0.273390523828241
"Cited1",7.57108669169586,0.102729615478601,0.600028287972345,0.171207
953921225,0.864060253513311,0.914429375977124,0.00918968197054464
"Olfr853",34.0203351966269,0.659176407861619,0.431429776541015,1.52788
806824267,0.126540331160913,0.235571111608674,0.952999315533141
"Eml5",1813.42463437672,1.32821083252696,0.28552382908185,4.6518388212
9224,3.28988180943425e-06,7.11943568483341e-05,12.6845422876617
"Rpusd3",480.19341903275,0.792861277115364,0.267631614963348,2.9625097
8130497,0.00305142114217128,0.0136994265323637,3.40169305879334
"Plcb1",1102.60664508994,0.592788482420567,0.226944533662342,2.6120412
4573692,0.00900033949023059,0.0311868881074313,2.0556467209227
"Olfr655",23.8069601466629,1.78527259300846,0.441126969284303,4.047071
97092242,5.18622989894171e-05,0.000593949890816522,13.2622823638174
"Zfp524",586.265484124374,-0.0635223123106866,0.230183127171136,-0.275
964242433197,0.782575530547084,0.859449008370341,-0.00962132972775544
"Fam174a",1400.74565722506,-0.0544293223669143,0.289666979096754,-0.18
7903096640967,0.850952615210222,0.90524528414827,-0.00541840310721194
"Olfr1356",196.062705104146,0.487049226285557,0.25015828053667,1.94696
423896375,0.0515390221451385,0.119050806040269,1.03654056719745
"Gpr183",133.431411382479,-1.19515799180131,0.473234567819099,-2.52550
864428436,0.0115530939196883,0.0379357065082658,-3.9103925984396
"Ercc6l",241.528569593978,0.904668419022641,0.224056394842406,4.037681
76158933,5.39820158201412e-05,0.00061301184884165,6.69194654875052
"Bzw1",7661.19723076546,-0.208419728907622,0.142848047866089,-1.459030
99147006,0.144556577961699,0.260640295644276,-0.280244085329504
"Tceanc",220.799228212596,-0.0343887755191242,0.184564753452433,-0.186
323633715834,0.852190974945455,0.905998889284977,-0.00339476359266592
"Fam83a",103.881621088263,-2.1409328471032,0.995027710625765,-2.151631
380956,0.031426398511657,0.0814438754639531,-5.36911947317166
"Nyx",18.2090722475674,0.278438168076739,0.379506486910771,0.733684871
484701,0.463140820940492,0.602262220001181,0.141185511031126
"Tmem199",1004.34632186549,0.0502255606455221,0.170976806850112,0.2937
56571846337,0.768943922268376,0.850514442726844,0.00813221569241565
"Rnf7",3582.96496272035,0.0266505920754795,0.238021585959804,0.1119671
22511234,0.910849475045165,0.945169934243248,0.00150284135997669
"Gen1",220.106868331997,0.499155542636591,0.221639168958723,2.25210888
933423,0.0243153916893569,0.066906789043054,1.34994362146557
"Msrb3",1509.61708009652,-1.65790108964947,0.271568330149697,-6.104913
22289158,1.02856549352828e-09,1.59166428514401e-07,-25.9516487609763
"Swsap1",566.179380593874,-0.0498588410072829,0.245949847094628,-0.202
719544639928,0.839354245937184,0.897124821651609,-0.00541268934294365
"Pcdhb9",105.976736808159,-1.34976684318859,0.434544274875157,-3.10616
64397175,0.0018953000347577,0.00958116673002478,-6.27365677094271
"Islr2",209.284662452905,-0.621871248091145,0.432609479917203,-1.43748
872126003,0.15057917532936,0.26888842939572,-0.816802229708649
"Msantd1",185.141443639882,0.198262527076533,0.289137506887687,0.68570
3246218923,0.49290025929554,0.62929342952753,0.0918268020448075
"Nhlh1",0.690414035312809,0.960478307395341,2.43237125495523,0.3948732
35505745,0.692936443222483,NA,NA

"Gm6563",0.605816608983402,0.0579936247068956,2.72190975225569,0.021306226137305,0.983001377228204,NA,NA
"Jagn1",1347.43706762281,0.104543213800949,0.217319150008777,0.481058451575606,0.630474956399448,0.74611560450816,0.0306180649879388
"Trap1a",1.71956964669503,0.3541008385878,1.52261455835158,0.232561048786476,0.816102276571538,0.882553161880229,0.0442400315671464
"Nat8f3",1206.91186147117,2.33693442016288,0.589793823343944,3.96229042703975,7.42341607745128e-05,0.000781965244074258,16.7177283768913
"Actrt2",0.257801208733911,1.8316319500156,4.71555462250302,0.388423440431567,0.697702700354001,NA,NA
"Zgrf1",136.605349135013,-0.0170288318820715,0.248868193902868,-0.068425103324846,0.945447239093675,0.966413120201755,-0.000581770892772861
"Gdf6",13.2825377693245,-0.984978107923637,0.605889920373137,-1.62567171825047,0.104019509529421,0.203305172093489,-1.5691165360967
"Pcmtd1",5342.72868399436,0.00116706760458013,0.20426907108873,0.00571338381459221,0.995441404065264,0.996975369656078,3.53529727319872e-06
"Usp42",1331.27010470784,0.0183535705605428,0.310178007067489,0.0591710893175911,0.952815836665001,0.970485461176106,0.000549852000294861
"Ffar2",181.769123640075,-2.52798421475733,0.691493624134815,-3.65583156015401,0.000256349642387714,0.00204597025267116,-15.6529828648944
"Taf7",365.352252835892,-0.385148270451072,0.153744016143764,-2.50512689931897,0.0122407466949704,0.0395586539246189,-1.24401766683421
"Mtln",1470.60577438432,0.246878156674957,0.32973540896355,0.748715940004635,0.454028434108932,0.594303900340825,0.128466622069971
"Pcdh19",339.047173890742,-1.28517590198008,0.321095080312383,-4.00247771074467,6.26825746951412e-05,0.000688559157271561,-9.35724920452428
"Nup160",677.364314683379,-0.0504884772137809,0.234475289489974,-0.215325364662529,0.829513655272802,0.891135547087361,-0.00581923796097113
"Cacna1c",500.912692154045,-1.08657529335873,0.303467981346997,-3.58052697531967,0.000342901938595807,0.00257150076874656,-6.47953706195997
"Gfod1",274.3693280885,-2.3803269761717,0.468289815426234,-5.08302102193946,3.7147856956945e-07,1.26335078408115e-05,-26.8480838687676
"2900026A02Rik",1946.18914297555,-0.468633286068509,0.224868816856713,-2.08402966947223,0.0371574685694303,0.0926261725377362,-1.11496459885561
"Olfr645",3.03959643369774,0.881525993836499,0.90312001637735,0.976089531679887,0.329020098507947,0.473833656895484,0.658410842454731
"Zfp52",379.716572343746,-0.208078855726045,0.19507109205052,-1.06668216976071,0.28611538130361,0.430221154057052,-0.175505336602008
"Rab11fip5",4415.81381331267,0.132130037047136,0.150299858762069,0.879109522360253,0.37934189516751,0.524196192202418,0.0853413708130725
"Plekhm3",741.593158049127,-0.497871621537488,0.184993527066844,-2.69129211941341,0.00711758411625993,0.026100931935083,-1.81513252078722
"Spryd4",518.546103962273,0.344416915723971,0.251087031455683,1.37170332425058,0.17015581314286,0.294617451775549,0.420904176352564
"Zfp46",806.965968702137,-0.670359841515763,0.152777087226757,-4.38782970459956,1.14487360578438e-05,0.000189494617453548,-5.74575471390089
"Samd3",52.0351153536803,-0.994758635955824,0.396352841763201,-2.50978050650672,0.0120806225661927,0.0391470797400074,-3.22344518048648
"Commd1",2025.04855653314,0.729172266249842,0.196010541756043,3.720066

58273196,0.000199170263660503,0.00169212278904248,4.65341075093983
"Ncald",802.875885746054,-0.272447881069476,0.25064323234248,-1.086994
76352588,0.277039123920338,0.420562818296384,-0.235983843869786
"6030498E09Rik",0.294579729475019,0,4.78471990595405,0,1,NA,NA
"Six1",233.754252146664,-5.14323852152164,1.37427451649632,-3.74251174
69501,0.000182190013138551,0.0015746191827019,-33.1931335607568
"Plpp7",100.844326027142,-0.263896135870241,0.205996542099611,-1.28107
070720941,0.200168831680415,0.331652946983747,-0.291253244738384
"Pcdh1",2762.09381549857,0.0244352638032366,0.139427952506372,0.175253
694571179,0.860880294960754,0.911855931722337,0.00225472171860419
"Kif18b",52.6908596753952,-1.11938776483044,0.485349783298476,-2.30635
266224493,0.0210909347746516,0.059939380627848,-3.15042904853625
"Flrt3",332.765672534816,-0.67239085572207,0.343859463291488,-1.955423
44330389,0.0505331113108678,0.117355835041552,-1.44062742022527
"Zbtb22",1022.34531837092,-0.225610858983948,0.171422550496508,-1.3161
0956860978,0.188137269831141,0.317134995000223,-0.259097769808955
"Ywhag",7055.55554982134,-1.33294038867968,0.425085020420601,-3.135703
03503238,0.00171442709417719,0.00891229260464894,-6.291910192251
"Gm45902",122.331540462826,-0.133210118225347,0.180104646275442,-0.739
626217202764,0.459526829084285,0.599201104359352,-0.0682246282261982
"Tacstd2",2958.09322579848,-0.403870536150741,0.332644334881936,-1.214
1211913141,0.224701448895175,0.360891997574805,-0.411615376088049
"Kctd16",2.71005152211695,1.20830939684059,1.30048007832113,0.92912564
9045291,0.352823973446758,0.497855104520544,0.842730796128315
"Ppp1r37",5055.84331870248,0.429533256979886,0.16182421908623,2.654319
97389096,0.00794684311570332,0.0283423352972349,1.53059822601113
"Vamp7",1161.99996229679,0.252547624883179,0.145370641126773,1.7372670
5011186,0.082340044696893,0.170016540453248,0.447478920949048
"Plagl2",845.646088681984,-0.481673150860905,0.164104651177972,-2.9351
584333739,0.00333377420929058,0.0146466718190058,-2.03436685466332
"Ccdc157",1543.66071862544,0.121784245278137,0.165972062693278,0.73376
3522016342,0.463092876280073,0.602240038798697,0.0617566906253261
"Gpr87",32.4047538377936,-3.53265571315898,1.03453494452882,-3.4147282
6204814,0.000638456723163802,0.00416342113319325,-19.3639631611126
"Fhad1",73.5429003431104,-1.14331305176021,0.483767000038911,-2.363354
77961136,0.0181103229104898,0.0532441308969737,-3.35318591862576
"Cd14",1508.71727316583,-1.16881281850702,0.307135978721599,-3.8055223
0765019,0.000141505259163079,0.00130267683813112,-7.76481396641034
"Bbs12",347.315585918279,-0.129839495464407,0.213971332516096,-0.60680
7902430758,0.543978403151356,0.674241364480652,-0.0511784605915203
"Crebzf",3098.75288786894,0.0683308849190171,0.152454043354344,0.44820
6445795589,0.654004211211507,0.764383195397004,0.0183595556234861
"Meioc",0.696995324810861,1.68743930216159,3.00491796123226,0.56155919
1941998,0.574416390603891,NA,NA
"Hspb3",0.260121955815912,0.890114603675218,4.78471990596089,0.1860327
50332218,0.852419078549083,NA,NA
"Spn",784.206712630936,-1.14130770836792,0.4206270741409,-2.7133481854
4184,0.00666070801827279,0.0247986413863439,-4.21937626144787
"Zfp24",2267.79244265341,0.35163578629358,0.169380340727599,2.07601298
228044,0.0378927503084118,0.0940469508036423,0.83125333591028

"Cbr1",2440.72982025797,-0.138596254466362,0.281611673981021,-0.492153
796421461,0.622610624320969,0.740499506463554,-0.0416385159388344
"Pcdhb11",34.1796951891886,-1.75301655922045,0.38415073160901,-4.56335
603443461,5.03423349235384e-06,9.98550199734641e-05,-16.1484225561372
"Foxd4",0.184223986600386,0,4.78471990595808,0,1,NA,NA
"Irf2bp2",4974.65460560981,-0.19254120904124,0.143056236769793,-1.3459
127220792,0.178330665443482,0.305302600300028,-0.228440875994361
"Kcnj16",10187.5343343871,1.57163744123464,0.58717960309915,2.67658725
360945,0.00743761968526781,0.0269802248601585,5.67777772130571
"Tlr6",99.5298686476035,-1.09201751916268,0.420234366786427,-2.5985916
561595,0.00936070514878418,0.0321555724085613,-3.75344933000151
"Zfp786",42.2232933514284,0.31946801365669,0.280692558936642,1.1381420
828074,0.255061158222926,0.395796455473984,0.296100590074719
"Ufsp1",394.170178859455,0.995944149856964,0.272627980344352,3.6531252
1700451,0.000259067815168778,0.00206175684088197,6.15911464868428
"Gm6583",0.954584338544839,-2.41145836854548,1.85897114068075,-1.29720
054054331,0.194562194800027,NA,NA
"Siglech",46.1017255712083,-1.35524856236334,0.444836182484155,-3.0466
2393871617,0.00231427048893668,0.0111802888982377,-6.08994896622458
"Wdfy4",1281.32186518636,-1.31400192926658,0.433287552727878,-3.032632
53466187,0.00242430613477313,0.0115597306871762,-5.86074781980607
"Olfr414",21.8978962932703,0.647211918562188,0.335418063947857,1.92956
78680645,0.0536604033289016,0.122643364682561,1.35815777797359
"Mafg",8639.8110017699,0.30562576389144,0.174809283009654,1.7483382954
8149,0.0804054657308615,0.166923527557532,0.547137199243873
"Fam181b",101.953267215893,-0.747802353237499,0.390443886818227,-1.915
2620350428,0.0554590946717857,0.125781252519539,-1.55035204354319
"Arhgef39",878.834805937863,1.10368779041495,0.461481635537329,2.39161
800908906,0.0167742904696976,0.0502266721768623,3.30136093478816
"Rps19bp1",1028.96740402048,0.231014596947067,0.144047985375938,1.6037
3361935026,0.108772782009413,0.210026906676523,0.360502813223338
"Usp29",27.8042121957028,-0.148721768349667,0.438346863801111,-0.33927
8732508841,0.734399762515177,0.826236908055152,-0.0283870790970503
"Gm5124",18.2106655351012,-1.01260843238664,0.387294691522425,-2.61456
832368696,0.00893402899370057,0.0310122302508766,-3.51716742462257
"Gm6365",0.849253894467468,-1.90787514749331,2.12379938589145,-0.89833
1151316577,0.369009029608871,NA,NA
"Zfp579",2944.22800286879,0.407927787653799,0.245688175735328,1.660347
65992665,0.0968445330136745,0.19256319648181,0.67199204374504
"Pusl1",402.559099730729,-0.157747365492786,0.161294726824454,-0.97800
6960292455,0.328070881629627,0.472711058972397,-0.118195517791792
"Tceal8",2053.35665102109,-0.0220823939661257,0.213017469030373,-0.103
664709127575,0.917435433754401,0.949331351328659,-0.00114822669454623
"Mical3",3321.63115705006,-0.232483358841984,0.235767980255244,-0.9860
68416034677,0.324099541134734,0.468999024272306,-0.17602584247386
"Map3k19",7.63307963548762,-1.74026538895089,1.10982579230025,-1.56805
275298565,0.116868821564013,0.222078365478114,-2.61862077082346
"Ccnb3",0.160750197886176,0,4.78471990596389,0,1,NA,NA
"Otop1",464.194689562339,-3.72829646503735,1.80250392795077,-2.0683985
2453247,0.0386025638494686,0.095407883120413,-8.75998327161403

"Pcdhb2",2.29187137379546,-1.52610474824367,1.85188218386104,-0.824083
06616021,0.4098923612683,0.553513680875572,-0.902643359546703
"Rap2a",1122.98249570697,-0.652437927452257,0.163873651780373,-3.98134
733902597,6.85257267123516e-05,0.000740229706059519,-4.70313142607271
"Krt9",0.29701819758227,-2.19685673226412,4.74443328935594,-0.46303880
7436229,0.643336550668347,NA,NA
"H1f4",64.4264160581881,-1.29991385636201,0.564672632877937,-2.3020663
3131272,0.0213314317779552,0.0604711045080794,-3.64702484761888
"Gm5812",1.36441025621702,-0.367976392298677,1.87447082394576,-0.19630
9479773116,0.84436794196738,0.900332830169527,-0.0386341257707802
"Kctd19",2.23623940147553,1.23882890366761,1.68810195946016,0.73385905
2011157,0.463034645771207,0.602204477271011,0.628282271709245
"B3gnt2",682.553706168797,-0.794838692525365,0.2771544808029,-2.867854
38295194,0.00413265691487004,0.0171986715608152,-3.22936851085033
"Lrrc3",566.535279820169,0.61131000042507,0.307210385786811,1.98987413
416839,0.046604802340651,0.110185574880322,1.34829878998105
"Pcdhb1",0.778227257102852,-1.00963258338768,2.98873359909971,-0.33781
2839421957,0.735504233657129,NA,NA
"AU021092",722.929264836032,0.163754319980885,0.406747654643551,0.4025
94380352087,0.687246644448329,0.79005030153336,0.0385901240614921
"Coa6",1464.79519860154,1.16883638360312,0.252606724169797,4.627099248
62277,3.70822837106011e-06,7.87922476044029e-05,11.0439795999835
"Dcun1d4",1220.89066597065,0.576610697597447,0.151956552819997,3.79457
606070125,0.000147895939797738,0.00134983830384445,3.81011113948368
"Trim32",856.887886052962,-0.190249883947213,0.143560871097045,-1.3252
2101944204,0.185097855727641,0.313420581066222,-0.220729681052347
"Pcdhb6",13.2905035593319,-2.00729922383882,0.603107121873003,-3.32826
317421849,0.000873892701396164,0.00531230678977397,-10.5136895859663
"Treml4",61.8999862087296,-1.53251894061375,0.511441395966618,-2.99647
02753818,0.00273124864152025,0.0126134263411563,-6.70169528888101
"Vmn1r73",2.69617630464225,-0.582135262188014,1.43966649019607,-0.4043
54248815454,0.685952238519623,0.789160770985108,-0.137841022277011
"Pcbp1",7620.55003988991,-0.0328839132275038,0.164858491922153,-0.1994
6751207109,0.841897054306875,0.898877930395107,-0.00350568945434474
"Tmem198",123.670671478878,0.0886208908717025,0.255502548326002,0.3468
49342412934,0.72870450561188,0.821857962194611,0.0173863282416569
"Senp8",493.057881436901,0.251008029192089,0.202140068682476,1.2417529
6282488,0.21432772229285,0.349003000606815,0.264229816659289
"Olfr1325",0.148895757179753,1.67550258368843,4.75885183143336,0.35208
1267296733,0.724777316609313,NA,NA
"Apon",10.4463265709347,-0.168731742866894,0.581661658628719,-0.290085
723141324,0.771750657716278,0.852431822854301,-0.0269400553273705
"Wdcp",524.698201756778,0.178368834674208,0.180087011251156,0.99045918
6562036,0.321949728506726,0.467132656324739,0.135764011739658
"Rpl31-
ps13",0.545279851113254,1.34838490166943,2.32183392221338,0.5807413220
94231,0.561414807417377,NA,NA
"Kcnf1",162.560844121092,-0.185941746136785,0.507593269633645,-0.36632
0353835637,0.714126043405987,0.810431848805653,-0.0390827287042835
"Kctd14",197.629978730102,-0.402219281552517,0.264946499455963,-1.5181

1510013693,0.128985385148955,0.238774052558455,-0.576073562796046
"Fam243",150.235599282142,0.858542348720989,0.481028516683688,1.784805
5134859,0.0742928687415729,0.157138117831084,1.58884433753332
"Mettl5",589.425346821161,0.364838580865787,0.149944985879695,2.433149
58966688,0.0149681155753457,0.0461128641946739,1.12248547878862
"Pabpc2",1.01292798083963,0.417343501910652,1.44208891587023,0.2894020
59275108,0.772273719317475,NA,NA
"Rinl",797.920726056113,-0.966498922212853,0.343672629548836,-2.812266
20659797,0.00491937710714136,0.0196271771710747,-3.79915270343025
"Fam229b",233.259237480732,0.768110397153298,0.142600556142745,5.38644
74159863,7.18639410689802e-08,3.53839172465398e-06,9.64119746253586
"Ttn",131.832212543175,-0.426297806542103,0.918391963013923,-0.4641785
02981564,0.642519861512308,0.755828190634509,-0.119338315128097
"Wfdc21",11.7618322029955,-3.27283208450508,1.2438694908804,-2.6311699
969332,0.00850914608755048,0.0298187150278683,-11.4962123652001
"Xrcc1",1469.04850822852,0.133150167510369,0.144815677295362,0.9194458
08610897,0.357862438291443,0.502990600604612,0.0914986375115609
"Wfdc15a",0.271349370025343,0.930789325864542,3.93187458944235,0.23672
9149084217,0.812866919918775,NA,NA
"Tubgcp6",1310.7530574149,0.053951691548378,0.181927743748005,0.296555
60189384,0.766805811250906,0.849450582794737,0.00880305535693392
"Nlgn2",2904.97958575551,-1.0818541523056,0.333072796217944,-3.2481012
096758,0.00116177937825224,0.0065907402726867,-5.43316849139221
"Adam6b",2.98956755391768,-3.29400118072891,1.75747358593695,-1.874282
04161191,0.0608915614009249,0.135095543015473,-6.5938427073187
"Cox6b2",1015.97559324482,-0.203555661668856,0.296629710483647,-0.6862
28164188151,0.492569240165558,0.628953179092865,-0.0943884472689276
"Sox12",923.055716939287,0.289293867326025,0.312900390906384,0.9245557
87508038,0.355197019946064,0.500334597233662,0.200329699381956
"Gypa",8.35888966062917,0.5781483457417,0.517469563200356,1.1172605827
5982,0.263882923875466,0.405772022848883,0.521468877251863
"Rtl8c",249.454115469132,-1.3439741562744,0.296833437451585,-4.5277047
2158688,5.96278230976563e-06,0.000113537431128721,-12.2078252644945
"Arf3",7093.89235288361,-0.0642653813161905,0.203882370663451,-0.31520
8132547535,0.752603631198226,0.839200823579036,-0.0112660581284002
"Mest",169.214359112515,-0.79696477226205,0.369061617104995,-2.1594355
3955468,0.0308163931933065,0.0802964097932769,-2.00996935862883
"Samd7",1.81338271209643,1.70582281746406,1.17847612283627,1.447481866
12268,0.147762011689686,0.264901039345852,2.26601325525009
"Tbc1d22a",1108.4903565104,-0.320909921934623,0.179194376320479,-1.790
84817573009,0.0733176619539061,0.155656334143866,-0.596926050259244
"Vmn2r72",4.40157144393808,0.365316719561525,0.726792159910254,0.50264
2625653303,0.615215554664913,0.734431695638062,0.112758031067144
"Olfr664",0.118377085491146,0,4.78471990596623,0,1,NA,NA
"Klhdc1",273.189773935674,0.491724330717485,0.34870736895581,1.4101346
1284152,0.158499938601742,0.279500027390501,0.62682701087315
"Abca16",0.10500178835157,-0.544048378937456,4.78471990596623,-0.11370
5376621746,0.90947135024012,NA,NA
"Cd209f",21.6075017733748,-1.77389380419905,1.34916087374999,-1.314812
6652002,0.18857286984935,0.317704468704971,-2.03400636645499

"Sox6",1314.78391018213,0.605835027454877,0.257513500158212,2.35263404
474974,0.0186409697127622,0.0544430238854758,1.76334376863503
"Rspo2",0.161850200053757,-0.0629309216593589,4.78471990595378,-0.0131
524776572714,0.98950614369312,NA,NA
"Spats2",885.142109762456,0.230480961514398,0.228113231480625,1.010379
62602346,0.312313445249484,0.457374760062674,0.180294234648313
"B3glct",867.957703072804,-0.385584136575322,0.217879090542817,-1.7697
1611004383,0.0767744448067897,0.160979473582405,-0.704261102776481
"Xkr4",6.33242329200827,-1.92313535384149,0.790948057943427,-2.4314306
5404561,0.0150393269233331,0.0462775473813993,-5.90998412748187
"Rnf133",0.162648236660872,0.412909841717156,4.78471990596623,0.086297
5994064531,0.931229846754807,NA,NA
"Nanos2",2.22033337615366,0.0344051840242058,1.19055695638785,0.028898
3940160167,0.97694562647783,0.985334462293851,0.000508306999228645
"Tlr11",160.33096544643,-1.34658445874691,0.442790984088219,-3.0411289
0085094,0.00235692904966667,0.0113218533161864,-6.03407256340991
"Prdm9",520.397726288921,0.296848775777465,0.321033941085352,0.9246647
71500104,0.355140309548501,0.500290857277413,0.205587260935499
"Erich1",335.133170162835,0.185262663918429,0.144425182056066,1.282758
73556808,0.199576615035342,0.331037417265768,0.204812296606365
"Casr",354.20679871052,1.45483515126602,0.572596816433311,2.5407670973
935,0.0110609572863661,0.0367029518668424,4.80808191969201
"Sec31b",327.290346533946,0.0190974874565899,0.387957641993723,0.04922
57024721757,0.960739428481159,0.976210192116611,0.000459816984730468
"Igfn1",31.4465022456819,0.995367564890536,0.695184137385498,1.4318041
9598467,0.15219986610076,0.271087328878471,1.29926747960689
"Smim11",1332.55383399794,0.249498080462726,0.155367464593892,1.605857
96463164,0.108305121274519,0.209310462346176,0.390199195498308
"Lax1",265.76914414347,-0.972355600293995,0.391993381162539,-2.4805408
6375201,0.013118322630053,0.0417056994359651,-3.08928797679911
"Gm9864",2446.07652354523,1.10347535337458,0.47114733588249,2.34210250
028836,0.0191754478645845,0.0556373336999507,3.18783086826143
"Olfr796",0.214228137860247,-0.920587860243252,4.78278000526876,-0.192
479658112881,0.847366500467667,NA,NA
"Btla",355.777982790905,-1.27554265810199,0.451304034810731,-2.8263488
905809,0.00470819522286249,0.0189591884459671,-5.05812179042784
"Slc6a7",30.3219318339492,0.98323507403295,0.383572465393204,2.5633619
7913744,0.0103663907938944,0.0348669808003023,3.29994831037779
"Tagap1",2024.76880869477,0.746280962821254,0.234134319858318,3.187405
26067624,0.00143555478336494,0.00777512421279153,3.62455666795333
"Pfdn4",763.899719111957,0.297229969115546,0.180864920080224,1.6433809
7727137,0.100304133427202,0.197808232382279,0.481648455675043
"Klf13",3947.00907629039,0.319023749188149,0.287437683860877,1.1098883
9355719,0.267047122502493,0.409056861855104,0.285175682259344
"Zfp217",1480.88222420962,-0.577875970331058,0.195713567562939,-2.9526
6177775447,0.00315046939764249,0.0140214225782318,-2.46589438873308
"Olfr901",0.25887104520169,2.12804998605385,4.7257511146108,0.45030936
5525931,0.652487386879203,NA,NA
"Pard3b",1800.67936609227,0.554440495359659,0.344588672589029,1.608992
22598912,0.107618041510182,0.208230284148608,0.869978500682548

"Dock8",2808.14160334171,-0.463162313838353,0.105044642296899,-4.40919
50213821,1.03755560165207e-05,0.000176068399660679,-4.00387054022262
"Rgs14",259.429152256903,-0.880554503208027,0.408426362333371,-2.15596
882183939,0.0310860997793082,0.0807940247263333,-2.21534504363813
"Gnpda1",3936.23020823035,1.14526641315747,0.299000432716488,3.8303169
0874978,0.000127978329006427,0.00120488723272203,7.69775850882664
"Mtcl1",799.05936831837,0.290995407195234,0.380291046952003,0.76519131
7354261,0.444157629169345,0.585424555772587,0.155804166884245
"D630039A03Rik",599.048156434625,0.43788478066767,0.329256941855577,1.
32991814295518,0.183545242981238,0.311684823867634,0.510469779957585
"Akr1b7",293.159768622192,-0.0697502039915725,0.47844518429459,-0.1457
8515215784,0.884090997529381,0.927895720325706,-0.00521982088458989
"Sema5b",65.6648809498436,0.832933608656801,0.551075810541677,1.511468
28208277,0.130669183810162,0.240971128906701,1.18532961865971
"Foxo6",19.6641205431596,-0.840781922547407,0.462404621325138,-1.81828
183320905,0.0690210679818003,0.148572839866322,-1.60310202298471
"Rbm12b2",1309.85272457202,-0.107105279873564,0.137243071105223,-0.780
405735685172,0.435152092858367,0.577183367792683,-0.0588645549776452
"Babam2",2039.39370111448,0.180475699624816,0.175723461354589,1.027043
84624338,0.304399818307566,0.449129843216643,0.144460555541926
"Rasal3",1004.09328245443,-1.25803281643096,0.49033185542919,-2.565676
29147773,0.010297487872929,0.0346531616407264,-4.22996716194442
"Ppp4r2",4207.36783892517,-0.29176321138422,0.108045172403734,-2.70038
174675667,0.00692599538908443,0.0255618130032876,-1.06979524811679
"Rps10",15576.2912459937,-0.229749484743385,0.215100460988814,-1.06810
317229089,0.285473975066994,0.429654308441239,-0.194086466463024
"Plpp2",2219.59185159372,0.587187087273965,0.211326970837285,2.7785714
4758906,0.00545985046331836,0.0212317838719149,2.26199496879968
"Acvr2a",1207.0334939631,0.0640860979794974,0.105416280969507,0.607933
58853207,0.543231521306769,0.673658220596539,0.0253160843087965
"Pld4",2391.13276910336,-1.07810856738558,0.418536789319715,-2.5758991
6799888,0.00999797973780245,0.0337676440200367,-3.65290373874818
"Serpinb6c",13.4035760163242,-1.44255224021985,0.590531264791903,-2.44
280417689348,0.0145736425947981,0.0452984237485801,-4.46395344590912
"Cd177",5.85490119774815,-1.64822767896781,0.839311780809963,-1.963784
75395307,0.0495550588802483,0.115563274494329,-3.55677160479206
"Opa3",3612.19810122712,0.754080539614571,0.132899472069808,5.67406723
194864,1.39446362210956e-08,1.02844968174828e-06,10.396853808497
"Ppp1r36",91.4586364541588,-1.53290682026653,0.557036811562128,-2.7518
9500666521,0.00592515141422223,0.0226171891688538,-5.8082530440615
"Gpr17",47.3175409794127,1.19808885639783,0.542750511966832,2.20743938
509826,0.0272833773614267,0.0732499619399642,3.1316575654534
"Epx",0.243148865927251,-0.0565070046367328,4.78471990596301,-0.011809
8876731134,0.990577291997576,NA,NA
"A930035D04Rik",14.6005080125099,-1.11169401712545,0.604057063022768,-
1.84037913829269,0.0657125940634993,0.142934422027009,-2.1626554576326
4
"Zfp622",1903.4774386153,0.551516564835428,0.150315791959054,3.6690527
1660119,0.000243450879145281,0.0019671799592104,3.43658478944754
"Fpr2",72.0873079033498,-1.2043608281053,0.709542687768094,-1.69737613

940282,0.0896255706645831,0.181485032383222,-2.05534062502647
"Bhlha15",25.5130503179877,-0.814450564450074,0.648702196155032,-1.255
50764168437,0.209294535777817,0.342926458705536,-0.871656970145416
"Dnah3",13.3965891567416,1.28782253833573,0.815326299883553,1.57951796
540803,0.114217299068778,0.218141727617107,1.96085186240783
"Ostn",0.0934659659323483,1.37778842356407,4.78471990596623,0.28795592
0229742,0.7733804806872,NA,NA
"Taf9",98.7745903971191,0.544877634516236,0.392741470930772,1.38736974
535669,0.165329045607073,0.288103112990142,0.678065797166099
"Ppp6r1",4782.06828039425,-0.0408581411072327,0.121409399143459,-0.336
531943947389,0.736469767040699,0.827704291313188,-0.0077262468829003
"Cdc42se2",2011.42592498816,-0.516788370450222,0.197515956649052,-2.61
643858662243,0.00888523463802235,0.0308648325266631,-1.79746119126142
"Ltn1",1899.15925415411,-0.0143890475112018,0.110220403551212,-0.13054
7947998721,0.896132921673395,0.936126424151506,-0.000949745384251349
"Doc2a",58.6430063819009,-0.0960226591299645,0.598800544840295,-0.1603
58336273015,0.872598807673474,0.92016936463047,-0.00798884843739019
"Tbc1d30",192.913067716551,-0.77562496476847,0.30649361604866,-2.53063
98703108,0.0113854680151667,0.0375243163217918,-2.54619535686719
"Hbb-
bs",11507.6213010999,-0.495302508937379,0.50556035546853,-0.9797099467
54737,0.327229314791721,0.472048788280162,-0.37181018747183
"Slc39a1",3818.85434720649,-0.5824976782192,0.153010448900009,-3.80691
438007516,0.000140711432726398,0.00129720639021244,-3.8721779307908
"Lrrc15",11.9893066815597,-4.09155783968871,1.38333243596468,-2.957754
57389274,0.00309888745710656,0.0138551037924017,-17.5081917366157
"Ankrd44",1548.76059911174,-0.595159258549237,0.190322276454001,-3.127
11296668985,0.00176532082033787,0.00909144885233697,-2.79749907353803
"1700024B05Rik",3.67397539426927,1.31662208193931,0.988903943799565,1.
33139531922644,0.183058968778017,0.311184287117156,1.53698509243594
"Cx3cr1",2588.39297155854,-1.28595237843739,0.493692124746883,-2.60476
58327479,0.00919370704261891,0.0316991865458857,-4.43841867352009
"Immt",10855.7481927353,0.563752458004941,0.267350158886229,2.10866700
193302,0.0349733350317591,0.0883532115119512,1.36789614123374
"Cemip",102.216714449632,-2.10749319117833,0.488323763803461,-4.315770
28888265,1.59047307746487e-05,0.000241331082213307,-17.5540289853547
"Zdhhc19",11.2817100786834,1.10806281422062,0.714393459921562,1.551053
97289371,0.120888750106194,0.227892183077499,1.63869486342686
"B630019K06Rik",157.047876229047,0.164872242111747,0.199686140717721,0
.825656910986187,0.408998741648269,0.552720922141527,0.097753093045476
7
"Tmem106c",1755.00365292392,-0.0883250275190507,0.176497006213953,-0.5
0043357342833,0.616769818209365,0.735611568109017,-0.0271204699998712
"Il1rapl1",3.40651076009376,-1.05758601451539,0.956880871189084,-1.105
24313564881,0.269054250381577,0.411420263033486,-0.939284494887929
"Mpp3",243.425012771512,0.108657506853084,0.312948515970434,0.34720569
4572935,0.728436793003376,0.821698616718532,0.0213383347745433
"Actn2",25.9829394762602,-0.459746779606058,0.601423394948005,-0.76443
1153606527,0.4446103494225,0.585783674102297,-0.245874745155398
"Rnase9",0.199266769027631,0.864206500620333,4.78471990595963,0.180617

991775008,0.856667432756454,NA,NA
"Nrros",1343.70311976243,-1.19947939704668,0.28171199601022,-4.257821
45860118,2.06428705485986e-05,0.000294652042427947,-9.75142614414018
"Trpm3",278.258493695817,-0.425154537900629,0.262120207777553,-1.62198
306458476,0.104806974341762,0.204304007648951,-0.675207547139409
"Acot4",2985.12071451472,1.6963393140844,0.36464074845898,4.6520837872
7103,3.28597503793846e-06,7.1188823655249e-05,16.2003368390029
"Rft1",846.844348816807,-0.00862627931974875,0.15193383251402,-0.05677
65531679901,0.954723191663728,0.972123494232565,-0.000243885886099403
"Mogat2",365.081075561837,2.10447611906988,0.680675788317247,3.0917452
261267,0.00198983550772892,0.00995581542974043,9.70078982126478
"Ezr",15842.954214967,0.250385659729956,0.29046559240488,0.86201486949
595,0.388679335378483,0.53286947554561,0.157612457415881
"Rexo4",1608.78464469879,-0.0024809083242378,0.0893440216546335,-0.027
7680395206291,0.97784715689601,0.985961086817372,-3.50760515777769e-05
"Ccdc171",163.175271639237,0.338520158677949,0.232243996215809,1.45760
563973152,0.144949274239959,0.261201090504158,0.454451365501482
"Gm28047",0.592773248650819,-2.91477056268671,3.54419867108079,-0.8224
06087579131,0.410845815842216,NA,NA
"Tchh",572.336487276392,1.11413468453039,0.422759502590201,2.635386496
82433,0.00840415278806041,0.029562361728595,3.92315042340324
"Olfr720",4.82757628190184,2.0559380239364,1.03777486189471,1.98110216
331776,0.0475798177471665,0.112042425897857,4.50019684538662
"2610001J05Rik",2251.93749236758,0.00991568765700425,0.107995451119961
,0.0918157899631343,0.926844398478596,0.955159200926832,0.000454904480
110109
"B4galt3",2292.17613835277,-0.2606106093672,0.101294401156874,-2.57280
369290692,0.0100878408182006,0.0340475541411244,-0.880863098771447
"Gm9877",0.416708554898905,0.412199748666724,3.75568298105334,0.109753
605601481,0.912604786043218,NA,NA
"Tmco1",4507.78159268107,0.405718714907128,0.270993410770218,1.4971534
3171627,0.134353339353778,0.246359649262375,0.56839683571104
"Bmpr1b",487.132524179484,-0.328293251772036,0.396008103477576,-0.8290
06398831497,0.407100786405964,0.550996191402435,-0.195671767384712
"Cebpe",10.7840524020536,-0.119232435143281,0.70273850625529,-0.169668
282130489,0.865271019548497,0.915213970877542,-0.010563682925609
"Zfp961",721.991928606321,0.0105733541497062,0.13915166459494,0.075984
3885481671,0.939431518421817,0.962511041050371,0.000404005130591001
"Asna1",3313.04191035552,0.581446510357529,0.133779073989878,4.3463188
4506483,1.38441287197363e-05,0.000219279302824873,4.89878237291023
"Atp6v1a",13288.4847691337,1.03216826155168,0.260911126443431,3.956014
7381276,7.62104613236595e-05,0.000800189324118855,7.36004320993371
"Pmp2",0.977758124461668,-0.0779408109878422,2.42715574519079,-0.03211
19941076198,0.974382738429254,NA,NA
"Tcp10c",0.319388375004306,3.16508737760244,4.6981254208954,0.67369154
5893046,0.500507437786643,NA,NA
"C130026I21Rik",99.0852442553806,-1.86318812273105,0.375246735323981,-
4.96523473048316,6.86180254432734e-07,2.03111775414358e-05,-20.1305164
819174
"Gm8174",3.46504743275007,-0.90184589802242,0.906129452660011,-0.99527

2690204455,0.319603656425843,0.464665729102311,-0.691208059172689
"Tmem171",545.136548001067,0.208836060384364,0.54361257771881,0.384163
40780913,0.700857337617306,0.800973102296822,0.0463465509632636
"Cherp",2980.33951130988,-0.0614378675268111,0.121931691523905,-0.5038
71198364914,0.614351891171113,0.733795019999878,-0.0190165899628169
"Pkdrej",8.6665509005413,-0.550158978652273,0.668332585359983,-0.82318
1437960176,0.41040482289483,0.55389168940288,-0.325026287193751
"Epha3",151.380129324558,-0.841882576350861,0.37505881325059,-2.244668
16031962,0.0247894435051538,0.067990514764874,-2.26330623464562
"Nav2",2645.2311420634,0.228907219127332,0.352291782327778,0.649765991
175897,0.515843389871045,0.649579326590757,0.0987575137616
"Robo2",826.299136644908,-0.484888549958706,0.466051187308737,-1.04041
908520553,0.298145238616382,0.442542315966978,-0.395290450841816
"Cyp2j5",27805.5599970927,2.1304616326919,0.781317316659086,2.72675593
803777,0.00639603283889817,0.0240131664467608,7.94481738278695
"Spdya",27.1358003350656,-0.329591690801769,0.347847902000468,-0.94751
6684465517,0.343375558500469,0.487983720090535,-0.236473216350384
"Nup188",1124.11261790674,-0.0939762542976181,0.164666088602132,-0.570
708001236884,0.568197594148409,0.695244222824329,-0.0341596255921593
"Pbx1",6665.56193320658,-0.0923495873706706,0.144788552435165,-0.63782
3818371441,0.523588373008044,0.656564801396286,-0.0388546004628403
"Magi3",943.805235828121,-0.250375607714477,0.165966964151118,-1.50858
701907991,0.131404353510278,0.241960721951402,-0.355277947782714
"St6galnac3",283.585083700547,-0.159902415700058,0.303894265550549,-0.
526177798749744,0.598764677945951,0.720794819447438,-0.052352172416428
5
"Adarb2",163.474722777061,0.717330906701108,0.413417577269863,1.735124
35402054,0.0827187814744914,0.170527680396451,1.26885625979745
"Gan",1756.12238976891,-0.0288486340703029,0.200405951821155,-0.143950
98452988,0.885539175075781,0.928542233698714,-0.00213882080390582
"Cpne8",1372.78986701634,0.438504146040921,0.241430951074656,1.8162714
6017962,0.0693287409417182,0.149054117419961,0.834668890332249
"Slc22a30",5763.75123797081,2.32969799620299,0.982293173076124,2.37169
315644062,0.0177067888202117,0.052326003094345,6.8732190977919
"D930048N14Rik",1387.32138063854,-0.0231159469179949,0.133131510716424
,-0.173632424011418,0.862154348382659,0.912858294041004,-0.00210758766
668499
"H1f3",48.2805559469562,-0.128636595037974,0.69229964022588,-0.1858105
76177683,0.852593310378807,0.90617957840098,-0.012672992081791
"Hook2",4118.65779786431,1.01954850050528,0.240365283391721,4.24166288
125614,2.21869783625111e-05,0.000311224247562763,8.23285093241109
"Dlg2",157.153549242771,-0.428250246443101,0.437240742829318,-0.979438
10924837,0.327363554959664,0.47211054693813,-0.321419844089333
"Lrrtm4",0.419868879130785,-0.463752194858535,3.14449544079227,-0.1474
8063833786,0.882752660984027,NA,NA
"Serp2",100.325369146727,-0.0883730626248614,0.410448125084337,-0.2153
08725327234,0.829526627327705,0.891135547087361,-0.0101857673103578
"Adam17",2371.49840072464,-0.0491267815610758,0.188372314573809,-0.260
796187976055,0.794249684302292,0.868052072435444,-0.00695161521108532
"A1cf",485.396338644935,1.41684119315639,0.714463153920218,1.983085041

38003,0.0473579311921103,0.111654769332983,3.10620270047984
"Isoc2b",818.284825339127,0.976749770377821,0.301846204923683,3.235918
67131401,0.00121251925655588,0.00681240417226804,4.8730145531464
"Plekhg3",5985.37414852814,0.447492590899451,0.156054741902642,2.86753
600335085,0.00413681733142407,0.017205559796951,1.81794881012606
"Pcdh15",5.68595111091,0.0509987770730557,1.53412342567551,0.033242942
6599751,0.973480853740748,0.983749354293898,0.000835570880166434
"Terb1",32.7889496180257,-0.121237971390508,0.326956211145981,-0.37080
797751347,0.710780559113644,0.807944418794408,-0.0258554536425991
"Olfr851",0.129581947825145,-0.550462304887101,4.78471990596623,-0.115
045878485115,0.908408757662264,NA,NA
"Sh2d6",6.64215925641615,-1.35937185342369,0.865515777799836,-1.570591
64984751,0.11627753078184,0.221058823633415,-2.05173588369102
"Asap2",957.851878728776,-0.593594380294207,0.129698634964469,-4.57672
03367778,4.72322201326036e-06,9.47462786157926e-05,-5.4992411605324
"Rnf103",2174.55426576825,0.137134084102796,0.12474597703045,1.0993066
6597226,0.271634325749135,0.41412809043458,0.120894659805484
"Zfp112",204.313659583993,0.115420130788476,0.12358986606721,0.9338963
98315609,0.350357336900283,0.495306507749544,0.0810917024132479
"Zmat1",954.092483127651,0.151842373041945,0.216591630695014,0.7010537
41341265,0.483269477453435,0.620828110509823,0.072383415631833
"Rap1b",6344.48606671753,-0.337803775660202,0.16399792085543,-2.059805
23349432,0.039417164066732,0.0968564417105408,-0.788611487434536
"Jun",5702.59046718031,-0.0777761050676369,0.149018289868091,-0.521923
215844734,0.601723793326383,0.72340171730798,-0.0251831705998795
"Rab7b",1057.66033274058,-1.13334380280609,0.339764122640545,-3.335678
27585233,0.000850915757001094,0.00520751896735949,-5.95872701969767
"Tln2",5289.32876550839,1.41495414775287,0.257209575636832,5.501172124
90844,3.77274669610714e-08,2.16994144780817e-06,18.4521487278342
"Tnrc6a",10375.771634792,0.435302519976992,0.303572848469639,1.4339310
0592304,0.15159195265101,0.270325580097805,0.569431499399317
"BC004004",5082.41984826731,0.879756330445999,0.222069701510397,3.9616
2251969709,7.4442166188137e-05,0.000783732913788497,6.29152660607476
"Zfp608",839.600847673817,-0.527479934521023,0.168450781858693,-3.1313
5937216075,0.00173999096168893,0.00899903017798692,-2.48476726930972
"Kcnt2",486.739922862782,-0.749451304724942,0.405539097940322,-1.84803
711536398,0.0645969737715001,0.141105959511171,-1.46760865936828
"Map1b",2578.9643918581,-0.208513193447374,0.418918601852803,-0.497741
548179425,0.618666219466425,0.736951980294355,-0.0636450126090467
"Klrc2",44.1480512744131,-1.35456122886706,0.308897842799899,-4.385143
05114304,1.15909581223639e-05,0.000191201777893276,-11.5979987676826
"Suclg1",9006.847354,0.799198792803567,0.442935358289232,1.80432376383
395,0.0711805665258885,0.152072754630574,1.50520798840486
"Swt1",1529.45787821745,0.608349237432064,0.302765514794757,2.00930821
94135,0.0445044567687383,0.106328623707494,1.36344493843688
"Trim30b",77.1221148892562,-0.813657847011743,0.454025985055172,-1.792
09532888931,0.0731176970179085,0.155333281374502,-1.5151792149435
"Repin1",1960.64538346957,0.495537133675705,0.177527314033484,2.791328
96463606,0.00524920918468369,0.0206515497746091,1.92266669163799
"Traf7",3595.72641878164,-0.284602913827466,0.166126101695794,-1.71317

397400093,0.0866805381492484,0.176700375361419,-0.493302166607441
"Gpr25",1.87807909077462,0.512398851888092,1.3864576493338,0.369574110059764,0.7116998445558,0.808714002514657,0.108787371645266
"A630001G21Rik",407.821174158698,-0.610104772122418,0.353440962029038,-1.72618580659107,0.0843139906655818,0.173120702400749,-1.06998114368597
"Zfp212",814.573878555822,-0.186163910261834,0.106613022473403,-1.74616482998853,0.0807823142172259,0.167545320987924,-0.332582084683879
"Oas1a",891.863709064676,-0.98054548205051,0.365374907167538,-2.68366946611606,0.00728190596490097,0.0265686007678965,-3.55744370166306
"Grk4",701.918942786372,0.488274316113101,0.209839654108314,2.32689249411871,0.0199709878006681,0.057393110326336,1.39540548296019
"1700030K09Rik",485.556344329925,-0.0104238358719437,0.25935236387783,-0.040191790489536,0.967940222583658,0.980359381846977,-0.000206767816806383
"Nup107",550.318698600355,-0.287144602249091,0.2682656952378,-1.07037391416952,0.284451036182379,0.42854476151167,-0.243314874701908
"Atad2b",1252.1245258242,0.0432770294786043,0.134873907480805,0.320870287566654,0.748308691384434,0.836324823146492,0.00773525821739153
"Best2",3.50401458042622,1.13676347673937,0.743501476677422,1.52893237256148,0.126281209929737,0.23523751134136,1.64507816542321
"Cysltr1",191.01628838464,-1.36866809633366,0.370799468017913,-3.69112745401119,0.000223262222119387,0.0018445622063067,-8.61646822592831
"Gm9892",2.10423388716889,2.3739358941992,1.42704155232426,1.66353662956254,0.0962050649417167,0.19152607272142,3.92347822691824
"Sae1",2360.54487318267,-0.433381294682968,0.174274322534734,-2.48677652783067,0.0128906344359611,0.0410890289737402,-1.3833592154645
"Junb",4676.76828725576,-0.331417459274128,0.21018634759603,-1.57677919172514,0.114846341167081,0.219035211234123,-0.503264961663333
"Tas2r137",0.515344611972341,0.872827577177397,2.92205320361615,0.298703519873368,0.765166271134417,NA,NA
"Reep1",54.8369827256509,-1.15149552530143,0.337944932682368,-3.4073466234918,0.000655977641391472,0.00425349253470267,-6.2871826801121
"Nrk",4.9080078844037,-1.22841479932529,1.16185245700128,-1.05728984082523,0.290379328336447,0.434155060494137,-1.02493221975708
"Dnah6",12.4947703477404,-1.73120097808395,0.534130719234683,-3.24115598624333,0.00119046023400434,0.00671826154634348,-8.66107013583976
"Prkcb",732.415230598501,-0.832711002860649,0.385189067698388,-2.16182408248585,0.030631738467182,0.0799006871749987,-2.1042364105589
"Ubxn8",1553.92441593909,0.663696528472961,0.181005702366743,3.66671612990524,0.00024568527275489,0.00198031064414007,4.13118007392757
"Gm9894",28.5920922030741,0.171721423329443,0.347787916186168,0.493753277033127,0.621480434128125,0.739601166054088,0.051798771855813
"Lamb2",6826.0837230923,-0.451693638027536,0.193096989783002,-2.33920600489494,0.0193247744637732,0.0559789708059203,-1.30213301548743
"Smarca5-ps",10.9050248177644,0.476833275136222,0.544255281567194,0.876120620755707,0.38096446371777,0.525879300075687,0.306452906844816
"Cyp2j6",1893.11662559447,0.702555399265159,0.181410004209557,3.87274892763685,0.000107614672027495,0.00104966026772991,4.81903032655647

"Msl1",7645.53414545882,0.0493273747101625,0.134815007515602,0.3658893
44362896,0.714447647841351,0.810574911557037,0.0103593167297155
"Senp7",896.396606621338,-0.0274720998955271,0.133211960562453,-0.2062
28478130141,0.836612443610882,0.895835544265197,-0.00302188778040053
"Prkg1",396.617543956631,-1.00987162164776,0.237751772052827,-4.247588
20061865,2.16084105887992e-05,0.000305304920043773,-8.17410241834263
"Arhgef15",1480.77676385193,-0.280003597682926,0.226507575165463,-1.23
617763105002,0.216392543548322,0.351719057527942,-0.292582074443516
"Bpi",0.695900470081077,-1.52920929221729,2.36531693916917,-0.64651348
2778521,0.517946868250518,NA,NA
"Rnaseh2a",1082.96449509779,0.107830974853588,0.156411753018672,0.6894
04554149556,0.490568718311524,0.627055780099521,0.050326848734703
"Ctif",634.145394746902,-0.773050536787931,0.292478542902214,-2.643101
70967444,0.00821503537063668,0.0290438367464504,-2.73578640740517
"Fbxo31",1080.49595391362,-0.266545183982784,0.170487007343945,-1.5634
3400083884,0.117950548032763,0.223520243516651,-0.399352199905136
"Glis3",1707.82367516633,0.581784059927507,0.31164618036274,1.86680953
140623,0.0619281909919452,0.136727009604437,1.15761587067818
"Rnf157",190.480483785879,-0.576282250488857,0.329650319198623,-1.7481
6227052288,0.0804359327548881,0.16695122597986,-1.03157609936804
"Cpvl",2.49634858980455,0.370070884084969,1.14245566010078,0.323925817
875788,0.745994197101412,0.834806879332208,0.0668180978199261
"Gas1",2332.20538572043,-0.825010731985493,0.271191568144839,-3.042169
55427193,0.00234879549470868,0.0113027530450855,-3.69828299263629
"Mrpl35",1451.2275576425,0.365136590544387,0.349080211605301,1.0459962
4500411,0.295562766058828,0.439939479999422,0.29820266019105
"Cyp2f2",472.525760965837,-3.58777689973775,0.800542807523855,-4.48168
026246472,7.40576409444175e-06,0.000134073898155455,-31.9926351076408
"Ube2ql1",72.2488402949692,0.117331507977068,0.369155972610771,0.31783
7219718451,0.750608416248837,0.837870343449178,0.0207549947711971
"Uba2",3032.45861038563,-0.17077162206562,0.0946486788537989,-1.804268
41804529,0.0711892382447612,0.152072754630574,-0.321630628124842
"Hrh1",42.2106297567532,-0.861454230074159,0.360156152195337,-2.391890
91960016,0.0167618231861568,0.0502030765719946,-2.5771945018935
"Creb5",660.464744738174,-0.306103861954013,0.590053359289258,-0.51877
3187432958,0.603918917668451,0.725029927756878,-0.0984253536826065
"Krcc1",4389.27579975544,0.264338345967405,0.175717613567378,1.5043360
7992318,0.132494853546273,0.24357765929146,0.373330189499556
"Cntn2",12.5367688613799,0.473333320055491,0.622355439088518,0.7605514
31427545,0.446925038676393,0.587959274280569,0.251386187872141
"Sv2b",4.20084054913786,-2.52557032671239,1.0102180329904,-2.500024988
899,0.0124184546530542,0.0399496830267696,-8.13267624489724
"Spink2",2.5094112829785,-2.70652407058353,1.27728348777104,-2.1189689
6538343,0.0340930898077035,0.0867816011841518,-6.61572093021081
"Gm6180",0.504287576072894,-2.37496424187981,3.64242411569496,-0.65202
8475115308,0.514382803150565,NA,NA
"Aph1c",1802.99827178188,1.54650585839034,0.404525753143938,3.82300965
110636,0.000131832617424719,0.0012316590199076,10.3606508650723
"Cd8b1",175.161685830311,-0.612184905691713,0.453782613673041,-1.34907
08706015,0.177314203554516,0.303909312667541,-0.729128100106899

"Brsk2",9.02065391795375,-0.300686150136984,0.663313448313004,-0.45330
9292766059,0.650326042233755,0.761839532052016,-0.0817924461019689
"Adh6a",28.3877209023067,0.0410781591296923,0.475130450112407,0.086456
5912792456,0.931103461953531,0.957120165603645,0.00180030497907669
"Jam2",1367.18956970544,-1.55471148670607,0.27176289333492,-5.72083799
825479,1.05999931189114e-08,8.3338171706667e-07,-21.762500113123
"Clec12a",1029.91656517938,-1.11729846804649,0.402071340238399,-2.7788
5627805259,0.00545506550438345,0.0212215114009021,-4.30466041378411
"Cfap300",164.360668344468,0.159513265528613,0.270472682323918,0.58975
7398632887,0.555353307507007,0.684727380155028,0.0604131776788058
"2700081O15Rik",2579.53807640286,-0.850199258492838,0.205286350193268,
-4.14152844401205,3.44999078289208e-05,0.000434825889551803,-6.5810225
0505499
"Lins1",432.514330080554,-0.0587053709952842,0.131191571136244,-0.4474
7822201411,0.654529808676862,0.764696639078522,-0.0157492464548891
"Myh7",14.9838091895039,0.871990841530988,0.879254024166309,0.99173938
0843656,0.32132467523618,0.466645949482722,0.664617857339559
"Tmem248",2513.58139286789,-0.258930148658801,0.164297562603195,-1.575
98289686232,0.115029744849111,0.219222169057939,-0.392970413280969
"Gpr141",55.0560099407645,-0.154689021302603,0.471605935631269,-0.3280
04822703395,0.74290801618658,0.832483936758962,-0.0283608943822302
"Yap1",5574.0720590177,-0.124726925640676,0.0880230165030297,-1.416980
81474387,0.156488536738754,0.277005309971151,-0.160114274790772
"Fank1",19.8655028703886,-1.18557418719926,0.560657612033295,-2.114613
55692581,0.0344628972530948,0.0874034301041679,-2.88950601094847
"Socs3",1976.56978772564,-0.713832151129427,0.438697285460011,-1.62716
336478105,0.103702406215212,0.202827717562866,-1.13884663054314
"Chmp3",5179.18309055166,-0.111982301709355,0.13526232304006,-0.827889
83060856,0.407732894658647,0.551468922034843,-0.0666484833930961
"Rnf26",842.879314017801,0.0179914624610076,0.161585677703096,0.111343
175439508,0.911344219184651,0.945417753576699,0.00100983167585048
"Supt7l",867.316241928307,0.0764998213405051,0.126191978252406,0.60621
7783411651,0.544370145350315,0.67459826452526,0.0301132312379871
"Mapk11",905.064383116834,-0.552116106155328,0.26283544089378,-2.10061
513880259,0.0356747640315996,0.0897878596989969,-1.33076849032752
"Ptprt",20.5973355845395,-3.36664939471774,1.01562875652718,-3.3148425
279229,0.000916947635415894,0.00550548320347427,-17.5133462999728
"Olfr352",0.579279898712118,2.44392492585724,2.1932281572297,1.1143049
1980561,0.265148397094193,NA,NA
"Spag16",2.46902748544529,0.38604242374192,1.16169303960067,0.33231018
0557354,0.739655053324594,0.830365858459405,0.0717609945492259
"Fes",2140.51209800457,-0.861001954634335,0.259352755726978,-3.3198103
1865617,0.000900786292748986,0.00543594278428342,-4.48988610013457
"Daw1",47.6660826710803,-0.747711511655888,0.383644784958332,-1.948968
26692717,0.0512992144649394,0.118693732483373,-1.59352933860797
"Gpr21",11.4750232081473,0.193078834330801,0.528785293119959,0.3651365
43778648,0.715009483359726,0.811034657748805,0.0404392982168156
"Cdh22",12.6477577708781,-1.62592215333723,0.653015208744306,-2.489868
73745826,0.0127790281741394,0.040806664156464,-5.20117843024607
"9030619P08Rik",316.688058999259,2.4573759085158,1.10345894180139,2.22

697539113159,0.025948918524487,0.070457041274258,6.51880909815978
"Rpl18-
ps2",2.63498924284781,1.16311261954763,1.08458471189588,1.072403664545
92,0.283538762713873,0.427566805521662,0.988232510605427
"Bcl3",1194.78517543873,-0.92467819046667,0.312206365203209,-2.9617531
6561792,0.00305892901999183,0.013726811975098,-3.96539390985743
"Mterf1b",220.476067225044,0.259239672606489,0.210084565622117,1.23397
77167295,0.217211207771932,0.352550081506463,0.270273584924871
"A830005F24Rik",7.36278630315088,-1.384666737144,0.811290106506799,-1.
7067467309641,0.0878691373078261,0.178707879339522,-2.38439995028967
"Gm609",52.5300541781247,-1.37260010103571,0.601231741354867,-2.282980
10005689,0.0224315405856879,0.0629132292404499,-3.79661025376067
"Spaca3",0.44373828255743,-1.48490811800342,3.84058370623116,-0.386636
051075838,0.699025664755136,NA,NA
"Mllt11",1054.378525539,0.656408835330786,0.215817444388269,3.04150036
245385,0.00235402281924892,0.0113185027086227,2.94157573434704
"Cib4",1.45935099734524,-1.79127010758002,1.92928681973892,-0.92846231
5324597,0.353167807015044,0.498058360383214,-1.24858336781729
"Prx",271.311563574807,-0.642332392878557,0.215744895756774,-2.9772773
5632136,0.002908207863259,0.0132085341061784,-2.77930297886655
"Arhgap20",67.3940041763524,-2.00749682866937,0.461736500679218,-4.347
71092542246,1.37565751586717e-05,0.000218069676783562,-16.924595338873
6
"Styx",1449.88320999555,-0.0723465177507111,0.183274255570839,-0.39474
4572964574,0.693031404065531,0.795100106882596,-0.016588134221371
"Zfy1",0.444218496146595,0.62475565855248,2.6722363674328,0.2337950587
62964,0.815144085818475,NA,NA
"Btn2a2",0.370590567642787,-1.94430622618768,3.78766728909947,-0.51332
5505591054,0.607723645441297,NA,NA
"Raet1e",37.2293280182824,-1.30331375000336,0.481816037272557,-2.70500
284170926,0.00683038051477013,0.0253047234043298,-4.79197734370229
"Dand5",334.062337939615,0.168493406308023,0.138014591421851,1.2208376
2718256,0.222147504118856,0.358012281548298,0.173074402876794
"Ceacam3",2.58427300114861,1.52804187193562,0.907382602025321,1.684010
54695666,0.0921796304517411,0.185462638999902,2.57460053752166
"Olfr212",2.70806094474602,0.785183174533656,0.907548005498252,0.86516
9853028968,0.386945569353857,0.531277002412613,0.496606162306617
"Ndfip2",2502.20062103277,0.228936212221569,0.211478584573164,1.082550
33332874,0.27900806273141,0.42272897716505,0.197119578046202
"Dspp",4.68276062287911,-2.33060162157371,1.43789314934522,-1.62084479
130803,0.105050929613516,0.204644122849868,-3.69745937348836
"Aldh1a1",738.738149654566,-1.52338918820894,0.358570754320692,-4.2485
0373281274,2.15203061616516e-05,0.000304722534161134,-12.3335247944807
"Trmt1l",2499.25969847019,0.317418832075993,0.184861467778655,1.717063
24682035,0.0859676165677651,0.175646713595126,0.552080420008005
"Ddx10",914.926360892884,-0.150801463421688,0.125231742327111,-1.20417
92329918,0.228520310801855,0.3650109789477,-0.15198191413585
"Rab4b",1927.45217126585,0.0386255029353079,0.113511025840298,0.340279
745067683,0.733645869121436,0.825806901981859,0.0073927013912071
"Pom121",1620.7940399753,-0.327870218318584,0.0959745242890469,-3.4162

2134360505,0.000634966125416261,0.00414343022535685,-1.79877184665399
"AI854703",1139.7724854388,1.16310964867439,0.377532011094818,3.080823
91557064,0.00206428686914144,0.0102337821954029,5.32943941955435
"Slc22a26",551.181396849016,2.36762423033776,1.10508402780148,2.142483
44087287,NA,NA,NA
"Nrgn",210.800939355838,-0.37049625455783,0.313485837031736,-1.1818596
2742656,0.237261403620143,0.375436036989575,-0.362963014676745
"Sec61b",2836.94974411828,0.0679376946085779,0.210429535467355,0.32285
246677797,0.746806976098407,0.835398055425001,0.0122183874805493
"Slamf8",455.133113572505,-1.4496467303099,0.496835883816725,-2.917757
70939414,0.0035255818012509,0.0153065673482053,-6.0587598099024
"Gatd3a",6429.57451930927,0.43707736638173,0.407463632133997,1.0726782
2674784,0.28341551275936,0.427414004778561,0.371516709555908
"Dis3l2",870.291316596399,0.414013031976704,0.089810177751666,4.609867
63795848,4.02925420041855e-06,8.43740065777616e-05,3.8835462328668
"Ficd",441.633486412742,-0.187205870600429,0.157565705964572,-1.188113
04436082,0.234788875836174,0.37273355325303,-0.184751872905467
"Tarm1",159.313159435861,2.30477360951487,0.592566554354362,3.88947636
780827,0.000100460750460609,0.000994306453036015,15.9339719034941
"Zfp943",982.509912934084,-0.09415882154352,0.115087136752516,-0.81815
2438234688,0.413270149363226,0.556833762164617,-0.0551289105514563
"Epp13",0.229026995095479,-1.56584971755908,4.76246957708833,-0.328789
44258084,0.742314843447175,NA,NA
"Rxfp2",10.1221615155537,-0.306500854185009,0.6364823164901,-0.4815543
90819869,0.630122532704088,0.745879622551258,-0.0898633090026381
"Atp6v1e2",0.72030912745275,2.01262664696318,2.99125397938293,0.672837
098031499,0.501050933430461,NA,NA
"Trim50",0.47963238652201,0.909935650126202,3.07466953702999,0.2959458
37159841,0.76727144509809,NA,NA
"Zfp952",691.289768648955,-0.13594158789442,0.115767613506515,-1.17426
267828152,0.240289827396833,0.378630277564526,-0.132025800102606
"Cacng8",54.7286303173468,-1.41566964575693,0.510619369596595,-2.77245
582531535,0.00556350700245511,0.0214976733123008,-5.43590326620201
"Phgdh",1081.11341243807,-1.14688933810231,0.277611555783373,-4.131273
76800286,3.60758625399943e-05,0.000451747998841715,-8.83378444820435
"Adamts18",9.38706139258624,-1.90122200634682,0.884262249309462,-2.150
06578402676,0.0315500116281604,0.0816522560981078,-4.76310454955591
"Cbx7",3301.6860545593,0.0973563763414444,0.135089750176207,0.72067922
4104389,0.471106897839169,0.609087082691342,0.0482687100332751
"Hunk",567.138943307736,0.524890396866183,0.331192698549052,1.58484893
889786,0.113000660171719,0.216326644283572,0.803589231407421
"Mapk14",4569.33228352266,0.239822356756946,0.0998974863490227,2.40068
459699829,0.0163644345710689,0.0493463880709405,0.72159926029695
"Adamts19",0.992184430240734,-0.0853726843548453,1.74544058918575,-0.0
489118248331053,0.96098956528835,NA,NA
"Thoc7",3902.00449999785,0.889673267460119,0.143732196380276,6.1897980
4014326,6.02413481982149e-10,1.05818541186378e-07,14.289522848191
"Ggcx",1177.09552515946,-0.220476002106074,0.164777817535948,-1.338019
91920408,0.180889943575524,0.30868126376996,-0.259157644316168
"Hhipl2",20.5355498896423,-0.110299205625411,0.491220542364899,-0.2245

4110956841,0.822336282625909,0.886096316034262,-0.0133384416229582
"Hs6st3",0.491854705783106,0,3.69637497973905,0,1,NA,NA
"Tg",11.9515162375278,0.119073125418892,0.650063360658354,0.1831715685
3495,0.85466340194826,0.907618501682864,0.0115418939094337
"Kdm3a",1727.81563048016,-0.0726360706162199,0.112521704402709,-0.6455
29420317521,0.518584159095934,0.651840764185954,-0.0310849676824159
"Tnfaip6",17.4929337131158,-1.5776659094758,0.490097843558696,-3.21908
355690786,0.00128601005846434,0.00712954908158592,-7.79920292305331
"Tcf4",7505.24896415653,0.0365078123620828,0.270908309061129,0.1347607
70124054,0.892801023735134,0.93382853574598,0.0024941384685407
"Usp21",2380.66830430887,0.185177991067745,0.183327232990921,1.0100953
7997508,0.312449595488113,0.457505423011958,0.144802993099902
"Trim60",7.42909571757771,0.282326853489048,0.791998608161985,0.356473
926316931,0.721485675099258,0.816039382989951,0.0573949775192952
"Nrd1",5813.30250573005,0.366670980595326,0.155888459736239,2.35213678
559483,0.0186659094262454,0.054491376252872,1.06690725432622
"Kcnip1",4.04974156591002,-2.20924194555266,1.52573957757621,-1.447981
01722068,0.147622359587343,0.26467501307899,-2.93664053683234
"Lgals7",10.5801766582011,-0.603128707507461,0.545987674142154,-1.1046
562698601,0.26930855882693,0.411550927189238,-0.535471240929432
"Cstf2t",2010.96654026966,-0.116748776697901,0.17969493183427,-0.64970
5450822491,0.515882502309476,0.649579326590757,-0.0503689615615807
"Gm4759",22.0867088739355,0.479508789981303,0.565997326394572,0.847192
676749543,0.396887734736053,0.541382191960512,0.294240880457015
"Shisa7",444.098209806004,0.660062904325555,0.559008770854331,1.180773
78878472,0.237692599296938,0.375813355586418,0.645978913459717
"Ebf4",369.099071400232,-0.6327545735535,0.356363774369986,-1.77558612
592468,0.0758011584437691,0.159435920964036,-1.16180901658676
"3110082I17Rik",276.169782588767,-0.875068315516586,0.129464075331719,
-6.75915935192404,1.38794676551591e-11,5.10607283660929e-09,-16.707535
1835611
"Smagp",2274.61337314384,0.747649053058347,0.260414247580747,2.8709990
3328647,0.00409176776298518,0.0170860383883816,3.04255304953679
"Ier2",2129.27516575149,-0.222367992433886,0.211161661172749,-1.053069
91429646,0.292308950400769,0.436203881441466,-0.184486610259026
"Eif3k",4989.02660739347,0.149975325555579,0.169936439131915,0.8825377
67189293,0.377486065586541,0.522706008579389,0.0972944075055995
"4930563E22Rik",57.1167827436127,-0.550494314303335,0.226453471820456,
-2.43093784289515,0.0150597978631545,0.0462830873967723,-1.69165728450
527
"Tanc2",2831.02161887181,1.16848445702658,0.326418125998082,3.57971682
31811,0.000343966746721453,0.00257651311086242,6.96570777877981
"Zfand2a",872.342331220639,-0.467905483749092,0.192052507596766,-2.436
34144434868,0.0148366723115148,0.0458382881841097,-1.44238207850296
"Fate1",0.219476833423174,-0.0674470221364578,4.7847199059589,-0.01409
63365593165,0.988753123168312,NA,NA
"Zfp472",342.679888009211,-0.333268784711308,0.115232875276768,-2.8921
3285627784,0.00382636104586292,0.0162364282202906,-1.37323332940899
"Rpia",1605.15197330201,0.483112317845133,0.145432351153125,3.32190406
064787,0.000894054291830741,0.00541038813845927,2.52157307211308

"Sh3pxd2a",5041.60507859096,-2.63732187207559,0.443707550262155,-5.943
8291516955,2.78439809646105e-09,3.15640663283707e-07,-39.4771781373049
"Tll1",48.8655608183367,-0.883626407217007,0.366884000046885,-2.408462
63970107,0.0160198658458171,0.0485551599971619,-2.67301831142212
"Dennd4a",952.025090274454,-0.735857858223715,0.163672010496267,-4.495
92973161712,6.92666104406166e-06,0.000127051775199543,-6.6013189427062
8
"Aldh7a1",9075.20738964396,1.27799752091967,0.299028297237708,4.273834
72642974,1.92139481115137e-05,0.000279786079371392,10.4559178657121
"Plxnb1",5498.94377346272,-0.207081592773563,0.20212860329189,-1.02450
414934358,0.305597211982588,0.450139374674274,-0.165292117883872
"Gper1",109.305189837227,0.219299727852553,0.262936742142694,0.8340398
7615219,0.404258513591147,0.548100444892579,0.131864206008736
"Krt42",3.3373200623072,0.297261916334539,1.03992947910874,0.285848148
654564,0.774994428689532,0.854443956493554,0.0467605969557899
"Tgm5",36.6623182623517,-2.81051861154267,0.753273699280639,-3.7310722
7057928,0.000190666512594556,0.00163339879726215,-18.0353572944276
"BC048403",287.751676440849,-0.166031143450082,0.159042861999194,-1.04
393961076306,0.296513330294348,0.440897149320796,-0.135970151001007
"Dpep2",312.008667023555,-1.08941079807486,0.420757905812092,-2.589162
99141717,0.00962095440478066,0.0328125536093079,-3.72245580421366
"Mast1",22.5222570266574,-0.384319404509929,0.389349876182424,-0.98707
9816945575,0.323603511291944,0.468489847280244,-0.291406671267493
"Nebl",585.938730330227,-0.408596857098829,0.409559085795176,-0.997650
574166903,0.318448828034189,0.463505169379712,-0.314185544324521
"B430305J03Rik",2.4092874866949,-0.594723312308319,1.22453276629563,-0
.485673661561081,0.627198560646341,0.743682652364532,-0.17612188367480
6
"Dusp7",1468.85553661314,0.201408262697324,0.183045219063782,1.1003197
1185843,0.271192846538201,0.413674868050863,0.177778029529683
"Spinkl",0.803055255277063,-1.26677951155413,1.98133284565746,-0.63935
724597236,0.522590555475749,NA,NA
"Tmem39b",581.893339723713,0.335858859175601,0.168885346546353,1.98867
969331738,0.0467365687792957,0.110443536305746,0.739981324441401
"Gm6457",0.443466371519398,2.74675837455538,4.52716168400014,0.6067285
78805336,0.544031052865392,NA,NA
"Gm5114",0.14728986473751,0,4.7847199059615,0,1,NA,NA
"Ptrh1",249.88962866125,-0.610210329217064,0.276375759408284,-2.207901
04936669,0.0272511716850206,0.0731806135382821,-1.59559306267302
"Sox14",0.446274309725509,2.33816462212349,3.62566291435865,0.64489299
6771348,0.51899653040558,NA,NA
"Chd8",5189.08648220366,0.0729719564274048,0.208258698698202,0.3503909
17083142,0.726045342001507,0.819664876286572,0.0145111820724687
"Oas1f",6.23734967822022,-0.123107798734922,0.824563039234262,-0.14930
0651226433,0.881316401428391,0.925956848531672,-0.00947039307222881
"Chchd3",5749.0464994532,0.191201306232101,0.390225259162842,0.4899767
55072928,0.624150347613954,0.741692069586597,0.0571349889533617
"Lysmd1",699.899338269765,0.20776034546277,0.174182635917406,1.1927730
0155962,0.232958286434112,0.370249483933185,0.206426154162631
"Ubxn7",2271.82654463925,-0.0868712782531174,0.131305354418858,-0.6615

97378397853,0.508229288127198,0.642747349143046,-0.0383974139965967
"1700016K19Rik",13.5717586495925,-0.418838191516164,0.614178308310675,
-0.681948850763222,0.495271294316428,0.631245894664774,-0.192690615423
39
"Krt16",1.84805884758714,0.587405899861807,1.28886042417809,0.45575602
2019529,0.648565431749073,0.760438953740984,0.16086665179901б
"Exoc6",2205.2289354954,0.747048325742262,0.186862147744515,3.99785796
513296,6.3918284494863e-05,0.00069858672145787,5.42839022779966
"Grwd1",649.2730502294,-0.177426230057433,0.108589164561009,-1.6339220
4714632,0.102275219747607,0.200720909374821,-0.284918110017465
"Camk2d",2233.37950340866,-0.353549791171872,0.164664439563074,-2.1470
9254839717,0.0317859144242106,0.0821420489653091,-0.88362884071706
"Ppfia2",31.1953056493846,-1.79674047700583,0.463671612572396,-3.87502
799025741,0.000106612444261028,0.00104144761432942,-12.3384748618174
"H2-
T24",303.651581979106,-1.13563355671014,0.2696804268856,-4.21103440774
321,2.54204020957713e-05,0.000346061422803521,-9.04974374982394
"Nudcd3",3790.42192029024,-0.159517114356475,0.16107681566685,-0.99031
703411867,0.322019183180881,0.467132656324739,-0.121415175614759
"Txlna",3141.82690247087,-0.195991935731696,0.0840449998877064,-2.3319
8805394209,0.0197013196160176,0.0567745092925083,-0.5622357617664
"Lipg",163.282405433596,-2.19652220812543,0.701895132357547,-3.1294164
9950995,0.00175153845774262,0.00903478752620785,-10.3383114896407
"Adgrg4",106.968582928848,2.53163979792177,0.831927554731702,3.0431012
694828,0.0023415351956439,0.0112812585235149,11.3534234019975
"Gm9925",10.9945592783996,0.130953960700165,0.641708407553493,0.204070
819641315,0.838298157037997,0.896655738983316,0.014284887841893
"Slc51b",878.128782029863,1.98325784709251,0.763423685429729,2.5978468
9019197,0.00938103127605134,0.0322083725691934,6.81353953590728
"Fpgt",909.993776138782,0.105901915823893,0.153239890711714,0.69108582
1922984,0.489511609641193,0.62612722474937,0.0495834563979704
"Aym1",0.291330136303653,-0.060738045090329,4.78471990595801,-0.012694
169415162,0.989871790225381,NA,NA
"Srcap",2658.93228496166,-0.289244437226818,0.256490744334418,-1.12769
931709385,0.259446926173807,0.400742711340105,-0.264495432255071
"Sh2d4a",2005.0954729111,0.0565325436117702,0.201855301947517,0.280064
695186797,0.779427870376543,0.857489398894196,0.00869167861475686
"Slc39a8",1493.65299894992,1.16494801987119,0.399748455833317,2.914202
67638742,0.00356598310205109,0.0154158870270921,4.86057864811949
"Ech1",15455.5980179183,1.03819015331486,0.35987545079429,2.8848596119
1142,0.0039158824120295,0.0165407008816619,4.25858459733572
"Mat2a",17320.9389125508,1.10184482305561,0.277907440638749,3.96479065
304298,7.34603886948898e-05,0.000776698269304374,7.88971425102383
"Kdm4d",5.5935343746795,-0.576644811350934,0.654378058119368,-0.881210
493224921,0.378203900851858,0.523177036858622,-0.373570904488616
"Nanp",548.245219483229,0.879745728578872,0.184355642537303,4.77200326
754773,1.82402544418744e-06,4.41251217255171e-05,8.8225135737873
"Cyhr1",6673.5909593475,0.533327473539316,0.154849636587796,3.44416354
659593,0.000572829088035107,0.00382630759142687,2.96842339385185
"Shisa6",15.6979093950751,-1.6502413349964,0.750935103041855,-2.197581

8260615,0.0279789195092747,0.0745507491397438,-4.28448063391178
"Cnn3",7712.05907874918,0.591666672496535,0.179762201409255,3.29138532
93859,0.00099695252354869,0.00586028950984394,3.04090415768325
"Adnp2",1007.85766270796,-0.655623658861455,0.108216615147671,-6.05843
805007945,1.37449663286185e-09,2.0411243243503e-07,-10.0996168424651
"Stum",53.2014280713896,-1.97866938880789,0.602808819027902,-3.2824161
2655687,0.00102921598582393,0.00601007885342767,-10.1195433041119
"Lgals4",1889.8205443576,0.859500909944008,0.264786900656829,3.2460099
3407127,0.00117034755480387,0.00662970267971117,4.3114244424086
"Pde5a",733.654645606306,-0.882523609682666,0.282902505961214,-3.11953
266968819,0.0018113817259441,0.00925775121689594,-4.13223509863878
"Cd8a",543.836536449786,-0.732527960325067,0.452705456586169,-1.618111
62129351,0.105638544462244,0.20548047744658,-1.15915535752934
"Zfp14",382.062049692772,-0.0916735604680112,0.214855229700286,-0.4266
75955693012,0.669615365873399,0.776320632881409,-0.0232107973706683
"Tusc1",328.495274401756,0.338681496557649,0.240015671626841,1.4110807
6094384,0.158220802139485,0.279083517289278,0.432240294556321
"Tdrd9",4.22753753073442,0.042260682989899,1.30729378370563,0.03232684
45980885,0.974211401495105,0.984077077375299,0.000678328518762072
"Mill1",4.87934168053422,0.61985173266136,0.886278732800166,0.69938689
6832062,0.484310274347795,0.621828113343429,0.294486332552379
"Ndst1",4860.82930840025,0.249409505316497,0.179089671876049,1.3926515
2872198,0.163725192791173,0.286249794242608,0.311983966573078
"Tmem179",15.0784703690907,0.0554696630242075,0.891927149804669,0.0621
908000405137,0.950410888813454,0.969301784186878,0.00172950397682521
"Sirt5",1975.53603857911,0.839061425499259,0.264932959292443,3.1670707
4778519,0.00153982852046504,0.00822113266484866,4.02837357936966
"Nt5dc3",595.703654729475,-0.390308712020759,0.173855247722065,-2.2450
2117212319,0.0247667734165062,0.0679378936515248,-1.04960309114672
"Rdh18-
ps",10.2454239847402,2.02154139076841,0.61467634242673,3.2887899716254
,0.00100619077547389,0.00589859889400323,10.3766536935027
"Tceal5",41.9351790441132,-0.603462881961653,0.426916395316898,-1.4135
3878319362,0.157497366348995,0.278184948733826,-0.772112112445683
"Olfr279",5.47644845698091,1.2464115814311,0.877489176739768,1.4204295
7847301,0.155482656759901,0.275675260662412,1.60604083117327
"Klk13",2.71571877993052,-1.2623822352496,1.3522875252137,-0.933516143
358723,0.350553539326559,0.495477498516476,-0.886486890784346
"Ercc6",1223.32126476219,0.260842856153334,0.175850254786007,1.4833237
3172137,0.137988420062484,0.251491214752114,0.360053711727793
"Rdh19",4.06515492803903,1.26236279281521,0.836448765767621,1.50919320
402932,0.131249416427169,0.241743918524177,1.79239901280545
"A930004D18Rik",179.348404773996,-0.334418496098351,0.352287967626733,
-0.949275952713446,0.342480280684343,0.487066412310964,-0.240565548687
894
"Pkp3",1540.52630255095,0.291994458388088,0.190410127137594,1.53350277
518111,0.12515202339808,0.233781773540502,0.424375165619106
"Iigp1",3029.89497287331,-1.09593399378023,0.542583137441227,-2.019845
28850003,0.0433994378265797,0.104469412190451,-2.47556250970947
"Skida1",249.185444612318,0.423656387985225,0.283291949418862,1.495476

27051988,0.134790188356916,0.246952138848571,0.592509187662338
"Utp18",1002.12804239863,-0.101183841928993,0.141664901055502,-0.714247785973118,0.475073972823439,0.612631767335411,-0.0495791950978457
"Capn12",24.75527272239,-1.89442247228253,0.744369273673461,-2.54500358798202,0.0109276615400252,0.0363162680598962,-6.28093778166421
"Fbxw28",0.157863633167365,0,4.78471990595081,0,1,NA,NA
"1810037I17Rik",4721.21368942876,0.223453279854679,0.220700093823368,1.01247478414538,0.311311097315047,0.456421067407232,0.175263234263003
"Slc25a40",600.088434233869,0.154617819651322,0.105346015931228,1.46771397365668,0.142181933415966,0.257238873317668,0.209932369001336
"Try4",1.44159808850869,1.75311515496299,4.01219438763397,0.436946714338241,0.662149992484934,0.770736108499268,0.456527377656072
"Skp2",329.300839569597,-0.152457753384509,0.247538970550381,-0.615893946094759,0.537964517384689,0.669254907163061,-0.0612255494727246
"H2-
T3",0.942862835333436,-2.09526822994977,2.13683668758549,-0.980546731588227,0.326816313866041,NA,NA
"Umodl1",0.0753649833681825,-0.0469963211071692,4.78471990596623,-0.00982216765678757,0.9921631700829,NA,NA
"Adm2",59.104192866428,1.8720419168768,0.622898418092816,3.00537272611575,0.0026525558121668,0.0123394484712482,8.22753802817338
"Vmn1r236",35.0412762832875,0.515892786659366,0.434015005201479,1.18865196013183,0.23457665201962,0.372457293247438,0.50951268729328
"Krt15",1761.37280759763,-1.33405881640062,0.759078502267389,-1.75747147681794,0.0788374794635467,0.164438722141659,-2.40826603851232
"Syne3",168.949630643136,-0.768169506017357,0.32023901909545,-2.39873800571565,0.0164516810579648,0.0495203445829637,-2.3086348881724
"Fam83e",27.9180265184409,-3.23548250418453,0.817502034445507,-3.95776691415708,7.56537276194708e-05,0.000794771757071359,-23.0931126617474
"Spock3",43.0765351024857,0.879145377385285,0.738236524039529,1.19087223235004,0.233703746872724,0.371282950910485,0.871049183550494
"Ceacam10",0.238306107034699,-0.547854530422708,4.78471990595988,-0.114500857143236,0.908840766966445,NA,NA
"Klf1",51.5811799450938,2.1382143707937,0.542925254442243,3.93832181004424,8.20534966757812e-05,0.000849192716658377,15.1197936416245
"Cthrc1",39.9501189092067,-1.14305465055839,0.437314905945475,-2.6138021709713,0.00895408659208878,0.0310575110419134,-3.96858812798005
"Gon4l",3368.44112376949,0.0516801114517098,0.157623915788777,0.327869734697896,0.743010158153274,0.832550546731368,0.00947096658846068
"Ffar4",134.457606040986,-2.51281176351204,0.646673453271434,-3.88575060689451,0.000102014193648848,0.00100559795134745,-17.3438613522358
"Ifi205",513.691973462169,-1.10627903227454,0.377329262002339,-2.93186652528339,0.00336931521039142,0.0147708760671054,-4.6630743928355
"Alkal2",19.7520905685309,-1.48101612058966,0.780452990108789,-1.89763655128443,0.0577439760052994,0.129879752490958,-3.02297048210603
"Gzmm",48.288013649973,-0.474964519342014,0.343227659352742,-1.38381772680471,0.16641427368859,0.289476805101269,-0.588804065554578
"Tprkb",3630.02357995486,1.42687585362333,0.249210962469602,5.72557418615716,1.03084485908612e-08,8.1704931148216e-07,20.0013270043671
"Fra10ac1",1158.39606094143,0.190776097284709,0.135814367637963,1.4046

8273425428,0.160115642188161,0.28150899832144,0.241826038425576
"Fgfr3",2121.40826984231,0.41739924865915,0.199783159756788,2.08926142
307131,0.0366841969595592,0.0917370979391868,0.99709518929139
"Msi1",171.059694882418,-0.612713763973849,0.384568673197823,-1.593249
28595696,0.111104278642405,0.213429677336908,-0.946304474261069
"Lifr",4019.08687852541,0.620724602660464,0.167545784073542,3.70480586
003886,0.000211552699341845,0.001765776768736,3.93487542120873
"Arfgap3",1389.40775447909,0.0359083711876746,0.0895769739080976,0.400
866088918288,0.688518717968664,0.791179747934525,0.00841082121778729
"Prr14l",1018.79669922523,0.237135784827748,0.190983720127548,1.241654
44399857,0.214364084947035,0.349033062905828,0.249606429725004
"P2ry10b",57.2410061144582,-0.813448620272321,0.338391705157062,-2.403
86690298678,0.0162226785189263,0.0490402768623295,-2.45263978779264
"Eapp",2173.39572120591,0.705119984175977,0.187616333059316,3.75830809
971673,0.000171066162145278,0.00150110172345123,4.58437703434637
"D130007C19Rik",106.411172375677,0.661350966700936,0.4111584520105,1.6
0850631542909,0.107724334159126,0.20839458948652,1.0372112255598
"Cpsf3",2139.13555550433,0.12176916459901,0.241992398492388,0.50319417
2038592,0.614827761885762,0.734168665109975,0.0376286795651308
"Obox1",0.110428945274772,-0.058772207075247,4.78471990596623,-0.01228
331192427,0.990199581508171,NA,NA
"Mrps21",4809.8123955119,1.07085194660545,0.23657995415757,4.526384961
13763,6.00012373716668e-06,0.00011413698792905,9.72131303124985
"Lrrc36",29.1623112184176,-0.704274688512074,0.435736424141032,-1.6162
8601487794,0.106032489088291,0.205877461884435,-1.11308792730047
"Taf4b",235.131555155639,0.0934921790548665,0.159770022405609,0.585167
215020584,0.5584352871058,0.687223632162894,0.0350684975037585
"Kcnn4",616.665394882598,-1.63946335067229,0.273124296175851,-6.002627
27859526,1.94149957148549e-09,2.50697739369696e-07,-24.9182327298064
"Bsx",1.44336225556438,1.60312834832099,1.35944642751763,1.17925084495
483,0.23829830523785,0.376404759845074,1.56640103959296
"Lexm",3.35972355330723,1.48484170753773,1.31162351209482,1.1320639603
0997,0.257607545257059,0.39854256267731,1.36596673444123
"Rhob",10999.8752471636,-0.894154663443615,0.167893681003699,-5.325719
5750203,1.00554162945585e-07,4.58085296521733e-06,-10.9924024285962
"Zfp747",372.386294952332,-0.313660562837961,0.194913255676314,-1.6092
3156175097,0.107565717490395,0.208149698256026,-0.492289549202756
"Pnma1",8.57092990872579,-1.28888306316173,0.639592575667793,-2.015162
6397727,0.0438876106678307,0.105319462498629,-2.90096263829693
"Ceacam2",427.877981441492,2.84575288123217,0.874725372197151,3.253310
09215401,0.00114068943160247,0.00650136291651122,14.3304814166078
"Mdm4",7923.73269041562,-0.00899025793640455,0.211857141060253,-0.0424
354727502326,0.966151550641982,0.979463574815025,-0.000186549918306561
"Cklf",356.312738910485,-0.344083205510487,0.24744523721494,-1.3905428
5054435,0.164364096674563,0.287012776889152,-0.429494479123282
"Slfn5",8228.31154897024,-0.743407208227867,0.192795471340362,-3.85593
708741972,0.000115287137263269,0.001111705540237,-5.05655169400697
"Dnajc8",3253.32453802702,-0.262658765112845,0.138717036600831,-1.8934
8598808858,0.0582932698634224,0.130704022055774,-0.534463278388387
"Spcs3",3556.49278215784,-0.0385668417101806,0.172604721654482,-0.2234

40247407502,0.823192883102709,0.886645567723203,-0.00463997525901228
"Tmem74",1.13299309414135,-0.507518434826614,1.88661463481257,-0.26901
0122926898,0.787921892754041,NA,NA
"Slc30a7",1452.45847536297,0.0620330255837663,0.154107151997388,0.4025
31775973757,0.687292707693551,0.79005030153336,0.0146186198536117
"Fabp1",33.1075470776352,1.30739456269408,0.725086311199498,1.80308818
757216,0.0713743659865081,0.152310882701077,2.46029635722547
"Cadps",2.39117673823583,0.0277022979575923,1.95463050906075,0.0141726
51981629,0.98869223835332,0.992458878882467,0.000209698060902923
"Atpif1",8374.62027284609,1.04515794251149,0.380401558911185,2.7475122
4864332,0.00600492658947339,0.0228590508866755,3.94903327756639
"Tmem120b",1246.9997314297,-1.91656706297016,0.621324987098885,-3.0846
4507748041,0.00203795190074723,0.010134145922155,-8.80057844975643
"Gimap4",2383.95991853732,-0.799627817043791,0.25646155189496,-3.11792
4738174,0.00182129306123689,0.00929379013556582,-3.7409858393401
"Cpa1",1.13482250925957,3.07935120279507,2.79907020080808,1.1001336093
343,0.271273912001909,NA,NA
"Tle5",19736.5425033311,0.741210097971347,0.19423687487767,3.816011241
11042,0.000135626328110484,0.00126166132895907,4.94781897281617
"Sytl5",18.3517188535069,-0.652273458309835,0.507554769751089,-1.28512
920611448,0.198747137110833,0.329998439869456,-0.723154287935421
"Vapb",7384.17901923153,-0.166034665250888,0.257030020860663,-0.645973
82319358,0.518296308208646,0.651604965984408,-0.0711154333658291
"Vsnl1",4.44280193978197,-4.31871341766896,1.43428378518061,-3.0110592
2153693,0.00260338072000721,0.0121991630085798,-19.0299267002848
"Lclat1",1716.94232819339,0.209848351549428,0.181689862274315,1.154981
07006432,0.248098178472886,0.387458412804512,0.19896703413929
"Thnsl2",5814.13291569628,2.10834458269105,0.529307089712002,3.9832162
1544538,6.79888466639107e-05,0.000734837323865262,15.2135223160114
"Kcnn2",90.7675154021214,2.11411242547609,0.354232871402964,5.96814298
205303,2.39969041035958e-09,2.90615923112988e-07,31.8200627476721
"Tmem62",886.983733822118,0.485479055007669,0.122919228126924,3.949577
80329024,7.82891608394928e-05,0.000817612243196803,3.45133000724316
"Dedd2",1663.56761086765,0.65809913099784,0.174817127096641,3.76450032
057806,0.000166882454203308,0.0014743425881541,4.29050564301373
"Parp4",1833.64697213554,0.28860765306926,0.223827668629696,1.28941901
97135,0.197252452664054,0.328307740885025,0.321452322648896
"Trim65",933.21388403316,-0.379035586532451,0.119768530654231,-3.16473
437940655,0.00155224586448125,0.0082625415958655,-1.81786340580823
"Zfp867",272.246203320921,-0.401598159683854,0.140316242223294,-2.8620
9317838463,0.00420853159943444,0.0174352403892995,-1.62617609145866
"Sh3bp2",1227.91843649627,-0.0382166940515506,0.284601714828995,-0.134
281320386679,0.893180123631054,0.93382853574598,-0.00261640805387282
"Tmprss11e",1.61238894140696,-3.36478072347166,1.86499860694686,-1.804
17331730883,0.071204140892416,0.152073811374445,-6.33719192682294
"Ugt1a6a",2457.66137587535,-2.95328979860463,0.728133829029161,-4.0559
7114275315,4.9926423742084e-05,0.000578117353584453,-22.0189422064951
"Adam12",822.243024014122,-1.9535488723988,0.33309718663536,-5.8648014
7770611,4.49670376026884e-09,4.48564132124303e-07,-28.5554422656601
"Usp17la",25.7136687709962,0.722606200551671,0.408742527194723,1.76787

623619948,0.0770815963592398,0.161484577824482,1.31756086104057
"Pla2r1",1161.16764056241,-0.92659845036511,0.158161623987003,-5.85855
422451437,4.66914162968562e-09,4.57482027616132e-07,-13.526047043474
"Pabpc1l",41.3095305181015,-1.05618914186468,0.386826684237792,-2.7303
9370059438,0.00632587300236151,0.0238203692159221,-3.94720507462768
"Oscar",14.6608744167952,-0.649028097681888,0.425844549074841,-1.52409
629075192,0.127484659158073,0.236687857956971,-0.935257965689724
"9130230L23Rik",82.391355744844,0.706868111316924,0.252776046153091,2.
79642047604787,0.00516721256484055,0.0203824217255232,2.7518958242924
"Cggbp1",3135.81094126258,-0.367908868095621,0.163080335553392,-2.2559
9773784601,0.0240707728041255,0.0663741943338762,-0.99793328257807
"Kdm2a",5209.36745655147,-0.186318595091036,0.18448812020027,-1.009921
91198425,0.312532703657623,0.45755839134377,-0.145673337234416
"Mgmt",362.415772282507,0.0987497616009765,0.343632229703611,0.2873704
88170303,0.773828655656437,0.853786279311295,0.0156098068396712
"Mettl7a1",12818.2024783476,1.0979734024009,0.380295790831742,2.887156
33691218,0.00388741019648743,0.0164418056423236,4.51039575228978
"Xlr",87.2427421975048,-0.81907604151598,0.344743543616871,-2.37589958
298467,0.0175062262585404,0.0519041214724126,-2.423119406611
"Ugt2b5",419.715176597547,1.56198053631849,0.574402092163117,2.7193155
4155086,0.00654171700633298,0.0244396241022189,5.79736811799135
"Slc8a1",4959.57318642288,1.17286773251381,0.329639945222615,3.5580267
1827814,0.000373651313844934,0.0027440502136906,6.91794952052278
"Mmrn1",181.444424473356,-1.56151867460171,0.321392448326033,-4.858604
12319844,1.1821623444363e-06,3.13957766841986e-05,-16.1911328820412
"Zfp869",2335.87297668213,-0.102454435720507,0.10161982485504,-1.00821
307128464,0.313352184085072,0.45848273189935,-0.0798973141281081
"Pm20d2",21.6026475461689,-0.339235030683903,0.590388817812156,-0.5745
95961930697,0.565564583683615,0.69302276600447,-0.124394917387944
"Ano9",634.022653508075,0.462402023647171,0.197718293202187,2.33869115
577646,0.019351423216564,0.0560230583957024,1.33263889406061
"Olfr63",42.0075517883537,0.448451113789406,0.369918016148425,1.212298
65595265,0.225398078195999,0.361474891319756,0.456327124697191
"Irs4",0.711728778165164,2.22459538812479,3.26906584164948,0.680498801
762378,0.496188680276677,NA,NA
"5830411N06Rik",48.6257038281567,0.281781656431108,0.525308720800627,0
.536411533396291,0.591674162163794,0.714677173104297,0.094657318199314
6
"Tmem119",1641.69593557376,-1.51602972192632,0.39578683032409,-3.83041
982646294,0.000127924810217656,0.00120488723272203,-10.1897956296636
"1600014C10Rik",2989.6987792998,-0.289099486249539,0.395013904640226,-
0.731871670474101,0.464246898895287,0.603177586103839,-0.1461524011930
28
"Srsf12",19.2006043942301,-1.05558426438162,0.461329058758248,-2.28813
738120664,0.0221295219386964,0.0623077861026284,-2.92995239647976
"Emcn",4260.74324838738,-0.240204406411602,0.218847496489512,-1.097588
09337402,0.272384394365132,0.414982881804293,-0.211264101103979
"Adam10",4633.70027427216,-0.583564803391624,0.158560138737017,-3.6804
0043380326,0.000232867974738772,0.00190615439607749,-3.65467231185519
"Ap1s3",683.626994135921,0.0718150879416838,0.214888582402024,0.334196

852801275,0.738231018708132,0.829133403323375,0.0134562958012732
"Ankrd24",2164.66325203812,0.878874326145975,0.233947487163034,3.75671624775137,0.000172157511139165,0.00150999871893452,5.70885691995456
"Zscan22",434.630341327424,-0.25172247966799,0.14931622797201,-1.68583470857017,0.0918276405305674,0.184964394118286,-0.424804826961977
"Zfp771",1391.53151416596,0.423739117595696,0.17178389742419,2.46669870662759,0.0136365044428055,0.0430512570637666,1.33281370165843
"Hmgb2",2484.05202716274,-0.0254485061862213,0.232503864634713,-0.109454121230214,0.912842309022502,0.946353538447837,-0.00140320672112841
"Lrrc8c",811.754877193034,-0.942239769790624,0.164611072157972,-5.72403640556079,1.04022443818671e-08,8.21145590921641e-07,-13.203196405691
"Vmac",957.053104279645,-0.0667763631488,0.181295611747021,-0.368328623651295,0.712628211861562,0.809438708777626,-0.0141174729728959
"1700013H16Rik",1.06988733269841,-0.248207897351763,1.48751185243201,-0.166861122448171,0.867479324003717,NA,NA
"Phactr1",1547.86184319731,0.512894734215074,0.454285211056357,1.12901481653438,0.258891583414684,0.400021245219076,0.469933450709264
"Msra",7171.41984978445,1.58245155404264,0.437820541160516,3.61438398903828,0.00030106258394437,0.00231471540289721,9.60305746405117
"Zfp182",853.030517228513,-0.0511631638935365,0.206577785204747,-0.24767021218098,0.804389581142527,0.875466818482667,-0.0068045999067091
"Gm9955",0.510055759146321,-1.07200145627739,2.68040967671552,-0.399939406871185,0.689201146600275,NA,NA
"Fsd1l",462.591118718842,-0.222077675660531,0.209374215049142,-1.06067347217711,0.288838319750298,0.432747007721784,-0.186012705180952
"AU018091",10.0326519185074,-0.126913734216077,0.595965146624243,-0.21295496042841,0.831362093207673,0.892330627692681,-0.0144578293677616
"Akr1c20",13.4288119902396,0.952397652048317,0.758786660168129,1.25515866585911,0.209421167194757,0.343024920198014,1.01901994481391
"Defb42",66.6619362341712,2.09526015523263,0.393884814610957,5.31947431713541,1.04067502172698e-07,4.70790755768737e-06,25.701020506747
"Set",9025.87010480732,-0.421212903518907,0.0931127522664137,-4.52368653343781,6.07717108402236e-06,0.00011526525466563,-3.81967424187525
"Kctd18",829.411948900009,-0.331240315406965,0.127361582561786,-2.60078674231513,0.00930102530056022,0.0320040182574136,-1.14009399238816
"Klhl18",1335.61357348536,-0.11473522005477,0.17010269765287,-0.674505587716234,0.499989934468014,0.635389870411235,-0.0520343153502259
"Cadm4",2335.63014083921,0.559364812020073,0.356113783691406,1.57074743420995,0.116241326405579,0.221011543857239,0.844383750859201
"Actn4",18684.0205917416,-0.278273266694134,0.111213411937539,-2.50215564693242,0.0123439643170332,0.0397493998767156,-0.897475957809022
"Usp46",1280.68579306324,-0.297650060547107,0.188605092673944,-1.57816555389455,0.114527581420292,0.218504221385041,-0.452710843156789
"Nsd3",4541.6770529793,0.261418049871428,0.116665406012113,2.24075035443057,0.025042250383846,0.0685681502575802,0.700581325173558
"Cyp2c50",2.36970484144572,1.24617065125756,0.949237747806544,1.31281194214743,0.189246324934164,0.318563830058391,1.42553500347048
"Elp6",375.852747357779,-0.0773989373924681,0.149941308027539,-0.51619489259259,0.60571829709883,0.726519153348486,-0.0247282199789644
"Atrnl1",1951.5940645505,0.0674869964099421,0.18775082738434,0.3594497

94976356,0.719258629282114,0.81414043269295,0.0138768385838906
"Smim10l2a",100.414410847422,0.731667520179072,0.341008507671783,2.145
59902089978,0.0319049836338672,0.0823847159547021,1.82650212495068
"Rnd1",194.445576644075,-0.68661410491799,0.254280436312954,-2.7002238
7437208,0.00692928305555509,0.0255642688774292,-2.51751157182465
"Tafa5",440.49604858364,0.784790895328383,0.140423619177518,5.58873856
068529,2.28725000028307e-08,1.51445687513816e-06,10.5165540425403
"Tmem158",782.827630807329,-0.911664646995036,0.417637077736544,-2.182
91118196679,0.0290423514113826,0.0767196542228882,-2.34078773665258
"Pcnx3",4065.88857335835,0.00353095933431148,0.161969253288363,0.02180
01828286824,0.982607348346294,0.988843275184532,3.96153435980284e-05
"4930578G10Rik",7.08401265165482,-1.14380869574602,0.716160227750928,-
1.5971407674203,0.110234332557432,0.212113788236929,-1.77362683599086
"Dsp",1981.65878854233,-0.436922718418882,0.285021662429474,-1.5329456
5295364,0.125289246087208,0.233970857217545,-0.634655901628162
"Olfr1535",0.755847661106642,-1.10113480911238,2.66493952447235,-0.413
193169676298,0.679465092017212,NA,NA
"Txk",62.7924939322002,-0.253247214278295,0.237118356905372,-1.0680202
8144687,0.285511363273895,0.429677412867738,-0.213923127263273
"Zfp667",275.88434403624,-0.521326456142688,0.26518538455258,-1.965894
37620127,0.0493108128346728,0.115158968577129,-1.12681677796257
"Dmac2l",650.21235181829,0.0862041762326512,0.151135012304147,0.570378
596715712,0.568420943101264,0.695299344328008,0.0313277023394206
"Arhgef33",28.6515273574811,0.239291975752768,0.368023204154979,0.6502
08935336587,0.515557270099313,0.649291800309805,0.103343754943537
"Stfa3",0.625009877263103,0.169160961181024,3.10580880606035,0.0544659
931580274,0.956563901883757,NA,NA
"Klhl5",2195.44630258849,-0.32497001571147,0.116339306680088,-2.793295
10364953,0.00521740704729082,0.0205513136581973,-1.26245340103466
"Zkscan4",129.169305138293,-0.796654967794444,0.198361975342329,-4.016
16774797485,5.91521589954975e-05,0.000659433273924648,-5.8348043642004
9
"Afp",54.5147619098077,1.51706562442957,0.465305025071838,3.2603680224
5011,0.00111267736427714,0.00639215770438293,7.665252295252
"Kcnmb4",28.2677173054396,-1.51511874100953,0.445633099661027,-3.39992
415770285,0.0006740454641188,0.00433604040230563,-8.24344849631135
"Olfr1346",1.64649868079026,-0.474976331789551,1.46592898870217,-0.324
010463978927,0.745930112119722,0.834806879332208,-0.0857592865705614
"Zfp174",148.542144547518,-0.0978264139906319,0.232172154753039,-0.421
35291415411,0.673497396663862,0.779418888440524,-0.0243789930862298
"Miga1",1472.4038866153,0.476921206002687,0.213964197752966,2.22897667
465526,0.0258154568007949,0.0702217880443498,1.26674881524238
"9130008F23Rik",297.286444720818,-0.56453435410511,0.222839064167797,-
2.53337248661221,0.0112970834397268,0.0373023764450117,-1.856583138288
89
"Gm9959",2.59966396986309,-0.635297447565001,1.05530727789996,-0.60200
2337015274,0.54717258176574,0.676940654407375,-0.247875065794022
"Nt5c1a",4.21209914879407,-0.531970394492585,0.822392696524004,-0.6468
56905151343,0.517724559124754,0.651097854704012,-0.228266013902975
"Lmntd1",81.8622028617223,-1.36487649093464,0.581567435931815,-2.34689

290803871,0.0189306949923761,0.0551240577899266,-3.95564278618504
"Zfp647",133.993339129599,-0.467585301595812,0.243463220621055,-1.9205
5826914242,0.0547874204716691,0.124547641874385,-0.974011497700453
"Nyap2",0.737060880139507,-2.37720539583195,2.07115579946241,-1.147767
53948157,0.251064541014145,NA,NA
"Kbtbd13",28.410300209859,-1.75976453209099,0.442654155020101,-3.97548
404806249,7.02362519748395e-05,0.000752454088464517,-12.6565266870027
"Sec14l3",192.196966799338,2.27301852918849,0.787701255571346,2.885635
27493655,NA,NA,NA
"Agtr1b",5.96649051554543,-0.103081203373742,0.710876879615172,-0.1450
05705389581,0.884706367154542,0.928068259900966,-0.00769501110752671
"AV320801",0.161595369035782,0,4.78471990595931,0,1,NA,NA
"Naaladl1",12.1365108968066,0.361438426466714,0.549080289331414,0.6582
61521109815,0.510370098958728,0.644674539355874,0.158674967827464
"Lrtm2",161.221306676186,0.586007785121042,0.411322057849668,1.4246933
1254591,0.154245873073103,0.273855949114857,0.758969766705842
"Agap1",3062.25654952611,0.411071874484995,0.17281029666637,2.37874641
971489,0.0173716216534993,0.0516093065671079,1.21843893086593
"Cntn1",338.253871973909,0.282486505125749,0.412671941196918,0.6845304
39133862,0.493640275027331,0.629822215474011,0.130598510506511
"Ep300",2886.43524409132,-0.0229495104839012,0.203765878951338,-0.1126
26856871271,0.910326391814783,0.945075737626967,-0.00129642264512638
"Gabrg3",38.90282696668,-0.820913005001302,0.728264776170794,-1.127217
78103515,0.259650414446821,0.400907806531843,-0.750334013634437
"Smyd1",51.5238935851836,-0.596206688608984,0.281088834464214,-2.12106
144217866,0.0339166321821769,0.0864226992012658,-1.45981500767772
"Sprr2e",0.444298480794803,-2.00300349116891,3.08005446449912,-0.65031
4309131751,0.515489216029,NA,NA
"Olfr420",18.7002314809008,0.586766409261365,0.392500750900544,1.49494
340562433,0.134929212880302,0.247051346862629,0.820392784076538
"Commd5",746.123384406682,0.481707060561057,0.172337346497788,2.795140
28938144,0.00518771977046094,0.0204550374286041,1.87361218354687
"Pdlim1",900.752626654608,-1.56422258343718,0.309109565750547,-5.06041
467736236,4.18345634617891e-07,1.37785526753034e-05,-17.5074006643774
"Nfic",7037.9105573967,0.762702792665522,0.346593960803015,2.200565730
85819,0.0277667818531923,0.0741030266320207,1.98478064011769
"Ddx17",25765.6766218952,-0.118797067734113,0.140512807286313,-0.84545
3663821898,0.397857593159467,0.542287826034483,-0.0726988604246613
"Smyd3",476.882063316553,0.221685190218047,0.31752672862953,0.69816229
699735,0.485075702268423,0.622441990314558,0.105102022355909
"Rab39",40.1390633152413,-1.00970892173238,0.464005618601168,-2.176070
46392312,0.0295499920627466,0.0777866538732866,-2.57857990919188
"Gabra5",0.255569238715336,0.372431776565014,3.99923617985339,0.093125
72696786,0.925803679079472,NA,NA
"Olfr354",50.4732214496376,0.37392726953373,0.299171937807681,1.249874
1435238,0.211345526090734,0.345302727622587,0.397609495213601
"Gm8430",4.66370722097137,1.26447924206387,1.06577147958288,1.18644499
903371,0.235446609915077,0.373321785811512,1.24590977603386
"Spink6",1.59415649271101,0,3.16544989769921,0,1,1,0
"Zfp819",2.06012092373774,-2.02652346606649,1.38113701588668,-1.467286

33202657,0.142298180253356,0.25742530326405,-2.75004439546217
"Phxr2",0.172325363106324,-1.42169013425738,4.76762349444656,-0.298196
813551531,0.765552953647034,NA,NA
"Arntl",691.513363304134,-2.07235797421731,0.16765094230148,-12.361147
1893231,4.240613305824e-35,4.13417391184782e-31,-144.983926113678
"Cgrrf1",1373.62513499073,0.672472145372691,0.209298192058367,3.212985
92577024,0.00131362726570717,0.00724147707852937,3.3138957111413
"Sugct",3392.18036720146,1.98989215441872,0.606892378981351,3.27882211
630122,0.00104241321520902,0.00607259422472229,10.1563486268106
"Klf2",2684.26743937095,-0.388027553093003,0.187285566896281,-2.071849
74007043,0.0382794563465864,0.0948209855101526,-0.91410154549347
"Zfp78",125.760549198656,0.472769771699132,0.289866943844062,1.6309889
1315274,0.102892658915179,0.20162824759077,0.75706024622948
"Ifng",27.01111959284,-0.709634242793514,0.636288514867414,-1.11527117
99951,0.264734231614259,0.406550425585528,-0.638704390960149
"C1ra",2603.67842093651,-1.44003102483469,0.250433464534632,-5.7501541
4777186,8.91621184355298e-09,7.24367910523317e-07,-20.359110258364
"Cstl1",0.320285278254356,2.17908760996114,4.70929709902183,0.46272035
1708911,0.643564828478562,NA,NA
"Fam72a",40.3896566808477,-0.382008127089514,0.282563069690748,-1.3519
393298905,0.176394724514855,0.302839159865339,-0.456329122298841
"Klk15",0.873074129025028,1.56664681629844,1.72459701994527,0.90841326
8827378,0.363659917936811,NA,NA
"Actbl2",1.14372199522911,-0.818600638732389,1.90704224802036,-0.42925
1444000336,0.66774024877969,NA,NA
"Fev",0.243455786905374,-0.599306271643202,4.78471990596188,-0.1252542
01587945,0.90032230840203,NA,NA
"Gm12830",0.133767146871686,-0.546530893334942,4.78471990596623,-0.114
22421877892,0.909060053743706,NA,NA
"Sertad3",440.487026396885,0.0939906345184482,0.152103537040368,0.6179
38519690723,0.536615866190636,0.66817857372178,0.0378970064721185
"Zfp811",121.913676983395,0.130810648794886,0.246055561951796,0.531630
529939056,0.594981915432643,0.717703377079044,0.0433897508170592
"Ankrd17",7159.38679551083,1.03743484346628,0.337871011123096,3.070505
63473264,0.00213696653966863,0.0105112445990058,4.72583698548264
"Foxd2",240.450325438375,-0.482748138779131,0.346064115189412,-1.39496
734157168,0.163025688089799,0.285287638339159,-0.605490401427099
"Pld5",3.28539156749824,0.611900422216189,1.02034363231483,0.599700339
020086,0.548705962273784,0.678505127626474,0.237333681810009
"9430025C20Rik",11.9012683356116,-0.119280295780595,0.553275080523358,
-0.215589496038326,0.829307744217042,0.891096792502143,-0.013753283365
1458
"Zfp935",0.108229817038987,-0.909962624367568,4.78339755770097,-0.1902
33534509919,0.849126134161093,NA,NA
"Wdr86",367.859133682749,-0.344643595054458,0.363115471689852,-0.94912
9469616288,0.342554767772562,0.48711751319603,-0.24788486604362
"Kcmf1",3497.10105557254,-0.249430751961068,0.249710366296492,-0.99888
02453836,0.317852706929569,0.462913958747589,-0.192115076248373
"Zfp101",1044.36105764201,0.608689156188583,0.154280709843322,3.945335
46550785,7.96883353150566e-05,0.00082955854883768,4.31841635458197

"Ntrk2",830.388736319966,-3.54887902843796,0.768794783074773,-4.616159
09286522,3.90907321168926e-06,8.23100534357637e-05,-33.3772686643781
"Tmem245",4524.35732922728,0.23207917783403,0.207681185947815,1.117478
10363691,0.263789956711438,0.405702492150625,0.209366787608268
"Adh7",523.537094872505,-2.0349949190471,0.723636844532584,-2.81217703
938438,0.00492074111433397,0.019628602751081,-7.9990917937717
"Mrfap1",17279.9380194694,0.0155655060315392,0.188061628239099,0.08276
8112651611,0.934035924520252,0.958802786298719,0.000654838821443223
"Zfp93",324.701686434749,0.532592700917336,0.198839392953652,2.6785069
7493066,0.00739511919424283,0.026853212403408,1.92658476403478
"Them7",3258.06885435969,2.11385119431825,0.710514885785113,2.97509768
846357,0.00292895258631903,0.0132810970995461,9.13482514187003
"Pgbd1",193.674844582348,0.256039620896844,0.199902475798694,1.2808226
6051913,0.2002559627421,0.331769119002929,0.282492604397436
"Sec23ip",1462.8208806132,-0.275997873728861,0.136623539079718,-2.0201
3412613927,0.0433694768972401,0.104410181570859,-0.623597346934464
"Tead1",3885.75216010961,0.0524496366902437,0.214202633528455,0.244859
90590436,0.806564907214012,0.876953246019005,0.00688672118002089
"Tns1",39951.4129084401,0.755440602750707,0.264005350107181,2.86145944
559083,0.0042169545007438,0.017453232616324,3.05819761138834
"Fat2",403.728832529308,-3.56160701097332,0.807740682231968,-4.4093445
9451987,1.03683930851043e-05,0.000176068399660679,-30.7888033223345
"Snupn",499.543008425163,-0.0666482643141793,0.170901364967407,-0.3899
80877723767,0.696550687050574,0.797506851713595,-0.0150801597190648
"Zfp457",74.4327076548948,0.443739561566157,0.222545817149608,1.993924
51967699,0.0461603038965857,0.109240636651976,0.982529093204361
"Prl2c5",0.277425164135561,1.60101573119522,3.85332092994256,0.4154898
48964926,0.677783344714188,NA,NA
"Slc6a2",17.682504076627,-1.10988320749599,1.61587493667114,-0.6868620
72248276,0.492169649587855,0.628648793165018,-0.515188401552563
"Stam2",1363.43212305338,-0.29233182962881,0.155993268430263,-1.874002
85006207,0.0609300322892037,0.135124405092686,-0.585119523172247
"Fut9",9301.88943614404,2.15109384658386,0.60673913740301,3.5453355717
1054,0.000392113543805872,0.00285169929203788,12.6050662382674
"Rnf212",0.268570282422638,2.78587384602754,4.71560524763364,0.5907775
77793547,0.554669460441736,NA,NA
"Fbxo6",1296.73227501094,-0.0620980164834134,0.141540886561135,-0.4387
2846915221,0.660858296087449,0.769692076764415,-0.0162550714138823
"Map6",265.33586457875,-2.14272338631555,0.512914463930808,-4.17754525
753557,2.94672044324152e-05,0.000384843673811732,-16.8475340621081
"Nell1",4.01881940768505,-3.07919268172254,1.39828946967978,-2.2021139
0308739,0.027657263642439,0.0739626013578764,-8.01882034301393
"H2-
Q5",788.732960243192,-1.31003022540344,0.335033500755442,-3.9101469627
6506,9.22399835090299e-05,0.000927537492758672,-9.14791137980799
"Atp10b",453.527218265013,-1.31400548625207,0.457701085290732,-2.87088
129891021,0.00409329199543425,0.0170864926840028,-5.34730200239928
"Pcdh9",158.545701223325,-0.744475559771772,0.473140300467764,-1.57347
737877276,0.115608322256791,0.21997961036039,-1.12730009264201
"Nap1l5",149.437439989153,0.811254555598683,0.34768870358171,2.3332784

3913695,0.0196335363007273,0.0566377704973489,2.32917607485407
"Maf",16797.0085357779,1.16357404980177,0.373982293210796,3.1113078638
3492,0.00186260640544576,0.00944773665280473,5.42455903456978
"Srsf11",15301.4489787444,0.518424645837972,0.258378667695529,2.006452
97253749,0.0448079414158497,0.106896518013733,1.15914257263035
"Cd47",9452.44831948722,-0.128274387409714,0.140512831181831,-0.912901
59290663,0.36129429969299,0.506180660732479,-0.0873371093195381
"Gm7353",1.62239356528916,-3.61398961953465,2.30593005175584,-1.567258
99676913,0.117054165385848,0.22225359009575,-5.43521000066495
"Alk",13.310044656165,3.63834697378026,0.831107027676856,4.37771171776
795,1.19931838388185e-05,0.000195898824286807,31.0638871412695
"Zfp458",179.400873828637,-0.0376660131380967,0.217773376839933,-0.172
959678013267,0.86268312216592,0.913269384080308,-0.00341722599599559
"Soga1",451.691782301596,-0.67048097710764,0.311789854174476,-2.150425
89786274,0.0315215416460993,0.0815994449038295,-1.68018028550807
"Ano5",226.819224016672,-0.526610545406821,0.598231050206584,-0.880279
526154601,0.378707901049206,0.523505611815485,-0.340826309800516
"Pprc1",1090.57007467821,-0.268359967047312,0.193004069313987,-1.39043
683379926,0.164396267972977,0.287012776889152,-0.334974571320696
"Epm2a",445.392860316687,-0.074587621356933,0.378543571640942,-0.19703
840441301,0.84379748447429,0.899917269871446,-0.00786544683355808
"Gm9974",19.0196413961847,0.621103728942466,0.411523014763664,1.509280
6639239,0.131227073921972,0.24172560107044,0.881937529340463
"Gucy2g",36.3463226187567,-0.621120731368565,0.74878515670245,-0.82950
4599295074,0.406818935328853,0.550729403668818,-0.370505777708103
"Cpsf6",3368.15472456004,-0.348378335898002,0.0924601804393138,-3.7678
7428104426,0.000164643577979301,0.00145985469915435,-2.27470790589307
"Zcchc24",1676.73197457026,-1.74165478835687,0.315053693618016,-5.5281
2051925514,3.23679671113622e-08,1.94619501646229e-06,-22.9021236573138
"Epha6",8.51081948648446,-0.457128411569614,0.745097675275135,-0.61351
4746775736,0.539536035012465,0.670397247685001,-0.182798719573519
"Lair1",574.139024585429,-1.17722611607443,0.516270819694814,-2.280249
18543785,0.0225929136933303,0.0632562652487873,-3.24980462307326
"Timd4",9.35334053992145,-2.72060143194192,0.768976245704805,-3.537952
50131342,0.000403242590369408,0.00291093077638753,-15.8863600440241
"Kxd1",2284.13116125102,0.352564572740197,0.263000487291119,1.34054722
244654,0.180067502599655,0.307573908514067,0.415687687207918
"4930502E18Rik",28.6405631959229,2.06022643902129,0.682070028746728,3.
02054972684676,0.00252316272626874,0.0119149011471998,9.12673197487437
"Zfp459",88.2000876366496,0.0120852184825063,0.309096188305837,0.03909
85684706941,0.968811802292984,0.980732699294357,0.00023512196601762
"Spink5",1.10930247376288,0.957194047304539,1.3147862887736,0.72802253
5280153,0.466599791508768,NA,NA
"Unc80",3.96319986514656,-1.70991668714196,1.6714064587799,-1.02304061
238951,0.3062886441587,0.450760277440248,-1.36249515873091
"Olfr305",0.862079785797724,2.92389887133546,1.88888454966079,1.547950
01730547,0.121634330946951,NA,NA
"Tcp10b",42.9612223652468,0.483621468779369,0.362466496098336,1.334251
50733977,0.182121444200922,0.310249719326935,0.566019811107651
"Cdca7",239.001615487477,-1.44953833327493,0.352856617320996,-4.108009

49201493,3.99083706538288e-05,0.000487089909119285,-11.0557184980712
"B4galnt4",87.7085365977006,-0.535152416619791,0.505509065973964,-1.05
864059151682,0.289763493230938,0.433500237168483,-0.447314069981485
"Hmcn2",416.135610698922,1.68984627762971,0.39992266077135,4.22539458
136961,2.38522465657863e-05,0.000329603900453367,13.5485434977222
"Zfp580",346.11484174807,-0.310586179078898,0.161233991931673,-1.92630
707307995,0.0540660427788588,0.123353580868499,-0.649963823144052
"Dach1",1161.8057751916,-0.0236842435280369,0.176430139565966,-0.13424
148269849,0.893211624306521,0.93382853574598,-0.00162148105833154
"Klhl25",766.748362063142,-1.30451842011886,0.244391016832,-5.33783294
095304,9.4064066946699e-08,4.34611653394962e-06,-16.1058816655105
"Gpc3",2452.92856320524,-1.31504872792332,0.33947083543467,-3.87381945
856848,0.000107142799265654,0.00104558073077164,-9.02541984312152
"Mettl4",836.699374385375,-0.00748464135583999,0.150800469679662,-0.04
96327456521802,0.960415051523341,0.975931450625501,-0.0001823477938053
69
"Zzef1",2942.49208249955,0.0967557835113618,0.130263686670163,0.742768
656289871,0.457621754957462,0.597444061650789,0.0498383835625793
"Kbtbd11",2255.22150910766,0.0971273646709877,0.232025369533961,0.4186
06658685965,0.675503622400392,0.781149969133672,0.0239893057482635
"Gm5069",115.218596717606,0.583525426770787,0.248741954575707,2.345906
73602343,0.0189808562799128,0.0552124027667821,1.69022088180049
"Cope",4378.44509246603,0.235978940150693,0.112661246568531,2.09458839
963348,0.0362075973102261,0.0907540470953579,0.566255586473556
"Gja6",5.54493365014897,-0.429685838776169,0.64280632761073,-0.6684530
32149332,0.5038444532435,0.638778613042673,-0.192584051306045
"Tmem191c",943.541968488486,0.199034360342038,0.187177925426446,1.0633
4312600473,0.287626374536987,0.431660949101152,0.167211719580256
"Klhl26",1256.94923025318,-0.517179979777444,0.175261863647971,-2.9508
9855267227,0.00316850966651686,0.0140702147090533,-2.20509777037412
"Slain1",287.316081160872,0.21924866400575,0.447644201869841,0.4897833
2141896,0.62428723409822,0.741734001995496,0.0655037367632447
"Ubl7",1530.20560642596,0.233651700751073,0.139065993312822,1.68014979
927901,0.092928173767064,0.186632904748982,0.392210471057696
"Rras2",2083.32006418337,-0.263619984400998,0.173674387412989,-1.51789
78796345,0.129040144589072,0.238825319335333,-0.377509848158336
"Paqr3",188.17678196248,-0.548987727752661,0.281194784182036,-1.952339
65434175,0.050897892434646,0.118007936494353,-1.1731886398607
"Ces2a",5.75256273419465,0.0412352839755194,0.678224529418309,0.060798
8684969645,0.951519391464719,0.969930765084564,0.00125893739328122
"Nap1l3",129.726740357082,0.198242917289958,0.176212162862454,1.125024
02824884,0.260578852361702,0.401959372100354,0.180679439312565
"Ghr",17547.5260616617,1.09999656695071,0.49385975910261,2.22734601610
284,0.0259241573479995,0.0703996128093726,2.91891515744206
"Rtl6",490.333214506775,-0.37869186567798,0.218668503221146,-1.7318080
1121137,0.0833077513011276,0.171415632637124,-0.667885237625143
"Gsdmc4",15.3894810525886,-2.15160692363805,1.18434654537784,-1.816703
84570728,0.0692624725800304,0.148944489948763,-4.09705029964343
"Gemin6",270.798163063533,0.117915157337014,0.120859449813328,0.975638
706937172,0.329243536050686,0.474028765207942,0.0880221642500808

"Nkain3",50.5470175684916,0.916847641018482,0.591517224482161,1.549993
1414865,0.12114316217957,0.228217331031619,1.35460287141892
"Eef1d",7068.79737584502,0.307154363584813,0.175126066627,1.7539043130
5135,0.0794469044424932,0.165479728962689,0.552542004277398
"Gm7936",0.088158751752442,-0.0671475021926688,4.78471990596623,-0.014
0337372954559,0.988803065214312,NA,NA
"Myh8",26.3646889225518,0.272429601243069,0.388606581288723,0.70104217
0566488,0.483276698190486,0.620828110509823,0.129867471524199
"Usp26",0.463154290068376,0,4.78471990594789,0,1,NA,NA
"Abcd2",1126.84511327397,-3.72552389453585,1.19161358187137,-3.1264530
3075944,0.00176928764365047,0.0091046636252037,-17.506119465351
"Ftdc",0.185015883193048,0,4.78471990595786,0,1,NA,NA
"Gm5160",0.720632436308967,-0.747203655793053,2.32176426809532,-0.3218
2580551385,0.747584663521559,NA,NA
"Tcf7l1",824.276100788444,-0.663165372281858,0.2538322177633,-2.612613
08011051,0.00898529620817601,0.0311458320830251,-2.30057031053344
"Fmnl1",3497.96755105285,0.854113475152657,0.320073844051891,2.6684888
2226748,0.00761933137542037,0.0274402887251471,3.07117251030453
"Dnaaf3",257.292579669682,-1.178958503373,0.407692271230155,-2.8917852
6690157,0.00383059661160868,0.0162508643892833,-4.85684827148873
"Cldn17",2.27527086699315,0.24375552975372,1.23284867314395,0.19771731
5242029,0.843266241544704,0.899650097266286,0.0257769942095389
"Mta3",1193.00834835054,-0.29549201466767,0.122354697347245,-2.4150443
0213299,0.0157332959186509,0.047835303886146,-0.898293164763982
"Olfr967",12.9197142739169,0.267084961722744,0.612599151095802,0.43598
6503156247,0.662846522096756,0.771313627379049,0.0693513367500141
"Tescl",3.52501041131364,1.02618040026686,0.754296865026413,1.36044632
80262,0.17368872644029,0.299516302180497,1.23714916692269
[truncated: 555,813 more chars]
